# Supplementary material for: Scutellaria barbata D. Don Inhibits the Main Proteases (Mpro and TMPRSS2) of Severe Acute Respiratory Syndrome Coronavirus 2 (SARS-CoV-2) Infection
Source: Viruses. 2021 May 2;13(5):826. doi: 10.3390/v13050826 (PMC8147405; doi:10.3390/v13050826)
Supplement: Supplementary file 1 [file viruses-13-00826-s001.zip › viruses-1186444-suppl/viruses-1186444-suppl table.pdf]

SPECTRUM - MS *Scutellaria barbata* **(SB)**
20200817-025.raw
ITMS + p ESI E Full ms [200.00-2000.00]
Scan #: 1-5340
RT: 0.00-30.01
AV: 1148
Data points: 45000

Mass	Intensity	Ratio	MSX Ratio	average molecular weight
200.04	3208.411947	7.24094E-05	0.014484774	547.6803639
200.08	4080.054074	9.20811E-05	0.018423594	
200.12	4282.170501	9.66426E-05	0.019340122	
200.16	3727.838347	8.41321E-05	0.016839885	
200.2	2751.352678	6.20942E-05	0.012431258	
200.24	1749.784465	3.94902E-05	0.007907517	
200.28	987.093369	2.22773E-05	0.004461702	
200.32	509.565744	1.15002E-05	0.002303718	
200.36	262.194464	5.91736E-06	0.001185603	
200.4	166.246546	3.75195E-06	0.000751891	
200.44	151.340754	3.41555E-06	0.000684613	
200.48	181.402474	4.094E-06	0.000820765	
200.52	231.898398	5.23362E-06	0.001049446	
200.56	273.018178	6.16164E-06	0.001235779	
200.6	264.25171	5.96379E-06	0.001196337	
200.64	203.327042	4.58881E-06	0.000920698	
200.68	129.104828	2.91372E-06	0.000584725	
200.72	85.131728	1.9213E-06	0.000385644	
200.76	67.732899	1.52864E-06	0.000306889	
200.8	77.971915	1.75972E-06	0.000353351	
200.84	146.948381	3.31642E-06	0.00066607	
200.88	360.086542	8.12665E-06	0.001632482	
200.92	947.359964	2.13806E-05	0.004295789	
200.96	2118.244966	4.78058E-05	0.009607061	
201	3945.630857	8.90474E-05	0.017898526	
201.04	5875.503846	0.000132602	0.026658294	
201.08	7060.534391	0.000159346	0.032041379	
201.12	7062.086521	0.000159381	0.032054798	
201.16	5942.578048	0.000134116	0.026978716	
201.2	4167.585299	9.40566E-05	0.018924187	
201.24	2452.326433	5.53456E-05	0.011137747	
201.28	1261.408415	2.84682E-05	0.005730086	
201.32	597.58367	1.34866E-05	0.002715129	
201.36	293.846419	6.6317E-06	0.00133536	
201.4	190.917301	4.30874E-06	0.00086778	
201.44	161.675447	3.64879E-06	0.000735012	
201.48	178.494114	4.02836E-06	0.000811635	
201.52	234.147617	5.28439E-06	0.001064909	

201.56	276.698373	6.2447E-06	0.001258681
201.6	257.15784	5.80369E-06	0.001170025
201.64	201.603122	4.5499E-06	0.000917442
201.68	143.454138	3.23756E-06	0.000652951
201.72	101.833962	2.29825E-06	0.000463603
201.76	77.479861	1.74861E-06	0.0003528
201.8	70.642558	1.5943E-06	0.000321731
201.84	127.758839	2.88334E-06	0.000581973
201.88	353.17302	7.97062E-06	0.001609109
201.92	962.79781	2.1729E-05	0.004387521
201.96	2241.29477	5.05829E-05	0.010215723
202	4236.448189	9.56107E-05	0.019313368
202.04	6419.399771	0.000144877	0.029270931
202.08	7836.854728	0.000176867	0.035741262
202.12	7901.718788	0.000178331	0.036044219
202.16	6639.413737	0.000149842	0.030292123
202.2	4679.063101	0.0001056	0.021352307
202.24	2823.48185	6.37221E-05	0.012887148
202.28	1502.608513	3.39118E-05	0.006859675
202.32	758.590048	1.71203E-05	0.003463783
202.36	416.14858	9.39189E-06	0.001900544
202.4	276.537487	6.24107E-06	0.001263192
202.44	267.091169	6.02788E-06	0.001220283
202.48	363.793288	8.21031E-06	0.001662423
202.52	493.548735	1.11387E-05	0.002255811
202.56	552.261986	1.24638E-05	0.002524664
202.6	506.495363	1.14309E-05	0.002315899
202.64	369.523282	8.33963E-06	0.001689942
202.68	238.481875	5.3822E-06	0.001090865
202.72	153.341266	3.4607E-06	0.000701553
202.76	112.881905	2.54759E-06	0.000516549
202.8	116.736941	2.63459E-06	0.000534295
202.84	182.712478	4.12357E-06	0.000836424
202.88	467.950957	1.0561E-05	0.002142616
202.92	1282.843223	2.8952E-05	0.005874937
202.96	2853.982934	6.44104E-05	0.013072739
203	5071.988034	0.000114468	0.023236944
203.04	7328.928122	0.000165404	0.033583567
203.08	8721.38252	0.000196829	0.039972126
203.12	8563.95021	0.000193276	0.039258308
203.16	7051.629214	0.000159145	0.032331989
203.2	4893.27504	0.000110434	0.02244027
203.24	2882.810611	6.5061E-05	0.013223002
203.28	1496.92626	3.37835E-05	0.006867518
203.32	728.337269	1.64376E-05	0.003342084
203.36	378.71192	8.547E-06	0.001738118

203.4	284.818712	6.42796E-06	0.001307447
203.44	347.802076	7.84941E-06	0.001596884
203.48	539.489122	1.21755E-05	0.002477474
203.52	736.739378	1.66272E-05	0.003383964
203.56	851.257709	1.92117E-05	0.003910734
203.6	821.308776	1.85358E-05	0.003773888
203.64	622.919326	1.40584E-05	0.002862857
203.68	440.812839	9.94853E-06	0.002026317
203.72	383.081999	8.64563E-06	0.001761287
203.76	436.951835	9.86139E-06	0.002009358
203.8	643.72727	1.4528E-05	0.002960812
203.84	1140.345659	2.5736E-05	0.005246029
203.88	2182.212622	4.92495E-05	0.010040988
203.92	4058.729916	9.15999E-05	0.018679048
203.96	6768.894882	0.000152765	0.031157853
204	9808.708136	0.000221369	0.045159252
204.04	12144.66913	0.000274088	0.05592497
204.08	12864.95089	0.000290344	0.059253408
204.12	11566.10428	0.000261031	0.05328162
204.16	8890.101287	0.000200637	0.040962091
204.2	5888.929683	0.000132905	0.027139189
204.24	3460.657153	7.81022E-05	0.015951596
204.28	1829.12024	4.12807E-05	0.008432821
204.32	899.67024	2.03043E-05	0.004148575
204.36	469.704932	1.06006E-05	0.002166336
204.4	338.78913	7.646E-06	0.001562842
204.44	409.179756	9.23462E-06	0.001887925
204.48	646.523374	1.45911E-05	0.002983595
204.52	1032.526989	2.33027E-05	0.004765867
204.56	1368.748137	3.08907E-05	0.00631901
204.6	1352.765782	3.053E-05	0.006246446
204.64	1010.743039	2.28111E-05	0.004668056
204.68	601.501774	1.35751E-05	0.002778543
204.72	316.259377	7.13753E-06	0.001461196
204.76	186.160148	4.20138E-06	0.000860274
204.8	178.855862	4.03653E-06	0.000826681
204.84	364.495726	8.22616E-06	0.001685047
204.88	1083.582694	2.4455E-05	0.005010331
204.92	2999.054999	6.76845E-05	0.013869907
204.96	6923.653836	0.000156257	0.03202648
205	12995.51701	0.000293291	0.060124597
205.04	19197.38577	0.000433258	0.088835273
205.08	22481.64915	0.00050738	0.104053389
205.12	21207.87383	0.000478632	0.098177031
205.16	16390.76058	0.000369917	0.075892097
205.2	10481.60413	0.000236555	0.048541128

205.24	5643.251354	0.00012736	0.026139433
205.28	2609.584975	5.88947E-05	0.012089904
205.32	1102.917263	2.48913E-05	0.005110683
205.36	499.999617	1.12843E-05	0.002317343
205.4	316.895333	7.15189E-06	0.001468997
205.44	318.250415	7.18247E-06	0.001475566
205.48	487.112483	1.09935E-05	0.002258934
205.52	820.807395	1.85245E-05	0.003807151
205.56	1261.665707	2.8474E-05	0.005853123
205.6	1475.965326	3.33105E-05	0.006848635
205.64	1243.526118	2.80647E-05	0.005771215
205.68	784.319215	1.7701E-05	0.00364074
205.72	407.220803	9.19041E-06	0.00189065
205.76	210.210519	4.74416E-06	0.000976158
205.8	155.861006	3.51757E-06	0.000723915
205.84	221.636366	5.00202E-06	0.001029617
205.88	556.931367	1.25692E-05	0.00258774
205.92	1535.928443	3.46638E-05	0.007137963
205.96	3588.627837	8.09903E-05	0.016680769
206	6749.298914	0.000152322	0.031378389
206.04	10175.77026	0.000229653	0.047317696
206.08	12283.90741	0.000277231	0.057131699
206.12	11992.2799	0.000270649	0.055786183
206.16	9627.902057	0.000217288	0.044796164
206.2	6467.030718	0.000145952	0.030095277
206.24	3730.02592	8.41815E-05	0.01736159
206.28	1929.861354	4.35543E-05	0.008984377
206.32	953.915413	2.15285E-05	0.004441769
206.36	501.856969	1.13262E-05	0.002337277
206.4	328.801181	7.42058E-06	0.001531609
206.44	320.159423	7.22555E-06	0.001491643
206.48	381.970344	8.62054E-06	0.001779969
206.52	478.722725	1.08041E-05	0.002231264
206.56	556.262112	1.25541E-05	0.002593167
206.6	507.464428	1.14528E-05	0.002366141
206.64	372.985959	8.41777E-06	0.001739449
206.68	249.75168	5.63655E-06	0.001164962
206.72	180.576859	4.07537E-06	0.00084246
206.76	144.266247	3.25589E-06	0.000673187
206.8	150.382114	3.39391E-06	0.000701862
206.84	326.440701	7.36731E-06	0.001523855
206.88	1004.563007	2.26716E-05	0.004690298
206.92	2827.128109	6.38043E-05	0.013202395
206.96	6622.044595	0.00014945	0.030930239
207	12492.98161	0.000281949	0.058363483
207.04	18629.59359	0.000420444	0.087048721

207.08	22404.71358	0.000505643	0.104708589
207.12	21854.16314	0.000493218	0.102155317
207.16	17475.40852	0.000394396	0.081702998
207.2	11688.98051	0.000263804	0.054660191
207.24	6612.751803	0.000149241	0.030928621
207.28	3213.728347	7.25294E-05	0.015033888
207.32	1417.009854	3.19799E-05	0.006630081
207.36	637.587865	1.43895E-05	0.002983801
207.4	362.561878	8.18252E-06	0.001697054
207.44	312.181814	7.04551E-06	0.00146152
207.48	360.089601	8.12672E-06	0.001686132
207.52	452.95633	1.02226E-05	0.002121393
207.56	491.979412	1.11033E-05	0.002304599
207.6	447.839048	1.01071E-05	0.002098235
207.64	349.619488	7.89042E-06	0.001638368
207.68	270.430352	6.10324E-06	0.00126752
207.72	256.521027	5.78932E-06	0.001202558
207.76	347.129062	7.83422E-06	0.001627637
207.8	765.023873	1.72655E-05	0.003587776
207.84	1905.690452	4.30088E-05	0.008938945
207.88	4211.239924	9.50418E-05	0.019757293
207.92	7715.877576	0.000174137	0.036206484
207.96	11714.29846	0.000264375	0.054979508
208	15026.27615	0.000339122	0.070537402
208.04	16988.99308	0.000383418	0.079766262
208.08	17017.2526	0.000384056	0.079914308
208.12	14984.21495	0.000338173	0.070380536
208.16	11439.35093	0.00025817	0.053740712
208.2	7555.089535	0.000170508	0.035499734
208.24	4344.742088	9.80548E-05	0.020418927
208.28	2251.485108	5.08129E-05	0.010583307
208.32	1137.660676	2.56754E-05	0.005348703
208.36	626.6521	1.41427E-05	0.002946766
208.4	442.524702	9.98717E-06	0.002081325
208.44	417.844168	9.43016E-06	0.001965623
208.48	456.642651	1.03058E-05	0.002148551
208.52	512.883316	1.15751E-05	0.002413632
208.56	575.261846	1.29829E-05	0.002707705
208.6	555.260538	1.25315E-05	0.002614062
208.64	424.255148	9.57485E-06	0.001997696
208.68	289.561701	6.535E-06	0.001363725
208.72	210.833483	4.75822E-06	0.000993135
208.76	192.58551	4.34639E-06	0.000907352
208.8	264.200895	5.96265E-06	0.001245001
208.84	572.828081	1.29279E-05	0.002699869
208.88	1521.454103	3.43371E-05	0.007172333

208.92	3829.48736	8.64262E-05	0.018056161
208.96	8232.447951	0.000185795	0.038823699
209	14836.89756	0.000334848	0.069983255
209.04	22068.98293	0.000498066	0.10411576
209.08	27004.18269	0.000609447	0.127423135
209.12	27447.71327	0.000619457	0.129540776
209.16	23225.10774	0.000524158	0.109632957
209.2	16439.25994	0.000371011	0.077615543
209.24	9796.868595	0.000221102	0.046263315
209.28	4879.771834	0.00011013	0.023047934
209.32	2101.42632	4.74263E-05	0.009927266
209.36	882.037702	1.99064E-05	0.004167596
209.4	480.507376	1.08444E-05	0.002270814
209.44	417.110703	9.41361E-06	0.001971586
209.48	469.015614	1.0585E-05	0.002217352
209.52	548.066432	1.23691E-05	0.002591573
209.56	601.938711	1.35849E-05	0.002846855
209.6	564.95874	1.27503E-05	0.00267247
209.64	446.18864	1.00699E-05	0.002111045
209.68	311.046013	7.01988E-06	0.001471927
209.72	215.457266	4.86257E-06	0.001019778
209.76	187.655807	4.23513E-06	0.000888361
209.8	269.720149	6.08721E-06	0.001277096
209.84	543.469894	1.22654E-05	0.002573763
209.88	1192.666204	2.69168E-05	0.005649301
209.92	2532.642035	5.71582E-05	0.01199865
209.96	4900.17277	0.00011059	0.023219492
210	8258.595104	0.000186385	0.039140848
210.04	11818.23824	0.000266721	0.056022116
210.08	14245.52799	0.000321502	0.067541082
210.12	14140.18309	0.000319124	0.067054385
210.16	11553.71036	0.000260751	0.054799462
210.2	7914.353982	0.000178616	0.037545072
210.24	4624.492613	0.000104368	0.021942403
210.28	2395.833694	5.40706E-05	0.011369972
210.32	1192.063095	2.69032E-05	0.005658282
210.36	641.245405	1.4472E-05	0.003044333
210.4	463.871593	1.04689E-05	0.002202664
210.44	471.360018	1.06379E-05	0.002238648
210.48	572.721621	1.29255E-05	0.002720565
210.52	679.309306	1.53311E-05	0.003227496
210.56	704.069835	1.58899E-05	0.003345772
210.6	590.806514	1.33337E-05	0.002808073
210.64	420.271243	9.48494E-06	0.001997907
210.68	282.940831	6.38558E-06	0.001345314
210.72	195.313624	4.40796E-06	0.000928845

210.76	157.638862	3.55769E-06	0.000749819
210.8	163.118741	3.68136E-06	0.000776031
210.84	288.621195	6.51378E-06	0.001373365
210.88	775.471163	1.75013E-05	0.003690675
210.92	2145.966768	4.84315E-05	0.010215168
210.96	5226.435659	0.000117953	0.024883444
211	10204.35468	0.000230298	0.048592893
211.04	15971.3478	0.000360451	0.076069595
211.08	20043.78392	0.00045236	0.095484209
211.12	20319.16032	0.000458575	0.096814385
211.16	16882.92304	0.000381024	0.08045704
211.2	11684.46447	0.000263702	0.055693881
211.24	6860.537134	0.000154833	0.032706874
211.28	3480.612242	7.85526E-05	0.016596587
211.32	1588.753666	3.5856E-05	0.007577081
211.36	752.687794	1.69871E-05	0.003590397
211.4	543.567737	1.22676E-05	0.002593364
211.44	720.357082	1.62575E-05	0.003437476
211.48	1176.834512	2.65595E-05	0.005616806
211.52	1606.924608	3.62661E-05	0.007670995
211.56	1646.026031	3.71485E-05	0.00785914
211.6	1276.823328	2.88161E-05	0.006097492
211.64	798.008949	1.801E-05	0.003811626
211.68	436.3023	9.84674E-06	0.002084357
211.72	242.711005	5.47765E-06	0.001159728
211.76	174.997725	3.94945E-06	0.000836337
211.8	183.190075	4.13434E-06	0.000875654
211.84	298.787093	6.74321E-06	0.001428481
211.88	693.115467	1.56427E-05	0.003314365
211.92	1787.918541	4.03508E-05	0.008551148
211.96	3987.26226	8.9987E-05	0.019073635
212	7381.897441	0.000166599	0.035319018
212.04	11036.87968	0.000249087	0.052816408
212.08	13286.22861	0.000299852	0.063592543
212.12	13008.73747	0.000293589	0.062276117
212.16	10635.1876	0.000240021	0.050922934
212.2	7289.354856	0.000164511	0.034909146
212.24	4296.367404	9.6963E-05	0.020579433
212.28	2280.061973	5.14578E-05	0.010923467
212.32	1149.230434	2.59365E-05	0.005506844
212.36	619.331638	1.39775E-05	0.002968252
212.4	453.66404	1.02386E-05	0.002174671
212.44	479.570214	1.08232E-05	0.002299287
212.48	655.153908	1.47859E-05	0.00314171
212.52	863.575926	1.94897E-05	0.004141952
212.56	908.243949	2.04978E-05	0.004357013

212.6	733.287874	1.65493E-05	0.003518378
212.64	510.03745	1.15108E-05	0.002447664
212.68	336.841989	7.60205E-06	0.001616805
212.72	223.859237	5.05219E-06	0.001074702
212.76	174.198745	3.93142E-06	0.00083645
212.8	183.654286	4.14482E-06	0.000882018
212.84	336.385983	7.59176E-06	0.001615831
212.88	898.851127	2.02858E-05	0.004318445
212.92	2405.014175	5.42778E-05	0.011556833
212.96	5491.911407	0.000123945	0.026395283
213	10239.14688	0.000231083	0.049220739
213.04	15315.91084	0.000345659	0.073639145
213.08	18314.59945	0.000413335	0.088073422
213.12	17961.12464	0.000405358	0.086389804
213.16	14744.00524	0.000332752	0.070929344
213.2	10141.79779	0.000228886	0.048798548
213.24	5955.564383	0.000134409	0.028661331
213.28	3067.727034	6.92343E-05	0.014766297
213.32	1426.152002	3.21863E-05	0.006865974
213.36	665.217466	1.5013E-05	0.00320318
213.4	403.697178	9.11088E-06	0.001944262
213.44	377.007466	8.50853E-06	0.001816061
213.48	480.004964	1.0833E-05	0.002312638
213.52	609.272769	1.37504E-05	0.002935993
213.56	638.076267	1.44005E-05	0.003075369
213.6	567.325615	1.28037E-05	0.002734881
213.64	433.345961	9.78001E-06	0.002089402
213.68	291.516797	6.57913E-06	0.001405828
213.72	189.841244	4.28445E-06	0.000915673
213.76	143.418438	3.23675E-06	0.000691889
213.8	157.909608	3.5638E-06	0.00076194
213.84	255.762528	5.7722E-06	0.001234328
213.88	575.903073	1.29973E-05	0.002779869
213.92	1407.848104	3.17732E-05	0.006796917
213.96	3082.298447	6.95632E-05	0.014883739
214	5654.620141	0.000127617	0.027310017
214.04	8527.161568	0.000192446	0.041191176
214.08	10470.97687	0.000236315	0.050590393
214.12	10460.35105	0.000236076	0.050548497
214.16	8651.549707	0.000195253	0.041815474
214.2	6058.483132	0.000136732	0.029287893
214.24	3683.627531	8.31343E-05	0.017810702
214.28	2017.875814	4.55406E-05	0.00975845
214.32	1043.901779	2.35594E-05	0.005049253
214.36	578.074108	1.30463E-05	0.002796611
214.4	395.417619	8.92402E-06	0.001913311

214.44	365.963865	8.25929E-06	0.001771123
214.48	432.181188	9.75373E-06	0.002091979
214.52	519.767884	1.17304E-05	0.002516413
214.56	552.559608	1.24705E-05	0.002675671
214.6	494.135773	1.1152E-05	0.00239321
214.64	373.68025	8.43344E-06	0.001810154
214.68	253.469911	5.72046E-06	0.001228069
214.72	178.819084	4.0357E-06	0.000866545
214.76	169.155246	3.8176E-06	0.000819867
214.8	230.763806	5.20802E-06	0.001118682
214.84	470.021554	1.06077E-05	0.002278965
214.88	1190.141113	2.68598E-05	0.00577164
214.92	3011.038547	6.79549E-05	0.014604877
214.96	6920.695191	0.00015619	0.033574699
215	12937.33459	0.000291978	0.062775189
215.04	19322.68856	0.000436086	0.093775969
215.08	23022.39247	0.000519583	0.111751983
215.12	22106.37241	0.00049891	0.107325526
215.16	17389.72949	0.000392462	0.084442118
215.2	11453.4013	0.000258487	0.05562647
215.24	6445.259602	0.000145461	0.031308925
215.28	3164.342403	7.14148E-05	0.015374178
215.32	1422.994382	3.2115E-05	0.006915002
215.36	663.046886	1.4964E-05	0.003222657
215.4	407.854864	9.20472E-06	0.001982696
215.44	371.60866	8.38669E-06	0.001806828
215.48	465.928602	1.05154E-05	0.00226585
215.52	572.071693	1.29109E-05	0.002782549
215.56	616.353998	1.39103E-05	0.002998494
215.6	566.915121	1.27945E-05	0.002758491
215.64	423.8977	9.56678E-06	0.002062981
215.68	293.284633	6.61903E-06	0.001427591
215.72	201.999772	4.55885E-06	0.000983436
215.76	161.046456	3.63459E-06	0.0007842
215.8	177.941292	4.01589E-06	0.000866628
215.84	268.927248	6.06931E-06	0.001310001
215.88	593.886566	1.34032E-05	0.002893481
215.92	1419.308722	3.20318E-05	0.006916311
215.96	3129.214198	7.0622E-05	0.015251528
216	5946.832286	0.000134212	0.028989732
216.04	9209.212984	0.000207839	0.044901561
216.08	11502.8309	0.000259603	0.056094988
216.12	11636.22079	0.000262613	0.056755985
216.16	9679.212997	0.000218446	0.047219364
216.2	6696.497143	0.000151131	0.032674439
216.24	3944.097488	8.90128E-05	0.019248125

216.28	2071.94671	4.6761E-05	0.010113459
216.32	1045.786617	2.36019E-05	0.005105573
216.36	570.640695	1.28786E-05	0.002786406
216.4	403.386185	9.10386E-06	0.001970076
216.44	408.454341	9.21825E-06	0.001995197
216.48	488.734955	1.10301E-05	0.002387789
216.52	599.405408	1.35277E-05	0.002929027
216.56	680.70037	1.53625E-05	0.003326894
216.6	627.889464	1.41706E-05	0.00306935
216.64	458.513416	1.0348E-05	0.002241793
216.68	300.786091	6.78832E-06	0.001470894
216.72	217.711019	4.91343E-06	0.00106484
216.76	232.57903	5.24898E-06	0.00113777
216.8	369.286819	8.33429E-06	0.001806874
216.84	778.369667	1.75667E-05	0.003809167
216.88	1839.086724	4.15056E-05	0.00900174
216.92	3997.874886	9.02265E-05	0.019571925
216.96	7425.653525	0.000167587	0.036359601
217	11698.9569	0.000264029	0.057294328
217.04	15747.50134	0.000355399	0.077135836
217.08	17875.21938	0.000403419	0.087574154
217.12	16960.24114	0.000382769	0.083106809
217.16	13531.42659	0.000305385	0.066317504
217.2	9099.083925	0.000205354	0.044602813
217.24	5191.451715	0.000117164	0.025452671
217.28	2578.113922	5.81844E-05	0.012642315
217.32	1187.362967	2.67971E-05	0.005823552
217.36	588.161958	1.3274E-05	0.002885236
217.4	389.242086	8.78465E-06	0.001909783
217.44	374.904392	8.46107E-06	0.001839775
217.48	448.562346	1.01234E-05	0.002201643
217.52	577.92703	1.3043E-05	0.002837115
217.56	695.401785	1.56942E-05	0.003414441
217.6	694.722793	1.56789E-05	0.003411734
217.64	575.990086	1.29993E-05	0.002829166
217.68	389.250909	8.78485E-06	0.001912286
217.72	248.064414	5.59847E-06	0.001218899
217.76	180.326878	4.06973E-06	0.000886224
217.8	181.381939	4.09354E-06	0.000891573
217.84	276.107751	6.23137E-06	0.001357441
217.88	606.679013	1.36919E-05	0.002983191
217.92	1457.004537	3.28826E-05	0.007165768
217.96	3165.208485	7.14343E-05	0.01556983
218	5768.65369	0.00013019	0.028381524
218.04	8661.359957	0.000195475	0.042621332
218.08	10682.00572	0.000241078	0.052574288

218.12	10835.205	0.000244535	0.05333808
218.16	8998.221522	0.000203077	0.044303349
218.2	6211.895035	0.000140194	0.030590291
218.24	3699.421845	8.34908E-05	0.018221032
218.28	1977.325729	4.46255E-05	0.009740851
218.32	1021.485329	2.30535E-05	0.00503304
218.36	606.2291	1.36817E-05	0.002987546
218.4	475.119449	1.07228E-05	0.002341856
218.44	488.574753	1.10265E-05	0.002408618
218.48	619.953379	1.39915E-05	0.003056859
218.52	768.917268	1.73534E-05	0.003792063
218.56	877.38612	1.98014E-05	0.00432779
218.6	815.051577	1.83946E-05	0.004021055
218.64	630.413694	1.42276E-05	0.003110713
218.68	426.783812	9.63192E-06	0.002106307
218.72	277.185845	6.2557E-06	0.001368246
218.76	233.300975	5.26528E-06	0.001151832
218.8	343.242371	7.7465E-06	0.001694935
218.84	717.524095	1.61935E-05	0.00354379
218.88	1711.578051	3.86279E-05	0.008454882
218.92	3851.292105	8.69183E-05	0.019028154
218.96	7495.325811	0.000169159	0.037039069
219	12494.53936	0.000281984	0.061754573
219.04	17497.55302	0.000394895	0.086497888
219.08	20355.245	0.00045939	0.100643058
219.12	19574.00038	0.000441758	0.096797996
219.16	15585.60972	0.000351746	0.077088544
219.2	10304.47592	0.000232558	0.050976641
219.24	5719.242773	0.000129075	0.028298478
219.28	2758.653931	6.2259E-05	0.013652148
219.32	1238.203656	2.79445E-05	0.006128795
219.36	597.046723	1.34745E-05	0.002955769
219.4	401.144667	9.05328E-06	0.001986289
219.44	395.208599	8.91931E-06	0.001957253
219.48	465.275383	1.05006E-05	0.002304675
219.52	557.9368	1.25919E-05	0.002764164
219.56	625.601007	1.41189E-05	0.003099955
219.6	585.449512	1.32128E-05	0.002901526
219.64	464.294493	1.04785E-05	0.002301493
219.68	319.032291	7.20011E-06	0.001581721
219.72	220.064158	4.96654E-06	0.001091249
219.76	192.004735	4.33328E-06	0.000952281
219.8	246.620804	5.56589E-06	0.001223382
219.84	440.097812	9.93239E-06	0.002183538
219.88	913.544951	2.06174E-05	0.004533362
219.92	1987.019294	4.48443E-05	0.009862149

219.96	3952.557037	8.92037E-05	0.019621247
220	6870.777992	0.000155064	0.034114056
220.04	10243.18855	0.000231174	0.050867637
220.08	12624.87006	0.000284926	0.062706455
220.12	12744.83401	0.000287633	0.063313809
220.16	10664.22668	0.000240677	0.052987392
220.2	7494.753159	0.000169146	0.03724598
220.24	4535.337623	0.000102356	0.022542941
220.28	2455.023973	5.54065E-05	0.012204938
220.32	1236.568727	2.79076E-05	0.00614861
220.36	661.347167	1.49257E-05	0.003289024
220.4	447.938273	1.01093E-05	0.002228099
220.44	468.362023	1.05703E-05	0.002330112
220.48	599.750497	1.35355E-05	0.002984314
220.52	748.478998	1.68921E-05	0.003725052
220.56	796.859631	1.7984E-05	0.003966554
220.6	715.568102	1.61494E-05	0.003562552
220.64	551.058197	1.24366E-05	0.002744015
220.68	392.223981	8.85195E-06	0.001953448
220.72	308.740334	6.96784E-06	0.001537941
220.76	314.136351	7.08962E-06	0.001565104
220.8	409.03363	9.23132E-06	0.002038275
220.84	716.158793	1.61627E-05	0.003569372
220.88	1580.475025	3.56691E-05	0.007878595
220.92	3501.566124	7.90255E-05	0.017458307
220.96	6965.780698	0.000157208	0.034736673
221	11803.45718	0.000266388	0.058871657
221.04	16571.19415	0.000373989	0.082666478
221.08	19201.01408	0.00043334	0.095802839
221.12	18332.09431	0.00041373	0.091483941
221.16	14637.46379	0.000330347	0.07305958
221.2	9830.292261	0.000221856	0.049074548
221.24	5589.670813	0.000126151	0.027909666
221.28	2771.175668	6.25416E-05	0.0138392
221.32	1265.171779	2.85532E-05	0.006319386
221.36	615.869378	1.38993E-05	0.003076752
221.4	416.506051	9.39996E-06	0.002081151
221.44	397.85512	8.97904E-06	0.001988318
221.48	459.056934	1.03603E-05	0.002294594
221.52	595.316133	1.34355E-05	0.002976222
221.56	672.406588	1.51753E-05	0.003362235
221.6	621.259022	1.4021E-05	0.003107043
221.64	484.137801	1.09263E-05	0.002421709
221.68	338.300774	7.63498E-06	0.001692522
221.72	246.247071	5.55745E-06	0.001232199
221.76	219.758453	4.95964E-06	0.00109985

221.8	276.825325	6.24756E-06	0.001385709
221.84	472.956609	1.0674E-05	0.002367914
221.88	1007.420351	2.27361E-05	0.00504468
221.92	2272.48063	5.12867E-05	0.011381549
221.96	4778.02147	0.000107833	0.023934677
222	8646.302073	0.000195135	0.043319969
222.04	12998.02859	0.000293347	0.065134857
222.08	15792.51607	0.000356415	0.079152663
222.12	15564.39921	0.000351267	0.078023384
222.16	12654.97007	0.000285605	0.063450017
222.2	8605.116094	0.000194205	0.043152459
222.24	5001.160154	0.000112869	0.025084055
222.28	2564.528689	5.78778E-05	0.012865086
222.32	1234.167238	2.78534E-05	0.006192376
222.36	642.626238	1.45032E-05	0.003224927
222.399999	456.763307	1.03085E-05	0.002292613
222.439999	455.294583	1.02754E-05	0.002285652
222.479999	550.856735	1.24321E-05	0.002765887
222.519999	711.42022	1.60558E-05	0.003572728
222.559999	787.252699	1.77672E-05	0.003954267
222.599999	690.047292	1.55734E-05	0.00346664
222.639999	515.018373	1.16232E-05	0.0025878
222.679999	345.455785	7.79646E-06	0.001736115
222.719999	256.726528	5.79396E-06	0.001290431
222.759999	265.57532	5.99367E-06	0.001335149
222.799999	359.756667	8.11921E-06	0.001808959
222.839999	649.019125	1.46475E-05	0.003264039
222.879999	1522.533289	3.43615E-05	0.007658481
222.919999	3590.91921	8.1042E-05	0.018065893
222.959999	7462.803093	0.000168425	0.037552054
222.999999	13181.79898	0.000297495	0.06634135
223.039999	19033.93702	0.000429569	0.095811169
223.079999	22441.39358	0.000506471	0.112983549
223.119999	21876.99256	0.000493733	0.110161762
223.159999	17682.40725	0.000399067	0.089055859
223.199999	11993.96795	0.000270687	0.060417372
223.239999	6875.190866	0.000155163	0.034638695
223.279999	3384.380077	7.63807E-05	0.017054293
223.319999	1512.49283	3.41349E-05	0.007622996
223.359999	707.638505	1.59704E-05	0.003567152
223.399999	440.626917	9.94434E-06	0.002221565
223.439999	422.239066	9.52935E-06	0.002129237
223.479999	500.151048	1.12877E-05	0.002522578
223.519999	599.017628	1.3519E-05	0.003021765
223.559999	665.891117	1.50282E-05	0.003359712
223.599999	607.356353	1.37072E-05	0.003064927

223.639999	468.035105	1.05629E-05	0.002362287
223.679999	323.669719	7.30477E-06	0.001633932
223.719999	220.717398	4.98128E-06	0.001114413
223.759999	195.095136	4.40303E-06	0.000985221
223.799999	270.359499	6.10164E-06	0.001365546
223.839999	515.229497	1.1628E-05	0.002602814
223.879999	1090.490059	2.46108E-05	0.005509875
223.919999	2259.487613	5.09935E-05	0.011418462
223.959999	4315.279272	9.73898E-05	0.021811429
223.999999	7351.928647	0.000165923	0.037166705
224.039999	10757.43945	0.00024278	0.054392526
224.079999	13215.37668	0.000298253	0.066832452
224.119999	13257.14831	0.000299195	0.067055666
224.159999	10950.68674	0.000247142	0.055399294
224.199999	7606.853326	0.000171676	0.038489778
224.239999	4492.015834	0.000101379	0.022733124
224.279999	2355.880408	5.31689E-05	0.01192473
224.319999	1193.678533	2.69397E-05	0.006043105
224.359999	670.404977	1.51301E-05	0.003394591
224.399999	492.263054	1.11097E-05	0.002493015
224.439999	502.074058	1.13311E-05	0.002543155
224.479999	639.826648	1.444E-05	0.00324149
224.519999	820.012302	1.85065E-05	0.004155087
224.559999	894.212575	2.01811E-05	0.004531875
224.599999	776.108767	1.75157E-05	0.003934025
224.639999	548.180011	1.23717E-05	0.00277917
224.679999	341.377177	7.70441E-06	0.001731026
224.719999	232.007968	5.2361E-06	0.001176656
224.759999	208.214443	4.69911E-06	0.001056172
224.799999	268.811065	6.06669E-06	0.001363792
224.839999	509.744022	1.15042E-05	0.002586607
224.879999	1213.729336	2.73922E-05	0.006159954
224.919999	2973.137549	6.70996E-05	0.015092036
224.959999	6514.342828	0.00014702	0.033073539
224.999999	11952.71177	0.000269756	0.060695112
225.039999	17940.9291	0.000404902	0.091119096
225.079999	22076.8124	0.000498243	0.112144513
225.119999	22323.9201	0.00050382	0.113419909
225.159999	18765.64264	0.000423514	0.095358505
225.199999	13218.93211	0.000298333	0.067184565
225.239999	7853.746473	0.000177248	0.039923366
225.279999	4030.837636	9.09704E-05	0.02049381
225.319999	1835.826108	4.1432E-05	0.009335467
225.359999	843.24756	1.90309E-05	0.004288809
225.399999	529.34274	1.19465E-05	0.002692747
225.439999	504.231361	1.13798E-05	0.002565462

225.479999	586.582039	1.32383E-05	0.002984981
225.519999	693.579334	1.56531E-05	0.003530091
225.559999	766.571706	1.73005E-05	0.003902291
225.599999	708.509698	1.59901E-05	0.003607361
225.639999	531.253807	1.19897E-05	0.002705347
225.679999	355.971025	8.03377E-06	0.001813061
225.719999	260.963968	5.88959E-06	0.001329399
225.759999	244.418354	5.51618E-06	0.001245333
225.799999	292.529264	6.60198E-06	0.001490727
225.839999	460.509791	1.03931E-05	0.00234717
225.879999	911.53271	2.0572E-05	0.004646809
225.919999	1965.558923	4.43599E-05	0.010021795
225.959999	3868.604182	8.7309E-05	0.019728343
225.999999	6742.646255	0.000152172	0.034390902
226.039999	10242.98927	0.00023117	0.052253667
226.079999	12962.14513	0.000292538	0.066136891
226.119999	13510.98516	0.000304924	0.068949441
226.159999	11708.03109	0.000264234	0.059759151
226.199999	8587.876902	0.000193816	0.043841274
226.239999	5405.660837	0.000121998	0.027600881
226.279999	2959.745925	6.67973E-05	0.015114903
226.319999	1456.059535	3.28612E-05	0.007437155
226.359999	718.47185	1.62149E-05	0.003670407
226.399999	476.927399	1.07636E-05	0.002436876
226.439999	463.020835	1.04497E-05	0.002366238
226.479999	562.319916	1.26908E-05	0.002874207
226.519999	701.152643	1.5824E-05	0.003584461
226.559999	757.689369	1.71E-05	0.003874175
226.599999	692.84747	1.56366E-05	0.003543254
226.639999	550.581247	1.24259E-05	0.002816195
226.679999	382.560236	8.63385E-06	0.001957121
226.719999	256.130005	5.7805E-06	0.001310554
226.759999	189.549684	4.27787E-06	0.00097005
226.799999	196.460908	4.43385E-06	0.001005597
226.839999	329.935563	7.44619E-06	0.001689093
226.879999	788.832632	1.78029E-05	0.004039111
226.919999	2049.537032	4.62552E-05	0.010496229
226.959999	4852.772936	0.00010952	0.024856733
226.999999	9691.961062	0.000218734	0.049652632
227.039999	15632.09591	0.000352795	0.080098493
227.079999	20548.62946	0.000463754	0.105309245
227.119999	22291.87401	0.000503097	0.114263287
227.159999	20209.9593	0.000456111	0.103610091
227.199999	15472.37594	0.00034919	0.079335962
227.239999	10099.12618	0.000227923	0.05179327
227.279999	5687.71566	0.000128364	0.029174529

227.319999	2829.513007	6.38582E-05	0.014516239
227.359999	1311.354469	2.95954E-05	0.00672882
227.399999	677.519037	1.52907E-05	0.003477096
227.439999	486.872634	1.0988E-05	0.002499119
227.479999	495.168665	1.11753E-05	0.00254215
227.519999	595.537447	1.34405E-05	0.003057971
227.559999	682.63895	1.54062E-05	0.003505837
227.599999	653.684787	1.47528E-05	0.003357727
227.639999	508.052303	1.1466E-05	0.002610128
227.679999	331.595826	7.48366E-06	0.001703879
227.719999	224.789907	5.0732E-06	0.001155268
227.759999	197.3442	4.45378E-06	0.001014394
227.799999	230.17902	5.19482E-06	0.00118338
227.839999	361.049159	8.14838E-06	0.001856526
227.879999	717.366511	1.619E-05	0.003689369
227.919999	1516.036413	3.42148E-05	0.007798244
227.959999	3117.512793	7.03579E-05	0.016038792
227.999999	5695.198905	0.000128533	0.029305457
228.039999	8848.469544	0.000199698	0.045539048
228.079999	11536.4751	0.000260362	0.059383404
228.119999	12596.77355	0.000284292	0.064852607
228.159999	11895.33969	0.000268461	0.061252118
228.199999	9911.773127	0.000223695	0.051047179
228.239999	7316.766521	0.000165129	0.037689095
228.279999	4846.932963	0.000109389	0.024971213
228.319999	2864.289577	6.4643E-05	0.014759296
228.359999	1555.653508	3.51089E-05	0.008017476
228.399999	851.813015	1.92242E-05	0.004390815
228.439999	551.081362	1.24371E-05	0.00284114
228.479999	498.293993	1.12458E-05	0.002569441
228.519999	568.421037	1.28285E-05	0.002931562
228.559999	617.443473	1.39348E-05	0.003184947
228.599999	570.305511	1.2871E-05	0.002942311
228.639999	456.507491	1.03027E-05	0.002355618
228.679999	328.515685	7.41414E-06	0.001695466
228.719999	219.460668	4.95292E-06	0.001132832
228.759999	169.661278	3.82902E-06	0.000875926
228.799999	185.544301	4.18748E-06	0.000958095
228.839999	321.448364	7.25464E-06	0.001660152
228.879999	785.609933	1.77301E-05	0.00405807
228.919999	2000.722268	4.51535E-05	0.010336543
228.959999	4498.084523	0.000101516	0.023242989
228.999999	8410.112366	0.000189805	0.043465235
229.039999	12922.49857	0.000291643	0.066797865
229.079999	16199.85842	0.000365608	0.083753535
229.119999	16709.92912	0.00037712	0.086405694

229.159999	14314.59417	0.00032306	0.074032533
229.199999	10361.06651	0.000233835	0.053594945
229.239999	6404.865876	0.000144549	0.03313639
229.279999	3438.790002	7.76087E-05	0.017794123
229.319999	1682.078454	3.79622E-05	0.008705484
229.359999	830.381774	1.87406E-05	0.004298335
229.399999	497.6372	1.1231E-05	0.002576386
229.439999	445.790868	1.00609E-05	0.002308368
229.479999	532.305478	1.20134E-05	0.002756833
229.519999	611.221773	1.37944E-05	0.003166096
229.559999	631.579541	1.42539E-05	0.003272119
229.599999	553.980917	1.25026E-05	0.002870592
229.639999	425.542996	9.60391E-06	0.002205442
229.679999	302.346616	6.82354E-06	0.001567231
229.719999	216.792646	4.89271E-06	0.001123953
229.759999	169.269539	3.82018E-06	0.000877724
229.799999	176.745904	3.98891E-06	0.000916651
229.839999	272.096352	6.14084E-06	0.00141141
229.879999	600.795963	1.35591E-05	0.003116972
229.919999	1502.402395	3.39071E-05	0.007795927
229.959999	3406.474927	7.68794E-05	0.017679185
229.999999	6383.823612	0.000144074	0.033137021
230.039999	9590.261688	0.000216439	0.049789592
230.079999	11754.73436	0.000265288	0.061037458
230.119999	11882.05439	0.000268161	0.061709305
230.159999	9842.024497	0.000222121	0.051123319
230.199999	6795.556551	0.000153366	0.03530491
230.239999	4036.89775	9.11072E-05	0.020976512
230.279999	2142.8247	4.83606E-05	0.011136472
230.319999	1108.5169	2.50177E-05	0.005762073
230.359999	638.687778	1.44143E-05	0.003320477
230.399999	471.827589	1.06485E-05	0.002453412
230.439999	498.885732	1.12592E-05	0.00259456
230.479999	615.047729	1.38808E-05	0.00319924
230.519999	682.225381	1.53969E-05	0.003549288
230.559999	663.077755	1.49647E-05	0.003450271
230.599999	571.31192	1.28937E-05	0.00297329
230.639999	428.528353	9.67129E-06	0.002230586
230.679999	291.103531	6.5698E-06	0.001515522
230.719999	214.31854	4.83687E-06	0.001115963
230.759999	194.702148	4.39416E-06	0.001013995
230.799999	250.759489	5.65929E-06	0.001306165
230.839999	483.668125	1.09157E-05	0.002519784
230.879999	1141.016415	2.57512E-05	0.005945426
230.919999	2658.488443	5.99984E-05	0.013854826
230.959999	5611.975751	0.000126654	0.029252118

230.999999	9805.888838	0.000221305	0.051121513
231.039999	14087.26591	0.00031793	0.07345454
231.079999	16595.75446	0.000374543	0.08654941
231.119999	16128.73466	0.000364003	0.084128391
231.159999	13041.5001	0.000294328	0.068036974
231.199999	8856.530967	0.00019988	0.046212156
231.239999	5160.801346	0.000116472	0.026933008
231.279999	2642.471906	5.96369E-05	0.013792825
231.319999	1259.759958	2.8431E-05	0.006576665
231.359999	655.653266	1.47972E-05	0.003423476
231.399999	458.988928	1.03587E-05	0.002397013
231.439999	436.303669	9.84677E-06	0.002278936
231.479999	500.900089	1.13046E-05	0.002616793
231.519999	598.785684	1.35138E-05	0.003128705
231.559999	633.075608	1.42876E-05	0.003308445
231.599999	570.783014	1.28818E-05	0.00298342
231.639999	433.404348	9.78133E-06	0.002265748
231.679999	294.258907	6.64101E-06	0.00153859
231.719999	199.969646	4.51304E-06	0.001045761
231.759999	171.765601	3.87651E-06	0.00089842
231.799999	227.382302	5.1317E-06	0.001189528
231.839999	392.003261	8.84697E-06	0.002051081
231.879999	806.374315	1.81987E-05	0.004219925
231.919999	1715.326951	3.87125E-05	0.008978212
231.959999	3474.689193	7.84189E-05	0.018190047
231.999999	6172.214307	0.000139298	0.032317201
232.039999	9035.321409	0.000203915	0.047316348
232.079999	10785.05504	0.000243404	0.056489123
232.119999	10618.32485	0.000239641	0.055625423
232.159999	8697.692098	0.000196295	0.0455718
232.199999	5959.627198	0.0001345	0.031231012
232.239999	3503.870297	7.90775E-05	0.018364952
232.279999	1853.366187	4.18279E-05	0.009715783
232.319999	961.47616	2.16992E-05	0.005041153
232.359999	589.267546	1.32989E-05	0.003090143
232.399999	508.220075	1.14698E-05	0.002665586
232.439999	598.06609	1.34975E-05	0.003137363
232.479999	799.628147	1.80465E-05	0.004195449
232.519999	976.808453	2.20452E-05	0.005125951
232.559999	1019.220701	2.30024E-05	0.005349436
232.599999	862.661119	1.94691E-05	0.004528503
232.639999	590.577857	1.33285E-05	0.003100747
232.679999	363.301946	8.19922E-06	0.001907794
232.719999	258.96022	5.84437E-06	0.001360102
232.759999	257.096871	5.80232E-06	0.001350548
232.799999	327.34444	7.38771E-06	0.001719858

232.839999	549.888199	1.24102E-05	0.002889593
232.879999	1136.673693	2.56531E-05	0.005974104
232.919999	2548.635131	5.75191E-05	0.013397359
232.959999	5355.646814	0.000120869	0.028157756
232.999999	9487.71125	0.000214124	0.049890991
233.039999	13782.87072	0.00031106	0.072489467
233.079999	16449.35546	0.000371239	0.086528395
233.119999	16298.87447	0.000367843	0.085751535
233.159999	13397.3596	0.00030236	0.070498198
233.199999	9212.787144	0.00020792	0.048486891
233.239999	5392.354526	0.000121698	0.028384825
233.279999	2758.300006	6.2251E-05	0.014521911
233.319999	1325.395103	2.99123E-05	0.006979143
233.359999	690.942193	1.55936E-05	0.003638923
233.399999	467.347936	1.05474E-05	0.002461761
233.439999	462.044617	1.04277E-05	0.002434243
233.479999	581.567798	1.31252E-05	0.003064466
233.519999	754.544841	1.7029E-05	0.003976618
233.559999	839.344915	1.89428E-05	0.004424291
233.599999	756.219978	1.70668E-05	0.003986812
233.639999	569.500511	1.28528E-05	0.003002936
233.679999	401.07653	9.05174E-06	0.00211521
233.719999	275.850628	6.22556E-06	0.001455039
233.759999	199.451582	4.50134E-06	0.001052234
233.799999	196.877805	4.44326E-06	0.001038834
233.839999	313.759326	7.08111E-06	0.001655847
233.879999	646.587479	1.45926E-05	0.003412912
233.919999	1447.170873	3.26606E-05	0.007639975
233.959999	3082.716991	6.95726E-05	0.016277212
233.999999	5639.511564	0.000127276	0.029782565
234.039999	8369.154287	0.00018888	0.044205511
234.079999	10202.00772	0.000230245	0.053895774
234.119999	10251.8384	0.00023137	0.054168277
234.159999	8502.908711	0.000191899	0.044935024
234.199999	5931.47053	0.000133865	0.031351189
234.239999	3537.2977	7.98319E-05	0.01869982
234.279999	1864.785493	4.20856E-05	0.009859817
234.319999	949.356682	2.14257E-05	0.00502046
234.359999	550.980903	1.24349E-05	0.002914236
234.399999	420.723415	9.49514E-06	0.002225661
234.439999	417.405393	9.42026E-06	0.002208485
234.479999	472.094877	1.06545E-05	0.002498273
234.519999	583.516051	1.31691E-05	0.003088428
234.559999	674.861013	1.52307E-05	0.003572507
234.599999	622.169292	1.40415E-05	0.003294135
234.639999	483.428913	1.09103E-05	0.002559997

234.679999	353.28451	7.97314E-06	0.001871136
234.719999	282.089471	6.36637E-06	0.001494314
234.759999	306.574727	6.91896E-06	0.001624296
234.799999	500.330113	1.12918E-05	0.002651304
234.839999	952.172428	2.14892E-05	0.005046525
234.879999	1880.843057	4.2448E-05	0.009970188
234.919999	3632.635685	8.19835E-05	0.01925957
234.959999	6598.725941	0.000148924	0.034991195
234.999999	10876.72873	0.000245473	0.057686065
235.039999	15519.53286	0.000350254	0.082323756
235.079999	18665.67428	0.000421258	0.099029394
235.119999	18837.13166	0.000425128	0.099956053
235.159999	16156.89447	0.000364639	0.085748414
235.199999	11745.302	0.000265075	0.062345665
235.239999	7272.872673	0.000164139	0.038611966
235.279999	3894.083219	8.7884E-05	0.020677355
235.319999	1843.046744	4.1595E-05	0.009788135
235.359999	882.452793	1.99157E-05	0.004687366
235.399999	562.612214	1.26974E-05	0.002988962
235.439999	499.070386	1.12633E-05	0.002651837
235.479999	528.079909	1.1918E-05	0.002806457
235.519999	602.441082	1.35963E-05	0.00320219
235.559999	656.760167	1.48222E-05	0.003491509
235.599999	615.524593	1.38915E-05	0.003272845
235.639999	496.888097	1.12141E-05	0.002642484
235.679999	362.02346	8.17037E-06	0.001925592
235.719999	274.377671	6.19232E-06	0.001459654
235.759999	254.27155	5.73855E-06	0.001352922
235.799999	350.52655	7.9109E-06	0.001865389
235.839999	587.156412	1.32513E-05	0.003125187
235.879999	1120.693592	2.52925E-05	0.005965993
235.919999	2179.154169	4.91805E-05	0.011602657
235.959999	3930.857685	8.8714E-05	0.020932951
235.999999	6456.539649	0.000145715	0.034388765
236.039999	9319.917867	0.000210338	0.049648079
236.079999	11213.8875	0.000253082	0.059747556
236.119999	11202.37455	0.000252822	0.059696328
236.159999	9473.482464	0.000213803	0.050491788
236.199999	6823.505633	0.000153997	0.036374095
236.239999	4189.251735	9.45456E-05	0.022335447
236.279999	2250.206752	5.0784E-05	0.011999251
236.319999	1128.632554	2.54717E-05	0.006019464
236.359999	600.878762	1.3561E-05	0.003205277
236.399999	405.291822	9.14687E-06	0.002162321
236.439999	368.575232	8.31823E-06	0.001966762
236.479999	397.381299	8.96834E-06	0.002120834

236.519999	496.281838	1.12004E-05	0.002649116
236.559999	568.915384	1.28396E-05	0.003037342
236.599999	556.809006	1.25664E-05	0.002973211
236.639999	478.506732	1.07992E-05	0.00255553
236.679999	371.75499	8.38999E-06	0.001985743
236.719999	298.898422	6.74572E-06	0.001596847
236.759999	308.709929	6.96715E-06	0.001649543
236.799999	487.396461	1.09999E-05	0.002604767
236.839999	920.619965	2.07771E-05	0.004920851
236.879999	1738.099984	3.92265E-05	0.009291972
236.919999	3163.853787	7.14038E-05	0.016916982
236.959999	5626.690394	0.000126987	0.030090736
236.999999	9449.8599	0.00021327	0.050545031
237.039999	13869.92319	0.000313025	0.074199409
237.079999	17059.02485	0.000384998	0.091275427
237.119999	17543.55877	0.000395934	0.093883795
237.159999	15064.64678	0.000339988	0.080631576
237.199999	10804.03648	0.000243832	0.057836963
237.239999	6585.879977	0.000148634	0.035261963
237.279999	3473.259968	7.83866E-05	0.018599582
237.319999	1649.505926	3.72271E-05	0.008834724
237.359999	796.490892	1.79757E-05	0.00426671
237.399999	498.841834	1.12582E-05	0.002672688
237.439999	424.130792	9.57204E-06	0.002272785
237.479999	447.15492	1.00917E-05	0.002396568
237.519999	529.091013	1.19408E-05	0.00283619
237.559999	599.403563	1.35277E-05	0.003213641
237.599999	591.721253	1.33543E-05	0.003172987
237.639999	485.926274	1.09667E-05	0.002606122
237.679999	352.225207	7.94923E-06	0.001889374
237.719999	261.290482	5.89696E-06	0.001401826
237.759999	234.152032	5.28449E-06	0.001256439
237.799999	274.850676	6.203E-06	0.001475073
237.839999	450.587332	1.01691E-05	0.002418626
237.879999	904.130831	2.0405E-05	0.004853935
237.919999	1912.969624	4.31731E-05	0.010271734
237.959999	3878.660024	8.7536E-05	0.020830056
237.999999	6908.170104	0.000155908	0.037106051
238.039999	10350.05268	0.000233586	0.055602876
238.079999	12733.84063	0.000287385	0.068420632
238.119999	12694.68945	0.000286501	0.068221727
238.159999	10438.51561	0.000235583	0.056106389
238.199999	7169.633945	0.000161809	0.038542821
238.239999	4209.295586	9.49979E-05	0.022632309
238.279999	2204.250527	4.97469E-05	0.011853683
238.319999	1094.394742	2.4699E-05	0.005886257

238.359999	593.57327	1.33961E-05	0.0031931
238.399999	435.251895	9.82303E-06	0.00234181
238.439999	428.634432	9.67368E-06	0.002306593
238.479999	468.625069	1.05762E-05	0.002522216
238.519999	516.713424	1.16615E-05	0.002781502
238.559999	539.640703	1.21789E-05	0.002905408
238.599999	511.12512	1.15354E-05	0.002752342
238.639999	407.926529	9.20633E-06	0.002196999
238.679999	293.47934	6.62342E-06	0.001580878
238.719999	207.092228	4.67378E-06	0.001115726
238.759999	176.993336	3.99449E-06	0.000953725
238.799999	213.616781	4.82103E-06	0.001151263
238.839999	370.082605	8.35225E-06	0.001994851
238.879999	860.083982	1.94109E-05	0.004636875
238.919999	2017.814557	4.55393E-05	0.010880241
238.959999	4347.093189	9.81078E-05	0.023443849
238.999999	8078.869452	0.000182329	0.04357659
239.039999	12418.25447	0.000280263	0.066993997
239.079999	15702.33794	0.00035438	0.084725145
239.119999	16332.04651	0.000368592	0.088137609
239.159999	14026.86253	0.000316567	0.075710103
239.199999	10128.24983	0.00022858	0.054676453
239.239999	6279.84868	0.000141727	0.033906872
239.279999	3364.769566	7.59382E-05	0.018170483
239.319999	1608.799614	3.63084E-05	0.008689318
239.359999	778.140667	1.75616E-05	0.004203533
239.399999	472.005261	1.06525E-05	0.002550209
239.439999	415.760824	9.38314E-06	0.0022467
239.479999	480.26466	1.08389E-05	0.002595701
239.519999	576.801565	1.30176E-05	0.003117977
239.559999	588.347621	1.32782E-05	0.003180922
239.599999	509.501399	1.14987E-05	0.002755097
239.639999	394.101067	8.89431E-06	0.002131433
239.679999	275.779301	6.22395E-06	0.001491757
239.719999	197.734114	4.46258E-06	0.001069771
239.759999	164.361434	3.70941E-06	0.000889368
239.799999	174.824344	3.94554E-06	0.000946141
239.839999	288.271203	6.50588E-06	0.00156037
239.879999	622.687204	1.40532E-05	0.003371077
239.919999	1424.483264	3.21486E-05	0.007713093
239.959999	3052.67566	6.88946E-05	0.016531957
239.999999	5709.593685	0.000128858	0.030925818
240.039999	8784.713167	0.000198259	0.047590028
240.079999	10967.42216	0.000247519	0.059424467
240.119999	11244.74033	0.000253778	0.060937204
240.159999	9520.576575	0.000214866	0.051602255

240.199999	6712.320383	0.000151488	0.036387351
240.239999	4053.816624	9.1489E-05	0.021979316
240.279999	2205.678879	4.97791E-05	0.011960922
240.319999	1146.634399	2.58779E-05	0.006218987
240.359999	631.584162	1.4254E-05	0.003426085
240.399999	439.771316	9.92503E-06	0.002385976
240.439999	428.782432	9.67702E-06	0.002326743
240.479999	497.109428	1.12191E-05	0.002697961
240.519999	549.840132	1.24091E-05	0.002984643
240.559999	550.049086	1.24138E-05	0.002986274
240.599999	490.676748	1.10739E-05	0.002664378
240.639999	402.886162	9.09258E-06	0.002188038
240.679999	315.702187	7.12496E-06	0.001714835
240.719999	278.378076	6.28261E-06	0.001512349
240.759999	341.519469	7.70762E-06	0.001855686
240.799999	588.903871	1.32907E-05	0.00320041
240.839999	1255.012625	2.83239E-05	0.006821525
240.879999	2706.212313	6.10754E-05	0.014711853
240.919999	5262.104122	0.000118758	0.028611264
240.959999	8925.638404	0.000201439	0.048538796
240.999999	13208.15388	0.00029809	0.071839602
241.039999	17118.00336	0.000386329	0.09312086
241.079999	19202.37784	0.000433371	0.104477061
241.119999	18422.48114	0.00041577	0.100250399
241.159999	15296.21581	0.000345214	0.083251877
241.199999	11081.96797	0.000250105	0.060325225
241.239999	7044.402429	0.000158982	0.038352902
241.279999	3967.317233	8.95368E-05	0.021603445
241.319999	2019.45367	4.55763E-05	0.010998462
241.359999	1009.553072	2.27842E-05	0.005499196
241.399999	592.696022	1.33763E-05	0.003229045
241.439999	495.021434	1.11719E-05	0.002697354
241.479999	568.405061	1.28281E-05	0.003097732
241.519999	713.49279	1.61025E-05	0.003889085
241.559999	799.352233	1.80403E-05	0.004357807
241.599999	712.756508	1.60859E-05	0.003886359
241.639999	534.925356	1.20725E-05	0.002917204
241.679999	374.502887	8.45201E-06	0.002042681
241.719999	261.395943	5.89934E-06	0.001425989
241.759999	224.041565	5.05631E-06	0.001222413
241.799999	270.614573	6.10739E-06	0.001476768
241.839999	437.792248	9.88036E-06	0.002389467
241.879999	872.798034	1.96978E-05	0.004764513
241.919999	1781.595025	4.02081E-05	0.009727148
241.959999	3448.346789	7.78244E-05	0.018830388
241.999999	5997.009249	0.000135344	0.032753283

242.039999	8883.740954	0.000200494	0.048527485
242.079999	10872.44657	0.000245376	0.059400616
242.119999	11102.30874	0.000250564	0.060666469
242.159999	9503.640013	0.000214484	0.051939425
242.199999	6932.955099	0.000156467	0.037896341
242.239999	4395.617056	9.9203E-05	0.024030924
242.279999	2519.95643	5.68719E-05	0.013778925
242.319999	1379.123264	3.11249E-05	0.007542184
242.359999	780.251383	1.76092E-05	0.004267763
242.399999	508.377871	1.14734E-05	0.002781147
242.439999	423.028222	9.54716E-06	0.002314613
242.479999	459.564531	1.03717E-05	0.002514937
242.519999	552.94111	1.24791E-05	0.003026434
242.559999	607.314769	1.37062E-05	0.003324588
242.599999	573.321116	1.29391E-05	0.003139016
242.639999	475.697659	1.07358E-05	0.002604943
242.679999	347.97117	7.85322E-06	0.001905821
242.719999	248.829532	5.61574E-06	0.001363051
242.759999	231.745667	5.23018E-06	0.001269678
242.799999	295.935736	6.67886E-06	0.001621627
242.839999	531.197264	1.19884E-05	0.002911259
242.879999	1173.67414	2.64882E-05	0.006433452
242.919999	2647.762556	5.97563E-05	0.014516003
242.959999	5471.190224	0.000123477	0.030000006
242.999999	9707.45048	0.000219084	0.053237323
243.039999	14339.42113	0.000323621	0.07865279
243.079999	17218.23508	0.000388592	0.094458842
243.119999	17015.88844	0.000384025	0.093364134
243.159999	14197.2006	0.000320411	0.077911149
243.199999	10101.04409	0.000227966	0.055441451
243.239999	6152.129942	0.000138845	0.033772659
243.279999	3288.770647	7.4223E-05	0.018056965
243.319999	1618.897843	3.65363E-05	0.008890005
243.359999	818.1427	1.84643E-05	0.004493482
243.399999	501.131425	1.13098E-05	0.002752815
243.439999	421.891376	9.5215E-06	0.002317914
243.479999	481.488409	1.08665E-05	0.002645781
243.519999	598.146541	1.34993E-05	0.003287358
243.559999	717.840645	1.62007E-05	0.003945834
243.599999	718.394376	1.62132E-05	0.003949526
243.639999	591.5044	1.33494E-05	0.003252455
243.679999	431.584614	9.74026E-06	0.002373507
243.719999	281.341504	6.34949E-06	0.001547497
243.759999	196.906836	4.44391E-06	0.001083248
243.799999	189.7567	4.28254E-06	0.001044084
243.839999	262.385453	5.92167E-06	0.001443941

243.879999	518.465982	1.17011E-05	0.002853653
243.919999	1211.724906	2.73469E-05	0.006670466
243.959999	2702.597898	6.09939E-05	0.014880065
243.999999	5156.88127	0.000116384	0.028397605
244.039999	8033.085414	0.000181296	0.044243366
244.079999	10178.28802	0.00022971	0.056067564
244.119999	10446.59896	0.000235765	0.057554997
244.159999	8827.382427	0.000199222	0.048641976
244.199999	6258.897971	0.000141255	0.034494375
244.239999	3807.798346	8.59367E-05	0.020989181
244.279999	2078.98704	4.69198E-05	0.011461579
244.319999	1076.286208	2.42903E-05	0.005934601
244.359999	601.345568	1.35715E-05	0.00331634
244.399999	424.007411	9.56926E-06	0.002338726
244.439999	382.714852	8.63734E-06	0.002111312
244.479999	423.565369	9.55928E-06	0.002337053
244.519999	543.488281	1.22658E-05	0.002999227
244.559999	633.386013	1.42946E-05	0.003495897
244.599999	630.85746	1.42376E-05	0.003482511
244.639999	528.388924	1.1925E-05	0.002917332
244.679999	405.003422	9.14036E-06	0.002236464
244.719999	316.743264	7.14845E-06	0.00174937
244.759999	332.163954	7.49648E-06	0.001834838
244.799999	572.367133	1.29175E-05	0.003162211
244.839999	1222.185878	2.7583E-05	0.00675343
244.879999	2517.659466	5.68201E-05	0.013914098
244.919999	4688.902279	0.000105822	0.025917923
244.959999	7853.850918	0.00017725	0.043419276
244.999999	11770.00227	0.000265633	0.065079976
245.039999	15547.36633	0.000350882	0.085980224
245.079999	17788.26566	0.000401456	0.098388929
245.119999	17455.62636	0.000393949	0.096564821
245.159999	14620.922	0.000329974	0.080896387
245.199999	10546.15514	0.000238012	0.05836055
245.239999	6569.027177	0.000148254	0.03635776
245.279999	3588.876628	8.09959E-05	0.019866686
245.319999	1752.695858	3.95559E-05	0.009703854
245.359999	833.076315	1.88014E-05	0.004613105
245.399999	487.099718	1.09932E-05	0.002697722
245.439999	404.713528	9.13382E-06	0.002241805
245.479999	435.03146	9.81805E-06	0.002410136
245.519999	509.902803	1.15078E-05	0.002825394
245.559999	538.938061	1.21631E-05	0.002986766
245.599999	498.255072	1.12449E-05	0.002761753
245.639999	401.209112	9.05473E-06	0.002224204
245.679999	293.825637	6.63124E-06	0.001629162

245.719999	214.098112	4.8319E-06	0.001187294
245.759999	180.832191	4.08113E-06	0.001002979
245.799999	205.275335	4.63278E-06	0.001138737
245.839999	325.428186	7.34446E-06	0.001805562
245.879999	654.710092	1.47759E-05	0.003633097
245.919999	1424.640457	3.21522E-05	0.007906857
245.959999	2906.733727	6.56009E-05	0.016135205
245.999999	5206.468911	0.000117503	0.028905677
246.039999	7832.498561	0.000176769	0.043492142
246.079999	9698.919226	0.000218891	0.053864721
246.119999	9982.694583	0.000225296	0.055449731
246.159999	8588.743923	0.000193836	0.047714666
246.199999	6225.305229	0.000140496	0.03459023
246.239999	3854.5379	8.69916E-05	0.0214208
246.279999	2084.205332	4.70376E-05	0.011584423
246.319999	1067.038523	2.40816E-05	0.005931773
246.359999	586.708077	1.32412E-05	0.003262098
246.399999	400.397916	9.03642E-06	0.002226575
246.439999	364.10433	8.21733E-06	0.002025078
246.479999	415.431165	9.3757E-06	0.002310923
246.519999	507.865195	1.14618E-05	0.002825565
246.559999	586.651768	1.32399E-05	0.003264433
246.599999	569.938898	1.28627E-05	0.003171948
246.639999	473.160542	1.06786E-05	0.002633764
246.679999	351.941141	7.94282E-06	0.001959335
246.719999	255.235936	5.76032E-06	0.001421186
246.759999	235.797105	5.32161E-06	0.001313161
246.799999	312.302896	7.04824E-06	0.001739506
246.839999	567.534761	1.28085E-05	0.003161642
246.879999	1268.236933	2.86223E-05	0.007066284
246.919999	2901.860677	6.5491E-05	0.016171026
246.959999	5936.574858	0.00013398	0.033087756
246.999999	10334.43946	0.000233234	0.057608774
247.039999	14911.82958	0.000336539	0.083138649
247.079999	17700.86132	0.000399484	0.098704453
247.119999	17308.03673	0.000390618	0.096529589
247.159999	14108.43571	0.000318408	0.078697657
247.199999	9717.292221	0.000219306	0.054212381
247.239999	5731.591565	0.000129354	0.031981492
247.279999	2960.398925	6.68121E-05	0.016521291
247.319999	1389.839792	3.13667E-05	0.007757624
247.359999	677.443431	1.5289E-05	0.003781876
247.399999	437.051861	9.86365E-06	0.002440267
247.439999	362.126521	8.17269E-06	0.002022251
247.479999	379.447417	8.5636E-06	0.00211932
247.519999	480.914235	1.08536E-05	0.002686474

247.559999	555.239208	1.2531E-05	0.003102168
247.599999	532.91443	1.20271E-05	0.002977919
247.639999	432.866325	9.76919E-06	0.002419242
247.679999	309.397519	6.98267E-06	0.001729468
247.719999	216.970816	4.89673E-06	0.001213018
247.759999	173.620933	3.91838E-06	0.000970818
247.799999	191.285246	4.31704E-06	0.001069763
247.839999	303.675772	6.85354E-06	0.001698581
247.879999	622.499932	1.4049E-05	0.003482455
247.919999	1426.601008	3.21964E-05	0.007982131
247.959999	3080.246221	6.95169E-05	0.017237402
247.999999	5741.329491	0.000129574	0.032134305
248.039999	8838.040884	0.000199462	0.049474622
248.079999	11098.9803	0.000250489	0.062141193
248.119999	11450.43916	0.00025842	0.064119285
248.159999	9771.303673	0.000220525	0.054725413
248.199999	6948.886009	0.000156827	0.038924381
248.239999	4211.507541	9.50479E-05	0.02359468
248.279999	2256.81319	5.09331E-05	0.012645678
248.319999	1137.303415	2.56674E-05	0.006373717
248.359999	620.217376	1.39974E-05	0.003476405
248.399999	436.512849	9.85149E-06	0.002447109
248.439999	416.723863	9.40488E-06	0.002336548
248.479999	460.51737	1.03932E-05	0.002582511
248.519999	534.31694	1.20588E-05	0.00299685
248.559999	628.969186	1.4195E-05	0.003528299
248.599999	626.147149	1.41313E-05	0.003513033
248.639999	497.961452	1.12383E-05	0.00279429
248.679999	346.634415	7.82306E-06	0.001945438
248.719999	241.804918	5.4572E-06	0.001357315
248.759999	202.953194	4.58037E-06	0.001139413
248.799999	229.46476	5.1787E-06	0.001288461
248.839999	378.7172	8.54712E-06	0.002126865
248.879999	851.717439	1.92221E-05	0.00478399
248.919999	2043.593401	4.61211E-05	0.011480453
248.959999	4519.648827	0.000102002	0.025394462
248.999999	8640.493941	0.000195004	0.048555974
249.039999	13372.24241	0.000301793	0.0751585
249.079999	16814.29895	0.000379475	0.094519709
249.119999	17463.19357	0.00039412	0.098183163
249.159999	15143.15072	0.00034176	0.085152876
249.199999	11072.86031	0.000249899	0.062274839
249.239999	6829.955861	0.000154143	0.038418499
249.279999	3603.180854	8.13188E-05	0.020271144
249.319999	1692.316242	3.81932E-05	0.009522334
249.359999	796.211228	1.79694E-05	0.004480844

249.399999	491.778185	1.10987E-05	0.002768028
249.439999	424.726734	9.58549E-06	0.002391005
249.479999	489.077363	1.10378E-05	0.002753709
249.519999	607.299226	1.37059E-05	0.003419896
249.559999	684.610767	1.54507E-05	0.00385588
249.599999	645.629745	1.4571E-05	0.003636913
249.639999	497.72584	1.1233E-05	0.002804201
249.679999	335.131777	7.56346E-06	0.001888444
249.719999	235.059263	5.30496E-06	0.001324755
249.759999	208.515224	4.7059E-06	0.001175345
249.799999	242.032557	5.46234E-06	0.001364492
249.839999	349.246758	7.88201E-06	0.001969242
249.879999	642.749395	1.4506E-05	0.003624749
249.919999	1370.338332	3.09266E-05	0.007729183
249.959999	2821.729021	6.36825E-05	0.015918076
249.999999	5175.476459	0.000116803	0.029200824
250.039999	8045.190392	0.000181569	0.045399448
250.079999	10217.8104	0.000230602	0.057668885
250.119999	10638.03331	0.000240086	0.060050209
250.159999	9208.546562	0.000207824	0.051989269
250.199999	6599.164838	0.000148934	0.037263276
250.239999	3994.953326	9.01605E-05	0.022561771
250.279999	2136.769598	4.82239E-05	0.012069481
250.319999	1076.180213	2.42879E-05	0.006079744
250.359999	601.865943	1.35833E-05	0.003400709
250.399999	446.870076	1.00852E-05	0.002525343
250.439999	445.768911	1.00604E-05	0.002519522
250.479999	522.68014	1.17962E-05	0.002954703
250.519999	634.399255	1.43175E-05	0.003586822
250.559999	666.03899	1.50316E-05	0.003766311
250.599999	582.368941	1.31433E-05	0.0032937
250.639999	456.606292	1.0305E-05	0.002582837
250.679999	331.846204	7.48931E-06	0.001877419
250.719999	251.287131	5.6712E-06	0.001421883
250.759999	220.202533	4.96966E-06	0.001246193
250.799999	260.659021	5.88271E-06	0.001475384
250.839999	438.51221	9.89661E-06	0.002482466
250.879999	941.647871	2.12517E-05	0.005331622
250.919999	2162.973112	4.88153E-05	0.012248733
250.959999	4684.296079	0.000105718	0.026530999
250.999999	8757.224962	0.000197638	0.049607231
251.039999	13712.67447	0.000309476	0.077690845
251.079999	17496.98639	0.000394883	0.099147126
251.119999	18300.31105	0.000413013	0.103715706
251.159999	15968.9261	0.000360396	0.090517164
251.199999	11732.73012	0.000264791	0.066515594

251.239999	7303.003345	0.000164819	0.041409028
251.279999	3903.444742	8.80953E-05	0.02213659
251.319999	1865.166537	4.20942E-05	0.010579117
251.359999	877.556714	1.98052E-05	0.004978243
251.399999	504.054838	1.13758E-05	0.00285988
251.439999	427.257775	9.64261E-06	0.002424539
251.479999	494.259063	1.11547E-05	0.002805194
251.519999	642.086795	1.4491E-05	0.003644777
251.559999	751.864413	1.69685E-05	0.004268604
251.599999	731.404576	1.65068E-05	0.004153106
251.639999	585.012467	1.32029E-05	0.003322382
251.679999	403.768294	9.11249E-06	0.002293431
251.719999	284.716967	6.42567E-06	0.001617468
251.759999	229.144977	5.17148E-06	0.001301973
251.799999	224.601919	5.06895E-06	0.001276362
251.839999	328.553133	7.41499E-06	0.00186739
251.879999	645.262318	1.45627E-05	0.003668046
251.919999	1379.69097	3.11377E-05	0.00784421
251.959999	2994.131844	6.75734E-05	0.01702579
251.999999	5731.535319	0.000129353	0.032596898
252.039999	9124.233041	0.000205921	0.051900387
252.079999	11862.49052	0.00026772	0.067486829
252.119999	12535.76001	0.000282915	0.071328439
252.159999	10847.93715	0.000244823	0.061734525
252.199999	7803.579563	0.000176116	0.044416435
252.239999	4749.402145	0.000107187	0.027036947
252.279999	2528.071884	5.70551E-05	0.014393851
252.319999	1242.475237	2.80409E-05	0.007075289
252.359999	650.28865	1.46761E-05	0.003703663
252.399999	450.911979	1.01765E-05	0.002568537
252.439999	409.357358	9.23863E-06	0.002332199
252.479999	431.10544	9.72945E-06	0.002456491
252.519999	520.108589	1.17381E-05	0.002964112
252.559999	605.304161	1.36609E-05	0.00345019
252.599999	589.198137	1.32974E-05	0.003358918
252.639999	486.324362	1.09757E-05	0.002772892
252.679999	343.613769	7.75488E-06	0.001959504
252.719999	232.518784	5.24763E-06	0.00132618
252.759999	193.593914	4.36914E-06	0.001104345
252.799999	226.494278	5.11166E-06	0.001292228
252.839999	388.991696	8.779E-06	0.002219683
252.879999	815.856557	1.84127E-05	0.004656215
252.919999	1839.039709	4.15046E-05	0.010497334
252.959999	3914.064406	8.8335E-05	0.022345217
252.999999	7339.716508	0.000165647	0.041908736
253.039999	11550.00598	0.000260668	0.065959316

253.079999	14944.28426	0.000337272	0.085356716
253.119999	16150.11879	0.000364486	0.092258616
253.159999	14501.28098	0.000327274	0.082852613
253.199999	10928.23358	0.000246635	0.062447984
253.239999	7061.404773	0.000159366	0.040357863
253.279999	3963.686849	8.94549E-05	0.022657135
253.319999	1969.48313	4.44485E-05	0.011259692
253.359999	938.438253	2.11792E-05	0.005365973
253.399999	521.095875	1.17604E-05	0.002980087
253.439999	400.481404	9.03831E-06	0.002290669
253.479999	403.836721	9.11403E-06	0.002310225
253.519999	481.733162	1.0872E-05	0.002756281
253.559999	554.962398	1.25247E-05	0.00317577
253.599999	537.701777	1.21352E-05	0.003077482
253.639999	431.942865	9.74835E-06	0.002472571
253.679999	303.85277	6.85753E-06	0.001739619
253.719999	213.685806	4.82259E-06	0.001223588
253.759999	175.694455	3.96518E-06	0.001006204
253.799999	203.647975	4.59605E-06	0.001166478
253.839999	319.654268	7.21415E-06	0.00183124
253.879999	605.638747	1.36684E-05	0.003470139
253.919999	1308.696471	2.95355E-05	0.007499643
253.959999	2674.944051	6.03698E-05	0.015331505
253.999999	4824.588811	0.000108884	0.027656599
254.039999	7451.600073	0.000168172	0.042722474
254.079999	9616.292845	0.000217026	0.055142051
254.119999	10099.82866	0.000227939	0.057923875
254.159999	8726.086692	0.000196936	0.050053158
254.199999	6388.193039	0.000144173	0.03664868
254.239999	3981.942998	8.98669E-05	0.022847762
254.279999	2138.01228	4.8252E-05	0.012269508
254.319999	1065.593676	2.4049E-05	0.006116133
254.359999	559.935641	1.2637E-05	0.003214339
254.399999	367.080079	8.28449E-06	0.002107573
254.439999	337.64537	7.62019E-06	0.00193888
254.479999	397.249129	8.96536E-06	0.002281505
254.519999	502.929399	1.13504E-05	0.002888908
254.559999	561.400511	1.267E-05	0.003225282
254.599999	532.62119	1.20205E-05	0.003060424
254.639999	456.691568	1.03069E-05	0.002624547
254.679999	350.203052	7.9036E-06	0.002012888
254.719999	257.375237	5.8086E-06	0.001479567
254.759999	224.964091	5.07713E-06	0.001293449
254.799999	267.091938	6.02789E-06	0.001535907
254.839999	429.380402	9.69052E-06	0.002469531
254.879999	849.387136	1.91695E-05	0.004885918

254.919999	1881.668896	4.24666E-05	0.010825597
254.959999	3940.776588	8.89378E-05	0.022675591
254.999999	7170.524744	0.000161829	0.041266333
255.039999	10941.04614	0.000246924	0.062975541
255.079999	14012.89455	0.000316252	0.080669435
255.119999	15062.95044	0.00033995	0.086727995
255.159999	13743.69368	0.000310176	0.079144514
255.199999	10862.88665	0.00024516	0.062564888
255.239999	7467.893551	0.00016854	0.043018138
255.279999	4478.405305	0.000101071	0.025801499
255.319999	2380.07796	5.3715E-05	0.013714525
255.359999	1197.825606	2.70333E-05	0.006903212
255.399999	645.468526	1.45673E-05	0.003720495
255.439999	454.344888	1.02539E-05	0.002619264
255.479999	432.103108	9.75197E-06	0.002491432
255.519999	495.417446	1.11809E-05	0.002856939
255.559999	550.684151	1.24282E-05	0.003176144
255.599999	572.486872	1.29202E-05	0.003302411
255.639999	513.099287	1.15799E-05	0.002960295
255.679999	384.566363	8.67913E-06	0.002219079
255.719999	295.064052	6.65918E-06	0.001702887
255.759999	287.065301	6.47866E-06	0.001656983
255.799999	371.712082	8.38902E-06	0.002145912
255.839999	568.157999	1.28225E-05	0.003280517
255.879999	931.706435	2.10273E-05	0.00538047
255.919999	1661.675976	3.75017E-05	0.009597438
255.959999	3004.384996	6.78048E-05	0.017355313
255.999999	5141.869568	0.000116045	0.029707477
256.039999	7896.99499	0.000178224	0.045632518
256.079999	10564.73121	0.000238431	0.061057479
256.119999	12883.32268	0.000290759	0.074469105
256.159999	15263.62763	0.000344479	0.088241693
256.199999	17796.22326	0.000401636	0.102899135
256.239999	19527.2931	0.000440704	0.112925943
256.279999	19118.9402	0.000431488	0.110581706
256.319999	16017.60257	0.000361495	0.092658393
256.359999	11271.75717	0.000254388	0.065214871
256.399999	6764.60354	0.000152668	0.039143993
256.439999	3566.080739	8.04815E-05	0.020638669
256.479999	1815.143646	4.09653E-05	0.010506771
256.519999	1102.868467	2.48902E-05	0.006384835
256.559999	880.930246	1.98814E-05	0.005100764
256.599999	753.235799	1.69995E-05	0.004362067
256.639999	578.40677	1.30538E-05	0.003350136
256.679999	396.698792	8.95294E-06	0.00229804
256.719999	265.89053	6.00078E-06	0.00154052

256.759999	206.339393	4.65679E-06	0.001195678
256.799999	249.469617	5.63018E-06	0.001445831
256.839999	433.223636	9.77725E-06	0.00251119
256.879999	914.504566	2.06391E-05	0.005301771
256.919999	1994.143092	4.5005E-05	0.011562693
256.959999	4127.635919	9.3155E-05	0.023937107
256.999999	7462.446647	0.000168417	0.043283176
257.039999	11096.55411	0.000250434	0.064371496
257.079999	13607.04043	0.000307092	0.078947198
257.119999	14180.60722	0.000320037	0.082287798
257.159999	12881.62477	0.00029072	0.074761639
257.199999	10634.61085	0.000240008	0.061730148
257.239999	8361.645941	0.000188711	0.048543941
257.279999	6249.510756	0.000141043	0.036287479
257.319999	4338.925244	9.79235E-05	0.025197675
257.359999	2739.426355	6.1825E-05	0.015911291
257.399999	1607.627569	3.62819E-05	0.009338965
257.439999	963.896486	2.17538E-05	0.005600299
257.479999	691.236578	1.56002E-05	0.004016752
257.519999	644.317874	1.45414E-05	0.00374469
257.559999	707.20714	1.59607E-05	0.004110833
257.599999	681.995601	1.53917E-05	0.0039649
257.639999	556.484434	1.25591E-05	0.003235721
257.679999	433.141516	9.7754E-06	0.002518925
257.719999	407.683303	9.20084E-06	0.002371242
257.759999	633.754192	1.43029E-05	0.003686728
257.799999	1385.60989	3.12713E-05	0.008061737
257.839999	2957.443001	6.67454E-05	0.017209626
257.879999	5372.942929	0.00012126	0.031270487
257.919999	8062.868355	0.000181968	0.046933111
257.959999	10365.80509	0.000233942	0.060347622
257.999999	11889.76506	0.000268335	0.069230542
258.039999	12653.79896	0.000285579	0.073690706
258.079999	12609.9277	0.000284589	0.073446601
258.119999	11400.12472	0.000257285	0.066410387
258.159999	9141.671604	0.000206315	0.053262227
258.199999	6434.887668	0.000145226	0.03749747
258.239999	3999.739868	9.02686E-05	0.023310952
258.279999	2241.751999	5.05932E-05	0.013067217
258.319999	1206.628021	2.72319E-05	0.007034548
258.359999	698.790417	1.57707E-05	0.004074525
258.399999	468.852251	1.05813E-05	0.002734219
258.439999	386.090942	8.71353E-06	0.002251926
258.479999	451.478083	1.01892E-05	0.002633713
258.519999	615.078051	1.38815E-05	0.003588634
258.559999	734.111013	1.65679E-05	0.004283787

258.599999	704.470128	1.58989E-05	0.004111458
258.639999	550.593867	1.24261E-05	0.003213896
258.679999	378.889185	8.551E-06	0.002211973
258.719999	281.002743	6.34184E-06	0.001640761
258.759999	280.731351	6.33572E-06	0.00163943
258.799999	407.755529	9.20247E-06	0.0023816
258.839999	767.435298	1.73199E-05	0.004483095
258.879999	1587.048211	3.58175E-05	0.009272426
258.919999	3228.955181	7.2873E-05	0.018868282
258.959999	6039.637345	0.000136306	0.035297854
258.999999	9938.392165	0.000224296	0.058092578
259.039999	14027.09223	0.000316572	0.082004793
259.079999	16454.36803	0.000371352	0.096209919
259.119999	16051.9406	0.00036227	0.093871387
259.159999	13167.02912	0.000297162	0.077012376
259.199999	9231.700207	0.000208347	0.054003442
259.239999	5610.871439	0.00012663	0.032827445
259.279999	3021.816456	6.81982E-05	0.017682426
259.319999	1493.735628	3.37115E-05	0.008742075
259.359999	744.629715	1.68053E-05	0.004358611
259.399999	439.973166	9.92958E-06	0.002575733
259.439999	354.346685	7.99711E-06	0.002074771
259.479999	405.936422	9.16142E-06	0.002377205
259.519999	499.760649	1.12789E-05	0.002927101
259.559999	561.702942	1.26769E-05	0.003290404
259.599999	542.093408	1.22343E-05	0.003176022
259.639999	494.738525	1.11656E-05	0.002899026
259.679999	419.923145	9.47708E-06	0.002461008
259.719999	339.762167	7.66796E-06	0.001991522
259.759999	322.679646	7.28243E-06	0.001891684
259.799999	433.151604	9.77563E-06	0.002539708
259.839999	759.697276	1.71453E-05	0.004455037
259.879999	1382.029954	3.11905E-05	0.008105785
259.919999	2363.733957	5.33462E-05	0.01386574
259.959999	3901.127654	8.8043E-05	0.022887663
259.999999	6018.792157	0.000135836	0.035317296
260.039999	8268.397559	0.000186606	0.04852508
260.079999	9816.049308	0.000221535	0.057616708
260.119999	9860.534384	0.000222539	0.057886721
260.159999	8348.191904	0.000188407	0.049015981
260.199999	5982.919056	0.000135026	0.035133804
260.239999	3691.576161	8.33137E-05	0.021681566
260.279999	2016.208492	4.5503E-05	0.011843525
260.319999	1018.404301	2.2984E-05	0.005983186
260.359999	529.484426	1.19497E-05	0.003111231
260.399999	360.738039	8.14136E-06	0.002120009

260.439999	342.927057	7.73939E-06	0.002015646
260.479999	400.646485	9.04203E-06	0.002355269
260.519999	473.003048	1.0675E-05	0.002781056
260.559999	517.864906	1.16875E-05	0.003045292
260.599999	514.321377	1.16075E-05	0.003024919
260.639999	431.870918	9.74673E-06	0.002540386
260.679999	342.655849	7.73327E-06	0.002015908
260.719999	257.710687	5.81617E-06	0.001516392
260.759999	223.235262	5.03811E-06	0.001313737
260.799999	284.264174	6.41545E-06	0.001673148
260.839999	484.647582	1.09378E-05	0.002853021
260.879999	1034.739646	2.33526E-05	0.006092235
260.919999	2343.435074	5.28881E-05	0.013799554
260.959999	5075.947192	0.000114557	0.02989481
260.999999	9401.71913	0.000212184	0.055379947
261.039999	14132.50383	0.000318951	0.08325895
261.079999	17299.28416	0.000390421	0.101931048
261.119999	17324.03687	0.000390979	0.102092536
261.159999	14361.6513	0.000324122	0.084647823
261.199999	10113.4693	0.000228247	0.059618094
261.239999	6184.521986	0.000139576	0.036462846
261.279999	3323.65587	7.50103E-05	0.019598687
261.319999	1586.314344	3.58009E-05	0.009355493
261.359999	757.386875	1.70932E-05	0.00446747
261.399999	459.778044	1.03766E-05	0.00271243
261.439999	392.20923	8.85162E-06	0.002314166
261.479999	395.577536	8.92763E-06	0.002334398
261.519999	451.645357	1.0193E-05	0.002665675
261.559999	534.37719	1.20601E-05	0.003154453
261.599999	519.668886	1.17282E-05	0.003068098
261.639999	427.436654	9.64665E-06	0.002523949
261.679999	314.903531	7.10693E-06	0.001859743
261.719999	235.834438	5.32245E-06	0.001392993
261.759999	208.454253	4.70452E-06	0.001231456
261.799999	240.814408	5.43485E-06	0.001422843
261.839999	333.406759	7.52453E-06	0.001970222
261.879999	582.712922	1.3151E-05	0.003443989
261.919999	1176.768502	2.6558E-05	0.006956078
261.959999	2352.904774	5.31018E-05	0.013910543
261.999999	4376.431256	9.877E-05	0.025877729
262.039999	7031.435845	0.00015869	0.041583052
262.079999	9306.665368	0.000210038	0.055046883
262.119999	10007.6259	0.000225858	0.059201945
262.159999	8920.793562	0.00020133	0.052780642
262.199999	6775.884847	0.000152922	0.040096222
262.239999	4425.979534	9.98882E-05	0.02619468

262.279999	2498.813865	5.63947E-05	0.014791215
262.319999	1278.876747	2.88625E-05	0.007571202
262.359999	663.25274	1.49687E-05	0.003927186
262.399999	417.490617	9.42218E-06	0.00247238
262.439999	347.042163	7.83226E-06	0.002055498
262.479999	369.766327	8.34511E-06	0.002190425
262.519999	465.924767	1.05153E-05	0.002760469
262.559999	531.426438	1.19936E-05	0.003149028
262.599999	529.402094	1.19479E-05	0.00313751
262.639999	456.815083	1.03097E-05	0.002707734
262.679999	365.750615	8.25448E-06	0.002168287
262.719999	291.315129	6.57458E-06	0.001727273
262.759999	270.022937	6.09404E-06	0.00160127
262.799999	315.812187	7.12744E-06	0.001873092
262.839999	486.269724	1.09744E-05	0.002884519
262.879999	905.297176	2.04313E-05	0.005370979
262.919999	1891.492604	4.26884E-05	0.011223622
262.959999	3959.734478	8.93657E-05	0.023499602
262.999999	7232.747166	0.000163233	0.042930287
263.039999	11017.33164	0.000248646	0.065403799
263.079999	13775.90824	0.000310903	0.081792377
263.119999	14368.01664	0.000324266	0.085320902
263.159999	12596.50874	0.000284286	0.074812613
263.199999	9453.174103	0.000213345	0.056152397
263.239999	6084.416051	0.000137317	0.036147273
263.279999	3399.030242	7.67114E-05	0.020196572
263.319999	1671.27122	3.77183E-05	0.009931973
263.359999	788.099668	1.77863E-05	0.004684203
263.399999	442.420561	9.98482E-06	0.00263
263.439999	375.937325	8.48438E-06	0.002235126
263.479999	484.408933	1.09324E-05	0.002880478
263.519999	680.776791	1.53642E-05	0.00404877
263.559999	789.65659	1.78214E-05	0.004697021
263.599999	716.486457	1.61701E-05	0.004262439
263.639999	535.029516	1.20749E-05	0.003183419
263.679999	357.113538	8.05956E-06	0.002125143
263.719999	243.66562	5.49919E-06	0.001450247
263.759999	185.91933	4.19594E-06	0.001106721
263.799999	196.460827	4.43385E-06	0.001169649
263.839999	294.404796	6.64431E-06	0.001753034
263.879999	522.732531	1.17973E-05	0.003113083
263.919999	1082.570137	2.44321E-05	0.00644812
263.959999	2265.383393	5.11265E-05	0.013495363
263.999999	4357.169853	9.83353E-05	0.025960507
264.039999	6974.628843	0.000157408	0.041561918
264.079999	9047.485496	0.000204189	0.053922269

264.119999	9549.932122	0.000215529	0.056925431
264.159999	8334.822534	0.000188105	0.049689905
264.199999	6139.558106	0.000138561	0.036607889
264.239999	3868.163234	8.72991E-05	0.023067903
264.279999	2132.852992	4.81355E-05	0.012721255
264.319999	1081.050932	2.43978E-05	0.00644883
264.359999	556.577391	1.25612E-05	0.003320673
264.399999	366.298454	8.26685E-06	0.002185754
264.439999	385.319176	8.69612E-06	0.002299601
264.479999	542.612453	1.2246E-05	0.003238824
264.519999	785.044239	1.77174E-05	0.004686595
264.559999	913.962932	2.06269E-05	0.005457045
264.599999	824.140762	1.85997E-05	0.004921483
264.639999	621.452281	1.40253E-05	0.003711659
264.679999	418.351555	9.44161E-06	0.002499006
264.719999	293.222976	6.61763E-06	0.00175182
264.759999	243.150491	5.48757E-06	0.001452888
264.799999	273.198433	6.16571E-06	0.001632679
264.839999	435.265538	9.82334E-06	0.002601613
264.879999	860.421429	1.94185E-05	0.005143576
264.919999	1866.752652	4.213E-05	0.011161081
264.959999	3920.921256	8.84897E-05	0.023446239
264.999999	7417.940999	0.000167413	0.044364338
265.039999	11901.01313	0.000268589	0.071186905
265.079999	15713.52017	0.000354632	0.09400592
265.119999	17158.15091	0.000387236	0.102663893
265.159999	15516.38358	0.000350183	0.092854568
265.199999	11802.24871	0.00026636	0.070638755
265.239999	7670.94035	0.000173122	0.045918996
265.279999	4291.244339	9.68474E-05	0.02569168
265.319999	2090.213599	4.71732E-05	0.012515996
265.359999	952.514395	2.14969E-05	0.005704424
265.399999	482.134187	1.08811E-05	0.002887843
265.439999	340.366496	7.6816E-06	0.002039003
265.479999	369.593779	8.34122E-06	0.002214426
265.519999	504.239665	1.138E-05	0.003021614
265.559999	649.843059	1.46661E-05	0.003894717
265.599999	679.570427	1.5337E-05	0.004073496
265.639999	575.879284	1.29968E-05	0.003452468
265.679999	411.855238	9.295E-06	0.002469495
265.719999	286.77181	6.47204E-06	0.001719751
265.759999	216.163582	4.87851E-06	0.001296513
265.799999	206.476844	4.65989E-06	0.0012386
265.839999	281.185499	6.34597E-06	0.001687011
265.879999	498.373449	1.12476E-05	0.00299051
265.919999	1058.085243	2.38795E-05	0.006350039

265.959999	2352.34775	5.30892E-05	0.014119607
265.999999	4736.361694	0.000106893	0.028433561
266.039999	8049.490581	0.000181666	0.048330367
266.079999	11238.92434	0.000253647	0.067490358
266.119999	12719.77472	0.000287068	0.076394429
266.159999	11700.99965	0.000264075	0.070286272
266.199999	8889.2651	0.000200618	0.0534046
266.239999	5666.914958	0.000127894	0.034050599
266.279999	3094.989514	6.98496E-05	0.018599552
266.319999	1522.089108	3.43514E-05	0.009148473
266.359999	753.140358	1.69973E-05	0.004527408
266.399999	453.594982	1.0237E-05	0.002727139
266.439999	403.350947	9.10307E-06	0.002425422
266.479999	486.449134	1.09785E-05	0.002925545
266.519999	616.135833	1.39053E-05	0.003706048
266.559999	753.314517	1.70013E-05	0.004531856
266.599999	812.424343	1.83353E-05	0.004888187
266.639999	712.159675	1.60725E-05	0.004285559
266.679999	515.610463	1.16366E-05	0.003103251
266.719999	352.719615	7.96039E-06	0.002123195
266.759999	268.448823	6.05852E-06	0.00161617
266.799999	260.583284	5.881E-06	0.001569051
266.839999	374.914988	8.46131E-06	0.002257816
266.879999	767.35702	1.73182E-05	0.004621876
266.919999	1857.364192	4.19181E-05	0.011188785
266.959999	4155.786521	9.37903E-05	0.025038262
266.999999	7964.307601	0.000179743	0.047991469
267.039999	12718.63785	0.000287042	0.07665168
267.079999	16708.57975	0.000377089	0.100713029
267.119998	18293.73683	0.000412864	0.110284273
267.159998	16717.33557	0.000377287	0.100795988
267.199998	12820.57493	0.000289343	0.07731232
267.239998	8352.035531	0.000188494	0.050373085
267.279998	4670.196002	0.0001054	0.028171263
267.319998	2239.955442	5.05527E-05	0.013513741
267.359998	982.561619	2.2175E-05	0.005928721
267.399998	485.28925	1.09523E-05	0.002928646
267.439998	360.110852	8.1272E-06	0.002173538
267.479998	371.401138	8.38201E-06	0.002242019
267.519998	438.590563	9.89838E-06	0.002648014
267.559998	544.489116	1.22884E-05	0.003287874
267.599998	574.230748	1.29596E-05	0.003467986
267.639998	501.117682	1.13095E-05	0.003026882
267.679998	384.841696	8.68534E-06	0.002324892
267.719998	285.177476	6.43606E-06	0.001723062
267.759998	258.298097	5.82943E-06	0.001560888

267.799998	355.405609	8.02101E-06	0.002148026
267.839998	627.878565	1.41703E-05	0.003795385
267.879998	1225.113056	2.76491E-05	0.00740664
267.919998	2541.040419	5.73477E-05	0.015364607
267.959998	5128.072315	0.000115733	0.031011935
267.999998	9252.19473	0.000208809	0.055960851
268.039998	14070.84045	0.000317559	0.085118586
268.079998	17453.06115	0.000393891	0.105594374
268.119998	17533.74253	0.000395712	0.10609834
268.159998	14415.75203	0.000325343	0.087244097
268.199998	9908.440281	0.00022362	0.059974802
268.239998	5769.35595	0.000130206	0.034926545
268.279998	2897.658017	6.53961E-05	0.017544467
268.319998	1328.252083	2.99768E-05	0.008043375
268.359998	616.103764	1.39046E-05	0.003731439
268.399998	355.230802	8.01706E-06	0.00215178
268.439998	308.799203	6.96917E-06	0.001870803
268.479998	364.996144	8.23745E-06	0.002211592
268.519998	453.409889	1.02328E-05	0.002747719
268.559998	521.807503	1.17765E-05	0.003162688
268.599998	519.037068	1.17139E-05	0.003146365
268.639998	456.0293	1.02919E-05	0.002764828
268.679998	362.124698	8.17265E-06	0.002195828
268.719998	273.326536	6.1686E-06	0.001657626
268.759998	235.055943	5.30489E-06	0.001425741
268.799998	277.247742	6.2571E-06	0.001681907
268.839998	445.0768	1.00448E-05	0.002700434
268.879998	865.824545	1.95405E-05	0.005254037
268.919998	1841.318911	4.1556E-05	0.01117524
268.959998	3758.512379	8.48244E-05	0.022814368
268.999998	6862.11055	0.000154868	0.041659566
269.039998	10596.18767	0.000239141	0.064338547
269.079998	13571.49707	0.00030629	0.082416455
269.119998	14560.35937	0.000328607	0.088434723
269.159998	13351.39711	0.000301322	0.081103946
269.199998	10556.99119	0.000238257	0.064138673
269.239998	7274.629753	0.000164178	0.044203355
269.279998	4383.48653	9.89292E-05	0.026639651
269.319998	2307.247252	5.20714E-05	0.014023858
269.359998	1097.507073	2.47692E-05	0.006671833
269.399998	558.906938	1.26138E-05	0.003398145
269.439998	405.254283	9.14602E-06	0.002464305
269.479998	432.888426	9.76969E-06	0.002632736
269.519998	499.038695	1.12626E-05	0.003035498
269.559998	585.433521	1.32124E-05	0.00356154
269.599998	610.030011	1.37675E-05	0.003711725

269.639998	499.04305	1.12627E-05	0.003036876
269.679998	352.000159	7.94415E-06	0.002142379
269.719998	247.968764	5.59631E-06	0.001509437
269.759998	210.506988	4.75085E-06	0.001281589
269.799998	239.635873	5.40825E-06	0.001459145
269.839998	325.591746	7.34815E-06	0.001982825
269.879998	548.939838	1.23888E-05	0.003343491
269.919998	1129.49192	2.54911E-05	0.006880547
269.959998	2347.922271	5.29893E-05	0.014305001
269.999998	4341.824568	9.79889E-05	0.026457012
270.039998	6768.09383	0.000152746	0.041247651
270.079998	8664.974617	0.000195556	0.052815874
270.119998	9127.125102	0.000205987	0.055641073
270.159998	8160.126237	0.000184163	0.04975339
270.199998	6415.861509	0.000144797	0.039124165
270.239998	4584.960348	0.000103476	0.0279634
270.279998	3117.965154	7.03681E-05	0.019019098
270.319998	2078.244761	4.69031E-05	0.012678843
270.359998	1353.295615	3.0542E-05	0.008257334
270.399998	883.152714	1.99315E-05	0.005389485
270.439998	620.746987	1.40094E-05	0.003788701
270.479998	555.844184	1.25446E-05	0.003393071
270.519998	594.053487	1.3407E-05	0.003626851
270.559998	648.362685	1.46326E-05	0.003959008
270.599998	632.092372	1.42654E-05	0.003860229
270.639998	524.327683	1.18333E-05	0.003202577
270.679998	382.345569	8.62901E-06	0.0023357
270.719998	287.562932	6.48989E-06	0.001756944
270.759998	282.565977	6.37712E-06	0.001726669
270.799998	581.047944	1.31134E-05	0.00355112
270.839998	1882.255307	4.24799E-05	0.011505251
270.879998	6172.643726	0.000139308	0.037735743
270.919998	16643.07297	0.000375611	0.101760525
270.959998	35784.08252	0.000807597	0.218826454
270.999998	60913.99986	0.001374744	0.372555657
271.039998	81776.64887	0.001845585	0.500227371
271.079998	87558.27476	0.001976068	0.535672592
271.119998	76232.52322	0.001720462	0.466451618
271.159998	55047.24852	0.00124234	0.336872837
271.199998	34138.94527	0.000770468	0.20895104
271.239998	18679.21148	0.000421564	0.11434496
271.279998	9177.422658	0.000207122	0.05618796
271.319998	4066.047472	9.1765E-05	0.024897687
271.359998	1736.830519	3.91978E-05	0.010636727
271.399998	890.958711	2.01077E-05	0.005457229
271.439998	726.377329	1.63933E-05	0.004449804

271.479998	876.550598	1.97825E-05	0.00537056
271.519998	1087.166636	2.45358E-05	0.00666197
271.559998	1159.962698	2.61787E-05	0.007109099
271.599998	1019.214207	2.30022E-05	0.00624741
271.639998	789.253965	1.78124E-05	0.00483855
271.679998	533.03367	1.20298E-05	0.003268264
271.719998	343.515838	7.75267E-06	0.002106557
271.759998	260.065899	5.86933E-06	0.001595048
271.799998	301.878471	6.81298E-06	0.001851767
271.839998	557.644813	1.25853E-05	0.003421179
271.879998	1378.04196	3.11005E-05	0.008455601
271.919998	3426.693087	7.73357E-05	0.021029121
271.959998	7550.94835	0.000170414	0.046345896
271.999998	13575.80044	0.000306387	0.083337238
272.039998	19327.42354	0.000436193	0.118661951
272.079998	21866.94989	0.000493507	0.134273275
272.119998	20007.6771	0.000451545	0.122874534
272.159998	15273.15889	0.000344694	0.093811897
272.199998	9956.708302	0.000224709	0.0611658
272.239998	5633.702163	0.000127145	0.034613903
272.279998	2828.688014	6.38395E-05	0.017382232
272.319998	1312.251446	2.96157E-05	0.008064944
272.359998	651.081719	1.4694E-05	0.00400206
272.399998	415.784455	9.38368E-06	0.002556113
272.439998	424.509991	9.5806E-06	0.002610138
272.479998	602.963939	1.36081E-05	0.003707923
272.519998	833.857859	1.8819E-05	0.005128557
272.559998	994.052187	2.24344E-05	0.006114713
272.599998	954.182762	2.15346E-05	0.005870325
272.639998	796.180666	1.79687E-05	0.004898983
272.679998	564.415884	1.27381E-05	0.00347342
272.719998	363.39675	8.20136E-06	0.002236674
272.759998	281.104065	6.34413E-06	0.001730424
272.799998	343.390783	7.74985E-06	0.00211416
272.839998	624.821363	1.41013E-05	0.003847412
272.879998	1370.941554	3.09402E-05	0.008442973
272.919998	3064.164128	6.91539E-05	0.018873487
272.959998	6263.831218	0.000141366	0.038587248
272.999998	11030.70009	0.000248948	0.067962677
273.039998	16060.45267	0.000362462	0.098966636
273.079998	19257.48383	0.000434615	0.11868455
273.119998	19423.0318	0.000438351	0.119722362
273.159998	16736.11226	0.000377711	0.103175466
273.199998	12487.37086	0.000281823	0.076993925
273.239998	8145.474135	0.000183832	0.050230257
273.279998	4607.283485	0.00010398	0.028415646

273.319998	2282.776327	5.15191E-05	0.014081195
273.359998	1031.381881	2.32769E-05	0.00636296
273.399998	502.618508	1.13434E-05	0.003101285
273.439998	373.80447	8.43625E-06	0.002306807
273.479998	456.553115	1.03038E-05	0.002817874
273.519998	612.30791	1.38189E-05	0.003779756
273.559998	726.734796	1.64014E-05	0.004486765
273.599998	742.349814	1.67538E-05	0.00458384
273.639998	633.138692	1.42891E-05	0.003910058
273.679998	477.925063	1.07861E-05	0.002951941
273.719998	343.862509	7.7605E-06	0.002124203
273.759998	278.515289	6.2857E-06	0.001720774
273.799998	315.364025	7.11733E-06	0.001948724
273.839998	491.579053	1.10943E-05	0.003038051
273.879998	838.933793	1.89336E-05	0.005185525
273.919998	1587.187271	3.58206E-05	0.00981198
273.959998	3032.685565	6.84435E-05	0.018750778
273.999998	5305.872928	0.000119746	0.032810448
274.039998	8082.881365	0.000182419	0.049990205
274.079998	10209.90529	0.000230423	0.063154429
274.119998	10706.46651	0.00024163	0.066235625
274.159998	9485.346012	0.000214071	0.058689718
274.199998	7103.749119	0.000160322	0.043960216
274.239998	4503.539126	0.000101639	0.027873371
274.279998	2460.865832	5.55383E-05	0.015233049
274.319998	1208.092741	2.7265E-05	0.007479327
274.359998	607.230936	1.37044E-05	0.003759927
274.399998	369.84154	8.34681E-06	0.002290364
274.439998	316.312648	7.13874E-06	0.001959155
274.479998	378.02708	8.53154E-06	0.002341738
274.519998	501.715737	1.1323E-05	0.003108397
274.559998	629.883595	1.42156E-05	0.003903034
274.599998	660.26827	1.49013E-05	0.004091907
274.639998	582.104614	1.31373E-05	0.003608025
274.679998	452.326058	1.02084E-05	0.002804035
274.719998	345.000632	7.78618E-06	0.00213902
274.759998	296.3367	6.68791E-06	0.001837569
274.799998	326.059783	7.35872E-06	0.002022175
274.839998	490.201584	1.10632E-05	0.003040601
274.879998	980.38917	2.2126E-05	0.006082
274.919998	2153.033469	4.8591E-05	0.013358628
274.959998	4489.292066	0.000101317	0.027858141
274.999998	8176.083356	0.000184523	0.050743773
275.039998	12701.83982	0.000286663	0.078843745
275.079998	16661.91348	0.000376036	0.103440031
275.119998	18818.68246	0.000424711	0.116846614

275.159998	18759.91746	0.000423385	0.116498673
275.199998	16573.9268	0.00037405	0.102938681
275.239998	12816.43108	0.000289249	0.079612894
275.279998	8566.255211	0.000193328	0.053219455
275.319998	4943.797598	0.000111575	0.030718726
275.359998	2443.63358	5.51494E-05	0.01518594
275.399998	1096.695872	2.47509E-05	0.006816397
275.439998	552.40175	1.24669E-05	0.003433893
275.479998	443.147509	1.00012E-05	0.002755137
275.519998	496.217745	1.11989E-05	0.003085533
275.559998	562.066101	1.2685E-05	0.003495492
275.599998	574.816452	1.29728E-05	0.003575305
275.639998	515.925307	1.16437E-05	0.003209474
275.679998	433.760482	9.78937E-06	0.002698733
275.719998	375.462606	8.47367E-06	0.00233636
275.759998	383.852453	8.66302E-06	0.002388913
275.799998	515.553785	1.16353E-05	0.003209024
275.839998	836.169303	1.88712E-05	0.005205425
275.879998	1417.503385	3.19911E-05	0.008825698
275.919998	2325.859911	5.24914E-05	0.014483432
275.959998	3662.75928	8.26634E-05	0.022811785
275.999998	5504.429116	0.000124227	0.034286734
276.039998	7670.693519	0.000173117	0.04778718
276.079998	9477.065913	0.000213884	0.059049143
276.119998	10117.51199	0.000228338	0.06304873
276.159998	9261.526491	0.00020902	0.057722894
276.199998	7270.34785	0.000164082	0.045319344
276.239998	4952.239049	0.000111765	0.030874001
276.279998	3007.860392	6.78832E-05	0.018754776
276.319998	1644.859058	3.71222E-05	0.0102576
276.359998	858.599141	1.93774E-05	0.005355135
276.399998	502.748236	1.13463E-05	0.003136125
276.439998	397.653112	8.97448E-06	0.002480904
276.479998	446.765307	1.00829E-05	0.002787712
276.519998	538.359112	1.215E-05	0.003359722
276.559998	590.628282	1.33297E-05	0.00368645
276.599998	597.442101	1.34834E-05	0.003729518
276.639998	537.289581	1.21259E-05	0.003354503
276.679998	427.002075	9.63684E-06	0.002666321
276.719998	321.27369	7.2507E-06	0.002006414
276.759998	271.933334	6.13716E-06	0.001698519
276.799998	311.055802	7.0201E-06	0.001943163
276.839998	490.722103	1.10749E-05	0.003065979
276.879998	957.643798	2.16127E-05	0.005984121
276.919998	2034.368406	4.59129E-05	0.012714189
276.959998	4327.754587	9.76714E-05	0.027051069

276.999998	8024.41642	0.0001811	0.050164672
277.039998	12574.38194	0.000283786	0.078620152
277.079998	16904.01908	0.0003815	0.105706065
277.119998	19648.12687	0.000443431	0.122883558
277.159998	20106.25863	0.00045377	0.125766962
277.199998	18428.19701	0.000415899	0.115287129
277.239998	15088.59822	0.000340529	0.094408161
277.279998	10799.71259	0.000243734	0.067582693
277.319998	6630.083281	0.000149632	0.041495875
277.359998	3488.52271	7.87311E-05	0.021836857
277.399998	1633.527657	3.68664E-05	0.010226751
277.439998	786.121248	1.77417E-05	0.004922247
277.479998	539.62073	1.21785E-05	0.003379287
277.519998	556.074566	1.25498E-05	0.003482828
277.559998	650.609869	1.46834E-05	0.004075513
277.599998	692.70207	1.56333E-05	0.00433981
277.639998	610.027017	1.37675E-05	0.003822398
277.679998	482.728603	1.08945E-05	0.003025188
277.719998	375.488715	8.47426E-06	0.002353471
277.759998	327.388831	7.38871E-06	0.002052288
277.799998	371.082337	8.37481E-06	0.002326523
277.839998	518.941085	1.17118E-05	0.003254
277.879998	844.346144	1.90557E-05	0.005295202
277.919998	1529.270898	3.45135E-05	0.009591996
277.959998	2776.730929	6.26669E-05	0.017418905
277.999998	4656.411513	0.000105089	0.029214665
278.039998	7036.899769	0.000158813	0.044156373
278.079998	9184.421562	0.00020728	0.057640311
278.119998	10221.49684	0.000230685	0.064158097
278.159998	9749.631073	0.000220036	0.0612051
278.199998	7982.82685	0.000180161	0.050120869
278.239998	5654.068359	0.000127604	0.035504661
278.279998	3592.867852	8.1086E-05	0.022564618
278.319998	2046.914726	4.6196E-05	0.012857275
278.359998	1058.392526	2.38864E-05	0.006649031
278.399998	589.480163	1.33037E-05	0.003703763
278.439998	455.982601	1.02909E-05	0.002865396
278.479998	499.021623	1.12622E-05	0.003136304
278.519998	625.40948	1.41146E-05	0.003931204
278.559998	722.074108	1.62962E-05	0.004539471
278.599998	712.872309	1.60885E-05	0.004482266
278.639998	629.708547	1.42116E-05	0.003959933
278.679998	505.514262	1.14088E-05	0.003179391
278.719998	385.376802	8.69742E-06	0.002424144
278.759998	321.399571	7.25354E-06	0.002021997
278.799998	369.671463	8.34297E-06	0.00232602

278.839998	552.878713	1.24777E-05	0.003479283
278.879998	1004.487238	2.26699E-05	0.006322175
278.919998	2099.323228	4.73788E-05	0.013214895
278.959998	4408.68645	9.94979E-05	0.027755937
278.999998	8371.373571	0.00018893	0.052711537
279.039998	13630.13116	0.000307613	0.085836352
279.079998	18432.15905	0.000415988	0.116093974
279.119998	20971.8797	0.000473306	0.132109201
279.159998	20531.22311	0.000463361	0.129351886
279.199998	17469.74007	0.000394268	0.110079541
279.239998	13050.97299	0.000294542	0.082247986
279.279998	8550.112119	0.000192964	0.053891019
279.319998	4928.908992	0.000111239	0.031071168
279.359998	2548.450229	5.7515E-05	0.016067382
279.399998	1262.45529	2.84919E-05	0.007960625
279.439998	733.663685	1.65578E-05	0.004626903
279.479998	653.752212	1.47543E-05	0.004123525
279.519998	717.925737	1.62026E-05	0.004528946
279.559998	798.512591	1.80213E-05	0.005038039
279.599998	805.36174	1.81759E-05	0.00508198
279.639998	690.296399	1.5579E-05	0.004356519
279.679998	502.496504	1.13406E-05	0.003171752
279.719998	335.956529	7.58207E-06	0.002120857
279.759998	255.23822	5.76037E-06	0.001611521
279.799998	248.753965	5.61403E-06	0.001570806
279.839998	333.67326	7.53054E-06	0.002107346
279.879998	599.617691	1.35325E-05	0.003787486
279.919998	1176.592525	2.65541E-05	0.007433011
279.959998	2367.085803	5.34218E-05	0.014955975
279.999998	4497.177163	0.000101495	0.028418607
280.039998	7358.121839	0.000166063	0.046504159
280.079998	9956.59001	0.000224706	0.062935759
280.119998	11163.3035	0.00025194	0.070573491
280.159998	10444.91832	0.000235727	0.066041348
280.199998	8307.675431	0.000187493	0.052535444
280.239998	5698.090345	0.000128598	0.036038293
280.279998	3446.46903	7.7782E-05	0.02180074
280.319998	1878.848667	4.2403E-05	0.011886408
280.359998	988.632546	2.23121E-05	0.006255409
280.399998	616.244378	1.39078E-05	0.003899741
280.439998	605.619771	1.3668E-05	0.003833052
280.479998	847.47419	1.91263E-05	0.005364548
280.519998	1119.560693	2.52669E-05	0.007087878
280.559998	1215.525303	2.74327E-05	0.007696522
280.599998	1111.706788	2.50897E-05	0.007040162
280.639998	881.892552	1.99031E-05	0.005585602

280.679998	633.043185	1.42869E-05	0.004010048
280.719998	457.814915	1.03322E-05	0.002900468
280.759998	441.492738	9.96388E-06	0.002797458
280.799998	712.695484	1.60845E-05	0.00451654
280.839998	1403.797124	3.16817E-05	0.008897501
280.879998	2738.826135	6.18115E-05	0.017361611
280.919998	4783.88136	0.000107966	0.030329679
280.959998	7340.174072	0.000165658	0.046543132
280.999998	10223.63382	0.000230733	0.064836022
281.039998	13072.77207	0.000295034	0.082916427
281.079998	14862.95555	0.000335436	0.094284408
281.119998	14940.80504	0.000337193	0.09479174
281.159998	13289.70508	0.00029993	0.084328356
281.199998	10349.42604	0.000233572	0.065680483
281.239998	6986.733667	0.000157681	0.044346161
281.279998	4130.672961	9.32235E-05	0.026221916
281.319998	2173.772764	4.9059E-05	0.013801284
281.359998	1090.175125	2.46037E-05	0.006922507
281.399998	612.940411	1.38332E-05	0.003892666
281.439998	471.185687	1.0634E-05	0.002992834
281.479998	556.596988	1.25616E-05	0.003535844
281.519998	726.95901	1.64065E-05	0.004618744
281.559998	814.000029	1.83708E-05	0.005172496
281.599998	776.866812	1.75328E-05	0.004937237
281.639998	635.834449	1.43499E-05	0.004041505
281.679998	462.457843	1.0437E-05	0.002939902
281.719998	324.188008	7.31647E-06	0.002061196
281.759998	260.977007	5.88989E-06	0.001659535
281.799998	287.238602	6.48258E-06	0.00182679
281.839998	451.643267	1.0193E-05	0.002872784
281.879998	826.136691	1.86448E-05	0.005255583
281.919998	1531.613671	3.45664E-05	0.009744956
281.959998	2732.134871	6.16605E-05	0.017385788
281.999998	4474.857378	0.000100991	0.028479546
282.039998	6642.452923	0.000149911	0.042280873
282.079998	8497.931639	0.000191786	0.05409913
282.119998	9089.670377	0.000205141	0.057874435
282.159998	8140.44877	0.000183719	0.051838037
282.199998	6200.829892	0.000139944	0.039492223
282.239998	4164.542759	9.39879E-05	0.026527153
282.279998	2477.057827	5.59037E-05	0.015780509
282.319998	1338.93287	3.02178E-05	0.008531103
282.359998	713.584768	1.61046E-05	0.004547299
282.399998	443.864691	1.00174E-05	0.002828916
282.439998	372.480773	8.40637E-06	0.002374296
282.479998	386.837223	8.73038E-06	0.002466157

282.519998	471.359407	1.06379E-05	0.003005427
282.559998	556.421929	1.25577E-05	0.003548295
282.599998	569.827507	1.28602E-05	0.003634296
282.639998	492.907076	1.11242E-05	0.003144151
282.679998	374.527698	8.45257E-06	0.002389372
282.719998	276.511178	6.24047E-06	0.001764306
282.759998	241.279129	5.44533E-06	0.001539723
282.799998	293.623402	6.62667E-06	0.001874023
282.839998	471.221467	1.06348E-05	0.00300795
282.879998	899.226236	2.02943E-05	0.005740847
282.919998	1842.453083	4.15816E-05	0.011764266
282.959998	3647.96756	8.23295E-05	0.023295968
282.999998	6497.172806	0.000146632	0.041496895
283.039998	9940.908559	0.000224352	0.06350072
283.079998	12823.23221	0.000289402	0.081924056
283.119998	13899.18488	0.000313685	0.088810565
283.159998	12765.06663	0.00028809	0.0815755
283.199998	10161.42974	0.000229329	0.064946062
283.239998	7248.121413	0.00016358	0.0463324
283.279998	4663.837814	0.000105256	0.029817011
283.319998	2667.086119	6.01924E-05	0.017053716
283.359998	1384.769917	3.12523E-05	0.00885566
283.399998	732.805442	1.65384E-05	0.004686982
283.439998	483.501397	1.0912E-05	0.003092884
283.479998	457.392122	1.03227E-05	0.00292628
283.519998	510.135093	1.1513E-05	0.003264177
283.559998	562.521551	1.26953E-05	0.003599887
283.599998	576.455237	1.30098E-05	0.003689577
283.639998	527.785523	1.19114E-05	0.003378545
283.679998	414.778471	9.36097E-06	0.002655521
283.719998	299.765969	6.7653E-06	0.001919451
283.759998	238.466633	5.38186E-06	0.001527157
283.799998	245.493993	5.54046E-06	0.001572382
283.839998	354.854182	8.00856E-06	0.002273151
283.879998	626.455205	1.41382E-05	0.004013558
283.919998	1220.081235	2.75355E-05	0.007817889
283.959998	2396.373798	5.40828E-05	0.015357357
283.999998	4326.517497	9.76435E-05	0.027730746
284.039998	6878.741924	0.000155244	0.044095399
284.079998	9161.880232	0.000206771	0.05873947
284.119998	10338.87873	0.000233334	0.066294882
284.159998	10633.25748	0.000239978	0.068192094
284.199998	10853.32341	0.000244944	0.069613196
284.239998	11500.42244	0.000259549	0.07377407
284.279998	12101.47824	0.000273114	0.077640707
284.319998	11515.10908	0.00025988	0.073889073

284.359998	9464.09082	0.000213591	0.060736835
284.399998	6633.299231	0.000149704	0.04257591
284.439998	4015.702471	9.06288E-05	0.025778459
284.479998	2266.27473	5.11467E-05	0.014550203
284.519998	1394.438322	3.14705E-05	0.008953995
284.559998	1031.87174	2.32879E-05	0.006626807
284.599998	851.916638	1.92266E-05	0.005471882
284.639998	677.335644	1.52865E-05	0.004351156
284.679998	488.501912	1.10248E-05	0.003138542
284.719998	333.299667	7.52211E-06	0.002141695
284.759998	248.050416	5.59815E-06	0.00159413
284.799998	279.036154	6.29746E-06	0.001793516
284.839998	588.315055	1.32775E-05	0.003781949
284.879998	1583.611399	3.57399E-05	0.010181584
284.919998	4007.550324	9.04448E-05	0.02576954
284.959998	8530.856128	0.00019253	0.054863217
284.999998	14720.93557	0.000332231	0.094685838
285.039998	20322.93442	0.00045866	0.130736537
285.079998	23119.75619	0.000521781	0.148749239
285.119998	22479.45622	0.00050733	0.144649933
285.159998	19017.39245	0.000429196	0.122389547
285.199998	14504.46325	0.000327346	0.093358945
285.239998	10320.04151	0.000232909	0.066434952
285.279998	6996.318866	0.000157897	0.045044905
285.319998	4678.01578	0.000105576	0.03012303
285.359998	3085.06654	6.96257E-05	0.019868377
285.399998	1955.425807	4.41312E-05	0.012595055
285.439998	1209.878482	2.73053E-05	0.007794017
285.479998	830.618616	1.87459E-05	0.005351581
285.519998	752.70792	1.69876E-05	0.004850291
285.559998	757.959531	1.71061E-05	0.004884815
285.599998	739.208217	1.66829E-05	0.004764636
285.639998	656.009002	1.48052E-05	0.00422896
285.679998	493.130521	1.11293E-05	0.00317941
285.719998	349.613118	7.89028E-06	0.002254411
285.759998	302.960052	6.83739E-06	0.001953852
285.799998	353.172071	7.9706E-06	0.002277998
285.839998	527.598364	1.19072E-05	0.003403543
285.879998	970.021072	2.1892E-05	0.006258492
285.919998	1876.468257	4.23493E-05	0.012108505
285.959998	3469.854202	7.83098E-05	0.022393463
285.999998	5791.678804	0.00013071	0.037383095
286.039998	8299.375135	0.000187305	0.053576818
286.079998	9989.099717	0.00022544	0.064493894
286.119998	10062.84683	0.000227104	0.064979121
286.159998	8581.236759	0.000193667	0.055419623

286.199998	6317.174005	0.00014257	0.040803482
286.239998	4055.896827	9.15359E-05	0.026201248
286.279998	2348.394395	5.3E-05	0.015172837
286.319998	1267.290141	2.8601E-05	0.008189031
286.359998	701.678944	1.58359E-05	0.004534773
286.399998	477.778483	1.07828E-05	0.003088192
286.439998	462.680523	1.04421E-05	0.002991022
286.479998	604.140302	1.36346E-05	0.003906042
286.519998	799.554025	1.80448E-05	0.005170202
286.559998	927.051377	2.09223E-05	0.005995482
286.599998	902.979459	2.0379E-05	0.005840618
286.639998	744.211813	1.67958E-05	0.004814355
286.679998	534.643404	1.20662E-05	0.003459126
286.719998	414.097474	9.3456E-06	0.002679571
286.759998	479.370293	1.08187E-05	0.003102376
286.799998	1161.651407	2.62169E-05	0.007518994
286.839998	3988.252612	9.00093E-05	0.025818269
286.879998	12678.51439	0.000286136	0.082086813
286.919998	32504.19347	0.000733574	0.210477157
286.959998	65660.54128	0.001481867	0.42523654
286.999998	104433.7844	0.002356925	0.676437484
287.039998	131398.6628	0.002965485	0.85121275
287.079998	131365.2039	0.00296473	0.851114589
287.119998	106420.6088	0.002401765	0.689594736
287.159998	71120.59561	0.001605093	0.46091844
287.199998	40270.00293	0.000908838	0.261018236
287.239998	19716.09628	0.000444965	0.127811697
287.279998	8669.227348	0.000195652	0.056207017
287.319998	3459.291691	7.80714E-05	0.022431473
287.359998	1320.392223	2.97994E-05	0.008563159
287.399998	618.806892	1.39656E-05	0.004013716
287.439998	466.781956	1.05346E-05	0.003028071
287.479998	523.991638	1.18258E-05	0.00339967
287.519998	649.732746	1.46636E-05	0.004216068
287.559998	741.514054	1.67349E-05	0.004812299
287.599998	736.182976	1.66146E-05	0.004778366
287.639998	661.039924	1.49188E-05	0.004291229
287.679998	522.917863	1.18015E-05	0.003395063
287.719998	381.663236	8.61361E-06	0.002478307
287.759998	317.481928	7.16512E-06	0.002061836
287.799998	384.628342	8.68053E-06	0.002498255
287.839998	802.444108	1.811E-05	0.005212796
287.879998	2156.928393	4.86789E-05	0.014013673
287.919998	5603.924735	0.000126473	0.036414041
287.959998	11943.30746	0.000269544	0.077617836
287.999998	20109.63424	0.000453846	0.130707772

288.039998	26908.71349	0.000607292	0.174924439
288.079998	28778.22701	0.000649484	0.187103493
288.119998	25033.14393	0.000564963	0.162777193
288.159998	18117.24381	0.000408881	0.117823136
288.199998	11220.00987	0.00025322	0.072978002
288.239998	6063.531972	0.000136845	0.039444338
288.279998	2923.178806	6.59721E-05	0.019018429
288.319998	1330.78044	3.00339E-05	0.008659363
288.359998	726.423258	1.63944E-05	0.004727478
288.399998	654.081666	1.47617E-05	0.004257278
288.439998	936.611229	2.1138E-05	0.006097048
288.479998	1340.848156	3.02611E-05	0.008729715
288.519998	1626.03829	3.66974E-05	0.010587939
288.559998	1595.334295	3.60045E-05	0.010389451
288.599998	1268.110714	2.86195E-05	0.008259585
288.639998	865.869719	1.95415E-05	0.005640451
288.679998	545.734604	1.23165E-05	0.003555518
288.719998	353.664674	7.98172E-06	0.002304482
288.759998	293.634569	6.62692E-06	0.00191359
288.799998	385.972036	8.71085E-06	0.002515694
288.839998	804.710387	1.81612E-05	0.005245679
288.879998	2163.054171	4.88171E-05	0.014102289
288.919998	5552.586706	0.000125314	0.036205763
288.959998	11950.22294	0.0002697	0.077932479
288.999998	20780.03448	0.000468976	0.135534188
289.039998	29432.22089	0.000664244	0.191993151
289.079998	33923.00481	0.000765595	0.221318192
289.119998	32309.45817	0.000729179	0.210820366
289.159998	25911.4839	0.000584786	0.169096747
289.199998	17785.21453	0.000401388	0.11608127
289.239998	10724.82092	0.000242044	0.070008884
289.279998	5740.207919	0.000129548	0.037475789
289.319998	2692.452146	6.07649E-05	0.017580499
289.359998	1181.621554	2.66676E-05	0.007716523
289.399998	597.077294	1.34752E-05	0.003899724
289.439998	438.262745	9.89098E-06	0.002862845
289.479998	507.704802	1.14582E-05	0.003316917
289.519998	716.11937	1.61618E-05	0.004679169
289.559998	932.482967	2.10448E-05	0.006093744
289.599998	1035.32137	2.33658E-05	0.006766724
289.639998	950.829699	2.14589E-05	0.006215356
289.679998	687.155241	1.55081E-05	0.004492397
289.719998	430.199131	9.709E-06	0.00281289
289.759998	284.735385	6.42608E-06	0.001862021
289.799998	244.864346	5.52625E-06	0.001601506
289.839998	330.772575	7.46508E-06	0.002163678

289.879998	666.824887	1.50493E-05	0.004362494
289.919998	1532.824366	3.45937E-05	0.010029409
289.959998	3292.079514	7.42976E-05	0.021543346
289.999998	5923.787858	0.000133692	0.038770576
290.039998	8924.697821	0.000201418	0.058419276
290.079998	11149.89705	0.000251638	0.072995048
290.119998	11366.48513	0.000256526	0.074423246
290.159998	9641.341635	0.000217592	0.063136393
290.199998	6911.705142	0.000155988	0.045267589
290.239998	4279.235266	9.65764E-05	0.028030328
290.279998	2334.089801	5.26772E-05	0.015291125
290.319998	1134.507876	2.56043E-05	0.007433429
290.359998	569.404604	1.28507E-05	0.00373132
290.399998	380.182383	8.58019E-06	0.002491686
290.439998	383.130409	8.64672E-06	0.002511353
290.479998	482.379271	1.08866E-05	0.003162348
290.519998	565.91234	1.27719E-05	0.003710479
290.559998	608.14958	1.37251E-05	0.003987962
290.599998	576.431027	1.30092E-05	0.003780487
290.639998	480.301723	1.08397E-05	0.003150462
290.679998	373.671944	8.43326E-06	0.002451379
290.719998	278.494666	6.28524E-06	0.001827244
290.759998	226.966341	5.12231E-06	0.001489364
290.799998	236.242469	5.33166E-06	0.001550448
290.839998	325.487498	7.3458E-06	0.002136452
290.879998	642.549338	1.45014E-05	0.00421818
290.919998	1496.945328	3.3784E-05	0.009828433
290.959998	3337.078341	7.53132E-05	0.021913131
290.999998	6486.869215	0.0001464	0.042602284
291.039998	10603.20781	0.0002393	0.069645764
291.079998	14722.80284	0.000332273	0.096718069
291.119998	17626.7578	0.000397811	0.115810846
291.159998	18290.6637	0.000412795	0.120189334
291.199998	16497.03479	0.000372315	0.108418155
291.239998	12769.8413	0.000288198	0.083934648
291.279998	8446.49062	0.000190626	0.055525404
291.319998	4755.924063	0.000107335	0.03126871
291.359998	2341.736145	5.28497E-05	0.015398295
291.399998	1088.973052	2.45766E-05	0.007161623
291.439998	579.903738	1.30876E-05	0.003814256
291.479998	477.881875	1.07851E-05	0.003143649
291.519998	532.195781	1.20109E-05	0.003501423
291.559998	585.386697	1.32114E-05	0.003851905
291.599998	588.828419	1.3289E-05	0.003875083
291.639998	521.690878	1.17738E-05	0.003433722
291.679998	382.442505	8.63119E-06	0.002517547

291.719998	267.424057	6.03539E-06	0.001760644
291.759998	243.509143	5.49566E-06	0.001603414
291.799998	287.599862	6.49073E-06	0.001893995
291.839998	390.960725	8.82344E-06	0.002575032
291.879998	618.880425	1.39673E-05	0.004076766
291.919998	1187.38954	2.67977E-05	0.007822793
291.959998	2378.653183	5.36829E-05	0.015673256
291.999998	4254.682796	9.60223E-05	0.028038501
292.039998	6469.843316	0.000146015	0.042642323
292.079998	8318.869746	0.000187745	0.054836648
292.119998	9167.195788	0.000206891	0.060436952
292.159998	8689.539078	0.000196111	0.057295729
292.199998	7153.26417	0.000161439	0.047172536
292.239998	5120.009134	0.000115551	0.033768763
292.279998	3183.828422	7.18546E-05	0.021001654
292.319998	1765.664041	3.98486E-05	0.011648536
292.359998	953.377191	2.15164E-05	0.006290533
292.399998	608.123441	1.37245E-05	0.004013043
292.439998	584.605358	1.31937E-05	0.003858374
292.479998	746.439467	1.68461E-05	0.004927147
292.519998	1000.698118	2.25844E-05	0.006606378
292.559998	1191.889751	2.68993E-05	0.007869657
292.599998	1108.493755	2.50172E-05	0.007320021
292.639998	848.915029	1.91588E-05	0.00560664
292.679998	579.745106	1.3084E-05	0.003829437
292.719998	398.817461	9.00075E-06	0.002634701
292.759998	330.595352	7.46108E-06	0.002184305
292.799998	366.680759	8.27547E-06	0.002423059
292.839998	513.291197	1.15843E-05	0.003392337
292.879998	921.217992	2.07906E-05	0.006089153
292.919998	1850.924584	4.17728E-05	0.012236085
292.959998	3727.396526	8.41221E-05	0.024644424
292.999998	6803.433458	0.000153544	0.044988397
293.039998	10684.02995	0.000241124	0.070658881
293.079998	14582.49689	0.000329107	0.096454574
293.119998	17557.12854	0.00039624	0.116145849
293.159998	18879.46774	0.000426083	0.124910576
293.199998	18004.81532	0.000406344	0.119139944
293.239998	14974.83296	0.000337961	0.099103719
293.279998	10632.758	0.000239967	0.070377386
293.319998	6433.737541	0.0001452	0.042590209
293.359998	3387.589019	7.64532E-05	0.022428301
293.399998	1623.996019	3.66513E-05	0.0107535
293.439998	835.863136	1.88643E-05	0.00553553
293.479998	659.564946	1.48855E-05	0.004368585
293.519998	745.761549	1.68308E-05	0.004940176

293.559998	830.967385	1.87538E-05	0.005505359
293.599998	809.476409	1.82688E-05	0.005363706
293.639998	662.961352	1.49621E-05	0.004393475
293.679998	491.997527	1.11037E-05	0.003260934
293.719998	345.604719	7.79982E-06	0.002290962
293.759998	255.467855	5.76555E-06	0.001693689
293.799998	268.251536	6.05406E-06	0.001778684
293.839998	368.363457	8.31345E-06	0.002442824
293.879998	612.605467	1.38257E-05	0.004063083
293.919998	1129.203688	2.54846E-05	0.00749042
293.959998	2058.227213	4.64513E-05	0.01365483
293.999998	3629.943724	8.19228E-05	0.024085295
294.039998	5771.730941	0.00013026	0.038301629
294.079998	7875.825983	0.000177746	0.052271668
294.119998	9114.413422	0.0002057	0.060500371
294.159998	9131.319319	0.000206081	0.060620833
294.199998	7977.032654	0.000180031	0.052964977
294.239998	6120.115004	0.000138122	0.040641156
294.279998	4131.108139	9.32334E-05	0.027436712
294.319998	2436.796018	5.49951E-05	0.016186155
294.359998	1305.738758	2.94687E-05	0.008674408
294.399998	724.317199	1.63468E-05	0.004812507
294.439998	500.603543	1.12979E-05	0.003326561
294.479998	464.076123	1.04736E-05	0.003084251
294.519998	538.26906	1.2148E-05	0.003577824
294.559998	655.410579	1.47917E-05	0.004357044
294.599998	706.450422	1.59436E-05	0.004696985
294.639998	633.138511	1.42891E-05	0.004210127
294.679998	472.085395	1.06543E-05	0.003139612
294.719998	353.009825	7.96694E-06	0.002348017
294.759998	336.722784	7.59936E-06	0.002239989
294.799998	487.510575	1.10024E-05	0.003243518
294.839998	876.378038	1.97786E-05	0.005831532
294.879998	1610.149134	3.63388E-05	0.010715592
294.919998	2840.45663	6.41051E-05	0.018905891
294.959998	4861.745433	0.000109723	0.032363844
294.999998	7846.860759	0.000177093	0.052242351
295.039998	11521.60465	0.000260027	0.076718238
295.079998	15275.0627	0.000344737	0.10172496
295.119998	18152.31443	0.000409672	0.120902533
295.159998	19482.9407	0.000439703	0.129782688
295.199998	18710.3455	0.000422266	0.124653051
295.239998	15899.62126	0.000358832	0.105941649
295.279998	11757.99458	0.000265362	0.078355961
295.319998	7469.217412	0.00016857	0.049782043
295.359998	4096.799406	9.24591E-05	0.027308707

295.399998	2011.210299	4.53902E-05	0.013408269
295.439998	967.423797	2.18334E-05	0.006450462
295.479998	598.711188	1.35121E-05	0.003992549
295.519998	573.925028	1.29527E-05	0.003827778
295.559998	650.20675	1.46743E-05	0.004337124
295.599998	661.719591	1.49341E-05	0.004414517
295.639998	576.025962	1.30001E-05	0.003843351
295.679998	437.822263	9.88104E-06	0.002921625
295.719998	314.023313	7.08707E-06	0.002095788
295.759998	235.599538	5.31715E-06	0.001572601
295.799998	234.848627	5.30021E-06	0.001567801
295.839998	349.985835	7.89869E-06	0.002336749
295.879998	609.377166	1.37528E-05	0.004069177
295.919998	1119.550291	2.52667E-05	0.007476919
295.959998	2124.592105	4.79491E-05	0.014191011
295.999998	3697.59969	8.34497E-05	0.024701104
296.039998	5761.305654	0.000130025	0.038492495
296.079998	7838.007552	0.000176893	0.052374453
296.119998	9051.056114	0.00020427	0.060488352
296.159998	8831.296873	0.00019931	0.05902767
296.199998	7456.838633	0.00016829	0.049847634
296.239998	5542.181116	0.000125079	0.037053494
296.279998	3668.256992	8.27875E-05	0.024528266
296.319998	2177.478015	4.91426E-05	0.014561949
296.359998	1199.305903	2.70667E-05	0.008021476
296.399998	698.619073	1.57669E-05	0.004673297
296.439998	543.909405	1.22753E-05	0.003638883
296.479998	600.768506	1.35585E-05	0.004019827
296.519998	708.014155	1.59789E-05	0.004738061
296.559998	764.76539	1.72597E-05	0.005118534
296.599998	750.19343	1.69308E-05	0.005021681
296.639998	660.603196	1.49089E-05	0.004422574
296.679998	531.082298	1.19858E-05	0.003555943
296.719998	424.673964	9.5843E-06	0.002843853
296.759998	383.513037	8.65536E-06	0.002568563
296.799998	430.979883	9.72662E-06	0.00288686
296.839998	640.522022	1.44557E-05	0.004291027
296.879998	1088.226699	2.45598E-05	0.007291302
296.919998	1903.28708	4.29545E-05	0.012754061
296.959998	3380.557772	7.62945E-05	0.022656409
296.999998	5741.866084	0.000129586	0.038487018
297.039998	8751.159187	0.000197501	0.058665837
297.079998	11504.65824	0.000259644	0.077135072
297.119998	13033.4038	0.000294146	0.087396591
297.159998	12898.37034	0.000291098	0.086502756
297.199998	11179.59621	0.000252308	0.074985912

297.239998	8604.26098	0.000194186	0.057719901
297.279998	5868.497839	0.000132444	0.039372899
297.319998	3534.967896	7.97793E-05	0.023719982
297.359998	1901.047155	4.2904E-05	0.012757929
297.399998	1007.198576	2.27311E-05	0.00676022
297.439998	619.9062	1.39904E-05	0.00416131
297.479998	527.475273	1.19044E-05	0.003541316
297.519998	579.39894	1.30762E-05	0.003890439
297.559998	683.051619	1.54155E-05	0.004587043
297.599998	727.144601	1.64106E-05	0.004883807
297.639998	643.942783	1.45329E-05	0.00432557
297.679998	493.118628	1.1129E-05	0.003312881
297.719998	343.072227	7.74266E-06	0.002305145
297.759998	258.290993	5.82927E-06	0.001735723
297.799998	251.18432	5.66888E-06	0.001688193
297.839998	346.088284	7.81073E-06	0.002326348
297.879998	543.598019	1.22683E-05	0.003654466
297.919998	931.779175	2.1029E-05	0.006264947
297.959998	1766.492738	3.98673E-05	0.011878855
297.999998	3150.801968	7.11092E-05	0.021190545
298.039998	4919.470855	0.000111026	0.033090071
298.079998	6569.856809	0.000148273	0.044197072
298.119998	7436.974427	0.000167842	0.050037103
298.159998	7051.71728	0.000159147	0.047451399
298.199998	5670.402597	0.000127973	0.038161575
298.239998	4012.098025	9.05475E-05	0.027004875
298.279998	2594.146399	5.85463E-05	0.017463182
298.319998	1540.315875	3.47628E-05	0.010370434
298.359998	870.654302	1.96495E-05	0.005862611
298.399998	536.606931	1.21105E-05	0.003613765
298.439998	423.553742	9.55902E-06	0.002852793
298.479998	431.838552	9.74599E-06	0.002908984
298.519998	510.874744	1.15297E-05	0.003441855
298.559998	586.790942	1.32431E-05	0.003953846
298.599998	572.154787	1.29127E-05	0.003855743
298.639998	493.003576	1.11264E-05	0.003322789
298.679998	386.727848	8.72791E-06	0.002606852
298.719998	301.67482	6.80838E-06	0.002033799
298.759998	246.544437	5.56416E-06	0.00166235
298.799998	241.794108	5.45696E-06	0.001630539
298.839998	342.109728	7.72094E-06	0.002307326
298.879998	641.759103	1.44836E-05	0.004328861
298.919998	1343.319309	3.03168E-05	0.009062311
298.959998	2763.449961	6.23672E-05	0.018645303
298.999998	5026.503185	0.000113441	0.033918911
299.039998	8088.425367	0.000182544	0.054588105

299.079998	11138.3511	0.000251377	0.075181854
299.119998	13041.13616	0.00029432	0.088037081
299.159998	13109.26344	0.000295858	0.088508824
299.199998	11433.198	0.000258031	0.077202981
299.239998	8705.986255	0.000196482	0.058795268
299.279998	5799.58795	0.000130889	0.039172346
299.319998	3346.492799	7.55257E-05	0.022606347
299.359998	1701.788559	3.8407E-05	0.011497519
299.399998	851.632667	1.92202E-05	0.005754517
299.439998	528.237648	1.19216E-05	0.0035698
299.479998	494.627688	1.11631E-05	0.003343113
299.519998	558.853948	1.26126E-05	0.003777713
299.559998	646.703247	1.45952E-05	0.004372136
299.599998	676.498701	1.52676E-05	0.004574183
299.639998	620.412248	1.40018E-05	0.004195511
299.679998	500.769476	1.13017E-05	0.003386884
299.719998	365.832345	8.25633E-06	0.002474586
299.759998	271.293963	6.12273E-06	0.001835349
299.799998	255.801038	5.77307E-06	0.001730767
299.839998	331.458058	7.48055E-06	0.002242967
299.879998	539.819753	1.2183E-05	0.003653432
299.919998	996.356109	2.24864E-05	0.006744112
299.959998	1915.123833	4.32217E-05	0.012964774
299.999998	3414.848211	7.70684E-05	0.02312051
300.039998	5256.246619	0.000118626	0.035592594
300.079998	6934.995015	0.000156513	0.046966474
300.119998	7754.125262	0.000175	0.052520942
300.159998	7394.18454	0.000166876	0.050089634
300.199998	6069.818775	0.000136987	0.041123604
300.239998	4243.504732	9.577E-05	0.028753982
300.279998	2546.775808	5.74772E-05	0.017259248
300.319998	1386.004132	3.12802E-05	0.009394064
300.359998	763.662167	1.72348E-05	0.005176642
300.399998	498.554619	1.12517E-05	0.003380006
300.439998	435.689446	9.8329E-06	0.002954198
300.479998	468.038249	1.0563E-05	0.003173962
300.519998	575.660905	1.29919E-05	0.003904315
300.559998	714.215598	1.61189E-05	0.004844682
300.599998	769.819157	1.73737E-05	0.005222548
300.639998	686.294483	1.54887E-05	0.004656526
300.679998	517.012579	1.16683E-05	0.003508411
300.719998	368.888842	8.32531E-06	0.002503586
300.759998	315.629961	7.12333E-06	0.002142412
300.799998	489.94344	1.10573E-05	0.003326048
300.839998	1317.071831	2.97245E-05	0.008942312
300.879998	4048.299999	9.13645E-05	0.027489748

300.919998	11312.73508	0.000255313	0.076828689
300.959998	25339.81392	0.000571884	0.172114299
300.999998	44726.08834	0.001009406	0.303831058
301.039998	62624.84833	0.001413356	0.425476566
301.079998	69785.28535	0.001574957	0.474187956
301.119998	63105.76043	0.001424209	0.428857846
301.159998	47198.99046	0.001065215	0.320800289
301.199998	29940.22257	0.000675709	0.203523586
301.239998	16422.81528	0.00037064	0.111651613
301.279998	8063.89096	0.000181991	0.054830184
301.319998	3673.953184	8.2916E-05	0.024984251
301.359998	1601.8106	3.61506E-05	0.010894355
301.399998	783.330349	1.76787E-05	0.005328353
301.439998	581.812135	1.31307E-05	0.003958115
301.479998	679.486921	1.53351E-05	0.004623218
301.519998	851.201249	1.92104E-05	0.005792328
301.559998	958.669988	2.16358E-05	0.006524506
301.599998	922.812221	2.08266E-05	0.006281298
301.639998	755.170502	1.70431E-05	0.005140895
301.679998	533.968179	1.20509E-05	0.003635521
301.719998	374.001199	8.44069E-06	0.002546724
301.759998	317.241396	7.1597E-06	0.00216051
301.799998	346.56519	7.82149E-06	0.002360527
301.839998	543.328897	1.22622E-05	0.003701215
301.879998	1197.968184	2.70365E-05	0.008161771
301.919998	2790.088858	6.29684E-05	0.019011425
301.959998	5899.346187	0.00013314	0.040202962
301.999998	10543.16514	0.000237945	0.071859254
302.039998	15319.7849	0.000345746	0.104429183
302.079998	17857.50916	0.000403019	0.121744011
302.119998	16974.59107	0.000383093	0.115740019
302.159998	13499.26684	0.00030466	0.092055959
302.199998	9234.19656	0.000208403	0.062979372
302.239998	5583.920173	0.000126021	0.038088678
302.279998	3057.123256	6.8995E-05	0.020855813
302.319998	1542.155641	3.48043E-05	0.010522037
302.359998	765.643387	1.72795E-05	0.005224631
302.399998	451.079895	1.01802E-05	0.003078506
302.439998	373.040474	8.419E-06	0.002546244
302.479998	419.81029	9.47453E-06	0.002865857
302.519998	534.983706	1.20738E-05	0.003652577
302.559998	620.986844	1.40148E-05	0.00424032
302.599998	598.818614	1.35145E-05	0.004089488
302.639998	517.341942	1.16757E-05	0.00353353
302.679998	425.683407	9.60708E-06	0.002907871
302.719998	336.94313	7.60434E-06	0.002301985

302.759998	300.682758	6.78599E-06	0.002054527
302.799998	432.733891	9.7662E-06	0.002957206
302.839998	956.413673	2.15849E-05	0.006536778
302.879998	2626.681374	5.92805E-05	0.01795489
302.919998	6843.964053	0.000154459	0.04678864
302.959998	14789.07092	0.000333769	0.101118574
302.999998	25731.06796	0.000580714	0.175956451
303.039998	35944.67731	0.000811221	0.245832501
303.079998	40588.97562	0.000916037	0.277632384
303.119998	37903.62865	0.000855432	0.259298577
303.159998	29898.52659	0.000674768	0.204562696
303.199998	20395.09964	0.000460289	0.139559621
303.239998	12338.90363	0.000278472	0.084443809
303.279998	6748.329038	0.0001523	0.046189661
303.319998	3298.13084	7.44342E-05	0.022577387
303.359998	1481.462469	3.34345E-05	0.010142703
303.399998	707.648248	1.59706E-05	0.004845491
303.439998	453.737522	1.02402E-05	0.003107294
303.479998	458.120531	1.03391E-05	0.003137723
303.519998	576.721773	1.30158E-05	0.003950558
303.559998	679.344451	1.53319E-05	0.004654139
303.599998	668.259474	1.50817E-05	0.0045788
303.639998	560.436062	1.26483E-05	0.003840518
303.679998	436.76286	9.85713E-06	0.002993413
303.719998	320.264124	7.22792E-06	0.002195262
303.759998	249.176889	5.62358E-06	0.001708217
303.799998	257.187135	5.80436E-06	0.001763363
303.839998	378.78865	8.54873E-06	0.002597447
303.879998	739.537115	1.66903E-05	0.005071855
303.919998	1675.375496	3.78109E-05	0.011491486
303.959998	3622.126794	8.17464E-05	0.024847622
303.999998	6708.579065	0.000151403	0.046026598
304.039998	10306.84884	0.000232611	0.070723115
304.079998	12941.70313	0.000292076	0.088814534
304.119998	13539.1896	0.000305561	0.092927104
304.159998	11907.38693	0.000268733	0.081737871
304.199998	8811.765797	0.000198869	0.060496035
304.239998	5583.763745	0.000126018	0.038339646
304.279998	3091.907848	6.97801E-05	0.021232675
304.319998	1559.262001	3.51904E-05	0.010709134
304.359998	806.427253	1.81999E-05	0.005539334
304.399998	494.827796	1.11676E-05	0.00339941
304.439998	394.868604	8.91163E-06	0.002713058
304.479998	412.029589	9.29893E-06	0.002831339
304.519998	481.803122	1.08736E-05	0.003311236
304.559998	617.097399	1.3927E-05	0.004241616

304.599998	712.095366	1.6071E-05	0.004895227
304.639998	658.983608	1.48723E-05	0.00453071
304.679998	516.478849	1.16562E-05	0.003551413
304.719998	379.987343	8.57579E-06	0.002613213
304.759998	293.518081	6.62429E-06	0.00201882
304.799998	292.509647	6.60154E-06	0.002012148
304.839998	457.791375	1.03317E-05	0.00314952
304.879998	921.544996	2.0798E-05	0.00634089
304.919998	2091.842406	4.721E-05	0.014395264
304.959998	4619.225003	0.000104249	0.031791919
304.999998	8584.828513	0.000193748	0.059093024
305.039998	13090.15785	0.000295427	0.090116939
305.079998	16543.61237	0.000373366	0.113906586
305.119998	17527.04378	0.000395561	0.120693561
305.159998	15749.38248	0.000355442	0.108466566
305.199998	12302.38978	0.000277648	0.084738108
305.239998	8357.600134	0.000188619	0.057574183
305.279998	4935.815206	0.000111394	0.034006505
305.319998	2578.312595	5.81889E-05	0.017766242
305.359998	1252.406914	2.82651E-05	0.008631025
305.399998	635.711557	1.43471E-05	0.004381612
305.439998	431.579259	9.74014E-06	0.002975029
305.479998	436.816094	9.85833E-06	0.003011523
305.519998	521.501262	1.17696E-05	0.003595835
305.559998	639.278147	1.44276E-05	0.004408503
305.599998	665.825208	1.50267E-05	0.004592174
305.639998	571.801698	1.29048E-05	0.003944213
305.679998	451.931427	1.01995E-05	0.003117772
305.719998	332.506213	7.5042E-06	0.002294185
305.759998	260.059279	5.86918E-06	0.001794559
305.799998	254.90713	5.7529E-06	0.001759236
305.839998	316.665869	7.14671E-06	0.002185749
305.879998	493.200269	1.11308E-05	0.003404702
305.919998	926.247554	2.09041E-05	0.006394987
305.959998	1860.955557	4.19992E-05	0.012850067
305.999998	3527.37779	7.9608E-05	0.024360049
306.039998	5662.960397	0.000127805	0.039113481
306.079998	7650.9973	0.000172672	0.052851553
306.119998	8664.595606	0.000195548	0.059861109
306.159998	8100.266197	0.000182812	0.055969637
306.199998	6369.664376	0.000143754	0.044017613
306.239998	4301.672408	9.70828E-05	0.029730623
306.279998	2565.643126	5.7903E-05	0.017734528
306.319998	1384.427565	3.12446E-05	0.009570846
306.359998	744.07714	1.67928E-05	0.005144637
306.399998	464.906417	1.04923E-05	0.003214838

306.439998	376.817357	8.50424E-06	0.00260604
306.479998	393.657248	8.8843E-06	0.002722859
306.519998	474.393299	1.07064E-05	0.003281724
306.559998	581.499975	1.31236E-05	0.004023184
306.599998	630.701968	1.42341E-05	0.004364164
306.639998	595.273217	1.34345E-05	0.004119551
306.679998	471.225517	1.06349E-05	0.003261512
306.719998	339.905045	7.67118E-06	0.002352905
306.759998	256.97411	5.79955E-06	0.001779069
306.799998	239.369464	5.40224E-06	0.001657406
306.839998	326.88203	7.37727E-06	0.002263642
306.879998	571.450555	1.28968E-05	0.003957783
306.919998	1198.005558	2.70373E-05	0.008298293
306.959998	2607.249573	5.8842E-05	0.018062138
306.999998	5101.821917	0.000115141	0.035348291
307.039998	8440.845583	0.000190498	0.058490545
307.079998	11838.53795	0.000267179	0.082045423
307.119998	14227.49131	0.000321095	0.098614592
307.159998	14670.99526	0.000331104	0.101701882
307.199998	12963.05993	0.000292558	0.08987388
307.239998	9825.149447	0.00022174	0.068127378
307.279998	6354.363142	0.000143409	0.044066756
307.319998	3506.39905	7.91345E-05	0.024319627
307.359998	1695.26881	3.82599E-05	0.011759549
307.399998	779.961098	1.76026E-05	0.00541105
307.439998	446.537307	1.00777E-05	0.003098296
307.479998	403.878341	9.11497E-06	0.002802671
307.519998	443.811367	1.00162E-05	0.003080183
307.559998	519.046562	1.17142E-05	0.003602806
307.599998	589.624806	1.3307E-05	0.004093236
307.639998	561.859957	1.26804E-05	0.003900997
307.679998	438.921223	9.90584E-06	0.003047829
307.719998	309.755063	6.99074E-06	0.002151191
307.759998	234.397036	5.29001E-06	0.001628055
307.799998	222.200521	5.01476E-06	0.001543542
307.839998	263.225914	5.94064E-06	0.001828767
307.879998	369.105688	8.3302E-06	0.002564702
307.919998	662.414196	1.49498E-05	0.004603332
307.959998	1390.845507	3.13894E-05	0.009666694
307.999998	2725.634446	6.15138E-05	0.018946242
308.039998	4540.427701	0.000102471	0.031565206
308.079998	6384.115127	0.000144081	0.044388347
308.119998	7648.992108	0.000172627	0.053189861
308.159998	7732.957484	0.000174522	0.053780724
308.199998	6616.677125	0.000149329	0.046023255
308.239998	4863.365376	0.000109759	0.033832232

308.279998	3091.10542	6.97619E-05	0.021506212
308.319998	1724.735132	3.89249E-05	0.012001316
308.359998	891.108154	2.01111E-05	0.006201449
308.399998	502.322006	1.13367E-05	0.003496241
308.439998	388.080112	8.75843E-06	0.002701449
308.479998	401.923769	9.07086E-06	0.002798179
308.519998	494.587421	1.11621E-05	0.003443746
308.559998	577.6659	1.30371E-05	0.004022732
308.599998	598.380158	1.35046E-05	0.004167522
308.639998	558.056268	1.25946E-05	0.003887183
308.679998	470.220662	1.06122E-05	0.003275782
308.719998	380.427495	8.58572E-06	0.002650583
308.759998	326.331804	7.36485E-06	0.002273972
308.799998	351.182611	7.9257E-06	0.002447457
308.839998	496.804696	1.12122E-05	0.003462773
308.879998	859.163663	1.93901E-05	0.005989222
308.919998	1639.018174	3.69904E-05	0.011427061
308.959998	3179.596459	7.17591E-05	0.02217068
308.999998	5796.380113	0.000130816	0.040422213
309.039998	9250.059625	0.000208761	0.064515487
309.079998	12822.81525	0.000289393	0.089445612
309.119998	15657.24033	0.000353362	0.109231293
309.159998	16865.5097	0.000380631	0.117675898
309.199998	15921.56065	0.000359327	0.111104046
309.239998	13178.41107	0.000297418	0.09197366
309.279998	9418.900739	0.000212571	0.065744104
309.319998	5741.297277	0.000129573	0.040079546
309.359998	3014.460061	6.80322E-05	0.02104643
309.399998	1458.824391	3.29236E-05	0.010186572
309.439998	813.234002	1.83536E-05	0.005679325
309.479998	679.60306	1.53377E-05	0.00474671
309.519998	768.445184	1.73427E-05	0.005367924
309.559998	869.493543	1.96233E-05	0.006074576
309.599998	868.64507	1.96041E-05	0.006069432
309.639998	767.705002	1.7326E-05	0.005364833
309.679998	641.877575	1.44863E-05	0.004486112
309.719998	522.396125	1.17898E-05	0.003651522
309.759998	449.284427	1.01397E-05	0.003140881
309.799998	495.375094	1.11799E-05	0.003463541
309.839998	698.238794	1.57583E-05	0.004882545
309.879998	1008.990701	2.27715E-05	0.007056437
309.919998	1415.754488	3.19516E-05	0.009902442
309.959998	2047.833353	4.62167E-05	0.014325343
309.999998	3179.675818	7.17609E-05	0.022245865
310.039998	4921.046811	0.000111061	0.034433406
310.079998	6818.107311	0.000153875	0.047713617

310.119998	8222.312941	0.000185566	0.057547774
310.159998	8759.040803	0.000197679	0.061312227
310.199998	8273.694977	0.000186726	0.057922334
310.239998	6867.394595	0.000154988	0.048083331
310.279998	4980.15239	0.000112395	0.034873952
310.319998	3211.635885	7.24821E-05	0.02249266
310.359998	1921.586148	4.33675E-05	0.013459544
310.399998	1132.332517	2.55552E-05	0.007932324
310.439998	739.376319	1.66867E-05	0.005180217
310.479998	638.890056	1.44189E-05	0.004476767
310.519998	667.180478	1.50573E-05	0.004675603
310.559998	726.375315	1.63933E-05	0.005091097
310.599998	722.911836	1.63151E-05	0.005067474
310.639998	640.752302	1.44609E-05	0.00449213
310.679998	513.321707	1.1585E-05	0.003599214
310.719998	376.007107	8.48596E-06	0.002636756
310.759998	292.962788	6.61176E-06	0.002054671
310.799998	291.956639	6.58905E-06	0.002047878
310.839998	407.682373	9.20082E-06	0.002859984
310.879998	679.034899	1.53249E-05	0.004764196
310.919998	1274.597212	2.87659E-05	0.008943889
310.959998	2609.120481	5.88842E-05	0.018310635
310.999998	5110.538518	0.000115338	0.035870036
311.039998	8528.083251	0.000192467	0.059864923
311.079998	12150.92186	0.000274229	0.085307275
311.119998	15094.0059	0.000340651	0.105983242
311.159998	16451.46185	0.000371287	0.115529531
311.199998	15796.25784	0.0003565	0.110942655
311.239998	13354.17296	0.000301385	0.093803093
311.279998	9865.021983	0.00022264	0.069303318
311.319998	6366.11955	0.000143674	0.04472873
311.359998	3541.017143	7.99158E-05	0.024882591
311.399998	1775.791936	4.00772E-05	0.012480025
311.439998	923.17013	2.08347E-05	0.006488748
311.479998	642.891811	1.45092E-05	0.004519317
311.519998	629.918518	1.42164E-05	0.004428688
311.559998	669.907555	1.51189E-05	0.004710438
311.599998	701.112089	1.58231E-05	0.004930485
311.639998	646.567941	1.45921E-05	0.004547494
311.679998	526.012157	1.18714E-05	0.003700066
311.719998	402.742872	9.08935E-06	0.002833331
311.759998	313.129019	7.06689E-06	0.002203172
311.799998	304.063732	6.86229E-06	0.002139664
311.839998	405.054254	9.14151E-06	0.002850689
311.879997	592.409518	1.33699E-05	0.004169791
311.919997	898.570503	2.02795E-05	0.006325577

311.959997	1494.892936	3.37377E-05	0.010524798
311.999997	2701.995933	6.09803E-05	0.019025849
312.039997	4555.385309	0.000102809	0.032080427
312.079997	6584.736582	0.000148608	0.046377688
312.119997	8045.156039	0.000181568	0.056670993
312.159997	8300.303068	0.000187326	0.058475771
312.199997	7436.828544	0.000167839	0.052399292
312.239997	5977.948957	0.000134914	0.042125541
312.279997	4278.646766	9.65631E-05	0.030154724
312.319997	2735.110057	6.17276E-05	0.019278771
312.359997	1611.014763	3.63584E-05	0.011356897
312.399997	925.95597	2.08975E-05	0.00652839
312.439997	591.809556	1.33563E-05	0.004173048
312.479997	508.401577	1.14739E-05	0.003585369
312.519997	576.233186	1.30048E-05	0.004064254
312.559997	736.84373	1.66295E-05	0.005197728
312.599997	876.671182	1.97852E-05	0.006184868
312.639997	824.713524	1.86126E-05	0.005819054
312.679997	632.489732	1.42744E-05	0.004463323
312.719997	474.615368	1.07114E-05	0.003349671
312.759997	410.512192	9.26469E-06	0.002897624
312.799997	467.209974	1.05443E-05	0.00329825
312.839997	686.095953	1.54842E-05	0.004844086
312.879997	1138.085589	2.5685E-05	0.008036325
312.919997	1973.737489	4.45445E-05	0.013938867
312.959997	3450.457908	7.7872E-05	0.02437083
312.999997	5951.780441	0.000134323	0.042043222
313.039997	9531.846658	0.000215121	0.067341321
313.079997	13480.12344	0.000304228	0.095247579
313.119997	16558.57112	0.000373704	0.11701416
313.159997	17633.57203	0.000397965	0.124626768
313.199997	16405.24049	0.000370243	0.11596024
313.239997	13534.38511	0.000305452	0.095679852
313.279997	9910.621248	0.000223669	0.070070998
313.319997	6414.359242	0.000144763	0.045357191
313.359997	3688.462096	8.32435E-05	0.026085168
313.399997	1959.70502	4.42278E-05	0.013860996
313.439997	1043.092289	2.35411E-05	0.007378735
313.479997	657.739626	1.48443E-05	0.004653381
313.519997	587.731721	1.32643E-05	0.004158619
313.559997	613.379998	1.38431E-05	0.004340652
313.599997	623.656249	1.40751E-05	0.004413937
313.639997	595.498896	1.34396E-05	0.00421519
313.679997	514.072167	1.16019E-05	0.003639282
313.719997	385.740869	8.70563E-06	0.002731132
313.759997	302.133513	6.81873E-06	0.002139446

313.799997	289.381461	6.53094E-06	0.002049408
313.839997	354.50566	8.0007E-06	0.002510939
313.879997	491.072032	1.10828E-05	0.003478673
313.919997	792.809652	1.78926E-05	0.005616848
313.959997	1516.287723	3.42205E-05	0.010743869
313.999997	2823.817697	6.37296E-05	0.020011104
314.039997	4672.424379	0.00010545	0.033115552
314.079997	6692.345793	0.000151037	0.047437676
314.119997	8121.219368	0.000183285	0.057573361
314.159997	8249.794435	0.000186186	0.058492309
314.199997	7228.701233	0.000163142	0.051259127
314.239997	5558.02082	0.000125437	0.039417256
314.279997	3748.566242	8.45999E-05	0.026588062
314.319997	2262.510668	5.10617E-05	0.016049718
314.359997	1264.179873	2.85308E-05	0.008968936
314.399997	736.966541	1.66323E-05	0.005229198
314.439997	508.03253	1.14656E-05	0.003605239
314.479997	446.442263	1.00756E-05	0.003168568
314.519997	500.787255	1.13021E-05	0.003554727
314.559997	619.382013	1.39786E-05	0.004397105
314.599997	668.118068	1.50785E-05	0.004743694
314.639997	620.000124	1.39925E-05	0.004402612
314.679997	534.970715	1.20735E-05	0.003799303
314.719997	423.505849	9.55794E-06	0.003008074
314.759997	322.92698	7.28801E-06	0.002293975
314.799997	313.290058	7.07052E-06	0.0022258
314.839997	426.187462	9.61846E-06	0.003028275
314.879997	727.061616	1.64088E-05	0.005166793
314.919997	1398.442751	3.15609E-05	0.00993916
314.959997	2732.346491	6.16653E-05	0.019422088
314.999997	5046.513288	0.000113893	0.035876224
315.039997	8193.388483	0.000184913	0.058255106
315.079997	11338.31933	0.00025589	0.080625844
315.119997	13333.16427	0.000300911	0.094823056
315.159997	13495.17169	0.000304567	0.095987407
315.199997	12028.33378	0.000271463	0.085565055
315.239997	9426.843834	0.000212751	0.067067541
315.279997	6415.190373	0.000144782	0.04564684
315.319997	3803.994913	8.58509E-05	0.027070495
315.359997	2012.195734	4.54125E-05	0.014321272
315.399997	999.12575	2.25489E-05	0.007111916
315.439997	540.792405	1.22049E-05	0.003849923
315.479997	406.458427	9.1732E-06	0.002893961
315.519997	421.711714	9.51745E-06	0.003002945
315.559997	499.211019	1.12665E-05	0.003555256
315.599997	565.570074	1.27641E-05	0.004028359

315.639997	570.474473	1.28748E-05	0.004063806
315.679997	516.702563	1.16613E-05	0.003681226
315.719997	403.700287	9.11095E-06	0.00287651
315.759997	306.456329	6.91629E-06	0.002183888
315.799997	272.2693	6.14474E-06	0.001940509
315.839997	299.510663	6.75954E-06	0.002134933
315.879997	392.504451	8.85828E-06	0.002798153
315.919997	672.614218	1.518E-05	0.004795655
315.959997	1367.682612	3.08667E-05	0.00975264
315.999997	2601.188451	5.87052E-05	0.018550843
316.039997	4239.127457	9.56712E-05	0.030235926
316.079997	5821.644182	0.000131386	0.041528613
316.119997	6941.269594	0.000156655	0.049521711
316.159997	7242.989809	0.000163464	0.051680838
316.199997	6416.454648	0.00014481	0.045789062
316.239997	4856.600267	0.000109607	0.034662024
316.279997	3228.139756	7.28546E-05	0.023042458
316.319997	1908.866057	4.30804E-05	0.013627207
316.359997	1033.339909	2.3321E-05	0.007377845
316.399997	590.590293	1.33288E-05	0.004217232
316.439997	418.597952	9.44717E-06	0.002989463
316.479997	403.234621	9.10044E-06	0.002880108
316.519997	485.512127	1.09573E-05	0.003468215
316.559997	602.565352	1.35991E-05	0.004304919
316.599997	682.75357	1.54088E-05	0.004878425
316.639997	652.516639	1.47264E-05	0.004662965
316.679997	518.935991	1.17117E-05	0.003708849
316.719997	398.780594	8.99992E-06	0.002850455
316.759997	316.081273	7.13351E-06	0.002259612
316.799997	310.179432	7.00032E-06	0.002217701
316.839997	435.250787	9.823E-06	0.003112321
316.879997	865.267317	1.95279E-05	0.006187994
316.919997	1899.296406	4.28645E-05	0.013584609
316.959997	4127.344697	9.31484E-05	0.029524323
316.999997	7810.851473	0.00017628	0.055880772
317.039997	12317.46085	0.000277988	0.088133294
317.079997	16390.67539	0.000369915	0.117292556
317.119997	18748.7569	0.000423133	0.13418404
317.159997	18919.52108	0.000426987	0.135423271
317.199997	16958.23318	0.000382724	0.121399957
317.239997	13423.30307	0.000302945	0.096106351
317.279997	9282.525019	0.000209494	0.066468148
317.319997	5541.475061	0.000125063	0.039685109
317.359997	2837.295308	6.40338E-05	0.020321768
317.399997	1293.282459	2.91876E-05	0.009264139
317.439997	634.867007	1.43281E-05	0.004548301

317.479997	487.865915	1.10105E-05	0.003495599
317.519997	567.667665	1.28115E-05	0.004067897
317.559997	701.721337	1.58369E-05	0.005029157
317.599997	767.226488	1.73152E-05	0.005499318
317.639997	731.147419	1.6501E-05	0.005241371
317.679997	652.074476	1.47164E-05	0.00467511
317.719997	523.248843	1.1809E-05	0.003751955
317.759997	429.8223	9.70049E-06	0.003082428
317.799997	420.709105	9.49482E-06	0.003017453
317.839997	536.153323	1.21002E-05	0.003845938
317.879997	816.351407	1.84239E-05	0.005856594
317.919997	1353.932854	3.05564E-05	0.009714484
317.959997	2259.091546	5.09845E-05	0.016211047
317.999997	3686.159621	8.31915E-05	0.026454893
318.039997	5507.872354	0.000124305	0.039533964
318.079997	7407.214465	0.000167171	0.053173596
318.119997	8668.725557	0.000195641	0.062237334
318.159997	8734.473402	0.000197125	0.062717257
318.199997	7580.559059	0.000171083	0.054438502
318.239997	5748.508142	0.000129736	0.041287128
318.279997	3824.329839	8.63098E-05	0.027470682
318.319997	2242.218006	5.06037E-05	0.016108181
318.359997	1223.562248	2.76141E-05	0.008791223
318.399997	696.272351	1.57139E-05	0.005003305
318.439997	465.640161	1.05088E-05	0.003346438
318.479997	437.73466	9.87906E-06	0.003146283
318.519997	529.911572	1.19594E-05	0.003809297
318.559997	665.815582	1.50265E-05	0.004786851
318.599997	747.239117	1.68641E-05	0.005372917
318.639997	721.834754	1.62908E-05	0.005190902
318.679997	587.30114	1.32546E-05	0.004223966
318.719997	463.251703	1.04549E-05	0.0033322
318.759997	402.42868	9.08225E-06	0.00289506
318.799997	438.361449	9.89321E-06	0.003153954
318.839997	593.458539	1.33935E-05	0.004270394
318.879997	958.253925	2.16265E-05	0.006896244
318.919997	1772.206461	3.99962E-05	0.012755598
318.959997	3400.446211	7.67433E-05	0.024478054
318.999997	6003.21054	0.000135484	0.043219428
319.039997	9240.566151	0.000208547	0.066534741
319.079997	12020.01693	0.000271275	0.086558439
319.119997	13242.75524	0.000298871	0.095375566
319.159997	12618.48902	0.000284782	0.090890933
319.199997	10586.68165	0.000238927	0.076265389
319.239997	7897.052423	0.000178225	0.0568967
319.279997	5249.699909	0.000118478	0.037827788

319.319997	3112.376014	7.0242E-05	0.022429673
319.359997	1643.129728	3.70831E-05	0.011842874
319.399997	850.737686	1.92E-05	0.006132469
319.439997	513.531864	1.15897E-05	0.003702213
319.479997	449.057991	1.01346E-05	0.003237806
319.519997	535.239328	1.20796E-05	0.003859676
319.559997	624.379425	1.40914E-05	0.004503039
319.599997	636.543205	1.43659E-05	0.00459134
319.639997	601.770204	1.35811E-05	0.004341068
319.679997	497.275851	1.12228E-05	0.003587712
319.719997	376.585129	8.499E-06	0.002717301
319.759997	280.707126	6.33517E-06	0.002025734
319.799997	274.302345	6.19062E-06	0.001979761
319.839997	347.854538	7.85059E-06	0.002510933
319.879997	483.839198	1.09196E-05	0.003492954
319.919997	788.8447	1.78031E-05	0.005695576
319.959997	1491.982227	3.3672E-05	0.01077368
319.999997	2758.488223	6.22552E-05	0.019921676
320.039997	4541.273302	0.00010249	0.032800967
320.079997	6300.157801	0.000142186	0.045510826
320.119997	7335.41463	0.00016555	0.052995895
320.159997	7155.907556	0.000161499	0.051705477
320.199997	5949.745478	0.000134277	0.042995645
320.239997	4306.784503	9.71981E-05	0.031126728
320.279997	2723.006725	6.14545E-05	0.019682637
320.319997	1554.52326	3.50834E-05	0.011237922
320.359997	837.048959	1.8891E-05	0.00605193
320.399997	483.493441	1.09118E-05	0.003496132
320.439997	369.33505	8.33538E-06	0.002670988
320.479997	372.680234	8.41087E-06	0.002695517
320.519997	429.95579	9.7035E-06	0.003110167
320.559997	530.202512	1.19659E-05	0.003835799
320.599997	613.708713	1.38506E-05	0.004440487
320.639997	631.665175	1.42558E-05	0.004570981
320.679997	539.753709	1.21815E-05	0.00390636
320.719997	399.621961	9.01891E-06	0.002892545
320.759997	348.958899	7.87552E-06	0.002526151
320.799997	435.069537	9.81891E-06	0.003149907
320.839997	764.015559	1.72428E-05	0.005532169
320.879997	1873.508299	4.22825E-05	0.0135676
320.919997	4646.620528	0.000104868	0.033654159
320.959997	9870.32482	0.000222759	0.071496883
320.999997	17312.27202	0.000390714	0.12541915
321.039997	24705.34232	0.000557565	0.179000719
321.079997	28782.59704	0.000649583	0.208568148
321.119997	27831.64978	0.000628122	0.201702396

321.159997	22832.00537	0.000515287	0.16548943
321.199997	16352.38267	0.00036905	0.118539019
321.239997	10372.76903	0.000234099	0.075201946
321.279997	5941.247654	0.000134086	0.043079048
321.319997	3095.469514	6.98604E-05	0.022447555
321.359997	1526.083896	3.44416E-05	0.011068149
321.399997	804.162973	1.81488E-05	0.005833037
321.439997	598.070029	1.34976E-05	0.004338671
321.479997	628.207212	1.41778E-05	0.004557867
321.519997	722.380944	1.63031E-05	0.005241783
321.559997	820.3866	1.8515E-05	0.005953678
321.599997	830.94148	1.87532E-05	0.006031026
321.639997	722.029048	1.62952E-05	0.005241185
321.679997	550.093039	1.24148E-05	0.003993604
321.719997	393.397479	8.87843E-06	0.002856369
321.759997	299.862168	6.76747E-06	0.002177502
321.799997	274.702111	6.19964E-06	0.001995045
321.839997	323.469824	7.30026E-06	0.002349517
321.879997	539.580579	1.21776E-05	0.00391972
321.919997	1100.400167	2.48345E-05	0.007994722
321.959997	2329.446979	5.25724E-05	0.016926201
321.999997	4471.280458	0.000100911	0.032493204
322.039997	7002.871674	0.000158045	0.05089683
322.079997	9024.504643	0.00020367	0.065598194
322.119997	9755.320272	0.000220164	0.070919224
322.159997	8902.305434	0.000200913	0.064726013
322.199997	7031.386297	0.000158689	0.051129465
322.239997	4866.62609	0.000109833	0.035392577
322.279997	3012.026272	6.79772E-05	0.021907704
322.319997	1708.374392	3.85556E-05	0.012427251
322.359997	921.406383	2.07949E-05	0.006703431
322.399997	549.439865	1.24001E-05	0.00399779
322.439997	407.448527	9.19555E-06	0.002965012
322.479997	399.177198	9.00887E-06	0.002905181
322.519997	477.922158	1.0786E-05	0.003478713
322.559997	580.708683	1.31058E-05	0.004227402
322.599997	654.426807	1.47695E-05	0.004764641
322.639997	654.748011	1.47768E-05	0.004767571
322.679997	563.066146	1.27076E-05	0.004100494
322.719997	412.960579	9.31995E-06	0.003007733
322.759997	312.827111	7.06007E-06	0.002278709
322.799997	333.59293	7.52873E-06	0.002430273
322.839997	499.258111	1.12676E-05	0.003637619
322.879997	869.461722	1.96225E-05	0.006335726
322.919997	1631.113653	3.6812E-05	0.011887319
322.959997	3111.247731	7.02165E-05	0.02267713

322.999997	5628.662806	0.000127031	0.041031038
323.039997	8872.346358	0.000200236	0.064684398
323.079997	11753.44536	0.000265259	0.085699842
323.119997	13381.53001	0.000302002	0.097583045
323.159997	13060.33087	0.000294753	0.095252532
323.199997	10906.1602	0.000246137	0.079551427
323.239997	7835.976534	0.000176847	0.057164048
323.279997	4869.262775	0.000109892	0.03552604
323.319997	2649.394213	5.97931E-05	0.019332317
323.359997	1294.744457	2.92206E-05	0.009448766
323.399997	651.875159	1.47119E-05	0.004757833
323.439997	423.506656	9.55796E-06	0.003091425
323.479997	401.460307	9.0604E-06	0.002930858
323.519997	500.396572	1.12933E-05	0.003653593
323.559997	618.339667	1.39551E-05	0.004515301
323.599997	704.431055	1.5898E-05	0.005144602
323.639997	700.543906	1.58103E-05	0.005116846
323.679997	585.976697	1.32247E-05	0.004280564
323.719997	433.528372	9.78413E-06	0.003167319
323.759997	310.146504	6.99957E-06	0.002266182
323.799997	262.404116	5.9221E-06	0.001917574
323.839997	319.955767	7.22096E-06	0.002338434
323.879997	487.244511	1.09964E-05	0.003561524
323.919997	816.155946	1.84195E-05	0.005966445
323.959997	1545.993357	3.48909E-05	0.011303261
323.999997	2951.09824	6.66022E-05	0.021579105
324.039997	4897.930942	0.000110539	0.035819212
324.079997	6887.791362	0.000155448	0.050377541
324.119997	8231.274592	0.000185768	0.060211254
324.159997	8359.882452	0.000188671	0.061159559
324.199997	7276.292517	0.000164216	0.053238758
324.239997	5545.345014	0.000125151	0.040578867
324.279997	3765.921292	8.49916E-05	0.027561075
324.319997	2235.411347	5.04501E-05	0.016361983
324.359997	1213.916258	2.73964E-05	0.008886296
324.399997	687.243503	1.55101E-05	0.005031486
324.439997	453.756555	1.02407E-05	0.003322478
324.479997	411.546794	9.28804E-06	0.003013783
324.519997	487.576721	1.10039E-05	0.003570995
324.559997	576.209564	1.30042E-05	0.004220658
324.599997	670.85324	1.51402E-05	0.004914516
324.639997	693.01811	1.56405E-05	0.005077517
324.679997	596.154759	1.34544E-05	0.004368369
324.719997	463.731517	1.04658E-05	0.003398446
324.759997	394.108217	8.89447E-06	0.002888569
324.799997	448.916941	1.01314E-05	0.003290688

324.839997	676.278443	1.52627E-05	0.004957923
324.879997	1249.394421	2.81971E-05	0.009160671
324.919997	2552.477979	5.76059E-05	0.0187173
324.959997	5447.549265	0.000122944	0.039951752
324.999997	10279.79644	0.000232001	0.075400223
325.039997	16207.6141	0.000365783	0.118894195
325.079997	21279.23794	0.000480243	0.156117315
325.119997	23485.69761	0.000530039	0.172326435
325.159997	22167.32908	0.000500286	0.16267291
325.199997	17992.88635	0.000406074	0.132055388
325.239997	12683.46088	0.000286248	0.093099313
325.279997	7815.938215	0.000176395	0.057377713
325.319997	4219.869979	9.52366E-05	0.030982366
325.359997	2017.977445	4.55429E-05	0.014817851
325.399997	966.880251	2.18211E-05	0.007100599
325.439997	568.72944	1.28354E-05	0.004177163
325.479997	502.649318	1.13441E-05	0.003692276
325.519997	602.269192	1.35924E-05	0.00442459
325.559997	737.277276	1.66393E-05	0.005417097
325.599997	772.76363	1.74402E-05	0.005678529
325.639997	736.080617	1.66123E-05	0.005409634
325.679997	609.710512	1.37603E-05	0.00448146
325.719997	464.451346	1.0482E-05	0.003414204
325.759997	353.131197	7.96968E-06	0.002596203
325.799997	339.173889	7.65468E-06	0.002493895
325.839997	424.241413	9.57454E-06	0.003119767
325.879997	568.003183	1.2819E-05	0.004177469
325.919997	930.665486	2.10038E-05	0.006845566
325.959997	1739.995136	3.92693E-05	0.01280021
325.999997	3223.424457	7.27482E-05	0.023715913
326.039997	5350.931856	0.000120763	0.039373594
326.079997	7542.662138	0.000170227	0.055507743
326.119997	8795.024308	0.000198491	0.06473203
326.159997	8672.427312	0.000195725	0.063837536
326.199997	7386.423033	0.000166701	0.054377957
326.239997	5567.899035	0.00012566	0.040995229
326.279997	3783.023658	8.53776E-05	0.027856995
326.319997	2331.478231	5.26182E-05	0.017170376
326.359997	1356.447192	3.06131E-05	0.009990899
326.399997	764.024833	1.7243E-05	0.005628107
326.439997	488.185045	1.10177E-05	0.003596604
326.479997	426.843593	9.63327E-06	0.003145068
326.519997	511.506771	1.1544E-05	0.003769345
326.559997	605.328885	1.36614E-05	0.004461276
326.599997	586.260009	1.32311E-05	0.004321268
326.639997	503.130004	1.13549E-05	0.003708979

326.679997	432.203952	9.75424E-06	0.003186515
326.719997	350.804466	7.91717E-06	0.002586697
326.759997	282.178158	6.36837E-06	0.002080928
326.799997	286.037026	6.45546E-06	0.002109643
326.839997	364.257782	8.22079E-06	0.002686883
326.879997	583.807918	1.31757E-05	0.004306883
326.919997	1192.750471	2.69187E-05	0.008800267
326.959997	2592.706018	5.85138E-05	0.01913166
326.999997	4998.622256	0.000112812	0.036889505
327.039997	8117.485841	0.0001832	0.059913841
327.079997	11252.36386	0.00025395	0.083062024
327.119997	13482.4527	0.00030428	0.099536131
327.159997	13984.98018	0.000315622	0.103258737
327.199997	12508.0707	0.00028229	0.0923652
327.239997	9671.125176	0.000218264	0.071424653
327.279997	6415.320578	0.000144785	0.047385185
327.319997	3636.688081	8.2075E-05	0.026864784
327.359997	1827.67261	4.1248E-05	0.013502953
327.399997	886.721504	2.00121E-05	0.006551951
327.439997	522.291452	1.17874E-05	0.003859663
327.479997	455.096495	1.02709E-05	0.003363512
327.519997	493.145078	1.11296E-05	0.003645166
327.559997	533.963287	1.20508E-05	0.003947363
327.599997	537.88528	1.21393E-05	0.003976842
327.639997	520.762909	1.17529E-05	0.003850718
327.679997	430.338989	9.71215E-06	0.003182478
327.719997	323.359058	7.29776E-06	0.002391623
327.759997	270.03645	6.09435E-06	0.001997483
327.799997	279.688235	6.31217E-06	0.002069131
327.839997	352.091905	7.94622E-06	0.00260509
327.879997	490.717015	1.10748E-05	0.003631205
327.919997	730.715865	1.64912E-05	0.005407807
327.959997	1287.71993	2.9062E-05	0.009531188
327.999997	2490.337063	5.62034E-05	0.018434728
328.039997	4228.610428	9.54338E-05	0.031306119
328.079997	6083.135437	0.000137288	0.045041412
328.119997	7339.296451	0.000165638	0.054349041
328.159997	7583.055671	0.000171139	0.056160975
328.199997	6827.735965	0.000154092	0.050573155
328.239997	5438.158437	0.000122732	0.04028544
328.279997	3818.866001	8.61865E-05	0.028293299
328.319997	2365.67613	5.339E-05	0.01752901
328.359997	1315.762857	2.96949E-05	0.009750629
328.399997	726.090316	1.63868E-05	0.005381441
328.439997	462.117746	1.04294E-05	0.003425417
328.479997	393.6146	8.88333E-06	0.002917997

328.519997	436.917313	9.86062E-06	0.003239409
328.559997	515.469791	1.16334E-05	0.003822281
328.599997	548.672151	1.23828E-05	0.004068977
328.639997	516.729887	1.16619E-05	0.003832558
328.679997	432.620532	9.76364E-06	0.003209114
328.719997	340.389402	7.68211E-06	0.002525265
328.759997	278.720461	6.29033E-06	0.00206801
328.799997	281.823797	6.36037E-06	0.00209129
328.839997	374.798654	8.45868E-06	0.002781553
328.879997	616.809374	1.39205E-05	0.004578183
328.919997	1309.404299	2.95514E-05	0.009720057
328.959997	2849.924945	6.43188E-05	0.021158324
328.999997	5551.308091	0.000125285	0.04121886
329.039997	9282.384254	0.00020949	0.068930748
329.079997	12965.83023	0.000292621	0.096295628
329.119997	15254.05407	0.000344263	0.113303756
329.159997	15308.65327	0.000345495	0.113723127
329.199997	13274.30011	0.000299582	0.098622546
329.239997	10099.79574	0.000227938	0.075046412
329.279997	6704.12888	0.000151303	0.049821002
329.319997	3866.123284	8.7253E-05	0.028734163
329.359997	1991.986319	4.49564E-05	0.014806825
329.399997	975.417749	2.20138E-05	0.007251352
329.439997	537.742774	1.21361E-05	0.003998119
329.479997	435.011883	9.81761E-06	0.003234707
329.519997	486.429202	1.0978E-05	0.00361748
329.559997	577.021627	1.30226E-05	0.00429172
329.599997	645.478965	1.45676E-05	0.004801468
329.639997	605.38268	1.36626E-05	0.004503754
329.679997	473.864458	1.06945E-05	0.00352575
329.719997	331.681619	7.48559E-06	0.002468149
329.759997	251.662538	5.67967E-06	0.001872929
329.799997	238.190628	5.37563E-06	0.001772883
329.839997	294.200679	6.6397E-06	0.002190038
329.879997	444.053297	1.00217E-05	0.003305947
329.919997	792.729216	1.78908E-05	0.005902531
329.959997	1523.139043	3.43751E-05	0.011342417
329.999997	2831.785405	6.39095E-05	0.021090119
330.039997	4719.393032	0.00010651	0.035152602
330.079997	6715.470548	0.000151559	0.050026534
330.119997	8035.365187	0.000181347	0.059866271
330.159997	8146.320867	0.000183851	0.060700284
330.199997	7142.870915	0.000161205	0.053229774
330.239997	5526.728702	0.000124731	0.041191023
330.279997	3797.354259	8.5701E-05	0.028305325
330.319997	2288.491125	5.16481E-05	0.017060386

330.359997	1242.160422	2.80338E-05	0.009261256
330.399997	677.030012	1.52796E-05	0.005048388
330.439997	462.257548	1.04325E-05	0.003447318
330.479997	449.290487	1.01399E-05	0.003351021
330.519997	532.156972	1.201E-05	0.003969559
330.559997	629.77954	1.42132E-05	0.004698331
330.599997	644.957152	1.45558E-05	0.004812142
330.639997	592.727797	1.3377E-05	0.004422985
330.679997	498.018935	1.12396E-05	0.003716709
330.719997	363.818084	8.21087E-06	0.002715498
330.759997	274.320247	6.19103E-06	0.002047744
330.799997	252.29056	5.69385E-06	0.001883524
330.839997	323.349445	7.29755E-06	0.00241432
330.879997	590.289626	1.3322E-05	0.004407988
330.919997	1262.639798	2.8496E-05	0.009429903
330.959997	2820.983966	6.36657E-05	0.021070793
330.999997	5662.562278	0.000127796	0.042300523
331.039997	9689.139565	0.00021867	0.072388643
331.079997	14028.5957	0.000316606	0.104821869
331.119997	17533.39196	0.000395704	0.131025585
331.159997	19039.94906	0.000429705	0.14230115
331.199997	17851.33659	0.00040288	0.133433789
331.239997	14411.76002	0.000325253	0.107736919
331.279997	10011.62645	0.000225948	0.074852206
331.319997	6013.553304	0.000135718	0.044965929
331.359997	3103.234183	7.00357E-05	0.02320702
331.399997	1417.068354	3.19813E-05	0.010598589
331.439997	697.286147	1.57368E-05	0.005215797
331.479997	489.531308	1.1048E-05	0.003662204
331.519997	462.522382	1.04385E-05	0.003460567
331.559997	537.640932	1.21338E-05	0.004023085
331.599997	625.07957	1.41072E-05	0.004677939
331.639997	617.457126	1.39351E-05	0.004621452
331.679997	509.524556	1.14993E-05	0.003814074
331.719997	362.383786	8.1785E-06	0.002712971
331.759997	255.643185	5.76951E-06	0.001914093
331.799997	222.997441	5.03274E-06	0.001669864
331.839997	278.154315	6.27756E-06	0.002083144
331.879997	402.094072	9.0747E-06	0.003011712
331.919997	691.940982	1.56161E-05	0.005183311
331.959997	1402.635882	3.16555E-05	0.010508372
331.999997	2808.464307	6.33831E-05	0.021043198
332.039997	4813.576621	0.000108636	0.036071404
332.079997	6893.457125	0.000155576	0.051663586
332.119997	8358.726036	0.000188645	0.062652712
332.159997	8778.768538	0.000198125	0.065809059

332.199997	7944.957904	0.000179307	0.059565663
332.239997	6187.760285	0.000139649	0.046397027
332.279997	4148.481618	9.36255E-05	0.031109865
332.319997	2431.220249	5.48693E-05	0.018234151
332.359997	1285.404524	2.90098E-05	0.009641694
332.399997	695.222951	1.56902E-05	0.005215427
332.439997	471.022619	1.06303E-05	0.003533945
332.479997	441.741571	9.96949E-06	0.003314657
332.519997	561.812844	1.26793E-05	0.004216132
332.559997	746.084966	1.68381E-05	0.005599678
332.599997	835.349577	1.88527E-05	0.0062704
332.639997	761.538455	1.71869E-05	0.005717038
332.679997	611.288856	1.37959E-05	0.004589633
332.719997	429.908417	9.70243E-06	0.003228194
332.759997	319.648339	7.21402E-06	0.002400537
332.799997	300.953389	6.7921E-06	0.00226041
332.839997	378.588358	8.54421E-06	0.002843855
332.879997	586.34905	1.32331E-05	0.004405028
332.919997	1013.01626	2.28624E-05	0.007611339
332.959997	1932.950254	4.3624E-05	0.014525045
332.999997	3731.811799	8.42218E-05	0.028045857
333.039997	6325.667214	0.000142761	0.047545289
333.079997	9190.446242	0.000207416	0.069085978
333.119997	11503.41922	0.000259616	0.086483331
333.159997	12511.4324	0.000282366	0.094072923
333.199997	11888.19118	0.0002683	0.089397531
333.239997	9739.586055	0.000219809	0.073249114
333.279997	6802.323953	0.000153519	0.051164804
333.319997	4083.188014	9.21519E-05	0.03071606
333.359997	2092.471156	4.72242E-05	0.015742646
333.399997	980.9009	2.21376E-05	0.007380665
333.439997	513.562536	1.15904E-05	0.0038647
333.479997	402.698506	9.08834E-06	0.003030781
333.519997	470.476455	1.0618E-05	0.003541315
333.559997	638.327613	1.44062E-05	0.00480532
333.599997	714.867658	1.61336E-05	0.005382158
333.639997	670.078593	1.51227E-05	0.005045551
333.679997	540.243533	1.21925E-05	0.004068408
333.719997	406.870427	9.1825E-06	0.003064383
333.759997	313.501733	7.0753E-06	0.002361451
333.799997	263.448973	5.94568E-06	0.001984667
333.839997	278.058443	6.27539E-06	0.002094977
333.879997	384.473253	8.67703E-06	0.002897085
333.919997	604.34752	1.36393E-05	0.004554429
333.959997	1043.429129	2.35487E-05	0.007864338
333.999997	1897.821631	4.28312E-05	0.014305618

334.039997	3255.887153	7.34808E-05	0.024545539
334.079997	4835.772345	0.000109137	0.03646037
334.119997	6122.561268	0.000138178	0.046167926
334.159997	6585.419448	0.000148624	0.04966411
334.199997	6126.386459	0.000138264	0.046207831
334.239997	4998.101286	0.0001128	0.037702333
334.279997	3510.999156	7.92384E-05	0.026487799
334.319997	2084.929684	4.7054E-05	0.015731079
334.359997	1098.652434	2.47951E-05	0.008290475
334.399997	589.956724	1.33145E-05	0.004452369
334.439997	387.165093	8.73778E-06	0.002922262
334.479997	359.639191	8.11656E-06	0.002714825
334.519997	445.839001	1.0062E-05	0.003365929
334.559997	541.922329	1.22304E-05	0.004091813
334.599997	568.799685	1.2837E-05	0.004295266
334.639997	543.750076	1.22717E-05	0.004106596
334.679997	467.101712	1.05418E-05	0.003528141
334.719997	356.546308	8.04675E-06	0.002693409
334.759997	266.970049	6.02514E-06	0.002016977
334.799997	251.573377	5.67766E-06	0.001900881
334.839997	310.522692	7.00806E-06	0.00234658
334.879997	467.45924	1.05499E-05	0.003532952
334.919997	842.902159	1.90231E-05	0.006371226
334.959997	1651.884359	3.72807E-05	0.012487553
334.999997	3218.543058	7.2638E-05	0.024333741
335.039997	5561.102525	0.000125506	0.042049643
335.079997	8167.806369	0.000184336	0.061767311
335.119997	10454.09892	0.000235934	0.079066353
335.159997	11862.09527	0.000267711	0.089726007
335.199997	11896.48052	0.000268487	0.08999684
335.239997	10467.49038	0.000236237	0.079195983
335.279997	7943.734821	0.000179279	0.060108673
335.319997	5206.994455	0.000117515	0.039404999
335.359997	2960.70438	6.6819E-05	0.022408411
335.399997	1497.993176	3.38076E-05	0.011339075
335.439997	745.974517	1.68356E-05	0.005647336
335.479997	471.586129	1.0643E-05	0.003570528
335.519997	446.747215	1.00825E-05	0.003382868
335.559997	523.380491	1.1812E-05	0.003963624
335.599997	564.125941	1.27315E-05	0.004272704
335.639997	570.614286	1.2878E-05	0.004322362
335.679997	510.733377	1.15265E-05	0.003869229
335.719997	395.986495	8.93686E-06	0.003000284
335.759997	299.050262	6.74915E-06	0.002266094
335.799997	274.598316	6.1973E-06	0.002081054
335.839997	317.354778	7.16226E-06	0.002405372

335.879997	465.24888	1.05E-05	0.003526746
335.919997	794.773006	1.79369E-05	0.00602537
335.959997	1482.875059	3.34664E-05	0.01124338
335.999997	2858.818836	6.45196E-05	0.021678572
336.039997	5181.951347	0.000116949	0.039299684
336.079997	7962.671352	0.000179706	0.060395727
336.119997	10130.2363	0.000228625	0.076845543
336.159997	10777.81709	0.00024324	0.081767665
336.199997	9715.181088	0.000219258	0.073714574
336.239997	7587.704802	0.000171244	0.057579058
336.279997	5113.276566	0.0001154	0.038806554
336.319997	3001.752918	6.77454E-05	0.022784127
336.359997	1585.503184	3.57826E-05	0.012035835
336.399997	804.705356	1.81611E-05	0.006109387
336.439997	498.62787	1.12533E-05	0.003786072
336.479997	415.480229	9.37681E-06	0.003155109
336.519997	466.375249	1.05254E-05	0.003542021
336.559997	546.5499	1.23349E-05	0.004151424
336.599997	582.324691	1.31423E-05	0.004423684
336.639997	572.565989	1.2922E-05	0.004350068
336.679997	460.135692	1.03846E-05	0.003496294
336.719997	323.4022	7.29874E-06	0.002457631
336.759997	246.088627	5.55388E-06	0.001870324
336.799997	241.542686	5.45128E-06	0.001835992
336.839997	317.898449	7.17453E-06	0.002416667
336.879997	556.259937	1.2554E-05	0.004229196
336.919997	1108.61408	2.50199E-05	0.008429696
336.959997	2271.053621	5.12545E-05	0.017270722
336.999997	4344.183437	9.80422E-05	0.033040211
337.039997	7054.250347	0.000159205	0.053658319
337.079997	9653.558595	0.000217867	0.073438733
337.119997	11252.70626	0.000253958	0.085614289
337.159997	11348.76091	0.000256126	0.086355349
337.199997	9997.544504	0.000225631	0.076082656
337.239997	7839.902479	0.000176936	0.059669788
337.279997	5456.113774	0.000123137	0.041531609
337.319997	3362.835981	7.58945E-05	0.025600741
337.359997	1847.383789	4.16929E-05	0.01406551
337.399997	962.997039	2.17335E-05	0.007332883
337.439997	559.071012	1.26175E-05	0.004257634
337.479997	458.065931	1.03379E-05	0.003488838
337.519997	532.335696	1.20141E-05	0.004054991
337.559997	619.885031	1.39899E-05	0.004722445
337.599997	643.376638	1.45201E-05	0.004901991
337.639997	626.630143	1.41422E-05	0.004774962
337.679997	573.398462	1.29408E-05	0.004369851

337.719997	518.105075	1.16929E-05	0.003948929
337.759997	463.934641	1.04704E-05	0.003536468
337.799997	422.771766	9.54137E-06	0.003223075
337.839997	442.358661	9.98342E-06	0.003372798
337.879997	565.381647	1.27599E-05	0.004311307
337.919997	832.373323	1.87855E-05	0.006347998
337.959997	1379.541685	3.11343E-05	0.01052216
337.999997	2410.096913	5.43925E-05	0.018384675
338.039997	3901.366026	8.80484E-05	0.02976388
338.079997	5598.782323	0.000126357	0.042718679
338.119997	6910.959035	0.000155971	0.052736819
338.159997	7204.112046	0.000162587	0.054980343
338.199997	6408.481928	0.000144631	0.048914038
338.239997	5004.241631	0.000112939	0.038200407
338.279997	3496.124374	7.89027E-05	0.026691191
338.319997	2273.238547	5.13038E-05	0.017357111
338.359997	1443.017633	3.25669E-05	0.011019336
338.399997	950.001153	2.14402E-05	0.007255365
338.439997	664.50924	1.4997E-05	0.005075601
338.479997	520.887131	1.17557E-05	0.003979068
338.519997	491.898492	1.11015E-05	0.003758067
338.559997	582.952259	1.31564E-05	0.004454238
338.599997	688.602926	1.55408E-05	0.005262118
338.639997	657.519852	1.48393E-05	0.005025183
338.679997	530.934747	1.19825E-05	0.004058219
338.719997	401.767676	9.06734E-06	0.003071288
338.759997	319.173257	7.2033E-06	0.002440188
338.799997	305.588734	6.89671E-06	0.002336606
338.839997	400.005409	9.02756E-06	0.0030589
338.879997	710.165645	1.60274E-05	0.005431382
338.919997	1446.767891	3.26515E-05	0.011066259
338.959997	2980.034507	6.72552E-05	0.022796832
338.999997	5607.657734	0.000126557	0.042902831
339.039997	8962.669648	0.000202275	0.068579305
339.079997	12189.74743	0.000275106	0.093282813
339.119997	14037.20958	0.0003168	0.107433306
339.159997	13740.71146	0.000310109	0.105176472
339.199997	11618.49034	0.000262213	0.088942698
339.239997	8578.094291	0.000193596	0.065675384
339.279997	5658.189462	0.000127697	0.043325192
339.319997	3412.333647	7.70116E-05	0.026131582
339.359997	1950.803239	4.40269E-05	0.014940973
339.399997	1103.950526	2.49146E-05	0.008456024
339.439997	679.35495	1.53321E-05	0.005204326
339.479997	533.255442	1.20348E-05	0.004085585
339.519997	559.056685	1.26171E-05	0.004283768

339.559997	645.931094	1.45778E-05	0.004950026
339.599997	701.558712	1.58332E-05	0.005376956
339.639997	647.517488	1.46136E-05	0.004963352
339.679997	496.700137	1.12098E-05	0.003807755
339.719997	364.084429	8.21688E-06	0.002791438
339.759997	298.854439	6.74473E-06	0.002291589
339.799997	259.707516	5.86124E-06	0.001991648
339.839997	292.860837	6.60946E-06	0.002246159
339.879997	420.992965	9.50122E-06	0.003229276
339.919997	654.820954	1.47784E-05	0.005023473
339.959997	1188.920096	2.68323E-05	0.009121899
339.999997	2282.293268	5.15082E-05	0.017512781
340.039997	3875.07965	8.74552E-05	0.029738249
340.079997	5538.141444	0.000124988	0.042505966
340.119997	6734.058879	0.000151978	0.051690869
340.159997	6929.869138	0.000156397	0.053200171
340.199997	6125.146806	0.000138236	0.047027898
340.239997	4755.343085	0.000107321	0.036515057
340.279997	3258.944243	7.35498E-05	0.025027537
340.319997	1994.354479	4.50098E-05	0.015317736
340.359997	1125.800784	2.54078E-05	0.008647784
340.399997	641.596045	1.44799E-05	0.004928968
340.439997	429.411933	9.69123E-06	0.003299282
340.479997	361.694962	8.16295E-06	0.002779322
340.519997	413.807817	9.33907E-06	0.003180139
340.559997	482.178241	1.08821E-05	0.003706005
340.599997	541.580249	1.22227E-05	0.004163056
340.639997	555.418023	1.2535E-05	0.004269926
340.679997	481.824377	1.08741E-05	0.00370459
340.719997	373.578818	8.43115E-06	0.002872663
340.759997	290.250883	6.55056E-06	0.002232168
340.799997	282.956716	6.38594E-06	0.002176328
340.839997	353.030244	7.9674E-06	0.002715609
340.879997	554.893551	1.25232E-05	0.0042689
340.919997	1063.089657	2.39925E-05	0.008179507
340.959997	2218.664455	5.00722E-05	0.017072606
340.999997	4193.44741	9.46403E-05	0.032272331
341.039997	6699.717121	0.000151203	0.051566367
341.079997	9200.632465	0.000207645	0.070823713
341.119997	10758.88652	0.000242813	0.082828398
341.159997	10802.84556	0.000243805	0.083176573
341.199997	9402.886094	0.00021221	0.072406066
341.239997	7054.624233	0.000159213	0.054329858
341.279997	4620.631355	0.000104281	0.035589092
341.319997	2667.095185	6.01926E-05	0.020544946
341.359997	1417.564843	3.19925E-05	0.010920947

341.399997	739.253638	1.66839E-05	0.005695892
341.439997	421.509168	9.51287E-06	0.003248076
341.479997	346.831227	7.8275E-06	0.002672934
341.519997	406.311367	9.16988E-06	0.003131698
341.559997	499.380547	1.12703E-05	0.003849492
341.599997	555.66193	1.25405E-05	0.00428384
341.639997	543.355299	1.22628E-05	0.004189453
341.679997	474.405973	1.07067E-05	0.003658259
341.719997	366.751138	8.27706E-06	0.002828438
341.759997	289.875742	6.54209E-06	0.002235825
341.799997	274.704717	6.1997E-06	0.002119058
341.839997	314.02229	7.08705E-06	0.002422636
341.879997	414.245368	9.34894E-06	0.003196216
341.919997	660.356712	1.49033E-05	0.005095747
341.959997	1287.833057	2.90646E-05	0.00993893
341.999997	2495.137912	5.63118E-05	0.019258631
342.039997	4190.757135	9.45796E-05	0.032349989
342.079997	6037.272301	0.000136253	0.046609367
342.119997	7300.493797	0.000164762	0.056368367
342.159997	7546.392994	0.000170312	0.058273809
342.199997	6729.897446	0.000151884	0.051974845
342.239997	5146.003404	0.000116138	0.039747113
342.279997	3424.649772	7.72896E-05	0.026454676
342.319997	2035.297928	4.59338E-05	0.015724072
342.359997	1115.161619	2.51676E-05	0.008616395
342.399997	629.496105	1.42069E-05	0.004864425
342.439997	428.520199	9.6711E-06	0.003311773
342.479997	397.775128	8.97723E-06	0.003074522
342.519997	463.36341	1.04575E-05	0.003581891
342.559997	561.147744	1.26643E-05	0.00433829
342.599997	586.203561	1.32298E-05	0.004532529
342.639997	565.77186	1.27687E-05	0.004375061
342.679997	515.103259	1.16252E-05	0.003983711
342.719997	412.120605	9.30099E-06	0.003187635
342.759997	326.335558	7.36494E-06	0.002524406
342.799997	301.64015	6.8076E-06	0.002333645
342.839997	351.892919	7.94173E-06	0.002722744
342.879997	568.274344	1.28252E-05	0.004397491
342.919997	1127.146382	2.54381E-05	0.008723241
342.959997	2335.046029	5.26987E-05	0.018073558
342.999997	4483.128048	0.000101178	0.034704038
343.039997	7550.257435	0.000170399	0.058453602
343.079997	10695.2274	0.000241376	0.082811409
343.119997	12668.67936	0.000285914	0.098102964
343.159997	12819.77113	0.000289324	0.099284553
343.199997	11133.79094	0.000251274	0.086237289

343.239997	8327.084489	0.000187931	0.064505332
343.279997	5407.023261	0.000122029	0.041890109
343.319997	3013.210649	6.8004E-05	0.023347122
343.359997	1482.164008	3.34504E-05	0.011485521
343.399997	714.794522	1.61319E-05	0.0055397
343.439997	426.136041	9.6173E-06	0.003302964
343.479997	368.587227	8.3185E-06	0.002857238
343.519997	445.605904	1.00567E-05	0.003454679
343.559997	526.469796	1.18817E-05	0.004082073
343.599997	573.608169	1.29455E-05	0.004448087
343.639997	568.053182	1.28202E-05	0.004405523
343.679997	474.109388	1.07E-05	0.003677372
343.719997	366.403145	8.26921E-06	0.002842292
343.759997	286.181636	6.45872E-06	0.00222025
343.799997	243.576496	5.49718E-06	0.001889931
343.839997	278.158019	6.27764E-06	0.002158503
343.879997	397.156493	8.96327E-06	0.003082289
343.919997	659.734193	1.48893E-05	0.005120722
343.959997	1273.31911	2.8737E-05	0.009884392
343.999997	2348.775573	5.30086E-05	0.018234956
344.039997	3937.23986	8.8858E-05	0.030570713
344.079997	5790.509139	0.000130684	0.044965656
344.119997	7080.529985	0.000159798	0.054989584
344.159997	7260.462798	0.000163859	0.056393552
344.199997	6455.592047	0.000145694	0.050147778
344.239997	5026.129171	0.000113433	0.039048084
344.279997	3440.073173	7.76377E-05	0.026729093
344.319997	2095.93208	4.73023E-05	0.016287117
344.359997	1155.015867	2.60671E-05	0.008976466
344.399997	621.598309	1.40286E-05	0.004831453
344.439997	396.744883	8.95398E-06	0.003084109
344.479997	371.501581	8.38427E-06	0.002888214
344.519997	508.537303	1.1477E-05	0.003954049
344.559997	686.971642	1.5504E-05	0.005342056
344.599997	777.904891	1.75562E-05	0.006049877
344.639997	700.763579	1.58153E-05	0.00545057
344.679997	541.799369	1.22277E-05	0.004214629
344.719997	407.314785	9.19253E-06	0.003168848
344.759997	305.225403	6.88851E-06	0.002374883
344.799997	267.248347	6.03142E-06	0.002079635
344.839997	328.361872	7.41067E-06	0.002555495
344.879997	568.40659	1.28281E-05	0.004424171
344.919997	1296.204213	2.92535E-05	0.010090125
344.959997	3121.005465	7.04367E-05	0.02429786
344.999997	6459.915722	0.000145791	0.050297998
345.039997	11301.83178	0.000255067	0.088008179

345.079997	16768.01489	0.000378431	0.130588879
345.119997	21053.01774	0.000475137	0.163979379
345.159997	22529.71319	0.000508464	0.175501517
345.199997	20647.22299	0.000465979	0.160855971
345.239997	16083.03379	0.000362972	0.125312339
345.279997	10624.99925	0.000239791	0.082795186
345.319997	5974.291243	0.000134831	0.04655999
345.359997	2933.278094	6.62E-05	0.022862832
345.399997	1311.832906	2.96062E-05	0.010225996
345.439997	635.66106	1.4346E-05	0.004955677
345.479997	483.558328	1.09132E-05	0.003770305
345.519997	541.336032	1.22172E-05	0.004221287
345.559997	696.331726	1.57152E-05	0.005430557
345.599997	797.997107	1.80097E-05	0.006224146
345.639997	761.701834	1.71905E-05	0.005941741
345.679997	623.07489	1.40619E-05	0.004860929
345.719997	463.554912	1.04618E-05	0.00361685
345.759997	336.186476	7.58726E-06	0.002623371
345.799997	277.008597	6.2517E-06	0.002161837
345.839997	294.486602	6.64615E-06	0.002298505
345.879997	439.466977	9.91816E-06	0.003430492
345.919997	806.138219	1.81934E-05	0.006293467
345.959997	1546.775836	3.49086E-05	0.012076971
345.999997	2944.530876	6.6454E-05	0.02299307
346.039997	4951.200193	0.000111742	0.038667095
346.079997	7091.628513	0.000160048	0.055389473
346.119997	8751.065357	0.000197499	0.068358476
346.159997	9357.113975	0.000211177	0.07310104
346.199997	8603.158104	0.000194161	0.06721864
346.239997	6824.750725	0.000154025	0.053329656
346.279997	4804.979994	0.000108442	0.037551194
346.319997	2958.421461	6.67675E-05	0.023122904
346.359997	1592.941863	3.59505E-05	0.012451808
346.399997	799.569991	1.80452E-05	0.006250851
346.439997	446.544251	1.00779E-05	0.003491381
346.479997	361.006685	8.14742E-06	0.002822917
346.519997	423.050946	9.54767E-06	0.003308459
346.559997	555.419419	1.2535E-05	0.004344144
346.599997	641.805366	1.44847E-05	0.005020381
346.639997	606.929384	1.36976E-05	0.004748119
346.679997	487.620318	1.10049E-05	0.003815183
346.719997	360.947789	8.14609E-06	0.002824412
346.759997	288.750511	6.5167E-06	0.00225973
346.799997	311.668784	7.03393E-06	0.002439367
346.839997	458.777215	1.0354E-05	0.003591168
346.879997	746.850406	1.68554E-05	0.005846792

346.919997	1239.96357	2.79843E-05	0.009708296
346.959997	2307.060675	5.20671E-05	0.018065217
346.999997	4351.320831	9.82033E-05	0.034076528
347.039997	7403.778366	0.000167093	0.057987942
347.079997	10700.9449	0.000241505	0.083821701
347.119997	13332.34556	0.000300892	0.104445793
347.159997	14426.62885	0.000325589	0.113031448
347.199997	13340.34138	0.000301073	0.104532518
347.239997	10588.64158	0.000238971	0.082980252
347.279997	7167.14374	0.000161752	0.056173391
347.319997	4102.904601	9.25968E-05	0.032160735
347.359997	2026.434202	4.57338E-05	0.015886092
347.399997	919.982361	2.07627E-05	0.007212969
347.439997	480.776437	1.08505E-05	0.003769882
347.479997	370.26936	8.35646E-06	0.002903704
347.519997	387.226298	8.73916E-06	0.003037032
347.559997	459.242232	1.03645E-05	0.003602271
347.599997	507.147593	1.14456E-05	0.003978496
347.639997	520.062735	1.17371E-05	0.004080282
347.679997	484.764689	1.09405E-05	0.00380378
347.719997	391.302688	8.83116E-06	0.00307077
347.759997	325.997237	7.3573E-06	0.002558576
347.799997	298.64441	6.73999E-06	0.002344168
347.839997	313.840464	7.08294E-06	0.002463731
347.879997	390.038334	8.80262E-06	0.003062256
347.919997	603.425293	1.36185E-05	0.004738137
347.959997	1102.829367	2.48893E-05	0.008660489
347.999997	2050.787495	4.62834E-05	0.016106629
348.039997	3405.266388	7.68521E-05	0.026747611
348.079997	4943.122562	0.000111559	0.038831592
348.119997	6239.439298	0.000140815	0.049020675
348.159997	6774.111599	0.000152882	0.053227488
348.199997	6267.680738	0.000141453	0.049253873
348.239997	4951.125424	0.00011174	0.038912339
348.279997	3448.399552	7.78256E-05	0.027105091
348.319997	2108.62455	4.75887E-05	0.016576102
348.359997	1132.171402	2.55515E-05	0.008901131
348.399997	594.843079	1.34248E-05	0.004677193
348.439997	362.30687	8.17676E-06	0.002849111
348.479997	310.464586	7.00675E-06	0.002441713
348.519997	353.037582	7.96757E-06	0.002776856
348.559997	448.679954	1.01261E-05	0.003529547
348.599997	528.887264	1.19362E-05	0.004160976
348.639997	563.955231	1.27277E-05	0.00443738
348.679997	526.756563	1.18882E-05	0.004145164
348.719997	420.788743	9.49662E-06	0.00331166

348.759997	318.648362	7.19145E-06	0.00250809
348.799997	281.748714	6.35868E-06	0.002217906
348.839997	339.349602	7.65865E-06	0.002671643
348.879997	513.789669	1.15955E-05	0.004045444
348.919997	897.679194	2.02594E-05	0.007068899
348.959997	1695.916888	3.82745E-05	0.013356263
348.999997	3219.232357	7.26536E-05	0.025356103
349.039997	5819.825103	0.000131345	0.045844779
349.079997	9231.456693	0.000208341	0.072727717
349.119997	12523.06249	0.000282628	0.098671113
349.159997	14853.10018	0.000335214	0.117043243
349.199997	15276.05721	0.000344759	0.120389958
349.239997	13342.38749	0.000301119	0.105162834
349.279997	9826.271086	0.000221765	0.077458166
349.319997	6090.715845	0.000137459	0.048017168
349.359997	3188.017925	7.19491E-05	0.025136146
349.399997	1470.791137	3.31937E-05	0.011597881
349.439997	670.506072	1.51324E-05	0.005287862
349.479997	422.51502	9.53558E-06	0.003332493
349.519997	440.205403	9.93482E-06	0.003472419
349.559997	513.081553	1.15795E-05	0.004047743
349.599997	566.635411	1.27882E-05	0.004470745
349.639997	594.533949	1.34178E-05	0.004691401
349.679997	575.495602	1.29881E-05	0.004541691
349.719997	475.175031	1.0724E-05	0.003750411
349.759997	355.205517	8.01649E-06	0.002803849
349.799997	285.567995	6.44487E-06	0.002254416
349.839997	274.97839	6.20588E-06	0.002171065
349.879997	342.199338	7.72296E-06	0.00270211
349.919997	530.222365	1.19664E-05	0.004187276
349.959997	976.733802	2.20435E-05	0.007714351
349.999997	1801.927484	4.0667E-05	0.014233448
350.039997	3146.017636	7.10012E-05	0.024853272
350.079997	4820.614182	0.000108795	0.038086795
350.119997	6364.967747	0.000143648	0.050294197
350.159997	7298.973936	0.000164728	0.057681042
350.199997	7161.65977	0.000161629	0.056602365
350.239997	6017.145562	0.000135799	0.047562099
350.279997	4217.509748	9.51833E-05	0.033340813
350.319997	2473.707386	5.58281E-05	0.01955771
350.359997	1285.991525	2.9023E-05	0.010168511
350.399997	651.958566	1.47138E-05	0.005155714
350.439997	388.925647	8.77751E-06	0.003075991
350.479997	335.085099	7.5624E-06	0.002650471
350.519997	366.980603	8.28224E-06	0.002903091
350.559997	443.801716	1.0016E-05	0.003511204

350.599997	527.674611	1.19089E-05	0.004175254
350.639997	590.121932	1.33182E-05	0.004669904
350.679997	591.110505	1.33405E-05	0.004678261
350.719997	504.013261	1.13749E-05	0.003989397
350.759997	397.660862	8.97465E-06	0.003147949
350.799997	350.791676	7.91688E-06	0.002777241
350.839997	376.627776	8.49996E-06	0.002982127
350.879997	502.015811	1.13298E-05	0.003975399
350.919997	854.627257	1.92877E-05	0.006768456
350.959997	1678.743152	3.78869E-05	0.013296784
350.999997	3243.568325	7.32028E-05	0.025694189
351.039997	5752.423648	0.000129824	0.045573483
351.079997	9007.090329	0.000203277	0.071366658
351.119997	12324.76948	0.000278153	0.097665038
351.159997	14544.86518	0.000328257	0.115270849
351.199997	14853.28422	0.000335218	0.117728538
351.239997	13199.29852	0.00029789	0.104630806
351.279997	10066.308	0.000227183	0.079804686
351.319997	6516.446268	0.000147067	0.051667619
351.359997	3559.419008	8.03311E-05	0.028225145
351.399997	1667.487169	3.76329E-05	0.013224188
351.439997	732.601122	1.65338E-05	0.005810634
351.479997	416.401698	9.39761E-06	0.003303071
351.519997	398.174115	8.98624E-06	0.003158841
351.559997	499.640949	1.12762E-05	0.003964261
351.599997	586.421025	1.32347E-05	0.004653322
351.639997	605.191721	1.36583E-05	0.004802817
351.679997	543.740594	1.22715E-05	0.00431563
351.719997	429.839141	9.70087E-06	0.00341199
351.759997	339.723056	7.66708E-06	0.002696971
351.799997	283.217417	6.39182E-06	0.002248643
351.839997	275.155664	6.20988E-06	0.002184884
351.879997	353.982934	7.9889E-06	0.002811135
351.919997	560.213081	1.26432E-05	0.004449405
351.959997	989.377706	2.23289E-05	0.007858871
351.999997	1847.204117	4.16888E-05	0.014674466
352.039997	3344.650342	7.54841E-05	0.026573422
352.079997	5263.107899	0.000118781	0.041820419
352.119997	7036.315847	0.0001588	0.055916602
352.159997	8136.560981	0.000183631	0.064667439
352.199997	8159.636439	0.000184152	0.064858203
352.239997	7132.378905	0.000160968	0.056699318
352.279997	5501.372069	0.000124158	0.043738487
352.319997	3868.006213	8.72955E-05	0.030755955
352.359997	2555.047955	5.76639E-05	0.020318442
352.399997	1666.948021	3.76207E-05	0.013257533

352.439997	1136.023841	2.56385E-05	0.009036024
352.479997	861.081941	1.94334E-05	0.006849892
352.519997	821.025721	1.85294E-05	0.006531986
352.559997	895.293713	2.02055E-05	0.007123662
352.599997	868.72028	1.96058E-05	0.006913007
352.639997	751.502241	1.69604E-05	0.005980901
352.679997	590.213257	1.33203E-05	0.0046978
352.719997	438.785006	9.90277E-06	0.003492904
352.759997	317.874424	7.17398E-06	0.002530694
352.799997	278.609	6.28782E-06	0.002218342
352.839997	324.180344	7.3163E-06	0.002581483
352.879997	474.915611	1.07182E-05	0.003782233
352.919997	897.838452	2.0263E-05	0.007151205
352.959997	1892.492627	4.27109E-05	0.015075247
352.999997	3814.423788	8.60862E-05	0.03038844
353.039997	6602.594226	0.000149011	0.052606968
353.079997	9448.022511	0.000213229	0.075286792
353.119997	11462.80024	0.000258699	0.091351944
353.159997	12248.25825	0.000276426	0.097622651
353.199997	11976.34631	0.000270289	0.095466235
353.239997	10612.18498	0.000239502	0.084601768
353.279997	8250.687522	0.000186207	0.065783041
353.319997	5653.916297	0.000127601	0.04508399
353.359997	3492.045305	7.88106E-05	0.027848513
353.399997	1996.782151	4.50646E-05	0.015925827
353.439997	1112.889232	2.51164E-05	0.008877126
353.479997	755.477115	1.70501E-05	0.006026857
353.519997	783.775803	1.76887E-05	0.006253319
353.559997	946.961628	2.13716E-05	0.007556145
353.599997	976.733166	2.20435E-05	0.007794584
353.639997	888.518991	2.00526E-05	0.007091414
353.679997	687.543343	1.55169E-05	0.005488016
353.719997	457.766009	1.03311E-05	0.003654331
353.759997	304.282141	6.86722E-06	0.002429349
353.799997	260.566863	5.88063E-06	0.002080567
353.839997	296.939146	6.7015E-06	0.00237126
353.879997	401.232347	9.05526E-06	0.003204474
353.919997	592.108637	1.33631E-05	0.004729457
353.959997	1022.220536	2.30701E-05	0.00816589
353.999997	1887.71727	4.26031E-05	0.015081515
354.039997	3177.854028	7.17197E-05	0.025391656
354.079997	4537.163191	0.000102397	0.03625689
354.119997	5608.750974	0.000126582	0.04482511
354.159997	6101.812983	0.000137709	0.048771168
354.199997	5815.179287	0.00013124	0.046485384
354.239997	4859.499397	0.000109672	0.038850256

354.279997	3634.12934	8.20172E-05	0.029057067
354.319997	2481.92629	5.60136E-05	0.019846745
354.359997	1567.462134	3.53754E-05	0.01253564
354.399997	957.822298	2.16167E-05	0.007660964
354.439997	597.118512	1.34761E-05	0.004776481
354.479997	439.269378	9.9137E-06	0.003514208
354.519997	437.021124	9.86296E-06	0.003496616
354.559997	532.146069	1.20098E-05	0.004258193
354.599997	603.785181	1.36266E-05	0.004831989
354.639997	586.485839	1.32362E-05	0.004694075
354.679997	505.907734	1.14176E-05	0.004049606
354.719997	387.364593	8.74228E-06	0.003101061
354.759997	292.821233	6.60857E-06	0.002344455
354.799997	271.199435	6.12059E-06	0.002171587
354.839997	391.542673	8.83657E-06	0.003135569
354.879997	734.905922	1.65858E-05	0.00588597
354.919997	1473.190948	3.32479E-05	0.011800333
354.959997	2935.071228	6.62405E-05	0.023512717
354.999997	5316.432345	0.000119984	0.042594488
355.039997	8108.177745	0.00018299	0.064968868
355.079997	10352.08713	0.000233632	0.082958116
355.119997	11402.50821	0.000257339	0.091386128
355.159997	10948.00526	0.000247081	0.087753369
355.199997	9160.85642	0.000206748	0.073436807
355.239997	6738.046118	0.000152068	0.054020748
355.279997	4450.27968	0.000100437	0.03568312
355.319997	2644.909621	5.96919E-05	0.021209735
355.359997	1381.303405	3.11741E-05	0.011078026
355.399997	696.333673	1.57153E-05	0.005585211
355.439997	414.971307	9.36532E-06	0.003328811
355.479997	351.138748	7.92471E-06	0.002817077
355.519997	388.139361	8.75976E-06	0.003114272
355.559997	483.93072	1.09216E-05	0.003883299
355.599997	578.149512	1.3048E-05	0.004639879
355.639997	580.345656	1.30976E-05	0.004658028
355.679997	495.599816	1.1185E-05	0.00397828
355.719997	401.471804	9.06066E-06	0.003223058
355.759997	333.787358	7.53312E-06	0.002679981
355.799997	315.077871	7.11087E-06	0.002530047
355.839997	340.763451	7.69056E-06	0.002736608
355.879997	427.967736	9.65864E-06	0.003437315
355.919997	715.944035	1.61579E-05	0.005750905
355.959997	1326.877798	2.99458E-05	0.010659501
355.999997	2352.804882	5.30995E-05	0.018903432
356.039997	3872.127381	8.73885E-05	0.031113809
356.079997	5537.917371	0.000124983	0.044503975

356.119997	6585.68484	0.00014863	0.05293002
356.159997	6533.251251	0.000147446	0.052514502
356.199997	5560.740286	0.000125498	0.044702445
356.239997	4148.597604	9.36281E-05	0.033354063
356.279997	2721.420122	6.14187E-05	0.021882241
356.319997	1605.424129	3.62322E-05	0.012910253
356.359997	877.144532	1.97959E-05	0.007054478
356.399997	478.060937	1.07892E-05	0.00384526
356.439997	317.409615	7.16349E-06	0.002553355
356.479997	298.038908	6.72632E-06	0.0023978
356.519997	375.530405	8.4752E-06	0.003021578
356.559997	490.07499	1.10603E-05	0.003943664
356.599996	563.727049	1.27225E-05	0.004536856
356.639996	561.451834	1.26712E-05	0.004519052
356.679996	501.253447	1.13126E-05	0.004034975
356.719996	415.712426	9.38205E-06	0.003346765
356.759996	350.622895	7.91307E-06	0.002823067
356.799996	320.666167	7.23699E-06	0.002582158
356.839996	362.006352	8.16998E-06	0.002915375
356.879996	542.58628	1.22454E-05	0.004370144
356.919996	951.389985	2.14715E-05	0.007663624
356.959996	1870.076171	4.2205E-05	0.015065502
356.999996	3642.855968	8.22142E-05	0.029350464
357.039996	6183.718803	0.000139558	0.049827759
357.079996	8926.366656	0.000201456	0.071935787
357.119996	10804.53215	0.000243843	0.087081297
357.159996	10907.15382	0.000246159	0.087918244
357.199996	9509.381527	0.000214613	0.07665994
357.239996	7200.384784	0.000162503	0.058052448
357.279996	4718.872182	0.000106498	0.038049738
357.319996	2662.152111	6.00811E-05	0.021468166
357.359996	1326.933536	2.9947E-05	0.010701875
357.399996	655.400821	1.47915E-05	0.005286476
357.439996	372.282619	8.4019E-06	0.003003175
357.479996	306.077317	6.90774E-06	0.002469378
357.519996	357.726858	8.0734E-06	0.002886401
357.559996	470.639294	1.06217E-05	0.003797885
357.599996	537.279515	1.21257E-05	0.004336132
357.639996	546.467919	1.2333E-05	0.004410781
357.679996	515.132237	1.16258E-05	0.004158322
357.719996	416.675283	9.40378E-06	0.00336392
357.759996	305.231674	6.88865E-06	0.002464485
357.799996	267.272822	6.03198E-06	0.002158241
357.839996	290.288644	6.55141E-06	0.002344357
357.879996	372.513279	8.40711E-06	0.003008735
357.919996	589.25244	1.32986E-05	0.004759837

357.959996	1077.010512	2.43066E-05	0.0087008
357.999996	1989.742975	4.49057E-05	0.01607625
358.039996	3324.14761	7.50214E-05	0.026860655
358.079996	4842.071878	0.000109279	0.039130559
358.119996	5957.699431	0.000134457	0.048151732
358.159996	6218.301861	0.000140338	0.050263605
358.199996	5581.26262	0.000125961	0.045119345
358.239996	4358.643348	9.83685E-05	0.035239534
358.279996	3025.604389	6.82837E-05	0.024464675
358.319996	1895.783729	4.27852E-05	0.015330792
358.359996	1104.044423	2.49167E-05	0.008929165
358.399996	605.455872	1.36643E-05	0.004897284
358.439996	370.050515	8.35152E-06	0.00299352
358.479996	347.40235	7.84039E-06	0.002810622
358.519996	413.012344	9.32111E-06	0.003341806
358.559996	514.682671	1.16157E-05	0.004164915
358.599996	588.702037	1.32862E-05	0.004764426
358.639996	584.73866	1.31967E-05	0.004732878
358.679996	509.637831	1.15018E-05	0.004125471
358.719996	412.694119	9.31393E-06	0.003341093
358.759996	328.581898	7.41564E-06	0.002660433
358.799996	269.178328	6.07498E-06	0.002179703
358.839996	282.871679	6.38402E-06	0.002290842
358.879996	420.716946	9.495E-06	0.003407564
358.919996	835.449536	1.88549E-05	0.006767412
358.959996	1795.811482	4.0529E-05	0.014548277
358.999996	3570.405369	8.05791E-05	0.028927888
359.039996	6186.634316	0.000139624	0.0501305
359.079996	9056.591879	0.000204395	0.073394035
359.119996	11138.29011	0.000251376	0.090274038
359.159996	11565.90906	0.000261026	0.093750261
359.199996	10329.82077	0.00023313	0.083740175
359.239996	8004.867446	0.000180659	0.064899834
359.279996	5405.013069	0.000121984	0.043826273
359.319996	3232.554084	7.29542E-05	0.026213918
359.359996	1747.141175	3.94305E-05	0.01416976
359.399996	903.24738	2.0385E-05	0.007326382
359.439996	510.489963	1.1521E-05	0.004141125
359.479996	379.859847	8.57291E-06	0.003081789
359.519996	402.466269	9.0831E-06	0.003265557
359.559996	463.43009	1.0459E-05	0.003760628
359.599996	509.5304	1.14994E-05	0.004135181
359.639996	519.769074	1.17305E-05	0.004218744
359.679996	502.67353	1.13446E-05	0.004080441
359.719996	441.98308	9.97494E-06	0.003588186
359.759996	353.036711	7.96755E-06	0.002866405

359.799996	314.51409	7.09814E-06	0.002553913
359.839996	337.434153	7.61542E-06	0.002740332
359.879996	409.028615	9.23121E-06	0.003322126
359.919996	594.28598	1.34122E-05	0.004827321
359.959996	1055.446634	2.382E-05	0.008574233
359.999996	2011.852204	4.54047E-05	0.016345693
360.039996	3493.349302	7.884E-05	0.028385563
360.079996	5207.861163	0.000117534	0.042321704
360.119996	6623.034565	0.000149473	0.053828096
360.159996	7110.804332	0.000160481	0.057798818
360.199996	6432.208381	0.000145166	0.052288787
360.239996	4966.489427	0.000112087	0.04037813
360.279996	3391.560606	7.65428E-05	0.027576839
360.319996	2051.051067	4.62894E-05	0.016678984
360.359996	1110.658342	2.5066E-05	0.009032788
360.399996	581.161071	1.3116E-05	0.004727005
360.439996	356.71602	8.05058E-06	0.002901752
360.479996	332.11158	7.4953E-06	0.002701904
360.519996	401.180775	9.05409E-06	0.003264181
360.559996	496.971208	1.12159E-05	0.004044022
360.599996	613.508969	1.3846E-05	0.004992883
360.639996	621.688836	1.40307E-05	0.005060014
360.679996	520.466427	1.17462E-05	0.00423662
360.719996	411.206768	9.28036E-06	0.003347613
360.759996	318.104644	7.17918E-06	0.00258996
360.799996	285.884844	6.45202E-06	0.00232789
360.839996	344.890191	7.78369E-06	0.002808667
360.879996	552.396097	1.24668E-05	0.004499023
360.919996	1030.113698	2.32482E-05	0.008390752
360.959996	2144.601305	4.84007E-05	0.017470704
360.999996	4182.511843	9.43935E-05	0.034076041
361.039996	7084.866468	0.000159896	0.057728695
361.079996	10292.53864	0.000232288	0.083874644
361.119996	12702.34445	0.000286674	0.103523794
361.159996	13364.41758	0.000301616	0.108931738
361.199996	12022.05027	0.000271321	0.098001119
361.239996	9232.365971	0.000208362	0.075268558
361.279996	6120.421816	0.000138129	0.049903387
361.319996	3547.218985	8.00558E-05	0.028925758
361.359996	1802.2888	4.06751E-05	0.014698371
361.399996	843.338213	1.9033E-05	0.006878515
361.439996	446.483246	1.00765E-05	0.003642052
361.479996	352.300515	7.95093E-06	0.002874103
361.519996	391.886489	8.84433E-06	0.003197403
361.559996	496.504508	1.12054E-05	0.00405143
361.599996	579.606806	1.30809E-05	0.00473006

361.639996	569.173445	1.28455E-05	0.004645429
361.679996	475.884881	1.07401E-05	0.003884464
361.719996	385.4698	8.69952E-06	0.003146789
361.759996	346.330892	7.81621E-06	0.002827591
361.799996	354.115917	7.9919E-06	0.00289147
361.839996	405.458932	9.15064E-06	0.003311069
361.879996	488.822646	1.1032E-05	0.003992277
361.919996	673.462358	1.51991E-05	0.005500861
361.959996	1129.439534	2.54899E-05	0.009226316
361.999996	2006.585351	4.52858E-05	0.016393473
362.039996	3327.032644	7.50865E-05	0.027184313
362.079996	4913.764439	0.000110897	0.04015352
362.119996	6142.132076	0.000138619	0.050196844
362.159996	6500.270867	0.000146702	0.053129617
362.199996	5875.966536	0.000132612	0.048032204
362.239996	4593.816864	0.000103676	0.037555611
362.279996	3122.262497	7.04651E-05	0.025528102
362.319996	1839.257353	4.15095E-05	0.015039713
362.359996	985.27245	2.22362E-05	0.008057519
362.399996	539.289037	1.2171E-05	0.004410771
362.439996	360.870171	8.14434E-06	0.002951834
362.479996	361.75927	8.1644E-06	0.002959433
362.519996	449.729005	1.01498E-05	0.00367949
362.559996	574.077778	1.29561E-05	0.004697377
362.599996	635.307341	1.4338E-05	0.005198959
362.639996	599.156091	1.35221E-05	0.004903661
362.679996	496.075856	1.11957E-05	0.004060471
362.719996	388.600296	8.77017E-06	0.003181115
362.759996	316.820278	7.15019E-06	0.002593804
362.799996	290.742004	6.56164E-06	0.002380564
362.839996	356.838283	8.05334E-06	0.002922075
362.879996	524.505483	1.18374E-05	0.00429554
362.919996	920.344822	2.07709E-05	0.007538175
362.959996	1842.334759	4.15789E-05	0.015091488
362.999996	3629.351628	8.19094E-05	0.029733116
363.039996	6276.653268	0.000141655	0.051426547
363.079996	9358.59235	0.00021121	0.076686269
363.119996	11827.47271	0.00026693	0.09692747
363.159996	12711.79747	0.000286888	0.104186086
363.199996	11621.37897	0.000262278	0.095259491
363.239996	9179.153235	0.000207161	0.075249053
363.279996	6307.926679	0.000142361	0.051716947
363.319996	3780.113441	8.53119E-05	0.030995517
363.359996	1975.771521	4.45904E-05	0.016202372
363.399996	961.574765	2.17014E-05	0.00788629
363.439996	529.812808	1.19571E-05	0.004345702

363.479996	407.886053	9.20542E-06	0.003345986
363.519996	464.869408	1.04915E-05	0.003813854
363.559996	572.887876	1.29293E-05	0.004700569
363.599996	609.579512	1.37574E-05	0.005002176
363.639996	573.744358	1.29486E-05	0.004708633
363.679996	492.225161	1.11088E-05	0.004040062
363.719996	397.845746	8.97882E-06	0.003265778
363.759996	324.316164	7.31936E-06	0.002662492
363.799996	308.338962	6.95878E-06	0.002531604
363.839996	344.71039	7.77963E-06	0.002830542
363.879996	435.878923	9.83718E-06	0.003579553
363.919996	631.821366	1.42593E-05	0.005189255
363.959996	1025.763899	2.31501E-05	0.008425697
363.999996	1808.554335	4.08166E-05	0.014857225
364.039996	2997.396102	6.76471E-05	0.024626234
364.079996	4445.342496	0.000100325	0.036526394
364.119996	5635.568693	0.000127187	0.046311307
364.159996	6028.561154	0.000136056	0.049546236
364.199996	5447.85914	0.000122951	0.044778605
364.239996	4240.978425	9.5713E-05	0.034862494
364.279996	2857.91125	6.44991E-05	0.023495723
364.319996	1664.754929	3.75712E-05	0.013687939
364.359996	902.055669	2.03581E-05	0.007417692
364.399996	511.17144	1.15364E-05	0.004203874
364.439996	347.685322	7.84677E-06	0.002859678
364.479996	350.004092	7.8991E-06	0.002879066
364.519996	421.82998	9.52012E-06	0.003470272
364.559996	501.429111	1.13166E-05	0.004125564
364.599996	560.687673	1.26539E-05	0.004613626
364.639996	574.586836	1.29676E-05	0.004728515
364.679996	503.591708	1.13654E-05	0.004144721
364.719996	394.810101	8.91031E-06	0.00324977
364.759996	310.901723	7.01662E-06	0.002559382
364.799996	295.354401	6.66574E-06	0.002431661
364.839996	354.456431	7.99959E-06	0.00291857
364.879996	507.170343	1.14461E-05	0.004176463
364.919996	928.684554	2.09591E-05	0.007648401
364.959996	1897.1147	4.28152E-05	0.015625848
364.999996	3718.273931	8.39163E-05	0.030629436
365.039996	6521.036326	0.000147171	0.053723194
365.079996	9779.952843	0.00022072	0.080580425
365.119996	12316.56312	0.000277968	0.101491556
365.159996	13161.3855	0.000297034	0.108464985
365.199996	12205.67538	0.000275465	0.100599851
365.239996	9859.237755	0.000222509	0.081269282
365.279996	6856.99748	0.000154753	0.056528132

365.319996	4055.925453	9.15366E-05	0.033440146
365.359996	2064.685551	4.65971E-05	0.017024708
365.399996	993.691298	2.24262E-05	0.008194544
365.439996	542.927417	1.22531E-05	0.004477779
365.479996	413.044497	9.32184E-06	0.003406946
365.519996	418.353861	9.44166E-06	0.003451117
365.559996	484.59616	1.09367E-05	0.003998006
365.599996	523.249284	1.1809E-05	0.004317373
365.639996	516.194033	1.16498E-05	0.004259626
365.679996	484.84289	1.09422E-05	0.004001354
365.719996	420.75048	9.49575E-06	0.003472786
365.759996	338.529946	7.64015E-06	0.002794461
365.799996	272.378675	6.14721E-06	0.002248648
365.839996	260.141566	5.87103E-06	0.002147859
365.879996	341.223902	7.70095E-06	0.002817623
365.919996	532.911328	1.20271E-05	0.004400944
365.959996	977.369237	2.20579E-05	0.008072295
365.999996	1848.208924	4.17115E-05	0.015266409
366.039996	3170.354495	7.15505E-05	0.026190339
366.079996	4717.473946	0.000106467	0.038975372
366.119996	6048.723497	0.000136511	0.049979503
366.159996	6700.51765	0.000151221	0.055371209
366.199996	6256.909471	0.00014121	0.051711003
366.239996	4967.304199	0.000112105	0.041057385
366.279996	3444.969296	7.77482E-05	0.028477595
366.319996	2131.091342	4.80958E-05	0.01761844
366.359996	1182.692143	2.66917E-05	0.009778776
366.399996	623.375525	1.40687E-05	0.005154778
366.439996	386.78247	8.72914E-06	0.003198707
366.479996	344.565555	7.77636E-06	0.002849882
366.519996	400.125475	9.03027E-06	0.003309776
366.559996	481.489472	1.08665E-05	0.003983241
366.599996	532.521182	1.20183E-05	0.004405895
366.639996	529.060077	1.19401E-05	0.004377736
366.679996	483.934902	1.09217E-05	0.004004782
366.719996	416.214261	9.39338E-06	0.003444739
366.759996	354.118267	7.99196E-06	0.00293113
366.799996	322.479962	7.27792E-06	0.002669542
366.839996	366.609693	8.27387E-06	0.003035186
366.879996	533.579562	1.20421E-05	0.004418023
366.919996	928.865984	2.09632E-05	0.007691821
366.959996	1776.145524	4.00851E-05	0.014709639
366.999996	3393.115801	7.65779E-05	0.028104088
367.039996	5880.160886	0.000132707	0.048708792
367.079996	9062.512303	0.000204528	0.07507824
367.119996	12236.31762	0.000276157	0.101382626

367.159996	14248.72353	0.000321574	0.118069051
367.199996	14334.9491	0.00032352	0.118796482
367.239996	12394.05444	0.000279717	0.102723103
367.279996	9112.051308	0.000205646	0.075529774
367.319996	5742.144604	0.000129592	0.047601808
367.359996	3171.682562	7.15805E-05	0.026295797
367.399996	1582.903342	3.57239E-05	0.013124969
367.439996	787.94348	1.77828E-05	0.006534107
367.479996	508.901779	1.14852E-05	0.004220583
367.519996	469.34129	1.05924E-05	0.003892911
367.559996	496.72601	1.12104E-05	0.0041205
367.599996	533.369542	1.20374E-05	0.004424951
367.639996	539.115294	1.21671E-05	0.004473106
367.679996	470.072615	1.06089E-05	0.003900674
367.719996	357.408285	8.06621E-06	0.002966106
367.759996	281.366871	6.35006E-06	0.002335297
367.799996	250.998261	5.66468E-06	0.00208347
367.839996	281.859528	6.36118E-06	0.002339895
367.879996	362.116977	8.17248E-06	0.00300649
367.919996	528.107315	1.19186E-05	0.004385108
367.959996	866.026097	1.9545E-05	0.007191779
367.999996	1572.431487	3.54876E-05	0.013059432
368.039996	2698.194544	6.08945E-05	0.022411609
368.079996	4111.015139	9.27799E-05	0.03415042
368.119996	5446.146588	0.000122912	0.045246344
368.159996	6099.55627	0.000137658	0.050680349
368.199996	5911.506369	0.000133414	0.049123205
368.239996	4951.683882	0.000111753	0.04115178
368.279996	3591.393419	8.10527E-05	0.029850105
368.319996	2272.043827	5.12769E-05	0.018886295
368.359996	1296.595536	2.92624E-05	0.010779081
368.399996	701.293321	1.58272E-05	0.005830745
368.439996	428.0004	9.65937E-06	0.003558899
368.479996	347.170309	7.83515E-06	0.002887096
368.519996	371.381396	8.38156E-06	0.003088773
368.559996	455.821164	1.02872E-05	0.003791468
368.599996	521.863162	1.17777E-05	0.004341269
368.639996	531.819569	1.20024E-05	0.004424575
368.679996	443.857328	1.00172E-05	0.003693157
368.719996	343.062698	7.74245E-06	0.002854795
368.759996	272.621096	6.15268E-06	0.002268862
368.799996	243.302461	5.491E-06	0.00202508
368.839996	273.527981	6.17315E-06	0.002276903
368.879996	438.483484	9.89596E-06	0.003650422
368.919996	924.69912	2.08692E-05	0.007699055
368.959996	2082.320917	4.69951E-05	0.017339306

368.999996	4115.574742	9.28828E-05	0.034273749
369.039996	6830.057659	0.000154145	0.056885629
369.079996	9621.228307	0.000217138	0.080141189
369.119996	11773.78163	0.000265718	0.098081772
369.159996	12671.47444	0.000285978	0.105571463
369.199996	11875.38337	0.000268011	0.098949609
369.239996	9688.426015	0.000218654	0.080735906
369.279996	6915.84248	0.000156081	0.057637566
369.319996	4285.35313	9.67145E-05	0.035718581
369.359996	2298.407968	5.18719E-05	0.019159392
369.399996	1113.267607	2.51249E-05	0.009281138
369.439996	568.617769	1.28329E-05	0.004740991
369.479996	399.443926	9.01489E-06	0.003330823
369.519996	419.529839	9.4682E-06	0.003498691
369.559996	485.806728	1.0964E-05	0.004051849
369.599996	524.097346	1.18281E-05	0.004371683
369.639996	524.634273	1.18403E-05	0.004376635
369.679996	467.787284	1.05573E-05	0.003902825
369.719996	377.557634	8.52095E-06	0.003150366
369.759996	301.332674	6.80066E-06	0.002514612
369.799996	270.704073	6.10941E-06	0.002259261
369.839996	312.667144	7.05646E-06	0.002609762
369.879996	391.548007	8.83669E-06	0.003268516
369.919996	580.906581	1.31103E-05	0.004849745
369.959996	1078.718172	2.43452E-05	0.009006738
369.999996	2029.53446	4.58038E-05	0.016947393
370.039996	3540.815183	7.99113E-05	0.029570365
370.079996	5143.844	0.000116089	0.042962363
370.119996	6316.574588	0.000142556	0.052762934
370.159996	6643.391501	0.000149932	0.055498863
370.199996	5986.339182	0.000135103	0.050015255
370.239996	4699.650171	0.000106065	0.039269342
370.279996	3278.849943	7.39991E-05	0.027400378
370.319996	2076.333444	4.686E-05	0.017353178
370.359996	1186.765718	2.67836E-05	0.009919592
370.399996	641.159631	1.44701E-05	0.005359718
370.439996	390.722552	8.81806E-06	0.003266564
370.479996	333.284122	7.52176E-06	0.002786661
370.519996	380.211072	8.58083E-06	0.003179371
370.559996	446.015014	1.00659E-05	0.003730034
370.599996	507.407392	1.14515E-05	0.004243918
370.639996	527.425602	1.19033E-05	0.004411825
370.679996	473.193882	1.06793E-05	0.003958613
370.719996	412.572633	9.31119E-06	0.003451844
370.759996	354.76176	8.00648E-06	0.002968482
370.799996	314.736934	7.10317E-06	0.002633857

370.839996	366.735689	8.27671E-06	0.003069336
370.879996	624.821762	1.41014E-05	0.005229911
370.919996	1215.324426	2.74282E-05	0.01017366
370.959996	2489.700298	5.61891E-05	0.020843896
370.999996	4691.308344	0.000105876	0.039280105
371.039996	7588.555072	0.000171263	0.063545467
371.079996	10378.45497	0.000234227	0.086917055
371.119996	12269.73802	0.000276911	0.102767171
371.159996	12585.1951	0.00028403	0.105420694
371.199996	11220.66544	0.000253235	0.094000753
371.239996	8731.981052	0.000197069	0.073159765
371.279996	5986.917416	0.000135116	0.050166012
371.319996	3639.009713	8.21274E-05	0.030495539
371.359996	1971.265161	4.44887E-05	0.016521327
371.399996	980.274391	2.21234E-05	0.008216641
371.439996	499.137976	1.12648E-05	0.004184215
371.479996	354.000291	7.98929E-06	0.002967863
371.519996	383.131758	8.64675E-06	0.003212441
371.559996	505.26251	1.14031E-05	0.004236925
371.599996	589.461967	1.33033E-05	0.004943519
371.639996	608.235508	1.3727E-05	0.005101513
371.679996	556.509107	1.25596E-05	0.004668165
371.719996	447.4786	1.0099E-05	0.003753989
371.759996	337.620387	7.61962E-06	0.002832671
371.799996	286.977728	6.47669E-06	0.002408032
371.839996	313.133535	7.06699E-06	0.002627789
371.879996	395.766792	8.93191E-06	0.003321597
371.919996	599.243761	1.35241E-05	0.005029882
371.959996	1122.624382	2.53361E-05	0.009424004
371.999996	2163.780241	4.88335E-05	0.018166064
372.039996	3654.119116	8.24684E-05	0.030681535
372.079996	5163.046535	0.000116523	0.043355791
372.119996	6208.813855	0.000140124	0.052143047
372.159996	6375.422933	0.000143884	0.053548023
372.199996	5627.429777	0.000127003	0.04727061
372.239996	4335.500363	9.78462E-05	0.036422271
372.279996	2993.515403	6.75595E-05	0.02515104
372.319996	1882.382116	4.24827E-05	0.015817174
372.359996	1104.801441	2.49338E-05	0.009284361
372.399996	623.268997	1.40663E-05	0.005238295
372.439996	372.381972	8.40414E-06	0.003130039
372.479996	294.123314	6.63795E-06	0.002472505
372.519996	347.41218	7.84061E-06	0.002920784
372.559996	448.140405	1.01139E-05	0.003768036
372.599996	511.787605	1.15503E-05	0.004303654
372.639996	515.496121	1.1634E-05	0.004335305

372.679996	488.008537	1.10137E-05	0.004104576
372.719996	414.3735	9.35183E-06	0.003485615
372.759996	364.752967	8.23197E-06	0.003068548
372.799996	408.637193	9.22237E-06	0.0034381
372.839996	570.231593	1.28693E-05	0.004798202
372.879996	869.900607	1.96324E-05	0.007320546
372.919996	1345.42892	3.03645E-05	0.011323513
372.959996	2078.104398	4.68999E-05	0.017491795
372.999996	3335.084841	7.52682E-05	0.028075046
373.039996	5173.810179	0.000116766	0.043558272
373.079996	7236.701297	0.000163322	0.060932271
373.119996	8833.949121	0.00019937	0.074388906
373.159996	9443.913634	0.000213136	0.07953382
373.199996	8927.561103	0.000201483	0.075193313
373.239996	7366.475373	0.000166251	0.062051556
373.279996	5268.978775	0.000118914	0.044388035
373.319996	3290.577858	7.42638E-05	0.027724146
373.359996	1793.537378	4.04776E-05	0.015112732
373.399996	880.637245	1.98748E-05	0.007421234
373.439996	449.447326	1.01434E-05	0.003787951
373.479996	318.354471	7.18482E-06	0.002683385
373.519996	350.485647	7.90997E-06	0.002954533
373.559996	469.378346	1.05932E-05	0.003957202
373.599996	544.04724	1.22784E-05	0.004587206
373.639996	540.635644	1.22014E-05	0.004558929
373.679996	495.312192	1.11785E-05	0.004177184
373.719996	433.276446	9.77845E-06	0.003654401
373.759996	370.560287	8.36303E-06	0.003125766
373.799996	350.459842	7.90939E-06	0.00295653
373.839996	407.171321	9.18929E-06	0.003435324
373.879996	506.477099	1.14305E-05	0.004273629
373.919996	677.13193	1.52819E-05	0.005714217
373.959996	1026.246787	2.3161E-05	0.008661273
373.999996	1745.01673	3.93826E-05	0.014729091
374.039996	2914.829091	6.57836E-05	0.02460571
374.079996	4291.081334	9.68437E-05	0.036227302
374.119996	5456.462314	0.000123145	0.046070915
374.159996	6086.394135	0.000137361	0.051395155
374.199996	5801.450916	0.000130931	0.048994255
374.239996	4810.605042	0.000108569	0.040630733
374.279996	3545.283565	8.00121E-05	0.029946933
374.319996	2278.365812	5.14195E-05	0.019247363
374.359996	1291.379678	2.91446E-05	0.010910588
374.399996	688.739833	1.55439E-05	0.005819636
374.439996	419.371975	9.46464E-06	0.00354394
374.479996	373.30633	8.425E-06	0.003154995

374.519996	410.833424	9.27194E-06	0.003472526
374.559996	474.907127	1.0718E-05	0.004014531
374.599996	549.809742	1.24084E-05	0.004648202
374.639996	545.372504	1.23083E-05	0.004611181
374.679996	489.650596	1.10507E-05	0.004140488
374.719996	405.583018	9.15344E-06	0.003429978
374.759996	356.194287	8.03881E-06	0.003012624
374.799996	352.336097	7.95173E-06	0.00298031
374.839996	448.631474	1.0125E-05	0.00379525
374.879996	636.706955	1.43696E-05	0.005386872
374.919996	980.642842	2.21317E-05	0.008297633
374.959996	1814.049553	4.09406E-05	0.015351077
374.999996	3495.15547	7.88808E-05	0.029580296
375.039996	5868.40581	0.000132442	0.049670952
375.079996	8314.601861	0.000187649	0.07038338
375.119996	10123.18387	0.000228466	0.085702225
375.159996	10661.08413	0.000240606	0.090265678
375.199996	9518.320188	0.000214815	0.080598673
375.239996	7271.115061	0.000164099	0.061576485
375.279996	4824.699606	0.000108887	0.040863019
375.319996	2748.092186	6.20206E-05	0.023277576
375.359996	1374.166794	3.1013E-05	0.011641051
375.399996	671.93522	1.51646E-05	0.005692807
375.439996	376.262961	8.49173E-06	0.003188135
375.479996	285.10606	6.43445E-06	0.002416006
375.519996	295.501245	6.66905E-06	0.002504362
375.559996	389.575503	8.79218E-06	0.00330199
375.599996	460.995123	1.0404E-05	0.003907749
375.639996	488.426282	1.10231E-05	0.004140718
375.679996	471.521356	1.06416E-05	0.003997829
375.719996	397.921248	8.98053E-06	0.003374164
375.759996	329.032548	7.42581E-06	0.002790321
375.799996	312.08366	7.04329E-06	0.00264687
375.839996	339.145137	7.65403E-06	0.002876692
375.879996	426.563875	9.62695E-06	0.003618579
375.919996	584.654631	1.31948E-05	0.004960204
375.959996	926.582471	2.09117E-05	0.007861954
375.999996	1578.943824	3.56346E-05	0.013398595
376.039996	2756.044811	6.22001E-05	0.023389723
376.079996	4419.376336	9.97392E-05	0.037509906
376.119996	6106.918383	0.000137825	0.051838604
376.159996	7605.605813	0.000171648	0.064567084
376.199996	8602.095376	0.000194137	0.073034456
376.239996	8846.575657	0.000199655	0.075118155
376.279996	8156.177023	0.000184074	0.069263196
376.319996	6511.340409	0.000146952	0.055300932

376.359996	4504.523249	0.000101661	0.038261064
376.399996	2701.253123	6.09635E-05	0.022946669
376.439996	1449.580181	3.2715E-05	0.012315237
376.479996	800.030931	1.80556E-05	0.006797566
376.519996	560.416289	1.26478E-05	0.004762155
376.559996	518.484298	1.17015E-05	0.004406305
376.599996	581.340528	1.312E-05	0.004941009
376.639996	610.191143	1.37712E-05	0.005186771
376.679996	516.335639	1.1653E-05	0.004389443
376.719996	404.3783	9.12625E-06	0.003438043
376.759996	309.104135	6.97605E-06	0.002628296
376.799996	265.024502	5.98123E-06	0.002253729
376.839996	300.920024	6.79135E-06	0.002559251
376.879996	436.00228	9.83996E-06	0.003708486
376.919996	783.577027	1.76842E-05	0.006665544
376.959996	1548.138847	3.49393E-05	0.013170732
376.999996	3119.076561	7.03932E-05	0.026538241
377.039996	5725.242	0.000129211	0.048717616
377.079996	8988.323377	0.000202854	0.076492162
377.119996	12107.33044	0.000273246	0.103046373
377.159996	13817.42008	0.00031184	0.117613545
377.199996	13462.14259	0.000303822	0.114601584
377.239996	11378.13431	0.000256789	0.096870947
377.279996	8348.275364	0.000188409	0.071082927
377.319996	5321.424701	0.000120097	0.045315054
377.359996	3010.497178	6.79427E-05	0.025638868
377.399996	1566.06188	3.53438E-05	0.013338763
377.439996	798.680525	1.80251E-05	0.006803396
377.479996	491.719683	1.10974E-05	0.004189057
377.519996	436.731601	9.85642E-06	0.003720997
377.559996	502.710251	1.13455E-05	0.004283596
377.599996	564.158383	1.27323E-05	0.004807705
377.639996	540.79625	1.2205E-05	0.004609103
377.679996	487.265793	1.09969E-05	0.004153313
377.719996	405.406786	9.14947E-06	0.003455936
377.759996	317.907201	7.17472E-06	0.002710323
377.799996	282.727802	6.38077E-06	0.002410656
377.839996	303.682089	6.85368E-06	0.002589595
377.879996	392.780785	8.86452E-06	0.003349723
377.919996	553.334645	1.2488E-05	0.004719462
377.959996	865.046067	1.95229E-05	0.007378869
377.999996	1514.420381	3.41784E-05	0.012919419
378.039996	2659.006945	6.00101E-05	0.022686212
378.079996	4078.192742	9.20391E-05	0.034798154
378.119996	5441.507922	0.000122807	0.046435879
378.159996	6309.013436	0.000142386	0.053844555

378.199996	6176.024994	0.000139384	0.052715135
378.239996	5159.962587	0.000116453	0.044047246
378.279996	3732.669145	8.42411E-05	0.031866739
378.319996	2320.739586	5.23759E-05	0.019814835
378.359996	1268.377187	2.86255E-05	0.010830746
378.399996	655.945702	1.48038E-05	0.005601751
378.439996	372.602415	8.40912E-06	0.003182346
378.479996	287.655391	6.49198E-06	0.002457085
378.519996	311.371434	7.02722E-06	0.002659943
378.559996	401.97547	9.07203E-06	0.003434306
378.599996	480.522109	1.08447E-05	0.004105809
378.639996	504.122519	1.13773E-05	0.004307917
378.679996	479.735124	1.0827E-05	0.004099951
378.719996	423.55965	9.55915E-06	0.003620242
378.759996	367.69407	8.29834E-06	0.00314308
378.799996	325.795461	7.35275E-06	0.002785222
378.839996	331.751185	7.48716E-06	0.002836436
378.879996	424.795242	9.58704E-06	0.003632337
378.919996	713.259899	1.60973E-05	0.006099582
378.959996	1367.858129	3.08707E-05	0.011698742
378.999996	2603.554338	5.87586E-05	0.022269507
379.039996	4603.922888	0.000103904	0.03938382
379.079996	7088.402322	0.000159975	0.060643462
379.119996	9188.334239	0.000207368	0.078617319
379.159996	10012.60149	0.00022597	0.085678962
379.199996	9347.553783	0.000210961	0.079996512
379.239996	7496.324278	0.000169182	0.064160428
379.279996	5183.427495	0.000116983	0.044369213
379.319996	3088.400626	6.97009E-05	0.026438945
379.359996	1625.713943	3.66901E-05	0.013918756
379.399996	857.443982	1.93513E-05	0.007341889
379.439996	521.929566	1.17792E-05	0.004469508
379.479996	388.917345	8.77732E-06	0.003330818
379.519996	390.982753	8.82394E-06	0.00334886
379.559996	510.516292	1.15216E-05	0.004373154
379.599996	632.755392	1.42804E-05	0.005420843
379.639996	722.376936	1.6303E-05	0.006189287
379.679996	811.394794	1.83121E-05	0.006952719
379.719996	823.356555	1.8582E-05	0.007055961
379.759996	701.650624	1.58353E-05	0.006013605
379.799996	549.776919	1.24077E-05	0.004712444
379.839996	427.373752	9.64523E-06	0.003663644
379.879996	422.295032	9.53061E-06	0.003620488
379.919996	544.012775	1.22776E-05	0.00466451
379.959996	829.184017	1.87135E-05	0.007110392
379.999996	1393.727314	3.14545E-05	0.011952704

380.039996	2379.586062	5.37039E-05	0.020409646
380.079996	3664.685356	8.27068E-05	0.031435217
380.119996	4797.796445	0.00010828	0.041159235
380.159996	5325.023446	0.000120178	0.045687006
380.199996	5039.48233	0.000113734	0.043241703
380.239996	4072.702561	9.19152E-05	0.034949844
380.279996	2910.255979	6.56804E-05	0.024976951
380.319996	1829.705302	4.12939E-05	0.015704896
380.359996	1019.204103	2.3002E-05	0.008749047
380.399996	554.481506	1.25139E-05	0.004760278
380.439996	358.165174	8.08329E-06	0.003075206
380.479996	311.567785	7.03165E-06	0.002675403
380.519996	375.930205	8.48422E-06	0.003228416
380.559996	487.365698	1.09992E-05	0.004185842
380.599996	566.913899	1.27945E-05	0.00486957
380.639996	586.820859	1.32437E-05	0.005041093
380.679996	548.744531	1.23844E-05	0.004714493
380.719996	480.158963	1.08365E-05	0.004125679
380.759996	404.116063	9.12034E-06	0.003472659
380.799996	373.767845	8.43542E-06	0.003212208
380.839996	451.79608	1.01964E-05	0.0038832
380.879996	702.35203	1.58511E-05	0.00603737
380.919996	1266.595486	2.85853E-05	0.010888711
380.959996	2557.529162	5.77199E-05	0.021988962
380.999996	4711.310487	0.000106328	0.040510859
381.039996	7428.463627	0.00016765	0.063881385
381.079996	10273.29572	0.000231854	0.088354912
381.119996	12620.55489	0.000284828	0.108553778
381.159996	13520.38572	0.000305136	0.116305741
381.199996	12532.24896	0.000282835	0.107816855
381.239996	10039.22768	0.000226571	0.086378074
381.279996	7055.245035	0.000159227	0.06071009
381.319996	4323.216113	9.7569E-05	0.037204998
381.359996	2343.436892	5.28881E-05	0.020169408
381.399996	1210.011154	2.73083E-05	0.010415373
381.439996	677.39049	1.52878E-05	0.005831363
381.479996	516.217664	1.16503E-05	0.004444362
381.519996	502.801291	1.13475E-05	0.004329308
381.559996	546.312236	1.23295E-05	0.004704446
381.599996	588.367742	1.32786E-05	0.005067129
381.639996	591.5533	1.33505E-05	0.005095098
381.679996	514.808773	1.16185E-05	0.004434555
381.719996	434.751198	9.81173E-06	0.003745333
381.759996	364.831592	8.23374E-06	0.003143313
381.799996	300.610786	6.78437E-06	0.002590271
381.839996	296.728334	6.69675E-06	0.002557085

381.879996	363.578597	8.20546E-06	0.003133502
381.919996	558.838263	1.26122E-05	0.004816852
381.959996	980.884096	2.21372E-05	0.00845552
381.999996	1733.42431	3.9121E-05	0.014944211
382.039996	2930.248123	6.61316E-05	0.025264923
382.079996	4389.994616	9.90761E-05	0.037854982
382.119996	5568.436117	0.000125672	0.048021726
382.159996	5982.33594	0.000135013	0.051596564
382.199996	5614.434531	0.00012671	0.048428549
382.239996	4538.44189	0.000102426	0.039151433
382.279996	3129.203446	7.06218E-05	0.026997287
382.319996	1900.579007	4.28934E-05	0.016399013
382.359996	1083.032517	2.44425E-05	0.009345848
382.399996	598.029507	1.34967E-05	0.005161135
382.439996	399.836905	9.02376E-06	0.003451048
382.479996	363.704233	8.2083E-06	0.00313951
382.519996	396.753799	8.95418E-06	0.003425153
382.559996	448.477854	1.01215E-05	0.003872089
382.599996	469.648897	1.05993E-05	0.0040553
382.639996	454.250951	1.02518E-05	0.003922753
382.679996	431.883379	9.74701E-06	0.003729984
382.719996	369.173243	8.33173E-06	0.003188718
382.759996	327.225263	7.38502E-06	0.00282669
382.799996	336.534587	7.59512E-06	0.002907411
382.839996	390.799166	8.81979E-06	0.003376569
382.879996	509.349379	1.14953E-05	0.004401323
382.919996	805.795095	1.81857E-05	0.006963658
382.959996	1439.966474	3.2498E-05	0.012445448
382.999996	2553.689007	5.76332E-05	0.022073517
383.039996	4290.081239	9.68212E-05	0.037086376
383.079996	6438.901401	0.000145317	0.05566805
383.119996	8365.698149	0.000188802	0.072333881
383.159996	9312.623153	0.000210173	0.08052986
383.199996	8943.824444	0.00020185	0.077348789
383.239996	7449.524567	0.000168125	0.064432375
383.279996	5427.34197	0.000122488	0.046947027
383.319996	3405.852682	7.68654E-05	0.029464026
383.359996	1869.447227	4.21908E-05	0.016174273
383.399996	947.076003	2.13742E-05	0.008194863
383.439996	483.997404	1.09231E-05	0.004188372
383.479996	341.123167	7.69867E-06	0.002952288
383.519996	343.238443	7.74641E-06	0.002970905
383.559996	394.340262	8.89971E-06	0.003413573
383.599996	470.792937	1.06251E-05	0.004075804
383.639996	521.360577	1.17664E-05	0.004514055
383.679996	493.813452	1.11447E-05	0.004275991

383.719996	409.117294	9.23321E-06	0.003542966
383.759996	323.647534	7.30427E-06	0.002803088
383.799996	281.30457	6.34865E-06	0.002436613
383.839996	286.37927	6.46318E-06	0.002480827
383.879996	371.09523	8.3751E-06	0.003215034
383.919996	559.663875	1.26308E-05	0.00484923
383.959996	1000.957944	2.25902E-05	0.008673743
383.999996	1855.429533	4.18745E-05	0.016079793
384.039996	3078.65709	6.9481E-05	0.026683484
384.079996	4571.831032	0.00010318	0.03962932
384.119996	5833.442776	0.000131653	0.050570426
384.159996	6243.904782	0.000140916	0.05413438
384.199996	5730.166267	0.000129322	0.049685462
384.239996	4606.980542	0.000103973	0.039950637
384.279996	3294.937981	7.43622E-05	0.028575891
384.319996	2100.033261	4.73948E-05	0.018214779
384.359996	1243.728869	2.80692E-05	0.010788688
384.399996	736.648548	1.66251E-05	0.006390701
384.439996	456.109862	1.02938E-05	0.003957335
384.479996	348.460472	7.86427E-06	0.003023654
384.519996	371.83352	8.39176E-06	0.003226801
384.559996	471.884232	1.06498E-05	0.004095475
384.599996	550.983608	1.24349E-05	0.004782475
384.639996	582.248518	1.31405E-05	0.005054377
384.679996	539.628044	1.21787E-05	0.004684884
384.719996	446.225996	1.00707E-05	0.003874399
384.759996	372.804604	8.41368E-06	0.003237248
384.799996	343.18786	7.74527E-06	0.002980381
384.839996	385.162109	8.69257E-06	0.003345249
384.879996	528.345107	1.1924E-05	0.004589314
384.919996	879.452467	1.9848E-05	0.007639899
384.959996	1647.503116	3.71819E-05	0.014313525
384.999996	3016.057344	6.80682E-05	0.026206262
385.039996	4943.374343	0.000111565	0.042957015
385.079996	6997.749108	0.000157929	0.060815472
385.119996	8540.687695	0.000192751	0.074232428
385.159996	8980.49268	0.000202677	0.078063154
385.199996	8169.074497	0.000184365	0.071017256
385.239996	6544.923765	0.00014771	0.056903728
385.279996	4612.752785	0.000104103	0.04010896
385.319996	2873.715288	6.48558E-05	0.024990218
385.359996	1585.810914	3.57895E-05	0.013791858
385.399996	785.138369	1.77195E-05	0.006829087
385.439996	400.752926	9.04444E-06	0.003486087
385.479996	295.628244	6.67192E-06	0.002571891
385.519996	340.803716	7.69147E-06	0.002965214

385.559996	443.869844	1.00175E-05	0.003862357
385.599996	548.768059	1.23849E-05	0.004775629
385.639996	583.914272	1.31781E-05	0.005082015
385.679996	529.300564	1.19456E-05	0.00460717
385.719996	425.968996	9.61353E-06	0.00370813
385.759996	334.164057	7.54162E-06	0.002909254
385.799996	284.582438	6.42263E-06	0.00247785
385.839996	292.419503	6.5995E-06	0.002546351
385.879996	345.371803	7.79456E-06	0.003007765
385.919996	497.565585	1.12294E-05	0.004333635
385.959996	854.184998	1.92778E-05	0.007440446
385.999996	1572.118201	3.54805E-05	0.01369548
386.039996	2610.133802	5.89071E-05	0.022740491
386.079996	3815.365998	8.61075E-05	0.033244382
386.119996	4808.496117	0.000108521	0.041902151
386.159996	5112.845339	0.00011539	0.044558924
386.199996	4636.670677	0.000104643	0.040413203
386.239996	3674.755888	8.29341E-05	0.032032475
386.279996	2586.04427	5.83634E-05	0.022544621
386.319996	1625.920015	3.66948E-05	0.014175916
386.359996	935.426862	2.11113E-05	0.008156555
386.399996	527.202472	1.18982E-05	0.004597474
386.439996	332.578735	7.50584E-06	0.002900556
386.479996	277.050575	6.25265E-06	0.002416522
386.519996	317.664439	7.16924E-06	0.002771056
386.559996	412.026872	9.29887E-06	0.003594572
386.599996	519.213401	1.17179E-05	0.004530149
386.639996	573.015786	1.29322E-05	0.005000093
386.679996	548.626559	1.23817E-05	0.00478777
386.719996	468.554427	1.05746E-05	0.004089417
386.759996	369.973404	8.34978E-06	0.003229363
386.799996	309.961315	6.9954E-06	0.002705819
386.839996	354.404022	7.99841E-06	0.003094103
386.879996	500.688942	1.12999E-05	0.004371687
386.919996	827.972778	1.86862E-05	0.007230061
386.959996	1546.989295	3.49134E-05	0.013510087
386.999996	2899.955837	6.5448E-05	0.025328362
387.039996	4965.024603	0.000112054	0.04336926
387.079996	7280.941566	0.000164321	0.06360526
387.119996	9160.037429	0.000206729	0.08002904
387.159996	9851.419979	0.000222333	0.086078376
387.199996	9107.357312	0.00020554	0.07958523
387.239996	7230.662147	0.000163186	0.063192136
387.279996	4925.160209	0.000111154	0.043047723
387.319996	2930.631159	6.61403E-05	0.025617446
387.359996	1525.065491	3.44186E-05	0.01333239

387.399996	715.260054	1.61424E-05	0.006253575
387.439996	381.312598	8.60569E-06	0.00333419
387.479996	302.955655	6.83729E-06	0.002649312
387.519996	349.4246	7.88603E-06	0.003055993
387.559996	460.189971	1.03858E-05	0.004025139
387.599996	545.049702	1.2301E-05	0.004767872
387.639996	598.453112	1.35063E-05	0.005235564
387.679996	583.110802	1.316E-05	0.005101868
387.719996	531.675337	1.19992E-05	0.004652319
387.759996	504.564353	1.13873E-05	0.004415545
387.799996	521.551991	1.17707E-05	0.004564678
387.839996	625.404732	1.41145E-05	0.005474173
387.879996	761.898531	1.7195E-05	0.006669592
387.919996	903.266679	2.03855E-05	0.007907932
387.959996	1154.706498	2.60601E-05	0.010110283
387.999996	1728.685396	3.9014E-05	0.01513744
388.039996	2715.761872	6.1291E-05	0.023783345
388.079996	3935.823639	8.88261E-05	0.034471616
388.119996	4996.432996	0.000112763	0.043765394
388.159996	5506.756052	0.00012428	0.048240452
388.199996	5241.450381	0.000118292	0.045921045
388.239996	4287.086811	9.67536E-05	0.037563609
388.279996	3064.003529	6.91503E-05	0.026849675
388.319996	1965.892984	4.43675E-05	0.017228774
388.359996	1164.049462	2.6271E-05	0.010202596
388.399996	639.719865	1.44376E-05	0.005607558
388.439996	392.977138	8.86895E-06	0.003445054
388.479996	328.449506	7.41265E-06	0.002879665
388.519996	387.115538	8.73666E-06	0.003394367
388.559996	461.509388	1.04156E-05	0.004047095
388.599996	507.296322	1.1449E-05	0.00444907
388.639996	534.395752	1.20606E-05	0.004687219
388.679996	503.287724	1.13585E-05	0.004414823
388.719996	425.023073	9.59218E-06	0.003728672
388.759996	346.779194	7.82632E-06	0.003042561
388.799996	320.553333	7.23444E-06	0.002812751
388.839996	374.084462	8.44257E-06	0.003282807
388.879996	523.510605	1.18149E-05	0.00459458
388.919996	817.758449	1.84557E-05	0.007177779
388.959996	1469.581268	3.31664E-05	0.012900404
388.999996	2653.163564	5.98782E-05	0.023292622
389.039996	4470.919099	0.000100902	0.039255076
389.079996	6681.482856	0.000150792	0.058670058
389.119996	8455.464427	0.000190828	0.074255012
389.159996	9286.919423	0.000209593	0.081565147
389.199996	8881.193063	0.000200436	0.078009751

389.239996	7357.693531	0.000166053	0.064634428
389.279996	5323.05656	0.000120134	0.046765752
389.319996	3307.748414	7.46513E-05	0.029063234
389.359996	1798.753648	4.05954E-05	0.015806211
389.399996	900.142132	2.0315E-05	0.007910643
389.439996	463.793916	1.04672E-05	0.00407634
389.479996	340.754475	7.69035E-06	0.002995239
389.519996	344.774388	7.78108E-06	0.003030885
389.559996	441.729608	9.96922E-06	0.00388361
389.599996	529.139646	1.19419E-05	0.004652582
389.639996	531.516155	1.19956E-05	0.004673957
389.679996	500.320978	1.12915E-05	0.00440009
389.719996	425.498144	9.6029E-06	0.003742442
389.759996	338.490256	7.63925E-06	0.002977475
389.799996	309.737097	6.99033E-06	0.002724832
389.839996	347.398619	7.8403E-06	0.003056464
389.879996	409.708326	9.24655E-06	0.003605043
389.919996	526.419415	1.18806E-05	0.004632465
389.959996	804.335554	1.81527E-05	0.00707884
389.999996	1360.588681	3.07066E-05	0.011975571
390.039996	2257.527221	5.09492E-05	0.019872243
390.079996	3417.170408	7.71208E-05	0.030083272
390.119996	4498.752294	0.000101531	0.039609107
390.159996	5045.112079	0.000113861	0.044424067
390.199996	4899.593461	0.000110577	0.043147145
390.239996	4055.731267	9.15322E-05	0.035719528
390.279996	2903.848165	6.55358E-05	0.025577315
390.319996	1866.831649	4.21318E-05	0.01644488
390.359996	1075.695268	2.42769E-05	0.009476748
390.399996	583.069711	1.31591E-05	0.005137301
390.439996	349.951989	7.89793E-06	0.003083667
390.479996	298.740327	6.74215E-06	0.002632676
390.519996	317.325927	7.1616E-06	0.00279675
390.559996	379.81992	8.57201E-06	0.003347883
390.599996	497.068589	1.12181E-05	0.004381808
390.639996	561.374217	1.26694E-05	0.004949188
390.679996	527.966384	1.19155E-05	0.004655134
390.719996	469.677739	1.06E-05	0.004141621
390.759996	433.661775	9.78714E-06	0.003824424
390.799996	416.028915	9.38919E-06	0.003669297
390.839996	467.914791	1.05602E-05	0.004127343
390.879996	646.879904	1.45992E-05	0.005706527
390.919996	975.343199	2.20121E-05	0.008604985
390.959996	1589.08822	3.58635E-05	0.014021197
390.999996	2767.865966	6.24669E-05	0.02442455
391.039996	4632.589154	0.000104551	0.040883656

391.079996	7173.909474	0.000161905	0.063317863
391.119996	10158.0113	0.000229252	0.089665106
391.159996	13037.91682	0.000294248	0.115097899
391.199996	15054.91057	0.000339768	0.132917382
391.239996	15403.73893	0.000347641	0.136011037
391.279996	13721.55308	0.000309676	0.121170157
391.319996	10478.1231	0.000236477	0.092538039
391.359996	6790.130657	0.000153244	0.059973489
391.399996	3726.166734	8.40944E-05	0.032914545
391.439996	1784.097225	4.02646E-05	0.015761171
391.479996	850.962784	1.9205E-05	0.007518391
391.519996	515.736899	1.16395E-05	0.004557083
391.559996	469.84074	1.06037E-05	0.004151966
391.599996	533.748473	1.2046E-05	0.004717198
391.639996	566.668801	1.27889E-05	0.005008655
391.679996	516.426626	1.1655E-05	0.004565042
391.719996	398.938333	9.00348E-06	0.003526844
391.759996	325.693857	7.35046E-06	0.002879615
391.799996	326.64261	7.37187E-06	0.002888298
391.839996	341.926199	7.7168E-06	0.00302375
391.879996	401.273651	9.05619E-06	0.003548939
391.919996	547.144903	1.23483E-05	0.004839545
391.959996	863.297469	1.94834E-05	0.007636722
391.999996	1455.564971	3.28501E-05	0.012877229
392.039996	2356.718561	5.31879E-05	0.020851767
392.079996	3553.972274	8.02082E-05	0.031448032
392.119996	4868.087195	0.000109866	0.043080635
392.159996	5950.579407	0.000134296	0.052665632
392.199996	6423.180773	0.000144962	0.056854191
392.239996	6249.124817	0.000141034	0.055319192
392.279996	5337.610416	0.000120462	0.047255003
392.319996	3883.99774	8.76564E-05	0.034389366
392.359996	2438.540785	5.50345E-05	0.021593324
392.399996	1318.287564	2.97519E-05	0.011674651
392.439996	670.839539	1.51399E-05	0.005941508
392.479996	429.391573	9.69077E-06	0.003803433
392.519996	407.654155	9.20019E-06	0.003611257
392.559996	462.349166	1.04346E-05	0.004096197
392.599996	549.446771	1.24002E-05	0.004868337
392.639996	601.062675	1.35651E-05	0.005326219
392.679996	540.222542	1.21921E-05	0.004787582
392.719996	452.243744	1.02065E-05	0.004008301
392.759996	400.203818	9.03204E-06	0.003547425
392.799996	372.979769	8.41763E-06	0.003306447
392.839996	388.706114	8.77256E-06	0.003446211
392.879996	474.756176	1.07146E-05	0.004209546

392.919996	706.756798	1.59505E-05	0.006267277
392.959996	1259.938084	2.8435E-05	0.011173836
392.999996	2307.487375	5.20768E-05	0.020466173
393.039996	3834.94727	8.65494E-05	0.034017383
393.079996	5703.264937	0.000128715	0.050595192
393.119996	7442.522092	0.000167967	0.066031327
393.159996	8636.288418	0.000194909	0.076630422
393.199996	8828.269586	0.000199242	0.078341854
393.239996	7918.823516	0.000178717	0.0702786
393.279996	6287.219756	0.000141894	0.05580399
393.319996	4351.098903	9.81982E-05	0.038623332
393.359996	2621.238796	5.91577E-05	0.023270276
393.399996	1390.901339	3.13907E-05	0.012349104
393.439996	704.144827	1.58916E-05	0.006252378
393.479996	413.101094	9.32312E-06	0.00366846
393.519996	387.253823	8.73978E-06	0.003439278
393.559996	478.693897	1.08035E-05	0.004251808
393.599996	566.064686	1.27753E-05	0.005028355
393.639996	622.618063	1.40516E-05	0.005531281
393.679996	559.629672	1.26301E-05	0.004972203
393.719996	443.113887	1.00005E-05	0.003937382
393.759996	350.680277	7.91437E-06	0.00311636
393.799996	302.656112	6.83053E-06	0.002689861
393.839996	299.963371	6.76976E-06	0.0026662
393.879996	349.663858	7.89143E-06	0.003108275
393.919996	462.759306	1.04438E-05	0.004114035
393.959996	735.509996	1.65994E-05	0.006539514
393.999996	1276.881922	2.88174E-05	0.011354074
394.039996	2107.336922	4.75597E-05	0.018740408
394.079996	3184.328438	7.18659E-05	0.028320897
394.119996	4187.496912	9.4506E-05	0.037246694
394.159996	4772.005301	0.000107698	0.042450051
394.199996	4739.408845	0.000106962	0.042164363
394.239996	4168.592714	9.40793E-05	0.037089835
394.279996	3220.693075	7.26866E-05	0.028658855
394.319996	2157.458634	4.86908E-05	0.01919977
394.359996	1281.128619	2.89133E-05	0.011402244
394.399996	710.650649	1.60384E-05	0.006325543
394.439996	422.955506	9.54552E-06	0.003765134
394.479996	328.329282	7.40993E-06	0.002923071
394.519996	351.527482	7.93349E-06	0.003129919
394.559996	434.888441	9.81483E-06	0.003872538
394.599996	540.414215	1.21964E-05	0.004812698
394.639996	568.357833	1.2827E-05	0.005062065
394.679996	496.318328	1.12012E-05	0.004420895
394.719996	389.169974	8.78302E-06	0.003466835

394.759996	317.392293	7.1631E-06	0.002827706
394.799996	305.186284	6.88763E-06	0.002719236
394.839996	328.194763	7.4069E-06	0.00292454
394.879996	426.807966	9.63246E-06	0.003803666
394.919996	707.918752	1.59767E-05	0.006309534
394.959996	1316.538598	2.97124E-05	0.011735226
394.999996	2567.423163	5.79432E-05	0.02288755
395.039996	4555.308498	0.000102807	0.040612864
395.079996	6658.598029	0.000150275	0.059370758
395.119996	8150.74803	0.000183951	0.07268273
395.159996	8596.169715	0.000194004	0.076662453
395.199996	7883.537715	0.00017792	0.070314167
395.239996	6401.091607	0.000144464	0.057097841
395.279996	4680.930509	0.000105642	0.041758202
395.319996	2985.290273	6.73738E-05	0.026634227
395.359996	1705.917119	3.85002E-05	0.015221428
395.399996	906.317119	2.04543E-05	0.008087636
395.439996	499.106834	1.12641E-05	0.004454294
395.479996	359.386495	8.11085E-06	0.00320768
395.519996	359.286271	8.10859E-06	0.00320711
395.559996	421.0697	9.50296E-06	0.003758989
395.599996	502.040465	1.13304E-05	0.004482288
395.639996	541.089193	1.22116E-05	0.004831409
395.679996	513.274904	1.15839E-05	0.004583517
395.719996	442.825931	9.99396E-06	0.003954812
395.759996	361.553887	8.15977E-06	0.00322931
395.799996	300.263792	6.77654E-06	0.002682153
395.839996	290.883908	6.56484E-06	0.002598628
395.879996	345.160809	7.7898E-06	0.003083825
395.919996	477.884275	1.07852E-05	0.004270069
395.959996	803.409304	1.81318E-05	0.007179479
395.999996	1446.330484	3.26417E-05	0.012926099
396.039996	2373.960135	5.3577E-05	0.021218624
396.079996	3432.086698	7.74574E-05	0.030679333
396.119996	4308.515488	9.72372E-05	0.038517597
396.159996	4644.862141	0.000104828	0.041528687
396.199996	4387.426773	9.90181E-05	0.039230975
396.239996	3664.313581	8.26985E-05	0.032768435
396.279996	2688.112089	6.06669E-05	0.024041097
396.319996	1779.922886	4.01704E-05	0.015920325
396.359996	1085.587712	2.45002E-05	0.0097109
396.399996	636.873711	1.43734E-05	0.005697597
396.439996	419.163136	9.45993E-06	0.003750294
396.479996	329.594598	7.43849E-06	0.002949213
396.519996	344.847642	7.78273E-06	0.003086008
396.559996	430.0459	9.70554E-06	0.003848828

396.599996	498.191281	1.12435E-05	0.004459165
396.639996	509.052048	1.14886E-05	0.004556837
396.679996	468.208209	1.05668E-05	0.004191641
396.719996	387.679455	8.74939E-06	0.003471056
396.759996	329.887885	7.44511E-06	0.002953922
396.799996	290.240214	6.55032E-06	0.002599166
396.839996	310.763625	7.0135E-06	0.002783238
396.879996	414.413304	9.35273E-06	0.003711912
396.919996	629.187956	1.41999E-05	0.005636223
396.959996	1147.930479	2.59072E-05	0.010284119
396.999996	2135.015547	4.81843E-05	0.019129178
397.039996	3531.056157	7.9691E-05	0.031640522
397.079996	5078.14297	0.000114607	0.045507994
397.119996	6354.067593	0.000143402	0.056947982
397.159996	6966.834272	0.000157232	0.062446159
397.199996	6603.843032	0.00014904	0.059198504
397.239996	5359.698042	0.000120961	0.048050515
397.279996	3853.001847	8.69569E-05	0.034546231
397.319996	2480.883125	5.59901E-05	0.022245977
397.359996	1447.932025	3.26778E-05	0.012984854
397.399996	804.641784	1.81596E-05	0.007216643
397.439996	452.807463	1.02192E-05	0.004061532
397.479996	334.652023	7.55263E-06	0.003002019
397.519996	355.957957	8.03348E-06	0.003193467
397.559996	437.34849	9.87035E-06	0.003924055
397.599996	513.911033	1.15983E-05	0.004611467
397.639996	571.957032	1.29083E-05	0.005132846
397.679996	522.871887	1.18005E-05	0.004692819
397.719996	392.483892	8.85781E-06	0.00352293
397.759996	295.419632	6.66721E-06	0.002651949
397.799996	263.595392	5.94898E-06	0.002366505
397.839996	264.163604	5.9618E-06	0.002371844
397.879996	316.700694	7.14749E-06	0.002843845
397.919996	458.979795	1.03585E-05	0.004121868
397.959996	761.398263	1.71837E-05	0.006838424
397.999996	1342.864442	3.03066E-05	0.012062019
398.039996	2310.177103	5.21375E-05	0.020752802
398.079996	3649.30103	8.23596E-05	0.032785726
398.119996	4841.372548	0.000109263	0.043499799
398.159996	5556.290018	0.000125398	0.049928358
398.199996	5670.883256	0.000127984	0.050963203
398.239996	5135.101369	0.000115892	0.046152864
398.279996	4154.485484	9.3761E-05	0.037343111
398.319996	3004.049445	6.77972E-05	0.027004985
398.359996	1895.364018	4.27757E-05	0.017040138
398.399996	1047.439753	2.36393E-05	0.00941788

398.439996	547.051298	1.23462E-05	0.004919214
398.479996	344.785404	7.78133E-06	0.003100703
398.519996	348.729225	7.87033E-06	0.003136485
398.559996	444.472617	1.00311E-05	0.003998006
398.599996	497.179664	1.12207E-05	0.004472552
398.639996	510.041478	1.15109E-05	0.004588715
398.679996	482.271041	1.08842E-05	0.004339307
398.719996	399.156087	9.0084E-06	0.003591828
398.759996	345.274118	7.79236E-06	0.00310728
398.799996	351.889791	7.94166E-06	0.003167135
398.839996	389.682755	8.7946E-06	0.003507637
398.879996	490.507777	1.10701E-05	0.004415632
398.919996	711.463983	1.60568E-05	0.006405359
398.959996	1264.261357	2.85326E-05	0.011383373
398.999996	2427.037284	5.47749E-05	0.021855165
399.039996	4066.006323	9.17641E-05	0.036617546
399.079996	5712.911478	0.000128932	0.051454363
399.119996	6999.044324	0.000157959	0.063044468
399.159996	7641.652637	0.000172461	0.068839714
399.199996	7268.156066	0.000164032	0.065481637
399.239996	6112.816646	0.000137958	0.055078254
399.279996	4591.498542	0.000103624	0.041374881
399.319996	3030.89505	6.84031E-05	0.027314717
399.359996	1786.241421	4.0313E-05	0.016099392
399.399996	953.508956	2.15194E-05	0.008594836
399.439996	503.943255	1.13733E-05	0.00454295
399.479996	322.311583	7.27412E-06	0.002905867
399.519996	325.232807	7.34005E-06	0.002932497
399.559996	401.937349	9.07117E-06	0.003624475
399.599996	474.730649	1.0714E-05	0.004281318
399.639996	526.204608	1.18757E-05	0.004746007
399.679996	496.951248	1.12155E-05	0.00448261
399.719996	434.867325	9.81435E-06	0.003922992
399.759996	358.267773	8.0856E-06	0.003232301
399.799996	283.46326	6.39737E-06	0.002557669
399.839996	274.652967	6.19853E-06	0.002478422
399.879996	329.262443	7.43099E-06	0.002971506
399.919996	463.485343	1.04602E-05	0.004183251
399.959996	752.725788	1.6988E-05	0.006794509
399.999996	1331.396129	3.00478E-05	0.012019102
400.039996	2292.42841	5.17369E-05	0.020696836
400.079996	3516.108261	7.93537E-05	0.031747814
400.119996	4481.815551	0.000101148	0.040471471
400.159996	4807.417533	0.000108497	0.043416046
400.199996	4449.715869	0.000100424	0.04018964
400.239996	3593.882427	8.11089E-05	0.032463034

400.279996	2551.102743	5.75748E-05	0.023046055
400.319996	1630.454075	3.67971E-05	0.014730606
400.359996	974.593211	2.19952E-05	0.008806003
400.399996	575.925165	1.29978E-05	0.005204331
400.439996	358.922554	8.10038E-06	0.003243717
400.479996	270.202185	6.09809E-06	0.002442162
400.519996	309.172451	6.97759E-06	0.002794665
400.559996	422.736502	9.54057E-06	0.003821572
400.599996	533.981023	1.20512E-05	0.004827714
400.639996	599.915633	1.35393E-05	0.005424369
400.679996	575.067588	1.29785E-05	0.005200215
400.719996	471.384085	1.06385E-05	0.004263053
400.759996	361.448826	8.1574E-06	0.003269158
400.799996	304.410322	6.87012E-06	0.002753543
400.839996	353.722595	7.98303E-06	0.003199916
400.879996	510.946583	1.15314E-05	0.004622689
400.919996	846.627042	1.91072E-05	0.007660456
400.959996	1596.788625	3.60373E-05	0.014449514
400.999996	3043.308018	6.86832E-05	0.027541972
401.039996	5322.285871	0.000120117	0.048171552
401.079996	8075.135978	0.000182245	0.073094652
401.119996	10466.2776	0.000236209	0.094748276
401.159996	11549.74542	0.000260662	0.104567033
401.199996	11000.11023	0.000248257	0.099600774
401.239996	8960.177473	0.000202219	0.08113824
401.279996	6261.716568	0.000141318	0.056708175
401.319996	3804.975314	8.5873E-05	0.034462549
401.359995	2060.991805	4.65137E-05	0.018668744
401.399995	1029.188917	2.32274E-05	0.009323462
401.439995	541.850391	1.22288E-05	0.004909133
401.479995	391.817711	8.84278E-06	0.003550199
401.519995	384.528509	8.67827E-06	0.0034845
401.559995	467.675465	1.05548E-05	0.004238379
401.599995	556.849933	1.25673E-05	0.005047039
401.639995	578.95398	1.30662E-05	0.005247902
401.679995	501.351445	1.13148E-05	0.00454493
401.719995	397.718888	8.97596E-06	0.003605823
401.759995	313.134393	7.06701E-06	0.002839241
401.799995	287.84652	6.4963E-06	0.002610211
401.839995	303.793234	6.85619E-06	0.002755091
401.879995	339.907017	7.67123E-06	0.003082913
401.919995	460.602786	1.03952E-05	0.004178024
401.959995	771.295716	1.74071E-05	0.006996946
401.999995	1389.214739	3.13526E-05	0.012603762
402.039995	2418.927806	5.45918E-05	0.0219481
402.079995	3749.161242	8.46133E-05	0.034021334

402.119995	4988.865631	0.000112592	0.045275393
402.159995	5559.55012	0.000125471	0.050459538
402.199995	5262.443675	0.000118766	0.047767694
402.239995	4268.227565	9.6328E-05	0.038746955
402.279995	3069.054204	6.92643E-05	0.027863634
402.319995	1978.461468	4.46511E-05	0.017964038
402.359995	1150.791448	2.59718E-05	0.010449997
402.399995	626.136219	1.4131E-05	0.005686323
402.439995	357.744125	8.07379E-06	0.003249215
402.479995	295.446146	6.66781E-06	0.002683659
402.519995	346.826465	7.82739E-06	0.003150681
402.559995	439.329413	9.91505E-06	0.003991404
402.599995	522.365098	1.17891E-05	0.004746273
402.639995	523.445577	1.18134E-05	0.004756562
402.679995	494.310186	1.11559E-05	0.004492255
402.719995	440.128629	9.93309E-06	0.004000254
402.759995	380.898077	8.59634E-06	0.003462261
402.799995	345.389611	7.79496E-06	0.003139811
402.839995	369.800193	8.34588E-06	0.003362052
402.879995	496.135501	1.11971E-05	0.004511082
402.919995	751.853594	1.69683E-05	0.006836863
402.959995	1287.669243	2.90609E-05	0.011710381
402.999995	2388.504177	5.39052E-05	0.0217238
403.039995	4130.726225	9.32247E-05	0.037573298
403.079995	6203.415337	0.000140002	0.056432184
403.119995	8103.187959	0.000182878	0.073721644
403.159995	9096.014877	0.000205284	0.082762452
403.199995	8741.528191	0.000197284	0.079544954
403.239995	7366.067487	0.000166242	0.067035376
403.279995	5441.119461	0.000122798	0.049522173
403.319995	3434.971038	7.75225E-05	0.031266379
403.359995	1907.171337	4.30422E-05	0.017361502
403.399995	975.316722	2.20115E-05	0.008879455
403.439995	508.196922	1.14693E-05	0.004627173
403.479995	339.414146	7.6601E-06	0.003090699
403.519995	333.264561	7.52132E-06	0.003035002
403.559995	381.70369	8.61452E-06	0.003476476
403.599995	455.987443	1.0291E-05	0.004153448
403.639995	487.070849	1.09925E-05	0.004437017
403.679995	469.5219	1.05965E-05	0.004277577
403.719995	446.072179	1.00672E-05	0.004064341
403.759995	419.543439	9.46851E-06	0.003823006
403.799995	398.957518	9.00392E-06	0.003635781
403.839995	396.981563	8.95932E-06	0.003618132
403.879995	431.02066	9.72754E-06	0.003928757
403.919995	575.345392	1.29847E-05	0.005244798

403.959995	853.088643	1.9253E-05	0.007777451
403.999995	1402.828781	3.16599E-05	0.012790596
404.039995	2358.016903	5.32172E-05	0.02150186
404.079995	3509.376665	7.92017E-05	0.03200384
404.119995	4553.930818	0.000102776	0.041533786
404.159995	5098.541399	0.000115067	0.046505469
404.199995	4863.388339	0.00010976	0.044364951
404.239995	3999.372551	9.02603E-05	0.03648681
404.279995	2860.579257	6.45593E-05	0.026100029
404.319995	1844.312478	4.16236E-05	0.016829239
404.359995	1078.926831	2.43499E-05	0.009846116
404.399995	580.614971	1.31037E-05	0.005299125
404.439995	333.710252	7.53138E-06	0.003045989
404.479995	271.001759	6.11613E-06	0.002473853
404.519995	314.847036	7.10566E-06	0.002874381
404.559995	370.524869	8.36223E-06	0.003383024
404.599995	420.734206	9.49538E-06	0.003841833
404.639995	478.008892	1.0788E-05	0.004365254
404.679995	494.874802	1.11686E-05	0.004519723
404.719995	428.710971	9.67541E-06	0.003915832
404.759995	330.362714	7.45583E-06	0.00301782
404.799995	297.689069	6.71843E-06	0.002719619
404.839995	334.676053	7.55317E-06	0.003057826
404.879995	447.889827	1.01082E-05	0.004092628
404.919995	714.063855	1.61154E-05	0.006525458
404.959995	1262.082933	2.84835E-05	0.011534659
404.999995	2227.17933	5.02643E-05	0.020357056
405.039995	3639.813913	8.21455E-05	0.033272225
405.079995	5302.626036	0.000119673	0.048477091
405.119995	6746.279075	0.000152254	0.061681189
405.159995	7387.742041	0.000166731	0.06755275
405.199995	7007.02599	0.000158139	0.064077848
405.239995	5791.455216	0.000130705	0.052966925
405.279995	4135.711387	9.33372E-05	0.037827718
405.319995	2586.412902	5.83717E-05	0.023659232
405.359995	1435.565858	3.23987E-05	0.013133146
405.399995	745.828333	1.68323E-05	0.006823817
405.439995	436.820436	9.85843E-06	0.003997001
405.479995	334.047723	7.53899E-06	0.00305691
405.519995	342.272096	7.7246E-06	0.003132482
405.559995	452.80966	1.02193E-05	0.004144532
405.599995	592.198354	1.33651E-05	0.005420881
405.639995	629.351836	1.42036E-05	0.005761546
405.679995	576.362827	1.30077E-05	0.005276966
405.719995	483.678099	1.09159E-05	0.004428816
405.759995	398.111356	8.98482E-06	0.00364568

405.799995	357.409859	8.06624E-06	0.003273281
405.839995	387.505311	8.74546E-06	0.003549255
405.879995	465.32782	1.05018E-05	0.004262471
405.919995	592.348137	1.33685E-05	0.00542653
405.959995	832.673636	1.87923E-05	0.007628916
405.999995	1304.899491	2.94498E-05	0.011956604
406.039995	2123.83735	4.79321E-05	0.01946233
406.079995	3134.035566	7.07308E-05	0.02872237
406.119995	4007.846881	9.04515E-05	0.036734172
406.159995	4424.452298	9.98537E-05	0.040556589
406.199995	4197.828804	9.47391E-05	0.038483042
406.239995	3469.525717	7.83024E-05	0.031809551
406.279995	2566.876737	5.79308E-05	0.023536138
406.319995	1656.45293	3.73838E-05	0.0151898
406.359995	942.995495	2.12821E-05	0.008648192
406.399995	511.990598	1.15549E-05	0.004695917
406.439995	311.34423	7.02661E-06	0.002855894
406.479995	293.933651	6.63367E-06	0.002696455
406.519995	383.821654	8.66232E-06	0.003521406
406.559995	479.265308	1.08164E-05	0.004397495
406.599995	552.005556	1.2458E-05	0.005065421
406.639995	595.68457	1.34438E-05	0.005466775
406.679995	561.809384	1.26793E-05	0.005156399
406.719995	469.146758	1.0588E-05	0.004306347
406.759995	385.483862	8.69983E-06	0.003538744
406.799995	365.950333	8.25899E-06	0.003359757
406.839995	393.835072	8.88831E-06	0.00361612
406.879995	478.672292	1.0803E-05	0.004395511
406.919995	732.295775	1.65269E-05	0.006725124
406.959995	1292.938932	2.91798E-05	0.011875024
406.999995	2385.62141	5.38402E-05	0.021912942
407.039995	4236.143287	9.56039E-05	0.038914592
407.079995	6729.999398	0.000151887	0.061830042
407.119995	9615.977117	0.000217019	0.08835286
407.159995	12151.66546	0.000274246	0.111662067
407.199995	13459.8311	0.00030377	0.123694996
407.239995	12837.66962	0.000289728	0.11798896
407.279995	10370.35411	0.000234044	0.095321621
407.319995	7110.431118	0.000160473	0.065363669
407.359995	4215.208193	9.51314E-05	0.038752718
407.399995	2179.637998	4.91914E-05	0.020040574
407.439995	1037.055615	2.34049E-05	0.009536093
407.479995	555.815099	1.2544E-05	0.005111418
407.519995	461.075884	1.04058E-05	0.004240588
407.559995	506.235182	1.1425E-05	0.004656382
407.599995	551.016814	1.24357E-05	0.005068784

407.639995	552.332752	1.24654E-05	0.005081388
407.679995	513.931586	1.15987E-05	0.004728566
407.719995	465.546265	1.05067E-05	0.004283804
407.759995	378.014974	8.53127E-06	0.003478711
407.799995	332.933249	7.51384E-06	0.003064144
407.839995	350.800936	7.91709E-06	0.003228905
407.879995	385.645686	8.70349E-06	0.003549978
407.919995	469.443281	1.05947E-05	0.004321782
407.959995	708.518786	1.59903E-05	0.006523395
407.999995	1241.687611	2.80232E-05	0.011433449
408.039995	2174.877109	4.90839E-05	0.020028213
408.079995	3387.268365	7.64459E-05	0.031196054
408.119995	4729.310573	0.000106734	0.04356026
408.159995	5761.543266	0.00013003	0.053073047
408.199995	6048.75469	0.000136512	0.055724185
408.239995	5561.134384	0.000125507	0.051237
408.279995	4406.643147	9.94518E-05	0.04060418
408.319995	3044.28111	6.87052E-05	0.028053701
408.359995	1826.641792	4.12248E-05	0.016834543
408.399995	977.384415	2.20582E-05	0.009008571
408.439995	533.219591	1.2034E-05	0.004915176
408.479995	372.717669	8.41172E-06	0.003436019
408.519995	378.796001	8.5489E-06	0.003492396
408.559995	496.395062	1.12029E-05	0.004577075
408.599995	609.114471	1.37469E-05	0.005616969
408.639995	607.483708	1.37101E-05	0.005602479
408.679995	542.485604	1.22431E-05	0.005003528
408.719995	429.3933	9.69081E-06	0.003960827
408.759995	348.416374	7.86327E-06	0.003214191
408.799995	333.254909	7.5211E-06	0.003074625
408.839995	357.775601	8.0745E-06	0.003301177
408.879995	439.958821	9.92926E-06	0.004059875
408.919995	673.675564	1.52039E-05	0.006217187
408.959995	1180.81759	2.66494E-05	0.010898542
408.999995	2172.37807	4.90275E-05	0.020052267
409.039995	3614.462913	8.15734E-05	0.03336678
409.079995	5260.004924	0.000118711	0.048562289
409.119995	6881.276933	0.000155301	0.063536677
409.159995	7929.32189	0.000178954	0.073220716
409.199995	8110.874336	0.000183051	0.074904524
409.239995	7208.388184	0.000162683	0.066576505
409.279995	5507.741323	0.000124302	0.050874342
409.319995	3652.900543	8.24409E-05	0.033744699
409.359995	2175.816346	4.91051E-05	0.020101682
409.399995	1221.406516	2.75654E-05	0.011285293
409.439995	672.774624	1.51836E-05	0.006216767

409.479995	426.898905	9.63451E-06	0.003945141
409.519995	403.478271	9.10594E-06	0.003729066
409.559995	446.655261	1.00804E-05	0.004128523
409.599995	515.737267	1.16395E-05	0.004767528
409.639995	577.274551	1.30283E-05	0.005336906
409.679995	563.368998	1.27145E-05	0.005208857
409.719995	499.46934	1.12723E-05	0.004618498
409.759995	409.224466	9.23563E-06	0.00378439
409.799995	334.997183	7.56042E-06	0.00309826
409.839995	306.439208	6.91591E-06	0.002834415
409.879995	333.619076	7.52932E-06	0.003086117
409.919995	444.802415	1.00386E-05	0.004115011
409.959995	694.947826	1.5684E-05	0.006429814
409.999995	1183.791514	2.67165E-05	0.010953775
410.039995	2021.428072	4.56208E-05	0.018706359
410.079995	3053.430666	6.89117E-05	0.0282593
410.119995	3968.380698	8.95608E-05	0.036730685
410.159995	4501.071841	0.000101583	0.041665251
410.199995	4443.939693	0.000100294	0.041140405
410.239995	3863.092302	8.71846E-05	0.035766615
410.279995	2968.40793	6.69928E-05	0.027485819
410.319995	2022.056248	4.5635E-05	0.01872495
410.359995	1251.608007	2.8247E-05	0.011591459
410.399995	755.294293	1.70459E-05	0.006995654
410.439995	517.445714	1.1678E-05	0.00479313
410.479995	429.615743	9.69583E-06	0.003979944
410.519995	415.916272	9.38665E-06	0.003853408
410.559995	439.756097	9.92468E-06	0.004074678
410.599995	465.591331	1.05077E-05	0.004314481
410.639995	515.094892	1.1625E-05	0.004773679
410.679995	548.865583	1.23871E-05	0.005087147
410.719995	506.234463	1.1425E-05	0.004692479
410.759995	417.113619	9.41367E-06	0.00386676
410.799995	360.264693	8.13067E-06	0.00334008
410.839995	351.788858	7.93938E-06	0.003261817
410.879995	418.900736	9.45401E-06	0.003884462
410.919995	626.813323	1.41463E-05	0.005812999
410.959995	1086.022196	2.451E-05	0.010072633
410.999995	1961.244245	4.42626E-05	0.018191908
411.039995	3237.6374	7.3069E-05	0.030034268
411.079995	4809.299762	0.000108539	0.044618292
411.119995	6300.568118	0.000142195	0.058459227
411.159995	7098.522974	0.000160204	0.065869384
411.199995	7078.880262	0.00015976	0.065693503
411.239995	6277.092659	0.000141665	0.058258412
411.279995	4822.928911	0.000108847	0.044766506

411.319995	3215.108335	7.25605E-05	0.029845591
411.359995	1891.43863	4.26871E-05	0.01755978
411.399995	1025.731575	2.31493E-05	0.009523635
411.439995	577.984872	1.30443E-05	0.005366952
411.479995	391.822786	8.84289E-06	0.003638674
411.519995	355.936791	8.033E-06	0.003305739
411.559995	418.566776	9.44647E-06	0.003887789
411.599995	506.250961	1.14254E-05	0.004702686
411.639995	527.323313	1.1901E-05	0.004898908
411.679995	492.592654	1.11171E-05	0.0045767
411.719995	429.993516	9.70435E-06	0.003995477
411.759995	362.815717	8.18825E-06	0.003371592
411.799995	350.617398	7.91295E-06	0.003258551
411.839995	371.9255	8.39384E-06	0.003456919
411.879995	417.031935	9.41183E-06	0.003876544
411.919995	499.928467	1.12827E-05	0.004647565
411.959995	711.947961	1.60677E-05	0.006619239
411.999995	1221.120411	2.7559E-05	0.011354302
412.039995	2010.18976	4.53672E-05	0.018693094
412.079995	2914.59485	6.57783E-05	0.027105941
412.119995	3728.149647	8.41391E-05	0.034675424
412.159995	4221.797777	9.52801E-05	0.039270644
412.199995	4279.631425	9.65853E-05	0.039812469
412.239995	3770.648163	8.50983E-05	0.035080913
412.279995	3001.518499	6.77401E-05	0.027927885
412.319995	2176.660868	4.91242E-05	0.020254892
412.359995	1425.946644	3.21816E-05	0.013270417
412.399995	869.455002	1.96224E-05	0.008092273
412.439995	525.001431	1.18486E-05	0.004886816
412.479995	374.63489	8.45499E-06	0.003487513
412.519995	355.531469	8.02385E-06	0.003309999
412.559995	415.517095	9.37764E-06	0.00386884
412.599995	508.421025	1.14744E-05	0.004734319
412.639995	557.099804	1.2573E-05	0.005188109
412.679995	514.037272	1.16011E-05	0.004787544
412.719995	434.980323	9.8169E-06	0.004051631
412.759995	344.348462	7.77147E-06	0.00320775
412.799995	283.484607	6.39785E-06	0.002641034
412.839995	306.832392	6.92478E-06	0.002858826
412.879995	381.133835	8.60166E-06	0.003551453
412.919995	590.022217	1.3316E-05	0.005498434
412.959995	1058.206679	2.38823E-05	0.009862414
412.999995	1937.33127	4.37229E-05	0.018057544
413.039995	3293.996846	7.43409E-05	0.030705773
413.079995	4744.130995	0.000107068	0.044227826
413.119995	5892.632376	0.000132989	0.054940211

413.159995	6466.533158	0.000145941	0.060296837
413.199995	6240.357936	0.000140836	0.058193512
413.239995	5401.51755	0.000121905	0.050375909
413.279995	4256.334682	9.60595E-05	0.039699488
413.319995	3042.856007	6.8673E-05	0.028383933
413.359995	1960.915043	4.42551E-05	0.018293297
413.399995	1138.434532	2.56929E-05	0.010621437
413.439995	623.320495	1.40675E-05	0.005816057
413.479995	395.022065	8.9151E-06	0.003686215
413.519995	355.467756	8.02241E-06	0.003317428
413.559995	414.330824	9.35087E-06	0.003867146
413.599995	482.235965	1.08834E-05	0.004501372
413.639995	521.005386	1.17584E-05	0.00486373
413.679995	487.290313	1.09975E-05	0.004549431
413.719995	414.350901	9.35132E-06	0.003868829
413.759995	338.644148	7.64273E-06	0.003162255
413.799995	279.072909	6.29829E-06	0.002606231
413.839995	268.384833	6.05707E-06	0.002506659
413.879995	318.318438	7.184E-06	0.002973315
413.919995	428.441129	9.66932E-06	0.004002325
413.959995	705.931039	1.59319E-05	0.006595161
413.999995	1320.286101	2.9797E-05	0.012335965
414.039995	2362.635413	5.33214E-05	0.022077188
414.079995	3628.167196	8.18827E-05	0.03390598
414.119995	4758.937924	0.000107403	0.044477564
414.159995	5539.407121	0.000125017	0.051776917
414.199995	5721.824849	0.000129134	0.053487144
414.239995	5212.555691	0.00011764	0.048731243
414.279995	4180.511495	9.43483E-05	0.039086622
414.319995	3015.397268	6.80533E-05	0.02819585
414.359995	1957.057638	4.41681E-05	0.018301479
414.399995	1146.683455	2.5879E-05	0.010724278
414.439995	652.665827	1.47298E-05	0.006104601
414.479995	437.072377	9.86411E-06	0.004088478
414.519995	404.566612	9.1305E-06	0.003784777
414.559995	460.97044	1.04035E-05	0.004312859
414.599995	541.649873	1.22243E-05	0.005068188
414.639995	569.621575	1.28556E-05	0.005330432
414.679995	542.683732	1.22476E-05	0.005078841
414.719995	488.115113	1.10161E-05	0.004568588
414.759995	406.58962	9.17616E-06	0.003805905
414.799995	346.253178	7.81445E-06	0.003241435
414.839995	355.929037	8.03282E-06	0.003332336
414.879995	441.365477	9.961E-06	0.004132621
414.919995	651.886886	1.47122E-05	0.006104377
414.959995	1146.615636	2.58775E-05	0.010738135

414.999995	2040.330924	4.60474E-05	0.019109682
415.039995	3421.991443	7.72296E-05	0.032053365
415.079995	5080.078281	0.00011465	0.047589042
415.119995	6437.223176	0.000145279	0.060308284
415.159995	7161.2974	0.000161621	0.067098369
415.199995	7060.630906	0.000159349	0.06616154
415.239995	6159.306966	0.000139007	0.057721257
415.279995	4801.230224	0.000108357	0.044998527
415.319995	3330.234023	7.51587E-05	0.031214929
415.359995	2000.336426	4.51448E-05	0.018751347
415.399995	1067.539961	2.40929E-05	0.010008186
415.439995	563.804421	1.27243E-05	0.005286175
415.479995	341.54938	7.70829E-06	0.003202642
415.519995	345.80934	7.80444E-06	0.003242899
415.559995	436.355974	9.84795E-06	0.004092413
415.599995	518.808562	1.17088E-05	0.004866172
415.639995	569.030695	1.28422E-05	0.005337744
415.679995	541.75913	1.22267E-05	0.005082415
415.719995	437.312191	9.86953E-06	0.00410296
415.759995	314.136632	7.08963E-06	0.002947583
415.799995	245.825245	5.54793E-06	0.002306831
415.839995	250.350767	5.65007E-06	0.002349524
415.879995	306.744263	6.92279E-06	0.00287905
415.919995	433.607075	9.78591E-06	0.004070155
415.959995	719.14727	1.62302E-05	0.006751094
415.999995	1321.250498	2.98188E-05	0.012404614
416.039995	2263.113602	5.10753E-05	0.021249377
416.079995	3429.475467	7.73985E-05	0.032203961
416.119995	4448.266282	0.000100391	0.041774775
416.159995	5035.07371	0.000113635	0.047290175
416.199995	5047.004236	0.000113904	0.047406784
416.239995	4508.303234	0.000101746	0.042350806
416.279995	3563.415953	8.04213E-05	0.033477793
416.319995	2523.754787	5.69576E-05	0.0237126
416.359995	1611.638616	3.63724E-05	0.015144028
416.399995	950.762102	2.14574E-05	0.008934851
416.439995	541.25214	1.22153E-05	0.005086942
416.479995	364.110612	8.21747E-06	0.003422412
416.519995	372.376126	8.40401E-06	0.003500438
416.559995	443.402545	1.0007E-05	0.004168507
416.599995	511.053357	1.15338E-05	0.004804965
416.639995	553.365691	1.24887E-05	0.005203289
416.679995	530.144617	1.19646E-05	0.00498542
416.719995	485.646755	1.09604E-05	0.004567406
416.759995	428.35631	9.66741E-06	0.004028988
416.799995	380.371419	8.58445E-06	0.003578

416.839995	394.381912	8.90065E-06	0.003710147
416.879995	508.814253	1.14832E-05	0.004787128
416.919995	758.341481	1.71147E-05	0.007135465
416.959995	1326.08921	2.9928E-05	0.012478773
416.999995	2482.535635	5.60274E-05	0.023363414
417.039995	4326.244295	9.76373E-05	0.040718662
417.079995	6515.590486	0.000147048	0.061330696
417.119995	8398.008765	0.000189531	0.07905732
417.159995	9412.22588	0.000212421	0.088613472
417.199995	9133.858971	0.000206138	0.086000971
417.239995	7767.846994	0.000175309	0.073146129
417.279995	5795.713129	0.000130801	0.054580713
417.319995	3720.967487	8.39771E-05	0.035045303
417.359995	2068.840518	4.66908E-05	0.019486892
417.399995	1046.886918	2.36268E-05	0.009861818
417.439995	542.669259	1.22473E-05	0.005112508
417.479995	353.39032	7.97553E-06	0.003329623
417.519995	349.858976	7.89583E-06	0.003296667
417.559995	412.771097	9.31567E-06	0.003889851
417.599995	497.496797	1.12278E-05	0.004688733
417.639995	522.984808	1.1803E-05	0.004929421
417.679995	530.339539	1.1969E-05	0.004999222
417.719995	463.657126	1.04641E-05	0.004371062
417.759995	340.521865	7.6851E-06	0.003210529
417.799995	285.360994	6.4402E-06	0.002690716
417.839995	274.392065	6.19265E-06	0.002587535
417.879995	296.889252	6.70038E-06	0.002799953
417.919995	395.911193	8.93516E-06	0.003734184
417.959995	630.664379	1.42332E-05	0.005948915
417.999995	1112.805009	2.51145E-05	0.010497844
418.039995	1976.830998	4.46143E-05	0.018650571
418.079995	3084.827292	6.96203E-05	0.029106836
418.119995	4168.426741	9.40756E-05	0.039334883
418.159995	4814.497448	0.000108657	0.045435802
418.199995	4937.62411	0.000111435	0.046602241
418.239995	4528.892587	0.000102211	0.042748643
418.279995	3587.310972	8.09606E-05	0.033864204
418.319995	2492.310653	5.6248E-05	0.023529655
418.359995	1568.482434	3.53985E-05	0.014809301
418.399995	926.370679	2.09069E-05	0.008747445
418.439995	532.010147	1.20067E-05	0.005024095
418.479995	347.580643	7.84441E-06	0.003282729
418.519995	321.838653	7.26345E-06	0.003039899
418.559995	361.947704	8.16866E-06	0.003419072
418.599995	431.2113	9.73184E-06	0.004073748
418.639995	475.581913	1.07332E-05	0.004493356

418.679995	484.866306	1.09428E-05	0.004581514
418.719995	439.697383	9.92336E-06	0.004155108
418.759995	372.854295	8.4148E-06	0.003523782
418.799995	320.487931	7.23297E-06	0.003029166
418.839995	331.144113	7.47346E-06	0.003130185
418.879995	421.988008	9.52368E-06	0.00398928
418.919995	627.941295	1.41718E-05	0.005936834
418.959995	1163.619134	2.62613E-05	0.011002419
418.999995	2185.803751	4.93305E-05	0.020669499
419.039995	3776.593012	8.52324E-05	0.035715803
419.079995	5567.243402	0.000125645	0.052655276
419.119995	7110.616411	0.000160477	0.067258997
419.159995	8014.215571	0.00018087	0.075813335
419.199995	7999.505213	0.000180538	0.075681399
419.239995	6972.885879	0.000157368	0.065975094
419.279995	5374.635945	0.000121298	0.050857845
419.319995	3650.014883	8.23758E-05	0.0345418
419.359995	2166.727439	4.89E-05	0.020506712
419.399995	1150.806676	2.59721E-05	0.0108927
419.439995	601.529317	1.35757E-05	0.005694183
419.479995	374.079239	8.44245E-06	0.003541438
419.519995	352.304361	7.95102E-06	0.003335611
419.559995	398.510052	8.99382E-06	0.003773446
419.599995	456.667859	1.03064E-05	0.004324548
419.639995	510.146189	1.15133E-05	0.004831437
419.679995	503.889938	1.13721E-05	0.00477264
419.719995	444.73961	1.00372E-05	0.004212794
419.759995	354.205605	7.99393E-06	0.003355531
419.799995	307.947785	6.94995E-06	0.00291759
419.839995	322.049798	7.26822E-06	0.003051488
419.879995	375.58359	8.4764E-06	0.00355907
419.919995	479.334107	1.08179E-05	0.004542654
419.959995	730.736057	1.64917E-05	0.006925852
419.999995	1245.19542	2.81023E-05	0.011802977
420.039995	2019.606876	4.55797E-05	0.019145303
420.079995	2939.098529	6.63314E-05	0.027864477
420.119995	3818.90766	8.61874E-05	0.036209061
420.159995	4405.933098	9.94358E-05	0.041778934
420.199995	4463.969139	0.000100746	0.042333286
420.239995	3977.230974	8.97606E-05	0.037720979
420.279995	3220.447872	7.2681E-05	0.03054638
420.319995	2363.336724	5.33372E-05	0.022418699
420.359995	1549.308762	3.49657E-05	0.014698199
420.399995	925.283105	2.08824E-05	0.00877894
420.439995	538.680405	1.21573E-05	0.005111401
420.479995	391.383893	8.83299E-06	0.003714095

420.519995	400.757205	9.04453E-06	0.003803407
420.559995	474.295774	1.07042E-05	0.004501756
420.599995	549.583876	1.24033E-05	0.005216846
420.639995	595.895819	1.34485E-05	0.005656993
420.679995	542.200505	1.22367E-05	0.005147739
420.719995	439.758153	9.92473E-06	0.004175532
420.759995	372.878482	8.41535E-06	0.003540842
420.799995	331.650066	7.48488E-06	0.003149637
420.839995	315.741131	7.12584E-06	0.002998837
420.879995	376.602763	8.4994E-06	0.003577227
420.919995	584.700589	1.31959E-05	0.005554409
420.959995	1123.778076	2.53621E-05	0.010676432
420.999995	2125.309216	4.79653E-05	0.020193378
421.039995	3577.908496	8.07484E-05	0.033998311
421.079995	5193.141649	0.000117202	0.049351406
421.119995	6459.399237	0.00014578	0.061390723
421.159995	7119.638672	0.00016068	0.067672126
421.199995	7079.764089	0.00015978	0.06729951
421.239995	6400.7767	0.000144457	0.060850904
421.279995	5309.741195	0.000119833	0.050483441
421.319995	4007.884017	9.04524E-05	0.038109388
421.359995	2708.597425	6.11293E-05	0.025757429
421.399995	1689.350888	3.81263E-05	0.016066421
421.439995	1022.574667	2.30781E-05	0.009726028
421.479995	670.663758	1.51359E-05	0.006379499
421.519995	539.515715	1.21761E-05	0.005132477
421.559995	499.359465	1.12698E-05	0.004750917
421.599995	497.449534	1.12267E-05	0.004733195
421.639995	508.084199	1.14668E-05	0.004834842
421.679995	492.645905	1.11183E-05	0.004688378
421.719995	429.139132	9.68507E-06	0.004084389
421.759995	348.710097	7.8699E-06	0.003319209
421.799995	290.685037	6.56036E-06	0.002767158
421.839995	310.980437	7.0184E-06	0.00296064
421.879995	375.927409	8.48416E-06	0.003579297
421.919995	470.850523	1.06264E-05	0.004483508
421.959995	680.686486	1.53621E-05	0.006482211
421.999995	1177.274673	2.65694E-05	0.011212308
422.039995	1949.685664	4.40017E-05	0.018570473
422.079995	2823.130478	6.37141E-05	0.026892457
422.119995	3552.015855	8.01641E-05	0.033838849
422.159995	3955.045304	8.92599E-05	0.037681944
422.199995	3971.62709	8.96341E-05	0.037843513
422.239995	3610.949664	8.14941E-05	0.03441007
422.279995	2980.426083	6.72641E-05	0.028404269
422.319995	2224.268692	5.01986E-05	0.021199892

422.359995	1538.469733	3.47211E-05	0.014664812
422.399995	1001.9408	2.26124E-05	0.009551481
422.439995	654.567885	1.47727E-05	0.006240573
422.479995	470.009579	1.06075E-05	0.00448144
422.519995	420.024262	9.47936E-06	0.00400522
422.559995	463.466198	1.04598E-05	0.004419887
422.599995	532.832724	1.20253E-05	0.005081889
422.639995	562.375671	1.2692E-05	0.005364162
422.679995	524.293334	1.18326E-05	0.005001391
422.719995	476.20372	1.07473E-05	0.004543079
422.759995	393.134149	8.87249E-06	0.003750934
422.799995	307.740445	6.94527E-06	0.002936461
422.839995	294.336526	6.64277E-06	0.002808827
422.879995	370.557711	8.36297E-06	0.003536533
422.919995	580.830153	1.31085E-05	0.005543858
422.959995	1063.381475	2.3999E-05	0.010150633
422.999995	1940.150603	4.37865E-05	0.018521688
423.039995	3212.084687	7.24923E-05	0.030667133
423.079995	4858.157333	0.000109642	0.046387271
423.119995	6319.622197	0.000142625	0.060347519
423.159995	7148.014448	0.000161321	0.068264479
423.199995	7295.152714	0.000164641	0.069676255
423.239995	6444.46015	0.000145442	0.061557078
423.279995	4988.975904	0.000112594	0.047658888
423.319995	3493.547874	7.88445E-05	0.033376457
423.359995	2209.410044	4.98633E-05	0.02111013
423.399995	1303.291104	2.94135E-05	0.012453661
423.439995	748.37041	1.68897E-05	0.007151765
423.479995	482.840111	1.0897E-05	0.004614674
423.519995	441.714794	9.96889E-06	0.004222023
423.559995	493.764016	1.11436E-05	0.004719969
423.599995	520.274795	1.17419E-05	0.004973859
423.639995	518.055066	1.16918E-05	0.004953106
423.679995	520.043375	1.17367E-05	0.004972586
423.719995	484.363605	1.09314E-05	0.004631858
423.759995	396.427788	8.94682E-06	0.003791306
423.799995	322.802351	7.2852E-06	0.003087467
423.839995	300.960056	6.79225E-06	0.002878827
423.879995	343.133333	7.74404E-06	0.003282544
423.919995	460.551919	1.0394E-05	0.004406231
423.959995	697.987374	1.57526E-05	0.006678473
423.999995	1156.804568	2.61075E-05	0.011069566
424.039995	1897.652168	4.28274E-05	0.018160516
424.079995	2766.810249	6.24431E-05	0.02648085
424.119995	3586.764867	8.09483E-05	0.034331787
424.159995	4124.099297	9.30752E-05	0.039478767

424.199995	4029.962012	9.09506E-05	0.038581257
424.239995	3434.708575	7.75166E-05	0.032885637
424.279995	2658.984572	6.00096E-05	0.025460864
424.319995	1864.797327	4.20859E-05	0.01785788
424.359995	1213.980764	2.73979E-05	0.011626554
424.399995	759.021497	1.71301E-05	0.007269997
424.439995	477.482531	1.07761E-05	0.004573814
424.479995	343.349258	7.74891E-06	0.003289259
424.519995	328.120591	7.40522E-06	0.003143666
424.559995	408.76035	9.22515E-06	0.00391663
424.599995	498.421338	1.12487E-05	0.004776187
424.639995	537.844742	1.21384E-05	0.005154453
424.679995	520.072924	1.17373E-05	0.004984606
424.719995	466.494088	1.05281E-05	0.004471504
424.759995	403.40883	9.10438E-06	0.003867174
424.799995	392.733857	8.86346E-06	0.003765196
424.839995	460.737667	1.03982E-05	0.004417574
424.879995	599.953446	1.35401E-05	0.005752923
424.919995	852.047939	1.92295E-05	0.008171014
424.959995	1355.489075	3.05915E-05	0.013000164
424.999995	2215.734438	5.0006E-05	0.021252567
425.039995	3623.921093	8.17869E-05	0.034762683
425.079995	5356.257563	0.000120883	0.051385061
425.119995	6859.916239	0.000154819	0.065816548
425.159995	7679.227166	0.000173309	0.07368425
425.199995	7489.684418	0.000169032	0.071872297
425.239995	6408.18142	0.000144624	0.061499794
425.279995	4855.246403	0.000109576	0.046600544
425.319995	3332.867883	7.52182E-05	0.031991798
425.359995	2113.977562	4.77095E-05	0.020293725
425.399995	1257.446826	2.83788E-05	0.012072351
425.439995	716.224863	1.61642E-05	0.006876896
425.479995	439.929153	9.92859E-06	0.004224416
425.519995	349.289642	7.88298E-06	0.003354366
425.559995	374.80245	8.45877E-06	0.003599714
425.599995	481.663352	1.08705E-05	0.004626472
425.639995	536.239372	1.21022E-05	0.00515117
425.679995	518.819827	1.1709E-05	0.004984304
425.719995	453.457762	1.02339E-05	0.00435678
425.759995	381.990482	8.62099E-06	0.003670474
425.799995	331.543564	7.48248E-06	0.003186038
425.839995	319.142516	7.2026E-06	0.003067156
425.879995	345.372236	7.79457E-06	0.003319552
425.919995	446.586633	1.00788E-05	0.004292779
425.959995	722.026412	1.62951E-05	0.006941074
425.999995	1289.09809	2.90931E-05	0.012393681

426.039995	2185.601884	4.9326E-05	0.021014845
426.079995	3220.757143	7.2688E-05	0.030970904
426.119995	4035.975983	9.10864E-05	0.038813718
426.159995	4466.837242	0.00010081	0.042961315
426.199995	4408.691854	9.9498E-05	0.042406062
426.239995	3884.009789	8.76567E-05	0.037362788
426.279995	3057.770809	6.90096E-05	0.029417424
426.319995	2147.567333	4.84676E-05	0.020662708
426.359995	1346.230069	3.03825E-05	0.012953899
426.399995	778.311205	1.75654E-05	0.007489886
426.439995	479.60839	1.08241E-05	0.004615826
426.479995	363.030063	8.19308E-06	0.003494186
426.519995	370.947992	8.37178E-06	0.003570731
426.559995	471.60196	1.06434E-05	0.004540048
426.599995	612.415176	1.38214E-05	0.005896191
426.639995	690.579483	1.55854E-05	0.006649362
426.679995	654.547679	1.47722E-05	0.006303015
426.719995	547.522045	1.23568E-05	0.005272898
426.759995	404.109948	9.1202E-06	0.003892136
426.799995	316.271533	7.13781E-06	0.003046416
426.839995	325.782438	7.35246E-06	0.003138322
426.879995	414.77867	9.36098E-06	0.003996014
426.919995	596.967458	1.34727E-05	0.005751775
426.959995	1067.984554	2.41029E-05	0.010290984
426.999995	1930.18909	4.35617E-05	0.018600837
427.039995	3213.015148	7.25133E-05	0.030966069
427.079995	4609.070279	0.00010402	0.044424988
427.119995	5801.633999	0.000130935	0.05592487
427.159995	6385.395763	0.000144109	0.061557808
427.199995	6137.942664	0.000138525	0.059177801
427.239995	5272.648769	0.000118996	0.050839995
427.279995	4076.840117	9.20086E-05	0.039313435
427.319995	2848.102166	6.42777E-05	0.027467146
427.359995	1829.331006	4.12855E-05	0.017643751
427.399995	1095.62384	2.47267E-05	0.010568193
427.439995	631.175825	1.42448E-05	0.00608878
427.479995	394.246193	8.89759E-06	0.003803541
427.519995	347.416276	7.8407E-06	0.003352057
427.559995	403.209939	9.09989E-06	0.003890748
427.599995	482.516003	1.08897E-05	0.004656442
427.639995	508.57687	1.14779E-05	0.004908397
427.679995	496.87874	1.12139E-05	0.004795944
427.719995	456.537489	1.03034E-05	0.004406977
427.759995	378.47433	8.54164E-06	0.003653771
427.799995	312.611331	7.0552E-06	0.003018216
427.839995	279.73998	6.31334E-06	0.0027011

427.879995	306.601235	6.91956E-06	0.002960743
427.919995	413.891859	9.34096E-06	0.003997185
427.959995	667.255019	1.5059E-05	0.006444657
427.999995	1131.010569	2.55253E-05	0.010924842
428.039995	1854.767993	4.18595E-05	0.017917553
428.079995	2775.738379	6.26445E-05	0.026816878
428.119995	3745.862279	8.45389E-05	0.036192791
428.159995	4396.568458	9.92244E-05	0.04248393
428.199995	4552.025586	0.000102733	0.043990217
428.239995	4255.458424	9.60398E-05	0.04112807
428.279995	3572.742877	8.06318E-05	0.034533
428.319995	2677.371235	6.04245E-05	0.025881038
428.359995	1727.668822	3.89911E-05	0.016702218
428.399995	955.300404	2.15598E-05	0.009236218
428.439995	498.453878	1.12494E-05	0.004819697
428.479995	319.366477	7.20766E-06	0.003088337
428.519995	312.629917	7.05562E-06	0.003023475
428.559995	376.28905	8.49232E-06	0.003639469
428.599995	464.794829	1.04898E-05	0.004495916
428.639995	556.346017	1.2556E-05	0.005381984
428.679995	566.184877	1.2778E-05	0.005477675
428.719995	551.274022	1.24415E-05	0.005333914
428.759995	541.531964	1.22216E-05	0.005240143
428.799995	474.4712	1.07082E-05	0.004591656
428.839995	424.470048	9.5797E-06	0.004108158
428.879995	426.981121	9.63637E-06	0.004132846
428.919995	569.321737	1.28488E-05	0.005511107
428.959995	1003.225271	2.26414E-05	0.009712254
428.999995	1846.403007	4.16707E-05	0.017876749
429.039995	3107.344093	7.01284E-05	0.0300879
429.079995	4639.49087	0.000104707	0.044927613
429.119995	5984.445472	0.000135061	0.057957203
429.159995	6610.063735	0.00014918	0.064022059
429.199995	6474.026448	0.00014611	0.062710308
429.239995	5796.524813	0.000130819	0.056152958
429.279995	4646.297611	0.00010486	0.0450145
429.319995	3276.077132	7.39365E-05	0.031742417
429.359995	2073.223535	4.67898E-05	0.020089654
429.399995	1201.395552	2.71138E-05	0.011642676
429.439995	651.37139	1.47005E-05	0.006313002
429.479995	377.666538	8.52341E-06	0.003660633
429.519995	315.326856	7.11649E-06	0.003056674
429.559995	400.094827	9.02958E-06	0.003878748
429.599995	483.51413	1.09122E-05	0.004687898
429.639995	534.604607	1.20653E-05	0.005183727
429.679995	537.196823	1.21238E-05	0.005209347

429.719995	470.50644	1.06187E-05	0.004563057
429.759995	369.224988	8.33289E-06	0.003581144
429.799995	291.473415	6.57815E-06	0.002827288
429.839995	276.802962	6.24706E-06	0.002685235
429.879995	324.234585	7.31752E-06	0.003145657
429.919995	436.796143	9.85788E-06	0.0042381
429.959995	706.282898	1.59398E-05	0.006853485
429.999995	1257.883693	2.83887E-05	0.012207133
430.039995	2094.419401	4.72681E-05	0.020327186
430.079995	3087.943966	6.96906E-05	0.02997253
430.119995	3990.796716	9.00667E-05	0.038739499
430.159995	4484.197184	0.000101202	0.043533088
430.199995	4481.668208	0.000101145	0.043512582
430.239995	4082.22254	9.21301E-05	0.039638044
430.279995	3308.887073	7.4677E-05	0.032132007
430.319995	2421.611329	5.46524E-05	0.023518018
430.359995	1594.652988	3.59891E-05	0.015488268
430.399995	924.086386	2.08553E-05	0.008976139
430.439995	536.206976	1.21014E-05	0.005208946
430.479995	373.105136	8.42046E-06	0.003624841
430.519995	345.445336	7.79622E-06	0.003356429
430.559995	404.333051	9.12523E-06	0.003928961
430.599995	469.130307	1.05876E-05	0.004559028
430.639995	483.695993	1.09163E-05	0.004701015
430.679995	487.571942	1.10038E-05	0.004739125
430.719995	467.771266	1.05569E-05	0.004547088
430.759995	406.104073	9.1652E-06	0.003948003
430.799995	330.537026	7.45976E-06	0.003213665
430.839995	318.85151	7.19603E-06	0.003100339
430.879995	399.10837	9.00732E-06	0.003881074
430.919995	614.758359	1.38742E-05	0.005978687
430.959995	1063.16111	2.39941E-05	0.010340482
430.999995	1880.326959	4.24364E-05	0.018290071
431.039995	3222.519138	7.27278E-05	0.031348577
431.079995	4714.749513	0.000106405	0.045869209
431.119995	5906.61094	0.000133304	0.057470012
431.159995	6567.466666	0.000148219	0.06390592
431.199995	6496.475222	0.000146616	0.06322099
431.239995	5575.717034	0.000125836	0.054265588
431.279995	4211.68341	9.50518E-05	0.040993952
431.319995	2841.838949	6.41363E-05	0.027663289
431.359995	1729.003926	3.90212E-05	0.016832189
431.399995	968.414373	2.18558E-05	0.009428576
431.439995	552.028769	1.24585E-05	0.005375104
431.479995	368.400922	8.3143E-06	0.003587452
431.519995	339.301479	7.65756E-06	0.003304391

431.559995	374.091662	8.44273E-06	0.003643544
431.599995	423.9948	9.56897E-06	0.004129968
431.639995	466.021102	1.05174E-05	0.004539751
431.679995	472.557194	1.0665E-05	0.004603849
431.719995	420.364498	9.48704E-06	0.004095745
431.759995	344.649778	7.77827E-06	0.003358344
431.799995	299.892738	6.76816E-06	0.002922492
431.839995	292.126556	6.59289E-06	0.002847073
431.879995	301.313941	6.80024E-06	0.002936886
431.919995	380.429338	8.58576E-06	0.003708362
431.959995	629.272777	1.42018E-05	0.006134614
431.999995	1123.851662	2.53638E-05	0.010957147
432.039995	1933.168803	4.36289E-05	0.018849441
432.079995	2900.218937	6.54539E-05	0.028281322
432.119995	3665.810279	8.27322E-05	0.035750252
432.159995	4143.067383	9.35033E-05	0.040408368
432.199995	4144.166922	9.35281E-05	0.040422834
432.239995	3699.215354	8.34861E-05	0.036086048
432.279995	3009.510655	6.79205E-05	0.029360658
432.319995	2234.976192	5.04403E-05	0.02180635
432.359995	1516.014307	3.42143E-05	0.014792908
432.399995	957.39151	2.1607E-05	0.009342864
432.439995	635.325295	1.43384E-05	0.006200501
432.479995	489.625818	1.10502E-05	0.004778979
432.519995	532.366961	1.20148E-05	0.005196633
432.559995	681.53987	1.53814E-05	0.006653381
432.599995	847.736411	1.91322E-05	0.008276603
432.639995	956.998326	2.15981E-05	0.00934421
432.679995	1046.986437	2.3629E-05	0.010223807
432.719995	1194.497722	2.69581E-05	0.01166533
432.759995	1438.376935	3.24622E-05	0.014048326
432.799995	1830.905452	4.1321E-05	0.017883722
432.839995	2535.123618	5.72142E-05	0.024764598
432.879995	3740.766135	8.44239E-05	0.036545409
432.919995	5791.769915	0.000130712	0.056587916
432.959995	9370.165467	0.000211472	0.091558736
432.999995	15508.29584	0.000350001	0.151550274
433.039995	24238.27467	0.000547024	0.236883313
433.079995	33063.15976	0.000746189	0.323159729
433.119995	38008.63501	0.000857802	0.371531177
433.159995	36405.45578	0.00082162	0.355893101
433.199995	29649.56406	0.000669149	0.2898755
433.239995	20726.95204	0.000467778	0.202660328
433.279995	12646.79159	0.00028542	0.12366698
433.319995	6925.832657	0.000156306	0.067730687
433.359995	3479.045746	7.85172E-05	0.034026221

433.399995	1624.989001	3.66737E-05	0.015894398
433.439995	778.88321	1.75783E-05	0.007619142
433.479995	453.743042	1.02403E-05	0.004438986
433.519995	393.566811	8.88225E-06	0.003850635
433.559995	423.96756	9.56836E-06	0.004148457
433.599995	503.991128	1.13744E-05	0.00493193
433.639995	578.408128	1.30539E-05	0.005660678
433.679995	613.636465	1.38489E-05	0.006006
433.719995	595.721045	1.34446E-05	0.005831189
433.759995	588.540454	1.32825E-05	0.005761434
433.799995	614.881225	1.3877E-05	0.006019848
433.839995	748.874211	1.6901E-05	0.007332351
433.879995	1037.222933	2.34087E-05	0.010156557
433.919995	1530.103561	3.45323E-05	0.014984258
433.959995	2485.14724	5.60863E-05	0.024339216
433.999995	4195.587728	9.46886E-05	0.041094839
434.039995	6819.096442	0.000153898	0.066797674
434.079995	9756.424488	0.000220189	0.095579601
434.119995	11685.82539	0.000263733	0.114491683
434.159995	11616.18412	0.000262161	0.11381986
434.199995	9829.92517	0.000221848	0.096326279
434.239995	7409.328757	0.000167218	0.072612843
434.279995	5051.821269	0.000114013	0.049513378
434.319995	3136.936856	7.07963E-05	0.030748247
434.359995	1823.967278	4.11644E-05	0.017880169
434.399995	1006.259851	2.27099E-05	0.009865173
434.439995	553.382413	1.24891E-05	0.005425752
434.479995	375.336806	8.47083E-06	0.003680406
434.519995	407.028725	9.18607E-06	0.003991532
434.559995	561.963839	1.26827E-05	0.005511412
434.599995	724.427368	1.63493E-05	0.007105413
434.639995	793.860627	1.79163E-05	0.007787153
434.679995	718.358073	1.62123E-05	0.00704718
434.719995	576.901849	1.30199E-05	0.005659998
434.759995	473.556231	1.06875E-05	0.004646499
434.799995	427.165782	9.64054E-06	0.004191705
434.839995	517.555976	1.16805E-05	0.005079156
434.879995	726.583654	1.6398E-05	0.007131154
434.919995	1134.020473	2.55933E-05	0.011131022
434.959995	2063.74083	4.65758E-05	0.020258591
434.999995	3751.425858	8.46645E-05	0.036829038
435.039995	6271.832791	0.000141547	0.061578402
435.079995	9044.520382	0.000204122	0.088809498
435.119995	11239.60392	0.000253662	0.110373497
435.159995	12183.35726	0.000274961	0.119652203
435.199995	11684.40268	0.000263701	0.11476254

435.239995	10003.07701	0.000225756	0.098257829
435.279995	7619.927408	0.000171971	0.0748556
435.319995	5194.175675	0.000117225	0.051030521
435.359995	3208.638687	7.24145E-05	0.031526379
435.399995	1806.607654	4.07726E-05	0.017752398
435.439995	940.18941	2.12188E-05	0.009239499
435.479995	532.975667	1.20285E-05	0.005238179
435.519995	431.702513	9.74292E-06	0.004243238
435.559995	469.805198	1.06028E-05	0.004618177
435.599995	521.602484	1.17718E-05	0.005127814
435.639995	540.289165	1.21936E-05	0.005312008
435.679995	524.368235	1.18343E-05	0.005155951
435.719995	470.42719	1.06169E-05	0.00462599
435.759995	388.109836	8.7591E-06	0.003816865
435.799995	316.490424	7.14275E-06	0.00311281
435.839995	293.730921	6.6291E-06	0.002889226
435.879995	336.097722	7.58526E-06	0.003306262
435.919995	457.788762	1.03317E-05	0.004503775
435.959995	748.251351	1.6887E-05	0.007362052
435.999995	1333.554469	3.00965E-05	0.013122059
436.039995	2315.69193	5.22619E-05	0.022788297
436.079995	3530.712831	7.96833E-05	0.03474828
436.119995	4617.766211	0.000104217	0.045450922
436.159995	5343.979458	0.000120606	0.052603588
436.199995	5462.786778	0.000123287	0.053778002
436.239995	4960.864762	0.00011196	0.048841346
436.279995	4034.907547	9.10622E-05	0.039728635
436.319995	2931.249229	6.61542E-05	0.028864406
436.359995	1915.409457	4.32281E-05	0.018863024
436.399995	1157.936158	2.6133E-05	0.011404444
436.439995	685.660242	1.54744E-05	0.006753646
436.479995	447.201815	1.00927E-05	0.004405271
436.519995	372.968101	8.41737E-06	0.003674351
436.559995	397.324327	8.96706E-06	0.003914658
436.599995	479.282437	1.08167E-05	0.004722587
436.639995	539.075908	1.21662E-05	0.005312246
436.679995	527.956845	1.19153E-05	0.005203152
436.719995	466.821865	1.05355E-05	0.004601072
436.759995	424.004187	9.56918E-06	0.004179437
436.799995	396.865292	8.9567E-06	0.003912285
436.839995	386.864285	8.73099E-06	0.003814045
436.879995	404.11589	9.12033E-06	0.003984491
436.919995	530.769046	1.19787E-05	0.005233741
436.959995	893.729439	2.01702E-05	0.008813583
436.999995	1664.778148	3.75717E-05	0.016418843
437.039995	2901.036478	6.54724E-05	0.028614037

437.079995	4385.500418	9.89746E-05	0.043259833
437.119995	5789.817879	0.000130668	0.05711765
437.159995	6628.515468	0.000149596	0.065397545
437.199995	6657.244193	0.000150245	0.065686995
437.239995	6110.82391	0.000137913	0.060300985
437.279995	5128.532714	0.000115744	0.050612466
437.319995	3867.056486	8.72741E-05	0.0381667
437.359995	2653.165824	5.98783E-05	0.026188354
437.399995	1687.583359	3.80864E-05	0.016658994
437.439995	1024.231948	2.31155E-05	0.010111639
437.479995	644.909578	1.45547E-05	0.006367395
437.519995	475.752426	1.07371E-05	0.004697683
437.559995	464.248332	1.04774E-05	0.004584508
437.599995	531.006779	1.19841E-05	0.005244235
437.639995	619.792872	1.39879E-05	0.006121648
437.679995	643.72225	1.45279E-05	0.006358578
437.719995	592.247236	1.33662E-05	0.005850651
437.759995	522.662225	1.17958E-05	0.005163711
437.799995	535.297889	1.20809E-05	0.00528903
437.839995	595.337603	1.34359E-05	0.005882792
437.879995	643.290749	1.45182E-05	0.006357219
437.919995	687.691188	1.55202E-05	0.00679662
437.959995	824.307785	1.86035E-05	0.008147579
437.999995	1191.119771	2.68819E-05	0.011774278
438.039995	1831.22857	4.13283E-05	0.018103439
438.079995	2735.690755	6.17407E-05	0.027047379
438.119995	3578.072272	8.07521E-05	0.035379113
438.159995	4136.753267	9.33608E-05	0.04090695
438.199995	4217.546167	9.51841E-05	0.041709691
438.239995	3880.166068	8.75699E-05	0.038376652
438.279995	3240.332259	7.31298E-05	0.032051322
438.319995	2387.855503	5.38906E-05	0.023621316
438.359995	1574.05583	3.55242E-05	0.015572409
438.399995	947.158439	2.1376E-05	0.009371259
438.439995	571.435005	1.28965E-05	0.005654338
438.479995	412.314308	9.30536E-06	0.004080214
438.519995	373.828473	8.43679E-06	0.0036997
438.559995	406.889498	9.18293E-06	0.004027265
438.599995	479.535063	1.08224E-05	0.004746721
438.639995	514.023238	1.16008E-05	0.00508857
438.679995	486.919345	1.09891E-05	0.004820695
438.719995	458.908242	1.03569E-05	0.004543788
438.759995	409.663582	9.24554E-06	0.004056572
438.799995	381.112411	8.60118E-06	0.003774196
438.839995	386.014512	8.71181E-06	0.003823091
438.879995	440.025893	9.93077E-06	0.004358417

438.919995	600.385567	1.35499E-05	0.005947307
438.959995	983.686168	2.22004E-05	0.009745099
438.999995	1830.995328	4.1323E-05	0.018140803
439.039995	3124.469721	7.05149E-05	0.030958874
439.079995	4653.797337	0.00010503	0.046116451
439.119995	6145.490591	0.000138695	0.060903819
439.159995	7044.501454	0.000158985	0.06981967
439.199995	7182.38965	0.000162097	0.071192795
439.239995	6562.974078	0.000148117	0.065058991
439.279995	5432.581915	0.000122606	0.053858278
439.319995	4124.864222	9.30924E-05	0.04089737
439.359995	2824.353149	6.37417E-05	0.02800556
439.399995	1731.143666	3.90695E-05	0.017167139
439.439995	971.711574	2.19302E-05	0.009636997
439.479995	562.023177	1.26841E-05	0.0055744
439.519995	425.506527	9.60309E-06	0.00422075
439.559995	425.808843	9.60991E-06	0.004224133
439.599995	472.597145	1.06659E-05	0.004688712
439.639995	521.06889	1.17598E-05	0.005170078
439.679995	513.604104	1.15913E-05	0.005096476
439.719995	476.410464	1.07519E-05	0.004727834
439.759995	417.492733	9.42223E-06	0.00414352
439.799995	365.741654	8.25428E-06	0.003630232
439.839995	360.141204	8.12789E-06	0.003574969
439.879995	393.612029	8.88328E-06	0.003907575
439.919995	484.475181	1.09339E-05	0.004810054
439.959995	652.509624	1.47262E-05	0.006478954
439.999995	989.22553	2.23254E-05	0.009823195
440.039995	1698.698252	3.83373E-05	0.016869925
440.079995	2604.418743	5.87781E-05	0.025867067
440.119995	3405.72605	7.68625E-05	0.03382872
440.159995	3951.224317	8.91736E-05	0.039250664
440.199995	4127.853777	9.31599E-05	0.041008992
440.239995	3765.056594	8.49721E-05	0.037408109
440.279995	3103.498865	7.00416E-05	0.030837936
440.319995	2309.442175	5.21209E-05	0.022949871
440.359995	1553.55646	3.50616E-05	0.015439728
440.399995	944.404966	2.13139E-05	0.009386643
440.439995	546.449279	1.23326E-05	0.00543177
440.479995	369.455779	8.3381E-06	0.003672767
440.519995	349.242046	7.88191E-06	0.003472137
440.559995	407.970495	9.20733E-06	0.004056379
440.599995	476.520908	1.07544E-05	0.004738394
440.639995	531.409775	1.19932E-05	0.005284674
440.679995	535.744701	1.2091E-05	0.005328267
440.719995	473.473608	1.06856E-05	0.004709375

440.759995	427.416212	9.64619E-06	0.004251654
440.799995	389.157256	8.78274E-06	0.003871431
440.839995	355.829001	8.03056E-06	0.003540194
440.879995	390.119821	8.80446E-06	0.003881711
440.919995	594.886281	1.34258E-05	0.005919684
440.959995	1072.446033	2.42036E-05	0.010672825
440.999995	1908.215249	4.30658E-05	0.018992
441.039995	3148.538493	7.10581E-05	0.031339477
441.079995	4520.137764	0.000102013	0.044995988
441.119995	5663.51378	0.000127818	0.056382907
441.159995	6295.572242	0.000142082	0.062681025
441.199995	6197.274177	0.000139864	0.061707928
441.239995	5465.773483	0.000123355	0.054429113
441.279995	4300.990286	9.70674E-05	0.042833884
441.319995	3062.447854	6.91152E-05	0.030501912
441.359995	2024.69395	4.56945E-05	0.020167734
441.399995	1246.041559	2.81214E-05	0.012412796
441.439995	721.990856	1.62943E-05	0.007192968
441.479995	455.107497	1.02711E-05	0.004534504
441.519995	423.309312	9.5535E-06	0.004218062
441.559995	476.059157	1.0744E-05	0.004744117
441.599995	510.260979	1.15159E-05	0.005085412
441.639995	498.208427	1.12439E-05	0.004965743
441.679995	444.25886	1.00263E-05	0.004428418
441.719995	384.623548	8.68042E-06	0.003834314
441.759995	317.260994	7.16014E-06	0.003163063
441.799995	265.868549	6.00028E-06	0.002650925
441.839995	251.794127	5.68264E-06	0.002510819
441.879995	277.766787	6.26881E-06	0.002770062
441.919995	388.683688	8.77205E-06	0.003876544
441.959995	631.662524	1.42557E-05	0.006300468
441.999995	1087.342689	2.45398E-05	0.010846596
442.039995	1836.234533	4.14413E-05	0.018318692
442.079995	2764.368079	6.23879E-05	0.027580459
442.119995	3676.690892	8.29778E-05	0.036686141
442.159995	4339.552213	9.79376E-05	0.04330411
442.199995	4485.857462	0.00010124	0.044768131
442.239995	4177.287263	9.42756E-05	0.041692421
442.279995	3531.062424	7.96912E-05	0.035245806
442.319995	2653.932851	5.98956E-05	0.026493007
442.359995	1760.059646	3.97221E-05	0.017571466
442.399995	1065.63675	2.40499E-05	0.010639692
442.439995	627.985684	1.41728E-05	0.006270597
442.479995	389.254906	8.78494E-06	0.003887161
442.519995	333.230727	7.52055E-06	0.003327995
442.559995	397.365537	8.96799E-06	0.003968872

442.599995	473.075724	1.06767E-05	0.00472549
442.639995	514.351282	1.16082E-05	0.00513825
442.679995	509.180252	1.14915E-05	0.005087052
442.719995	472.27087	1.06585E-05	0.004718729
442.759995	414.967498	9.36524E-06	0.004146553
442.799995	391.300768	8.83111E-06	0.003910417
442.839995	430.025197	9.70507E-06	0.004297793
442.879995	478.644176	1.08023E-05	0.004784137
442.919995	617.866615	1.39444E-05	0.006176249
442.959995	981.383233	2.21485E-05	0.009810878
442.999995	1713.990453	3.86824E-05	0.017136293
443.039995	2863.675429	6.46292E-05	0.028633305
443.079995	4133.570247	9.32889E-05	0.041334455
443.119995	5154.825256	0.000116337	0.051551351
443.159995	5770.153767	0.000130224	0.057710215
443.199995	5729.72918	0.000129312	0.057311081
443.239995	5021.10496	0.000113319	0.050227666
443.279995	3971.032225	8.96207E-05	0.039727048
443.319995	2803.948023	6.32812E-05	0.028053822
443.359995	1784.624696	4.02765E-05	0.017856986
443.399995	1054.647458	2.38019E-05	0.010553773
443.439995	596.228949	1.34561E-05	0.005966954
443.479995	380.49315	8.5872E-06	0.003808252
443.519995	350.836753	7.9179E-06	0.003511746
443.559995	422.274337	9.53014E-06	0.00422719
443.599995	519.194049	1.17175E-05	0.005197877
443.639995	563.003706	1.27062E-05	0.005636983
443.679995	541.11592	1.22122E-05	0.005418323
443.719995	473.188748	1.06792E-05	0.004738579
443.759995	391.561977	8.83701E-06	0.003921511
443.799995	331.255967	7.47599E-06	0.003317842
443.839995	302.709227	6.83173E-06	0.003032193
443.879995	316.345367	7.13947E-06	0.00316907
443.919995	399.570637	9.01775E-06	0.004003161
443.959995	599.551451	1.3531E-05	0.006007241
443.999995	988.746322	2.23146E-05	0.009907694
444.039995	1652.703608	3.72992E-05	0.016562345
444.079995	2484.672307	5.60756E-05	0.024902049
444.119995	3232.321098	7.2949E-05	0.032398103
444.159995	3727.629565	8.41274E-05	0.037366029
444.199995	3848.155172	8.68475E-05	0.03857766
444.239995	3535.236522	7.97854E-05	0.03544385
444.279995	2861.51427	6.45804E-05	0.028691776
444.319995	2033.407291	4.58912E-05	0.020390365
444.359995	1275.096204	2.87771E-05	0.012787412
444.399995	760.895562	1.71724E-05	0.007631394

444.439995	466.883425	1.05369E-05	0.004683024
444.479995	350.951634	7.92049E-06	0.003520499
444.519995	358.613368	8.0934E-06	0.00359768
444.559995	397.483078	8.97064E-06	0.003987987
444.599995	449.824474	1.01519E-05	0.00451354
444.639995	506.67067	1.14349E-05	0.005084392
444.679995	482.597521	1.08916E-05	0.004843256
444.719995	385.140058	8.69207E-06	0.003865539
444.759995	308.846379	6.97023E-06	0.003100081
444.799995	284.179408	6.41353E-06	0.00285274
444.839995	297.721728	6.71916E-06	0.002988953
444.879995	385.494379	8.70007E-06	0.003870488
444.919995	637.573523	1.43891E-05	0.006402019
444.959995	1212.602959	2.73668E-05	0.012177113
444.999995	2198.626663	4.96199E-05	0.022080874
445.039995	3681.085046	8.3077E-05	0.036972571
445.079995	5406.878359	0.000122026	0.054311204
445.119995	6771.887614	0.000152832	0.068028611
445.159995	7364.705006	0.000166211	0.073990547
445.199995	7269.731781	0.000164068	0.073042948
445.239995	6451.893548	0.00014561	0.064831507
445.279995	5042.440331	0.000113801	0.050673244
445.319995	3449.506452	7.78506E-05	0.034668409
445.359995	2065.283419	4.66106E-05	0.020758483
445.399995	1130.416008	2.55119E-05	0.011363007
445.439995	595.961258	1.345E-05	0.005991175
445.479995	378.274094	8.53712E-06	0.003803116
445.519995	328.725749	7.41888E-06	0.00330526
445.559995	359.452376	8.11234E-06	0.003614534
445.599995	478.823444	1.08064E-05	0.004815322
445.639995	529.837523	1.19577E-05	0.005328827
445.679995	491.994321	1.11036E-05	0.004948664
445.719995	413.509399	9.33233E-06	0.004159607
445.759995	329.638081	7.43947E-06	0.003316219
445.799995	270.034562	6.0943E-06	0.002716841
445.839995	257.680792	5.8155E-06	0.002592781
445.879995	284.076819	6.41122E-06	0.002858634
445.919995	395.742968	8.93137E-06	0.003982675
445.959995	665.08011	1.50099E-05	0.006693829
445.999995	1170.119592	2.6408E-05	0.011777954
446.039995	1944.981617	4.38955E-05	0.01957916
446.079994	2851.262887	6.4349E-05	0.028704816
446.119994	3728.016522	8.41361E-05	0.037534814
446.159994	4373.978472	9.87146E-05	0.044042506
446.199994	4536.472598	0.000102382	0.045682789
446.239994	4108.380516	9.27204E-05	0.041375562

446.279994	3273.113778	7.38696E-05	0.032966533
446.319994	2293.951094	5.17713E-05	0.023106558
446.359994	1429.125923	3.22534E-05	0.014396619
446.399994	810.857923	1.82999E-05	0.00816909
446.439994	486.233625	1.09736E-05	0.004899061
446.479994	360.87367	8.14442E-06	0.003636319
446.519994	344.015738	7.76396E-06	0.003466762
446.559994	393.033096	8.87021E-06	0.003961081
446.599994	499.228502	1.12669E-05	0.005031794
446.639994	566.28744	1.27803E-05	0.005708201
446.679994	551.542514	1.24475E-05	0.00556007
446.719994	509.375051	1.14959E-05	0.005135442
446.759994	451.627359	1.01926E-05	0.004553646
446.799994	389.919111	8.79993E-06	0.003931809
446.839994	437.499249	9.87375E-06	0.004411986
446.879994	813.580224	1.83614E-05	0.00820533
446.919994	2028.745547	4.5786E-05	0.020462661
446.959994	4948.778737	0.000111687	0.04991964
446.999994	10163.30086	0.000229372	0.102529079
447.039994	17248.43449	0.000389273	0.174020663
447.079994	24246.80643	0.000547217	0.244649616
447.119994	28280.57762	0.000638253	0.285375787
447.159994	27391.54395	0.000618189	0.276429387
447.199994	22580.44264	0.000509609	0.227897204
447.239994	16150.40229	0.000364492	0.163015438
447.279994	10317.34866	0.000232848	0.104148336
447.319994	5986.153718	0.000135099	0.060432552
447.359994	3200.527506	7.22314E-05	0.03231346
447.399994	1591.635358	3.5921E-05	0.016071052
447.439994	776.932883	1.75343E-05	0.007845544
447.479994	418.523298	9.44549E-06	0.004226667
447.519994	348.273513	7.86005E-06	0.003517529
447.559994	416.879974	9.4084E-06	0.004210824
447.599994	471.171776	1.06337E-05	0.00475964
447.639994	524.822742	1.18445E-05	0.00530208
447.679994	544.741151	1.2294E-05	0.0055038
447.719994	524.294847	1.18326E-05	0.005297694
447.759994	474.483336	1.07084E-05	0.004794806
447.799994	445.273196	1.00492E-05	0.00450003
447.839994	399.468685	9.01545E-06	0.00403748
447.879994	424.113787	9.57166E-06	0.004286954
447.919994	678.067691	1.5303E-05	0.006854539
447.959994	1341.048843	3.02656E-05	0.01355778
447.999994	2728.853351	6.15864E-05	0.027590715
448.039994	4896.178307	0.0001105	0.049508386
448.079994	7197.159798	0.00016243	0.072781576

448.119994	8751.480976	0.000197509	0.088507614
448.159994	8753.595146	0.000197556	0.088536897
448.199994	7655.823787	0.000172781	0.077440568
448.239994	5952.008571	0.000134329	0.060211425
448.279994	4152.301621	9.37117E-05	0.042009064
448.319994	2640.873848	5.96008E-05	0.02672025
448.359994	1567.403312	3.53741E-05	0.015860336
448.399994	916.742001	2.06896E-05	0.009277212
448.439994	546.399703	1.23315E-05	0.005529929
448.479994	394.954109	8.91356E-06	0.003997555
448.519994	397.259724	8.9656E-06	0.00402125
448.559994	465.645792	1.0509E-05	0.004713907
448.599994	534.227397	1.20568E-05	0.005408666
448.639994	582.865144	1.31545E-05	0.005901614
448.679994	656.067986	1.48065E-05	0.006643399
448.719994	739.894711	1.66984E-05	0.007492903
448.759994	803.893455	1.81428E-05	0.008141743
448.799994	1006.024026	2.27046E-05	0.010189807
448.839994	1415.230078	3.19398E-05	0.014335847
448.879994	2099.715379	4.73877E-05	0.021271369
448.919994	3592.056584	8.10677E-05	0.036392918
448.959994	6888.276863	0.000155459	0.069794788
448.999994	12415.39767	0.000280198	0.125809005
449.039994	19888.92314	0.000448865	0.201558468
449.079994	27267.32777	0.000615386	0.276357365
449.119994	31250.97236	0.000705291	0.316760252
449.159994	29890.24027	0.000674581	0.302994838
449.199994	24032.15842	0.000542372	0.243633653
449.239994	16597.76364	0.000374588	0.168280094
449.279994	10104.90825	0.000228054	0.102459967
449.319994	5610.947325	0.000126631	0.05689796
449.359994	2916.623059	6.58241E-05	0.029578726
449.399994	1423.552869	3.21276E-05	0.014438146
449.439994	687.855134	1.55239E-05	0.006977076
449.479994	396.69793	8.95292E-06	0.004024158
449.519994	337.746015	7.62246E-06	0.003426447
449.559994	388.153643	8.76009E-06	0.003938185
449.599994	491.60347	1.10948E-05	0.004988225
449.639994	529.877798	1.19586E-05	0.005377067
449.679994	542.752931	1.22492E-05	0.00550821
449.719994	503.590296	1.13653E-05	0.005111217
449.759994	412.736318	9.31488E-06	0.004189462
449.799994	368.803068	8.32337E-06	0.003743852
449.839994	435.410026	9.8266E-06	0.004420397
449.879994	589.358266	1.3301E-05	0.005983852
449.919994	919.54862	2.07529E-05	0.009337159

449.959994	1693.009873	3.82089E-05	0.017192465
449.999994	3214.288801	7.2542E-05	0.032643909
450.039994	5473.751891	0.000123535	0.055595671
450.079994	7762.226698	0.000175183	0.078846204
450.119994	9287.608757	0.000209608	0.094348929
450.159994	9501.522442	0.000214436	0.096530566
450.199994	8602.058604	0.000194136	0.087400243
450.239994	7004.410204	0.00015808	0.07117384
450.279994	5251.704263	0.000118524	0.053368828
450.319994	3590.476511	8.10321E-05	0.036490354
450.359994	2109.599149	4.76107E-05	0.021441961
450.399994	1075.492595	2.42724E-05	0.010932275
450.439994	559.221369	1.26208E-05	0.005684934
450.479994	373.648237	8.43272E-06	0.003798772
450.519994	346.796817	7.82672E-06	0.003526094
450.559994	429.203235	9.68652E-06	0.004364358
450.599994	531.781526	1.20016E-05	0.005407907
450.639994	545.477128	1.23107E-05	0.005547675
450.679994	574.771434	1.29718E-05	0.005846127
450.719994	683.556597	1.54269E-05	0.006953221
450.759994	798.694602	1.80254E-05	0.00812514
450.799994	848.508337	1.91497E-05	0.008632663
450.839994	846.242381	1.90985E-05	0.008610373
450.879994	841.057712	1.89815E-05	0.008558379
450.919994	973.102377	2.19616E-05	0.009902909
450.959994	1447.34911	3.26647E-05	0.014730452
450.999994	2403.254091	5.42381E-05	0.024461382
451.039994	3931.438844	8.87271E-05	0.04001947
451.079994	5830.311238	0.000131582	0.059354009
451.119994	7381.392458	0.000166588	0.075151063
451.159994	8024.150281	0.000181094	0.081702321
451.199994	7809.849791	0.000176257	0.079527352
451.239994	6799.502816	0.000153455	0.069245172
451.279994	5336.902085	0.000120446	0.054355072
451.319994	3795.657635	8.56627E-05	0.038661292
451.359994	2390.642415	5.39535E-05	0.024352438
451.399994	1357.882947	3.06455E-05	0.013833391
451.439994	763.658605	1.72347E-05	0.007780438
451.479994	460.350732	1.03895E-05	0.00469064
451.519994	363.019533	8.19285E-06	0.003699233
451.559994	404.072321	9.11935E-06	0.004117933
451.599994	477.011754	1.07655E-05	0.004861696
451.639994	514.68957	1.16158E-05	0.005246172
451.679994	523.842136	1.18224E-05	0.005339936
451.719994	499.806851	1.12799E-05	0.005095376
451.759994	430.974181	9.72649E-06	0.004394038

451.799994	359.43877	8.11203E-06	0.003665016
451.839994	308.238424	6.95651E-06	0.00314323
451.879994	331.579269	7.48328E-06	0.003381546
451.919994	398.788407	9.0001E-06	0.004067325
451.959994	570.624419	1.28782E-05	0.00582043
451.999994	959.58644	2.16565E-05	0.009788751
452.039994	1609.949059	3.63343E-05	0.01642456
452.079994	2480.727222	5.59866E-05	0.025310403
452.119994	3364.85304	7.594E-05	0.034334013
452.159994	4003.312009	9.03492E-05	0.040852283
452.199994	4249.882309	9.59139E-05	0.043372276
452.239994	4004.819505	9.03832E-05	0.040874897
452.279994	3246.00026	7.32577E-05	0.033132994
452.319994	2281.212602	5.14838E-05	0.023287148
452.359994	1445.458597	3.2622E-05	0.014756882
452.399994	858.147084	1.93672E-05	0.008761714
452.439994	522.87962	1.18007E-05	0.005339093
452.479994	352.804798	7.96231E-06	0.003602787
452.519994	327.784624	7.39764E-06	0.003347581
452.559994	411.272531	9.28185E-06	0.004200593
452.599994	502.017352	1.13298E-05	0.005127882
452.639994	553.090261	1.24825E-05	0.005650068
452.679994	536.128419	1.20997E-05	0.005477279
452.719994	495.296176	1.11781E-05	0.00506057
452.759994	436.505306	9.85132E-06	0.004460282
452.799994	384.486914	8.67733E-06	0.003929097
452.839994	374.257674	8.44647E-06	0.003824901
452.879994	438.917654	9.90576E-06	0.004486121
452.919994	764.408207	1.72516E-05	0.007813607
452.959994	1525.333975	3.44247E-05	0.015592995
452.999994	2838.211335	6.40545E-05	0.029016677
453.039994	4773.668648	0.000107735	0.048808287
453.079994	7076.88906	0.000159716	0.072363911
453.119994	9193.430801	0.000207483	0.094014663
453.159994	10458.60377	0.000236036	0.106962126
453.199994	10593.88702	0.000239089	0.108355258
453.239994	9734.468903	0.000219693	0.099573837
453.279994	8202.030702	0.000185108	0.083905938
453.319994	6358.672727	0.000143506	0.065054314
453.359994	4434.885439	0.000100089	0.045376433
453.399994	2812.195298	6.34673E-05	0.028776087
453.439994	1622.801117	3.66244E-05	0.01660695
453.479994	911.761588	2.05772E-05	0.009331344
453.519994	614.379359	1.38657E-05	0.006288366
453.559994	540.54753	1.21994E-05	0.005533162
453.599994	527.385077	1.19023E-05	0.005398904

453.639994	533.116423	1.20317E-05	0.005458058
453.679994	512.585715	1.15683E-05	0.005248327
453.719994	437.074167	9.86416E-06	0.004475564
453.759994	359.557305	8.11471E-06	0.00368213
453.799994	335.24544	7.56602E-06	0.003433461
453.839994	341.395848	7.70483E-06	0.003496759
453.879994	351.058898	7.92291E-06	0.00359605
453.919994	435.071199	9.81895E-06	0.004457018
453.959994	647.520336	1.46136E-05	0.006634004
453.999994	1096.55032	2.47476E-05	0.011235416
454.039994	1944.816399	4.38918E-05	0.019928631
454.079994	3183.183599	7.184E-05	0.032621116
454.119994	4458.541683	0.000100623	0.04569495
454.159994	5497.650941	0.000124074	0.056349594
454.199994	6129.434002	0.000138333	0.062830751
454.239994	6079.789598	0.000137212	0.062327352
454.279994	5278.093242	0.000119119	0.054113475
454.319994	3997.888814	9.02268E-05	0.040991831
454.359994	2726.825093	6.15406E-05	0.027961607
454.399994	1696.693742	3.8292E-05	0.017399891
454.439994	963.563786	2.17463E-05	0.009882385
454.479994	573.261535	1.29377E-05	0.005879932
454.519994	436.869547	9.85954E-06	0.004481357
454.559994	443.915394	1.00186E-05	0.004554033
454.599994	474.105408	1.06999E-05	0.004864174
454.639994	510.816881	1.15284E-05	0.005241283
454.679994	518.177523	1.16945E-05	0.005317276
454.719994	444.796522	1.00384E-05	0.004564678
454.759994	380.960977	8.59776E-06	0.003909917
454.799994	355.106861	8.01427E-06	0.003644889
454.839994	366.753479	8.27712E-06	0.003764763
454.879994	411.008181	9.27588E-06	0.004219413
454.919994	495.826386	1.11901E-05	0.005090605
454.959994	797.52772	1.79991E-05	0.008188866
454.999994	1431.660209	3.23106E-05	0.014701312
455.039994	2445.513602	5.51918E-05	0.025114493
455.079994	3813.524528	8.60659E-05	0.039166886
455.119994	5135.516044	0.000115901	0.052749064
455.159994	6035.084545	0.000136203	0.061994362
455.199994	6370.74386	0.000143779	0.065448116
455.239994	6070.564846	0.000137004	0.062369788
455.279994	5369.78618	0.000121189	0.055174743
455.319994	4460.573613	0.000100669	0.045836578
455.359994	3443.617765	7.77177E-05	0.035389512
455.399994	2435.662099	5.49695E-05	0.02503311
455.439994	1558.74879	3.51788E-05	0.016021827

455.479994	928.630771	2.09579E-05	0.009545906
455.519994	590.727845	1.33319E-05	0.006072949
455.559994	509.811132	1.15057E-05	0.005241549
455.599994	535.95987	1.20959E-05	0.005510877
455.639994	575.206598	1.29816E-05	0.005914941
455.679994	576.306158	1.30064E-05	0.005926769
455.719994	527.064975	1.18951E-05	0.005420845
455.759994	451.625299	1.01926E-05	0.004645358
455.799994	374.650774	8.45535E-06	0.003853947
455.839994	351.499216	7.93285E-06	0.003616109
455.879994	372.769856	8.4129E-06	0.003835271
455.919994	421.652712	9.51611E-06	0.004338587
455.959994	566.472141	1.27845E-05	0.005829215
455.999994	931.078733	2.10132E-05	0.009581996
456.039994	1612.594811	3.6394E-05	0.016597128
456.079994	2586.963764	5.83842E-05	0.026627851
456.119994	3557.717866	8.02927E-05	0.036623123
456.159994	4301.285951	9.7074E-05	0.04428129
456.199994	4684.964543	0.000105733	0.048235451
456.239994	4514.921741	0.000101895	0.0464888
456.279994	3920.50885	8.84804E-05	0.040371847
456.319994	3096.311268	6.98794E-05	0.031887382
456.359994	2183.422472	4.92768E-05	0.022487962
456.399994	1364.665531	3.07986E-05	0.014056481
456.439994	831.439625	1.87644E-05	0.008564838
456.479994	540.930382	1.2208E-05	0.005572728
456.519994	411.262154	9.28161E-06	0.004237242
456.559994	405.49589	9.15148E-06	0.004178198
456.599994	464.082471	1.04737E-05	0.004782289
456.639994	528.039507	1.19171E-05	0.005441832
456.679994	523.68384	1.18188E-05	0.005397416
456.719994	494.420653	1.11584E-05	0.005096258
456.759994	439.316699	9.91477E-06	0.004528668
456.799994	389.055263	8.78044E-06	0.004010903
456.839994	372.293046	8.40214E-06	0.003838432
456.879994	446.298391	1.00723E-05	0.004601847
456.919994	667.997126	1.50758E-05	0.006888418
456.959994	1112.541378	2.51085E-05	0.011473584
456.999994	1894.046251	4.2746E-05	0.019534915
457.039994	3039.709177	6.8602E-05	0.031353859
457.079994	4313.075248	9.73401E-05	0.044492213
457.119994	5471.280539	0.000123479	0.056444802
457.159994	6164.76912	0.00013913	0.063604785
457.199994	6211.922876	0.000140194	0.0640969
457.239994	5704.772709	0.000128749	0.058869089
457.279994	4701.194681	0.000106099	0.04851714

457.319994	3417.829164	7.71356E-05	0.035275672
457.359994	2269.991211	5.12305E-05	0.023430799
457.399994	1411.336475	3.18519E-05	0.014569058
457.439994	820.034037	1.8507E-05	0.008465854
457.479994	501.01928	1.13073E-05	0.005172867
457.519994	392.22917	8.85207E-06	0.004049997
457.559994	402.816895	9.09102E-06	0.004159685
457.599994	447.138643	1.00913E-05	0.004617777
457.639994	476.777552	1.07602E-05	0.0049243
457.679994	517.171793	1.16718E-05	0.005341971
457.719994	488.475779	1.10242E-05	0.005046005
457.759994	397.559866	8.97237E-06	0.004107193
457.799994	319.491416	7.21048E-06	0.003300956
457.839994	312.556832	7.05397E-06	0.003229591
457.879994	348.715626	7.87003E-06	0.003603527
457.919994	417.595657	9.42455E-06	0.004315691
457.959994	638.134551	1.44018E-05	0.006595452
457.999994	1066.797096	2.40761E-05	0.011026865
458.039994	1714.010035	3.86828E-05	0.017718278
458.079994	2519.041444	5.68513E-05	0.026042423
458.119994	3286.563169	7.41732E-05	0.033980204
458.159994	3793.041541	8.56037E-05	0.039220174
458.199994	3918.076635	8.84255E-05	0.040516578
458.239994	3686.965113	8.32097E-05	0.038129997
458.279994	3093.463066	6.98152E-05	0.031994888
458.319994	2357.732547	5.32107E-05	0.024387546
458.359994	1602.346149	3.61627E-05	0.016575544
458.399994	1005.567503	2.26943E-05	0.010403048
458.439994	603.196581	1.36133E-05	0.006240884
458.479994	414.046667	9.34446E-06	0.004284246
458.519994	383.382306	8.6524E-06	0.003967301
458.559994	414.154155	9.34688E-06	0.004286106
458.599994	442.125166	9.97815E-06	0.004575979
458.639994	480.200059	1.08374E-05	0.004970486
458.679994	503.032811	1.13527E-05	0.005207279
458.719994	530.687038	1.19769E-05	0.005494028
458.759994	545.312514	1.23069E-05	0.005645934
458.799994	487.084591	1.09928E-05	0.005043506
458.839994	433.886397	9.79221E-06	0.004493058
458.879994	448.077005	1.01125E-05	0.004640412
458.919994	584.395428	1.3189E-05	0.006052692
458.959994	911.23255	2.05652E-05	0.009438627
458.999994	1607.220947	3.62727E-05	0.016649187
459.039994	2647.653594	5.97539E-05	0.027429409
459.079994	3678.883969	8.30273E-05	0.038116166
459.119994	4552.709322	0.000102748	0.047173803

459.159994	5082.198631	0.000114698	0.052664799
459.199994	5175.770267	0.00011681	0.053639117
459.239994	4742.498653	0.000107032	0.049153186
459.279994	3957.203143	8.93086E-05	0.041017636
459.319994	3018.398807	6.81211E-05	0.031289363
459.359994	2038.59674	4.60083E-05	0.021134367
459.399994	1216.978098	2.74655E-05	0.012617651
459.439994	702.832092	1.58619E-05	0.00728761
459.479994	440.103942	9.93253E-06	0.0045638
459.519994	366.344579	8.26789E-06	0.003799259
459.559994	406.472928	9.17353E-06	0.004215786
459.599994	460.124575	1.03844E-05	0.004772657
459.639994	501.838411	1.13258E-05	0.005205788
459.679994	487.302133	1.09977E-05	0.005055437
459.719994	424.679513	9.58443E-06	0.004406152
459.759994	362.451792	8.18003E-06	0.003760852
459.799994	319.88181	7.21929E-06	0.003319428
459.839994	297.146758	6.70619E-06	0.003083774
459.879994	289.54955	6.53473E-06	0.003005192
459.919994	375.439856	8.47315E-06	0.003896973
459.959994	582.508235	1.31464E-05	0.006046818
459.999994	1016.458938	2.29401E-05	0.010552429
460.039994	1737.760653	3.92188E-05	0.018042234
460.079994	2543.770607	5.74094E-05	0.026412898
460.119994	3210.235123	7.24505E-05	0.03333594
460.159994	3672.203873	8.28765E-05	0.038136462
460.199994	3778.164272	8.52679E-05	0.039240289
460.239994	3531.384375	7.96984E-05	0.036680403
460.279994	2963.900223	6.68911E-05	0.030788635
460.319994	2175.998027	4.91092E-05	0.022605967
460.359994	1438.272337	3.24598E-05	0.014943195
460.399994	904.580987	2.04151E-05	0.009399127
460.439994	552.819386	1.24764E-05	0.005744617
460.479994	380.231547	8.5813E-06	0.003951515
460.519994	345.075859	7.78788E-06	0.003586475
460.559994	389.109057	8.78165E-06	0.004044476
460.599994	458.778407	1.0354E-05	0.004769048
460.639994	518.293773	1.16972E-05	0.005388184
460.679994	507.066038	1.14438E-05	0.005271918
460.719994	460.757742	1.03987E-05	0.004790871
460.759994	410.566124	9.26591E-06	0.004269359
460.799994	383.162766	8.64745E-06	0.003984745
460.839994	408.091832	9.21006E-06	0.004244366
460.879994	526.671432	1.18862E-05	0.005478131
460.919994	872.156254	1.96834E-05	0.00907245
460.959994	1634.278448	3.68834E-05	0.017001766

460.999994	2946.725042	6.65035E-05	0.030658104
461.039994	4820.659153	0.000108796	0.050159105
461.079994	6755.970738	0.000152473	0.07030218
461.119994	8005.869417	0.000180681	0.08331577
461.159994	8246.268221	0.000186107	0.085825005
461.199994	7751.30658	0.000174936	0.080680571
461.239994	6685.091117	0.000150873	0.069588752
461.279994	5347.32914	0.000120682	0.055668086
461.319994	3880.824307	8.75848E-05	0.040404619
461.359994	2531.627703	5.71353E-05	0.026359947
461.399994	1480.40857	3.34108E-05	0.015415724
461.439994	820.971152	1.85282E-05	0.008549641
461.479994	502.54081	1.13416E-05	0.005233943
461.519994	395.296172	8.92128E-06	0.004117351
461.559994	414.031226	9.34411E-06	0.004312866
461.599994	489.401519	1.10451E-05	0.005098423
461.639994	521.768671	1.17756E-05	0.005436084
461.679994	501.271193	1.1313E-05	0.005222982
461.719994	472.561109	1.0665E-05	0.004924265
461.759994	437.040515	9.8634E-06	0.004554522
461.799994	379.650926	8.56819E-06	0.003956791
461.839994	318.329343	7.18425E-06	0.003317974
461.879994	328.894188	7.42268E-06	0.003428389
461.919994	446.539233	1.00778E-05	0.004655123
461.959994	750.76622	1.69437E-05	0.007827334
461.999994	1378.089857	3.11016E-05	0.014368924
462.039994	2230.412251	5.03373E-05	0.023257845
462.079994	3130.531956	7.06517E-05	0.032646758
462.119994	3855.338823	8.70096E-05	0.040208888
462.159994	4215.09876	9.51289E-05	0.043964775
462.199994	4129.014671	9.31861E-05	0.043070619
462.239994	3658.776048	8.25735E-05	0.038168765
462.279994	2948.58466	6.65454E-05	0.03076263
462.319994	2201.052091	4.96747E-05	0.022965598
462.359994	1490.249074	3.36328E-05	0.015550482
462.399994	940.012346	2.12148E-05	0.009809709
462.439994	603.340897	1.36166E-05	0.006296844
462.479994	451.743722	1.01952E-05	0.004715088
462.519994	451.873834	1.01982E-05	0.004716854
462.559994	523.123194	1.18062E-05	0.005461058
462.599994	647.122492	1.46047E-05	0.006756113
462.639994	737.223877	1.66381E-05	0.007697458
462.679994	813.985288	1.83705E-05	0.008499669
462.719994	897.845316	2.02631E-05	0.00937615
462.759994	1097.690134	2.47733E-05	0.011464109
462.799994	1826.029744	4.12109E-05	0.019072426

462.839994	4539.815859	0.000102457	0.047421346
462.879994	13658.76417	0.000308259	0.142687055
462.919994	38103.44369	0.000859942	0.398084184
462.959994	90132.19683	0.002034158	0.94173384
462.999994	171982.9516	0.003881416	1.79709543
463.039994	264544.373	0.005970398	2.764533063
463.079994	327364.9195	0.00738817	3.421313836
463.119994	329299.4732	0.00743183	3.441829266
463.159994	270681.6937	0.006108909	2.829402162
463.199994	184406.8349	0.004161805	1.927748239
463.239994	105345.9523	0.002377511	1.101358371
463.279994	51605.49301	0.001164664	0.539565576
463.319994	22122.98784	0.000499285	0.231328733
463.359994	8709.555527	0.000196563	0.091079216
463.399994	3298.323519	7.44386E-05	0.034494832
463.439994	1323.052209	2.98594E-05	0.013838061
463.479994	658.060723	1.48515E-05	0.00688338
463.519994	480.346511	1.08408E-05	0.005024905
463.559994	474.847475	1.07166E-05	0.004967808
463.599994	503.133758	1.1355E-05	0.005264191
463.639994	537.359816	1.21275E-05	0.005622777
463.679994	526.015772	1.18714E-05	0.005504551
463.719994	466.715033	1.05331E-05	0.004884413
463.759994	416.633273	9.40283E-06	0.004360658
463.799994	488.426746	1.10231E-05	0.005112519
463.839994	1075.454985	2.42715E-05	0.011258102
463.879994	3058.073986	6.90165E-05	0.03201536
463.919994	8332.578459	0.000188055	0.08724233
463.959994	20033.19996	0.000452121	0.209766251
463.999994	39553.71434	0.000892672	0.414199914
464.039994	63066.07342	0.001423313	0.660474357
464.079994	80121.9031	0.00180824	0.839167893
464.119994	81525.75126	0.001839923	0.853944889
464.159994	67104.70054	0.00151446	0.702951569
464.199994	45785.72782	0.00103332	0.479667185
464.239994	26503.4537	0.000598146	0.277683311
464.279994	13460.81521	0.000303792	0.141044478
464.319994	6218.408845	0.000140341	0.065163052
464.359994	2719.345673	6.13718E-05	0.028498629
464.399994	1225.63109	2.76608E-05	0.012845669
464.439994	692.53993	1.56297E-05	0.00725904
464.479994	501.01339	1.13072E-05	0.005251956
464.519994	464.96896	1.04937E-05	0.004874534
464.559994	528.202117	1.19208E-05	0.00553792
464.599994	665.894539	1.50283E-05	0.006982154
464.639994	802.599859	1.81136E-05	0.008416285

464.679994	892.956195	2.01528E-05	0.009364593
464.719994	956.88261	2.15955E-05	0.010035864
464.759994	1108.556391	2.50186E-05	0.011627632
464.799994	1399.77567	3.1591E-05	0.014683491
464.839994	1989.851847	4.49082E-05	0.02087512
464.879994	3369.479731	7.60445E-05	0.03535155
464.919994	6720.835047	0.00015168	0.070519012
464.959994	13332.23887	0.00030089	0.139901841
464.999994	23532.49912	0.000531096	0.24695951
465.039994	35419.51635	0.000799369	0.371738624
465.079994	44189.64689	0.000997299	0.46382371
465.119994	45236.8647	0.001020933	0.474856363
465.159994	38226.69421	0.000862723	0.401304339
465.199994	27295.12768	0.000616013	0.28656925
465.239994	17018.4993	0.000384084	0.178691156
465.279994	9627.378483	0.000217277	0.101094419
465.319994	5020.7276	0.000113311	0.052725794
465.359994	2483.871546	5.60575E-05	0.026086928
465.399994	1212.022328	2.73537E-05	0.012730391
465.439994	654.110943	1.47624E-05	0.006870999
465.479994	442.635015	9.98966E-06	0.004649985
465.519994	385.296189	8.6956E-06	0.004047975
465.559994	422.630382	9.53818E-06	0.004440595
465.599994	485.454015	1.0956E-05	0.005101123
465.639994	555.112271	1.25281E-05	0.005833589
465.679994	581.509746	1.31239E-05	0.006111521
465.719994	564.183448	1.27328E-05	0.005929936
465.759994	518.702297	1.17064E-05	0.005452367
465.799994	486.771944	1.09858E-05	0.005117169
465.839994	523.819723	1.18219E-05	0.005507105
465.879994	693.533947	1.56521E-05	0.007291998
465.919994	1231.082576	2.77838E-05	0.012945037
465.959994	2470.964085	5.57662E-05	0.025984826
465.999994	4636.1729	0.000104632	0.048758495
466.039994	7559.935782	0.000170617	0.079514448
466.079994	10261.11216	0.000231579	0.107934343
466.119994	11425.81825	0.000257865	0.12019594
466.159994	10728.32001	0.000242123	0.112868168
466.199994	8681.202465	0.000195923	0.091339137
466.239994	6235.792849	0.000140733	0.065615427
466.279994	4062.658281	9.16885E-05	0.042752531
466.319994	2452.391236	5.53471E-05	0.025809438
466.359994	1401.408984	3.16278E-05	0.014749963
466.399994	767.158002	1.73137E-05	0.008075104
466.439994	447.054143	1.00894E-05	0.004706095
466.479994	332.68193	7.50817E-06	0.00350241

466.519994	325.483703	7.34571E-06	0.003426922
466.559994	381.651533	8.61334E-06	0.004018641
466.599994	463.990935	1.04716E-05	0.004886062
466.639994	493.952258	1.11478E-05	0.005202016
466.679994	505.490695	1.14082E-05	0.005323988
466.719994	478.354251	1.07958E-05	0.005038611
466.759994	412.314895	9.30537E-06	0.004343376
466.799994	341.455804	7.70618E-06	0.003597246
466.839994	328.684842	7.41796E-06	0.003463
466.879994	435.49797	9.82858E-06	0.004588769
466.919994	717.73555	1.61983E-05	0.007563306
466.959994	1340.850113	3.02611E-05	0.014130732
466.999994	2486.338676	5.61132E-05	0.026204864
467.039994	4122.7188	9.3044E-05	0.043455279
467.079994	5790.783191	0.00013069	0.061042643
467.119994	7060.721029	0.000159351	0.074435869
467.159994	7604.42341	0.000171621	0.080174579
467.199994	7286.48474	0.000164446	0.076829082
467.239994	6301.481533	0.000142216	0.066448845
467.279994	4949.932023	0.000111713	0.052201283
467.319994	3496.508666	7.89113E-05	0.036876842
467.359994	2233.310085	5.04027E-05	0.023556205
467.399994	1269.422236	2.86491E-05	0.013390585
467.439994	683.383959	1.5423E-05	0.007209338
467.479994	420.730131	9.49529E-06	0.00443886
467.519994	355.393405	8.02073E-06	0.003749854
467.559994	392.770242	8.86428E-06	0.004144581
467.599994	464.296013	1.04785E-05	0.004899753
467.639994	505.443459	1.14072E-05	0.005334442
467.679994	513.220924	1.15827E-05	0.005416988
467.719994	462.44203	1.04367E-05	0.00488144
467.759994	382.337048	8.62881E-06	0.004036214
467.799994	311.203413	7.02343E-06	0.003285559
467.839994	283.230433	6.39212E-06	0.002990488
467.879994	306.718818	6.92222E-06	0.003238767
467.919994	380.319648	8.58328E-06	0.004016291
467.959994	589.113221	1.32955E-05	0.006221746
467.999994	1040.674923	2.34866E-05	0.010991721
468.039994	1737.250968	3.92073E-05	0.018350601
468.079994	2668.700034	6.02288E-05	0.028191916
468.119994	3609.215456	8.1455E-05	0.038130698
468.159994	4257.897881	9.60948E-05	0.044987752
468.199994	4419.100688	9.97329E-05	0.046694965
468.239994	4043.610644	9.12587E-05	0.042730954
468.279994	3272.986544	7.38667E-05	0.03459032
468.319994	2354.704945	5.31424E-05	0.024887653

468.359994	1549.588061	3.4972E-05	0.016379506
468.399994	944.090963	2.13068E-05	0.009980113
468.439994	556.45076	1.25583E-05	0.005882819
468.479994	376.348022	8.49365E-06	0.003979105
468.519994	331.425722	7.47982E-06	0.003504444
468.559994	368.291356	8.31182E-06	0.003894588
468.599994	442.818669	9.9938E-06	0.004683095
468.639994	506.450693	1.14299E-05	0.005356502
468.679994	509.085234	1.14893E-05	0.005384826
468.719994	459.598773	1.03725E-05	0.0048618
468.759994	378.574006	8.54389E-06	0.004005033
468.799994	325.436291	7.34464E-06	0.003443169
468.839994	293.734709	6.62918E-06	0.003108026
468.879994	322.650574	7.28177E-06	0.003414278
468.919994	448.218067	1.01157E-05	0.004743434
468.959994	804.031053	1.81459E-05	0.008509683
468.999994	1531.09125	3.45546E-05	0.016206106
469.039994	2613.073547	5.89734E-05	0.027660897
469.079994	3930.715769	8.87108E-05	0.041612452
469.119994	5166.838661	0.000116608	0.054703309
469.159994	5976.603482	0.000134884	0.063281996
469.199994	6230.329662	0.00014061	0.065974147
469.239994	6020.1167	0.000135866	0.063753596
469.279994	5244.12216	0.000118353	0.055540476
469.319994	4062.03797	9.16745E-05	0.043024694
469.359994	2779.708989	6.27342E-05	0.029444905
469.399994	1700.299084	3.83734E-05	0.018012465
469.439994	987.983343	2.22974E-05	0.010467295
469.479994	581.522068	1.31241E-05	0.006161523
469.519994	395.934771	8.9357E-06	0.004195488
469.559994	400.140409	9.03061E-06	0.004240414
469.599994	494.2406	1.11543E-05	0.00523807
469.639994	544.107919	1.22798E-05	0.005767065
469.679994	577.844623	1.30411E-05	0.006125166
469.719994	628.248683	1.41787E-05	0.006660018
469.759994	615.492784	1.38908E-05	0.006525349
469.799994	543.01121	1.2255E-05	0.005757402
469.839994	442.031724	9.97604E-06	0.004687143
469.879994	365.736787	8.25417E-06	0.003878469
469.919994	370.136475	8.35346E-06	0.00392546
469.959994	569.027835	1.28422E-05	0.006035304
469.999994	991.06066	2.23669E-05	0.010512423
470.039994	1620.444683	3.65712E-05	0.017189917
470.079994	2500.408593	5.64307E-05	0.026526962
470.119994	3468.324757	7.82753E-05	0.036798764
470.159994	4241.332917	9.5721E-05	0.045004174

470.199994	4578.567157	0.000103332	0.048586651
470.239994	4484.463292	0.000101208	0.047592092
470.279994	3884.813492	8.76748E-05	0.041231718
470.319994	2959.37979	6.67891E-05	0.03141224
470.359994	2020.70789	4.56046E-05	0.021450562
470.399994	1230.131115	2.77623E-05	0.013059407
470.439994	712.271775	1.6075E-05	0.007562314
470.479994	482.882811	1.0898E-05	0.005127287
470.519994	428.825249	9.67799E-06	0.004553687
470.559994	444.808387	1.00387E-05	0.004723813
470.599994	524.798402	1.1844E-05	0.005573772
470.639994	547.193719	1.23494E-05	0.005812122
470.679994	516.691679	1.1661E-05	0.005488605
470.719994	480.594498	1.08463E-05	0.005105593
470.759994	470.259328	1.06131E-05	0.004996222
470.799994	455.160963	1.02723E-05	0.004836222
470.839994	443.77093	1.00153E-05	0.0047156
470.879994	498.275192	1.12454E-05	0.005295223
470.919994	716.225134	1.61642E-05	0.007612047
470.959994	1185.302562	2.67506E-05	0.012598476
470.999994	2037.938242	4.59934E-05	0.021662904
471.039994	3310.88307	7.4722E-05	0.035197059
471.079994	4882.789	0.000110198	0.051911954
471.119994	6281.127906	0.000141756	0.066784231
471.159994	7093.4078	0.000160088	0.075427218
471.199994	7137.297514	0.000161079	0.075900359
471.239994	6518.058999	0.000147104	0.069321058
471.279994	5490.236878	0.000123907	0.05839489
471.319994	4256.330185	9.60594E-05	0.045274736
471.359994	3064.421707	6.91597E-05	0.03259913
471.399994	2028.784518	4.57868E-05	0.021583916
471.439994	1224.057335	2.76253E-05	0.013023657
471.479994	709.975785	1.60232E-05	0.007554602
471.519994	471.292051	1.06364E-05	0.005015278
471.559994	409.82663	9.24922E-06	0.00436156
471.599994	426.40752	9.62342E-06	0.004538407
471.639994	453.005671	1.02237E-05	0.004821909
471.679994	464.023441	1.04724E-05	0.004939604
471.719994	443.22201	1.00029E-05	0.00471857
471.759994	384.877272	8.68614E-06	0.004097775
471.799994	323.409522	7.2989E-06	0.003443622
471.839994	300.172445	6.77447E-06	0.003196468
471.879994	339.052326	7.65194E-06	0.003610797
471.919994	442.321803	9.98259E-06	0.004710982
471.959994	626.207481	1.41326E-05	0.006670036
471.999994	1024.247079	2.31158E-05	0.010910672

472.039994	1746.205575	3.94094E-05	0.018602826
472.079994	2760.598693	6.23029E-05	0.029411937
472.119994	3947.62111	8.90923E-05	0.042062261
472.159994	5117.230809	0.000115489	0.054529178
472.199994	6072.615191	0.00013705	0.064715229
472.239994	6426.03328	0.000145027	0.06848737
472.279994	5811.747152	0.000131163	0.061945679
472.319994	4506.316124	0.000101701	0.048035547
472.359994	3026.098142	6.82948E-05	0.032259741
472.399994	1778.88668	4.0147E-05	0.01896544
472.439994	936.944633	2.11455E-05	0.009989997
472.479994	514.588596	1.16135E-05	0.005487169
472.519994	375.204475	8.46784E-06	0.004001225
472.559994	379.896048	8.57372E-06	0.004051599
472.599994	401.317979	9.05719E-06	0.004280427
472.639994	448.847229	1.01299E-05	0.004787775
472.679994	527.469976	1.19043E-05	0.005626907
472.719994	607.42032	1.37086E-05	0.006480344
472.759994	622.932799	1.40587E-05	0.006646403
472.799994	578.707713	1.30606E-05	0.006175064
472.839994	509.239283	1.14928E-05	0.005434266
472.879994	486.650676	1.0983E-05	0.005193654
472.919994	579.539911	1.30794E-05	0.006185514
472.959994	901.592631	2.03477E-05	0.009623643
472.999994	1577.230435	3.55959E-05	0.016836858
473.039994	2627.131647	5.92907E-05	0.028046873
473.079994	3882.981918	8.76335E-05	0.041457653
473.119994	5030.454569	0.00011353	0.05371348
473.159994	5808.713461	0.000131095	0.062028707
473.199994	6141.047876	0.000138595	0.065583104
473.239994	5883.924647	0.000132792	0.062842478
473.279994	5055.310717	0.000114091	0.05399714
473.319994	3928.292035	8.86561E-05	0.041962695
473.359994	2746.908179	6.19939E-05	0.029345428
473.399994	1704.241474	3.84624E-05	0.018208079
473.439994	977.773161	2.2067E-05	0.01044739
473.479994	591.293201	1.33447E-05	0.006318431
473.519994	443.085679	9.99983E-06	0.004735118
473.559994	434.592516	9.80815E-06	0.004644746
473.599994	474.240916	1.0703E-05	0.00506892
473.639994	489.41356	1.10454E-05	0.005231535
473.679994	479.510136	1.08219E-05	0.005126106
473.719994	439.751455	9.92458E-06	0.004701471
473.759994	391.538036	8.83647E-06	0.004186365
473.799994	331.522617	7.482E-06	0.003544973
473.839994	297.834096	6.7217E-06	0.003185011

473.879994	298.751922	6.74241E-06	0.003195095
473.919994	353.834154	7.98554E-06	0.003784509
473.959994	546.178383	1.23265E-05	0.005842261
473.999994	974.5209	2.19936E-05	0.010424956
474.039994	1736.576189	3.91921E-05	0.018578626
474.079994	2813.965395	6.35073E-05	0.030107531
474.119994	4027.897334	9.0904E-05	0.043099419
474.159994	5053.665837	0.000114054	0.054079938
474.199994	5626.047108	0.000126972	0.060210144
474.239994	5596.943692	0.000126315	0.059903731
474.279994	4858.584466	0.000109651	0.052005505
474.319994	3671.945466	8.28707E-05	0.039307227
474.359994	2535.996521	5.72339E-05	0.027149477
474.399994	1571.135651	3.54583E-05	0.016821438
474.439994	875.572756	1.97605E-05	0.009375151
474.479994	493.716097	1.11425E-05	0.005286886
474.519994	398.704091	8.9982E-06	0.004269824
474.559994	448.192965	1.01151E-05	0.004800217
474.599994	501.173352	1.13108E-05	0.005368098
474.639994	500.372836	1.12927E-05	0.005359976
474.679994	475.360935	1.07282E-05	0.005092478
474.719994	457.466678	1.03244E-05	0.004901192
474.759994	450.18833	1.01601E-05	0.00482362
474.799994	460.718407	1.03978E-05	0.004936862
474.839994	494.071434	1.11505E-05	0.005294705
474.879994	585.885163	1.32226E-05	0.006279154
474.919994	786.445788	1.7749E-05	0.008429348
474.959994	1210.621027	2.7322E-05	0.012976871
474.999994	2100.463208	4.74045E-05	0.022517151
475.039994	3379.385715	7.6268E-05	0.036230364
475.079994	4765.147944	0.000107543	0.05109141
475.119994	5939.225042	0.00013404	0.063685101
475.159994	6808.763349	0.000153664	0.07301513
475.199994	7310.308441	0.000164983	0.078400148
475.239994	7337.154797	0.000165589	0.078694688
475.279994	7073.304223	0.000159635	0.075871143
475.319994	6670.653216	0.000150547	0.071558166
475.359994	5949.664773	0.000134276	0.063829271
475.399994	4831.386629	0.000109038	0.051836506
475.439994	3531.040766	7.96907E-05	0.037888133
475.479994	2346.384739	5.29546E-05	0.02517887
475.519994	1434.905789	3.23838E-05	0.015399156
475.559994	914.933818	2.06488E-05	0.009819735
475.599994	728.258498	1.64358E-05	0.007816857
475.639994	650.625694	1.46837E-05	0.006984162
475.679994	556.245766	1.25537E-05	0.00597154

475.719994	481.822998	1.08741E-05	0.005173014
475.759994	393.27613	8.87569E-06	0.0042227
475.799994	331.503966	7.48158E-06	0.003559737
475.839994	314.753709	7.10355E-06	0.003380155
475.879994	350.373792	7.90745E-06	0.003762997
475.919994	440.633552	9.94449E-06	0.004732779
475.959994	654.278613	1.47662E-05	0.0070281
475.999994	1046.885323	2.36267E-05	0.01124633
476.039994	1662.531438	3.7521E-05	0.017861506
476.079994	2441.915388	5.51106E-05	0.026237068
476.119994	3259.618562	7.35651E-05	0.035025791
476.159994	3813.753256	8.60711E-05	0.040983614
476.199994	3957.565238	8.93167E-05	0.042532629
476.239994	3910.146205	8.82466E-05	0.042026538
476.279994	3697.6019	8.34497E-05	0.039745434
476.319994	3271.180549	7.3826E-05	0.035164794
476.359994	2720.24327	6.13921E-05	0.029244741
476.399994	2110.691578	4.76354E-05	0.022693489
476.439994	1488.198356	3.35866E-05	0.016001982
476.479994	1021.439851	2.30525E-05	0.010984043
476.519994	713.404823	1.61006E-05	0.007672235
476.559994	554.332166	1.25105E-05	0.005962006
476.599994	553.568337	1.24933E-05	0.005954291
476.639994	610.508869	1.37783E-05	0.006567305
476.679994	666.32405	1.5038E-05	0.007168316
476.719994	616.505528	1.39137E-05	0.006632925
476.759994	559.345507	1.26236E-05	0.006018451
476.799994	570.107418	1.28665E-05	0.006134762
476.839994	770.703655	1.73937E-05	0.008294016
476.879994	1555.561049	3.51068E-05	0.016741753
476.919994	3772.263048	8.51347E-05	0.040602451
476.959994	8818.918127	0.000199031	0.094929681
476.999994	17766.10648	0.000400956	0.191256142
477.039994	29437.8385	0.000664371	0.316931543
477.079994	39434.37995	0.000889979	0.424591189
477.119994	43403.56743	0.000979558	0.467366736
477.159994	39318.91592	0.000887373	0.423418974
477.199994	29947.32789	0.000675869	0.322524911
477.239994	19710.86012	0.000444847	0.212298616
477.279994	11610.37242	0.00026203	0.125061645
477.319994	6268.016363	0.00014146	0.067521877
477.359994	3221.208202	7.26982E-05	0.034703204
477.399994	1634.979703	3.68992E-05	0.017615685
477.439994	869.672653	1.96273E-05	0.009370858
477.479994	551.141135	1.24385E-05	0.005939129
477.519994	424.045064	9.57011E-06	0.004569917

477.559994	419.90494	9.47667E-06	0.004525678
477.599994	494.727764	1.11653E-05	0.005332555
477.639994	551.657664	1.24501E-05	0.005946687
477.679994	550.515045	1.24244E-05	0.005934867
477.719994	539.850269	1.21837E-05	0.005820382
477.759994	503.528195	1.13639E-05	0.005429231
477.799994	439.612896	9.92145E-06	0.004740469
477.839994	406.900489	9.18318E-06	0.004388089
477.879994	524.583751	1.18391E-05	0.005657681
477.919994	1030.856916	2.3265E-05	0.011118811
477.959994	2324.211964	5.24542E-05	0.025071022
477.999994	4734.868017	0.000106859	0.051078781
478.039994	8128.444793	0.000183448	0.087695326
478.079994	11179.75435	0.000252311	0.120625073
478.119994	12735.34033	0.000287419	0.137420719
478.159994	12035.10596	0.000271616	0.129875703
478.199994	9688.310466	0.000218652	0.104559228
478.239994	6918.343522	0.000156137	0.074671138
478.279994	4586.141992	0.000103503	0.049503336
478.319994	2819.864952	6.36404E-05	0.030440487
478.359994	1633.229721	3.68597E-05	0.017632215
478.399994	950.937342	2.14613E-05	0.010267101
478.439994	591.109459	1.33405E-05	0.006382637
478.479994	429.794398	9.69986E-06	0.004641189
478.519994	435.509179	9.82884E-06	0.004703294
478.559994	523.148247	1.18067E-05	0.005650227
478.599994	608.202966	1.37263E-05	0.006569404
478.639994	711.061206	1.60477E-05	0.007681052
478.679994	777.88542	1.75558E-05	0.008403606
478.719994	795.68145	1.79574E-05	0.008596577
478.759994	813.742622	1.8365E-05	0.008792445
478.799994	945.074377	2.1329E-05	0.010212331
478.839994	1349.251761	3.04507E-05	0.014581029
478.879994	2262.507794	5.10616E-05	0.024452402
478.919994	4280.192063	9.6598E-05	0.046262701
478.959994	8363.386784	0.00018875	0.090403694
478.999994	14674.40783	0.000331181	0.158635671
479.039994	21682.76803	0.00048935	0.23441816
479.079994	26844.29157	0.000605838	0.290244995
479.119994	27532.89821	0.000621379	0.297715181
479.159994	23797.61509	0.000537079	0.257346783
479.199994	18116.07955	0.000408855	0.195923161
479.239994	12487.52726	0.000281826	0.135062337
479.279994	7926.881551	0.000178899	0.085742556
479.319994	4713.430788	0.000106376	0.050987936
479.359994	2684.84358	6.05932E-05	0.029045946

479.399994	1467.459155	3.31185E-05	0.015877013
479.439994	800.477946	1.80657E-05	0.008661406
479.479994	492.546545	1.11161E-05	0.005329943
479.519994	393.719992	8.88571E-06	0.004260876
479.559994	418.641564	9.44816E-06	0.004530958
479.599994	493.669292	1.11414E-05	0.005343429
479.639994	560.045002	1.26394E-05	0.006062379
479.679994	563.259403	1.2712E-05	0.006097683
479.719994	499.151789	1.12652E-05	0.005404123
479.759994	386.592518	8.72485E-06	0.004185836
479.799994	355.86029	8.03127E-06	0.003853404
479.839994	422.436905	9.53381E-06	0.004574705
479.879994	590.060053	1.33168E-05	0.006390482
479.919994	1034.528032	2.33479E-05	0.011205103
479.959994	2022.596646	4.56472E-05	0.021908825
479.999994	3714.323666	8.38271E-05	0.040237013
480.039994	5871.775028	0.000132518	0.063613836
480.079994	7777.875697	0.000175536	0.084271236
480.119994	8643.894892	0.000195081	0.093662129
480.159994	8222.968618	0.000185581	0.089108547
480.199994	7029.910164	0.000158655	0.076186265
480.239994	5512.36613	0.000124406	0.059744941
480.279994	4001.156696	9.03005E-05	0.04336954
480.319994	2681.719417	6.05227E-05	0.029070249
480.359994	1639.769433	3.70073E-05	0.017776832
480.399994	900.782404	2.03294E-05	0.009766246
480.439994	517.967402	1.16898E-05	0.005616249
480.479994	402.131685	9.07555E-06	0.004360621
480.519994	421.864817	9.5209E-06	0.004574983
480.559994	474.441903	1.07075E-05	0.005145593
480.599994	549.617301	1.24041E-05	0.005961409
480.639994	621.317502	1.40223E-05	0.006739664
480.679994	660.919296	1.4916E-05	0.007169836
480.719994	700.587031	1.58113E-05	0.007600795
480.759994	741.861015	1.67428E-05	0.008049254
480.799994	822.556368	1.8564E-05	0.008925548
480.839994	1016.714173	2.29458E-05	0.011033269
480.879994	1501.743371	3.38923E-05	0.016298108
480.919994	2779.55594	6.27307E-05	0.030168451
480.959994	5373.047191	0.000121262	0.058322263
480.999994	9353.127458	0.000211087	0.101532874
481.039994	14215.31405	0.00032082	0.154327173
481.079994	18470.9212	0.000416863	0.200544432
481.119994	20347.58933	0.000459217	0.22093836
481.159994	19189.71161	0.000433085	0.208383206
481.199994	15976.20903	0.000360561	0.173501847

481.239994	12433.71485	0.000280612	0.135041537
481.279994	9195.432023	0.000207528	0.099879119
481.319994	6393.084397	0.000144283	0.069446298
481.359994	4057.603486	9.15745E-05	0.044080281
481.399994	2287.255464	5.16202E-05	0.024849949
481.439994	1218.859872	2.7508E-05	0.013243437
481.479994	683.426301	1.5424E-05	0.007426338
481.519994	508.50634	1.14763E-05	0.005526058
481.559994	500.890694	1.13044E-05	0.005443749
481.599994	499.818478	1.12802E-05	0.005432548
481.639994	524.906655	1.18464E-05	0.005705706
481.679994	531.430789	1.19937E-05	0.005777103
481.719994	504.566743	1.13874E-05	0.005485523
481.759994	442.706871	9.99128E-06	0.004813398
481.799994	399.108958	9.00733E-06	0.004339733
481.839994	407.11865	9.1881E-06	0.004427194
481.879994	509.695938	1.15031E-05	0.005543127
481.919994	822.416746	1.85608E-05	0.008944821
481.959994	1597.372977	3.60505E-05	0.017374891
481.999994	2981.137458	6.72801E-05	0.032429017
482.039994	4775.884876	0.000107785	0.051956714
482.079994	6476.192038	0.000146159	0.070460153
482.119994	7325.627127	0.000165329	0.079708513
482.159994	7198.663387	0.000162464	0.078333548
482.199994	6413.090503	0.000144735	0.069790985
482.239994	5348.713097	0.000120713	0.058212639
482.279994	4157.627211	9.38319E-05	0.045253226
482.319994	2858.668452	6.45162E-05	0.031117436
482.359994	1732.689137	3.91044E-05	0.018862389
482.399994	985.297061	2.22368E-05	0.010727024
482.439994	591.511906	1.33496E-05	0.006440381
482.479994	441.207632	9.95744E-06	0.004804266
482.519994	431.374489	9.73552E-06	0.004697584
482.559994	505.191832	1.14015E-05	0.005501896
482.599994	596.124487	1.34537E-05	0.006492755
482.639994	621.498244	1.40263E-05	0.006769677
482.679994	590.860654	1.33349E-05	0.00643649
482.719994	525.005256	1.18486E-05	0.005719574
482.759994	436.166373	9.84367E-06	0.004752129
482.799994	367.255702	8.28845E-06	0.004001663
482.839994	339.386682	7.65948E-06	0.003698306
482.879994	379.163663	8.5572E-06	0.004132099
482.919994	585.56172	1.32153E-05	0.006381939
482.959994	1124.93278	2.53882E-05	0.012261468
482.999994	2027.024808	4.57471E-05	0.022095862
483.039994	3187.052871	7.19273E-05	0.034743784

483.079994	4434.274327	0.000100075	0.048344421
483.119994	5450.508698	0.00012301	0.059428779
483.159994	5888.818273	0.000132902	0.064213135
483.199994	5636.216219	0.000127202	0.061463788
483.239994	4909.999977	0.000110812	0.053548723
483.279994	3875.680512	8.74687E-05	0.042271878
483.319994	2715.228526	6.12789E-05	0.02961733
483.359994	1661.49116	3.74975E-05	0.018124811
483.399994	908.9634	2.0514E-05	0.009916486
483.439994	520.878431	1.17555E-05	0.005683079
483.479994	386.884548	8.73145E-06	0.004221479
483.519994	397.201144	8.96428E-06	0.004334407
483.559994	455.840016	1.02877E-05	0.004974708
483.599994	508.205086	1.14695E-05	0.005546641
483.639994	526.132449	1.18741E-05	0.005742778
483.679994	483.907786	1.09211E-05	0.00528233
483.719994	442.094773	9.97746E-06	0.004826298
483.759994	436.884892	9.85988E-06	0.004769817
483.799994	421.281165	9.50773E-06	0.004599839
483.839994	364.880013	8.23483E-06	0.003984342
483.879994	323.697116	7.30539E-06	0.003534934
483.919994	371.148626	8.37631E-06	0.004053463
483.959994	590.225747	1.33206E-05	0.006446624
483.999994	1025.716728	2.3149E-05	0.011204115
484.039994	1711.16506	3.86186E-05	0.018692953
484.079994	2532.351173	5.71516E-05	0.027665965
484.119994	3193.057943	7.20629E-05	0.034887076
484.159994	3610.057564	8.1474E-05	0.039446437
484.199994	3760.39123	8.48668E-05	0.0410925
484.239994	3482.946591	7.86053E-05	0.038063807
484.279994	2830.851242	6.38884E-05	0.030939859
484.319994	2067.302964	4.66561E-05	0.022596506
484.359994	1332.482959	3.00723E-05	0.014565811
484.399994	779.92333	1.76018E-05	0.008526304
484.439994	487.187653	1.09951E-05	0.005326489
484.479994	389.4	8.78822E-06	0.004257715
484.519994	397.339172	8.96739E-06	0.004344881
484.559994	446.903989	1.0086E-05	0.004887272
484.599994	495.447535	1.11816E-05	0.005418584
484.639994	556.520102	1.25599E-05	0.006087022
484.679994	544.324055	1.22846E-05	0.005954117
484.719994	442.672175	9.99049E-06	0.004842592
484.759994	370.916124	8.37106E-06	0.004057955
484.799994	357.189113	8.06126E-06	0.003908099
484.839994	345.902764	7.80654E-06	0.003784925
484.879994	399.001075	9.0049E-06	0.004366295

484.919994	610.533952	1.37789E-05	0.006681665
484.959994	1185.158098	2.67474E-05	0.012971403
484.999994	2183.685954	4.92828E-05	0.023902134
485.039994	3515.204591	7.93333E-05	0.038479809
485.079994	4888.287696	0.000110322	0.05351492
485.119994	5789.754265	0.000130667	0.063389024
485.159994	6167.254591	0.000139186	0.067527647
485.199994	6039.204133	0.000136296	0.066131025
485.239994	5299.142245	0.000119594	0.058031918
485.279994	4296.53312	9.69668E-05	0.047056033
485.319994	3212.202148	7.24949E-05	0.035183238
485.359994	2198.975426	4.96278E-05	0.024087355
485.399994	1396.905197	3.15262E-05	0.01530282
485.439994	817.816315	1.8457E-05	0.008959754
485.479994	488.950864	1.10349E-05	0.005357243
485.519994	355.459374	8.02222E-06	0.00389495
485.559994	374.413848	8.45E-06	0.004102981
485.599994	462.884246	1.04467E-05	0.005072894
485.639994	514.495396	1.16114E-05	0.005638982
485.679994	507.540903	1.14545E-05	0.005563217
485.719994	452.374373	1.02095E-05	0.004958939
485.759994	363.2572	8.19821E-06	0.003982362
485.799994	296.093568	6.68242E-06	0.003246319
485.839994	272.931639	6.15969E-06	0.002992622
485.879994	295.812647	6.67608E-06	0.003243773
485.919994	380.773574	8.59353E-06	0.004175768
485.959994	585.919309	1.32234E-05	0.006426035
485.999994	954.523143	2.15423E-05	0.010469537
486.039994	1596.620014	3.60335E-05	0.017513717
486.079994	2410.073081	5.4392E-05	0.026438859
486.119994	3178.784177	7.17407E-05	0.034874604
486.159994	3732.430798	8.42358E-05	0.040952059
486.199994	3925.08507	8.85837E-05	0.043069396
486.239994	3712.096912	8.37769E-05	0.040735658
486.279994	3110.205443	7.0193E-05	0.034133454
486.319994	2298.90598	5.18831E-05	0.025231792
486.359994	1522.760931	3.43666E-05	0.016714536
486.399994	939.744902	2.12087E-05	0.010315928
486.439994	590.770524	1.33329E-05	0.00648564
486.479994	442.189799	9.97961E-06	0.00485488
486.519994	430.804691	9.72266E-06	0.004730269
486.559994	493.706274	1.11423E-05	0.005421379
486.599994	551.293625	1.24419E-05	0.006054243
486.639994	542.951056	1.22536E-05	0.005963116
486.679994	511.343632	1.15403E-05	0.00561644
486.719994	473.911881	1.06955E-05	0.005205729

486.759994	443.457217	1.00082E-05	0.004871597
486.799994	456.182549	1.02954E-05	0.005011803
486.839994	529.454267	1.1949E-05	0.005817273
486.879994	747.392969	1.68676E-05	0.008212506
486.919994	1231.028621	2.77826E-05	0.013527904
486.959994	2156.624635	4.8672E-05	0.023701324
486.999994	3713.379102	8.38058E-05	0.040813421
487.039994	5690.893476	0.000128436	0.062553244
487.079994	7539.75785	0.000170162	0.082882424
487.119994	8684.181807	0.00019599	0.095470593
487.159994	8751.543945	0.00019751	0.096219047
487.199994	7885.816095	0.000177972	0.086707901
487.239994	6615.025423	0.000149292	0.072740989
487.279994	5183.995616	0.000116996	0.057009596
487.319994	3817.460265	8.61548E-05	0.041984938
487.359994	2660.89594	6.00527E-05	0.029267291
487.399994	1757.464739	3.96635E-05	0.019332005
487.439994	1103.495995	2.49044E-05	0.012139385
487.479994	690.704571	1.55882E-05	0.007598955
487.519994	487.346753	1.09987E-05	0.005362104
487.559994	451.093412	1.01805E-05	0.004963629
487.599994	502.756869	1.13465E-05	0.005532564
487.639994	540.313093	1.21941E-05	0.005946338
487.679994	527.843188	1.19127E-05	0.005809578
487.719994	525.337671	1.18561E-05	0.005782476
487.759994	514.903675	1.16207E-05	0.005668092
487.799994	498.180241	1.12432E-05	0.00548445
487.839994	499.680556	1.12771E-05	0.005501418
487.879994	503.022014	1.13525E-05	0.005538661
487.919994	574.856807	1.29737E-05	0.006330136
487.959994	827.696243	1.868E-05	0.009115069
487.999994	1377.521226	3.10887E-05	0.015171303
488.039994	2217.54612	5.00469E-05	0.024424902
488.079994	3177.830196	7.17192E-05	0.035004707
488.119994	4151.407316	9.36915E-05	0.045732685
488.159994	4911.89731	0.000110855	0.05411482
488.199994	5141.442629	0.000116035	0.056648383
488.239994	4790.439151	0.000108114	0.052785354
488.279994	4065.902367	9.17618E-05	0.044805428
488.319994	3108.503749	7.01546E-05	0.034257894
488.359994	2094.322067	4.72659E-05	0.02308279
488.399994	1257.904774	2.83892E-05	0.013865265
488.439994	746.706132	1.68521E-05	0.008231248
488.479994	510.229652	1.15152E-05	0.005624931
488.519994	435.494938	9.82851E-06	0.004801426
488.559994	420.928853	9.49978E-06	0.004641211

488.599994	455.128896	1.02716E-05	0.005018716
488.639994	516.789769	1.16632E-05	0.005699118
488.679994	517.319925	1.16752E-05	0.005705432
488.719994	478.575855	1.08008E-05	0.005278562
488.759994	432.430409	9.75935E-06	0.004769981
488.799994	381.714076	8.61476E-06	0.004210892
488.839994	388.299977	8.76339E-06	0.004283895
488.879994	480.224926	1.0838E-05	0.005298485
488.919994	689.021667	1.55503E-05	0.007602833
488.959994	1072.331661	2.4201E-05	0.011833336
488.999994	1764.883897	3.9831E-05	0.019477345
489.039994	2866.709264	6.46976E-05	0.031639731
489.079994	4233.397859	9.55419E-05	0.046727628
489.119994	5380.60294	0.000121433	0.059395168
489.159994	6169.583878	0.000139239	0.068110107
489.199994	6451.608526	0.000145604	0.071229387
489.239994	6143.313735	0.000138646	0.067831185
489.279994	5360.778746	0.000120985	0.059195691
489.319994	4169.050436	9.40897E-05	0.046039953
489.359994	2964.088925	6.68954E-05	0.032735912
489.399994	1934.921443	4.36685E-05	0.021371355
489.439994	1163.470453	2.62579E-05	0.01285167
489.479994	701.443711	1.58306E-05	0.007748766
489.519994	502.169381	1.13333E-05	0.005547859
489.559994	488.033191	1.10142E-05	0.005392126
489.599994	509.068464	1.1489E-05	0.005624998
489.639994	523.127067	1.18062E-05	0.005780812
489.679994	522.792785	1.17987E-05	0.00577759
489.719994	500.1918	1.12886E-05	0.005528269
489.759994	438.98133	9.9072E-06	0.004852149
489.799994	397.612075	8.97355E-06	0.004395245
489.839994	385.78155	8.70655E-06	0.004264817
489.879994	368.777605	8.3228E-06	0.004077172
489.919994	426.286882	9.6207E-06	0.004713374
489.959994	580.338044	1.30974E-05	0.006417212
489.999994	939.677949	2.12072E-05	0.010391539
490.039994	1584.07868	3.57504E-05	0.01751915
490.079994	2432.051973	5.4888E-05	0.026899523
490.119994	3243.279792	7.31963E-05	0.035874974
490.159994	3932.080393	8.87416E-05	0.043497571
490.199994	4395.033265	9.91898E-05	0.048622829
490.239994	4361.917329	9.84424E-05	0.048260401
490.279994	3890.520094	8.78036E-05	0.043048358
490.319994	3135.101387	7.07549E-05	0.034692528
490.359994	2273.653778	5.13132E-05	0.02516194
490.399994	1465.006064	3.30631E-05	0.016214167

490.439994	871.32196	1.96645E-05	0.009644269
490.479994	550.25307	1.24184E-05	0.006090999
490.519994	438.265833	9.89105E-06	0.004851757
490.559994	432.055867	9.7509E-06	0.004783401
490.599994	467.547951	1.05519E-05	0.005176765
490.639994	477.327824	1.07726E-05	0.00528548
490.679994	466.916839	1.05377E-05	0.00517062
490.719994	439.500565	9.91892E-06	0.00486741
490.759994	386.663247	8.72645E-06	0.004282593
490.799994	359.73146	8.11864E-06	0.003984627
490.839993	384.696034	8.68205E-06	0.004261499
490.879993	450.294778	1.01625E-05	0.004988581
490.919993	650.509861	1.46811E-05	0.007207246
490.959993	1222.691808	2.75945E-05	0.013547771
490.999993	2284.935023	5.15678E-05	0.02531979
491.039993	3776.731225	8.52356E-05	0.04185407
491.079993	5335.025867	0.000120404	0.059128046
491.119993	6576.559479	0.000148424	0.072893889
491.159993	7312.197412	0.000165026	0.081054223
491.199993	7404.440718	0.000167108	0.082083406
491.239993	6785.034795	0.000153129	0.075222983
491.279993	5610.579458	0.000126623	0.062207329
491.319993	4126.670336	9.31332E-05	0.045758204
491.359993	2692.896486	6.07749E-05	0.029862366
491.399993	1567.655073	3.53798E-05	0.017385629
491.439993	856.45314	1.9329E-05	0.009499021
491.479993	496.192151	1.11984E-05	0.005503773
491.519993	384.507973	8.67781E-06	0.004265317
491.559993	417.184146	9.41527E-06	0.004628168
491.599993	517.815788	1.16864E-05	0.005745025
491.639993	610.050394	1.3768E-05	0.006768893
491.679993	665.433545	1.50179E-05	0.007384005
491.719993	661.999513	1.49404E-05	0.007346497
491.759993	618.098583	1.39496E-05	0.006859867
491.799993	548.203483	1.23722E-05	0.006084642
491.839993	433.161985	9.77586E-06	0.00480816
491.879993	355.284446	8.01828E-06	0.003944029
491.919993	393.025392	8.87004E-06	0.004363348
491.959993	579.994102	1.30897E-05	0.006439589
491.999993	980.814278	2.21356E-05	0.010890721
492.039993	1672.564765	3.77475E-05	0.018573259
492.079993	2514.634264	5.67518E-05	0.027926422
492.119993	3353.829913	7.56913E-05	0.037249187
492.159993	3932.114406	8.87423E-05	0.043675432
492.199993	4060.231873	9.16338E-05	0.045102145
492.239993	3733.607767	8.42623E-05	0.041477287

492.279993	3065.418633	6.91822E-05	0.034057027
492.319993	2290.820213	5.17006E-05	0.02545325
492.359993	1551.885375	3.50239E-05	0.017244363
492.399993	931.009003	2.10116E-05	0.0103461
492.439993	530.66791	1.19764E-05	0.005897676
492.479993	353.221187	7.97171E-06	0.003925908
492.519993	344.323941	7.77091E-06	0.003827329
492.559993	431.157956	9.73063E-06	0.004792921
492.599993	532.230512	1.20117E-05	0.005916964
492.639993	581.870803	1.3132E-05	0.006469355
492.679993	598.941411	1.35173E-05	0.00665969
492.719993	586.036911	1.3226E-05	0.006516733
492.759993	527.889203	1.19137E-05	0.005870607
492.799993	507.619306	1.14563E-05	0.005645645
492.839993	525.089236	1.18505E-05	0.005840416
492.879993	604.223428	1.36365E-05	0.006721149
492.919993	845.140407	1.90736E-05	0.009401779
492.959993	1438.970273	3.24756E-05	0.016009149
492.999993	2581.667236	5.82646E-05	0.028724464
493.039993	4197.805273	9.47386E-05	0.046709928
493.079993	5967.983145	0.000134689	0.066412484
493.119993	7257.974079	0.000163802	0.080774221
493.159993	7617.309742	0.000171912	0.084780156
493.199993	7236.930694	0.000163327	0.080553095
493.239993	6283.648993	0.000141813	0.069947944
493.279993	4975.045203	0.00011228	0.055385399
493.319993	3619.452265	8.1686E-05	0.040297335
493.359993	2363.938279	5.33508E-05	0.026321147
493.399993	1375.047101	3.10329E-05	0.015311632
493.439993	758.005576	1.71071E-05	0.008441342
493.479993	461.609583	1.04179E-05	0.005141018
493.519993	376.949143	8.50722E-06	0.004198482
493.559993	394.819342	8.91052E-06	0.004397877
493.599993	459.009352	1.03592E-05	0.005113302
493.639993	512.618308	1.15691E-05	0.005710961
493.679993	499.502889	1.12731E-05	0.005565296
493.719993	414.621583	9.35743E-06	0.004619951
493.759993	366.973564	8.28208E-06	0.004089361
493.799993	335.504388	7.57187E-06	0.003738988
493.839993	290.263021	6.55083E-06	0.003235063
493.879993	300.271732	6.77671E-06	0.003346884
493.919993	407.239983	9.19084E-06	0.004539539
493.959993	637.271789	1.43823E-05	0.007104299
493.999993	1118.165669	2.52354E-05	0.012466308
494.039993	1858.1015	4.19348E-05	0.02071745
494.079993	2731.635544	6.16492E-05	0.030459641

494.119993	3553.95695	8.02079E-05	0.039632306
494.159993	4090.298456	9.23123E-05	0.045617065
494.199993	4289.265545	9.68027E-05	0.047839918
494.239993	4112.623704	9.28162E-05	0.045873472
494.279993	3544.999641	8.00057E-05	0.039545219
494.319993	2661.987797	6.00774E-05	0.029697439
494.359993	1747.567896	3.94402E-05	0.019497644
494.399993	1048.854883	2.36712E-05	0.011703038
494.439993	635.250352	1.43367E-05	0.007088646
494.479993	423.322353	9.5538E-06	0.004724161
494.519993	361.909802	8.1678E-06	0.00403914
494.559993	390.947103	8.82313E-06	0.004363568
494.599993	438.701937	9.90089E-06	0.004896981
494.639993	493.675917	1.11416E-05	0.00551107
494.679993	558.557218	1.26059E-05	0.006235866
494.719993	610.088415	1.37688E-05	0.006811723
494.759993	611.850982	1.38086E-05	0.006831955
494.799993	601.638035	1.35781E-05	0.00671846
494.839993	585.61362	1.32165E-05	0.006540045
494.879993	647.30212	1.46087E-05	0.007229557
494.919993	959.090437	2.16453E-05	0.010712709
494.959993	1737.607483	3.92154E-05	0.019410045
494.999993	3081.027824	6.95345E-05	0.03441958
495.039993	4864.568891	0.000109787	0.05434873
495.079993	6547.968899	0.000147779	0.073162197
495.119993	7567.367715	0.000170785	0.084559043
495.159993	7747.841733	0.000174858	0.086582684
495.199993	7176.876003	0.000161972	0.08020858
495.239993	6105.786653	0.000137799	0.068243625
495.279993	4716.011208	0.000106434	0.052714534
495.319993	3257.5062	7.35174E-05	0.036414627
495.359993	2047.00603	4.61981E-05	0.022884678
495.399993	1169.887795	2.64027E-05	0.013079916
495.439993	644.89385	1.45544E-05	0.00721081
495.479993	418.506048	9.4451E-06	0.004679857
495.519993	374.495908	8.45185E-06	0.004188061
495.559993	419.690068	9.47182E-06	0.004693855
495.599993	474.834137	1.07163E-05	0.005311021
495.639993	500.804803	1.13025E-05	0.005601955
495.679993	473.105722	1.06773E-05	0.005292542
495.719993	421.504696	9.51277E-06	0.004715672
495.759993	382.67016	8.63633E-06	0.004281548
495.799993	345.15813	7.78974E-06	0.003862152
495.839993	296.506013	6.69173E-06	0.003318026
495.879993	283.926751	6.40783E-06	0.003177515
495.919993	372.606817	8.40922E-06	0.004170299

495.959993	689.925088	1.55706E-05	0.007722419
495.999993	1251.884059	2.82533E-05	0.014013626
496.039993	2042.896262	4.61053E-05	0.022870084
496.079993	2950.223116	6.65824E-05	0.03303021
496.119993	3723.040577	8.40238E-05	0.041685907
496.159993	4233.538618	9.55451E-05	0.047405641
496.199993	4539.444	0.000102449	0.050835157
496.239993	4649.634223	0.000104936	0.052073325
496.279993	4587.396913	0.000103531	0.051380443
496.319993	4346.350285	9.80911E-05	0.04868456
496.359993	3854.109148	8.69819E-05	0.043174323
496.399993	3108.774865	7.01607E-05	0.03482778
496.439993	2233.10993	5.03982E-05	0.025019672
496.479993	1456.758918	3.2877E-05	0.016322783
496.519993	947.586192	2.13857E-05	0.010618428
496.559993	720.486229	1.62604E-05	0.00807425
496.599993	669.997226	1.51209E-05	0.007509041
496.639993	620.322942	1.39998E-05	0.006952873
496.679993	563.18285	1.27103E-05	0.006312928
496.719993	516.813449	1.16638E-05	0.005793623
496.759993	487.556526	1.10035E-05	0.005466085
496.799993	466.420184	1.05265E-05	0.005229542
496.839993	469.295733	1.05914E-05	0.005262207
496.879993	588.92625	1.32912E-05	0.006604154
496.919993	994.734091	2.24498E-05	0.011155736
496.959993	1993.73338	4.49958E-05	0.022361105
496.999993	3646.333973	8.22927E-05	0.040899461
497.039993	5640.175418	0.000127291	0.06326867
497.079993	7511.629111	0.000169527	0.084268483
497.119993	8671.887112	0.000195712	0.097292553
497.159993	8634.553984	0.00019487	0.096881496
497.199993	7675.778497	0.000173232	0.086130765
497.239993	6389.916139	0.000144212	0.071707729
497.279993	5020.571688	0.000113307	0.056345458
497.319993	3666.764705	8.27538E-05	0.041155105
497.359993	2541.534058	5.73589E-05	0.028528014
497.399993	1693.905925	3.82291E-05	0.019015153
497.439993	1094.547257	2.47024E-05	0.012287965
497.479993	720.874355	1.62691E-05	0.008093567
497.519993	544.24051	1.22828E-05	0.006110914
497.559993	526.857463	1.18904E-05	0.005916207
497.599993	570.450563	1.28743E-05	0.006406239
497.639993	546.085935	1.23244E-05	0.006133114
497.679993	486.997337	1.09909E-05	0.005469927
497.719993	412.764666	9.31552E-06	0.004636522
497.759993	348.533444	7.86591E-06	0.003915337

497.799993	289.912088	6.54291E-06	0.003257062
497.839993	275.776237	6.22389E-06	0.003098499
497.879993	330.237	7.45299E-06	0.003710694
497.919993	482.982169	1.09002E-05	0.005427445
497.959993	832.074076	1.87788E-05	0.009351068
497.999993	1466.184436	3.30897E-05	0.016478691
498.039993	2409.189265	5.4372E-05	0.027079453
498.079993	3510.505281	7.92272E-05	0.03946149
498.119993	4570.119914	0.000103141	0.051376714
498.159993	5310.355671	0.000119847	0.059703145
498.199993	5614.467645	0.000126711	0.063127277
498.239993	5301.276643	0.000119642	0.059610643
498.279993	4502.3217	0.000101611	0.050630793
498.319993	3407.274288	7.68974E-05	0.038319529
498.359993	2292.087257	5.17292E-05	0.025779772
498.399993	1369.141802	3.08996E-05	0.015400372
498.439993	772.793334	1.74409E-05	0.008693227
498.479993	462.947926	1.04481E-05	0.005208164
498.519993	366.566454	8.27289E-06	0.004124203
498.559993	396.28224	8.94354E-06	0.00445889
498.599993	462.488513	1.04377E-05	0.005204248
498.639993	519.16255	1.17168E-05	0.005842453
498.679993	514.979025	1.16224E-05	0.005795838
498.719993	439.533895	9.91967E-06	0.004947137
498.759993	362.919462	8.19059E-06	0.004085137
498.799993	322.30537	7.27398E-06	0.003628263
498.839993	318.244029	7.18232E-06	0.003582831
498.879993	380.865361	8.5956E-06	0.004288173
498.919993	625.078942	1.41072E-05	0.007038345
498.959993	1227.218409	2.76966E-05	0.0138195
498.999993	2275.679876	5.13589E-05	0.025628103
499.039993	3780.739852	8.5326E-05	0.042581103
499.079993	5443.580492	0.000122854	0.061313988
499.119993	6637.113416	0.00014979	0.074763386
499.159993	6955.471577	0.000156975	0.078355792
499.199993	6580.457477	0.000148512	0.074137069
499.239993	5676.288242	0.000128106	0.063955598
499.279993	4324.585945	9.75999E-05	0.048729668
499.319993	2909.877117	6.56719E-05	0.032791279
499.359993	1784.730646	4.02789E-05	0.020113664
499.399993	1013.441546	2.2872E-05	0.011422259
499.439993	565.585973	1.27645E-05	0.006375096
499.479993	371.651135	8.38765E-06	0.004189463
499.519993	325.000879	7.33482E-06	0.003663888
499.559993	380.527455	8.58797E-06	0.004290209
499.599993	475.026164	1.07207E-05	0.005356051

499.639993	562.870383	1.27032E-05	0.006347027
499.679993	598.338898	1.35037E-05	0.006747516
499.719993	594.397163	1.34147E-05	0.006703602
499.759993	541.973997	1.22316E-05	0.006112863
499.799993	444.439794	1.00304E-05	0.005013187
499.839993	358.682837	8.09497E-06	0.004046191
499.879993	352.959041	7.96579E-06	0.003981941
499.919993	432.103117	9.75197E-06	0.004875203
499.959993	646.83878	1.45983E-05	0.007298541
499.999993	1097.923131	2.47786E-05	0.012389298
500.039993	1863.520883	4.20571E-05	0.021030217
500.079993	2786.873738	6.28959E-05	0.03145296
500.119993	3605.3483	8.13677E-05	0.040693608
500.159993	4193.361322	9.46383E-05	0.047334304
500.199993	4296.980752	9.69769E-05	0.04850783
500.239993	3922.005554	8.85142E-05	0.044278344
500.279993	3321.083703	7.49522E-05	0.037497103
500.319993	2524.256566	5.6969E-05	0.028502707
500.359993	1688.030936	3.80965E-05	0.019061968
500.399993	993.828898	2.24293E-05	0.011223639
500.439993	552.334697	1.24654E-05	0.006238197
500.479993	389.615644	8.79308E-06	0.004400762
500.519993	396.665215	8.95218E-06	0.004480746
500.559993	454.916895	1.02668E-05	0.00513917
500.599993	505.965432	1.14189E-05	0.005716319
500.639993	516.926191	1.16663E-05	0.005840618
500.679993	482.850114	1.08973E-05	0.005456037
500.719993	447.863666	1.01077E-05	0.005061107
500.759993	419.25308	9.46196E-06	0.00473817
500.799993	368.929518	8.32623E-06	0.004169774
500.839993	380.254561	8.58182E-06	0.004298117
500.879993	551.849944	1.24545E-05	0.006238202
500.919993	985.294762	2.22367E-05	0.011138823
500.959993	1861.15953	4.20038E-05	0.021042212
500.999993	3132.186832	7.06891E-05	0.035415236
501.039993	4611.266667	0.00010407	0.052143165
501.079993	5854.785911	0.000132134	0.066209886
501.119993	6541.123932	0.000147624	0.073977366
501.159993	6558.540057	0.000148017	0.074180256
501.199993	5984.905594	0.000135071	0.067697576
501.239993	5051.992942	0.000114016	0.057149602
501.279993	3908.100247	8.82004E-05	0.044213085
501.319993	2832.684469	6.39297E-05	0.032049258
501.359993	1927.574121	4.35027E-05	0.021810495
501.399993	1210.087974	2.731E-05	0.013693234
501.439993	705.535766	1.5923E-05	0.007984409

501.479993	454.572106	1.02591E-05	0.005144713
501.519993	372.819655	8.41402E-06	0.004219799
501.559993	359.362952	8.11032E-06	0.004067813
501.599993	400.891027	9.04755E-06	0.004538252
501.639993	470.560088	1.06199E-05	0.005327359
501.679993	481.764727	1.08728E-05	0.005454646
501.719993	463.538357	1.04614E-05	0.005248701
501.759993	416.356696	9.39659E-06	0.004714833
501.799993	342.56615	7.73124E-06	0.003879537
501.839993	280.545044	6.33151E-06	0.003177405
501.879993	275.438718	6.21627E-06	0.003119821
501.919993	369.277285	8.33407E-06	0.004183038
501.959993	626.993484	1.41504E-05	0.007102919
501.999993	1103.199041	2.48977E-05	0.012498628
502.039993	1763.298293	3.97952E-05	0.019978775
502.079993	2532.932809	5.71648E-05	0.028701285
502.119993	3187.727153	7.19426E-05	0.036123799
502.159993	3637.178615	8.20861E-05	0.041220333
502.199993	3823.784084	8.62975E-05	0.043338594
502.239993	3662.863401	8.26657E-05	0.041518033
502.279993	3172.78897	7.16054E-05	0.035965974
502.319993	2463.728607	5.56029E-05	0.02793046
502.359993	1689.584641	3.81316E-05	0.019155776
502.399993	1038.546436	2.34385E-05	0.011775526
502.439993	620.7209	1.40088E-05	0.007038584
502.479993	417.960695	9.43279E-06	0.004739789
502.519993	373.354318	8.42609E-06	0.004234277
502.559993	414.744912	9.36021E-06	0.00470407
502.599993	471.944324	1.06511E-05	0.005353256
502.639993	520.610916	1.17495E-05	0.00590575
502.679993	549.032083	1.23909E-05	0.006228652
502.719993	550.80042	1.24308E-05	0.006249211
502.759993	521.075051	1.17599E-05	0.005912426
502.799993	500.704131	1.13002E-05	0.005681738
502.839993	550.396814	1.24217E-05	0.006246122
502.879993	810.904248	1.8301E-05	0.009203196
502.919993	1558.804551	3.518E-05	0.017692749
502.959993	2953.715826	6.66613E-05	0.033527943
502.999993	4895.663571	0.000110488	0.055575618
503.039993	6967.85376	0.000157255	0.07910543
503.079993	8505.191828	0.00019195	0.096566372
503.119993	9214.449603	0.000207957	0.104627472
503.159993	9022.158108	0.000203618	0.102452201
503.199993	8276.636909	0.000186792	0.093993817
503.239993	7129.760017	0.000160909	0.08097572
503.279993	5728.993418	0.000129295	0.065071789

503.319993	4274.220485	9.64632E-05	0.048551859
503.359993	2885.701376	6.51263E-05	0.032781954
503.399993	1744.133939	3.93627E-05	0.01981517
503.439993	988.173608	2.23017E-05	0.011227569
503.479993	615.009515	1.38799E-05	0.006988256
503.519993	470.48825	1.06183E-05	0.005346508
503.559993	477.064786	1.07667E-05	0.005421673
503.599993	525.905	1.18689E-05	0.0059772
503.639993	542.513073	1.22438E-05	0.006166449
503.679993	528.864277	1.19357E-05	0.006011788
503.719993	469.751756	1.06016E-05	0.005340259
503.759993	391.830869	8.84308E-06	0.004454788
503.799993	325.320759	7.34204E-06	0.003698918
503.839993	304.332622	6.86836E-06	0.003460556
503.879993	347.584302	7.84449E-06	0.003952683
503.919993	485.053311	1.0947E-05	0.005516401
503.959993	858.373168	1.93723E-05	0.009762858
503.999993	1556.10156	3.5119E-05	0.017699998
504.039993	2467.487613	5.56878E-05	0.028068857
504.079993	3420.699779	7.72004E-05	0.038915192
504.119993	4253.563575	9.5997E-05	0.04839401
504.159993	4749.129806	0.000107181	0.054036496
504.199993	4794.794964	0.000108212	0.054560411
504.239993	4447.048344	0.000100364	0.050607385
504.279993	3865.821537	8.72462E-05	0.043996517
504.319993	3002.2159	6.77558E-05	0.03417062
504.359993	2032.917763	4.58801E-05	0.023140098
504.399993	1232.225128	2.78096E-05	0.014027164
504.439993	720.549931	1.62618E-05	0.008203106
504.479993	460.156779	1.03851E-05	0.005239074
504.519993	395.519045	8.92631E-06	0.004503504
504.559993	441.61203	9.96657E-06	0.005028732
504.599993	504.320643	1.13818E-05	0.005743263
504.639993	549.095886	1.23923E-05	0.006253665
504.679993	544.57561	1.22903E-05	0.006202675
504.719993	496.116622	1.11967E-05	0.005651179
504.759993	454.719246	1.02624E-05	0.005180039
504.799993	434.736832	9.8114E-06	0.004952797
504.839993	440.442227	9.94017E-06	0.005018194
504.879993	527.266926	1.18997E-05	0.00600791
504.919993	792.068147	1.78759E-05	0.009025887
504.959993	1359.473231	3.06814E-05	0.015492888
504.999993	2360.500432	5.32732E-05	0.026902969
505.039993	3678.998232	8.30299E-05	0.041933403
505.079993	5051.690268	0.00011401	0.057583974
505.119993	6217.35533	0.000140317	0.070876947

505.159993	6786.925119	0.000153171	0.077376087
505.199993	6591.769045	0.000148767	0.07515711
505.239993	5724.67555	0.000129198	0.065275973
505.279993	4566.893006	0.000103068	0.052078407
505.319993	3365.837284	7.59623E-05	0.038385248
505.359993	2270.330156	5.12382E-05	0.025893731
505.399993	1364.568314	3.07964E-05	0.015564503
505.439993	782.019399	1.76491E-05	0.008920555
505.479993	485.213254	1.09506E-05	0.005535303
505.519993	402.922157	9.09339E-06	0.004596891
505.559993	438.383003	9.89369E-06	0.005001856
505.599993	478.57348	1.08007E-05	0.005460852
505.639993	484.404663	1.09323E-05	0.005527828
505.679993	467.290001	1.05461E-05	0.005332944
505.719993	447.813845	1.01065E-05	0.005111077
505.759993	388.394369	8.76552E-06	0.004433249
505.799993	348.741952	7.87062E-06	0.00398096
505.839993	327.878015	7.39975E-06	0.00374309
505.879993	323.129485	7.29258E-06	0.003689172
505.919993	388.009588	8.75684E-06	0.004430258
505.959993	596.547506	1.34632E-05	0.006811864
505.999993	968.509857	2.18579E-05	0.011060107
506.039993	1574.510661	3.55345E-05	0.017981884
506.079993	2365.188252	5.3379E-05	0.027014046
506.119993	3101.684876	7.00007E-05	0.035428758
506.159993	3590.836891	8.10402E-05	0.0410193
506.199993	3714.350463	8.38277E-05	0.042433589
506.239993	3386.994389	7.64397E-05	0.038696856
506.279993	2785.0043	6.28537E-05	0.031821555
506.319993	2061.675147	4.65291E-05	0.023558632
506.359993	1381.80932	3.11855E-05	0.015791095
506.399993	854.958331	1.92952E-05	0.009771099
506.439993	500.588769	1.12976E-05	0.005721552
506.479993	341.419593	7.70536E-06	0.003902613
506.519993	346.318773	7.81593E-06	0.003958926
506.559993	436.588249	9.85319E-06	0.004991231
506.599993	542.862459	1.22516E-05	0.006206686
506.639993	572.800243	1.29273E-05	0.006549489
506.679993	530.345331	1.19692E-05	0.006064532
506.719993	506.469943	1.14303E-05	0.005791972
506.759993	473.964495	1.06967E-05	0.005420669
506.799993	467.443294	1.05495E-05	0.005346509
506.839993	479.360526	1.08185E-05	0.005483248
506.879993	513.103303	1.158E-05	0.005869684
506.919993	702.09714	1.58454E-05	0.008032327
506.959993	1268.37188	2.86254E-05	0.014511926

506.999993	2283.694525	5.15398E-05	0.026130681
507.039993	3688.365523	8.32413E-05	0.042206654
507.079993	5313.961281	0.000119929	0.060813447
507.119993	6900.488356	0.000155734	0.078976033
507.159993	7939.289241	0.000179179	0.090872268
507.199993	8125.304538	0.000183377	0.093008715
507.239993	7466.550573	0.00016851	0.085474832
507.279993	6134.464305	0.000138446	0.070231046
507.319993	4505.624173	0.000101686	0.051587169
507.359993	2965.871504	6.69356E-05	0.03396044
507.399993	1749.52506	3.94843E-05	0.020034356
507.439993	965.860324	2.17981E-05	0.011061239
507.479993	562.183168	1.26877E-05	0.006438749
507.519993	443.326041	1.00053E-05	0.005077865
507.559993	534.22587	1.20567E-05	0.006119516
507.599993	669.033964	1.50992E-05	0.007664336
507.639993	744.427271	1.68007E-05	0.0085287
507.679993	711.461327	1.60567E-05	0.008151661
507.719993	607.2049	1.37038E-05	0.006957677
507.759993	488.346265	1.10213E-05	0.005596173
507.799993	397.039504	8.96063E-06	0.004550207
507.839993	350.422854	7.90856E-06	0.004016281
507.879993	355.751243	8.02881E-06	0.004077672
507.919993	438.83269	9.90384E-06	0.00503036
507.959993	634.107908	1.43109E-05	0.007269381
507.999993	1072.671564	2.42087E-05	0.012298021
508.039993	1802.510773	4.06802E-05	0.020667147
508.079993	2703.223795	6.1008E-05	0.030996942
508.119993	3610.650513	8.14874E-05	0.041405353
508.159993	4350.814937	9.81918E-05	0.049897161
508.199993	4715.38452	0.00010642	0.054082471
508.239993	4468.858894	0.000100856	0.051259012
508.279993	3779.824408	8.53054E-05	0.043359014
508.319993	2880.198367	6.50021E-05	0.033041849
508.359993	1937.064696	4.37169E-05	0.022223899
508.399993	1172.185499	2.64546E-05	0.013449515
508.439993	710.111268	1.60262E-05	0.008148372
508.479993	484.057848	1.09245E-05	0.005554895
508.519993	415.831834	9.38475E-06	0.004772331
508.559993	458.909673	1.0357E-05	0.005267131
508.599993	525.671065	1.18637E-05	0.006033859
508.639993	531.930515	1.20049E-05	0.006106188
508.679993	530.277718	1.19676E-05	0.006087694
508.719993	517.577559	1.1681E-05	0.005942361
508.759993	470.546852	1.06196E-05	0.005402821
508.799993	401.287063	9.05649E-06	0.004607942

508.839993	362.680487	8.18519E-06	0.004164954
508.879993	366.295889	8.26679E-06	0.004206803
508.919993	461.759396	1.04213E-05	0.005303591
508.959993	758.184209	1.71112E-05	0.008708897
508.999993	1387.260592	3.13085E-05	0.015936046
509.039993	2373.041383	5.35562E-05	0.027262267
509.079993	3622.547372	8.17558E-05	0.041620267
509.119993	4919.369275	0.000111023	0.056524188
509.159993	5887.78704	0.000132879	0.067656748
509.199993	6320.773949	0.000142651	0.072637921
509.239993	6009.364068	0.000135623	0.069064644
509.279993	5088.110177	0.000114832	0.058481416
509.319993	3757.665914	8.48053E-05	0.043193028
509.359993	2366.438489	5.34072E-05	0.027203501
509.399993	1305.69386	2.94677E-05	0.015010842
509.439993	704.810935	1.59066E-05	0.008103459
509.479993	437.18121	9.86657E-06	0.005026821
509.519993	387.799128	8.75209E-06	0.004459363
509.559993	412.823569	9.31685E-06	0.004747496
509.599993	433.351189	9.78013E-06	0.004983956
509.639993	482.950526	1.08995E-05	0.005554832
509.679993	528.350281	1.19241E-05	0.00607749
509.719993	511.967895	1.15544E-05	0.00588951
509.759993	448.202063	1.01153E-05	0.005156373
509.799993	354.580641	8.00239E-06	0.004079619
509.839993	295.223365	6.66278E-06	0.003396952
509.879993	280.201091	6.32375E-06	0.003224353
509.919993	318.997706	7.19933E-06	0.003671084
509.959993	482.96313	1.08998E-05	0.005558465
509.999993	816.578292	1.8429E-05	0.009398808
510.039993	1351.971851	3.05121E-05	0.015562402
510.079993	2116.546818	4.77675E-05	0.024365253
510.119993	2900.806571	6.54672E-05	0.033396109
510.159993	3658.011402	8.25562E-05	0.042116882
510.199993	4155.189048	9.37768E-05	0.047844938
510.239993	3985.819335	8.99544E-05	0.045898328
510.279993	3334.308893	7.52507E-05	0.03839893
510.319993	2495.73246	5.63252E-05	0.028743878
510.359993	1659.33401	3.74489E-05	0.019112399
510.399993	974.546019	2.19941E-05	0.011225812
510.439993	560.490393	1.26495E-05	0.006456804
510.479993	396.04659	8.93822E-06	0.004562782
510.519993	354.739256	8.00597E-06	0.004087208
510.559993	380.019597	8.57651E-06	0.004378824
510.599993	452.942391	1.02223E-05	0.005219496
510.639993	508.857726	1.14842E-05	0.005864297

510.679993	532.989481	1.20288E-05	0.006142883
510.719993	519.956819	1.17347E-05	0.005993146
510.759993	470.154562	1.06107E-05	0.005419538
510.799993	402.00266	9.07264E-06	0.004634304
510.839993	369.434218	8.33762E-06	0.004259188
510.879993	429.998418	9.70447E-06	0.004957817
510.919993	680.104886	1.5349E-05	0.007842121
510.959993	1283.712756	2.89716E-05	0.014803333
510.999993	2265.34387	5.11257E-05	0.02612521
511.039993	3487.127959	7.86996E-05	0.040218654
511.079993	4652.807804	0.000105007	0.053667177
511.119993	5614.141645	0.000126703	0.064760619
511.159993	6125.099928	0.000138235	0.070660188
511.199993	6182.623301	0.000139533	0.071329369
511.239993	5737.272798	0.000129482	0.066196507
511.279993	4790.56176	0.000108116	0.055277704
511.319993	3581.69581	8.08339E-05	0.041331982
511.359993	2357.046211	5.31952E-05	0.027201923
511.399993	1386.809362	3.12984E-05	0.016005979
511.439993	787.115146	1.77641E-05	0.009085267
511.479993	489.590184	1.10494E-05	0.005651531
511.519993	412.287086	9.30475E-06	0.004759563
511.559993	431.725475	9.74344E-06	0.004984355
511.599993	473.594547	1.06884E-05	0.00546817
511.639993	493.294024	1.1133E-05	0.005696067
511.679993	482.333954	1.08856E-05	0.005569947
511.719993	473.853083	1.06942E-05	0.005472438
511.759993	430.886889	9.72452E-06	0.004976619
511.799993	339.67125	7.66591E-06	0.003923411
511.839993	271.011316	6.11635E-06	0.003130591
511.879993	266.23963	6.00866E-06	0.003075712
511.919993	348.908484	7.87438E-06	0.004031052
511.959993	578.099495	1.30469E-05	0.006679491
511.999993	1028.527602	2.32124E-05	0.011884767
512.039993	1727.373786	3.89844E-05	0.019961582
512.079993	2586.696203	5.83781E-05	0.029894273
512.119993	3456.989899	7.80194E-05	0.039955319
512.159993	4180.186401	9.4341E-05	0.048317678
512.199993	4527.039055	0.000102169	0.052330944
512.239993	4339.951398	9.79467E-05	0.050172195
512.279993	3562.840602	8.04083E-05	0.041191589
512.319993	2592.377654	5.85064E-05	0.029973974
512.359993	1753.198812	3.95673E-05	0.020272679
512.399993	1073.653347	2.42309E-05	0.012415893
512.439993	630.8599	1.42376E-05	0.007295931
512.479993	419.309159	9.46322E-06	0.004849713

512.519993	376.488413	8.49682E-06	0.00435479
512.559993	410.705065	9.26904E-06	0.00475094
512.599993	480.750691	1.08499E-05	0.005561645
512.639993	529.039838	1.19397E-05	0.006120764
512.679993	523.982366	1.18256E-05	0.006062724
512.719993	483.037818	1.09015E-05	0.005589412
512.759993	406.358991	9.17096E-06	0.0047025
512.799993	366.045335	8.26113E-06	0.004236309
512.839993	355.913917	8.03248E-06	0.004119378
512.879993	373.807244	8.43631E-06	0.004326814
512.919993	525.505378	1.18599E-05	0.006083192
512.959993	877.28164	1.9799E-05	0.010156108
512.999993	1503.896174	3.39408E-05	0.017411652
513.039993	2391.734963	5.39781E-05	0.027692938
513.079993	3315.754575	7.4832E-05	0.038394783
513.119993	4141.502269	9.34679E-05	0.047960267
513.159993	4762.561752	0.000107484	0.055156686
513.199993	4918.338763	0.000111	0.056965228
513.239993	4562.616089	0.000102972	0.052849292
513.279993	3785.393891	8.54311E-05	0.043850058
513.319993	2761.434379	6.23217E-05	0.031990989
513.359993	1791.022579	4.04209E-05	0.020750465
513.399993	1055.055122	2.38111E-05	0.012224631
513.439993	588.32717	1.32777E-05	0.006817315
513.479993	372.407111	8.40471E-06	0.00431565
513.519993	350.791652	7.91688E-06	0.004065476
513.559993	386.29811	8.71821E-06	0.004477324
513.599993	453.65376	1.02383E-05	0.005258408
513.639993	493.618634	1.11403E-05	0.005722096
513.679993	511.68961	1.15481E-05	0.005932039
513.719993	548.033573	1.23684E-05	0.006353871
513.759993	560.237985	1.26438E-05	0.006495874
513.799993	553.148256	1.24838E-05	0.006414169
513.839993	529.948576	1.19602E-05	0.00614563
513.879993	535.256465	1.208E-05	0.006207667
513.919993	673.685101	1.52041E-05	0.007813709
513.959993	1050.152425	2.37005E-05	0.012181097
513.999993	1774.406506	4.00459E-05	0.020583584
514.039993	2918.942752	6.58765E-05	0.033863141
514.079993	4193.123567	9.4633E-05	0.048648911
514.119993	5287.212105	0.000119325	0.061347375
514.159993	6034.831208	0.000136198	0.070027426
514.199993	6334.706068	0.000142965	0.073512855
514.239993	6082.056229	0.000137264	0.070586401
514.279993	5182.266159	0.000116957	0.060148403
514.319993	3852.433591	8.69441E-05	0.044717068

514.359993	2533.240915	5.71717E-05	0.029406845
514.399993	1483.607331	3.3483E-05	0.01722363
514.439993	829.379758	1.87179E-05	0.00962926
514.479993	505.349285	1.1405E-05	0.005867659
514.519993	411.851067	9.2949E-06	0.004782414
514.559993	429.978644	9.70402E-06	0.0049933
514.599993	468.368233	1.05704E-05	0.005439537
514.639993	496.080165	1.11958E-05	0.005761826
514.679993	507.600741	1.14558E-05	0.005896093
514.719993	486.876646	1.09881E-05	0.005655809
514.759993	447.55014	1.01006E-05	0.005199376
514.799993	413.460487	9.33123E-06	0.004803716
514.839993	429.309834	9.68892E-06	0.004988246
514.879993	493.734189	1.11429E-05	0.005737253
514.919993	737.080895	1.66349E-05	0.008565637
514.959993	1352.174547	3.05167E-05	0.015714878
514.999993	2394.408961	5.40385E-05	0.027829814
515.039993	3936.836436	8.88489E-05	0.045760744
515.079993	5655.590233	0.000127639	0.065744189
515.119993	7013.493197	0.000158285	0.081535651
515.159993	7484.038673	0.000168904	0.087012753
515.199993	7053.200764	0.000159181	0.082010007
515.239993	6002.290072	0.000135463	0.069796123
515.279993	4666.396707	0.000105314	0.054266235
515.319993	3334.659134	7.52586E-05	0.038782267
515.359993	2120.399879	4.78545E-05	0.02466228
515.399993	1206.123738	2.72205E-05	0.014029462
515.439993	657.974909	1.48496E-05	0.007654066
515.479993	420.725915	9.4952E-06	0.004894585
515.519993	408.196199	9.21242E-06	0.004749187
515.559993	540.593292	1.22004E-05	0.006290058
515.599993	730.253813	1.64808E-05	0.008497506
515.639993	840.898651	1.89779E-05	0.00978577
515.679993	796.150161	1.7968E-05	0.009265738
515.719993	657.414546	1.48369E-05	0.007651702
515.759993	515.522629	1.16346E-05	0.006000675
515.799993	398.626804	8.99645E-06	0.00464037
515.839993	361.38504	8.15596E-06	0.004207169
515.879993	453.752586	1.02406E-05	0.005282902
515.919993	657.581956	1.48407E-05	0.007656618
515.959993	979.077226	2.20964E-05	0.011400863
515.999993	1622.156293	3.66098E-05	0.018890661
516.039993	2821.884812	6.3686E-05	0.032864528
516.079993	4799.53647	0.000108319	0.055901193
516.119993	7668.239522	0.000173061	0.089320492
516.159993	11344.73595	0.000256035	0.132154966

516.199993	14960.76169	0.000337644	0.1742916
516.239993	17084.64836	0.000385577	0.199050121
516.279993	16540.79111	0.000373303	0.192728672
516.319993	13427.96959	0.000303051	0.156471068
516.359993	9085.570699	0.000205049	0.105878933
516.399993	5189.689682	0.000117124	0.060482868
516.439993	2590.394813	5.84616E-05	0.03019191
516.479993	1232.254679	2.78103E-05	0.014363449
516.519993	673.174256	1.51926E-05	0.007847284
516.559993	555.915676	1.25462E-05	0.006480887
516.599993	571.419634	1.28961E-05	0.006662149
516.639993	576.974959	1.30215E-05	0.006727439
516.679993	573.10124	1.29341E-05	0.006682789
516.719993	517.291987	1.16746E-05	0.006032478
516.759993	440.87694	9.94998E-06	0.005141751
516.799993	426.173223	9.61814E-06	0.004970653
516.839993	429.37811	9.69047E-06	0.00500842
516.879993	479.049723	1.08115E-05	0.00558824
516.919993	710.443579	1.60337E-05	0.008288151
516.959993	1307.055369	2.94984E-05	0.015249503
516.999993	2401.28942	5.41938E-05	0.028018172
517.039993	3952.024078	8.91917E-05	0.046115665
517.079993	5636.900047	0.000127217	0.065781356
517.119993	7133.134585	0.000160985	0.083248517
517.159993	8294.442248	0.000187194	0.096809252
517.199993	8905.434115	0.000200983	0.103948532
517.239993	8838.538741	0.000199474	0.103175676
517.279993	7939.309588	0.000179179	0.092685794
517.319993	6153.697311	0.00013888	0.071845596
517.359993	4094.790065	9.24137E-05	0.047811155
517.399993	2374.867141	5.35974E-05	0.027731317
517.439993	1236.635519	2.79091E-05	0.014441306
517.479993	665.564202	1.50209E-05	0.007772993
517.519993	478.649768	1.08025E-05	0.005590488
517.559993	446.253356	1.00713E-05	0.005212511
517.599993	474.378588	1.07061E-05	0.005541459
517.639993	510.789551	1.15278E-05	0.005967255
517.679993	491.856198	1.11005E-05	0.005746512
517.719993	413.053223	9.32204E-06	0.004826204
517.759993	353.908822	7.98723E-06	0.004135468
517.799993	329.117864	7.42773E-06	0.003846079
517.839993	297.064029	6.70432E-06	0.003471766
517.879993	279.646334	6.31123E-06	0.003268459
517.919993	364.253857	8.2207E-06	0.004257666
517.959993	619.300969	1.39768E-05	0.007239403
517.999993	1073.581061	2.42292E-05	0.012550741

518.039993	1732.381358	3.90974E-05	0.020254035
518.079993	2528.274037	5.70596E-05	0.029561449
518.119993	3324.469332	7.50286E-05	0.03887384
518.159993	3962.415707	8.94262E-05	0.046337081
518.199993	4328.825092	9.76956E-05	0.050625835
518.239993	4163.027818	9.39537E-05	0.048690585
518.279993	3592.477186	8.10772E-05	0.042020694
518.319993	2764.267745	6.23857E-05	0.032335741
518.359993	1861.008244	4.20004E-05	0.021771308
518.399993	1102.319005	2.48778E-05	0.012896654
518.439993	629.009063	1.41959E-05	0.007359701
518.479993	409.148989	9.23392E-06	0.004787604
518.519993	353.660442	7.98162E-06	0.004138631
518.559993	403.597038	9.10862E-06	0.004723367
518.599993	477.433906	1.0775E-05	0.005587924
518.639993	538.888047	1.2162E-05	0.006307675
518.679993	571.788897	1.29045E-05	0.006693295
518.719993	566.355403	1.27819E-05	0.006630202
518.759993	531.634887	1.19983E-05	0.006224217
518.799993	527.788486	1.19115E-05	0.006179661
518.839993	657.735209	1.48442E-05	0.007701748
518.879993	1115.299994	2.51708E-05	0.013060608
518.919993	2339.863789	5.28075E-05	0.02740285
518.959993	4861.179219	0.00010971	0.056935124
518.999993	8599.546342	0.00019408	0.100727406
519.039993	12856.06209	0.000290143	0.150596036
519.079993	16209.94384	0.000365836	0.189898072
519.119993	17292.82738	0.000390275	0.202599569
519.159993	15637.07486	0.000352907	0.183215195
519.199993	12550.18548	0.00028324	0.147058313
519.239993	9361.773886	0.000211282	0.109706166
519.279993	6586.191089	0.000148641	0.07718638
519.319993	4322.744648	9.75583E-05	0.050663989
519.359993	2607.955884	5.88579E-05	0.030568455
519.399993	1457.070711	3.28841E-05	0.017079979
519.439993	801.142912	1.80807E-05	0.009391828
519.479993	494.455209	1.11592E-05	0.005796963
519.519993	407.149746	9.1888E-06	0.004773767
519.559993	419.808183	9.47449E-06	0.004922564
519.599993	458.355339	1.03444E-05	0.005374972
519.639993	468.829136	1.05808E-05	0.005498218
519.679993	428.483284	9.67027E-06	0.005025446
519.719993	384.640949	8.68081E-06	0.004511591
519.759993	347.276753	7.83755E-06	0.004073646
519.799993	318.020621	7.17728E-06	0.003730751
519.839993	355.891907	8.03198E-06	0.004175347

519.879993	457.322118	1.03211E-05	0.005365745
519.919993	738.663878	1.66706E-05	0.008667386
519.959993	1463.382611	3.30265E-05	0.017172462
519.999993	2609.136091	5.88846E-05	0.030619975
520.039993	4048.1208	9.13604E-05	0.047511087
520.079993	5381.666577	0.000121457	0.063167209
520.119993	6033.34929	0.000136164	0.070821769
520.159993	6054.323512	0.000136638	0.071073438
520.199993	5546.515766	0.000125177	0.065117145
520.239993	4744.724931	0.000107082	0.055708252
520.279993	3862.96552	8.71818E-05	0.045358921
520.319993	3039.036828	6.85868E-05	0.035687098
520.359993	2293.540037	5.1762E-05	0.026934876
520.399993	1619.302349	3.65454E-05	0.019018225
520.439993	1078.495294	2.43401E-05	0.012667581
520.479993	715.084174	1.61385E-05	0.008399742
520.519993	559.367154	1.26241E-05	0.006571116
520.559993	569.854857	1.28608E-05	0.006694834
520.599993	619.53929	1.39821E-05	0.007279101
520.639993	635.583728	1.43442E-05	0.007468185
520.679993	613.466352	1.38451E-05	0.007208857
520.719993	576.823017	1.30181E-05	0.006778781
520.759993	549.682199	1.24056E-05	0.00646032
520.799993	552.820935	1.24764E-05	0.006497708
520.839993	620.00817	1.39927E-05	0.007287969
520.879993	801.75794	1.80946E-05	0.009425094
520.919993	1269.778735	2.86571E-05	0.014928076
520.959993	2243.790183	5.06392E-05	0.026381007
520.999993	3752.615327	8.46913E-05	0.044124167
521.039993	5442.10571	0.000122821	0.06399452
521.079993	6991.630815	0.000157791	0.082221925
521.119993	7974.585596	0.000179975	0.093788721
521.159993	8193.347242	0.000184912	0.096368963
521.199993	7707.556086	0.000173949	0.090662116
521.239993	6682.042945	0.000150804	0.078605285
521.279993	5375.93537	0.000121327	0.063245532
521.319993	3875.833054	8.74722E-05	0.045600982
521.359993	2493.868216	5.62831E-05	0.029343773
521.399993	1455.454973	3.28476E-05	0.017126734
521.439993	840.398551	1.89666E-05	0.009889957
521.479993	531.183532	1.19881E-05	0.00625154
521.519993	420.30852	9.48578E-06	0.004947023
521.559993	470.54486	1.06195E-05	0.005538728
521.599993	567.803003	1.28145E-05	0.006684055
521.639993	600.612607	1.3555E-05	0.007070825
521.679993	554.30275	1.25098E-05	0.006526134

521.719993	482.290414	1.08846E-05	0.005678725
521.759993	432.327295	9.75702E-06	0.005090825
521.799993	396.763857	8.95441E-06	0.00467241
521.839993	378.65471	8.54571E-06	0.004459493
521.879993	432.55585	9.76218E-06	0.005094688
521.919993	603.879903	1.36287E-05	0.007113106
521.959993	911.821615	2.05785E-05	0.010741177
521.999993	1402.723004	3.16575E-05	0.016525217
522.039993	2121.439778	4.78779E-05	0.0249942
522.079993	2943.046358	6.64205E-05	0.034676792
522.119993	3761.187826	8.48848E-05	0.044320036
522.159993	4327.495268	9.76655E-05	0.050997038
522.199993	4455.242802	0.000100549	0.052506491
522.239993	4057.993705	9.15833E-05	0.047828444
522.279993	3322.506817	7.49844E-05	0.039162826
522.319993	2500.289505	5.64281E-05	0.029473499
522.359993	1746.550193	3.94172E-05	0.020589971
522.399993	1171.521126	2.64396E-05	0.013812047
522.439993	775.192893	1.7495E-05	0.0091401
522.479993	516.443868	1.16554E-05	0.006089723
522.519993	406.770377	9.18024E-06	0.004796859
522.559993	424.390997	9.57791E-06	0.005005035
522.599993	480.644411	1.08475E-05	0.00566889
522.639993	536.833347	1.21156E-05	0.006332087
522.679993	578.341322	1.30524E-05	0.006822206
522.719993	553.01051	1.24807E-05	0.006523899
522.759993	493.996051	1.11488E-05	0.005828148
522.799993	440.975034	9.95219E-06	0.005203006
522.839993	409.9136	9.25118E-06	0.004836886
522.879993	437.405367	9.87163E-06	0.005161678
522.919993	590.991296	1.33379E-05	0.006974629
522.959993	965.15393	2.17822E-05	0.011391209
522.999993	1610.975105	3.63575E-05	0.019014954
523.039993	2534.130133	5.71918E-05	0.029913592
523.079993	3624.41876	8.17981E-05	0.042786941
523.119993	4564.629522	0.000103017	0.053890425
523.159993	5256.311778	0.000118628	0.062061233
523.199993	5420.441292	0.000122332	0.064004002
523.239993	4848.686835	0.000109428	0.057257163
523.279993	3894.294357	8.78888E-05	0.04599045
523.319993	2836.024254	6.40051E-05	0.033495157
523.359993	1862.821377	4.20413E-05	0.022002726
523.399993	1146.607716	2.58773E-05	0.013544199
523.439993	713.477451	1.61022E-05	0.008428531
523.479993	497.572087	1.12295E-05	0.005878423
523.519993	415.902712	9.38634E-06	0.004913939

523.559993	427.530795	9.64877E-06	0.005051712
523.599993	457.860977	1.03333E-05	0.005410508
523.639993	463.384373	1.04579E-05	0.005476196
523.679993	472.915967	1.06731E-05	0.005589265
523.719993	471.149549	1.06332E-05	0.005568814
523.759993	404.749355	9.13463E-06	0.004784353
523.799993	328.353369	7.41048E-06	0.003881608
523.839993	284.070323	6.41107E-06	0.003358376
523.879993	298.687164	6.74095E-06	0.003531451
523.919993	374.461312	8.45107E-06	0.004427685
523.959993	555.945879	1.25469E-05	0.006574086
523.999993	932.152719	2.10374E-05	0.011023591
524.039993	1512.310877	3.41308E-05	0.017885878
524.079993	2236.721174	5.04797E-05	0.026455391
524.119993	2995.509961	6.76045E-05	0.035432863
524.159993	3482.306167	7.85908E-05	0.041194153
524.199993	3620.167624	8.17021E-05	0.042828262
524.239993	3412.114305	7.70067E-05	0.040369975
524.279993	2851.10153	6.43454E-05	0.033735001
524.319993	2117.243027	4.77832E-05	0.025053701
524.359993	1417.517877	3.19914E-05	0.016775012
524.399993	887.109769	2.00208E-05	0.010498924
524.439993	569.541626	1.28538E-05	0.006741026
524.479993	401.165616	9.05375E-06	0.00474851
524.519993	358.17275	8.08346E-06	0.004239936
524.559993	417.689415	9.42667E-06	0.004944853
524.599993	499.291893	1.12683E-05	0.005911362
524.639993	521.775845	1.17758E-05	0.006178032
524.679993	519.516596	1.17248E-05	0.00615175
524.719993	526.824983	1.18897E-05	0.006238766
524.759993	499.093255	1.12638E-05	0.005910812
524.799993	470.692031	1.06229E-05	0.005574879
524.839993	437.85812	9.88185E-06	0.005186389
524.879993	456.97374	1.03133E-05	0.005413224
524.919993	542.924592	1.22531E-05	0.006431872
524.959993	718.871475	1.62239E-05	0.008516913
524.999993	1170.688375	2.64208E-05	0.013870923
525.039993	1937.252993	4.37211E-05	0.022955327
525.079993	2857.580294	6.44916E-05	0.033863253
525.119993	3788.354535	8.54979E-05	0.044896649
525.159993	4456.905035	0.000100586	0.052823816
525.199993	4697.969603	0.000106027	0.055685185
525.239993	4339.377103	9.79337E-05	0.051438694
525.279993	3615.014639	8.15858E-05	0.042855412
525.319993	2750.384186	6.20723E-05	0.032607841
525.359993	1867.282381	4.2142E-05	0.022139701

525.399993	1107.440479	2.49934E-05	0.013131526
525.439993	644.654888	1.4549E-05	0.007644607
525.479993	423.480234	9.55736E-06	0.005022201
525.519993	342.780895	7.73609E-06	0.004065469
525.559993	353.833099	7.98552E-06	0.00419687
525.599993	406.753151	9.17985E-06	0.00482493
525.639993	438.927408	9.90598E-06	0.005206979
525.679993	450.264535	1.01618E-05	0.005341878
525.719993	424.801599	9.58718E-06	0.005040172
525.759993	383.944452	8.66509E-06	0.004555758
525.799993	330.005554	7.44777E-06	0.003916035
525.839993	297.186181	6.70708E-06	0.00352685
525.879993	309.687078	6.98921E-06	0.003675484
525.919993	398.459985	8.99269E-06	0.004729434
525.959993	574.462153	1.29648E-05	0.006818972
525.999993	850.936428	1.92044E-05	0.010101541
526.039993	1326.850819	2.99452E-05	0.01575236
526.079993	1989.861743	4.49084E-05	0.023625415
526.119993	2650.730611	5.98233E-05	0.031474233
526.159993	3204.313246	7.23169E-05	0.038050253
526.199993	3379.838965	7.62783E-05	0.040137619
526.239993	3116.706744	7.03397E-05	0.037015579
526.279993	2596.863923	5.86076E-05	0.030844008
526.319993	1928.435689	4.35221E-05	0.022906555
526.359993	1248.638028	2.818E-05	0.014832836
526.399993	750.103288	1.69288E-05	0.008911313
526.439993	475.378068	1.07286E-05	0.005647975
526.479993	343.236452	7.74637E-06	0.004078308
526.519993	334.204416	7.54253E-06	0.003971292
526.559993	397.767166	8.97705E-06	0.004726956
526.599993	460.805152	1.03997E-05	0.005476498
526.639993	518.537381	1.17027E-05	0.006163092
526.679993	507.567425	1.14551E-05	0.006033167
526.719993	470.126461	1.06101E-05	0.005588551
526.759993	419.259755	9.46211E-06	0.00498426
526.799993	362.072906	8.17148E-06	0.004304736
526.839993	347.022776	7.83182E-06	0.004126116
526.879993	385.253798	8.69464E-06	0.004581033
526.919993	526.96723	1.18929E-05	0.006266615
526.959993	874.367605	1.97333E-05	0.010398638
526.999993	1573.807849	3.55187E-05	0.018718329
527.039993	2632.202009	5.94051E-05	0.031308881
527.079993	3906.027976	8.81536E-05	0.046464005
527.119993	5138.258848	0.000115963	0.061126598
527.159993	5979.650332	0.000134952	0.071141495
527.199993	6117.633433	0.000138066	0.07278864

527.239993	5460.775649	0.000123242	0.064978164
527.279993	4328.349082	9.76848E-05	0.051507246
527.319993	3075.467161	6.9409E-05	0.036600759
527.359993	1939.346914	4.37684E-05	0.023081681
527.399993	1098.41131	2.47896E-05	0.013074042
527.439993	622.574672	1.40506E-05	0.007410871
527.479993	395.411748	8.92389E-06	0.004707175
527.519993	299.414283	6.75736E-06	0.003564644
527.559993	310.784007	7.01396E-06	0.003700286
527.599993	426.338864	9.62187E-06	0.005076501
527.639993	518.803098	1.17087E-05	0.006177959
527.679993	505.204383	1.14018E-05	0.00601648
527.719993	436.144217	9.84317E-06	0.005194436
527.759993	369.316492	8.33496E-06	0.004398858
527.799993	321.046174	7.24556E-06	0.003824209
527.839993	273.697888	6.17698E-06	0.003260457
527.879993	275.119686	6.20907E-06	0.003277643
527.919993	323.824174	7.30826E-06	0.003858177
527.959993	491.173901	1.10851E-05	0.005852495
527.999993	835.763945	1.8862E-05	0.009959151
528.039993	1441.949999	3.25428E-05	0.017183901
528.079993	2241.665551	5.05913E-05	0.026716237
528.119993	3139.708823	7.08589E-05	0.037421978
528.159993	3921.554149	8.8504E-05	0.04674428
528.199993	4384.791619	9.89586E-05	0.052269953
528.239993	4237.015805	9.56235E-05	0.050512181
528.279993	3544.064585	7.99846E-05	0.042254265
528.319993	2597.160478	5.86143E-05	0.030967104
528.359993	1657.531701	3.74082E-05	0.019764987
528.399993	949.497939	2.14288E-05	0.011323002
528.439993	556.47709	1.25589E-05	0.006636632
528.479993	373.92562	8.43898E-06	0.004459832
528.519993	310.924454	7.01713E-06	0.003708694
528.559993	356.057387	8.03572E-06	0.00424736
528.599993	450.881153	1.01758E-05	0.005378906
528.639993	522.262579	1.17867E-05	0.006230942
528.679993	541.027524	1.22102E-05	0.006455308
528.719993	499.858988	1.12811E-05	0.005964554
528.759993	433.953363	9.79372E-06	0.005178529
528.799993	366.6617	8.27504E-06	0.004375843
528.839993	342.731969	7.73498E-06	0.004090568
528.879993	364.601938	8.22856E-06	0.00435192
528.919993	475.648144	1.07347E-05	0.005677806
528.959993	738.994256	1.66781E-05	0.008822032
528.999993	1257.379918	2.83773E-05	0.015011599
529.039993	2163.20053	4.88204E-05	0.025827956

529.079993	3233.257547	7.29701E-05	0.03860703
529.119993	4165.817765	9.40167E-05	0.049746118
529.159993	4835.413999	0.000109129	0.057746467
529.199993	5112.059353	0.000115372	0.061054893
529.239993	4829.925846	0.000109005	0.057689646
529.279993	4103.524526	9.26108E-05	0.049017061
529.319993	3075.969479	6.94203E-05	0.036745577
529.359993	2034.169959	4.59084E-05	0.024302061
529.399993	1199.840643	2.70787E-05	0.014335481
529.439993	691.26115	1.56008E-05	0.008259688
529.479993	429.320455	9.68916E-06	0.005130219
529.519993	365.651169	8.25224E-06	0.004369725
529.559993	406.208705	9.16756E-06	0.004854775
529.599993	475.684878	1.07355E-05	0.005685545
529.639993	508.799559	1.14829E-05	0.006081802
529.679993	470.161002	1.06109E-05	0.00562037
529.719993	398.080338	8.98412E-06	0.004759067
529.759993	346.534165	7.82079E-06	0.004143143
529.799993	332.495295	7.50396E-06	0.003975596
529.839993	343.748006	7.75791E-06	0.004110453
529.879993	377.227255	8.51349E-06	0.00451113
529.919993	451.105048	1.01808E-05	0.005395016
529.959993	661.033646	1.49186E-05	0.007906266
529.999993	1121.323861	2.53067E-05	0.01341256
530.039993	1903.309946	4.29551E-05	0.022767897
530.079993	3090.542938	6.97492E-05	0.036972682
530.119993	4632.956693	0.000104559	0.055429017
530.159993	6425.715347	0.000145019	0.076883508
530.199993	7870.032626	0.000177616	0.094171829
530.239993	8265.256957	0.000186535	0.098908496
530.279993	7373.794131	0.000166416	0.088247215
530.319993	5621.714529	0.000126874	0.06728396
530.359993	3642.054752	8.21961E-05	0.043593525
530.399993	2031.534306	4.58489E-05	0.024318256
530.439993	1033.760338	2.33305E-05	0.012375447
530.479993	572.072119	1.29109E-05	0.006848959
530.519993	409.407857	9.23977E-06	0.00490188
530.559993	380.420772	8.58557E-06	0.004555158
530.599993	421.197414	9.50584E-06	0.005043798
530.639993	491.530862	1.10932E-05	0.005886478
530.679993	518.427112	1.17002E-05	0.00620905
530.719993	503.871221	1.13717E-05	0.006035173
530.759993	429.932316	9.70297E-06	0.00514995
530.799993	342.827811	7.73715E-06	0.004106877
530.839993	310.920307	7.01704E-06	0.003724925
530.879993	367.28862	8.28919E-06	0.004400566

530.919993	521.026578	1.17588E-05	0.006243005
530.959993	882.400855	1.99146E-05	0.010573834
530.999993	1557.023767	3.51399E-05	0.018659264
531.039993	2535.040682	5.72123E-05	0.030382039
531.079993	3741.281177	8.44355E-05	0.044842007
531.119993	4901.557286	0.000110621	0.058753195
531.159993	5860.469599	0.000132263	0.070252621
531.199993	6385.678571	0.000144116	0.07655435
531.239993	6121.559295	0.000138155	0.073393497
531.279993	5200.787463	0.000117375	0.062358739
531.319993	3902.755349	8.80798E-05	0.046798533
531.359993	2557.011298	5.77082E-05	0.03066382
531.399993	1499.422958	3.38399E-05	0.017982516
531.439993	848.684065	1.91536E-05	0.010178998
531.479993	523.409858	1.18126E-05	0.006278177
531.519993	443.969786	1.00198E-05	0.005325713
531.559993	484.885016	1.09432E-05	0.005816956
531.599993	485.812672	1.09641E-05	0.005828524
531.639993	460.491874	1.03927E-05	0.005525154
531.679993	455.677337	1.0284E-05	0.005467799
531.719993	458.216803	1.03413E-05	0.005498684
531.759993	424.957141	9.59069E-06	0.005099946
531.799993	381.556793	8.61121E-06	0.004579439
531.839993	355.180595	8.01593E-06	0.004263193
531.879993	337.777123	7.62316E-06	0.004054606
531.919993	396.293766	8.9438E-06	0.004757385
531.959993	589.571721	1.33058E-05	0.00707816
531.999993	997.363916	2.25091E-05	0.011974849
532.039993	1687.015893	3.80736E-05	0.020256677
532.079993	2578.916103	5.82025E-05	0.030968409
532.119993	3491.208408	7.87917E-05	0.041926645
532.159993	4286.586493	9.67423E-05	0.051482374
532.199993	4761.073203	0.000107451	0.05718531
532.239993	4750.846967	0.00010722	0.057066771
532.279993	4098.812826	9.25045E-05	0.049238293
532.319993	3057.183697	6.89964E-05	0.036728151
532.359993	2007.865743	4.53147E-05	0.024123751
532.399993	1171.37179	2.64362E-05	0.014074649
532.439993	650.698989	1.46854E-05	0.007819078
532.479993	404.762786	9.13493E-06	0.004864169
532.519993	350.988761	7.92133E-06	0.004218265
532.559993	406.237287	9.16821E-06	0.004882622
532.599993	487.363424	1.09991E-05	0.005858128
532.639993	529.059883	1.19401E-05	0.006359799
532.679993	502.964022	1.13512E-05	0.006046556
532.719993	449.399488	1.01423E-05	0.005403017

532.759993	413.256771	9.32663E-06	0.004968855
532.799993	382.457622	8.63154E-06	0.004598882
532.839993	372.742842	8.41229E-06	0.004482403
532.879993	451.601859	1.0192E-05	0.005431126
532.919993	673.296661	1.51954E-05	0.008097915
532.959993	1130.147537	2.55059E-05	0.013593601
532.999993	1986.806148	4.48394E-05	0.023899425
533.039993	3107.847542	7.01398E-05	0.037387313
533.079993	4207.511102	9.49577E-05	0.050620031
533.119993	5078.435612	0.000114613	0.061102598
533.159993	5563.219792	0.000125554	0.066940435
533.199993	5626.07123	0.000126973	0.067701785
533.239993	5130.160649	0.000115781	0.061738836
533.279993	4241.496034	9.57247E-05	0.051048045
533.319993	3159.74452	7.1311E-05	0.038031599
533.359993	2129.066062	4.80501E-05	0.025627977
533.399993	1278.082813	2.88445E-05	0.015385682
533.439993	711.425156	1.60559E-05	0.008564846
533.479993	437.425211	9.87208E-06	0.005266556
533.519993	350.122604	7.90178E-06	0.004215757
533.559993	385.973122	8.71088E-06	0.004647775
533.599993	446.467164	1.00761E-05	0.005376629
533.639993	480.910115	1.08535E-05	0.005791846
533.679993	478.39061	1.07966E-05	0.005761935
533.719993	433.126379	9.77506E-06	0.005217144
533.759993	386.626325	8.72562E-06	0.004657386
533.799993	324.456356	7.32253E-06	0.003908765
533.839993	280.052847	6.3204E-06	0.003374084
533.879993	286.822098	6.47318E-06	0.003455899
533.919993	400.016193	9.02781E-06	0.004820127
533.959993	615.850722	1.38989E-05	0.007421453
533.999993	944.652302	2.13195E-05	0.011384606
534.039993	1511.997925	3.41237E-05	0.018223414
534.079993	2162.823221	4.88119E-05	0.026069463
534.119993	2741.103115	6.18629E-05	0.033042201
534.159993	3199.689187	7.22125E-05	0.038573044
534.199993	3366.094408	7.59681E-05	0.040582138
534.239993	3169.945954	7.15413E-05	0.038220205
534.279993	2727.31613	6.15517E-05	0.032885856
534.319993	2102.670322	4.74543E-05	0.025355803
534.359993	1435.110178	3.23884E-05	0.017307085
534.399993	887.02163	2.00188E-05	0.010698069
534.439993	542.352161	1.22401E-05	0.006541617
534.479993	361.752611	8.16425E-06	0.00436363
534.519993	305.504942	6.89482E-06	0.00368542
534.559993	334.147308	7.54124E-06	0.004031245

534.599993	420.854046	9.49809E-06	0.005077679
534.639993	511.136885	1.15356E-05	0.006167419
534.679993	558.151741	1.25967E-05	0.006735207
534.719993	508.493847	1.1476E-05	0.006136445
534.759993	439.846175	9.92672E-06	0.00530841
534.799993	472.632692	1.06667E-05	0.005704531
534.839993	671.1016	1.51458E-05	0.008100595
534.879993	1411.828108	3.1863E-05	0.017042878
534.919993	3108.32168	7.01505E-05	0.0375249
534.959993	5880.346024	0.000132711	0.070995193
534.999993	9202.884291	0.000207696	0.111117508
535.039993	12156.03285	0.000274345	0.146785402
535.079993	13594.93623	0.000306819	0.164172588
535.119993	13126.39601	0.000296244	0.158526341
535.159993	11369.85095	0.000256602	0.137322963
535.199993	9055.268564	0.000204365	0.109376039
535.239993	6839.533248	0.000154359	0.08261897
535.279993	4950.523385	0.000111726	0.059804916
535.319993	3372.649225	7.6116E-05	0.040746414
535.359993	2088.160896	4.71269E-05	0.025229848
535.399993	1172.743788	2.64672E-05	0.014170535
535.439993	630.011037	1.42185E-05	0.007613138
535.479993	390.325239	8.8091E-06	0.004717095
535.519993	375.304632	8.4701E-06	0.004535909
535.559992	430.396463	9.71345E-06	0.005202134
535.599992	486.855611	1.09877E-05	0.005884987
535.639992	519.352478	1.17211E-05	0.00627827
535.679992	507.803953	1.14604E-05	0.006139122
535.719992	487.91706	1.10116E-05	0.005899139
535.759992	445.457685	1.00534E-05	0.005386188
535.799992	396.566506	8.94995E-06	0.004795385
535.839992	396.353731	8.94515E-06	0.00479317
535.879992	503.812101	1.13703E-05	0.006093136
535.919992	857.01722	1.93417E-05	0.010365596
535.959992	1542.007809	3.4801E-05	0.018651927
535.999992	2575.80445	5.81323E-05	0.031158922
536.039992	3736.097981	8.43185E-05	0.045198103
536.079992	4581.96299	0.000103409	0.05543524
536.119992	5010.486207	0.00011308	0.060624285
536.159992	4970.485885	0.000112177	0.060144789
536.199992	4427.770309	9.99286E-05	0.053581719
536.239992	3670.918972	8.28475E-05	0.044426158
536.279992	2872.551953	6.48295E-05	0.034766762
536.319992	2072.153278	4.67656E-05	0.025081333
536.359992	1370.075819	3.09207E-05	0.016584628
536.399992	860.240783	1.94144E-05	0.010413903

536.439992	542.294667	1.22388E-05	0.006565401
536.479992	392.549555	8.8593E-06	0.004752835
536.519992	382.855391	8.64051E-06	0.004635808
536.559992	416.854141	9.40782E-06	0.005047858
536.599992	463.578078	1.04623E-05	0.005614076
536.639992	484.702296	1.09391E-05	0.005870335
536.679992	450.207036	1.01605E-05	0.005452962
536.719992	395.273328	8.92077E-06	0.004787955
536.759992	369.184348	8.33198E-06	0.004472272
536.799992	371.49489	8.38412E-06	0.004500597
536.839992	437.672302	9.87765E-06	0.00530272
536.879992	662.686285	1.49559E-05	0.008029527
536.919992	1092.23832	2.46503E-05	0.013235238
536.959992	1883.024332	4.24972E-05	0.022819316
536.999992	3096.482406	6.98833E-05	0.037527329
537.039992	4474.309802	0.000100979	0.054229729
537.079992	5654.097709	0.000127605	0.068534151
537.119992	6332.020526	0.000142905	0.07675707
537.159992	6369.048533	0.000143741	0.077211675
537.199992	5967.910898	0.000134687	0.07235409
537.239992	5157.608011	0.0001164	0.062534751
537.279992	4070.911848	9.18748E-05	0.049362497
537.319992	2937.279765	6.62903E-05	0.03561911
537.359992	1898.782559	4.28529E-05	0.023027422
537.399992	1088.991653	2.4577E-05	0.013207693
537.439992	600.090854	1.35432E-05	0.007278665
537.479992	388.809769	8.77489E-06	0.00471633
537.519992	356.356697	8.04247E-06	0.004322991
537.559992	382.566963	8.634E-06	0.004641295
537.599992	435.774958	9.83483E-06	0.005287207
537.639992	479.036083	1.08112E-05	0.005812521
537.679992	488.217248	1.10184E-05	0.005924364
537.719992	446.246003	1.00712E-05	0.005415459
537.759992	374.382065	8.44928E-06	0.004543686
537.799992	300.298022	6.77731E-06	0.003644836
537.839992	272.296257	6.14535E-06	0.003305214
537.879992	311.863095	7.03832E-06	0.003785769
537.919992	446.539633	1.00778E-05	0.005421038
537.959992	740.823006	1.67193E-05	0.008994338
537.999992	1226.196289	2.76735E-05	0.014888366
538.039992	1942.010478	4.38285E-05	0.02358147
538.079992	2821.990017	6.36884E-05	0.034269445
538.119992	3555.432902	8.02412E-05	0.043179377
538.159992	4043.111934	9.12474E-05	0.049105703
538.199992	4215.380421	9.51353E-05	0.051201799
538.239992	4097.60129	9.24772E-05	0.049774902

538.279992	3651.660533	8.24129E-05	0.044361211
538.319992	2903.155009	6.55202E-05	0.035270815
538.359992	2078.938691	4.69188E-05	0.025259178
538.399992	1327.601247	2.99621E-05	0.0161316
538.439992	789.482146	1.78175E-05	0.009593661
538.479992	481.15612	1.0859E-05	0.005847367
538.519992	379.49109	8.56459E-06	0.0046122
538.559992	389.272723	8.78534E-06	0.004731434
538.599992	441.93284	9.97381E-06	0.005371893
538.639992	500.117428	1.1287E-05	0.006079605
538.679992	509.762539	1.15046E-05	0.006197314
538.719992	480.347414	1.08408E-05	0.005840141
538.759992	443.569098	1.00107E-05	0.005393384
538.799992	395.234046	8.91988E-06	0.004806032
538.839992	388.545715	8.76894E-06	0.004725053
538.879992	423.620059	9.56051E-06	0.00515197
538.919992	561.364743	1.26692E-05	0.006827696
538.959992	906.442503	2.04571E-05	0.011025583
538.999992	1500.880481	3.38728E-05	0.018257429
539.039992	2300.298837	5.19145E-05	0.027984014
539.079992	3234.578502	7.29999E-05	0.039352802
539.119992	4026.422445	9.08707E-05	0.048990236
539.159992	4495.36686	0.000101454	0.054700029
539.199992	4579.794512	0.00010336	0.055731486
539.239992	4186.783731	9.44899E-05	0.050952721
539.279992	3472.324784	7.83655E-05	0.042260964
539.319992	2565.611989	5.79023E-05	0.031227862
539.359992	1744.652768	3.93744E-05	0.021236967
539.399992	1118.295978	2.52384E-05	0.013613583
539.439992	672.723868	1.51824E-05	0.008190016
539.479992	416.17	9.39238E-06	0.005067
539.519992	335.434815	7.5703E-06	0.004084326
539.559992	363.547291	8.20476E-06	0.004426958
539.599992	418.089138	9.43569E-06	0.005091498
539.639992	461.000951	1.04041E-05	0.005614495
539.679992	470.014264	1.06076E-05	0.005724692
539.719992	448.069655	1.01123E-05	0.005457815
539.759992	412.085334	9.30019E-06	0.005019872
539.799992	353.638459	7.98113E-06	0.004308213
539.839992	305.105895	6.88582E-06	0.003717238
539.879992	310.011128	6.99652E-06	0.003777281
539.919992	396.270872	8.94328E-06	0.004828657
539.959992	577.514885	1.30337E-05	0.00703768
539.999992	883.290968	1.99346E-05	0.010764709
540.039992	1384.399922	3.1244E-05	0.016872998
540.079992	2043.607669	4.61214E-05	0.024909234

540.119992	2697.81989	6.0886E-05	0.032885766
540.159992	3066.682827	6.92108E-05	0.037384883
540.199992	3064.719072	6.91664E-05	0.037363711
540.239992	2801.764377	6.32319E-05	0.034160412
540.279992	2356.394759	5.31805E-05	0.028732386
540.319992	1804.413467	4.07231E-05	0.022003504
540.359992	1211.9963	2.73531E-05	0.014780504
540.399992	757.530941	1.70964E-05	0.009238904
540.439992	494.755899	1.1166E-05	0.006034527
540.479992	364.44827	8.22509E-06	0.004445496
540.519992	338.172345	7.63208E-06	0.004125291
540.559992	390.426344	8.81138E-06	0.004763079
540.599992	442.585641	9.98854E-06	0.005399805
540.639992	474.510712	1.0709E-05	0.005789738
540.679992	479.410522	1.08196E-05	0.005849956
540.719992	449.483183	1.01442E-05	0.005485177
540.759992	387.272182	8.74019E-06	0.004726347
540.799992	317.822278	7.17281E-06	0.003879053
540.839992	290.718447	6.56111E-06	0.003548511
540.879992	311.997034	7.04134E-06	0.003808519
540.919992	401.329034	9.05744E-06	0.004899349
540.959992	671.502617	1.51549E-05	0.008198183
540.999992	1175.244966	2.65236E-05	0.01434929
541.039992	1904.518428	4.29823E-05	0.023255158
541.079992	2764.966953	6.24015E-05	0.033764178
541.119992	3465.494581	7.82114E-05	0.042321744
541.159992	3980.973995	8.9845E-05	0.04862054
541.199992	4185.784372	9.44673E-05	0.051125714
541.239992	3894.575507	8.78951E-05	0.047572367
541.279992	3273.870156	7.38867E-05	0.039993387
541.319992	2434.958907	5.49536E-05	0.029747499
541.359992	1612.560267	3.63932E-05	0.019701844
541.399992	968.132617	2.18494E-05	0.011829268
541.439992	545.292899	1.23065E-05	0.006663232
541.479992	335.213766	7.56531E-06	0.004096463
541.519992	286.779751	6.47222E-06	0.003504836
541.559992	325.651779	7.34951E-06	0.003980199
541.599992	406.579156	9.17593E-06	0.004969681
541.639992	486.661413	1.09833E-05	0.005948978
541.679992	497.290664	1.12232E-05	0.00607936
541.719992	450.781294	1.01735E-05	0.005511192
541.759992	395.264796	8.92058E-06	0.004832811
541.799992	353.895242	7.98692E-06	0.004327315
541.839992	311.941015	7.04007E-06	0.003814594
541.879992	327.445109	7.38998E-06	0.004004482
541.919992	404.780278	9.13533E-06	0.004950616

541.959992	546.084971	1.23244E-05	0.006679319
541.999992	854.480702	1.92844E-05	0.010452166
542.039992	1336.139562	3.01548E-05	0.016345112
542.079992	1974.732028	4.4567E-05	0.024158853
542.119992	2688.828033	6.06831E-05	0.032897524
542.159992	3180.465941	7.17787E-05	0.038915532
542.199992	3319.192779	7.49096E-05	0.040615962
542.239992	3150.744706	7.11079E-05	0.038557558
542.279992	2684.716105	6.05903E-05	0.032856909
542.319992	2046.082901	4.61772E-05	0.025042841
542.359992	1415.008712	3.19348E-05	0.017320145
542.399992	905.751427	2.04415E-05	0.011087496
542.439992	564.48098	1.27395E-05	0.006910441
542.479992	400.265061	9.03342E-06	0.004900452
542.519992	357.116224	8.05962E-06	0.004372503
542.559992	391.085773	8.82626E-06	0.004788776
542.599992	449.612368	1.01471E-05	0.00550583
542.639992	497.034843	1.12174E-05	0.006087001
542.679992	496.658761	1.12089E-05	0.006082844
542.719992	450.509007	1.01674E-05	0.00551803
542.759992	403.15958	9.09875E-06	0.004938438
542.799992	379.834349	8.57233E-06	0.004653062
542.839992	493.657189	1.11412E-05	0.006047865
542.879992	924.579339	2.08665E-05	0.011327988
542.919992	1813.001983	4.09169E-05	0.022214619
542.959992	3242.346342	7.31752E-05	0.039731228
542.999992	5071.043144	0.000114446	0.062144384
543.039992	6803.259366	0.00015354	0.083378408
543.079992	7931.808703	0.00017901	0.097216681
543.119992	8255.446425	0.000186314	0.101190819
543.159992	7850.723655	0.00017718	0.096237032
543.199992	7035.007051	0.00015877	0.086244031
543.239992	5993.68735	0.000135269	0.073483626
543.279992	4797.539609	0.000108274	0.058822982
543.319992	3579.197031	8.07775E-05	0.043888026
543.359992	2399.102393	5.41444E-05	0.0294199
543.399992	1428.653712	3.22427E-05	0.017520696
543.439992	820.772103	1.85237E-05	0.01006651
543.479992	504.165328	1.13783E-05	0.006183883
543.519992	396.577106	8.95019E-06	0.004864609
543.559992	413.597878	9.33433E-06	0.005073767
543.599992	479.614339	1.08242E-05	0.00588405
543.639992	543.410811	1.2264E-05	0.006667215
543.679992	555.85793	1.25449E-05	0.006820432
543.719992	510.441973	1.152E-05	0.006263635
543.759992	430.10444	9.70686E-06	0.005278201

543.799992	347.021974	7.8318E-06	0.004258934
543.839992	317.388005	7.16301E-06	0.003895529
543.879992	416.199545	9.39304E-06	0.005108689
543.919992	658.490756	1.48612E-05	0.008083314
543.959992	1079.797996	2.43695E-05	0.013256053
543.999992	1671.992014	3.77345E-05	0.020527584
544.039992	2442.759121	5.51297E-05	0.029992746
544.079992	3218.299377	7.26325E-05	0.039517909
544.119992	3795.27937	8.56542E-05	0.046606146
544.159992	4059.018407	9.16064E-05	0.049848533
544.199992	3904.827175	8.81265E-05	0.047958446
544.239992	3550.202329	8.01231E-05	0.043606207
544.279992	2951.505411	6.66114E-05	0.036255234
544.319992	2179.49769	4.91882E-05	0.026774135
544.359992	1461.899226	3.2993E-05	0.017960085
544.399992	924.890453	2.08735E-05	0.011363527
544.439992	579.979303	1.30893E-05	0.007126352
544.479992	392.748409	8.86378E-06	0.004826153
544.519992	368.236989	8.3106E-06	0.004525286
544.559992	403.42256	9.10469E-06	0.004958047
544.599992	480.155404	1.08364E-05	0.005901524
544.639992	542.735685	1.22488E-05	0.00667118
544.679992	511.940956	1.15538E-05	0.006293121
544.719992	488.881535	1.10334E-05	0.0060101
544.759992	446.697241	1.00813E-05	0.005491908
544.799992	380.651237	8.59077E-06	0.00468025
544.839992	333.129284	7.51826E-06	0.004096251
544.879992	386.656776	8.7263E-06	0.004754789
544.919992	590.812373	1.33338E-05	0.007265861
544.959992	1094.355207	2.46981E-05	0.013459462
544.999992	1907.686787	4.30538E-05	0.023464339
545.039992	2938.56522	6.63193E-05	0.036146684
545.079992	4125.559794	9.31081E-05	0.050751383
545.119992	5088.356477	0.000114837	0.062600009
545.159992	5585.746903	0.000126063	0.068724246
545.199992	5596.78887	0.000126312	0.068865154
545.239992	5064.931508	0.000114308	0.062325538
545.279992	4107.438793	9.26992E-05	0.050547004
545.319992	2990.712785	6.74962E-05	0.036807039
545.359992	1953.082479	4.40784E-05	0.024038569
545.399992	1169.45784	2.6393E-05	0.014394761
545.439992	696.820917	1.57263E-05	0.008577741
545.479992	441.526477	9.96464E-06	0.00543551
545.519992	332.057114	7.49407E-06	0.004088163
545.559992	340.766251	7.69062E-06	0.004195694
545.599992	402.512581	9.08415E-06	0.004956311

545.639992	440.415419	9.93956E-06	0.005423423
545.679992	434.13851	9.7979E-06	0.005346519
545.719992	395.592955	8.92798E-06	0.004872178
545.759992	336.225255	7.58814E-06	0.004141301
545.799992	283.584054	6.4001E-06	0.003493173
545.839992	267.914893	6.04647E-06	0.003300403
545.879992	290.847183	6.56402E-06	0.003583165
545.919992	367.622835	8.29674E-06	0.004529354
545.959992	569.144879	1.28448E-05	0.007012751
545.999992	1013.568639	2.28748E-05	0.012489658
546.039992	1697.510921	3.83105E-05	0.020919042
546.079992	2586.195063	5.83668E-05	0.031872953
546.119992	3600.957965	8.12686E-05	0.04438241
546.159992	4384.214421	9.89456E-05	0.054040135
546.199992	4759.770043	0.000107421	0.058673557
546.239992	4708.49373	0.000106264	0.058045726
546.279992	4072.282964	9.19058E-05	0.050206275
546.319992	3048.399162	6.87981E-05	0.03758579
546.359992	1995.43951	4.50343E-05	0.024604935
546.399992	1160.166276	2.61833E-05	0.014306575
546.439992	644.996104	1.45567E-05	0.007954343
546.479992	400.060381	9.02881E-06	0.004934062
546.519992	341.178254	7.69992E-06	0.004208159
546.559992	370.031262	8.35109E-06	0.004564372
546.599992	415.534456	9.37803E-06	0.005126033
546.639992	465.218906	1.04993E-05	0.005739361
546.679992	480.685533	1.08484E-05	0.005930605
546.719992	460.871083	1.04012E-05	0.005686554
546.759992	413.185501	9.32502E-06	0.005098549
546.799992	340.784056	7.69102E-06	0.00420545
546.839992	301.089734	6.79518E-06	0.003715874
546.879992	358.174742	8.08351E-06	0.004420707
546.919992	513.043247	1.15787E-05	0.006332607
546.959992	862.672671	1.94693E-05	0.010648939
546.999992	1530.827625	3.45486E-05	0.01889811
547.039992	2448.58468	5.52611E-05	0.030230057
547.079992	3464.2279	7.81828E-05	0.042772245
547.119992	4419.963236	9.97524E-05	0.05457654
547.159992	5163.859198	0.000116541	0.063766633
547.199992	5466.491927	0.000123371	0.06750867
547.239992	5197.2721	0.000117295	0.06418862
547.279992	4336.182017	9.78616E-05	0.053557689
547.319992	3269.664716	7.37918E-05	0.040387716
547.359992	2245.510615	5.0678E-05	0.027739135
547.399992	1322.136329	2.98388E-05	0.016333745
547.439992	682.07098	1.53934E-05	0.008426958

547.479992	400.574722	9.04041E-06	0.004949446
547.519992	356.58571	8.04764E-06	0.004406245
547.559992	381.363826	8.60685E-06	0.004712767
547.599992	423.659826	9.56141E-06	0.005235829
547.639992	458.59026	1.03497E-05	0.005667933
547.679992	457.939316	1.03351E-05	0.005660301
547.719992	419.827982	9.47493E-06	0.00518961
547.759992	350.192756	7.90336E-06	0.004329146
547.799992	287.606569	6.49088E-06	0.003555704
547.839992	254.486767	5.74341E-06	0.003146471
547.879992	309.650997	6.98839E-06	0.0038288
547.919992	388.8637	8.77611E-06	0.004808607
547.959992	536.07608	1.20985E-05	0.006629489
547.999992	800.995504	1.80774E-05	0.009906389
548.039992	1298.977895	2.93161E-05	0.016066407
548.079992	2085.894295	4.70757E-05	0.025801265
548.119992	2969.144577	6.70095E-05	0.036729223
548.159992	3729.713394	8.41744E-05	0.046141058
548.199992	4234.533926	9.55675E-05	0.052390121
548.239992	4126.631836	9.31323E-05	0.051058869
548.279992	3585.664774	8.09235E-05	0.044368714
548.319992	2823.261661	6.37171E-05	0.034937351
548.359992	1984.597282	4.47896E-05	0.024560823
548.399992	1244.643317	2.80899E-05	0.015404482
548.439992	736.133941	1.66135E-05	0.009111518
548.479992	479.422958	1.08199E-05	0.005934503
548.519992	405.762909	9.1575E-06	0.005023074
548.559992	404.46295	9.12817E-06	0.005007346
548.599992	446.995563	1.00881E-05	0.005534314
548.639992	486.587721	1.09816E-05	0.006024949
548.679992	487.579896	1.1004E-05	0.006037674
548.719992	447.449031	1.00983E-05	0.00554114
548.759992	399.941744	9.02613E-06	0.004953178
548.799992	386.520176	8.72322E-06	0.004787304
548.839992	400.756338	9.04451E-06	0.00496399
548.879992	434.010317	9.79501E-06	0.005376284
548.919992	584.365793	1.31883E-05	0.007239334
548.959992	933.878163	2.10763E-05	0.011570062
548.999992	1625.632615	3.66883E-05	0.020141857
549.039992	2617.309143	5.9069E-05	0.032431255
549.079992	3773.556258	8.51639E-05	0.046761798
549.119992	4930.794124	0.000111281	0.061106707
549.159992	5614.142898	0.000126703	0.06958043
549.199992	5764.071398	0.000130087	0.071443814
549.239992	5307.056822	0.000119773	0.065784056
549.279992	4332.898257	9.77875E-05	0.053712705

549.319992	3166.199178	7.14567E-05	0.039252597
549.359992	2027.57374	4.57595E-05	0.025138447
549.399992	1179.67539	2.66236E-05	0.014627022
549.439992	680.575079	1.53596E-05	0.008439196
549.479992	436.663967	9.8549E-06	0.005415069
549.519992	361.061385	8.14865E-06	0.004477847
549.559992	373.369558	8.42643E-06	0.004630829
549.599992	428.624264	9.67345E-06	0.005316529
549.639992	495.405507	1.11806E-05	0.006145312
549.679992	508.232361	1.14701E-05	0.006304883
549.719992	454.743424	1.02629E-05	0.005641735
549.759992	400.486863	9.03843E-06	0.004968968
549.799992	338.649911	7.64286E-06	0.004202043
549.839992	294.533127	6.6472E-06	0.003654898
549.879992	308.369415	6.95947E-06	0.003826872
549.919992	388.42649	8.76624E-06	0.004820733
549.959992	551.450724	1.24455E-05	0.006844513
549.999992	839.307093	1.8942E-05	0.010418095
550.039992	1328.46343	2.99816E-05	0.016491062
550.079992	1972.289861	4.45118E-05	0.02448507
550.119992	2579.819195	5.82229E-05	0.032029595
550.159992	3100.561301	6.99753E-05	0.038497638
550.199992	3308.775135	7.46744E-05	0.04108588
550.239992	3119.170849	7.03953E-05	0.038734332
550.279992	2668.967513	6.02349E-05	0.033146049
550.319992	2038.610701	4.60086E-05	0.025319455
550.359992	1404.254026	3.16921E-05	0.01744204
550.399992	907.369713	2.04781E-05	0.01127113
550.439992	571.002054	1.28867E-05	0.007093367
550.479992	394.252556	8.89773E-06	0.004898023
550.519992	351.862083	7.94104E-06	0.0043717
550.559992	401.293057	9.05663E-06	0.004986216
550.599992	463.93678	1.04704E-05	0.005765006
550.639992	476.313829	1.07497E-05	0.005919237
550.679992	469.088526	1.05867E-05	0.00582987
550.719992	445.727656	1.00595E-05	0.005539942
550.759992	400.169543	9.03127E-06	0.004974062
550.799992	347.42888	7.84099E-06	0.004318815
550.839992	361.995786	8.16974E-06	0.00450022
550.879992	495.218114	1.11764E-05	0.006156846
550.919992	787.174736	1.77654E-05	0.009787335
550.959992	1297.654014	2.92862E-05	0.016135549
550.999992	1998.546452	4.51044E-05	0.024852529
551.039992	2758.581502	6.22573E-05	0.034306285
551.079992	3484.282555	7.86354E-05	0.043334398
551.119992	4055.356996	9.15238E-05	0.050440573

551.159992	4428.559275	9.99464E-05	0.055086465
551.199992	4455.158493	0.000100547	0.055421352
551.239992	4007.961746	9.04541E-05	0.049861925
551.279992	3237.312819	7.30616E-05	0.040277421
551.319992	2372.449119	5.35429E-05	0.029519256
551.359992	1584.174276	3.57526E-05	0.019712557
551.399992	984.353125	2.22155E-05	0.012249615
551.439992	590.239424	1.33209E-05	0.007345667
551.479992	386.158482	8.71506E-06	0.004806181
551.519992	330.751359	7.4646E-06	0.004116875
551.559992	346.792198	7.82662E-06	0.004316849
551.599992	413.645747	9.33541E-06	0.005149411
551.639992	477.427106	1.07749E-05	0.005943846
551.679992	479.226251	1.08155E-05	0.005966678
551.719992	435.363808	9.82555E-06	0.005420955
551.759992	376.964233	8.50756E-06	0.00469413
551.799992	323.746182	7.3065E-06	0.004031727
551.839992	287.397034	6.48615E-06	0.003579317
551.879992	292.18133	6.59413E-06	0.003639166
551.919992	364.250591	8.22063E-06	0.004537129
551.959992	544.911294	1.22979E-05	0.006787943
551.999992	836.158589	1.88709E-05	0.010416756
552.039992	1242.342026	2.80379E-05	0.015478058
552.079992	1792.75399	4.046E-05	0.022337135
552.119992	2342.268592	5.28617E-05	0.029186023
552.159992	2746.828953	6.19921E-05	0.034229559
552.199992	2880.296681	6.50043E-05	0.035895365
552.239992	2771.703304	6.25535E-05	0.034544535
552.279992	2443.441575	5.51451E-05	0.03045552
552.319992	1926.713781	4.34832E-05	0.024016666
552.359992	1365.887874	3.08262E-05	0.017027152
552.399992	883.813081	1.99464E-05	0.011018408
552.439992	574.843549	1.29734E-05	0.007167035
552.479992	414.042357	9.34436E-06	0.005162572
552.519992	355.68012	8.0272E-06	0.004435191
552.559992	377.285414	8.51481E-06	0.004704941
552.599992	424.06897	9.57065E-06	0.005288739
552.639992	454.621916	1.02602E-05	0.005670188
552.679992	470.853569	1.06265E-05	0.005873059
552.719992	459.827027	1.03777E-05	0.005735938
552.759992	411.897218	9.29595E-06	0.005138427
552.799992	374.274019	8.44684E-06	0.004669415
552.839992	411.638375	9.2901E-06	0.005135941
552.879992	519.316958	1.17203E-05	0.006479897
552.919992	750.52053	1.69382E-05	0.009365471
552.959992	1141.194621	2.57552E-05	0.01424158

552.999992	1726.315183	3.89605E-05	0.021545172
553.039992	2445.934883	5.52013E-05	0.030528551
553.079992	3106.927467	7.0119E-05	0.03878143
553.119992	3595.815797	8.11526E-05	0.0448871
553.159992	3895.291548	8.79113E-05	0.048629017
553.199992	3934.233878	8.87902E-05	0.049118726
553.239992	3689.944677	8.32769E-05	0.046072118
553.279992	3061.602851	6.90961E-05	0.038229496
553.319992	2205.188103	4.9768E-05	0.027537643
553.359992	1474.799098	3.32842E-05	0.018418123
553.399992	947.984097	2.13947E-05	0.011839816
553.439992	575.410528	1.29862E-05	0.00718709
553.479992	382.572015	8.63412E-06	0.004778811
553.519992	345.196453	7.7906E-06	0.004312255
553.559992	369.490077	8.33888E-06	0.004616068
553.599992	422.728843	9.5404E-06	0.005281566
553.639992	434.324326	9.8021E-06	0.005426832
553.679992	437.044337	9.86348E-06	0.005461213
553.719992	405.977793	9.16235E-06	0.005073378
553.759992	362.632158	8.1841E-06	0.004532029
553.799992	330.416999	7.45705E-06	0.004129715
553.839992	317.464818	7.16474E-06	0.003968119
553.879992	346.315269	7.81585E-06	0.004329045
553.919992	442.133012	9.97833E-06	0.005527194
553.959992	589.691424	1.33085E-05	0.007372384
553.999992	842.432849	1.90125E-05	0.010532945
554.039992	1265.678497	2.85646E-05	0.015825931
554.079992	1833.296051	4.13749E-05	0.022925025
554.119992	2399.560959	5.41547E-05	0.030008229
554.159992	2810.714106	6.34339E-05	0.035152531
554.199992	3005.488076	6.78297E-05	0.037591208
554.239992	2893.307689	6.52979E-05	0.036190721
554.279992	2534.133682	5.71919E-05	0.031700308
554.319992	1997.980006	4.50916E-05	0.02499519
554.359992	1414.734465	3.19286E-05	0.017699931
554.399992	915.53969	2.06625E-05	0.011455265
554.439992	595.170552	1.34322E-05	0.007447332
554.479992	411.32474	9.28303E-06	0.005147252
554.519992	347.255291	7.83707E-06	0.004345811
554.559992	357.870425	8.07664E-06	0.00447898
554.599992	403.177317	9.09915E-06	0.005046389
554.639992	459.324924	1.03663E-05	0.005749578
554.679992	501.281233	1.13132E-05	0.006275216
554.719992	461.006154	1.04043E-05	0.005771455
554.759992	391.677588	8.83962E-06	0.004903866
554.799992	334.709087	7.55392E-06	0.004190914

554.839992	341.768001	7.71323E-06	0.004279607
554.879992	398.97864	9.00439E-06	0.004996357
554.919992	520.255487	1.17414E-05	0.006515561
554.959992	774.237714	1.74735E-05	0.009697075
554.999992	1224.503754	2.76353E-05	0.015337616
555.039992	1916.352191	4.32494E-05	0.024005146
555.079992	2695.034101	6.08232E-05	0.033761722
555.119992	3323.788553	7.50133E-05	0.04164137
555.159992	3702.15818	8.35526E-05	0.046385035
555.199992	3693.007873	8.3346E-05	0.046273723
555.239992	3385.110888	7.63972E-05	0.042418802
555.279992	2774.000342	6.26053E-05	0.034763484
555.319992	2040.077684	4.60417E-05	0.025567883
555.359992	1413.613242	3.19033E-05	0.017717806
555.399992	906.084331	2.04491E-05	0.011357408
555.439992	545.135658	1.2303E-05	0.006833552
555.479992	364.75614	8.23204E-06	0.004572732
555.519992	316.347092	7.13951E-06	0.003966142
555.559992	356.32915	8.04185E-06	0.004467732
555.599992	438.767596	9.90237E-06	0.005501759
555.639992	481.959521	1.08772E-05	0.006043782
555.679992	466.388593	1.05257E-05	0.005848944
555.719992	449.764514	1.01506E-05	0.005640868
555.759992	408.817507	9.22644E-06	0.005127687
555.799992	343.32148	7.74829E-06	0.004306498
555.839992	291.702649	6.58332E-06	0.003659274
555.879992	301.822026	6.8117E-06	0.003786489
555.919992	381.711034	8.61469E-06	0.004789076
555.959992	574.118727	1.29571E-05	0.007203607
555.999992	984.221693	2.22125E-05	0.012350157
556.039992	1735.918288	3.91773E-05	0.021784122
556.079992	2943.728534	6.64359E-05	0.036943648
556.119992	4675.229722	0.000105513	0.058678124
556.159992	6904.898559	0.000155834	0.086668604
556.199992	9088.20687	0.000205108	0.114081163
556.239992	10300.20424	0.000232461	0.129304251
556.279992	9849.260498	0.000222284	0.123652192
556.319992	7913.680347	0.000178601	0.099359164
556.359992	5371.619816	0.00012123	0.067447509
556.399992	3091.313246	6.97666E-05	0.038818155
556.439992	1552.336579	3.50341E-05	0.01949436
556.479992	766.017938	1.7288E-05	0.009620403
556.519992	494.549529	1.11613E-05	0.006211483
556.559992	439.612718	9.92145E-06	0.00552188
556.599992	474.249212	1.07031E-05	0.00595737
556.639992	466.451599	1.05272E-05	0.00585984

556.679992	442.750059	9.99225E-06	0.005562487
556.719992	450.241439	1.01613E-05	0.005657011
556.759992	429.818992	9.70042E-06	0.005400804
556.799992	367.308439	8.28964E-06	0.004615671
556.839992	312.247554	7.04699E-06	0.003924047
556.879992	335.066578	7.56199E-06	0.004211119
556.919992	473.280185	1.06813E-05	0.005948615
556.959992	781.308679	1.7633E-05	0.009820903
556.999992	1314.306099	2.96621E-05	0.016521766
557.039992	2117.921593	4.77985E-05	0.026625699
557.079992	3079.276907	6.9495E-05	0.038714269
557.119992	4068.572714	9.1822E-05	0.051155883
557.159992	4996.481499	0.000112764	0.062827382
557.199992	5716.308579	0.000129009	0.071883882
557.239992	5862.476498	0.000132308	0.073727269
557.279992	5293.805515	0.000119474	0.066580368
557.319992	4130.480431	9.32192E-05	0.051952918
557.359992	2750.263942	6.20696E-05	0.034595126
557.399992	1611.126571	3.63609E-05	0.020267556
557.439992	898.394751	2.02755E-05	0.011302385
557.479992	548.205665	1.23722E-05	0.006897275
557.519992	420.275483	9.48503E-06	0.005288095
557.559992	397.19658	8.96417E-06	0.004998065
557.599992	414.968106	9.36525E-06	0.005222065
557.639992	469.197441	1.05891E-05	0.005904924
557.679992	499.326119	1.12691E-05	0.006284549
557.719992	467.216885	1.05444E-05	0.005880842
557.759992	429.273954	9.68812E-06	0.005403643
557.799992	368.325148	8.31259E-06	0.00463676
557.839992	331.568558	7.48304E-06	0.004174339
557.879992	335.381388	7.56909E-06	0.004222644
557.919992	372.314158	8.40261E-06	0.004687985
557.959992	491.878109	1.1101E-05	0.006193916
557.999992	762.67101	1.72124E-05	0.009604532
558.039992	1276.865566	2.88171E-05	0.016081082
558.079992	1945.847555	4.39151E-05	0.024508121
558.119992	2637.728464	5.95299E-05	0.033224804
558.159992	3238.426735	7.30868E-05	0.040794117
558.199992	3536.007618	7.98028E-05	0.044545904
558.239992	3424.706384	7.72909E-05	0.043146845
558.279992	2923.792372	6.59859E-05	0.03683862
558.319992	2250.77211	5.07968E-05	0.028360864
558.359992	1539.062349	3.47345E-05	0.019394352
558.399992	954.138024	2.15336E-05	0.012024343
558.439992	582.612184	1.31487E-05	0.007342785
558.479992	392.126404	8.84975E-06	0.004942406

558.519992	351.240249	7.927E-06	0.00442739
558.559992	385.794073	8.70683E-06	0.00486329
558.599992	435.39131	9.82618E-06	0.005488902
558.639992	490.735268	1.10752E-05	0.006187056
558.679992	515.025306	1.16234E-05	0.006493763
558.719992	481.417195	1.08649E-05	0.006070446
558.759992	399.872432	9.02456E-06	0.005042565
558.799992	324.882518	7.33215E-06	0.004097203
558.839992	308.633879	6.96544E-06	0.003892565
558.879992	365.884965	8.25751E-06	0.004614959
558.919992	534.941282	1.20729E-05	0.006747774
558.959992	864.271302	1.95054E-05	0.010902738
558.999992	1438.383277	3.24623E-05	0.018146429
559.039992	2255.927326	5.09131E-05	0.02846248
559.079992	3296.733085	7.44027E-05	0.041597046
559.119992	4535.958112	0.00010237	0.057237257
559.159992	5663.505525	0.000127817	0.071470394
559.199992	6278.381123	0.000141694	0.079235461
559.239992	6155.090647	0.000138912	0.077685047
559.279992	5320.34585	0.000120073	0.067154312
559.319992	4040.417651	9.11866E-05	0.051002488
559.359992	2703.522062	6.10147E-05	0.034129197
559.399992	1599.559037	3.60998E-05	0.020194239
559.439992	860.257463	1.94148E-05	0.010861423
559.479992	487.695552	1.10066E-05	0.006157978
559.519992	348.782127	7.87153E-06	0.004404277
559.559992	332.767646	7.5101E-06	0.004202353
559.599992	369.145926	8.33111E-06	0.004662089
559.639992	417.884423	9.43107E-06	0.005278004
559.679992	450.034931	1.01567E-05	0.00568448
559.719992	435.374164	9.82579E-06	0.00549969
559.759992	373.89397	8.43827E-06	0.004723404
559.799992	318.978634	7.1989E-06	0.004029946
559.839992	305.335038	6.89099E-06	0.00385785
559.879992	334.584028	7.5511E-06	0.004227707
559.919992	423.158774	9.5501E-06	0.005347294
559.959992	613.780787	1.38522E-05	0.007756665
559.999992	937.344905	2.11546E-05	0.011846558
560.039992	1457.594252	3.28959E-05	0.018423004
560.079992	2097.284558	4.73328E-05	0.026510149
560.119992	2756.903091	6.22195E-05	0.034850365
560.159992	3290.450664	7.42609E-05	0.041597979
560.199992	3552.511395	8.01752E-05	0.044914165
560.239992	3476.147592	7.84518E-05	0.043951841
560.279992	2998.772211	6.76781E-05	0.037918692
560.319992	2251.035942	5.08027E-05	0.028465794

560.359992	1515.813649	3.42098E-05	0.019169805
560.399992	984.270109	2.22136E-05	0.012448504
560.439992	635.909232	1.43516E-05	0.008043203
560.479992	428.973836	9.68134E-06	0.005426198
560.519992	329.129222	7.42799E-06	0.004163536
560.559992	334.003826	7.538E-06	0.004225502
560.599992	392.124661	8.84971E-06	0.004961146
560.639992	438.704483	9.90095E-06	0.005550868
560.679992	418.951043	9.45514E-06	0.005301309
560.719992	396.857022	8.95651E-06	0.005022094
560.759992	372.051777	8.39669E-06	0.004708528
560.799992	348.983462	7.87607E-06	0.0044169
560.839992	340.644309	7.68787E-06	0.004311664
560.879992	394.604779	8.90568E-06	0.004995018
560.919992	612.42555	1.38216E-05	0.007752807
560.959992	1045.658578	2.35991E-05	0.013238127
560.999992	1807.500854	4.07928E-05	0.022884747
561.039992	2874.822384	6.48807E-05	0.036400688
561.079992	3940.812901	8.89387E-05	0.049901702
561.119992	4822.730659	0.000108842	0.061073598
561.159992	5296.775403	0.000119541	0.067081539
561.199992	5239.217268	0.000118242	0.066357317
561.239992	4713.906054	0.000106386	0.059708243
561.279992	3760.999216	8.48805E-05	0.047641734
561.319992	2685.793557	6.06146E-05	0.034024198
561.359992	1748.637267	3.94643E-05	0.022153683
561.399992	1081.636163	2.4411E-05	0.013704347
561.439992	649.15724	1.46506E-05	0.008225419
561.479992	406.571955	9.17576E-06	0.005152007
561.519992	340.975253	7.69534E-06	0.004321085
561.559992	385.204168	8.69352E-06	0.004881934
561.599992	421.968216	9.52323E-06	0.005348249
561.639992	451.498881	1.01897E-05	0.005722944
561.679992	450.120823	1.01586E-05	0.005705882
561.719992	419.850197	9.47543E-06	0.005322541
561.759992	362.031876	8.17056E-06	0.004589891
561.799992	319.942509	7.22066E-06	0.004056565
561.839992	308.248794	6.95675E-06	0.003908578
561.879992	330.606633	7.46133E-06	0.004192373
561.919992	402.013604	9.07289E-06	0.005098237
561.959992	574.757656	1.29715E-05	0.007289453
561.999992	904.570981	2.04149E-05	0.011473178
562.039992	1471.66689	3.32135E-05	0.018667299
562.079992	2244.29958	5.06507E-05	0.028469754
562.119992	2886.655463	6.51478E-05	0.036620877
562.159992	3214.27232	7.25416E-05	0.040780013

562.199992	3256.774257	7.35009E-05	0.041322182
562.239992	3011.292972	6.79607E-05	0.038210217
562.279992	2472.842275	5.58086E-05	0.031380062
562.319992	1847.903615	4.17046E-05	0.023451336
562.359992	1282.942108	2.89542E-05	0.016282693
562.399992	794.514136	1.79311E-05	0.010084438
562.439992	488.152527	1.10169E-05	0.006196358
562.479992	354.566119	8.00206E-06	0.004501001
562.519992	302.794481	6.83365E-06	0.003844065
562.559992	362.645267	8.1844E-06	0.004604215
562.599992	456.245388	1.02968E-05	0.005792992
562.639992	465.763175	1.05116E-05	0.005914261
562.679992	447.218997	1.00931E-05	0.005679191
562.719992	440.422902	9.93973E-06	0.005593286
562.759992	423.150219	9.54991E-06	0.005374308
562.799992	383.095745	8.64594E-06	0.004865933
562.839992	361.77915	8.16485E-06	0.004595505
562.879992	386.577464	8.72451E-06	0.004910855
562.919992	458.388659	1.03452E-05	0.005823516
562.959992	731.571618	1.65106E-05	0.00929478
562.999992	1288.666156	2.90834E-05	0.016373954
563.039992	2095.997045	4.73037E-05	0.026633894
563.079992	3022.582662	6.82155E-05	0.038410772
563.119992	3853.049841	8.6958E-05	0.04896777
563.159992	4436.875812	0.000100134	0.056391523
563.199992	4507.56197	0.000101729	0.057293995
563.239992	4025.781123	9.08563E-05	0.051173886
563.279992	3259.154049	7.35546E-05	0.041431816
563.319992	2365.552896	5.33872E-05	0.030074096
563.359992	1565.492984	3.5331E-05	0.01990407
563.399992	959.768935	2.16606E-05	0.012203609
563.439992	566.859607	1.27932E-05	0.007208218
563.479992	377.445077	8.51841E-06	0.004799953
563.519992	332.399817	7.5018E-06	0.004227415
563.559992	385.742752	8.70568E-06	0.004906171
563.599992	450.649402	1.01705E-05	0.00573211
563.639992	495.942984	1.11927E-05	0.006308677
563.679992	506.553808	1.14322E-05	0.00644411
563.719992	475.273779	1.07263E-05	0.006046611
563.759992	399.082521	9.00674E-06	0.005077638
563.799992	325.367231	7.34309E-06	0.004140031
563.839992	276.785949	6.24667E-06	0.003522124
563.879992	273.498007	6.17247E-06	0.003480532
563.919992	349.97417	7.89843E-06	0.004454082
563.959992	509.28564	1.14939E-05	0.006482081
563.999992	823.009467	1.85742E-05	0.010475836

564.039992	1312.850629	2.96292E-05	0.016712059
564.079992	1929.261928	4.35408E-05	0.024560468
564.119992	2523.408686	5.69498E-05	0.032126532
564.159992	2911.601298	6.57108E-05	0.037071396
564.199992	2994.517441	6.75821E-05	0.038129813
564.239992	2830.249247	6.38748E-05	0.036040707
564.279992	2416.144644	5.4529E-05	0.030769635
564.319992	1892.203888	4.27044E-05	0.02409895
564.359992	1386.503758	3.12915E-05	0.017659647
564.399992	969.177193	2.1873E-05	0.012345109
564.439992	681.169266	1.5373E-05	0.008677159
564.479992	480.22659	1.0838E-05	0.00611786
564.519992	395.289437	8.92113E-06	0.005036157
564.559992	420.232952	9.48407E-06	0.005354328
564.599992	503.33433	1.13596E-05	0.006413605
564.639992	573.064786	1.29333E-05	0.007302644
564.679992	600.528022	1.35531E-05	0.007653154
564.719992	541.262257	1.22155E-05	0.006898357
564.759992	466.655348	1.05318E-05	0.005947917
564.799992	418.986225	9.45594E-06	0.005340712
564.839992	391.167406	8.8281E-06	0.004986466
564.879992	446.411554	1.00749E-05	0.005691102
564.919992	683.080784	1.54162E-05	0.008708909
564.959992	1196.269579	2.69981E-05	0.015252868
564.999992	2210.897598	4.98969E-05	0.028191736
565.039992	3971.890806	8.964E-05	0.050650209
565.079992	6212.670944	0.000140211	0.079230617
565.119992	8264.288653	0.000186513	0.105402498
565.159992	9357.800338	0.000211193	0.119357563
565.199992	8976.43767	0.000202586	0.114501433
565.239992	7494.133231	0.000169132	0.095600254
565.279992	5511.686005	0.000124391	0.070315786
565.319992	3676.546513	8.29745E-05	0.046907163
565.359992	2236.301488	5.04702E-05	0.028533837
565.399992	1267.345271	2.86022E-05	0.016171694
565.439992	709.228737	1.60063E-05	0.009050605
565.479992	437.209865	9.86722E-06	0.005579714
565.519992	368.21696	8.31014E-06	0.004699553
565.559992	407.172492	9.18932E-06	0.005197109
565.599992	455.842814	1.02877E-05	0.005818744
565.639992	485.229644	1.0951E-05	0.006194299
565.679992	513.009248	1.15779E-05	0.006549389
565.719992	493.618301	1.11403E-05	0.006302278
565.759992	419.878419	9.47607E-06	0.005361182
565.799992	353.703805	7.9826E-06	0.004516556
565.839992	297.716552	6.71905E-06	0.003801906

565.879992	290.677037	6.56018E-06	0.003712272
565.919992	360.206064	8.12935E-06	0.004600561
565.959992	560.376743	1.26469E-05	0.007157652
565.999992	969.277822	2.18753E-05	0.012381392
566.039992	1627.113135	3.67217E-05	0.020785938
566.079992	2495.914305	5.63293E-05	0.031886895
566.119992	3409.673104	7.69516E-05	0.043563824
566.159992	4105.565963	9.26569E-05	0.052458633
566.199992	4262.115251	9.619E-05	0.05446278
566.239992	3836.103269	8.65755E-05	0.049022515
566.279992	3115.996799	7.03237E-05	0.039822909
566.319992	2303.682913	5.19909E-05	0.029443494
566.359992	1528.408875	3.44941E-05	0.019536055
566.399992	940.590676	2.12278E-05	0.012023438
566.439992	582.363168	1.31431E-05	0.007444792
566.479992	423.217681	9.55143E-06	0.005410696
566.519992	405.837419	9.15919E-06	0.005188862
566.559992	450.162472	1.01595E-05	0.005755989
566.599992	529.477697	1.19496E-05	0.006770629
566.639992	602.89968	1.36066E-05	0.007710047
566.679992	601.200652	1.35683E-05	0.007688862
566.719992	566.053041	1.2775E-05	0.007239864
566.759992	525.65095	1.18632E-05	0.006723593
566.799992	515.084828	1.16247E-05	0.006588907
566.839992	515.450505	1.1633E-05	0.00659405
566.879992	544.735912	1.22939E-05	0.006969183
566.919992	651.297354	1.46989E-05	0.008333086
566.959992	943.686368	2.12977E-05	0.012074936
566.999992	1380.329807	3.11521E-05	0.017663252
567.039992	2016.01211	4.54986E-05	0.025799517
567.079992	2872.413818	6.48264E-05	0.036761742
567.119992	3729.102878	8.41607E-05	0.047729192
567.159992	4403.591888	9.93829E-05	0.056366025
567.199992	4702.963621	0.000106139	0.060202232
567.239992	4516.258852	0.000101926	0.057816317
567.279992	3876.452505	8.74861E-05	0.049629133
567.319992	2903.117172	6.55193E-05	0.037170415
567.359992	1974.399824	4.45595E-05	0.025281251
567.399992	1254.08391	2.83029E-05	0.016059081
567.439992	762.278509	1.72036E-05	0.00976199
567.479992	495.447952	1.11816E-05	0.006345317
567.519992	388.274756	8.76282E-06	0.004973076
567.559992	358.325944	8.08692E-06	0.004589811
567.599992	411.69917	9.29148E-06	0.005273842
567.639992	472.594181	1.06658E-05	0.00605433
567.679992	511.776429	1.15501E-05	0.00655675

567.719992	474.26749	1.07036E-05	0.006076623
567.759992	401.179592	9.05406E-06	0.005140536
567.799992	352.59949	7.95768E-06	0.00451837
567.839992	308.455717	6.96142E-06	0.00395297
567.879992	280.835259	6.33806E-06	0.003599258
567.919992	308.640664	6.96559E-06	0.003955898
567.959992	442.46337	9.98578E-06	0.005671525
567.999992	710.338574	1.60314E-05	0.009105808
568.039992	1105.125836	2.49412E-05	0.014167571
568.079992	1646.519795	3.71597E-05	0.021109658
568.119992	2227.538595	5.02724E-05	0.02856078
568.159992	2682.584969	6.05422E-05	0.034397659
568.199992	2933.093257	6.61958E-05	0.03761247
568.239992	2832.207458	6.3919E-05	0.036321319
568.279992	2420.983118	5.46382E-05	0.031049805
568.319992	1887.733991	4.26035E-05	0.024212436
568.359992	1338.500606	3.02081E-05	0.017169072
568.399992	864.034486	1.95001E-05	0.011083831
568.439992	552.194994	1.24623E-05	0.007084054
568.479992	385.727818	8.70534E-06	0.004948811
568.519992	316.962307	7.1534E-06	0.00406685
568.559992	353.448944	7.97685E-06	0.004535318
568.599992	438.899083	9.90534E-06	0.005632177
568.639992	480.010663	1.08332E-05	0.006160175
568.679992	451.327906	1.01858E-05	0.005792485
568.719992	407.084647	9.18733E-06	0.00522502
568.759992	353.836546	7.9856E-06	0.004541889
568.799992	312.04503	7.04242E-06	0.004005729
568.839992	309.069041	6.97526E-06	0.003967806
568.879992	358.979864	8.10168E-06	0.004608881
568.919992	478.061038	1.07892E-05	0.006138176
568.959992	700.680352	1.58134E-05	0.00899718
568.999992	1048.098639	2.36541E-05	0.013459198
569.039992	1602.020014	3.61554E-05	0.020573846
569.079992	2300.154875	5.19113E-05	0.029541677
569.119992	2936.450145	6.62716E-05	0.037716487
569.159992	3294.0581	7.43423E-05	0.042312665
569.199992	3345.046133	7.5493E-05	0.042970633
569.239992	3175.658905	7.16702E-05	0.040797543
569.279992	2733.574764	6.1693E-05	0.035120576
569.319992	2121.553931	4.78805E-05	0.027259336
569.359992	1507.043971	3.40119E-05	0.019365006
569.399992	977.873217	2.20692E-05	0.012566223
569.439992	613.060492	1.38359E-05	0.007878727
569.479992	419.762097	9.47345E-06	0.005394938
569.519992	333.377507	7.52387E-06	0.004284992

569.559992	346.270664	7.81485E-06	0.004451024
569.599992	416.121562	9.39128E-06	0.005349275
569.639992	452.786805	1.02188E-05	0.005821019
569.679992	427.118921	9.63948E-06	0.005491418
569.719992	377.465559	8.51887E-06	0.004853372
569.759992	341.890502	7.71599E-06	0.004396264
569.799992	289.036793	6.52316E-06	0.003716895
569.839992	255.601937	5.76858E-06	0.003287167
569.879992	270.621835	6.10756E-06	0.003480575
569.919992	335.703077	7.57635E-06	0.004317914
569.959992	459.293589	1.03656E-05	0.005907987
569.999992	670.028889	1.51216E-05	0.008619323
570.039992	1027.327944	2.31854E-05	0.013216583
570.079992	1585.026415	3.57718E-05	0.020392809
570.119992	2261.980408	5.10497E-05	0.029104481
570.159992	2804.411638	6.32917E-05	0.036086375
570.199992	3128.35268	7.06026E-05	0.040257581
570.239992	3195.611099	7.21205E-05	0.041125988
570.279992	2905.329511	6.55692E-05	0.037392826
570.319992	2272.498563	5.12871E-05	0.029250074
570.359992	1595.40592	3.60061E-05	0.020536433
570.399992	1024.126539	2.31131E-05	0.013183717
570.439992	624.807458	1.4101E-05	0.008043794
570.479992	427.964784	9.65857E-06	0.00551002
570.519992	384.213923	8.67117E-06	0.004947078
570.559992	412.022783	9.29878E-06	0.005305512
570.599992	459.204613	1.03636E-05	0.005913475
570.639992	475.785297	1.07378E-05	0.006127425
570.679992	469.742428	1.06014E-05	0.006050025
570.719992	432.353606	9.75762E-06	0.005568868
570.759992	372.819826	8.41402E-06	0.004802388
570.799992	320.239276	7.22735E-06	0.004125374
570.839992	296.564117	6.69304E-06	0.003820654
570.879992	336.098672	7.58528E-06	0.004330284
570.919992	448.983215	1.01329E-05	0.00578509
570.959992	689.140775	1.55529E-05	0.008880111
570.999992	1094.575656	2.4703E-05	0.01410544
571.039992	1723.160679	3.88893E-05	0.022207366
571.079992	2449.655085	5.52853E-05	0.03157233
571.119992	3143.048319	7.09342E-05	0.040511953
571.159992	3619.643739	8.16903E-05	0.046658241
571.199992	3712.235371	8.378E-05	0.047855125
571.239992	3422.881705	7.72497E-05	0.044128102
571.279992	2778.420557	6.27051E-05	0.035822159
571.319992	2025.562406	4.57141E-05	0.026117392
571.359992	1385.094069	3.12596E-05	0.01786051

571.399992	877.826107	1.98113E-05	0.011320184
571.439992	552.410937	1.24671E-05	0.007124225
571.479992	389.934278	8.80027E-06	0.00502918
571.519992	337.309747	7.61261E-06	0.004350759
571.559992	368.676105	8.32051E-06	0.004755668
571.599992	420.566994	9.49161E-06	0.005425405
571.639992	459.086679	1.03609E-05	0.005922732
571.679992	473.480507	1.06858E-05	0.006108856
571.719992	431.765277	9.74434E-06	0.005571035
571.759992	356.468198	8.04499E-06	0.004599804
571.799992	278.318649	6.28126E-06	0.003591627
571.839992	237.622336	5.36281E-06	0.003066666
571.879992	256.710589	5.7936E-06	0.003313244
571.919992	322.44123	7.27705E-06	0.00416189
571.959992	467.701318	1.05554E-05	0.006037248
571.999992	779.441226	1.75909E-05	0.010061996
572.039992	1248.897868	2.81859E-05	0.016123454
572.079992	1891.927109	4.26982E-05	0.024426763
572.119992	2641.037888	5.96045E-05	0.034100953
572.159992	3393.393635	7.65842E-05	0.043818397
572.199992	3980.985607	8.98453E-05	0.05140948
572.239992	4164.063103	9.39771E-05	0.053777457
572.279992	3800.260453	8.57666E-05	0.049082501
572.319992	2912.949782	6.57412E-05	0.037625014
572.359992	1902.042621	4.29265E-05	0.024569384
572.399992	1128.576643	2.54704E-05	0.014579258
572.439992	656.124137	1.48078E-05	0.008476581
572.479992	411.299867	9.28247E-06	0.005314026
572.519992	336.387604	7.5918E-06	0.004346457
572.559992	369.182405	8.33193E-06	0.004770531
572.599992	438.367168	9.89334E-06	0.005664924
572.639992	446.354073	1.00736E-05	0.00576854
572.679992	433.531714	9.78421E-06	0.00560322
572.719992	444.062635	1.00219E-05	0.005739728
572.759992	419.690194	9.47182E-06	0.005425081
572.799992	368.533068	8.31728E-06	0.004764137
572.839992	317.839725	7.1732E-06	0.004109096
572.879992	318.160213	7.18043E-06	0.004113526
572.919992	435.200078	9.82186E-06	0.00562714
572.959992	734.308787	1.65723E-05	0.00949528
572.999992	1203.605454	2.71637E-05	0.015564799
573.039992	1892.537919	4.27119E-05	0.024475652
573.079992	2722.106392	6.14341E-05	0.035206682
573.119992	3507.884181	7.91681E-05	0.045372798
573.159992	4069.244165	9.18372E-05	0.052637393
573.199992	4294.631921	9.69239E-05	0.055556756

573.239992	4084.742373	9.21869E-05	0.052845244
573.279992	3499.59306	7.89809E-05	0.045278193
573.319992	2616.918572	5.90602E-05	0.033860397
573.359992	1724.132807	3.89113E-05	0.022310169
573.399992	1044.110974	2.35641E-05	0.013511672
573.439992	613.566889	1.38474E-05	0.007940624
573.479992	407.691008	9.20102E-06	0.0052766
573.519992	327.656059	7.39474E-06	0.004241032
573.559992	341.401006	7.70495E-06	0.004419248
573.599992	399.714921	9.02101E-06	0.005174451
573.639992	440.262902	9.93612E-06	0.005699756
573.679992	447.184501	1.00923E-05	0.005789769
573.719992	409.817455	9.24901E-06	0.005306341
573.759992	357.611457	8.07079E-06	0.004630698
573.799992	304.536855	6.87297E-06	0.003943712
573.839992	299.864287	6.76752E-06	0.003883473
573.879992	324.396664	7.32118E-06	0.004201479
573.919992	408.970923	9.2299E-06	0.005297226
573.959992	583.481421	1.31684E-05	0.007558113
573.999992	911.221037	2.0565E-05	0.011804304
574.039992	1540.949459	3.47771E-05	0.019963436
574.079992	2607.081516	5.88382E-05	0.033777832
574.119992	3854.789867	8.69972E-05	0.049946854
574.159992	5196.656759	0.000117281	0.067338233
574.199992	6420.701074	0.000144906	0.083205185
574.239992	6988.213028	0.000157714	0.090565821
574.279992	6609.953294	0.000149177	0.085669618
574.319992	5390.244964	0.00012165	0.069866211
574.359992	3749.798799	8.46277E-05	0.048606786
574.399992	2259.974768	5.10045E-05	0.029296974
574.439992	1228.650105	2.77289E-05	0.015928601
574.479992	669.529446	1.51103E-05	0.008680592
574.519992	476.765748	1.07599E-05	0.0061818
574.559992	445.014766	1.00434E-05	0.005770515
574.599992	463.405908	1.04584E-05	0.006009411
574.639992	487.917427	1.10116E-05	0.006327715
574.679992	515.579812	1.16359E-05	0.006686929
574.719992	525.035297	1.18493E-05	0.006810038
574.759992	470.060999	1.06086E-05	0.006097411
574.799992	407.749326	9.20233E-06	0.005289502
574.839992	414.54273	9.35565E-06	0.005378003
574.879992	491.75327	1.10982E-05	0.006380125
574.919992	626.248837	1.41336E-05	0.008125668
574.959992	896.373183	2.02299E-05	0.01163138
574.999992	1486.426445	3.35466E-05	0.01928928
575.039992	2482.820683	5.60338E-05	0.032221679

575.079992	3662.031423	8.26469E-05	0.047528606
575.119992	4742.992036	0.000107043	0.06156241
575.159992	5659.721049	0.000127732	0.073466348
575.199992	6200.352103	0.000139933	0.080489639
575.239992	6192.332	0.000139752	0.080391117
575.279992	5568.258809	0.000125668	0.072294197
575.319992	4434.496339	0.00010008	0.057578258
575.359992	3042.038005	6.86546E-05	0.039501087
575.399992	1832.314883	4.13528E-05	0.023794398
575.439992	1022.413138	2.30744E-05	0.013277956
575.479992	594.888619	1.34258E-05	0.007726284
575.519992	430.326981	9.71188E-06	0.005589381
575.559992	395.762449	8.93181E-06	0.005140791
575.599992	432.289779	9.75618E-06	0.005615656
575.639992	460.224349	1.03866E-05	0.005978955
575.679992	426.833519	9.63304E-06	0.005545547
575.719992	410.482541	9.26402E-06	0.005333481
575.759992	418.385591	9.44238E-06	0.005436545
575.799992	430.470197	9.71511E-06	0.005593962
575.839992	429.589604	9.69524E-06	0.005582906
575.879992	471.289543	1.06363E-05	0.00612526
575.919992	568.817986	1.28374E-05	0.007393332
575.959992	724.718492	1.63559E-05	0.009420336
575.999992	1018.680068	2.29902E-05	0.013242349
576.039992	1520.125918	3.43071E-05	0.019762276
576.079992	2161.31385	4.87778E-05	0.028099939
576.119992	2760.320078	6.22966E-05	0.035890305
576.159992	3206.707698	7.23709E-05	0.041697232
576.199992	3452.954108	7.79284E-05	0.044902323
576.239992	3379.243782	7.62648E-05	0.043946842
576.279992	2969.766976	6.70235E-05	0.038624304
576.319992	2360.66104	5.32768E-05	0.030704503
576.359992	1708.263986	3.85531E-05	0.022220487
576.399992	1107.36232	2.49916E-05	0.014405173
576.439992	690.995494	1.55948E-05	0.00898947
576.479992	466.080085	1.05188E-05	0.006063865
576.519992	368.687444	8.32076E-06	0.004797086
576.559992	357.493674	8.06813E-06	0.004651763
576.599992	410.176866	9.25712E-06	0.005337656
576.639992	454.127846	1.0249E-05	0.005910002
576.679992	451.625212	1.01926E-05	0.005877841
576.719992	456.90023	1.03116E-05	0.005946907
576.759992	450.963324	1.01776E-05	0.005870041
576.799992	434.128828	9.79768E-06	0.005651303
576.839992	434.10616	9.79717E-06	0.0056514
576.879992	466.598413	1.05305E-05	0.006074821

576.919992	614.581393	1.38702E-05	0.008002022
576.959992	907.591991	2.04831E-05	0.011817922
576.999992	1418.422875	3.20118E-05	0.018470825
577.039992	2203.957587	4.97403E-05	0.028702115
577.079992	3180.866262	7.17877E-05	0.041427257
577.119992	4102.893453	9.25966E-05	0.053439343
577.159992	4809.482413	0.000108543	0.062646861
577.199992	5177.748551	0.000116855	0.067448458
577.239992	5002.100059	0.00011289	0.065164871
577.279992	4372.505204	9.86814E-05	0.05696677
577.319992	3361.725645	7.58695E-05	0.043800959
577.359992	2286.259915	5.15977E-05	0.029790449
577.399992	1390.437945	3.13802E-05	0.018118955
577.439992	782.310969	1.76557E-05	0.010195089
577.479992	455.453756	1.0279E-05	0.005935892
577.519992	360.483727	8.13562E-06	0.004698481
577.559992	383.3667	8.65205E-06	0.004997079
577.599992	444.288544	1.0027E-05	0.00579158
577.639992	528.612999	1.19301E-05	0.006891279
577.679992	517.848356	1.16871E-05	0.006751413
577.719992	440.389902	9.93899E-06	0.005741951
577.759992	394.802929	8.91015E-06	0.005147929
577.799992	357.861347	8.07643E-06	0.004666562
577.839992	319.841212	7.21837E-06	0.004171063
577.879992	309.020495	6.97416E-06	0.004030229
577.919992	344.497214	7.77482E-06	0.004493225
577.959992	473.184036	1.06791E-05	0.006172095
577.999992	736.428454	1.66202E-05	0.009606455
578.039992	1188.433229	2.68213E-05	0.015503774
578.079992	1843.250748	4.15996E-05	0.024047898
578.119992	2504.60315	5.65254E-05	0.032678466
578.159992	2937.749246	6.63009E-05	0.038332533
578.199992	3112.951872	7.0255E-05	0.040621433
578.239992	3024.954119	6.8269E-05	0.039475867
578.279992	2630.892734	5.93756E-05	0.034335713
578.319992	2034.038938	4.59054E-05	0.026548025
578.359992	1449.796952	3.27199E-05	0.01892388
578.399992	979.108721	2.20971E-05	0.012780974
578.439992	644.615424	1.45481E-05	0.008415187
578.479992	432.872667	9.76933E-06	0.005651364
578.519992	343.388917	7.74981E-06	0.00448342
578.559992	364.007769	8.21515E-06	0.004752956
578.599992	408.840728	9.22697E-06	0.005338722
578.639992	466.774018	1.05344E-05	0.006095648
578.679992	499.615892	1.12756E-05	0.006524984
578.719992	500.577668	1.12973E-05	0.006537997

578.759992	463.843228	1.04683E-05	0.006058631
578.799992	393.335142	8.87703E-06	0.005138023
578.839992	363.25944	8.19826E-06	0.004745481
578.879992	417.935988	9.43223E-06	0.005460131
578.919992	571.355477	1.28947E-05	0.007464998
578.959992	875.892257	1.97677E-05	0.011444689
578.999992	1460.93816	3.29713E-05	0.019090405
579.039992	2406.593669	5.43135E-05	0.031449669
579.079992	3544.587922	7.99964E-05	0.046324322
579.119992	4646.691787	0.000104869	0.060731944
579.159992	5499.535575	0.000124117	0.071883518
579.199992	5728.95393	0.000129295	0.074887379
579.239992	5328.771847	0.000120263	0.069661115
579.279992	4463.674867	0.000100739	0.058356043
579.319992	3314.046346	7.47934E-05	0.043329318
579.359992	2196.732124	4.95772E-05	0.028723037
579.399992	1309.191796	2.95466E-05	0.01711932
579.439992	760.658245	1.7167E-05	0.009947245
579.479992	489.890887	1.10562E-05	0.006406821
579.519992	403.494012	9.1063E-06	0.005277282
579.559992	438.25847	9.89088E-06	0.00573236
579.599992	507.864699	1.14618E-05	0.006643259
579.639992	576.888002	1.30196E-05	0.007546657
579.679992	575.341927	1.29847E-05	0.007526951
579.719992	501.850521	1.13261E-05	0.006565948
579.759992	419.831942	9.47502E-06	0.005493239
579.799992	351.182782	7.92571E-06	0.004595324
579.839992	284.211601	6.41426E-06	0.003719244
579.879992	260.52916	5.87978E-06	0.003409567
579.919992	331.262962	7.47614E-06	0.004335565
579.959992	481.165149	1.08592E-05	0.006297917
579.999992	774.864476	1.74876E-05	0.010142815
580.039992	1251.042771	2.82343E-05	0.016377019
580.079992	1846.856033	4.1681E-05	0.024178296
580.119992	2475.539592	5.58695E-05	0.032411003
580.159992	2937.430292	6.62937E-05	0.038460958
580.199992	3051.161575	6.88605E-05	0.039952842
580.239992	2919.46965	6.58884E-05	0.038231064
580.279992	2576.516799	5.81484E-05	0.03374235
580.319991	1982.783145	4.47487E-05	0.025968538
580.359991	1338.764287	3.0214E-05	0.017535023
580.399991	871.634832	1.96716E-05	0.011417387
580.439991	581.945151	1.31337E-05	0.00762332
580.479991	414.948866	9.36482E-06	0.005436089
580.519991	361.143286	8.1505E-06	0.004731529
580.559991	414.443935	9.35342E-06	0.005430223

580.599991	489.609956	1.10498E-05	0.006415522
580.639991	537.182293	1.21235E-05	0.007039363
580.679991	550.464582	1.24232E-05	0.007213915
580.719991	493.969283	1.11482E-05	0.006473982
580.759991	422.641497	9.53843E-06	0.005539538
580.799991	365.424456	8.24712E-06	0.004789928
580.839991	344.277234	7.76986E-06	0.004513044
580.879991	394.592111	8.90539E-06	0.005172965
580.919991	573.129815	1.29347E-05	0.00751405
580.959991	886.084384	1.99977E-05	0.011617858
580.999991	1395.373673	3.14916E-05	0.018296643
581.039991	2176.706404	4.91252E-05	0.028543724
581.079991	3016.121891	6.80697E-05	0.039553923
581.119991	3758.55551	8.48254E-05	0.049293714
581.159991	4142.898734	9.34995E-05	0.054338142
581.199991	4170.62084	9.41251E-05	0.054705509
581.239991	3847.382285	8.68301E-05	0.050469103
581.279991	3083.219907	6.9584E-05	0.040447775
581.319991	2232.09186	5.03752E-05	0.029284113
581.359991	1489.862098	3.36241E-05	0.019547714
581.399991	933.564038	2.10692E-05	0.012249656
581.439991	576.651949	1.30142E-05	0.007566995
581.479991	402.601423	9.08615E-06	0.005283416
581.519991	357.049208	8.0581E-06	0.004685948
581.559991	393.918144	8.89018E-06	0.005170175
581.599991	443.525751	1.00098E-05	0.005821675
581.639991	469.0287	1.05853E-05	0.006156848
581.679991	483.422507	1.09102E-05	0.006346229
581.719991	472.623161	1.06664E-05	0.006204885
581.759991	438.914431	9.90569E-06	0.005762733
581.799991	380.381242	8.58467E-06	0.004994564
581.839991	331.281986	7.47657E-06	0.004350169
581.879991	340.755983	7.69039E-06	0.004474883
581.919991	399.61636	9.01878E-06	0.005248211
581.959991	502.92123	1.13502E-05	0.006605381
581.999991	709.731109	1.60176E-05	0.009322268
582.039991	1088.708547	2.45706E-05	0.014301092
582.079991	1614.478096	3.64365E-05	0.02120897
582.119991	2061.337903	4.65215E-05	0.02708111
582.159991	2448.001362	5.5248E-05	0.032163164
582.199991	2613.07657	5.89735E-05	0.03433437
582.239991	2449.916392	5.52912E-05	0.032192748
582.279991	2065.767841	4.66215E-05	0.027146768
582.319991	1603.163543	3.61812E-05	0.021069018
582.359991	1152.01922	2.59995E-05	0.015141051
582.399991	780.26639	1.76095E-05	0.010255788

582.439991	513.788973	1.15955E-05	0.006753684
582.479991	364.880236	8.23484E-06	0.004796629
582.519991	324.619422	7.32621E-06	0.004267663
582.559991	355.545912	8.02418E-06	0.004674564
582.599991	415.537043	9.37809E-06	0.005463676
582.639991	473.786241	1.06927E-05	0.006229992
582.679991	475.24295	1.07256E-05	0.006249576
582.719991	429.514079	9.69353E-06	0.005648616
582.759991	406.914785	9.1835E-06	0.005351776
582.799991	396.530733	8.94915E-06	0.005215562
582.839991	399.82887	9.02358E-06	0.005259304
582.879991	435.076835	9.81908E-06	0.005723344
582.919991	537.870405	1.2139E-05	0.007076058
582.959991	798.97339	1.80317E-05	0.010511769
582.999991	1282.555105	2.89455E-05	0.016875216
583.039991	1996.809828	4.50652E-05	0.026274823
583.079991	2855.211804	6.44382E-05	0.037572598
583.119991	3584.91225	8.09065E-05	0.047178184
583.159991	3979.977167	8.98225E-05	0.052380912
583.199991	3908.40972	8.82074E-05	0.051442534
583.239991	3483.597415	7.86199E-05	0.045854294
583.279991	2810.719111	6.3434E-05	0.036999792
583.319991	2043.679072	4.6123E-05	0.026904462
583.359991	1355.442514	3.05904E-05	0.017845244
583.399991	826.393993	1.86506E-05	0.010880737
583.439991	516.274214	1.16516E-05	0.006798004
583.479991	378.221415	8.53593E-06	0.004980545
583.519991	332.816589	7.51121E-06	0.004382939
583.559991	355.029569	8.01252E-06	0.004675788
583.599991	425.959936	9.61332E-06	0.005610335
583.639991	486.378123	1.09769E-05	0.006406544
583.679991	498.988123	1.12615E-05	0.006573093
583.719991	454.770842	1.02635E-05	0.005991036
583.759991	378.169527	8.53476E-06	0.004982251
583.799991	308.466795	6.96167E-06	0.00406422
583.839991	274.332494	6.1913E-06	0.00361473
583.879991	296.137312	6.68341E-06	0.003902307
583.919991	361.15458	8.15076E-06	0.004759389
583.959991	491.333927	1.10887E-05	0.00647537
583.999991	721.497673	1.62832E-05	0.009509387
584.039991	1089.974145	2.45992E-05	0.014366916
584.079991	1643.840809	3.70992E-05	0.021668899
584.119991	2248.321899	5.07415E-05	0.029639121
584.159991	2667.47501	6.02012E-05	0.03516713
584.199991	2781.713071	6.27794E-05	0.036675719
584.239991	2630.469913	5.9366E-05	0.034684016

584.279991	2270.127026	5.12336E-05	0.02993477
584.319991	1728.304492	3.90054E-05	0.02279165
584.359991	1175.00324	2.65182E-05	0.015496167
584.399991	742.654706	1.67607E-05	0.009794943
584.439991	471.771291	1.06472E-05	0.006222662
584.479991	375.188477	8.46748E-06	0.004949073
584.519991	369.157363	8.33137E-06	0.004869851
584.559991	389.600038	8.79273E-06	0.005139878
584.599991	448.39169	1.01196E-05	0.005915904
584.639991	508.69702	1.14806E-05	0.006712008
584.679991	520.564565	1.17484E-05	0.006869064
584.719991	497.160901	1.12202E-05	0.006560692
584.759991	451.298648	1.01852E-05	0.005955887
584.799991	409.694151	9.24623E-06	0.005407193
584.839991	374.832369	8.45944E-06	0.004947421
584.879991	392.136687	8.84998E-06	0.005176176
584.919991	509.033188	1.14882E-05	0.00671966
584.959991	762.88615	1.72173E-05	0.010071419
584.999991	1189.533217	2.68461E-05	0.015704973
585.039991	1754.489305	3.95964E-05	0.023165466
585.079991	2473.875236	5.58319E-05	0.032666138
585.119991	3138.281303	7.08266E-05	0.041442082
585.159991	3540.364285	7.99011E-05	0.046754921
585.199991	3719.881562	8.39525E-05	0.049129029
585.239991	3686.610662	8.32017E-05	0.048692943
585.279991	3317.356406	7.48681E-05	0.043818809
585.319991	2676.453713	6.04038E-05	0.035355571
585.359991	1935.813941	4.36886E-05	0.025573573
585.399991	1264.109573	2.85292E-05	0.016700989
585.439991	768.327005	1.73401E-05	0.010151571
585.479991	450.358178	1.0164E-05	0.005950793
585.519991	348.880137	7.87374E-06	0.004610231
585.559991	370.899895	8.37069E-06	0.004901544
585.599991	451.351803	1.01864E-05	0.005965145
585.639991	484.391593	1.0932E-05	0.006402242
585.679991	482.885219	1.0898E-05	0.006382768
585.719991	463.793321	1.04672E-05	0.00613083
585.759991	398.798829	9.00033E-06	0.005272036
585.799991	320.043197	7.22293E-06	0.004231192
585.839991	266.102339	6.00556E-06	0.003518297
585.879991	268.484883	6.05933E-06	0.00355004
585.919991	341.563914	7.70862E-06	0.004516636
585.959991	465.146096	1.04977E-05	0.006151232
585.999991	696.519975	1.57195E-05	0.009211618
586.039991	1083.756995	2.44589E-05	0.014333885
586.079991	1639.141418	3.69931E-05	0.021680938

586.119991	2184.615837	4.93037E-05	0.028897906
586.159991	2570.143913	5.80046E-05	0.033999957
586.199991	2743.650571	6.19204E-05	0.036297721
586.239991	2600.176274	5.86824E-05	0.034401944
586.279991	2243.590355	5.06347E-05	0.029686117
586.319991	1778.021879	4.01275E-05	0.023527542
586.359991	1275.722978	2.87913E-05	0.016882061
586.399991	866.928533	1.95654E-05	0.011473132
586.439991	572.98238	1.29314E-05	0.007583498
586.479991	382.147123	8.62453E-06	0.005058113
586.519991	308.491459	6.96222E-06	0.004083483
586.559991	323.080999	7.29149E-06	0.004276895
586.599991	391.888273	8.84437E-06	0.005188109
586.639991	416.854486	9.40783E-06	0.005519006
586.679991	408.063225	9.20942E-06	0.005402982
586.719991	404.431014	9.12744E-06	0.005355254
586.759991	394.576438	8.90504E-06	0.005225122
586.799991	382.438162	8.6311E-06	0.005064727
586.839991	364.581896	8.22811E-06	0.004828581
586.879991	383.61512	8.65766E-06	0.005081007
586.919991	478.302551	1.07946E-05	0.00633558
586.959991	670.414903	1.51303E-05	0.008880899
586.999991	1053.363341	2.37729E-05	0.013954718
587.039991	1665.158776	3.75803E-05	0.022061147
587.079991	2407.231194	5.43279E-05	0.031894797
587.119991	3115.180157	7.03053E-05	0.041277634
587.159991	3559.880399	8.03415E-05	0.047173339
587.199991	3629.488609	8.19125E-05	0.048099021
587.239991	3274.594966	7.3903E-05	0.043398825
587.279991	2704.441039	6.10355E-05	0.035844909
587.319991	2080.647854	4.69573E-05	0.027578975
587.359991	1437.310693	3.24381E-05	0.019052842
587.399991	909.036432	2.05157E-05	0.012050914
587.439991	550.307584	1.24197E-05	0.007295814
587.479991	357.783358	8.07467E-06	0.004743708
587.519991	310.473172	7.00695E-06	0.004116721
587.559991	319.941662	7.22064E-06	0.004242558
587.599991	377.720015	8.52461E-06	0.005009063
587.639991	427.639104	9.65122E-06	0.005671442
587.679991	435.87073	9.837E-06	0.005781005
587.719991	436.37169	9.8483E-06	0.005788044
587.759991	389.731716	8.7957E-06	0.005169762
587.799991	304.903145	6.88124E-06	0.004044792
587.839991	260.579933	5.88093E-06	0.003457044
587.879991	283.309072	6.39389E-06	0.003758841
587.919991	372.27085	8.40163E-06	0.004939489

587.959991	497.633124	1.12309E-05	0.006603312
587.999991	722.349863	1.63024E-05	0.009585829
588.039991	1169.903494	2.64031E-05	0.015526074
588.079991	1847.139498	4.16874E-05	0.024515506
588.119991	2506.590553	5.65703E-05	0.033270099
588.159991	2936.237122	6.62668E-05	0.03897547
588.199991	3063.397887	6.91366E-05	0.040666161
588.239991	2795.441764	6.30892E-05	0.037111606
588.279991	2318.04892	5.23151E-05	0.030775947
588.319991	1791.413887	4.04297E-05	0.02378561
588.359991	1275.236131	2.87803E-05	0.016933179
588.399991	846.93172	1.91141E-05	0.011246718
588.439991	595.652439	1.3443E-05	0.007910426
588.479991	454.981307	1.02683E-05	0.006042686
588.519991	410.268795	9.2592E-06	0.005449222
588.559991	442.963182	9.99706E-06	0.005883871
588.599991	563.126555	1.2709E-05	0.007480507
588.639991	672.167143	1.51699E-05	0.008929596
588.679991	689.449426	1.55599E-05	0.00915981
588.719991	621.532742	1.40271E-05	0.008258051
588.759991	501.888098	1.13269E-05	0.006668835
588.799991	404.592036	9.13108E-06	0.005376379
588.839991	345.836844	7.80506E-06	0.004595929
588.879991	381.776626	8.61617E-06	0.005073888
588.919991	493.239417	1.11317E-05	0.006555696
588.959991	718.552408	1.62167E-05	0.009551003
588.999991	1161.78291	2.62198E-05	0.015443475
589.039991	1898.149644	4.28386E-05	0.025233645
589.079991	2820.675148	6.36587E-05	0.037500072
589.119991	3705.297316	8.36234E-05	0.049264216
589.159991	4324.082419	9.75885E-05	0.05749525
589.199991	4508.090166	0.000101741	0.059945982
589.239991	4209.447315	9.50014E-05	0.055978602
589.279991	3535.331768	7.97875E-05	0.047017185
589.319991	2667.138324	6.01936E-05	0.03547329
589.359991	1771.177743	3.9973E-05	0.023558496
589.399991	1062.921837	2.39887E-05	0.014138919
589.439991	638.677686	1.44141E-05	0.008496227
589.479991	434.389245	9.80356E-06	0.005779003
589.519991	373.514058	8.42969E-06	0.004969472
589.559991	400.13145	9.03041E-06	0.005323968
589.599991	452.124168	1.02038E-05	0.006016168
589.639991	476.960846	1.07643E-05	0.006347086
589.679991	474.693982	1.07132E-05	0.006317349
589.719991	465.292971	1.0501E-05	0.006192658
589.759991	430.710881	9.72054E-06	0.005732788

589.799991	357.08012	8.0588E-06	0.004753081
589.839991	289.037532	6.52317E-06	0.003847629
589.879991	273.783791	6.17892E-06	0.003644821
589.919991	326.090725	7.35941E-06	0.004341465
589.959991	471.128342	1.06327E-05	0.006272874
589.999991	747.577461	1.68718E-05	0.009954351
590.039991	1155.330871	2.60742E-05	0.015384826
590.079991	1772.758577	4.00087E-05	0.023608329
590.119991	2413.070986	5.44597E-05	0.032137729
590.159991	2919.92027	6.58985E-05	0.038890678
590.199991	3163.496764	7.13957E-05	0.042137751
590.239991	2984.077692	6.73465E-05	0.039750584
590.279991	2455.523847	5.54178E-05	0.032711991
590.319991	1776.413658	4.00912E-05	0.023666626
590.359991	1207.538158	2.72525E-05	0.016088759
590.399991	806.802717	1.82084E-05	0.010750247
590.439991	518.832022	1.17093E-05	0.006913648
590.479991	352.785743	7.96188E-06	0.004701333
590.519991	296.072896	6.68195E-06	0.003945827
590.559991	344.141226	7.76679E-06	0.004586754
590.599991	436.714518	9.85604E-06	0.005820976
590.639991	492.342599	1.11115E-05	0.006562888
590.679991	486.713742	1.09845E-05	0.006488296
590.719991	480.331351	1.08404E-05	0.006403647
590.759991	453.526382	1.02355E-05	0.0060467
590.799991	393.191722	8.87379E-06	0.005242635
590.839991	346.994828	7.83119E-06	0.00462698
590.879991	368.316333	8.31239E-06	0.004911623
590.919991	474.531922	1.07095E-05	0.006328472
590.959991	701.963152	1.58423E-05	0.009362184
590.999991	1114.524105	2.51533E-05	0.014865574
591.039991	1819.670159	4.10674E-05	0.024272488
591.079991	2672.405657	6.03125E-05	0.035649496
591.119991	3509.361356	7.92014E-05	0.046817529
591.159991	4161.399066	9.3917E-05	0.055519961
591.199991	4399.397188	9.92883E-05	0.058699222
591.239991	4163.963201	9.39748E-05	0.055561689
591.279991	3580.744467	8.08124E-05	0.047782764
591.319991	2748.955637	6.20401E-05	0.036685551
591.359991	1858.41602	4.19419E-05	0.024802739
591.399991	1124.629733	2.53813E-05	0.015010516
591.439991	648.079364	1.46262E-05	0.008650548
591.479991	404.383439	9.12637E-06	0.005398066
591.519991	331.43764	7.48009E-06	0.00442462
591.559991	376.71934	8.50203E-06	0.005029461
591.599991	457.099652	1.03161E-05	0.006103006

591.639991	491.245044	1.10867E-05	0.006559345
591.679991	493.731531	1.11428E-05	0.006592991
591.719991	466.131665	1.05199E-05	0.00622486
591.759991	379.664367	8.5685E-06	0.005070493
591.799991	314.418719	7.09599E-06	0.004199408
591.839991	311.207083	7.02351E-06	0.004156794
591.879991	335.065425	7.56196E-06	0.004475773
591.919991	384.043584	8.66733E-06	0.005130365
591.959991	522.527835	1.17927E-05	0.006980822
591.999991	827.311387	1.86713E-05	0.011053389
592.039991	1260.138459	2.84396E-05	0.016837363
592.079991	1762.211256	3.97707E-05	0.023547408
592.119991	2288.206939	5.16416E-05	0.030578049
592.159991	2753.387556	6.21401E-05	0.036796894
592.199991	2901.99698	6.5494E-05	0.038785564
592.239991	2753.510125	6.21429E-05	0.036803504
592.279991	2440.688077	5.50829E-05	0.032624517
592.319991	2012.688778	4.54236E-05	0.026905296
592.359991	1497.781728	3.38028E-05	0.020023454
592.399991	1036.841002	2.34001E-05	0.013862194
592.439991	696.925248	1.57286E-05	0.009318271
592.479991	505.461198	1.14076E-05	0.006758748
592.519991	457.145085	1.03171E-05	0.006113104
592.559991	463.856439	1.04686E-05	0.00620327
592.599991	512.714292	1.15712E-05	0.006857121
592.639991	563.336387	1.27137E-05	0.007534657
592.679991	581.864502	1.31319E-05	0.007782997
592.719991	570.180662	1.28682E-05	0.00762723
592.759991	532.111665	1.2009E-05	0.007118466
592.799991	512.041703	1.15561E-05	0.006850437
592.839991	502.158286	1.1333E-05	0.006718663
592.879991	536.162856	1.21004E-05	0.007174114
592.919991	677.472724	1.52896E-05	0.009065519
592.959991	931.278377	2.10177E-05	0.012462629
592.999991	1429.207481	3.22552E-05	0.019127346
593.039991	2175.962625	4.91084E-05	0.029123272
593.079991	3082.16202	6.95601E-05	0.041254706
593.119991	3947.323801	8.90856E-05	0.05283845
593.159991	4623.093392	0.000104337	0.061888403
593.199991	4887.675753	0.000110308	0.065434725
593.239991	4560.015269	0.000102913	0.061052222
593.279991	3803.064812	8.58299E-05	0.050921148
593.319991	2842.582128	6.41531E-05	0.038063328
593.359991	1912.989871	4.31735E-05	0.025617438
593.399991	1166.843558	2.6334E-05	0.015626615
593.439991	682.668055	1.54069E-05	0.009143051

593.479991	451.354623	1.01864E-05	0.006045451
593.519991	402.633974	9.08689E-06	0.00539325
593.559991	406.022801	9.16337E-06	0.005439009
593.599991	440.635901	9.94454E-06	0.005903078
593.639991	500.4964	1.12955E-05	0.006705464
593.679991	505.014532	1.13975E-05	0.006766453
593.719991	476.571313	1.07556E-05	0.006385785
593.759991	430.700391	9.72031E-06	0.00577153
593.799991	398.196305	8.98674E-06	0.005336324
593.839991	410.617438	9.26706E-06	0.005503153
593.879991	425.789789	9.60948E-06	0.005706879
593.919991	454.450224	1.02563E-05	0.006091427
593.959991	600.732306	1.35577E-05	0.008052726
593.999991	920.677832	2.07784E-05	0.012342379
594.039991	1426.929433	3.22038E-05	0.019130351
594.079991	2027.919342	4.57673E-05	0.027189446
594.119991	2561.31725	5.78054E-05	0.034343322
594.159991	2888.080894	6.518E-05	0.038727326
594.199991	2974.392353	6.71279E-05	0.039887393
594.239991	2747.735913	6.20126E-05	0.03685035
594.279991	2305.439191	5.20306E-05	0.030920716
594.319991	1775.480615	4.00701E-05	0.023814476
594.359991	1252.113413	2.82585E-05	0.016795695
594.399991	817.246044	1.84441E-05	0.010963176
594.439991	537.690112	1.21349E-05	0.00721348
594.479991	393.020065	8.86992E-06	0.005272987
594.519991	332.98163	7.51493E-06	0.004467777
594.559991	361.688591	8.16281E-06	0.004853279
594.599991	457.451924	1.03241E-05	0.006138682
594.639991	538.736359	1.21585E-05	0.007229948
594.679991	578.646829	1.30593E-05	0.007766076
594.719991	563.552921	1.27186E-05	0.007564008
594.759991	528.504919	1.19276E-05	0.007094071
594.799991	495.469895	1.11821E-05	0.006651092
594.839991	490.536653	1.10707E-05	0.006585312
594.879991	624.369189	1.40911E-05	0.008382539
594.919991	967.118236	2.18265E-05	0.012985028
594.959991	1751.104593	3.952E-05	0.023512814
594.999991	3407.906031	7.69117E-05	0.045762456
595.039991	6132.786838	0.000138408	0.082358567
595.079991	9905.391571	0.000223551	0.133030662
595.119991	14032.77633	0.0003167	0.18847463
595.159991	16967.08953	0.000382924	0.227900793
595.199991	17230.78031	0.000388875	0.231458225
595.239991	14515.8805	0.000327603	0.195002528
595.279991	10370.3814	0.000234045	0.139322343

595.319991	6534.532083	0.000147475	0.087794987
595.359991	3664.385859	8.27001E-05	0.049236322
595.399991	1883.483901	4.25076E-05	0.02530903
595.439991	967.262965	2.18298E-05	0.012998323
595.479991	552.239041	1.24633E-05	0.007421625
595.519991	418.922105	9.45449E-06	0.005630337
595.559991	440.511658	9.94173E-06	0.005920899
595.599991	474.550425	1.07099E-05	0.006378841
595.639991	489.244518	1.10416E-05	0.006576799
595.679991	462.752595	1.04437E-05	0.006221092
595.719991	426.491821	9.62533E-06	0.005733999
595.759991	395.882737	8.93452E-06	0.005322831
595.799991	355.243438	8.01735E-06	0.004776737
595.839991	328.122475	7.40527E-06	0.004412354
595.879991	351.625822	7.93571E-06	0.004728728
595.919991	483.944371	1.0922E-05	0.006508609
595.959991	840.985394	1.89799E-05	0.011311243
595.999991	1497.606943	3.37989E-05	0.020144146
596.039991	2575.189707	5.81184E-05	0.034640917
596.079991	4228.189988	9.54244E-05	0.056880551
596.119991	6056.783622	0.000136693	0.081485531
596.159991	7220.636216	0.00016296	0.097150054
596.199991	7225.4616	0.000163069	0.0972215
596.239991	6210.975351	0.000140173	0.083576786
596.279991	4663.632624	0.000105252	0.062759478
596.319991	3144.012105	7.0956E-05	0.042312466
596.359991	1902.639362	4.29399E-05	0.02560765
596.399991	1082.068293	2.44208E-05	0.014564549
596.439991	645.770366	1.45741E-05	0.008692599
596.479991	478.116834	1.07904E-05	0.006436276
596.519991	439.230078	9.91281E-06	0.00591319
596.559991	518.561901	1.17032E-05	0.006981673
596.599991	695.862673	1.57047E-05	0.009369395
596.639991	833.992275	1.8822E-05	0.011229984
596.679991	843.726911	1.90417E-05	0.011361826
596.719991	756.759399	1.7079E-05	0.010191384
596.759991	638.497992	1.441E-05	0.008599318
596.799991	546.148617	1.23258E-05	0.007356045
596.839991	491.139746	1.10843E-05	0.006615577
596.879991	551.433704	1.24451E-05	0.007428226
596.919991	772.191799	1.74273E-05	0.0104027
596.959991	1127.81692	2.54533E-05	0.015194576
596.999991	1691.141668	3.81667E-05	0.022785527
597.039991	2669.896073	6.02558E-05	0.035975143
597.079991	4003.16822	9.03459E-05	0.053943748
597.119991	5243.814704	0.000118346	0.07066652

597.159991	6045.742583	0.000136444	0.081478891
597.199991	6110.47505	0.000137905	0.082356811
597.239991	5423.819884	0.000122408	0.073106989
597.279991	4229.288929	9.54492E-05	0.057009874
597.319991	3020.828135	6.81759E-05	0.040722818
597.359991	2002.457725	4.51927E-05	0.0269963
597.399991	1258.99281	2.84137E-05	0.016974353
597.439991	766.592563	1.73009E-05	0.010336265
597.479991	494.863394	1.11684E-05	0.006672882
597.519991	395.516086	8.92625E-06	0.005333611
597.559991	420.902499	9.49918E-06	0.005676332
597.599991	487.318175	1.09981E-05	0.00657246
597.639991	552.777331	1.24754E-05	0.007455807
597.679991	570.103012	1.28664E-05	0.007690008
597.719991	531.337393	1.19915E-05	0.007167586
597.759991	454.254803	1.02519E-05	0.006128175
597.799991	373.664785	8.43309E-06	0.005041303
597.839991	334.302001	7.54473E-06	0.004510542
597.879991	330.434108	7.45744E-06	0.004458653
597.919991	370.700677	8.3662E-06	0.005002317
597.959991	519.778419	1.17307E-05	0.007014474
597.999991	867.331034	1.95745E-05	0.011705523
598.039991	1369.692974	3.09121E-05	0.01848665
598.079991	1953.014801	4.40768E-05	0.026361466
598.119991	2622.741039	5.91916E-05	0.035403686
598.159991	3090.360335	6.97451E-05	0.041718745
598.199991	3185.786683	7.18988E-05	0.043009842
598.239991	2972.071836	6.70755E-05	0.040127259
598.279991	2498.329962	5.63838E-05	0.033733316
598.319991	1904.663101	4.29856E-05	0.02571914
598.359991	1335.067799	3.01306E-05	0.018028958
598.399991	877.872395	1.98124E-05	0.011855714
598.439991	549.649043	1.24048E-05	0.007423536
598.479991	361.372147	8.15567E-06	0.004881003
598.519991	330.374613	7.45609E-06	0.004462622
598.559991	388.955478	8.77818E-06	0.005254269
598.599991	456.270265	1.02974E-05	0.006164014
598.639991	502.130955	1.13324E-05	0.006784026
598.679991	492.683311	1.11192E-05	0.006656828
598.719991	447.418837	1.00976E-05	0.006045647
598.759991	419.007719	9.45642E-06	0.005662126
598.799991	417.074218	9.41278E-06	0.005636375
598.839991	424.832398	9.58788E-06	0.005741603
598.879991	459.970225	1.03809E-05	0.006216906
598.919991	556.009779	1.25484E-05	0.007515467
598.959991	813.304072	1.83551E-05	0.010993995

598.999991	1261.802185	2.84771E-05	0.017057793
599.039991	1905.560126	4.30058E-05	0.025762217
599.079991	2716.338946	6.1304E-05	0.036725992
599.119991	3509.985316	7.92155E-05	0.047459577
599.159991	3929.230946	8.86773E-05	0.053131872
599.199991	3965.05613	8.94858E-05	0.053619887
599.239991	3731.202915	8.42081E-05	0.050460833
599.279991	3173.114886	7.16128E-05	0.042916108
599.319991	2430.475588	5.48524E-05	0.032874169
599.359991	1692.158076	3.81897E-05	0.022889349
599.399991	1081.097636	2.43989E-05	0.014624681
599.439991	666.138057	1.50338E-05	0.009011866
599.479991	443.635452	1.00122E-05	0.006002134
599.519991	347.693816	7.84697E-06	0.004704412
599.559991	362.640624	8.18429E-06	0.004906975
599.599991	438.715761	9.9012E-06	0.005936762
599.639991	495.915183	1.11921E-05	0.00671124
599.679991	481.934983	1.08766E-05	0.00652248
599.719991	442.231618	9.98055E-06	0.005985536
599.759991	386.759432	8.72862E-06	0.005235078
599.799991	308.440733	6.96108E-06	0.004175254
599.839991	250.210158	5.64689E-06	0.003387233
599.879991	255.569834	5.76786E-06	0.003460021
599.919991	314.424154	7.09612E-06	0.004257101
599.959991	454.20656	1.02508E-05	0.006150076
599.999991	696.919484	1.57285E-05	0.009437101
600.039991	1058.866644	2.38971E-05	0.014339243
600.079991	1528.503998	3.44962E-05	0.020700483
600.119991	2001.120624	4.51625E-05	0.027102922
600.159991	2413.638731	5.44725E-05	0.032692194
600.199991	2608.578957	5.8872E-05	0.03533497
600.239991	2485.803866	5.61011E-05	0.033674142
600.279991	2123.866388	4.79327E-05	0.028773044
600.319991	1688.231321	3.8101E-05	0.02287281
600.359991	1208.528514	2.72748E-05	0.016374702
600.399991	795.199646	1.79465E-05	0.010775107
600.439991	524.237263	1.18313E-05	0.007103989
600.479991	369.002176	8.32787E-06	0.005000716
600.519991	321.00544	7.24465E-06	0.004350555
600.559991	352.830029	7.96288E-06	0.004782189
600.599991	406.496784	9.17407E-06	0.005509944
600.639991	457.470067	1.03245E-05	0.006201285
600.679991	495.83747	1.11904E-05	0.006721826
600.719991	486.480212	1.09792E-05	0.006595413
600.759991	448.714629	1.01269E-05	0.006083815
600.799991	402.90839	9.09308E-06	0.005463123

600.839991	370.019218	8.35082E-06	0.005017506
600.879991	388.818674	8.7751E-06	0.00527278
600.919991	498.564119	1.12519E-05	0.00676149
600.959991	701.7725	1.5838E-05	0.009518021
600.999991	1023.722071	2.3104E-05	0.013885493
601.039991	1607.455989	3.6278E-05	0.021804554
601.079991	2340.093466	5.28127E-05	0.031744627
601.119991	2893.077269	6.52927E-05	0.039248762
601.159991	3308.941563	7.46782E-05	0.044893547
601.199991	3453.640538	7.79439E-05	0.046859846
601.239991	3159.184205	7.12984E-05	0.042867442
601.279991	2630.723734	5.93718E-05	0.035699058
601.319991	2051.594582	4.63016E-05	0.027842097
601.359991	1476.266206	3.33173E-05	0.020035674
601.399991	986.026969	2.22533E-05	0.013383108
601.439991	609.125995	1.37471E-05	0.008268071
601.479991	400.166499	9.0312E-06	0.005432086
601.519991	335.502778	7.57183E-06	0.004554607
601.559991	352.372152	7.95255E-06	0.004783935
601.599991	398.332393	8.98981E-06	0.005408268
601.639991	439.803397	9.92575E-06	0.005971728
601.679991	465.617029	1.05083E-05	0.006322651
601.719991	463.726317	1.04657E-05	0.006297395
601.759991	402.78924	9.09039E-06	0.005470234
601.799991	327.718928	7.39616E-06	0.004451009
601.839991	294.704093	6.65106E-06	0.004002874
601.879991	295.118514	6.66041E-06	0.00400877
601.919991	351.783259	7.93926E-06	0.004778798
601.959991	478.97051	1.08097E-05	0.006507005
601.999991	698.737598	1.57695E-05	0.00949326
602.039991	1060.233815	2.3928E-05	0.014405613
602.079991	1545.710917	3.48845E-05	0.021003285
602.119991	2038.348823	4.60027E-05	0.027699142
602.159991	2440.928258	5.50884E-05	0.033172001
602.199991	2719.498748	6.13753E-05	0.036960204
602.239991	2804.142836	6.32856E-05	0.038113118
602.279991	2621.188929	5.91566E-05	0.035628826
602.319991	2157.964073	4.87022E-05	0.029334335
602.359991	1558.08018	3.51637E-05	0.021181206
602.399991	1040.970606	2.34933E-05	0.014152338
602.439991	687.988345	1.55269E-05	0.009354049
602.479991	466.342548	1.05247E-05	0.006340922
602.519991	376.617434	8.49973E-06	0.005121258
602.559991	369.393404	8.33669E-06	0.005023359
602.599991	403.478762	9.10595E-06	0.005487248
602.639991	463.207805	1.0454E-05	0.006299971

602.679991	469.915748	1.06053E-05	0.006391629
602.719991	426.717746	9.63043E-06	0.00580445
602.759991	411.36139	9.28385E-06	0.005595936
602.799991	378.670286	8.54606E-06	0.005151565
602.839991	357.236611	8.06233E-06	0.004860297
602.879991	386.453063	8.72171E-06	0.005258143
602.919991	461.844352	1.04232E-05	0.006284346
602.959991	636.908506	1.43741E-05	0.00866703
602.999991	1002.526177	2.26256E-05	0.013643248
603.039991	1580.842057	3.56774E-05	0.021514901
603.079991	2352.489578	5.30924E-05	0.032018972
603.119991	3104.806229	7.00712E-05	0.042261313
603.159991	3602.431734	8.13019E-05	0.049038033
603.199991	3766.843186	8.50124E-05	0.051279481
603.239991	3527.710206	7.96155E-05	0.048027257
603.279991	3051.857529	6.88762E-05	0.041551617
603.319991	2401.309324	5.41942E-05	0.032696448
603.359991	1668.928771	3.76654E-05	0.022725794
603.399991	1056.516243	2.38441E-05	0.01438753
603.439991	646.902389	1.45997E-05	0.008810034
603.479991	438.773024	9.9025E-06	0.005975958
603.519991	348.454698	7.86414E-06	0.004746164
603.559991	383.44122	8.65373E-06	0.005223048
603.599991	440.021178	9.93067E-06	0.005994149
603.639991	478.856967	1.08071E-05	0.006523619
603.679991	496.071427	1.11956E-05	0.006758585
603.719991	440.879634	9.95004E-06	0.006007038
603.759991	354.486136	8.00026E-06	0.004830236
603.799991	297.710336	6.71891E-06	0.004056876
603.839991	274.499404	6.19507E-06	0.003740831
603.879991	262.382932	5.92162E-06	0.003575946
603.919991	279.213326	6.30146E-06	0.003805575
603.959991	377.885335	8.52835E-06	0.00515078
603.999991	607.013827	1.36995E-05	0.008274472
604.039991	980.788826	2.2135E-05	0.013370448
604.079991	1458.568327	3.29179E-05	0.019885018
604.119991	1958.998103	4.42119E-05	0.026709268
604.159991	2414.152565	5.44841E-05	0.03291709
604.199991	2641.059294	5.9605E-05	0.036013358
604.239991	2563.988075	5.78656E-05	0.034964733
604.279991	2221.599725	5.01384E-05	0.030297639
604.319991	1739.36809	3.92551E-05	0.023722651
604.359991	1244.4589	2.80857E-05	0.016973876
604.399991	833.778766	1.88172E-05	0.011373131
604.439991	555.234632	1.25309E-05	0.00757416
604.479991	403.70445	9.11105E-06	0.005507446

604.519991	341.754744	7.71293E-06	0.004662619
604.559991	363.783171	8.21008E-06	0.004963486
604.599991	405.711277	9.15634E-06	0.005535922
604.639991	436.390293	9.84872E-06	0.005954931
604.679991	510.704196	1.15259E-05	0.00696947
604.719991	556.425508	1.25577E-05	0.007593922
604.759991	519.129091	1.1716E-05	0.00708538
604.799991	423.0525	9.54771E-06	0.005774452
604.839991	334.761971	7.55511E-06	0.004569634
604.879991	328.602682	7.4161E-06	0.004485853
604.919991	401.119482	9.05271E-06	0.005476164
604.959991	583.362456	1.31657E-05	0.007964708
604.999991	983.954963	2.22065E-05	0.013434928
605.039991	1609.159995	3.63165E-05	0.021972935
605.079991	2384.503273	5.38149E-05	0.03256233
605.119991	3162.19688	7.13664E-05	0.043185223
605.159991	3835.441794	8.65606E-05	0.052382999
605.199991	4131.685486	9.32464E-05	0.056432712
605.239991	3921.577189	8.85045E-05	0.053566484
605.279991	3355.707306	7.57336E-05	0.045840057
605.319991	2580.980184	5.82491E-05	0.035259361
605.359991	1766.395994	3.98651E-05	0.024132735
605.399991	1104.55858	2.49283E-05	0.015091622
605.439991	686.559869	1.54947E-05	0.009381111
605.479991	455.620618	1.02827E-05	0.006225983
605.519991	349.733513	7.893E-06	0.004779368
605.559991	390.954083	8.82329E-06	0.005343031
605.599991	484.016945	1.09236E-05	0.006615325
605.639991	492.35704	1.11118E-05	0.006729758
605.679991	465.677102	1.05097E-05	0.006365505
605.719991	437.232288	9.86772E-06	0.005977078
605.759991	381.143089	8.60187E-06	0.005210668
605.799991	330.896711	7.46788E-06	0.00452404
605.839991	284.381604	6.4181E-06	0.00388834
605.879991	256.958052	5.79919E-06	0.00351361
605.919991	287.835196	6.49604E-06	0.00393608
605.959991	384.996609	8.68884E-06	0.005265088
605.999991	599.95414	1.35401E-05	0.008205318
606.039991	972.559391	2.19493E-05	0.01330216
606.079991	1449.362652	3.27101E-05	0.019824935
606.119991	1953.937005	4.40976E-05	0.026728459
606.159991	2333.576893	5.26656E-05	0.031923768
606.199991	2448.630617	5.52622E-05	0.033499934
606.239991	2290.698167	5.16979E-05	0.031341314
606.279991	1988.641873	4.48809E-05	0.027210378
606.319991	1555.362108	3.51024E-05	0.02128326

606.359991	1111.594506	2.50871E-05	0.015211838
606.399991	774.484823	1.7479E-05	0.010599292
606.439991	541.454727	1.22199E-05	0.007410623
606.479991	416.653573	9.40329E-06	0.005702908
606.519991	380.549474	8.58847E-06	0.00520908
606.559991	382.710714	8.63725E-06	0.005239009
606.599991	437.946678	9.88385E-06	0.005995541
606.639991	486.77761	1.09859E-05	0.006664482
606.679991	492.885591	1.11237E-05	0.006748551
606.719991	445.105809	1.00454E-05	0.006094756
606.759991	398.231809	8.98754E-06	0.005453278
606.799991	364.932875	8.23603E-06	0.004997621
606.839991	358.377824	8.08809E-06	0.004908175
606.879991	384.714393	8.68247E-06	0.005269216
606.919991	470.652168	1.0622E-05	0.006446682
606.959991	656.215425	1.48099E-05	0.008988997
606.999991	1077.686841	2.43219E-05	0.014763388
607.039991	1829.792755	4.12959E-05	0.025068247
607.079991	2834.206427	6.39641E-05	0.03883132
607.119991	3945.130671	8.90361E-05	0.054055599
607.159991	4775.736198	0.000107782	0.065440744
607.199991	5096.657074	0.000115024	0.069842846
607.239991	4943.90363	0.000111577	0.067754028
607.279991	4365.480623	9.85228E-05	0.059830935
607.319991	3341.712194	7.54178E-05	0.045802731
607.359991	2252.875774	5.08443E-05	0.030880774
607.399991	1379.025628	3.11227E-05	0.01890392
607.439991	826.577511	1.86547E-05	0.011331613
607.479991	519.997488	1.17356E-05	0.007129154
607.519991	404.815888	9.13613E-06	0.005550382
607.559991	398.623362	8.99637E-06	0.005465837
607.599991	435.144768	9.82061E-06	0.005967003
607.639991	487.961194	1.10126E-05	0.006691699
607.679991	500.671051	1.12994E-05	0.006866449
607.719991	466.314462	1.05241E-05	0.006395686
607.759991	420.707096	9.49477E-06	0.005770543
607.799991	371.463075	8.3834E-06	0.005095433
607.839991	341.601391	7.70947E-06	0.004686123
607.879991	317.194413	7.15864E-06	0.004351592
607.919991	320.517073	7.23362E-06	0.004397465
607.959991	446.794122	1.00835E-05	0.006130377
607.999991	670.775087	1.51385E-05	0.009204183
608.039991	1002.844191	2.26328E-05	0.013761645
608.079991	1507.673031	3.40261E-05	0.020690579
608.119991	2092.26939	4.72196E-05	0.028715186
608.159991	2595.28149	5.85719E-05	0.035621079

608.199991	2876.807173	6.49255E-05	0.039487707
608.239991	2773.27223	6.25889E-05	0.038069067
608.279991	2447.087659	5.52274E-05	0.033593698
608.319991	2055.762457	4.63957E-05	0.028223429
608.359991	1618.073248	3.65177E-05	0.022215883
608.399991	1163.781098	2.62649E-05	0.015979576
608.439991	780.383916	1.76122E-05	0.010715953
608.479991	537.940625	1.21406E-05	0.007387294
608.519991	420.150343	9.48221E-06	0.005770113
608.559991	419.713184	9.47234E-06	0.005764488
608.599991	504.784161	1.13923E-05	0.006933339
608.639991	568.381621	1.28276E-05	0.007807379
608.679991	596.067081	1.34524E-05	0.008188209
608.719991	578.664079	1.30596E-05	0.007949665
608.759991	556.806026	1.25663E-05	0.007649883
608.799991	536.308912	1.21037E-05	0.00736876
608.839991	527.326426	1.1901E-05	0.007245819
608.879991	564.5139	1.27403E-05	0.007757309
608.919991	749.889985	1.6924E-05	0.010305345
608.959991	1077.443043	2.43164E-05	0.014807708
608.999991	1585.961414	3.57929E-05	0.0217979
609.039991	2400.957194	5.41863E-05	0.033001599
609.079991	3520.95763	7.94631E-05	0.04839939
609.119991	4640.582588	0.000104731	0.063794041
609.159991	5272.755197	0.000118999	0.072489272
609.199991	5232.646423	0.000118094	0.071942584
609.239991	4718.633959	0.000106493	0.064879792
609.279991	3843.642612	8.67457E-05	0.052852395
609.319991	2812.110828	6.34654E-05	0.038670752
609.359991	1911.192517	4.3133E-05	0.026283496
609.399991	1225.50324	2.76579E-05	0.016854725
609.439991	763.693678	1.72355E-05	0.010504005
609.479991	486.055859	1.09696E-05	0.006685754
609.519991	377.692757	8.524E-06	0.005195548
609.559991	384.561094	8.67901E-06	0.005290376
609.599991	446.134457	1.00686E-05	0.006137839
609.639991	468.145461	1.05654E-05	0.006441085
609.679991	455.302155	1.02755E-05	0.006264788
609.719991	456.674664	1.03065E-05	0.006284086
609.759991	448.962036	1.01324E-05	0.006178361
609.799991	403.655087	9.10993E-06	0.005555237
609.839991	352.873046	7.96385E-06	0.004856676
609.879991	355.73192	8.02837E-06	0.004896345
609.919991	429.004755	9.68204E-06	0.00590527
609.959991	589.632224	1.33072E-05	0.008116846
609.999991	865.349969	1.95297E-05	0.011913143

610.039991	1267.167534	2.85982E-05	0.017446049
610.079991	1773.509411	4.00256E-05	0.02441884
610.119991	2295.058533	5.17963E-05	0.031601942
610.159991	2714.246164	6.12568E-05	0.037376421
610.199991	2847.830624	6.42716E-05	0.039218512
610.239991	2642.566389	5.9639E-05	0.036394129
610.279991	2222.795411	5.01654E-05	0.030614938
610.319991	1712.463851	3.86479E-05	0.023587601
610.359991	1208.654028	2.72776E-05	0.016649179
610.399991	790.783286	1.78469E-05	0.010893734
610.439991	524.217801	1.18309E-05	0.007222033
610.479991	397.781491	8.97737E-06	0.005480507
610.519991	377.460114	8.51875E-06	0.005200867
610.559991	424.496332	9.58029E-06	0.005849342
610.599991	481.493086	1.08666E-05	0.006635163
610.639991	549.215078	1.2395E-05	0.007568894
610.679991	606.499679	1.36879E-05	0.008358898
610.719991	566.907301	1.27943E-05	0.00781374
610.759991	540.366203	1.21953E-05	0.007448409
610.799991	506.038677	1.14206E-05	0.006975695
610.839991	485.671873	1.09609E-05	0.006695379
610.879991	545.798324	1.23179E-05	0.007524763
610.919991	705.48573	1.59218E-05	0.009726964
610.959991	979.051282	2.20958E-05	0.013499664
610.999991	1485.37686	3.35229E-05	0.020482484
611.039991	2298.107802	5.18651E-05	0.031691645
611.079991	3355.533446	7.57297E-05	0.046276914
611.119991	4354.085877	9.82657E-05	0.060052105
611.159991	5050.875737	0.000113991	0.069666879
611.199991	5116.189237	0.000115465	0.070572369
611.239991	4569.10124	0.000103118	0.063029999
611.279991	3678.835224	8.30262E-05	0.050752247
611.319991	2675.452197	6.03812E-05	0.036912253
611.359991	1818.13776	4.10328E-05	0.025085835
611.399991	1160.36939	2.61879E-05	0.016011295
611.439991	706.19083	1.59377E-05	0.009744974
611.479991	458.644222	1.0351E-05	0.006329406
611.519991	407.486954	9.19641E-06	0.00562379
611.559991	452.616694	1.02149E-05	0.006247041
611.599991	494.046518	1.11499E-05	0.006819304
611.639991	504.503692	1.13859E-05	0.0069641
611.679991	508.447252	1.14749E-05	0.007018995
611.719991	513.930577	1.15987E-05	0.007095155
611.759991	476.820915	1.07612E-05	0.006583262
611.799991	405.508155	9.15175E-06	0.005599043
611.839991	347.005896	7.83144E-06	0.004791588

611.879991	338.94777	7.64958E-06	0.004680624
611.919991	418.638732	9.44809E-06	0.005781477
611.959991	543.181807	1.22589E-05	0.00750193
611.999991	770.139923	1.7381E-05	0.010637163
612.039991	1174.787091	2.65133E-05	0.016227205
612.079991	1671.269359	3.77182E-05	0.023086569
612.119991	2179.26505	4.9183E-05	0.030105883
612.159991	2626.592901	5.92785E-05	0.036287952
612.199991	2750.076224	6.20654E-05	0.037996431
612.239991	2582.460307	5.82825E-05	0.035682897
612.279991	2176.40015	4.91183E-05	0.030074164
612.319991	1682.594552	3.79738E-05	0.023252126
612.359991	1206.419603	2.72272E-05	0.016672854
612.399991	824.713995	1.86126E-05	0.011398384
612.439991	567.55484	1.28089E-05	0.007844696
612.479991	435.220572	9.82232E-06	0.006015976
612.519991	395.680221	8.92995E-06	0.005469774
612.559991	403.377569	9.10367E-06	0.005576544
612.599991	451.351984	1.01864E-05	0.00624018
612.639991	496.57604	1.1207E-05	0.006865874
612.679991	539.051698	1.21656E-05	0.007453648
612.719991	553.318597	1.24876E-05	0.007651421
612.759991	517.556447	1.16805E-05	0.00715736
612.799991	455.92359	1.02896E-05	0.006305443
612.839991	437.04798	9.86356E-06	0.006044787
612.879991	468.978446	1.05842E-05	0.006486838
612.919991	599.047145	1.35197E-05	0.00828647
612.959991	858.912844	1.93845E-05	0.011881902
612.999991	1209.416669	2.72948E-05	0.016731743
613.039991	1775.036819	4.00601E-05	0.024558449
613.079991	2495.676968	5.6324E-05	0.034531089
613.119991	3203.798019	7.23053E-05	0.0443318
613.159991	3723.40998	8.40322E-05	0.051525169
613.199991	3825.942417	8.63462E-05	0.052947484
613.239991	3593.331436	8.10965E-05	0.049731608
613.279991	3076.05309	6.94222E-05	0.042575267
613.319991	2428.085117	5.47985E-05	0.033609015
613.359991	1762.747295	3.97828E-05	0.024401148
613.399991	1174.57452	2.65085E-05	0.01626032
613.439991	745.128556	1.68165E-05	0.010315922
613.479991	494.686834	1.11644E-05	0.006849132
613.519991	390.207294	8.80644E-06	0.005402924
613.559991	387.527256	8.74595E-06	0.005366165
613.599991	410.382094	9.26175E-06	0.005683011
613.639991	458.73388	1.0353E-05	0.006353005
613.679991	498.173058	1.12431E-05	0.006899648

613.719991	479.434323	1.08202E-05	0.006640551
613.759991	430.989794	9.72684E-06	0.005969945
613.799991	370.148697	8.35374E-06	0.005127526
613.839991	330.65389	7.4624E-06	0.004580718
613.879991	318.891272	7.19693E-06	0.004418052
613.919991	329.569039	7.43791E-06	0.004566284
613.959991	390.850066	8.82094E-06	0.005415705
613.999991	556.393548	1.2557E-05	0.007710015
614.039991	908.244456	2.04978E-05	0.012586476
614.079991	1409.554175	3.18117E-05	0.019534913
614.119991	1956.052207	4.41454E-05	0.027110557
614.159991	2369.030455	5.34657E-05	0.032836505
614.199991	2529.633089	5.70903E-05	0.035064859
614.239991	2436.218958	5.49821E-05	0.033772185
614.279991	2199.840581	4.96473E-05	0.030497366
614.319991	1821.03042	4.10981E-05	0.025247397
614.359991	1376.710588	3.10704E-05	0.019088435
614.399991	968.299707	2.18532E-05	0.013426591
614.439991	630.879574	1.42381E-05	0.008748442
614.479991	443.095115	1E-05	0.006144824
614.519991	375.749171	8.48014E-06	0.005211213
614.559991	360.063474	8.12613E-06	0.004993995
614.599991	410.942835	9.27441E-06	0.005700051
614.639991	472.980939	1.06745E-05	0.006560987
614.679991	495.159726	1.11751E-05	0.006869089
614.719991	475.753582	1.07371E-05	0.006600307
614.759991	424.204477	9.5737E-06	0.00588553
614.799991	376.292319	8.49239E-06	0.005221123
614.839991	381.677564	8.61393E-06	0.005296189
614.879991	370.992845	8.37279E-06	0.005148262
614.919991	424.922349	9.58991E-06	0.005897025
614.959991	589.906435	1.33134E-05	0.008187188
614.999991	899.752985	2.03062E-05	0.012488295
615.039991	1379.269281	3.11282E-05	0.01914508
615.079991	2004.128979	4.52304E-05	0.027820314
615.119991	2640.111494	5.95836E-05	0.036651088
615.159991	3085.569076	6.9637E-05	0.042837894
615.199991	3220.805031	7.26891E-05	0.044718323
615.239991	3047.023094	6.87671E-05	0.04230825
615.279991	2617.814715	5.90804E-05	0.036351008
615.319991	2063.859996	4.65784E-05	0.028660649
615.359991	1536.568014	3.46782E-05	0.021339577
615.399991	1060.279814	2.3929E-05	0.01472593
615.439991	684.691935	1.54525E-05	0.009510113
615.479991	458.42417	1.0346E-05	0.006367753
615.519991	352.910729	7.9647E-06	0.004902434

615.559991	338.135548	7.63125E-06	0.004697491
615.599991	378.480908	8.54179E-06	0.005258324
615.639991	417.456075	9.4214E-06	0.005800192
615.679991	435.143845	9.82059E-06	0.006046341
615.719991	427.155311	9.6403E-06	0.005935726
615.759991	397.367271	8.96803E-06	0.005522151
615.799991	379.00048	8.55351E-06	0.005267253
615.839991	342.673558	7.73366E-06	0.0047627
615.879991	301.46826	6.80372E-06	0.004190274
615.919991	305.019816	6.88387E-06	0.004239915
615.959991	400.604971	9.0411E-06	0.005568954
615.999991	604.686219	1.36469E-05	0.008406506
616.039991	904.807895	2.04203E-05	0.012579693
616.079991	1315.133656	2.96807E-05	0.018285707
616.119991	1791.616156	4.04343E-05	0.024912369
616.159991	2149.954593	4.85215E-05	0.029896995
616.199991	2314.508456	5.22352E-05	0.032187349
616.239991	2287.30904	5.16214E-05	0.031811158
616.279991	2026.141128	4.57272E-05	0.028180748
616.319991	1581.801935	3.56991E-05	0.022002048
616.359991	1100.986561	2.48477E-05	0.015315149
616.399991	714.033987	1.61148E-05	0.009933133
616.439991	465.47951	1.05052E-05	0.00647584
616.479991	368.040354	8.30616E-06	0.00512058
616.519991	342.693276	7.73411E-06	0.004768233
616.559991	356.710524	8.05046E-06	0.004963591
616.599991	402.20971	9.07731E-06	0.005597071
616.639991	462.637465	1.04411E-05	0.006438389
616.679991	492.687817	1.11193E-05	0.006857036
616.719991	467.198072	1.0544E-05	0.006502702
616.759991	408.979413	9.2301E-06	0.005692754
616.799991	366.547378	8.27246E-06	0.005102456
616.839991	357.080471	8.05881E-06	0.004970996
616.879991	369.622877	8.34187E-06	0.005145935
616.919991	421.247443	9.50697E-06	0.005865039
616.959991	607.779887	1.37167E-05	0.008462683
616.999991	969.200526	2.18735E-05	0.013495953
617.039991	1466.949331	3.3107E-05	0.020428345
617.079991	2030.99888	4.58368E-05	0.028284982
617.119991	2610.144939	5.89073E-05	0.036352894
617.159991	3059.195333	6.90418E-05	0.042609823
617.199991	3204.379177	7.23184E-05	0.0446349
617.239991	3004.445238	6.78061E-05	0.041852664
617.279991	2634.326742	5.94531E-05	0.0366992
617.319991	2120.099704	4.78477E-05	0.02953734
617.359991	1573.115208	3.5503E-05	0.021918144

617.399991	1083.337679	2.44494E-05	0.015095073
617.439991	719.460378	1.62372E-05	0.010025508
617.479991	468.348616	1.057E-05	0.006526749
617.519991	353.106142	7.96911E-06	0.004921087
617.559991	353.817271	7.98516E-06	0.004931317
617.599991	401.557904	9.0626E-06	0.005597063
617.639991	456.75121	1.03082E-05	0.00636678
617.679991	481.597159	1.0869E-05	0.00671355
617.719991	460.188109	1.03858E-05	0.006415519
617.759991	421.524251	9.51322E-06	0.005876884
617.799991	370.193126	8.35474E-06	0.00516156
617.839991	320.279705	7.22827E-06	0.004465912
617.879991	273.755977	6.17829E-06	0.003817442
617.919991	279.170644	6.30049E-06	0.0038932
617.959991	383.085921	8.64572E-06	0.005342706
617.999991	578.887052	1.30647E-05	0.008073969
618.039991	817.362139	1.84467E-05	0.011400814
618.079991	1168.757032	2.63772E-05	0.016303231
618.119991	1639.936708	3.70111E-05	0.022877293
618.159991	2071.548888	4.6752E-05	0.028900199
618.199991	2225.503394	5.02265E-05	0.031050029
618.239991	2114.631331	4.77243E-05	0.029505061
618.279991	1812.230496	4.08995E-05	0.025287354
618.319991	1461.520753	3.29845E-05	0.020394968
618.359991	1105.847143	2.49574E-05	0.015432676
618.399991	783.666313	1.76863E-05	0.010937181
618.439991	561.489342	1.2672E-05	0.007836892
618.479991	402.950136	9.09402E-06	0.005624471
618.519991	312.613107	7.05524E-06	0.004363808
618.559991	336.262677	7.58898E-06	0.00469424
618.599991	401.762578	9.06722E-06	0.005608983
618.639991	460.782021	1.03992E-05	0.006433366
618.679991	492.782951	1.11214E-05	0.006880603
618.719991	472.42854	1.06621E-05	0.006596826
618.759991	442.67667	9.9906E-06	0.006181781
618.799991	400.424034	9.03701E-06	0.005592103
618.839991	373.755424	8.43514E-06	0.005220002
618.879991	361.093835	8.14938E-06	0.005043491
618.919991	393.538873	8.88162E-06	0.005497015
618.959991	524.66157	1.18409E-05	0.007329031
618.999991	832.205241	1.87817E-05	0.01162588
619.039991	1361.467962	3.07264E-05	0.019020892
619.079991	2074.500523	4.68186E-05	0.028984451
619.119991	2830.94469	6.38905E-05	0.039555872
619.159991	3414.426472	7.70588E-05	0.047711757
619.199991	3683.775505	8.31377E-05	0.051478852

619.239991	3561.106768	8.03692E-05	0.049767835
619.279991	3106.991461	7.01205E-05	0.043424204
619.319991	2428.369081	5.48049E-05	0.033941775
619.359991	1719.758754	3.88126E-05	0.024038947
619.399991	1129.201197	2.54845E-05	0.015785098
619.439991	715.36862	1.61449E-05	0.01000078
619.479991	477.367256	1.07735E-05	0.006673976
619.519991	374.920333	8.46143E-06	0.005242025
619.559991	355.321626	8.01911E-06	0.004968322
619.599991	368.918508	8.32598E-06	0.005158775
619.639991	422.581502	9.53708E-06	0.005909554
619.679991	444.705939	1.00364E-05	0.006219352
619.719991	429.22496	9.68701E-06	0.006003233
619.759991	382.073212	8.62286E-06	0.005344104
619.799991	336.323988	7.59036E-06	0.004704508
619.839991	287.211761	6.48197E-06	0.004017784
619.879991	269.726263	6.08735E-06	0.003773424
619.919991	273.133678	6.16425E-06	0.00382134
619.959991	346.314332	7.81583E-06	0.004845503
619.999991	556.145886	1.25514E-05	0.007781891
620.039991	877.339534	1.98003E-05	0.012276997
620.079991	1266.728263	2.85883E-05	0.017727028
620.119991	1652.899913	3.73036E-05	0.023132738
620.159991	2009.44472	4.53504E-05	0.028124484
620.199991	2271.571328	5.12662E-05	0.031795297
620.239991	2387.369373	5.38796E-05	0.033418283
620.279991	2325.746493	5.24889E-05	0.032557789
620.319991	2119.370819	4.78312E-05	0.029670679
620.359991	1832.914036	4.13663E-05	0.025662008
620.399991	1489.331748	3.36121E-05	0.020852973
620.439991	1154.485716	2.60551E-05	0.016165647
620.479991	840.910794	1.89782E-05	0.011775584
620.519991	580.341384	1.30975E-05	0.008127259
620.559991	429.878319	9.70175E-06	0.006020521
620.599991	419.126777	9.45911E-06	0.005870322
620.639991	448.606402	1.01244E-05	0.006283621
620.679991	466.142681	1.05202E-05	0.006529672
620.719991	464.327401	1.04792E-05	0.006504663
620.759991	431.270561	9.73318E-06	0.006041966
620.799991	400.965766	9.04924E-06	0.005617767
620.839991	353.029519	7.96738E-06	0.004946471
620.879991	327.310323	7.38694E-06	0.004586402
620.919991	373.423272	8.42764E-06	0.005232892
620.959991	525.647512	1.18631E-05	0.00736653
620.999991	845.822325	1.9089E-05	0.011854289
621.039991	1415.057791	3.19359E-05	0.019833461

621.079991	2123.409854	4.79224E-05	0.029763645
621.119991	2900.237601	6.54543E-05	0.040654988
621.159991	3456.732591	7.80136E-05	0.048458952
621.199991	3656.300616	8.25176E-05	0.05125994
621.239991	3545.538779	8.00179E-05	0.049710302
621.279991	3132.497068	7.06961E-05	0.04392207
621.319991	2523.864692	5.69601E-05	0.035390455
621.359991	1901.732403	4.29195E-05	0.02666843
621.399991	1358.614532	3.0662E-05	0.01905339
621.439991	917.424203	2.0705E-05	0.012866907
621.479991	612.024025	1.38125E-05	0.008584211
621.519991	435.883758	9.83729E-06	0.006114072
621.559991	359.401683	8.1112E-06	0.005041595
621.599991	370.07286	8.35203E-06	0.005191621
621.639991	418.546893	9.44602E-06	0.005872024
621.679991	454.04208	1.02471E-05	0.006370415
621.719991	456.880352	1.03112E-05	0.00641065
621.759991	442.105338	9.9777E-06	0.006203736
621.799991	395.218057	8.91952E-06	0.005546158
621.839991	311.960407	7.04051E-06	0.004378072
621.879991	253.175346	5.71381E-06	0.003553307
621.919991	249.483217	5.63049E-06	0.003501713
621.959991	323.734768	7.30624E-06	0.004544191
621.999991	517.466112	1.16785E-05	0.00726402
622.039991	844.851739	1.90671E-05	0.011860515
622.079991	1288.546799	2.90807E-05	0.018090526
622.119991	1779.936215	4.01707E-05	0.024990984
622.159991	2230.719118	5.03442E-05	0.031322162
622.199991	2440.290735	5.5074E-05	0.034267019
622.239991	2309.342762	5.21186E-05	0.032430308
622.279991	1993.066975	4.49807E-05	0.027990618
622.319991	1643.191042	3.70845E-05	0.023078446
622.359991	1272.380385	2.87159E-05	0.017871598
622.399991	908.653542	2.0507E-05	0.012763584
622.439991	637.256786	1.4382E-05	0.008951931
622.479991	458.153537	1.03399E-05	0.006436373
622.519991	370.752054	8.36736E-06	0.005208847
622.559991	365.324851	8.24487E-06	0.005132928
622.599991	383.293864	8.65041E-06	0.005385744
622.639991	427.268306	9.64285E-06	0.006004024
622.679991	478.67527	1.0803E-05	0.006726833
622.719991	478.188955	1.07921E-05	0.006720431
622.759991	430.136511	9.70758E-06	0.006045494
622.799991	373.724862	8.43445E-06	0.005252975
622.839991	336.187808	7.58729E-06	0.004725668
622.879991	336.763944	7.60029E-06	0.004734071

622.919991	407.963392	9.20717E-06	0.005735327
622.959991	556.899588	1.25684E-05	0.00782964
622.999991	916.399213	2.06819E-05	0.012884795
623.039991	1572.84392	3.54969E-05	0.022115986
623.079991	2566.824148	5.79296E-05	0.036094803
623.119991	3735.330641	8.43012E-05	0.052529769
623.159991	4711.584297	0.000106334	0.066263029
623.199991	5259.474154	0.000118699	0.073973219
623.239991	5193.071648	0.0001172	0.073043972
623.279991	4623.105689	0.000104337	0.065031199
623.319991	3643.809168	8.22357E-05	0.051259154
623.359991	2493.555902	5.62761E-05	0.035080259
623.399991	1521.603406	3.43405E-05	0.021407848
623.439991	874.618691	1.97389E-05	0.012306036
623.479991	514.037908	1.16011E-05	0.007233066
623.519991	360.230745	8.12991E-06	0.005069159
623.559991	353.736806	7.98335E-06	0.004978096
623.599991	398.671775	8.99747E-06	0.00561082
623.639991	438.719921	9.9013E-06	0.006174845
623.679991	481.228946	1.08607E-05	0.006773581
623.719991	475.789748	1.07379E-05	0.00669745
623.759991	411.772258	9.29313E-06	0.00579668
623.799991	359.094886	8.10427E-06	0.005055444
623.839991	342.927312	7.73939E-06	0.004828142
623.879991	355.195137	8.01626E-06	0.005001184
623.919991	359.474636	8.11284E-06	0.005061764
623.959991	404.320342	9.12495E-06	0.005693602
623.999991	570.459396	1.28745E-05	0.008033672
624.039991	847.022797	1.91161E-05	0.011929226
624.079991	1294.04832	2.92049E-05	0.018226174
624.119991	1920.841319	4.33507E-05	0.027056047
624.159991	2469.784975	5.57396E-05	0.034790432
624.199991	2720.98187	6.14088E-05	0.038331354
624.239991	2730.006089	6.16124E-05	0.038460946
624.279991	2483.124774	5.60407E-05	0.034985067
624.319991	1961.30999	4.4264E-05	0.027634922
624.359991	1456.07544	3.28616E-05	0.020517465
624.399991	1047.000407	2.36293E-05	0.01475416
624.439991	734.285985	1.65718E-05	0.010348102
624.479991	567.349111	1.28043E-05	0.007996016
624.519991	513.750631	1.15946E-05	0.007241082
624.559991	558.330603	1.26007E-05	0.007869921
624.599991	654.515959	1.47715E-05	0.009226287
624.639991	744.096142	1.67932E-05	0.010489713
624.679991	781.259661	1.76319E-05	0.011014322
624.719991	783.117012	1.76739E-05	0.011041214

624.759991	750.751708	1.69434E-05	0.010585571
624.799991	771.395293	1.74093E-05	0.010877341
624.839991	860.279807	1.94153E-05	0.012131466
624.879991	993.734943	2.24272E-05	0.014014317
624.919991	1242.059873	2.80316E-05	0.017517483
624.959991	1743.860757	3.93565E-05	0.024596243
624.999991	2725.934179	6.15205E-05	0.038450335
625.03999	4336.700795	9.78733E-05	0.061174724
625.07999	6171.037494	0.000139272	0.087055966
625.11999	7758.655496	0.000175102	0.10945979
625.15999	8504.151085	0.000191927	0.119984984
625.19999	8191.970311	0.000184881	0.115587824
625.23999	7053.317193	0.000159184	0.099527918
625.27999	5432.950699	0.000122614	0.076668162
625.31999	3706.488624	8.36503E-05	0.052308196
625.35999	2312.981301	5.22008E-05	0.032644271
625.39999	1367.666393	3.08663E-05	0.0193038
625.43999	821.419966	1.85383E-05	0.011594597
625.47999	561.207074	1.26657E-05	0.007922118
625.51999	471.95929	1.06515E-05	0.006662704
625.55999	431.073494	9.72873E-06	0.006085903
625.59999	406.274414	9.16905E-06	0.005736156
625.63999	432.730901	9.76613E-06	0.006110084
625.67999	499.910858	1.12823E-05	0.007059104
625.71999	553.464567	1.24909E-05	0.007815821
625.75999	542.272664	1.22383E-05	0.007658263
625.79999	489.348616	1.10439E-05	0.006911283
625.83999	450.172434	1.01598E-05	0.006358387
625.87999	453.691116	1.02392E-05	0.006408496
625.91999	505.83516	1.1416E-05	0.007145499
625.95999	704.847225	1.59074E-05	0.009957409
625.99999	1083.857879	2.44612E-05	0.015312688
626.03999	1700.756698	3.83837E-05	0.024029737
626.07999	2438.185659	5.50265E-05	0.034450962
626.11999	3149.800197	7.10866E-05	0.044508743
626.15999	3708.264624	8.36904E-05	0.05240356
626.19999	3772.862612	8.51483E-05	0.053319836
626.23999	3374.065179	7.6148E-05	0.047686892
626.27999	2686.699024	6.06351E-05	0.037974521
626.31999	1944.309631	4.38804E-05	0.027483147
626.35999	1335.943302	3.01504E-05	0.018884991
626.39999	879.513883	1.98494E-05	0.012433666
626.43999	579.38054	1.30758E-05	0.008191211
626.47999	431.972186	9.74901E-06	0.00610756
626.51999	379.880758	8.57338E-06	0.005371394
626.55999	399.116608	9.00751E-06	0.005643743

626.59999	465.525268	1.05063E-05	0.00658322
626.63999	518.474994	1.17013E-05	0.007332476
626.67999	519.139604	1.17163E-05	0.007342344
626.71999	508.204195	1.14695E-05	0.00718814
626.75999	494.74932	1.11658E-05	0.006998279
626.79999	457.986792	1.03361E-05	0.006478682
626.83999	435.210705	9.8221E-06	0.006156885
626.87999	469.433152	1.05945E-05	0.00664145
626.91999	582.830677	1.31537E-05	0.008246303
626.95999	838.239925	1.89179E-05	0.011860771
626.99999	1261.502635	2.84704E-05	0.017850914
627.03999	1890.369416	4.2663E-05	0.02675141
627.07999	2704.445234	6.10356E-05	0.03827418
627.11999	3518.645824	7.94109E-05	0.049800185
627.15999	4056.690708	9.15539E-05	0.057418917
627.19999	4074.129889	9.19474E-05	0.057669431
627.23999	3729.933016	8.41794E-05	0.052800682
627.27999	3079.731421	6.95052E-05	0.043599252
627.31999	2358.054873	5.3218E-05	0.033384724
627.35999	1703.29552	3.8441E-05	0.02411635
627.39999	1130.486773	2.55135E-05	0.016007177
627.43999	722.071897	1.62962E-05	0.010224861
627.47999	502.775135	1.13469E-05	0.007119974
627.51999	428.113422	9.66192E-06	0.00606305
627.55999	407.782986	9.20309E-06	0.005775494
627.59999	433.376492	9.7807E-06	0.00613837
627.63999	492.188392	1.1108E-05	0.006971829
627.67999	538.444082	1.21519E-05	0.007627525
627.71999	553.840771	1.24994E-05	0.007846133
627.75999	556.361516	1.25563E-05	0.007882346
627.79999	551.37535	1.24438E-05	0.007812201
627.83999	568.039776	1.28199E-05	0.008048825
627.87999	626.547863	1.41403E-05	0.008878419
627.91999	709.438678	1.6011E-05	0.010053654
627.95999	859.476306	1.93972E-05	0.012180655
627.99999	1138.897166	2.57033E-05	0.016141686
628.03999	1554.158687	3.50752E-05	0.022028626
628.07999	2096.6852	4.73193E-05	0.029720283
628.11999	2639.945801	5.95799E-05	0.037423326
628.15999	3023.937534	6.82461E-05	0.042869443
628.19999	3104.91746	7.00737E-05	0.044020274
628.23999	2883.249836	6.50709E-05	0.040880162
628.27999	2411.139125	5.44161E-05	0.034188516
628.31999	1788.649585	4.03673E-05	0.0253636
628.35999	1221.887421	2.75763E-05	0.017327842
628.39999	833.02061	1.88001E-05	0.011813992

628.43999	595.313094	1.34354E-05	0.008443335
628.47999	424.566412	9.58187E-06	0.006022015
628.51999	356.847984	8.05356E-06	0.005061825
628.55999	375.754144	8.48025E-06	0.005330344
628.59999	433.272842	9.77836E-06	0.00614668
628.63999	479.125053	1.08132E-05	0.006797601
628.67999	504.859667	1.1394E-05	0.007163167
628.71999	521.208067	1.17629E-05	0.007395596
628.75999	514.438497	1.16102E-05	0.007300004
628.79999	496.12784	1.11969E-05	0.00704062
628.83999	470.491019	1.06183E-05	0.006677229
628.87999	499.743063	1.12785E-05	0.007092826
628.91999	626.863	1.41474E-05	0.008897598
628.95999	852.874134	1.92482E-05	0.012106336
628.99999	1186.049485	2.67675E-05	0.016836748
629.03999	1725.758078	3.8948E-05	0.024499822
629.07999	2418.727552	5.45873E-05	0.034339786
629.11999	3115.275983	7.03074E-05	0.044231816
629.15999	3651.626268	8.24121E-05	0.051850408
629.19999	3805.772837	8.5891E-05	0.054042611
629.23999	3560.638269	8.03586E-05	0.050564874
629.27999	3113.191381	7.02604E-05	0.04421346
629.31999	2510.007481	5.66474E-05	0.035649324
629.35999	1843.451449	4.16041E-05	0.026183976
629.39999	1247.914451	2.81637E-05	0.017726227
629.43999	823.168449	1.85778E-05	0.011693588
629.47999	559.002969	1.26159E-05	0.007941468
629.51999	411.204042	9.2803E-06	0.005842136
629.55999	388.256944	8.76242E-06	0.005516468
629.59999	436.658981	9.85478E-06	0.006204573
629.63999	498.654057	1.12539E-05	0.007085923
629.67999	541.834285	1.22284E-05	0.007700007
629.71999	536.776696	1.21143E-05	0.007628618
629.75999	494.086629	1.11508E-05	0.007022357
629.79999	469.063784	1.05861E-05	0.006667136
629.83999	445.609289	1.00568E-05	0.006334163
629.87999	438.752721	9.90204E-06	0.006237095
629.91999	480.487131	1.08439E-05	0.006830805
629.95999	590.573108	1.33284E-05	0.008396367
629.99999	796.154764	1.79681E-05	0.011319905
630.03999	1081.472704	2.44073E-05	0.015377596
630.07999	1470.187886	3.31801E-05	0.020906113
630.11999	1971.241928	4.44882E-05	0.028032894
630.15999	2362.45133	5.33172E-05	0.033598389
630.19999	2548.998899	5.75274E-05	0.036253738
630.23999	2488.661664	5.61656E-05	0.035397824

630.27999	2213.496015	4.99555E-05	0.031485966
630.31999	1771.391805	3.99778E-05	0.025198835
630.35999	1258.85162	2.84105E-05	0.01790886
630.39999	871.994824	1.96797E-05	0.012406088
630.43999	623.821256	1.40788E-05	0.008875824
630.47999	457.471192	1.03245E-05	0.006509383
630.51999	392.381787	8.85551E-06	0.005583576
630.55999	378.604644	8.54458E-06	0.00538787
630.59999	389.030533	8.77988E-06	0.005536591
630.63999	434.944892	9.8161E-06	0.006190425
630.67999	477.69437	1.07809E-05	0.006799296
630.71999	465.98363	1.05166E-05	0.006633031
630.75999	426.590919	9.62756E-06	0.006072681
630.79999	386.230286	8.71668E-06	0.005498481
630.83999	382.285975	8.62766E-06	0.005442674
630.87999	425.645447	9.60622E-06	0.006060375
630.91999	499.98327	1.12839E-05	0.007119254
630.95999	652.850779	1.47339E-05	0.009296522
630.99999	969.991851	2.18914E-05	0.013813451
631.03999	1441.606314	3.2535E-05	0.020530915
631.07999	2024.461862	4.56893E-05	0.028833593
631.11999	2637.074266	5.95151E-05	0.037561165
631.15999	3091.774236	6.9777E-05	0.044040475
631.19999	3236.286365	7.30385E-05	0.046101885
631.23999	3070.020622	6.92861E-05	0.04373615
631.27999	2643.57094	5.96617E-05	0.037663246
631.31999	2049.5471	4.62554E-05	0.029201973
631.35999	1478.964377	3.33782E-05	0.021073639
631.39999	1023.55892	2.31003E-05	0.014585529
631.43999	680.603527	1.53603E-05	0.009699091
631.47999	464.519171	1.04836E-05	0.006620152
631.51999	366.856741	8.27945E-06	0.005228635
631.55999	335.73629	7.5771E-06	0.004785393
631.59999	359.99708	8.12463E-06	0.005131518
631.63999	417.252975	9.41682E-06	0.005948039
631.67999	449.622534	1.01474E-05	0.006409881
631.71999	419.583939	9.46943E-06	0.005982025
631.75999	355.08223	8.01371E-06	0.005062742
631.79999	285.545368	6.44436E-06	0.004071547
631.83999	257.313934	5.80722E-06	0.003669232
631.87999	261.599691	5.90394E-06	0.003730582
631.91999	301.865551	6.81269E-06	0.004305072
631.95999	392.827986	8.86558E-06	0.005602692
631.99999	574.90598	1.29748E-05	0.008200091
632.03999	915.353431	2.06583E-05	0.013056841
632.07999	1382.271124	3.11959E-05	0.019718326

632.11999	1859.68987	4.19706E-05	0.026530462
632.15999	2189.798861	4.94207E-05	0.031241796
632.19999	2321.523746	5.23936E-05	0.033123206
632.23999	2293.626183	5.17639E-05	0.032727238
632.27999	2120.347167	4.78533E-05	0.030256673
632.31999	1740.097546	3.92716E-05	0.024832204
632.35999	1297.884291	2.92914E-05	0.018522735
632.39999	905.261342	2.04305E-05	0.01292024
632.43999	598.956432	1.35176E-05	0.008549079
632.47999	404.493029	9.12884E-06	0.005773811
632.51999	325.029706	7.33547E-06	0.00463983
632.55999	320.422013	7.23148E-06	0.004574344
632.59999	373.246313	8.42365E-06	0.005328801
632.63999	437.408715	9.87171E-06	0.006245236
632.67999	452.677847	1.02163E-05	0.006463654
632.71999	445.059162	1.00444E-05	0.006355271
632.75999	418.468279	9.44425E-06	0.005975941
632.79999	379.304362	8.56037E-06	0.005417003
632.83999	332.481354	7.50364E-06	0.004748604
632.87999	366.069366	8.26168E-06	0.005228649
632.91999	483.034263	1.09014E-05	0.006899721
632.95999	713.650504	1.61061E-05	0.010194516
632.99999	1140.217324	2.57331E-05	0.016289063
633.03999	1771.948671	3.99904E-05	0.025315531
633.07999	2588.137367	5.84107E-05	0.036978617
633.11999	3336.178311	7.52929E-05	0.047669439
633.15999	3788.098144	8.54921E-05	0.054130176
633.19999	3808.601839	8.59548E-05	0.054426603
633.23999	3474.530772	7.84153E-05	0.049655716
633.27999	2881.738303	6.50368E-05	0.041186516
633.31999	2224.779272	5.02102E-05	0.031799104
633.35999	1624.523678	3.66632E-05	0.023221027
633.39999	1125.182847	2.53938E-05	0.016084439
633.43999	724.504888	1.63511E-05	0.010357419
633.47999	478.371239	1.07962E-05	0.006839159
633.51999	406.093183	9.16496E-06	0.005806184
633.55999	423.228408	9.55168E-06	0.00605156
633.59999	464.998564	1.04944E-05	0.006649233
633.63999	467.673568	1.05547E-05	0.006687906
633.67999	444.743759	1.00372E-05	0.006360403
633.71999	432.524739	9.76148E-06	0.006186046
633.75999	389.246075	8.78474E-06	0.005567418
633.79999	337.007545	7.60579E-06	0.00482055
633.83999	295.33799	6.66537E-06	0.004224776
633.87999	277.160709	6.25513E-06	0.003965002
633.91999	304.666395	6.8759E-06	0.004358768

633.95999	380.387326	8.58481E-06	0.005442427
633.99999	567.747379	1.28133E-05	0.008123611
634.03999	962.232203	2.17162E-05	0.013768965
634.07999	1481.98551	3.34463E-05	0.02120766
634.11999	1978.606037	4.46544E-05	0.028316236
634.15999	2372.088736	5.35347E-05	0.033949589
634.19999	2600.661024	5.86933E-05	0.037223288
634.23999	2547.388168	5.7491E-05	0.036463093
634.27999	2226.658867	5.02526E-05	0.031874212
634.31999	1821.497781	4.11087E-05	0.02607605
634.35999	1332.236697	3.00667E-05	0.019073128
634.39999	876.756842	1.97872E-05	0.012552987
634.43999	566.987607	1.27961E-05	0.008118371
634.47999	391.727407	8.84074E-06	0.005609274
634.51999	322.015196	7.26743E-06	0.004611332
634.55999	341.667434	7.71096E-06	0.004893065
634.59999	393.334406	8.87701E-06	0.00563335
634.63999	466.638018	1.05314E-05	0.006683629
634.67999	476.583622	1.07558E-05	0.006826509
634.71999	410.513807	9.26472E-06	0.005880506
634.75999	377.489401	8.51941E-06	0.005407781
634.79999	376.175975	8.48977E-06	0.005389305
634.83999	351.614021	7.93544E-06	0.005037734
634.87999	342.559424	7.73109E-06	0.004908314
634.91999	407.917274	9.20612E-06	0.005845153
634.95999	572.966165	1.2931E-05	0.008210698
634.99999	918.376221	2.07265E-05	0.01316131
635.03999	1543.689049	3.48389E-05	0.022124102
635.07999	2315.950246	5.22678E-05	0.033194216
635.11999	3036.052757	6.85195E-05	0.043518093
635.15999	3734.785469	8.42889E-05	0.053536941
635.19999	4131.335695	9.32385E-05	0.059225089
635.23999	4037.479466	9.11203E-05	0.057883251
635.27999	3611.389228	8.1504E-05	0.051777876
635.31999	2949.177704	6.65588E-05	0.042286157
635.35999	2210.489869	4.98877E-05	0.031696634
635.39999	1521.578275	3.43399E-05	0.021819574
635.43999	963.129607	2.17365E-05	0.013812237
635.47999	615.477588	1.38905E-05	0.008827117
635.51999	439.951897	9.9291E-06	0.006310142
635.55999	379.800135	8.57156E-06	0.005447741
635.59999	396.860028	8.95658E-06	0.005692801
635.63999	451.863235	1.01979E-05	0.006482208
635.67999	481.937458	1.08767E-05	0.006914073
635.71999	460.783068	1.03992E-05	0.006611
635.75999	410.146766	9.25644E-06	0.005884875

635.79999	377.504822	8.51976E-06	0.005416862
635.83999	331.395301	7.47913E-06	0.00475553
635.87999	296.369408	6.68864E-06	0.004253175
635.91999	305.950483	6.90488E-06	0.004390949
635.95999	384.544771	8.67864E-06	0.005519268
635.99999	561.459757	1.26714E-05	0.008058988
636.03999	854.931025	1.92946E-05	0.012272139
636.07999	1278.056703	2.8844E-05	0.018347066
636.11999	1767.482624	3.98896E-05	0.025374585
636.15999	2168.693231	4.89444E-05	0.03113646
636.19999	2405.019624	5.42779E-05	0.034531627
636.23999	2462.219079	5.55689E-05	0.035355128
636.27999	2301.338682	5.1938E-05	0.033047116
636.31999	1967.063069	4.43939E-05	0.028248709
636.35999	1543.254529	3.48291E-05	0.022163849
636.39999	1134.760285	2.561E-05	0.016298178
636.43999	806.79304	1.82082E-05	0.011588424
636.47999	560.344503	1.26462E-05	0.00804905
636.51999	408.300412	9.21477E-06	0.005865386
636.55999	385.528224	8.70084E-06	0.005538603
636.59999	460.269001	1.03876E-05	0.006612765
636.63999	537.395439	1.21283E-05	0.007721339
636.67999	531.670689	1.19991E-05	0.007639566
636.71999	468.673171	1.05773E-05	0.006734779
636.75999	412.910333	9.31881E-06	0.005933846
636.79999	391.380805	8.83292E-06	0.005624803
636.83999	373.894825	8.43829E-06	0.005373838
636.87999	391.819028	8.84281E-06	0.005631809
636.91999	450.07903	1.01577E-05	0.006469615
636.95999	594.652759	1.34205E-05	0.008548312
636.99999	922.086064	2.08102E-05	0.013256096
637.03999	1442.383029	3.25526E-05	0.020737293
637.07999	2098.58373	4.73621E-05	0.030173454
637.11999	2754.044722	6.2155E-05	0.039600163
637.15999	3273.890721	7.38872E-05	0.047077938
637.19999	3478.370104	7.8502E-05	0.050021453
637.23999	3346.404127	7.55237E-05	0.048126709
637.27999	2911.016356	6.56976E-05	0.041867756
637.31999	2278.703473	5.14272E-05	0.032775559
637.35999	1651.979792	3.72829E-05	0.023762618
637.39999	1120.686917	2.52923E-05	0.016121339
637.43999	768.083468	1.73346E-05	0.011049751
637.47999	535.175791	1.20782E-05	0.007699593
637.51999	387.104224	8.7364E-06	0.005569632
637.55999	337.756428	7.62269E-06	0.004859924
637.59999	389.087745	8.78117E-06	0.005598873

637.63999	444.075751	1.00222E-05	0.006390537
637.67999	462.538869	1.04389E-05	0.006656651
637.71999	423.491284	9.55761E-06	0.006095078
637.75999	369.780096	8.34542E-06	0.005322376
637.79999	313.00382	7.06406E-06	0.004505458
637.83999	290.42096	6.5544E-06	0.004180656
637.87999	300.985196	6.79282E-06	0.004333002
637.91999	315.345632	7.11691E-06	0.00454002
637.95999	373.256506	8.42388E-06	0.005374098
637.99999	525.627985	1.18627E-05	0.007568397
638.03999	792.192734	1.78787E-05	0.011407317
638.07999	1160.502754	2.61909E-05	0.016711909
638.11999	1556.936469	3.51379E-05	0.022422188
638.15999	1889.148131	4.26354E-05	0.027208233
638.19999	2014.03816	4.5454E-05	0.029008765
638.23999	1956.542887	4.41564E-05	0.028182411
638.27999	1749.87818	3.94923E-05	0.025207153
638.31999	1413.195176	3.18938E-05	0.02035848
638.35999	1067.912832	2.41013E-05	0.015385309
638.39999	819.703858	1.84996E-05	0.011810128
638.43999	606.897249	1.36968E-05	0.008744601
638.47999	436.253642	9.84564E-06	0.006286242
638.51999	361.11289	8.14981E-06	0.00520382
638.55999	386.822866	8.73005E-06	0.005574663
638.59999	478.446725	1.07979E-05	0.006895524
638.63999	538.848221	1.21611E-05	0.007766535
638.67999	581.65327	1.31271E-05	0.008384019
638.71999	596.638093	1.34653E-05	0.00860055
638.75999	536.090856	1.20988E-05	0.007728245
638.79999	470.205578	1.06119E-05	0.006778872
638.83999	482.196615	1.08825E-05	0.00695218
638.87999	703.345051	1.58735E-05	0.010141273
638.91999	1397.791243	3.15462E-05	0.020155499
638.95999	3123.346268	7.04896E-05	0.045040018
638.99999	6402.56102	0.000144497	0.092333508
639.03999	11108.94963	0.000250714	0.160215965
639.07999	15923.54263	0.000359372	0.229667568
639.11999	18989.54657	0.000428568	0.27390613
639.15999	18794.166	0.000424158	0.271104918
639.19999	15693.9447	0.00035419	0.226398546
639.23999	11409.72041	0.000257501	0.164605254
639.27999	7298.404147	0.000164715	0.105298886
639.31999	4253.288435	9.59908E-05	0.061368835
639.35999	2321.971738	5.24037E-05	0.033504809
639.39999	1257.18299	2.83729E-05	0.018141612
639.43999	734.168987	1.65692E-05	0.010594991

639.47999	519.517363	1.17248E-05	0.007497764
639.51999	404.837131	9.13661E-06	0.005843045
639.55999	330.502461	7.45898E-06	0.004770465
639.59999	351.240933	7.92702E-06	0.005070121
639.63999	405.733284	9.15684E-06	0.005857078
639.67999	467.876857	1.05593E-05	0.006754591
639.71999	467.967146	1.05614E-05	0.006756317
639.75999	451.050234	1.01796E-05	0.006512485
639.79999	411.446795	9.28578E-06	0.005941043
639.83999	356.639517	8.04886E-06	0.005149981
639.87999	353.183111	7.97085E-06	0.005100388
639.91999	488.126794	1.10163E-05	0.007049577
639.95999	960.573192	2.16788E-05	0.013873564
639.99999	1978.920082	4.46615E-05	0.02858334
640.03999	3566.436353	8.04895E-05	0.051516499
640.07999	5326.711206	0.000120216	0.076948146
640.11999	6553.241184	0.000147898	0.094672163
640.15999	6758.802258	0.000152537	0.097647926
640.19999	6006.27756	0.000135553	0.086781233
640.23999	4715.091845	0.000106413	0.068129894
640.27999	3356.478993	7.57511E-05	0.048501885
640.31999	2193.670119	4.95081E-05	0.031701013
640.35999	1381.927659	3.11882E-05	0.019971664
640.39999	877.55332	1.98052E-05	0.012683222
640.43999	593.682666	1.33986E-05	0.008580993
640.47999	438.052717	9.88624E-06	0.006331939
640.51999	377.914287	8.529E-06	0.005462994
640.55999	388.269307	8.7627E-06	0.005613033
640.59999	409.894684	9.25075E-06	0.005926032
640.63999	433.421559	9.78172E-06	0.006266562
640.67999	450.58313	1.0169E-05	0.006515096
640.71999	461.088586	1.04061E-05	0.006667414
640.75999	468.698289	1.05779E-05	0.006777874
640.79999	439.212859	9.91242E-06	0.00635188
640.83999	412.343173	9.30601E-06	0.005963664
640.87999	428.900549	9.67969E-06	0.006203518
640.91999	544.802166	1.22954E-05	0.007880384
640.95999	814.63128	1.83851E-05	0.011784109
640.99999	1390.7875	3.13881E-05	0.020119795
641.03999	2308.316702	5.20955E-05	0.033395294
641.07999	3458.585985	7.80555E-05	0.050039799
641.11999	4560.188381	0.000102917	0.06598221
641.15999	5077.45694	0.000114591	0.073471248
641.19999	4782.687206	0.000107939	0.069210222
641.23999	4064.46415	9.17293E-05	0.058820492
641.27999	3154.641396	7.11959E-05	0.045656482

641.31999	2222.350922	5.01554E-05	0.032165638
641.35999	1475.584938	3.33019E-05	0.021358504
641.39999	955.755589	2.15701E-05	0.013835044
641.43999	611.677162	1.38047E-05	0.008854888
641.47999	409.539086	9.24273E-06	0.005929024
641.51999	342.034368	7.71924E-06	0.004952046
641.55999	330.421399	7.45715E-06	0.00478421
641.59999	359.867677	8.12171E-06	0.00521089
641.63999	407.505476	9.19683E-06	0.005901055
641.67999	451.22572	1.01835E-05	0.006534571
641.71999	452.616907	1.02149E-05	0.006555127
641.75999	430.895081	9.7247E-06	0.006240925
641.79999	373.259621	8.42395E-06	0.005406491
641.83999	306.282724	6.91237E-06	0.004436638
641.87999	290.615946	6.5588E-06	0.004209961
641.91999	348.451003	7.86405E-06	0.005048093
641.95999	515.598515	1.16363E-05	0.007470065
641.99999	798.348437	1.80176E-05	0.011567307
642.03999	1212.273728	2.73593E-05	0.017565784
642.07999	1804.18198	4.07179E-05	0.026144132
642.11999	2402.405402	5.42189E-05	0.034815068
642.15999	2807.814229	6.33685E-05	0.040692687
642.19999	2933.556816	6.62063E-05	0.04251768
642.23999	2747.244492	6.20015E-05	0.039819831
642.27999	2322.579922	5.24174E-05	0.033666643
642.31999	1768.543788	3.99136E-05	0.025637284
642.35999	1257.810192	2.8387E-05	0.018234688
642.39999	856.93834	1.93399E-05	0.012423955
642.43999	591.708066	1.3354E-05	0.008579161
642.47999	448.238029	1.01161E-05	0.006499397
642.51999	400.107674	9.02987E-06	0.005801874
642.55999	416.385719	9.39725E-06	0.006038294
642.59999	475.087838	1.07221E-05	0.006890003
642.63999	509.291652	1.1494E-05	0.007386506
642.67999	518.446185	1.17006E-05	0.007519747
642.71999	505.708351	1.14131E-05	0.007335449
642.75999	484.958594	1.09448E-05	0.007034905
642.79999	464.030919	1.04725E-05	0.006731743
642.83999	412.313204	9.30533E-06	0.005981841
642.87999	399.944464	9.02619E-06	0.005802757
642.91999	491.808618	1.10994E-05	0.007136049
642.95999	682.402885	1.54009E-05	0.009902151
642.99999	1022.190043	2.30694E-05	0.014833627
643.03999	1609.632145	3.63272E-05	0.023359814
643.07999	2332.80518	5.26482E-05	0.033856981
643.11999	3100.036009	6.99635E-05	0.044994922

643.15999	3694.54438	8.33807E-05	0.053627143
643.19999	3938.617651	8.88891E-05	0.057173477
643.23999	3754.706793	8.47385E-05	0.054507193
643.27999	3229.419304	7.28835E-05	0.046884494
643.31999	2522.090678	5.69201E-05	0.036617821
643.35999	1767.353356	3.98867E-05	0.025661509
643.39999	1148.760376	2.59259E-05	0.016680738
643.43999	731.660883	1.65126E-05	0.010624846
643.47999	487.227977	1.09961E-05	0.007075743
643.51999	371.550587	8.38538E-06	0.005396159
643.55999	344.837881	7.78251E-06	0.005008512
643.59999	356.766848	8.05173E-06	0.005182094
643.63999	404.074075	9.11939E-06	0.005869603
643.67999	449.397248	1.01423E-05	0.006528376
643.71999	423.376406	9.55502E-06	0.006150755
643.75999	374.384601	8.44934E-06	0.005439346
643.79999	327.264603	7.38591E-06	0.004755046
643.83999	290.953351	6.56641E-06	0.004227719
643.87999	263.122869	5.93832E-06	0.003823563
643.91999	290.96268	6.56662E-06	0.004228379
643.95999	400.566317	9.04022E-06	0.005821542
643.99999	592.523051	1.33724E-05	0.008611838
644.03999	880.420476	1.98699E-05	0.012796987
644.07999	1292.638715	2.91731E-05	0.018789781
644.11999	1827.230488	4.1238E-05	0.02656225
644.15999	2224.275723	5.01988E-05	0.032336062
644.19999	2338.577605	5.27784E-05	0.03399987
644.23999	2296.090029	5.18196E-05	0.033384229
644.27999	2076.97279	4.68744E-05	0.030200227
644.31999	1683.289513	3.79895E-05	0.024477394
644.35999	1252.271428	2.8262E-05	0.018210916
644.39999	847.432512	1.91254E-05	0.012324389
644.43999	567.229986	1.28016E-05	0.008249857
644.47999	421.614418	9.51525E-06	0.006132388
644.51999	354.80248	8.0074E-06	0.005160928
644.55999	362.556176	8.18239E-06	0.00527404
644.59999	414.409168	9.35264E-06	0.00602871
644.63999	492.523759	1.11156E-05	0.007165544
644.67999	550.042894	1.24137E-05	0.008002865
644.71999	533.476183	1.20398E-05	0.007762309
644.75999	472.317274	1.06595E-05	0.006872847
644.79999	417.985599	9.43335E-06	0.006082626
644.83999	362.287719	8.17633E-06	0.005272424
644.87999	338.308309	7.63515E-06	0.004923754
644.91999	402.243227	9.07807E-06	0.005854628
644.95999	533.237946	1.20344E-05	0.007761731

644.99999	775.426278	1.75003E-05	0.011287688
645.03999	1214.814357	2.74167E-05	0.017684847
645.07999	1829.500726	4.12893E-05	0.02663489
645.11999	2496.219941	5.63362E-05	0.036343613
645.15999	2941.113937	6.63768E-05	0.042823685
645.19999	3028.675434	6.8353E-05	0.044101346
645.23999	2879.005312	6.49751E-05	0.041924559
645.27999	2526.924466	5.70292E-05	0.036799779
645.31999	2053.418874	4.63428E-05	0.029905938
645.35999	1539.56368	3.47458E-05	0.022423554
645.39999	1016.366876	2.2938E-05	0.014804175
645.43999	644.172669	1.45381E-05	0.009383458
645.47999	439.298684	9.91436E-06	0.006399521
645.51999	354.276324	7.99552E-06	0.00516127
645.55999	349.967115	7.89827E-06	0.005098807
645.59999	373.139396	8.42124E-06	0.00543675
645.63999	414.6507	9.35809E-06	0.006041956
645.67999	471.483001	1.06407E-05	0.006870497
645.71999	440.720545	9.94645E-06	0.006422621
645.75999	388.75595	8.77368E-06	0.005665692
645.79999	347.191833	7.83564E-06	0.005060254
645.83999	306.450166	6.91615E-06	0.004466728
645.87999	285.494366	6.44321E-06	0.004161541
645.91999	306.794045	6.92391E-06	0.004472295
645.95999	383.270609	8.64988E-06	0.005587479
645.99999	565.963003	1.2773E-05	0.008251356
646.03999	884.107583	1.99531E-05	0.012890485
646.07999	1227.99621	2.77142E-05	0.017905567
646.11999	1553.315218	3.50562E-05	0.022650486
646.15999	1872.742728	4.22652E-05	0.027310078
646.19999	2074.844328	4.68263E-05	0.030259185
646.23999	2022.238257	4.56391E-05	0.029493812
646.27999	1788.799399	4.03707E-05	0.026090783
646.31999	1484.634016	3.35061E-05	0.021655676
646.35999	1126.610272	2.5426E-05	0.016434365
646.39999	761.0127	1.7175E-05	0.011101918
646.43999	502.527991	1.13414E-05	0.007331507
646.47999	357.359745	8.06511E-06	0.005213933
646.51999	290.158841	6.54848E-06	0.004233724
646.55999	299.545379	6.76032E-06	0.004370954
646.59999	368.389615	8.31404E-06	0.005375858
646.63999	425.621543	9.60569E-06	0.00621142
646.67999	489.087184	1.1038E-05	0.007138064
646.71999	478.395474	1.07967E-05	0.006982454
646.75999	417.456769	9.42142E-06	0.006093396
646.79999	408.120815	9.21072E-06	0.005957493

646.83999	382.702394	8.63706E-06	0.005586796
646.87999	355.547729	8.02422E-06	0.005190705
646.91999	389.602332	8.79278E-06	0.005688226
646.95999	538.34767	1.21498E-05	0.007860407
646.99999	818.140266	1.84643E-05	0.011946393
647.03999	1311.875483	2.96072E-05	0.019157044
647.07999	2046.437209	4.61852E-05	0.029885543
647.11999	2816.047461	6.35543E-05	0.041127238
647.15999	3489.363106	7.87501E-05	0.050963891
647.19999	3860.604788	8.71285E-05	0.056389547
647.23999	3800.027079	8.57613E-05	0.055508155
647.27999	3323.824585	7.50141E-05	0.04855512
647.31999	2602.888958	5.87436E-05	0.038025892
647.35999	1854.508793	4.18537E-05	0.027094398
647.39999	1256.402863	2.83553E-05	0.018357197
647.43999	814.617813	1.83848E-05	0.011903048
647.47999	521.406223	1.17674E-05	0.007619164
647.51999	393.64403	8.884E-06	0.005752566
647.55999	368.389751	8.31404E-06	0.005383842
647.59999	389.991723	8.80157E-06	0.005699897
647.63999	460.189603	1.03858E-05	0.006726284
647.67999	487.084889	1.09928E-05	0.007119834
647.71999	460.786696	1.03993E-05	0.006735843
647.75999	407.522828	9.19722E-06	0.005957593
647.79999	324.224772	7.3173E-06	0.004740148
647.83999	274.032278	6.18453E-06	0.004006584
647.87999	303.321394	6.84554E-06	0.004435089
647.91999	350.446923	7.9091E-06	0.005124463
647.95999	417.106879	9.41352E-06	0.006099585
647.99999	578.850478	1.30638E-05	0.008465374
648.03999	828.949891	1.87082E-05	0.012123691
648.07999	1197.798489	2.70326E-05	0.017519315
648.11999	1655.820717	3.73696E-05	0.024219964
648.15999	2073.167845	4.67885E-05	0.030326441
648.19999	2264.004074	5.10954E-05	0.03312005
648.23999	2202.540757	4.97083E-05	0.032222893
648.27999	2009.998037	4.53629E-05	0.029407832
648.31999	1633.937069	3.68757E-05	0.023907243
648.35999	1206.787736	2.72355E-05	0.01765842
648.39999	832.506548	1.87885E-05	0.012182472
648.43999	584.796896	1.31981E-05	0.008558144
648.47999	436.74059	9.85663E-06	0.006391825
648.51999	403.256134	9.10093E-06	0.005902135
648.55999	418.773921	9.45114E-06	0.006129634
648.59999	463.502085	1.04606E-05	0.006784743
648.63999	530.561565	1.1974E-05	0.007766838

648.67999	568.286342	1.28254E-05	0.008319601
648.71999	564.521413	1.27405E-05	0.008264992
648.75999	560.05709	1.26397E-05	0.008200137
648.79999	521.466308	1.17688E-05	0.007635577
648.83999	532.361286	1.20147E-05	0.007795587
648.87999	579.138327	1.30703E-05	0.008481086
648.91999	736.19488	1.66149E-05	0.010781736
648.95999	1120.746822	2.52937E-05	0.016414596
648.99999	1742.866394	3.93341E-05	0.025527809
649.03999	2729.020207	6.15902E-05	0.039974493
649.07999	3896.532725	8.79393E-05	0.05707965
649.11999	4935.438538	0.000111386	0.072302862
649.15999	5565.91822	0.000125615	0.081544246
649.19999	5511.897043	0.000124396	0.080757777
649.23999	4835.871988	0.000109139	0.070857335
649.27999	3830.930018	8.64588E-05	0.056135939
649.31999	2769.086465	6.24944E-05	0.040578879
649.35999	1848.356757	4.17148E-05	0.027087946
649.39999	1177.995581	2.65857E-05	0.017264766
649.43999	748.757319	1.68984E-05	0.010974503
649.47999	514.803843	1.16184E-05	0.007545922
649.51999	411.637204	9.29008E-06	0.006034092
649.55999	362.415322	8.17921E-06	0.005312887
649.59999	369.075218	8.32951E-06	0.005410852
649.63999	403.709142	9.11115E-06	0.005918969
649.67999	410.230524	9.25833E-06	0.006014953
649.71999	397.450787	8.96991E-06	0.00582793
649.75999	372.264504	8.40149E-06	0.005458953
649.79999	331.289102	7.47673E-06	0.004858381
649.83999	301.894868	6.81335E-06	0.004427585
649.87999	337.487627	7.61663E-06	0.004949893
649.91999	425.995673	9.61413E-06	0.006248415
649.95999	579.484635	1.30782E-05	0.008500281
649.99999	907.296455	2.04764E-05	0.013309671
650.03999	1482.196873	3.34511E-05	0.021744565
650.07999	2231.422861	5.03601E-05	0.032738097
650.11999	2966.114341	6.69411E-05	0.043519727
650.15999	3386.647445	7.64319E-05	0.049692973
650.19999	3469.620336	7.83045E-05	0.050913584
650.23999	3228.287466	7.2858E-05	0.047375154
650.27999	2750.990145	6.2086E-05	0.040373294
650.31999	2146.27315	4.84384E-05	0.031500457
650.35999	1511.620532	3.41152E-05	0.022187142
650.39999	991.356537	2.23735E-05	0.014551748
650.43999	670.90476	1.51414E-05	0.009848563
650.47999	499.903768	1.12821E-05	0.007338801

650.51999	414.571685	9.35631E-06	0.006086464
650.55999	391.676807	8.8396E-06	0.00575069
650.59999	433.481721	9.78308E-06	0.006364871
650.63999	497.657171	1.12314E-05	0.007307617
650.67999	481.2512	1.08612E-05	0.007067146
650.71999	443.329438	1.00053E-05	0.006510667
650.75999	415.210729	9.37073E-06	0.006098095
650.79999	370.683652	8.36581E-06	0.005444471
650.83999	376.821617	8.50434E-06	0.005534964
650.87999	399.731197	9.02138E-06	0.005871833
650.91999	474.138502	1.07006E-05	0.006965264
650.95999	637.646803	1.43908E-05	0.009367835
650.99999	1026.848946	2.31746E-05	0.015086632
651.03999	1692.335975	3.81937E-05	0.024865604
651.07999	2480.497372	5.59814E-05	0.03644835
651.11999	3259.620082	7.35651E-05	0.047899697
651.15999	3734.389009	8.428E-05	0.054879737
651.19999	3864.703168	8.7221E-05	0.056798293
651.23999	3625.248482	8.18168E-05	0.053282378
651.27999	3126.924094	7.05703E-05	0.045961038
651.31999	2474.027054	5.58353E-05	0.036366676
651.35999	1748.278893	3.94562E-05	0.025700203
651.39999	1139.58433	2.57188E-05	0.016753246
651.43999	734.281131	1.65717E-05	0.01079547
651.47999	493.364353	1.11345E-05	0.007253934
651.51999	395.048457	8.91569E-06	0.005808752
651.55999	393.912392	8.89005E-06	0.005792404
651.59999	424.160627	9.57271E-06	0.006237581
651.63999	441.045595	9.95378E-06	0.006486284
651.67999	439.269631	9.9137E-06	0.006460562
651.71999	409.744321	9.24736E-06	0.006026688
651.75999	374.982301	8.46283E-06	0.005515733
651.79999	350.966085	7.92082E-06	0.005162788
651.83999	321.838542	7.26345E-06	0.004734606
651.87999	301.404155	6.80227E-06	0.004434265
651.91999	307.960918	6.95025E-06	0.004531006
651.95999	433.627384	9.78637E-06	0.006380319
651.99999	670.343109	1.51287E-05	0.009863919
652.03999	1021.596954	2.3056E-05	0.015033447
652.07999	1474.349553	3.3274E-05	0.02169732
652.11999	1864.961426	4.20896E-05	0.027447458
652.15999	2139.085993	4.82762E-05	0.0314838
652.19999	2215.309389	4.99964E-05	0.032607682
652.23999	2146.290553	4.84388E-05	0.031593715
652.27999	1934.622013	4.36617E-05	0.028479669
652.31999	1602.604695	3.61686E-05	0.023593472

652.35999	1210.152691	2.73115E-05	0.017816904
652.39999	904.73461	2.04186E-05	0.013321094
652.43999	717.304661	1.61886E-05	0.010562069
652.47999	597.604324	1.34871E-05	0.008800061
652.51999	517.799076	1.1686E-05	0.007625351
652.55999	471.783481	1.06475E-05	0.00694813
652.59999	493.61853	1.11403E-05	0.007270148
652.63999	542.557117	1.22448E-05	0.007991419
652.67999	551.863263	1.24548E-05	0.008128989
652.71999	542.534615	1.22443E-05	0.007992067
652.75999	535.060087	1.20756E-05	0.007882443
652.79999	510.302277	1.15168E-05	0.007518174
652.83999	437.890449	9.88258E-06	0.006451742
652.87999	414.341692	9.35111E-06	0.006105156
652.91999	471.93276	1.06509E-05	0.006954163
652.95999	685.601036	1.54731E-05	0.010103289
652.99999	1181.8369	2.66724E-05	0.017417085
653.03999	1970.865136	4.44797E-05	0.02904701
653.07999	2892.550043	6.52808E-05	0.042633601
653.11999	3733.531055	8.42606E-05	0.055032279
653.15999	4256.015479	9.60523E-05	0.062737546
653.19999	4362.09651	9.84464E-05	0.064305215
653.23999	4051.437487	9.14353E-05	0.059729195
653.27999	3379.76171	7.62765E-05	0.04982992
653.31999	2539.008974	5.73019E-05	0.037436474
653.35999	1735.56017	3.91692E-05	0.025591572
653.39999	1109.596284	2.5042E-05	0.01636247
653.43999	715.775271	1.61541E-05	0.010555703
653.47999	510.151573	1.15134E-05	0.007523783
653.51999	429.499257	9.6932E-06	0.0063347
653.55999	391.075461	8.82603E-06	0.005768339
653.59999	416.137185	9.39164E-06	0.006138374
653.63999	465.787727	1.05122E-05	0.006871181
653.67999	492.136607	1.11068E-05	0.007260318
653.71999	485.034843	1.09466E-05	0.007155986
653.75999	446.435861	1.00754E-05	0.006586917
653.79999	409.882112	9.25047E-06	0.006047956
653.83999	362.909559	8.19036E-06	0.005355187
653.87999	339.187096	7.65498E-06	0.005005438
653.91999	339.681919	7.66615E-06	0.005013047
653.95999	377.332138	8.51586E-06	0.005569032
653.99999	548.432727	1.23774E-05	0.008094795
654.03999	920.625858	2.07772E-05	0.013589148
654.07999	1412.794994	3.18848E-05	0.02085522
654.11999	1880.691428	4.24446E-05	0.027763852
654.15999	2240.734752	5.05703E-05	0.033081042

654.19999	2386.832573	5.38675E-05	0.035240109
654.23999	2277.924809	5.14096E-05	0.03363421
654.27999	2001.586698	4.5173E-05	0.029555806
654.31999	1615.567401	3.64611E-05	0.02385723
654.35999	1187.842787	2.6808E-05	0.017542055
654.39999	816.267628	1.8422E-05	0.012055372
654.43999	534.241327	1.20571E-05	0.007890637
654.47999	395.290361	8.92115E-06	0.005838716
654.51999	350.157145	7.90256E-06	0.005172383
654.55999	371.761791	8.39015E-06	0.005491854
654.59999	437.656744	9.8773E-06	0.006465683
654.63999	492.837488	1.11227E-05	0.007281335
654.67999	525.971987	1.18705E-05	0.007771349
654.71999	549.833362	1.2409E-05	0.008124403
654.75999	545.128228	1.23028E-05	0.008055371
654.79999	514.208247	1.1605E-05	0.00759893
654.83999	498.81249	1.12575E-05	0.007371863
654.87999	509.79109	1.15053E-05	0.007534574
654.91999	598.667414	1.35111E-05	0.008848683
654.95999	863.988509	1.9499E-05	0.012771076
654.99999	1381.508987	3.11787E-05	0.02042207
655.03999	2147.878062	4.84746E-05	0.031752812
655.07999	3020.189647	6.81615E-05	0.044651217
655.11999	3733.395504	8.42575E-05	0.055198796
655.15999	4056.704242	9.15542E-05	0.059982625
655.19999	3926.24365	8.86099E-05	0.058057173
655.23999	3486.289867	7.86807E-05	0.051554745
655.27999	2845.454552	6.42179E-05	0.042080735
655.31999	2202.927922	4.9717E-05	0.032580554
655.35999	1582.36841	3.57119E-05	0.023404118
655.39999	1095.144652	2.47159E-05	0.016198794
655.43999	756.476549	1.70726E-05	0.011190079
655.47999	500.092215	1.12864E-05	0.007397999
655.51999	384.778045	8.6839E-06	0.005692473
655.55999	367.651981	8.29739E-06	0.005439439
655.59999	384.429494	8.67604E-06	0.005688011
655.63999	419.371704	9.46464E-06	0.006205393
655.67999	440.152	9.93362E-06	0.006513274
655.71999	424.873792	9.58881E-06	0.006287574
655.75999	386.130875	8.71444E-06	0.005714578
655.79999	371.213109	8.37776E-06	0.005494137
655.83999	350.657974	7.91386E-06	0.005190227
655.87999	316.030465	7.13237E-06	0.004677977
655.91999	339.854289	7.67004E-06	0.005030931
655.95999	448.548587	1.01231E-05	0.00664036
655.99999	660.481863	1.49062E-05	0.009778438

656.03999	1037.045357	2.34047E-05	0.015354399
656.07999	1511.155924	3.41047E-05	0.022375401
656.11999	1985.396244	4.48076E-05	0.02939918
656.15999	2340.115247	5.28131E-05	0.03465387
656.19999	2478.34704	5.59328E-05	0.036703129
656.23999	2317.919026	5.23122E-05	0.03432936
656.27999	1888.410295	4.26188E-05	0.027969859
656.31999	1417.144275	3.1983E-05	0.020991064
656.35999	1061.827858	2.3964E-05	0.015728995
656.39999	794.48399	1.79304E-05	0.011769512
656.43999	593.051329	1.33843E-05	0.008786018
656.47999	456.757348	1.03084E-05	0.006767243
656.51999	371.752489	8.38994E-06	0.005508161
656.55999	327.53411	7.39199E-06	0.004853284
656.59999	369.220394	8.33279E-06	0.00547131
656.63999	443.23813	1.00033E-05	0.006568545
656.67999	495.216723	1.11764E-05	0.007339286
656.71999	527.471095	1.19043E-05	0.007817784
656.75999	509.506399	1.14988E-05	0.007551984
656.79999	437.479981	9.87331E-06	0.006484792
656.83999	383.874451	8.66351E-06	0.005690541
656.87999	424.218216	9.57401E-06	0.006288978
656.91999	475.572108	1.0733E-05	0.007050722
656.95999	639.324696	1.44287E-05	0.009479058
656.99999	934.328156	2.10865E-05	0.01385382
657.03999	1451.060395	3.27484E-05	0.021517016
657.07999	2145.612327	4.84235E-05	0.031818101
657.11999	2807.662013	6.3365E-05	0.041638422
657.15999	3233.32245	7.29716E-05	0.047954005
657.19999	3338.151536	7.53374E-05	0.049511759
657.23999	3096.783712	6.98901E-05	0.045934565
657.27999	2612.378407	5.89577E-05	0.038751744
657.31999	2100.761318	4.74113E-05	0.031164367
657.35999	1628.183957	3.67458E-05	0.024155248
657.39999	1146.592417	2.5877E-05	0.017011535
657.43999	754.281941	1.70231E-05	0.011191662
657.47999	512.344707	1.15629E-05	0.00760238
657.51999	385.823258	8.70749E-06	0.005725351
657.55999	349.487408	7.88744E-06	0.005186468
657.59999	361.380386	8.15585E-06	0.005363288
657.63999	392.658191	8.86175E-06	0.00582784
657.67999	428.338511	9.667E-06	0.006357795
657.71999	444.731396	1.0037E-05	0.006601515
657.75999	423.821865	9.56507E-06	0.00629152
657.79999	372.615165	8.40941E-06	0.005531707
657.83999	309.46018	6.98409E-06	0.004594411

657.87999	272.468177	6.14923E-06	0.004045454
657.91999	302.020422	6.81618E-06	0.004484501
657.95999	372.04592	8.39656E-06	0.005524599
657.99999	547.665409	1.236E-05	0.00813291
658.03999	802.372156	1.81084E-05	0.011916066
658.07999	1166.871452	2.63347E-05	0.017330315
658.11999	1580.699609	3.56742E-05	0.023477897
658.15999	1887.455159	4.25972E-05	0.028035795
658.19999	2003.928347	4.52259E-05	0.029767668
658.23999	1923.330529	4.34069E-05	0.028572152
658.27999	1694.027296	3.82318E-05	0.025167253
658.31999	1409.342147	3.18069E-05	0.020939111
658.35999	1076.74252	2.43006E-05	0.015998529
658.39999	752.183208	1.69757E-05	0.011176819
658.43999	542.957042	1.22538E-05	0.008068382
658.47999	431.590834	9.7404E-06	0.006413861
658.51999	353.316825	7.97387E-06	0.005250952
658.55999	325.803362	7.35293E-06	0.004842344
658.59999	363.071055	8.19401E-06	0.005396574
658.63999	437.167085	9.86625E-06	0.006498308
658.67999	473.815403	1.06934E-05	0.007043498
658.71999	451.345745	1.01862E-05	0.006709883
658.75999	409.302546	9.23739E-06	0.006085222
658.79999	382.08646	8.62316E-06	0.005680937
658.83999	352.208831	7.94886E-06	0.005237029
658.87999	361.11371	8.14983E-06	0.005369762
658.91999	414.472714	9.35407E-06	0.006163585
658.95999	538.215299	1.21468E-05	0.008004235
658.99999	786.702402	1.77548E-05	0.011700398
659.03999	1208.690431	2.72785E-05	0.017977596
659.07999	1782.638811	4.02317E-05	0.026515892
659.11999	2380.621664	5.37273E-05	0.035412747
659.15999	2879.220617	6.498E-05	0.042832215
659.19999	3124.570952	7.05172E-05	0.046484947
659.23999	3113.024062	7.02566E-05	0.046315971
659.27999	2766.411877	6.24341E-05	0.041161528
659.31999	2262.34361	5.10579E-05	0.033663523
659.35999	1740.292838	3.9276E-05	0.025897013
659.39999	1250.263246	2.82167E-05	0.018606092
659.43999	854.941078	1.92948E-05	0.012723782
659.47999	569.625681	1.28557E-05	0.008478049
659.51999	401.951009	9.07147E-06	0.005982819
659.55999	342.702689	7.73432E-06	0.00510125
659.59999	371.329564	8.38039E-06	0.005527706
659.63999	383.504667	8.65517E-06	0.005709294
659.67999	398.90352	9.0027E-06	0.005938899

659.71999	402.331519	9.08006E-06	0.005990298
659.75999	374.405696	8.44982E-06	0.00557485
659.79999	360.054151	8.12592E-06	0.005361482
659.83999	328.704198	7.4184E-06	0.004894954
659.87999	282.978635	6.38643E-06	0.00421428
659.91999	267.412153	6.03512E-06	0.003982696
659.95999	329.996237	7.44756E-06	0.004915089
659.99999	485.711813	1.09618E-05	0.007234814
660.03999	736.899157	1.66308E-05	0.010976985
660.07999	1081.970451	2.44186E-05	0.016118207
660.11999	1479.573334	3.33919E-05	0.022042667
660.15999	1776.290085	4.00884E-05	0.026464753
660.19999	1970.566587	4.44729E-05	0.029361037
660.23999	1978.82636	4.46594E-05	0.029485892
660.27999	1796.850931	4.05524E-05	0.026775953
660.31999	1491.557255	3.36624E-05	0.022227936
660.35999	1147.49213	2.58973E-05	0.01710154
660.39999	823.430755	1.85837E-05	0.012272666
660.43999	575.122239	1.29797E-05	0.008572318
660.47999	400.901863	9.0478E-06	0.005975889
660.51999	347.760359	7.84847E-06	0.005184069
660.55999	392.014937	8.84723E-06	0.005844127
660.59999	451.477996	1.01892E-05	0.006731005
660.63999	467.921426	1.05603E-05	0.006976579
660.67999	455.31117	1.02757E-05	0.006788975
660.71999	445.384076	1.00517E-05	0.006641358
660.75999	422.932164	9.54499E-06	0.006306947
660.79999	380.398376	8.58506E-06	0.005673009
660.83999	369.934351	8.3489E-06	0.005517289
660.87999	374.992826	8.46307E-06	0.005593071
660.91999	443.10772	1.00003E-05	0.006609414
660.95999	599.630601	1.35328E-05	0.008944657
660.99999	870.138442	1.96378E-05	0.012980594
661.03999	1283.862165	2.8975E-05	0.019153621
661.07999	1843.313448	4.1601E-05	0.0275016
661.11999	2406.873769	5.43198E-05	0.035911898
661.15999	2797.579891	6.31375E-05	0.041743977
661.19999	2941.111141	6.63768E-05	0.043888327
661.23999	2740.241332	6.18434E-05	0.040893349
661.27999	2348.047394	5.29922E-05	0.035042654
661.31999	1953.838052	4.40954E-05	0.029161172
661.35999	1557.378899	3.51479E-05	0.023245397
661.39999	1138.050491	2.56842E-05	0.016987539
661.43999	754.359796	1.70248E-05	0.011260917
661.47999	523.372707	1.18118E-05	0.007813265
661.51999	439.410256	9.91688E-06	0.006560213

661.55999	417.637022	9.42549E-06	0.006235524
661.59999	439.669043	9.92272E-06	0.00656487
661.63999	461.71824	1.04203E-05	0.006894512
661.67999	460.89642	1.04018E-05	0.006882656
661.71999	431.439982	9.737E-06	0.006443167
661.75999	410.264385	9.2591E-06	0.006127299
661.79999	364.653311	8.22972E-06	0.005446427
661.83999	304.52269	6.87265E-06	0.004548597
661.87999	311.340871	7.02653E-06	0.004650719
661.91999	380.968104	8.59792E-06	0.005691135
661.95999	440.230697	9.93539E-06	0.006576833
661.99999	538.385813	1.21506E-05	0.008043709
662.03999	735.12692	1.65908E-05	0.010983767
662.07999	1056.569789	2.38453E-05	0.015787502
662.11999	1411.747362	3.18612E-05	0.021095919
662.15999	1708.662056	3.85621E-05	0.025534295
662.19999	1899.074055	4.28595E-05	0.028381531
662.23999	1892.941751	4.27211E-05	0.028291593
662.27999	1698.943048	3.83428E-05	0.025393655
662.31999	1371.954815	3.09631E-05	0.020507486
662.35999	1028.432342	2.32103E-05	0.015373564
662.39999	734.809072	1.65836E-05	0.010984988
662.43999	478.492626	1.07989E-05	0.007153631
662.47999	337.168128	7.60942E-06	0.005041085
662.51999	313.553225	7.07646E-06	0.004688296
662.55999	327.814875	7.39833E-06	0.004901834
662.59999	374.177957	8.44468E-06	0.005595442
662.63999	421.958294	9.52301E-06	0.006310328
662.67999	449.582084	1.01464E-05	0.006723844
662.71999	456.636041	1.03056E-05	0.006829753
662.75999	432.744443	9.76644E-06	0.006472805
662.79999	419.817673	9.4747E-06	0.006279831
662.83999	395.519269	8.92632E-06	0.005916721
662.87999	363.245923	8.19795E-06	0.00543426
662.91999	395.505366	8.92601E-06	0.005917227
662.95999	586.901287	1.32455E-05	0.008781266
662.99999	944.443659	2.13148E-05	0.014131698
663.03999	1549.461071	3.49692E-05	0.023185964
663.07999	2368.220543	5.34474E-05	0.035439927
663.11999	3232.689056	7.29573E-05	0.048379437
663.15999	3877.133018	8.75015E-05	0.058027488
663.19999	4119.212321	9.29649E-05	0.06165431
663.23999	3827.726638	8.63865E-05	0.057294954
663.27999	3164.856616	7.14264E-05	0.047375706
663.31999	2413.22938	5.44632E-05	0.036126546
663.35999	1750.939505	3.95163E-05	0.02621351

663.39999	1279.856921	2.88846E-05	0.019162035
663.43999	975.304863	2.20113E-05	0.014603158
663.47999	740.581886	1.67139E-05	0.011089339
663.51999	570.557892	1.28767E-05	0.008543946
663.55999	481.954952	1.08771E-05	0.007217576
663.59999	448.404306	1.01199E-05	0.006715539
663.63999	434.05275	9.79597E-06	0.006500995
663.67999	459.810034	1.03773E-05	0.006887188
663.71999	482.587995	1.08913E-05	0.007228799
663.75999	437.210816	9.86724E-06	0.006549479
663.79999	362.336942	8.17744E-06	0.005428185
663.83999	315.490968	7.12019E-06	0.004726668
663.87999	292.390232	6.59884E-06	0.004380838
663.91999	312.230888	7.04662E-06	0.004678389
663.95999	370.690034	8.36596E-06	0.005554661
663.99999	544.819237	1.22958E-05	0.008164419
664.03999	817.658579	1.84534E-05	0.012253806
664.07999	1228.479037	2.77251E-05	0.018411658
664.11999	1740.892672	3.92895E-05	0.026092957
664.15999	2106.257362	4.75353E-05	0.031571041
664.19999	2150.903621	4.85429E-05	0.032242193
664.23999	2054.041363	4.63569E-05	0.030792075
664.27999	1811.019937	4.08722E-05	0.027150583
664.31999	1463.373523	3.30263E-05	0.021940033
664.35999	1141.469625	2.57614E-05	0.01711483
664.39999	870.767714	1.9652E-05	0.013056798
664.43999	676.000743	1.52564E-05	0.010136959
664.47999	540.285707	1.21935E-05	0.008102334
664.51999	442.439595	9.98525E-06	0.006635395
664.55999	422.424732	9.53354E-06	0.006335608
664.59999	432.904554	9.77005E-06	0.006493177
664.63999	429.626269	9.69607E-06	0.006444394
664.67999	444.243486	1.0026E-05	0.006664053
664.71999	457.20562	1.03185E-05	0.006858909
664.75999	448.939179	1.01319E-05	0.006735303
664.79999	400.449679	9.03759E-06	0.006008191
664.83999	345.956486	7.80776E-06	0.005190908
664.87999	342.574846	7.73144E-06	0.005140478
664.91999	404.279517	9.12403E-06	0.006066747
664.95999	550.470774	1.24234E-05	0.008261036
664.99999	880.828264	1.98791E-05	0.013219579
665.03999	1411.394787	3.18532E-05	0.021183661
665.07999	2067.017755	4.66497E-05	0.03102579
665.11999	2757.697714	6.22374E-05	0.041395336
665.15999	3202.502541	7.2276E-05	0.048075118
665.19999	3273.896561	7.38873E-05	0.049149821

665.23999	2949.296552	6.65615E-05	0.044279382
665.27999	2465.835143	5.56505E-05	0.037023141
665.31999	1947.097905	4.39433E-05	0.029236348
665.35999	1395.263666	3.14892E-05	0.020951626
665.39999	964.835571	2.1775E-05	0.014489081
665.43999	686.718462	1.54983E-05	0.010313175
665.47999	492.410071	1.1113E-05	0.007395485
665.51999	383.342682	8.65151E-06	0.005757753
665.55999	360.091758	8.12677E-06	0.005408853
665.59999	371.433109	8.38273E-06	0.005579544
665.63999	421.625791	9.51551E-06	0.006333902
665.67999	432.46661	9.76017E-06	0.006497149
665.71999	397.923515	8.98058E-06	0.005978551
665.75999	378.682598	8.54634E-06	0.00568981
665.79999	328.505378	7.41391E-06	0.00493618
665.83999	273.492755	6.17235E-06	0.004109798
665.87999	266.373258	6.01167E-06	0.004003053
665.91999	297.751748	6.71984E-06	0.004474877
665.95999	375.241865	8.46869E-06	0.005639806
665.99999	548.905335	1.2388E-05	0.008250427
666.03999	835.792167	1.88627E-05	0.012563289
666.07999	1161.022619	2.62027E-05	0.01745307
666.11999	1521.058711	3.43282E-05	0.022866685
666.15999	1904.002545	4.29707E-05	0.028625351
666.19999	2074.906136	4.68277E-05	0.031196641
666.23999	1986.15388	4.48247E-05	0.029864025
666.27999	1754.454131	3.95956E-05	0.026381747
666.31999	1414.324396	3.19193E-05	0.021268489
666.35999	1096.752558	2.47522E-05	0.016493861
666.39999	849.183276	1.91649E-05	0.012771478
666.43999	622.273282	1.40438E-05	0.009359377
666.47999	457.108504	1.03163E-05	0.006875609
666.51999	391.583327	8.83749E-06	0.005890364
666.55999	403.752113	9.11212E-06	0.006073776
666.59999	470.709569	1.06233E-05	0.007081465
666.63999	542.262212	1.22381E-05	0.008158409
666.67999	601.035465	1.35645E-05	0.009043203
666.71999	620.276271	1.39988E-05	0.009333261
666.75999	619.139828	1.39731E-05	0.00931672
666.79999	604.228887	1.36366E-05	0.009092888
666.83999	609.178642	1.37483E-05	0.009167925
666.87999	656.194852	1.48094E-05	0.009876095
666.91999	764.616222	1.72563E-05	0.011508587
666.95999	985.188448	2.22343E-05	0.014829409
666.99999	1391.545786	3.14053E-05	0.020947302
667.03999	2031.700521	4.58527E-05	0.030585552

667.07999	2879.393129	6.49839E-05	0.043349454
667.11999	3682.244893	8.31031E-05	0.055439765
667.15999	4231.399135	9.54968E-05	0.063711634
667.19999	4319.708303	9.74898E-05	0.065045193
667.23999	3879.40056	8.75527E-05	0.058418641
667.27999	3132.604572	7.06985E-05	0.047175709
667.31999	2315.3065	5.22532E-05	0.034869633
667.35999	1597.014117	3.60424E-05	0.024053245
667.39999	1042.716856	2.35327E-05	0.015705702
667.43999	676.974215	1.52784E-05	0.010197392
667.47999	468.644714	1.05767E-05	0.007059708
667.51999	381.218295	8.60357E-06	0.005743052
667.55999	360.40162	8.13376E-06	0.005429774
667.59999	403.588599	9.10843E-06	0.006080789
667.63999	471.03693	1.06306E-05	0.007097445
667.67999	503.122514	1.13548E-05	0.007581356
667.71999	485.082975	1.09476E-05	0.007309963
667.75999	418.693047	9.44932E-06	0.006309877
667.79999	373.022572	8.4186E-06	0.005621941
667.83999	358.948561	8.10097E-06	0.005410151
667.87999	380.008985	8.57627E-06	0.005727921
667.91999	424.961918	9.5908E-06	0.006405886
667.95999	490.555631	1.10712E-05	0.00739509
667.99999	668.417802	1.50853E-05	0.010076953
668.03999	981.96908	2.21617E-05	0.014804885
668.07999	1371.027948	3.09422E-05	0.020671859
668.11999	1785.883629	4.03049E-05	0.026928513
668.15999	2085.145705	4.70588E-05	0.03144283
668.19999	2217.987469	5.00569E-05	0.033448012
668.23999	2103.767789	4.74791E-05	0.031727439
668.27999	1821.936614	4.11186E-05	0.027478719
668.31999	1452.687047	3.27851E-05	0.021910953
668.35999	1056.938493	2.38536E-05	0.015942812
668.39999	729.314077	1.64596E-05	0.011001598
668.43999	553.014865	1.24808E-05	0.008342649
668.47999	444.540118	1.00327E-05	0.006706627
668.51999	388.653215	8.77136E-06	0.005863831
668.55999	372.750635	8.41246E-06	0.005624236
668.59999	385.7351	8.7055E-06	0.0058205
668.63999	415.635566	9.38032E-06	0.006272054
668.67999	436.384242	9.84858E-06	0.006585551
668.71999	463.322559	1.04565E-05	0.006992501
668.75999	476.923597	1.07635E-05	0.007198199
668.79999	471.151803	1.06332E-05	0.007111511
668.83999	458.616688	1.03503E-05	0.006922721
668.87999	461.441493	1.04141E-05	0.006965778

668.91999	507.597879	1.14558E-05	0.007662998
668.95999	639.568234	1.44342E-05	0.009655878
668.99999	887.892574	2.00385E-05	0.013405755
669.03999	1297.674395	2.92867E-05	0.019593976
669.07999	1853.959305	4.18413E-05	0.027995163
669.11999	2488.974956	5.61727E-05	0.037586275
669.15999	3022.808183	6.82206E-05	0.045650476
669.19999	3168.782508	7.1515E-05	0.047857842
669.23999	2925.08992	6.60152E-05	0.044180015
669.27999	2490.105857	5.61982E-05	0.037612345
669.31999	1964.783803	4.43424E-05	0.029679277
669.35999	1431.315526	3.23028E-05	0.0216222
669.39999	979.41017	2.21039E-05	0.014796366
669.43999	665.727324	1.50245E-05	0.010058027
669.47999	440.238011	9.93556E-06	0.006651658
669.51999	318.283765	7.18322E-06	0.00480931
669.55999	318.576271	7.18982E-06	0.004814018
669.59999	429.798201	9.69995E-06	0.006495084
669.63999	556.922386	1.2569E-05	0.00841668
669.67999	606.013418	1.36769E-05	0.009159132
669.71999	555.271633	1.25317E-05	0.008392735
669.75999	467.672325	1.05547E-05	0.007069124
669.799989	415.633811	9.38028E-06	0.006282909
669.839989	352.958544	7.96578E-06	0.0053358
669.879989	326.576088	7.37037E-06	0.004937262
669.919989	355.389916	8.02066E-06	0.005373197
669.959989	419.806076	9.47444E-06	0.006347495
669.999989	543.152852	1.22582E-05	0.008212996
670.039989	776.115908	1.75159E-05	0.011736323
670.079989	1099.250473	2.48086E-05	0.016623714
670.119989	1501.382446	3.38841E-05	0.02270642
670.159989	1882.578701	4.24872E-05	0.028473207
670.199989	2056.268607	4.64071E-05	0.03110205
670.239989	2057.859375	4.6443E-05	0.031127969
670.279989	1879.970782	4.24283E-05	0.028438855
670.319989	1523.527908	3.43839E-05	0.023048217
670.359989	1149.400598	2.59404E-05	0.017389386
670.399989	811.879048	1.8323E-05	0.012283725
670.439989	572.156641	1.29128E-05	0.008657243
670.479989	435.258603	9.82318E-06	0.006586246
670.519989	357.048394	8.05808E-06	0.005403107
670.559989	372.00515	8.39564E-06	0.005629779
670.599989	436.265036	9.84589E-06	0.006602657
670.639989	496.117798	1.11967E-05	0.007508946
670.679989	515.099078	1.16251E-05	0.007796701
670.719989	509.259715	1.14933E-05	0.007708774

670.759989	474.017759	1.06979E-05	0.007175737
670.799989	443.428112	1.00076E-05	0.006713068
670.839989	413.961233	9.34253E-06	0.006267342
670.879989	409.730981	9.24706E-06	0.006203666
670.919989	452.396703	1.021E-05	0.006850069
670.959989	606.727029	1.3693E-05	0.009187444
670.999989	893.506543	2.01652E-05	0.013530848
671.039989	1242.429193	2.80399E-05	0.018815892
671.079989	1692.4876	3.81971E-05	0.025633301
671.119989	2209.484523	4.9865E-05	0.03346539
671.159989	2681.688647	6.0522E-05	0.04061993
671.199989	2992.574832	6.75382E-05	0.04533167
671.239989	2945.358388	6.64726E-05	0.044619092
671.279989	2534.947736	5.72102E-05	0.038404089
671.319989	1935.105761	4.36726E-05	0.029318317
671.359989	1409.151641	3.18026E-05	0.021350986
671.399989	990.053499	2.23441E-05	0.015001848
671.439989	682.109415	1.53943E-05	0.010336321
671.479989	491.864675	1.11007E-05	0.007453898
671.519989	405.685695	9.15576E-06	0.006148277
671.559989	386.38391	8.72015E-06	0.005856102
671.599989	401.093132	9.05211E-06	0.006079399
671.639989	448.124469	1.01135E-05	0.006792661
671.679989	444.742113	1.00372E-05	0.006741793
671.719989	405.776715	9.15782E-06	0.006151488
671.759989	372.772126	8.41295E-06	0.005651482
671.799989	351.357042	7.92964E-06	0.005327131
671.839989	328.733024	7.41905E-06	0.004984412
671.879989	318.907031	7.19729E-06	0.004835713
671.919989	340.252742	7.67903E-06	0.005159694
671.959989	392.005165	8.84701E-06	0.005944837
671.999989	525.071698	1.18501E-05	0.007963292
672.039989	741.899391	1.67436E-05	0.011252393
672.079989	1072.865594	2.42131E-05	0.016273128
672.119989	1497.405204	3.37943E-05	0.022713858
672.159989	1837.241387	4.1464E-05	0.027870428
672.199989	1971.814391	4.45011E-05	0.029913642
672.239989	1867.120564	4.21383E-05	0.028327057
672.279989	1662.417239	3.75184E-05	0.025222897
672.319989	1430.644252	3.22876E-05	0.021707631
672.359989	1118.876917	2.52515E-05	0.016978094
672.399989	831.436555	1.87644E-05	0.012617159
672.439989	607.291124	1.37057E-05	0.009216271
672.479989	433.666115	9.78724E-06	0.006581723
672.519989	378.420896	8.54043E-06	0.005743612
672.559989	382.550568	8.63363E-06	0.005806636

672.599989	384.797375	8.68434E-06	0.005841088
672.639989	421.617639	9.51532E-06	0.006400387
672.679989	482.023918	1.08786E-05	0.007317822
672.719989	519.440058	1.1723E-05	0.007886322
672.759989	486.625473	1.09825E-05	0.007388559
672.799989	445.252557	1.00487E-05	0.006760785
672.839989	430.956111	9.72608E-06	0.006544095
672.879989	397.101174	8.96202E-06	0.006030364
672.919989	404.006094	9.11785E-06	0.006135587
672.959989	550.317436	1.24199E-05	0.008358094
672.999989	832.324567	1.87844E-05	0.012641905
673.039989	1235.595611	2.78857E-05	0.018768172
673.079989	1750.544419	3.95073E-05	0.026591606
673.119989	2274.701954	5.13369E-05	0.034555863
673.159989	2700.354093	6.09432E-05	0.041024544
673.199989	2950.386512	6.65861E-05	0.044825771
673.239989	2905.911702	6.55824E-05	0.04415268
673.279989	2623.889655	5.92175E-05	0.039869981
673.319989	2254.097163	5.08718E-05	0.034253022
673.359989	1809.299728	4.08334E-05	0.027495561
673.399989	1316.975781	2.97223E-05	0.020015003
673.439989	894.970961	2.01982E-05	0.013602308
673.479989	624.636133	1.40972E-05	0.00949416
673.519989	464.084584	1.04737E-05	0.007054275
673.559989	390.356942	8.80981E-06	0.005933937
673.599989	419.357482	9.46431E-06	0.006375162
673.639989	496.037644	1.11949E-05	0.007541318
673.679989	501.668143	1.1322E-05	0.007627372
673.719989	452.241594	1.02065E-05	0.006876298
673.759989	394.985589	8.91427E-06	0.006006082
673.799989	343.632753	7.75531E-06	0.00522553
673.839989	312.800609	7.05947E-06	0.004756956
673.879989	285.717059	6.44824E-06	0.004345337
673.919989	297.479252	6.71369E-06	0.004524491
673.959989	378.140688	8.53411E-06	0.005751648
673.999989	510.111034	1.15125E-05	0.007759422
674.039989	720.639544	1.62638E-05	0.010962472
674.079989	1042.186504	2.35207E-05	0.015854832
674.119989	1404.965842	3.17081E-05	0.021375079
674.159989	1727.939551	3.89972E-05	0.026290344
674.199989	1858.514114	4.19441E-05	0.028278695
674.239989	1854.186911	4.18464E-05	0.028214527
674.279989	1724.611555	3.89221E-05	0.02624438
674.319989	1441.379389	3.25299E-05	0.021935579
674.359989	1102.263172	2.48765E-05	0.016775746
674.399989	792.305232	1.78812E-05	0.012059098

674.439989	539.619789	1.21785E-05	0.008213645
674.479989	383.071617	8.64539E-06	0.005831144
674.519989	330.146785	7.45095E-06	0.005025817
674.559989	335.042336	7.56144E-06	0.005100644
674.599989	385.643091	8.70343E-06	0.005871332
674.639989	458.555811	1.0349E-05	0.006981826
674.679989	475.643571	1.07346E-05	0.007242429
674.719989	433.916217	9.79288E-06	0.006607455
674.759989	402.85426	9.09186E-06	0.006134823
674.799989	420.78373	9.4965E-06	0.00640824
674.839989	418.144627	9.43694E-06	0.006368426
674.879989	390.655815	8.81656E-06	0.005950118
674.919989	406.589153	9.17615E-06	0.006193168
674.959989	515.72919	1.16393E-05	0.007856055
674.999989	709.000467	1.60012E-05	0.010800778
675.039989	1037.720057	2.34199E-05	0.015809367
675.079989	1476.879953	3.33311E-05	0.022501174
675.119989	1938.100687	4.37402E-05	0.029529906
675.159989	2278.044941	5.14123E-05	0.034711529
675.199989	2482.529435	5.60272E-05	0.037829586
675.239989	2529.822423	5.70946E-05	0.038552536
675.279989	2402.311582	5.42168E-05	0.036611538
675.319989	2080.118745	4.69454E-05	0.031703155
675.359989	1630.896187	3.68071E-05	0.024858012
675.399989	1164.082769	2.62717E-05	0.017743924
675.439989	807.607788	1.82266E-05	0.012310963
675.479989	561.040103	1.26619E-05	0.008552856
675.519989	396.836552	8.95605E-06	0.00604999
675.559989	333.58865	7.52863E-06	0.005086042
675.599989	380.877784	8.59588E-06	0.005807377
675.639989	455.261166	1.02746E-05	0.006941938
675.679989	467.884358	1.05595E-05	0.007134842
675.719989	469.358713	1.05928E-05	0.007157748
675.759989	465.979584	1.05165E-05	0.007106637
675.799989	428.949091	9.68078E-06	0.006542273
675.839989	373.657828	8.43294E-06	0.005699316
675.879989	306.160499	6.90962E-06	0.004670071
675.919989	265.698255	5.99644E-06	0.004053113
675.959989	297.342853	6.71061E-06	0.004536107
675.999989	425.655266	9.60645E-06	0.006493958
676.039989	648.817671	1.46429E-05	0.009899193
676.079989	978.175933	2.20761E-05	0.014925188
676.119989	1310.243938	2.95704E-05	0.019993125
676.159989	1568.119378	3.53903E-05	0.023929485
676.199989	1761.077768	3.97451E-05	0.026875617
676.239989	1787.973326	4.03521E-05	0.027287682

676.279989	1628.510038	3.67532E-05	0.024855456
676.319989	1355.616941	3.05944E-05	0.020691595
676.359989	1050.320796	2.37043E-05	0.016032625
676.399989	755.975162	1.70613E-05	0.011540267
676.439989	524.977043	1.1848E-05	0.008014461
676.479989	385.093994	8.69104E-06	0.005879311
676.519989	339.725458	7.66713E-06	0.005186967
676.559989	318.111439	7.17933E-06	0.004857249
676.599989	333.22142	7.52034E-06	0.005088264
676.639989	380.012386	8.57635E-06	0.005803102
676.679989	427.802934	9.65492E-06	0.006533289
676.719989	462.776446	1.04442E-05	0.007067812
676.759989	435.203189	9.82193E-06	0.006647089
676.799989	375.596514	8.47669E-06	0.005737024
676.839989	357.214925	8.06184E-06	0.005456578
676.879989	382.693195	8.63685E-06	0.005846113
676.919989	428.009319	9.65957E-06	0.006538759
676.959989	532.528585	1.20184E-05	0.008135995
676.999989	787.48085	1.77723E-05	0.012031878
677.039989	1273.457409	2.87402E-05	0.019458238
677.079989	1891.342509	4.2685E-05	0.028901136
677.119989	2477.943673	5.59237E-05	0.03786708
677.159989	2948.466927	6.65428E-05	0.045060116
677.199989	3145.863336	7.09978E-05	0.048079678
677.239989	2910.221508	6.56796E-05	0.044480883
677.279989	2419.441276	5.46034E-05	0.036981803
677.319989	1984.815844	4.47945E-05	0.03034023
677.359989	1556.056637	3.5118E-05	0.023787549
677.399989	1115.996251	2.51865E-05	0.017061322
677.439989	732.122473	1.6523E-05	0.01119333
677.479989	495.875403	1.11912E-05	0.007581826
677.519989	408.890825	9.2281E-06	0.00625222
677.559989	399.682862	9.02029E-06	0.006111785
677.599989	476.975889	1.07647E-05	0.007294148
677.639989	616.281842	1.39086E-05	0.009425039
677.679989	699.597693	1.57889E-05	0.010699852
677.719989	655.496886	1.47937E-05	0.010025953
677.759989	572.915316	1.29299E-05	0.008763369
677.799989	465.26171	1.05003E-05	0.007117109
677.839989	351.053512	7.92279E-06	0.005370383
677.879989	298.521158	6.73721E-06	0.004567018
677.919989	331.59564	7.48365E-06	0.005073317
677.959989	391.491467	8.83542E-06	0.005990059
677.999989	527.487409	1.19047E-05	0.008071357
678.039989	759.0109	1.71298E-05	0.011614702
678.079989	1088.090356	2.45567E-05	0.016651396

678.119989	1490.259309	3.36331E-05	0.022807262
678.159989	1790.629886	4.0412E-05	0.027405817
678.199989	1971.387488	4.44915E-05	0.030174114
678.239989	1971.000792	4.44827E-05	0.030169975
678.279989	1789.459462	4.03856E-05	0.027392749
678.319989	1516.494439	3.42252E-05	0.023215615
678.359989	1219.256421	2.75169E-05	0.018666377
678.399989	909.939981	2.05361E-05	0.013931675
678.439989	650.930258	1.46906E-05	0.009966683
678.479989	432.654705	9.76441E-06	0.00662496
678.519989	342.308949	7.72544E-06	0.005241863
678.559989	382.297423	8.62792E-06	0.005854562
678.599989	460.736549	1.03982E-05	0.007056206
678.639989	507.579214	1.14554E-05	0.007774063
678.679989	552.47484	1.24686E-05	0.008462181
678.719989	564.147204	1.2732E-05	0.008641474
678.759989	516.749742	1.16623E-05	0.007915917
678.799989	435.965351	9.83913E-06	0.006678802
678.839989	369.69714	8.34355E-06	0.005663935
678.879989	372.45399	8.40577E-06	0.005706508
678.919989	435.156122	9.82087E-06	0.006667583
678.959989	551.613167	1.24491E-05	0.008452469
678.999989	742.469664	1.67565E-05	0.011377667
679.039989	1120.470034	2.52874E-05	0.017171188
679.079989	1637.992185	3.69672E-05	0.025103687
679.119989	2121.306097	4.78749E-05	0.032512818
679.159989	2502.129134	5.64696E-05	0.038351872
679.199989	2679.416555	6.04707E-05	0.041071698
679.239989	2591.340713	5.8483E-05	0.039723959
679.279989	2269.38283	5.12168E-05	0.034790554
679.319989	1872.812656	4.22668E-05	0.028712664
679.359989	1486.431319	3.35467E-05	0.022790275
679.399989	1154.901719	2.60645E-05	0.017708237
679.439989	836.145969	1.88706E-05	0.012821474
679.479989	604.054259	1.36327E-05	0.009263122
679.519989	474.842373	1.07165E-05	0.007282097
679.559989	423.111056	9.54903E-06	0.006489137
679.599989	427.130849	9.63975E-06	0.006551173
679.639989	449.653665	1.01481E-05	0.006897025
679.679989	466.939126	1.05382E-05	0.00716258
679.719989	477.553382	1.07777E-05	0.007325828
679.759989	434.320719	9.80201E-06	0.006663017
679.799989	384.814954	8.68474E-06	0.005903884
679.839989	331.018635	7.47063E-06	0.005078833
679.879989	282.002452	6.3644E-06	0.00432703
679.919989	278.194524	6.27846E-06	0.004268852

679.959989	320.555015	7.23448E-06	0.004919157
679.999989	469.402393	1.05938E-05	0.007203755
680.039989	692.369892	1.56258E-05	0.010626185
680.079989	989.469538	2.23309E-05	0.015186832
680.119989	1370.838289	3.09379E-05	0.021041492
680.159989	1687.488967	3.80843E-05	0.0259034
680.199989	1798.043231	4.05793E-05	0.027602061
680.239989	1803.767489	4.07085E-05	0.027691563
680.279989	1678.867965	3.78897E-05	0.025775613
680.319989	1416.683741	3.19726E-05	0.021751584
680.359989	1134.983395	2.5615E-05	0.017427417
680.399989	872.227179	1.9685E-05	0.013393641
680.439989	670.877027	1.51408E-05	0.010302379
680.479989	539.908746	1.2185E-05	0.008291641
680.519989	425.784068	9.60935E-06	0.006539357
680.559989	358.694749	8.09524E-06	0.005509297
680.599989	353.737895	7.98337E-06	0.005433483
680.639989	370.086807	8.35234E-06	0.005684939
680.679989	422.968804	9.54582E-06	0.006497646
680.719989	446.237668	1.0071E-05	0.006855506
680.759989	429.634892	9.69626E-06	0.006600827
680.799989	423.158825	9.55011E-06	0.006501712
680.839989	415.272045	9.37211E-06	0.006380908
680.879989	404.548929	9.13011E-06	0.006216506
680.919989	431.010381	9.7273E-06	0.006623516
680.959989	538.243575	1.21474E-05	0.008271899
680.999989	807.056605	1.82141E-05	0.012403831
681.039989	1170.845485	2.64244E-05	0.01799604
681.079989	1586.041814	3.57948E-05	0.024379091
681.119989	2056.629262	4.64153E-05	0.03161436
681.159989	2500.53805	5.64337E-05	0.038440352
681.199989	2762.850998	6.23537E-05	0.042475339
681.239989	2705.330806	6.10555E-05	0.041593481
681.279989	2406.514931	5.43117E-05	0.037001468
681.319989	1998.393375	4.5101E-05	0.030728182
681.359989	1527.893427	3.44824E-05	0.023494946
681.399989	1154.448183	2.60543E-05	0.017753391
681.439989	850.596855	1.91968E-05	0.013081458
681.479989	606.946614	1.36979E-05	0.009334872
681.519989	451.768263	1.01958E-05	0.006948628
681.559989	405.977784	9.16235E-06	0.006244693
681.599989	427.257499	9.64261E-06	0.006572401
681.639989	425.479279	9.60247E-06	0.006545431
681.679989	423.357761	9.55459E-06	0.006513176
681.719989	424.529791	9.58105E-06	0.006531591
681.759989	425.898284	9.61193E-06	0.00655303

681.799989	403.602834	9.10875E-06	0.006210348
681.839989	336.545469	7.59536E-06	0.005178822
681.879989	297.484535	6.71381E-06	0.004578014
681.919989	317.375048	7.16271E-06	0.004884397
681.959989	352.858973	7.96354E-06	0.005430813
681.999989	445.008143	1.00432E-05	0.006849472
682.039989	663.270581	1.49691E-05	0.01020952
682.079989	954.448573	2.15406E-05	0.014692395
682.119989	1241.476043	2.80184E-05	0.019111901
682.159989	1519.141532	3.42849E-05	0.023387793
682.199989	1700.140088	3.83698E-05	0.026175873
682.239989	1713.321983	3.86673E-05	0.026380372
682.279989	1596.089572	3.60215E-05	0.024576761
682.319989	1371.54758	3.09539E-05	0.021120477
682.359989	1059.263347	2.39061E-05	0.016312565
682.399989	785.61717	1.77303E-05	0.012099146
682.439989	607.1522	1.37026E-05	0.009351188
682.479989	456.748622	1.03082E-05	0.007035127
682.519989	380.86539	8.5956E-06	0.00586667
682.559989	367.7932	8.30058E-06	0.005665644
682.599989	383.364321	8.652E-06	0.005905854
682.639989	402.203821	9.07718E-06	0.006196446
682.679989	441.073806	9.95442E-06	0.006795684
682.719989	480.845635	1.0852E-05	0.007408888
682.759989	459.708763	1.0375E-05	0.007083626
682.799989	413.372661	9.32925E-06	0.006370009
682.839989	403.070777	9.09675E-06	0.006211622
682.879989	395.600002	8.92814E-06	0.006096849
682.919989	414.988672	9.36572E-06	0.006396035
682.959989	519.287315	1.17196E-05	0.008004012
682.999989	703.478672	1.58765E-05	0.010843672
683.039989	1034.246523	2.33415E-05	0.01594318
683.079989	1492.577212	3.36854E-05	0.023009815
683.119989	1972.089303	4.45073E-05	0.030403832
683.159989	2333.710027	5.26686E-05	0.03598107
683.199989	2560.40218	5.77847E-05	0.039478514
683.239989	2524.379994	5.69717E-05	0.038925371
683.279989	2207.764256	4.98262E-05	0.034045221
683.319989	1783.47423	4.02505E-05	0.027503991
683.359989	1355.353437	3.05884E-05	0.020902915
683.399989	1039.391557	2.34576E-05	0.016030937
683.439989	814.224467	1.83759E-05	0.012558833
683.479989	647.418039	1.46113E-05	0.009986547
683.519989	533.960578	1.20507E-05	0.008236926
683.559989	479.904282	1.08308E-05	0.007403482
683.599989	473.767895	1.06923E-05	0.007309244

683.639989	486.156154	1.09719E-05	0.007500807
683.679989	493.236309	1.11317E-05	0.007610491
683.719989	471.563615	1.06425E-05	0.007276513
683.759989	444.133455	1.00235E-05	0.00685365
683.799989	420.58387	9.49199E-06	0.006490624
683.839989	360.971453	8.14662E-06	0.005570987
683.879989	326.802884	7.37549E-06	0.005043947
683.919989	359.870732	8.12178E-06	0.005554648
683.959989	425.647944	9.60628E-06	0.006570312
683.999989	553.483586	1.24914E-05	0.008544086
684.039989	771.838363	1.74193E-05	0.011915509
684.079989	1157.707552	2.61278E-05	0.017873537
684.119989	1617.064895	3.64949E-05	0.024966892
684.159989	1927.434213	4.34995E-05	0.029760621
684.199989	2106.679667	4.75448E-05	0.032530169
684.239989	2076.011401	4.68527E-05	0.032058481
684.279989	1788.461492	4.03631E-05	0.027619651
684.319989	1452.360757	3.27778E-05	0.022430476
684.359989	1180.87342	2.66507E-05	0.018238651
684.399989	900.635393	2.03261E-05	0.013911173
684.439989	676.03987	1.52573E-05	0.010442691
684.479989	545.570432	1.23128E-05	0.008427841
684.519989	484.060868	1.09246E-05	0.007478093
684.559989	456.877786	1.03111E-05	0.007058563
684.599989	480.720438	1.08492E-05	0.007427356
684.639989	536.339601	1.21044E-05	0.008287182
684.679989	527.797588	1.19117E-05	0.008155673
684.719989	481.306734	1.08624E-05	0.007437718
684.759989	437.389044	9.87126E-06	0.006759445
684.799989	394.757251	8.90912E-06	0.006100966
684.839989	352.100663	7.94642E-06	0.005442027
684.879989	328.018555	7.40292E-06	0.005070113
684.919989	363.021159	8.19288E-06	0.005611469
684.959989	492.910492	1.11243E-05	0.007619703
684.999989	742.046685	1.6747E-05	0.011471667
685.039989	1097.352961	2.47657E-05	0.016965514
685.079989	1551.431241	3.50136E-05	0.023987146
685.119989	2098.460933	4.73593E-05	0.032446831
685.159989	2548.096775	5.7507E-05	0.039401492
685.199989	2723.044875	6.14553E-05	0.042109191
685.239989	2613.538695	5.89839E-05	0.040418146
685.279989	2243.674787	5.06366E-05	0.034700259
685.319989	1799.681434	4.06163E-05	0.027835164
685.359989	1464.537067	3.30526E-05	0.022652903
685.399989	1162.042548	2.62257E-05	0.017975082
685.439989	878.789841	1.98331E-05	0.013594374

685.479989	666.81339	1.5049E-05	0.010315822
685.519989	512.133572	1.15581E-05	0.007923337
685.559989	415.022862	9.36649E-06	0.006421289
685.599989	414.265662	9.3494E-06	0.006409948
685.639989	451.96959	1.02003E-05	0.00699375
685.679989	467.871533	1.05592E-05	0.007240238
685.719989	458.968197	1.03583E-05	0.007102875
685.759989	432.922742	9.77046E-06	0.006700193
685.799989	397.691075	8.97533E-06	0.006155284
685.839989	325.435194	7.34462E-06	0.005037233
685.879989	302.206421	6.82038E-06	0.004677961
685.919989	330.367134	7.45593E-06	0.005114169
685.959989	393.294438	8.87611E-06	0.006088655
685.999989	501.510556	1.13184E-05	0.007764419
686.039989	718.711907	1.62203E-05	0.011127793
686.079989	1040.335011	2.34789E-05	0.016108412
686.119989	1453.599375	3.28057E-05	0.022508656
686.159989	1765.001079	3.98336E-05	0.027332233
686.199989	1863.744103	4.20621E-05	0.028863018
686.239989	1752.959208	3.95618E-05	0.027148922
686.279989	1534.156788	3.46238E-05	0.023761609
686.319989	1339.649628	3.0234E-05	0.020750216
686.359989	1060.984584	2.39449E-05	0.016434852
686.399989	778.905406	1.75788E-05	0.012066095
686.439989	582.981896	1.31571E-05	0.009031552
686.479989	453.658565	1.02384E-05	0.007028485
686.519989	387.82738	8.75272E-06	0.00600892
686.559989	365.390517	8.24636E-06	0.005661617
686.599989	379.191129	8.55782E-06	0.005875796
686.639989	419.160869	9.45988E-06	0.00649553
686.679989	472.591447	1.06657E-05	0.007323944
686.719989	501.806801	1.13251E-05	0.007777159
686.759989	453.250467	1.02292E-05	0.007025027
686.799989	387.329667	8.74149E-06	0.006003656
686.839989	334.843005	7.55694E-06	0.005190409
686.879989	337.828762	7.62432E-06	0.005236996
686.919989	373.942738	8.43937E-06	0.00579717
686.959989	477.032828	1.0766E-05	0.007395788
686.999989	693.898574	1.56603E-05	0.010758643
687.039989	1098.591562	2.47937E-05	0.017034251
687.079989	1593.300151	3.59586E-05	0.024706411
687.119989	2039.689355	4.60329E-05	0.031630159
687.159989	2367.492084	5.3431E-05	0.036715644
687.199989	2551.98397	5.75947E-05	0.039579093
687.239989	2467.763779	5.5694E-05	0.038275138
687.279989	2118.841403	4.78193E-05	0.032865247

687.319989	1702.957198	3.84334E-05	0.026416025
687.359989	1265.069014	2.85508E-05	0.019624709
687.399989	889.891819	2.00836E-05	0.013805479
687.439989	596.431222	1.34606E-05	0.00925337
687.479989	401.140207	9.05318E-06	0.006223877
687.519989	331.320818	7.47745E-06	0.005140896
687.559989	337.407267	7.61481E-06	0.00523564
687.599989	378.731753	8.54745E-06	0.005877225
687.639989	432.24598	9.75519E-06	0.006708059
687.679989	450.859666	1.01753E-05	0.006997333
687.719989	437.965739	9.88428E-06	0.006797615
687.759989	384.576575	8.67936E-06	0.005969315
687.799989	321.709997	7.26055E-06	0.004993804
687.839989	285.216646	6.43694E-06	0.004427586
687.879989	264.848529	5.97726E-06	0.004111639
687.919989	282.794288	6.38227E-06	0.004390493
687.959989	339.161186	7.6544E-06	0.005265918
687.999989	471.200161	1.06343E-05	0.00731642
688.039989	694.995537	1.56851E-05	0.010791963
688.079989	990.210112	2.23477E-05	0.01537698
688.119989	1290.180406	2.91176E-05	0.020036385
688.159989	1557.78683	3.51571E-05	0.024193695
688.199989	1707.11113	3.85271E-05	0.026514364
688.239989	1738.730036	3.92407E-05	0.027007029
688.279989	1583.921639	3.57469E-05	0.024603879
688.319989	1278.375291	2.88511E-05	0.019858823
688.359989	1009.419992	2.27812E-05	0.015681669
688.399989	770.021167	1.73783E-05	0.011963225
688.439989	551.450396	1.24455E-05	0.008567957
688.479989	397.208764	8.96445E-06	0.006171843
688.519989	344.694369	7.77927E-06	0.005356184
688.559989	347.660986	7.84622E-06	0.005402596
688.599989	384.166117	8.67009E-06	0.005970227
688.639989	427.681419	9.65217E-06	0.006646873
688.679989	452.590752	1.02143E-05	0.007034414
688.719989	442.021284	9.9758E-06	0.006870536
688.759989	415.951818	9.38745E-06	0.006465702
688.799989	393.715004	8.8856E-06	0.006120401
688.839989	361.948484	8.16867E-06	0.005626909
688.879989	351.363473	7.92978E-06	0.00546267
688.919989	367.009363	8.28289E-06	0.005706249
688.959989	471.199913	1.06343E-05	0.007326625
688.999989	737.893877	1.66532E-05	0.01147408
689.039989	1201.436251	2.71147E-05	0.018683142
689.079989	1831.903745	4.13435E-05	0.028488989
689.119989	2572.965138	5.80682E-05	0.040015984

689.159989	3179.723434	7.17619E-05	0.04945545
689.199989	3404.143627	7.68268E-05	0.052949016
689.239989	3237.09777	7.30568E-05	0.05035366
689.279989	2831.887996	6.39118E-05	0.044053102
689.319989	2303.17801	5.19795E-05	0.035830522
689.359989	1682.118309	3.79631E-05	0.02617022
689.399989	1124.57855	2.53802E-05	0.01749709
689.439989	740.514993	1.67124E-05	0.011522191
689.479989	506.214395	1.14246E-05	0.007877001
689.519989	376.070575	8.48739E-06	0.005852224
689.559989	342.325653	7.72581E-06	0.005327412
689.599989	351.215019	7.92643E-06	0.005466069
689.639989	392.228779	8.85206E-06	0.006104733
689.679989	442.58633	9.98856E-06	0.006888908
689.719989	439.600513	9.92117E-06	0.00684283
689.759989	411.550965	9.28813E-06	0.006406582
689.799989	358.091357	8.08162E-06	0.005574704
689.839989	312.269484	7.04749E-06	0.004861639
689.879989	292.790108	6.60786E-06	0.004558634
689.919989	299.490163	6.75908E-06	0.004663222
689.959989	340.187264	7.67755E-06	0.005297204
689.999989	464.144708	1.04751E-05	0.007227819
690.039989	700.478507	1.58088E-05	0.010908721
690.079989	1081.55907	2.44093E-05	0.016844357
690.119989	1498.433183	3.38175E-05	0.023338167
690.159989	1878.350509	4.23918E-05	0.029257093
690.199989	2114.519241	4.77218E-05	0.032937554
690.239989	2166.514674	4.88952E-05	0.033749435
690.279989	1993.35525	4.49873E-05	0.031053799
690.319989	1646.289645	3.71545E-05	0.025648469
690.359989	1266.653709	2.85866E-05	0.019735052
690.399989	911.957222	2.05816E-05	0.014209539
690.439989	641.596006	1.44799E-05	0.009997521
690.479989	460.807743	1.03998E-05	0.007180846
690.519989	373.931263	8.43911E-06	0.005827373
690.559989	361.053446	8.14847E-06	0.00562701
690.599989	358.160407	8.08318E-06	0.005582245
690.639989	384.550286	8.67876E-06	0.005993902
690.679989	446.675991	1.00809E-05	0.006962645
690.719989	487.614893	1.10048E-05	0.007601228
690.759989	483.756011	1.09177E-05	0.00754151
690.799989	467.231676	1.05448E-05	0.007284326
690.839989	404.018989	9.11815E-06	0.00629918
690.879989	343.645312	7.7556E-06	0.005358186
690.919989	368.195409	8.30966E-06	0.005741308
690.959989	476.309229	1.07496E-05	0.007427568

690.999989	701.625526	1.58347E-05	0.010941785
691.039989	1137.206453	2.56652E-05	0.017735656
691.079989	1715.408805	3.87144E-05	0.026754738
691.119989	2234.691944	5.04339E-05	0.034855866
691.159989	2600.731009	5.86949E-05	0.04056755
691.199989	2794.038542	6.30576E-05	0.043585383
691.239989	2721.917949	6.14299E-05	0.042462801
691.279989	2429.018867	5.48196E-05	0.037895673
691.319989	2013.135374	4.54337E-05	0.031409198
691.359989	1557.441837	3.51493E-05	0.024300815
691.399989	1109.24765	2.50342E-05	0.017308628
691.439989	726.791192	1.64027E-05	0.011341458
691.479989	506.505341	1.14311E-05	0.007904391
691.519989	372.32631	8.40289E-06	0.005810764
691.559989	319.534392	7.21145E-06	0.004987148
691.599989	319.729469	7.21585E-06	0.004990481
691.639989	362.340041	8.17751E-06	0.005655893
691.679989	415.964235	9.38773E-06	0.006493307
691.719989	422.593898	9.53736E-06	0.00659718
691.759989	391.13134	8.82729E-06	0.006106366
691.799989	357.969173	8.07887E-06	0.005588959
691.839989	332.639651	7.50721E-06	0.005193791
691.879989	316.740538	7.14839E-06	0.00494583
691.919989	343.0794	7.74282E-06	0.005357415
691.959989	414.955283	9.36496E-06	0.00648018
691.999989	538.516277	1.21536E-05	0.008410265
692.039989	718.065243	1.62057E-05	0.011215015
692.079989	1001.686241	2.26067E-05	0.015645619
692.119989	1364.361598	3.07917E-05	0.021311579
692.159989	1700.753605	3.83836E-05	0.02656762
692.199989	1892.432612	4.27096E-05	0.029563563
692.239989	1877.012155	4.23615E-05	0.029324359
692.279989	1749.966564	3.94943E-05	0.027341119
692.319989	1512.798338	3.41418E-05	0.023637017
692.359989	1197.521714	2.70264E-05	0.018711996
692.399989	879.025736	1.98384E-05	0.013736099
692.439989	602.021612	1.35868E-05	0.009408036
692.479989	419.262958	9.46218E-06	0.006552371
692.519989	341.267057	7.70192E-06	0.005333735
692.559989	349.766696	7.89375E-06	0.005466893
692.599989	390.452861	8.81198E-06	0.006103175
692.639989	449.391006	1.01421E-05	0.007024844
692.679989	487.572922	1.10038E-05	0.007622141
692.719989	486.248093	1.09739E-05	0.007601869
692.759989	441.128162	9.95565E-06	0.006896875
692.799989	377.236665	8.51371E-06	0.005898295

692.839989	308.387296	6.95987E-06	0.004822077
692.879989	310.271481	7.0024E-06	0.004851819
692.919989	377.907123	8.52884E-06	0.005909802
692.959989	477.144441	1.07685E-05	0.00746213
692.999989	683.880311	1.54342E-05	0.010695918
693.039989	1035.601184	2.33721E-05	0.016197783
693.079989	1460.160814	3.29538E-05	0.022839616
693.119989	1874.752321	4.23105E-05	0.029326287
693.159989	2208.673369	4.98467E-05	0.034551725
693.199989	2440.991396	5.50898E-05	0.038188232
693.239989	2533.778805	5.71839E-05	0.039642137
693.279989	2353.695284	5.31196E-05	0.036826772
693.319989	1957.445844	4.41768E-05	0.030628677
693.359989	1439.764635	3.24935E-05	0.022529681
693.399989	983.417984	2.21944E-05	0.015389579
693.439989	686.422566	1.54916E-05	0.010742496
693.479989	506.89619	1.14399E-05	0.00793337
693.519989	400.230063	9.03264E-06	0.006264313
693.559989	350.06372	7.90045E-06	0.005479436
693.599989	352.891744	7.96428E-06	0.005524021
693.639989	368.746454	8.32209E-06	0.005772537
693.679989	408.847406	9.22712E-06	0.006400666
693.719989	446.519718	1.00773E-05	0.006990844
693.759989	440.137193	9.93328E-06	0.006891315
693.799989	386.690534	8.72707E-06	0.006054839
693.839989	331.209159	7.47493E-06	0.005186405
693.879989	282.153058	6.3678E-06	0.00441849
693.919989	290.352741	6.55286E-06	0.004547158
693.959989	372.815024	8.41392E-06	0.005838921
693.999989	508.079086	1.14666E-05	0.007957846
694.039989	704.50392	1.58997E-05	0.011035008
694.079989	978.551455	2.20845E-05	0.01532844
694.119989	1293.194836	2.91856E-05	0.020258313
694.159989	1527.323956	3.44696E-05	0.023927399
694.199989	1685.472218	3.80388E-05	0.026406507
694.239989	1699.004971	3.83442E-05	0.02662006
694.279989	1523.964569	3.43938E-05	0.023878898
694.319989	1334.984127	3.01287E-05	0.020918981
694.359989	1091.483469	2.46333E-05	0.017104351
694.399989	813.211755	1.83531E-05	0.012744362
694.439989	572.213373	1.29141E-05	0.008968039
694.479989	406.151219	9.16627E-06	0.006365789
694.519989	336.586363	7.59629E-06	0.005275772
694.559989	348.031649	7.85459E-06	0.005455484
694.599989	389.626126	8.79332E-06	0.006107839
694.639989	421.880064	9.52125E-06	0.006613838

694.679989	459.544633	1.03713E-05	0.007204722
694.719989	463.496801	1.04605E-05	0.007267103
694.759989	430.848806	9.72366E-06	0.006755608
694.799989	408.960325	9.22966E-06	0.006412771
694.839989	381.860776	8.61807E-06	0.005988177
694.879989	375.155609	8.46674E-06	0.005883368
694.919989	402.402849	9.08167E-06	0.006311035
694.959989	480.612905	1.08468E-05	0.007538067
694.999989	697.493576	1.57415E-05	0.010940313
695.039989	1067.795971	2.40987E-05	0.016749538
695.079989	1520.884553	3.43242E-05	0.023858096
695.119989	1989.526333	4.49008E-05	0.03121147
695.159989	2346.383135	5.29546E-05	0.036811919
695.199989	2496.577729	5.63443E-05	0.039170544
695.239989	2478.598083	5.59385E-05	0.038890687
695.279989	2263.485973	5.10837E-05	0.035517492
695.319989	1851.177628	4.17785E-05	0.029049427
695.359989	1439.591887	3.24896E-05	0.022591957
695.399989	1071.88113	2.41909E-05	0.016822327
695.439989	750.487005	1.69374E-05	0.011778978
695.479989	529.738991	1.19555E-05	0.008314791
695.519989	411.516772	9.28736E-06	0.006459545
695.559989	363.764222	8.20965E-06	0.005710305
695.599989	368.824096	8.32385E-06	0.005790067
695.639989	409.892132	9.25069E-06	0.006435153
695.679989	422.507384	9.5354E-06	0.006633589
695.719989	414.607827	9.35712E-06	0.006509936
695.759989	392.716273	8.86306E-06	0.006166562
695.799989	388.54974	8.76903E-06	0.006101488
695.839989	361.253717	8.15299E-06	0.005673179
695.879989	314.749665	7.10346E-06	0.004943157
695.919989	310.975423	7.01828E-06	0.004884163
695.959989	352.691067	7.95975E-06	0.005539665
695.999989	479.205557	1.0815E-05	0.007527241
696.039989	716.211487	1.61639E-05	0.011250718
696.079989	991.445853	2.23756E-05	0.015575174
696.119989	1283.281176	2.89619E-05	0.020160936
696.159989	1551.518541	3.50156E-05	0.024376468
696.199989	1740.338954	3.9277E-05	0.027344665
696.239989	1785.927958	4.03059E-05	0.028062584
696.279989	1766.354904	3.98642E-05	0.027756624
696.319989	1690.752118	3.81579E-05	0.026570123
696.359989	1496.63826	3.3777E-05	0.02352098
696.399989	1263.348245	2.8512E-05	0.019855764
696.439989	962.595333	2.17244E-05	0.015129766
696.479989	684.610286	1.54507E-05	0.010761104

696.519989	524.529915	1.18379E-05	0.008245341
696.559989	465.998924	1.05169E-05	0.007325684
696.599989	433.058435	9.77353E-06	0.006808238
696.639989	446.376959	1.00741E-05	0.007018025
696.679989	450.632214	1.01701E-05	0.007085334
696.719989	432.028347	9.75028E-06	0.006793214
696.759989	398.432098	8.99206E-06	0.006265306
696.799989	367.762366	8.29988E-06	0.005783359
696.839989	357.340512	8.06468E-06	0.00561979
696.879989	372.084476	8.39743E-06	0.005852
696.919989	412.573666	9.31121E-06	0.00648917
696.959989	508.738183	1.14815E-05	0.008002155
696.999989	726.517521	1.63965E-05	0.011428352
697.039989	1092.626648	2.46591E-05	0.017188352
697.079989	1538.544556	3.47228E-05	0.024204574
697.119989	1966.839505	4.43888E-05	0.030944339
697.159989	2255.13668	5.08953E-05	0.035482162
697.199989	2338.494437	5.27766E-05	0.036795819
697.239989	2291.973818	5.17267E-05	0.036065893
697.279989	2105.841732	4.75259E-05	0.033138869
697.319989	1767.503585	3.98901E-05	0.02781616
697.359989	1376.345607	3.10622E-05	0.021661538
697.399989	1028.736599	2.32172E-05	0.016191642
697.439989	747.112279	1.68613E-05	0.011759733
697.479989	545.582699	1.2313E-05	0.0085881
697.519989	410.243002	9.25861E-06	0.006458068
697.559989	363.971161	8.21432E-06	0.005729982
697.599989	340.169068	7.67714E-06	0.005355574
697.639989	373.361131	8.42624E-06	0.005878482
697.679989	428.615305	9.67325E-06	0.006748833
697.719989	442.407288	9.98452E-06	0.006966397
697.759989	435.628871	9.83154E-06	0.006860053
697.799989	376.655546	8.50059E-06	0.005931712
697.839989	328.918236	7.42323E-06	0.005180224
697.879989	328.900671	7.42283E-06	0.005180245
697.919989	334.833808	7.55673E-06	0.005273995
697.959989	388.040464	8.75753E-06	0.006112407
697.999989	519.695023	1.17288E-05	0.008186697
698.039989	751.184657	1.69532E-05	0.011834006
698.079989	1017.642651	2.29668E-05	0.016032648
698.119989	1314.087664	2.96571E-05	0.020704234
698.159989	1553.179151	3.50531E-05	0.024472665
698.199989	1636.941611	3.69435E-05	0.025793946
698.239989	1616.538722	3.6483E-05	0.025473909
698.279989	1470.940571	3.31971E-05	0.023180857
698.319989	1176.240636	2.65461E-05	0.018537681

698.359989	909.19246	2.05192E-05	0.014329793
698.399989	713.95983	1.61131E-05	0.011253374
698.439989	546.918261	1.23432E-05	0.008620973
698.479989	424.195566	9.5735E-06	0.0066869
698.519989	360.42008	8.13418E-06	0.005681887
698.559989	353.669961	7.98184E-06	0.005575793
698.599989	376.719594	8.50204E-06	0.005939523
698.639989	404.588666	9.131E-06	0.006379284
698.679989	432.411247	9.75892E-06	0.006818362
698.719989	448.520395	1.01225E-05	0.00707278
698.759989	443.893415	1.00181E-05	0.007000217
698.799989	425.27092	9.59777E-06	0.006706923
698.839989	397.533837	8.97178E-06	0.006269842
698.879989	402.874778	9.09232E-06	0.006354442
698.919989	463.06908	1.04508E-05	0.00730429
698.959989	560.041743	1.26394E-05	0.008834408
698.999989	711.265221	1.60523E-05	0.011220533
699.039989	999.898113	2.25663E-05	0.015774751
699.079989	1417.588021	3.1993E-05	0.022365657
699.119989	1794.51258	4.04996E-05	0.028314114
699.159989	2066.094551	4.66289E-05	0.032601045
699.199989	2206.953401	4.98079E-05	0.034825658
699.239989	2169.806055	4.89695E-05	0.034241433
699.279989	1945.554488	4.39085E-05	0.030704304
699.319989	1599.098907	3.60894E-05	0.025238063
699.359989	1244.741493	2.80921E-05	0.019646478
699.399989	965.112486	2.17812E-05	0.015233802
699.439989	800.32769	1.80623E-05	0.012633482
699.479989	627.180806	1.41546E-05	0.009900857
699.519989	466.624304	1.05311E-05	0.007366687
699.559989	367.886815	8.30269E-06	0.005808232
699.599989	348.934811	7.87497E-06	0.005509331
699.639989	410.42435	9.26271E-06	0.006480559
699.679989	472.735434	1.0669E-05	0.007464872
699.719989	484.349035	1.09311E-05	0.007648697
699.759989	438.223642	9.8901E-06	0.006920694
699.799989	352.147448	7.94748E-06	0.005561645
699.839989	292.918683	6.61077E-06	0.004626479
699.879989	279.164554	6.30036E-06	0.004409492
699.919989	294.511924	6.64672E-06	0.004652175
699.959989	360.741132	8.14142E-06	0.005698672
699.999989	484.071316	1.09248E-05	0.007647371
700.039989	673.817513	1.52071E-05	0.010645594
700.079989	901.36628	2.03426E-05	0.014241434
700.119989	1147.508695	2.58977E-05	0.018131478
700.159989	1424.681416	3.21531E-05	0.022512297

700.199989	1656.709558	3.73896E-05	0.026180216
700.239989	1695.313639	3.82609E-05	0.026791789
700.279989	1544.403998	3.4855E-05	0.024408292
700.319989	1280.232843	2.88931E-05	0.020234396
700.359989	982.363557	2.21706E-05	0.015527385
700.399989	771.139759	1.74036E-05	0.012189446
700.439989	618.575728	1.39604E-05	0.009778417
700.479989	489.112674	1.10386E-05	0.007732313
700.519989	418.887946	9.45372E-06	0.006622518
700.559989	408.81574	9.2264E-06	0.006463648
700.599989	435.834416	9.83618E-06	0.006891225
700.639989	490.378009	1.10671E-05	0.007754087
700.679989	504.792085	1.13925E-05	0.007982465
700.719989	459.43871	1.03689E-05	0.00726569
700.759989	436.319173	9.84712E-06	0.006900465
700.799989	429.222816	9.68696E-06	0.006788622
700.839989	397.731783	8.97625E-06	0.006290917
700.879989	372.007112	8.39568E-06	0.005884366
700.919989	378.403353	8.54004E-06	0.005985882
700.959989	502.81539	1.13478E-05	0.007954384
700.999989	741.845367	1.67424E-05	0.011736434
701.039989	1003.171723	2.26402E-05	0.015871677
701.079989	1378.863842	3.1119E-05	0.021816934
701.119989	1802.407959	4.06778E-05	0.028520045
701.159989	2067.638695	4.66637E-05	0.032718737
701.199989	2157.166205	4.86842E-05	0.034137386
701.239989	2117.698044	4.77935E-05	0.03351471
701.279989	1935.913952	4.36909E-05	0.030639541
701.319989	1642.378575	3.70662E-05	0.025995265
701.359989	1315.662799	2.96927E-05	0.020825255
701.399989	1032.350497	2.32987E-05	0.016341717
701.439989	783.088964	1.76732E-05	0.012396708
701.479989	563.766532	1.27234E-05	0.008925228
701.519989	407.714049	9.20154E-06	0.006455063
701.559989	370.998251	8.37291E-06	0.005874101
701.599989	392.008871	8.84709E-06	0.006207121
701.639989	393.451675	8.87966E-06	0.006230322
701.679989	397.272285	8.96588E-06	0.00629118
701.719989	412.816555	9.31669E-06	0.006537711
701.759989	385.174763	8.69286E-06	0.0061003
701.799989	362.695168	8.18552E-06	0.005744601
701.839989	341.902369	7.71626E-06	0.00541558
701.879989	296.115227	6.68291E-06	0.0046906
701.919989	308.992581	6.97353E-06	0.004894862
701.959989	379.576028	8.5665E-06	0.006013342
701.999989	518.554114	1.1703E-05	0.008215537

702.039989	711.000494	1.60463E-05	0.011265138
702.079989	950.021759	2.14407E-05	0.015053064
702.119989	1236.402093	2.79039E-05	0.019591867
702.159989	1457.745651	3.28993E-05	0.023100564
702.199989	1548.168483	3.494E-05	0.024534872
702.239989	1521.785172	3.43446E-05	0.024118132
702.279989	1370.294665	3.09256E-05	0.021718459
702.319989	1135.911432	2.56359E-05	0.018004632
702.359989	899.367394	2.02975E-05	0.01425613
702.399989	689.687111	1.55653E-05	0.010933051
702.439989	513.251002	1.15834E-05	0.008136615
702.479989	392.055939	8.84816E-06	0.006215653
702.519989	342.035037	7.71925E-06	0.00542293
702.559989	320.781973	7.2396E-06	0.005086255
702.599989	347.680976	7.84668E-06	0.005513074
702.639989	381.350489	8.60655E-06	0.006047306
702.679989	420.073208	9.48047E-06	0.006661735
702.719989	444.730865	1.0037E-05	0.00705317
702.759989	435.043986	9.81834E-06	0.006899934
702.799989	400.353043	9.03541E-06	0.006350086
702.839989	350.17851	7.90304E-06	0.005554573
702.879989	337.647811	7.62024E-06	0.005356115
702.919989	371.097496	8.37515E-06	0.005887063
702.959989	473.168401	1.06788E-05	0.007506735
702.999989	671.354545	1.51515E-05	0.01065153
703.039989	997.936421	2.2522E-05	0.015833891
703.079989	1369.020724	3.08969E-05	0.021722986
703.119989	1706.259757	3.85079E-05	0.027075679
703.159989	1954.321859	4.41063E-05	0.031013801
703.199989	2085.753714	4.70726E-05	0.033101421
703.239989	2082.559189	4.70005E-05	0.033052603
703.279989	1876.501863	4.235E-05	0.029783931
703.319989	1548.47883	3.4947E-05	0.024578931
703.359989	1173.802096	2.64911E-05	0.018632765
703.399989	835.869519	1.88644E-05	0.013269226
703.439989	588.561558	1.3283E-05	0.009343804
703.479989	442.426452	9.98495E-06	0.007024212
703.519989	374.52082	8.45241E-06	0.005946442
703.559989	365.195494	8.24195E-06	0.005798709
703.599989	368.486047	8.31622E-06	0.00585129
703.639989	379.006769	8.55365E-06	0.006018694
703.679989	367.606535	8.29637E-06	0.005837988
703.719989	353.945316	7.98805E-06	0.005621352
703.759989	366.826444	8.27876E-06	0.005826261
703.799989	353.158965	7.97031E-06	0.005609501
703.839989	313.19228	7.06831E-06	0.004974962

703.879989	299.037451	6.74886E-06	0.004750387
703.919989	299.478536	6.75881E-06	0.004757664
703.959989	331.796049	7.48817E-06	0.005271375
703.999989	457.59322	1.03272E-05	0.007270378
704.039989	693.24501	1.56456E-05	0.01101511
704.079989	994.155157	2.24367E-05	0.015797229
704.119989	1307.289584	2.95037E-05	0.020774148
704.159989	1549.403807	3.49679E-05	0.024622986
704.199989	1600.015036	3.61101E-05	0.02542874
704.239989	1549.598758	3.49723E-05	0.024628882
704.279989	1411.626647	3.18584E-05	0.022437267
704.319989	1195.163669	2.69732E-05	0.018997749
704.359989	928.479167	2.09545E-05	0.014759498
704.399989	698.005588	1.5753E-05	0.011096423
704.439989	513.204002	1.15823E-05	0.008159035
704.479989	376.712318	8.50187E-06	0.005989399
704.519989	324.026528	7.31283E-06	0.005152033
704.559989	315.214832	7.11396E-06	0.005012211
704.599989	350.033542	7.89977E-06	0.005566177
704.639989	395.837376	8.9335E-06	0.0062949
704.679989	412.197675	9.30273E-06	0.006555446
704.719989	420.292388	9.48541E-06	0.006684561
704.759989	431.162197	9.73073E-06	0.006857829
704.799989	425.848314	9.6108E-06	0.006773694
704.839989	397.854569	8.97902E-06	0.006328775
704.879989	390.333807	8.80929E-06	0.006209492
704.919989	438.121458	9.88779E-06	0.006970102
704.959989	578.084073	1.30466E-05	0.009197297
704.999989	867.247772	1.95726E-05	0.013798665
705.039989	1339.019302	3.02198E-05	0.021306168
705.079989	1959.056407	4.42132E-05	0.031173824
705.119989	2685.292191	6.06033E-05	0.042732601
705.159989	3316.285782	7.4844E-05	0.05277696
705.199989	3464.472966	7.81883E-05	0.055138409
705.239989	3127.577281	7.05851E-05	0.049779409
705.279989	2596.69397	5.86038E-05	0.041332063
705.319989	2010.919792	4.53837E-05	0.032010002
705.359989	1465.721539	3.30793E-05	0.02333281
705.399989	1009.910082	2.27923E-05	0.016077663
705.439989	701.932834	1.58416E-05	0.011175331
705.479989	519.799466	1.17311E-05	0.008276092
705.519989	434.562256	9.80746E-06	0.006919362
705.559989	403.412369	9.10446E-06	0.006423739
705.599989	415.777575	9.38352E-06	0.006621012
705.639989	421.164412	9.50509E-06	0.006707175
705.679989	435.830606	9.83609E-06	0.006941132

705.719989	436.011196	9.84017E-06	0.006944401
705.759989	389.978852	8.80128E-06	0.006211591
705.799989	336.263692	7.589E-06	0.005356318
705.839989	314.533011	7.09857E-06	0.005010456
705.879989	309.951408	6.99517E-06	0.004937752
705.919989	345.128875	7.78908E-06	0.005498466
705.959989	407.142589	9.18864E-06	0.006486813
705.999989	550.225699	1.24178E-05	0.008766986
706.039989	821.279199	1.85351E-05	0.013086541
706.079989	1169.305284	2.63896E-05	0.018633163
706.119989	1522.709044	3.43654E-05	0.024266112
706.159989	1781.631423	4.02089E-05	0.028393944
706.199989	1842.274943	4.15776E-05	0.029362086
706.239989	1726.340289	3.89611E-05	0.027515884
706.279989	1544.038445	3.48468E-05	0.024611595
706.319989	1257.444319	2.83788E-05	0.02004449
706.359989	953.014881	2.15082E-05	0.015192545
706.399989	700.130435	1.5801E-05	0.011161804
706.439989	528.802996	1.19343E-05	0.0084309
706.479989	430.107376	9.70692E-06	0.006857748
706.519989	374.981949	8.46282E-06	0.005979152
706.559989	352.815096	7.96255E-06	0.005626016
706.599989	379.827745	8.57218E-06	0.006057105
706.639989	422.838071	9.54287E-06	0.006743371
706.679989	447.520784	1.00999E-05	0.007137412
706.719989	428.464647	9.66985E-06	0.006833876
706.759989	393.418415	8.87891E-06	0.006275255
706.799989	366.994609	8.28256E-06	0.005854111
706.839989	333.702588	7.5312E-06	0.005323355
706.879989	342.277485	7.72473E-06	0.005460454
706.919989	409.961379	9.25226E-06	0.006540606
706.959989	545.481019	1.23107E-05	0.008703205
706.999989	754.600463	1.70303E-05	0.012040409
707.039989	1048.599236	2.36654E-05	0.016732401
707.079989	1508.311983	3.40405E-05	0.024069357
707.119989	2047.032997	4.61987E-05	0.032668013
707.159989	2476.443855	5.58899E-05	0.039523093
707.199989	2581.888138	5.82696E-05	0.041208274
707.239989	2393.664765	5.40217E-05	0.038206292
707.279989	2052.138903	4.63139E-05	0.032756906
707.319989	1639.066448	3.69914E-05	0.02616479
707.359989	1239.932551	2.79836E-05	0.019794444
707.399989	914.602212	2.06413E-05	0.014601654
707.439989	652.704649	1.47306E-05	0.01042104
707.479989	480.828166	1.08516E-05	0.007677305
707.519989	401.969632	9.07189E-06	0.006418547

707.559989	372.911445	8.41609E-06	0.00595489
707.599989	361.16027	8.15088E-06	0.005767566
707.639989	383.105971	8.64617E-06	0.006118374
707.679989	419.18614	9.46045E-06	0.006694969
707.719989	419.483898	9.46717E-06	0.006700104
707.759989	398.279191	8.98861E-06	0.006361776
707.799989	358.370209	8.08792E-06	0.005724627
707.839989	322.261104	7.27298E-06	0.005148109
707.879989	291.722398	6.58377E-06	0.004660518
707.919989	314.342039	7.09426E-06	0.00502217
707.959989	388.675021	8.77185E-06	0.006210122
707.999989	498.789328	1.1257E-05	0.007969942
708.039989	692.697447	1.56332E-05	0.011068942
708.079989	946.714284	2.1366E-05	0.015128853
708.119989	1263.800305	2.85222E-05	0.020197149
708.159989	1587.399389	3.58254E-05	0.02537011
708.199989	1718.978728	3.8795E-05	0.027474587
708.239989	1668.126353	3.76473E-05	0.026663315
708.279989	1452.642421	3.27841E-05	0.023220333
708.319989	1160.725715	2.6196E-05	0.018555124
708.359989	915.635646	2.06646E-05	0.014637991
708.399989	711.404161	1.60554E-05	0.011373646
708.439989	569.171411	1.28454E-05	0.009100199
708.479989	463.77514	1.04668E-05	0.007415489
708.519989	374.911773	8.46124E-06	0.005994955
708.559989	341.57808	7.70894E-06	0.005462247
708.599989	338.906298	7.64864E-06	0.005419828
708.639989	366.958975	8.28175E-06	0.005868781
708.679989	416.927141	9.40946E-06	0.006668299
708.719989	452.063121	1.02024E-05	0.00723067
708.759989	440.233552	9.93546E-06	0.007041855
708.799989	409.660251	9.24546E-06	0.006553183
708.839989	367.336915	8.29028E-06	0.005876484
708.879989	332.179075	7.49682E-06	0.005314345
708.919989	353.520904	7.97847E-06	0.0056561
708.959989	427.056289	9.63807E-06	0.006833003
708.999989	605.964343	1.36758E-05	0.009696122
709.039989	971.419669	2.19236E-05	0.015544701
709.079989	1447.494354	3.26679E-05	0.023164176
709.119989	1890.107703	4.26571E-05	0.030249001
709.159989	2282.282649	5.15079E-05	0.03652737
709.199989	2496.061819	5.63326E-05	0.039951107
709.239989	2463.939445	5.56077E-05	0.039439191
709.279989	2228.314238	5.02899E-05	0.035669654
709.319989	1808.863017	4.08235E-05	0.028956938
709.359989	1348.74275	3.04392E-05	0.021592383

709.399989	956.834808	2.15944E-05	0.015319088
709.439989	656.725742	1.48214E-05	0.010514883
709.479989	460.121771	1.03843E-05	0.007367458
709.519989	357.11451	8.05958E-06	0.005718431
709.559989	330.671908	7.4628E-06	0.005295307
709.599989	353.129812	7.96965E-06	0.005655262
709.639989	409.008317	9.23075E-06	0.006550508
709.679989	452.369004	1.02093E-05	0.007245363
709.719989	419.426009	9.46586E-06	0.006718111
709.759989	369.006545	8.32796E-06	0.005910855
709.799989	328.824087	7.4211E-06	0.005267498
709.839989	286.631526	6.46887E-06	0.004591866
709.879989	278.549802	6.28648E-06	0.004462647
709.919989	307.727247	6.94498E-06	0.004930377
709.959989	389.411904	8.78848E-06	0.006239472
709.999989	496.140376	1.11972E-05	0.00795001
710.039989	661.30107	1.49246E-05	0.010597094
710.079989	923.588211	2.08441E-05	0.014800978
710.119989	1265.944747	2.85706E-05	0.020288561
710.159989	1548.711195	3.49523E-05	0.024821693
710.199989	1654.98813	3.73508E-05	0.026526521
710.239989	1654.226547	3.73336E-05	0.026515808
710.279989	1550.094708	3.49835E-05	0.024848065
710.319989	1340.528233	3.02539E-05	0.021489918
710.359989	1073.39484	2.4225E-05	0.01720849
710.399989	818.398349	1.84701E-05	0.013121167
710.439989	590.02803	1.33161E-05	0.009460298
710.479989	420.054686	9.48005E-06	0.006735385
710.519989	357.504856	8.06839E-06	0.00573275
710.559989	348.118295	7.85655E-06	0.005582547
710.599989	370.276617	8.35663E-06	0.005938219
710.639989	411.4584	9.28604E-06	0.006599033
710.679989	449.537765	1.01454E-05	0.007210162
710.719989	473.527962	1.06869E-05	0.00759537
710.759989	472.176657	1.06564E-05	0.007574121
710.799989	427.153165	9.64025E-06	0.006852291
710.839989	388.626761	8.77076E-06	0.00623461
710.879989	375.826377	8.48188E-06	0.006029597
710.919989	411.089974	9.27773E-06	0.006595722
710.959989	522.752258	1.17978E-05	0.008387757
710.999989	669.764606	1.51157E-05	0.01074723
711.039989	961.581901	2.17016E-05	0.01543068
711.079989	1328.434733	2.99809E-05	0.021318833
711.119989	1748.46392	3.94604E-05	0.028061076
711.159989	2162.527719	4.88052E-05	0.034708333
711.199989	2388.496604	5.3905E-05	0.038337265

711.239989	2351.245108	5.30643E-05	0.037741471
711.279989	2075.215723	4.68347E-05	0.033312605
711.319989	1659.480262	3.74522E-05	0.026640469
711.359989	1226.687063	2.76846E-05	0.01969373
711.399989	875.792035	1.97654E-05	0.01406111
711.439989	641.576481	1.44795E-05	0.010301286
711.479989	481.093665	1.08576E-05	0.007724975
711.519989	374.502837	8.45201E-06	0.006013772
711.559989	321.243954	7.25003E-06	0.00515883
711.599989	319.609317	7.21314E-06	0.005132868
711.639989	371.763325	8.39018E-06	0.005970788
711.679989	417.935209	9.43222E-06	0.006712719
711.719989	409.676635	9.24583E-06	0.006580443
711.759989	416.312688	9.3956E-06	0.00668741
711.799989	398.93521	9.00341E-06	0.006408629
711.839989	328.962832	7.42423E-06	0.005284866
711.879989	290.382775	6.55353E-06	0.00466533
711.919989	287.054215	6.47841E-06	0.004612112
711.959989	343.099424	7.74328E-06	0.005512903
711.999989	469.638505	1.05991E-05	0.00754655
712.039989	635.681616	1.43464E-05	0.010215245
712.079989	891.332886	2.01161E-05	0.014324302
712.119989	1239.850479	2.79817E-05	0.019926327
712.159989	1451.072649	3.27487E-05	0.023322306
712.199989	1540.262563	3.47616E-05	0.024757197
712.239989	1579.565214	3.56486E-05	0.025390349
712.279989	1466.986715	3.31078E-05	0.023582057
712.319989	1235.21303	2.7877E-05	0.019857371
712.359989	999.683147	2.25615E-05	0.016071879
712.399989	795.82171	1.79606E-05	0.012795122
712.439989	610.277269	1.37731E-05	0.009812513
712.479989	454.675024	1.02614E-05	0.007311029
712.519989	365.097229	8.23974E-06	0.005870977
712.559989	335.199236	7.56498E-06	0.005390502
712.599989	356.395974	8.04336E-06	0.005731699
712.639989	414.976211	9.36544E-06	0.006674184
712.679989	454.817515	1.02646E-05	0.007315373
712.719989	459.920335	1.03798E-05	0.007397863
712.759989	431.513545	9.73866E-06	0.006941327
712.799989	411.387609	9.28445E-06	0.006617953
712.839989	401.956519	9.0716E-06	0.006466598
712.879989	414.91989	9.36416E-06	0.006675525
712.919989	422.640122	9.5384E-06	0.006800115
712.959989	472.190637	1.06567E-05	0.00759779
712.999989	619.55306	1.39824E-05	0.009969486
713.039989	884.050919	1.99518E-05	0.01422643

713.079989	1233.861592	2.78465E-05	0.019856809
713.119989	1574.208046	3.55277E-05	0.025335501
713.159989	1859.574447	4.1968E-05	0.029929902
713.199989	2014.769996	4.54706E-05	0.032429598
713.239989	2024.55363	4.56914E-05	0.032588902
713.279989	1843.609611	4.16077E-05	0.029677941
713.319989	1528.957774	3.45064E-05	0.024614139
713.359989	1181.974034	2.66755E-05	0.01902924
713.399989	875.571761	1.97604E-05	0.014097094
713.439989	650.859365	1.4689E-05	0.010479712
713.479989	477.199466	1.07697E-05	0.007683985
713.519989	364.747067	8.23183E-06	0.005873578
713.559989	335.015892	7.56084E-06	0.005395114
713.599989	334.724757	7.55427E-06	0.005390728
713.639989	354.847556	8.00842E-06	0.005715125
713.679989	375.583461	8.4764E-06	0.006049434
713.719989	387.930773	8.75506E-06	0.006248659
713.759989	379.67091	8.56864E-06	0.006115955
713.799989	365.548989	8.24993E-06	0.005888801
713.839989	351.599393	7.93511E-06	0.005664398
713.879989	297.305161	6.70976E-06	0.004789966
713.919989	282.109458	6.36682E-06	0.004545398
713.959989	319.444394	7.20942E-06	0.005147234
713.999989	446.254035	1.00713E-05	0.007190931
714.039989	664.822438	1.50041E-05	0.010713539
714.079989	934.368479	2.10874E-05	0.015058087
714.119989	1190.297727	2.68634E-05	0.019183664
714.159989	1444.848005	3.26082E-05	0.023287477
714.199989	1623.300069	3.66356E-05	0.02616516
714.239989	1611.49339	3.63692E-05	0.025976309
714.279989	1479.843202	3.3398E-05	0.023855523
714.319989	1270.762214	2.86793E-05	0.020486221
714.359989	1025.8988	2.31531E-05	0.016539653
714.399989	816.699087	1.84318E-05	0.01316765
714.439989	615.485463	1.38906E-05	0.009924036
714.479989	441.656723	9.96758E-06	0.007121634
714.519988	363.717273	8.20859E-06	0.005865203
714.559988	369.678035	8.34312E-06	0.005961659
714.599988	408.578594	9.22105E-06	0.006589362
714.639988	452.596542	1.02145E-05	0.007299671
714.679988	471.076209	1.06315E-05	0.007598145
714.719988	477.320033	1.07724E-05	0.007699284
714.759988	452.829608	1.02197E-05	0.007304657
714.799988	427.97277	9.65875E-06	0.006904074
714.839988	410.210742	9.25789E-06	0.006617906
714.879988	404.661716	9.13265E-06	0.00652875

714.919988	464.489238	1.04829E-05	0.007494417
714.959988	535.018754	1.20746E-05	0.008632876
714.999988	731.044633	1.64987E-05	0.011796541
715.039988	1060.950093	2.39442E-05	0.017121036
715.079988	1458.534035	3.29171E-05	0.023538346
715.119988	1863.598746	4.20588E-05	0.030077109
715.159988	2185.48948	4.93235E-05	0.03527416
715.199988	2294.555289	5.17849E-05	0.037036572
715.239988	2234.038092	5.04191E-05	0.036061776
715.279988	2073.177066	4.67887E-05	0.033467034
715.319988	1751.255774	3.95234E-05	0.028271881
715.359988	1362.296435	3.07451E-05	0.021993838
715.399988	1005.498432	2.26927E-05	0.016234357
715.439988	713.632863	1.61057E-05	0.011522662
715.479988	512.043042	1.15561E-05	0.008268157
715.519988	390.176829	8.80575E-06	0.006300688
715.559988	338.521138	7.63995E-06	0.005466843
715.599988	326.268757	7.36343E-06	0.005269271
715.639988	350.928695	7.91997E-06	0.005667849
715.679988	360.901522	8.14504E-06	0.005829245
715.719988	350.717748	7.91521E-06	0.005665075
715.759988	324.266195	7.31824E-06	0.005238101
715.799988	327.500263	7.39122E-06	0.005290639
715.839988	306.487685	6.917E-06	0.004951465
715.879988	282.675147	6.37958E-06	0.004567017
715.919988	287.194906	6.48159E-06	0.004640299
715.959988	324.818314	7.3307E-06	0.005248486
715.999988	424.741805	9.58583E-06	0.006863455
716.039988	610.304066	1.37737E-05	0.009862529
716.079988	893.505064	2.01652E-05	0.014439871
716.119988	1229.165945	2.77406E-05	0.019865572
716.159988	1477.758513	3.3351E-05	0.023884617
716.199988	1618.043977	3.6517E-05	0.026153474
716.239988	1612.470766	3.63912E-05	0.026064846
716.279988	1454.400485	3.28238E-05	0.023511026
716.319988	1199.327914	2.70672E-05	0.019388748
716.359988	908.046289	2.04933E-05	0.014680609
716.399988	697.71686	1.57465E-05	0.011280791
716.439988	554.736192	1.25196E-05	0.008969559
716.479988	418.374686	9.44213E-06	0.0067651
716.519988	349.310755	7.88346E-06	0.005648655
716.559988	373.53876	8.43025E-06	0.006040779
716.599988	416.610685	9.40232E-06	0.006737704
716.639988	417.474855	9.42183E-06	0.006752057
716.679988	412.443308	9.30827E-06	0.006671052
716.719988	407.493577	9.19656E-06	0.00659136

716.759988	402.677027	9.08786E-06	0.006513814
716.799988	370.232131	8.35562E-06	0.005989311
716.839988	316.03365	7.13244E-06	0.005112818
716.879988	318.28531	7.18326E-06	0.005149533
716.919988	386.770933	8.72888E-06	0.006257909
716.959988	490.276422	1.10649E-05	0.007933059
716.999988	638.761466	1.4416E-05	0.010336241
717.039988	901.5921	2.03477E-05	0.014590099
717.079988	1271.716612	2.87009E-05	0.020580821
717.119988	1680.951716	3.79367E-05	0.027205194
717.159988	2039.907194	4.60379E-05	0.033016514
717.199988	2118.150254	4.78037E-05	0.034284814
717.239988	2006.495578	4.52838E-05	0.03247936
717.279988	1865.236592	4.20958E-05	0.030194469
717.319988	1660.148777	3.74672E-05	0.026876004
717.359988	1338.968602	3.02187E-05	0.021677655
717.399988	1026.299524	2.31622E-05	0.016616527
717.439988	739.41104	1.66875E-05	0.011972263
717.479988	514.43185	1.161E-05	0.00832995
717.519988	399.748806	9.02177E-06	0.006473303
717.559988	375.808336	8.48147E-06	0.006085964
717.599988	368.765246	8.32252E-06	0.005972239
717.639988	386.039651	8.71238E-06	0.00625235
717.679988	418.689507	9.44924E-06	0.00678153
717.719988	431.446936	9.73716E-06	0.006988552
717.759988	404.957159	9.13932E-06	0.006559838
717.799988	384.153255	8.6698E-06	0.006223185
717.839988	363.373076	8.20082E-06	0.00588688
717.879988	300.984258	6.7928E-06	0.004876412
717.919988	284.628989	6.42368E-06	0.004611688
717.959988	325.573091	7.34773E-06	0.005275377
717.999988	407.771924	9.20284E-06	0.006607642
718.039988	582.971524	1.31569E-05	0.009447148
718.079988	817.573735	1.84515E-05	0.013249653
718.119988	1092.339372	2.46526E-05	0.017703509
718.159988	1321.788215	2.98309E-05	0.021423372
718.199988	1442.481095	3.25548E-05	0.023380849
718.239988	1460.070874	3.29518E-05	0.023667276
718.279988	1407.497213	3.17653E-05	0.022816345
718.319988	1298.735435	2.93106E-05	0.021054426
718.359988	1123.801768	2.53626E-05	0.018219506
718.399988	864.437446	1.95091E-05	0.014015372
718.439988	598.411707	1.35053E-05	0.009702761
718.479988	413.828816	9.33954E-06	0.006710273
718.519988	327.954916	7.40149E-06	0.005318115
718.559988	324.7784	7.3298E-06	0.005266898

718.599988	366.523838	8.27193E-06	0.005944211
718.639988	406.110336	9.16534E-06	0.006586583
718.679988	424.693145	9.58473E-06	0.006888356
718.719988	425.914754	9.6123E-06	0.006908554
718.759988	422.220206	9.52892E-06	0.006849008
718.799988	402.617645	9.08652E-06	0.00653139
718.839988	371.33087	8.38042E-06	0.006024181
718.879988	342.446677	7.72854E-06	0.005555896
718.919988	368.918035	8.32597E-06	0.005985704
718.959988	471.891597	1.06499E-05	0.007656878
718.999988	637.201003	1.43807E-05	0.010339751
719.039988	970.725606	2.19079E-05	0.015752674
719.079988	1385.173609	3.12614E-05	0.022479475
719.119988	1812.227249	4.08994E-05	0.029411608
719.159988	2118.353637	4.78083E-05	0.03438181
719.199988	2203.780948	4.97363E-05	0.035770322
719.239988	2112.061089	4.76663E-05	0.034283492
719.279988	1858.072194	4.19341E-05	0.03016236
719.319988	1523.224199	3.4377E-05	0.024728098
719.359988	1180.771313	2.66484E-05	0.019169767
719.399988	849.014237	1.91611E-05	0.013784473
719.439988	579.705673	1.30831E-05	0.009412541
719.479988	429.375408	9.6904E-06	0.006972052
719.519988	361.35861	8.15536E-06	0.005867945
719.559988	342.155504	7.72197E-06	0.005556423
719.599988	360.400674	8.13374E-06	0.00585304
719.639988	381.117962	8.6013E-06	0.006189841
719.679988	390.126266	8.80461E-06	0.006336499
719.719988	390.310304	8.80876E-06	0.006339841
719.759988	362.366984	8.17812E-06	0.005886282
719.799988	333.749421	7.53226E-06	0.00542172
719.839988	329.40835	7.43429E-06	0.005351498
719.879988	322.655819	7.28189E-06	0.005242089
719.919988	296.950434	6.70176E-06	0.004824729
719.959988	306.547158	6.91834E-06	0.00498093
719.999988	396.068148	8.93871E-06	0.006435869
720.039988	564.417491	1.27381E-05	0.009171953
720.079988	826.668655	1.86568E-05	0.01343436
720.119988	1156.132615	2.60923E-05	0.018789589
720.159988	1378.83946	3.11185E-05	0.022410289
720.199988	1433.584628	3.2354E-05	0.023301356
720.239988	1390.113471	3.13729E-05	0.022596035
720.279988	1315.74429	2.96945E-05	0.021388366
720.319988	1138.660402	2.5698E-05	0.018510768
720.359988	885.050461	1.99744E-05	0.014388727
720.399988	692.14084	1.56207E-05	0.011253119

720.439988	510.838075	1.15289E-05	0.008305883
720.479988	385.980059	8.71103E-06	0.006276124
720.519988	327.609005	7.39368E-06	0.005327293
720.559988	319.8171	7.21783E-06	0.005200877
720.599988	374.128878	8.44357E-06	0.006084435
720.639988	423.883912	9.56647E-06	0.00689398
720.679988	461.772633	1.04216E-05	0.007510613
720.719988	460.032052	1.03823E-05	0.007482719
720.759988	432.743112	9.76641E-06	0.007039237
720.799988	404.336004	9.1253E-06	0.006577516
720.839988	377.53288	8.52039E-06	0.006141839
720.879988	392.128513	8.84979E-06	0.00637964
720.919988	440.77485	9.94767E-06	0.007171477
720.959988	513.445293	1.15877E-05	0.008354301
720.999988	673.846957	1.52078E-05	0.010964814
721.039988	1022.374238	2.30736E-05	0.016636961
721.079988	1500.337441	3.38605E-05	0.024416148
721.119988	1963.613139	4.4316E-05	0.031957163
721.159988	2305.527292	5.20325E-05	0.037523786
721.199988	2363.975208	5.33516E-05	0.038477193
721.239988	2161.987546	4.8793E-05	0.035191497
721.279988	1875.655415	4.23309E-05	0.030532453
721.319988	1549.632008	3.4973E-05	0.02522675
721.359988	1173.217438	2.64779E-05	0.019100086
721.399988	822.551019	1.85638E-05	0.013391947
721.439988	567.005016	1.27965E-05	0.009231917
721.479988	414.094552	9.34554E-06	0.006742618
721.519988	335.193199	7.56484E-06	0.005458186
721.559988	336.670414	7.59818E-06	0.005482544
721.599988	379.465859	8.56402E-06	0.006179794
721.639988	403.665996	9.11018E-06	0.00657427
721.679988	411.545962	9.28802E-06	0.006702978
721.719988	391.823364	8.84291E-06	0.006382103
721.759988	362.224357	8.1749E-06	0.005900315
721.799988	336.663848	7.59803E-06	0.005484261
721.839988	290.55798	6.55749E-06	0.004733458
721.879988	276.497261	6.24016E-06	0.004504645
721.919988	304.604657	6.8745E-06	0.004962841
721.959988	355.124662	8.01467E-06	0.00578627
721.999988	510.404987	1.15191E-05	0.008316811
722.039988	759.203068	1.71342E-05	0.012371546
722.079988	1053.755433	2.37818E-05	0.017172356
722.119988	1343.406886	3.03188E-05	0.021893828
722.159988	1599.320899	3.60944E-05	0.026065964
722.199988	1663.412228	3.75409E-05	0.027112035
722.239988	1527.316001	3.44694E-05	0.024895175

722.279988	1300.133384	2.93422E-05	0.021193284
722.319988	1063.862902	2.40099E-05	0.017342833
722.359988	845.785584	1.90882E-05	0.013788554
722.399988	650.887847	1.46896E-05	0.01061179
722.439988	475.108931	1.07225E-05	0.007746397
722.479988	361.160697	8.15089E-06	0.005888858
722.519988	322.781492	7.28473E-06	0.005263362
722.559988	326.057975	7.35867E-06	0.005317084
722.599988	351.889885	7.94166E-06	0.005738647
722.639988	359.254359	8.10787E-06	0.005859071
722.679988	371.924312	8.39381E-06	0.006066041
722.719988	409.11162	9.23308E-06	0.006672931
722.759988	403.791013	9.113E-06	0.006586512
722.799988	388.939606	8.77783E-06	0.006344612
722.839988	395.556554	8.92716E-06	0.006452909
722.879988	396.219024	8.94211E-06	0.006464073
722.919988	413.942675	9.34211E-06	0.006753598
722.959988	508.031296	1.14656E-05	0.00828914
722.999988	716.038232	1.616E-05	0.011683669
723.039988	1079.197841	2.4356E-05	0.017610356
723.079988	1527.573186	3.44752E-05	0.024928327
723.119988	1952.972438	4.40759E-05	0.031872141
723.159988	2297.965337	5.18619E-05	0.037504434
723.199988	2400.413075	5.4174E-05	0.039178621
723.239988	2270.975001	5.12527E-05	0.037068033
723.279988	1958.867232	4.42089E-05	0.031975416
723.319988	1545.44798	3.48786E-05	0.025228395
723.359988	1176.426884	2.65503E-05	0.019205436
723.399988	852.853478	1.92477E-05	0.013923797
723.439988	577.498705	1.30333E-05	0.00942884
723.479988	394.013948	8.89235E-06	0.006433434
723.519988	318.07578	7.17853E-06	0.005193808
723.559988	330.986829	7.46991E-06	0.005404929
723.599988	368.88683	8.32526E-06	0.006024159
723.639988	388.026161	8.75721E-06	0.006337067
723.679988	399.130916	9.00783E-06	0.006518785
723.719988	387.433103	8.74383E-06	0.006328081
723.759988	366.87374	8.27983E-06	0.005992609
723.799988	342.130757	7.72141E-06	0.00558876
723.839988	307.284995	6.93499E-06	0.005019826
723.879988	295.719193	6.67397E-06	0.004831154
723.919988	320.922316	7.24277E-06	0.005243186
723.959988	389.626958	8.79334E-06	0.006366025
723.999988	512.8824	1.1575E-05	0.00838033
724.039988	690.122954	1.55751E-05	0.011277005
724.079988	901.830247	2.03531E-05	0.014737238

724.119988	1154.757553	2.60613E-05	0.018871486
724.159988	1374.210948	3.1014E-05	0.022459117
724.199988	1454.549099	3.28271E-05	0.02377342
724.239988	1432.206353	3.23229E-05	0.023409539
724.279988	1329.469581	3.00043E-05	0.021731497
724.319988	1164.199177	2.62744E-05	0.01903104
724.359988	950.047141	2.14412E-05	0.015531177
724.399988	717.431689	1.61914E-05	0.011729075
724.439988	522.290232	1.17874E-05	0.008539237
724.479988	401.601592	9.06359E-06	0.006566389
724.519988	334.533507	7.54996E-06	0.005470094
724.559988	324.725661	7.32861E-06	0.005310015
724.599988	358.720084	8.09581E-06	0.005866226
724.639988	405.585339	9.1535E-06	0.006632989
724.679988	425.750173	9.60859E-06	0.006963152
724.719988	423.139666	9.54967E-06	0.006920839
724.759988	405.479975	9.15112E-06	0.006632364
724.799988	370.393317	8.35926E-06	0.006058792
724.839988	346.042316	7.80969E-06	0.005660778
724.879988	340.418551	7.68277E-06	0.005569088
724.919988	370.672569	8.36556E-06	0.006064364
724.959988	464.87603	1.04916E-05	0.007605993
724.999988	639.971282	1.44433E-05	0.010471364
725.039988	919.81759	2.0759E-05	0.015051106
725.079988	1280.359822	2.88959E-05	0.020951866
725.119988	1690.051256	3.81421E-05	0.027657601
725.159988	2012.076692	4.54098E-05	0.032929347
725.199988	2108.912523	4.75952E-05	0.034516052
725.239988	1934.931155	4.36687E-05	0.031670288
725.279988	1687.879016	3.80931E-05	0.027628147
725.319988	1417.876997	3.19995E-05	0.023209883
725.359988	1078.654197	2.43437E-05	0.017657962
725.399988	787.404145	1.77706E-05	0.012890804
725.439988	561.024579	1.26615E-05	0.00918519
725.479988	405.066844	9.14179E-06	0.006632189
725.519988	348.678924	7.8692E-06	0.00570926
725.559988	364.132749	8.21797E-06	0.005962629
725.599988	394.925655	8.91292E-06	0.006467216
725.639988	409.919843	9.25132E-06	0.006713128
725.679988	435.44144	9.82731E-06	0.00713148
725.719988	405.327354	9.14767E-06	0.00663865
725.759988	376.468577	8.49637E-06	0.006166326
725.799988	346.683741	7.82417E-06	0.005678782
725.839988	306.982997	6.92818E-06	0.005028749
725.879988	267.218289	6.03074E-06	0.004377597
725.919988	268.211198	6.05315E-06	0.004394105

725.959988	320.386297	7.23067E-06	0.005249179
725.999988	413.693526	9.33649E-06	0.006778289
726.039988	576.317315	1.30067E-05	0.009443369
726.079988	827.861814	1.86837E-05	0.013565852
726.119988	1101.561153	2.48607E-05	0.018051852
726.159988	1301.580248	2.93749E-05	0.021330843
726.199988	1380.838302	3.11636E-05	0.022631004
726.239988	1356.672632	3.06182E-05	0.02223617
726.279988	1244.993789	2.80978E-05	0.020406852
726.319988	1077.030159	2.43071E-05	0.017654711
726.359988	885.796468	1.99912E-05	0.014520802
726.399988	678.258145	1.53073E-05	0.011119253
726.439988	503.09489	1.13542E-05	0.008248109
726.479988	372.415037	8.40489E-06	0.006105983
726.519988	310.146031	6.99956E-06	0.005085323
726.559988	311.164764	7.02256E-06	0.005102308
726.599988	353.183288	7.97085E-06	0.005791623
726.639988	404.737079	9.13435E-06	0.006637386
726.679988	426.670722	9.62936E-06	0.006997466
726.719988	413.002492	9.32089E-06	0.006773678
726.759988	430.303495	9.71135E-06	0.007057821
726.799988	430.021558	9.70499E-06	0.007053585
726.839988	384.751757	8.68331E-06	0.006311378
726.879988	348.080165	7.85568E-06	0.00571014
726.919988	381.130474	8.60158E-06	0.006252663
726.959988	452.655845	1.02158E-05	0.007426487
726.999988	569.81087	1.28598E-05	0.009349102
727.039988	791.448187	1.78619E-05	0.012986303
727.079988	1138.464039	2.56935E-05	0.018681264
727.119988	1582.182137	3.57076E-05	0.025963744
727.159988	1989.657246	4.49038E-05	0.032652241
727.199988	2130.658846	4.8086E-05	0.03496814
727.239988	2044.325163	4.61376E-05	0.033553087
727.279988	1786.057301	4.03088E-05	0.029315802
727.319988	1399.072826	3.15751E-05	0.022965219
727.359988	1029.860499	2.32425E-05	0.016905677
727.399988	753.796995	1.70121E-05	0.012374636
727.439988	561.119386	1.26637E-05	0.009212069
727.479988	429.714939	9.69807E-06	0.00705515
727.519988	361.557649	8.15985E-06	0.005936456
727.559988	340.109841	7.67581E-06	0.005584609
727.599988	364.951083	8.23644E-06	0.005992832
727.639988	401.428081	9.05967E-06	0.00659218
727.679988	433.097125	9.7744E-06	0.007112634
727.719988	418.447472	9.44378E-06	0.006872425
727.759988	380.107215	8.57849E-06	0.006243082

727.799988	327.820285	7.39845E-06	0.00538459
727.839988	296.610153	6.69408E-06	0.004872218
727.879988	291.537847	6.5796E-06	0.004789161
727.919988	309.945076	6.99503E-06	0.005091821
727.959988	360.780268	8.14231E-06	0.005927275
727.999988	445.275367	1.00492E-05	0.00731585
728.039988	601.712047	1.35798E-05	0.009886639
728.079988	839.506458	1.89465E-05	0.013794561
728.119988	1076.125765	2.42867E-05	0.017683602
728.159988	1304.982462	2.94516E-05	0.021445503
728.199988	1396.550175	3.15182E-05	0.022951547
728.239988	1379.419911	3.11316E-05	0.022671266
728.279988	1251.962084	2.8255E-05	0.02057758
728.319988	1064.364515	2.40212E-05	0.017495138
728.359988	871.757221	1.96743E-05	0.014330007
728.399988	665.590269	1.50214E-05	0.010941621
728.439988	487.170873	1.09948E-05	0.008009029
728.479988	378.794131	8.54886E-06	0.00622767
728.519988	342.791894	7.73634E-06	0.005636075
728.559988	374.971483	8.46258E-06	0.0061655
728.599988	395.050586	8.91574E-06	0.006496009
728.639988	380.726592	8.59247E-06	0.006260816
728.679988	398.896578	9.00254E-06	0.006559971
728.719988	454.567141	1.02589E-05	0.0074759
728.759988	433.132936	9.77521E-06	0.00712378
728.799988	402.667098	9.08764E-06	0.006623069
728.839988	382.12513	8.62403E-06	0.006285539
728.879988	365.101068	8.23982E-06	0.006005842
728.919988	406.570051	9.17572E-06	0.006688366
728.959988	554.499675	1.25143E-05	0.009122413
728.999988	798.42448	1.80193E-05	0.01313609
729.039988	1121.16556	2.53031E-05	0.018447005
729.079988	1506.553142	3.40008E-05	0.024789307
729.119988	1863.803539	4.20634E-05	0.030669302
729.159988	2082.313895	4.69949E-05	0.034266818
729.199988	2180.059139	4.92009E-05	0.035877294
729.239988	2141.83795	4.83383E-05	0.035250221
729.279988	1857.394043	4.19188E-05	0.030570539
729.319988	1503.557034	3.39332E-05	0.024748152
729.359988	1177.905437	2.65837E-05	0.019389076
729.399988	883.251864	1.99338E-05	0.014539687
729.439988	613.327885	1.3842E-05	0.010096876
729.479988	407.747044	9.20228E-06	0.006712881
729.519988	303.662001	6.85323E-06	0.004999567
729.559988	301.278846	6.79944E-06	0.004960602
729.599988	336.404072	7.59217E-06	0.005539248

729.639988	395.257013	8.9204E-06	0.006508681
729.679988	455.311631	1.02757E-05	0.007498009
729.719988	456.739916	1.0308E-05	0.007521942
729.759988	414.945991	9.36475E-06	0.006834022
729.799988	380.507613	8.58753E-06	0.006267177
729.839988	354.56544	8.00205E-06	0.005840215
729.879988	317.179258	7.15829E-06	0.005224696
729.919988	334.964693	7.55969E-06	0.005517966
729.959988	415.662525	9.38092E-06	0.006847699
729.999988	502.439147	1.13394E-05	0.008277727
730.039988	688.934228	1.55483E-05	0.01135087
730.079988	944.224228	2.13098E-05	0.015557877
730.119988	1218.507466	2.75E-05	0.020078312
730.159988	1480.687211	3.3417E-05	0.024399791
730.199988	1556.902362	3.51371E-05	0.025657123
730.239988	1501.490552	3.38866E-05	0.024745314
730.279988	1361.908243	3.07364E-05	0.022446157
730.319988	1170.585377	2.64185E-05	0.019293945
730.359988	922.324566	2.08156E-05	0.015202867
730.399988	688.328227	1.55346E-05	0.011346478
730.439988	487.388066	1.09997E-05	0.008034599
730.479988	341.162542	7.69956E-06	0.005624377
730.519988	265.232173	5.98592E-06	0.004372835
730.559988	263.155742	5.93906E-06	0.004338839
730.599988	313.192174	7.06831E-06	0.005164108
730.639988	385.681441	8.70429E-06	0.006359704
730.679988	463.522123	1.0461E-05	0.007643679
730.719988	495.785205	1.11892E-05	0.008176159
730.759988	453.79031	1.02414E-05	0.007484016
730.799988	381.390147	8.60744E-06	0.00629032
730.839988	335.96645	7.58229E-06	0.005541444
730.879988	346.446682	7.81882E-06	0.005714618
730.919988	373.734259	8.43466E-06	0.006165063
730.959988	457.87318	1.03336E-05	0.007553419
730.999988	686.634437	1.54964E-05	0.011327856
731.039988	1054.784563	2.3805E-05	0.01740242
731.079988	1444.240316	3.25945E-05	0.023829181
731.119988	1760.934474	3.97418E-05	0.029056052
731.159988	1998.553248	4.51046E-05	0.032978652
731.199988	2090.063725	4.71698E-05	0.034490577
731.239988	1991.985627	4.49563E-05	0.032873874
731.279988	1773.029665	4.00148E-05	0.02926203
731.319988	1465.651273	3.30777E-05	0.024190389
731.359988	1153.899357	2.60419E-05	0.019046005
731.399988	898.055146	2.02679E-05	0.014823908
731.439988	659.144501	1.4876E-05	0.010880882

731.479988	465.660124	1.05093E-05	0.007687343
731.519988	355.982218	8.03402E-06	0.005877048
731.559988	337.337338	7.61323E-06	0.005569537
731.599988	382.404072	8.63033E-06	0.006313947
731.639988	402.295916	9.07926E-06	0.006642749
731.679988	400.938196	9.04862E-06	0.006620692
731.719988	388.805297	8.77479E-06	0.006420692
731.759988	376.571716	8.4987E-06	0.006219008
731.799988	357.047861	8.05807E-06	0.005896898
731.839988	313.148272	7.06732E-06	0.005172148
731.879988	296.933164	6.70137E-06	0.004904597
731.919988	331.770766	7.4876E-06	0.005480327
731.959988	412.385273	9.30696E-06	0.006812323
731.999988	526.236918	1.18764E-05	0.00869355
732.039988	691.666352	1.56099E-05	0.011427105
732.079988	914.193791	2.06321E-05	0.015104333
732.119988	1192.847327	2.69209E-05	0.019709331
732.159988	1413.384535	3.18981E-05	0.023354527
732.199988	1470.439945	3.31858E-05	0.024298629
732.239988	1390.331078	3.13778E-05	0.022976106
732.279988	1241.182922	2.80118E-05	0.020512458
732.319988	1092.172718	2.46488E-05	0.018050821
732.359988	930.595246	2.10022E-05	0.015381199
732.399988	723.879635	1.6337E-05	0.011965186
732.439988	547.295205	1.23517E-05	0.009046872
732.479988	423.495698	9.55771E-06	0.00700083
732.519988	359.387925	8.11088E-06	0.005941385
732.559988	352.686642	7.95965E-06	0.005830918
732.599988	376.145773	8.48909E-06	0.006219104
732.639988	400.94246	9.04871E-06	0.006629449
732.679988	437.920103	9.88325E-06	0.007241257
732.719988	451.313302	1.01855E-05	0.007463129
732.759988	415.399783	9.37499E-06	0.006869621
732.799988	397.033223	8.96049E-06	0.006566245
732.839988	383.277804	8.65005E-06	0.0063391
732.879988	381.634477	8.61296E-06	0.006312265
732.919988	408.580498	9.22109E-06	0.006758323
732.959988	516.65704	1.16602E-05	0.008546482
732.999988	756.835636	1.70807E-05	0.012520172
733.039988	1161.586507	2.62154E-05	0.019216929
733.079988	1644.673386	3.7118E-05	0.027210454
733.119988	2098.427254	4.73586E-05	0.034719522
733.159988	2394.852949	5.40485E-05	0.039626194
733.199988	2382.567447	5.37712E-05	0.039425064
733.239988	2163.19779	4.88204E-05	0.035797041
733.279988	1878.719841	4.24001E-05	0.031091137

733.319988	1550.57836	3.49944E-05	0.025662088
733.359988	1188.056429	2.68128E-05	0.019663419
733.399988	857.788141	1.93591E-05	0.014197952
733.439988	597.170109	1.34773E-05	0.009884789
733.479988	421.100966	9.50366E-06	0.006970746
733.519988	329.098786	7.4273E-06	0.005448074
733.559988	326.982112	7.37953E-06	0.005413329
733.599988	379.218769	8.55844E-06	0.006278471
733.639988	428.257577	9.66518E-06	0.00709076
733.679988	434.423296	9.80433E-06	0.00719324
733.719988	402.836873	9.09147E-06	0.006670591
733.759988	358.053752	8.08077E-06	0.005929349
733.799988	331.045144	7.47123E-06	0.005482387
733.839988	311.536164	7.03094E-06	0.005159583
733.879988	323.279563	7.29597E-06	0.005354366
733.919988	354.102733	7.99161E-06	0.005865199
733.959988	398.486178	8.99328E-06	0.006600706
733.999988	504.786837	1.13923E-05	0.008361974
734.039988	700.17817	1.5802E-05	0.011599334
734.079988	975.291402	2.2011E-05	0.016157811
734.119988	1287.119073	2.90485E-05	0.021325073
734.159988	1504.587144	3.39564E-05	0.024929457
734.199988	1586.373814	3.58022E-05	0.02628601
734.239988	1539.173452	3.4737E-05	0.025505295
734.279988	1385.579173	3.12706E-05	0.02296137
734.319988	1145.740555	2.58578E-05	0.018987876
734.359988	867.469711	1.95776E-05	0.014376994
734.399988	665.860909	1.50276E-05	0.011036235
734.439988	508.819629	1.14834E-05	0.008433832
734.479988	384.880151	8.68621E-06	0.006379847
734.519988	337.373741	7.61406E-06	0.005592676
734.559988	343.663603	7.75601E-06	0.005697254
734.599988	377.932742	8.52942E-06	0.006265709
734.639988	414.121113	9.34614E-06	0.006866046
734.679988	413.690265	9.33641E-06	0.006859276
734.719988	395.369589	8.92294E-06	0.006555863
734.759988	407.113386	9.18798E-06	0.006750962
734.799988	451.34619	1.01863E-05	0.00748486
734.839988	515.658355	1.16377E-05	0.008551841
734.879988	569.040328	1.28424E-05	0.009437658
734.919988	586.543412	1.32375E-05	0.00972848
734.959988	642.322073	1.44963E-05	0.010654211
734.999988	821.651988	1.85435E-05	0.013629502
735.039988	1156.473201	2.61E-05	0.019184536
735.079988	1608.695217	3.6306E-05	0.026687822
735.119988	2048.654944	4.62353E-05	0.033988485

735.159988	2268.603508	5.11992E-05	0.037639619
735.199988	2223.792206	5.01879E-05	0.036898138
735.239988	2065.57617	4.66172E-05	0.034274813
735.279988	1885.531032	4.25538E-05	0.031288964
735.319988	1619.878742	3.65584E-05	0.026882127
735.359988	1252.783291	2.82736E-05	0.020791255
735.399988	947.231137	2.13777E-05	0.015721151
735.439988	684.307161	1.54439E-05	0.011358032
735.479988	472.509055	1.06639E-05	0.007843064
735.519988	348.621131	7.86789E-06	0.005786993
735.559988	323.036235	7.29048E-06	0.005362584
735.599988	354.795141	8.00723E-06	0.00589012
735.639988	375.521292	8.47499E-06	0.006234543
735.679988	385.259761	8.69478E-06	0.006396573
735.719988	413.261474	9.32674E-06	0.006861866
735.759988	389.709239	8.79519E-06	0.006471152
735.799988	388.222213	8.76163E-06	0.006446811
735.839988	401.749062	9.06692E-06	0.0066718
735.879988	381.147904	8.60198E-06	0.006330023
735.919988	360.270705	8.13081E-06	0.005983624
735.959988	384.055035	8.66759E-06	0.006378997
735.999988	494.501414	1.11602E-05	0.008213913
736.039988	692.982321	1.56396E-05	0.011511405
736.079988	925.884388	2.08959E-05	0.015381069
736.119988	1196.949296	2.70135E-05	0.019885161
736.159988	1431.061201	3.22971E-05	0.023775802
736.199988	1552.223167	3.50315E-05	0.025790201
736.239988	1500.977199	3.3875E-05	0.024940104
736.279988	1346.651295	3.0392E-05	0.022377054
736.319988	1159.034558	2.61578E-05	0.019260508
736.359988	942.953622	2.12811E-05	0.015670587
736.399988	698.814623	1.57713E-05	0.011613965
736.439988	541.957253	1.22312E-05	0.00900756
736.479988	442.824915	9.99394E-06	0.007360338
736.519988	375.088082	8.46522E-06	0.0062348
736.559988	350.70607	7.91495E-06	0.005829834
736.599988	376.268978	8.49187E-06	0.006255109
736.639988	426.625196	9.62834E-06	0.007092618
736.679988	481.989678	1.08778E-05	0.008013484
736.719988	492.377644	1.11123E-05	0.008186637
736.759988	456.196874	1.02957E-05	0.00758548
736.799988	422.968729	9.54582E-06	0.007033356
736.839988	424.15145	9.57251E-06	0.007053406
736.879988	442.560819	9.98798E-06	0.007359944
736.919988	458.214823	1.03413E-05	0.007620689
736.959988	535.296214	1.20809E-05	0.008903133

736.999988	731.074996	1.64993E-05	0.012160017
737.039988	1071.999865	2.41935E-05	0.017831609
737.079988	1510.80715	3.40968E-05	0.025132079
737.119988	1928.244097	4.35178E-05	0.032077828
737.159988	2214.70507	4.99828E-05	0.036845327
737.199988	2273.917255	5.13191E-05	0.037832473
737.239988	2105.873505	4.75266E-05	0.035038533
737.279988	1795.231696	4.05159E-05	0.029871547
737.319988	1454.04689	3.28158E-05	0.024195755
737.359988	1114.627488	2.51556E-05	0.018548725
737.399988	804.380257	1.81537E-05	0.013386569
737.439988	553.573106	1.24934E-05	0.009213114
737.479988	391.972072	8.84626E-06	0.006523942
737.519988	324.793255	7.33013E-06	0.005406118
737.559988	335.048315	7.56157E-06	0.005577114
737.599988	380.069641	8.57764E-06	0.006326869
737.639988	403.518375	9.10685E-06	0.006717575
737.679988	432.912356	9.77023E-06	0.007207302
737.719988	428.160931	9.663E-06	0.007128585
737.759988	391.778032	8.84188E-06	0.006523189
737.799988	354.376273	7.99778E-06	0.005900761
737.839988	333.46252	7.52578E-06	0.005552825
737.879988	333.353296	7.52332E-06	0.005551307
737.919988	342.563005	7.73117E-06	0.005704985
737.959988	360.88252	8.14462E-06	0.006010401
737.999988	433.031543	9.77292E-06	0.007212414
738.039988	620.13997	1.39957E-05	0.010329383
738.079988	885.259756	1.99791E-05	0.014746159
738.119988	1166.914423	2.63356E-05	0.019438857
738.159988	1400.372039	3.16044E-05	0.023329138
738.199988	1455.674563	3.28525E-05	0.02425175
738.239988	1387.510546	3.13142E-05	0.02311738
738.279988	1284.316202	2.89852E-05	0.021399214
738.319988	1095.051847	2.47138E-05	0.018246689
738.359988	851.950772	1.92273E-05	0.0141967
738.399988	613.928113	1.38555E-05	0.010230903
738.439988	461.913712	1.04247E-05	0.007698052
738.479988	368.77172	8.32266E-06	0.006146121
738.519988	315.575659	7.1221E-06	0.005259815
738.559988	312.866661	7.06096E-06	0.005214946
738.599988	377.157669	8.51192E-06	0.006286906
738.639988	465.947615	1.05158E-05	0.007767382
738.679988	506.382291	1.14283E-05	0.008441888
738.719988	470.946576	1.06286E-05	0.007851565
738.759988	401.14672	9.05332E-06	0.006688233
738.799988	340.07269	7.67497E-06	0.005670265

738.839988	322.650782	7.28178E-06	0.005380069
738.879988	338.543724	7.64046E-06	0.005645383
738.919988	362.915765	8.1905E-06	0.006052127
738.959988	417.946851	9.43248E-06	0.006970224
738.999988	578.766527	1.3062E-05	0.009652784
739.039988	872.683275	1.96952E-05	0.014555574
739.079988	1278.180158	2.88467E-05	0.021320053
739.119988	1720.7717	3.88354E-05	0.028704035
739.159988	2019.43872	4.55759E-05	0.033687896
739.199988	2142.090865	4.8344E-05	0.035735889
739.239988	2066.189665	4.6631E-05	0.034471517
739.279988	1751.526109	3.95295E-05	0.029223372
739.319988	1354.807301	3.05761E-05	0.022605532
739.359988	1049.016568	2.36748E-05	0.017504231
739.399988	784.405701	1.77029E-05	0.013089557
739.439988	533.311487	1.20361E-05	0.008899972
739.479988	382.603906	8.63484E-06	0.006385289
739.519988	314.092517	7.08863E-06	0.005242184
739.559988	282.711524	6.38041E-06	0.004718692
739.599988	313.491902	7.07508E-06	0.005232726
739.639988	357.585047	8.0702E-06	0.00596904
739.679988	386.029076	8.71214E-06	0.006444195
739.719988	409.077157	9.2323E-06	0.006829318
739.759988	387.542343	8.74629E-06	0.006470156
739.799988	338.552078	7.64065E-06	0.005652552
739.839988	295.774092	6.67521E-06	0.004938587
739.879988	269.070179	6.07254E-06	0.00449295
739.919988	268.013004	6.04868E-06	0.004475539
739.959988	298.641189	6.73992E-06	0.004987268
739.999988	414.623612	9.35748E-06	0.006924533
740.039988	623.143275	1.40635E-05	0.010407534
740.079988	883.038112	1.99289E-05	0.01474901
740.119988	1143.356686	2.5804E-05	0.019098032
740.159988	1390.192013	3.13747E-05	0.023222296
740.199988	1495.735061	3.37567E-05	0.024986677
740.239988	1429.767781	3.22679E-05	0.023885966
740.279988	1300.239638	2.93446E-05	0.021723218
740.319988	1094.562918	2.47028E-05	0.018287947
740.359988	858.826238	1.93825E-05	0.014350036
740.399988	661.720577	1.49341E-05	0.011057216
740.439988	529.895442	1.1959E-05	0.008854923
740.479988	434.479197	9.80559E-06	0.007260843
740.519988	351.478913	7.93239E-06	0.005874093
740.559988	335.867265	7.58006E-06	0.005613486
740.599988	381.836162	8.61751E-06	0.006382128
740.639988	426.384028	9.62289E-06	0.0071271

740.679988	460.797506	1.03996E-05	0.007702744
740.719988	468.037701	1.0563E-05	0.007824195
740.759988	435.983955	9.83955E-06	0.007288745
740.799988	411.89905	9.29599E-06	0.006886468
740.839988	379.510014	8.56501E-06	0.006345304
740.879988	358.347171	8.0874E-06	0.00599179
740.919988	385.317267	8.69607E-06	0.006443095
740.959988	464.964214	1.04936E-05	0.007775334
740.999988	605.365184	1.36622E-05	0.010123726
741.039988	818.106551	1.84635E-05	0.013682211
741.079988	1168.160529	2.63638E-05	0.019537652
741.119988	1583.55766	3.57387E-05	0.026486658
741.159988	1983.418243	4.4763E-05	0.033176535
741.199988	2196.561966	4.95733E-05	0.036743762
741.239988	2085.631995	4.70698E-05	0.034890025
741.279988	1743.86226	3.93565E-05	0.029174217
741.319988	1415.827653	3.19533E-05	0.023687589
741.359988	1083.807191	2.446E-05	0.018133679
741.399988	772.420308	1.74325E-05	0.012924419
741.439988	551.870693	1.2455E-05	0.0092346
741.479988	403.722744	9.11146E-06	0.006755965
741.519988	338.64532	7.64275E-06	0.005667254
741.559988	323.323757	7.29697E-06	0.005411139
741.599988	325.48832	7.34582E-06	0.005447659
741.639988	343.931153	7.76205E-06	0.005756644
741.679988	366.969066	8.28198E-06	0.006142579
741.719988	355.207183	8.01653E-06	0.005946022
741.759988	339.603072	7.66437E-06	0.005685122
741.799988	333.633429	7.52964E-06	0.005585488
741.839988	319.179686	7.20344E-06	0.005343801
741.879988	311.694142	7.0345E-06	0.005218757
741.919988	310.322645	7.00355E-06	0.005196074
741.959988	327.040659	7.38085E-06	0.005476297
741.999988	412.811508	9.31658E-06	0.006912903
742.039988	578.09612	1.30468E-05	0.009681265
742.079988	801.929937	1.80984E-05	0.013430491
742.119988	1046.523378	2.36186E-05	0.017527817
742.159988	1235.104694	2.78746E-05	0.020687407
742.199988	1361.71655	3.0732E-05	0.022809324
742.239988	1373.713594	3.10028E-05	0.02301152
742.279988	1287.257374	2.90516E-05	0.021564426
742.319988	1118.16596	2.52354E-05	0.018732778
742.359988	854.482725	1.92845E-05	0.01431603
742.399988	618.209487	1.39521E-05	0.010358059
742.439988	459.286531	1.03655E-05	0.00769573
742.479988	348.335579	7.86145E-06	0.005836968

742.519988	298.731454	6.74195E-06	0.005006035
742.559988	310.864018	7.01577E-06	0.005209629
742.599988	364.634984	8.2293E-06	0.006111081
742.639988	404.077098	9.11946E-06	0.006772474
742.679988	447.373282	1.00966E-05	0.007498537
742.719988	487.940707	1.10121E-05	0.008178938
742.759988	456.518457	1.0303E-05	0.007652646
742.799988	404.159975	9.12133E-06	0.006775322
742.839988	360.779303	8.14229E-06	0.006048416
742.879988	344.603883	7.77723E-06	0.005777548
742.919988	382.467263	8.63175E-06	0.006412702
742.959988	482.077568	1.08798E-05	0.00808327
742.999988	636.186464	1.43578E-05	0.010667877
743.039988	832.588537	1.87904E-05	0.013961992
743.079988	1124.173266	2.5371E-05	0.018852701
743.119988	1463.036594	3.30187E-05	0.024536855
743.159988	1692.283433	3.81925E-05	0.028383123
743.199988	1789.464564	4.03857E-05	0.030014669
743.239988	1753.32612	3.95701E-05	0.029410102
743.279988	1580.89191	3.56785E-05	0.026519136
743.319988	1296.949708	2.92703E-05	0.021757235
743.359988	963.192196	2.17379E-05	0.01615909
743.399988	702.919106	1.58639E-05	0.011793227
743.439988	521.178541	1.17623E-05	0.008744545
743.479988	399.765892	9.02216E-06	0.006707795
743.519988	341.361187	7.70405E-06	0.005728113
743.559988	341.762874	7.71311E-06	0.005735161
743.599988	347.80842	7.84955E-06	0.005836926
743.639988	355.764324	8.02911E-06	0.005970764
743.679988	354.342597	7.99702E-06	0.005947223
743.719988	360.126714	8.12756E-06	0.006044628
743.759988	379.069167	8.55506E-06	0.006362914
743.799988	363.755092	8.20945E-06	0.006106186
743.839988	314.343408	7.09429E-06	0.005277019
743.879988	302.97196	6.83766E-06	0.005086395
743.919988	311.540816	7.03104E-06	0.005230533
743.959988	350.90284	7.91939E-06	0.005891708
743.999988	436.724854	9.85627E-06	0.007333066
744.039988	599.867254	1.35382E-05	0.010072939
744.079988	815.780184	1.8411E-05	0.013699273
744.119988	1062.108369	2.39703E-05	0.017836784
744.159988	1258.052422	2.83925E-05	0.021128556
744.199988	1372.465504	3.09746E-05	0.023051323
744.239988	1369.103038	3.08987E-05	0.022996084
744.279988	1218.030808	2.74893E-05	0.020459705
744.319988	1000.974301	2.25906E-05	0.016814632

744.359988	792.018869	1.78748E-05	0.013305258
744.399988	613.07893	1.38363E-05	0.010299769
744.439988	481.658543	1.08704E-05	0.008092332
744.479988	372.219398	8.40047E-06	0.006253984
744.519988	312.361701	7.04957E-06	0.005248545
744.559988	320.605227	7.23561E-06	0.005387348
744.599988	360.413718	8.13404E-06	0.006056603
744.639988	387.962334	8.75577E-06	0.006519896
744.679988	417.157382	9.41466E-06	0.00701091
744.719988	427.355039	9.64481E-06	0.007182681
744.759988	423.887093	9.56654E-06	0.007124777
744.799988	414.318819	9.3506E-06	0.006964326
744.839988	396.583207	8.95033E-06	0.006666564
744.879988	390.691808	8.81737E-06	0.006567882
744.919988	399.948664	9.02628E-06	0.00672386
744.959988	506.683337	1.14351E-05	0.00851872
744.999988	687.459509	1.5515E-05	0.011558677
745.039988	895.476771	2.02097E-05	0.015057007
745.079988	1289.205364	2.90956E-05	0.021678526
745.119988	1768.870091	3.99209E-05	0.029745886
745.159988	2118.035046	4.78011E-05	0.035619468
745.199988	2199.828786	4.96471E-05	0.036996997
745.239988	2052.940185	4.6332E-05	0.034528459
745.279988	1814.997262	4.0962E-05	0.030528129
745.319988	1534.125393	3.46231E-05	0.025805269
745.359988	1211.922773	2.73514E-05	0.020386646
745.399988	923.933426	2.08519E-05	0.015542999
745.439988	706.904431	1.59538E-05	0.011892637
745.479988	537.412636	1.21287E-05	0.009041669
745.519988	436.613344	9.85376E-06	0.007346171
745.559988	377.901942	8.52872E-06	0.006358673
745.599988	367.564308	8.29541E-06	0.006185061
745.639988	392.028336	8.84753E-06	0.006597075
745.679988	417.502832	9.42246E-06	0.007026138
745.719988	423.396508	9.55547E-06	0.007125705
745.759988	396.027427	8.93779E-06	0.006665444
745.799988	351.334951	7.92914E-06	0.005913553
745.839988	309.603899	6.98733E-06	0.005211429
745.879988	295.814846	6.67613E-06	0.004979591
745.919988	319.504507	7.21077E-06	0.005378659
745.959988	381.422206	8.60817E-06	0.006421349
745.999988	498.666507	1.12542E-05	0.008395639
746.039988	675.815899	1.52522E-05	0.011378769
746.079988	909.325905	2.05222E-05	0.015311217
746.119988	1169.579417	2.63958E-05	0.019694418
746.159988	1426.438068	3.21927E-05	0.02402092

746.199988	1615.138801	3.64514E-05	0.027200059
746.239988	1613.285542	3.64096E-05	0.027170305
746.279988	1438.776579	3.24712E-05	0.024232594
746.319988	1212.465793	2.73637E-05	0.020422049
746.359988	979.583931	2.21078E-05	0.016500411
746.399988	770.981216	1.74E-05	0.012987339
746.439988	567.052834	1.27976E-05	0.009552635
746.479988	424.873945	9.58881E-06	0.007157857
746.519988	371.818663	8.39143E-06	0.00626437
746.559988	354.556718	8.00185E-06	0.005973862
746.599988	374.493657	8.4518E-06	0.006310114
746.639988	438.542891	9.8973E-06	0.007389722
746.679988	434.368303	9.80309E-06	0.007319769
746.719988	423.872396	9.56621E-06	0.00714328
746.759988	410.842292	9.27214E-06	0.006924062
746.799988	365.936759	8.25868E-06	0.006167584
746.839988	338.456654	7.6385E-06	0.005704734
746.879988	365.094585	8.23968E-06	0.006154049
746.919988	446.755134	1.00826E-05	0.007530926
746.959988	611.211813	1.37942E-05	0.010303715
746.999988	930.067151	2.09903E-05	0.015679769
747.039988	1401.093959	3.16207E-05	0.023621957
747.079988	1914.165582	4.32001E-05	0.032273894
747.119988	2326.09163	5.24966E-05	0.039221295
747.159988	2610.204823	5.89087E-05	0.044014214
747.199988	2594.403793	5.85521E-05	0.043750113
747.239988	2342.938869	5.28769E-05	0.039511709
747.279988	1923.292368	4.3406E-05	0.032436457
747.319988	1418.662598	3.20172E-05	0.023927123
747.359988	1027.180078	2.3182E-05	0.017325317
747.399988	781.400433	1.76351E-05	0.013180488
747.439988	595.239418	1.34337E-05	0.010040903
747.479988	454.016618	1.02465E-05	0.007659071
747.519988	374.590726	8.45399E-06	0.006319527
747.559988	349.536025	7.88854E-06	0.005897158
747.599988	355.49549	8.02304E-06	0.005998023
747.639988	362.368335	8.17815E-06	0.006114311
747.679988	351.267804	7.92763E-06	0.005927327
747.719988	367.127012	8.28555E-06	0.006195268
747.759988	396.50417	8.94855E-06	0.006691365
747.799988	384.383816	8.67501E-06	0.00648717
747.839988	352.98986	7.96649E-06	0.005957659
747.879988	327.934757	7.40103E-06	0.005535083
747.919988	331.178691	7.47424E-06	0.005590135
747.959988	401.805739	9.0682E-06	0.006782648
747.999988	546.378415	1.2331E-05	0.009223588

748.039988	792.675392	1.78896E-05	0.013382121
748.079988	1081.3342	2.44042E-05	0.018256299
748.119988	1413.206644	3.18941E-05	0.023860618
748.159988	1678.064573	3.78716E-05	0.028334001
748.199988	1779.782664	4.01672E-05	0.03005311
748.239988	1724.993544	3.89307E-05	0.029129508
748.279988	1525.508587	3.44286E-05	0.025762235
748.319988	1268.462384	2.86274E-05	0.021422477
748.359988	1046.114515	2.36093E-05	0.017668291
748.399988	822.583564	1.85646E-05	0.01389372
748.439988	620.317044	1.39997E-05	0.010477929
748.479988	463.470822	1.04599E-05	0.007829019
748.519988	378.182092	8.53504E-06	0.00638865
748.559988	358.172908	8.08346E-06	0.006050957
748.599988	363.126305	8.19525E-06	0.006134968
748.639988	399.323931	9.01218E-06	0.006746882
748.679988	441.747649	9.96963E-06	0.007464062
748.719988	475.960776	1.07418E-05	0.008042579
748.759988	455.447254	1.02788E-05	0.007696362
748.799988	390.799929	8.81981E-06	0.006604274
748.839988	378.352393	8.53889E-06	0.00639426
748.879988	449.869148	1.01529E-05	0.007603319
748.919988	543.833409	1.22736E-05	0.009191916
748.959988	785.332618	1.77239E-05	0.013274464
748.999988	1286.665969	2.90383E-05	0.021749655
749.039988	2093.590901	4.72494E-05	0.035391713
749.079988	3008.769863	6.79037E-05	0.050865336
749.119988	3627.000462	8.18563E-05	0.061320227
749.159988	3813.127175	8.6057E-05	0.064470438
749.199988	3499.218896	7.89725E-05	0.059166193
749.239988	2844.799633	6.42032E-05	0.048103579
749.279988	2164.59668	4.88519E-05	0.036603775
749.319988	1594.623374	3.59884E-05	0.026966849
749.359988	1167.740289	2.63543E-05	0.019748837
749.399988	832.384703	1.87858E-05	0.014078051
749.439988	580.512184	1.31014E-05	0.009818677
749.479988	433.05394	9.77342E-06	0.007324986
749.519988	346.913635	7.82936E-06	0.00586826
749.559988	325.366656	7.34307E-06	0.005504073
749.599988	329.106199	7.42747E-06	0.00556763
749.639988	356.513206	8.04601E-06	0.006031608
749.679988	430.94768	9.72589E-06	0.007291304
749.719988	442.607701	9.98904E-06	0.007488982
749.759988	399.642614	9.01938E-06	0.006762368
749.799988	357.646975	8.07159E-06	0.006052081
749.839988	337.643303	7.62014E-06	0.005713885

749.879988	331.436805	7.48007E-06	0.005609153
749.919988	377.214634	8.51321E-06	0.006384225
749.959988	491.092609	1.10833E-05	0.008312014
749.999988	694.146186	1.56659E-05	0.011749434
750.039988	1026.491625	2.31665E-05	0.017375791
750.079988	1502.567395	3.39109E-05	0.025435852
750.119988	1865.33011	4.20979E-05	0.031578478
750.159988	2047.149021	4.62013E-05	0.034658368
750.199988	2092.337932	4.72212E-05	0.035425308
750.239988	1961.626381	4.42712E-05	0.033214006
750.279988	1624.62376	3.66655E-05	0.027509387
750.319988	1247.994448	2.81655E-05	0.021133135
750.359988	925.733527	2.08925E-05	0.015676908
750.399988	679.160219	1.53277E-05	0.011501906
750.439988	502.58424	1.13426E-05	0.00851196
750.479988	414.652511	9.35813E-06	0.007023089
750.519988	373.888356	8.43814E-06	0.006332992
750.559988	354.770959	8.00669E-06	0.006009498
750.599988	397.83095	8.97849E-06	0.006739255
750.639988	482.646069	1.08926E-05	0.008176458
750.679988	557.849762	1.25899E-05	0.00945098
750.719988	570.406254	1.28733E-05	0.009664225
750.759988	534.951475	1.20731E-05	0.009064008
750.799988	483.157934	1.09042E-05	0.008186874
750.839988	449.287117	1.01398E-05	0.007613356
750.879988	454.758419	1.02633E-05	0.00770648
750.919988	500.369699	1.12926E-05	0.008479874
750.959988	616.141841	1.39055E-05	0.010442446
750.999988	896.590776	2.02348E-05	0.015196338
751.039988	1301.223487	2.93668E-05	0.022055642
751.079988	1777.890889	4.01245E-05	0.030136725
751.119988	2197.961418	4.96049E-05	0.037259253
751.159988	2425.947108	5.47502E-05	0.041126195
751.199988	2429.124634	5.4822E-05	0.041182255
751.239988	2235.358654	5.04489E-05	0.037899254
751.279988	1856.836933	4.19062E-05	0.031483307
751.319988	1404.877244	3.17061E-05	0.023821444
751.359988	1032.798742	2.33088E-05	0.017513322
751.399988	766.586736	1.73008E-05	0.012999817
751.439988	568.385792	1.28277E-05	0.009639228
751.479988	458.872734	1.03561E-05	0.007782416
751.519988	415.806253	9.38417E-06	0.00705239
751.559988	397.523861	8.97156E-06	0.006742665
751.599988	431.784667	9.74478E-06	0.007324175
751.639988	473.848371	1.06941E-05	0.008038111
751.679988	477.476802	1.0776E-05	0.008100093

751.719988	444.960971	1.00421E-05	0.007548884
751.759988	409.427595	9.24021E-06	0.006946421
751.799988	384.931557	8.68737E-06	0.006531164
751.839988	333.437578	7.52522E-06	0.005657763
751.879988	315.739789	7.12581E-06	0.005357752
751.919988	339.366308	7.65902E-06	0.005758974
751.959988	406.709084	9.17886E-06	0.006902134
751.999988	528.597936	1.19297E-05	0.008971149
752.039988	746.251766	1.68419E-05	0.012665754
752.079988	978.460584	2.20825E-05	0.016607801
752.119988	1256.315099	2.83533E-05	0.02132507
752.159988	1498.581753	3.38209E-05	0.02543873
752.199988	1592.056495	3.59305E-05	0.02702692
752.239988	1508.680977	3.40488E-05	0.02561289
752.279988	1312.021958	2.96105E-05	0.022275393
752.319988	1072.281958	2.41999E-05	0.018206076
752.359988	838.850542	1.89317E-05	0.014243445
752.399988	613.015495	1.38349E-05	0.010409383
752.439988	450.282377	1.01622E-05	0.00764648
752.479988	382.954819	8.64276E-06	0.006503502
752.519988	358.070979	8.08116E-06	0.006081237
752.559988	342.101567	7.72076E-06	0.005810332
752.599988	363.283846	8.19881E-06	0.006170425
752.639988	400.819132	9.04593E-06	0.006808328
752.679988	429.217836	9.68685E-06	0.007291097
752.719988	421.044168	9.50238E-06	0.007152632
752.759988	432.898918	9.76993E-06	0.007354409
752.799988	447.8165	1.01066E-05	0.007608244
752.839988	414.868232	9.363E-06	0.007048839
752.879988	382.689557	8.63677E-06	0.006502451
752.919988	412.754833	9.3153E-06	0.007013677
752.959988	504.121804	1.13773E-05	0.008566672
752.999988	657.051088	1.48287E-05	0.011166032
753.039988	904.695998	2.04177E-05	0.015375366
753.079988	1247.938827	2.81642E-05	0.021209926
753.119988	1627.919311	3.67399E-05	0.027669531
753.159988	1950.629895	4.4023E-05	0.033156362
753.199988	2063.642412	4.65735E-05	0.035079186
753.239988	1953.200158	4.4081E-05	0.033203577
753.279988	1708.531479	3.85592E-05	0.029045855
753.319988	1402.712578	3.16573E-05	0.023848054
753.359988	1050.880478	2.37169E-05	0.01786737
753.399988	754.171764	1.70206E-05	0.012823325
753.439988	547.627794	1.23592E-05	0.009311913
753.479988	406.175038	9.1668E-06	0.006907004
753.519988	342.692709	7.7341E-06	0.005827797

753.559988	337.2255	7.61071E-06	0.005735126
753.599988	348.025581	7.85445E-06	0.005919115
753.639988	397.699962	8.97553E-06	0.006764321
753.679988	437.699883	9.87828E-06	0.007445059
753.719988	434.098918	9.79701E-06	0.007384201
753.759988	396.218665	8.9421E-06	0.0067402
753.799988	364.371069	8.22335E-06	0.006198759
753.839988	323.541841	7.30189E-06	0.005504456
753.879988	295.89762	6.678E-06	0.005034408
753.919988	291.148618	6.57082E-06	0.004953872
753.959988	341.900075	7.71621E-06	0.005817712
753.999988	440.28504	9.93662E-06	0.007492211
754.039988	588.42211	1.32799E-05	0.010013551
754.079988	797.071898	1.79888E-05	0.013564996
754.119988	1065.903973	2.4056E-05	0.018141086
754.159988	1305.589676	2.94653E-05	0.022221581
754.199988	1416.357735	3.19652E-05	0.024108169
754.239988	1392.709357	3.14315E-05	0.023706902
754.279988	1229.804672	2.7755E-05	0.020935025
754.319988	1029.23002	2.32283E-05	0.017521562
754.359988	844.076646	1.90496E-05	0.014370282
754.399988	642.735569	1.45056E-05	0.01094306
754.439988	465.661242	1.05093E-05	0.007928655
754.479988	341.067727	7.69742E-06	0.005807552
754.519988	288.296554	6.50645E-06	0.004909248
754.559988	294.467936	6.64573E-06	0.005014603
754.599988	339.58251	7.6639E-06	0.005783182
754.639988	393.472147	8.88012E-06	0.006701292
754.679988	420.17409	9.48274E-06	0.007156437
754.719988	432.170074	9.75348E-06	0.007361144
754.759988	415.411942	9.37527E-06	0.007076078
754.799988	416.445455	9.39859E-06	0.007094059
754.839988	412.773437	9.31572E-06	0.007031879
754.879988	401.387203	9.05875E-06	0.006838269
754.919988	408.527048	9.21989E-06	0.006960277
754.959988	438.509612	9.89655E-06	0.0074715
754.999988	545.202165	1.23045E-05	0.009289862
755.039988	773.741908	1.74623E-05	0.013184718
755.079988	1116.024545	2.51871E-05	0.01901829
755.119988	1493.604929	3.37086E-05	0.025454025
755.159988	1753.405182	3.95719E-05	0.029883125
755.199988	1934.579043	4.36608E-05	0.032972601
755.239988	1939.654826	4.37753E-05	0.033060863
755.279988	1729.828162	3.90398E-05	0.029485989
755.319988	1411.263673	3.18503E-05	0.024057135
755.359988	1090.896571	2.462E-05	0.018596975

755.399988	795.496823	1.79533E-05	0.013561889
755.439988	583.764621	1.31748E-05	0.009952736
755.479988	433.16654	9.77597E-06	0.007385546
755.519988	359.703625	8.11801E-06	0.006133319
755.559988	351.167506	7.92536E-06	0.005988086
755.599988	375.429849	8.47293E-06	0.006402145
755.639988	393.494228	8.88062E-06	0.006710549
755.679988	395.037542	8.91545E-06	0.006737225
755.719988	378.491979	8.54204E-06	0.006455388
755.759988	350.529188	7.91096E-06	0.005978784
755.799988	334.379063	7.54647E-06	0.005703622
755.839988	346.745501	7.82556E-06	0.005914873
755.879988	319.71091	7.21543E-06	0.005453999
755.919988	291.790609	6.58531E-06	0.004977966
755.959988	318.061412	7.1782E-06	0.005426434
755.999988	407.325108	9.19276E-06	0.006949727
756.039988	548.284666	1.2374E-05	0.009355255
756.079988	723.079242	1.63189E-05	0.012338387
756.119988	979.805736	2.21129E-05	0.016719968
756.159988	1242.666399	2.80452E-05	0.021206696
756.199988	1393.2974	3.14448E-05	0.023778543
756.239988	1392.765004	3.14328E-05	0.023770715
756.279988	1254.421905	2.83106E-05	0.021410706
756.319988	1029.769171	2.32405E-05	0.017577221
756.359988	780.110327	1.7606E-05	0.013316477
756.399988	579.106901	1.30696E-05	0.009885873
756.439988	465.310688	1.05014E-05	0.00794369
756.479988	372.831881	8.4143E-06	0.006365247
756.519988	304.31038	6.86786E-06	0.005195674
756.559988	313.885831	7.08397E-06	0.005359445
756.599988	385.001025	8.68894E-06	0.00657405
756.639988	429.748859	9.69883E-06	0.007338525
756.679988	410.706444	9.26907E-06	0.007013722
756.719988	371.597084	8.38643E-06	0.006346178
756.759988	360.75516	8.14174E-06	0.006161344
756.799988	379.686714	8.569E-06	0.006485019
756.839988	375.293561	8.46985E-06	0.006410323
756.879988	384.215978	8.67122E-06	0.006563072
756.919988	401.477662	9.06079E-06	0.006858294
756.959988	457.934705	1.03349E-05	0.007823142
756.999988	637.193781	1.43806E-05	0.010886096
757.039988	950.419536	2.14496E-05	0.016238239
757.079988	1299.813128	2.9335E-05	0.02220892
757.119988	1684.399607	3.80146E-05	0.028781578
757.159988	2047.9938	4.62204E-05	0.034996213
757.199988	2215.924403	5.00103E-05	0.037867819

757.239988	2185.849552	4.93316E-05	0.037355845
757.279988	1991.071604	4.49357E-05	0.034028916
757.319988	1591.762114	3.59239E-05	0.027205853
757.359988	1186.25267	2.67721E-05	0.020276095
757.399988	868.576912	1.96026E-05	0.014846987
757.439988	628.338948	1.41807E-05	0.010741056
757.479988	445.961004	1.00647E-05	0.007623823
757.519988	351.886196	7.94158E-06	0.006015907
757.559988	307.351228	6.93649E-06	0.005254807
757.599988	345.510212	7.79768E-06	0.005907525
757.639988	408.709108	9.224E-06	0.006988468
757.679988	441.140616	9.95593E-06	0.007543408
757.719988	425.778831	9.60924E-06	0.00728111
757.759988	382.169966	8.62504E-06	0.006535713
757.799988	350.60936	7.91276E-06	0.005996293
757.839988	299.25912	6.75386E-06	0.005118346
757.879988	298.118272	6.72811E-06	0.005099103
757.919988	312.221954	7.04641E-06	0.005340618
757.959988	339.38052	7.65935E-06	0.005805478
757.999988	455.667811	1.02838E-05	0.007795111
758.039988	665.306323	1.5015E-05	0.011381998
758.079988	939.993396	2.12143E-05	0.016082168
758.119988	1282.178263	2.8937E-05	0.021937701
758.159988	1619.947889	3.656E-05	0.027718304
758.199988	1860.505973	4.1989E-05	0.031836081
758.239988	1860.922718	4.19984E-05	0.031844892
758.279988	1617.049245	3.64945E-05	0.027673086
758.319988	1256.515111	2.83578E-05	0.021504283
758.359988	929.931568	2.09873E-05	0.015915898
758.399988	683.046583	1.54154E-05	0.011691047
758.439988	535.490736	1.20853E-05	0.00916596
758.479988	423.557253	9.5591E-06	0.007250384
758.519988	342.323938	7.72577E-06	0.005860154
758.559988	341.10481	7.69826E-06	0.005839592
758.599988	416.397275	9.39751E-06	0.007128948
758.639988	520.239669	1.17411E-05	0.008907256
758.679988	571.772704	1.29041E-05	0.009790093
758.719988	591.783686	1.33557E-05	0.010133262
758.759988	567.698122	1.28122E-05	0.009721351
758.799988	485.175582	1.09497E-05	0.00830866
758.839988	430.800381	9.72256E-06	0.007377871
758.879988	419.155339	9.45975E-06	0.007178817
758.919988	422.112324	9.52649E-06	0.007229842
758.959988	450.528591	1.01678E-05	0.007716955
758.999988	594.867223	1.34253E-05	0.010189821
759.039988	893.494204	2.01649E-05	0.01530598

759.079988	1227.182221	2.76958E-05	0.021023323
759.119988	1542.81587	3.48192E-05	0.026431955
759.159988	1857.504074	4.19213E-05	0.031824958
759.199988	2064.308829	4.65886E-05	0.035370046
759.239988	2058.65059	4.64609E-05	0.035274955
759.279987	1841.56856	4.15616E-05	0.031556919
759.319987	1476.276246	3.33175E-05	0.025298643
759.359987	1100.365437	2.48337E-05	0.01885773
759.399987	779.092335	1.7583E-05	0.013352552
759.439987	567.622216	1.28104E-05	0.009728763
759.479987	451.778224	1.0196E-05	0.007743662
759.519987	396.267748	8.94321E-06	0.006792548
759.559987	361.361291	8.15542E-06	0.006194531
759.599987	356.22865	8.03958E-06	0.006106868
759.639987	383.641716	8.65826E-06	0.00657716
759.679987	434.417858	9.80421E-06	0.007448059
759.719987	469.229305	1.05899E-05	0.008045322
759.759987	454.493956	1.02573E-05	0.007793083
759.799987	394.882925	8.91196E-06	0.006771305
759.839987	337.531599	7.61762E-06	0.005788171
759.879987	314.325413	7.09389E-06	0.005390503
759.919987	313.606201	7.07766E-06	0.005378452
759.959987	362.689223	8.18539E-06	0.006220569
759.999987	445.016657	1.00434E-05	0.007632988
760.039987	555.76055	1.25427E-05	0.009532985
760.079987	744.682057	1.68064E-05	0.012774236
760.119987	995.548012	2.24681E-05	0.017078476
760.159987	1232.720503	2.78208E-05	0.021148247
760.199987	1377.324225	3.10843E-05	0.023630276
760.239987	1377.885783	3.1097E-05	0.023641155
760.279987	1263.663629	2.85191E-05	0.021682522
760.319987	1069.443598	2.41359E-05	0.018350971
760.359987	829.656851	1.87242E-05	0.014237132
760.399987	619.907406	1.39904E-05	0.010638335
760.439987	483.483651	1.09116E-05	0.008297581
760.479987	399.382767	9.01351E-06	0.006854596
760.519987	338.731467	7.6447E-06	0.005813945
760.559987	340.53061	7.6853E-06	0.005845133
760.599987	374.193432	8.44502E-06	0.006423286
760.639987	408.090857	9.21004E-06	0.007005526
760.679987	445.462715	1.00535E-05	0.007647476
760.719987	478.185291	1.0792E-05	0.008209672
760.759987	486.023825	1.09689E-05	0.008344686
760.799987	461.129992	1.04071E-05	0.007917692
760.839987	408.259044	9.21384E-06	0.007010256
760.879987	380.722126	8.59237E-06	0.006537761

760.919987	416.151609	9.39196E-06	0.007146532
760.959987	531.200559	1.19885E-05	0.009122736
760.999987	759.638695	1.7144E-05	0.013046574
761.039987	1015.934751	2.29282E-05	0.017449302
761.079987	1258.542995	2.84036E-05	0.021617383
761.119987	1541.150831	3.47816E-05	0.026472992
761.159987	1805.369607	4.07447E-05	0.031013218
761.199987	1936.09183	4.36949E-05	0.033260554
761.239987	1869.345129	4.21885E-05	0.032115585
761.279987	1646.360459	3.71561E-05	0.028286167
761.319987	1403.77596	3.16813E-05	0.024119583
761.359987	1170.715179	2.64214E-05	0.020116205
761.399987	924.154614	2.08569E-05	0.01588043
761.439987	717.709027	1.61977E-05	0.012333571
761.479987	537.871942	1.2139E-05	0.009243621
761.519987	421.655353	9.51617E-06	0.007246757
761.559987	355.82974	8.03058E-06	0.00611577
761.599987	364.479957	8.2258E-06	0.006264773
761.639987	400.068891	9.029E-06	0.006876846
761.679987	413.760046	9.33799E-06	0.007112559
761.719987	400.615629	9.04134E-06	0.006886967
761.759987	394.793243	8.90993E-06	0.006787231
761.799987	374.716042	8.45682E-06	0.006442405
761.839987	335.628108	7.57466E-06	0.005770678
761.879987	309.310691	6.98071E-06	0.005318464
761.919987	313.039922	7.06488E-06	0.005382869
761.959987	382.603008	8.63482E-06	0.006579385
761.999987	466.416525	1.05264E-05	0.008021095
762.039987	623.2275	1.40654E-05	0.010718379
762.079987	877.198118	1.97971E-05	0.015087004
762.119987	1092.761825	2.46621E-05	0.018795489
762.159987	1222.140249	2.7582E-05	0.021021899
762.199987	1222.239027	2.75842E-05	0.021024702
762.239987	1155.302828	2.60736E-05	0.019874322
762.279987	1125.238732	2.53951E-05	0.019358154
762.319987	1071.764563	2.41882E-05	0.018439174
762.359987	923.298161	2.08376E-05	0.015885717
762.399987	708.169434	1.59824E-05	0.01218498
762.439987	538.163799	1.21456E-05	0.009260297
762.479987	450.098845	1.01581E-05	0.007745351
762.519987	398.231734	8.98754E-06	0.006853175
762.559987	387.871413	8.75372E-06	0.006675235
762.599987	423.94558	9.56786E-06	0.007296451
762.639987	449.542469	1.01455E-05	0.0077374
762.679987	427.716884	9.65297E-06	0.00736213
762.719987	401.123133	9.05279E-06	0.006904744

762.759987	389.864328	8.79869E-06	0.006711292
762.799987	417.777359	9.42865E-06	0.007192176
762.839987	437.46542	9.87299E-06	0.007531508
762.879987	404.376764	9.12622E-06	0.006962211
762.919987	392.144168	8.85015E-06	0.006751955
762.959987	503.287038	1.13585E-05	0.008666071
762.999987	783.843536	1.76903E-05	0.013497666
763.039987	1150.519304	2.59656E-05	0.019812805
763.079987	1535.285975	3.46493E-05	0.026440161
763.119987	1832.980104	4.13678E-05	0.031568601
763.159987	2050.188441	4.62699E-05	0.035311334
763.199987	2041.437428	4.60724E-05	0.035162455
763.239987	1849.50961	4.17409E-05	0.03185829
763.279987	1588.178165	3.5843E-05	0.027358221
763.319987	1294.674389	2.9219E-05	0.022303445
763.359987	1015.63711	2.29215E-05	0.017497367
763.399987	766.769222	1.73049E-05	0.013210571
763.439987	593.174782	1.33871E-05	0.010220269
763.479987	460.718394	1.03978E-05	0.007938491
763.519987	400.095705	9.0296E-06	0.006894282
763.559987	387.96206	8.75576E-06	0.006685551
763.599987	385.040444	8.68983E-06	0.006635551
763.639987	417.900155	9.43142E-06	0.007202213
763.679987	458.155491	1.03399E-05	0.007896399
763.719987	445.901844	1.00634E-05	0.007685607
763.759987	441.568366	9.96558E-06	0.007611314
763.799987	420.351718	9.48675E-06	0.007245982
763.839987	356.790941	8.05227E-06	0.006150649
763.879987	326.829037	7.37608E-06	0.005634437
763.919987	353.775544	7.98422E-06	0.006099306
763.959987	391.659062	8.8392E-06	0.006752795
763.999987	486.327173	1.09757E-05	0.008385455
764.039987	694.74415	1.56794E-05	0.011979695
764.079987	934.475207	2.10898E-05	0.016114297
764.119987	1168.622845	2.63742E-05	0.020153046
764.159987	1357.982076	3.06478E-05	0.023419795
764.199987	1457.815126	3.29009E-05	0.025142834
764.239987	1462.433961	3.30051E-05	0.025223815
764.279987	1321.702788	2.9829E-05	0.022797701
764.319987	1106.305087	2.49678E-05	0.019083362
764.359987	879.401378	1.98469E-05	0.015170149
764.399987	676.649682	1.5271E-05	0.011673183
764.439987	476.371189	1.0751E-05	0.00821852
764.479987	358.739982	8.09626E-06	0.00618943
764.519987	349.112103	7.87897E-06	0.006023633
764.559987	392.810686	8.86519E-06	0.00677797

764.599987	430.57976	9.71759E-06	0.007430066
764.639987	451.476237	1.01892E-05	0.007791062
764.679987	457.133509	1.03169E-05	0.007889101
764.719987	459.223082	1.0364E-05	0.007925577
764.759987	439.381582	9.91623E-06	0.007583536
764.799987	448.118632	1.01134E-05	0.007734738
764.839987	441.264678	9.95873E-06	0.007616834
764.879987	449.665227	1.01483E-05	0.007762245
764.919987	513.562527	1.15904E-05	0.008865722
764.959987	667.544449	1.50655E-05	0.011524542
764.999987	1012.469893	2.285E-05	0.017480277
765.039987	1481.018258	3.34245E-05	0.025571094
765.079987	1976.53996	4.46078E-05	0.0341285
765.119987	2370.623757	5.35017E-05	0.040935202
765.159987	2506.282283	5.65633E-05	0.043279974
765.199987	2299.388095	5.1894E-05	0.039709278
765.239987	1939.717639	4.37767E-05	0.033499701
765.279987	1572.888091	3.54979E-05	0.027165827
765.319987	1234.364369	2.78579E-05	0.021320196
765.359987	919.944883	2.07619E-05	0.015890308
765.399987	673.093763	1.51908E-05	0.011627029
765.439987	528.800526	1.19343E-05	0.009134984
765.479987	438.704014	9.90094E-06	0.00757897
765.519987	395.916638	8.93529E-06	0.006840141
765.559987	403.619784	9.10914E-06	0.00697359
765.599987	419.083205	9.45812E-06	0.00724114
765.639987	413.190713	9.32514E-06	0.007139699
765.679987	411.998488	9.29823E-06	0.00711947
765.719987	412.358561	9.30636E-06	0.007126065
765.759987	385.575495	8.7019E-06	0.006663568
765.799987	322.901878	7.28745E-06	0.005580726
765.839987	298.332467	6.73295E-06	0.005156361
765.879987	297.650775	6.71756E-06	0.005144847
765.919987	328.370238	7.41086E-06	0.005676125
765.959987	383.199315	8.64827E-06	0.006624232
765.999987	498.425473	1.12488E-05	0.008616557
766.039987	712.615937	1.60827E-05	0.012320029
766.079987	1018.501246	2.29862E-05	0.017609233
766.119987	1313.929966	2.96536E-05	0.022718192
766.159987	1519.076975	3.42835E-05	0.026266609
766.199987	1538.826593	3.47292E-05	0.026609492
766.239987	1408.320924	3.17838E-05	0.02435405
766.279987	1227.448321	2.77018E-05	0.021227334
766.319987	1011.823163	2.28354E-05	0.017499254
766.359987	766.984555	1.73098E-05	0.013265517
766.399987	588.63982	1.32848E-05	0.010181456

766.439987	458.310888	1.03434E-05	0.007927625
766.479987	372.138626	8.39865E-06	0.006437397
766.519987	335.296035	7.56716E-06	0.005800383
766.559987	328.315325	7.40962E-06	0.005679918
766.599987	341.671842	7.71106E-06	0.005911297
766.639987	419.031667	9.45696E-06	0.007250085
766.679987	461.418747	1.04136E-05	0.007983882
766.719987	436.950725	9.86137E-06	0.007560909
766.759987	391.130792	8.82728E-06	0.006768403
766.799987	368.33174	8.31273E-06	0.006374205
766.839987	403.942914	9.11643E-06	0.006990842
766.879987	440.344282	9.93796E-06	0.00762122
766.919987	487.831894	1.10097E-05	0.008443549
766.959987	596.670087	1.3466E-05	0.010327893
766.999987	876.279135	1.97764E-05	0.015168498
767.039987	1256.201682	2.83507E-05	0.021746138
767.079987	1646.852687	3.71672E-05	0.028510193
767.119987	2042.879563	4.61049E-05	0.035368026
767.159987	2241.452272	5.05865E-05	0.038807905
767.199987	2233.797651	5.04137E-05	0.038677391
767.239987	2050.724878	4.6282E-05	0.035509404
767.279987	1702.124015	3.84146E-05	0.029474729
767.319987	1292.559493	2.91713E-05	0.022383696
767.359987	951.906286	2.14832E-05	0.016485347
767.399987	708.515745	1.59902E-05	0.01227089
767.439987	529.246082	1.19443E-05	0.009166569
767.479987	406.226204	9.16796E-06	0.007036226
767.519987	340.589572	7.68663E-06	0.005899644
767.559987	342.792628	7.73635E-06	0.005938114
767.599987	358.14286	8.08279E-06	0.006204346
767.639987	374.878132	8.46048E-06	0.006494601
767.679987	398.838841	9.00124E-06	0.00691007
767.719987	441.594199	9.96617E-06	0.007651225
767.759987	453.000345	1.02236E-05	0.007849261
767.799987	417.834643	9.42995E-06	0.007240312
767.839987	371.046827	8.37401E-06	0.0064299
767.879987	345.765377	7.80344E-06	0.005992108
767.919987	349.374924	7.88491E-06	0.006054976
767.959987	404.854432	9.137E-06	0.007016851
767.999987	511.345859	1.15404E-05	0.008862999
768.039987	682.644573	1.54063E-05	0.011832683
768.079987	911.398835	2.0569E-05	0.015798639
768.119987	1157.016457	2.61122E-05	0.020057341
768.159987	1331.760595	3.0056E-05	0.023087802
768.199987	1346.055015	3.03786E-05	0.02333683
768.239987	1225.17874	2.76506E-05	0.021242279

768.279987	1075.785104	2.4279E-05	0.018653048
768.319987	931.198139	2.10158E-05	0.016146894
768.359987	767.448811	1.73203E-05	0.013308188
768.399987	624.261479	1.40887E-05	0.010825766
768.439987	498.158142	1.12427E-05	0.008639367
768.479987	404.432589	9.12748E-06	0.007014286
768.519987	347.93129	7.85232E-06	0.006034668
768.559987	356.786703	8.05218E-06	0.006188583
768.599987	391.9179	8.84504E-06	0.006798298
768.639987	442.988934	9.99764E-06	0.007684588
768.679987	467.857507	1.05589E-05	0.008116409
768.719987	464.923934	1.04927E-05	0.008065937
768.759987	427.567483	9.6496E-06	0.007418228
768.799987	370.094553	8.35252E-06	0.006421416
768.839987	335.662134	7.57543E-06	0.005824291
768.879987	324.906491	7.33269E-06	0.005637956
768.919987	345.445294	7.79622E-06	0.005994669
768.959987	417.011516	9.41137E-06	0.007236966
768.999987	593.392068	1.3392E-05	0.010298473
769.039987	833.16783	1.88034E-05	0.014460595
769.079987	1130.518021	2.55142E-05	0.019622474
769.119987	1428.341767	3.22357E-05	0.024793109
769.159987	1643.137072	3.70833E-05	0.028523002
769.199987	1766.213185	3.9861E-05	0.030661059
769.239987	1809.110887	4.08291E-05	0.031407386
769.279987	1707.409442	3.85339E-05	0.029643322
769.319987	1474.455426	3.32764E-05	0.025600204
769.359987	1144.406874	2.58277E-05	0.019870775
769.399987	799.52784	1.80442E-05	0.01388323
769.439987	568.888057	1.2839E-05	0.009878848
769.479987	416.10379	9.39088E-06	0.007226096
769.519987	352.417478	7.95357E-06	0.006120432
769.559987	330.386519	7.45636E-06	0.005738119
769.599987	336.682088	7.59845E-06	0.005847764
769.639987	363.515147	8.20403E-06	0.00631415
769.679987	399.336737	9.01247E-06	0.006936721
769.719987	392.957464	8.8685E-06	0.006826264
769.759987	357.705839	8.07292E-06	0.006214213
769.799987	323.096247	7.29183E-06	0.005613252
769.839987	288.189045	6.50403E-06	0.005007059
769.879987	276.390555	6.23775E-06	0.004802319
769.919987	307.93745	6.94972E-06	0.005350728
769.959987	348.415579	7.86325E-06	0.006054391
769.999987	407.347113	9.19326E-06	0.007078808
770.039987	534.507929	1.20631E-05	0.009289069
770.079987	756.12169	1.70646E-05	0.013141117

770.119987	978.549437	2.20845E-05	0.017007713
770.159987	1154.273979	2.60504E-05	0.020062942
770.199987	1292.835111	2.91775E-05	0.022472501
770.239987	1320.93329	2.98116E-05	0.022962105
770.279987	1226.436239	2.7679E-05	0.021320547
770.319987	1050.275254	2.37032E-05	0.018259087
770.359987	853.242754	1.92565E-05	0.014834437
770.399987	669.812374	1.51167E-05	0.01164593
770.439987	518.292043	1.16971E-05	0.009011936
770.479987	403.601139	9.10872E-06	0.007018083
770.519987	325.027296	7.33541E-06	0.005652083
770.559987	306.776415	6.92352E-06	0.005334985
770.599987	317.687583	7.16977E-06	0.005525022
770.639987	359.025177	8.1027E-06	0.006244263
770.679987	401.937501	9.07117E-06	0.006990969
770.719987	420.524225	9.49065E-06	0.007314631
770.759987	420.024585	9.47937E-06	0.007306319
770.799987	396.339974	8.94484E-06	0.006894683
770.839987	363.186309	8.19661E-06	0.006318274
770.879987	344.253387	7.76932E-06	0.005989213
770.919987	374.988003	8.46296E-06	0.006524263
770.959987	479.777475	1.08279E-05	0.008347885
770.999987	659.250091	1.48784E-05	0.011471213
771.039987	981.366023	2.21481E-05	0.017077043
771.079987	1423.873106	3.21348E-05	0.024778526
771.119987	1861.290228	4.20067E-05	0.032392227
771.159987	2206.799772	4.98044E-05	0.038407158
771.199987	2356.447729	5.31817E-05	0.041013759
771.239987	2324.124839	5.24523E-05	0.040453281
771.279987	2045.94451	4.61741E-05	0.035613173
771.319987	1603.060573	3.61788E-05	0.027905466
771.359987	1194.123567	2.69497E-05	0.020787925
771.399987	864.733188	1.95158E-05	0.015054506
771.439987	633.922747	1.43068E-05	0.011036802
771.479987	474.423526	1.07071E-05	0.008260297
771.519987	396.347179	8.945E-06	0.006901249
771.559987	331.057066	7.4715E-06	0.005764708
771.599987	313.870429	7.08362E-06	0.00546572
771.639987	362.958128	8.19146E-06	0.006320858
771.679987	409.657198	9.24539E-06	0.007134484
771.719987	405.923441	9.16113E-06	0.007069825
771.759987	387.254362	8.73979E-06	0.006745021
771.799987	347.431957	7.84106E-06	0.006051726
771.839987	309.676392	6.98896E-06	0.005394363
771.879987	303.953852	6.85982E-06	0.005294954
771.919987	304.117428	6.86351E-06	0.005298078

771.959987	352.606003	7.95783E-06	0.006143124
771.999987	481.000516	1.08555E-05	0.008380455
772.039987	653.946173	1.47587E-05	0.011394271
772.079987	864.355255	1.95073E-05	0.015061191
772.119987	1118.765638	2.5249E-05	0.019495244
772.159987	1462.321488	3.30026E-05	0.025483256
772.199987	1739.984629	3.9269E-05	0.030323544
772.239987	1836.901327	4.14563E-05	0.032014216
772.279987	1774.114525	4.00393E-05	0.030921545
772.319987	1564.069943	3.52989E-05	0.027262031
772.359987	1241.410967	2.80169E-05	0.021639145
772.399987	882.897772	1.99258E-05	0.015390667
772.439987	615.879959	1.38996E-05	0.01073657
772.479987	477.536709	1.07773E-05	0.008325278
772.519987	410.474616	9.26384E-06	0.007156502
772.559987	374.928368	8.46161E-06	0.006537102
772.599987	378.611831	8.54474E-06	0.006601667
772.639987	402.624649	9.08668E-06	0.00702073
772.679987	382.219319	8.62616E-06	0.006665259
772.719987	377.530907	8.52035E-06	0.006583842
772.759987	389.28681	8.78566E-06	0.006789207
772.799987	394.472045	8.90268E-06	0.006879995
772.839987	411.167259	9.27947E-06	0.007171547
772.879987	446.443928	1.00756E-05	0.007787243
772.919987	486.731307	1.09848E-05	0.008490408
772.959987	578.077679	1.30464E-05	0.010084351
772.999987	724.991887	1.63621E-05	0.01264787
773.039987	957.011462	2.15984E-05	0.016696439
773.079987	1285.319138	2.90079E-05	0.022425398
773.119987	1668.889258	3.76645E-05	0.029119182
773.159987	2010.520607	4.53746E-05	0.035081864
773.199987	2196.769698	4.9578E-05	0.038333734
773.239987	2217.241859	5.00401E-05	0.038692976
773.279987	2052.625952	4.63249E-05	0.035822125
773.319987	1700.740462	3.83833E-05	0.029682607
773.359987	1292.054981	2.91599E-05	0.022551085
773.399987	944.548979	2.13172E-05	0.016486687
773.439987	696.28368	1.57142E-05	0.012153954
773.479987	503.733435	1.13686E-05	0.008793355
773.519987	394.56868	8.90487E-06	0.006888091
773.559987	337.104451	7.60798E-06	0.005885227
773.599987	319.801936	7.21748E-06	0.005583446
773.639987	356.685847	8.0499E-06	0.006227727
773.679987	393.549787	8.88187E-06	0.006871725
773.719987	399.361899	9.01304E-06	0.006973571
773.759987	392.934009	8.86797E-06	0.006861683

773.799987	361.18467	8.15143E-06	0.00630758
773.839987	321.147253	7.24785E-06	0.005608673
773.879987	290.855642	6.56421E-06	0.005079908
773.919987	296.601488	6.69388E-06	0.005180529
773.959987	326.210875	7.36213E-06	0.00569799
773.999987	408.69353	9.22364E-06	0.0071391
774.039987	589.308261	1.32999E-05	0.010294629
774.079987	832.655769	1.87919E-05	0.014546419
774.119987	1073.176579	2.42201E-05	0.018749264
774.159987	1208.409504	2.72721E-05	0.021112984
774.199987	1291.377703	2.91446E-05	0.022563746
774.239987	1341.282293	3.02709E-05	0.02343692
774.279987	1282.553088	2.89454E-05	0.022411872
774.319987	1085.832635	2.45057E-05	0.018975277
774.359987	890.40098	2.00951E-05	0.015560849
774.399987	715.151959	1.614E-05	0.012498803
774.439987	555.054925	1.25268E-05	0.009701268
774.479987	420.774536	9.4963E-06	0.007354691
774.519987	357.819569	8.07549E-06	0.006254628
774.559987	364.757735	8.23207E-06	0.006376235
774.599987	395.488816	8.92563E-06	0.006913794
774.639987	428.981209	9.68151E-06	0.007499684
774.679987	451.356339	1.01865E-05	0.007891265
774.719987	464.461596	1.04823E-05	0.00812081
774.759987	438.692105	9.90067E-06	0.007670643
774.799987	410.349364	9.26101E-06	0.007175433
774.839987	403.672191	9.11032E-06	0.00705904
774.879987	400.409632	9.03669E-06	0.007002348
774.919987	410.465357	9.26363E-06	0.007178573
774.959987	451.445841	1.01885E-05	0.007895683
774.999987	618.043934	1.39484E-05	0.010810003
775.039987	874.329096	1.97324E-05	0.015293391
775.079987	1225.883941	2.76665E-05	0.021443745
775.119987	1623.021652	3.66293E-05	0.028392132
775.159987	1968.15145	4.44184E-05	0.034431395
775.199987	2210.518186	4.98883E-05	0.038673422
775.239987	2194.448874	4.95257E-05	0.038394268
775.279987	1967.509005	4.44039E-05	0.034425484
775.319987	1655.355867	3.73591E-05	0.028965238
775.359987	1279.920662	2.8886E-05	0.022397069
775.399987	926.673235	2.09137E-05	0.016216501
775.439987	647.389801	1.46107E-05	0.01132971
775.479987	466.913548	1.05376E-05	0.008171689
775.519987	362.982736	8.19201E-06	0.006353071
775.559987	331.019539	7.47065E-06	0.005793937
775.599987	333.074279	7.51702E-06	0.005830203

775.639987	363.438327	8.2023E-06	0.006362029
775.679987	414.326334	9.35077E-06	0.007253204
775.719987	407.793552	9.20333E-06	0.007139209
775.759987	347.219291	7.83626E-06	0.006079054
775.799987	304.526102	6.87273E-06	0.005331864
775.839987	286.039954	6.45552E-06	0.005008453
775.879987	274.429771	6.1935E-06	0.004805411
775.919987	279.989876	6.31898E-06	0.004903024
775.959987	336.549779	7.59546E-06	0.005893773
775.999987	435.665731	9.83237E-06	0.007629918
776.039987	585.805005	1.32208E-05	0.010259872
776.079987	835.410774	1.88541E-05	0.014632257
776.119987	1147.005086	2.58863E-05	0.02009088
776.159987	1422.432415	3.21023E-05	0.024916535
776.199987	1574.444207	3.5533E-05	0.027580724
776.239987	1512.117579	3.41264E-05	0.026490267
776.279987	1328.713562	2.99872E-05	0.023278474
776.319987	1148.514866	2.59204E-05	0.02012251
776.359987	950.524668	2.1452E-05	0.016654488
776.399987	720.2509	1.62551E-05	0.012620428
776.439987	510.083294	1.15119E-05	0.008938276
776.479987	392.794875	8.86483E-06	0.006883366
776.519987	370.704016	8.36627E-06	0.006496578
776.559987	363.50743	8.20386E-06	0.006370787
776.599987	368.421347	8.31476E-06	0.00645724
776.639987	383.484988	8.65472E-06	0.006721603
776.679987	404.216778	9.12261E-06	0.007085348
776.719987	419.717358	9.47244E-06	0.00735743
776.759987	429.449568	9.69208E-06	0.007528419
776.799987	407.518818	9.19713E-06	0.007144332
776.839987	364.103551	8.21731E-06	0.006383535
776.879987	382.933145	8.64227E-06	0.006714005
776.919987	408.180363	9.21206E-06	0.007157035
776.959987	467.641238	1.0554E-05	0.008200045
776.999987	624.322901	1.40901E-05	0.010948006
777.039987	870.516406	1.96463E-05	0.015265994
777.079987	1192.981241	2.69239E-05	0.020922043
777.119987	1601.629122	3.61465E-05	0.028090197
777.159987	1909.854793	4.31028E-05	0.033497742
777.199987	2020.254828	4.55943E-05	0.035435919
777.239987	1994.143292	4.5005E-05	0.034979714
777.279987	1860.853388	4.19969E-05	0.032643326
777.319987	1618.359844	3.65241E-05	0.028390934
777.359987	1252.078594	2.82577E-05	0.021966382
777.399987	907.626912	2.04839E-05	0.015924164
777.439987	637.493571	1.43873E-05	0.011185295

777.479987	441.134574	9.95579E-06	0.00774043
777.519987	350.547453	7.91137E-06	0.006151247
777.559987	331.455548	7.48049E-06	0.00581653
777.599987	342.479046	7.72928E-06	0.006010284
777.639987	358.329856	8.08701E-06	0.006288779
777.679987	384.012435	8.66663E-06	0.006739861
777.719987	387.981528	8.7562E-06	0.006809874
777.759987	353.718908	7.98294E-06	0.006208814
777.799987	334.967251	7.55974E-06	0.005879969
777.839987	316.523719	7.1435E-06	0.0055565
777.879987	310.250718	7.00193E-06	0.005446659
777.919987	342.133483	7.72148E-06	0.006006691
777.959987	382.521058	8.63297E-06	0.006716103
777.999987	465.688247	1.05099E-05	0.008176729
778.039987	607.934713	1.37202E-05	0.010674895
778.079987	811.563828	1.83159E-05	0.014251208
778.119987	1084.173765	2.44683E-05	0.019039267
778.159987	1337.880088	3.01941E-05	0.023495833
778.199987	1448.522314	3.26911E-05	0.025440238
778.239987	1458.824785	3.29236E-05	0.025622496
778.279987	1420.486786	3.20584E-05	0.024950418
778.319987	1232.529643	2.78165E-05	0.02165012
778.359987	980.129644	2.21202E-05	0.017217448
778.399987	715.003623	1.61366E-05	0.012560757
778.439987	504.180025	1.13786E-05	0.008857589
778.479987	398.629386	8.99651E-06	0.007003603
778.519987	355.427209	8.0215E-06	0.006244896
778.559987	343.730527	7.75752E-06	0.006039694
778.599987	359.405276	8.11128E-06	0.00631544
778.639987	390.090282	8.80379E-06	0.006854986
778.679987	426.279218	9.62053E-06	0.007491313
778.719987	443.084301	9.9998E-06	0.007787041
778.759987	418.684949	9.44914E-06	0.007358609
778.799987	371.418151	8.38239E-06	0.006528205
778.839987	341.08286	7.69777E-06	0.005995327
778.879987	350.759183	7.91615E-06	0.006165728
778.919987	369.01999	8.32827E-06	0.006487054
778.959987	451.317034	1.01856E-05	0.007934172
778.999987	625.816149	1.41238E-05	0.011002439
779.039987	848.96368	1.91599E-05	0.01492635
779.079987	1153.940802	2.60428E-05	0.020289453
779.119987	1443.25173	3.25722E-05	0.025377637
779.159987	1639.858011	3.70093E-05	0.028836174
779.199987	1752.814938	3.95586E-05	0.030824054
779.239987	1721.893746	3.88607E-05	0.030281845
779.279987	1513.359178	3.41544E-05	0.026615847

779.319987	1203.53302	2.71621E-05	0.021167939
779.359987	895.837492	2.02178E-05	0.015756948
779.399987	657.771575	1.4845E-05	0.011570184
779.439987	480.441624	1.08429E-05	0.008451388
779.479987	375.076755	8.46496E-06	0.006598267
779.519987	319.374026	7.20783E-06	0.005618645
779.559987	307.979494	6.95067E-06	0.005418463
779.599987	349.644158	7.89098E-06	0.006151809
779.639987	384.152043	8.66978E-06	0.006759304
779.679987	368.795221	8.32319E-06	0.006489428
779.719987	362.085737	8.17177E-06	0.006371693
779.759987	374.549985	8.45307E-06	0.006591367
779.799987	361.830218	8.166E-06	0.00636785
779.839987	339.740946	7.66748E-06	0.005979407
779.879987	327.568825	7.39277E-06	0.005765475
779.919987	323.753203	7.30666E-06	0.005698609
779.959987	366.026414	8.26071E-06	0.00644302
779.999987	447.051693	1.00893E-05	0.00786968
780.039987	597.642968	1.3488E-05	0.010521155
780.079987	812.943756	1.8347E-05	0.014312134
780.119987	1033.406277	2.33225E-05	0.01819438
780.159987	1200.852925	2.71016E-05	0.021143567
780.199987	1274.433649	2.87622E-05	0.022440262
780.239987	1209.257162	2.72912E-05	0.021293724
780.279987	1094.271985	2.46962E-05	0.019269946
780.319987	955.827664	2.15717E-05	0.016832828
780.359987	760.708506	1.71681E-05	0.013397323
780.399987	563.715631	1.27223E-05	0.009928464
780.439987	412.014411	9.29859E-06	0.007256993
780.479987	339.754456	7.66778E-06	0.005984553
780.519987	323.331588	7.29714E-06	0.005695566
780.559987	349.735797	7.89305E-06	0.006160999
780.599987	372.870518	8.41517E-06	0.00656888
780.639987	390.069571	8.80333E-06	0.006872229
780.679987	430.260308	9.71038E-06	0.007580696
780.719987	430.240737	9.70993E-06	0.00758074
780.759987	395.599461	8.92813E-06	0.006970726
780.799987	365.580341	8.25064E-06	0.006442099
780.839987	349.673851	7.89165E-06	0.006162117
780.879987	361.351331	8.1552E-06	0.00636823
780.919987	398.682527	8.99771E-06	0.007026491
780.959987	496.850013	1.12132E-05	0.00875707
780.999987	655.482966	1.47933E-05	0.011553596
781.039987	882.463792	1.9916E-05	0.015555176
781.079987	1183.153242	2.67021E-05	0.020856492
781.119987	1463.390995	3.30267E-05	0.025797813

781.159987	1649.002822	3.72157E-05	0.029071413
781.199987	1725.233801	3.89361E-05	0.030416899
781.239987	1694.396686	3.82402E-05	0.029874752
781.279987	1531.029695	3.45532E-05	0.02699573
781.319987	1239.686816	2.7978E-05	0.021859774
781.359987	953.854107	2.15272E-05	0.01682046
781.399987	700.048621	1.57991E-05	0.012345434
781.439987	502.239198	1.13348E-05	0.008857497
781.479987	373.7751	8.43558E-06	0.00659224
781.519987	330.625881	7.46177E-06	0.005831519
781.559987	325.570814	7.34768E-06	0.005742653
781.599987	344.144282	7.76686E-06	0.006070575
781.639987	387.770094	8.75143E-06	0.006840468
781.679987	414.919126	9.36415E-06	0.007319766
781.719987	390.451496	8.81195E-06	0.006888475
781.759987	359.44608	8.1122E-06	0.006341791
781.799987	351.927316	7.94251E-06	0.006209454
781.839987	324.414112	7.32157E-06	0.0057243
781.879987	295.414439	6.66709E-06	0.005212866
781.919987	288.092369	6.50184E-06	0.005083921
781.959987	329.747264	7.44194E-06	0.005819297
781.999987	426.325454	9.62157E-06	0.007524069
782.039987	605.215248	1.36589E-05	0.010681779
782.079987	836.552346	1.88798E-05	0.01476553
782.119987	1051.345882	2.37274E-05	0.018557683
782.159987	1180.79317	2.66489E-05	0.02084367
782.199987	1221.028136	2.75569E-05	0.02155501
782.239987	1209.543833	2.72977E-05	0.021353368
782.279987	1122.103295	2.53243E-05	0.0198107
782.319987	946.577127	2.13629E-05	0.016712645
782.359987	776.93138	1.75343E-05	0.013718103
782.399987	612.06603	1.38135E-05	0.010807665
782.439987	447.252679	1.00939E-05	0.007897848
782.479987	358.699488	8.09535E-06	0.006334448
782.519987	336.014934	7.58339E-06	0.005934153
782.559987	333.091991	7.51742E-06	0.005882834
782.599987	353.805999	7.98491E-06	0.006248989
782.639987	400.14257	9.03066E-06	0.007067756
782.679987	440.346145	9.938E-06	0.007778273
782.719987	426.526073	9.6261E-06	0.00753454
782.759987	370.704003	8.36627E-06	0.006548784
782.799987	358.459885	8.08994E-06	0.006332805
782.839987	382.431393	8.63094E-06	0.006756648
782.879987	387.130135	8.73699E-06	0.006840013
782.919987	428.115325	9.66197E-06	0.007564547
782.959987	519.150733	1.17165E-05	0.009173558

782.999987	613.696623	1.38503E-05	0.010844767
783.039987	789.401215	1.78157E-05	0.013950394
783.079987	1097.112387	2.47603E-05	0.019389294
783.119987	1425.463416	3.21707E-05	0.025193537
783.159987	1666.373679	3.76077E-05	0.029452872
783.199987	1780.062493	4.01735E-05	0.031463909
783.239987	1780.112929	4.01747E-05	0.031466407
783.279987	1598.875145	3.60844E-05	0.028264177
783.319987	1277.044923	2.88211E-05	0.022576163
783.359987	963.710905	2.17496E-05	0.017037776
783.399987	701.521262	1.58324E-05	0.012403069
783.439987	518.195563	1.1695E-05	0.009162293
783.479987	387.083846	8.73594E-06	0.006844437
783.519987	314.750534	7.10348E-06	0.005565719
783.559987	303.531816	6.85029E-06	0.005367613
783.599987	318.658578	7.19168E-06	0.005635401
783.639987	353.539777	7.9789E-06	0.006252585
783.679987	368.868533	8.32485E-06	0.006524018
783.719987	368.011691	8.30551E-06	0.006509195
783.759987	357.958027	8.07861E-06	0.006331694
783.799987	341.306455	7.70281E-06	0.006037463
783.839987	303.429233	6.84798E-06	0.005367717
783.879987	311.732422	7.03537E-06	0.005514883
783.919987	355.43205	8.02161E-06	0.006288298
783.959987	368.168661	8.30905E-06	0.006513966
783.999987	418.685211	9.44914E-06	0.007408127
784.039987	559.517446	1.26275E-05	0.009900488
784.079987	771.091308	1.74025E-05	0.013644918
784.119987	960.867052	2.16854E-05	0.01700398
784.159987	1116.547744	2.51989E-05	0.019759991
784.199987	1205.79854	2.72132E-05	0.021340586
784.239987	1195.4759	2.69802E-05	0.021158972
784.279987	1130.955331	2.55241E-05	0.02001803
784.319987	1013.222025	2.2867E-05	0.017935053
784.359987	867.278672	1.95733E-05	0.015352491
784.399987	737.685276	1.66485E-05	0.013059106
784.439987	579.634467	1.30815E-05	0.010261685
784.479987	456.505748	1.03027E-05	0.008082261
784.519987	409.077458	9.23231E-06	0.00724293
784.559987	395.790176	8.93243E-06	0.00700803
784.599987	407.060239	9.18678E-06	0.00720795
784.639987	431.379719	9.73564E-06	0.007638972
784.679987	451.881431	1.01983E-05	0.008002429
784.719987	437.951822	9.88396E-06	0.007756143
784.759987	395.54134	8.92682E-06	0.007005409
784.799987	348.66844	7.86896E-06	0.006175561

784.839987	326.394615	7.36627E-06	0.005781345
784.879987	329.37599	7.43356E-06	0.00583445
784.919987	379.107834	8.55594E-06	0.006715725
784.959987	461.312253	1.04112E-05	0.008172356
784.999987	590.226609	1.33206E-05	0.010456665
785.039987	762.86535	1.72168E-05	0.013515883
785.079987	1013.646944	2.28766E-05	0.017959961
785.119987	1329.64981	3.00083E-05	0.023560151
785.159987	1580.10616	3.56608E-05	0.027999429
785.199987	1685.219722	3.80331E-05	0.02986356
785.239987	1696.526339	3.82882E-05	0.030065454
785.279987	1619.326237	3.65459E-05	0.028698793
785.319987	1363.171769	3.07649E-05	0.024160282
785.359987	1004.253391	2.26646E-05	0.01779987
785.399987	734.292027	1.65719E-05	0.013015608
785.439987	539.449513	1.21746E-05	0.009562437
785.479987	424.04805	9.57017E-06	0.00751718
785.519987	348.881459	7.87377E-06	0.006185003
785.559987	297.608378	6.71661E-06	0.005276297
785.599987	289.09658	6.52451E-06	0.005125653
785.639987	304.066501	6.86236E-06	0.005391342
785.679987	333.504656	7.52674E-06	0.005913605
785.719987	362.490114	8.1809E-06	0.006427894
785.759987	362.782048	8.18749E-06	0.006433399
785.799987	339.95377	7.67228E-06	0.00602888
785.839987	319.423303	7.20894E-06	0.005665073
785.879987	288.367514	6.50805E-06	0.005114549
785.919987	285.275093	6.43826E-06	0.005059958
785.959987	313.094218	7.0661E-06	0.005553672
785.999987	370.299014	8.35713E-06	0.006568706
786.039987	497.254566	1.12223E-05	0.00882121
786.079987	684.782099	1.54546E-05	0.012148535
786.119987	852.891591	1.92486E-05	0.01513169
786.159987	1012.615509	2.28533E-05	0.017966367
786.199987	1126.817097	2.54307E-05	0.01999361
786.239987	1185.124652	2.67466E-05	0.021029256
786.279987	1143.258319	2.58017E-05	0.020287398
786.319987	994.342937	2.24409E-05	0.017645756
786.359987	797.147355	1.79905E-05	0.014147014
786.399987	628.726801	1.41895E-05	0.011158613
786.439987	470.512715	1.06188E-05	0.008351062
786.479987	366.398488	8.2691E-06	0.006503484
786.519987	328.578412	7.41556E-06	0.005832484
786.559987	358.743778	8.09635E-06	0.006368263
786.599987	392.390162	8.8557E-06	0.006965893
786.639987	431.421435	9.73658E-06	0.007659184

786.679987	502.238937	1.13348E-05	0.008916886
786.719987	497.226194	1.12217E-05	0.008828338
786.759987	449.004386	1.01334E-05	0.007972556
786.799987	398.878082	9.00212E-06	0.00708287
786.839987	373.212587	8.42289E-06	0.006627465
786.879987	409.684254	9.246E-06	0.007275495
786.919987	461.202111	1.04087E-05	0.008190806
786.959987	519.906215	1.17336E-05	0.009233842
786.999987	664.24149	1.4991E-05	0.011797921
787.039987	909.25793	2.05207E-05	0.0161506
787.079987	1232.263346	2.78105E-05	0.021889062
787.119987	1494.381615	3.37261E-05	0.026546496
787.159987	1717.530396	3.87623E-05	0.030512107
787.199987	1869.975882	4.22028E-05	0.033222005
787.239987	1892.026897	4.27004E-05	0.033615472
787.279987	1691.986322	3.81858E-05	0.030062896
787.319987	1363.73311	3.07776E-05	0.024231786
787.359987	1041.055781	2.34952E-05	0.018499164
787.399987	777.196752	1.75402E-05	0.013811191
787.439987	580.073036	1.30914E-05	0.010308724
787.479987	425.41139	9.60094E-06	0.00756055
787.519987	348.788853	7.87168E-06	0.006199104
787.559987	312.67023	7.05653E-06	0.005557442
787.599987	314.748473	7.10343E-06	0.005594665
787.639987	346.353414	7.81671E-06	0.006156757
787.679987	381.774175	8.61611E-06	0.006786738
787.719987	389.630739	8.79342E-06	0.006926755
787.759987	356.881688	8.05432E-06	0.006344873
787.799987	324.226094	7.31733E-06	0.005764594
787.839987	281.097328	6.34398E-06	0.004998037
787.879987	256.271194	5.78368E-06	0.004556849
787.919987	275.898417	6.22664E-06	0.004906096
787.959987	332.483716	7.50369E-06	0.005912611
787.999987	415.504949	9.37737E-06	0.007389366
788.039987	574.908296	1.29749E-05	0.010224724
788.079987	830.585478	1.87452E-05	0.014772683
788.119987	1067.591511	2.40941E-05	0.018989005
788.159987	1231.324966	2.77893E-05	0.021902406
788.199987	1288.557722	2.9081E-05	0.022921607
788.239987	1277.002703	2.88202E-05	0.022717212
788.279987	1175.571233	2.6531E-05	0.020913861
788.319987	1011.191518	2.28212E-05	0.017990396
788.359987	830.165734	1.87357E-05	0.014770464
788.399987	634.36819	1.43168E-05	0.01128737
788.439987	482.748111	1.0895E-05	0.008590016
788.479987	384.363483	8.67455E-06	0.006839708

788.519987	349.564223	7.88918E-06	0.006220774
788.559987	369.070475	8.32941E-06	0.006568237
788.599987	373.344061	8.42586E-06	0.006644629
788.639987	373.620433	8.43209E-06	0.006649886
788.679987	419.874998	9.47599E-06	0.007473527
788.719987	454.684319	1.02616E-05	0.008093523
788.759987	427.961447	9.65849E-06	0.007618233
788.799987	424.291434	9.57567E-06	0.007553286
788.839987	418.558756	9.44629E-06	0.00745161
788.879987	413.07717	9.32258E-06	0.007354394
788.919987	444.498844	1.00317E-05	0.007914224
788.959987	531.092323	1.1986E-05	0.009456485
788.999987	684.752504	1.54539E-05	0.012193135
789.039987	924.955449	2.0875E-05	0.016471175
789.079987	1238.111917	2.79425E-05	0.022048837
789.119987	1531.844503	3.45716E-05	0.027281138
789.159987	1736.612956	3.91929E-05	0.030929496
789.199987	1801.606494	4.06597E-05	0.032088673
789.239987	1749.820055	3.9491E-05	0.031167877
789.279987	1591.56486	3.59194E-05	0.028350465
789.319987	1355.793223	3.05984E-05	0.024151901
789.359987	1105.310897	2.49453E-05	0.019690843
789.399987	871.945489	1.96786E-05	0.015534283
789.439987	637.751866	1.43932E-05	0.011362545
789.479987	455.428395	1.02784E-05	0.008114579
789.519987	356.233141	8.03969E-06	0.006347493
789.559987	335.418837	7.56994E-06	0.005976919
789.599987	365.314436	8.24464E-06	0.006509966
789.639987	402.213364	9.0774E-06	0.007167874
789.679987	407.679106	9.20075E-06	0.007265648
789.719987	403.628298	9.10933E-06	0.007193819
789.759987	394.976577	8.91407E-06	0.007039977
789.799987	379.114278	8.55608E-06	0.006757593
789.839987	357.600965	8.07056E-06	0.006374448
789.879987	317.517407	7.16593E-06	0.005660221
789.919987	313.335188	7.07154E-06	0.00558595
789.959987	341.762982	7.71311E-06	0.006093052
789.999987	419.256666	9.46204E-06	0.007475011
790.039987	577.198887	1.30266E-05	0.010291515
790.079987	784.558205	1.77064E-05	0.013989461
790.119987	984.228949	2.22127E-05	0.01755068
790.159987	1142.993086	2.57958E-05	0.020382779
790.199987	1219.399537	2.75201E-05	0.021746421
790.239987	1238.12992	2.79429E-05	0.022081571
790.279987	1183.515656	2.67103E-05	0.021108615
790.319987	1015.93402	2.29282E-05	0.018120627

790.359987	817.457982	1.84489E-05	0.014581263
790.399987	648.738139	1.46411E-05	0.011572338
790.439987	543.646829	1.22694E-05	0.009698186
790.479987	474.141544	1.07007E-05	0.0084587
790.519987	414.040422	9.34432E-06	0.007386868
790.559987	373.821053	8.43662E-06	0.006669655
790.599987	372.687992	8.41105E-06	0.006649775
790.639987	397.014807	8.96007E-06	0.007084191
790.679987	438.627806	9.89922E-06	0.007827114
790.719987	443.830475	1.00166E-05	0.007920354
790.759987	412.601025	9.31183E-06	0.007363423
790.799987	368.444077	8.31527E-06	0.006575715
790.839987	344.374468	7.77205E-06	0.00614645
790.879987	343.02474	7.74159E-06	0.006122669
790.919987	384.879937	8.6862E-06	0.006870092
790.959987	458.91849	1.03572E-05	0.008192092
790.999987	635.813167	1.43494E-05	0.011350389
791.039987	927.730142	2.09376E-05	0.016562461
791.079987	1251.982595	2.82555E-05	0.022352363
791.119987	1522.434641	3.43592E-05	0.027182273
791.159987	1740.319606	3.92766E-05	0.031074066
791.199987	1773.527548	4.0026E-05	0.031668607
791.239987	1672.52661	3.77466E-05	0.029866616
791.279987	1457.763757	3.28997E-05	0.026032872
791.319987	1215.132763	2.74239E-05	0.021701044
791.359987	970.019896	2.1892E-05	0.017324451
791.399987	694.268302	1.56687E-05	0.012400184
791.439987	483.506641	1.09121E-05	0.00863625
791.479987	375.690937	8.47882E-06	0.006710817
791.519987	344.606188	7.77728E-06	0.006155874
791.559987	347.252647	7.83701E-06	0.006203462
791.599987	355.542478	8.0241E-06	0.006351876
791.639987	375.257145	8.46903E-06	0.006704424
791.679987	404.659821	9.13261E-06	0.007230103
791.719987	422.919396	9.5447E-06	0.007556731
791.759987	410.033468	9.25388E-06	0.007326855
791.799987	375.245361	8.46877E-06	0.006705568
791.839987	346.923706	7.82958E-06	0.006199778
791.879987	321.761375	7.26171E-06	0.0057504
791.919987	329.755163	7.44211E-06	0.005893559
791.959987	377.240348	8.51379E-06	0.00674258
791.999987	449.579927	1.01464E-05	0.008035943
792.039987	571.209064	1.28914E-05	0.010210499
792.079987	738.510354	1.66672E-05	0.013201716
792.119987	945.411663	2.13366E-05	0.016901167
792.159987	1166.044825	2.6316E-05	0.020846488

792.199987	1325.56046	2.99161E-05	0.023699497
792.239987	1310.809008	2.95831E-05	0.023436941
792.279987	1159.553839	2.61695E-05	0.020733583
792.319987	986.124687	2.22555E-05	0.017633446
792.359987	809.220154	1.8263E-05	0.014470848
792.399987	637.698114	1.4392E-05	0.011404187
792.439987	496.966729	1.12158E-05	0.008887886
792.479987	404.908619	9.13822E-06	0.007241859
792.519987	346.002772	7.8088E-06	0.006188631
792.559987	341.739227	7.71258E-06	0.006112681
792.599987	374.277776	8.44693E-06	0.006695035
792.639987	408.953	9.2295E-06	0.00731567
792.679987	421.648534	9.51602E-06	0.007543159
792.719987	416.677909	9.40384E-06	0.007454612
792.759987	385.333685	8.69644E-06	0.006894193
792.799987	378.560872	8.54359E-06	0.006773359
792.839987	384.065203	8.66782E-06	0.006872192
792.879987	388.952763	8.77812E-06	0.006959997
792.919987	377.622275	8.52241E-06	0.006757588
792.959987	408.22648	9.2131E-06	0.007305622
792.999987	529.370971	1.19472E-05	0.009474102
793.039987	714.429272	1.61237E-05	0.012786718
793.079987	1022.215815	2.307E-05	0.018296345
793.119987	1360.152859	3.06968E-05	0.02434621
793.159987	1540.685854	3.47711E-05	0.027579072
793.199987	1532.207262	3.45798E-05	0.027428684
793.239987	1470.585652	3.31891E-05	0.026326897
793.279987	1363.840412	3.078E-05	0.024417141
793.319987	1185.931941	2.67648E-05	0.021233076
793.359987	958.283234	2.16271E-05	0.01715809
793.399987	744.950573	1.68125E-05	0.013339035
793.439987	548.195561	1.2372E-05	0.009816448
793.479987	412.058455	9.29959E-06	0.007379035
793.519987	361.855405	8.16657E-06	0.006480338
793.559987	337.110699	7.60812E-06	0.006037499
793.599987	318.098888	7.17905E-06	0.005697293
793.639987	349.491458	7.88754E-06	0.006259863
793.679987	370.629328	8.36459E-06	0.006638806
793.719987	363.261096	8.1983E-06	0.006507152
793.759987	340.436186	7.68317E-06	0.006098593
793.799987	317.149101	7.15761E-06	0.005681713
793.839987	298.034419	6.72622E-06	0.005339544
793.879987	289.071	6.52393E-06	0.005179217
793.919987	294.074239	6.63685E-06	0.005269125
793.959987	344.335102	7.77116E-06	0.006169993
793.999987	434.509216	9.80627E-06	0.007786176

794.039987	560.703701	1.26543E-05	0.010048021
794.079987	729.476811	1.64633E-05	0.013073158
794.119987	938.020261	2.11698E-05	0.01681137
794.159987	1107.77719	2.5001E-05	0.019854784
794.199987	1210.698122	2.73238E-05	0.021700538
794.239987	1183.527426	2.67106E-05	0.021214599
794.279987	1079.824781	2.43701E-05	0.019356715
794.319987	930.321548	2.09961E-05	0.016677591
794.359987	763.255093	1.72256E-05	0.013683331
794.399987	592.45375	1.33709E-05	0.010621808
794.439987	467.811389	1.05579E-05	0.008387579
794.479987	383.540691	8.65598E-06	0.006877002
794.519987	325.594401	7.34821E-06	0.005838301
794.559987	330.139612	7.45079E-06	0.005920101
794.599987	375.601635	8.47681E-06	0.00673567
794.639987	412.188495	9.30252E-06	0.007392155
794.679987	428.184465	9.66353E-06	0.007679411
794.719987	423.820224	9.56503E-06	0.007601522
794.759987	404.971762	9.13965E-06	0.007263827
794.799987	378.589709	8.54424E-06	0.006790964
794.839987	359.247944	8.10773E-06	0.006444345
794.879987	371.913702	8.39357E-06	0.006671884
794.919987	392.327859	8.85429E-06	0.007038455
794.959987	406.684146	9.17829E-06	0.007296377
794.999987	490.982252	1.10808E-05	0.008809224
795.039987	676.221252	1.52614E-05	0.012133401
795.079987	935.625031	2.11158E-05	0.016788713
795.119987	1209.286978	2.72919E-05	0.021700353
795.159987	1424.899237	3.2158E-05	0.025570748
795.199987	1511.595985	3.41146E-05	0.027127942
795.239987	1496.1946	3.3767E-05	0.026852891
795.279987	1380.795406	3.11626E-05	0.024783015
795.319987	1162.184439	2.62289E-05	0.020860356
795.359987	915.969174	2.06721E-05	0.0164418
795.399987	662.921506	1.49612E-05	0.01190015
795.439987	479.99376	1.08328E-05	0.008616835
795.479987	386.218667	8.71642E-06	0.006933736
795.519987	328.129045	7.40542E-06	0.005891156
795.559987	306.531607	6.91799E-06	0.005503677
795.599987	336.506008	7.59447E-06	0.006042162
795.639987	373.226285	8.4232E-06	0.006701833
795.679987	397.425492	8.96934E-06	0.007136724
795.719987	401.007233	9.05017E-06	0.007201405
795.759987	379.686896	8.569E-06	0.006818871
795.799987	349.551352	7.88889E-06	0.006277976
795.839987	321.488512	7.25555E-06	0.005774255

795.879987	287.738884	6.49387E-06	0.005168338
795.919987	281.903857	6.36218E-06	0.005063784
795.959987	323.294871	7.29631E-06	0.005807575
795.999987	401.740986	9.06673E-06	0.007217121
796.039987	523.749646	1.18203E-05	0.009409431
796.079987	675.936737	1.5255E-05	0.012144161
796.119987	860.106964	1.94114E-05	0.015453816
796.159987	1023.401654	2.30967E-05	0.018388708
796.199987	1130.596275	2.5516E-05	0.020315825
796.239987	1137.959584	2.56822E-05	0.020449165
796.279987	1036.791713	2.33989E-05	0.018632111
796.319987	862.456602	1.94644E-05	0.015499926
796.359987	675.306085	1.52407E-05	0.012137098
796.399987	541.887787	1.22297E-05	0.009739695
796.439987	437.94256	9.88375E-06	0.007871817
796.479987	368.620778	8.31926E-06	0.006626122
796.519987	308.900341	6.97145E-06	0.0055529
796.559987	300.379642	6.77915E-06	0.0054
796.599987	329.34749	7.43291E-06	0.005921059
796.639987	374.402714	8.44975E-06	0.006731407
796.679987	429.508248	9.6934E-06	0.00772254
796.719987	409.425376	9.24016E-06	0.007361821
796.759987	357.860464	8.07641E-06	0.006434962
796.799987	354.893746	8.00946E-06	0.006381936
796.839987	375.36361	8.47143E-06	0.006750377
796.879987	382.37735	8.62972E-06	0.006876854
796.919987	371.943881	8.39426E-06	0.00668955
796.959987	390.379597	8.81032E-06	0.007021476
796.999987	499.661798	1.12767E-05	0.008987506
797.039987	699.85421	1.57947E-05	0.012589035
797.079987	959.458093	2.16536E-05	0.017259677
797.119987	1225.358943	2.76546E-05	0.02204407
797.159987	1416.791986	3.1975E-05	0.025489208
797.199987	1507.802648	3.4029E-05	0.027127923
797.239987	1489.13657	3.36077E-05	0.026793433
797.279987	1330.124891	3.00191E-05	0.023933601
797.319987	1118.9856	2.52539E-05	0.020135476
797.359987	919.179284	2.07446E-05	0.016540911
797.399987	727.835307	1.64262E-05	0.013098275
797.439987	539.804549	1.21826E-05	0.009714922
797.479987	394.379605	8.9006E-06	0.007098049
797.519987	334.088002	7.5399E-06	0.006013222
797.559987	300.47311	6.78126E-06	0.005408461
797.599987	308.025612	6.95171E-06	0.005544683
797.639987	357.778066	8.07455E-06	0.006440586
797.679987	394.478373	8.90283E-06	0.007101607

797.719987	438.254793	9.8908E-06	0.007890089
797.759987	445.625818	1.00572E-05	0.008023195
797.799987	390.751722	8.81872E-06	0.007035576
797.839987	323.061706	7.29105E-06	0.005817093
797.879987	301.451899	6.80335E-06	0.005428256
797.919987	324.818812	7.33071E-06	0.005849319
797.959987	360.463174	8.13515E-06	0.006491526
797.999987	427.871311	9.65646E-06	0.007705855
798.039987	557.194774	1.25751E-05	0.010035441
798.079987	703.081172	1.58676E-05	0.012663584
798.119987	838.445498	1.89225E-05	0.015102463
798.159987	998.72213	2.25398E-05	0.01799034
798.199987	1112.575897	2.51093E-05	0.020042234
798.239987	1137.697155	2.56762E-05	0.020495802
798.279987	1043.20783	2.35437E-05	0.018794503
798.319987	864.966211	1.95211E-05	0.01558407
798.359987	675.811855	1.52521E-05	0.012176692
798.399987	522.972819	1.18028E-05	0.00942333
798.439987	443.985891	1.00201E-05	0.008000483
798.479987	378.863891	8.55043E-06	0.006827347
798.519987	332.182912	7.49691E-06	0.005986429
798.559987	338.516694	7.63985E-06	0.006100879
798.599987	374.691965	8.45628E-06	0.006753182
798.639987	381.317775	8.60581E-06	0.006872945
798.679987	382.800867	8.63928E-06	0.006900022
798.719987	371.796259	8.39092E-06	0.006701998
798.759987	352.893739	7.96432E-06	0.00636158
798.799987	353.672787	7.9819E-06	0.006375943
798.839987	365.279303	8.24385E-06	0.006585513
798.879987	361.576548	8.16028E-06	0.006519084
798.919987	346.155832	7.81225E-06	0.006241367
798.959987	385.821453	8.70745E-06	0.006956906
798.999987	532.288013	1.2013E-05	0.009598386
799.039987	737.776952	1.66506E-05	0.013304494
799.079987	992.810022	2.24063E-05	0.017904457
799.119987	1265.877158	2.85691E-05	0.022830126
799.159987	1506.846431	3.40074E-05	0.027177373
799.199987	1698.807131	3.83397E-05	0.030641096
799.239987	1792.098847	4.04452E-05	0.032325401
799.279987	1678.832361	3.78889E-05	0.030283846
799.319987	1404.554291	3.16988E-05	0.025337512
799.359987	1082.486932	2.44302E-05	0.019528542
799.399987	772.772541	1.74404E-05	0.013941855
799.439987	555.850613	1.25448E-05	0.010028794
799.479987	408.536253	9.22009E-06	0.007371281
799.519987	313.800598	7.08204E-06	0.005662234

799.559987	268.227817	6.05353E-06	0.004840159
799.599987	292.748099	6.60692E-06	0.005282891
799.639987	367.410675	8.29195E-06	0.006630573
799.679987	394.502522	8.90337E-06	0.007119849
799.719987	421.879784	9.52124E-06	0.007614325
799.759987	422.544552	9.53624E-06	0.007626705
799.799987	390.648849	8.8164E-06	0.007051357
799.839987	365.761183	8.25472E-06	0.006602455
799.879987	327.670854	7.39507E-06	0.005915172
799.919987	298.556934	6.73801E-06	0.005389872
799.959987	313.198081	7.06844E-06	0.005654473
799.999987	375.528039	8.47514E-06	0.006780116
800.039987	493.379324	1.11349E-05	0.008908353
800.079987	651.27734	1.46984E-05	0.011759914
800.119987	878.380722	1.98238E-05	0.015861442
800.159987	1079.763356	2.43688E-05	0.019498903
800.199987	1167.110553	2.63401E-05	0.021077315
800.239987	1178.500615	2.65971E-05	0.021284077
800.279987	1123.923906	2.53654E-05	0.02029942
800.319987	959.062904	2.16447E-05	0.017322697
800.359987	758.105373	1.71094E-05	0.013693665
800.399987	567.472475	1.28071E-05	0.010250773
800.439987	422.201361	9.5285E-06	0.00762699
800.479987	351.206639	7.92624E-06	0.0063448
800.519987	339.657627	7.6656E-06	0.006136466
800.559987	348.108281	7.85632E-06	0.006289455
800.599987	377.757476	8.52546E-06	0.006825483
800.639987	426.597979	9.62772E-06	0.007708339
800.679987	428.631228	9.67361E-06	0.007745466
800.719987	427.884392	9.65675E-06	0.007732357
800.759987	450.844771	1.01749E-05	0.008147684
800.799987	442.951132	9.99679E-06	0.008005429
800.839987	417.13129	9.41407E-06	0.007539165
800.879987	399.400841	9.01392E-06	0.007219069
800.919987	405.261867	9.1462E-06	0.007325371
800.959987	493.921297	1.11471E-05	0.008928394
800.999987	662.404166	1.49495E-05	0.011974581
801.039987	868.085122	1.95915E-05	0.015693553
801.079987	1132.091198	2.55497E-05	0.020467371
801.119987	1452.390102	3.27784E-05	0.026259449
801.159987	1773.544505	4.00264E-05	0.032067573
801.199987	1974.644326	4.4565E-05	0.035705455
801.239987	2004.828826	4.52462E-05	0.03625306
801.279987	1812.837338	4.09132E-05	0.032782939
801.319987	1421.958412	3.20916E-05	0.025715657
801.359987	1026.907567	2.31759E-05	0.018572218

801.399987	721.701701	1.62878E-05	0.013053044
801.439987	552.464871	1.24684E-05	0.009992644
801.479987	443.074404	9.99957E-06	0.008014457
801.519987	341.511843	7.70745E-06	0.006177673
801.559987	310.22127	7.00126E-06	0.005611931
801.599987	340.984032	7.69553E-06	0.00616874
801.639987	398.042385	8.98326E-06	0.007201342
801.679987	421.339678	9.50905E-06	0.007623215
801.719987	381.536698	8.61075E-06	0.006903412
801.759987	334.299643	7.54468E-06	0.00604902
801.799987	314.720719	7.10281E-06	0.005695032
801.839987	294.501329	6.64648E-06	0.005329417
801.879987	302.704767	6.83162E-06	0.005478143
801.919987	308.29482	6.95778E-06	0.005579587
801.959987	341.982744	7.71807E-06	0.006189587
801.999987	432.800807	9.76771E-06	0.007833704
802.039987	544.638435	1.22917E-05	0.00985846
802.079987	757.494224	1.70956E-05	0.01371203
802.119987	1004.644391	2.26734E-05	0.018186807
802.159987	1212.551174	2.73656E-05	0.021951582
802.199987	1293.978886	2.92033E-05	0.023426888
802.239987	1317.439092	2.97328E-05	0.023852813
802.279987	1267.870637	2.86141E-05	0.022956499
802.319987	1079.095764	2.43537E-05	0.019539451
802.359987	804.64347	1.81597E-05	0.014570602
802.399987	578.421657	1.30542E-05	0.010474667
802.439987	437.274844	9.86868E-06	0.007919027
802.479987	370.300765	8.35717E-06	0.006706464
802.519987	318.876795	7.19661E-06	0.005775419
802.559987	329.682909	7.44048E-06	0.005971435
802.599987	382.035268	8.622E-06	0.00692002
802.639987	409.188761	9.23482E-06	0.007412236
802.679987	414.017837	9.34381E-06	0.007500086
802.719987	425.964856	9.61343E-06	0.007716895
802.759987	436.638532	9.85432E-06	0.007910657
802.799987	418.590126	9.447E-06	0.007584048
802.839987	394.320752	8.89927E-06	0.00714469
802.879987	380.78038	8.59368E-06	0.006899696
802.919987	406.010953	9.1631E-06	0.007357237
802.959987	459.476415	1.03697E-05	0.008326488
802.999987	561.735624	1.26776E-05	0.010180105
803.039987	788.764835	1.78013E-05	0.014295175
803.079987	1097.444746	2.47678E-05	0.019890524
803.119987	1374.349031	3.10171E-05	0.024910488
803.159987	1574.941093	3.55442E-05	0.028547701
803.199987	1678.804404	3.78883E-05	0.030431864

803.239987	1619.990445	3.65609E-05	0.029367199
803.279987	1412.717845	3.18831E-05	0.025611035
803.319987	1169.287598	2.63892E-05	0.021198966
803.359987	921.28999	2.07922E-05	0.016703647
803.399987	692.863911	1.5637E-05	0.012562744
803.439987	527.167066	1.18974E-05	0.009558868
803.479987	422.726393	9.54035E-06	0.007665477
803.519987	363.93547	8.21352E-06	0.006599725
803.559987	338.894691	7.64838E-06	0.006145933
803.599987	326.884935	7.37734E-06	0.005928428
803.639987	335.425117	7.57008E-06	0.006083617
803.679987	342.804048	7.73661E-06	0.006217759
803.719987	368.151499	8.30867E-06	0.006677841
803.759987	395.681767	8.92999E-06	0.007177566
803.799987	344.883074	7.78353E-06	0.006256402
803.839987	321.890944	7.26463E-06	0.0058396
803.879987	325.712759	7.35088E-06	0.005909228
803.919987	350.585014	7.91222E-06	0.006360788
803.959987	392.313857	8.85398E-06	0.007118243
803.999986	443.004591	9.998E-06	0.008038389
804.039986	539.795483	1.21824E-05	0.009795163
804.079986	694.373812	1.5671E-05	0.012600777
804.119986	881.790649	1.99008E-05	0.01600262
804.159986	1071.73459	2.41876E-05	0.019450665
804.199986	1169.3305	2.63902E-05	0.021222967
804.239986	1098.527338	2.47922E-05	0.019938904
804.279986	955.360738	2.15612E-05	0.01734121
804.319986	831.925882	1.87754E-05	0.015101436
804.359986	712.57049	1.60817E-05	0.012935495
804.399986	568.100968	1.28212E-05	0.010313411
804.439986	450.719849	1.01721E-05	0.008182859
804.479986	361.777589	8.16482E-06	0.006568431
804.519986	304.26846	6.86692E-06	0.005524571
804.559986	307.687435	6.94408E-06	0.005586926
804.599986	356.303254	8.04127E-06	0.006470004
804.639986	398.061682	8.9837E-06	0.007228642
804.679986	435.921089	9.83813E-06	0.007916548
804.719986	419.453103	9.46647E-06	0.007617859
804.759986	382.545295	8.63351E-06	0.006947907
804.799986	363.464188	8.20288E-06	0.006601678
804.839986	349.642759	7.89095E-06	0.006350952
804.879986	358.989349	8.10189E-06	0.006521049
804.919986	410.951347	9.2746E-06	0.00746531
804.959986	507.553864	1.14548E-05	0.009220643
804.999986	650.833294	1.46884E-05	0.011824163
805.039986	873.885742	1.97224E-05	0.015877308

805.079986	1199.845755	2.70788E-05	0.021800638
805.119986	1521.554572	3.43394E-05	0.027647311
805.159986	1768.33408	3.99088E-05	0.032132999
805.199986	1862.203962	4.20273E-05	0.033840421
805.239986	1772.485783	4.00025E-05	0.032211641
805.279986	1586.360677	3.5802E-05	0.028830594
805.319986	1288.03883	2.90692E-05	0.023410042
805.359986	980.207422	2.21219E-05	0.017816106
805.399986	754.957332	1.70383E-05	0.013722675
805.439986	609.148526	1.37476E-05	0.011072894
805.479986	515.725009	1.16392E-05	0.009375139
805.519986	434.67135	9.80993E-06	0.007902092
805.559986	385.50449	8.7003E-06	0.007008613
805.599986	385.257421	8.69472E-06	0.007004469
805.639986	380.417666	8.5855E-06	0.00691682
805.679986	379.232954	8.55876E-06	0.006895621
805.719986	377.449706	8.51851E-06	0.006863537
805.759986	336.890182	7.60314E-06	0.006126308
805.799986	304.182347	6.86497E-06	0.005531794
805.839986	315.949798	7.13055E-06	0.00574608
805.879986	352.970377	7.96605E-06	0.00641968
805.919986	410.452031	9.26333E-06	0.007465503
805.959986	431.136412	9.73015E-06	0.00784211
805.999986	482.289353	1.08846E-05	0.008772987
806.039986	620.325393	1.39999E-05	0.011284463
806.079986	784.65552	1.77086E-05	0.014274534
806.119986	1007.765364	2.27439E-05	0.018334281
806.159986	1256.371826	2.83546E-05	0.022858313
806.199986	1348.721944	3.04388E-05	0.024539741
806.239986	1298.613606	2.93079E-05	0.023629201
806.279986	1200.541648	2.70946E-05	0.021845796
806.319986	1034.973361	2.33579E-05	0.018833947
806.359986	840.6268	1.89718E-05	0.015298081
806.399986	683.103684	1.54167E-05	0.012432026
806.439986	536.00087	1.20968E-05	0.009755338
806.479986	405.327412	9.14768E-06	0.007377417
806.519986	336.307875	7.59E-06	0.006121487
806.559986	315.597433	7.12259E-06	0.0057448
806.599986	353.364495	7.97494E-06	0.00643259
806.639986	398.134445	8.98534E-06	0.007247934
806.679986	410.135532	9.25619E-06	0.007466781
806.719986	414.947305	9.36478E-06	0.007554757
806.759986	407.541153	9.19764E-06	0.007420285
806.799986	393.676476	8.88473E-06	0.0071682
806.839986	363.915344	8.21306E-06	0.006626627
806.879986	353.698679	7.98249E-06	0.006440909

806.919986	402.416118	9.08197E-06	0.007328424
806.959986	492.560093	1.11164E-05	0.008970486
806.999986	595.064429	1.34298E-05	0.010837828
807.039986	780.078619	1.76053E-05	0.014208171
807.079986	1046.550346	2.36192E-05	0.01906257
807.119986	1308.687826	2.95353E-05	0.0238385
807.159986	1509.954448	3.40776E-05	0.02750605
807.199986	1615.077669	3.64501E-05	0.029422483
807.239986	1591.665451	3.59217E-05	0.02899741
807.279986	1402.31911	3.16484E-05	0.025549111
807.319986	1128.444607	2.54674E-05	0.02056036
807.359986	864.462359	1.95097E-05	0.01575136
807.399986	651.623278	1.47062E-05	0.01187381
807.439986	505.014158	1.13975E-05	0.00920277
807.479986	416.091284	9.3906E-06	0.007582722
807.519986	350.223966	7.90407E-06	0.006382692
807.559986	321.377527	7.25304E-06	0.005857267
807.599986	323.84375	7.3087E-06	0.005902508
807.639986	366.383747	8.26877E-06	0.00667819
807.679986	391.83436	8.84316E-06	0.00714244
807.719986	399.999659	9.02744E-06	0.00729164
807.759986	391.374644	8.83278E-06	0.007134767
807.799986	373.780729	8.43571E-06	0.006814367
807.839986	335.818684	7.57896E-06	0.006122587
807.879986	292.275667	6.59625E-06	0.005328982
807.919986	288.268071	6.50581E-06	0.005256173
807.959986	347.099175	7.83354E-06	0.006329191
807.999986	437.272337	9.86863E-06	0.007973851
808.039986	550.463828	1.24232E-05	0.010038444
808.079986	751.490854	1.69601E-05	0.013705119
808.119986	965.445349	2.17888E-05	0.01760793
808.159986	1170.303369	2.64121E-05	0.021345216
808.199986	1219.117919	2.75138E-05	0.022236647
808.239986	1102.379524	2.48792E-05	0.02010834
808.279986	964.385929	2.17648E-05	0.01759209
808.319986	867.431707	1.95767E-05	0.015824258
808.359986	772.45694	1.74333E-05	0.014092364
808.399986	620.547876	1.40049E-05	0.011321562
808.439986	460.393107	1.03904E-05	0.00840004
808.479986	356.75854	8.05154E-06	0.006509512
808.519986	309.465954	6.98422E-06	0.005646878
808.559986	304.081376	6.86269E-06	0.005548899
808.599986	301.833128	6.81195E-06	0.005508145
808.639986	332.414308	7.50213E-06	0.006066521
808.679986	386.887856	8.73152E-06	0.007061005
808.719986	407.859481	9.20482E-06	0.007444122

808.759986	394.194342	8.89642E-06	0.007195066
808.799986	384.580272	8.67944E-06	0.007019932
808.839986	362.037877	8.17069E-06	0.006608781
808.879986	340.385389	7.68202E-06	0.006213836
808.919986	377.276998	8.51462E-06	0.006887643
808.959986	451.833361	1.01972E-05	0.008249167
808.999986	573.717058	1.2948E-05	0.010474928
809.039986	761.858284	1.71941E-05	0.013910698
809.079986	1038.023316	2.34267E-05	0.018954107
809.119986	1327.14423	2.99518E-05	0.024234597
809.159986	1537.344741	3.46957E-05	0.028074396
809.199986	1625.953444	3.66955E-05	0.029694002
809.239986	1537.193975	3.46923E-05	0.028074418
809.279986	1377.802492	3.10951E-05	0.025164628
809.319986	1154.710787	2.60602E-05	0.021091052
809.359986	901.449265	2.03445E-05	0.016465987
809.399986	690.84419	1.55914E-05	0.012619672
809.439986	530.323616	1.19687E-05	0.009687916
809.479986	422.83801	9.54286E-06	0.007724758
809.519986	368.191064	8.30956E-06	0.006726754
809.559986	336.67057	7.59819E-06	0.006151187
809.599986	339.083294	7.65264E-06	0.006195575
809.639986	343.577355	7.75406E-06	0.006277999
809.679986	350.878601	7.91884E-06	0.006411727
809.719986	355.546932	8.0242E-06	0.006497354
809.759986	360.973422	8.14667E-06	0.006596845
809.799986	345.570073	7.79904E-06	0.006315659
809.839986	308.945692	6.97247E-06	0.005646588
809.879986	281.886498	6.36179E-06	0.005152283
809.919986	292.895859	6.61025E-06	0.005353775
809.959986	338.185039	7.63237E-06	0.00618191
809.999986	419.501176	9.46756E-06	0.007668721
810.039986	573.997514	1.29543E-05	0.010493521
810.079986	735.186754	1.65921E-05	0.013440961
810.119986	920.177393	2.07671E-05	0.016823861
810.159986	1085.852472	2.45062E-05	0.019853925
810.199986	1176.450599	2.65509E-05	0.021511499
810.239986	1161.80143	2.62202E-05	0.021244687
810.279986	1081.754134	2.44137E-05	0.01978192
810.319986	935.046482	2.11027E-05	0.017099937
810.359986	757.361545	1.70926E-05	0.013851155
810.399986	587.515821	1.32594E-05	0.010745429
810.439986	485.155158	1.09493E-05	0.008873731
810.479986	433.761484	9.78939E-06	0.007934107
810.519986	388.994398	8.77906E-06	0.007115605
810.559986	368.707388	8.32121E-06	0.006744842

810.599986	392.258887	8.85274E-06	0.007176028
810.639986	410.064387	9.25458E-06	0.007502134
810.679986	451.460558	1.01888E-05	0.008259885
810.719986	476.661217	1.07576E-05	0.008721385
810.759986	489.067716	1.10376E-05	0.008948826
810.799986	482.72862	1.08945E-05	0.008833271
810.839986	447.748769	1.01051E-05	0.008193592
810.879986	442.351975	9.98327E-06	0.008095232
810.919986	489.190781	1.10404E-05	0.008952844
810.959986	589.988274	1.33152E-05	0.010798105
810.999986	715.662785	1.61515E-05	0.013098876
811.039986	913.602984	2.06187E-05	0.016722628
811.079986	1168.341405	2.63678E-05	0.021386426
811.119986	1433.682052	3.23562E-05	0.026244766
811.159986	1677.658336	3.78624E-05	0.030712473
811.199986	1768.445376	3.99113E-05	0.032376086
811.239986	1685.343684	3.80359E-05	0.03085621
811.279986	1477.721923	3.33501E-05	0.027056289
811.319986	1222.664094	2.75938E-05	0.022387422
811.359986	946.15525	2.13534E-05	0.017325299
811.399986	700.149435	1.58014E-05	0.012821254
811.439986	509.840627	1.15064E-05	0.009336747
811.479986	361.008516	8.14746E-06	0.0066115
811.519986	282.181829	6.36845E-06	0.005168125
811.559986	282.482936	6.37525E-06	0.005173895
811.599986	320.001892	7.222E-06	0.005861373
811.639986	388.902158	8.77698E-06	0.007123748
811.679986	418.386212	9.44239E-06	0.007664202
811.719986	434.113287	9.79733E-06	0.00795269
811.759986	438.359686	9.89317E-06	0.008030878
811.799986	417.297009	9.41781E-06	0.00764538
811.839986	391.095116	8.82647E-06	0.007165683
811.879986	383.307165	8.65071E-06	0.007023337
811.919986	374.85913	8.46005E-06	0.006868882
811.959986	374.736479	8.45728E-06	0.006866973
811.999986	424.858246	9.58846E-06	0.007785828
812.039986	531.991576	1.20063E-05	0.009749603
812.079986	688.78953	1.5545E-05	0.0126238
812.119986	886.169797	1.99996E-05	0.016242089
812.159986	1021.954036	2.30641E-05	0.018731722
812.199986	1114.729577	2.51579E-05	0.020433241
812.239986	1163.063134	2.62487E-05	0.021320256
812.279986	1102.462235	2.4881E-05	0.020210368
812.319986	909.42931	2.05246E-05	0.016672504
812.359986	699.242748	1.57809E-05	0.0128198
812.399986	554.837219	1.25219E-05	0.010172794

812.439986	431.586107	9.7403E-06	0.007913407
812.479986	353.376965	7.97523E-06	0.006479711
812.519986	332.534045	7.50483E-06	0.006097825
812.559986	334.785151	7.55563E-06	0.006139406
812.599986	339.540972	7.66297E-06	0.006226927
812.639986	384.292198	8.67294E-06	0.007047978
812.679986	435.510574	9.82887E-06	0.007987724
812.719986	442.613919	9.98918E-06	0.008118406
812.759986	422.11154	9.52647E-06	0.007742733
812.799986	393.147203	8.87278E-06	0.007211799
812.839986	362.258289	8.17566E-06	0.006645507
812.879986	360.641712	8.13918E-06	0.006616177
812.919986	369.880615	8.34769E-06	0.006786004
812.959986	421.592814	9.51476E-06	0.007735121
812.999986	531.95176	1.20054E-05	0.009760398
813.039986	690.605985	1.5586E-05	0.012672054
813.079986	894.570395	2.01892E-05	0.016415441
813.119986	1188.497064	2.68227E-05	0.021810092
813.159986	1435.30951	3.23929E-05	0.02634064
813.199986	1523.847109	3.43911E-05	0.027966847
813.239986	1492.231818	3.36776E-05	0.027387966
813.279986	1307.723167	2.95135E-05	0.024002731
813.319986	1065.499252	2.40468E-05	0.019557771
813.359986	816.858695	1.84354E-05	0.014994587
813.399986	618.981211	1.39695E-05	0.011362827
813.439986	466.200032	1.05215E-05	0.008558597
813.479986	385.89341	8.70908E-06	0.00708466
813.519986	346.604354	7.82238E-06	0.00636366
813.559986	331.655581	7.485E-06	0.0060895
813.599986	348.824847	7.87249E-06	0.006405059
813.639986	359.381556	8.11074E-06	0.006599223
813.679986	371.365441	8.3812E-06	0.006819615
813.719986	372.505515	8.40693E-06	0.006840887
813.759986	351.990556	7.94394E-06	0.006464458
813.799986	327.38363	7.38859E-06	0.006012836
813.839986	297.624514	6.71697E-06	0.005466539
813.879986	299.527408	6.75992E-06	0.005501761
813.919986	302.438455	6.82561E-06	0.005555504
813.959986	320.869003	7.24157E-06	0.005894345
813.999986	379.820197	8.57201E-06	0.006977618
814.039986	486.046435	1.09694E-05	0.008929523
814.079986	635.155958	1.43346E-05	0.011669499
814.119986	821.839265	1.85478E-05	0.015100108
814.159986	1021.186228	2.30468E-05	0.018763742
814.199986	1118.981598	2.52539E-05	0.020561689
814.239986	1104.508597	2.49272E-05	0.02029674

814.279986	990.52306	2.23547E-05	0.018203006
814.319986	833.592845	1.8813E-05	0.015319826
814.359986	687.391905	1.55135E-05	0.012633556
814.399986	573.061478	1.29332E-05	0.010532797
814.439986	485.561639	1.09584E-05	0.008925
814.479986	438.572948	9.89798E-06	0.008061707
814.519986	364.909273	8.23549E-06	0.006707974
814.559986	329.24492	7.4306E-06	0.006052669
814.599986	374.017867	8.44106E-06	0.006876089
814.639986	419.804052	9.47439E-06	0.007718219
814.679986	407.010227	9.18565E-06	0.007483368
814.719986	362.323325	8.17713E-06	0.006662073
814.759986	360.357434	8.13277E-06	0.006626252
814.799986	351.466086	7.9321E-06	0.006463075
814.839986	360.315261	8.13181E-06	0.006626127
814.879986	419.418214	9.46568E-06	0.007713397
814.919986	445.094687	1.00452E-05	0.008186007
814.959986	457.819048	1.03323E-05	0.008420442
814.999986	527.379508	1.19022E-05	0.00970031
815.039986	713.9688	1.61133E-05	0.013132969
815.079986	950.840364	2.14591E-05	0.017490918
815.119986	1245.489281	2.8109E-05	0.022912174
815.159986	1554.204198	3.50762E-05	0.028592734
815.199986	1716.88628	3.87477E-05	0.031587151
815.239986	1665.999168	3.75993E-05	0.030652437
815.279986	1448.914526	3.27E-05	0.026659641
815.319986	1176.841075	2.65597E-05	0.021654625
815.359986	948.881895	2.14149E-05	0.017460887
815.399986	767.686543	1.73256E-05	0.014127307
815.439986	580.80402	1.31079E-05	0.010688737
815.479986	445.819381	1.00615E-05	0.00820497
815.519986	369.864004	8.34732E-06	0.006807403
815.559986	337.301847	7.61243E-06	0.006208396
815.599986	347.495138	7.84248E-06	0.006396328
815.639986	372.123987	8.39832E-06	0.006850006
815.679986	366.074041	8.26178E-06	0.00673897
815.719986	352.077714	7.9459E-06	0.006481632
815.759986	338.10904	7.63065E-06	0.006224779
815.799986	321.074118	7.2462E-06	0.005911446
815.839986	308.630909	6.96537E-06	0.005682627
815.879986	312.399182	7.05041E-06	0.005752292
815.919986	352.652738	7.95888E-06	0.00649381
815.959986	394.831503	8.9108E-06	0.007270854
815.999986	427.631875	9.65106E-06	0.007875261
816.039986	494.121951	1.11516E-05	0.009100188
816.079986	623.570099	1.40731E-05	0.011484782

816.119986	808.368185	1.82437E-05	0.014889084
816.159986	1021.868385	2.30621E-05	0.018822401
816.199986	1174.117811	2.64982E-05	0.021627833
816.239986	1163.706491	2.62632E-05	0.021437102
816.279986	1060.303467	2.39296E-05	0.019533231
816.319986	941.174198	2.1241E-05	0.017339445
816.359986	794.136809	1.79226E-05	0.014631262
816.399986	619.502155	1.39813E-05	0.011414334
816.439986	518.041426	1.16915E-05	0.009545386
816.479986	437.827926	9.88117E-06	0.008067775
816.519986	377.333603	8.51589E-06	0.006953398
816.559986	378.71883	8.54716E-06	0.006979266
816.599986	400.120456	9.03016E-06	0.00737403
816.639986	398.452125	8.99251E-06	0.007343643
816.679986	416.982228	9.41071E-06	0.007685537
816.719986	441.880323	9.97262E-06	0.008144841
816.759986	415.9074	9.38645E-06	0.007666477
816.799986	379.29432	8.56014E-06	0.006991926
816.839986	383.659789	8.65867E-06	0.007072745
816.879986	401.494157	9.06116E-06	0.007401883
816.919986	414.194629	9.3478E-06	0.007636401
816.959986	458.997156	1.03589E-05	0.008462828
816.999986	540.99702	1.22095E-05	0.009975202
817.039986	705.445082	1.59209E-05	0.013008022
817.079986	954.282214	2.15368E-05	0.017597305
817.119986	1183.219333	2.67036E-05	0.021820055
817.159986	1337.149393	3.01776E-05	0.024659927
817.199986	1412.355985	3.18749E-05	0.026048174
817.239986	1370.110093	3.09215E-05	0.025270267
817.279986	1260.882649	2.84564E-05	0.023256818
817.319986	1092.885096	2.46649E-05	0.020159111
817.359986	884.073315	1.99523E-05	0.016308214
817.399986	696.87779	1.57276E-05	0.012855708
817.439986	558.925909	1.26142E-05	0.010311335
817.479986	451.060326	1.01798E-05	0.008321785
817.519986	374.402884	8.44975E-06	0.006907841
817.559986	327.343028	7.38768E-06	0.006039868
817.599986	346.961293	7.83043E-06	0.006402162
817.639986	402.574857	9.08555E-06	0.007428712
817.679986	410.677756	9.26842E-06	0.007578606
817.719986	383.678378	8.65909E-06	0.007080708
817.759986	365.323679	8.24485E-06	0.006742306
817.799986	334.96625	7.55972E-06	0.00618234
817.839986	307.005029	6.92868E-06	0.005666548
817.879986	283.463929	6.39739E-06	0.005232294
817.919986	309.260273	6.97957E-06	0.005708733

817.959986	366.414605	8.26947E-06	0.006764093
817.999986	429.718089	9.69814E-06	0.007933077
818.039986	541.107943	1.22121E-05	0.009989947
818.079986	689.595799	1.55632E-05	0.012731956
818.119986	825.666584	1.86341E-05	0.015244966
818.159986	966.419346	2.18107E-05	0.017844674
818.199986	1046.611262	2.36206E-05	0.019326341
818.239986	1030.646682	2.32603E-05	0.019032475
818.279986	956.523026	2.15874E-05	0.017664531
818.319986	809.278862	1.82643E-05	0.014946039
818.359986	640.634022	1.44582E-05	0.011832027
818.399986	498.459384	1.12495E-05	0.009206618
818.439986	418.973831	9.45566E-06	0.007738887
818.479986	359.014528	8.10246E-06	0.0066317
818.519986	336.568998	7.59589E-06	0.006217391
818.559986	337.58003	7.61871E-06	0.006236372
818.599986	329.057577	7.42637E-06	0.006079227
818.639986	324.088332	7.31422E-06	0.005987715
818.679986	333.926146	7.53625E-06	0.006169775
818.719986	352.13237	7.94714E-06	0.00650648
818.759986	356.417596	8.04385E-06	0.006585981
818.799986	353.385328	7.97541E-06	0.006530269
818.839986	365.985629	8.25979E-06	0.006763443
818.879986	380.019159	8.5765E-06	0.007023127
818.919986	415.882902	9.3859E-06	0.007686299
818.959986	470.973452	1.06292E-05	0.008704902
818.999986	558.248688	1.25989E-05	0.010318495
819.039986	708.825422	1.59972E-05	0.013102348
819.079986	953.874965	2.15276E-05	0.01763285
819.119986	1227.494614	2.77028E-05	0.022691953
819.159986	1393.835419	3.14569E-05	0.025768253
819.199986	1383.598217	3.12259E-05	0.025580244
819.239986	1292.814067	2.9177E-05	0.023902976
819.279986	1206.233634	2.7223E-05	0.02230327
819.319986	1071.355189	2.4179E-05	0.019810333
819.359986	845.119914	1.90732E-05	0.0156278
819.399986	646.875755	1.45991E-05	0.01196249
819.439986	516.581172	1.16585E-05	0.009553455
819.479986	438.509621	9.89655E-06	0.008110026
819.519986	396.266966	8.94319E-06	0.007329126
819.559986	337.047682	7.6067E-06	0.006234144
819.599986	340.773646	7.69079E-06	0.006303369
819.639986	375.487993	8.47424E-06	0.006945827
819.679986	394.830701	8.91078E-06	0.007303987
819.719986	393.940564	8.89069E-06	0.007287876
819.759986	362.415699	8.17922E-06	0.006704995

819.799986	342.582796	7.73162E-06	0.006338379
819.839986	316.004511	7.13178E-06	0.00584692
819.879986	306.790402	6.92383E-06	0.005676711
819.919986	323.032547	7.29039E-06	0.00597754
819.959986	370.127594	8.35326E-06	0.006849342
819.999986	426.33244	9.62173E-06	0.007889818
820.039986	508.441305	1.14748E-05	0.009409805
820.079986	650.985464	1.46918E-05	0.01204848
820.119986	791.779834	1.78694E-05	0.014655026
820.159986	942.456009	2.12699E-05	0.017444737
820.199986	1064.93661	2.40341E-05	0.019712798
820.239986	1099.678294	2.48182E-05	0.020356886
820.279986	1011.732603	2.28334E-05	0.018729777
820.319986	840.959429	1.89793E-05	0.015569085
820.359986	703.881429	1.58856E-05	0.01303193
820.399986	600.492667	1.35523E-05	0.011118293
820.439986	502.134745	1.13325E-05	0.009297621
820.479986	412.890697	9.31837E-06	0.007645534
820.519986	340.635163	7.68766E-06	0.00630788
820.559986	336.598816	7.59657E-06	0.006233438
820.599986	398.077788	8.98406E-06	0.00737232
820.639986	436.613826	9.85377E-06	0.008086394
820.679986	433.855903	9.79152E-06	0.008035707
820.719986	438.111361	9.88756E-06	0.008114921
820.759986	435.628292	9.83152E-06	0.008069321
820.799986	417.530738	9.42309E-06	0.00773447
820.839986	364.483413	8.22588E-06	0.006752134
820.879986	334.300395	7.54469E-06	0.006193289
820.919986	353.799698	7.98477E-06	0.006554854
820.959986	414.50995	9.35491E-06	0.007680009
820.999986	577.447602	1.30322E-05	0.010699426
821.039986	820.693665	1.85219E-05	0.015207231
821.079986	1078.878617	2.43488E-05	0.019992302
821.119986	1265.797284	2.85673E-05	0.023457166
821.159986	1393.749014	3.1455E-05	0.025829566
821.199986	1438.92264	3.24745E-05	0.026668042
821.239986	1366.216599	3.08336E-05	0.02532179
821.279986	1210.349727	2.73159E-05	0.022434008
821.319986	1011.81618	2.28353E-05	0.018755073
821.359986	791.899538	1.78721E-05	0.014679403
821.399986	611.818532	1.38079E-05	0.011341802
821.439986	490.750822	1.10756E-05	0.00909791
821.479986	385.27862	8.6952E-06	0.007142934
821.519986	313.095603	7.06613E-06	0.005804968
821.559986	283.085842	6.38885E-06	0.005248826
821.599986	297.788278	6.72067E-06	0.0055217

821.639986	325.788791	7.3526E-06	0.00604119
821.679986	358.727246	8.09597E-06	0.0066523
821.719986	398.801322	9.00039E-06	0.007395801
821.759986	341.406015	7.70506E-06	0.006331709
821.799986	286.009737	6.45484E-06	0.005304589
821.839986	273.163587	6.16492E-06	0.005066579
821.879986	279.513259	6.30822E-06	0.005184604
821.919986	283.534362	6.39898E-06	0.005259446
821.959986	320.651232	7.23665E-06	0.005948238
821.999986	400.785204	9.04516E-06	0.007435125
822.039986	511.551275	1.1545E-05	0.009490451
822.079986	673.311975	1.51957E-05	0.012492092
822.119986	875.839502	1.97665E-05	0.016250416
822.159986	1077.407097	2.43156E-05	0.019991295
822.199986	1190.351853	2.68646E-05	0.02208806
822.239986	1167.735706	2.63542E-05	0.021669451
822.279986	1046.699808	2.36226E-05	0.019424356
822.319986	855.201556	1.93007E-05	0.015871358
822.359986	660.545178	1.49076E-05	0.012259401
822.399986	514.372293	1.16087E-05	0.009546967
822.439986	406.614277	9.17672E-06	0.0075473
822.479986	335.985343	7.58272E-06	0.006236636
822.519986	322.06496	7.26856E-06	0.005978534
822.559986	342.495913	7.72966E-06	0.006358105
822.599986	372.893052	8.41568E-06	0.006922736
822.639986	397.975128	8.98174E-06	0.007388742
822.679986	410.954249	9.27466E-06	0.007630081
822.719986	421.712981	9.51747E-06	0.007830217
822.759986	406.550417	9.17528E-06	0.00754905
822.799986	375.779884	8.48083E-06	0.006978026
822.839986	353.683224	7.98214E-06	0.006568022
822.879986	367.482474	8.29357E-06	0.006824611
822.919986	420.40886	9.48804E-06	0.0078079
822.959986	504.015016	1.13749E-05	0.009361101
822.999986	632.289544	1.42699E-05	0.011744123
823.039986	810.890865	1.83007E-05	0.015062189
823.079986	1004.755218	2.26759E-05	0.018664101
823.119986	1195.796068	2.69875E-05	0.022213911
823.159986	1410.940107	3.1843E-05	0.026211845
823.199986	1565.418561	3.53293E-05	0.029083093
823.239986	1580.268593	3.56645E-05	0.02936041
823.279986	1482.082523	3.34485E-05	0.027537512
823.319986	1253.370182	2.82868E-05	0.023289104
823.359986	1013.28634	2.28685E-05	0.018828976
823.399986	825.226138	1.86242E-05	0.01533517
823.439986	615.489625	1.38907E-05	0.011438194

823.479986	448.569544	1.01236E-05	0.008336574
823.519986	358.323033	8.08685E-06	0.006659684
823.559986	335.101316	7.56277E-06	0.006228395
823.599986	334.835283	7.55677E-06	0.006223752
823.639986	338.443814	7.63821E-06	0.006291131
823.679986	361.889871	8.16735E-06	0.006727283
823.719986	355.462494	8.02229E-06	0.006608123
823.759986	334.679792	7.55326E-06	0.006222071
823.799986	324.051195	7.31338E-06	0.006024766
823.839986	316.152794	7.13513E-06	0.005878204
823.879986	299.681268	6.76339E-06	0.005572221
823.919986	316.384463	7.14036E-06	0.005883083
823.959986	354.784575	8.00699E-06	0.006597442
823.999986	419.860646	9.47567E-06	0.007807952
824.039986	547.647151	1.23596E-05	0.010184832
824.079986	702.682765	1.58586E-05	0.013068731
824.119986	858.12092	1.93666E-05	0.015960397
824.159986	1064.45065	2.40232E-05	0.019798934
824.199986	1172.212169	2.64552E-05	0.021804372
824.239986	1162.430378	2.62344E-05	0.02162347
824.279986	1126.816807	2.54307E-05	0.020962005
824.319986	996.873182	2.2498E-05	0.018545583
824.359986	836.836314	1.88862E-05	0.015569052
824.399986	725.175467	1.63662E-05	0.013492295
824.439986	599.873341	1.35383E-05	0.011161521
824.479986	443.157817	1.00015E-05	0.008245999
824.519986	348.117699	7.85653E-06	0.006477867
824.559986	312.844326	7.06046E-06	0.005821773
824.599986	342.921428	7.73926E-06	0.006381793
824.639986	411.045102	9.27672E-06	0.00764995
824.679986	417.037815	9.41196E-06	0.007761857
824.719986	385.219439	8.69387E-06	0.007170005
824.759986	364.796241	8.23294E-06	0.006790202
824.799986	340.87151	7.693E-06	0.006345182
824.839986	329.395503	7.434E-06	0.006131859
824.879986	310.884566	7.01623E-06	0.005787549
824.919986	325.852692	7.35404E-06	0.006066496
824.959986	420.215486	9.48368E-06	0.007823655
824.999986	558.884071	1.26132E-05	0.010405919
825.039986	736.383491	1.66191E-05	0.013711463
825.079986	931.908951	2.10319E-05	0.017352989
825.119986	1123.514815	2.53562E-05	0.020921878
825.159986	1344.480768	3.03431E-05	0.025037878
825.199986	1388.348573	3.13331E-05	0.025856068
825.239986	1287.55863	2.90584E-05	0.023980158
825.279986	1180.798634	2.6649E-05	0.02199287

825.319986	1030.09996	2.32479E-05	0.019186974
825.359986	854.69904	1.92894E-05	0.015920672
825.399986	678.937158	1.53227E-05	0.012647328
825.439986	552.752482	1.24749E-05	0.010297243
825.479986	439.81616	9.92604E-06	0.008193746
825.519986	351.283068	7.92797E-06	0.006544697
825.559986	330.427065	7.45728E-06	0.006156431
825.599986	355.266223	8.01786E-06	0.006619548
825.639986	366.014025	8.26043E-06	0.006820139
825.679986	383.893265	8.66394E-06	0.007153639
825.719986	366.738972	8.27679E-06	0.006834309
825.759986	334.715576	7.55406E-06	0.006237844
825.799986	303.98556	6.86053E-06	0.005665426
825.839986	269.135453	6.07401E-06	0.005016162
825.879986	253.315339	5.71697E-06	0.004721535
825.919986	272.046489	6.13971E-06	0.005070909
825.959986	307.251738	6.93424E-06	0.005727408
825.999986	382.993459	8.64363E-06	0.007139637
826.039986	529.663822	1.19538E-05	0.009874296
826.079986	705.814756	1.59293E-05	0.01315884
826.119986	836.123746	1.88701E-05	0.015589007
826.159986	928.08685	2.09456E-05	0.017304439
826.199986	1003.788338	2.26541E-05	0.018716821
826.239986	1035.612871	2.33723E-05	0.019311162
826.279986	962.306347	2.17179E-05	0.017945078
826.319986	817.790854	1.84564E-05	0.015250893
826.359986	665.895643	1.50283E-05	0.012418816
826.399986	543.040332	1.22557E-05	0.01012808
826.439986	438.037223	9.88589E-06	0.008170095
826.479986	348.53369	7.86592E-06	0.006501025
826.519986	291.391039	6.57629E-06	0.005435435
826.559986	254.7321	5.74895E-06	0.004751851
826.599986	257.856461	5.81946E-06	0.004810367
826.639986	303.929618	6.85927E-06	0.005670145
826.679986	379.689878	8.56907E-06	0.00708388
826.719986	411.919109	9.29644E-06	0.007685553
826.759986	441.240326	9.95818E-06	0.008233024
826.799986	435.462441	9.82778E-06	0.008125609
826.839986	362.265362	8.17582E-06	0.006760099
826.879986	344.041415	7.76454E-06	0.006420339
826.919986	361.765001	8.16453E-06	0.006751415
826.959986	425.284251	9.59807E-06	0.007937222
826.999986	508.84806	1.1484E-05	0.009497261
827.039986	610.202192	1.37714E-05	0.01138951
827.079986	786.952484	1.77604E-05	0.014689289
827.119986	986.190741	2.2257E-05	0.018409169

827.159986	1191.660604	2.68941E-05	0.02224574
827.199986	1301.808649	2.938E-05	0.024303142
827.239986	1314.737324	2.96718E-05	0.024545691
827.279986	1253.772167	2.82959E-05	0.023408625
827.319986	1060.343479	2.39305E-05	0.019798161
827.359986	829.221733	1.87144E-05	0.015483529
827.399986	621.952256	1.40366E-05	0.011613879
827.439986	477.729721	1.07817E-05	0.008921205
827.479986	420.801907	9.49691E-06	0.007858505
827.519986	381.844611	8.6177E-06	0.00713132
827.559986	341.146604	7.6992E-06	0.006371553
827.599986	330.428675	7.45731E-06	0.006171674
827.639986	325.811184	7.3531E-06	0.006085723
827.679986	321.43228	7.25428E-06	0.006004221
827.719986	302.883562	6.83566E-06	0.005658012
827.759986	296.343174	6.68805E-06	0.005536102
827.799986	305.405292	6.89257E-06	0.005705671
827.839986	326.399383	7.36638E-06	0.006098183
827.879986	340.273914	7.67951E-06	0.006357711
827.919986	340.235589	7.67864E-06	0.006357302
827.959986	341.863326	7.71538E-06	0.006388025
827.999986	374.000387	8.44067E-06	0.006988873
828.039986	482.087867	1.08801E-05	0.009009118
828.079986	692.538514	1.56296E-05	0.012942583
828.119986	878.25029	1.98209E-05	0.016414072
828.159986	1018.333848	2.29824E-05	0.019033085
828.199986	1107.199973	2.4988E-05	0.020695029
828.239986	1104.445166	2.49258E-05	0.020644535
828.279986	1030.642962	2.32602E-05	0.019265938
828.319986	907.904621	2.04901E-05	0.016972395
828.359986	766.404345	1.72967E-05	0.014327876
828.399986	624.688791	1.40984E-05	0.011679078
828.439986	524.783965	1.18436E-05	0.009811748
828.479986	440.830889	9.94894E-06	0.008242497
828.519986	392.516381	8.85855E-06	0.007339484
828.559986	390.197722	8.80622E-06	0.007296481
828.599986	442.666574	9.99037E-06	0.008278019
828.639986	512.502161	1.15665E-05	0.009584431
828.679986	548.973708	1.23896E-05	0.01026699
828.719986	575.540713	1.29892E-05	0.01076437
828.759986	596.579512	1.3464E-05	0.011158398
828.799986	612.448484	1.38221E-05	0.011455764
828.839986	649.151777	1.46505E-05	0.01214288
828.879986	735.424519	1.65975E-05	0.013757341
828.919986	832.816419	1.87955E-05	0.015579971
828.959986	936.03446	2.1125E-05	0.017511775

828.999986	1204.713866	2.71887E-05	0.022539443
829.039986	1662.457681	3.75194E-05	0.031105045
829.079986	2216.594861	5.00255E-05	0.041475106
829.119986	2838.752867	6.40667E-05	0.05311898
829.159986	3425.681704	7.73129E-05	0.064104734
829.199986	3730.000106	8.41809E-05	0.069802807
829.239986	3658.719839	8.25722E-05	0.068472179
829.279986	3166.267316	7.14582E-05	0.059258891
829.319986	2448.284939	5.52544E-05	0.045823562
829.359986	1737.302749	3.92085E-05	0.032517964
829.399986	1131.091255	2.55272E-05	0.021172221
829.439986	726.81666	1.64032E-05	0.013605503
829.479986	533.940929	1.20503E-05	0.009995485
829.519986	447.830842	1.01069E-05	0.008383891
829.559986	418.878483	9.4535E-06	0.007842249
829.599986	383.63423	8.65809E-06	0.007182752
829.639986	362.675553	8.18508E-06	0.006790671
829.679986	398.634161	8.99662E-06	0.007464314
829.719986	408.136175	9.21106E-06	0.007642605
829.759986	368.900771	8.32558E-06	0.00690823
829.799986	348.670828	7.86901E-06	0.006529708
829.839986	367.187679	8.28691E-06	0.006876813
829.879986	406.82589	9.18149E-06	0.007619538
829.919986	438.139593	9.8882E-06	0.008206415
829.959986	502.099549	1.13317E-05	0.009404847
829.999986	634.570016	1.43214E-05	0.01188673
830.039986	839.874534	1.89548E-05	0.015733241
830.079986	1110.612851	2.5065E-05	0.020805942
830.119986	1412.09274	3.1869E-05	0.026455066
830.159986	1695.074713	3.82555E-05	0.031758165
830.199986	1871.747587	4.22427E-05	0.035069919
830.239986	1831.596367	4.13366E-05	0.034319281
830.279986	1636.130568	3.69252E-05	0.030658245
830.319986	1301.341348	2.93695E-05	0.024386051
830.359986	948.587012	2.14083E-05	0.017776585
830.399986	677.699159	1.52947E-05	0.01270074
830.439986	500.483578	1.12952E-05	0.00938
830.479986	369.760062	8.34497E-06	0.00693033
830.519986	306.472483	6.91666E-06	0.005744422
830.559986	315.654829	7.12389E-06	0.005916818
830.599986	357.187244	8.06122E-06	0.006695648
830.639986	408.668039	9.22307E-06	0.007661049
830.679986	444.355123	1.00285E-05	0.008330454
830.719986	405.916854	9.16098E-06	0.007610208
830.759986	407.847096	9.20454E-06	0.007646764
830.799986	412.015577	9.29862E-06	0.007725291

830.839986	397.973954	8.98172E-06	0.00746237
830.879986	394.733101	8.90858E-06	0.007401958
830.919986	417.962838	9.43284E-06	0.007837935
830.959986	483.709278	1.09166E-05	0.009071295
830.999986	660.831406	1.4914E-05	0.012393571
831.039986	903.782355	2.03971E-05	0.016950813
831.079986	1122.730492	2.53385E-05	0.02105829
831.119986	1412.459847	3.18773E-05	0.026493821
831.159986	1729.405204	3.90303E-05	0.032440396
831.199986	1883.990813	4.2519E-05	0.035341832
831.239986	1878.165104	4.23876E-05	0.035234243
831.279986	1649.445357	3.72257E-05	0.030944966
831.319986	1332.732044	3.00779E-05	0.025004364
831.359986	1021.142696	2.30458E-05	0.01915933
831.399986	794.819126	1.7938E-05	0.01491362
831.439986	621.491819	1.40262E-05	0.011661947
831.479986	490.108044	1.10611E-05	0.009197047
831.519986	407.107514	9.18785E-06	0.007639881
831.559986	368.449904	8.3154E-06	0.006914755
831.599986	347.8276	7.84998E-06	0.006528047
831.639986	356.698852	8.0502E-06	0.006694865
831.679986	433.690722	9.7878E-06	0.008140314
831.719986	480.702575	1.08488E-05	0.009023153
831.759986	471.983717	1.0652E-05	0.00885992
831.799986	428.496357	9.67057E-06	0.008043977
831.839986	367.634224	8.29699E-06	0.00690177
831.879986	325.511486	7.34634E-06	0.006111274
831.919986	323.783356	7.30734E-06	0.006079122
831.959986	360.041615	8.12564E-06	0.006760205
831.999986	435.296727	9.82404E-06	0.008173602
832.039986	558.870492	1.26129E-05	0.010494461
832.079986	728.743444	1.64467E-05	0.01368499
832.119986	889.264007	2.00694E-05	0.01670019
832.159986	1074.072731	2.42403E-05	0.020171829
832.199986	1252.885842	2.82759E-05	0.023531194
832.239986	1268.572094	2.86299E-05	0.023826952
832.279986	1136.246427	2.56435E-05	0.021342571
832.319986	953.745223	2.15247E-05	0.017915439
832.359986	736.682331	1.66259E-05	0.013838729
832.399986	526.048162	1.18722E-05	0.009882398
832.439986	399.99481	9.02733E-06	0.007514707
832.479986	352.033204	7.9449E-06	0.006613969
832.519986	326.366372	7.36563E-06	0.006132038
832.559986	298.515343	6.73708E-06	0.005609019
832.599986	306.075471	6.9077E-06	0.005751348
832.639986	363.285818	8.19885E-06	0.006826694

832.679986	428.663903	9.67435E-06	0.008055635
832.719986	428.387005	9.6681E-06	0.008050818
832.759986	402.343674	9.08034E-06	0.007561741
832.799986	375.656839	8.47805E-06	0.007060521
832.839986	356.866058	8.05397E-06	0.006707668
832.879986	367.278884	8.28897E-06	0.00690372
832.919986	389.492352	8.7903E-06	0.007321616
832.959986	435.415588	9.82672E-06	0.008185267
832.999986	565.254777	1.2757E-05	0.010626592
833.039986	746.348022	1.6844E-05	0.014031755
833.079986	933.769903	2.10739E-05	0.017556233
833.119986	1173.702099	2.64888E-05	0.022068367
833.159986	1358.274522	3.06544E-05	0.02553999
833.199986	1413.348927	3.18973E-05	0.026576844
833.239986	1398.066066	3.15524E-05	0.026290724
833.279986	1328.59025	2.99844E-05	0.024985427
833.319986	1138.537645	2.56952E-05	0.021412331
833.359986	907.935255	2.04908E-05	0.017076241
833.399986	676.089157	1.52584E-05	0.012716342
833.439986	501.309253	1.13139E-05	0.009429416
833.479986	422.567308	9.53676E-06	0.007948695
833.519986	406.79254	9.18074E-06	0.007652331
833.559986	380.69932	8.59185E-06	0.007161825
833.599986	356.568899	8.04726E-06	0.006708199
833.639986	360.470468	8.13532E-06	0.006781925
833.679986	397.814247	8.97811E-06	0.007484873
833.719986	404.380447	9.1263E-06	0.007608782
833.759986	368.574041	8.3182E-06	0.006935385
833.799986	343.088268	7.74302E-06	0.006456134
833.839986	313.596694	7.07744E-06	0.005901453
833.879986	278.381591	6.28268E-06	0.005239005
833.919986	280.683857	6.33464E-06	0.005282586
833.959986	335.777401	7.57803E-06	0.006319772
833.999986	432.394249	9.75854E-06	0.008138619
834.039986	515.733787	1.16394E-05	0.009707719
834.079986	656.114025	1.48076E-05	0.012350706
834.119986	811.70846	1.83191E-05	0.015280352
834.159986	923.67234	2.0846E-05	0.017388898
834.199986	1059.94156	2.39214E-05	0.019955235
834.239986	1096.279998	2.47415E-05	0.020640359
834.279986	1018.275457	2.29811E-05	0.019172637
834.319986	926.27902	2.09048E-05	0.017441315
834.359986	783.707811	1.76872E-05	0.014757487
834.399986	643.897584	1.45319E-05	0.012125393
834.439986	517.328106	1.16754E-05	0.0097424
834.479986	419.29986	9.46301E-06	0.007896696

834.519986	367.747927	8.29956E-06	0.006926147
834.559986	356.211203	8.03919E-06	0.006709187
834.599986	372.727478	8.41194E-06	0.007020605
834.639986	384.021946	8.66684E-06	0.007233692
834.679986	405.338097	9.14792E-06	0.007635583
834.719986	413.239934	9.32625E-06	0.007784807
834.759986	388.491214	8.76771E-06	0.00731893
834.799986	382.341844	8.62892E-06	0.007203425
834.839986	391.075733	8.82603E-06	0.007368327
834.879986	396.026284	8.93776E-06	0.007461958
834.919986	405.075355	9.14199E-06	0.007632827
834.959986	458.830625	1.03552E-05	0.008646151
834.999986	560.97118	1.26603E-05	0.010571382
835.039986	678.35575	1.53095E-05	0.012784082
835.079986	841.285416	1.89866E-05	0.015855363
835.119986	1039.14117	2.3452E-05	0.019585208
835.159986	1243.768542	2.80701E-05	0.023443044
835.199986	1358.625304	3.06623E-05	0.025609136
835.239986	1343.886159	3.03296E-05	0.025332527
835.279986	1200.545964	2.70947E-05	0.022631619
835.319986	1009.366021	2.278E-05	0.019028577
835.359986	839.067733	1.89366E-05	0.01581887
835.399986	693.175164	1.5644E-05	0.013068996
835.439986	539.237795	1.21698E-05	0.010167176
835.479986	416.292309	9.39514E-06	0.007849449
835.519986	347.247838	7.8369E-06	0.006547887
835.559986	328.345177	7.41029E-06	0.006191745
835.599986	345.240996	7.79161E-06	0.006510668
835.639986	367.978553	8.30476E-06	0.006939792
835.679986	373.615518	8.43198E-06	0.007046438
835.719986	356.709962	8.05045E-06	0.006727919
835.759986	343.455612	7.75131E-06	0.006478239
835.799986	325.223506	7.33984E-06	0.006134639
835.839986	303.529783	6.85024E-06	0.005725708
835.879986	307.195542	6.93298E-06	0.005795135
835.919986	349.261729	7.88235E-06	0.006589015
835.959986	377.238915	8.51376E-06	0.00711716
835.999986	415.260228	9.37184E-06	0.007834862
836.039986	510.927423	1.15309E-05	0.00964031
836.079986	669.461232	1.51088E-05	0.012632172
836.119986	887.491187	2.00294E-05	0.016747015
836.159986	977.13165	2.20525E-05	0.018439418
836.199986	954.177969	2.15345E-05	0.018007122
836.239986	947.848827	2.13916E-05	0.017888535
836.279986	896.371889	2.02299E-05	0.016917831
836.319986	795.202017	1.79466E-05	0.015009102

836.359986	667.839364	1.50722E-05	0.012605789
836.399986	547.065024	1.23465E-05	0.010326609
836.439986	439.870027	9.92725E-06	0.008303552
836.479986	365.941447	8.25879E-06	0.006908311
836.519986	321.289157	7.25105E-06	0.006065647
836.559986	323.390098	7.29846E-06	0.006105603
836.599986	344.438545	7.7735E-06	0.006503308
836.639986	369.209039	8.33253E-06	0.006971331
836.679986	383.274377	8.64997E-06	0.007237256
836.719986	382.114993	8.6238E-06	0.007215708
836.759986	371.234086	8.37824E-06	0.007010573
836.799986	368.089808	8.30727E-06	0.006951527
836.839986	369.543916	8.34009E-06	0.006979322
836.879986	377.26577	8.51436E-06	0.0071255
836.919986	394.786548	8.90978E-06	0.007456775
836.959986	437.282505	9.86886E-06	0.008259838
836.999986	510.553206	1.15225E-05	0.009644311
837.039986	633.288074	1.42924E-05	0.011963335
837.079986	774.813295	1.74865E-05	0.014637563
837.119986	974.315237	2.19889E-05	0.018407379
837.159986	1156.497847	2.61005E-05	0.021850332
837.199986	1249.03951	2.81891E-05	0.023599899
837.239986	1237.878398	2.79372E-05	0.023390134
837.279986	1148.001394	2.59088E-05	0.021692913
837.319986	979.611156	2.21085E-05	0.018511855
837.359986	773.684805	1.7461E-05	0.014621133
837.399986	594.138476	1.34089E-05	0.011228594
837.439986	467.153311	1.0543E-05	0.008829129
837.479986	390.14407	8.80501E-06	0.007374018
837.519986	348.179787	7.85793E-06	0.006581176
837.559986	315.616727	7.12303E-06	0.005965965
837.599986	314.102438	7.08885E-06	0.005937624
837.639986	319.382702	7.20802E-06	0.006037728
837.679986	323.757337	7.30675E-06	0.00612072
837.719986	343.80207	7.75913E-06	0.006499981
837.759986	373.886586	8.4381E-06	0.007069102
837.799986	351.433897	7.93137E-06	0.006644905
837.839986	303.109493	6.84076E-06	0.005731461
837.879986	291.038577	6.56834E-06	0.005503477
837.919986	322.559667	7.27972E-06	0.006099825
837.959986	346.440638	7.81868E-06	0.006551743
837.999986	387.242565	8.73953E-06	0.007323722
838.039986	490.481664	1.10695E-05	0.009276674
838.079986	624.626915	1.4097E-05	0.01181438
838.119986	771.240859	1.74058E-05	0.014588176
838.159986	869.279828	1.96184E-05	0.016443387

838.199986	937.728087	2.11632E-05	0.017739008
838.239986	955.46005	2.15634E-05	0.018075306
838.279986	889.689335	2.0079E-05	0.016831865
838.319986	755.529221	1.70512E-05	0.014294397
838.359986	615.914757	1.39003E-05	0.011653487
838.399986	491.366009	1.10894E-05	0.009297392
838.439986	395.667188	8.92966E-06	0.007486982
838.479986	362.552669	8.18231E-06	0.006860702
838.519986	341.944497	7.71721E-06	0.006471036
838.559986	346.427414	7.81838E-06	0.006556184
838.599986	357.897112	8.07724E-06	0.006773573
838.639986	356.511153	8.04596E-06	0.006747664
838.679986	358.920395	8.10033E-06	0.006793587
838.719986	371.781746	8.3906E-06	0.007037361
838.759986	390.785241	8.81948E-06	0.007397426
838.799986	372.081338	8.39736E-06	0.007043703
838.839986	353.767701	7.98404E-06	0.006697336
838.879986	351.243792	7.92708E-06	0.006649871
838.919986	352.068458	7.94569E-06	0.006665802
838.959986	414.069034	9.34496E-06	0.007840049
838.999986	516.669333	1.16605E-05	0.009783165
839.039986	684.075287	1.54386E-05	0.012953625
839.079986	920.09766	2.07653E-05	0.017423767
839.119986	1161.052794	2.62033E-05	0.02198775
839.159986	1427.932619	3.22265E-05	0.027043148
839.199986	1613.080675	3.6405E-05	0.030551062
839.239986	1599.418306	3.60966E-05	0.030293746
839.279986	1371.14335	3.09448E-05	0.025971348
839.319986	1072.848156	2.42127E-05	0.020322193
839.359986	832.807742	1.87953E-05	0.015776032
839.399986	661.12664	1.49207E-05	0.012524442
839.439986	483.614007	1.09145E-05	0.009162063
839.479986	357.337915	8.06462E-06	0.006770086
839.519986	303.798494	6.85631E-06	0.005756008
839.559986	296.516847	6.69197E-06	0.005618312
839.599986	356.108348	8.03687E-06	0.006747755
839.639986	445.282221	1.00494E-05	0.008437878
839.679986	518.762395	1.17077E-05	0.009830759
839.719986	515.202642	1.16274E-05	0.009763765
839.759986	462.136508	1.04298E-05	0.00875851
839.799986	403.054279	9.09637E-06	0.007639134
839.839986	360.263618	8.13065E-06	0.006828443
839.879986	318.599953	7.19036E-06	0.006039037
839.919986	286.046264	6.45567E-06	0.005422243
839.959986	301.278374	6.79943E-06	0.005711252
839.999986	385.399132	8.69792E-06	0.007306254

840.039986	487.521343	1.10027E-05	0.009242689
840.079986	610.235159	1.37722E-05	0.011569714
840.119986	797.429167	1.79969E-05	0.015119526
840.159986	1001.418471	2.26006E-05	0.018988137
840.199986	1109.876326	2.50484E-05	0.021045634
840.239986	1124.82188	2.53857E-05	0.021330049
840.279986	1015.970201	2.2929E-05	0.019266807
840.319986	850.556632	1.91959E-05	0.01613068
840.359986	685.426754	1.54691E-05	0.012999635
840.399986	554.64552	1.25176E-05	0.010519771
840.439986	450.312104	1.01629E-05	0.008541322
840.479986	395.514951	8.92622E-06	0.007502311
840.519986	358.1821	8.08367E-06	0.006794487
840.559986	329.326509	7.43244E-06	0.006247412
840.599986	324.139765	7.31538E-06	0.006149311
840.639986	324.365669	7.32048E-06	0.006153889
840.679986	353.994272	7.98916E-06	0.006716325
840.719986	377.062517	8.50978E-06	0.007154339
840.759986	382.461803	8.63163E-06	0.007257129
840.799986	374.033081	8.44141E-06	0.007097534
840.839986	362.634404	8.18415E-06	0.006881563
840.879986	346.348335	7.8166E-06	0.006572822
840.919986	344.141708	7.7668E-06	0.006531257
840.959986	400.511056	9.03898E-06	0.007601418
840.999986	508.589796	1.14782E-05	0.009653135
841.039986	623.142535	1.40635E-05	0.011827931
841.079986	758.423455	1.71166E-05	0.014396397
841.119986	960.214274	2.16707E-05	0.018227658
841.159986	1157.874373	2.61316E-05	0.021980866
841.199986	1293.010513	2.91814E-05	0.024547432
841.239986	1290.585289	2.91267E-05	0.024502555
841.279986	1164.73133	2.62864E-05	0.022114192
841.319986	984.170579	2.22114E-05	0.01868686
841.359986	802.176705	1.8104E-05	0.01523199
841.399986	615.870267	1.38993E-05	0.0116949
841.439986	485.564777	1.09585E-05	0.009220937
841.479986	412.041951	9.29921E-06	0.007825102
841.519986	351.87737	7.94138E-06	0.006682832
841.559986	327.529074	7.39187E-06	0.006220706
841.599986	344.651137	7.7783E-06	0.006546214
841.639986	364.786826	8.23273E-06	0.006928995
841.679986	357.177796	8.06101E-06	0.006784787
841.719986	348.132634	7.85687E-06	0.006613283
841.759986	329.805501	7.44325E-06	0.006265431
841.799986	317.259507	7.16011E-06	0.006027376
841.839986	329.131461	7.42804E-06	0.00625322

841.879986	321.884809	7.26449E-06	0.00611583
841.919986	276.405266	6.23808E-06	0.005251966
841.959986	272.543645	6.15093E-06	0.005178837
841.999986	365.719865	8.25379E-06	0.006949689
842.039986	505.917064	1.14178E-05	0.009614281
842.079986	648.770341	1.46418E-05	0.012329603
842.119986	814.737389	1.83875E-05	0.015484471
842.159986	937.117928	2.11494E-05	0.017811217
842.199986	995.756451	2.24728E-05	0.018926622
842.239986	997.783217	2.25186E-05	0.018966046
842.279986	910.555601	2.055E-05	0.01730883
842.319986	799.275303	1.80385E-05	0.015194215
842.359986	674.537219	1.52234E-05	0.012823554
842.399986	549.829588	1.24089E-05	0.010453247
842.439986	457.687103	1.03294E-05	0.008701866
842.479986	378.743119	8.5477E-06	0.00720127
842.519986	357.536988	8.06911E-06	0.006798388
842.559986	356.533775	8.04647E-06	0.006779634
842.599986	362.922161	8.19065E-06	0.00690144
842.639986	362.916415	8.19052E-06	0.006901658
842.679986	345.753934	7.80318E-06	0.006575588
842.719986	344.604891	7.77725E-06	0.006554046
842.759986	350.739917	7.91571E-06	0.006671045
842.799986	348.060244	7.85523E-06	0.006620392
842.839986	350.344994	7.9068E-06	0.006664166
842.879986	354.724584	8.00564E-06	0.006747794
842.919986	382.553804	8.63371E-06	0.007277524
842.959986	463.114482	1.04518E-05	0.00881049
842.999986	566.7665	1.27911E-05	0.010782923
843.039986	663.19639	1.49674E-05	0.012618133
843.079986	797.536572	1.79993E-05	0.015174841
843.119986	1001.155939	2.25947E-05	0.019050039
843.159986	1175.329396	2.65255E-05	0.02236528
843.199986	1226.477925	2.76799E-05	0.02333969
843.239986	1205.675711	2.72104E-05	0.022944915
843.279986	1107.747843	2.50003E-05	0.021082274
843.319986	928.328497	2.09511E-05	0.017668465
843.359986	774.802525	1.74862E-05	0.014747173
843.399986	652.076909	1.47165E-05	0.012411869
843.439986	521.06856	1.17598E-05	0.009918678
843.479986	414.480672	9.35425E-06	0.007890124
843.519986	330.567117	7.46044E-06	0.00629303
843.559986	285.878932	6.45189E-06	0.005442556
843.599986	299.791276	6.76587E-06	0.005707689
843.639986	346.123441	7.81152E-06	0.006590114
843.679986	372.903923	8.41592E-06	0.007100345

843.719986	359.326387	8.1095E-06	0.006842144
843.759986	358.763023	8.09678E-06	0.00683174
843.799986	350.747797	7.91589E-06	0.006679427
843.839986	329.281131	7.43142E-06	0.006270926
843.879986	309.262555	6.97963E-06	0.005889966
843.919986	303.091917	6.84036E-06	0.005772719
843.959986	338.014825	7.62852E-06	0.006438169
843.999986	424.203669	9.57369E-06	0.008080191
844.039986	507.834281	1.14611E-05	0.009673637
844.079986	642.247643	1.44946E-05	0.012234631
844.119986	848.481419	1.9149E-05	0.016164091
844.159986	1003.406262	2.26455E-05	0.01911641
844.199986	1061.142796	2.39485E-05	0.020217336
844.239986	1054.213696	2.37921E-05	0.020086271
844.279986	945.492317	2.13384E-05	0.018015622
844.319986	778.380316	1.7567E-05	0.014832135
844.359986	643.505461	1.4523E-05	0.012262659
844.399986	519.137229	1.17162E-05	0.009893163
844.439986	441.054829	9.95399E-06	0.00840555
844.479986	390.507672	8.81321E-06	0.007442583
844.519986	361.838841	8.1662E-06	0.006896518
844.559986	352.375244	7.95262E-06	0.006716463
844.599986	383.710257	8.65981E-06	0.007314072
844.639986	409.907204	9.25103E-06	0.007813794
844.679986	418.782286	9.45133E-06	0.007983352
844.719986	437.040658	9.8634E-06	0.00833181
844.759986	438.665903	9.90008E-06	0.00836319
844.799986	398.87117	9.00197E-06	0.007604861
844.839986	352.488802	7.95518E-06	0.006720855
844.879986	351.428178	7.93124E-06	0.00670095
844.919986	395.67976	8.92994E-06	0.007545086
844.959986	440.492741	9.94131E-06	0.008400007
844.999986	565.303618	1.27581E-05	0.010780607
845.039986	729.802692	1.64706E-05	0.013918341
845.079986	873.351095	1.97103E-05	0.016656795
845.119986	1051.682096	2.3735E-05	0.020058923
845.159986	1259.741525	2.84306E-05	0.024028415
845.199986	1359.757909	3.06878E-05	0.025937364
845.239986	1278.228045	2.88478E-05	0.024383337
845.279986	1114.478812	2.51522E-05	0.02126068
845.319986	948.351949	2.1403E-05	0.018092369
845.359986	773.364555	1.74538E-05	0.014754711
845.399986	628.044859	1.41741E-05	0.011982782
845.439986	537.665765	1.21344E-05	0.010258879
845.479986	465.200895	1.04989E-05	0.00887664
845.519986	407.46389	9.19589E-06	0.007775311

845.559986	360.910102	8.14524E-06	0.006887288
845.599986	326.00229	7.35742E-06	0.006221432
845.639986	329.849275	7.44424E-06	0.006295146
845.679986	357.305301	8.06388E-06	0.006819464
845.719986	360.519969	8.13643E-06	0.006881144
845.759986	353.312756	7.97378E-06	0.006743901
845.799986	334.519794	7.54965E-06	0.00638549
845.839986	324.869671	7.33186E-06	0.006201577
845.879986	320.743365	7.23873E-06	0.006123098
845.919986	321.317017	7.25168E-06	0.006134339
845.959986	338.056788	7.62947E-06	0.006454227
845.999986	398.720071	8.99856E-06	0.007612779
846.039986	497.66197	1.12315E-05	0.00950233
846.079986	694.552555	1.56751E-05	0.013262375
846.119986	892.093596	2.01333E-05	0.017035196
846.159986	999.529669	2.2558E-05	0.019087671
846.199986	1074.322705	2.4246E-05	0.020516937
846.239986	1138.669194	2.56982E-05	0.021746826
846.279986	1102.880387	2.48905E-05	0.02106431
846.319986	951.681432	2.14781E-05	0.018177366
846.359986	773.750617	1.74625E-05	0.014779539
846.399986	623.519039	1.4072E-05	0.011910504
846.439986	503.448551	1.13621E-05	0.009617363
846.479986	411.642814	9.2902E-06	0.007863973
846.519986	346.941067	7.82998E-06	0.006628232
846.559986	303.822141	6.85684E-06	0.005804729
846.599986	317.625409	7.16836E-06	0.006068736
846.639986	368.722966	8.32156E-06	0.007045369
846.679986	410.526043	9.265E-06	0.007844491
846.719986	441.503635	9.96412E-06	0.008436821
846.759986	446.676979	1.00809E-05	0.008536083
846.799986	421.012782	9.50167E-06	0.008046016
846.839986	389.152894	8.78264E-06	0.00743749
846.879986	395.930756	8.93561E-06	0.007567386
846.919986	449.18322	1.01374E-05	0.0085856
846.959986	518.403333	1.16996E-05	0.009909128
846.999986	631.042831	1.42418E-05	0.012062769
847.039986	762.835469	1.72161E-05	0.014582754
847.079986	927.974198	2.09431E-05	0.017740468
847.119986	1182.413309	2.66854E-05	0.022605754
847.159986	1410.177933	3.18258E-05	0.026961503
847.199986	1507.082951	3.40128E-05	0.028815612
847.239986	1422.922384	3.21134E-05	0.027207736
847.279986	1239.685449	2.7978E-05	0.023705177
847.319986	1078.292718	2.43356E-05	0.02062001
847.359986	909.910922	2.05354E-05	0.017400895

847.399986	717.361949	1.61899E-05	0.013719287
847.439986	557.606609	1.25844E-05	0.010664528
847.479986	439.752054	9.92459E-06	0.008410893
847.519986	373.954003	8.43962E-06	0.007152747
847.559986	334.047929	7.539E-06	0.006389752
847.599986	314.136882	7.08963E-06	0.006009172
847.639986	328.755765	7.41956E-06	0.006289115
847.679986	355.716822	8.02803E-06	0.006805203
847.719986	361.853673	8.16653E-06	0.006922934
847.759986	355.300492	8.01864E-06	0.00679788
847.799986	327.200642	7.38446E-06	0.006260547
847.839986	319.533802	7.21143E-06	0.006114141
847.879986	310.482014	7.00715E-06	0.005941219
847.919986	311.759324	7.03597E-06	0.005965943
847.959986	321.43809	7.25441E-06	0.006151449
847.999986	397.917485	8.98044E-06	0.007615416
848.039986	541.718374	1.22258E-05	0.010367992
848.079986	726.953628	1.64063E-05	0.013913881
848.119986	947.923218	2.13933E-05	0.018144091
848.159986	1120.73064	2.52933E-05	0.02145279
848.199986	1215.246655	2.74264E-05	0.023263093
848.239986	1178.330469	2.65933E-05	0.022557481
848.279986	1018.664087	2.29898E-05	0.019501812
848.319986	836.371514	1.88757E-05	0.016012668
848.359986	663.651037	1.49777E-05	0.012706464
848.399986	500.672048	1.12995E-05	0.009586471
848.439986	422.428083	9.53361E-06	0.008088699
848.479986	377.808435	8.52661E-06	0.007234658
848.519986	341.404686	7.70503E-06	0.00653787
848.559986	321.452045	7.25472E-06	0.006156069
848.599986	358.178997	8.0836E-06	0.006859744
848.639986	411.228868	9.28086E-06	0.007876111
848.679986	441.233689	9.95803E-06	0.008451181
848.719985	464.051222	1.0473E-05	0.008888636
848.759985	464.205496	1.04765E-05	0.00889201
848.799985	434.366385	9.80304E-06	0.008320824
848.839985	407.960921	9.20711E-06	0.007815363
848.879985	382.433798	8.631E-06	0.007326682
848.919985	404.357403	9.12578E-06	0.00774706
848.959985	453.580915	1.02367E-05	0.00869054
848.999985	550.439481	1.24227E-05	0.010546832
849.039985	710.522647	1.60355E-05	0.013614786
849.079985	893.81589	2.01722E-05	0.017127794
849.119985	1097.830893	2.47765E-05	0.021038233
849.159985	1343.411391	3.03189E-05	0.025745617
849.199985	1497.819111	3.38037E-05	0.028706094

849.239985	1461.895897	3.2993E-05	0.028018936
849.279985	1286.150846	2.90266E-05	0.024651739
849.319985	1107.401763	2.49925E-05	0.021226643
849.359985	910.097739	2.05396E-05	0.017445547
849.399985	705.082702	1.59127E-05	0.013516276
849.439985	576.664914	1.30145E-05	0.011055057
849.479985	521.818954	1.17767E-05	0.010004094
849.519985	442.860211	9.99474E-06	0.00849073
849.559985	391.785827	8.84206E-06	0.007511861
849.599985	380.943028	8.59735E-06	0.007304311
849.639985	397.567322	8.97254E-06	0.007623429
849.679985	415.116551	9.3686E-06	0.007960314
849.719985	418.896235	9.4539E-06	0.008033172
849.759985	397.41658	8.96914E-06	0.007621615
849.799985	368.32825	8.31266E-06	0.007064095
849.839985	331.087477	7.47218E-06	0.00635016
849.879985	307.305455	6.93546E-06	0.005894305
849.919985	325.980659	7.35693E-06	0.006252801
849.959985	374.22123	8.44565E-06	0.007178466
849.999985	423.307222	9.55345E-06	0.008120436
850.039985	527.594634	1.19071E-05	0.010121491
850.079985	664.277495	1.49918E-05	0.012744244
850.119985	774.113367	1.74707E-05	0.014852158
850.159985	874.579405	1.9738E-05	0.016780491
850.199985	958.425145	2.16303E-05	0.018390098
850.239985	953.691677	2.15235E-05	0.018300134
850.279985	907.362277	2.04779E-05	0.017411951
850.319985	812.327214	1.83331E-05	0.015588996
850.359985	679.435094	1.53339E-05	0.013039338
850.399985	564.221653	1.27337E-05	0.010828736
850.439985	488.714687	1.10296E-05	0.009380021
850.479985	434.809963	9.81306E-06	0.008345807
850.519985	386.288518	8.71799E-06	0.007414828
850.559985	362.395297	8.17876E-06	0.006956523
850.599985	384.309235	8.67332E-06	0.007377529
850.639985	418.534138	9.44573E-06	0.008034918
850.679985	427.29527	9.64346E-06	0.008203498
850.719985	430.608557	9.71824E-06	0.008267497
850.759985	424.399596	9.57811E-06	0.008148671
850.799985	386.48942	8.72253E-06	0.007421127
850.839985	363.209942	8.19714E-06	0.006974457
850.879985	361.339131	8.15492E-06	0.006938859
850.919985	375.153429	8.46669E-06	0.007204476
850.959985	448.069011	1.01123E-05	0.008605157
850.999985	547.786141	1.23628E-05	0.010520717
851.039985	723.84579	1.63362E-05	0.013902752

851.079985	977.060268	2.20509E-05	0.018767071
851.119985	1234.967465	2.78715E-05	0.023721987
851.159985	1446.326989	3.26416E-05	0.027783211
851.199985	1544.702474	3.48618E-05	0.029674349
851.239985	1533.151289	3.46011E-05	0.029453831
851.279985	1395.083869	3.14851E-05	0.026802635
851.319985	1208.810347	2.72812E-05	0.023225002
851.359985	988.251003	2.23034E-05	0.018988264
851.399985	772.390956	1.74318E-05	0.014841424
851.439985	577.818606	1.30406E-05	0.011103255
851.479985	419.686667	9.47174E-06	0.008065
851.519985	353.180041	7.97078E-06	0.00678728
851.559985	355.230744	8.01706E-06	0.00682701
851.599985	365.078772	8.23932E-06	0.007016604
851.639985	345.25978	7.79203E-06	0.006636006
851.679985	332.803418	7.51091E-06	0.006396891
851.719985	340.414723	7.68269E-06	0.006543497
851.759985	324.996981	7.33473E-06	0.006247429
851.799985	313.714445	7.0801E-06	0.006030827
851.839985	291.974923	6.58947E-06	0.005613172
851.879985	277.788097	6.26929E-06	0.005340683
851.919985	268.719102	6.06462E-06	0.005166567
851.959985	278.042464	6.27503E-06	0.005346075
851.999985	354.429203	7.99897E-06	0.006815125
852.039985	491.643136	1.10957E-05	0.009453981
852.079985	686.976698	1.55041E-05	0.013210739
852.119985	927.780097	2.09387E-05	0.017842289
852.159985	1200.323084	2.70896E-05	0.02308469
852.199985	1347.456529	3.04102E-05	0.025915586
852.239985	1299.895869	2.93368E-05	0.025002027
852.279985	1148.192875	2.59131E-05	0.022085228
852.319985	942.08863	2.12616E-05	0.018121711
852.359985	756.669366	1.7077E-05	0.014555729
852.399985	645.549924	1.45692E-05	0.012418754
852.439985	570.148659	1.28675E-05	0.010968738
852.479985	521.898969	1.17785E-05	0.010040963
852.519985	464.45701	1.04821E-05	0.00893624
852.559985	436.910445	9.86046E-06	0.008406634
852.599985	438.508007	9.89651E-06	0.008437768
852.639985	454.062394	1.02476E-05	0.008737476
852.679985	437.552731	9.87496E-06	0.008420177
852.719985	417.273466	9.41728E-06	0.008030304
852.759985	416.312883	9.3956E-06	0.008012193
852.799985	374.077537	8.44241E-06	0.007199686
852.839985	343.824098	7.75963E-06	0.006617724
852.879985	330.239497	7.45305E-06	0.006356553

852.919985	339.71256	7.66684E-06	0.0065392
852.959985	399.302853	9.01171E-06	0.007686627
852.999985	511.677503	1.15478E-05	0.009850315
853.039985	642.804032	1.45072E-05	0.012375215
853.079985	814.700523	1.83867E-05	0.015685288
853.119985	1017.41959	2.29617E-05	0.019589122
853.159985	1249.241792	2.81936E-05	0.024053692
853.199985	1432.225761	3.23233E-05	0.027578274
853.239985	1416.04196	3.19581E-05	0.027267924
853.279985	1249.891359	2.82083E-05	0.024069584
853.319985	1097.347596	2.47656E-05	0.021132987
853.359985	939.585	2.12051E-05	0.018095605
853.399985	744.932208	1.68121E-05	0.014347431
853.439985	582.006039	1.31351E-05	0.01120999
853.479985	482.549654	1.08905E-05	0.009294801
853.519985	428.348287	9.66722E-06	0.008251169
853.559985	406.372302	9.17126E-06	0.007828218
853.599985	397.290155	8.96629E-06	0.007653621
853.639985	380.87555	8.59583E-06	0.007337745
853.679985	353.854228	7.986E-06	0.006817486
853.719985	328.114131	7.40508E-06	0.006321864
853.759985	310.509362	7.00776E-06	0.005982948
853.799985	279.298081	6.30337E-06	0.005381816
853.839985	273.200072	6.16575E-06	0.00526456
853.879985	282.633093	6.37864E-06	0.005446589
853.919985	288.427812	6.50941E-06	0.005558519
853.959985	323.762092	7.30686E-06	0.006239766
853.999985	412.088181	9.30026E-06	0.007942419
854.039985	528.725357	1.19326E-05	0.010190913
854.079985	628.45886	1.41834E-05	0.012113793
854.119985	702.412604	1.58525E-05	0.013539915
854.159985	797.356771	1.79952E-05	0.015370807
854.199985	899.625761	2.03033E-05	0.017343079
854.239985	979.358095	2.21027E-05	0.018881051
854.279985	909.45649	2.05252E-05	0.017534239
854.319985	783.600504	1.76848E-05	0.015108454
854.359985	689.735201	1.55664E-05	0.013299277
854.399985	582.601353	1.31485E-05	0.011234079
854.439985	479.840948	1.08293E-05	0.009253023
854.479985	421.242644	9.50686E-06	0.008123421
854.519985	371.681074	8.38832E-06	0.007167991
854.559985	351.223796	7.92663E-06	0.006773782
854.599985	351.97953	7.94369E-06	0.006788675
854.639985	353.443257	7.97672E-06	0.006817226
854.679985	365.386479	8.24626E-06	0.007047917
854.719985	400.598469	9.04095E-06	0.00772748

854.759985	404.368751	9.12604E-06	0.007800573
854.799985	367.80034	8.30074E-06	0.007095474
854.839985	320.090201	7.22399E-06	0.006175355
854.879985	317.425235	7.16385E-06	0.006124228
854.919985	356.569306	8.04727E-06	0.006879774
854.959985	394.866906	8.9116E-06	0.007619058
854.999985	462.384834	1.04354E-05	0.008922251
855.039985	583.725706	1.31739E-05	0.011264191
855.079985	763.892624	1.724E-05	0.014741573
855.119985	977.210254	2.20543E-05	0.018859051
855.159985	1107.076781	2.49852E-05	0.021366327
855.199985	1194.44313	2.69569E-05	0.023053556
855.239985	1170.091296	2.64073E-05	0.022584605
855.279985	1054.189213	2.37916E-05	0.020348464
855.319985	935.158894	2.11052E-05	0.018051728
855.359985	806.296364	1.8197E-05	0.015564974
855.399985	668.693523	1.50915E-05	0.012909253
855.439985	547.113428	1.23476E-05	0.01056262
855.479985	432.902064	9.77E-06	0.008358037
855.519985	348.662071	7.86882E-06	0.00673193
855.559985	315.399779	7.11813E-06	0.00608999
855.599985	321.834013	7.26335E-06	0.006214518
855.639985	360.599458	8.13823E-06	0.006963393
855.679985	377.185729	8.51256E-06	0.007284024
855.719985	378.277118	8.53719E-06	0.007305442
855.759985	375.15686	8.46677E-06	0.007245521
855.799985	350.678746	7.91433E-06	0.006773084
855.839985	313.102352	7.06628E-06	0.006047608
855.879985	297.38662	6.7116E-06	0.005744326
855.919985	296.144104	6.68356E-06	0.005720593
855.959985	335.579175	7.57355E-06	0.00648266
855.999985	394.022988	8.89255E-06	0.007612023
856.039985	468.476607	1.05729E-05	0.009050795
856.079985	578.615428	1.30585E-05	0.011179158
856.119985	750.4621	1.69369E-05	0.014500005
856.159985	888.549088	2.00533E-05	0.017168846
856.199985	958.534463	2.16328E-05	0.018521993
856.239985	946.275667	2.13561E-05	0.018285967
856.279985	861.696582	1.94473E-05	0.016652327
856.319985	747.560924	1.68714E-05	0.014447325
856.359985	652.495155	1.47259E-05	0.012610677
856.399985	561.16268	1.26647E-05	0.010846015
856.439985	488.743712	1.10303E-05	0.00944676
856.479985	452.17919	1.02051E-05	0.008740425
856.519985	450.16605	1.01596E-05	0.008701918
856.559985	439.107048	9.91003E-06	0.008488539

856.599985	410.81868	9.27161E-06	0.007942057
856.639985	408.652844	9.22273E-06	0.007900555
856.679985	397.230728	8.96494E-06	0.007680088
856.719985	377.65817	8.52322E-06	0.007302012
856.759985	381.445274	8.60869E-06	0.00737558
856.799985	396.761724	8.95436E-06	0.007672095
856.839985	390.609194	8.81551E-06	0.007553478
856.879985	360.040418	8.12561E-06	0.006962673
856.919985	370.190105	8.35468E-06	0.007159288
856.959985	430.583428	9.71767E-06	0.008327653
856.999985	493.767075	1.11436E-05	0.009550095
857.039985	579.141784	1.30704E-05	0.011201875
857.079985	715.74836	1.61534E-05	0.013844793
857.119985	911.513935	2.05716E-05	0.017632329
857.159985	1079.012616	2.43518E-05	0.020873399
857.199985	1137.56006	2.56731E-05	0.02200702
857.239985	1125.118954	2.53924E-05	0.021767353
857.279985	1031.950449	2.32897E-05	0.01996578
857.319985	880.23233	1.98656E-05	0.017031191
857.359985	742.502198	1.67572E-05	0.014366987
857.399985	616.710055	1.39183E-05	0.011933539
857.439985	511.773939	1.155E-05	0.009903453
857.479985	430.05752	9.7058E-06	0.008322529
857.519985	379.58536	8.56671E-06	0.007346127
857.559985	346.031098	7.80944E-06	0.006697063
857.599985	318.110691	7.17932E-06	0.006156981
857.639985	346.243835	7.81424E-06	0.006701806
857.679985	376.848436	8.50494E-06	0.00729452
857.719985	374.017776	8.44106E-06	0.007240066
857.759985	343.891025	7.76114E-06	0.006657197
857.799985	314.596363	7.1E-06	0.006090381
857.839985	333.58305	7.5285E-06	0.006458252
857.879985	332.853565	7.51204E-06	0.00644443
857.919985	328.977346	7.42456E-06	0.006369679
857.959985	350.782402	7.91667E-06	0.006792186
857.999985	390.485147	8.81271E-06	0.007561302
858.039985	484.909991	1.09437E-05	0.009390169
858.079985	608.39077	1.37305E-05	0.011781895
858.119985	734.318843	1.65726E-05	0.014221239
858.159985	849.155746	1.91643E-05	0.016446003
858.199985	922.96917	2.08301E-05	0.017876416
858.239985	910.605983	2.05511E-05	0.017637783
858.279985	818.259949	1.8467E-05	0.015849846
858.319985	721.571073	1.62849E-05	0.013977616
858.359985	640.06329	1.44453E-05	0.012399299
858.399985	560.842808	1.26574E-05	0.010865147

858.439985	460.08899	1.03836E-05	0.00891367
858.479985	389.607967	8.79291E-06	0.007548537
858.519985	344.939072	7.78479E-06	0.006683402
858.559985	321.667222	7.25958E-06	0.006232786
858.599985	309.499172	6.98497E-06	0.005997291
858.639985	338.318641	7.63538E-06	0.006556043
858.679985	379.332873	8.56101E-06	0.007351172
858.719985	395.931075	8.93561E-06	0.007673189
858.759985	378.354341	8.53893E-06	0.007332892
858.799985	344.558287	7.7762E-06	0.006678201
858.839985	337.935425	7.62673E-06	0.006550142
858.879985	330.294559	7.45429E-06	0.006402339
858.919985	331.542379	7.48245E-06	0.006426826
858.959985	378.83156	8.5497E-06	0.007343851
858.999985	508.20196	1.14694E-05	0.009852223
859.039985	671.115073	1.51461E-05	0.013011133
859.079985	846.42996	1.91027E-05	0.016410786
859.119985	984.817016	2.22259E-05	0.019094756
859.159985	1093.200224	2.4672E-05	0.021197201
859.199985	1153.84589	2.60407E-05	0.022374164
859.239985	1128.498361	2.54686E-05	0.021883671
859.279985	1042.301918	2.35233E-05	0.020213103
859.319985	908.336403	2.04999E-05	0.017615963
859.359985	738.374087	1.66641E-05	0.014320439
859.399985	576.08028	1.30013E-05	0.011173342
859.439985	420.964652	9.50059E-06	0.008165183
859.479985	326.334872	7.36492E-06	0.006330004
859.519985	288.448109	6.50987E-06	0.005595365
859.559985	296.661389	6.69523E-06	0.005754956
859.599985	342.033555	7.71922E-06	0.006635442
859.639985	371.928104	8.3939E-06	0.007215731
859.679985	390.09315	8.80386E-06	0.007568501
859.719985	404.514972	9.12934E-06	0.007848676
859.759985	373.028016	8.41872E-06	0.007238081
859.799985	344.09253	7.76569E-06	0.006676939
859.839985	318.242783	7.1823E-06	0.006175626
859.879985	320.85365	7.24122E-06	0.00622658
859.919985	332.442613	7.50277E-06	0.006451779
859.959985	354.536393	8.00139E-06	0.006880877
859.999985	404.533508	9.12976E-06	0.007851592
860.039985	484.822054	1.09418E-05	0.00941035
860.079985	604.233651	1.36367E-05	0.011728663
860.119985	758.028376	1.71076E-05	0.014714627
860.159985	909.324778	2.05222E-05	0.01765237
860.199985	966.616575	2.18152E-05	0.018765426
860.239985	939.673939	2.12071E-05	0.018243223

860.279985	876.683816	1.97855E-05	0.017021098
860.319985	799.978505	1.80544E-05	0.015532562
860.359985	689.150065	1.55532E-05	0.013381314
860.399985	570.92032	1.28849E-05	0.011086147
860.439985	475.311694	1.07271E-05	0.009230045
860.479985	392.412028	8.85619E-06	0.007620577
860.519985	349.44414	7.88647E-06	0.006786463
860.559985	354.225587	7.99438E-06	0.006879642
860.599985	358.801948	8.09766E-06	0.006968846
860.639985	323.372051	7.29806E-06	0.006280999
860.679985	344.802189	7.78171E-06	0.006697558
860.719985	390.452349	8.81197E-06	0.007584635
860.759985	427.354063	9.64479E-06	0.008301846
860.799985	423.90697	9.56699E-06	0.008235265
860.839985	393.521683	8.88124E-06	0.007645323
860.879985	369.720617	8.34408E-06	0.007183251
860.919985	372.641673	8.41E-06	0.00724034
860.959985	409.363115	9.23876E-06	0.007954199
860.999985	499.915442	1.12824E-05	0.009714142
861.039985	643.842791	1.45306E-05	0.012511458
861.079985	797.408743	1.79964E-05	0.015496344
861.119985	979.944555	2.2116E-05	0.019044516
861.159985	1123.018826	2.5345E-05	0.021826075
861.199985	1200.328913	2.70898E-05	0.023329694
861.239985	1183.156686	2.67022E-05	0.022997001
861.279985	1072.236008	2.41989E-05	0.020842005
861.319985	914.518263	2.06394E-05	0.017777131
861.359985	706.294791	1.59401E-05	0.013730155
861.399985	512.44926	1.15653E-05	0.00996232
861.439985	402.246103	9.07813E-06	0.007820268
861.479985	374.6621	8.4556E-06	0.007284332
861.519985	360.448534	8.13482E-06	0.007008311
861.559985	356.569921	8.04729E-06	0.00693322
861.599985	359.586012	8.11536E-06	0.00699219
861.639985	374.87708	8.46045E-06	0.007289865
861.679985	380.508797	8.58755E-06	0.007399723
861.719985	356.058333	8.03574E-06	0.006924558
861.759985	349.604546	7.89009E-06	0.006799362
861.799985	328.895833	7.42272E-06	0.006396901
861.839985	301.394402	6.80205E-06	0.00586228
861.879985	278.391832	6.28292E-06	0.005415119
861.919985	293.606128	6.62628E-06	0.005711324
861.959985	333.942171	7.53661E-06	0.006496256
861.999985	388.312578	8.76367E-06	0.007554287
862.039985	476.613635	1.07565E-05	0.009272538
862.079985	617.075058	1.39265E-05	0.012005778

862.119985	772.886958	1.7443E-05	0.015037943
862.159985	880.414415	1.98697E-05	0.017130884
862.199985	910.986	2.05597E-05	0.01772656
862.239985	890.982295	2.01082E-05	0.017338119
862.279985	834.081343	1.88241E-05	0.016231605
862.319985	760.184341	1.71563E-05	0.014794222
862.359985	648.396173	1.46334E-05	0.012619257
862.399985	527.997439	1.19162E-05	0.010276502
862.439985	441.050227	9.95389E-06	0.008584632
862.479985	376.900244	8.50611E-06	0.007336353
862.519985	332.237792	7.49814E-06	0.006467299
862.559985	341.513569	7.70749E-06	0.006648169
862.599985	361.499683	8.15854E-06	0.00703756
862.639985	355.907085	8.03233E-06	0.006929007
862.679985	353.722707	7.98303E-06	0.006886799
862.719985	384.100246	8.66861E-06	0.007478581
862.759985	420.912622	9.49941E-06	0.008195712
862.799985	423.528987	9.55846E-06	0.008247039
862.839985	373.689294	8.43365E-06	0.007276888
862.879985	344.674863	7.77883E-06	0.006712198
862.919985	395.215026	8.91945E-06	0.007696774
862.959985	474.177326	1.07015E-05	0.009234985
862.999985	544.303315	1.22842E-05	0.010601237
863.039985	656.714935	1.48211E-05	0.012791238
863.079985	846.240486	1.90985E-05	0.016483506
863.119985	1082.872179	2.44389E-05	0.021093717
863.159985	1297.646746	2.92861E-05	0.025278572
863.199985	1365.868007	3.08257E-05	0.026608777
863.239985	1352.407482	3.0522E-05	0.02634777
863.279985	1228.735072	2.77308E-05	0.023939478
863.319985	1013.847418	2.28811E-05	0.019753732
863.359985	800.402855	1.8064E-05	0.015595715
863.399985	587.882291	1.32677E-05	0.011455319
863.439985	430.217845	9.70942E-06	0.008383499
863.479985	380.942526	8.59734E-06	0.007423633
863.519985	373.350538	8.426E-06	0.007276021
863.559985	361.395334	8.15619E-06	0.007043359
863.599985	336.562231	7.59574E-06	0.006559682
863.639985	339.438814	7.66066E-06	0.006616053
863.679985	341.095515	7.69805E-06	0.006648652
863.719985	320.229977	7.22714E-06	0.006242229
863.759985	337.092931	7.60772E-06	0.006571242
863.799985	336.857548	7.60241E-06	0.006566958
863.839985	311.418016	7.02827E-06	0.006071301
863.879985	300.686316	6.78607E-06	0.005862351
863.919985	329.989792	7.44741E-06	0.006433966

863.959985	364.189072	8.21924E-06	0.007101095
863.999985	412.212423	9.30306E-06	0.008037844
864.039985	522.094645	1.17829E-05	0.010180939
864.079985	631.401748	1.42499E-05	0.012313017
864.119985	735.875407	1.66077E-05	0.014351031
864.159985	867.367696	1.95753E-05	0.016916174
864.199985	956.621673	2.15896E-05	0.018657748
864.239985	937.490667	2.11579E-05	0.018285467
864.279985	857.574097	1.93543E-05	0.016727493
864.319985	757.703291	1.71003E-05	0.014780138
864.359985	646.292405	1.45859E-05	0.012607485
864.399985	512.677427	1.15704E-05	0.010001467
864.439985	408.760375	9.22515E-06	0.007974591
864.479985	342.115958	7.72108E-06	0.00667472
864.519985	325.113295	7.33735E-06	0.006343289
864.559985	338.975704	7.65021E-06	0.006614065
864.599985	338.913772	7.64881E-06	0.006613163
864.639985	348.233618	7.85915E-06	0.006795333
864.679985	359.369919	8.11048E-06	0.007012968
864.719985	375.305995	8.47013E-06	0.007324294
864.759985	412.206492	9.30293E-06	0.008044798
864.799985	414.701422	9.35923E-06	0.008093865
864.839985	373.72746	8.43451E-06	0.0072945
864.879985	360.338229	8.13233E-06	0.007033491
864.919985	371.601987	8.38654E-06	0.007253685
864.959985	434.154718	9.79827E-06	0.008475109
864.999985	503.046723	1.13531E-05	0.0098204
865.039985	632.976882	1.42854E-05	0.012357448
865.079985	827.445824	1.86743E-05	0.016154763
865.119985	1018.501955	2.29862E-05	0.019885795
865.159985	1141.569205	2.57636E-05	0.022289659
865.199985	1194.098502	2.69491E-05	0.023316395
865.239985	1142.88091	2.57932E-05	0.022317334
865.279985	1026.531263	2.31674E-05	0.020046271
865.319985	912.804839	2.06007E-05	0.017826227
865.359985	784.758518	1.77109E-05	0.01532631
865.399985	611.502396	1.38008E-05	0.011943175
865.439985	460.911236	1.04021E-05	0.009002415
865.479985	365.008215	8.23773E-06	0.007129588
865.519985	345.620008	7.80016E-06	0.006751196
865.559985	343.276298	7.74727E-06	0.006705725
865.599985	333.287059	7.52182E-06	0.006510891
865.639985	359.868877	8.12174E-06	0.007030502
865.679985	372.67229	8.41069E-06	0.00728097
865.719985	363.640555	8.20686E-06	0.007104843
865.759985	355.458763	8.02221E-06	0.006945308

865.799985	338.071558	7.6298E-06	0.006605884
865.839985	310.203928	7.00087E-06	0.006061634
865.879985	287.881333	6.49708E-06	0.005625692
865.919985	296.127533	6.68319E-06	0.005787104
865.959985	329.713111	7.44117E-06	0.006443752
865.999985	411.775955	9.29321E-06	0.00804792
866.039985	547.271557	1.23512E-05	0.010696596
866.079985	650.845455	1.46887E-05	0.012721568
866.119985	800.586124	1.80681E-05	0.015649154
866.159985	924.602684	2.0867E-05	0.018074156
866.199985	945.575981	2.13403E-05	0.018484996
866.239985	946.377166	2.13584E-05	0.018501513
866.279985	911.467331	2.05705E-05	0.017819854
866.319985	827.182718	1.86684E-05	0.016172775
866.359985	675.017214	1.52342E-05	0.0131983
866.399985	501.296081	1.13136E-05	0.009802063
866.439985	401.713213	9.06611E-06	0.007855238
866.479985	367.462315	8.29311E-06	0.007185816
866.519985	354.559128	8.00191E-06	0.006933811
866.559985	333.192789	7.5197E-06	0.006516269
866.599985	330.108748	7.45009E-06	0.006456252
866.639985	356.433254	8.0442E-06	0.006971427
866.679985	386.433153	8.72126E-06	0.00755854
866.719985	408.578117	9.22104E-06	0.007992059
866.759985	414.654617	9.35818E-06	0.008111293
866.799985	392.529855	8.85885E-06	0.007678853
866.839985	369.86853	8.34742E-06	0.007235875
866.879985	346.605402	7.8224E-06	0.006781083
866.919985	345.016523	7.78654E-06	0.006750309
866.959985	397.327193	8.96712E-06	0.007774135
866.999985	489.5964	1.10495E-05	0.009579924
867.039985	646.728532	1.45958E-05	0.012655109
867.079985	902.257433	2.03627E-05	0.017656084
867.119985	1202.996103	2.71499E-05	0.023542261
867.159985	1456.143278	3.28631E-05	0.028497587
867.199985	1546.266955	3.48971E-05	0.030262757
867.239985	1492.347868	3.36802E-05	0.029208827
867.279985	1357.01839	3.0626E-05	0.02656133
867.319985	1148.543205	2.5921E-05	0.022481819
867.359985	885.92831	1.99942E-05	0.017342141
867.399985	651.721683	1.47085E-05	0.01275811
867.439985	493.650843	1.1141E-05	0.009664159
867.479985	420.369723	9.48716E-06	0.008229921
867.519985	392.663659	8.86187E-06	0.007687851
867.559985	358.172974	8.08347E-06	0.007012891
867.599985	361.187901	8.15151E-06	0.007072248

867.639985	387.985319	8.75629E-06	0.007597306
867.679985	401.467925	9.06057E-06	0.007861677
867.719985	385.232322	8.69416E-06	0.007544094
867.759985	348.472294	7.86453E-06	0.006824528
867.799985	308.520314	6.96287E-06	0.006042382
867.839985	268.777261	6.06593E-06	0.005264255
867.879985	251.103148	5.66705E-06	0.004918318
867.919985	263.618976	5.94951E-06	0.005163701
867.959985	319.873022	7.21909E-06	0.00626588
867.999985	432.880696	9.76951E-06	0.008479938
868.039985	572.779837	1.29268E-05	0.011221017
868.079985	712.916429	1.60895E-05	0.013967
868.119985	828.385358	1.86955E-05	0.01622994
868.159985	922.924784	2.08291E-05	0.018083014
868.199985	1019.549098	2.30098E-05	0.019977111
868.239985	1089.963074	2.45989E-05	0.021357791
868.279985	1047.072014	2.3631E-05	0.020518287
868.319985	894.089768	2.01784E-05	0.017521274
868.359985	719.277601	1.62331E-05	0.014096169
868.399985	563.197466	1.27106E-05	0.01103787
868.439985	459.072864	1.03606E-05	0.00899759
868.479985	408.02521	9.20856E-06	0.007997451
868.519985	397.08096	8.96156E-06	0.007783298
868.559985	391.57316	8.83726E-06	0.007675691
868.599985	385.299127	8.69566E-06	0.007553054
868.639985	382.873258	8.64092E-06	0.007505845
868.679985	394.861444	8.91147E-06	0.007741218
868.719985	401.033393	9.05077E-06	0.007862581
868.759985	367.933465	8.30375E-06	0.007213962
868.799985	344.165948	7.76735E-06	0.00674827
868.839985	331.465955	7.48072E-06	0.006499553
868.879985	344.00019	7.76361E-06	0.006745641
868.919985	365.872346	8.25723E-06	0.007174871
868.959985	402.865474	9.09211E-06	0.007900682
868.999985	469.505039	1.05961E-05	0.009207989
869.039985	611.346099	1.37972E-05	0.011990344
869.079985	808.968978	1.82573E-05	0.015867057
869.119985	950.774101	2.14576E-05	0.01864927
869.159985	1110.935682	2.50723E-05	0.021791815
869.199985	1239.619431	2.79765E-05	0.024317159
869.239985	1256.509007	2.83577E-05	0.02464961
869.279985	1174.484826	2.65065E-05	0.023041558
869.319985	1032.703122	2.33067E-05	0.020260955
869.359985	822.558313	1.8564E-05	0.016138795
869.399985	631.035385	1.42416E-05	0.012381638
869.439985	501.938623	1.13281E-05	0.009849064

869.479985	404.653236	9.13246E-06	0.007940491
869.519985	343.207011	7.7457E-06	0.006735045
869.559985	322.658799	7.28196E-06	0.006332101
869.599985	339.543896	7.66303E-06	0.006663773
869.639985	347.158557	7.83489E-06	0.006813529
869.679985	343.800645	7.7591E-06	0.006747935
869.719985	363.566351	8.20519E-06	0.007136214
869.759985	357.360769	8.06513E-06	0.007014732
869.799985	335.497791	7.57172E-06	0.00658588
869.839985	304.870252	6.8805E-06	0.005984931
869.879985	287.765692	6.49447E-06	0.00564941
869.919985	291.531276	6.57945E-06	0.005723599
869.959985	314.20894	7.09126E-06	0.006169111
869.999985	370.706187	8.36632E-06	0.0072787
870.039985	448.265117	1.01167E-05	0.00880195
870.079985	537.873342	1.21391E-05	0.010561946
870.119985	689.378369	1.55583E-05	0.013537596
870.159985	870.873192	1.96544E-05	0.017102468
870.199985	929.654657	2.0981E-05	0.018257675
870.239985	910.151704	2.05409E-05	0.017875474
870.279985	860.459705	1.94194E-05	0.016900295
870.319985	707.054282	1.59572E-05	0.013887896
870.359985	544.470646	1.22879E-05	0.010694935
870.399985	457.77461	1.03313E-05	0.008992394
870.439985	407.62848	9.19961E-06	0.008007706
870.479985	365.836154	8.25641E-06	0.007187042
870.519985	343.938188	7.76221E-06	0.006757155
870.559985	348.248491	7.85948E-06	0.006842152
870.599985	378.466994	8.54147E-06	0.007436206
870.639985	393.274499	8.87566E-06	0.007727502
870.679985	388.367073	8.7649E-06	0.007631426
870.719985	366.406963	8.26929E-06	0.00720024
870.759985	346.719717	7.82498E-06	0.00681368
870.799985	327.748787	7.39683E-06	0.006441163
870.839985	305.285543	6.88987E-06	0.005999974
870.879985	310.868296	7.01586E-06	0.006109976
870.919985	342.850875	7.73767E-06	0.006738889
870.959985	393.059066	8.8708E-06	0.007726108
870.999985	453.042985	1.02245E-05	0.008905582
871.039985	562.232272	1.26888E-05	0.011052452
871.079985	745.562241	1.68263E-05	0.014657054
871.119985	941.86134	2.12565E-05	0.018516961
871.159985	1079.777041	2.43691E-05	0.021229353
871.199985	1188.417499	2.68209E-05	0.023366392
871.239985	1197.252871	2.70203E-05	0.023541192
871.279985	1084.278041	2.44706E-05	0.021320783

871.319985	902.876225	2.03767E-05	0.01775459
871.359985	744.983249	1.68132E-05	0.01465038
871.399985	604.958738	1.36531E-05	0.01189729
871.439985	478.070817	1.07894E-05	0.009402308
871.479985	380.606513	8.58976E-06	0.007485803
871.519985	307.014258	6.92888E-06	0.006038661
871.559985	311.646282	7.03342E-06	0.00613005
871.599985	324.367138	7.32051E-06	0.00638056
871.639985	325.81077	7.3531E-06	0.006409252
871.679985	344.83847	7.78252E-06	0.00678387
871.719985	355.219071	8.0168E-06	0.006988404
871.759985	352.326648	7.95152E-06	0.006931818
871.799985	322.712018	7.28316E-06	0.006349459
871.839985	284.934629	6.43058E-06	0.005606435
871.879985	292.805162	6.6082E-06	0.005761561
871.919985	308.595321	6.96457E-06	0.006072545
871.959985	329.556234	7.43763E-06	0.006485312
871.999985	395.251872	8.92028E-06	0.007778488
872.039985	449.769467	1.01507E-05	0.00885179
872.079985	561.166677	1.26648E-05	0.011044675
872.119985	684.85228	1.54562E-05	0.013479628
872.159985	780.455111	1.76138E-05	0.015362038
872.199985	870.518792	1.96464E-05	0.017135587
872.239985	904.020716	2.04025E-05	0.017795866
872.279985	852.089101	1.92305E-05	0.016774349
872.319985	789.165143	1.78104E-05	0.015536331
872.359985	724.574969	1.63526E-05	0.014265396
872.399985	596.078132	1.34527E-05	0.011736094
872.439985	502.25557	1.13352E-05	0.009889289
872.479985	482.33608	1.08857E-05	0.009497515
872.519985	514.742593	1.1617E-05	0.010136085
872.559985	529.137007	1.19419E-05	0.010420011
872.599985	504.771324	1.1392E-05	0.009940647
872.639985	463.960942	1.0471E-05	0.009137372
872.679985	420.832519	9.4976E-06	0.008288369
872.719985	408.549495	9.22039E-06	0.008046821
872.759985	408.086498	9.20994E-06	0.00803807
872.799985	414.171998	9.34729E-06	0.00815831
872.839985	394.746741	8.90888E-06	0.00777603
872.879985	378.313886	8.53802E-06	0.007452665
872.919985	410.523124	9.26494E-06	0.008087547
872.959985	432.967844	9.77148E-06	0.008530112
872.999985	471.603308	1.06434E-05	0.009291714
873.039985	547.323671	1.23523E-05	0.010784081
873.079985	693.946338	1.56614E-05	0.013673657
873.119985	904.547437	2.04144E-05	0.017824199

873.159985	1053.593472	2.37781E-05	0.020762118
873.199985	1144.191359	2.58228E-05	0.022548473
873.239985	1110.133148	2.50542E-05	0.021878293
873.279985	1014.961554	2.29063E-05	0.020003586
873.319985	897.503975	2.02554E-05	0.017689458
873.359985	732.902412	1.65406E-05	0.014445886
873.399985	578.575765	1.30576E-05	0.011404551
873.439985	482.859439	1.08975E-05	0.009518282
873.479985	434.576871	9.80779E-06	0.008566912
873.519985	412.379967	9.30684E-06	0.008129712
873.559985	403.826874	9.11381E-06	0.00796146
873.599985	421.791199	9.51924E-06	0.008316008
873.639985	437.924477	9.88335E-06	0.008634486
873.679985	426.300772	9.62101E-06	0.008405688
873.719985	402.198657	9.07706E-06	0.007930812
873.759985	367.589178	8.29598E-06	0.007248692
873.799985	325.317987	7.34197E-06	0.006415417
873.839985	289.530202	6.53429E-06	0.005709927
873.879985	324.575687	7.32522E-06	0.006401364
873.919985	360.665145	8.13971E-06	0.007113455
873.959985	369.596964	8.34129E-06	0.007289953
873.999985	409.239917	9.23597E-06	0.008072242
874.039985	473.485216	1.06859E-05	0.009339905
874.079985	573.743994	1.29486E-05	0.011318115
874.119985	726.317656	1.6392E-05	0.014328555
874.159985	869.246556	1.96177E-05	0.017148994
874.199985	929.985942	2.09885E-05	0.018348135
874.239985	930.810509	2.10071E-05	0.018365244
874.279985	892.361112	2.01393E-05	0.017607428
874.319985	830.105868	1.87343E-05	0.016379802
874.359985	727.825428	1.6426E-05	0.014362242
874.399985	578.911416	1.30652E-05	0.011424232
874.439985	474.184357	1.07017E-05	0.009357977
874.479985	400.692882	9.04308E-06	0.007907993
874.519985	346.647834	7.82336E-06	0.006841684
874.559985	318.868463	7.19642E-06	0.006293698
874.599985	308.627253	6.96529E-06	0.00609184
874.639985	342.137652	7.72157E-06	0.006753594
874.679985	384.741623	8.68308E-06	0.007594918
874.719985	409.109666	9.23304E-06	0.008076321
874.759985	428.807297	9.67758E-06	0.008465563
874.799985	433.630874	9.78644E-06	0.008561182
874.839985	415.761997	9.38317E-06	0.008208772
874.879985	374.659559	8.45554E-06	0.007397587
874.919985	347.778839	7.84888E-06	0.006867145
874.959985	386.348044	8.71934E-06	0.007629071

874.999985	465.436569	1.05043E-05	0.009191223
875.039985	573.988278	1.29541E-05	0.011335369
875.079985	715.370808	1.61449E-05	0.014128099
875.119985	853.706101	1.9267E-05	0.0168609
875.159985	979.439938	2.21046E-05	0.019345057
875.199985	1100.473817	2.48362E-05	0.021736608
875.239985	1104.839509	2.49347E-05	0.021823837
875.279985	980.024909	2.21178E-05	0.019359265
875.319985	819.809259	1.8502E-05	0.016195129
875.359985	670.34749	1.51288E-05	0.013243154
875.399985	528.568628	1.19291E-05	0.010442697
875.439985	413.073255	9.32249E-06	0.008161279
875.479985	323.636696	7.30403E-06	0.006394532
875.519985	282.084883	6.36626E-06	0.00557379
875.559985	314.05959	7.08789E-06	0.006205871
875.599985	322.643133	7.28161E-06	0.006375774
875.639985	338.758371	7.6453E-06	0.006694534
875.679985	368.663538	8.32022E-06	0.007285852
875.719985	365.190261	8.24184E-06	0.00721754
875.759985	341.094499	7.69803E-06	0.006741625
875.799985	317.485415	7.1652E-06	0.006275285
875.839985	287.209612	6.48192E-06	0.005677125
875.879985	272.726128	6.15505E-06	0.005391084
875.919985	276.886064	6.24893E-06	0.005473565
875.959985	312.087233	7.04337E-06	0.006169714
875.999985	406.564674	9.1756E-06	0.008037824
876.039985	527.431884	1.19034E-05	0.010427857
876.079985	650.178427	1.46736E-05	0.012855266
876.119985	771.234801	1.74057E-05	0.015249477
876.159985	870.636192	1.9649E-05	0.017215708
876.199985	898.594538	2.028E-05	0.017769359
876.239985	868.181336	1.95936E-05	0.017168734
876.279985	825.158907	1.86227E-05	0.016318688
876.319985	734.855377	1.65847E-05	0.014533471
876.359985	596.950988	1.34724E-05	0.01180663
876.399985	510.288637	1.15165E-05	0.010093064
876.439985	478.285937	1.07942E-05	0.00946051
876.479985	460.539147	1.03937E-05	0.009109894
876.519985	420.495854	9.49001E-06	0.00831818
876.559985	384.824869	8.68496E-06	0.00761289
876.599985	372.956272	8.4171E-06	0.007378433
876.639985	390.238269	8.80713E-06	0.007720686
876.679985	428.037847	9.66022E-06	0.00846892
876.719985	447.613322	1.0102E-05	0.008856633
876.759985	441.029148	9.95341E-06	0.008726755
876.799985	423.602233	9.56011E-06	0.008382306

876.839985	406.293394	9.16948E-06	0.008040163
876.879985	378.430215	8.54064E-06	0.007489119
876.919985	363.916905	8.2131E-06	0.007202229
876.959985	404.155637	9.12123E-06	0.007998954
876.999985	508.588766	1.14781E-05	0.010066329
877.039985	635.484519	1.4342E-05	0.012578508
877.079985	791.500097	1.78631E-05	0.015667328
877.119985	967.351184	2.18318E-05	0.019149081
877.159985	1123.065137	2.5346E-05	0.022232511
877.199985	1218.407849	2.74978E-05	0.024121042
877.239985	1226.256826	2.76749E-05	0.024277536
877.279985	1155.452697	2.6077E-05	0.022876793
877.319985	1028.94322	2.32218E-05	0.020372963
877.359985	912.654326	2.05973E-05	0.018071279
877.399985	781.558092	1.76387E-05	0.015476175
877.439985	626.18818	1.41322E-05	0.012400153
877.479985	527.469479	1.19043E-05	0.010445742
877.519985	507.680329	1.14576E-05	0.010054306
877.559985	468.546229	1.05744E-05	0.009279702
877.599985	414.528354	9.35533E-06	0.008210235
877.639985	389.703835	8.79507E-06	0.007718908
877.679985	346.346431	7.81656E-06	0.006860435
877.719985	332.741563	7.50951E-06	0.00659125
877.759985	342.165047	7.72219E-06	0.006778228
877.799985	318.348084	7.18467E-06	0.006306706
877.839985	306.047519	6.90707E-06	0.006063299
877.879985	292.398555	6.59903E-06	0.005793155
877.919985	297.364248	6.7111E-06	0.005891806
877.959985	322.631027	7.28133E-06	0.006392719
877.999985	381.200489	8.60316E-06	0.007553578
878.039985	487.366829	1.09992E-05	0.009657729
878.079985	577.096659	1.30243E-05	0.011436349
878.119985	675.163471	1.52375E-05	0.013380352
878.159985	798.96489	1.80315E-05	0.015834563
878.199985	847.958569	1.91372E-05	0.016806327
878.239985	838.062684	1.89139E-05	0.01661095
878.279985	814.106107	1.83732E-05	0.01613685
878.319985	742.009092	1.67461E-05	0.014708445
878.359985	656.432322	1.48148E-05	0.013012695
878.399985	575.024087	1.29775E-05	0.011399429
878.439985	493.870264	1.1146E-05	0.00979106
878.479985	445.344727	1.00508E-05	0.008829436
878.519985	437.762771	9.8797E-06	0.00867951
878.559985	433.220431	9.77718E-06	0.008589841
878.599985	412.595852	9.31171E-06	0.008181272
878.639985	387.096548	8.73623E-06	0.007676001

878.679985	386.496911	8.7227E-06	0.007664459
878.719985	406.814009	9.18123E-06	0.008067726
878.759985	424.698022	9.58484E-06	0.008422776
878.799985	405.164569	9.144E-06	0.008035747
878.839985	381.18851	8.60289E-06	0.007560567
878.879985	371.506727	8.38439E-06	0.007368872
878.919985	357.642109	8.07148E-06	0.007094189
878.959985	416.10341	9.39087E-06	0.008254203
878.999985	568.963635	1.28407E-05	0.01128699
879.039985	733.292146	1.65494E-05	0.014547569
879.079985	866.406573	1.95536E-05	0.017189169
879.119985	979.455774	2.2105E-05	0.019432906
879.159985	1074.468511	2.42493E-05	0.021318977
879.199985	1144.743472	2.58353E-05	0.022714365
879.239985	1164.54455	2.62821E-05	0.023108316
879.279985	1083.065293	2.44433E-05	0.021492483
879.319985	918.349832	2.07259E-05	0.018224677
879.359985	745.182093	1.68177E-05	0.014788832
879.399985	579.892193	1.30874E-05	0.011509023
879.439985	442.37009	9.98368E-06	0.008780045
879.479985	376.340021	8.49347E-06	0.007469837
879.519985	361.459962	8.15765E-06	0.007174814
879.559985	340.786564	7.69108E-06	0.006764765
879.599985	315.829799	7.12784E-06	0.006269647
879.639985	328.002281	7.40255E-06	0.006511583
879.679985	338.015931	7.62855E-06	0.006710682
879.719985	337.043961	7.60661E-06	0.006691689
879.759985	334.209035	7.54263E-06	0.006635706
879.799985	313.009826	7.0642E-06	0.006215079
879.839985	308.375085	6.9596E-06	0.006123331
879.879985	309.389448	6.98249E-06	0.006143752
879.919985	314.400522	7.09558E-06	0.006243544
879.959985	348.549789	7.86628E-06	0.006922014
879.999985	409.992197	9.25295E-06	0.008142598
880.039985	492.000443	1.11038E-05	0.009771757
880.079985	589.501357	1.33042E-05	0.011708782
880.119985	710.92166	1.60445E-05	0.014121095
880.159985	839.191941	1.89394E-05	0.016669696
880.199985	893.03005	2.01544E-05	0.017739942
880.239985	899.175902	2.02931E-05	0.01786284
880.279985	876.1663	1.97739E-05	0.017406527
880.319985	798.302095	1.80166E-05	0.015860343
880.359985	669.869014	1.5118E-05	0.013309292
880.399985	512.096976	1.15573E-05	0.01017506
880.439985	414.778869	9.36098E-06	0.008241782
880.479985	372.248859	8.40114E-06	0.007397034

880.519985	312.351551	7.04934E-06	0.006207084
880.559985	296.969649	6.70219E-06	0.005901682
880.599985	330.635423	7.46198E-06	0.00657102
880.639985	370.802363	8.36849E-06	0.007369629
880.679985	419.962638	9.47797E-06	0.00834706
880.719985	412.576855	9.31128E-06	0.008200635
880.759985	362.734017	8.1864E-06	0.007210255
880.799985	337.575585	7.61861E-06	0.006710472
880.839985	341.450962	7.70607E-06	0.006787817
880.879985	354.976754	8.01133E-06	0.007057021
880.919985	360.846339	8.1438E-06	0.007174036
880.959985	391.772227	8.84175E-06	0.007789231
880.999985	473.223999	1.068E-05	0.009409086
881.039985	612.531405	1.3824E-05	0.01217948
881.079985	765.153738	1.72685E-05	0.015214889
881.119985	936.3138	2.11313E-05	0.018619209
881.159985	1068.248616	2.41089E-05	0.021243783
881.199985	1183.033805	2.66994E-05	0.023527533
881.239985	1196.932964	2.70131E-05	0.023805032
881.279985	1059.048217	2.39012E-05	0.021063687
881.319985	885.860918	1.99926E-05	0.017619919
881.359985	715.818968	1.6155E-05	0.014238403
881.399985	534.436328	1.20615E-05	0.010630991
881.439985	402.455309	9.08286E-06	0.008005992
881.479985	343.735164	7.75762E-06	0.00683819
881.519985	296.23034	6.68551E-06	0.005893407
881.559985	285.604478	6.4457E-06	0.005682267
881.599985	320.132574	7.22495E-06	0.006369513
881.639985	330.789093	7.46545E-06	0.006581838
881.679985	348.799506	7.87192E-06	0.006940513
881.719985	357.364632	8.06522E-06	0.007111267
881.759985	339.059627	7.6521E-06	0.006747319
881.799985	311.122911	7.02161E-06	0.006191656
881.839985	271.58309	6.12925E-06	0.00540502
881.879985	271.297084	6.1228E-06	0.005399572
881.919985	270.95374	6.11505E-06	0.005392983
881.959985	297.690047	6.71845E-06	0.005925404
881.999985	363.914663	8.21305E-06	0.007243907
882.039985	458.208326	1.03411E-05	0.009121285
882.079985	609.327542	1.37517E-05	0.012130076
882.119985	738.140292	1.66588E-05	0.014695059
882.159985	802.718528	1.81162E-05	0.015981421
882.199985	850.459828	1.91937E-05	0.016932676
882.239985	908.235535	2.04976E-05	0.018083812
882.279985	855.525317	1.9308E-05	0.017035075
882.319985	743.929808	1.67895E-05	0.014813676

882.359985	616.439407	1.39122E-05	0.01227555
882.399985	522.231254	1.1786E-05	0.010399994
882.439985	427.38006	9.64537E-06	0.008511463
882.479985	354.282826	7.99567E-06	0.007056019
882.519985	309.207287	6.97838E-06	0.006158558
882.559985	316.162446	7.13535E-06	0.006297371
882.599985	342.896267	7.73869E-06	0.006830169
882.639985	366.899864	8.28042E-06	0.007308629
882.679985	395.601624	8.92818E-06	0.007880724
882.719985	401.796334	9.06798E-06	0.00800449
882.759985	393.816542	8.88789E-06	0.007845874
882.799985	389.628518	8.79337E-06	0.00776279
882.839985	381.167365	8.60242E-06	0.007594557
882.879985	367.984102	8.30489E-06	0.00733222
882.919985	387.671769	8.74921E-06	0.007724854
882.959985	429.408768	9.69116E-06	0.008556905
882.999985	503.681072	1.13674E-05	0.010037397
883.039985	602.246881	1.35919E-05	0.012002168
883.079985	704.702218	1.59041E-05	0.014044634
883.119985	839.262022	1.8941E-05	0.016727153
883.159985	968.166132	2.18502E-05	0.019297188
883.199985	1051.491635	2.37307E-05	0.020958955
883.239985	1065.697361	2.40513E-05	0.021243074
883.279985	1040.387024	2.34801E-05	0.02073949
883.319985	914.699574	2.06435E-05	0.018234812
883.359985	759.240831	1.7135E-05	0.01513638
883.399985	602.507076	1.35977E-05	0.012012248
883.439985	474.298221	1.07043E-05	0.009456563
883.479985	381.644318	8.61318E-06	0.007609573
883.519985	323.566816	7.30245E-06	0.006451863
883.559985	321.258155	7.25035E-06	0.006406118
883.599985	342.788935	7.73627E-06	0.006835767
883.639985	366.272508	8.26626E-06	0.007304398
883.679985	366.953415	8.28163E-06	0.007318308
883.719985	371.898429	8.39323E-06	0.007417265
883.759985	367.297318	8.28939E-06	0.00732583
883.799985	325.750711	7.35174E-06	0.006497468
883.839985	286.503404	6.46598E-06	0.005714894
883.879985	260.070118	5.86942E-06	0.005187863
883.919985	288.547263	6.51211E-06	0.005756184
883.959985	343.265408	7.74702E-06	0.006848058
883.999985	389.133722	8.78221E-06	0.00776347
884.039985	453.380658	1.02322E-05	0.009045647
884.079985	537.145919	1.21226E-05	0.010717379
884.119985	695.71759	1.57014E-05	0.013881901
884.159985	820.885358	1.85262E-05	0.016380159

884.199985	879.142593	1.9841E-05	0.017543433
884.239985	896.929006	2.02424E-05	0.017899173
884.279985	810.060259	1.82819E-05	0.016166346
884.319985	657.155062	1.48311E-05	0.013115415
884.359985	543.106051	1.22571E-05	0.01083973
884.399985	460.706382	1.03975E-05	0.00919555
884.439985	412.536354	9.31037E-06	0.008234464
884.479985	389.684761	8.79464E-06	0.007778685
884.519985	339.739479	7.66745E-06	0.00678201
884.559985	307.336344	6.93615E-06	0.006135444
884.599985	318.462275	7.18725E-06	0.006357841
884.639985	318.175468	7.18078E-06	0.006352403
884.679985	302.951184	6.83719E-06	0.006048722
884.719985	329.895259	7.44528E-06	0.006586985
884.759985	372.446072	8.40559E-06	0.007436929
884.799985	371.83818	8.39187E-06	0.007425126
884.839985	362.232104	8.17507E-06	0.007233632
884.879985	379.308246	8.56046E-06	0.007574979
884.919985	386.529925	8.72344E-06	0.007719548
884.959985	406.207907	9.16755E-06	0.008112912
884.999985	507.636037	1.14566E-05	0.010139125
885.039985	644.703194	1.45501E-05	0.012877379
885.079985	776.522328	1.7525E-05	0.015511051
885.119985	928.619418	2.09576E-05	0.018550032
885.159985	1056.43549	2.38423E-05	0.02110423
885.199985	1141.429036	2.57605E-05	0.022803162
885.239985	1142.258752	2.57792E-05	0.022820769
885.279985	1027.600415	2.31915E-05	0.02053098
885.319985	887.261619	2.00243E-05	0.017727877
885.359985	750.661065	1.69414E-05	0.014999215
885.399985	589.113482	1.32955E-05	0.01177181
885.439985	451.714733	1.01946E-05	0.009026682
885.479985	378.246285	8.53649E-06	0.007558893
885.519985	322.115273	7.26969E-06	0.006437458
885.559985	302.704907	6.83163E-06	0.006049816
885.599985	302.563526	6.82844E-06	0.006047264
885.639985	342.540934	7.73067E-06	0.006846592
885.679985	388.513389	8.76821E-06	0.007765825
885.719985	404.498445	9.12897E-06	0.008085708
885.759985	383.376795	8.65228E-06	0.007663844
885.799985	327.516086	7.39158E-06	0.006547463
885.839985	289.761206	6.53951E-06	0.005792957
885.879985	279.602508	6.31024E-06	0.005590115
885.919985	302.600599	6.82927E-06	0.00605019
885.959985	327.780528	7.39755E-06	0.006553933
885.999985	364.361741	8.22314E-06	0.007285699

886.039985	428.929038	9.68033E-06	0.00857716
886.079985	541.381416	1.22182E-05	0.010826324
886.119985	688.153905	1.55307E-05	0.013762042
886.159985	781.519782	1.76378E-05	0.015629924
886.199985	816.718266	1.84322E-05	0.01633461
886.239985	835.594861	1.88582E-05	0.016712902
886.279985	833.733992	1.88162E-05	0.016676435
886.319985	747.065384	1.68602E-05	0.014943554
886.359985	645.371744	1.45651E-05	0.012909958
886.399985	553.902041	1.25008E-05	0.011080707
886.439985	436.19072	9.84422E-06	0.008726308
886.479985	374.61122	8.45445E-06	0.007494704
886.519985	338.845572	7.64727E-06	0.00677946
886.559985	323.801474	7.30775E-06	0.006478757
886.599985	321.5508	7.25695E-06	0.006434015
886.639985	318.548968	7.18921E-06	0.006374238
886.679985	331.256686	7.476E-06	0.006628821
886.719985	356.316265	8.04156E-06	0.007130614
886.759985	360.802775	8.14282E-06	0.007220723
886.799985	341.364875	7.70413E-06	0.006832022
886.839985	352.23205	7.94939E-06	0.007049834
886.879985	348.64407	7.86841E-06	0.006978336
886.919985	337.379375	7.61418E-06	0.006753171
886.959985	398.539341	8.99448E-06	0.007977742
886.999985	495.769013	1.11888E-05	0.00992448
887.039985	635.569702	1.43439E-05	0.012723633
887.079985	780.347608	1.76114E-05	0.015622684
887.119985	915.311455	2.06573E-05	0.018325508
887.159985	1003.211503	2.26411E-05	0.020086266
887.199985	1108.371213	2.50144E-05	0.02219277
887.239985	1140.647924	2.57428E-05	0.022840072
887.279985	999.75827	2.25632E-05	0.020019833
887.319985	825.183591	1.86232E-05	0.016524777
887.359985	654.35834	1.4768E-05	0.013104493
887.399985	533.476055	1.20398E-05	0.010684129
887.439985	449.210402	1.01381E-05	0.008996914
887.479985	414.423976	9.35297E-06	0.008300575
887.519985	394.399446	8.90105E-06	0.007899856
887.559985	358.050835	8.08071E-06	0.007172114
887.599985	349.441295	7.8864E-06	0.006999971
887.639985	337.667878	7.62069E-06	0.006764432
887.679985	320.373674	7.23039E-06	0.00641827
887.719985	341.779833	7.71349E-06	0.006847423
887.759985	354.312095	7.99633E-06	0.007098822
887.799985	316.168053	7.13547E-06	0.006334872
887.839985	272.503175	6.15002E-06	0.005460231

887.879985	253.494884	5.72103E-06	0.005079585
887.919985	272.430445	6.14838E-06	0.005459266
887.959985	321.803216	7.26265E-06	0.006448943
887.999985	372.069896	8.3971E-06	0.007456624
888.039985	452.874138	1.02207E-05	0.009076424
888.079985	556.309629	1.25551E-05	0.011149963
888.119985	661.901584	1.49382E-05	0.013266911
888.159985	754.965928	1.70385E-05	0.01513294
888.199985	812.880447	1.83456E-05	0.016294543
888.239985	842.470171	1.90134E-05	0.016888443
888.279985	784.590664	1.77071E-05	0.015728879
888.319985	721.065011	1.62734E-05	0.014456016
888.359985	653.561685	1.475E-05	0.013103289
888.399985	566.822415	1.27924E-05	0.011364761
888.439985	470.936413	1.06284E-05	0.009442677
888.479985	389.928088	8.80013E-06	0.007818743
888.519985	344.331496	7.77108E-06	0.006904762
888.559985	345.207959	7.79086E-06	0.006922649
888.599985	357.791909	8.07487E-06	0.007175325
888.639985	371.629475	8.38716E-06	0.007453165
888.679985	395.27952	8.92091E-06	0.007927833
888.719985	418.724538	9.45003E-06	0.00839843
888.759985	390.861152	8.82119E-06	0.007839922
888.799985	345.972491	7.80812E-06	0.006939854
888.839985	340.621979	7.68736E-06	0.006832836
888.879985	340.948519	7.69473E-06	0.006839694
888.919985	350.279555	7.90532E-06	0.007027198
888.959985	416.777811	9.40609E-06	0.008361642
888.999985	488.29513	1.10201E-05	0.009796905
889.039985	592.183227	1.33648E-05	0.011881798
889.079985	729.632028	1.64668E-05	0.014640283
889.119985	889.613466	2.00773E-05	0.017851161
889.159985	970.462472	2.1902E-05	0.01947437
889.199985	1016.438637	2.29396E-05	0.020397896
889.239985	1054.971332	2.38092E-05	0.021172122
889.279985	995.904278	2.24762E-05	0.01998761
889.319985	875.722717	1.97638E-05	0.01757638
889.359985	716.370371	1.61675E-05	0.014378711
889.399985	557.366887	1.2579E-05	0.011187757
889.439985	464.256177	1.04776E-05	0.00931921
889.479985	403.675705	9.1104E-06	0.008103517
889.519985	353.970003	7.98861E-06	0.007106028
889.559985	323.067406	7.29118E-06	0.006485943
889.599985	319.730348	7.21587E-06	0.006419237
889.639985	322.318895	7.27429E-06	0.006471498
889.679985	342.333962	7.726E-06	0.006873668

889.719985	339.667455	7.66582E-06	0.006820434
889.759985	319.747093	7.21625E-06	0.006420727
889.799985	289.482022	6.53321E-06	0.005813247
889.839985	269.069891	6.07253E-06	0.005403582
889.879985	267.240735	6.03125E-06	0.00536709
889.919985	296.632883	6.69459E-06	0.00595765
889.959985	341.132516	7.69889E-06	0.0068517
889.999985	361.866546	8.16682E-06	0.007268473
890.039985	417.856069	9.43043E-06	0.008393459
890.079985	509.923727	1.15083E-05	0.010243279
890.119985	651.144763	1.46954E-05	0.013080696
890.159985	761.99084	1.71971E-05	0.015308145
890.199985	791.902769	1.78721E-05	0.015909781
890.239985	794.588413	1.79328E-05	0.015964454
890.279985	725.641917	1.63767E-05	0.014579873
890.319985	611.75084	1.38064E-05	0.012292082
890.359985	528.770368	1.19336E-05	0.010625209
890.399985	446.941589	1.00868E-05	0.00898133
890.439985	383.394852	8.65269E-06	0.007704699
890.479985	338.900476	7.64851E-06	0.006810847
890.519985	339.544448	7.66305E-06	0.006824095
890.559985	361.013515	8.14757E-06	0.007255902
890.599985	373.244314	8.4236E-06	0.007502062
890.639985	382.548161	8.63358E-06	0.007689411
890.679985	390.636569	8.81612E-06	0.007852345
890.719985	394.555222	8.90456E-06	0.007931471
890.759985	391.432741	8.83409E-06	0.007869055
890.799985	375.154689	8.46672E-06	0.007542153
890.839985	361.261364	8.15317E-06	0.007263166
890.879985	362.411004	8.17911E-06	0.007286607
890.919985	370.061178	8.35177E-06	0.007440755
890.959985	404.426361	9.12734E-06	0.008132094
890.999985	465.126472	1.04973E-05	0.009353055
891.039985	569.258392	1.28474E-05	0.011447519
891.079985	693.438531	1.56499E-05	0.01394535
891.119985	805.382517	1.81764E-05	0.016197319
891.159985	966.578996	2.18143E-05	0.019440068
891.199985	1107.289786	2.499E-05	0.022271077
891.239985	1118.729212	2.52482E-05	0.02250217
891.279985	1022.458925	2.30755E-05	0.020566708
891.319985	885.48203	1.99841E-05	0.017812224
891.359985	733.039492	1.65437E-05	0.014746375
891.399985	590.492088	1.33266E-05	0.011879317
891.439985	471.500775	1.06411E-05	0.009485916
891.479985	380.890869	8.59618E-06	0.007663319
891.519985	332.351425	7.50071E-06	0.006687032

891.559985	333.863869	7.53484E-06	0.006717764
891.599985	349.399296	7.88546E-06	0.007030672
891.639985	367.700228	8.29848E-06	0.007399258
891.679985	368.024156	8.30579E-06	0.007406109
891.719985	371.96931	8.39483E-06	0.007485837
891.759985	380.871111	8.59573E-06	0.007665328
891.799985	367.816944	8.30112E-06	0.007402935
891.839985	343.666636	7.75608E-06	0.00691718
891.879985	307.599716	6.9421E-06	0.006191517
891.919985	280.533326	6.33125E-06	0.005646965
891.959985	329.165808	7.42881E-06	0.006626205
891.999985	418.450622	9.44385E-06	0.008423912
892.039985	486.171081	1.09722E-05	0.009787645
892.079985	561.089192	1.2663E-05	0.01129641
892.119985	676.349587	1.52643E-05	0.013617559
892.159985	797.717888	1.80034E-05	0.016061897
892.199985	914.927141	2.06486E-05	0.018422708
892.239985	961.387218	2.16972E-05	0.019359083
892.279985	870.29668	1.96414E-05	0.017525614
892.319985	753.118115	1.69968E-05	0.015166608
892.359985	642.447817	1.44992E-05	0.012938463
892.399985	569.079452	1.28433E-05	0.011461388
892.439985	501.570692	1.13198E-05	0.010102199
892.479985	418.732609	9.45021E-06	0.008434125
892.519985	357.956879	8.07859E-06	0.007210301
892.559985	331.69131	7.48581E-06	0.006681535
892.599985	358.896215	8.09979E-06	0.00722987
892.639985	414.840357	9.36237E-06	0.008357225
892.679985	459.238375	1.03644E-05	0.009252066
892.719985	434.556881	9.80734E-06	0.008755212
892.759985	404.238478	9.1231E-06	0.008144738
892.799985	398.051004	8.98346E-06	0.00802043
892.839985	375.945853	8.48457E-06	0.007575367
892.879985	354.505574	8.0007E-06	0.007143662
892.919985	376.478768	8.4966E-06	0.007586785
892.959985	451.108321	1.01809E-05	0.009091124
892.999985	570.838495	1.2883E-05	0.011504545
893.039985	681.939961	1.53904E-05	0.013744274
893.079985	806.571743	1.82032E-05	0.016256914
893.119985	927.757888	2.09382E-05	0.018700328
893.159985	1065.511065	2.40471E-05	0.021477908
893.199985	1144.632893	2.58328E-05	0.02307383
893.239985	1098.930969	2.48013E-05	0.02215355
893.279985	1019.67218	2.30126E-05	0.020556678
893.319985	954.059937	2.15318E-05	0.019234791
893.359985	853.613012	1.92649E-05	0.017210452

893.399985	777.919453	1.75566E-05	0.015685029
893.439985	733.965715	1.65646E-05	0.014799462
893.479984	689.005827	1.55499E-05	0.013893526
893.519984	611.771526	1.38068E-05	0.01233668
893.559984	523.301769	1.18102E-05	0.010553115
893.599984	468.495203	1.05733E-05	0.009448287
893.639984	449.164455	1.0137E-05	0.009058843
893.679984	406.886171	9.18285E-06	0.008206533
893.719984	372.376686	8.40402E-06	0.007510843
893.759984	348.596929	7.86735E-06	0.00703152
893.799984	325.943834	7.3561E-06	0.006574881
893.839984	314.687677	7.10206E-06	0.006348107
893.879984	296.850248	6.6995E-06	0.005988546
893.919984	296.934441	6.7014E-06	0.005990512
893.959984	336.487311	7.59405E-06	0.006788777
893.999984	383.700209	8.65958E-06	0.007741664
894.039984	469.611788	1.05985E-05	0.009475468
894.079984	601.084392	1.35656E-05	0.012128765
894.119984	733.642392	1.65573E-05	0.014804201
894.159984	848.017549	1.91386E-05	0.017112948
894.199984	920.917892	2.07838E-05	0.018584904
894.239984	932.135087	2.1037E-05	0.018812118
894.279984	902.124348	2.03597E-05	0.018207263
894.319984	798.522401	1.80215E-05	0.016117022
894.359984	667.293125	1.50599E-05	0.013468951
894.399984	591.076965	1.33398E-05	0.011931102
894.439984	516.445096	1.16554E-05	0.010425097
894.479984	476.226844	1.07478E-05	0.009613671
894.519984	460.509811	1.03931E-05	0.009296804
894.559984	409.350587	9.23847E-06	0.008264368
894.599984	360.844474	8.14376E-06	0.007285405
894.639984	340.627422	7.68749E-06	0.006877533
894.679984	357.417997	8.06643E-06	0.00721687
894.719984	367.0801	8.28449E-06	0.007412296
894.759984	345.523449	7.79798E-06	0.006977323
894.799984	325.366608	7.34307E-06	0.00657058
894.839984	311.843995	7.03788E-06	0.006297781
894.879984	307.274116	6.93475E-06	0.006205768
894.919984	314.335164	7.09411E-06	0.006348658
894.959984	368.48453	8.31618E-06	0.007442651
894.999984	467.797645	1.05575E-05	0.009448999
895.039984	557.486428	1.25817E-05	0.011261118
895.079984	725.539591	1.63744E-05	0.014656414
895.119984	934.653512	2.10938E-05	0.018881507
895.159984	1040.963753	2.34931E-05	0.021030085
895.199984	1125.518273	2.54014E-05	0.022739315

895.239984	1096.220052	2.47402E-05	0.02214838
895.279984	965.362705	2.17869E-05	0.019505368
895.319984	837.239816	1.88953E-05	0.016917372
895.359984	715.705252	1.61525E-05	0.014462276
895.399984	584.11339	1.31826E-05	0.011803723
895.439984	469.438401	1.05946E-05	0.009486802
895.479984	416.073738	9.3902E-06	0.00840874
895.519984	395.131063	8.91756E-06	0.007985851
895.559984	383.084769	8.64569E-06	0.007742734
895.599984	346.167103	7.81251E-06	0.006996883
895.639984	314.879397	7.10639E-06	0.006364766
895.679984	317.528329	7.16617E-06	0.006418597
895.719984	341.497374	7.70712E-06	0.006903421
895.759984	362.774234	8.18731E-06	0.007333864
895.799984	345.414524	7.79552E-06	0.006983231
895.839984	307.621218	6.94258E-06	0.006219443
895.879984	289.454941	6.53259E-06	0.005852421
895.919984	286.496401	6.46582E-06	0.005792862
895.959984	315.594657	7.12253E-06	0.006381503
895.999984	383.57848	8.65683E-06	0.007756521
896.039984	448.914025	1.01314E-05	0.009078107
896.079984	535.525937	1.20861E-05	0.010830089
896.119984	615.857453	1.3899E-05	0.012455212
896.159984	735.235914	1.65933E-05	0.014870207
896.199984	824.627219	1.86107E-05	0.016678898
896.239984	840.238157	1.8963E-05	0.016995403
896.279984	804.904659	1.81656E-05	0.016281443
896.319984	728.218379	1.64349E-05	0.014730906
896.359984	612.39062	1.38208E-05	0.012388415
896.399984	495.452837	1.11817E-05	0.010023258
896.439984	415.392923	9.37484E-06	0.008403981
896.479984	370.410972	8.35966E-06	0.007494268
896.519984	316.630605	7.14591E-06	0.006406453
896.559984	316.008784	7.13188E-06	0.006394157
896.599984	345.290719	7.79273E-06	0.006986962
896.639984	332.223197	7.49781E-06	0.006722841
896.679984	339.978168	7.67283E-06	0.006880076
896.719984	358.024101	8.08011E-06	0.007245592
896.759984	356.715848	8.05058E-06	0.007219438
896.799984	362.45321	8.18006E-06	0.007335881
896.839984	379.001771	8.55354E-06	0.007671159
896.879984	406.821754	9.1814E-06	0.008234614
896.919984	439.351844	9.91556E-06	0.008893463
896.959984	472.283469	1.06588E-05	0.009560499
896.999984	530.45792	1.19717E-05	0.010738611
897.039984	665.489576	1.50192E-05	0.013472799

897.079984	826.133532	1.86447E-05	0.016725772
897.119984	973.968262	2.19811E-05	0.01971969
897.159984	1060.926139	2.39436E-05	0.021481262
897.199984	1091.991041	2.46447E-05	0.022111239
897.239984	1087.193122	2.45364E-05	0.02201507
897.279984	998.584957	2.25367E-05	0.020221704
897.319984	844.760935	1.90651E-05	0.017107475
897.359984	665.871397	1.50278E-05	0.013485337
897.399984	521.82636	1.17769E-05	0.010568585
897.439984	425.964298	9.61342E-06	0.008627468
897.479984	358.528082	8.09148E-06	0.007261941
897.519984	313.975086	7.08598E-06	0.006359809
897.559984	292.412386	6.59934E-06	0.005923304
897.599984	314.430268	7.09625E-06	0.006369597
897.639984	355.757643	8.02895E-06	0.007207111
897.679984	385.195937	8.69334E-06	0.007803834
897.719984	385.140083	8.69208E-06	0.00780305
897.759984	360.683815	8.14013E-06	0.007307884
897.799984	334.024503	7.53847E-06	0.006768036
897.839984	312.922557	7.06223E-06	0.006340749
897.879984	327.300967	7.38673E-06	0.006632394
897.919984	350.845755	7.9181E-06	0.00710982
897.959984	358.574501	8.09253E-06	0.007266765
897.999984	393.725876	8.88584E-06	0.007979488
898.039984	491.62719	1.10953E-05	0.00996406
898.079984	621.19145	1.40194E-05	0.012590566
898.119984	733.754304	1.65598E-05	0.014872699
898.159984	808.163229	1.82391E-05	0.016381646
898.199984	815.045125	1.83944E-05	0.01652188
898.239984	808.905722	1.82559E-05	0.016398157
898.279984	784.14108	1.7697E-05	0.015896836
898.319984	718.673788	1.62195E-05	0.014570271
898.359984	629.050406	1.41968E-05	0.012753829
898.399984	544.732825	1.22939E-05	0.011044805
898.439984	451.623197	1.01925E-05	0.009157355
898.479984	392.36709	8.85518E-06	0.007956201
898.519984	380.564964	8.58882E-06	0.007717227
898.559984	380.484923	8.58701E-06	0.007715948
898.599984	377.695732	8.52407E-06	0.007659726
898.639984	377.314759	8.51547E-06	0.007652341
898.679984	401.520217	9.06175E-06	0.008143615
898.719984	396.170191	8.94101E-06	0.008035464
898.759984	385.581398	8.70204E-06	0.007821041
898.799984	385.918435	8.70964E-06	0.007828226
898.839984	371.738487	8.38962E-06	0.007540926
898.879984	377.971646	8.53029E-06	0.00766771

898.919984	399.417599	9.0143E-06	0.008103133
898.959984	443.570271	1.00108E-05	0.008999276
898.999984	481.272249	1.08616E-05	0.009764618
899.039984	543.909232	1.22753E-05	0.011035962
899.079984	686.855628	1.55014E-05	0.013936976
899.119984	838.391078	1.89213E-05	0.017012536
899.159984	962.709166	2.1727E-05	0.019536053
899.199984	1039.871869	2.34685E-05	0.021102839
899.239984	1059.850156	2.39193E-05	0.021509229
899.279984	973.823124	2.19778E-05	0.019764224
899.319984	821.796802	1.85468E-05	0.016679516
899.359984	676.356303	1.52644E-05	0.013728208
899.399984	534.883027	1.20716E-05	0.010857165
899.439984	418.581349	9.4468E-06	0.008496828
899.479984	344.579362	7.77668E-06	0.006994965
899.519984	316.780751	7.1493E-06	0.006430939
899.559984	304.649647	6.87552E-06	0.006184941
899.599984	328.062827	7.40392E-06	0.006660567
899.639984	363.5711	8.20529E-06	0.00738181
899.679984	386.173466	8.7154E-06	0.007841068
899.719984	386.865717	8.73102E-06	0.007855474
899.759984	337.881873	7.62552E-06	0.006861141
899.799984	278.631037	6.28831E-06	0.005658225
899.839984	268.668244	6.06347E-06	0.005456151
899.879984	274.272434	6.18995E-06	0.005570209
899.919984	267.57293	6.03875E-06	0.00543439
899.959984	313.786363	7.08172E-06	0.006373266
899.999984	396.700724	8.95298E-06	0.008057684
900.039984	482.547559	1.08904E-05	0.009801819
900.079984	582.60564	1.31486E-05	0.01183479
900.119984	638.923729	1.44196E-05	0.012979387
900.159984	680.137349	1.53498E-05	0.013817233
900.199984	718.733037	1.62208E-05	0.014601967
900.239984	755.160133	1.70429E-05	0.015342711
900.279984	719.032702	1.62276E-05	0.014609353
900.319984	661.197454	1.49223E-05	0.01343485
900.359984	582.399695	1.31439E-05	0.011834287
900.399984	492.466295	1.11143E-05	0.010007296
900.439984	421.610054	9.51515E-06	0.008567823
900.479984	397.811261	8.97805E-06	0.008084551
900.519984	408.916964	9.22869E-06	0.008310616
900.559984	400.787145	9.04521E-06	0.008145752
900.599984	410.958528	9.27476E-06	0.00835285
900.639984	418.669708	9.44879E-06	0.00850996
900.679984	386.927416	8.73241E-06	0.007865109
900.719984	343.319604	7.74825E-06	0.006978999

900.759984	329.235896	7.4304E-06	0.006693003
900.799984	315.932521	7.13016E-06	0.006422845
900.839984	291.261846	6.57337E-06	0.005921558
900.879984	297.670371	6.71801E-06	0.006052117
900.919984	354.231017	7.9945E-06	0.007202405
900.959984	420.521778	9.49059E-06	0.008550643
900.999984	525.047371	1.18496E-05	0.010676478
901.039984	638.403542	1.44079E-05	0.012982075
901.079984	749.208352	1.69086E-05	0.015235991
901.119984	863.995944	1.94992E-05	0.017571105
901.159984	1003.533482	2.26484E-05	0.020409789
901.199984	1094.784421	2.47078E-05	0.022266632
901.239984	1050.334104	2.37046E-05	0.021363513
901.279984	930.521413	2.10006E-05	0.018927395
901.319984	812.302288	1.83325E-05	0.016523477
901.359984	697.454534	1.57406E-05	0.014187927
901.399984	573.939478	1.2953E-05	0.011675847
901.439984	450.78314	1.01735E-05	0.009170843
901.479984	372.116091	8.39814E-06	0.007570757
901.519984	361.85369	8.16653E-06	0.007362293
901.559984	374.400146	8.44969E-06	0.007617902
901.599984	385.330787	8.69638E-06	0.007840655
901.639984	412.686905	9.31377E-06	0.008397666
901.679984	426.421043	9.62373E-06	0.008677524
901.719984	421.730722	9.51787E-06	0.008582458
901.759984	410.745707	9.26996E-06	0.008359278
901.799984	366.807931	8.27834E-06	0.007465411
901.839984	332.652184	7.5075E-06	0.00677056
901.879984	315.158259	7.11268E-06	0.006414786
901.919984	314.962446	7.10826E-06	0.006411085
901.959984	338.558099	7.64078E-06	0.006891682
901.999984	375.10802	8.46567E-06	0.00763603
902.039984	420.868308	9.49841E-06	0.008567947
902.079984	534.215578	1.20565E-05	0.010875929
902.119984	694.642604	1.56771E-05	0.01414264
902.159984	818.643312	1.84756E-05	0.016667982
902.199984	907.498436	2.0481E-05	0.018477936
902.239984	922.106015	2.08106E-05	0.018776199
902.279984	815.983205	1.84156E-05	0.016616031
902.319984	708.931271	1.59996E-05	0.014436751
902.359984	624.14553	1.40861E-05	0.012710729
902.399984	523.94762	1.18248E-05	0.01067067
902.439984	452.669095	1.02161E-05	0.009219427
902.479984	392.861816	8.86634E-06	0.008001698
902.519984	374.154247	8.44414E-06	0.007621005
902.559984	355.591087	8.0252E-06	0.00724322

902.599984	343.245305	7.74657E-06	0.006992053
902.639984	369.066429	8.32932E-06	0.007518373
902.679984	394.283569	8.89843E-06	0.008032436
902.719984	393.32792	8.87686E-06	0.008013322
902.759984	403.038241	9.09601E-06	0.008211515
902.799984	411.12113	9.27843E-06	0.008376568
902.839984	381.687508	8.61416E-06	0.007777204
902.879984	335.314707	7.56759E-06	0.006832622
902.919984	337.412874	7.61494E-06	0.00687568
902.959984	363.348726	8.20027E-06	0.00740452
902.999984	434.301451	9.80158E-06	0.008850826
903.039984	511.319391	1.15398E-05	0.010420871
903.079984	601.100744	1.3566E-05	0.012251189
903.119984	772.84843	1.74421E-05	0.01575232
903.159984	983.13217	2.21879E-05	0.020039245
903.199984	1098.921716	2.48011E-05	0.022400382
903.239984	1118.429313	2.52414E-05	0.022799034
903.279984	1036.2121	2.33859E-05	0.021123983
903.319984	904.290385	2.04086E-05	0.018435473
903.359984	784.689238	1.77093E-05	0.015997912
903.399984	657.588518	1.48409E-05	0.01340723
903.439984	516.72361	1.16617E-05	0.010535675
903.479984	435.012738	9.81763E-06	0.008870034
903.519984	403.942313	9.11642E-06	0.008236863
903.559984	387.640945	8.74852E-06	0.007904809
903.599984	366.600136	8.27365E-06	0.007476074
903.639984	329.555499	7.43761E-06	0.00672092
903.679984	323.242068	7.29512E-06	0.006592457
903.719984	316.808272	7.14992E-06	0.006461527
903.759984	314.312818	7.0936E-06	0.006410914
903.799984	318.886689	7.19683E-06	0.006504493
903.839984	304.06886	6.86241E-06	0.006202521
903.879984	290.651848	6.55961E-06	0.005929098
903.919984	307.142725	6.93178E-06	0.006265778
903.959984	342.956298	7.74005E-06	0.006996692
903.999984	390.281434	8.80811E-06	0.00796253
904.039984	426.132866	9.61723E-06	0.008694356
904.079984	506.44277	1.14297E-05	0.01033337
904.119984	626.545337	1.41403E-05	0.012784488
904.159984	709.146697	1.60045E-05	0.014470586
904.199984	791.869449	1.78714E-05	0.016159311
904.239984	840.514951	1.89693E-05	0.017152756
904.279984	781.347761	1.76339E-05	0.015946011
904.319984	656.159015	1.48086E-05	0.013391708
904.359984	538.056684	1.21432E-05	0.010981815
904.399984	458.860244	1.03558E-05	0.009365818

904.439984	409.160363	9.23418E-06	0.008351761
904.479984	340.503694	7.68469E-06	0.006950652
904.519984	295.922618	6.67856E-06	0.006040892
904.559984	294.351769	6.64311E-06	0.006009091
904.599984	296.182461	6.68443E-06	0.006046731
904.639984	317.020831	7.15472E-06	0.006472445
904.679984	360.659504	8.13958E-06	0.007363717
904.719984	409.388273	9.23932E-06	0.008359
904.759984	418.521484	9.44545E-06	0.008545862
904.799984	388.473168	8.7673E-06	0.007932651
904.839984	370.176947	8.35438E-06	0.007559375
904.879984	374.030551	8.44135E-06	0.007638407
904.919984	390.288886	8.80828E-06	0.007970785
904.959984	413.654408	9.3356E-06	0.008448348
904.999984	447.371309	1.00965E-05	0.009137375
905.039984	551.318343	1.24425E-05	0.011260948
905.079984	727.387861	1.64161E-05	0.014857911
905.119984	914.469906	2.06383E-05	0.018680149
905.159984	1015.545691	2.29195E-05	0.020745771
905.199984	1030.920963	2.32665E-05	0.02106079
905.239984	1004.64763	2.26735E-05	0.020524957
905.279984	935.32388	2.1109E-05	0.019109516
905.319984	828.374478	1.86953E-05	0.016925191
905.359984	698.847357	1.5772E-05	0.014279348
905.399984	566.91036	1.27944E-05	0.011584029
905.439984	463.499612	1.04605E-05	0.009471392
905.479984	389.445297	8.78924E-06	0.007958479
905.519984	335.065011	7.56195E-06	0.006847497
905.559984	311.920713	7.03962E-06	0.006374795
905.599984	305.684429	6.89887E-06	0.006247618
905.639984	324.191737	7.31656E-06	0.006626166
905.679984	317.046725	7.1553E-06	0.006480415
905.719984	302.116759	6.81835E-06	0.00617552
905.759984	291.272851	6.57362E-06	0.005954124
905.799984	269.202018	6.07551E-06	0.005503201
905.839984	262.66655	5.92802E-06	0.005369836
905.879984	256.314684	5.78467E-06	0.005240213
905.919984	265.173301	5.98459E-06	0.005421562
905.959984	302.98245	6.83789E-06	0.006194856
905.999984	363.927417	8.21333E-06	0.007441281
906.039984	425.919593	9.61241E-06	0.00870923
906.079984	553.450119	1.24906E-05	0.011317481
906.119984	717.796374	1.61997E-05	0.01467884
906.159984	799.779222	1.80499E-05	0.0163561
906.199984	819.455843	1.8494E-05	0.016759242
906.239984	783.09483	1.76734E-05	0.016016305

906.279984	737.216178	1.66379E-05	0.015078634
906.319984	660.23506	1.49006E-05	0.013504699
906.359984	552.394847	1.24668E-05	0.011299393
906.399984	486.496674	1.09796E-05	0.009951866
906.439984	415.405896	9.37513E-06	0.008497995
906.479984	371.507466	8.38441E-06	0.007600296
906.519984	344.02826	7.76424E-06	0.007038437
906.559984	324.107733	7.31466E-06	0.006631178
906.599984	306.815884	6.92441E-06	0.006277667
906.639984	309.274868	6.9799E-06	0.006328259
906.679984	326.496762	7.36858E-06	0.006680941
906.719984	333.924087	7.5362E-06	0.006833225
906.759984	325.388194	7.34356E-06	0.006658845
906.799984	327.377582	7.38846E-06	0.006699852
906.839984	336.646932	7.59765E-06	0.006889855
906.879984	338.713862	7.6443E-06	0.006932463
906.919984	337.097047	7.60781E-06	0.006899676
906.959984	376.461062	8.4962E-06	0.007705715
906.999984	516.338905	1.1653E-05	0.010569316
907.039984	661.448722	1.4928E-05	0.013540271
907.079984	783.697994	1.7687E-05	0.016043498
907.119984	918.199844	2.07225E-05	0.018797786
907.159984	1014.237079	2.28899E-05	0.020764818
907.199984	1075.703195	2.42771E-05	0.022024205
907.239984	1064.452348	2.40232E-05	0.021794814
907.279984	991.132508	2.23685E-05	0.020294474
907.319984	850.679561	1.91987E-05	0.017419321
907.359984	677.737504	1.52956E-05	0.013878608
907.399984	528.217725	1.19211E-05	0.010817241
907.439984	425.11614	9.59428E-06	0.008706233
907.479984	355.929921	8.03284E-06	0.007289644
907.519984	325.162171	7.33846E-06	0.006659797
907.559984	336.731013	7.59955E-06	0.006897047
907.599984	342.638785	7.73288E-06	0.007018362
907.639984	334.654369	7.55268E-06	0.006855117
907.679984	343.761758	7.75822E-06	0.007041985
907.719984	330.764874	7.4649E-06	0.006776041
907.759984	295.443828	6.66776E-06	0.006052722
907.799984	261.342955	5.89815E-06	0.005354337
907.839984	240.930125	5.43746E-06	0.004936341
907.879984	268.113886	6.05096E-06	0.005493543
907.919984	322.002093	7.26714E-06	0.00659798
907.959984	382.446178	8.63128E-06	0.007836855
907.999984	432.746277	9.76648E-06	0.008867964
908.039984	471.983936	1.0652E-05	0.00967246
908.079984	547.68197	1.23604E-05	0.011224249

908.119984	665.871159	1.50278E-05	0.013647031
908.159984	806.782103	1.82079E-05	0.01653573
908.199984	885.755689	1.99903E-05	0.018155165
908.239984	849.316835	1.91679E-05	0.017409051
908.279984	750.297472	1.69332E-05	0.015380058
908.319984	696.879411	1.57276E-05	0.014285691
908.359984	625.52962	1.41173E-05	0.01282362
908.399984	557.09643	1.25729E-05	0.011421213
908.439984	494.345956	1.11567E-05	0.010135192
908.479984	453.643773	1.02381E-05	0.009301116
908.519984	423.49447	9.55768E-06	0.008683344
908.559984	400.936449	9.04858E-06	0.008221175
908.599984	397.19048	8.96404E-06	0.008144723
908.639984	398.111053	8.98481E-06	0.008163959
908.679984	392.582044	8.86003E-06	0.008050932
908.719984	418.732185	9.4502E-06	0.008587588
908.759984	432.259335	9.75549E-06	0.0088654
908.799984	426.420304	9.62371E-06	0.00874603
908.839984	430.251965	9.71019E-06	0.008825007
908.879984	442.381929	9.98394E-06	0.009074207
908.919984	443.167869	1.00017E-05	0.009090728
908.959984	480.362715	1.08411E-05	0.009854142
908.999984	593.321451	1.33904E-05	0.012171909
909.039984	789.940055	1.78278E-05	0.016206226
909.079984	1026.41075	2.31647E-05	0.02105853
909.119984	1284.47154	2.89887E-05	0.026354237
909.159984	1493.053363	3.36961E-05	0.030635177
909.199984	1492.604508	3.3686E-05	0.030627315
909.239984	1345.669775	3.03699E-05	0.027613521
909.279984	1164.3473	2.62777E-05	0.023893784
909.319984	1001.736127	2.26078E-05	0.020557714
909.359984	819.646628	1.84983E-05	0.016821598
909.399984	617.525959	1.39367E-05	0.012674035
909.439984	468.204698	1.05667E-05	0.009609805
909.479984	388.85347	8.77588E-06	0.007981488
909.519984	354.93337	8.01035E-06	0.007285575
909.559984	347.119343	7.834E-06	0.007125493
909.599984	357.614412	8.07086E-06	0.007341253
909.639984	378.934871	8.55203E-06	0.00777927
909.679984	408.910783	9.22855E-06	0.008395024
909.719984	400.844943	9.04651E-06	0.008229793
909.759984	364.418796	8.22442E-06	0.007482252
909.799984	340.964939	7.6951E-06	0.007001005
909.839984	334.477461	7.54869E-06	0.0068681
909.879984	344.149794	7.76698E-06	0.007067021
909.919984	354.432764	7.99905E-06	0.007278499

909.959984	387.177193	8.73805E-06	0.007951276
909.999984	450.625967	1.017E-05	0.0092547
910.039984	538.880864	1.21618E-05	0.011067716
910.079984	674.651045	1.52259E-05	0.013856818
910.119984	840.924694	1.89785E-05	0.017272711
910.159984	999.894545	2.25662E-05	0.020538877
910.199984	1073.791594	2.4234E-05	0.022057769
910.239984	1096.713236	2.47513E-05	0.022529614
910.279984	1002.603389	2.26274E-05	0.020597235
910.319984	802.225913	1.81051E-05	0.016481454
910.359984	640.248033	1.44495E-05	0.013154252
910.399984	521.702538	1.17741E-05	0.01071914
910.439984	446.531562	1.00776E-05	0.009175046
910.479984	400.812421	9.04578E-06	0.008236
910.519984	358.719756	8.09581E-06	0.007371392
910.559984	321.269729	7.25061E-06	0.006602116
910.599984	330.028167	7.44828E-06	0.0067824
910.639984	364.283036	8.22136E-06	0.0074867
910.679984	408.907136	9.22846E-06	0.008404178
910.719984	427.051482	9.63796E-06	0.00877748
910.759984	426.79753	9.63223E-06	0.008772646
910.799984	416.389022	9.39732E-06	0.008559079
910.839984	397.381951	8.96836E-06	0.008168738
910.879984	396.321061	8.94441E-06	0.008147288
910.919984	435.287647	9.82384E-06	0.008948728
910.959984	490.866993	1.10782E-05	0.010091783
910.999984	553.083277	1.24823E-05	0.011371393
911.039984	719.56731	1.62396E-05	0.014794954
911.079984	945.040239	2.13282E-05	0.019431734
911.119984	1161.740383	2.62189E-05	0.023888529
911.159984	1299.225559	2.93217E-05	0.02671677
911.199984	1333.029572	3.00846E-05	0.027413106
911.239984	1202.193245	2.71318E-05	0.024723606
911.279984	1045.287768	2.35907E-05	0.021497723
911.319984	851.056158	1.92072E-05	0.017503862
911.359984	658.581693	1.48633E-05	0.013545791
911.399984	532.733176	1.2023E-05	0.010957804
911.439984	427.912369	9.65739E-06	0.008802128
911.479984	360.244456	8.13022E-06	0.007410529
911.519984	315.677339	7.1244E-06	0.006494031
911.559984	301.198052	6.79762E-06	0.006196439
911.599984	314.461697	7.09696E-06	0.006469591
911.639984	327.86631	7.39949E-06	0.006745667
911.679984	329.615078	7.43895E-06	0.006781945
911.719984	325.259939	7.34066E-06	0.00669263
911.759984	317.329612	7.16169E-06	0.00652974

911.799984	326.350719	7.36528E-06	0.006715663
911.839984	328.568025	7.41532E-06	0.006761588
911.879984	310.103414	6.9986E-06	0.006381885
911.919984	302.214497	6.82056E-06	0.006219805
911.959984	345.718904	7.80239E-06	0.007115471
911.999984	432.510698	9.76116E-06	0.008902181
912.039984	531.830102	1.20027E-05	0.01094691
912.079984	639.859942	1.44407E-05	0.013171117
912.119984	744.42811	1.68007E-05	0.015324259
912.159984	854.135002	1.92766E-05	0.017583377
912.199984	964.636265	2.17705E-05	0.019859047
912.239984	1005.696313	2.26972E-05	0.020705261
912.279984	943.480124	2.1293E-05	0.019425207
912.319984	788.870368	1.78037E-05	0.016242676
912.359984	605.355104	1.3662E-05	0.012464682
912.399984	464.379464	1.04804E-05	0.009562315
912.439984	389.855077	8.79849E-06	0.00802809
912.479984	353.348051	7.97457E-06	0.007276639
912.519984	313.51756	7.07565E-06	0.006456676
912.559984	304.823332	6.87944E-06	0.0062779
912.599984	331.991242	7.49258E-06	0.006837728
912.639984	375.011842	8.46349E-06	0.007724124
912.679984	415.47827	9.37677E-06	0.008557986
912.719984	445.100218	1.00453E-05	0.009168539
912.759984	473.206508	1.06796E-05	0.009747922
912.799984	456.433164	1.03011E-05	0.009402808
912.839984	433.27478	9.77841E-06	0.008926122
912.879984	422.747835	9.54083E-06	0.008709633
912.919984	420.635128	9.49315E-06	0.008666485
912.959984	434.553305	9.80726E-06	0.008953638
912.999984	496.286872	1.12005E-05	0.010226059
913.039984	588.997947	1.32929E-05	0.012136916
913.079984	726.361688	1.6393E-05	0.014968094
913.119984	872.104694	1.96822E-05	0.017972199
913.159984	1000.876826	2.25884E-05	0.020626819
913.199984	1050.809573	2.37153E-05	0.021656819
913.239984	1074.910863	2.42592E-05	0.022154509
913.279984	1022.941221	2.30864E-05	0.021084309
913.319984	874.536613	1.97371E-05	0.018026263
913.359984	697.833449	1.57491E-05	0.014384624
913.399984	533.837064	1.2048E-05	0.011004606
913.439984	412.639946	9.31271E-06	0.008506601
913.479984	348.989409	7.8762E-06	0.007194755
913.519984	335.595987	7.57393E-06	0.00691894
913.559984	325.723689	7.35113E-06	0.006715698
913.599984	339.710851	7.6668E-06	0.007004389

913.639984	347.697137	7.84704E-06	0.00716937
913.679984	358.571362	8.09246E-06	0.007393915
913.719984	367.855468	8.30199E-06	0.00758569
913.759984	370.906086	8.37083E-06	0.007648933
913.799984	355.478218	8.02265E-06	0.007331096
913.839984	331.543023	7.48246E-06	0.006837775
913.879984	295.609203	6.67149E-06	0.006096939
913.919984	283.894373	6.4071E-06	0.005855577
913.959984	306.598966	6.91951E-06	0.006324157
913.999984	377.541623	8.52059E-06	0.007787818
914.039984	480.230792	1.08381E-05	0.009906493
914.079984	569.364846	1.28498E-05	0.011745719
914.119984	651.778192	1.47097E-05	0.013446454
914.159984	738.742958	1.66724E-05	0.015241241
914.199984	799.162876	1.8036E-05	0.016488504
914.239984	837.851295	1.89091E-05	0.017287489
914.279984	788.154541	1.77876E-05	0.016262801
914.319984	649.778407	1.46646E-05	0.013408131
914.359984	534.783153	1.20693E-05	0.011035695
914.399984	485.417165	1.09552E-05	0.010017425
914.439984	411.605478	9.28936E-06	0.008494564
914.479984	380.394324	8.58497E-06	0.007850783
914.519984	392.940219	8.86811E-06	0.008110067
914.559984	410.717622	9.26932E-06	0.008477353
914.599984	387.268582	8.74011E-06	0.007993707
914.639984	373.942442	8.43936E-06	0.007718976
914.679984	351.422282	7.93111E-06	0.007254429
914.719984	359.299452	8.10889E-06	0.007417362
914.759984	368.5857	8.31847E-06	0.0076094
914.799984	345.93101	7.80718E-06	0.007142009
914.839984	320.676603	7.23722E-06	0.006620902
914.879984	326.320163	7.36459E-06	0.006737717
914.919984	362.639899	8.18428E-06	0.007487959
914.959984	384.651833	8.68106E-06	0.007942819
914.999984	445.620879	1.0057E-05	0.009202194
915.039984	541.508838	1.22211E-05	0.011182795
915.079984	671.934543	1.51646E-05	0.013876846
915.119984	789.814316	1.7825E-05	0.016312022
915.159984	855.516211	1.93078E-05	0.017669734
915.199984	922.522825	2.08201E-05	0.019054514
915.239984	934.44383	2.10891E-05	0.019301584
915.279984	855.991934	1.93185E-05	0.017681878
915.319984	748.974812	1.69033E-05	0.015471945
915.359984	637.422125	1.43857E-05	0.013168121
915.399984	527.763231	1.19109E-05	0.01090322
915.439984	418.404099	9.4428E-06	0.008644315

915.479984	346.340454	7.81642E-06	0.007155778
915.519984	328.382144	7.41113E-06	0.006785035
915.559984	335.120984	7.56321E-06	0.006924576
915.599984	355.059245	8.01319E-06	0.007336879
915.639984	344.842831	7.78262E-06	0.00712608
915.679984	306.672039	6.92116E-06	0.006337568
915.719984	304.879338	6.8807E-06	0.006300796
915.759984	311.173189	7.02275E-06	0.006431149
915.799984	294.700108	6.65097E-06	0.006090959
915.839984	288.194666	6.50415E-06	0.005956763
915.879984	279.178095	6.30066E-06	0.005770649
915.919984	271.019677	6.11654E-06	0.005602258
915.959984	305.343829	6.89118E-06	0.00631205
915.999984	348.094827	7.85602E-06	0.00719611
916.039984	396.274163	8.94336E-06	0.008192472
916.079984	468.356187	1.05701E-05	0.0096831
916.119984	581.242996	1.31178E-05	0.01201752
916.159984	699.303421	1.57823E-05	0.014459116
916.199984	769.468935	1.73658E-05	0.015910584
916.239984	764.244859	1.72479E-05	0.015803254
916.279984	702.74489	1.586E-05	0.014532176
916.319984	627.047892	1.41516E-05	0.012967392
916.359984	562.590954	1.26969E-05	0.011634926
916.399984	486.057997	1.09697E-05	0.010052589
916.439984	449.160817	1.01369E-05	0.009289892
916.479984	444.601525	1.0034E-05	0.009195994
916.519984	472.947208	1.06738E-05	0.009782714
916.559984	525.299706	1.18553E-05	0.010866078
916.599984	530.531978	1.19734E-05	0.010974789
916.639984	499.551662	1.12742E-05	0.010334369
916.679984	444.429434	1.00302E-05	0.009194441
916.719984	378.401533	8.54E-06	0.007828785
916.759984	359.351953	8.11007E-06	0.00743499
916.799984	368.989551	8.32758E-06	0.007634725
916.839984	354.877624	8.00909E-06	0.007343057
916.879984	333.591574	7.5287E-06	0.006902912
916.919984	345.388524	7.79494E-06	0.007147334
916.959984	385.802891	8.70703E-06	0.007984002
916.999984	428.341888	9.66708E-06	0.008864712
917.039984	506.872104	1.14394E-05	0.010490385
917.079984	624.353858	1.40908E-05	0.012922388
917.119984	723.099369	1.63193E-05	0.014966798
917.159984	839.157693	1.89386E-05	0.017369745
917.199984	970.158382	2.18951E-05	0.020082207
917.239984	1028.14281	2.32038E-05	0.021283408
917.279984	947.741149	2.13892E-05	0.019619883

917.319984	788.749826	1.7801E-05	0.016329199
917.359984	650.147428	1.46729E-05	0.013460351
917.399984	564.224074	1.27338E-05	0.011681943
917.439984	484.022713	1.09237E-05	0.010021856
917.479984	443.007636	9.99807E-06	0.009173025
917.519984	428.319751	9.66658E-06	0.00886928
917.559984	413.051915	9.32201E-06	0.0085535
917.599984	392.493393	8.85803E-06	0.008128127
917.639984	401.421681	9.05953E-06	0.008313385
917.679984	395.877293	8.9344E-06	0.008198919
917.719984	358.379692	8.08813E-06	0.007422639
917.759984	325.026642	7.3354E-06	0.006732135
917.799984	308.389943	6.95993E-06	0.006387825
917.839984	304.509476	6.87235E-06	0.006307722
917.879984	298.327057	6.73283E-06	0.006179926
917.919984	284.910309	6.43003E-06	0.005902252
917.959984	302.208038	6.82041E-06	0.006260868
917.999984	338.992568	7.65059E-06	0.007023242
918.039984	388.610414	8.7704E-06	0.008051574
918.079984	453.080379	1.02254E-05	0.009387729
918.119984	542.245994	1.22377E-05	0.011235711
918.159984	646.278228	1.45856E-05	0.013391914
918.199984	730.92527	1.6496E-05	0.015146595
918.239984	769.958112	1.73769E-05	0.015956148
918.279984	742.825357	1.67645E-05	0.015394536
918.319984	657.956807	1.48492E-05	0.013636288
918.359984	541.706071	1.22256E-05	0.011227457
918.399984	463.145039	1.04525E-05	0.009599611
918.439984	412.362742	9.30645E-06	0.008547418
918.479984	367.280511	8.28901E-06	0.007613289
918.519984	332.974227	7.51476E-06	0.006902461
918.559984	306.558711	6.9186E-06	0.006355152
918.599984	308.603673	6.96476E-06	0.006397824
918.639984	335.373747	7.56892E-06	0.006953111
918.679984	357.443544	8.067E-06	0.007410994
918.719984	392.3506	8.85481E-06	0.008135088
918.759984	386.887083	8.7315E-06	0.008022155
918.799984	337.144017	7.60887E-06	0.00699103
918.839984	303.730812	6.85478E-06	0.006298447
918.879984	308.652316	6.96585E-06	0.006400783
918.919984	322.622878	7.28115E-06	0.006690793
918.959984	338.970631	7.65009E-06	0.007030131
918.999984	384.979884	8.68846E-06	0.007984694
919.039984	457.890601	1.0334E-05	0.009497316
919.079984	603.368785	1.36172E-05	0.01251529
919.119984	759.559504	1.71422E-05	0.015755739

919.159984	867.239293	1.95724E-05	0.017990151
919.199984	936.510869	2.11357E-05	0.019427977
919.239984	968.900936	2.18667E-05	0.020100786
919.279984	925.704458	2.08919E-05	0.019205469
919.319984	802.976617	1.81221E-05	0.016659975
919.359984	638.155689	1.44023E-05	0.013240885
919.399984	510.051885	1.15112E-05	0.010583361
919.439984	415.97097	9.38789E-06	0.008631597
919.479984	348.498954	7.86514E-06	0.007231835
919.519984	327.555772	7.39248E-06	0.006797531
919.559984	318.059565	7.17816E-06	0.00660075
919.599984	330.227061	7.45276E-06	0.006853562
919.639984	339.271823	7.65689E-06	0.007041584
919.679984	332.517919	7.50447E-06	0.006901707
919.719984	338.167337	7.63197E-06	0.007019271
919.759984	341.875646	7.71566E-06	0.007096553
919.799984	325.831994	7.35357E-06	0.006763817
919.839984	307.941328	6.94981E-06	0.00639271
919.879984	296.22271	6.68533E-06	0.006149705
919.919984	282.604014	6.37798E-06	0.00586723
919.959984	277.21847	6.25643E-06	0.00575567
919.999984	307.503732	6.93993E-06	0.006384736
920.039984	401.436456	9.05986E-06	0.008335435
920.079984	550.3867	1.24215E-05	0.011428738
920.119984	685.941522	1.54807E-05	0.014244143
920.159984	811.864642	1.83227E-05	0.016859773
920.199984	906.516226	2.04588E-05	0.018826195
920.239984	930.92887	2.10098E-05	0.019334028
920.279984	858.543055	1.93761E-05	0.017831456
920.319984	751.250702	1.69547E-05	0.015603733
920.359984	646.880722	1.45992E-05	0.013436517
920.399984	542.804134	1.22503E-05	0.011275207
920.439984	449.857298	1.01527E-05	0.009344907
920.479984	366.892212	8.28025E-06	0.007621801
920.519984	308.98605	6.97338E-06	0.00641914
920.559984	313.637379	7.07836E-06	0.006516054
920.599984	370.857338	8.36973E-06	0.007705177
920.639984	420.678966	9.49414E-06	0.008740683
920.679984	439.852585	9.92686E-06	0.009139461
920.719984	474.715536	1.07137E-05	0.009864289
920.759984	483.868162	1.09202E-05	0.010054911
920.799984	439.551643	9.92007E-06	0.009134399
920.839984	383.80034	8.66184E-06	0.007976168
920.879984	355.16381	8.01555E-06	0.007381362
920.919984	349.967511	7.89828E-06	0.007273683
920.959984	385.890983	8.70902E-06	0.008020661

920.999984	464.910685	1.04924E-05	0.009663488
921.039984	593.975227	1.34052E-05	0.012346719
921.079984	754.293732	1.70234E-05	0.015679875
921.119984	894.38273	2.0185E-05	0.018592781
921.159984	997.915891	2.25216E-05	0.020745971
921.199984	1064.17411	2.40169E-05	0.022124393
921.239984	1111.858524	2.50931E-05	0.023116765
921.279984	1117.477283	2.52199E-05	0.023234594
921.319984	1013.874783	2.28817E-05	0.021081406
921.359984	874.983541	1.97472E-05	0.018194243
921.399984	742.012892	1.67462E-05	0.015429946
921.439984	669.594109	1.51118E-05	0.013924623
921.479984	642.726778	1.45054E-05	0.01336648
921.519984	659.507918	1.48842E-05	0.013716065
921.559984	630.257135	1.4224E-05	0.013108293
921.599984	558.175676	1.25972E-05	0.011609623
921.639984	496.188177	1.11983E-05	0.010320779
921.679984	471.679533	1.06452E-05	0.009811422
921.719984	462.019398	1.04271E-05	0.009610898
921.759984	448.960523	1.01324E-05	0.009339653
921.799984	394.956417	8.91362E-06	0.008216571
921.839984	342.554354	7.73097E-06	0.007126722
921.879984	321.037011	7.24536E-06	0.006679351
921.919984	322.224977	7.27217E-06	0.006704358
921.959984	342.309123	7.72544E-06	0.007122547
921.999984	411.083579	9.27758E-06	0.008553932
922.039984	472.478772	1.06632E-05	0.009831885
922.079984	557.011094	1.2571E-05	0.011591435
922.119984	665.550277	1.50205E-05	0.013850743
922.159984	805.871057	1.81874E-05	0.016771681
922.199984	934.04791	2.10802E-05	0.019440123
922.239984	935.101017	2.11039E-05	0.019462886
922.279984	868.568829	1.96024E-05	0.018078891
922.319984	759.698063	1.71453E-05	0.015813478
922.359984	630.648369	1.42329E-05	0.013127816
922.399984	535.387432	1.20829E-05	0.011145312
922.439984	508.285923	1.14713E-05	0.010581591
922.479984	499.415453	1.12711E-05	0.010397374
922.519984	511.327486	1.15399E-05	0.010645834
922.559984	526.709948	1.18871E-05	0.010966572
922.599984	525.830053	1.18673E-05	0.010948726
922.639984	491.074808	1.10829E-05	0.010225503
922.679984	447.979768	1.01103E-05	0.009328552
922.719984	409.689421	9.24612E-06	0.008531579
922.759984	411.357373	9.28376E-06	0.008566685
922.799984	397.178508	8.96377E-06	0.008271763

922.839984	367.021733	8.28317E-06	0.00764404
922.879984	379.309808	8.56049E-06	0.007900309
922.919984	462.76373	1.04439E-05	0.009638914
922.959984	603.981466	1.3631E-05	0.012580886
922.999984	784.026711	1.76944E-05	0.016331922
923.039984	1092.373078	2.46533E-05	0.022756017
923.079984	1495.662248	3.3755E-05	0.031158577
923.119984	1797.450522	4.0566E-05	0.037447243
923.159984	1941.817024	4.38241E-05	0.040456661
923.199984	1833.62427	4.13823E-05	0.038204181
923.239984	1581.882366	3.57009E-05	0.032960481
923.279984	1348.074548	3.04242E-05	0.028090022
923.319984	1099.364765	2.48111E-05	0.022908614
923.359984	809.860611	1.82774E-05	0.016876644
923.399984	599.472415	1.35293E-05	0.012492916
923.439984	486.472321	1.0979E-05	0.01013845
923.479984	438.638963	9.89947E-06	0.009141963
923.519984	423.536686	9.55863E-06	0.008827589
923.559984	436.66203	9.85485E-06	0.009101549
923.599984	423.071783	9.54814E-06	0.008818663
923.639984	397.492466	8.97085E-06	0.008285837
923.679984	382.099212	8.62345E-06	0.007965305
923.719984	377.755618	8.52542E-06	0.007875099
923.759984	379.497802	8.56474E-06	0.007911761
923.799984	379.159285	8.5571E-06	0.007905046
923.839984	401.749007	9.06692E-06	0.008376379
923.879984	409.849952	9.24974E-06	0.008545652
923.919984	416.623051	9.4026E-06	0.008687252
923.959984	507.985216	1.14645E-05	0.010592757
923.999984	759.978971	1.71517E-05	0.01584814
924.039984	1182.951753	2.66976E-05	0.024669625
924.079984	1675.972263	3.78244E-05	0.034952734
924.119984	2155.312239	4.86424E-05	0.044951409
924.159984	2492.904947	5.62614E-05	0.051994527
924.199984	2431.164218	5.4868E-05	0.050708996
924.239984	2064.893673	4.66018E-05	0.043071223
924.279984	1636.646727	3.69368E-05	0.034139979
924.319984	1218.052493	2.74897E-05	0.025409324
924.359984	903.281286	2.03858E-05	0.018843818
924.399984	681.566548	1.5382E-05	0.014219128
924.439984	519.772253	1.17305E-05	0.010844176
924.479984	425.301793	9.59847E-06	0.008873593
924.519984	416.895263	9.40875E-06	0.008698573
924.559984	457.811875	1.03322E-05	0.009552717
924.599984	459.914381	1.03796E-05	0.009597003
924.639984	445.574153	1.0056E-05	0.009298169

924.679984	470.631968	1.06215E-05	0.009821496
924.719984	529.290491	1.19453E-05	0.011046103
924.759984	544.905861	1.22978E-05	0.011372482
924.799984	542.806932	1.22504E-05	0.011329166
924.839984	552.123143	1.24607E-05	0.011524108
924.879984	566.8648	1.27933E-05	0.011832312
924.919984	602.790861	1.36042E-05	0.01258275
924.959984	705.735569	1.59275E-05	0.014732271
924.999984	923.303071	2.08377E-05	0.019274839
925.039984	1286.845342	2.90423E-05	0.026865295
925.079984	1760.755045	3.97378E-05	0.036760633
925.119984	2255.04304	5.08932E-05	0.047082298
925.159984	2555.469474	5.76734E-05	0.053357109
925.199984	2585.363254	5.8348E-05	0.053983613
925.239984	2274.394176	5.13299E-05	0.047492484
925.279984	1739.068004	3.92483E-05	0.036315706
925.319984	1266.742999	2.85886E-05	0.026453626
925.359984	904.185893	2.04062E-05	0.018883096
925.399984	673.624446	1.52028E-05	0.014068639
925.439984	528.242712	1.19217E-05	0.01103282
925.479984	388.246471	8.76218E-06	0.008109224
925.519984	310.44457	7.0063E-06	0.006484472
925.559984	301.820886	6.81168E-06	0.006304616
925.599984	339.711212	7.66681E-06	0.007096398
925.639984	379.163874	8.5572E-06	0.007920887
925.679984	404.007538	9.11789E-06	0.008440246
925.719984	419.603616	9.46987E-06	0.008766447
925.759984	399.479954	9.01571E-06	0.00834638
925.799984	375.611691	8.47703E-06	0.007848037
925.839984	355.712497	8.02794E-06	0.007432584
925.879984	360.810869	8.143E-06	0.00753944
925.919984	390.596023	8.81521E-06	0.008162177
925.959984	413.01225	9.32111E-06	0.008630976
925.999984	490.486529	1.10696E-05	0.010250447
926.039984	669.573999	1.51114E-05	0.013993717
926.079984	903.543935	2.03917E-05	0.018884371
926.119984	1167.31844	2.63448E-05	0.024398401
926.159984	1337.441327	3.01842E-05	0.027955387
926.199984	1341.168237	3.02683E-05	0.028034498
926.239984	1242.915383	2.80509E-05	0.025981836
926.279984	1116.485352	2.51975E-05	0.023339958
926.319984	964.375757	2.17646E-05	0.020161
926.359984	751.692903	1.69647E-05	0.015715384
926.399984	558.401465	1.26023E-05	0.011674811
926.439984	444.457882	1.00308E-05	0.00929293
926.479984	398.263499	8.98825E-06	0.008327436

926.519984	398.146706	8.98562E-06	0.008325353
926.559984	409.550288	9.24298E-06	0.008564175
926.599984	431.073395	9.72873E-06	0.009014637
926.639984	465.410201	1.05037E-05	0.009733111
926.679984	471.640714	1.06443E-05	0.009863836
926.719984	482.069338	1.08796E-05	0.010082374
926.759984	520.473531	1.17464E-05	0.010886059
926.799984	521.921221	1.1779E-05	0.010916809
926.839984	512.505386	1.15665E-05	0.010720325
926.879984	521.736222	1.17749E-05	0.010913882
926.919984	538.607565	1.21556E-05	0.011267289
926.959984	581.41797	1.31218E-05	0.012163378
926.999984	688.679569	1.55425E-05	0.014407933
927.039984	907.749411	2.04866E-05	0.018991934
927.079984	1142.377138	2.57819E-05	0.023901847
927.119984	1324.613134	2.98947E-05	0.027715949
927.159984	1463.46863	3.30284E-05	0.030622656
927.199984	1494.097787	3.37197E-05	0.031264911
927.239984	1366.483275	3.08396E-05	0.028595733
927.279984	1204.624757	2.71867E-05	0.025209685
927.319984	1030.747854	2.32625E-05	0.021571821
927.359984	845.746587	1.90873E-05	0.017700819
927.399984	658.019633	1.48506E-05	0.013772434
927.439984	514.800107	1.16183E-05	0.010775296
927.479984	445.978462	1.00651E-05	0.009335191
927.519984	407.751359	9.20238E-06	0.008535392
927.559984	403.334131	9.10269E-06	0.008443291
927.599984	391.784373	8.84203E-06	0.008201865
927.639984	390.043577	8.80274E-06	0.008165774
927.679984	412.696504	9.31399E-06	0.008640398
927.719984	400.888516	9.0475E-06	0.008393542
927.759984	380.891498	8.59619E-06	0.007975202
927.799984	392.679192	8.86222E-06	0.00822237
927.839984	399.500184	9.01616E-06	0.008365556
927.879984	387.207238	8.73873E-06	0.008108491
927.919984	404.572598	9.13064E-06	0.008472503
927.959984	415.934828	9.38707E-06	0.008710825
927.999984	464.215697	1.04767E-05	0.009722379
928.039984	567.260365	1.28023E-05	0.011881024
928.079984	670.884089	1.51409E-05	0.014051984
928.119984	817.338852	1.84462E-05	0.017120286
928.159984	964.401382	2.17652E-05	0.020201584
928.199984	1003.458898	2.26467E-05	0.021020638
928.239984	954.106229	2.15328E-05	0.019987651
928.279984	877.922824	1.98135E-05	0.018392471
928.319984	789.545025	1.78189E-05	0.01654167

928.359984	651.458561	1.47025E-05	0.013649224
928.399984	536.356493	1.21048E-05	0.011238114
928.439984	455.491775	1.02798E-05	0.009544191
928.479984	404.853953	9.13699E-06	0.008483512
928.519984	374.511126	8.45219E-06	0.007848031
928.559984	381.626018	8.61277E-06	0.007997471
928.599984	398.549893	8.99472E-06	0.008352493
928.639984	435.298332	9.82408E-06	0.009123031
928.679984	502.681455	1.13448E-05	0.010535707
928.719984	541.052553	1.22108E-05	0.011340416
928.759984	533.335153	1.20366E-05	0.011179142
928.799984	508.824648	1.14835E-05	0.010665841
928.839984	453.936091	1.02447E-05	0.009515692
928.879984	407.693781	9.20108E-06	0.0085467
928.919984	400.641758	9.04193E-06	0.008399226
928.959984	447.015849	1.00885E-05	0.009371836
928.999984	530.235891	1.19667E-05	0.011117051
929.039984	635.632504	1.43453E-05	0.013327395
929.079984	745.3705	1.6822E-05	0.015628959
929.119984	863.783007	1.94944E-05	0.018112617
929.159984	993.440193	2.24206E-05	0.020832288
929.199984	1078.180781	2.4333E-05	0.022610259
929.239984	1082.702001	2.44351E-05	0.02270605
929.279984	1019.915186	2.30181E-05	0.021390227
929.319984	863.197145	1.94812E-05	0.018104229
929.359984	724.827054	1.63583E-05	0.015202784
929.399984	580.411601	1.30991E-05	0.012174286
929.439984	461.826999	1.04228E-05	0.00968736
929.479984	439.504733	9.91901E-06	0.009219521
929.519984	410.234871	9.25843E-06	0.008605895
929.559984	370.272971	8.35655E-06	0.00776791
929.599984	351.72717	7.93799E-06	0.007379158
929.639984	356.769169	8.05178E-06	0.00748526
929.679984	357.123034	8.05977E-06	0.007493006
929.719984	349.405519	7.8856E-06	0.007331396
929.759984	350.069414	7.90058E-06	0.007345642
929.799984	339.739085	7.66744E-06	0.007129184
929.839984	322.953909	7.28862E-06	0.00677725
929.879984	337.281013	7.61196E-06	0.007078212
929.919984	364.474686	8.22569E-06	0.00764923
929.959984	364.998168	8.2375E-06	0.007660546
929.999984	387.888531	8.7541E-06	0.008141316
930.039984	441.661091	9.96768E-06	0.009270337
930.079984	552.624003	1.2472E-05	0.011599915
930.119984	640.187994	1.44482E-05	0.013438514
930.159984	682.243043	1.53973E-05	0.014321929

930.199984	728.300042	1.64367E-05	0.015289434
930.239984	757.794347	1.71024E-05	0.015909302
930.279984	742.931871	1.67669E-05	0.015597946
930.319984	702.117455	1.58458E-05	0.014741676
930.359984	600.394162	1.35501E-05	0.012606434
930.399984	487.10683	1.09933E-05	0.010228187
930.439984	404.68934	9.13327E-06	0.008497964
930.479984	347.882892	7.85123E-06	0.007305415
930.519984	334.247007	7.54349E-06	0.007019368
930.559984	353.987604	7.98901E-06	0.00743425
930.599984	381.423765	8.6082E-06	0.008010794
930.639984	382.208886	8.62592E-06	0.008027628
930.679984	400.524352	9.03928E-06	0.008412674
930.719984	413.798464	9.33885E-06	0.008691859
930.759984	413.120814	9.32356E-06	0.008677998
930.799984	384.973751	8.68832E-06	0.008087089
930.839984	345.863807	7.80566E-06	0.007265825
930.879984	334.547758	7.55028E-06	0.007028402
930.919984	326.791449	7.37523E-06	0.006865747
930.959984	355.065487	8.01333E-06	0.007460093
930.999984	425.353515	9.59964E-06	0.008937261
931.039984	544.749562	1.22942E-05	0.011446428
931.079984	668.836683	1.50947E-05	0.014054385
931.119984	770.278561	1.73841E-05	0.016186696
931.159984	855.436651	1.9306E-05	0.017976987
931.199984	931.21531	2.10162E-05	0.019570315
931.239984	920.421173	2.07726E-05	0.019344298
931.279984	847.900866	1.91359E-05	0.017820919
931.319984	767.237367	1.73155E-05	0.016126251
931.359984	672.483552	1.5177E-05	0.014135266
931.399984	556.897029	1.25684E-05	0.011706198
931.439984	451.029365	1.01791E-05	0.009481225
931.479984	361.206369	8.15192E-06	0.007593355
931.519984	327.611788	7.39374E-06	0.006887418
931.559984	329.584331	7.43826E-06	0.006929185
931.599984	313.194466	7.06836E-06	0.006584887
931.639984	317.796611	7.17223E-06	0.006681933
931.679984	344.31818	7.77078E-06	0.007239882
931.719984	358.96274	8.10129E-06	0.007548133
931.759984	344.437729	7.77348E-06	0.007243017
931.799984	331.172951	7.47411E-06	0.006964378
931.839984	342.883143	7.73839E-06	0.007210946
931.879984	334.979336	7.56002E-06	0.007045029
931.919984	321.112153	7.24705E-06	0.006753674
931.959984	335.775806	7.57799E-06	0.007062386
931.999984	371.320893	8.3802E-06	0.007810342

932.039984	425.095382	9.59381E-06	0.008941815
932.079984	533.094608	1.20312E-05	0.011214043
932.119984	627.391521	1.41594E-05	0.013198216
932.159984	697.447099	1.57404E-05	0.01467258
932.199984	783.038757	1.76721E-05	0.016473926
932.239984	780.691542	1.76191E-05	0.016425249
932.279984	717.062578	1.61831E-05	0.015087184
932.319984	674.790654	1.52291E-05	0.01419838
932.359984	605.2264	1.36591E-05	0.012735214
932.399984	495.240739	1.11769E-05	0.010421335
932.439984	377.8013	8.52645E-06	0.007950402
932.479984	324.021393	7.31271E-06	0.006818957
932.519984	315.763955	7.12635E-06	0.006645466
932.559984	292.461444	6.60045E-06	0.006155313
932.599984	286.101657	6.45692E-06	0.00602172
932.639984	298.719013	6.74167E-06	0.006287553
932.679984	337.666562	7.62066E-06	0.007107641
932.719984	381.59625	8.6121E-06	0.008032674
932.759984	383.341581	8.65149E-06	0.00806976
932.799984	356.849674	8.0536E-06	0.007512398
932.839984	335.350695	7.5684E-06	0.007060104
932.879984	351.0473	7.92265E-06	0.00739088
932.919984	375.310853	8.47024E-06	0.007902059
932.959984	397.631553	8.97399E-06	0.008372374
932.999984	460.383849	1.03902E-05	0.009694077
933.039984	544.440805	1.22873E-05	0.011464515
933.079984	670.63578	1.51353E-05	0.01412246
933.119984	846.448019	1.91032E-05	0.017825534
933.159984	955.732192	2.15695E-05	0.020127836
933.199984	988.684786	2.23132E-05	0.020822714
933.239984	1023.879739	2.31075E-05	0.02156488
933.279984	1001.537621	2.26033E-05	0.021095216
933.319984	868.668447	1.96046E-05	0.018297399
933.359984	717.635	1.6196E-05	0.015116719
933.399984	568.065876	1.28205E-05	0.011966613
933.439984	449.16544	1.0137E-05	0.009462317
933.479984	370.758048	8.36749E-06	0.007810887
933.519984	337.869095	7.62523E-06	0.007118309
933.559984	349.588878	7.88973E-06	0.00736554
933.599984	355.043902	8.01285E-06	0.007480793
933.639984	365.457669	8.24787E-06	0.007700542
933.679984	359.712433	8.11821E-06	0.007579809
933.719984	331.496001	7.4814E-06	0.006985535
933.759984	323.693352	7.30531E-06	0.006821404
933.799984	317.079654	7.15605E-06	0.006682316
933.839984	285.588017	6.44532E-06	0.006018901

933.879984	262.886776	5.93299E-06	0.005540699
933.919984	247.313278	5.58152E-06	0.00521269
933.959984	267.801419	6.04391E-06	0.005644765
933.999984	352.38888	7.95293E-06	0.007428033
934.039984	473.629841	1.06892E-05	0.009984108
934.079984	575.306546	1.29839E-05	0.012127971
934.119984	664.670987	1.50007E-05	0.014012452
934.159984	741.081314	1.67252E-05	0.015623987
934.199984	777.96683	1.75576E-05	0.016402336
934.239984	788.222249	1.77891E-05	0.016619268
934.279984	763.686792	1.72353E-05	0.01610264
934.319984	711.647015	1.60609E-05	0.015006003
934.359984	626.970834	1.41499E-05	0.013221061
934.399984	551.501705	1.24466E-05	0.011630126
934.439984	477.407874	1.07744E-05	0.010068059
934.479984	378.037166	8.53177E-06	0.00797277
934.519984	333.834033	7.53417E-06	0.007040832
934.559984	324.633942	7.32654E-06	0.006847087
934.599984	348.275072	7.86008E-06	0.007346034
934.639984	356.177645	8.03843E-06	0.007513041
934.679984	348.178815	7.85791E-06	0.007344632
934.719984	372.52129	8.40729E-06	0.007858459
934.759984	384.118464	8.66902E-06	0.008103452
934.799984	377.577736	8.5214E-06	0.007965808
934.839984	364.835688	8.23383E-06	0.007697317
934.879984	359.056725	8.10341E-06	0.007575716
934.919984	364.654229	8.22974E-06	0.007694147
934.959984	397.668726	8.97483E-06	0.008391106
934.999984	444.416893	1.00299E-05	0.009377929
935.039984	525.624822	1.18626E-05	0.011092024
935.079984	684.621167	1.54509E-05	0.01444787
935.119984	830.340913	1.87396E-05	0.01752381
935.159984	927.786627	2.09389E-05	0.019581176
935.199984	983.615023	2.21988E-05	0.020760337
935.239984	986.433976	2.22624E-05	0.020820725
935.279984	943.309102	2.12892E-05	0.019911337
935.319984	853.309509	1.9258E-05	0.018012399
935.359984	743.284753	1.67749E-05	0.015690572
935.399984	619.276311	1.39762E-05	0.013073341
935.439984	504.869139	1.13942E-05	0.010658584
935.479984	431.43163	9.73681E-06	0.009108592
935.519984	377.67833	8.52367E-06	0.007974067
935.559984	331.589431	7.48351E-06	0.007001274
935.599984	308.904774	6.97155E-06	0.006522583
935.639984	317.85792	7.17361E-06	0.006711917
935.679984	338.367927	7.63649E-06	0.007145313

935.719984	362.712797	8.18592E-06	0.007659731
935.759984	367.675174	8.29792E-06	0.007764858
935.799984	343.81527	7.75943E-06	0.007261276
935.839984	322.113808	7.26966E-06	0.006803238
935.879984	310.978428	7.01835E-06	0.006568333
935.919984	318.412364	7.18612E-06	0.006725637
935.959984	328.156693	7.40604E-06	0.006931757
935.999984	378.731449	8.54744E-06	0.008000405
936.039984	447.954851	1.01097E-05	0.009463099
936.079984	548.462305	1.2378E-05	0.011586826
936.119984	648.386333	1.46332E-05	0.013698409
936.159984	732.352575	1.65282E-05	0.015473018
936.199984	767.130916	1.73131E-05	0.016208502
936.239984	777.662023	1.75507E-05	0.016431713
936.279984	764.538484	1.72546E-05	0.016155107
936.319984	698.390998	1.57617E-05	0.014758006
936.359984	580.335413	1.30974E-05	0.012263846
936.399984	512.580537	1.15682E-05	0.010832489
936.439984	478.678914	1.08031E-05	0.01011647
936.479984	409.764908	9.24782E-06	0.008660401
936.519984	362.410354	8.1791E-06	0.007659888
936.559984	361.33518	8.15483E-06	0.007637489
936.599984	390.940804	8.82299E-06	0.008263612
936.639984	387.627605	8.74822E-06	0.008193928
936.679984	363.72111	8.20868E-06	0.007688905
936.719984	348.431001	7.8636E-06	0.007365993
936.759984	375.768942	8.48058E-06	0.00794427
936.799984	386.306034	8.71839E-06	0.008167387
936.839984	357.500326	8.06828E-06	0.007558691
936.879984	350.083712	7.9009E-06	0.007402197
936.919984	353.596177	7.98017E-06	0.007476784
936.959984	415.204734	9.37059E-06	0.00877987
936.999984	493.686086	1.11418E-05	0.010439873
937.039984	638.440375	1.44087E-05	0.013501537
937.079984	781.862418	1.76455E-05	0.016535288
937.119984	903.042769	2.03804E-05	0.019098896
937.159984	993.66074	2.24255E-05	0.021016318
937.199984	1032.102205	2.32931E-05	0.021830302
937.239984	1042.824508	2.35351E-05	0.022058034
937.279984	987.71317	2.22913E-05	0.020893199
937.319984	869.130759	1.96151E-05	0.018385598
937.359984	790.237695	1.78346E-05	0.016717407
937.399984	799.236514	1.80377E-05	0.016908497
937.439984	831.463782	1.8765E-05	0.017591042
937.479984	826.597917	1.86552E-05	0.017488843
937.519984	767.040807	1.7311E-05	0.016229448

937.559984	685.612649	1.54733E-05	0.014507168
937.599984	569.628044	1.28557E-05	0.012053515
937.639984	466.192607	1.05213E-05	0.009865208
937.679984	395.499931	8.92588E-06	0.008369621
937.719984	372.593924	8.40893E-06	0.007885218
937.759984	375.145416	8.46651E-06	0.007939554
937.799984	379.572421	8.56642E-06	0.008033589
937.839984	350.514429	7.91062E-06	0.007418898
937.879984	310.539946	7.00845E-06	0.006573089
937.919984	306.626794	6.92014E-06	0.006490537
937.959984	327.329765	7.38738E-06	0.006929064
937.999984	377.360718	8.51651E-06	0.007988482
938.039984	452.386182	1.02097E-05	0.009577131
938.079984	521.834874	1.17771E-05	0.011047849
938.119984	640.351731	1.44518E-05	0.013557566
938.159984	752.803832	1.69897E-05	0.015939089
938.199983	802.368214	1.81083E-05	0.016989238
938.239983	808.985004	1.82577E-05	0.017130071
938.279983	781.929955	1.76471E-05	0.016557892
938.319983	740.912294	1.67214E-05	0.015689985
938.359983	689.721356	1.55661E-05	0.014606559
938.399983	643.15855	1.45152E-05	0.013621057
938.439983	616.578411	1.39153E-05	0.013058689
938.479983	579.862523	1.30867E-05	0.012281596
938.519983	548.33712	1.23752E-05	0.011614377
938.559983	487.975655	1.10129E-05	0.010336296
938.599983	471.744717	1.06466E-05	0.009992918
938.639983	466.357349	1.0525E-05	0.009879219
938.679983	409.169063	9.23438E-06	0.008668124
938.719983	384.607155	8.68005E-06	0.008148134
938.759983	386.135464	8.71454E-06	0.008180861
938.799983	394.474093	8.90273E-06	0.008357884
938.839983	419.618152	9.4702E-06	0.008891
938.879983	450.512754	1.01674E-05	0.009546011
938.919983	482.133874	1.08811E-05	0.010216473
938.959983	518.698008	1.17063E-05	0.010991739
938.999983	579.599814	1.30808E-05	0.012282834
939.039983	702.910752	1.58637E-05	0.014896664
939.079983	893.266283	2.01598E-05	0.018931642
939.119983	1074.25947	2.42445E-05	0.022768532
939.159983	1182.577925	2.66891E-05	0.025065369
939.199983	1251.976226	2.82554E-05	0.026537433
939.239983	1178.302259	2.65926E-05	0.024976871
939.279983	1015.201618	2.29117E-05	0.021520488
939.319983	845.318523	1.90777E-05	0.017920029
939.359983	705.976393	1.59329E-05	0.014966732

939.399983	578.093812	1.30468E-05	0.012256138
939.439983	487.399097	1.09999E-05	0.010333763
939.479983	455.461213	1.02791E-05	0.009657032
939.519983	412.305114	9.30515E-06	0.008742376
939.559983	391.124158	8.82713E-06	0.008293616
939.599983	386.955759	8.73305E-06	0.008205576
939.639983	386.454408	8.72174E-06	0.008195293
939.679983	375.249996	8.46887E-06	0.007958027
939.719983	374.880514	8.46053E-06	0.00795053
939.759983	376.8283	8.50449E-06	0.007992179
939.799983	350.218113	7.90393E-06	0.007428118
939.839983	333.087524	7.51732E-06	0.007065079
939.879983	344.871927	7.78328E-06	0.007315348
939.919983	371.660633	8.38786E-06	0.00788392
939.959983	402.619508	9.08656E-06	0.008541004
939.999983	441.276982	9.95901E-06	0.009361466
940.039983	549.632225	1.24044E-05	0.011660664
940.079983	723.346486	1.63249E-05	0.015346733
940.119983	880.351279	1.98683E-05	0.018678588
940.159983	990.931139	2.23639E-05	0.021025677
940.199983	1050.579218	2.37101E-05	0.022292245
940.239983	1026.244761	2.31609E-05	0.021776818
940.279983	923.838372	2.08497E-05	0.019604598
940.319983	788.466993	1.77946E-05	0.01673262
940.359983	622.179515	1.40417E-05	0.013204276
940.399983	491.89746	1.11014E-05	0.010439794
940.439983	431.754784	9.7441E-06	0.009163745
940.479983	402.31607	9.07971E-06	0.008539289
940.519983	400.635133	9.04178E-06	0.008503972
940.559983	407.875394	9.20518E-06	0.008658023
940.599983	396.556798	8.94973E-06	0.00841812
940.639983	411.156245	9.27922E-06	0.008728409
940.679983	423.538427	9.55867E-06	0.008991652
940.719983	456.868146	1.03109E-05	0.009699649
940.759983	454.829293	1.02649E-05	0.009656773
940.799983	418.334045	9.44122E-06	0.008882297
940.839983	425.465791	9.60217E-06	0.009034106
940.879983	433.127963	9.77509E-06	0.009197191
940.919983	446.336147	1.00732E-05	0.009478061
940.959983	535.295856	1.20809E-05	0.011367626
940.999983	663.691368	1.49786E-05	0.014094853
941.039983	795.78052	1.79597E-05	0.016900756
941.079983	959.030013	2.1644E-05	0.020368708
941.119983	1158.33737	2.61421E-05	0.024602816
941.159983	1329.277985	3E-05	0.028234755
941.199983	1392.713551	3.14316E-05	0.029583426

941.239983	1319.75173	2.9785E-05	0.028034794
941.279983	1124.129162	2.537E-05	0.023880301
941.319983	913.380843	2.06137E-05	0.019404119
941.359983	748.197482	1.68858E-05	0.015895593
941.399983	577.751426	1.3039E-05	0.012274957
941.439983	440.114494	9.93277E-06	0.009351108
941.479983	340.398723	7.68233E-06	0.007232755
941.519983	294.368081	6.64348E-06	0.006254967
941.559983	303.29413	6.84493E-06	0.006444908
941.599983	312.703003	7.05727E-06	0.006645126
941.639983	323.440802	7.29961E-06	0.006873603
941.679983	352.989773	7.96649E-06	0.007501882
941.719983	371.014254	8.37327E-06	0.00788528
941.759983	404.032846	9.11846E-06	0.008587399
941.799983	398.85185	9.00153E-06	0.008477641
941.839983	355.92367	8.0327E-06	0.007565519
941.879983	332.877585	7.51258E-06	0.007075952
941.919983	342.207402	7.72314E-06	0.007274584
941.959983	380.316302	8.58321E-06	0.00808504
941.999983	460.257864	1.03874E-05	0.009784911
942.039983	576.282959	1.30059E-05	0.012252081
942.079983	692.924775	1.56383E-05	0.014732574
942.119983	811.974381	1.83251E-05	0.017264472
942.159983	942.613707	2.12735E-05	0.02004302
942.199983	956.900211	2.15959E-05	0.020347661
942.239983	893.331839	2.01613E-05	0.018996741
942.279983	842.892084	1.90229E-05	0.017924898
942.319983	754.527166	1.70286E-05	0.016046416
942.359983	616.467206	1.39128E-05	0.013110872
942.399983	480.865476	1.08525E-05	0.010227362
942.439983	411.024375	9.27625E-06	0.008742307
942.479983	377.203082	8.51295E-06	0.008023283
942.519983	381.122145	8.6014E-06	0.008106988
942.559983	400.073889	9.02911E-06	0.008510478
942.599983	406.47055	9.17347E-06	0.008646917
942.639983	415.61441	9.37984E-06	0.008841811
942.679983	422.842915	9.54298E-06	0.008995972
942.719983	426.409701	9.62347E-06	0.00907224
942.759983	429.448797	9.69206E-06	0.009137287
942.799983	414.018144	9.34381E-06	0.008809347
942.839983	385.109998	8.6914E-06	0.008194596
942.879983	374.223475	8.4457E-06	0.007963284
942.919983	390.156761	8.80529E-06	0.008302688
942.959983	440.544278	9.94247E-06	0.009375352
942.999983	531.81657	1.20024E-05	0.011318225
943.039983	657.401354	1.48366E-05	0.013991538

943.079983	782.349613	1.76565E-05	0.01665153
943.119983	902.98049	2.0379E-05	0.019219853
943.159983	1009.926379	2.27926E-05	0.021497098
943.199983	1096.665132	2.47502E-05	0.023344392
943.239983	1066.925099	2.4079E-05	0.022712288
943.279983	954.399371	2.15395E-05	0.020317746
943.319983	820.463922	1.85167E-05	0.017467199
943.359983	679.300513	1.53309E-05	0.014462526
943.399983	528.030171	1.19169E-05	0.011242408
943.439983	431.106282	9.72947E-06	0.009179169
943.479983	398.041743	8.98325E-06	0.008475514
943.519983	392.329398	8.85433E-06	0.008354235
943.559983	395.194518	8.91899E-06	0.008415602
943.599983	383.247651	8.64937E-06	0.008161541
943.639983	369.329825	8.33526E-06	0.007865484
943.679983	376.67518	8.50103E-06	0.008022256
943.719983	390.711065	8.8178E-06	0.008321538
943.759983	382.186272	8.62541E-06	0.008140319
943.799983	382.702165	8.63705E-06	0.008151652
943.839983	395.022462	8.91511E-06	0.008414434
943.879983	378.446925	8.54102E-06	0.008061698
943.919983	353.277363	7.97298E-06	0.007525853
943.959983	363.023168	8.19293E-06	0.007733795
943.999983	409.923464	9.2514E-06	0.008733323
944.039983	493.218868	1.11313E-05	0.010508357
944.079983	590.289795	1.3322E-05	0.012577051
944.119983	682.703238	1.54077E-05	0.014546681
944.159983	770.608893	1.73916E-05	0.016420424
944.199983	811.958392	1.83248E-05	0.017302247
944.239983	804.093744	1.81473E-05	0.017135383
944.279983	760.346541	1.716E-05	0.016203809
944.319983	664.342131	1.49933E-05	0.014158451
944.359983	586.749943	1.32421E-05	0.012505337
944.399983	539.439537	1.21744E-05	0.011497502
944.439983	487.953753	1.10124E-05	0.010400585
944.479983	457.668972	1.0329E-05	0.009755488
944.519983	483.407877	1.09098E-05	0.010304564
944.559983	494.634396	1.11632E-05	0.010544321
944.599983	448.356697	1.01188E-05	0.009558205
944.639983	378.86633	8.55049E-06	0.00807713
944.679983	353.337143	7.97433E-06	0.007533187
944.719983	378.213297	8.53575E-06	0.008063891
944.759983	394.640084	8.90648E-06	0.008414483
944.799983	367.529319	8.29462E-06	0.007836761
944.839983	332.160238	7.49639E-06	0.007082893
944.879983	339.618195	7.66471E-06	0.007242231

944.919983	367.866488	8.30223E-06	0.007844947
944.959983	375.44828	8.47334E-06	0.008006972
944.999983	410.902819	9.2735E-06	0.008763461
945.039983	520.045229	1.17367E-05	0.011091647
945.079983	680.483752	1.53576E-05	0.014514132
945.119983	810.877137	1.83004E-05	0.017296043
945.159983	902.471492	2.03675E-05	0.019250569
945.199983	957.324226	2.16055E-05	0.020421494
945.239983	937.465143	2.11573E-05	0.019998709
945.279983	838.92764	1.89334E-05	0.017897391
945.319983	714.144578	1.61172E-05	0.015235957
945.359983	604.944473	1.36528E-05	0.012906767
945.399983	500.941428	1.13056E-05	0.010688267
945.439983	423.196612	9.55096E-06	0.009029858
945.479983	383.114204	8.64635E-06	0.008174955
945.519983	375.399019	8.47223E-06	0.008010666
945.559983	364.492141	8.22608E-06	0.007778252
945.599983	380.354942	8.58408E-06	0.008117107
945.639983	411.990824	9.29806E-06	0.008792616
945.679983	406.657081	9.17768E-06	0.008679152
945.719983	391.795942	8.84229E-06	0.008362329
945.759983	366.577769	8.27315E-06	0.007824414
945.799983	327.300851	7.38672E-06	0.006986364
945.839983	309.304008	6.98056E-06	0.006602493
945.879983	316.973226	7.15364E-06	0.006766489
945.919983	321.173358	7.24844E-06	0.00685644
945.959983	346.572609	7.82166E-06	0.007398978
945.999983	399.204665	9.00949E-06	0.00852298
946.039983	452.851072	1.02202E-05	0.009668735
946.079983	538.269029	1.2148E-05	0.011492963
946.119983	634.582339	1.43216E-05	0.013549989
946.159983	719.254929	1.62326E-05	0.015358619
946.199983	773.892772	1.74657E-05	0.016526028
946.239983	762.334735	1.72048E-05	0.016279901
946.279983	694.10762	1.5665E-05	0.014823516
946.319983	585.37065	1.3211E-05	0.012501834
946.359983	490.705161	1.10745E-05	0.010480494
946.399983	432.126936	9.7525E-06	0.009229769
946.439983	396.987233	8.95945E-06	0.008479581
946.479983	362.789898	8.18766E-06	0.007749459
946.519983	349.847844	7.89558E-06	0.007473323
946.559983	358.713577	8.09567E-06	0.007663033
946.599983	373.608897	8.43183E-06	0.007981572
946.639983	368.044515	8.30625E-06	0.00786303
946.679983	379.971323	8.57542E-06	0.008118182
946.719983	400.525753	9.03931E-06	0.008557694

946.759983	406.606969	9.17655E-06	0.008687993
946.799983	400.546479	9.03978E-06	0.00855886
946.839983	366.03483	8.2609E-06	0.007821747
946.879983	338.142647	7.63141E-06	0.007226028
946.919983	371.950194	8.3944E-06	0.007948823
946.959983	434.19893	9.79927E-06	0.009279512
946.999983	517.32686	1.16753E-05	0.011056553
947.039983	624.758351	1.40999E-05	0.013353193
947.079983	721.221489	1.6277E-05	0.015415587
947.119983	806.600471	1.82038E-05	0.017241229
947.159983	928.022597	2.09442E-05	0.019837487
947.199983	995.422957	2.24653E-05	0.021279141
947.239983	958.790312	2.16386E-05	0.020496911
947.279983	885.254328	1.9979E-05	0.018925666
947.319983	775.065968	1.74922E-05	0.016570672
947.359983	632.074207	1.4265E-05	0.013514123
947.399983	522.975498	1.18028E-05	0.011182
947.439983	438.595107	9.89848E-06	0.009378216
947.479983	369.053059	8.32901E-06	0.007891574
947.519983	325.492691	7.34592E-06	0.006960403
947.559983	318.163048	7.1805E-06	0.006803951
947.599983	304.19141	6.86518E-06	0.006505441
947.639983	299.01888	6.74844E-06	0.006395091
947.679983	299.475583	6.75875E-06	0.006405129
947.719983	294.341978	6.64289E-06	0.006295598
947.759983	323.467508	7.30021E-06	0.006918848
947.799983	342.067395	7.71998E-06	0.007317001
947.839983	314.358698	7.09464E-06	0.006724582
947.879983	314.996562	7.10903E-06	0.006738511
947.919983	334.319222	7.54512E-06	0.007152169
947.959983	353.280329	7.97304E-06	0.007558128
947.999983	392.439338	8.85681E-06	0.008396255
948.039983	465.831824	1.05132E-05	0.009966911
948.079983	571.240477	1.28921E-05	0.012222744
948.119983	667.450098	1.50634E-05	0.014281929
948.159983	733.987824	1.65651E-05	0.015706349
948.199983	760.723232	1.71685E-05	0.016279137
948.239983	750.241056	1.69319E-05	0.0160555
948.279983	719.152193	1.62303E-05	0.015390834
948.319983	656.445393	1.48151E-05	0.014049416
948.359983	563.374445	1.27146E-05	0.012057996
948.399983	460.821334	1.04001E-05	0.009863451
948.439983	382.048461	8.6223E-06	0.008177736
948.479983	338.621704	7.64222E-06	0.007248493
948.519983	328.275054	7.40871E-06	0.00702731
948.559983	309.929222	6.99467E-06	0.006634865

948.599983	300.0656	6.77206E-06	0.006423979
948.639983	304.90319	6.88124E-06	0.00652782
948.679983	338.335947	7.63577E-06	0.007243903
948.719983	376.809309	8.50406E-06	0.008067973
948.759983	377.209706	8.5131E-06	0.008076886
948.799983	362.007278	8.17E-06	0.007751696
948.839983	332.127393	7.49565E-06	0.007112175
948.879983	304.095034	6.863E-06	0.006512165
948.919983	320.338867	7.2296E-06	0.006860314
948.959983	392.560546	8.85954E-06	0.008407353
948.999983	502.334745	1.1337E-05	0.010758809
949.039983	616.476852	1.3913E-05	0.013204016
949.079983	777.087404	1.75378E-05	0.016644756
949.119983	971.282855	2.19205E-05	0.020805186
949.159983	1100.112175	2.4828E-05	0.023565744
949.199983	1112.269054	2.51024E-05	0.023827163
949.239983	1054.979258	2.38094E-05	0.022600846
949.279983	965.730273	2.17952E-05	0.020689735
949.319983	836.592494	1.88807E-05	0.017923852
949.359983	701.756267	1.58377E-05	0.015035642
949.399983	605.37879	1.36626E-05	0.01297123
949.439983	487.518843	1.10026E-05	0.010446329
949.479983	371.765063	8.39022E-06	0.007966346
949.519983	310.145589	6.99955E-06	0.006646216
949.559983	292.817515	6.60848E-06	0.006275151
949.599983	318.245502	7.18236E-06	0.006820367
949.639983	351.370746	7.92995E-06	0.007530596
949.679983	338.900205	7.64851E-06	0.007263633
949.719983	305.296049	6.89011E-06	0.006543672
949.759983	303.524228	6.85012E-06	0.006505969
949.799983	328.534973	7.41458E-06	0.007042365
949.839983	330.943938	7.46894E-06	0.007094301
949.879983	328.79796	7.42051E-06	0.007048596
949.919983	341.789477	7.71371E-06	0.00732741
949.959983	345.363961	7.79438E-06	0.007404352
949.999983	376.543981	8.49807E-06	0.008073169
950.039983	461.481693	1.0415E-05	0.009894665
950.079983	561.123159	1.26638E-05	0.012031593
950.119983	641.211912	1.44713E-05	0.013749433
950.159983	710.092872	1.60258E-05	0.015227081
950.199983	738.890605	1.66757E-05	0.01584528
950.239983	739.352548	1.66862E-05	0.015855854
950.279983	698.294593	1.57595E-05	0.014975972
950.319983	637.053696	1.43774E-05	0.013663144
950.359983	522.619438	1.17948E-05	0.011209299
950.399983	410.093055	9.25523E-06	0.00879617

950.439983	350.903529	7.9194E-06	0.007526918
950.479983	344.747049	7.78046E-06	0.007395172
950.519983	349.35672	7.88449E-06	0.00749437
950.559983	352.324551	7.95147E-06	0.007558353
950.599983	354.32915	7.99672E-06	0.007601678
950.639983	350.039394	7.8999E-06	0.007509962
950.679983	345.685363	7.80164E-06	0.00741686
950.719983	359.754313	8.11915E-06	0.007719042
950.759983	394.194113	8.89641E-06	0.008458353
950.799983	413.212086	9.32562E-06	0.008866801
950.839983	420.512811	9.49039E-06	0.009023841
950.879983	398.943924	9.00361E-06	0.008561351
950.919983	380.130245	8.57901E-06	0.008157952
950.959983	449.435115	1.01431E-05	0.009645706
950.999983	584.487878	1.31911E-05	0.012544714
951.039983	735.382697	1.65966E-05	0.015783995
951.079983	887.445647	2.00284E-05	0.019048622
951.119983	1041.792642	2.35118E-05	0.022362551
951.159983	1127.354036	2.54428E-05	0.024200183
951.199983	1103.591895	2.49065E-05	0.023691093
951.239983	1053.445214	2.37748E-05	0.022615532
951.279983	959.875589	2.16631E-05	0.020607631
951.319983	808.213674	1.82403E-05	0.017352321
951.359983	670.763137	1.51382E-05	0.014401868
951.399983	566.113216	1.27764E-05	0.012155454
951.439983	484.827313	1.09419E-05	0.010410539
951.479983	412.437723	9.30814E-06	0.008856514
951.519983	358.891413	8.09968E-06	0.007707007
951.559983	308.080127	6.95294E-06	0.006616139
951.599983	289.909915	6.54286E-06	0.006226188
951.639983	334.517296	7.54959E-06	0.007184491
951.679983	352.619055	7.95812E-06	0.007573584
951.719983	356.952311	8.05592E-06	0.007666977
951.759983	341.064658	7.69735E-06	0.007326034
951.799983	300.261406	6.77648E-06	0.006449855
951.839983	282.520697	6.3761E-06	0.006069026
951.879983	329.36077	7.43321E-06	0.007075527
951.919983	380.886619	8.59608E-06	0.008182781
951.959983	376.412154	8.4951E-06	0.008086993
951.999983	391.454989	8.83459E-06	0.008410533
952.039983	466.0898	1.0519E-05	0.010014506
952.079983	571.493882	1.28978E-05	0.012279757
952.119983	676.679736	1.52717E-05	0.014540509
952.159983	770.602886	1.73914E-05	0.016559427
952.199983	823.02939	1.85746E-05	0.017686759
952.239983	816.717875	1.84322E-05	0.017551863

952.279983	746.761296	1.68534E-05	0.01604912
952.319983	669.857058	1.51177E-05	0.014396927
952.359983	608.090938	1.37238E-05	0.013069965
952.399983	508.916108	1.14855E-05	0.010938816
952.439983	431.076658	9.7288E-06	0.009266098
952.479983	401.060109	9.05137E-06	0.008621247
952.519983	390.709141	8.81776E-06	0.008399094
952.559983	386.578014	8.72453E-06	0.008310636
952.599983	407.235655	9.19074E-06	0.0087551
952.639983	406.01248	9.16314E-06	0.00872917
952.679983	403.646072	9.10973E-06	0.008678657
952.719983	426.32653	9.6216E-06	0.009166687
952.759983	428.089852	9.66139E-06	0.009204987
952.799983	410.521823	9.26491E-06	0.008827602
952.839983	422.497797	9.53519E-06	0.009085507
952.879983	417.6184	9.42507E-06	0.008980956
952.919983	438.209394	9.88978E-06	0.009424165
952.959983	528.869287	1.19358E-05	0.011374381
952.999983	626.232979	1.41332E-05	0.013468945
953.039983	726.705324	1.64007E-05	0.015630549
953.079983	871.667051	1.96723E-05	0.018749286
953.119983	1014.645386	2.28991E-05	0.021825621
953.159983	1039.561057	2.34614E-05	0.022362511
953.199983	1015.502363	2.29185E-05	0.021845889
953.239983	1003.423476	2.26459E-05	0.021586949
953.279983	908.992129	2.05147E-05	0.01955624
953.319983	771.381341	1.7409E-05	0.01659635
953.359983	641.429857	1.44762E-05	0.013801009
953.399983	530.03929	1.19622E-05	0.011404808
953.439983	471.640722	1.06443E-05	0.010148677
953.479983	415.471575	9.37661E-06	0.008940414
953.519983	375.040269	8.46414E-06	0.008070723
953.559983	371.066844	8.37446E-06	0.007985552
953.599983	376.796336	8.50377E-06	0.008109193
953.639983	351.650418	7.93626E-06	0.007568335
953.679983	329.858273	7.44444E-06	0.007099615
953.719983	344.600146	7.77715E-06	0.007417219
953.759983	340.819572	7.69182E-06	0.007336153
953.799983	349.698012	7.8922E-06	0.007527577
953.839983	368.471921	8.3159E-06	0.007932036
953.879983	384.397744	8.67532E-06	0.008275216
953.919983	378.773888	8.5484E-06	0.008154489
953.959983	363.394713	8.20131E-06	0.007823724
953.999983	391.151976	8.82776E-06	0.008421678
954.039983	459.278821	1.03653E-05	0.009888895
954.079983	577.1453	1.30254E-05	0.012427241

954.119983	722.380005	1.63031E-05	0.015555124
954.159983	836.087834	1.88693E-05	0.018004367
954.199983	835.754895	1.88618E-05	0.017997952
954.239983	823.510848	1.85855E-05	0.01773502
954.279983	758.630089	1.71212E-05	0.016338442
954.319983	636.348032	1.43615E-05	0.013705456
954.359983	570.6992	1.28799E-05	0.012292048
954.399983	494.201974	1.11535E-05	0.010644853
954.439983	435.726461	9.83374E-06	0.009385714
954.479983	389.080859	8.78101E-06	0.008381301
954.519983	353.848717	7.98587E-06	0.007622675
954.559983	341.06829	7.69744E-06	0.007347665
954.599983	361.557081	8.15984E-06	0.007789383
954.639983	372.763415	8.41275E-06	0.008031149
954.679983	390.023693	8.80229E-06	0.008403371
954.719983	411.98084	9.29783E-06	0.008876828
954.759983	415.289202	9.3725E-06	0.008948487
954.799983	410.437563	9.263E-06	0.008844316
954.839983	387.807305	8.75227E-06	0.008357018
954.879983	383.248169	8.64938E-06	0.008259117
954.919983	412.406817	9.30745E-06	0.008887868
954.959983	474.508219	1.0709E-05	0.010226656
954.999983	557.892841	1.25909E-05	0.012024275
955.039983	661.194689	1.49222E-05	0.014251339
955.079983	792.708759	1.78903E-05	0.017086698
955.119983	915.160779	2.06539E-05	0.019726957
955.159983	971.247408	2.19197E-05	0.020936821
955.199983	947.096861	2.13747E-05	0.020417072
955.239983	888.683453	2.00563E-05	0.019158625
955.279983	792.715788	1.78905E-05	0.017090428
955.319983	703.110256	1.58682E-05	0.015159227
955.359983	613.017856	1.3835E-05	0.013217366
955.399983	504.870578	1.13942E-05	0.010886043
955.439983	445.722004	1.00593E-05	0.009611081
955.479983	436.882454	9.85983E-06	0.009420869
955.519983	396.754851	8.9542E-06	0.008555921
955.559983	347.737594	7.84795E-06	0.00749919
955.599983	335.567392	7.57329E-06	0.007237034
955.639983	352.452355	7.95436E-06	0.007601503
955.679983	378.250455	8.53659E-06	0.008158244
955.719983	388.963275	8.77836E-06	0.008389653
955.759983	379.469748	8.5641E-06	0.008185228
955.799983	361.841692	8.16626E-06	0.007805314
955.839983	355.127149	8.01473E-06	0.007660795
955.879983	340.161657	7.67697E-06	0.007338267
955.919983	321.442456	7.25451E-06	0.00693473

955.959983	332.528868	7.50471E-06	0.007174206
955.999983	399.785896	9.02261E-06	0.008625616
956.039983	501.7223	1.13232E-05	0.010825407
956.079983	614.669406	1.38722E-05	0.013262964
956.119983	655.891022	1.48025E-05	0.014153011
956.159983	689.450297	1.55599E-05	0.014877785
956.199983	761.925204	1.71956E-05	0.016442423
956.239983	781.805633	1.76443E-05	0.016872151
956.279983	718.310437	1.62113E-05	0.015502509
956.319983	638.391727	1.44076E-05	0.013778287
956.359983	587.433693	1.32576E-05	0.012679
956.399983	509.743235	1.15042E-05	0.011002612
956.439983	440.391843	9.93903E-06	0.009506086
956.479983	384.443763	8.67636E-06	0.008298765
956.519983	340.720989	7.6896E-06	0.007355254
956.559983	345.89958	7.80647E-06	0.007467358
956.599983	382.062852	8.62263E-06	0.008248404
956.639983	392.484623	8.85783E-06	0.008473756
956.679983	413.915747	9.3415E-06	0.008936828
956.719983	443.854379	1.00172E-05	0.009583632
956.759983	468.681055	1.05775E-05	0.010120108
956.799983	441.410111	9.96201E-06	0.009531652
956.839983	381.67648	8.61391E-06	0.00824213
956.879983	366.231412	8.26533E-06	0.007908932
956.919983	370.841124	8.36937E-06	0.008008815
956.959983	408.931922	9.22902E-06	0.008831807
956.999983	460.713131	1.03977E-05	0.009950554
957.039983	529.310358	1.19458E-05	0.011432606
957.079983	637.240874	1.43816E-05	0.013764379
957.119983	757.222021	1.70894E-05	0.016356651
957.159983	878.052747	1.98164E-05	0.018967491
957.199983	935.795327	2.11196E-05	0.020215678
957.239983	914.585657	2.06409E-05	0.019758318
957.279983	880.402766	1.98695E-05	0.01902064
957.319983	766.910791	1.73081E-05	0.016569398
957.359983	609.603	1.37579E-05	0.013171254
957.399983	473.632544	1.06892E-05	0.010233866
957.439983	387.655646	8.74885E-06	0.008376497
957.479983	343.339064	7.74868E-06	0.00741921
957.519983	344.212702	7.7684E-06	0.007438399
957.559983	347.24978	7.83694E-06	0.007504344
957.599983	353.361825	7.97488E-06	0.007636749
957.639983	351.052187	7.92276E-06	0.007587151
957.679983	349.337435	7.88406E-06	0.007550406
957.719983	343.474765	7.75175E-06	0.007424003
957.759983	319.009547	7.1996E-06	0.00689549

957.799983	304.259554	6.86671E-06	0.006576939
957.839983	314.452326	7.09675E-06	0.006797552
957.879983	326.592673	7.37074E-06	0.007060286
957.919983	349.267363	7.88248E-06	0.007550783
957.959983	360.729497	8.14116E-06	0.007798908
957.999983	375.895557	8.48344E-06	0.008127135
958.039983	414.561762	9.35608E-06	0.0089635
958.079983	500.914905	1.1305E-05	0.010831048
958.119983	606.654114	1.36913E-05	0.013117945
958.159983	647.882787	1.46218E-05	0.014010035
958.199983	683.499824	1.54256E-05	0.014780847
958.239983	719.476031	1.62376E-05	0.015559491
958.279983	686.909351	1.55026E-05	0.014855819
958.319983	646.125581	1.45822E-05	0.01397437
958.359983	577.316702	1.30292E-05	0.012486696
958.399983	494.093797	1.1151E-05	0.010687127
958.439983	426.744544	9.63103E-06	0.009230764
958.479983	411.402859	9.28479E-06	0.008899285
958.519983	389.469663	8.78979E-06	0.008425187
958.559983	394.577355	8.90506E-06	0.008536035
958.599983	402.118332	9.07525E-06	0.008699535
958.639983	419.870992	9.4759E-06	0.00908398
958.679983	454.17925	1.02502E-05	0.009826655
958.719983	484.79559	1.09412E-05	0.01048951
958.759983	458.074037	1.03381E-05	0.00991175
958.799983	412.381773	9.30688E-06	0.008923439
958.839983	417.047586	9.41218E-06	0.009024777
958.879983	420.091733	9.48089E-06	0.009091031
958.919983	411.74877	9.2926E-06	0.008910856
958.959983	441.460987	9.96316E-06	0.009554271
958.999983	503.352344	1.136E-05	0.010894203
959.039983	604.016131	1.36318E-05	0.013073444
959.079983	742.844733	1.6765E-05	0.016078948
959.119983	884.211156	1.99554E-05	0.019139637
959.159983	996.14106	2.24815E-05	0.021563371
959.199983	1013.303482	2.28688E-05	0.021935799
959.239983	954.047067	2.15315E-05	0.020653889
959.279983	875.442197	1.97575E-05	0.018952985
959.319983	764.793484	1.72603E-05	0.016558173
959.359983	647.059927	1.46032E-05	0.014009765
959.399983	529.122788	1.19416E-05	0.011456737
959.439983	391.659555	8.83921E-06	0.008480692
959.479983	302.169292	6.81954E-06	0.006543212
959.519983	287.80057	6.49526E-06	0.00623233
959.559983	302.566521	6.8285E-06	0.00655236
959.599983	338.393709	7.63707E-06	0.007328537

959.639983	357.396205	8.06593E-06	0.007740393
959.679983	346.062635	7.81015E-06	0.007495246
959.719983	337.594821	7.61904E-06	0.00731215
959.759983	344.913979	7.78423E-06	0.007470991
959.799983	369.632114	8.34208E-06	0.00800673
959.839983	365.937604	8.2587E-06	0.007927032
959.879983	336.534639	7.59512E-06	0.007290402
959.919983	332.562883	7.50548E-06	0.007204661
959.959983	339.804258	7.66891E-06	0.007361846
959.999983	355.849846	8.03104E-06	0.007709794
960.039983	387.265101	8.74003E-06	0.008390782
960.079983	490.035846	1.10594E-05	0.010617934
960.119983	619.346	1.39778E-05	0.013420343
960.159983	677.275229	1.52852E-05	0.014676198
960.199983	708.840222	1.59975E-05	0.015360834
960.239983	696.992944	1.57302E-05	0.015104728
960.279983	636.607712	1.43673E-05	0.013796678
960.319983	565.738898	1.27679E-05	0.012261307
960.359983	514.772766	1.16177E-05	0.011157178
960.399983	458.283289	1.03428E-05	0.00993324
960.439983	422.332992	9.53147E-06	0.009154402
960.479983	447.766842	1.01055E-05	0.009706105
960.519983	508.985668	1.14871E-05	0.011033587
960.559983	508.712882	1.14809E-05	0.011028133
960.599983	488.572626	1.10264E-05	0.010591963
960.639983	467.430937	1.05493E-05	0.010134046
960.679983	433.318078	9.77939E-06	0.00939486
960.719983	403.279396	9.10145E-06	0.008743949
960.759983	360.509555	8.1362E-06	0.007816934
960.799983	338.58575	7.64141E-06	0.007341865
960.839983	355.646326	8.02644E-06	0.007712127
960.879983	376.103321	8.48813E-06	0.008156072
960.919983	409.061466	9.23195E-06	0.008871163
960.959983	438.314801	9.89215E-06	0.009505965
960.999983	461.350478	1.0412E-05	0.010005968
961.039983	513.416121	1.15871E-05	0.011135654
961.079983	607.623194	1.37132E-05	0.013179491
961.119983	737.69989	1.66489E-05	0.016001551
961.159983	829.334165	1.87169E-05	0.017989952
961.199983	825.905645	1.86395E-05	0.017916326
961.239983	803.449341	1.81327E-05	0.017429907
961.279983	780.390582	1.76123E-05	0.016930379
961.319983	701.054889	1.58218E-05	0.015209844
961.359983	593.830183	1.34019E-05	0.01288407
961.399983	493.803011	1.11444E-05	0.01071427
961.439983	428.343393	9.66711E-06	0.00929435

961.479983	393.891039	8.88957E-06	0.008547146
961.519983	372.067014	8.39703E-06	0.008073916
961.559983	336.100139	7.58531E-06	0.007293732
961.599983	325.790673	7.35264E-06	0.0070703
961.639983	344.853278	7.78286E-06	0.007484308
961.679983	348.956674	7.87547E-06	0.007573678
961.719983	356.927035	8.05535E-06	0.007746987
961.759983	343.252566	7.74673E-06	0.007450497
961.799983	316.900392	7.152E-06	0.006878794
961.839983	314.22085	7.09153E-06	0.006820914
961.879983	302.911678	6.83629E-06	0.006575695
961.919983	301.166584	6.79691E-06	0.006538084
961.959983	337.89959	7.62592E-06	0.007335833
961.999983	394.711559	8.90809E-06	0.008569583
962.039983	470.102253	1.06096E-05	0.010206814
962.079983	536.763055	1.2114E-05	0.011654632
962.119983	570.123472	1.28669E-05	0.012379495
962.159983	609.20488	1.37489E-05	0.013228647
962.199983	633.656711	1.43007E-05	0.013760181
962.239983	667.626339	1.50674E-05	0.014498451
962.279983	683.291249	1.54209E-05	0.014839254
962.319983	623.284001	1.40667E-05	0.01353662
962.359983	539.803006	1.21826E-05	0.011724049
962.399983	485.796439	1.09637E-05	0.010551512
962.439983	419.964306	9.47801E-06	0.009122015
962.479983	366.10295	8.26243E-06	0.007952427
962.519983	333.560316	7.52799E-06	0.007245842
962.559983	331.239511	7.47561E-06	0.007195727
962.599983	339.347155	7.65859E-06	0.007372161
962.639983	348.743857	7.87066E-06	0.007576615
962.679983	354.171704	7.99316E-06	0.007694857
962.719983	374.687881	8.45618E-06	0.008140937
962.759983	384.10336	8.66868E-06	0.008345856
962.799983	388.435774	8.76645E-06	0.008440342
962.839983	404.660295	9.13262E-06	0.008793251
962.879983	406.167499	9.16663E-06	0.008826369
962.919983	407.029265	9.18608E-06	0.008845463
962.959983	458.119917	1.03391E-05	0.009956167
962.999983	543.06776	1.22563E-05	0.0118028
963.039983	634.574977	1.43215E-05	0.013792151
963.079983	714.481899	1.61249E-05	0.015529531
963.119983	862.838462	1.94731E-05	0.018754895
963.159983	1000.751211	2.25856E-05	0.021753508
963.199983	991.289016	2.2372E-05	0.021548722
963.239983	891.301811	2.01154E-05	0.019375996
963.279983	793.30483	1.79038E-05	0.017246358

963.319983	717.657023	1.61965E-05	0.015602431
963.359983	601.116797	1.35664E-05	0.013069297
963.399983	472.169196	1.06562E-05	0.010266184
963.439983	396.119431	8.93986E-06	0.008613022
963.479983	342.425763	7.72807E-06	0.007445843
963.519983	297.481505	6.71374E-06	0.006468826
963.559983	273.000293	6.16124E-06	0.005936721
963.599983	275.964462	6.22813E-06	0.006001429
963.639983	305.462954	6.89387E-06	0.006643212
963.679983	324.140675	7.3154E-06	0.007049708
963.719983	308.899225	6.97143E-06	0.006718502
963.759983	318.713304	7.19292E-06	0.006932244
963.799983	337.84739	7.62475E-06	0.007348729
963.839983	327.585694	7.39315E-06	0.007125816
963.879983	313.186798	7.06819E-06	0.006812887
963.919983	318.080699	7.17864E-06	0.006919633
963.959983	328.163951	7.4062E-06	0.007139284
963.999983	366.924017	8.28096E-06	0.007982849
964.039983	479.277631	1.08166E-05	0.010427662
964.079983	599.04253	1.35196E-05	0.013033933
964.119983	672.157952	1.51697E-05	0.014625381
964.159983	730.763322	1.64923E-05	0.015901226
964.199983	791.183429	1.78559E-05	0.017216666
964.239983	824.398755	1.86055E-05	0.017940197
964.279983	768.760452	1.73499E-05	0.016730116
964.319983	649.409463	1.46563E-05	0.014133331
964.359983	525.423921	1.18581E-05	0.011435464
964.399983	419.411325	9.46553E-06	0.009128556
964.439983	352.978165	7.96623E-06	0.007682946
964.479983	330.982943	7.46982E-06	0.007204496
964.519983	305.877963	6.90324E-06	0.006658313
964.559983	321.855776	7.26384E-06	0.007006406
964.599983	345.472239	7.79683E-06	0.007520819
964.639983	347.421727	7.84082E-06	0.007563573
964.679983	358.534793	8.09163E-06	0.007805834
964.719983	394.2699	8.89812E-06	0.008584197
964.759983	415.339565	9.37364E-06	0.009043308
964.799983	401.451918	9.06021E-06	0.008741291
964.839983	386.035572	8.71229E-06	0.008405961
964.879983	391.447526	8.83443E-06	0.00852416
964.919983	429.566226	9.69471E-06	0.009354621
964.959983	472.411711	1.06617E-05	0.010288089
964.999983	516.710939	1.16614E-05	0.011253295
965.039983	646.447258	1.45894E-05	0.014079368
965.079983	816.065463	1.84175E-05	0.017774323
965.119983	930.028275	2.09894E-05	0.020257331

965.159983	1026.510064	2.31669E-05	0.022359767
965.199983	1078.186532	2.43332E-05	0.023486374
965.239983	1015.836975	2.2926E-05	0.022129117
965.279983	913.82112	2.06237E-05	0.019907616
965.319983	827.326559	1.86716E-05	0.018024076
965.359983	740.637782	1.67152E-05	0.01613615
965.399983	631.530621	1.42528E-05	0.013759621
965.439983	582.743126	1.31517E-05	0.012697178
965.479983	584.878929	1.31999E-05	0.012744242
965.519983	567.223525	1.28014E-05	0.012360051
965.559983	536.408942	1.2106E-05	0.011689072
965.599983	533.279634	1.20354E-05	0.011621361
965.639983	497.519363	1.12283E-05	0.010842514
965.679983	442.066529	9.97683E-06	0.009634421
965.719983	379.367974	8.56181E-06	0.008268308
965.759983	348.1115	7.85639E-06	0.007587389
965.799983	342.004693	7.71857E-06	0.007454594
965.839983	320.601388	7.23553E-06	0.006988361
965.879983	291.209009	6.57218E-06	0.006347939
965.919983	293.274972	6.61881E-06	0.006393239
965.959983	357.639535	8.07143E-06	0.007796675
965.999983	428.485675	9.67032E-06	0.009341534
966.039983	477.719435	1.07815E-05	0.010415324
966.079983	562.568449	1.26964E-05	0.012265725
966.119983	665.312256	1.50152E-05	0.014506457
966.159983	745.275831	1.68198E-05	0.016250654
966.199983	768.397381	1.73417E-05	0.01675551
966.239983	742.097294	1.67481E-05	0.016182686
966.279983	712.184222	1.6073E-05	0.015531024
966.319983	634.603104	1.43221E-05	0.013839739
966.359983	520.013401	1.1736E-05	0.01134118
966.399983	472.685972	1.06679E-05	0.010309424
966.439983	466.505693	1.05284E-05	0.010175051
966.479983	471.094846	1.0632E-05	0.010275571
966.519983	470.473186	1.06179E-05	0.010262436
966.559983	467.541656	1.05518E-05	0.010198913
966.599983	481.793651	1.08734E-05	0.010510239
966.639983	479.035245	1.08112E-05	0.010450498
966.679983	463.974093	1.04712E-05	0.010122347
966.719983	452.44859	1.02111E-05	0.009871308
966.759983	423.791689	9.56439E-06	0.009246468
966.799983	400.951679	9.04892E-06	0.008748497
966.839983	405.667772	9.15536E-06	0.008851765
966.879983	428.029592	9.66003E-06	0.009340091
966.919983	463.597922	1.04628E-05	0.010116651
966.959983	547.40026	1.23541E-05	0.011945883

966.999983	645.028575	1.45574E-05	0.014077002
967.039983	763.622452	1.72339E-05	0.016665866
967.079983	917.162546	2.06991E-05	0.020017667
967.119983	1062.741088	2.39846E-05	0.023195972
967.159983	1165.951503	2.63139E-05	0.025449752
967.199983	1185.056811	2.67451E-05	0.025867842
967.239983	1149.154065	2.59348E-05	0.025085182
967.279983	1072.991794	2.42159E-05	0.023423585
967.319983	933.149394	2.10599E-05	0.020371645
967.359983	766.13094	1.72905E-05	0.016726146
967.399983	610.546652	1.37792E-05	0.013329986
967.439983	523.400286	1.18124E-05	0.011427803
967.479983	462.005022	1.04268E-05	0.01008773
967.519983	413.453901	9.33108E-06	0.009028005
967.559983	383.256562	8.64957E-06	0.008368975
967.599983	374.287961	8.44716E-06	0.00817347
967.639983	364.819171	8.23346E-06	0.007967026
967.679983	355.520922	8.02361E-06	0.007764289
967.719983	359.516467	8.11379E-06	0.007851873
967.759983	347.258023	7.83713E-06	0.007584461
967.799983	349.118603	7.87912E-06	0.007625413
967.839983	361.715444	8.16341E-06	0.007900878
967.879983	383.565685	8.65654E-06	0.008378495
967.919983	380.905611	8.59651E-06	0.008320733
967.959983	395.6502	8.92927E-06	0.00864318
967.999983	436.635167	9.85425E-06	0.009538911
968.039983	487.150759	1.09943E-05	0.010642936
968.079983	592.979363	1.33827E-05	0.012955542
968.119983	687.234608	1.55099E-05	0.015015471
968.159983	778.721619	1.75747E-05	0.017015085
968.199983	875.860878	1.9767E-05	0.01913837
968.239983	902.287734	2.03634E-05	0.019716636
968.279983	880.833039	1.98792E-05	0.019248607
968.319983	789.735844	1.78232E-05	0.017258598
968.359983	634.202664	1.43131E-05	0.013860205
968.399983	507.006169	1.14424E-05	0.011080842
968.439983	427.833862	9.65561E-06	0.009350883
968.479983	346.543831	7.82101E-06	0.007574493
968.519983	321.414649	7.25388E-06	0.007025529
968.559983	351.505886	7.933E-06	0.007683585
968.599983	368.206941	8.30992E-06	0.008048986
968.639983	379.127283	8.55637E-06	0.008288047
968.679983	375.555995	8.47578E-06	0.008210314
968.719983	370.817036	8.36882E-06	0.008107047
968.759983	382.065796	8.62269E-06	0.00835332
968.799983	400.32155	9.0347E-06	0.008752817

968.839983	395.858917	8.93398E-06	0.008655601
968.879983	416.562727	9.40124E-06	0.009108674
968.919983	456.274961	1.02975E-05	0.009977444
968.959983	482.227648	1.08832E-05	0.010545391
968.999983	521.275778	1.17645E-05	0.01139977
969.039983	622.672464	1.40529E-05	0.013617774
969.079983	785.228473	1.77215E-05	0.017173563
969.119983	922.157067	2.08118E-05	0.020169132
969.159983	1020.79552	2.30379E-05	0.022327443
969.199983	1056.856584	2.38518E-05	0.023117146
969.239983	1020.686826	2.30355E-05	0.022326908
969.279983	934.3098	2.10861E-05	0.020438306
969.319983	834.790954	1.88401E-05	0.018262055
969.359983	693.292105	1.56466E-05	0.015167224
969.399983	511.378762	1.15411E-05	0.011187948
969.439983	393.579187	8.88253E-06	0.008611084
969.479983	342.151	7.72187E-06	0.0074862
969.519983	343.972844	7.76299E-06	0.007526372
969.559983	352.065628	7.94563E-06	0.007703766
969.599983	332.356402	7.50082E-06	0.007272796
969.639983	322.218265	7.27202E-06	0.007051239
969.679983	329.480842	7.43592E-06	0.007210466
969.719983	336.470726	7.59368E-06	0.007363739
969.759983	359.62905	8.11633E-06	0.007870889
969.799983	366.865077	8.27963E-06	0.008029589
969.839983	348.316085	7.86101E-06	0.007623921
969.879983	340.070912	7.67493E-06	0.007443758
969.919983	353.406683	7.9759E-06	0.007735981
969.959983	368.119456	8.30794E-06	0.008058373
969.999983	384.136635	8.66943E-06	0.008409346
970.039983	426.063795	9.61567E-06	0.009327581
970.079983	502.524594	1.13413E-05	0.011001949
970.119983	602.631146	1.36005E-05	0.013194161
970.159983	684.093603	1.5439E-05	0.014978339
970.199983	728.393397	1.64388E-05	0.015948947
970.239983	730.305898	1.6482E-05	0.015991483
970.279983	685.870053	1.54791E-05	0.015019092
970.319983	617.265695	1.39308E-05	0.01351736
970.359983	540.224105	1.21921E-05	0.011830731
970.399983	459.317146	1.03661E-05	0.01005931
970.439983	385.054128	8.69014E-06	0.008433255
970.479983	326.993985	7.3798E-06	0.007161947
970.519983	313.334375	7.07152E-06	0.006863052
970.559983	336.05904	7.58438E-06	0.0073611
970.599983	361.563103	8.15998E-06	0.007920072
970.639983	366.455831	8.2704E-06	0.008027579

970.679983	365.778029	8.2551E-06	0.008013061
970.719983	373.862465	8.43756E-06	0.008190503
970.759983	376.063198	8.48722E-06	0.008239056
970.799983	376.033258	8.48655E-06	0.00823874
970.839983	386.261451	8.71738E-06	0.008463184
970.879983	381.375661	8.60712E-06	0.008356478
970.919983	377.667855	8.52344E-06	0.008275576
970.959983	416.626248	9.40267E-06	0.00912962
970.999983	482.231991	1.08833E-05	0.010567688
971.039983	540.542534	1.21993E-05	0.011846
971.079983	655.036758	1.47833E-05	0.014355735
971.119983	803.258362	1.81284E-05	0.017604873
971.159983	888.211519	2.00457E-05	0.019467579
971.199983	921.751761	2.08027E-05	0.020203537
971.239983	922.49083	2.08193E-05	0.020220569
971.279983	900.2934	2.03184E-05	0.019734824
971.319983	797.109981	1.79897E-05	0.017473718
971.359983	657.30449	1.48344E-05	0.014409588
971.399983	562.226631	1.26887E-05	0.012325776
971.439983	480.858069	1.08523E-05	0.010542355
971.479983	431.275916	9.7333E-06	0.009455703
971.519983	352.798367	7.96217E-06	0.007735405
971.559983	309.805785	6.99189E-06	0.006793036
971.599983	309.585311	6.98691E-06	0.006788481
971.639983	312.948379	7.06281E-06	0.006862508
971.679983	324.215864	7.3171E-06	0.00710988
971.719983	349.787496	7.89422E-06	0.007670968
971.759983	367.479301	8.2935E-06	0.008059288
971.799983	357.535344	8.06907E-06	0.007841527
971.839983	331.233956	7.47549E-06	0.007264979
971.879983	286.435293	6.46445E-06	0.006282665
971.919983	281.098494	6.344E-06	0.006165862
971.959983	312.79772	7.05941E-06	0.006861463
971.999983	398.699511	8.99809E-06	0.008746146
972.039983	501.520835	1.13186E-05	0.011002158
972.079983	574.563624	1.29671E-05	0.012605059
972.119983	664.468612	1.49961E-05	0.014578039
972.159983	687.098557	1.55069E-05	0.015075146
972.199983	671.302051	1.51504E-05	0.014729172
972.239983	673.032599	1.51894E-05	0.01476775
972.279983	645.517352	1.45684E-05	0.01416459
972.319983	605.042281	1.3655E-05	0.013276992
972.359983	577.907567	1.30426E-05	0.012682072
972.399983	504.223545	1.13796E-05	0.011065545
972.439983	417.03487	9.4119E-06	0.009152504
972.479983	373.131345	8.42105E-06	0.008189307

972.519983	348.26263	7.8598E-06	0.007643815
972.559983	343.998693	7.76357E-06	0.007550539
972.599983	335.205835	7.56513E-06	0.007357844
972.639983	315.002716	7.10917E-06	0.006914665
972.679983	331.312828	7.47727E-06	0.00727299
972.719983	371.578097	8.386E-06	0.00815723
972.759983	375.56515	8.47598E-06	0.008245096
972.799983	344.831495	7.78237E-06	0.007570686
972.839983	351.706244	7.93752E-06	0.007721937
972.879983	405.141549	9.14348E-06	0.008895509
972.919983	459.680239	1.03743E-05	0.010093405
972.959983	488.855806	1.10328E-05	0.010734468
972.999983	537.07416	1.2121E-05	0.011793748
973.039983	670.793381	1.51389E-05	0.014730728
973.079983	821.56191	1.85415E-05	0.01804237
973.119983	929.960438	2.09879E-05	0.020423756
973.159983	1044.97046	2.35835E-05	0.022950545
973.199983	1168.664468	2.63751E-05	0.025668275
973.239983	1221.744652	2.75731E-05	0.026835219
973.279983	1162.509495	2.62362E-05	0.025535188
973.319983	972.759342	2.19538E-05	0.021368095
973.359983	785.562761	1.77291E-05	0.017256755
973.399983	626.328579	1.41354E-05	0.013759363
973.439983	473.12495	1.06778E-05	0.010394169
973.479983	400.32801	9.03485E-06	0.008795241
973.519983	388.803371	8.77475E-06	0.008542395
973.559983	366.7173	8.2763E-06	0.008057473
973.599983	331.32583	7.47756E-06	0.007280155
973.639983	312.648022	7.05603E-06	0.006870033
973.679983	310.495292	7.00745E-06	0.00682301
973.719983	323.16462	7.29338E-06	0.007101705
973.759983	357.370824	8.06536E-06	0.007853726
973.799983	369.139713	8.33097E-06	0.008112698
973.839983	367.414857	8.29204E-06	0.008075122
973.879983	379.727099	8.56991E-06	0.008346065
973.919983	377.001709	8.5084E-06	0.008286504
973.959983	383.890383	8.66387E-06	0.008438264
973.999983	406.526671	9.17474E-06	0.008936197
974.039983	439.128689	9.91052E-06	0.009653246
974.079983	513.05889	1.1579E-05	0.011278896
974.119983	642.482689	1.44999E-05	0.014124681
974.159983	756.712112	1.70779E-05	0.016636644
974.199983	862.630234	1.94684E-05	0.018966079
974.239983	908.843736	2.05113E-05	0.019982965
974.279983	845.515057	1.90821E-05	0.018591305
974.319983	721.982955	1.62941E-05	0.015875716

974.359983	595.440592	1.34383E-05	0.013093707
974.399983	496.761411	1.12112E-05	0.010924206
974.439983	427.603192	9.65041E-06	0.009403744
974.479983	373.203444	8.42268E-06	0.008207735
974.519983	340.794466	7.69126E-06	0.007495283
974.559983	366.193559	8.26448E-06	0.00805423
974.599983	384.811306	8.68466E-06	0.008464065
974.639983	340.623192	7.68739E-06	0.007492439
974.679983	320.626134	7.23609E-06	0.007052867
974.719983	347.536456	7.84341E-06	0.007645132
974.759983	371.540717	8.38516E-06	0.008173515
974.799983	375.548385	8.4756E-06	0.008262019
974.839983	356.990026	8.05677E-06	0.007854059
974.879983	367.51896	8.29439E-06	0.008086036
974.919983	402.864306	9.09209E-06	0.008864057
974.959983	459.714358	1.03751E-05	0.01011532
974.999983	490.839011	1.10776E-05	0.010800614
975.039983	529.879076	1.19586E-05	0.011660145
975.079983	620.142491	1.39958E-05	0.013646978
975.119983	725.907668	1.63827E-05	0.015975122
975.159983	838.734139	1.89291E-05	0.018458863
975.199983	952.552046	2.14978E-05	0.020964628
975.239983	960.874918	2.16856E-05	0.021148672
975.279983	902.012146	2.03572E-05	0.019853928
975.319983	775.452688	1.75009E-05	0.017068965
975.359983	648.305842	1.46314E-05	0.014270843
975.399983	544.370453	1.22857E-05	0.011983455
975.439983	441.021806	9.95325E-06	0.009708796
975.479983	378.441065	8.54089E-06	0.008331465
975.519983	335.551676	7.57293E-06	0.007387548
975.559983	283.130401	6.38986E-06	0.006233691
975.599983	258.972294	5.84464E-06	0.005702035
975.639983	288.434604	6.50957E-06	0.006350994
975.679983	324.696246	7.32794E-06	0.007149726
975.719983	348.011813	7.85414E-06	0.007663443
975.759983	339.08428	7.65266E-06	0.007467159
975.799983	330.576485	7.46065E-06	0.007280103
975.839983	341.867303	7.71547E-06	0.007529063
975.879983	316.830146	7.15042E-06	0.006977947
975.919983	280.862236	6.33867E-06	0.006186034
975.959983	281.491646	6.35287E-06	0.006200151
975.999983	339.053468	7.65196E-06	0.007468317
976.039983	430.129626	9.70743E-06	0.009474836
976.079983	503.96681	1.13738E-05	0.011101767
976.119983	578.902389	1.3065E-05	0.012753028
976.159983	640.169777	1.44477E-05	0.014103306

976.199983	687.100601	1.55069E-05	0.015137839
976.239983	685.918414	1.54802E-05	0.015112413
976.279983	657.816876	1.4846E-05	0.014493863
976.319983	602.773139	1.36038E-05	0.013281613
976.359983	515.722995	1.16391E-05	0.011364
976.399983	414.693096	9.35905E-06	0.009138172
976.439983	326.739007	7.37404E-06	0.007200312
976.479983	294.563301	6.64788E-06	0.006491525
976.519983	292.143359	6.59327E-06	0.006438459
976.559983	318.946487	7.19818E-06	0.007029453
976.599983	342.3224	7.72574E-06	0.007544957
976.639983	357.361007	8.06514E-06	0.007876738
976.679983	371.988414	8.39526E-06	0.008199483
976.719983	388.492262	8.76773E-06	0.008563616
976.759983	386.641278	8.72596E-06	0.008523164
976.799983	346.042218	7.80969E-06	0.007628506
976.839983	346.217305	7.81364E-06	0.007632678
976.879983	356.789945	8.05225E-06	0.007866084
976.919983	370.508706	8.36187E-06	0.008168873
976.959983	415.332065	9.37347E-06	0.009157501
976.999983	463.255813	1.0455E-05	0.010214572
977.039983	528.499462	1.19275E-05	0.011653641
977.079983	604.499318	1.36427E-05	0.013330017
977.119983	690.580716	1.55854E-05	0.01522885
977.159983	782.032957	1.76494E-05	0.017246282
977.199983	855.088213	1.92981E-05	0.018858152
977.239983	871.231819	1.96625E-05	0.01921497
977.279983	819.726439	1.85001E-05	0.018079762
977.319983	720.332356	1.62569E-05	0.015888191
977.359983	602.860934	1.36057E-05	0.013297698
977.399983	487.882146	1.10108E-05	0.010761976
977.439983	401.39152	9.05885E-06	0.00885448
977.479983	336.476267	7.5938E-06	0.007422788
977.519983	295.867558	6.67732E-06	0.006527212
977.559983	294.703184	6.65104E-06	0.006501791
977.599983	305.823755	6.90202E-06	0.006747411
977.639983	312.778447	7.05897E-06	0.006901135
977.679983	301.321976	6.80042E-06	0.006648632
977.719983	304.567985	6.87368E-06	0.00672053
977.759983	303.931518	6.85931E-06	0.00670676
977.799983	296.409189	6.68954E-06	0.006541035
977.839983	319.145975	7.20268E-06	0.007043069
977.879983	318.940769	7.19805E-06	0.007038828
977.919983	328.98571	7.42475E-06	0.007260811
977.959983	342.250695	7.72412E-06	0.007553882
977.999983	353.218276	7.97164E-06	0.007796268

978.039983	402.895046	9.09278E-06	0.008893102
978.079983	481.334183	1.0863E-05	0.010624924
978.119983	594.551936	1.34182E-05	0.013124618
978.159983	674.296666	1.52179E-05	0.014885577
978.199983	713.030177	1.60921E-05	0.01574129
978.239983	714.83147	1.61328E-05	0.015781702
978.279983	663.943402	1.49843E-05	0.014658819
978.319983	595.815834	1.34467E-05	0.013155208
978.359983	534.124448	1.20544E-05	0.011793586
978.399983	448.948253	1.01321E-05	0.009913282
978.439983	368.827211	8.32392E-06	0.008144453
978.479983	331.828481	7.48891E-06	0.007327745
978.519983	309.723433	6.99003E-06	0.006839881
978.559983	304.841684	6.87985E-06	0.006732348
978.599983	302.270301	6.82182E-06	0.006675833
978.639983	300.685599	6.78606E-06	0.006641105
978.679983	314.264251	7.09251E-06	0.006941294
978.719983	346.949958	7.83018E-06	0.007663551
978.759983	365.852932	8.25679E-06	0.008081417
978.799983	369.089779	8.32984E-06	0.008153249
978.839983	369.480194	8.33865E-06	0.008162207
978.879983	343.934292	7.76212E-06	0.007598182
978.919983	320.089687	7.22398E-06	0.007071697
978.959983	344.123072	7.76638E-06	0.007602974
978.999983	432.42362	9.7592E-06	0.009554255
979.039983	539.116377	1.21671E-05	0.011912083
979.079983	660.491216	1.49064E-05	0.014594525
979.119983	759.304722	1.71364E-05	0.016778641
979.159983	846.42839	1.91027E-05	0.018704609
979.199983	895.281345	2.02053E-05	0.019784983
979.239983	878.959456	1.98369E-05	0.019425076
979.279983	790.75927	1.78463E-05	0.017476559
979.319983	695.330192	1.56926E-05	0.01536811
979.359983	649.163663	1.46507E-05	0.014348329
979.399983	564.020608	1.27292E-05	0.012466939
979.439983	442.852736	9.99457E-06	0.009789081
979.479983	360.977774	8.14677E-06	0.007979594
979.519983	328.279247	7.40881E-06	0.007257073
979.559983	317.979285	7.17635E-06	0.007029665
979.599983	321.571391	7.25742E-06	0.007109367
979.639983	331.159449	7.47381E-06	0.007321641
979.679983	315.641756	7.12359E-06	0.006978843
979.719983	341.971984	7.71783E-06	0.007561314
979.759983	358.693459	8.09521E-06	0.007931365
979.799983	328.174592	7.40644E-06	0.007256833
979.839983	319.315227	7.2065E-06	0.007061217

979.879983	305.817193	6.90187E-06	0.006763002
979.919983	305.559957	6.89606E-06	0.00675759
979.959983	308.20276	6.95571E-06	0.006816315
979.999983	344.667934	7.77868E-06	0.007623102
980.039983	433.03966	9.7731E-06	0.009578031
980.079983	521.787367	1.1776E-05	0.011541436
980.119983	604.502265	1.36428E-05	0.013371556
980.159983	661.82131	1.49364E-05	0.014640047
980.199983	670.262051	1.51269E-05	0.014827368
980.239983	637.130112	1.43791E-05	0.014095007
980.279983	605.648139	1.36686E-05	0.013399089
980.319983	574.49152	1.29655E-05	0.012710313
980.359983	512.025872	1.15557E-05	0.011328756
980.399983	418.098913	9.43591E-06	0.009250966
980.439983	342.490673	7.72954E-06	0.007578348
980.479983	337.364644	7.61385E-06	0.007465228
980.519983	363.949294	8.21383E-06	0.008053823
980.559983	404.476725	9.12848E-06	0.008951019
980.599983	428.438559	9.66926E-06	0.009481678
980.639983	408.988731	9.23031E-06	0.009051607
980.679983	346.58321	7.8219E-06	0.007670781
980.719983	309.731991	6.99022E-06	0.006855448
980.759983	315.457817	7.11944E-06	0.006982465
980.799983	317.057413	7.15554E-06	0.007018158
980.839983	313.704253	7.07987E-06	0.006944218
980.879983	330.615668	7.46154E-06	0.00731887
980.919983	385.261699	8.69482E-06	0.008528923
980.959983	456.490806	1.03024E-05	0.010106205
980.999983	537.00815	1.21195E-05	0.011889255
981.039983	616.257713	1.39081E-05	0.013644381
981.079983	725.982406	1.63844E-05	0.016074418
981.119983	849.753997	1.91778E-05	0.018815688
981.159983	935.242264	2.11071E-05	0.020709457
981.199983	962.663591	2.1726E-05	0.021317528
981.239983	939.716496	2.12081E-05	0.020810228
981.279983	876.246963	1.97757E-05	0.019405473
981.319983	752.640915	1.69861E-05	0.016668757
981.359983	626.905979	1.41484E-05	0.013884668
981.399983	588.321311	1.32776E-05	0.013030629
981.439983	623.137363	1.40633E-05	0.013802326
981.479983	674.09119	1.52133E-05	0.014931549
981.519983	670.515858	1.51326E-05	0.014852958
981.559983	607.708238	1.37151E-05	0.013462221
981.599983	520.951593	1.17572E-05	0.01154082
981.639983	449.309919	1.01403E-05	0.009954123
981.679983	420.6348	9.49314E-06	0.009319227

981.719983	405.138236	9.14341E-06	0.008976264
981.759983	386.227462	8.71662E-06	0.008557625
981.799983	330.498766	7.4589E-06	0.007323145
981.839983	291.286594	6.57393E-06	0.00645455
981.879983	298.292436	6.73204E-06	0.00661006
981.919983	314.601714	7.10012E-06	0.006971752
981.959983	327.25468	7.38568E-06	0.007252444
981.999983	383.132362	8.64676E-06	0.008491122
982.039983	468.784725	1.05798E-05	0.010389805
982.079983	538.499478	1.21532E-05	0.011935398
982.119983	634.951954	1.433E-05	0.014073761
982.159983	744.601529	1.68046E-05	0.016504825
982.199983	804.690532	1.81607E-05	0.017837483
982.239983	803.364977	1.81308E-05	0.017808825
982.279983	761.555246	1.71872E-05	0.016882683
982.319983	686.963354	1.55038E-05	0.015229698
982.359983	608.266939	1.37277E-05	0.01348558
982.399983	558.220469	1.25983E-05	0.012376529
982.439983	513.97741	1.15998E-05	0.011396062
982.479983	460.219482	1.03865E-05	0.010204541
982.519983	479.5794	1.08234E-05	0.010634245
982.559983	472.758579	1.06695E-05	0.010483426
982.599983	450.207097	1.01605E-05	0.009983753
982.639983	439.229835	9.91281E-06	0.009740719
982.679983	467.540877	1.05517E-05	0.01036899
982.719983	506.411522	1.1429E-05	0.011231509
982.759983	528.316113	1.19234E-05	0.0117178
982.799983	495.389999	1.11803E-05	0.010987962
982.839983	459.940254	1.03802E-05	0.010202086
982.879983	445.612442	1.00569E-05	0.009884679
982.919983	411.036992	9.27653E-06	0.009118089
982.959982	398.740361	8.99901E-06	0.008845671
982.999982	480.406922	1.08421E-05	0.010657799
983.039982	636.041226	1.43546E-05	0.014111111
983.079982	753.929213	1.70151E-05	0.016727236
983.119982	825.894705	1.86393E-05	0.018324662
983.159982	913.338536	2.06128E-05	0.020265659
983.199982	981.239399	2.21452E-05	0.021773167
983.239982	985.272952	2.22362E-05	0.021863558
983.279982	894.050493	2.01775E-05	0.019840107
983.319982	760.032267	1.71529E-05	0.016866759
983.359982	637.817703	1.43947E-05	0.01415513
983.399982	540.312204	1.21941E-05	0.011991672
983.439982	459.423832	1.03686E-05	0.010196852
983.479982	429.096889	9.68412E-06	0.009524137
983.519982	411.918226	9.29642E-06	0.009143215

983.559982	356.256696	8.04022E-06	0.007908036
983.599982	353.290466	7.97327E-06	0.007842512
983.639982	379.756607	8.57058E-06	0.008430363
983.679982	356.497657	8.04566E-06	0.00791435
983.719982	343.456546	7.75134E-06	0.007625144
983.759982	361.808003	8.1655E-06	0.008032895
983.799982	346.175314	7.81269E-06	0.007686129
983.839982	326.396761	7.36632E-06	0.00724728
983.879982	330.268198	7.45369E-06	0.00733354
983.919982	325.337985	7.34243E-06	0.007224359
983.959982	318.843356	7.19585E-06	0.007080429
983.999982	351.772951	7.93903E-06	0.007812001
984.039982	413.384798	9.32952E-06	0.00918062
984.079982	503.207222	1.13567E-05	0.011175887
984.119982	608.895245	1.37419E-05	0.013523696
984.159982	688.149008	1.55306E-05	0.01528456
984.199982	731.563768	1.65104E-05	0.016249511
984.239982	723.679611	1.63324E-05	0.016075041
984.279982	684.660881	1.54518E-05	0.015208939
984.319982	632.680298	1.42787E-05	0.014054823
984.359982	554.263881	1.2509E-05	0.012313323
984.399982	483.287208	1.09071E-05	0.010736968
984.439982	443.034467	9.99867E-06	0.009843091
984.479982	417.07137	9.41272E-06	0.009266634
984.519982	364.754854	8.23201E-06	0.008104577
984.559982	309.714123	6.98982E-06	0.006881893
984.599982	306.040923	6.90692E-06	0.006800551
984.639982	352.606216	7.95783E-06	0.007835599
984.679982	384.785579	8.68407E-06	0.008551034
984.719982	378.572399	8.54385E-06	0.008413302
984.759982	367.557885	8.29527E-06	0.008168849
984.799982	374.762516	8.45787E-06	0.008329308
984.839982	376.895341	8.506E-06	0.008377052
984.879982	364.951147	8.23644E-06	0.008111904
984.919982	353.142215	7.96993E-06	0.007849741
984.959982	371.619379	8.38693E-06	0.008260792
984.999982	425.80007	9.60971E-06	0.009465569
985.039982	511.603225	1.15462E-05	0.011373441
985.079982	622.069063	1.40392E-05	0.013829767
985.119982	725.397914	1.63712E-05	0.016127616
985.159982	782.342637	1.76564E-05	0.017394363
985.199982	820.205972	1.85109E-05	0.018236945
985.239982	834.03976	1.88231E-05	0.018545286
985.279982	792.398207	1.78833E-05	0.017620081
985.319982	716.173632	1.6163E-05	0.015925768
985.359982	601.095947	1.35659E-05	0.013367293

985.399982	508.426524	1.14745E-05	0.011306951
985.439982	415.454852	9.37624E-06	0.009239719
985.479982	337.560452	7.61827E-06	0.007507652
985.519982	318.71754	7.19301E-06	0.007088856
985.559982	308.052023	6.95231E-06	0.006851914
985.599982	315.701268	7.12494E-06	0.007022339
985.639982	329.625666	7.43919E-06	0.007332365
985.679982	356.60244	8.04802E-06	0.007932773
985.719982	360.453428	8.13493E-06	0.008018765
985.759982	347.848764	7.85046E-06	0.007738671
985.799982	324.477217	7.323E-06	0.007219012
985.839982	300.468423	6.78115E-06	0.006685133
985.879982	274.297481	6.19051E-06	0.006103102
985.919982	267.182872	6.02995E-06	0.005945044
985.959982	295.737566	6.67438E-06	0.006580676
985.999982	343.015713	7.74139E-06	0.007633007
986.039982	407.055403	9.18667E-06	0.009058427
986.079982	501.348141	1.13147E-05	0.011157228
986.119982	560.11269	1.2641E-05	0.012465506
986.159982	595.424665	1.34379E-05	0.013251924
986.199982	641.257727	1.44723E-05	0.014272576
986.239982	693.274838	1.56462E-05	0.015430955
986.279982	670.411094	1.51302E-05	0.014922657
986.319982	602.837957	1.36052E-05	0.013419093
986.359982	539.730003	1.2181E-05	0.012014806
986.399982	460.886499	1.04016E-05	0.010260105
986.439982	384.062774	8.66776E-06	0.008550227
986.479982	336.129035	7.58596E-06	0.007483402
986.519982	311.624924	7.03294E-06	0.006938136
986.559982	307.609542	6.94232E-06	0.006849014
986.599982	306.989845	6.92833E-06	0.006835493
986.639982	313.33529	7.07154E-06	0.006977065
986.679982	336.918723	7.60379E-06	0.007502504
986.719982	366.8721	8.27979E-06	0.008169836
986.759982	370.845499	8.36947E-06	0.008258654
986.799982	357.769554	8.07436E-06	0.007967779
986.839982	361.709835	8.16329E-06	0.008055858
986.879982	352.745998	7.96099E-06	0.007856538
986.919982	367.257107	8.28848E-06	0.008180068
986.959982	401.606959	9.06371E-06	0.008945519
986.999982	451.246222	1.0184E-05	0.010051607
987.039982	528.082595	1.19181E-05	0.01176363
987.079982	606.819886	1.36951E-05	0.013518139
987.119982	673.782122	1.52063E-05	0.015010466
987.159982	719.639887	1.62413E-05	0.016032732
987.199982	754.161957	1.70204E-05	0.016802524

987.239982	778.47064	1.7569E-05	0.017344817
987.279982	727.822028	1.64259E-05	0.016216991
987.319982	627.294619	1.41572E-05	0.013977653
987.359982	521.57035	1.17711E-05	0.011622329
987.399982	415.310348	9.37298E-06	0.009254876
987.439982	357.139341	8.06014E-06	0.007958902
987.479982	340.714949	7.68946E-06	0.00759319
987.519982	333.058496	7.51667E-06	0.007422858
987.559982	326.776631	7.37489E-06	0.00728315
987.599982	325.264002	7.34076E-06	0.00724973
987.639982	307.877538	6.94837E-06	0.006862485
987.679982	303.547515	6.85064E-06	0.006766245
987.719982	314.991161	7.10891E-06	0.007021614
987.759982	327.721115	7.39621E-06	0.007305679
987.799982	336.214726	7.5879E-06	0.007495326
987.839982	323.860822	7.30909E-06	0.007220209
987.879982	288.750279	6.51669E-06	0.006437709
987.919982	281.2954	6.34845E-06	0.006271756
987.959982	313.479367	7.07479E-06	0.006989612
987.999982	363.872266	8.21209E-06	0.008113545
988.039982	426.963952	9.63598E-06	0.009520735
988.079982	510.188053	1.15142E-05	0.011376984
988.119982	601.69907	1.35795E-05	0.013418185
988.159982	636.508936	1.43651E-05	0.014195037
988.199982	638.689971	1.44143E-05	0.014244254
988.239982	605.233493	1.36593E-05	0.013498644
988.279982	591.910793	1.33586E-05	0.013202039
988.319982	577.287766	1.30286E-05	0.012876407
988.359982	497.202147	1.12212E-05	0.011090545
988.399982	402.633991	9.08689E-06	0.00898148
988.439982	344.003664	7.76368E-06	0.007673935
988.479982	321.344699	7.2523E-06	0.007168756
988.519982	334.290626	7.54447E-06	0.007457863
988.559982	355.116216	8.01448E-06	0.007922793
988.599982	362.135302	8.17289E-06	0.008079718
988.639982	362.529192	8.18178E-06	0.008088834
988.679982	357.741307	8.07372E-06	0.007982328
988.719982	341.240636	7.70133E-06	0.007614455
988.759982	312.585458	7.05462E-06	0.006975324
988.799982	295.024778	6.6583E-06	0.006583725
988.839982	265.672955	5.99587E-06	0.005928955
988.879982	265.378088	5.98921E-06	0.005922614
988.919982	291.011238	6.56772E-06	0.006494948
988.959982	338.297783	7.63491E-06	0.00755062
988.999982	394.60467	8.90568E-06	0.008807715
989.039982	473.791287	1.06928E-05	0.010575616

989.079982	554.31683	1.25102E-05	0.012373548
989.119982	667.930625	1.50743E-05	0.014910256
989.159982	768.735201	1.73493E-05	0.017161216
989.199982	785.26338	1.77223E-05	0.0175309
989.239982	758.301519	1.71138E-05	0.016929664
989.279982	698.229063	1.57581E-05	0.015589131
989.319982	627.147531	1.41538E-05	0.014002683
989.359982	549.455744	1.24005E-05	0.01226851
989.399982	482.063721	1.08795E-05	0.010764184
989.439982	404.367006	9.126E-06	0.009029629
989.479982	338.471245	7.63882E-06	0.007558464
989.519982	295.926368	6.67865E-06	0.006608654
989.559982	298.660992	6.74036E-06	0.006669993
989.599982	331.927046	7.49113E-06	0.007413223
989.639982	343.447624	7.75113E-06	0.007670833
989.679982	351.245701	7.92713E-06	0.007845318
989.719982	341.240114	7.70131E-06	0.007622144
989.759982	325.395831	7.34373E-06	0.007268531
989.799982	311.250702	7.02449E-06	0.006952845
989.839982	284.376481	6.41798E-06	0.006352774
989.879982	264.582587	5.97126E-06	0.005910831
989.919982	246.767052	5.56919E-06	0.005513051
989.959982	272.414694	6.14802E-06	0.006086294
989.999982	336.184604	7.58722E-06	0.007511346
990.039982	391.165402	8.82806E-06	0.008740131
990.079982	440.025171	9.93076E-06	0.009832242
990.119982	519.59474	1.17265E-05	0.011610671
990.159982	617.828787	1.39435E-05	0.013806331
990.199982	669.623832	1.51125E-05	0.014964375
990.239982	674.441741	1.52212E-05	0.015072651
990.279982	666.967549	1.50525E-05	0.014906218
990.319982	622.420912	1.40472E-05	0.013911196
990.359982	550.092002	1.24148E-05	0.012295131
990.399982	496.184509	1.11982E-05	0.01109069
990.439982	460.897705	1.04018E-05	0.010302378
990.479982	425.775089	9.60915E-06	0.009517671
990.519982	392.249303	8.85252E-06	0.008768598
990.559982	384.161556	8.66999E-06	0.008588146
990.599982	424.52892	9.58103E-06	0.009490965
990.639982	481.151572	1.08589E-05	0.010757281
990.679982	498.474794	1.12499E-05	0.011145033
990.719982	527.12778	1.18965E-05	0.01178614
990.759982	594.624079	1.34198E-05	0.013295838
990.799982	651.203313	1.46968E-05	0.014561541
990.839982	694.779645	1.56802E-05	0.015536578
990.879982	792.668503	1.78894E-05	0.017726272

990.919982	919.658598	2.07554E-05	0.020566954
990.959982	1084.567558	2.44772E-05	0.024255905
990.999982	1349.235004	3.04504E-05	0.030176301
991.039982	1661.165194	3.74902E-05	0.037154272
991.079982	2001.039133	4.51607E-05	0.044757832
991.119982	2436.644888	5.49917E-05	0.054503354
991.159982	2980.748047	6.72713E-05	0.066676652
991.199982	3479.390661	7.8525E-05	0.077833979
991.239982	3702.813352	8.35673E-05	0.082835288
991.279982	3587.115714	8.09562E-05	0.080250266
991.319982	3106.364546	7.01063E-05	0.069497797
991.359982	2333.129846	5.26555E-05	0.052200546
991.399982	1598.280789	3.6071E-05	0.03576076
991.439982	1009.654779	2.27865E-05	0.022591449
991.479982	618.985416	1.39696E-05	0.013850617
991.519982	456.475342	1.0302E-05	0.010214651
991.559982	399.488479	9.0159E-06	0.008939804
991.599982	391.610193	8.8381E-06	0.008763856
991.639982	432.108597	9.75209E-06	0.009670562
991.679982	422.584487	9.53714E-06	0.009457794
991.719982	384.611791	8.68015E-06	0.008608281
991.759982	360.367444	8.13299E-06	0.008065975
991.799982	383.194993	8.64818E-06	0.008577262
991.839982	433.665852	9.78723E-06	0.00970737
991.879982	450.997301	1.01784E-05	0.010095732
991.919982	477.297884	1.07719E-05	0.010684911
991.959982	554.054734	1.25042E-05	0.012403709
991.999982	645.570087	1.45696E-05	0.01445306
992.039982	791.306453	1.78587E-05	0.017716529
992.079982	1008.653502	2.27639E-05	0.022583614
992.119982	1250.891897	2.82309E-05	0.028008428
992.159982	1434.895776	3.23836E-05	0.032129711
992.199982	1596.866829	3.60391E-05	0.035757955
992.239982	1723.59146	3.88991E-05	0.038597201
992.279982	1699.262488	3.835E-05	0.038053925
992.319982	1477.445271	3.33439E-05	0.0330878
992.359982	1155.257641	2.60726E-05	0.025873361
992.399982	850.603037	1.91969E-05	0.019051029
992.439982	596.472998	1.34616E-05	0.013359795
992.479982	425.965488	9.61345E-06	0.009541154
992.519982	337.26653	7.61164E-06	0.007554701
992.559982	314.66251	7.10149E-06	0.007048659
992.599982	328.816399	7.42093E-06	0.007366013
992.639982	372.270002	8.40162E-06	0.008339779
992.679982	404.676335	9.13298E-06	0.009066128
992.719982	405.444924	9.15033E-06	0.009083713

992.759982	373.300505	8.42487E-06	0.008363876
992.799982	372.816317	8.41394E-06	0.008353364
992.839982	380.178116	8.58009E-06	0.008518657
992.879982	372.11217	8.39805E-06	0.008338259
992.919982	376.602749	8.4994E-06	0.008439224
992.959982	420.200554	9.48334E-06	0.009416578
992.999982	468.365719	1.05704E-05	0.010496369
993.039982	584.708662	1.31961E-05	0.013104215
993.079982	717.799481	1.61997E-05	0.016087632
993.119982	879.74117	1.98545E-05	0.019717933
993.159982	1014.004559	2.28847E-05	0.022728139
993.199982	1135.389659	2.56242E-05	0.025449918
993.239982	1184.10153	2.67235E-05	0.026542871
993.279982	1123.927773	2.53655E-05	0.025195028
993.319982	1010.058851	2.27956E-05	0.022643346
993.359982	829.67031	1.87245E-05	0.018600172
993.399982	643.23205	1.45169E-05	0.01442104
993.439982	493.282881	1.11327E-05	0.011059677
993.479982	399.920245	9.02564E-06	0.008966796
993.519982	339.80023	7.66882E-06	0.007619124
993.559982	308.603706	6.96476E-06	0.006919903
993.599982	303.518216	6.84998E-06	0.006806143
993.639982	305.065113	6.88489E-06	0.006841107
993.679982	317.973866	7.17623E-06	0.007130873
993.719982	316.242956	7.13716E-06	0.007092342
993.759982	320.261274	7.22785E-06	0.007182749
993.799982	316.426071	7.1413E-06	0.00709702
993.839982	302.302174	6.82254E-06	0.006780512
993.879982	310.852848	7.01552E-06	0.006972581
993.919982	327.654571	7.39471E-06	0.007349747
993.959982	345.999895	7.80874E-06	0.007761571
993.999982	376.296853	8.4925E-06	0.008441541
994.039982	404.032447	9.11845E-06	0.009064103
994.079982	495.594139	1.11849E-05	0.011118655
994.119982	624.122408	1.40856E-05	0.01400275
994.159982	750.352858	1.69344E-05	0.016835522
994.199982	803.003902	1.81227E-05	0.018017568
994.239982	817.139809	1.84417E-05	0.018335483
994.279982	768.011046	1.73329E-05	0.017233795
994.319982	688.973851	1.55492E-05	0.015460861
994.359982	587.479048	1.32586E-05	0.013183805
994.399982	490.530006	1.10706E-05	0.011008584
994.439982	389.859165	8.79858E-06	0.008749658
994.479982	308.770579	6.96852E-06	0.006930056
994.519982	280.215522	6.32407E-06	0.006289418
994.559982	292.007455	6.5902E-06	0.006554351

994.599982	322.369288	7.27543E-06	0.007236138
994.639982	344.099197	7.76584E-06	0.007724215
994.679982	353.994116	7.98915E-06	0.007946652
994.719982	356.43647	8.04427E-06	0.008001801
994.759982	363.720869	8.20867E-06	0.00816566
994.799982	381.739293	8.61532E-06	0.008570524
994.839982	366.679516	8.27545E-06	0.008232744
994.879982	328.124669	7.40532E-06	0.007367401
994.919982	322.929188	7.28806E-06	0.007251038
994.959982	358.959223	8.10121E-06	0.008060379
994.999982	426.049385	9.61534E-06	0.009567264
995.039982	481.45948	1.08659E-05	0.010811975
995.079982	577.554317	1.30346E-05	0.012970466
995.119982	701.76634	1.58379E-05	0.0157606
995.159982	814.437781	1.83807E-05	0.018291763
995.199982	871.718992	1.96735E-05	0.01957905
995.239982	871.26064	1.96631E-05	0.019569542
995.279982	825.497536	1.86303E-05	0.018542394
995.319982	749.490533	1.6915E-05	0.016835795
995.359982	678.625442	1.53156E-05	0.015244566
995.399982	578.756643	1.30617E-05	0.013001647
995.439982	447.684538	1.01036E-05	0.010057544
995.479982	359.442463	8.11212E-06	0.008075449
995.519982	330.85715	7.46699E-06	0.007433533
995.559982	305.140111	6.88659E-06	0.006856011
995.599982	286.863559	6.47411E-06	0.006445625
995.639982	304.983504	6.88305E-06	0.006853043
995.679982	298.055955	6.72671E-06	0.006697648
995.719982	272.989618	6.161E-06	0.006134626
995.759982	282.530378	6.37632E-06	0.006349281
995.799982	295.839512	6.67669E-06	0.006648644
995.839982	282.303742	6.3712E-06	0.006344698
995.879982	253.399109	5.71886E-06	0.005695303
995.919982	241.835253	5.45788E-06	0.005435617
995.959982	271.527956	6.12801E-06	0.00610325
995.999982	339.120122	7.65347E-06	0.007622855
996.039982	410.439619	9.26305E-06	0.009226369
996.079982	485.820849	1.09643E-05	0.01092132
996.119982	555.887276	1.25456E-05	0.012496924
996.159982	587.697254	1.32635E-05	0.013212576
996.199982	592.650258	1.33753E-05	0.013324464
996.239982	621.254628	1.40209E-05	0.013968133
996.279982	631.736406	1.42574E-05	0.014204373
996.319982	582.843176	1.3154E-05	0.013105552
996.359982	509.12613	1.14903E-05	0.011448443
996.399982	430.004777	9.70461E-06	0.009669672

996.439982	364.699051	8.23075E-06	0.008201448
996.479982	326.54926	7.36976E-06	0.00734382
996.519982	333.386579	7.52407E-06	0.007497887
996.559982	366.185318	8.26429E-06	0.008235863
996.599982	360.221173	8.12969E-06	0.008102049
996.639982	360.279719	8.13101E-06	0.008103691
996.679982	369.59665	8.34128E-06	0.008313588
996.719982	381.78023	8.61625E-06	0.008587987
996.759982	381.785353	8.61636E-06	0.008588446
996.799982	386.049476	8.7126E-06	0.008684718
996.839982	389.982221	8.80136E-06	0.008773543
996.879982	379.044319	8.5545E-06	0.008527812
996.919982	354.357695	7.99736E-06	0.007972728
996.959982	375.290379	8.46978E-06	0.008444033
996.999982	467.729822	1.0556E-05	0.010524342
997.039982	571.646664	1.29013E-05	0.012863081
997.079982	669.274983	1.51046E-05	0.015060498
997.119982	743.789937	1.67863E-05	0.016737958
997.159982	810.311062	1.82876E-05	0.018235655
997.199982	868.27633	1.95958E-05	0.019540919
997.239982	865.451015	1.9532E-05	0.019478115
997.279982	766.521579	1.72993E-05	0.01725227
997.319982	654.409324	1.47691E-05	0.014729525
997.359982	579.316553	1.30744E-05	0.013039851
997.399982	513.597365	1.15912E-05	0.01156104
997.439982	445.147162	1.00464E-05	0.010020633
997.479982	380.886655	8.59608E-06	0.008574419
997.519982	350.095972	7.90118E-06	0.007881583
997.559982	335.796257	7.57845E-06	0.007559962
997.599982	323.1319	7.29264E-06	0.007275134
997.639982	323.580976	7.30277E-06	0.007285537
997.679982	308.349623	6.95902E-06	0.006942876
997.719982	279.012942	6.29693E-06	0.006282576
997.759982	267.955759	6.04739E-06	0.006033842
997.799982	293.337574	6.62022E-06	0.006605656
997.839982	325.775372	7.3523E-06	0.007336415
997.879982	310.967774	7.01811E-06	0.007003231
997.919982	277.89422	6.27169E-06	0.00625864
997.959982	306.134932	6.90904E-06	0.006894944
997.999982	363.548871	8.20479E-06	0.008188382
998.039982	416.203962	9.39314E-06	0.009374733
998.079982	460.269883	1.03877E-05	0.010367706
998.119982	517.527632	1.16799E-05	0.011657919
998.159982	630.098371	1.42204E-05	0.014194277
998.199982	709.49901	1.60124E-05	0.015983582
998.239982	753.499156	1.70054E-05	0.016975497

998.279982	734.274301	1.65715E-05	0.016543045
998.319982	665.770774	1.50255E-05	0.015000276
998.359982	565.249366	1.27569E-05	0.012735969
998.399982	482.26171	1.0884E-05	0.010866561
998.439982	397.65315	8.97448E-06	0.008960477
998.479982	351.067395	7.9231E-06	0.007911059
998.519982	336.690921	7.59865E-06	0.007587399
998.559982	305.660949	6.89834E-06	0.006888408
998.599982	301.132009	6.79613E-06	0.006786615
998.639982	331.832886	7.48901E-06	0.007478821
998.679982	377.108079	8.5108E-06	0.00849957
998.719982	395.837261	8.9335E-06	0.008922061
998.759982	398.385739	8.99101E-06	0.008979862
998.799982	392.884166	8.86685E-06	0.008856208
998.839982	387.15981	8.73766E-06	0.008727522
998.879982	392.89156	8.86702E-06	0.008857084
998.919982	369.765213	8.34509E-06	0.008336073
998.959982	370.92767	8.37132E-06	0.008362615
998.999982	424.01748	9.56948E-06	0.009559914
999.039982	509.079284	1.14892E-05	0.011478181
999.079982	611.634351	1.38037E-05	0.013791036
999.119982	746.741318	1.68529E-05	0.016838081
999.159982	863.088303	1.94787E-05	0.019462338
999.199982	906.049554	2.04483E-05	0.020431917
999.239982	862.460362	1.94645E-05	0.019449736
999.279982	827.845847	1.86833E-05	0.018669875
999.319982	750.675716	1.69417E-05	0.016930185
999.359982	596.469351	1.34615E-05	0.013452867
999.399982	479.720225	1.08266E-05	0.010820121
999.439982	393.822668	8.88803E-06	0.008883052
999.479982	335.017466	7.56088E-06	0.007556946
999.519982	315.181009	7.1132E-06	0.007109782
999.559982	318.981888	7.19898E-06	0.007195809
999.599982	308.163282	6.95482E-06	0.006952034
999.639982	307.571341	6.94146E-06	0.006938958
999.679982	328.294412	7.40915E-06	0.007406777
999.719982	328.971144	7.42442E-06	0.007422342
999.759982	329.507836	7.43653E-06	0.007434748
999.799982	300.281297	6.77693E-06	0.006775575
999.839982	290.536662	6.55701E-06	0.006555959
999.879982	298.151007	6.72885E-06	0.006728045
999.919982	296.087606	6.68228E-06	0.00668175
999.959982	335.151177	7.5639E-06	0.007563593
999.999982	376.995642	8.50827E-06	0.008508266
1000.039982	414.823414	9.36199E-06	0.009362361
1000.079982	493.475434	1.11371E-05	0.011137944

1000.119982	620.40469	1.40017E-05	0.01400335
1000.159982	683.230058	1.54196E-05	0.015422018
1000.199982	684.767205	1.54542E-05	0.015457333
1000.239982	657.756546	1.48446E-05	0.014848212
1000.279982	615.614809	1.38936E-05	0.013897459
1000.319982	590.748267	1.33324E-05	0.013336632
1000.359982	551.944624	1.24566E-05	0.012461106
1000.399982	467.156732	1.05431E-05	0.010547294
1000.439982	376.049059	8.4869E-06	0.008490638
1000.479982	340.664926	7.68833E-06	0.007692023
1000.519982	335.106359	7.56288E-06	0.007566816
1000.559982	333.88458	7.53531E-06	0.00753953
1000.599982	358.737315	8.0962E-06	0.008101059
1000.639982	373.928564	8.43905E-06	0.008444448
1000.679982	363.378008	8.20094E-06	0.008206512
1000.719982	366.262509	8.26603E-06	0.008271986
1000.759982	350.221857	7.90402E-06	0.007910026
1000.799982	347.31849	7.83849E-06	0.007844765
1000.839982	332.294926	7.49943E-06	0.007505733
1000.879982	322.467193	7.27764E-06	0.00728404
1000.919982	340.861034	7.69276E-06	0.007699836
1000.959982	396.271815	8.9433E-06	0.008951888
1000.999982	452.657532	1.02158E-05	0.010226066
1001.039982	540.808894	1.22053E-05	0.012217997
1001.079982	649.784846	1.46647E-05	0.014680577
1001.119982	705.130983	1.59138E-05	0.015931647
1001.159982	769.47805	1.7366E-05	0.017386192
1001.199982	854.840382	1.92926E-05	0.019315707
1001.239982	885.092198	1.99753E-05	0.020000067
1001.279982	858.737277	1.93805E-05	0.019405311
1001.319982	762.571613	1.72102E-05	0.017232896
1001.359982	656.053596	1.48062E-05	0.014826352
1001.399982	569.002747	1.28416E-05	0.012859577
1001.439982	479.474917	1.08211E-05	0.010836663
1001.479982	387.124902	8.73687E-06	0.0087498
1001.519982	322.065049	7.26856E-06	0.007279608
1001.559982	319.409815	7.20863E-06	0.00721988
1001.599982	320.566527	7.23474E-06	0.007246315
1001.639982	316.643439	7.1462E-06	0.007157921
1001.679982	362.602463	8.18343E-06	0.008197181
1001.719982	422.23186	9.52918E-06	0.009545575
1001.759982	440.30834	9.93715E-06	0.009954635
1001.799982	415.931736	9.387E-06	0.009403896
1001.839982	375.931637	8.48425E-06	0.008499864
1001.879982	336.515662	7.59469E-06	0.007608968
1001.919982	308.76551	6.96841E-06	0.006981787

1001.959982	307.026314	6.92916E-06	0.006942737
1001.999982	328.366477	7.41077E-06	0.007425595
1002.039982	370.423975	8.35995E-06	0.008377007
1002.079982	455.698539	1.02845E-05	0.010305873
1002.119982	554.08609	1.2505E-05	0.012531462
1002.159982	607.355252	1.37072E-05	0.013736769
1002.199982	670.739683	1.51377E-05	0.015170963
1002.239982	714.931427	1.6135E-05	0.016171149
1002.279982	712.847333	1.6088E-05	0.016124652
1002.319982	645.381086	1.45654E-05	0.014599144
1002.359982	539.317426	1.21716E-05	0.012200368
1002.399982	429.70272	9.69779E-06	0.009721066
1002.439982	358.64521	8.09412E-06	0.008113872
1002.479982	314.225624	7.09163E-06	0.007109222
1002.519982	331.9898	7.49255E-06	0.007511428
1002.559982	355.393479	8.02074E-06	0.008041269
1002.599982	356.618388	8.04838E-06	0.008069306
1002.639982	368.196499	8.30968E-06	0.00833162
1002.679982	357.027849	8.05762E-06	0.008079216
1002.719982	359.262297	8.10805E-06	0.008130103
1002.759982	391.099282	8.82657E-06	0.008850927
1002.799982	384.855741	8.68566E-06	0.008709978
1002.839982	386.217079	8.71638E-06	0.008741136
1002.879982	408.241054	9.21343E-06	0.009239966
1002.919982	404.800695	9.13579E-06	0.009162464
1002.959982	377.87589	8.52813E-06	0.008553376
1002.999982	380.701978	8.59191E-06	0.008617689
1003.039982	449.425236	1.01429E-05	0.010173736
1003.079982	548.605987	1.23813E-05	0.012419407
1003.119982	629.003849	1.41957E-05	0.014240031
1003.159982	735.888512	1.6608E-05	0.01666046
1003.199982	818.022821	1.84616E-05	0.018520713
1003.239982	798.335882	1.80173E-05	0.018075705
1003.279982	750.301525	1.69333E-05	0.016988801
1003.319982	678.224842	1.53066E-05	0.015357408
1003.359982	567.303751	1.28033E-05	0.012846274
1003.399982	466.628621	1.05312E-05	0.010566964
1003.439982	384.057224	8.66764E-06	0.008697453
1003.479982	347.540636	7.84351E-06	0.007870803
1003.519982	326.429768	7.36707E-06	0.007392997
1003.559982	305.456317	6.89372E-06	0.006918265
1003.599982	315.84508	7.12818E-06	0.007153845
1003.639982	344.15822	7.76717E-06	0.007795444
1003.679982	355.829213	8.03057E-06	0.008060122
1003.719982	339.657563	7.6656E-06	0.007694114
1003.759982	325.19203	7.33913E-06	0.007366726

1003.799982	312.15777	7.04497E-06	0.007071737
1003.839982	289.007161	6.52249E-06	0.006547535
1003.879982	271.001204	6.11612E-06	0.00613985
1003.919982	274.288132	6.1903E-06	0.006214567
1003.959982	296.441187	6.69026E-06	0.006716758
1003.999982	327.298248	7.38667E-06	0.007416212
1004.039982	353.251524	7.97239E-06	0.008004603
1004.079982	417.575179	9.42409E-06	0.00946254
1004.119982	511.016997	1.15329E-05	0.011580457
1004.159982	552.728667	1.24743E-05	0.012526209
1004.199982	585.134076	1.32057E-05	0.013261125
1004.239982	601.719622	1.358E-05	0.013637553
1004.279982	594.465523	1.34163E-05	0.01347368
1004.319982	543.217887	1.22597E-05	0.012312633
1004.359982	476.957294	1.07643E-05	0.010811194
1004.399982	425.287947	9.59816E-06	0.009640388
1004.439982	359.662604	8.11708E-06	0.008153124
1004.479982	315.299494	7.11587E-06	0.007147749
1004.519982	322.970475	7.28899E-06	0.00732194
1004.559982	355.831653	8.03062E-06	0.008067244
1004.599982	350.572209	7.91193E-06	0.007948321
1004.639982	323.820351	7.30817E-06	0.007342084
1004.679982	323.905397	7.31009E-06	0.007344305
1004.719982	328.222601	7.40753E-06	0.00744249
1004.759982	315.388085	7.11787E-06	0.007151751
1004.799982	299.918117	6.76873E-06	0.006801224
1004.839982	300.6148	6.78446E-06	0.006817294
1004.879982	303.032229	6.83902E-06	0.00687239
1004.919982	326.43057	7.36708E-06	0.007403329
1004.959982	360.349536	8.13259E-06	0.008172925
1004.999982	382.538406	8.63336E-06	0.008676526
1005.039982	436.304279	9.84678E-06	0.009896407
1005.079982	515.183893	1.1627E-05	0.011686048
1005.119982	589.989024	1.33152E-05	0.013383404
1005.159982	659.164849	1.48764E-05	0.014953195
1005.199982	726.448779	1.63949E-05	0.01648019
1005.239982	773.184491	1.74497E-05	0.017541133
1005.279982	736.207277	1.66152E-05	0.016702901
1005.319982	654.187277	1.47641E-05	0.01484264
1005.359982	577.412435	1.30314E-05	0.013101242
1005.399982	500.761916	1.13015E-05	0.011362527
1005.439982	437.690933	9.87807E-06	0.009931811
1005.479982	344.143373	7.76684E-06	0.007809399
1005.519982	291.352498	6.57542E-06	0.006611716
1005.559982	291.288197	6.57397E-06	0.00661052
1005.599982	298.366878	6.73372E-06	0.006771434

1005.639982	289.593999	6.53573E-06	0.006572595
1005.679982	282.600372	6.3779E-06	0.006414123
1005.719982	295.142436	6.66095E-06	0.006699054
1005.759982	313.510539	7.0755E-06	0.007116251
1005.799982	329.769213	7.44243E-06	0.007485598
1005.839982	331.407556	7.47941E-06	0.007523087
1005.879982	312.997344	7.06391E-06	0.00710545
1005.919982	302.832822	6.83451E-06	0.006874975
1005.959982	304.198794	6.86534E-06	0.00690626
1005.999982	330.852957	7.46689E-06	0.007511692
1006.039982	359.415879	8.11152E-06	0.008160509
1006.079982	396.699987	8.95297E-06	0.0090074
1006.119982	459.333646	1.03665E-05	0.010429964
1006.159982	529.058011	1.19401E-05	0.012013653
1006.199982	596.470001	1.34615E-05	0.013544958
1006.239982	647.099931	1.46041E-05	0.014695274
1006.279982	678.420204	1.5311E-05	0.015407152
1006.319982	624.839835	1.41018E-05	0.014190888
1006.359982	503.861998	1.13715E-05	0.011443786
1006.399982	404.648061	9.13234E-06	0.00919079
1006.439982	352.875872	7.96392E-06	0.008015204
1006.479982	344.763903	7.78084E-06	0.007831261
1006.519982	314.912062	7.10713E-06	0.007153465
1006.559982	283.810826	6.40522E-06	0.006447233
1006.599982	267.218739	6.03075E-06	0.006070558
1006.639982	294.947113	6.65655E-06	0.006700745
1006.679982	339.557867	7.66335E-06	0.007714539
1006.719982	348.602746	7.86748E-06	0.007920348
1006.759982	330.104603	7.45E-06	0.007500363
1006.799982	306.31513	6.91311E-06	0.006960115
1006.839982	295.362459	6.66592E-06	0.006711514
1006.879982	293.344892	6.62039E-06	0.006665934
1006.919982	311.219492	7.02379E-06	0.007072395
1006.959982	367.771644	8.30009E-06	0.008357862
1006.999982	401.633969	9.06432E-06	0.009127769
1007.039982	449.36374	1.01415E-05	0.01021291
1007.079982	521.573282	1.17712E-05	0.011854522
1007.119982	608.316512	1.37289E-05	0.013826606
1007.159982	696.598911	1.57213E-05	0.015833831
1007.199982	752.528039	1.69835E-05	0.017105791
1007.239982	765.539781	1.72772E-05	0.017402253
1007.279982	751.935831	1.69701E-05	0.017093687
1007.319982	682.202601	1.53964E-05	0.015509064
1007.359982	593.752162	1.34002E-05	0.013498784
1007.399982	493.046981	1.11274E-05	0.011209726
1007.439982	429.49853	9.69318E-06	0.009765301

1007.479982	368.427299	8.31489E-06	0.008377086
1007.519982	312.765668	7.05869E-06	0.007111767
1007.559982	298.672333	6.74062E-06	0.006791577
1007.599982	301.469046	6.80374E-06	0.006855445
1007.639982	310.54593	7.00859E-06	0.007062135
1007.679982	336.694619	7.59873E-06	0.007657087
1007.719982	340.930474	7.69433E-06	0.007753726
1007.759982	321.719299	7.26076E-06	0.0073171
1007.799982	280.492045	6.33031E-06	0.006379691
1007.839982	268.384064	6.05705E-06	0.006104542
1007.879982	298.748536	6.74234E-06	0.006795468
1007.919982	321.127145	7.24739E-06	0.007304792
1007.959982	311.790276	7.03667E-06	0.007092684
1007.999982	322.327408	7.27448E-06	0.007332676
1008.039982	368.651732	8.31996E-06	0.008386848
1008.079982	436.674431	9.85513E-06	0.009934763
1008.119982	502.654375	1.13442E-05	0.011436324
1008.159982	597.472568	1.34841E-05	0.013594154
1008.199982	688.600157	1.55407E-05	0.01566818
1008.239982	758.301943	1.71138E-05	0.017254836
1008.279982	792.771806	1.78918E-05	0.018039899
1008.319982	759.102635	1.71319E-05	0.017274426
1008.359982	625.65174	1.41201E-05	0.014238132
1008.399982	503.155995	1.13555E-05	0.011450916
1008.439982	433.986109	9.79446E-06	0.009877127
1008.479982	381.85628	8.61796E-06	0.008691045
1008.519982	314.102975	7.08887E-06	0.007149264
1008.559982	275.408226	6.21558E-06	0.006268785
1008.599982	277.423651	6.26107E-06	0.00631491
1008.639982	314.116756	7.08918E-06	0.007150428
1008.679982	339.308244	7.65771E-06	0.007724183
1008.719982	323.629581	7.30387E-06	0.007367558
1008.759982	319.837014	7.21828E-06	0.007281508
1008.799982	321.948088	7.26592E-06	0.00732986
1008.839982	332.956239	7.51436E-06	0.007580785
1008.879982	364.908665	8.23548E-06	0.008308611
1008.919982	372.962079	8.41723E-06	0.008492316
1008.959982	374.817665	8.45911E-06	0.008534906
1008.999982	408.56306	9.2207E-06	0.009303685
1009.039982	505.615938	1.1411E-05	0.011514203
1009.079982	598.962196	1.35177E-05	0.013640483
1009.119982	700.90088	1.58184E-05	0.01596262
1009.159982	769.430106	1.7365E-05	0.017524029
1009.199982	818.063281	1.84625E-05	0.018632404
1009.239982	835.485132	1.88557E-05	0.019029962
1009.279982	766.911002	1.73081E-05	0.017468732

1009.319982	682.391777	1.54006E-05	0.015544166
1009.359982	596.750805	1.34678E-05	0.013593893
1009.399982	507.604709	1.14559E-05	0.011563617
1009.439982	436.721919	9.85621E-06	0.009949248
1009.479982	413.535612	9.33292E-06	0.009421399
1009.519982	393.812692	8.8878E-06	0.008972416
1009.559982	371.245287	8.37849E-06	0.008458587
1009.599982	383.877738	8.66359E-06	0.008746756
1009.639982	410.754653	9.27016E-06	0.009359525
1009.679982	407.604591	9.19907E-06	0.009288115
1009.719982	375.843507	8.48226E-06	0.008564712
1009.759982	335.60133	7.57405E-06	0.007647977
1009.799982	309.926836	6.99462E-06	0.007063164
1009.839982	309.964793	6.99547E-06	0.007064309
1009.879982	316.161532	7.13533E-06	0.007205822
1009.919982	316.075086	7.13337E-06	0.007204137
1009.959982	330.464498	7.45812E-06	0.007532406
1009.999982	355.368801	8.02018E-06	0.00810038
1010.039982	411.133587	9.27871E-06	0.009371871
1010.079982	476.701413	1.07585E-05	0.010866932
1010.119982	540.888737	1.22071E-05	0.012330641
1010.159982	642.568248	1.45019E-05	0.014649209
1010.199982	691.921946	1.56157E-05	0.015774995
1010.239982	686.352829	1.549E-05	0.015648645
1010.279982	683.452028	1.54246E-05	0.015583125
1010.319982	606.237111	1.36819E-05	0.013823124
1010.359982	507.213723	1.14471E-05	0.011565699
1010.399982	430.963033	9.72624E-06	0.009827388
1010.439982	387.104183	8.7364E-06	0.00882761
1010.479982	360.284758	8.13113E-06	0.008216339
1010.519982	357.246604	8.06256E-06	0.008147376
1010.559982	364.178693	8.21901E-06	0.008305798
1010.599982	368.807713	8.32348E-06	0.008411705
1010.639982	375.243861	8.46873E-06	0.008558838
1010.679982	357.816834	8.07543E-06	0.008161673
1010.719982	336.641163	7.59752E-06	0.007678967
1010.759982	326.144644	7.36063E-06	0.007439831
1010.799982	314.735857	7.10315E-06	0.007179864
1010.839982	302.91856	6.83645E-06	0.006910557
1010.879982	307.190148	6.93285E-06	0.007008283
1010.919982	320.682302	7.23735E-06	0.007316385
1010.959982	355.188056	8.0161E-06	0.008103956
1010.999982	419.483655	9.46716E-06	0.0095713
1011.039982	477.596205	1.07787E-05	0.010897678
1011.079982	552.075469	1.24596E-05	0.012597626
1011.119982	622.976157	1.40597E-05	0.014216048

1011.159982	711.7534	1.60633E-05	0.016242549
1011.199982	817.686955	1.84541E-05	0.018660741
1011.239982	831.203628	1.87591E-05	0.01896996
1011.279982	761.037511	1.71756E-05	0.017369297
1011.319982	673.390233	1.51975E-05	0.015369515
1011.359982	599.442955	1.35286E-05	0.013682277
1011.399982	497.895166	1.12368E-05	0.0113649
1011.439982	392.062337	8.8483E-06	0.008949525
1011.479982	341.805985	7.71408E-06	0.007802643
1011.519982	308.21852	6.95606E-06	0.007036196
1011.559982	306.248491	6.9116E-06	0.0069915
1011.599982	308.172898	6.95503E-06	0.007035711
1011.639982	300.922485	6.7914E-06	0.006870453
1011.679982	313.049122	7.06508E-06	0.007147603
1011.719982	326.765484	7.37464E-06	0.007461072
1011.759982	316.148589	7.13503E-06	0.007218941
1011.799982	296.920602	6.70108E-06	0.006780157
1011.839982	264.464988	5.96861E-06	0.006039275
1011.879982	234.116793	5.28369E-06	0.00534646
1011.919982	255.11866	5.75767E-06	0.005826304
1011.959982	304.20363	6.86545E-06	0.006947563
1011.999982	340.759949	7.69048E-06	0.007782763
1012.039982	417.028271	9.41175E-06	0.009525064
1012.079982	496.650088	1.12087E-05	0.011344102
1012.119982	568.319529	1.28262E-05	0.012981633
1012.159982	615.020054	1.38801E-05	0.014048928
1012.199982	623.880299	1.40801E-05	0.014251886
1012.239982	620.245679	1.39981E-05	0.014169417
1012.279982	613.838132	1.38535E-05	0.014023592
1012.319982	569.44778	1.28516E-05	0.013009975
1012.359982	515.836437	1.16417E-05	0.011785601
1012.399982	476.373588	1.07511E-05	0.010884402
1012.439982	414.581255	9.35652E-06	0.009472916
1012.479982	335.99562	7.58295E-06	0.007677588
1012.519982	280.938935	6.3404E-06	0.006419782
1012.559982	265.111344	5.98319E-06	0.006058343
1012.599982	270.542476	6.10577E-06	0.006182699
1012.639982	289.250927	6.52799E-06	0.006610504
1012.679982	307.305084	6.93545E-06	0.007023389
1012.719982	310.248949	7.00189E-06	0.007090951
1012.759982	322.093321	7.2692E-06	0.007361952
1012.799982	337.034263	7.60639E-06	0.007703756
1012.839982	347.898312	7.85158E-06	0.007952394
1012.879982	327.815722	7.39834E-06	0.007493635
1012.919982	308.944877	6.97246E-06	0.00706254
1012.959982	321.093545	7.24663E-06	0.00734055

1012.999982	375.184509	8.46739E-06	0.008577468
1013.039982	447.628232	1.01023E-05	0.01023408
1013.079982	540.666823	1.22021E-05	0.0123617
1013.119982	672.817823	1.51846E-05	0.015383782
1013.159982	818.655143	1.84759E-05	0.018719049
1013.199982	911.911903	2.05806E-05	0.020852245
1013.239982	984.648318	2.22221E-05	0.022516362
1013.279982	1000.010739	2.25688E-05	0.022868563
1013.319982	925.74034	2.08927E-05	0.02117096
1013.359982	762.149934	1.72007E-05	0.017430463
1013.399982	596.961807	1.34726E-05	0.013653129
1013.439982	485.30955	1.09528E-05	0.011099965
1013.479982	388.730006	8.77309E-06	0.008891356
1013.519982	328.918643	7.42324E-06	0.007523598
1013.559982	311.962709	7.04056E-06	0.007136034
1013.599982	309.786887	6.99146E-06	0.007086542
1013.639982	285.024414	6.4326E-06	0.006520345
1013.679982	264.842193	5.97712E-06	0.006058886
1013.719982	290.705101	6.56081E-06	0.006650823
1013.759982	324.570151	7.3251E-06	0.007425889
1013.799982	298.903082	6.74583E-06	0.006838919
1013.839982	289.291464	6.52891E-06	0.006619265
1013.879982	311.699062	7.03461E-06	0.007132254
1013.919982	308.137129	6.95423E-06	0.007051029
1013.959982	305.290898	6.88999E-06	0.006986174
1013.999982	298.656138	6.74025E-06	0.006834616
1014.039982	324.967482	7.33406E-06	0.007437034
1014.079982	394.274135	8.89822E-06	0.009023505
1014.119982	479.431782	1.08201E-05	0.010972887
1014.159982	561.196757	1.26654E-05	0.012844771
1014.199982	638.454954	1.4409E-05	0.014613647
1014.239982	691.018393	1.55953E-05	0.0158174
1014.279982	693.078832	1.56418E-05	0.015865189
1014.319982	637.276458	1.43824E-05	0.014588398
1014.359982	545.357317	1.2308E-05	0.012484697
1014.399982	449.484585	1.01442E-05	0.010290318
1014.439982	386.240463	8.71691E-06	0.008842781
1014.479982	340.849629	7.6925E-06	0.007803889
1014.519982	314.291224	7.09312E-06	0.007196107
1014.559982	344.262653	7.76953E-06	0.007882653
1014.599982	377.176052	8.51234E-06	0.008636618
1014.639982	376.922496	8.50662E-06	0.008631152
1014.679982	355.397616	8.02083E-06	0.008138575
1014.719982	348.033565	7.85463E-06	0.007970253
1014.759982	339.474768	7.66147E-06	0.007774556
1014.799982	337.7816	7.62326E-06	0.007736084

1014.839982	345.854282	7.80545E-06	0.007921282
1014.879982	351.558587	7.93419E-06	0.008052248
1014.919982	361.359121	8.15537E-06	0.00827705
1014.959982	375.154696	8.46672E-06	0.008593381
1014.999982	409.472725	9.24123E-06	0.009379847
1015.039982	512.427295	1.15648E-05	0.011738704
1015.079982	623.585074	1.40734E-05	0.014285674
1015.119982	689.14787	1.55531E-05	0.01578827
1015.159982	766.168147	1.72913E-05	0.017553484
1015.199982	854.439526	1.92835E-05	0.019576619
1015.239982	873.704391	1.97183E-05	0.020018798
1015.279982	814.632494	1.83851E-05	0.018666045
1015.319982	701.623182	1.58347E-05	0.016077245
1015.359982	588.278624	1.32766E-05	0.013480558
1015.399982	520.238265	1.17411E-05	0.011921865
1015.439982	456.277583	1.02975E-05	0.010456543
1015.479982	377.434616	8.51817E-06	0.008650035
1015.519982	323.953541	7.31118E-06	0.00742465
1015.559982	293.136545	6.61568E-06	0.006718623
1015.599982	271.500255	6.12738E-06	0.00622297
1015.639982	293.857436	6.63195E-06	0.006735677
1015.679982	301.096413	6.79533E-06	0.006901877
1015.719982	286.621181	6.46864E-06	0.006570328
1015.759982	299.08865	6.75001E-06	0.006856394
1015.799982	311.396697	7.02779E-06	0.007138829
1015.839982	297.634109	6.71719E-06	0.006823587
1015.879982	283.15301	6.39037E-06	0.006491848
1015.919982	275.3976	6.21534E-06	0.006314288
1015.959982	294.219966	6.64013E-06	0.006746111
1015.999982	328.655701	7.4173E-06	0.007535978
1016.039982	368.714805	8.32138E-06	0.008454854
1016.079982	442.59071	9.98866E-06	0.010149273
1016.119982	500.924128	1.13052E-05	0.011487399
1016.159982	542.492765	1.22433E-05	0.012441158
1016.199982	579.959522	1.30889E-05	0.013300918
1016.239982	587.23174	1.3253E-05	0.013468231
1016.279982	606.862792	1.3696E-05	0.013919019
1016.319982	567.363336	1.28046E-05	0.013013571
1016.359982	509.213031	1.14922E-05	0.011680241
1016.399982	472.348439	1.06602E-05	0.010835074
1016.439982	397.196551	8.96417E-06	0.009111544
1016.479982	323.352467	7.29761E-06	0.007417879
1016.519982	294.030741	6.63586E-06	0.006745489
1016.559982	299.601308	6.76158E-06	0.006873556
1016.599982	328.267547	7.40854E-06	0.007531523
1016.639982	319.228838	7.20455E-06	0.007324434

1016.679982	325.910943	7.35536E-06	0.007478043
1016.719982	349.484804	7.88739E-06	0.008019262
1016.759982	354.789327	8.0071E-06	0.0081413
1016.799982	356.71313	8.05052E-06	0.008185767
1016.839982	363.722943	8.20872E-06	0.008346955
1016.879982	360.912411	8.14529E-06	0.008282783
1016.919982	373.050391	8.41923E-06	0.008561681
1016.959982	392.164688	8.85061E-06	0.009000717
1016.999982	403.445146	9.10519E-06	0.009259983
1017.039982	440.873909	9.94991E-06	0.010119456
1017.079982	524.329464	1.18334E-05	0.0120355
1017.119982	599.179592	1.35226E-05	0.013754156
1017.159982	659.590349	1.4886E-05	0.01514148
1017.199982	704.81063	1.59066E-05	0.016180187
1017.239982	726.590854	1.63981E-05	0.016680847
1017.279982	734.575141	1.65783E-05	0.016864811
1017.319982	699.033976	1.57762E-05	0.016049467
1017.359982	583.266916	1.31635E-05	0.01339204
1017.399982	457.28508	1.03203E-05	0.01049986
1017.439982	389.801699	8.79728E-06	0.008950706
1017.479982	338.755555	7.64524E-06	0.00777888
1017.519982	296.065543	6.68179E-06	0.006798852
1017.559982	279.30749	6.30358E-06	0.006414272
1017.599982	289.19752	6.52679E-06	0.006641657
1017.639982	296.538636	6.69246E-06	0.006810519
1017.679982	298.354913	6.73345E-06	0.006852502
1017.719982	305.14405	6.88668E-06	0.007008708
1017.759982	319.373587	7.20782E-06	0.007335828
1017.799982	309.575273	6.98668E-06	0.007111046
1017.839982	286.375099	6.46309E-06	0.006578388
1017.879982	282.682475	6.37975E-06	0.00649382
1017.919982	291.266438	6.57348E-06	0.006691274
1017.959982	300.804031	6.78873E-06	0.006910653
1017.999982	335.772007	7.57791E-06	0.007714309
1018.039982	390.621686	8.81579E-06	0.008974824
1018.079982	425.645513	9.60623E-06	0.009779907
1018.119982	463.860642	1.04687E-05	0.010658381
1018.159982	567.563316	1.28091E-05	0.013041727
1018.199982	645.626372	1.45709E-05	0.014836078
1018.239982	610.331901	1.37743E-05	0.014025585
1018.279982	558.532835	1.26053E-05	0.012835733
1018.319982	490.770036	1.1076E-05	0.011278908
1018.359982	415.06905	9.36753E-06	0.009539518
1018.399982	372.050704	8.39667E-06	0.008551165
1018.439982	364.500992	8.22628E-06	0.008377972
1018.479982	346.862912	7.82821E-06	0.007972878

1018.519982	343.357768	7.74911E-06	0.00789262
1018.559982	350.435246	7.90884E-06	0.008055623
1018.599982	344.904771	7.78402E-06	0.007928803
1018.639982	341.853082	7.71515E-06	0.007858958
1018.679982	337.353057	7.61359E-06	0.00775581
1018.719982	312.098413	7.04363E-06	0.007175483
1018.759982	284.353006	6.41745E-06	0.006537843
1018.799982	244.117156	5.50938E-06	0.005612961
1018.839982	244.823836	5.52533E-06	0.00562943
1018.879982	269.556228	6.08351E-06	0.006198365
1018.919982	274.569507	6.19665E-06	0.006313892
1018.959982	335.259791	7.56635E-06	0.007709804
1018.999982	410.145482	9.25641E-06	0.009432284
1019.039982	476.336054	1.07502E-05	0.010954926
1019.079982	553.662522	1.24954E-05	0.012733804
1019.119982	641.745048	1.44833E-05	0.014760212
1019.159982	686.964261	1.55038E-05	0.01580088
1019.199982	660.201122	1.48998E-05	0.015185896
1019.239982	630.395066	1.42271E-05	0.014500868
1019.279982	592.679582	1.3376E-05	0.013633841
1019.319982	534.933858	1.20727E-05	0.012305957
1019.359982	474.630554	1.07118E-05	0.01091913
1019.399982	415.915807	9.38664E-06	0.009568741
1019.439982	356.648644	8.04906E-06	0.008205537
1019.479982	319.438146	7.20927E-06	0.007349711
1019.519982	294.700742	6.65099E-06	0.006780812
1019.559982	292.129956	6.59297E-06	0.006721925
1019.599982	306.469978	6.9166E-06	0.007052166
1019.639982	341.78766	7.71367E-06	0.007865168
1019.679982	323.541447	7.30188E-06	0.007445581
1019.719982	291.614266	6.58133E-06	0.006711111
1019.759982	318.078928	7.1786E-06	0.007320447
1019.799982	338.040604	7.62911E-06	0.007780162
1019.839982	311.303413	7.02568E-06	0.007165074
1019.879982	275.560408	6.21901E-06	0.006342648
1019.919982	267.189142	6.03009E-06	0.006150206
1019.959982	301.52476	6.80499E-06	0.006940821
1019.999982	333.050048	7.51648E-06	0.007666805
1020.039982	372.147941	8.39886E-06	0.008567174
1020.079982	394.100094	8.89429E-06	0.009072887
1020.119982	434.534318	9.80683E-06	0.010004148
1020.159982	467.432931	1.05493E-05	0.010761984
1020.199982	498.745738	1.1256E-05	0.011483368
1020.239982	524.150085	1.18293E-05	0.012068763
1020.279982	522.688196	1.17963E-05	0.012035574
1020.319982	487.184667	1.09951E-05	0.011218499

1020.359982	444.948092	1.00419E-05	0.010246311
1020.399982	398.431785	8.99205E-06	0.009175488
1020.439982	345.880229	7.80603E-06	0.00796559
1020.479982	303.779074	6.85587E-06	0.006996279
1020.519982	261.248998	5.89603E-06	0.006017012
1020.559982	250.48208	5.65303E-06	0.005769258
1020.599982	250.933746	5.66323E-06	0.005779887
1020.639982	252.602373	5.70088E-06	0.00581855
1020.679982	262.491613	5.92407E-06	0.00604658
1020.719982	278.89238	6.29421E-06	0.006424629
1020.759982	273.172424	6.16512E-06	0.006293109
1020.799982	261.561451	5.90308E-06	0.006025862
1020.839982	259.094924	5.84741E-06	0.005969271
1020.879982	277.403165	6.2606E-06	0.006391324
1020.919982	319.006946	7.19954E-06	0.007350157
1020.959982	377.603486	8.52198E-06	0.008700605
1020.999982	407.565951	9.1982E-06	0.009391358
1021.039982	424.370053	9.57744E-06	0.00977895
1021.079982	482.102145	1.08804E-05	0.011109732
1021.119982	580.952959	1.31113E-05	0.013388209
1021.159982	653.990338	1.47597E-05	0.015071965
1021.199982	720.108935	1.62519E-05	0.016596395
1021.239982	786.484855	1.77499E-05	0.018126875
1021.279982	789.139472	1.78098E-05	0.01818877
1021.319982	697.769877	1.57477E-05	0.016083435
1021.359982	575.517954	1.29886E-05	0.013266076
1021.399982	470.981382	1.06294E-05	0.010856863
1021.439982	395.419648	8.92407E-06	0.009115403
1021.479982	324.487059	7.32322E-06	0.007480524
1021.519982	270.628074	6.1077E-06	0.006239136
1021.559982	251.079536	5.66652E-06	0.005788685
1021.599982	257.514674	5.81175E-06	0.005937281
1021.639982	286.285675	6.46107E-06	0.006600886
1021.679982	311.23862	7.02422E-06	0.007176507
1021.719982	307.762674	6.94577E-06	0.007096637
1021.759982	304.913151	6.88147E-06	0.007031206
1021.799982	301.177697	6.79716E-06	0.006945339
1021.839982	288.690081	6.51533E-06	0.006657628
1021.879982	273.465183	6.17173E-06	0.006306766
1021.919982	270.748163	6.11041E-06	0.006244349
1021.959982	293.25513	6.61836E-06	0.006763699
1021.999982	326.908125	7.37786E-06	0.007540174
1022.039982	376.299827	8.49256E-06	0.008679739
1022.079982	458.224249	1.03415E-05	0.010569823
1022.119982	524.123271	1.18287E-05	0.012090384
1022.159982	538.645485	1.21565E-05	0.012425866

1022.199982	521.024317	1.17588E-05	0.012019838
1022.239982	534.906671	1.20721E-05	0.012340582
1022.279982	542.312994	1.22392E-05	0.012511939
1022.319982	506.608077	1.14334E-05	0.011688633
1022.359982	437.243802	9.86798E-06	0.010088632
1022.399982	372.87035	8.41516E-06	0.008603664
1022.439982	320.458201	7.2323E-06	0.007394588
1022.479982	269.740603	6.08767E-06	0.00622452
1022.519982	276.701087	6.24476E-06	0.00638539
1022.559982	288.580354	6.51286E-06	0.006659787
1022.599982	305.700316	6.89923E-06	0.007055153
1022.639982	325.026536	7.3354E-06	0.007501469
1022.679982	312.856108	7.06073E-06	0.007220864
1022.719982	278.002739	6.27413E-06	0.006416683
1022.759982	271.788176	6.13388E-06	0.006273488
1022.799982	285.283216	6.43844E-06	0.006585241
1022.839982	280.315562	6.32633E-06	0.006470825
1022.879982	294.122085	6.63793E-06	0.006789801
1022.919982	289.683536	6.53775E-06	0.006687599
1022.959982	305.256943	6.88922E-06	0.0070474
1022.999982	335.02971	7.56115E-06	0.00773506
1023.039982	363.596361	8.20586E-06	0.008394926
1023.079982	458.64401	1.0351E-05	0.010589856
1023.119982	572.124446	1.29121E-05	0.013210578
1023.159982	654.778082	1.47774E-05	0.015119674
1023.199982	695.18815	1.56894E-05	0.016053423
1023.239982	701.850136	1.58398E-05	0.016207896
1023.279982	685.134554	1.54625E-05	0.0158225
1023.319982	625.591641	1.41187E-05	0.014447981
1023.359982	548.300469	1.23744E-05	0.012663443
1023.399982	459.737636	1.03756E-05	0.010618428
1023.439982	384.583086	8.6795E-06	0.008882952
1023.479982	335.02573	7.56106E-06	0.007738598
1023.519982	301.989543	6.81548E-06	0.006975783
1023.559982	285.941149	6.45329E-06	0.006605333
1023.599982	296.547413	6.69266E-06	0.006850609
1023.639982	316.793054	7.14958E-06	0.007318594
1023.679982	335.200465	7.56501E-06	0.007744147
1023.719982	324.648057	7.32685E-06	0.007500647
1023.759982	289.973547	6.5443E-06	0.006699791
1023.799982	248.372038	5.60541E-06	0.00573882
1023.839982	239.190828	5.3982E-06	0.005526897
1023.879982	239.560383	5.40654E-06	0.005535652
1023.919982	249.989522	5.64192E-06	0.00577687
1023.959982	278.989295	6.2964E-06	0.006447261
1023.999982	344.685084	7.77906E-06	0.00796576

1024.039982	397.666947	8.97479E-06	0.009190543
1024.079982	445.715063	1.00592E-05	0.010301393
1024.119982	510.137487	1.15131E-05	0.011790788
1024.159982	571.989343	1.2909E-05	0.013220884
1024.199982	599.322932	1.35259E-05	0.01385321
1024.239982	573.307722	1.29388E-05	0.013252392
1024.279982	544.490158	1.22884E-05	0.012586746
1024.319982	535.919808	1.2095E-05	0.012389113
1024.359982	480.880819	1.08528E-05	0.011117185
1024.399982	419.196765	9.46069E-06	0.009691528
1024.439982	369.01196	8.32809E-06	0.008531624
1024.479982	313.363587	7.07218E-06	0.007245307
1024.519982	262.928396	5.93393E-06	0.006079427
1024.559982	267.654771	6.0406E-06	0.006188952
1024.599982	304.63834	6.87526E-06	0.007044394
1024.639982	320.711391	7.23801E-06	0.007416354
1024.679982	323.17834	7.29368E-06	0.007473693
1024.719982	332.006084	7.49291E-06	0.00767814
1024.759982	332.082508	7.49464E-06	0.007680207
1024.799982	317.163209	7.15793E-06	0.007335448
1024.839982	312.56446	7.05414E-06	0.007229369
1024.879982	302.726307	6.83211E-06	0.007002094
1024.919982	308.068574	6.95268E-06	0.007125939
1024.959982	348.330086	7.86132E-06	0.008057543
1024.999982	412.790905	9.31612E-06	0.009549018
1025.039982	456.375517	1.02998E-05	0.010557666
1025.079982	536.482954	1.21077E-05	0.012411333
1025.119982	619.510356	1.39815E-05	0.014332701
1025.159982	674.08599	1.52132E-05	0.015595945
1025.199982	703.757438	1.58828E-05	0.016283072
1025.239982	703.436012	1.58756E-05	0.01627627
1025.279982	693.900309	1.56604E-05	0.016056257
1025.319982	682.414561	1.54011E-05	0.015791103
1025.359982	614.2658	1.38631E-05	0.014214692
1025.399982	500.528511	1.12962E-05	0.011583155
1025.439982	422.050122	9.52508E-06	0.009767401
1025.479982	395.112893	8.91715E-06	0.009144356
1025.519982	387.413123	8.74337E-06	0.008966505
1025.559982	369.756747	8.34489E-06	0.00855819
1025.599982	367.237718	8.28804E-06	0.008500218
1025.639982	347.255507	7.83707E-06	0.008038016
1025.679982	320.190497	7.22625E-06	0.007411824
1025.719982	320.214492	7.2268E-06	0.007412668
1025.759982	309.568749	6.98654E-06	0.007166508
1025.799982	291.438623	6.57736E-06	0.00674706
1025.839982	269.301164	6.07775E-06	0.006234801

1025.879982	246.940312	5.5731E-06	0.005717331
1025.919982	247.283873	5.58085E-06	0.005725508
1025.959982	283.794608	6.40485E-06	0.006571119
1025.999982	332.624523	7.50687E-06	0.007702051
1026.039982	373.9148	8.43874E-06	0.008658481
1026.079982	425.094916	9.5938E-06	0.009844006
1026.119982	484.206661	1.09279E-05	0.011213306
1026.159982	541.390316	1.22184E-05	0.012538059
1026.199982	617.590692	1.39382E-05	0.014303341
1026.239982	669.286402	1.51049E-05	0.015501213
1026.279982	624.794913	1.41008E-05	0.014471318
1026.319982	538.411576	1.21512E-05	0.012471019
1026.359982	438.33363	9.89258E-06	0.010153348
1026.399982	390.422917	8.8113E-06	0.00904392
1026.439982	374.33409	8.4482E-06	0.008671569
1026.479982	349.310204	7.88344E-06	0.008092198
1026.519982	346.493084	7.81987E-06	0.008027249
1026.559982	349.80177	7.89454E-06	0.008104218
1026.599982	351.984245	7.94379E-06	0.008155099
1026.639982	339.56528	7.66352E-06	0.007867671
1026.679982	316.401984	7.14075E-06	0.007331267
1026.719982	288.046402	6.50081E-06	0.006674507
1026.759982	271.680576	6.13145E-06	0.00629553
1026.799982	272.940295	6.15988E-06	0.006324967
1026.839982	275.146559	6.20967E-06	0.006376342
1026.879982	316.723054	7.148E-06	0.007340136
1026.919982	360.577831	8.13774E-06	0.008356807
1026.959982	368.800261	8.32331E-06	0.008547704
1026.999982	381.14852	8.60199E-06	0.008834245
1027.039982	426.048359	9.61532E-06	0.009875316
1027.079982	518.160616	1.16942E-05	0.012010841
1027.119982	611.138743	1.37926E-05	0.014166604
1027.159982	663.889662	1.49831E-05	0.015390005
1027.199982	667.015141	1.50536E-05	0.01546306
1027.239982	695.40953	1.56944E-05	0.01612194
1027.279982	712.26105	1.60747E-05	0.016513258
1027.319982	670.032299	1.51217E-05	0.01553482
1027.359982	589.474358	1.33036E-05	0.013667602
1027.399982	479.506383	1.08218E-05	0.011118308
1027.439982	374.995657	8.46313E-06	0.008695358
1027.479982	332.503402	7.50414E-06	0.007710352
1027.519982	324.767121	7.32954E-06	0.00753125
1027.559982	302.318706	6.82291E-06	0.007010951
1027.599982	280.499811	6.33049E-06	0.006505211
1027.639982	259.033932	5.84604E-06	0.006007619
1027.679981	250.870703	5.6618E-06	0.005818521

1027.719981	264.031075	5.95881E-06	0.006123992
1027.759981	257.173909	5.80406E-06	0.005965177
1027.799981	261.393319	5.89928E-06	0.006063283
1027.839981	265.678876	5.996E-06	0.006162931
1027.879981	267.561322	6.03849E-06	0.006206839
1027.919981	285.209483	6.43678E-06	0.006616496
1027.959981	300.226306	6.77569E-06	0.006965138
1027.999981	293.790226	6.63044E-06	0.006816088
1028.039981	332.450963	7.50296E-06	0.007713338
1028.079981	400.721129	9.04372E-06	0.009297665
1028.119981	437.51495	9.8741E-06	0.010151763
1028.159981	510.507292	1.15214E-05	0.011845882
1028.199981	604.484999	1.36424E-05	0.014027099
1028.239981	640.428443	1.44536E-05	0.014861747
1028.279981	599.367113	1.35269E-05	0.013909421
1028.319981	525.325074	1.18559E-05	0.012191613
1028.359981	438.213869	9.88988E-06	0.010170353
1028.399981	396.526517	8.94905E-06	0.009203204
1028.439981	353.689856	7.98229E-06	0.008209304
1028.479981	300.72697	6.78699E-06	0.006980282
1028.519981	272.656573	6.15348E-06	0.006328976
1028.559981	276.016447	6.22931E-06	0.006407216
1028.599981	297.432646	6.71264E-06	0.006904622
1028.639981	318.036372	7.17764E-06	0.007383205
1028.679981	314.824621	7.10515E-06	0.007308929
1028.719981	325.519324	7.34652E-06	0.00755751
1028.759981	336.13864	7.58618E-06	0.007804359
1028.799981	348.929478	7.87485E-06	0.008101648
1028.839981	355.665648	8.02688E-06	0.008258373
1028.879981	347.188756	7.83557E-06	0.008061858
1028.919981	333.588682	7.52863E-06	0.00774636
1028.959981	332.907315	7.51325E-06	0.007730838
1028.999981	373.120544	8.42081E-06	0.008665014
1029.039981	444.378439	1.0029E-05	0.010320244
1029.079981	512.858594	1.15745E-05	0.01191109
1029.119981	633.013997	1.42862E-05	0.014702259
1029.159981	779.516663	1.75926E-05	0.018105605
1029.199981	877.618761	1.98066E-05	0.020384986
1029.239981	936.588024	2.11375E-05	0.021755547
1029.279981	952.59998	2.14989E-05	0.022128341
1029.319981	850.77768	1.92009E-05	0.019763836
1029.359981	678.659873	1.53164E-05	0.015766097
1029.399981	553.12623	1.24833E-05	0.012850297
1029.439981	451.88273	1.01984E-05	0.010498603
1029.479981	384.495949	8.67754E-06	0.008933352
1029.519981	338.348654	7.63606E-06	0.007861474

1029.559981	298.158876	6.72903E-06	0.006927941
1029.599981	287.773159	6.49464E-06	0.006686881
1029.639981	304.492215	6.87197E-06	0.00707565
1029.679981	308.166931	6.9549E-06	0.00716132
1029.719981	297.34168	6.71059E-06	0.006910026
1029.759981	290.064368	6.54635E-06	0.006741168
1029.799981	278.872674	6.29377E-06	0.006481322
1029.839981	268.062546	6.0498E-06	0.006230324
1029.879981	269.853846	6.09023E-06	0.006272201
1029.919981	300.493191	6.78171E-06	0.006984621
1029.959981	297.253931	6.70861E-06	0.006909597
1029.999981	306.026669	6.9066E-06	0.007113793
1030.039981	368.693296	8.32089E-06	0.008570854
1030.079981	452.450987	1.02112E-05	0.010518341
1030.119981	523.929985	1.18244E-05	0.01218052
1030.159981	607.123806	1.37019E-05	0.014115189
1030.199981	665.511217	1.50197E-05	0.015473255
1030.239981	723.442763	1.63271E-05	0.016820827
1030.279981	715.387995	1.61453E-05	0.01663419
1030.319981	621.174411	1.4019E-05	0.014444098
1030.359981	534.002917	1.20517E-05	0.012417591
1030.399981	467.598815	1.05531E-05	0.010873867
1030.439981	380.640623	8.59053E-06	0.008852024
1030.479981	301.607789	6.80687E-06	0.007014341
1030.519981	282.263301	6.37029E-06	0.006564711
1030.559981	310.05868	6.99759E-06	0.007211439
1030.599981	329.867138	7.44464E-06	0.007672448
1030.639981	342.646733	7.73306E-06	0.00797
1030.679981	318.935965	7.19794E-06	0.007418773
1030.719981	286.208658	6.45933E-06	0.006657761
1030.759981	271.846375	6.13519E-06	0.006323912
1030.799981	275.700555	6.22218E-06	0.00641382
1030.839981	328.611339	7.4163E-06	0.007645019
1030.879981	361.346166	8.15508E-06	0.008406908
1030.919981	365.551221	8.24998E-06	0.008505071
1030.959981	379.565059	8.56625E-06	0.008831466
1030.999981	421.93872	9.52257E-06	0.009817769
1031.039981	466.156867	1.05205E-05	0.010847068
1031.079981	540.534204	1.21991E-05	0.012578252
1031.119981	626.975212	1.415E-05	0.014590304
1031.159981	686.41661	1.54915E-05	0.015974181
1031.199981	751.120114	1.69517E-05	0.017480629
1031.239981	758.584436	1.71202E-05	0.017655029
1031.279981	709.008041	1.60013E-05	0.016501845
1031.319981	658.184984	1.48543E-05	0.015319555
1031.359981	595.770148	1.34457E-05	0.013867359

1031.399981	499.50207	1.12731E-05	0.01162704
1031.439981	399.095515	9.00703E-06	0.009290211
1031.479981	322.462654	7.27753E-06	0.00750663
1031.519981	298.878715	6.74528E-06	0.006957887
1031.559981	310.200117	7.00078E-06	0.007221729
1031.599981	347.588875	7.8446E-06	0.008092486
1031.639981	348.227568	7.85901E-06	0.00810767
1031.679981	318.55327	7.1893E-06	0.007417061
1031.719981	305.309477	6.89041E-06	0.007108973
1031.759981	276.884066	6.24889E-06	0.006447352
1031.799981	259.203499	5.84986E-06	0.006035887
1031.839981	275.131436	6.20933E-06	0.006407038
1031.879981	308.376489	6.95963E-06	0.007181501
1031.919981	305.91071	6.90398E-06	0.007124353
1031.959981	304.2104	6.8656E-06	0.00708503
1031.999981	326.581591	7.37049E-06	0.007606347
1032.039981	356.733026	8.05097E-06	0.00830892
1032.079981	419.16792	9.46004E-06	0.009763514
1032.119981	474.885208	1.07175E-05	0.011061744
1032.159981	533.441831	1.2039E-05	0.012426214
1032.199981	582.102756	1.31372E-05	0.013560268
1032.239981	566.892934	1.2794E-05	0.013206462
1032.279981	518.820028	1.1709E-05	0.012087013
1032.319981	485.833983	1.09646E-05	0.011318972
1032.359981	472.851208	1.06716E-05	0.011016926
1032.399981	436.553816	9.85241E-06	0.01017163
1032.439981	360.356383	8.13274E-06	0.008396568
1032.479981	312.436668	7.05126E-06	0.007280285
1032.519981	315.953437	7.13063E-06	0.007362517
1032.559981	324.132112	7.31521E-06	0.007553393
1032.599981	331.591887	7.48357E-06	0.007727531
1032.639981	342.28092	7.7248E-06	0.007976941
1032.679981	339.254696	7.65651E-06	0.00790672
1032.719981	326.915666	7.37803E-06	0.00761944
1032.759981	315.978962	7.13121E-06	0.007364823
1032.799981	297.815084	6.72127E-06	0.006941729
1032.839981	312.228389	7.04656E-06	0.007277969
1032.879981	331.353008	7.47818E-06	0.007724058
1032.919981	359.634123	8.11644E-06	0.008383634
1032.959981	376.19463	8.49019E-06	0.008770025
1032.999981	378.532336	8.54295E-06	0.008824865
1033.039981	408.866079	9.22754E-06	0.009532416
1033.079981	489.357075	1.10441E-05	0.011409446
1033.119981	615.670509	1.38948E-05	0.014355022
1033.159981	728.10814	1.64324E-05	0.016977284
1033.199981	770.155826	1.73813E-05	0.017958405

1033.239981	760.152867	1.71556E-05	0.017725843
1033.279981	703.08568	1.58677E-05	0.01639574
1033.319981	604.860666	1.36509E-05	0.014105709
1033.359981	495.274274	1.11777E-05	0.011550537
1033.399981	408.565242	9.22075E-06	0.009528721
1033.439981	347.849707	7.85048E-06	0.008113003
1033.479981	294.214911	6.64002E-06	0.006862329
1033.519981	272.554082	6.15117E-06	0.006357353
1033.559981	298.698067	6.7412E-06	0.006967434
1033.599981	324.825332	7.33086E-06	0.007577172
1033.639981	338.931938	7.64922E-06	0.007906541
1033.679981	329.253462	7.43079E-06	0.007681061
1033.719981	305.59709	6.8969E-06	0.007129464
1033.759981	278.5081	6.28554E-06	0.00649774
1033.799981	278.193448	6.27844E-06	0.00649065
1033.839981	272.497721	6.14989E-06	0.006358006
1033.879981	274.488014	6.19481E-06	0.006404692
1033.919981	278.862828	6.29355E-06	0.006507023
1033.959981	288.728664	6.5162E-06	0.006737494
1033.999981	313.482668	7.07487E-06	0.007315413
1034.039981	358.855577	8.09887E-06	0.008374556
1034.079981	420.141124	9.482E-06	0.009805146
1034.119981	492.066295	1.11053E-05	0.011484162
1034.159981	574.740354	1.29711E-05	0.013414181
1034.199981	624.007485	1.4083E-05	0.014564617
1034.239981	606.777821	1.36941E-05	0.014163017
1034.279981	572.076251	1.2911E-05	0.013353552
1034.319981	510.656985	1.15248E-05	0.011920348
1034.359981	442.958589	9.99696E-06	0.010340454
1034.399981	374.843124	8.45969E-06	0.0087507
1034.439981	300.861411	6.79002E-06	0.007023871
1034.479981	270.97605	6.11555E-06	0.006326416
1034.519981	266.174389	6.00719E-06	0.006214553
1034.559981	284.726891	6.42589E-06	0.006647968
1034.599981	301.69673	6.80887E-06	0.007044462
1034.639981	310.623106	7.01033E-06	0.007253169
1034.679981	305.209395	6.88815E-06	0.007127032
1034.719981	292.183966	6.59419E-06	0.006823135
1034.759981	285.020565	6.43252E-06	0.006656111
1034.799981	291.939955	6.58868E-06	0.006817964
1034.839981	297.337219	6.71049E-06	0.00694428
1034.879981	310.111109	6.99878E-06	0.007242893
1034.919981	318.061366	7.1782E-06	0.007428865
1034.959981	310.613649	7.01012E-06	0.007255191
1034.999981	338.632299	7.64246E-06	0.007909945
1035.039981	400.181807	9.03155E-06	0.009348011

1035.079981	451.137847	1.01816E-05	0.010538722
1035.119981	476.923344	1.07635E-05	0.011141509
1035.159981	518.735676	1.17071E-05	0.012118765
1035.199981	584.297661	1.31868E-05	0.013650959
1035.239981	655.290368	1.4789E-05	0.015310155
1035.279981	665.535024	1.50202E-05	0.015550111
1035.319981	608.841268	1.37407E-05	0.014226021
1035.359981	521.798338	1.17763E-05	0.01219267
1035.399981	423.801664	9.56461E-06	0.0099032
1035.439981	333.193996	7.51972E-06	0.007786223
1035.479981	279.49429	6.3078E-06	0.006531597
1035.519981	245.842426	5.54832E-06	0.005745397
1035.559981	237.41476	5.35812E-06	0.005548655
1035.599981	231.335944	5.22093E-06	0.005406795
1035.639981	235.658153	5.31848E-06	0.005508027
1035.679981	255.220546	5.75997E-06	0.005965488
1035.719981	276.673125	6.24413E-06	0.006467167
1035.759981	268.282976	6.05477E-06	0.006271292
1035.799981	257.145095	5.80341E-06	0.006011168
1035.839981	267.90517	6.04625E-06	0.006262944
1035.879981	295.294148	6.66438E-06	0.006903495
1035.919981	311.315427	7.02596E-06	0.007278328
1035.959981	313.974374	7.08596E-06	0.007340775
1035.999981	323.157995	7.29323E-06	0.007555782
1036.039981	349.292306	7.88304E-06	0.008167145
1036.079981	411.209794	9.28043E-06	0.00961527
1036.119981	486.692089	1.0984E-05	0.011380703
1036.159981	522.118622	1.17835E-05	0.012209581
1036.199981	540.756924	1.22041E-05	0.01264592
1036.239981	540.588414	1.22003E-05	0.012642467
1036.279981	524.341581	1.18337E-05	0.012262984
1036.319981	511.125358	1.15354E-05	0.011954352
1036.359981	450.693199	1.01715E-05	0.010541354
1036.399981	383.794966	8.66172E-06	0.008977004
1036.439981	345.755063	7.80321E-06	0.008087559
1036.479981	295.710521	6.67377E-06	0.006917234
1036.519981	281.718704	6.358E-06	0.006590193
1036.559981	272.253748	6.14439E-06	0.006369027
1036.599981	273.325865	6.16858E-06	0.006394354
1036.639981	279.065268	6.29811E-06	0.006528877
1036.679981	294.931159	6.65619E-06	0.006900334
1036.719981	287.219391	6.48214E-06	0.006720166
1036.759981	270.674056	6.10874E-06	0.006333293
1036.799981	276.218023	6.23386E-06	0.006463262
1036.839981	283.93859	6.4081E-06	0.006644173
1036.879981	280.91029	6.33975E-06	0.006573564

1036.919981	292.623857	6.60411E-06	0.006847937
1036.959981	328.90146	7.42285E-06	0.007697196
1036.999981	378.958238	8.55256E-06	0.008869004
1037.039981	414.307082	9.35033E-06	0.00969667
1037.079981	497.955194	1.12382E-05	0.011654866
1037.119981	617.654606	1.39396E-05	0.014457042
1037.159981	671.905577	1.5164E-05	0.015727466
1037.199981	645.2817	1.45631E-05	0.015104857
1037.239981	628.567681	1.41859E-05	0.01471418
1037.279981	627.598076	1.4164E-05	0.014692049
1037.319981	575.970518	1.29989E-05	0.013483969
1037.359981	502.046901	1.13305E-05	0.011753806
1037.399981	403.568575	9.10798E-06	0.009448619
1037.439981	314.107768	7.08897E-06	0.007354386
1037.479981	295.189036	6.66201E-06	0.006911697
1037.519981	279.207208	6.30132E-06	0.006537743
1037.559981	264.550154	5.97053E-06	0.006194781
1037.599981	248.448966	5.60715E-06	0.005817976
1037.639981	242.67291	5.47679E-06	0.005682936
1037.679981	246.563099	5.56459E-06	0.005774259
1037.719981	246.384943	5.56056E-06	0.005770309
1037.759981	252.768443	5.70463E-06	0.005920038
1037.799981	267.355929	6.03385E-06	0.00626193
1037.839981	269.510045	6.08247E-06	0.006312627
1037.879981	272.172046	6.14254E-06	0.006375223
1037.919981	254.779341	5.75001E-06	0.005968055
1037.959981	240.135325	5.41952E-06	0.005625245
1037.999981	257.833469	5.81894E-06	0.006040062
1038.039981	285.490607	6.44313E-06	0.006688222
1038.079981	330.58752	7.4609E-06	0.007745011
1038.119981	401.641063	9.06448E-06	0.009410017
1038.159981	465.350012	1.05023E-05	0.010903069
1038.199981	532.306393	1.20134E-05	0.012472326
1038.239981	543.671953	1.22699E-05	0.01273912
1038.279981	522.94284	1.18021E-05	0.012253875
1038.319981	481.690533	1.08711E-05	0.011287664
1038.359981	420.067761	9.48034E-06	0.00984401
1038.399981	350.703203	7.91488E-06	0.008218814
1038.439981	316.570835	7.14456E-06	0.0074192
1038.479981	312.513738	7.053E-06	0.007324399
1038.519981	295.017778	6.65814E-06	0.006914612
1038.559981	258.320858	5.82994E-06	0.006054744
1038.599981	242.530221	5.47357E-06	0.005684849
1038.639981	255.690935	5.77059E-06	0.005993564
1038.679981	274.233049	6.18906E-06	0.006428451
1038.719981	297.119605	6.70558E-06	0.006965215

1038.759981	308.89595	6.97135E-06	0.007241561
1038.799981	287.608363	6.49092E-06	0.006742768
1038.839981	296.169033	6.68412E-06	0.006943734
1038.879981	322.239397	7.27249E-06	0.007555249
1038.919981	314.742275	7.10329E-06	0.007379755
1038.959981	330.773792	7.4651E-06	0.007755944
1038.999981	346.264808	7.81471E-06	0.008119488
1039.039981	395.752791	8.93159E-06	0.009280278
1039.079981	494.415396	1.11583E-05	0.011594332
1039.119981	586.26871	1.32313E-05	0.013748875
1039.159981	654.914565	1.47805E-05	0.015359314
1039.199981	737.533031	1.66451E-05	0.017297581
1039.239981	745.682164	1.6829E-05	0.017489378
1039.279981	688.851715	1.55464E-05	0.016157087
1039.319981	586.07103	1.32268E-05	0.013746885
1039.359981	531.338826	1.19916E-05	0.012463565
1039.399981	450.894863	1.01761E-05	0.010577006
1039.439981	361.188236	8.15152E-06	0.008473011
1039.479981	303.632557	6.85256E-06	0.007123103
1039.519981	283.130331	6.38986E-06	0.006642384
1039.559981	269.435556	6.08079E-06	0.006321341
1039.599981	245.187749	5.53355E-06	0.005752674
1039.639981	258.678054	5.838E-06	0.006069422
1039.679981	279.574904	6.30962E-06	0.006559982
1039.719981	299.986066	6.77027E-06	0.007039183
1039.759981	289.266678	6.52835E-06	0.006787913
1039.799981	241.330928	5.4465E-06	0.005663274
1039.839981	224.108396	5.05781E-06	0.005259318
1039.879981	249.73583	5.63619E-06	0.005860961
1039.919981	282.352105	6.37229E-06	0.006626676
1039.959981	303.187187	6.84251E-06	0.007115939
1039.999981	309.931004	6.99471E-06	0.007274499
1040.039981	344.664723	7.7786E-06	0.008090058
1040.079981	401.592583	9.06339E-06	0.009426646
1040.119981	440.914434	9.95082E-06	0.010350052
1040.159981	468.213019	1.05669E-05	0.010991283
1040.199981	514.601544	1.16138E-05	0.012080716
1040.239981	558.859947	1.26127E-05	0.013120225
1040.279981	547.666691	1.23601E-05	0.012857938
1040.319981	522.378365	1.17894E-05	0.012264699
1040.359981	455.844621	1.02878E-05	0.010702993
1040.399981	385.392676	8.69778E-06	0.009049166
1040.439981	325.167666	7.33858E-06	0.007635353
1040.479981	295.718426	6.67395E-06	0.006944114
1040.519981	297.563736	6.7156E-06	0.006987715
1040.559981	293.761887	6.6298E-06	0.006898701

1040.599981	278.43822	6.28396E-06	0.006539092
1040.639981	247.328081	5.58185E-06	0.005808697
1040.679981	240.231076	5.42168E-06	0.005642235
1040.719981	269.565494	6.08372E-06	0.006331447
1040.759981	287.957243	6.49879E-06	0.006763685
1040.799981	283.426821	6.39655E-06	0.006657528
1040.839981	276.765051	6.2462E-06	0.006501296
1040.879981	307.637249	6.94294E-06	0.007226772
1040.919981	338.844766	7.64725E-06	0.00796018
1040.959981	366.470578	8.27073E-06	0.008609499
1040.999981	381.502376	8.60998E-06	0.008962986
1041.039981	411.931821	9.29673E-06	0.009678265
1041.079981	476.645339	1.07572E-05	0.011199128
1041.119981	558.516935	1.26049E-05	0.013123265
1041.159981	630.201575	1.42228E-05	0.014808181
1041.199981	648.972202	1.46464E-05	0.01524983
1041.239981	626.056013	1.41292E-05	0.014711901
1041.279981	620.184169	1.39967E-05	0.014574476
1041.319981	595.055656	1.34296E-05	0.013984487
1041.359981	500.120203	1.1287E-05	0.011753847
1041.399981	421.364987	9.50962E-06	0.009903319
1041.439981	391.482705	8.83522E-06	0.009201351
1041.479981	347.477906	7.84209E-06	0.008167382
1041.519981	320.830111	7.24069E-06	0.007541322
1041.559981	293.516184	6.62425E-06	0.006899555
1041.599981	265.805252	5.99885E-06	0.006248407
1041.639981	279.625346	6.31075E-06	0.006573534
1041.679981	299.947938	6.76941E-06	0.007051556
1041.719981	319.13145	7.20235E-06	0.007502834
1041.759981	306.628983	6.92019E-06	0.007209176
1041.799981	272.131967	6.14164E-06	0.00639836
1041.839981	271.422236	6.12562E-06	0.006381918
1041.879981	273.344376	6.169E-06	0.00642736
1041.919981	277.266309	6.25751E-06	0.006519829
1041.959981	285.618122	6.446E-06	0.006716477
1041.999981	285.851486	6.45127E-06	0.006722223
1042.039981	314.16518	7.09027E-06	0.007388345
1042.079981	388.921335	8.77741E-06	0.009146766
1042.119981	466.293595	1.05236E-05	0.010966851
1042.159981	521.296979	1.17649E-05	0.012260957
1042.199981	566.458046	1.27842E-05	0.013323661
1042.239981	614.941589	1.38784E-05	0.014464597
1042.279981	583.268215	1.31636E-05	0.013720106
1042.319981	564.065805	1.27302E-05	0.01326892
1042.359981	515.547616	1.16352E-05	0.012128058
1042.399981	438.341644	9.89276E-06	0.010312213

1042.439981	350.746629	7.91586E-06	0.008251812
1042.479981	286.5034	6.46598E-06	0.006740657
1042.519981	263.697721	5.95129E-06	0.006204339
1042.559981	294.284549	6.64159E-06	0.006924258
1042.599981	320.336738	7.22955E-06	0.007537533
1042.639981	325.728769	7.35124E-06	0.007664702
1042.679981	325.78228	7.35245E-06	0.007666255
1042.719981	323.640038	7.3041E-06	0.007616136
1042.759981	327.117354	7.38258E-06	0.007698262
1042.799981	311.077405	7.02058E-06	0.007321065
1042.839981	277.471008	6.26213E-06	0.006530404
1042.879981	261.380731	5.899E-06	0.006151948
1042.919981	277.496858	6.26272E-06	0.006531513
1042.959981	295.180979	6.66182E-06	0.006948015
1042.999981	339.380492	7.65934E-06	0.007988697
1043.039981	400.994043	9.04988E-06	0.009439384
1043.079981	468.549182	1.05745E-05	0.011030052
1043.119981	528.578002	1.19293E-05	0.012443659
1043.159981	614.377756	1.38657E-05	0.014464091
1043.199981	699.375641	1.57839E-05	0.0164658
1043.239981	740.149097	1.67041E-05	0.017426421
1043.279981	715.778686	1.61541E-05	0.016853278
1043.319981	636.645337	1.43682E-05	0.014990629
1043.359981	522.32898	1.17882E-05	0.012299375
1043.399981	405.19568	9.1447E-06	0.009541582
1043.439981	326.988727	7.37968E-06	0.007700253
1043.479981	295.444668	6.66777E-06	0.006957689
1043.519981	259.510772	5.8568E-06	0.006111684
1043.559981	230.717077	5.20696E-06	0.005433778
1043.599981	240.288803	5.42298E-06	0.005659426
1043.639981	266.995788	6.02572E-06	0.006288685
1043.679981	296.010034	6.68053E-06	0.00697234
1043.719981	311.201938	7.02339E-06	0.007330457
1043.759981	289.413872	6.53167E-06	0.006817494
1043.799981	259.925861	5.86616E-06	0.006123103
1043.839981	273.08918	6.16324E-06	0.006433439
1043.879981	310.516096	7.00792E-06	0.007315423
1043.919981	329.848634	7.44422E-06	0.007771174
1043.959981	342.94002	7.73968E-06	0.008079915
1043.999981	355.60942	8.02561E-06	0.008378736
1044.039981	335.72586	7.57687E-06	0.00791055
1044.079981	338.902774	7.64856E-06	0.007985712
1044.119981	403.941374	9.11639E-06	0.009518609
1044.159981	474.968283	1.07194E-05	0.01119274
1044.199981	508.973787	1.14868E-05	0.011994547
1044.239981	537.855234	1.21386E-05	0.012675657

1044.279981	559.363085	1.2624E-05	0.013183038
1044.319981	515.76741	1.16402E-05	0.012156043
1044.359981	419.084941	9.45816E-06	0.009877727
1044.399981	329.162742	7.42874E-06	0.007758581
1044.439981	294.930486	6.65617E-06	0.00695197
1044.479981	302.611509	6.82952E-06	0.007133297
1044.519981	335.456416	7.57078E-06	0.007907835
1044.559981	337.839655	7.62457E-06	0.007964321
1044.599981	294.755025	6.65221E-06	0.006948899
1044.639981	302.956836	6.83731E-06	0.007142531
1044.679981	348.121767	7.85662E-06	0.008207657
1044.719981	332.693676	7.50843E-06	0.00784421
1044.759981	293.543354	6.62486E-06	0.006921393
1044.799981	278.549185	6.28647E-06	0.006568101
1044.839981	283.737144	6.40355E-06	0.006690687
1044.879981	268.48241	6.05927E-06	0.006331214
1044.919981	277.166847	6.25527E-06	0.006536256
1044.959981	322.848929	7.28625E-06	0.00761384
1044.999981	360.828546	8.1434E-06	0.00850985
1045.039981	393.758914	8.88659E-06	0.009286842
1045.079981	447.149661	1.00915E-05	0.010546472
1045.119981	532.246387	1.20121E-05	0.012554043
1045.159981	587.027521	1.32484E-05	0.013846691
1045.199981	637.36684	1.43845E-05	0.01503466
1045.239981	650.800944	1.46877E-05	0.015352141
1045.279981	576.939679	1.30207E-05	0.013610303
1045.319981	496.457667	1.12044E-05	0.011712139
1045.359981	431.02862	9.72772E-06	0.010168965
1045.399981	365.170201	8.24138E-06	0.008615541
1045.439981	309.217848	6.97862E-06	0.007295724
1045.479981	279.740538	6.31335E-06	0.006600486
1045.519981	273.484292	6.17216E-06	0.006453116
1045.559981	274.707267	6.19976E-06	0.006482221
1045.599981	285.348074	6.43991E-06	0.006733568
1045.639981	297.848531	6.72203E-06	0.00702882
1045.679981	303.644127	6.85283E-06	0.007165862
1045.719981	303.380138	6.84687E-06	0.007159906
1045.759981	292.458258	6.60038E-06	0.006902409
1045.799981	291.941877	6.58872E-06	0.006890485
1045.839981	284.350722	6.4174E-06	0.006711573
1045.879981	294.701203	6.651E-06	0.006956143
1045.919981	321.672354	7.2597E-06	0.007593062
1045.959981	335.465253	7.57098E-06	0.007918946
1045.999981	337.224524	7.61069E-06	0.007960779
1046.039981	343.476057	7.75178E-06	0.008108668
1046.079981	377.109322	8.51083E-06	0.008903011

1046.119981	441.344533	9.96053E-06	0.010419911
1046.159981	511.033587	1.15333E-05	0.012065694
1046.199981	571.385057	1.28954E-05	0.01349113
1046.239981	598.994155	1.35185E-05	0.014143557
1046.279981	569.130663	1.28445E-05	0.013438929
1046.319981	526.270753	1.18772E-05	0.012427349
1046.359981	508.932703	1.14859E-05	0.012018388
1046.399981	447.750315	1.01051E-05	0.010573977
1046.439981	348.015876	7.85423E-06	0.008218984
1046.479981	284.426987	6.41912E-06	0.006717482
1046.519981	263.050698	5.93669E-06	0.006212862
1046.559981	270.055841	6.09478E-06	0.006378557
1046.599981	292.76884	6.60738E-06	0.006915289
1046.639981	298.109683	6.72792E-06	0.00704171
1046.679981	288.958902	6.5214E-06	0.006825819
1046.719981	329.443086	7.43507E-06	0.007782438
1046.759981	384.78959	8.68417E-06	0.009090236
1046.799981	369.787024	8.34558E-06	0.008736151
1046.839981	346.449022	7.81887E-06	0.008185108
1046.879981	324.400162	7.32126E-06	0.00766448
1046.919981	332.295567	7.49945E-06	0.007851322
1046.959981	391.390044	8.83313E-06	0.009247932
1046.999981	461.424897	1.04137E-05	0.010903162
1047.039981	516.652564	1.16601E-05	0.012208621
1047.079981	581.489626	1.31234E-05	0.013741261
1047.119981	636.845828	1.43727E-05	0.015049966
1047.159981	671.009777	1.51438E-05	0.015857935
1047.199981	658.951306	1.48716E-05	0.015573553
1047.239981	647.091154	1.46039E-05	0.015293836
1047.279981	637.669121	1.43913E-05	0.015071724
1047.319981	594.309866	1.34127E-05	0.014047437
1047.359981	544.331585	1.22848E-05	0.012866614
1047.399981	469.699112	1.06005E-05	0.011102916
1047.439981	380.408608	8.58529E-06	0.008992579
1047.479981	328.103959	7.40485E-06	0.007756431
1047.519981	295.087046	6.6597E-06	0.006976173
1047.559981	283.815466	6.40532E-06	0.006709957
1047.599981	301.286296	6.79961E-06	0.007123273
1047.639981	312.544987	7.05371E-06	0.007389743
1047.679981	296.937289	6.70146E-06	0.007020987
1047.719981	297.536149	6.71498E-06	0.007035415
1047.759981	304.405001	6.87E-06	0.007198108
1047.799981	282.8078	6.38258E-06	0.006687665
1047.839981	269.743184	6.08773E-06	0.006378965
1047.879981	269.607934	6.08468E-06	0.00637601
1047.919981	259.064512	5.84673E-06	0.0061269

1047.959981	240.029988	5.41714E-06	0.005676949
1047.999981	286.668971	6.46972E-06	0.006780266
1048.039981	354.902446	8.00965E-06	0.008394437
1048.079981	428.892378	9.6795E-06	0.010144894
1048.119981	491.334922	1.10887E-05	0.011622335
1048.159981	522.903992	1.18012E-05	0.012369561
1048.199981	555.412385	1.25349E-05	0.013139065
1048.239981	589.312802	1.33E-05	0.013941559
1048.279981	567.280178	1.28027E-05	0.013420839
1048.319981	488.163285	1.10172E-05	0.011549515
1048.359981	424.106083	9.57148E-06	0.01003436
1048.399981	369.636327	8.34218E-06	0.008745938
1048.439981	313.131272	7.06694E-06	0.007409259
1048.479981	280.804743	6.33737E-06	0.006644608
1048.519981	266.363921	6.01146E-06	0.006303139
1048.559981	272.30512	6.14555E-06	0.006443975
1048.599981	286.480039	6.46546E-06	0.006779676
1048.639981	317.425187	7.16384E-06	0.007512294
1048.679981	334.049084	7.53902E-06	0.007906022
1048.719981	332.925343	7.51366E-06	0.007879727
1048.759981	332.362236	7.50095E-06	0.007866699
1048.799981	317.381877	7.16287E-06	0.007512415
1048.839981	298.017354	6.72584E-06	0.007054326
1048.879981	299.652622	6.76274E-06	0.007093305
1048.919981	308.560635	6.96378E-06	0.007304452
1048.959981	314.442672	7.09653E-06	0.007443979
1048.999981	343.73332	7.75758E-06	0.008137704
1049.039981	396.164368	8.94088E-06	0.009379338
1049.079981	446.114916	1.00682E-05	0.010562339
1049.119981	489.212565	1.10408E-05	0.011583172
1049.159981	564.624121	1.27428E-05	0.013369215
1049.199981	637.324211	1.43835E-05	0.015091189
1049.239981	674.705098	1.52272E-05	0.015976939
1049.279981	663.263587	1.49689E-05	0.015706604
1049.319981	616.893316	1.39224E-05	0.014609077
1049.359981	539.467531	1.2175E-05	0.01277599
1049.399981	447.975078	1.01102E-05	0.010609616
1049.439981	364.457693	8.2253E-06	0.008631961
1049.479981	308.425146	6.96073E-06	0.007305143
1049.519981	265.853004	5.99993E-06	0.006297049
1049.559981	250.608172	5.65588E-06	0.005936183
1049.599981	260.598561	5.88135E-06	0.006173061
1049.639981	281.512801	6.35335E-06	0.006668732
1049.679981	298.999012	6.74799E-06	0.007083231
1049.719981	291.417213	6.57688E-06	0.006903883
1049.759981	247.011543	5.57471E-06	0.005852104

1049.799981	232.570383	5.24879E-06	0.005510179
1049.839981	252.434535	5.6971E-06	0.005981039
1049.879981	265.597231	5.99416E-06	0.006293148
1049.919981	273.258121	6.16706E-06	0.006474914
1049.959981	293.276527	6.61884E-06	0.00694952
1049.999981	319.062303	7.20079E-06	0.007560831
1050.039981	355.077875	8.01361E-06	0.008414614
1050.079981	383.868285	8.66337E-06	0.009097234
1050.119981	399.216582	9.00976E-06	0.009461331
1050.159981	449.89105	1.01534E-05	0.010662709
1050.199981	521.860977	1.17777E-05	0.012368914
1050.239981	532.887983	1.20265E-05	0.012630753
1050.279981	498.034669	1.12399E-05	0.011805093
1050.319981	457.85064	1.03331E-05	0.01085301
1050.359981	419.887738	9.47628E-06	0.009953507
1050.399981	368.296419	8.31194E-06	0.008730859
1050.439981	334.406217	7.54708E-06	0.007927757
1050.479981	301.35266	6.80111E-06	0.00714443
1050.519981	270.746688	6.11038E-06	0.006419072
1050.559981	258.1449	5.82597E-06	0.006120532
1050.599981	258.788472	5.8405E-06	0.006136024
1050.639981	243.44189	5.49414E-06	0.005772368
1050.679981	252.939079	5.70848E-06	0.005997788
1050.719981	279.263062	6.30258E-06	0.006622245
1050.759981	300.876139	6.79036E-06	0.007135034
1050.799981	295.00422	6.65783E-06	0.006996052
1050.839981	286.879957	6.47448E-06	0.006803644
1050.879981	269.296828	6.07765E-06	0.006386885
1050.919981	259.042714	5.84623E-06	0.006143923
1050.959981	273.580825	6.17434E-06	0.006488982
1050.999981	302.74808	6.8326E-06	0.007181065
1051.039981	369.16627	8.33157E-06	0.008756812
1051.079981	442.049173	9.97643E-06	0.01048603
1051.119981	508.046853	1.14659E-05	0.012052047
1051.159981	548.307053	1.23745E-05	0.013007607
1051.199981	569.944519	1.28629E-05	0.013521432
1051.239981	568.500468	1.28303E-05	0.013487686
1051.279981	523.383242	1.1812E-05	0.012417751
1051.319981	489.881086	1.10559E-05	0.011623324
1051.359981	451.41851	1.01879E-05	0.010711137
1051.399981	405.346401	9.1481E-06	0.009618316
1051.439981	339.33222	7.65826E-06	0.008052196
1051.479981	285.086458	6.434E-06	0.006765227
1051.519981	267.666042	6.04085E-06	0.006352074
1051.559981	265.722049	5.99698E-06	0.006306181
1051.599981	280.583053	6.33237E-06	0.006659119

1051.639981	261.769824	5.90778E-06	0.006212858
1051.679981	237.521069	5.36052E-06	0.005637551
1051.719981	231.21309	5.21816E-06	0.00548804
1051.759981	251.958012	5.68634E-06	0.005980666
1051.799981	273.771974	6.17865E-06	0.006498706
1051.839981	268.793968	6.06631E-06	0.006380783
1051.879981	257.531226	5.81212E-06	0.006113654
1051.919981	255.170169	5.75884E-06	0.006057834
1051.959981	271.306455	6.12301E-06	0.00644116
1051.999981	287.674849	6.49242E-06	0.006830026
1052.039981	290.55408	6.5574E-06	0.006898648
1052.079981	350.80978	7.91729E-06	0.00832962
1052.119981	450.647465	1.01705E-05	0.010700571
1052.159981	523.623448	1.18175E-05	0.01243385
1052.199981	561.351764	1.26689E-05	0.013330245
1052.239981	568.79772	1.2837E-05	0.013507575
1052.279981	558.68229	1.26087E-05	0.013267863
1052.319981	507.369311	1.14506E-05	0.012049715
1052.359981	468.750384	1.0579E-05	0.011132962
1052.399981	408.219236	9.21294E-06	0.009695697
1052.439981	333.762151	7.53255E-06	0.007927553
1052.479981	290.810093	6.56318E-06	0.006907614
1052.519981	270.871842	6.1132E-06	0.006434265
1052.559981	268.639297	6.06281E-06	0.006381476
1052.599981	269.503695	6.08232E-06	0.006402253
1052.639981	252.491437	5.69838E-06	0.005998343
1052.679981	259.611257	5.85906E-06	0.00616772
1052.719981	294.369674	6.64351E-06	0.006993759
1052.759981	345.065974	7.78766E-06	0.008198535
1052.799981	350.7074	7.91498E-06	0.008332888
1052.839981	314.637201	7.10092E-06	0.007476136
1052.879981	309.772401	6.99113E-06	0.007360823
1052.919981	324.524985	7.32408E-06	0.007711667
1052.959981	339.033304	7.65151E-06	0.008056733
1052.999981	360.310787	8.13171E-06	0.008562693
1053.039981	385.221034	8.6939E-06	0.009155027
1053.079981	439.299543	9.91438E-06	0.010440634
1053.119981	519.010248	1.17133E-05	0.012335551
1053.159981	562.256396	1.26893E-05	0.013363909
1053.199981	606.759878	1.36937E-05	0.014422231
1053.239981	641.322809	1.44738E-05	0.015244345
1053.279981	628.120869	1.41758E-05	0.0149311
1053.319981	573.822624	1.29504E-05	0.013640891
1053.359981	513.276365	1.15839E-05	0.012202051
1053.399981	437.43406	9.87228E-06	0.010399457
1053.439981	348.345812	7.86168E-06	0.008281808

1053.479981	293.02637	6.6132E-06	0.006966871
1053.519981	268.668783	6.06348E-06	0.006387998
1053.559981	274.191611	6.18812E-06	0.006519558
1053.599981	281.618209	6.35573E-06	0.006696398
1053.639981	293.142125	6.61581E-06	0.006970681
1053.679981	300.385311	6.77928E-06	0.00714319
1053.719981	296.130321	6.68325E-06	0.007042273
1053.759981	274.741707	6.20054E-06	0.006533878
1053.799981	254.688646	5.74797E-06	0.006057209
1053.839981	253.300368	5.71664E-06	0.00602442
1053.879981	263.483764	5.94646E-06	0.006266857
1053.919981	260.771265	5.88524E-06	0.006202576
1053.959981	259.946587	5.86663E-06	0.006183196
1053.999981	264.685053	5.97357E-06	0.006296146
1054.039981	292.489422	6.60108E-06	0.006957801
1054.079981	357.482338	8.06788E-06	0.008504189
1054.119981	445.734422	1.00596E-05	0.010604031
1054.159981	511.093629	1.15347E-05	0.012159389
1054.199981	531.257925	1.19898E-05	0.012639596
1054.239981	535.149256	1.20776E-05	0.012732661
1054.279981	547.17941	1.23491E-05	0.013019385
1054.319981	529.164967	1.19425E-05	0.012591233
1054.359981	482.152593	1.08815E-05	0.011473031
1054.399981	376.029208	8.48646E-06	0.008948118
1054.439981	283.055426	6.38817E-06	0.006735938
1054.479981	272.432204	6.14842E-06	0.006483381
1054.519981	313.612908	7.07781E-06	0.007463688
1054.559981	342.845637	7.73755E-06	0.008159709
1054.599981	325.794395	7.35273E-06	0.007754184
1054.639981	306.273268	6.91216E-06	0.007289841
1054.679981	289.0553	6.52358E-06	0.006880284
1054.719981	306.005916	6.90613E-06	0.00728403
1054.759981	310.695313	7.01196E-06	0.007395935
1054.799981	293.260792	6.61849E-06	0.006981181
1054.839981	288.259548	6.50562E-06	0.006862384
1054.879981	297.852843	6.72212E-06	0.007091034
1054.919981	289.854058	6.5416E-06	0.006900867
1054.959981	292.3055	6.59693E-06	0.006959495
1054.999981	312.637758	7.0558E-06	0.007443868
1055.039981	363.199609	8.19691E-06	0.008648067
1055.079981	422.472338	9.53461E-06	0.010059778
1055.119981	493.188202	1.11306E-05	0.011744088
1055.159981	573.104157	1.29342E-05	0.013647611
1055.199981	664.640124	1.5E-05	0.015828002
1055.239981	712.974258	1.60908E-05	0.016979693
1055.279981	642.578773	1.45021E-05	0.015303784

1055.319981	566.272259	1.278E-05	0.013486964
1055.359981	512.671736	1.15703E-05	0.012210818
1055.399981	429.641905	9.69642E-06	0.010233601
1055.439981	342.572775	7.73139E-06	0.008160019
1055.479981	271.22512	6.12117E-06	0.006460776
1055.519981	227.070931	5.12467E-06	0.005409196
1055.559981	222.390461	5.01904E-06	0.005297901
1055.599981	256.980129	5.79968E-06	0.006122146
1055.639981	306.641371	6.92047E-06	0.007305523
1055.679981	347.820341	7.84982E-06	0.008286898
1055.719981	349.438801	7.88635E-06	0.008325774
1055.759981	300.361019	6.77873E-06	0.007156712
1055.799981	270.636381	6.10789E-06	0.006448706
1055.839981	260.46033	5.87823E-06	0.006206467
1055.879981	255.315127	5.76211E-06	0.006084093
1055.919981	277.453817	6.26175E-06	0.006611903
1055.959981	303.04064	6.83921E-06	0.007221927
1055.999981	305.061622	6.88482E-06	0.007270365
1056.039981	310.06405	6.99771E-06	0.007389865
1056.079981	354.099738	7.99154E-06	0.008439703
1056.119981	432.786757	9.76739E-06	0.01031554
1056.159981	473.488222	1.0686E-05	0.011286094
1056.199981	461.672767	1.04193E-05	0.011004876
1056.239981	457.715357	1.033E-05	0.010910957
1056.279981	494.656666	1.11637E-05	0.011792005
1056.319981	497.537507	1.12287E-05	0.01186113
1056.359981	423.149405	9.54989E-06	0.010088124
1056.399981	362.442325	8.17982E-06	0.00864116
1056.439981	334.529393	7.54986E-06	0.007975977
1056.479981	273.251101	6.1669E-06	0.006515203
1056.519981	251.723692	5.68105E-06	0.006002146
1056.559981	257.911286	5.8207E-06	0.006149917
1056.599981	294.6939	6.65083E-06	0.007027268
1056.639981	333.961961	7.53706E-06	0.007963955
1056.679981	339.539367	7.66293E-06	0.008097265
1056.719981	350.739901	7.91571E-06	0.00836469
1056.759981	369.198044	8.33229E-06	0.008805226
1056.799981	349.291792	7.88303E-06	0.008330785
1056.839981	316.384292	7.14035E-06	0.00754621
1056.879981	288.043396	6.50074E-06	0.0068705
1056.919981	305.274496	6.88962E-06	0.007281777
1056.959981	344.41081	7.77287E-06	0.008215615
1056.999981	363.674003	8.20762E-06	0.00867545
1057.039981	403.922029	9.11596E-06	0.009635932
1057.079981	462.080728	1.04285E-05	0.011023778
1057.119981	531.064773	1.19854E-05	0.012669998

1057.159981	579.819027	1.30857E-05	0.013833687
1057.199981	612.38053	1.38206E-05	0.014611112
1057.239981	622.54075	1.40499E-05	0.014854092
1057.279981	567.874347	1.28161E-05	0.013550241
1057.319981	549.113679	1.23927E-05	0.013103082
1057.359981	506.951442	1.14412E-05	0.012097454
1057.399981	423.395343	9.55544E-06	0.010103925
1057.439981	343.730118	7.75751E-06	0.008203101
1057.479981	283.011597	6.38718E-06	0.006754312
1057.519981	251.779062	5.6823E-06	0.006009149
1057.559981	240.773265	5.43392E-06	0.005746693
1057.599981	236.705009	5.3421E-06	0.005649807
1057.639981	237.851299	5.36797E-06	0.005677382
1057.679981	260.945265	5.88917E-06	0.006228858
1057.719981	292.640973	6.6045E-06	0.006985711
1057.759981	296.066929	6.68182E-06	0.00706776
1057.799981	300.697778	6.78633E-06	0.00717858
1057.839981	306.084013	6.90789E-06	0.007307442
1057.879981	288.314618	6.50686E-06	0.006883476
1057.919981	266.639289	6.01768E-06	0.006366221
1057.959981	264.194709	5.96251E-06	0.006308093
1057.999981	273.586588	6.17447E-06	0.006532587
1058.039981	335.273458	7.56665E-06	0.008005823
1058.079981	412.987276	9.32055E-06	0.009861885
1058.119981	451.103188	1.01808E-05	0.010772477
1058.159981	491.376957	1.10897E-05	0.01173467
1058.199981	537.546792	1.21317E-05	0.012837746
1058.239981	550.146126	1.2416E-05	0.013139141
1058.279981	527.127603	1.18965E-05	0.012589866
1058.319981	492.109167	1.11062E-05	0.011753933
1058.359981	468.141966	1.05653E-05	0.011181904
1058.399981	428.231991	9.6646E-06	0.010229012
1058.439981	365.808341	8.25578E-06	0.008738253
1058.479981	309.61277	6.98753E-06	0.00739616
1058.519981	269.647639	6.08557E-06	0.006441699
1058.559981	262.246772	5.91854E-06	0.006265134
1058.599981	301.823494	6.81174E-06	0.007210903
1058.639981	340.597606	7.68681E-06	0.008137568
1058.679981	323.344527	7.29744E-06	0.007725649
1058.719981	298.776354	6.74297E-06	0.007138913
1058.759981	292.172133	6.59392E-06	0.006981377
1058.799981	282.158322	6.36792E-06	0.006742354
1058.839981	294.71066	6.65121E-06	0.007042566
1058.879981	314.033528	7.0873E-06	0.007504599
1058.919981	312.519562	7.05313E-06	0.007468702
1058.959981	308.15427	6.95461E-06	0.007364656

1058.999981	337.746488	7.62247E-06	0.008072193
1059.039981	371.114539	8.37554E-06	0.00887003
1059.079981	446.776126	1.00831E-05	0.010678825
1059.119981	556.789529	1.2566E-05	0.013308864
1059.159981	648.89588	1.46447E-05	0.015511055
1059.199981	671.59957	1.51571E-05	0.016054365
1059.239981	653.70315	1.47532E-05	0.015627147
1059.279981	589.293972	1.32995E-05	0.014087941
1059.319981	525.49925	1.18598E-05	0.012563308
1059.359981	465.04333	1.04954E-05	0.011118386
1059.399981	411.368458	9.28401E-06	0.009835483
1059.439981	356.525656	8.04629E-06	0.008524559
1059.479981	311.013967	7.01915E-06	0.007436651
1059.519981	273.04901	6.16234E-06	0.006529118
1059.559981	234.527017	5.29295E-06	0.005608196
1059.599981	226.724797	5.11686E-06	0.005421828
1059.639981	257.876115	5.8199E-06	0.006167004
1059.679981	317.929196	7.17522E-06	0.007603436
1059.719981	354.068815	7.99084E-06	0.008468053
1059.759981	334.879332	7.55776E-06	0.008009412
1059.799981	285.787165	6.44982E-06	0.006835517
1059.839981	244.693683	5.5224E-06	0.005852856
1059.879981	234.899372	5.30135E-06	0.005618796
1059.919981	233.055336	5.25973E-06	0.005574897
1059.959981	242.234733	5.4669E-06	0.005794696
1059.999981	264.817432	5.97656E-06	0.006335154
1060.039981	286.582764	6.46777E-06	0.006856099
1060.079981	337.727211	7.62203E-06	0.008079964
1060.119981	408.484133	9.21892E-06	0.009773159
1060.159981	457.06332	1.03153E-05	0.01093585
1060.199981	493.36988	1.11347E-05	0.011804978
1060.239981	515.220012	1.16278E-05	0.012328256
1060.279981	509.472724	1.14981E-05	0.012191194
1060.319981	490.986124	1.10809E-05	0.011749271
1060.359981	455.733414	1.02853E-05	0.010906087
1060.399981	394.406339	8.9012E-06	0.009438834
1060.439981	345.036022	7.78698E-06	0.008257627
1060.479981	286.628414	6.4688E-06	0.006860037
1060.519981	246.288719	5.55839E-06	0.005894787
1060.559981	252.95409	5.70882E-06	0.006054548
1060.599981	246.893454	5.57204E-06	0.005909707
1060.639981	246.146162	5.55518E-06	0.005892042
1060.679981	272.823454	6.15725E-06	0.006530867
1060.719981	293.311584	6.61963E-06	0.007021578
1060.759981	269.739627	6.08765E-06	0.006457533
1060.799981	232.72903	5.25237E-06	0.005571714

1060.839981	236.323773	5.3335E-06	0.005657988
1060.879981	275.086577	6.20832E-06	0.006586283
1060.919981	302.11087	6.81822E-06	0.007233587
1060.959981	323.612281	7.30348E-06	0.007748698
1060.999981	341.415212	7.70527E-06	0.008175287
1061.039981	385.902339	8.70928E-06	0.009240892
1061.079981	419.757873	9.47335E-06	0.010051983
1061.119981	482.995485	1.09005E-05	0.011566776
1061.159981	551.002032	1.24353E-05	0.013195894
1061.199981	600.489996	1.35522E-05	0.014381619
1061.239981	618.185268	1.39516E-05	0.014805975
1061.279981	614.344097	1.38649E-05	0.014714531
1061.319981	572.662006	1.29242E-05	0.013716695
1061.359981	490.444403	1.10686E-05	0.011747819
1061.399981	409.519736	9.24229E-06	0.009809766
1061.439981	355.939072	8.03305E-06	0.008526599
1061.479981	333.394325	7.52425E-06	0.007986836
1061.519981	319.916805	7.22008E-06	0.007664256
1061.559981	278.413128	6.2834E-06	0.006670202
1061.599981	247.152286	5.57788E-06	0.00592148
1061.639981	241.392057	5.44788E-06	0.00578369
1061.679981	261.389877	5.89921E-06	0.006263068
1061.719981	282.620835	6.37836E-06	0.006772031
1061.759981	271.147155	6.11941E-06	0.006497348
1061.799981	268.03615	6.0492E-06	0.006423043
1061.839981	263.436616	5.9454E-06	0.006313061
1061.879981	240.763443	5.4337E-06	0.005769933
1061.919981	227.6057	5.13674E-06	0.005454811
1061.959981	245.886177	5.54931E-06	0.005893144
1061.999981	284.141965	6.41269E-06	0.006810275
1062.039981	315.878244	7.12893E-06	0.007571211
1062.079981	348.16661	7.85764E-06	0.008345437
1062.119981	403.03688	9.09598E-06	0.009661023
1062.159981	440.871415	9.94985E-06	0.010568336
1062.199981	478.926727	1.08087E-05	0.011481011
1062.239981	515.228355	1.1628E-05	0.012351712
1062.279981	476.920435	1.07634E-05	0.011433776
1062.319981	445.881877	1.00629E-05	0.010690055
1062.359981	430.074996	9.70619E-06	0.010311472
1062.399981	371.180774	8.37703E-06	0.00889976
1062.439981	309.204442	6.97831E-06	0.007414039
1062.479981	272.70133	6.15449E-06	0.006539022
1062.519981	245.011398	5.52957E-06	0.005875274
1062.559981	235.798582	5.32165E-06	0.005654568
1062.599981	242.506051	5.47302E-06	0.005815635
1062.639981	290.128149	6.54779E-06	0.006957942

1062.679981	332.941557	7.51403E-06	0.007985006
1062.719981	335.029322	7.56115E-06	0.00803538
1062.759981	326.173178	7.36127E-06	0.007823268
1062.799981	294.384831	6.64386E-06	0.00706109
1062.839981	255.844456	5.77405E-06	0.006136894
1062.879981	258.243229	5.82819E-06	0.006194666
1062.919981	284.235719	6.4148E-06	0.006818424
1062.959981	312.50879	7.05289E-06	0.007496938
1062.999981	341.431276	7.70563E-06	0.008191083
1063.039981	404.38714	9.12645E-06	0.009701786
1063.079981	490.37095	1.1067E-05	0.011765095
1063.119981	548.233482	1.23729E-05	0.013153841
1063.159981	598.265127	1.3502E-05	0.014354797
1063.199981	657.880168	1.48474E-05	0.015785797
1063.239981	673.470388	1.51993E-05	0.016160491
1063.279981	668.007231	1.5076E-05	0.016030001
1063.319981	613.579343	1.38476E-05	0.014724463
1063.359981	549.543273	1.24024E-05	0.013188244
1063.399981	469.027536	1.05853E-05	0.011256406
1063.439981	374.390721	8.44948E-06	0.008985512
1063.479981	311.058501	7.02016E-06	0.007465797
1063.519981	266.622049	6.01729E-06	0.006399506
1063.559981	266.649869	6.01792E-06	0.006400415
1063.599981	294.307817	6.64212E-06	0.007064556
1063.639981	312.12617	7.04425E-06	0.007492549
1063.679981	329.753154	7.44207E-06	0.00791598
1063.719981	372.529627	8.40747E-06	0.008943199
1063.759981	429.08638	9.68388E-06	0.010301326
1063.799981	427.100798	9.63907E-06	0.010254043
1063.839981	352.820539	7.96267E-06	0.008471005
1063.879981	278.119821	6.27678E-06	0.006677737
1063.919981	248.159358	5.60061E-06	0.005958602
1063.959981	263.956857	5.95714E-06	0.006338157
1063.999981	297.592227	6.71624E-06	0.007146081
1064.039981	353.052411	7.9679E-06	0.008478165
1064.079981	391.942978	8.84561E-06	0.009412433
1064.119981	413.083913	9.32273E-06	0.009920502
1064.159981	471.247696	1.06354E-05	0.011317771
1064.199981	535.922052	1.2095E-05	0.012871514
1064.239981	574.720381	1.29706E-05	0.013803872
1064.279981	558.885542	1.26133E-05	0.013424049
1064.319981	535.424619	1.20838E-05	0.012861017
1064.359981	506.22644	1.14248E-05	0.012160127
1064.399981	426.792406	9.63211E-06	0.010252418
1064.439981	346.988063	7.83104E-06	0.008335669
1064.479981	289.42503	6.53192E-06	0.006953098

1064.519981	255.965835	5.77679E-06	0.006149511
1064.559981	265.322349	5.98796E-06	0.006374538
1064.599981	306.606302	6.91968E-06	0.007366688
1064.639981	308.796597	6.96911E-06	0.007419592
1064.679981	303.702597	6.85414E-06	0.007297471
1064.719981	301.961098	6.81484E-06	0.007255898
1064.759981	288.426705	6.50939E-06	0.006930937
1064.799981	271.977991	6.13816E-06	0.006535917
1064.839981	257.781644	5.81777E-06	0.006194997
1064.879981	256.347482	5.78541E-06	0.006160763
1064.919981	287.922055	6.498E-06	0.00691985
1064.959981	316.502464	7.14302E-06	0.00760703
1064.999981	343.309892	7.74803E-06	0.008251648
1065.039981	399.842336	9.02388E-06	0.009610798
1065.079981	444.742131	1.00372E-05	0.010690432
1065.119981	503.142728	1.13552E-05	0.012094683
1065.159981	555.668296	1.25407E-05	0.013357809
1065.199981	585.647663	1.32173E-05	0.014079017
1065.239981	577.544456	1.30344E-05	0.013884736
1065.279981	543.848533	1.22739E-05	0.013075144
1065.319981	527.057043	1.18949E-05	0.012671921
1065.359981	460.459483	1.03919E-05	0.011071145
1065.399981	370.932671	8.37143E-06	0.008918925
1065.439981	318.797786	7.19482E-06	0.007665651
1065.479981	292.769452	6.6074E-06	0.007040051
1065.519981	267.782887	6.04349E-06	0.006439456
1065.559981	245.652367	5.54403E-06	0.005907498
1065.599981	257.990025	5.82248E-06	0.00620443
1065.639981	301.681055	6.80852E-06	0.007255432
1065.679981	303.264911	6.84427E-06	0.007293798
1065.719981	278.623673	6.28815E-06	0.006701405
1065.759981	254.398343	5.74142E-06	0.006118972
1065.799981	266.476182	6.014E-06	0.006409717
1065.839981	293.744368	6.6294E-06	0.007065881
1065.879981	280.330787	6.32668E-06	0.006743477
1065.919981	249.912487	5.64018E-06	0.006011977
1065.959981	245.873227	5.54902E-06	0.005915029
1065.999981	255.105818	5.75738E-06	0.00613737
1066.039981	283.493742	6.39806E-06	0.006820587
1066.079981	337.671828	7.62078E-06	0.008124364
1066.119981	398.829097	9.00102E-06	0.009596164
1066.159981	440.538636	9.94234E-06	0.010600129
1066.199981	472.273416	1.06586E-05	0.011364149
1066.239981	535.994864	1.20967E-05	0.01289794
1066.279981	541.805511	1.22278E-05	0.013038254
1066.319981	463.603108	1.04629E-05	0.011156774

1066.359981	396.3574	8.94523E-06	0.00953884
1066.399981	361.895355	8.16747E-06	0.008709794
1066.439981	326.290547	7.36392E-06	0.007853182
1066.479981	278.625986	6.2882E-06	0.00670624
1066.519981	244.239773	5.51215E-06	0.00587882
1066.559981	262.367191	5.92126E-06	0.006315381
1066.599981	298.571545	6.73834E-06	0.007187117
1066.639981	301.156543	6.79668E-06	0.007249615
1066.679981	285.055921	6.43332E-06	0.006862288
1066.719981	297.029807	6.70355E-06	0.00715081
1066.759981	279.753144	6.31364E-06	0.006735137
1066.799981	242.477622	5.47238E-06	0.005837937
1066.839981	248.673982	5.61223E-06	0.005987346
1066.879981	282.913896	6.38497E-06	0.006811999
1066.919981	283.65913	6.40179E-06	0.006830199
1066.959981	264.077208	5.95985E-06	0.006358927
1066.999981	286.68815	6.47015E-06	0.006903652
1067.039981	367.283259	8.28907E-06	0.008844771
1067.079981	469.063744	1.05861E-05	0.011296232
1067.119981	555.129651	1.25285E-05	0.013369416
1067.159981	603.172178	1.36128E-05	0.014526988
1067.199981	623.248483	1.40659E-05	0.015011075
1067.239981	615.509269	1.38912E-05	0.01482523
1067.279981	577.097146	1.30243E-05	0.013900552
1067.319981	526.362301	1.18793E-05	0.012678976
1067.359981	462.426198	1.04363E-05	0.011139305
1067.399981	405.398557	9.14928E-06	0.009765942
1067.439981	350.863694	7.9185E-06	0.008452529
1067.479981	298.153785	6.72892E-06	0.007182983
1067.519981	276.885516	6.24892E-06	0.006670847
1067.559981	269.483667	6.08187E-06	0.006492762
1067.599981	275.868944	6.22598E-06	0.006646854
1067.639981	303.450771	6.84846E-06	0.007311691
1067.679981	323.281867	7.29602E-06	0.007789816
1067.719981	291.0854	6.56939E-06	0.007014271
1067.759981	260.769203	5.8852E-06	0.006283979
1067.799981	299.647635	6.76263E-06	0.007221136
1067.839981	311.874049	7.03856E-06	0.007516059
1067.879981	291.362141	6.57564E-06	0.007021992
1067.919981	263.133369	5.93855E-06	0.0063419
1067.959981	247.776118	5.59196E-06	0.005971991
1067.999981	265.561089	5.99334E-06	0.006400891
1068.039981	311.262153	7.02475E-06	0.007502717
1068.079981	364.262939	8.22091E-06	0.008780586
1068.119981	440.071608	9.9318E-06	0.010608357
1068.159981	543.366812	1.2263E-05	0.01309888

1068.199981	580.284698	1.30962E-05	0.013989379
1068.239981	551.115555	1.24379E-05	0.013286673
1068.279981	539.866138	1.2184E-05	0.013015952
1068.319981	515.240516	1.16283E-05	0.012422703
1068.359981	431.316112	9.7342E-06	0.010399634
1068.399981	355.587788	8.02512E-06	0.008574039
1068.439981	312.650238	7.05608E-06	0.007538998
1068.479981	288.416084	6.50915E-06	0.006954896
1068.519981	285.077382	6.4338E-06	0.006874643
1068.559981	293.061928	6.614E-06	0.007067455
1068.599981	308.224729	6.9562E-06	0.007433398
1068.639981	348.455274	7.86415E-06	0.008403945
1068.679981	354.04657	7.99034E-06	0.008539114
1068.719981	327.130958	7.38289E-06	0.007890242
1068.759981	311.155993	7.02236E-06	0.007505215
1068.799981	299.763188	6.76524E-06	0.007230686
1068.839981	290.277537	6.55116E-06	0.007002141
1068.879981	259.079574	5.84707E-06	0.006249811
1068.919981	268.281861	6.05475E-06	0.006472041
1068.959981	320.520214	7.23369E-06	0.00773253
1068.999981	343.560033	7.75367E-06	0.008288675
1069.039981	372.976351	8.41756E-06	0.008998705
1069.079981	442.203505	9.97992E-06	0.01066933
1069.119981	567.049621	1.27975E-05	0.013682084
1069.159981	641.384486	1.44752E-05	0.015476256
1069.199981	677.186991	1.52832E-05	0.016340762
1069.239981	692.815874	1.56359E-05	0.016718518
1069.279981	642.170087	1.44929E-05	0.015496951
1069.319981	585.475802	1.32134E-05	0.014129324
1069.359981	506.508708	1.14312E-05	0.012224064
1069.399981	386.392387	8.72034E-06	0.009325529
1069.439981	299.55405	6.76052E-06	0.007229968
1069.479981	269.28308	6.07734E-06	0.006499598
1069.519981	251.416845	5.67413E-06	0.006068593
1069.559981	245.117806	5.53197E-06	0.005916771
1069.599981	278.879015	6.29391E-06	0.006731967
1069.639981	284.7641	6.42673E-06	0.006874286
1069.679981	277.956257	6.27309E-06	0.006710194
1069.719981	285.463364	6.44251E-06	0.006891682
1069.759981	274.115802	6.18641E-06	0.006617976
1069.799981	262.168784	5.91678E-06	0.006329776
1069.839981	260.952519	5.88933E-06	0.006300646
1069.879981	239.36427	5.40212E-06	0.005779618
1069.919981	219.863299	4.96201E-06	0.005308952
1069.959981	239.176722	5.39789E-06	0.005775521
1069.999981	269.290555	6.07751E-06	0.006502939

1070.039981	293.978467	6.63468E-06	0.007099378
1070.079981	323.625929	7.30379E-06	0.007815636
1070.119981	390.668097	8.81683E-06	0.009435071
1070.159981	491.751246	1.10981E-05	0.011876786
1070.199981	550.139801	1.24159E-05	0.013287484
1070.239981	512.347064	1.1563E-05	0.012375142
1070.279981	457.290184	1.03204E-05	0.01104572
1070.319981	420.901349	9.49916E-06	0.010167138
1070.359981	392.801603	8.86499E-06	0.009488725
1070.399981	362.034946	8.17062E-06	0.008745836
1070.439981	324.94793	7.33362E-06	0.007850202
1070.479981	295.716861	6.67392E-06	0.007144295
1070.519981	280.685141	6.33467E-06	0.006781394
1070.559981	274.413005	6.19312E-06	0.006630106
1070.599981	298.210927	6.73021E-06	0.007205358
1070.639981	312.519418	7.05313E-06	0.007551361
1070.679981	314.627867	7.10071E-06	0.007602591
1070.719981	326.344847	7.36515E-06	0.007886012
1070.759981	332.888839	7.51284E-06	0.008044446
1070.799981	330.167213	7.45141E-06	0.007978974
1070.839981	314.283962	7.09295E-06	0.007595416
1070.879981	293.479976	6.62343E-06	0.007092903
1070.919981	280.917032	6.33991E-06	0.006789532
1070.959981	284.924962	6.43036E-06	0.006886658
1070.999981	317.494551	7.16541E-06	0.007674154
1071.039981	362.436249	8.17968E-06	0.008760766
1071.079981	432.857585	9.76899E-06	0.010463373
1071.119981	524.491499	1.1837E-05	0.012678893
1071.159981	570.991658	1.28865E-05	0.013803489
1071.199981	584.062999	1.31815E-05	0.01412001
1071.239981	634.953457	1.433E-05	0.015350885
1071.279981	651.539311	1.47043E-05	0.015752459
1071.319981	579.166788	1.3071E-05	0.01400321
1071.359981	489.30547	1.10429E-05	0.011830967
1071.399981	399.657678	9.01972E-06	0.009663725
1071.439981	328.215494	7.40737E-06	0.007936549
1071.479981	271.668365	6.13118E-06	0.006569433
1071.519981	250.780551	5.65977E-06	0.006064554
1071.559981	266.061269	6.00463E-06	0.006434324
1071.599981	263.668542	5.95063E-06	0.006376697
1071.639981	274.057932	6.18511E-06	0.006628207
1071.679981	283.736219	6.40353E-06	0.006862536
1071.719981	300.853015	6.78983E-06	0.0072768
1071.759981	309.210813	6.97846E-06	0.007479231
1071.799981	304.077994	6.86262E-06	0.007355353
1071.839981	293.146516	6.61591E-06	0.007091195

1071.879981	274.761332	6.20098E-06	0.006646707
1071.919981	274.889553	6.20387E-06	0.006650057
1071.959981	296.61489	6.69418E-06	0.007175898
1071.999981	309.731842	6.99022E-06	0.007493512
1072.039981	336.476265	7.5938E-06	0.008140858
1072.079981	401.105473	9.05239E-06	0.009704888
1072.119981	485.380707	1.09544E-05	0.011744395
1072.159981	536.521219	1.21085E-05	0.012982288
1072.199981	578.289764	1.30512E-05	0.01399349
1072.239981	596.283466	1.34573E-05	0.014429441
1072.279981	555.472481	1.25362E-05	0.013442359
1072.319981	488.818004	1.10319E-05	0.011829771
1072.359981	425.316454	9.5988E-06	0.010293369
1072.399981	379.022505	8.55401E-06	0.00917332
1072.43998	310.096661	6.99845E-06	0.007505417
1072.47998	258.772867	5.84014E-06	0.006263437
1072.51998	248.691094	5.61261E-06	0.006019638
1072.55998	265.4749	5.9914E-06	0.006426134
1072.59998	295.341904	6.66546E-06	0.007149367
1072.63998	292.41071	6.5993E-06	0.007078676
1072.67998	294.927902	6.65611E-06	0.007139878
1072.71998	302.164663	6.81944E-06	0.007315345
1072.75998	333.517191	7.52702E-06	0.008074684
1072.79998	330.390322	7.45645E-06	0.007999279
1072.83998	301.08579	6.79509E-06	0.007290041
1072.87998	295.75718	6.67483E-06	0.007161289
1072.91998	299.62635	6.76215E-06	0.007255245
1072.95998	294.289895	6.64171E-06	0.007126292
1072.99998	312.309014	7.04838E-06	0.007562911
1073.03998	347.343356	7.83906E-06	0.00841162
1073.07998	401.451309	9.0602E-06	0.009722316
1073.11998	449.207941	1.0138E-05	0.010879288
1073.15998	513.947518	1.15991E-05	0.012447668
1073.19998	596.858094	1.34703E-05	0.014456278
1073.23998	627.755285	1.41676E-05	0.015205194
1073.27998	615.309455	1.38867E-05	0.014904293
1073.31998	577.980016	1.30442E-05	0.014000605
1073.35998	484.152463	1.09266E-05	0.011728225
1073.39998	385.689086	8.70447E-06	0.009343373
1073.43998	351.154425	7.92507E-06	0.008507083
1073.47998	308.054102	6.95235E-06	0.007463211
1073.51998	271.056198	6.11736E-06	0.006567109
1073.55998	258.879206	5.84254E-06	0.00627232
1073.59998	262.844966	5.93204E-06	0.006368643
1073.63998	285.048148	6.43314E-06	0.006906876
1073.67998	291.075804	6.56918E-06	0.007053192

1073.71998	303.259663	6.84415E-06	0.007348699
1073.75998	306.711633	6.92205E-06	0.007432625
1073.79998	286.102216	6.45693E-06	0.00693345
1073.83998	264.789485	5.97593E-06	0.006417192
1073.87998	268.214849	6.05324E-06	0.006500448
1073.91998	275.398453	6.21536E-06	0.006674799
1073.95998	266.653974	6.01801E-06	0.006463101
1073.99998	278.274861	6.28028E-06	0.006745016
1074.03998	320.242808	7.22743E-06	0.007762553
1074.07998	410.59036	9.26645E-06	0.009952911
1074.11998	508.670539	1.148E-05	0.012330882
1074.15998	551.966372	1.24571E-05	0.013380931
1074.19998	559.136357	1.26189E-05	0.013555253
1074.23998	568.306214	1.28259E-05	0.013778073
1074.27998	571.248047	1.28923E-05	0.01384991
1074.31998	539.492438	1.21756E-05	0.013080483
1074.35998	497.596068	1.12301E-05	0.012065116
1074.39998	437.409357	9.87172E-06	0.010606176
1074.43998	361.968507	8.16913E-06	0.008777235
1074.47998	297.002831	6.70294E-06	0.007202175
1074.51998	267.672768	6.041E-06	0.006491177
1074.55998	277.748216	6.26839E-06	0.006735761
1074.59998	272.961927	6.16037E-06	0.006619934
1074.63998	280.191219	6.32353E-06	0.006795513
1074.67998	311.76541	7.03611E-06	0.007561568
1074.71998	330.808244	7.46588E-06	0.008023732
1074.75998	321.694088	7.26019E-06	0.007802959
1074.79998	329.651082	7.43977E-06	0.00799626
1074.83998	337.227761	7.61076E-06	0.00818035
1074.87998	319.546605	7.21172E-06	0.007751735
1074.91998	309.183568	6.97784E-06	0.007500622
1074.95998	300.549133	6.78298E-06	0.007291427
1074.99998	309.374026	6.98214E-06	0.007505801
1075.03998	375.887821	8.48326E-06	0.009119849
1075.07998	441.445197	9.9628E-06	0.01071081
1075.11998	506.280878	1.14261E-05	0.012284379
1075.15998	592.992292	1.3383E-05	0.014388877
1075.19998	655.530168	1.47944E-05	0.015906942
1075.23998	718.69606	1.622E-05	0.017440359
1075.27998	723.313442	1.63242E-05	0.017553061
1075.31998	633.634106	1.43002E-05	0.015377333
1075.35998	518.928514	1.17115E-05	0.012594072
1075.39998	413.151631	9.32426E-06	0.010027306
1075.43998	330.275766	7.45386E-06	0.008016183
1075.47998	291.382673	6.5761E-06	0.007072465
1075.51998	275.277247	6.21262E-06	0.006681801

1075.55998	259.358664	5.85336E-06	0.006295644
1075.59998	252.949054	5.70871E-06	0.006140286
1075.63998	263.791745	5.95341E-06	0.006403728
1075.67998	262.416085	5.92237E-06	0.00637057
1075.71998	254.136725	5.73551E-06	0.006169805
1075.75998	263.032029	5.93627E-06	0.006385998
1075.79998	274.645655	6.19837E-06	0.006668206
1075.83998	262.614923	5.92685E-06	0.006376345
1075.87998	262.644913	5.92753E-06	0.006377311
1075.91998	270.397715	6.1025E-06	0.006565801
1075.95998	272.486186	6.14963E-06	0.00661676
1075.99998	304.657514	6.8757E-06	0.007398248
1076.03998	330.796479	7.46562E-06	0.008033301
1076.07998	352.411454	7.95344E-06	0.008558533
1076.11998	412.417159	9.30768E-06	0.010016181
1076.15998	491.216356	1.10861E-05	0.011930384
1076.19998	546.516339	1.23341E-05	0.013273972
1076.23998	551.915268	1.2456E-05	0.013405601
1076.27998	534.058206	1.20529E-05	0.012972349
1076.31998	503.77216	1.13694E-05	0.012237151
1076.35998	422.463056	9.5344E-06	0.01026245
1076.39998	373.611819	8.4319E-06	0.009076095
1076.43998	341.358724	7.70399E-06	0.008292884
1076.47998	317.584525	7.16744E-06	0.007715606
1076.51998	302.629758	6.82993E-06	0.007352558
1076.55998	275.790363	6.2242E-06	0.006700729
1076.59998	273.044585	6.16224E-06	0.006634263
1076.63998	279.688693	6.31218E-06	0.00679595
1076.67998	283.822105	6.40547E-06	0.006896641
1076.71998	304.312388	6.86791E-06	0.007394812
1076.75998	316.267339	7.13771E-06	0.007685604
1076.79998	318.648482	7.19145E-06	0.007743756
1076.83998	286.480568	6.46547E-06	0.006962274
1076.87998	259.886327	5.86527E-06	0.006316194
1076.91998	253.171232	5.71372E-06	0.006153221
1076.95998	275.955323	6.22793E-06	0.006707228
1076.99998	325.409855	7.34405E-06	0.007909539
1077.03998	374.364809	8.44889E-06	0.009099795
1077.07998	454.148187	1.02495E-05	0.011039523
1077.11998	525.415069	1.18579E-05	0.012772366
1077.15998	607.68889	1.37147E-05	0.014772917
1077.19998	654.757158	1.4777E-05	0.015917738
1077.23998	631.985741	1.4263E-05	0.015364715
1077.27998	572.402213	1.29183E-05	0.013916648
1077.31998	498.974752	1.12612E-05	0.012131878
1077.35998	442.60193	9.98891E-06	0.010761651

1077.39998	373.519263	8.42981E-06	0.009082277
1077.43998	289.345	6.53011E-06	0.007035805
1077.47998	262.332257	5.92047E-06	0.006379192
1077.51998	267.738979	6.0425E-06	0.00651091
1077.55998	250.739226	5.65884E-06	0.006097734
1077.59998	244.7477	5.52361E-06	0.005952247
1077.63998	265.193406	5.98505E-06	0.006449725
1077.67998	294.756045	6.65223E-06	0.007168979
1077.71998	309.381752	6.98232E-06	0.007524981
1077.75998	301.323789	6.80046E-06	0.007329262
1077.79998	282.734054	6.38091E-06	0.006877349
1077.83998	279.859946	6.31605E-06	0.00680769
1077.87998	288.053464	6.50097E-06	0.007007261
1077.91998	284.392282	6.41834E-06	0.006918454
1077.95998	280.320957	6.32645E-06	0.006819664
1077.99998	286.748018	6.4715E-06	0.00697628
1078.03998	315.652006	7.12383E-06	0.007679769
1078.07998	390.624854	8.81586E-06	0.009504201
1078.11998	465.660538	1.05093E-05	0.011330297
1078.15998	493.055846	1.11276E-05	0.011997315
1078.19998	535.169389	1.2078E-05	0.013022529
1078.23998	539.41101	1.21738E-05	0.01312623
1078.27998	524.987523	1.18482E-05	0.012775717
1078.31998	473.592524	1.06883E-05	0.011525433
1078.35998	410.633569	9.26743E-06	0.009993623
1078.39998	360.275393	8.13091E-06	0.008768377
1078.43998	324.264675	7.3182E-06	0.007892242
1078.47998	274.647093	6.1984E-06	0.006684853
1078.51998	256.993611	5.79999E-06	0.006255403
1078.55998	257.401684	5.8092E-06	0.006265568
1078.59998	279.582866	6.3098E-06	0.006805746
1078.63998	312.994328	7.06385E-06	0.007619347
1078.67998	311.283407	7.02523E-06	0.007577978
1078.71998	295.791296	6.6756E-06	0.0072011
1078.75998	277.791918	6.26938E-06	0.006763153
1078.79998	267.536845	6.03793E-06	0.006513723
1078.83998	250.452211	5.65236E-06	0.006097989
1078.87998	239.844321	5.41295E-06	0.005839926
1078.91998	271.687872	6.13162E-06	0.006615524
1078.95998	297.165233	6.70661E-06	0.007236159
1078.99998	312.064331	7.04286E-06	0.007599243
1079.03998	339.054007	7.65198E-06	0.008256789
1079.07998	399.149936	9.00826E-06	0.009720631
1079.11998	451.878837	1.01983E-05	0.011005163
1079.15998	515.985912	1.16451E-05	0.012566908
1079.19998	580.394328	1.30987E-05	0.014136108

1079.23998	606.082848	1.36784E-05	0.014762325
1079.27998	563.27164	1.27123E-05	0.013720084
1079.31998	510.731124	1.15265E-05	0.012440771
1079.35998	447.41424	1.00975E-05	0.010898855
1079.39998	357.138141	8.06011E-06	0.008700083
1079.43998	284.275437	6.4157E-06	0.006925364
1079.47998	265.077125	5.98242E-06	0.006457904
1079.51998	264.021305	5.95859E-06	0.00643242
1079.55998	257.190911	5.80444E-06	0.006266242
1079.59998	230.782513	5.20844E-06	0.005623032
1079.63998	219.974677	4.96452E-06	0.005359896
1079.67998	262.788416	5.93077E-06	0.006403332
1079.71998	274.493684	6.19494E-06	0.006688801
1079.75998	271.037872	6.11695E-06	0.006604835
1079.79998	281.284814	6.34821E-06	0.006854793
1079.83998	283.192112	6.39125E-06	0.006901529
1079.87998	264.259025	5.96396E-06	0.006440359
1079.91998	241.817834	5.45749E-06	0.005893654
1079.95998	238.896607	5.39156E-06	0.005822673
1079.99998	273.616242	6.17514E-06	0.006669148
1080.03998	308.527408	6.96303E-06	0.007520355
1080.07998	358.338396	8.0872E-06	0.008734821
1080.11998	413.039984	9.32174E-06	0.010068595
1080.15998	470.699685	1.0623E-05	0.011474579
1080.19998	531.405672	1.19931E-05	0.012954931
1080.23998	566.447361	1.27839E-05	0.01380971
1080.27998	543.096367	1.22569E-05	0.013240915
1080.31998	497.67324	1.12318E-05	0.012133929
1080.35998	417.275803	9.41733E-06	0.01017411
1080.39998	331.667398	7.48527E-06	0.008087087
1080.43998	285.382531	6.44069E-06	0.006958775
1080.47998	267.698984	6.04159E-06	0.00652782
1080.51998	269.084758	6.07287E-06	0.006561855
1080.55998	272.407365	6.14785E-06	0.006643126
1080.59998	266.33499	6.01081E-06	0.006495281
1080.63998	279.048848	6.29774E-06	0.006805594
1080.67998	306.26307	6.91193E-06	0.007469585
1080.71998	301.829011	6.81186E-06	0.007361714
1080.75998	277.752493	6.26849E-06	0.00677473
1080.79998	262.360423	5.92111E-06	0.006399535
1080.83998	279.332411	6.30414E-06	0.00681377
1080.87998	299.119925	6.75072E-06	0.007296718
1080.91998	315.151674	7.11253E-06	0.00768808
1080.95998	309.119933	6.97641E-06	0.007541216
1080.99998	346.062055	7.81014E-06	0.00844276
1081.03998	410.997904	9.27565E-06	0.010027349

1081.07998	452.588588	1.02143E-05	0.011042469
1081.11998	516.471132	1.1656E-05	0.012601571
1081.15998	601.956786	1.35853E-05	0.014687911
1081.19998	642.672716	1.45042E-05	0.015681971
1081.23998	639.457786	1.44317E-05	0.0156041
1081.27998	604.980098	1.36536E-05	0.014763319
1081.31998	526.723685	1.18874E-05	0.012854105
1081.35998	461.72358	1.04205E-05	0.011268266
1081.39998	393.998208	8.89199E-06	0.009615799
1081.43998	335.768794	7.57783E-06	0.008194973
1081.47998	318.09941	7.17906E-06	0.00776401
1081.51998	303.317051	6.84544E-06	0.007403484
1081.55998	294.279341	6.64147E-06	0.007183153
1081.59998	293.82012	6.63111E-06	0.007172209
1081.63998	279.445862	6.3067E-06	0.006821583
1081.67998	265.749648	5.9976E-06	0.006487483
1081.71998	255.807217	5.77321E-06	0.006244999
1081.75998	257.328221	5.80754E-06	0.006282364
1081.79998	265.377561	5.9892E-06	0.006479119
1081.83998	270.906317	6.11398E-06	0.006614346
1081.87998	257.955276	5.82169E-06	0.006298371
1081.91998	238.753832	5.38834E-06	0.005829754
1081.95998	236.435201	5.33601E-06	0.005773353
1081.99998	282.452133	6.37455E-06	0.006897264
1082.03998	348.381661	7.86249E-06	0.008507527
1082.07998	411.90213	9.29606E-06	0.010059078
1082.11998	461.138632	1.04073E-05	0.0112619
1082.15998	513.533001	1.15897E-05	0.012541936
1082.19998	579.374471	1.30757E-05	0.014150495
1082.23998	551.196404	1.24397E-05	0.013462779
1082.27998	515.53405	1.16349E-05	0.012592204
1082.31998	496.429825	1.12037E-05	0.01212602
1082.35998	445.25014	1.00487E-05	0.010876284
1082.39998	363.327796	8.1998E-06	0.008875466
1082.43998	301.821019	6.81168E-06	0.007373235
1082.47998	276.158046	6.2325E-06	0.006746559
1082.51998	254.431599	5.74217E-06	0.00621601
1082.55998	259.007955	5.84545E-06	0.006328049
1082.59998	285.791471	6.44992E-06	0.006982678
1082.63998	310.203588	7.00086E-06	0.007579414
1082.67998	339.135661	7.65382E-06	0.008286637
1082.71998	329.494986	7.43624E-06	0.008051369
1082.75998	319.752062	7.21636E-06	0.007813584
1082.79998	319.534955	7.21146E-06	0.007808568
1082.83998	304.362487	6.86904E-06	0.007438068
1082.87998	282.818458	6.38282E-06	0.006911827

1082.91998	291.347493	6.57531E-06	0.007120531
1082.95998	319.893054	7.21954E-06	0.007818474
1082.99998	336.504007	7.59443E-06	0.008224764
1083.03998	363.979157	8.2145E-06	0.008896635
1083.07998	401.835994	9.06888E-06	0.009822321
1083.11998	468.454741	1.05724E-05	0.011451146
1083.15998	589.123948	1.32957E-05	0.014401378
1083.19998	666.425007	1.50403E-05	0.016291635
1083.23998	683.655907	1.54292E-05	0.016713485
1083.27998	644.778494	1.45518E-05	0.015763622
1083.31998	561.192765	1.26653E-05	0.013720615
1083.35998	468.854092	1.05814E-05	0.011463448
1083.39998	400.094078	9.02957E-06	0.009782632
1083.43998	353.829018	7.98543E-06	0.008651732
1083.47998	304.605666	6.87453E-06	0.007448411
1083.51998	254.188842	5.73669E-06	0.006215816
1083.55998	232.870794	5.25557E-06	0.005694725
1083.59998	229.662626	5.18317E-06	0.005616478
1083.63998	247.290622	5.581E-06	0.0060478
1083.67998	306.372207	6.91439E-06	0.00749299
1083.71998	334.577142	7.55094E-06	0.008183105
1083.75998	339.872328	7.67045E-06	0.008312921
1083.79998	315.70717	7.12507E-06	0.007722152
1083.83998	276.90247	6.2493E-06	0.006773244
1083.87998	253.30698	5.71679E-06	0.00619631
1083.91998	246.153932	5.55535E-06	0.006021556
1083.95998	257.29366	5.80676E-06	0.006294295
1083.99998	273.356772	6.16928E-06	0.006687501
1084.03998	306.822263	6.92455E-06	0.00750649
1084.07998	369.238583	8.3332E-06	0.009033856
1084.11998	458.929811	1.03574E-05	0.011228671
1084.15998	521.106234	1.17606E-05	0.012750417
1084.19998	560.482004	1.26493E-05	0.013714369
1084.23998	597.284249	1.34799E-05	0.014615418
1084.27998	545.875766	1.23197E-05	0.013357956
1084.31998	465.794135	1.05123E-05	0.011398724
1084.35998	411.286476	9.28216E-06	0.010065206
1084.39998	349.710843	7.89249E-06	0.008558612
1084.43998	275.058664	6.20769E-06	0.006731868
1084.47998	229.88207	5.18812E-06	0.00562641
1084.51998	231.235201	5.21866E-06	0.005659737
1084.55998	234.956716	5.30265E-06	0.005751037
1084.59998	255.797576	5.77299E-06	0.00626139
1084.63998	303.047457	6.83936E-06	0.007418242
1084.67998	352.164207	7.94786E-06	0.00862088
1084.71998	343.0116	7.74129E-06	0.008397136

1084.75998	343.819626	7.75953E-06	0.008417228
1084.79998	313.872238	7.08366E-06	0.007684353
1084.83998	286.007302	6.45479E-06	0.00700241
1084.87998	270.891348	6.11364E-06	0.006632566
1084.91998	281.619382	6.35576E-06	0.006895488
1084.95998	322.638152	7.28149E-06	0.007900129
1084.99998	361.022214	8.14777E-06	0.008840329
1085.03998	390.283708	8.80816E-06	0.009557205
1085.07998	445.216436	1.00479E-05	0.010902791
1085.11998	532.737302	1.20231E-05	0.013046548
1085.15998	617.70921	1.39408E-05	0.015128038
1085.19998	638.439267	1.44087E-05	0.015636305
1085.23998	639.238779	1.44267E-05	0.015656463
1085.27998	622.318893	1.40449E-05	0.015242617
1085.31998	561.634723	1.26753E-05	0.013756771
1085.35998	478.207093	1.07925E-05	0.011713713
1085.39998	380.466272	8.58659E-06	0.009319889
1085.43998	326.140995	7.36055E-06	0.007989433
1085.47998	297.596926	6.71635E-06	0.007290461
1085.51998	283.907708	6.4074E-06	0.006955362
1085.55998	271.802076	6.13419E-06	0.006659036
1085.59998	286.719678	6.47086E-06	0.00702477
1085.63998	311.7325	7.03537E-06	0.007637877
1085.67998	303.808095	6.85653E-06	0.007443993
1085.71998	296.682083	6.6957E-06	0.007269657
1085.75998	305.189999	6.88771E-06	0.007478403
1085.79998	304.39733	6.86982E-06	0.007459254
1085.83998	286.56896	6.46746E-06	0.007022629
1085.87998	266.855742	6.02256E-06	0.00653978
1085.91998	253.368959	5.71818E-06	0.006209491
1085.95998	271.262577	6.12202E-06	0.006648267
1085.99998	286.632089	6.46889E-06	0.007025211
1086.03998	316.027457	7.1323E-06	0.007745962
1086.07998	362.038305	8.1707E-06	0.008874034
1086.11998	419.907613	9.47673E-06	0.010292866
1086.15998	515.719609	1.16391E-05	0.012641896
1086.19998	588.476504	1.32811E-05	0.014425925
1086.23998	609.056943	1.37456E-05	0.014930984
1086.27998	582.736209	1.31515E-05	0.01428626
1086.31998	550.466736	1.24233E-05	0.013495644
1086.35998	504.236667	1.13799E-05	0.012362689
1086.39998	442.258994	9.98117E-06	0.010843542
1086.43998	380.870001	8.59571E-06	0.009338718
1086.47998	327.555716	7.39248E-06	0.008031777
1086.51998	317.024011	7.15479E-06	0.007773823
1086.55998	308.865575	6.97067E-06	0.007574047

1086.59998	312.574321	7.05437E-06	0.007665275
1086.63998	340.842085	7.69233E-06	0.008358795
1086.67998	358.233374	8.08483E-06	0.008785621
1086.71998	377.216841	8.51326E-06	0.009251528
1086.75998	401.580519	9.06311E-06	0.009849428
1086.79998	392.458501	8.85724E-06	0.00962605
1086.83998	375.928018	8.48417E-06	0.009220937
1086.87998	389.756184	8.79625E-06	0.009560473
1086.91998	425.05255	9.59284E-06	0.010426654
1086.95998	434.759467	9.81192E-06	0.01066516
1086.99998	445.897921	1.00633E-05	0.010938801
1087.03998	493.131828	1.11293E-05	0.012097992
1087.07998	568.342211	1.28267E-05	0.01394364
1087.11998	628.220674	1.41781E-05	0.015413258
1087.15998	671.334614	1.51511E-05	0.016471656
1087.19998	742.835518	1.67648E-05	0.01822665
1087.23998	762.009938	1.71975E-05	0.018697813
1087.27998	736.583602	1.66237E-05	0.018074579
1087.31998	658.15033	1.48535E-05	0.016150547
1087.35998	533.489836	1.20401E-05	0.013091947
1087.39998	424.580599	9.58219E-06	0.010419676
1087.43998	353.267481	7.97275E-06	0.008669893
1087.47998	312.553597	7.0539E-06	0.007670974
1087.51998	295.274489	6.66393E-06	0.007247161
1087.55998	305.221794	6.88843E-06	0.007491582
1087.59998	315.483792	7.12003E-06	0.007743744
1087.63998	302.141497	6.81891E-06	0.007416522
1087.67998	287.984929	6.49942E-06	0.007069288
1087.71998	301.641192	6.80762E-06	0.007404786
1087.75998	321.471627	7.25517E-06	0.00789188
1087.79998	309.301123	6.9805E-06	0.007593383
1087.83998	294.991867	6.65756E-06	0.007242355
1087.87998	327.877397	7.39974E-06	0.008050025
1087.91998	352.70969	7.96017E-06	0.008660024
1087.95998	367.146786	8.28599E-06	0.009014827
1087.99998	390.56853	8.81459E-06	0.009590271
1088.03998	405.469054	9.15087E-06	0.009956514
1088.07998	434.749977	9.8117E-06	0.010675916
1088.11998	500.910199	1.13048E-05	0.012301028
1088.15998	575.698984	1.29927E-05	0.014138162
1088.19998	625.781588	1.4123E-05	0.015368669
1088.23998	636.958366	1.43753E-05	0.015643736
1088.27998	583.909508	1.3178E-05	0.01434138
1088.31998	514.675304	1.16155E-05	0.012641386
1088.35998	445.906793	1.00635E-05	0.010952705
1088.39998	378.518323	8.54263E-06	0.0092978

1088.43998	338.89947	7.64849E-06	0.008324921
1088.47998	300.661378	6.78551E-06	0.00738589
1088.51998	281.432509	6.35154E-06	0.006913778
1088.55998	279.820546	6.31516E-06	0.00687443
1088.59998	270.699311	6.10931E-06	0.006650591
1088.63998	269.825952	6.0896E-06	0.006629378
1088.67998	289.120122	6.52504E-06	0.007103679
1088.71998	316.028053	7.13231E-06	0.007765092
1088.75998	315.213562	7.11393E-06	0.007745363
1088.79998	332.938729	7.51396E-06	0.008181203
1088.83998	360.505113	8.1361E-06	0.008858909
1088.87998	366.397963	8.26909E-06	0.009004048
1088.91998	347.830476	7.85005E-06	0.008548076
1088.95998	349.317685	7.88361E-06	0.00858494
1088.99998	379.657184	8.56833E-06	0.009330915
1089.03998	437.809062	9.88074E-06	0.010760522
1089.07998	542.476938	1.22429E-05	0.013333551
1089.11998	620.349846	1.40004E-05	0.01524815
1089.15998	674.148931	1.52146E-05	0.016571136
1089.19998	704.814535	1.59067E-05	0.017325558
1089.23998	685.656459	1.54743E-05	0.016855238
1089.27998	653.327929	1.47447E-05	0.016061108
1089.31998	587.82042	1.32663E-05	0.014451233
1089.35998	498.874871	1.12589E-05	0.012265007
1089.39998	403.025034	9.09571E-06	0.00990887
1089.43998	326.665646	7.37239E-06	0.008031775
1089.47998	296.349829	6.6882E-06	0.007286663
1089.51998	295.047942	6.65882E-06	0.007254919
1089.55998	316.690335	7.14726E-06	0.007787368
1089.59998	318.556411	7.18937E-06	0.007833542
1089.63998	294.242872	6.64065E-06	0.00723592
1089.67998	285.078466	6.43382E-06	0.007010809
1089.71998	281.686774	6.35728E-06	0.006927653
1089.75998	264.330539	5.96557E-06	0.006501042
1089.79998	248.531069	5.609E-06	0.006112688
1089.83998	243.325226	5.49151E-06	0.005984869
1089.87998	268.273071	6.05455E-06	0.006598732
1089.91998	298.53636	6.73755E-06	0.00734339
1089.95998	305.857732	6.90278E-06	0.007523757
1089.99998	325.519638	7.34652E-06	0.008007712
1090.03998	369.896568	8.34805E-06	0.009099709
1090.07998	405.775126	9.15778E-06	0.009982712
1090.11998	428.724589	9.67572E-06	0.010547692
1090.15998	483.482715	1.09115E-05	0.011895315
1090.19998	513.636963	1.15921E-05	0.012637675
1090.23998	538.99334	1.21643E-05	0.013262038

1090.27998	540.67837	1.22024E-05	0.013303986
1090.31998	517.148473	1.16713E-05	0.012725474
1090.35998	469.15247	1.05881E-05	0.01154486
1090.39998	397.381567	8.96835E-06	0.009779087
1090.43998	342.9267	7.73938E-06	0.008439327
1090.47998	288.919378	6.52051E-06	0.007110483
1090.51998	249.536081	5.63168E-06	0.006141461
1090.55998	271.680222	6.13144E-06	0.006686708
1090.59998	308.668858	6.96623E-06	0.007597366
1090.63998	311.930456	7.03984E-06	0.007677926
1090.67998	313.056984	7.06526E-06	0.007705938
1090.71998	300.214147	6.77542E-06	0.007390081
1090.75998	289.955816	6.5439E-06	0.007137823
1090.79998	291.652059	6.58218E-06	0.007179843
1090.83998	281.037651	6.34263E-06	0.006918793
1090.87998	275.411627	6.21566E-06	0.006780536
1090.91998	269.54876	6.08334E-06	0.006636437
1090.95998	298.758	6.74255E-06	0.007355854
1090.99998	343.680619	7.75639E-06	0.008462224
1091.03998	383.822659	8.66234E-06	0.009450962
1091.07998	416.36043	9.39667E-06	0.010252524
1091.11998	475.672466	1.07353E-05	0.011713462
1091.15998	535.502269	1.20855E-05	0.013187258
1091.19998	585.30144	1.32094E-05	0.014414139
1091.23998	624.839468	1.41018E-05	0.0153884
1091.27998	627.427774	1.41602E-05	0.015452711
1091.31998	546.692137	1.23381E-05	0.013464793
1091.35998	449.31423	1.01404E-05	0.011066823
1091.39998	380.902166	8.59643E-06	0.009382145
1091.43998	330.417248	7.45706E-06	0.00813893
1091.47998	286.759132	6.47175E-06	0.00706379
1091.51998	240.690089	5.43204E-06	0.00592918
1091.55998	239.599263	5.40742E-06	0.005902525
1091.59998	272.988114	6.16096E-06	0.006725305
1091.63998	282.145963	6.36764E-06	0.006951172
1091.67998	304.870333	6.8805E-06	0.007511303
1091.71998	329.123849	7.42787E-06	0.008109151
1091.75998	324.19894	7.31672E-06	0.0079881
1091.79998	300.298605	6.77732E-06	0.007399479
1091.83998	274.228823	6.18896E-06	0.006757357
1091.87998	262.249688	5.91861E-06	0.006462412
1091.91998	271.915731	6.13676E-06	0.00670085
1091.95998	255.468735	5.76557E-06	0.006295776
1091.99998	253.408076	5.71907E-06	0.006245221
1092.03998	275.953835	6.22789E-06	0.006801109
1092.07998	303.795729	6.85625E-06	0.007487569

1092.11998	342.843655	7.7375E-06	0.008450282
1092.15998	389.978834	8.80128E-06	0.009612405
1092.19998	464.318511	1.0479E-05	0.011445188
1092.23998	496.33872	1.12017E-05	0.012234916
1092.27998	472.082015	1.06542E-05	0.011637406
1092.31998	464.089826	1.04739E-05	0.011440808
1092.35998	451.343466	1.01862E-05	0.01112699
1092.39998	400.489912	9.0385E-06	0.009873657
1092.43998	339.631815	7.66502E-06	0.008373571
1092.47998	287.5397	6.48937E-06	0.007089507
1092.51998	274.757731	6.2009E-06	0.006774606
1092.55998	279.924197	6.3175E-06	0.006902247
1092.59998	280.643881	6.33374E-06	0.006920246
1092.63998	293.56085	6.62526E-06	0.007239023
1092.67998	322.978214	7.28917E-06	0.007964728
1092.71998	325.762844	7.35201E-06	0.008033692
1092.75998	285.655136	6.44684E-06	0.007044847
1092.79998	236.091411	5.32825E-06	0.005822716
1092.83998	231.727951	5.22978E-06	0.005715309
1092.87998	229.60239	5.18181E-06	0.005663092
1092.91998	245.057668	5.53061E-06	0.006044514
1092.95998	296.103847	6.68265E-06	0.007303871
1092.99998	358.228763	8.08472E-06	0.008836603
1093.03998	379.331969	8.56099E-06	0.009357509
1093.07998	383.363933	8.65199E-06	0.009457317
1093.11998	429.154026	9.68541E-06	0.010587314
1093.15998	482.694916	1.08938E-05	0.011908614
1093.19998	501.229459	1.13121E-05	0.012366334
1093.23998	538.909407	1.21624E-05	0.01329646
1093.27998	564.889108	1.27488E-05	0.013937964
1093.31998	526.408454	1.18803E-05	0.012988975
1093.35998	460.677641	1.03969E-05	0.011367503
1093.39998	385.283277	8.69531E-06	0.009507448
1093.43998	329.742721	7.44183E-06	0.008137199
1093.47998	290.573746	6.55784E-06	0.007170872
1093.51998	265.343168	5.98843E-06	0.006548463
1093.55998	244.338263	5.51437E-06	0.006030299
1093.59998	241.759125	5.45617E-06	0.005966864
1093.63998	269.339934	6.07863E-06	0.00664783
1093.67998	272.033021	6.13941E-06	0.006714546
1093.71998	257.628381	5.81431E-06	0.006359231
1093.75998	277.140191	6.25467E-06	0.006841106
1093.79998	285.723273	6.44838E-06	0.007053234
1093.83998	263.011342	5.9358E-06	0.006492815
1093.87998	234.688236	5.29659E-06	0.00579383
1093.91998	228.108343	5.14809E-06	0.005631596

1093.95998	255.564352	5.76773E-06	0.006309667
1093.99998	284.278795	6.41578E-06	0.007018859
1094.03998	310.808691	7.01452E-06	0.007674164
1094.07998	361.730706	8.16376E-06	0.008931804
1094.11998	414.67125	9.35855E-06	0.010239379
1094.15998	463.988271	1.04716E-05	0.011457572
1094.19998	497.922865	1.12374E-05	0.012295991
1094.23998	486.282498	1.09747E-05	0.012008976
1094.27998	478.473668	1.07985E-05	0.011816565
1094.31998	439.627959	9.92179E-06	0.010857614
1094.35998	417.812771	9.42945E-06	0.010319215
1094.39998	365.441985	8.24752E-06	0.009026082
1094.43998	294.558967	6.64779E-06	0.007275602
1094.47998	262.334638	5.92053E-06	0.006479899
1094.51998	277.251165	6.25717E-06	0.0068486
1094.55998	272.349702	6.14655E-06	0.006727771
1094.59998	241.279635	5.44535E-06	0.005960475
1094.63998	245.188029	5.53355E-06	0.006057248
1094.67998	264.05331	5.95932E-06	0.006523543
1094.71998	277.218376	6.25643E-06	0.006849042
1094.75998	290.721343	6.56118E-06	0.007182913
1094.79998	292.99976	6.6126E-06	0.00723947
1094.83998	286.336282	6.46221E-06	0.007075087
1094.87998	298.424587	6.73503E-06	0.007374047
1094.91998	318.014946	7.17715E-06	0.00785841
1094.95998	350.283848	7.90542E-06	0.008656117
1094.99998	363.610253	8.20618E-06	0.008985764
1095.03998	338.491494	7.63928E-06	0.008365319
1095.07998	377.223776	8.51342E-06	0.009322871
1095.11998	459.377155	1.03675E-05	0.011353659
1095.15998	506.212507	1.14245E-05	0.012511667
1095.19998	533.334351	1.20366E-05	0.013182499
1095.23998	562.673743	1.26988E-05	0.013908193
1095.27998	556.426592	1.25578E-05	0.013754278
1095.31998	473.435658	1.06848E-05	0.011703257
1095.35998	429.357307	9.69E-06	0.010614034
1095.39998	385.940362	8.71014E-06	0.009541083
1095.43998	348.010747	7.85412E-06	0.008603715
1095.47998	322.933073	7.28815E-06	0.007984022
1095.51998	284.258096	6.41531E-06	0.0070281
1095.55998	262.674352	5.92819E-06	0.006494692
1095.59998	267.508615	6.0373E-06	0.006614462
1095.63998	271.583873	6.12927E-06	0.006715473
1095.67998	259.371066	5.85364E-06	0.00641372
1095.71998	258.657776	5.83755E-06	0.006396315
1095.75998	267.777768	6.04337E-06	0.006622084

1095.79998	256.81548	5.79597E-06	0.006351221
1095.83998	242.172863	5.4655E-06	0.005989318
1095.87998	232.849958	5.2551E-06	0.005758958
1095.91998	233.300575	5.26527E-06	0.005770314
1095.95998	229.208936	5.17293E-06	0.00566932
1095.99998	238.955053	5.39288E-06	0.005910599
1096.03998	300.588223	6.78386E-06	0.007435379
1096.07998	375.744965	8.48004E-06	0.009294803
1096.11998	416.269058	9.39461E-06	0.010297623
1096.15998	456.876474	1.03111E-05	0.011302578
1096.19998	495.053995	1.11727E-05	0.012247491
1096.23998	490.996312	1.10811E-05	0.012147548
1096.27998	464.462508	1.04823E-05	0.011491505
1096.31998	422.989286	9.54628E-06	0.010465776
1096.35998	393.765535	8.88674E-06	0.009743066
1096.39998	362.573355	8.18278E-06	0.008971595
1096.43998	316.078762	7.13346E-06	0.007821408
1096.47998	269.242754	6.07643E-06	0.006662688
1096.51998	243.848109	5.50331E-06	0.006034492
1096.55998	220.183881	4.96924E-06	0.005449074
1096.59998	225.803268	5.09607E-06	0.005588345
1096.63998	246.094572	5.55401E-06	0.006090751
1096.67998	238.718797	5.38755E-06	0.005908419
1096.71998	216.366894	4.8831E-06	0.005355393
1096.75998	214.916808	4.85037E-06	0.005319695
1096.79998	227.612905	5.13691E-06	0.005634159
1096.83998	255.23831	5.76037E-06	0.006318208
1096.87998	265.858681	6.00006E-06	0.006581346
1096.91998	275.649804	6.22103E-06	0.006823974
1096.95998	303.56711	6.85109E-06	0.007515368
1096.99998	342.880667	7.73834E-06	0.008488958
1097.03998	372.791593	8.41339E-06	0.009229822
1097.07998	389.999853	8.80175E-06	0.009656228
1097.11998	454.751431	1.02631E-05	0.011259859
1097.15998	551.309224	1.24423E-05	0.013651173
1097.19998	586.594733	1.32386E-05	0.01452542
1097.23998	585.935371	1.32237E-05	0.014509622
1097.27998	562.54307	1.26958E-05	0.013930862
1097.31998	511.022848	1.15331E-05	0.012655472
1097.35998	463.80764	1.04675E-05	0.011486607
1097.39998	401.998614	9.07255E-06	0.009956215
1097.43998	315.869688	7.12874E-06	0.007823363
1097.47998	262.784164	5.93067E-06	0.006508794
1097.51998	270.485668	6.10448E-06	0.006699794
1097.55998	280.129116	6.32212E-06	0.00693891
1097.59998	285.252182	6.43774E-06	0.007066068

1097.63998	293.973145	6.63456E-06	0.007282363
1097.67998	288.924251	6.52062E-06	0.007157552
1097.71998	265.627114	5.99483E-06	0.006580649
1097.75998	248.2658	5.60301E-06	0.006150764
1097.79998	242.548626	5.47398E-06	0.00600934
1097.83998	261.923093	5.91124E-06	0.006489595
1097.87998	278.617482	6.28801E-06	0.006903479
1097.91998	284.268596	6.41555E-06	0.007043756
1097.95998	276.9955	6.2514E-06	0.00686379
1097.99998	286.211426	6.45939E-06	0.007092414
1098.03998	321.11164	7.24704E-06	0.007957542
1098.07998	368.243441	8.31074E-06	0.009125859
1098.11998	429.585058	9.69514E-06	0.010646423
1098.15998	464.998641	1.04944E-05	0.011524499
1098.19998	488.728134	1.10299E-05	0.012113051
1098.23998	513.048161	1.15788E-05	0.012716282
1098.27998	495.178069	1.11755E-05	0.012273805
1098.31998	477.429918	1.07749E-05	0.011834319
1098.35998	447.60083	1.01017E-05	0.011095333
1098.39998	406.311718	9.16989E-06	0.010072206
1098.43998	358.932506	8.10061E-06	0.00889803
1098.47998	326.478463	7.36816E-06	0.008093781
1098.51998	304.803539	6.87899E-06	0.007556709
1098.55998	291.925859	6.58836E-06	0.007237709
1098.59998	284.852431	6.42872E-06	0.007062595
1098.63998	293.069398	6.61417E-06	0.00726659
1098.67998	313.769964	7.08135E-06	0.007780139
1098.71998	325.558256	7.3474E-06	0.008072731
1098.75998	336.378747	7.5916E-06	0.008341346
1098.79998	346.722145	7.82504E-06	0.008598149
1098.83998	343.982841	7.76321E-06	0.008530529
1098.87998	315.198592	7.11359E-06	0.007816985
1098.91998	271.671562	6.13125E-06	0.006737752
1098.95998	262.969134	5.93485E-06	0.006522159
1098.99998	297.20448	6.70749E-06	0.007371533
1099.03998	331.743809	7.487E-06	0.008228508
1099.07998	404.526498	9.1296E-06	0.01003416
1099.11998	476.213919	1.07475E-05	0.011812775
1099.15998	525.230904	1.18537E-05	0.013029146
1099.19998	588.638373	1.32847E-05	0.014602595
1099.23998	619.336519	1.39776E-05	0.015364696
1099.27998	570.83861	1.2883E-05	0.01416206
1099.31998	483.374883	1.09091E-05	0.011992589
1099.35998	420.52798	9.49073E-06	0.01043373
1099.39998	376.467877	8.49636E-06	0.009340893
1099.43998	342.966887	7.74028E-06	0.008509979

1099.47998	326.200957	7.3619E-06	0.008094263
1099.51998	278.316507	6.28122E-06	0.006906322
1099.55998	239.367193	5.40218E-06	0.005940025
1099.59998	233.47309	5.26916E-06	0.005793971
1099.63998	228.611668	5.15945E-06	0.005673534
1099.67998	236.611552	5.33999E-06	0.005872284
1099.71998	250.863025	5.66163E-06	0.006226207
1099.75998	244.149531	5.51011E-06	0.006059804
1099.79998	232.549483	5.24832E-06	0.0057721
1099.83998	209.777053	4.73438E-06	0.005207056
1099.87998	211.1308	4.76493E-06	0.005240849
1099.91998	219.199696	4.94703E-06	0.005441339
1099.95998	235.47343	5.31431E-06	0.005845525
1099.99998	298.783478	6.74313E-06	0.007417439
1100.03998	337.440605	7.61556E-06	0.008377425
1100.07998	345.129141	7.78908E-06	0.008568615
1100.11998	394.883506	8.91197E-06	0.009804237
1100.15998	452.016777	1.02014E-05	0.01122316
1100.19998	478.047799	1.07889E-05	0.011869918
1100.23998	488.510156	1.1025E-05	0.012130139
1100.27998	467.3339	1.05471E-05	0.011604736
1100.31998	433.04583	9.77324E-06	0.010753693
1100.35998	385.389697	8.69771E-06	0.009570611
1100.39998	338.876891	7.64798E-06	0.008415836
1100.43998	306.682079	6.92139E-06	0.007616571
1100.47998	291.988861	6.58978E-06	0.007251923
1100.51998	283.851626	6.40614E-06	0.00705008
1100.55998	290.062618	6.54631E-06	0.007204606
1100.59998	293.017735	6.613E-06	0.00727827
1100.63998	285.252913	6.43776E-06	0.007085657
1100.67998	305.963093	6.90516E-06	0.007600372
1100.71998	330.356395	7.45568E-06	0.00820662
1100.75998	338.600337	7.64174E-06	0.008411719
1100.79998	338.349089	7.63607E-06	0.008405783
1100.83998	310.35701	7.00433E-06	0.007710641
1100.87998	281.194115	6.34616E-06	0.00698636
1100.91998	295.364708	6.66597E-06	0.0073387
1100.95998	330.538528	7.45979E-06	0.008212935
1100.99998	365.565435	8.2503E-06	0.009083583
1101.03998	405.515737	9.15193E-06	0.010076636
1101.07998	434.243471	9.80027E-06	0.010790881
1101.11998	503.140598	1.13552E-05	0.012503418
1101.15998	582.221539	1.31399E-05	0.014469164
1101.19998	612.981326	1.38341E-05	0.015234149
1101.23998	601.142094	1.35669E-05	0.014940456
1101.27998	552.807045	1.24761E-05	0.013739663

1101.31998	486.71045	1.09844E-05	0.012097314
1101.35998	436.968496	9.86177E-06	0.010861359
1101.39998	375.425173	8.47282E-06	0.009331967
1101.43998	316.515585	7.14332E-06	0.007867934
1101.47998	282.555078	6.37687E-06	0.007024
1101.51998	246.205256	5.55651E-06	0.006120606
1101.55998	247.456121	5.58474E-06	0.006151926
1101.59998	263.868377	5.95514E-06	0.006560184
1101.63998	283.595765	6.40036E-06	0.007050894
1101.67998	304.503266	6.87221E-06	0.007570981
1101.71998	297.823355	6.72146E-06	0.007405165
1101.75998	291.906668	6.58793E-06	0.007258314
1101.79998	307.703935	6.94445E-06	0.007651394
1101.83998	307.219777	6.93352E-06	0.007639632
1101.87998	311.074511	7.02052E-06	0.007735769
1101.91998	309.64352	6.98822E-06	0.007700462
1101.95998	308.384044	6.9598E-06	0.007669419
1101.99998	313.671956	7.07914E-06	0.007801211
1102.03998	338.384956	7.63688E-06	0.008416144
1102.07998	388.558884	8.76923E-06	0.009664396
1102.11998	457.50756	1.03253E-05	0.011379729
1102.15998	528.737249	1.19329E-05	0.013151924
1102.19998	580.608121	1.31035E-05	0.014442696
1102.23998	579.693743	1.30829E-05	0.014420474
1102.27998	574.62741	1.29685E-05	0.014294962
1102.31998	517.769272	1.16853E-05	0.012880974
1102.35998	427.07893	9.63858E-06	0.010625181
1102.39998	373.663513	8.43307E-06	0.009296611
1102.43998	349.693249	7.89209E-06	0.008700555
1102.47998	313.453855	7.07422E-06	0.007799183
1102.51998	292.980246	6.61216E-06	0.007290034
1102.55998	294.042199	6.63612E-06	0.007316723
1102.59998	288.818045	6.51822E-06	0.00718699
1102.63998	314.076071	7.08826E-06	0.007815798
1102.67998	337.382779	7.61426E-06	0.008396091
1102.71998	344.624957	7.77771E-06	0.008576631
1102.75998	333.672622	7.53053E-06	0.008304363
1102.79998	288.645025	6.51432E-06	0.007183988
1102.83998	272.932017	6.1597E-06	0.006793158
1102.87998	286.24438	6.46014E-06	0.007124756
1102.91998	286.287527	6.46111E-06	0.007126088
1102.95998	313.428361	7.07364E-06	0.007801944
1102.99998	348.274325	7.86007E-06	0.008669653
1103.03998	381.141327	8.60183E-06	0.009488161
1103.07998	428.798046	9.67737E-06	0.010674918
1103.11998	477.567149	1.0778E-05	0.011889455

1103.15998	520.826326	1.17543E-05	0.012966901
1103.19998	555.794424	1.25435E-05	0.013837996
1103.23998	573.032701	1.29325E-05	0.014267706
1103.27998	546.165403	1.23262E-05	0.013599241
1103.31998	509.429595	1.14971E-05	0.012684998
1103.35998	460.053292	1.03828E-05	0.011455924
1103.39998	378.194743	8.53533E-06	0.009417881
1103.43998	334.72032	7.55417E-06	0.008335575
1103.47998	307.262986	6.9345E-06	0.007652079
1103.51998	286.903261	6.47501E-06	0.0071453
1103.55998	283.677298	6.4022E-06	0.007065213
1103.59998	267.558914	6.03843E-06	0.006664013
1103.63998	240.783061	5.43414E-06	0.005997332
1103.67998	254.934885	5.75353E-06	0.006350051
1103.71998	272.420151	6.14814E-06	0.006785829
1103.75998	272.058369	6.13998E-06	0.006777062
1103.79998	268.434694	6.0582E-06	0.006687038
1103.83998	264.588014	5.97138E-06	0.006591451
1103.87998	267.29597	6.0325E-06	0.006659153
1103.91998	254.70389	5.74831E-06	0.006345676
1103.95998	243.723309	5.5005E-06	0.006072327
1103.99998	255.989303	5.77732E-06	0.006378163
1104.03998	295.573753	6.67069E-06	0.007364706
1104.07998	345.814594	7.80455E-06	0.008616852
1104.11998	389.488883	8.79022E-06	0.009705459
1104.15998	447.978074	1.01102E-05	0.011163324
1104.19998	507.661981	1.14572E-05	0.012651066
1104.23998	541.490177	1.22207E-05	0.013494562
1104.27998	535.630337	1.20884E-05	0.013349012
1104.31998	480.73299	1.08495E-05	0.011981291
1104.35998	417.526921	9.423E-06	0.010406385
1104.39998	354.415042	7.99865E-06	0.008833713
1104.43998	312.957145	7.06301E-06	0.007800667
1104.47998	324.46231	7.32266E-06	0.008087734
1104.51998	313.893467	7.08414E-06	0.007824572
1104.55998	265.774707	5.99816E-06	0.006625333
1104.59998	242.756711	5.47868E-06	0.006051751
1104.63998	244.24537	5.51228E-06	0.006089082
1104.67998	257.07471	5.80182E-06	0.006409152
1104.71998	272.626821	6.15281E-06	0.00679713
1104.75998	279.682337	6.31204E-06	0.00697329
1104.79998	279.159767	6.30025E-06	0.006960513
1104.83998	304.129981	6.86379E-06	0.00758339
1104.87998	314.718788	7.10276E-06	0.007847702
1104.91998	295.814827	6.67613E-06	0.007376588
1104.95998	297.523522	6.71469E-06	0.007419465

1104.99998	335.382758	7.56912E-06	0.008363879
1105.03998	373.508725	8.42957E-06	0.009315014
1105.07998	391.54613	8.83665E-06	0.009765206
1105.11998	417.579319	9.42418E-06	0.010414854
1105.15998	454.566091	1.02589E-05	0.011337752
1105.19998	509.572598	1.15003E-05	0.01271018
1105.23998	552.143394	1.24611E-05	0.013772514
1105.27998	544.414228	1.22867E-05	0.013580211
1105.31998	515.195306	1.16272E-05	0.012851821
1105.35998	442.622706	9.98938E-06	0.011041859
1105.39998	375.580603	8.47633E-06	0.009369736
1105.43998	327.116818	7.38257E-06	0.008160989
1105.47998	289.289717	6.52887E-06	0.007217531
1105.51998	255.412096	5.7643E-06	0.006372544
1105.55998	226.325169	5.10784E-06	0.005647028
1105.59998	223.565693	5.04557E-06	0.005578378
1105.63998	240.380547	5.42505E-06	0.005998157
1105.67998	246.65408	5.56664E-06	0.006154921
1105.71998	265.109557	5.98315E-06	0.006615692
1105.75998	292.549936	6.60244E-06	0.007300719
1105.79998	276.632115	6.2432E-06	0.006903732
1105.83998	223.391313	5.04163E-06	0.005575237
1105.87998	222.761348	5.02741E-06	0.005559716
1105.91998	249.577157	5.63261E-06	0.006229215
1105.95998	265.567238	5.99348E-06	0.006628552
1105.99998	286.664682	6.46962E-06	0.007155402
1106.03998	313.609881	7.07774E-06	0.007828261
1106.07998	362.173827	8.17376E-06	0.009040831
1106.11998	404.949953	9.13916E-06	0.010109003
1106.15998	425.823645	9.61025E-06	0.01063047
1106.19998	463.591805	1.04626E-05	0.011573751
1106.23998	481.070532	1.08571E-05	0.012010549
1106.27998	454.358059	1.02542E-05	0.011344048
1106.31998	409.577576	9.2436E-06	0.010226374
1106.35998	364.896879	8.23521E-06	0.009111112
1106.39998	320.347299	7.22979E-06	0.007999042
1106.43998	264.945845	5.97946E-06	0.006615912
1106.47998	256.485871	5.78853E-06	0.006404891
1106.51998	284.537268	6.42161E-06	0.00710564
1106.55998	299.274436	6.75421E-06	0.007473935
1106.59998	308.273541	6.9573E-06	0.007698953
1106.63998	310.632434	7.01054E-06	0.007758145
1106.67998	296.954142	6.70184E-06	0.007416794
1106.71998	285.528848	6.44399E-06	0.007131691
1106.75998	267.441683	6.03579E-06	0.006680167
1106.79998	260.34512	5.87563E-06	0.006503144

1106.83998	244.793097	5.52464E-06	0.006114892
1106.87998	242.98703	5.48388E-06	0.006069996
1106.91998	271.619435	6.13007E-06	0.0067855
1106.95998	288.081196	6.50159E-06	0.007197001
1106.99998	306.519599	6.91772E-06	0.007657916
1107.03998	334.271723	7.54405E-06	0.008351562
1107.07998	372.946926	8.41689E-06	0.009318173
1107.11998	425.354817	9.59967E-06	0.010627982
1107.15998	482.116195	1.08807E-05	0.012046666
1107.19998	540.247703	1.21926E-05	0.013499689
1107.23998	558.197941	1.25978E-05	0.013948732
1107.27998	521.617785	1.17722E-05	0.013035107
1107.31998	467.362459	1.05477E-05	0.011679701
1107.35998	408.199617	9.2125E-06	0.01020155
1107.39998	368.419569	8.31472E-06	0.009207717
1107.43998	333.801888	7.53344E-06	0.008342837
1107.47998	280.103663	6.32155E-06	0.00700099
1107.51998	251.7288	5.68117E-06	0.006292007
1107.55998	258.33936	5.83036E-06	0.006457473
1107.59998	262.237556	5.91834E-06	0.006555149
1107.63998	263.071652	5.93716E-06	0.006576237
1107.67998	260.20342	5.87243E-06	0.006504772
1107.71998	260.234229	5.87312E-06	0.006505777
1107.75998	255.589903	5.76831E-06	0.006389901
1107.79998	241.551304	5.45148E-06	0.006039146
1107.83998	245.516628	5.54097E-06	0.006138506
1107.87998	272.693981	6.15432E-06	0.006818252
1107.91998	294.809997	6.65345E-06	0.007371491
1107.95998	295.010804	6.65798E-06	0.007376779
1107.99998	304.289788	6.8674E-06	0.007609075
1108.03998	352.663387	7.95912E-06	0.008819025
1108.07998	395.686867	8.9301E-06	0.009895267
1108.11998	417.847049	9.43023E-06	0.010449822
1108.15998	430.982814	9.72668E-06	0.01077872
1108.19998	456.515716	1.03029E-05	0.0114177
1108.23998	462.686172	1.04422E-05	0.011572444
1108.27998	469.201229	1.05892E-05	0.011735819
1108.31998	460.778298	1.03991E-05	0.011525557
1108.35998	414.606314	9.35709E-06	0.010371021
1108.39998	354.739782	8.00598E-06	0.008873831
1108.43998	307.375862	6.93705E-06	0.007689298
1108.47998	294.591885	6.64853E-06	0.007369761
1108.51998	250.798943	5.66018E-06	0.006274426
1108.55998	215.564967	4.865E-06	0.005393145
1108.59998	241.642807	5.45354E-06	0.006045796
1108.63998	285.008721	6.43225E-06	0.007131049

1108.67998	288.294784	6.50641E-06	0.007213528
1108.71998	266.182188	6.00736E-06	0.006660481
1108.75998	259.415313	5.85464E-06	0.006491393
1108.79998	270.23977	6.09894E-06	0.006762499
1108.83998	300.553067	6.78306E-06	0.007521333
1108.87998	305.397001	6.89238E-06	0.007642828
1108.91998	263.900572	5.95587E-06	0.006604581
1108.95998	257.456306	5.81043E-06	0.006443535
1108.99998	294.82734	6.65384E-06	0.007379111
1109.03998	339.471047	7.66139E-06	0.008496786
1109.07998	364.414375	8.22432E-06	0.009121434
1109.11998	419.230286	9.46144E-06	0.010493876
1109.15998	481.700537	1.08713E-05	0.012058022
1109.19998	511.330344	1.154E-05	0.012800183
1109.23998	551.182138	1.24394E-05	0.013798294
1109.27998	581.648979	1.3127E-05	0.014561526
1109.31998	547.064866	1.23465E-05	0.01369621
1109.35998	459.172868	1.03629E-05	0.011496178
1109.39998	383.970907	8.66569E-06	0.009613715
1109.43998	343.636259	7.75539E-06	0.008604142
1109.47998	295.622009	6.67178E-06	0.007402203
1109.51998	263.161668	5.93919E-06	0.006589653
1109.55998	261.69015	5.90598E-06	0.006553041
1109.59998	261.596153	5.90386E-06	0.006550924
1109.63998	268.624631	6.06248E-06	0.006727174
1109.67998	272.428899	6.14834E-06	0.006822691
1109.71998	264.560895	5.97077E-06	0.006625884
1109.75998	266.587083	6.0165E-06	0.00667687
1109.79998	249.51685	5.63125E-06	0.006249559
1109.83998	238.133246	5.37434E-06	0.005964653
1109.87998	243.085135	5.48609E-06	0.006088905
1109.91998	232.041118	5.23684E-06	0.005812479
1109.95998	231.419363	5.22281E-06	0.005797113
1109.99998	230.968268	5.21263E-06	0.005786022
1110.03998	255.373381	5.76342E-06	0.006397628
1110.07998	315.373406	7.11754E-06	0.007901037
1110.11998	367.325128	8.29002E-06	0.009202913
1110.15998	396.59689	8.95064E-06	0.009936642
1110.19998	428.126682	9.66222E-06	0.010727
1110.23998	464.838471	1.04908E-05	0.011647258
1110.27998	473.30984	1.06819E-05	0.011859948
1110.31998	439.063498	9.90905E-06	0.011002218
1110.35998	371.678279	8.38826E-06	0.009313989
1110.39998	322.383061	7.27574E-06	0.008078978
1110.43998	287.145489	6.48047E-06	0.007196177
1110.47998	256.523656	5.78938E-06	0.006428992

1110.51998	237.217624	5.35367E-06	0.005945359
1110.55998	250.538889	5.65431E-06	0.006279455
1110.59998	274.302983	6.19064E-06	0.00687532
1110.63998	299.485454	6.75897E-06	0.007506782
1110.67998	288.726646	6.51616E-06	0.007237367
1110.71998	266.23684	6.00859E-06	0.006673866
1110.75998	265.495678	5.99187E-06	0.006655527
1110.79998	293.606703	6.62629E-06	0.007360488
1110.83998	306.558256	6.91859E-06	0.00768545
1110.87998	263.591244	5.94889E-06	0.0066085
1110.91998	235.244026	5.30913E-06	0.005898019
1110.95998	263.833188	5.95435E-06	0.006615042
1110.99998	319.559548	7.21201E-06	0.008012547
1111.03998	374.115382	8.44326E-06	0.009380803
1111.07998	418.252588	9.43938E-06	0.010487904
1111.11998	485.667527	1.09608E-05	0.012178808
1111.15998	521.500448	1.17695E-05	0.01307784
1111.19998	539.721027	1.21808E-05	0.013535251
1111.23998	559.233143	1.26211E-05	0.014025085
1111.27998	558.895409	1.26135E-05	0.01401712
1111.31998	519.025116	1.17137E-05	0.01301764
1111.35998	446.60313	1.00792E-05	0.011201631
1111.39998	376.665309	8.50081E-06	0.009447802
1111.43998	320.982773	7.24413E-06	0.00805142
1111.47998	291.707912	6.58344E-06	0.007317363
1111.51998	279.736991	6.31327E-06	0.00701733
1111.55998	265.868682	6.00029E-06	0.006669678
1111.59998	246.508728	5.56336E-06	0.006184229
1111.63998	235.924022	5.32448E-06	0.005918901
1111.67998	228.353866	5.15363E-06	0.005729186
1111.71998	225.328508	5.08535E-06	0.005653486
1111.75998	231.539432	5.22552E-06	0.005809527
1111.79998	250.884039	5.6621E-06	0.006295126
1111.83998	232.777123	5.25346E-06	0.005841002
1111.87998	216.6471	4.88942E-06	0.005436452
1111.91998	221.57816	5.00071E-06	0.00556039
1111.95998	246.851257	5.57109E-06	0.006194828
1111.99998	280.586254	6.33244E-06	0.007041674
1112.03998	302.739776	6.83242E-06	0.007597919
1112.07998	320.120477	7.22467E-06	0.008034415
1112.11998	366.024694	8.26067E-06	0.009186853
1112.15998	430.533891	9.71655E-06	0.010806358
1112.19998	458.590748	1.03498E-05	0.011510997
1112.23998	485.109807	1.09483E-05	0.012177084
1112.27998	517.579847	1.16811E-05	0.012992605
1112.31998	470.434674	1.06171E-05	0.011809563

1112.35998	407.257712	9.19124E-06	0.010223967
1112.39998	354.964107	8.01105E-06	0.008911487
1112.43998	303.306796	6.84521E-06	0.007614887
1112.47998	278.314268	6.28117E-06	0.006987671
1112.51998	243.523333	5.49598E-06	0.00611439
1112.55998	226.519678	5.11223E-06	0.005687667
1112.59998	260.949336	5.88926E-06	0.006552394
1112.63998	295.399582	6.66676E-06	0.0074177
1112.67998	303.12605	6.84113E-06	0.007611991
1112.71998	265.082947	5.98255E-06	0.006656906
1112.75998	212.692242	4.80017E-06	0.005341434
1112.79998	195.919174	4.42162E-06	0.004920382
1112.83998	230.475943	5.20152E-06	0.005788461
1112.87998	271.807548	6.13432E-06	0.006826759
1112.91998	298.872512	6.74514E-06	0.007506797
1112.95998	313.063278	7.0654E-06	0.00786351
1112.99998	328.149318	7.40587E-06	0.008242737
1113.03998	383.667393	8.65884E-06	0.009637634
1113.07998	451.055758	1.01797E-05	0.01133082
1113.11998	491.726597	1.10976E-05	0.012352943
1113.15998	549.165594	1.23939E-05	0.013796396
1113.19998	568.343992	1.28267E-05	0.014278718
1113.23998	536.502294	1.21081E-05	0.013479232
1113.27998	514.522088	1.1612E-05	0.012927459
1113.31998	503.707406	1.1368E-05	0.012656193
1113.35998	467.634416	1.05539E-05	0.011750242
1113.39998	412.970737	9.32017E-06	0.010377082
1113.43998	363.019113	8.19284E-06	0.009122231
1113.47998	296.916614	6.70099E-06	0.007461423
1113.51998	247.590918	5.58778E-06	0.006222107
1113.55998	244.405162	5.51588E-06	0.006142268
1113.59998	250.9955	5.66462E-06	0.006308119
1113.63998	252.06145	5.68868E-06	0.006335137
1113.67998	250.169736	5.64598E-06	0.006287818
1113.71998	256.804125	5.79571E-06	0.0064548
1113.75998	261.389962	5.89921E-06	0.006570301
1113.79998	257.681099	5.8155E-06	0.006477308
1113.83998	246.066589	5.55338E-06	0.006185577
1113.87998	221.329673	4.9951E-06	0.005563945
1113.91998	234.331025	5.28852E-06	0.005890993
1113.95998	267.108189	6.02826E-06	0.00671524
1113.99998	302.093328	6.81783E-06	0.007595058
1114.03998	325.876576	7.35458E-06	0.008193297
1114.07998	334.16475	7.54163E-06	0.008401982
1114.11998	371.201911	8.37751E-06	0.009333551
1114.15998	415.691799	9.38158E-06	0.010452586

1114.19998	487.543812	1.10032E-05	0.012259748
1114.23998	503.143296	1.13552E-05	0.012652466
1114.27998	469.972503	1.06066E-05	0.01181875
1114.31998	433.880598	9.79208E-06	0.010911511
1114.35998	397.112709	8.96228E-06	0.009987207
1114.39998	355.503696	8.02322E-06	0.00894108
1114.43998	319.322131	7.20666E-06	0.008031385
1114.47998	285.233944	6.43733E-06	0.007174279
1114.51998	279.32726	6.30403E-06	0.007025964
1114.55998	296.429347	6.69E-06	0.007456403
1114.59998	298.033312	6.7262E-06	0.007497019
1114.63998	282.661119	6.37927E-06	0.007110587
1114.67998	291.540381	6.57966E-06	0.007334216
1114.71998	317.263721	7.1602E-06	0.007981618
1114.75998	323.433699	7.29945E-06	0.008137133
1114.79998	317.47407	7.16495E-06	0.007987483
1114.83998	320.610939	7.23574E-06	0.008066695
1114.87998	341.864851	7.71541E-06	0.00860176
1114.91998	347.690928	7.8469E-06	0.008748666
1114.95998	358.256271	8.08535E-06	0.009014836
1114.99998	403.017542	9.09554E-06	0.010141532
1115.03998	444.798356	1.00385E-05	0.011193306
1115.07998	510.077614	1.15117E-05	0.012836512
1115.11998	577.376	1.30306E-05	0.014530651
1115.15998	618.163566	1.39511E-05	0.015557698
1115.19998	658.453779	1.48604E-05	0.016572301
1115.23998	642.231173	1.44943E-05	0.016164582
1115.27998	635.342749	1.43388E-05	0.015991778
1115.31998	607.514228	1.37107E-05	0.015291873
1115.35998	516.633914	1.16597E-05	0.013004771
1115.39998	416.632783	9.40282E-06	0.010487907
1115.43998	342.731019	7.73496E-06	0.008627886
1115.47998	284.691291	6.42509E-06	0.007167055
1115.51998	252.951024	5.70875E-06	0.006368227
1115.55998	255.107557	5.75742E-06	0.00642275
1115.59998	250.194293	5.64654E-06	0.006299276
1115.63998	253.953432	5.73138E-06	0.006394151
1115.67998	270.534409	6.10558E-06	0.006811879
1115.71998	263.298319	5.94228E-06	0.006629916
1115.75998	243.690635	5.49976E-06	0.00613641
1115.79998	254.967651	5.75426E-06	0.006420608
1115.83998	262.890994	5.93308E-06	0.006620372
1115.87998	269.937757	6.09212E-06	0.006798074
1115.91998	260.134605	5.87088E-06	0.006551427
1115.95998	263.274841	5.94175E-06	0.006630751
1115.99998	292.323842	6.59734E-06	0.007362633

1116.03998	336.931155	7.60407E-06	0.008486443
1116.07998	379.695524	8.5692E-06	0.009563912
1116.11998	402.826447	9.09123E-06	0.010146905
1116.15998	440.951025	9.95165E-06	0.011107634
1116.19998	502.511432	1.1341E-05	0.012658805
1116.23998	549.909577	1.24107E-05	0.013853312
1116.27998	561.775826	1.26785E-05	0.014152753
1116.31998	517.618167	1.16819E-05	0.013040762
1116.35998	442.097651	9.97753E-06	0.011138513
1116.39998	389.787985	8.79697E-06	0.009820939
1116.43998	330.333784	7.45517E-06	0.008323254
1116.47998	285.099891	6.43431E-06	0.007183775
1116.51998	266.713487	6.01935E-06	0.006720727
1116.55998	260.255739	5.87361E-06	0.006558237
1116.59998	272.931777	6.15969E-06	0.00687791
1116.63998	290.480362	6.55574E-06	0.007320398
1116.67998	279.307112	6.30357E-06	0.007039073
1116.71998	275.945669	6.22771E-06	0.006954608
1116.75998	303.853649	6.85755E-06	0.007658241
1116.79998	313.474239	7.07468E-06	0.007900999
1116.83998	278.803415	6.2922E-06	0.007027386
1116.87998	275.497384	6.21759E-06	0.006944304
1116.91998	308.730137	6.96761E-06	0.007782262
1116.95998	337.123045	7.6084E-06	0.008498276
1116.99998	366.568758	8.27295E-06	0.009240881
1117.03998	389.097321	8.78138E-06	0.009809158
1117.07998	452.019415	1.02014E-05	0.011395834
1117.11998	545.393305	1.23088E-05	0.01375037
1117.159979	596.269694	1.3457E-05	0.015033596
1117.199979	603.748231	1.36258E-05	0.015222695
1117.239979	601.558606	1.35763E-05	0.01516803
1117.279979	572.768063	1.29266E-05	0.014442606
1117.319979	513.177958	1.15817E-05	0.012940478
1117.359979	410.412006	9.26243E-06	0.010349466
1117.399979	342.086709	7.72042E-06	0.008626798
1117.439979	308.767268	6.96845E-06	0.007786821
1117.479979	261.404721	5.89954E-06	0.006592618
1117.519979	225.614506	5.09181E-06	0.005690194
1117.559979	230.72547	5.20715E-06	0.005819305
1117.599979	238.054209	5.37255E-06	0.006004364
1117.639979	259.065485	5.84675E-06	0.006534558
1117.679979	272.7793	6.15625E-06	0.006880716
1117.719979	248.447268	5.60711E-06	0.006267178
1117.759979	248.408696	5.60624E-06	0.006266429
1117.799979	243.598505	5.49768E-06	0.006145305
1117.839979	252.602274	5.70088E-06	0.006372673

1117.879979	271.774402	6.13357E-06	0.006856595
1117.919979	261.892634	5.91055E-06	0.006607524
1117.959979	275.091764	6.20844E-06	0.006940785
1117.999979	297.998207	6.7254E-06	0.007519002
1118.039979	303.986939	6.86056E-06	0.007670382
1118.079979	325.359677	7.34291E-06	0.008209966
1118.119979	382.332755	8.62872E-06	0.009647942
1118.159979	425.585778	9.60488E-06	0.01073979
1118.199979	461.497622	1.04154E-05	0.011646454
1118.239979	491.167917	1.1085E-05	0.012395663
1118.279979	464.410604	1.04811E-05	0.011720805
1118.319979	415.405381	9.37512E-06	0.010484385
1118.359979	369.443714	8.33783E-06	0.009324695
1118.399979	316.718901	7.1479E-06	0.007994216
1118.439979	293.335911	6.62018E-06	0.007404277
1118.479979	276.664251	6.24393E-06	0.006983707
1118.519979	283.69462	6.40259E-06	0.007161428
1118.559979	291.158804	6.57105E-06	0.007350112
1118.599979	277.869286	6.27112E-06	0.007014878
1118.639979	273.707127	6.17719E-06	0.00691005
1118.679979	276.913069	6.24954E-06	0.006991238
1118.719979	278.973499	6.29604E-06	0.007043509
1118.759979	276.566214	6.24171E-06	0.00698298
1118.799979	253.588982	5.72315E-06	0.00640306
1118.839979	236.163602	5.32988E-06	0.005963287
1118.879979	242.644616	5.47615E-06	0.006127156
1118.919979	255.462261	5.76543E-06	0.006451052
1118.959979	280.189726	6.32349E-06	0.007075734
1118.999979	312.402461	7.05049E-06	0.007889496
1119.039979	376.533901	8.49785E-06	0.009509429
1119.079979	415.797617	9.38397E-06	0.010501416
1119.119979	457.990173	1.03362E-05	0.011567448
1119.159979	474.609098	1.07113E-05	0.011987621
1119.199979	462.794695	1.04446E-05	0.011689632
1119.239979	519.645572	1.17277E-05	0.013126085
1119.279979	536.842298	1.21158E-05	0.013560953
1119.319979	497.594973	1.123E-05	0.012569992
1119.359979	438.306912	9.89198E-06	0.011072683
1119.399979	375.961112	8.48492E-06	0.009498018
1119.439979	309.877121	6.99349E-06	0.007828798
1119.479979	270.690849	6.10912E-06	0.006839032
1119.519979	277.486384	6.26248E-06	0.007010973
1119.559979	257.275133	5.80634E-06	0.006500547
1119.599979	232.234462	5.24121E-06	0.005868057
1119.639979	234.200555	5.28558E-06	0.005917947
1119.679979	239.568313	5.40672E-06	0.0060538

1119.719979	251.955971	5.6863E-06	0.006367058
1119.759979	249.382272	5.62821E-06	0.006302245
1119.799979	226.003813	5.10059E-06	0.005711642
1119.839979	220.707165	4.98105E-06	0.005577983
1119.879979	232.424793	5.2455E-06	0.005874335
1119.919979	238.872034	5.39101E-06	0.006037499
1119.959979	273.638243	6.17563E-06	0.006916463
1119.999979	305.198772	6.88791E-06	0.00771446
1120.039979	314.276437	7.09278E-06	0.007944199
1120.079979	329.595266	7.43851E-06	0.008331722
1120.119979	368.330811	8.31271E-06	0.009311236
1120.159979	434.401323	9.80383E-06	0.010981861
1120.199979	498.547104	1.12515E-05	0.012603945
1120.239979	483.574691	1.09136E-05	0.012225859
1120.279979	449.83683	1.01522E-05	0.011373296
1120.319979	405.420747	9.14978E-06	0.010250683
1120.359979	356.99527	8.05689E-06	0.009026613
1120.399979	301.503345	6.80451E-06	0.007623773
1120.439979	268.477507	6.05916E-06	0.006788929
1120.479979	255.497941	5.76623E-06	0.006460948
1120.519979	261.981645	5.91256E-06	0.006625143
1120.559979	263.453293	5.94577E-06	0.006662596
1120.599979	267.850477	6.04501E-06	0.006774041
1120.639979	296.803683	6.69845E-06	0.007506546
1120.679979	335.700428	7.57629E-06	0.008490598
1120.719979	344.219371	7.76855E-06	0.008706371
1120.759979	325.216338	7.33968E-06	0.008226019
1120.799979	294.583899	6.64835E-06	0.007451469
1120.839979	258.547922	5.83507E-06	0.006540176
1120.879979	252.228989	5.69246E-06	0.006380561
1120.919979	268.151219	6.0518E-06	0.006783583
1120.959979	279.755916	6.3137E-06	0.007077407
1120.999979	296.823374	6.69889E-06	0.007509456
1121.039979	342.932208	7.7395E-06	0.008676291
1121.079979	404.35089	9.12564E-06	0.010230568
1121.119979	455.463917	1.02792E-05	0.011524201
1121.159979	500.793712	1.13022E-05	0.012671593
1121.199979	527.741256	1.19104E-05	0.013353923
1121.239979	556.838333	1.25671E-05	0.014090696
1121.279979	537.270308	1.21254E-05	0.013596016
1121.319979	475.270844	1.07262E-05	0.012027503
1121.359979	398.672191	8.99748E-06	0.01008941
1121.399979	326.449841	7.36752E-06	0.008261935
1121.439979	283.406988	6.3961E-06	0.007172843
1121.479979	266.817866	6.02171E-06	0.006753225
1121.519979	255.966638	5.77681E-06	0.006478808

1121.559979	260.234621	5.87313E-06	0.006587071
1121.599979	245.015902	5.52967E-06	0.006202075
1121.639979	229.885277	5.18819E-06	0.005819282
1121.679979	260.239123	5.87323E-06	0.00658789
1121.719979	285.039923	6.43295E-06	0.007215973
1121.759979	281.442388	6.35176E-06	0.007125153
1121.799979	281.476528	6.35253E-06	0.007126272
1121.839979	275.067631	6.20789E-06	0.006964263
1121.879979	276.128843	6.23184E-06	0.00699138
1121.919979	257.439061	5.81004E-06	0.006518401
1121.959979	247.637068	5.58882E-06	0.006270437
1121.999979	267.740625	6.04253E-06	0.006779722
1122.039979	305.248939	6.88904E-06	0.007729782
1122.079979	348.096911	7.85606E-06	0.00881513
1122.119979	388.508869	8.7681E-06	0.009838865
1122.159979	421.746713	9.51824E-06	0.010680983
1122.199979	450.257686	1.01617E-05	0.011403447
1122.239979	455.96523	1.02905E-05	0.011548411
1122.279979	449.790111	1.01511E-05	0.011392417
1122.319979	427.970858	9.65871E-06	0.010840159
1122.359979	361.314632	8.15437E-06	0.009152136
1122.399979	310.318928	7.00347E-06	0.00786069
1122.439979	295.370423	6.6661E-06	0.007482296
1122.479979	290.199357	6.5494E-06	0.007351565
1122.519979	263.732423	5.95207E-06	0.006681321
1122.559979	231.57698	5.22637E-06	0.005866914
1122.599979	237.320806	5.356E-06	0.006012646
1122.639979	264.470507	5.96873E-06	0.006700736
1122.679979	262.487604	5.92398E-06	0.006650733
1122.719979	258.902908	5.84308E-06	0.00656014
1122.759979	277.248881	6.25712E-06	0.007025245
1122.799979	256.886257	5.79757E-06	0.006509506
1122.839979	254.988549	5.75474E-06	0.006461648
1122.879979	278.221074	6.27906E-06	0.007050633
1122.919979	264.405014	5.96725E-06	0.006700747
1122.959979	259.697536	5.86101E-06	0.006581682
1122.999979	295.292685	6.66434E-06	0.007484059
1123.039979	345.681476	7.80155E-06	0.008761452
1123.079979	400.4201	9.03692E-06	0.010149188
1123.119979	480.517186	1.08446E-05	0.012179791
1123.159979	529.391216	1.19476E-05	0.013419091
1123.199979	538.362388	1.21501E-05	0.01364698
1123.239979	551.490898	1.24464E-05	0.013980273
1123.279979	558.582719	1.26064E-05	0.014160555
1123.319979	505.29358	1.14038E-05	0.012810085
1123.359979	420.378672	9.48736E-06	0.010657722

1123.399979	366.512117	8.27167E-06	0.009292392
1123.439979	341.410668	7.70516E-06	0.008656288
1123.479979	293.59109	6.62594E-06	0.007444113
1123.519979	238.74281	5.38809E-06	0.00605363
1123.559979	225.937239	5.09909E-06	0.005729132
1123.599979	249.12385	5.62238E-06	0.006317304
1123.639979	251.224722	5.66979E-06	0.006370805
1123.679979	251.289427	5.67125E-06	0.006372673
1123.719979	264.427592	5.96776E-06	0.006706094
1123.759979	259.407261	5.85446E-06	0.006579008
1123.799979	250.92748	5.66308E-06	0.006364173
1123.839979	235.721473	5.31991E-06	0.005978722
1123.879979	238.630652	5.38556E-06	0.006052725
1123.919979	258.555804	5.83524E-06	0.006558348
1123.959979	283.877723	6.40672E-06	0.007200902
1123.999979	294.932904	6.65622E-06	0.007481597
1124.039979	284.453387	6.41972E-06	0.007216018
1124.079979	278.194006	6.27845E-06	0.007057481
1124.119979	297.044854	6.70389E-06	0.007535975
1124.159979	377.516738	8.52003E-06	0.009577873
1124.199979	441.735444	9.96935E-06	0.011207547
1124.239979	454.135695	1.02492E-05	0.011522572
1124.279979	438.27415	9.89124E-06	0.01112052
1124.319979	429.150957	9.68534E-06	0.010889421
1124.359979	405.051105	9.14144E-06	0.010278268
1124.399979	365.344114	8.24531E-06	0.009271024
1124.439979	318.567011	7.18961E-06	0.008084289
1124.479979	278.260443	6.27995E-06	0.007061679
1124.519979	267.616069	6.03972E-06	0.006791788
1124.559979	259.078432	5.84704E-06	0.006575346
1124.599979	231.115058	5.21594E-06	0.005865852
1124.639979	222.971867	5.03216E-06	0.005659373
1124.679979	235.490632	5.3147E-06	0.005977332
1124.719979	273.395501	6.17016E-06	0.006939697
1124.759979	323.836467	7.30854E-06	0.008220351
1124.799979	326.301437	7.36417E-06	0.008283217
1124.839979	292.173193	6.59394E-06	0.00741713
1124.879979	262.612739	5.9268E-06	0.006666943
1124.919979	242.129507	5.46453E-06	0.006147154
1124.959979	254.38351	5.74108E-06	0.006458487
1124.999979	291.385288	6.57616E-06	0.00739818
1125.039979	349.199527	7.88095E-06	0.00886638
1125.079979	434.417254	9.80419E-06	0.011030501
1125.119979	493.169244	1.11301E-05	0.012522746
1125.159979	520.31206	1.17427E-05	0.013212437
1125.199979	516.356643	1.16535E-05	0.013112462

1125.239979	506.019692	1.14202E-05	0.01285042
1125.279979	505.853679	1.14164E-05	0.012846661
1125.319979	460.801466	1.03996E-05	0.011702931
1125.359979	423.6381	9.56092E-06	0.010759479
1125.399979	387.03934	8.73494E-06	0.0098303
1125.439979	331.614179	7.48407E-06	0.008422872
1125.479979	288.750255	6.51669E-06	0.007334405
1125.519979	235.925962	5.32452E-06	0.005992854
1125.559979	217.125602	4.90022E-06	0.005515494
1125.599979	228.381068	5.15424E-06	0.005801615
1125.639979	247.016165	5.57481E-06	0.00627523
1125.679979	275.176895	6.21036E-06	0.006990877
1125.719979	276.690231	6.24451E-06	0.007029573
1125.759979	280.966179	6.34102E-06	0.007138461
1125.799979	272.710347	6.15469E-06	0.006928953
1125.839979	251.601456	5.67829E-06	0.006392851
1125.879979	251.011784	5.66499E-06	0.006378095
1125.919979	275.476628	6.21712E-06	0.006999984
1125.959979	270.849536	6.1127E-06	0.006882652
1125.999979	251.508698	5.6762E-06	0.006391402
1126.039979	265.92205	6.00149E-06	0.006757918
1126.079979	316.669101	7.14678E-06	0.008047846
1126.119979	369.376016	8.3363E-06	0.009387676
1126.159979	423.784029	9.56422E-06	0.010770836
1126.199979	460.649854	1.03962E-05	0.011708229
1126.239979	470.972118	1.06292E-05	0.011971013
1126.279979	449.438205	1.01432E-05	0.011424077
1126.319979	411.706312	9.29164E-06	0.010465358
1126.359979	384.728655	8.68279E-06	0.009779947
1126.399979	360.843437	8.14373E-06	0.009173102
1126.439979	310.662455	7.01122E-06	0.007897717
1126.479979	286.024378	6.45517E-06	0.007271622
1126.519979	266.971149	6.02517E-06	0.006787471
1126.559979	227.284195	5.12949E-06	0.005778676
1126.599979	222.698892	5.026E-06	0.005662296
1126.639979	248.112439	5.59955E-06	0.006308679
1126.679979	269.874767	6.0907E-06	0.006862267
1126.719979	261.76288	5.90762E-06	0.006656238
1126.759979	275.961379	6.22806E-06	0.007017533
1126.799979	296.567857	6.69312E-06	0.007541811
1126.839979	303.90927	6.85881E-06	0.00772878
1126.879979	304.499566	6.87213E-06	0.007744067
1126.919979	295.060675	6.65911E-06	0.007504282
1126.959979	287.878595	6.49702E-06	0.00732188
1126.999979	330.619856	7.46163E-06	0.008409256
1127.039979	378.927273	8.55186E-06	0.009638289

1127.079979	395.594575	8.92802E-06	0.010062591
1127.119979	440.501857	9.94151E-06	0.011205278
1127.159979	505.96494	1.14189E-05	0.012870954
1127.199979	560.799724	1.26565E-05	0.014266371
1127.239979	585.190939	1.32069E-05	0.014887396
1127.279979	560.311828	1.26455E-05	0.014254971
1127.319979	509.487161	1.14984E-05	0.012962394
1127.359979	426.783832	9.63192E-06	0.010858637
1127.399979	336.756672	7.60013E-06	0.008568385
1127.439979	297.474698	6.71359E-06	0.007569169
1127.479979	280.45747	6.32953E-06	0.007136423
1127.519979	258.630246	5.83692E-06	0.006581249
1127.559979	241.668957	5.45413E-06	0.006149861
1127.599979	237.925405	5.36964E-06	0.006054812
1127.639979	237.197306	5.35321E-06	0.006036497
1127.679979	243.104454	5.48653E-06	0.006187049
1127.719979	255.735661	5.7716E-06	0.006508746
1127.759979	273.581948	6.17436E-06	0.0069632
1127.799979	287.263487	6.48314E-06	0.007311682
1127.839979	280.920491	6.33998E-06	0.007150488
1127.879979	241.908789	5.45954E-06	0.006157711
1127.919979	228.973323	5.16761E-06	0.005828649
1127.959979	257.051474	5.80129E-06	0.006543627
1127.999979	297.676745	6.71815E-06	0.007578073
1128.039979	331.911873	7.49079E-06	0.008449909
1128.079979	350.633229	7.9133E-06	0.008926839
1128.119979	385.170693	8.69277E-06	0.009806483
1128.159979	426.696454	9.62994E-06	0.010864118
1128.199979	437.898919	9.88277E-06	0.011149739
1128.239979	462.783662	1.04444E-05	0.01178377
1128.279979	466.479591	1.05278E-05	0.0118783
1128.319979	418.895211	9.45388E-06	0.010667003
1128.359979	339.390275	7.65957E-06	0.008642747
1128.399979	297.111284	6.70539E-06	0.007566359
1128.439979	294.162941	6.63885E-06	0.007491541
1128.479979	276.909356	6.24946E-06	0.007052389
1128.519979	259.740025	5.86197E-06	0.006615351
1128.559979	235.642006	5.31811E-06	0.006001808
1128.599979	241.912818	5.45964E-06	0.006161744
1128.639979	246.48945	5.56292E-06	0.006278538
1128.679979	265.788061	5.99847E-06	0.006770349
1128.719979	268.417426	6.05781E-06	0.006837568
1128.759979	255.095795	5.75716E-06	0.006498448
1128.799979	281.037897	6.34263E-06	0.007159565
1128.839979	301.149732	6.79653E-06	0.007672195
1128.879979	289.240093	6.52775E-06	0.007369042

1128.919979	285.529856	6.44401E-06	0.007274773
1128.959979	293.842647	6.63162E-06	0.007486833
1128.999979	322.546298	7.27942E-06	0.008218466
1129.039979	360.565813	8.13747E-06	0.009187527
1129.079979	417.18495	9.41528E-06	0.010630608
1129.119979	456.092692	1.02934E-05	0.011622457
1129.159979	462.118728	1.04294E-05	0.011776434
1129.199979	495.972601	1.11934E-05	0.012639599
1129.239979	529.021421	1.19393E-05	0.013482308
1129.279979	512.514628	1.15667E-05	0.013062089
1129.319979	481.437382	1.08654E-05	0.012270481
1129.359979	410.644242	9.26767E-06	0.010466534
1129.399979	351.230432	7.92678E-06	0.008952507
1129.439979	316.721654	7.14797E-06	0.008073199
1129.479979	279.26239	6.30256E-06	0.007118619
1129.519979	242.727208	5.47801E-06	0.006187527
1129.559979	216.889271	4.89489E-06	0.00552907
1129.599979	229.477736	5.17899E-06	0.00585019
1129.639979	256.218681	5.7825E-06	0.006532142
1129.679979	264.229921	5.9633E-06	0.006736622
1129.719979	258.624803	5.8368E-06	0.006593951
1129.759979	256.040419	5.77848E-06	0.00652829
1129.799979	275.178303	6.21039E-06	0.0070165
1129.839979	274.258381	6.18963E-06	0.006993291
1129.879979	251.307644	5.67166E-06	0.006408299
1129.919979	267.806311	6.04402E-06	0.006829254
1129.959979	280.399982	6.32824E-06	0.007150655
1129.999979	273.453801	6.17147E-06	0.006973763
1130.039979	276.372246	6.23734E-06	0.00704844
1130.079979	297.096252	6.70505E-06	0.007577241
1130.119979	362.758142	8.18695E-06	0.009252231
1130.159979	424.2328	9.57434E-06	0.01082054
1130.199979	433.030574	9.7729E-06	0.011045328
1130.239979	433.170779	9.77606E-06	0.011049295
1130.279979	451.014107	1.01788E-05	0.011504849
1130.319979	448.061778	1.01121E-05	0.011429943
1130.359979	389.884449	8.79915E-06	0.009946206
1130.399979	357.1307	8.05994E-06	0.009110959
1130.439979	332.298812	7.49952E-06	0.008477759
1130.479979	280.237019	6.32456E-06	0.007149788
1130.519979	229.786724	5.18597E-06	0.005862839
1130.559979	216.274742	4.88102E-06	0.005518286
1130.599979	242.01257	5.46189E-06	0.006175209
1130.639979	277.278111	6.25778E-06	0.007075297
1130.679979	305.710842	6.89947E-06	0.00780109
1130.719979	297.214082	6.70771E-06	0.007584539

1130.759979	278.579403	6.28715E-06	0.007109257
1130.799979	268.46614	6.05891E-06	0.006851412
1130.839979	268.804409	6.06654E-06	0.006860287
1130.879979	282.177289	6.36835E-06	0.007201838
1130.919979	303.82669	6.85695E-06	0.007754656
1130.959979	341.173493	7.69981E-06	0.008708178
1130.999979	343.818898	7.75951E-06	0.00877601
1131.039979	337.504167	7.617E-06	0.00861513
1131.079979	379.76823	8.57084E-06	0.009694306
1131.119979	417.319349	9.41832E-06	0.010653246
1131.159979	457.950182	1.03353E-05	0.011690875
1131.199979	523.255862	1.18092E-05	0.013358517
1131.239979	556.720338	1.25644E-05	0.014213355
1131.279979	511.787363	1.15503E-05	0.013066655
1131.319979	469.302327	1.05915E-05	0.011982375
1131.359979	433.779952	9.78981E-06	0.011075798
1131.399979	400.061769	9.02884E-06	0.010215226
1131.439979	349.928954	7.89741E-06	0.008935444
1131.479979	287.184263	6.48135E-06	0.007333516
1131.519979	264.289469	5.96465E-06	0.006749115
1131.559979	247.85398	5.59372E-06	0.006329629
1131.599979	239.010447	5.39413E-06	0.006104001
1131.639979	233.569276	5.27133E-06	0.005965251
1131.679979	251.855831	5.68404E-06	0.006432509
1131.719979	252.643387	5.70181E-06	0.006452852
1131.759979	251.436016	5.67456E-06	0.006422241
1131.799979	264.502265	5.96945E-06	0.006756221
1131.839979	260.357727	5.87591E-06	0.006650591
1131.879979	252.915897	5.70796E-06	0.006460725
1131.919979	254.871098	5.75209E-06	0.006510901
1131.959979	278.457085	6.28439E-06	0.007113676
1131.999979	301.341021	6.80085E-06	0.007698559
1132.039979	305.547269	6.89578E-06	0.007806294
1132.079979	303.13906	6.84143E-06	0.007745042
1132.119979	336.370401	7.59141E-06	0.008594389
1132.159979	396.642296	8.95166E-06	0.010134716
1132.199979	471.537267	1.06419E-05	0.012048803
1132.239979	487.113825	1.09935E-05	0.012447258
1132.279979	485.40915	1.0955E-05	0.012404137
1132.319979	451.194514	1.01828E-05	0.011530224
1132.359979	401.392784	9.05888E-06	0.010257909
1132.399979	357.833215	8.0758E-06	0.009145033
1132.439979	300.337502	6.7782E-06	0.007675904
1132.479979	259.408172	5.85448E-06	0.006630083
1132.519979	241.95912	5.46068E-06	0.00618433
1132.559979	230.237282	5.19613E-06	0.005884934

1132.599979	234.326094	5.28841E-06	0.005989657
1132.639979	255.094819	5.75713E-06	0.006520761
1132.679979	261.384186	5.89908E-06	0.006681766
1132.719979	240.043676	5.41745E-06	0.006136455
1132.759979	245.753656	5.54632E-06	0.006282647
1132.799979	270.833934	6.11234E-06	0.006924064
1132.839979	284.721709	6.42577E-06	0.007279372
1132.879979	285.62516	6.44616E-06	0.007302728
1132.919979	271.530026	6.12805E-06	0.006942595
1132.959979	284.517886	6.42117E-06	0.007274931
1132.999979	315.407627	7.11831E-06	0.008065046
1133.039979	353.501918	7.97805E-06	0.009039445
1133.079979	428.461741	9.66978E-06	0.010956639
1133.119979	497.079578	1.12184E-05	0.012711786
1133.159979	512.814374	1.15735E-05	0.013114634
1133.199979	520.288481	1.17422E-05	0.013306245
1133.239979	529.980606	1.19609E-05	0.013554597
1133.279979	536.655461	1.21116E-05	0.013725796
1133.319979	510.688732	1.15255E-05	0.013062117
1133.359979	446.096103	1.00678E-05	0.011410405
1133.399979	373.711131	8.43414E-06	0.009559254
1133.439979	309.950582	6.99515E-06	0.007928586
1133.479979	287.651538	6.49189E-06	0.007358432
1133.519979	285.862999	6.45153E-06	0.007312938
1133.559979	262.119944	5.91568E-06	0.00670578
1133.599979	258.858719	5.84208E-06	0.006622583
1133.639979	276.248982	6.23455E-06	0.007067741
1133.679979	278.153069	6.27753E-06	0.007116707
1133.719979	280.896632	6.33945E-06	0.007187156
1133.759979	287.463495	6.48765E-06	0.007355439
1133.799979	291.850708	6.58666E-06	0.007467959
1133.839979	296.188231	6.68456E-06	0.007579217
1133.879979	278.576044	6.28707E-06	0.007128787
1133.919979	266.940409	6.02447E-06	0.006831271
1133.959979	243.183511	5.48831E-06	0.006223527
1133.999979	210.042749	4.74037E-06	0.005375582
1134.039979	223.611967	5.04661E-06	0.005723058
1134.079979	294.097719	6.63738E-06	0.007527315
1134.119979	385.719769	8.70516E-06	0.009872694
1134.159979	460.167139	1.03853E-05	0.011778627
1134.199979	480.179913	1.0837E-05	0.012291316
1134.239979	476.978843	1.07647E-05	0.012209808
1134.279979	465.276462	1.05006E-05	0.011910668
1134.319979	402.036429	9.0734E-06	0.010292141
1134.359979	363.250259	8.19805E-06	0.009299543
1134.399979	336.816688	7.60148E-06	0.008623123

1134.439979	307.273212	6.93473E-06	0.007867033
1134.479979	281.933213	6.36284E-06	0.007218514
1134.519979	294.464929	6.64566E-06	0.007539638
1134.559979	315.681823	7.1245E-06	0.008083171
1134.599979	324.308785	7.3192E-06	0.008304361
1134.639979	336.676571	7.59832E-06	0.008621359
1134.679979	342.679755	7.7338E-06	0.008775393
1134.719979	336.33862	7.59069E-06	0.008613312
1134.759979	328.877599	7.42231E-06	0.00842254
1134.799979	315.644922	7.12367E-06	0.008083936
1134.839979	334.281361	7.54426E-06	0.008561533
1134.879979	333.186697	7.51956E-06	0.008533798
1134.919979	326.280548	7.3637E-06	0.008357207
1134.959979	331.17397	7.47414E-06	0.008482844
1134.999979	357.113791	8.05956E-06	0.009147601
1135.039979	416.211245	9.39331E-06	0.01066178
1135.079979	515.874327	1.16426E-05	0.013215242
1135.119979	582.632714	1.31492E-05	0.01492593
1135.159979	630.055765	1.42195E-05	0.016141386
1135.199979	681.976509	1.53913E-05	0.017472158
1135.239979	719.929898	1.62478E-05	0.018445169
1135.279979	695.925499	1.57061E-05	0.017830786
1135.319979	637.251232	1.43819E-05	0.016328027
1135.359979	563.906514	1.27266E-05	0.014449255
1135.399979	452.639743	1.02154E-05	0.01159862
1135.439979	356.280657	8.04076E-06	0.009129798
1135.479979	275.880573	6.22624E-06	0.007069771
1135.519979	238.80661	5.38953E-06	0.006119922
1135.559979	240.06363	5.4179E-06	0.006152352
1135.599979	234.635168	5.29539E-06	0.006013444
1135.639979	237.419642	5.35823E-06	0.006085021
1135.679979	270.381264	6.10213E-06	0.006930065
1135.719979	292.486643	6.60102E-06	0.007496906
1135.759979	289.740401	6.53904E-06	0.007426777
1135.799979	285.706244	6.44799E-06	0.007323629
1135.839979	290.109681	6.54737E-06	0.007436766
1135.879979	271.663792	6.13107E-06	0.006964164
1135.919979	245.688392	5.54484E-06	0.0062985
1135.959979	259.288522	5.85178E-06	0.006647389
1135.999979	323.7811	7.30729E-06	0.00830108
1136.039979	366.388279	8.26887E-06	0.00939377
1136.079979	398.20306	8.98689E-06	0.010209824
1136.119979	427.229389	9.64197E-06	0.010954437
1136.159979	468.978604	1.05842E-05	0.012025337
1136.199979	486.503211	1.09797E-05	0.012475135
1136.239979	484.28974	1.09297E-05	0.012418813

1136.279979	452.983754	1.02232E-05	0.011616432
1136.319979	417.362035	9.41928E-06	0.010703316
1136.359979	386.957741	8.7331E-06	0.009923942
1136.399979	376.202629	8.49037E-06	0.009648456
1136.439979	351.712442	7.93766E-06	0.009020674
1136.479979	279.524898	6.30849E-06	0.00716947
1136.519979	238.795282	5.38928E-06	0.006125021
1136.559979	234.953704	5.30258E-06	0.006026698
1136.599979	224.981113	5.07751E-06	0.005771098
1136.639979	216.441886	4.88479E-06	0.00555225
1136.679979	241.388923	5.44781E-06	0.006192419
1136.719979	288.428642	6.50943E-06	0.007399402
1136.759979	319.875678	7.21915E-06	0.008206439
1136.799979	323.626648	7.3038E-06	0.008302963
1136.839979	288.907507	6.52024E-06	0.00741247
1136.879979	262.17976	5.91703E-06	0.006726955
1136.919979	264.724233	5.97446E-06	0.00679248
1136.959979	268.648951	6.06303E-06	0.006893425
1136.999979	284.008157	6.40967E-06	0.007287793
1137.039979	330.895725	7.46786E-06	0.00849125
1137.079979	389.780632	8.79681E-06	0.010002672
1137.119979	437.822908	9.88105E-06	0.011235943
1137.159979	497.889871	1.12367E-05	0.012777904
1137.199979	527.984234	1.19159E-05	0.013550726
1137.239979	550.521629	1.24245E-05	0.014129645
1137.279979	555.317169	1.25327E-05	0.014253229
1137.319979	527.810673	1.1912E-05	0.013547701
1137.359979	488.219492	1.10184E-05	0.012531926
1137.399979	404.855124	9.13702E-06	0.010392442
1137.439979	329.406402	7.43424E-06	0.008456006
1137.479979	289.396786	6.53128E-06	0.007429203
1137.519979	245.740452	5.54602E-06	0.006308708
1137.559979	212.715415	4.80069E-06	0.005461074
1137.599979	224.555044	5.06789E-06	0.005765237
1137.639979	264.713306	5.97421E-06	0.006796501
1137.679979	274.138749	6.18693E-06	0.007038746
1137.719979	255.15136	5.75841E-06	0.006551459
1137.759979	254.166592	5.73619E-06	0.006526403
1137.799979	301.785682	6.81088E-06	0.007749422
1137.839979	295.078017	6.6595E-06	0.007577445
1137.879979	256.509649	5.78907E-06	0.006587262
1137.919979	229.660701	5.18312E-06	0.005897978
1137.959979	217.321002	4.90463E-06	0.005581275
1137.999979	229.957953	5.18983E-06	0.005906027
1138.039979	277.313126	6.25857E-06	0.007122504
1138.079979	324.576178	7.32523E-06	0.0083367

1138.119979	358.627533	8.09372E-06	0.009211629
1138.159979	411.371485	9.28408E-06	0.01056677
1138.199979	431.815622	9.74548E-06	0.011092302
1138.239979	442.755221	9.99237E-06	0.011373713
1138.279979	447.358661	1.00963E-05	0.011492373
1138.319979	413.47581	9.33157E-06	0.010622316
1138.359979	343.553852	7.75353E-06	0.00882631
1138.399979	284.332255	6.41698E-06	0.007305093
1138.439979	271.805067	6.13426E-06	0.006983489
1138.479979	263.653424	5.95029E-06	0.006774287
1138.519979	250.658001	5.657E-06	0.00644061
1138.559979	237.419256	5.35822E-06	0.006100657
1138.599979	225.308575	5.0849E-06	0.005789668
1138.639979	241.585176	5.45224E-06	0.006208139
1138.679979	261.97302	5.91237E-06	0.006732293
1138.719979	266.656032	6.01806E-06	0.00685288
1138.759979	251.660198	5.67962E-06	0.006467724
1138.799979	249.088543	5.62158E-06	0.006401857
1138.839979	262.570488	5.92585E-06	0.006748595
1138.879979	268.159007	6.05198E-06	0.006892473
1138.919979	253.010389	5.71009E-06	0.006503338
1138.959979	250.804991	5.66032E-06	0.006446877
1138.999979	262.531812	5.92498E-06	0.006748549
1139.039979	297.865928	6.72242E-06	0.007657104
1139.079979	355.364498	8.02008E-06	0.009135515
1139.119979	420.085371	9.48074E-06	0.010799702
1139.159979	475.358092	1.07282E-05	0.012221102
1139.199979	537.380076	1.21279E-05	0.013816126
1139.239979	566.805112	1.2792E-05	0.01457316
1139.279979	516.513418	1.1657E-05	0.013280574
1139.319979	439.676298	9.92288E-06	0.011305337
1139.359979	401.778473	9.06758E-06	0.010331238
1139.399979	373.880171	8.43795E-06	0.009614205
1139.439979	313.228804	7.06914E-06	0.008054858
1139.479979	244.235541	5.51206E-06	0.006280877
1139.519979	206.445216	4.65918E-06	0.00530923
1139.559979	196.406726	4.43263E-06	0.005051243
1139.599979	217.200414	4.90191E-06	0.005586217
1139.639979	249.675936	5.63484E-06	0.006421687
1139.679979	288.083183	6.50164E-06	0.007409785
1139.719979	278.004252	6.27417E-06	0.007150795
1139.759979	258.936486	5.84384E-06	0.00666057
1139.799979	271.873802	6.13581E-06	0.0069936
1139.839979	262.770326	5.93036E-06	0.006759662
1139.879979	254.593415	5.74582E-06	0.006549544
1139.919979	256.838885	5.7965E-06	0.006607541

1139.959979	250.03524	5.64295E-06	0.006432734
1139.999979	262.518314	5.92467E-06	0.006754127
1140.039979	278.830471	6.29282E-06	0.007174061
1140.079979	327.204149	7.38454E-06	0.008418968
1140.119979	379.83739	8.5724E-06	0.009773565
1140.159979	370.066991	8.3519E-06	0.009522498
1140.199979	340.526657	7.68521E-06	0.008762679
1140.239979	336.13003	7.58599E-06	0.008649845
1140.279979	348.145416	7.85716E-06	0.008959359
1140.319979	380.459925	8.58645E-06	0.009791301
1140.359979	379.477949	8.56429E-06	0.009766372
1140.399979	355.576448	8.02487E-06	0.009151556
1140.439979	315.704045	7.125E-06	0.008125635
1140.479979	261.231446	5.89563E-06	0.006723848
1140.519979	233.664506	5.27348E-06	0.006014512
1140.559979	225.877737	5.09775E-06	0.005814285
1140.599979	219.778649	4.9601E-06	0.005657488
1140.639979	233.262511	5.26441E-06	0.006004796
1140.679979	243.278231	5.49045E-06	0.006262847
1140.719979	261.054116	5.89163E-06	0.006720698
1140.759979	283.373986	6.39536E-06	0.007295566
1140.799979	270.636503	6.10789E-06	0.00696788
1140.839979	250.349995	5.65005E-06	0.006445804
1140.879979	255.009714	5.75521E-06	0.006566008
1140.919979	247.782399	5.5921E-06	0.006380143
1140.959979	262.829772	5.9317E-06	0.006767834
1140.999979	280.766063	6.3365E-06	0.007229945
1141.039979	318.009458	7.17703E-06	0.008189279
1141.079979	355.766321	8.02915E-06	0.009161903
1141.119979	391.656475	8.83914E-06	0.010086521
1141.159979	462.080686	1.04285E-05	0.011900607
1141.199979	507.201199	1.14468E-05	0.013063116
1141.239979	499.012423	1.1262E-05	0.012852662
1141.279979	469.825603	1.06033E-05	0.012101345
1141.319979	457.070113	1.03154E-05	0.011773213
1141.359979	428.010281	9.6596E-06	0.011025076
1141.399979	382.748184	8.63809E-06	0.00985952
1141.439979	313.121478	7.06672E-06	0.008066232
1141.479979	246.039651	5.55277E-06	0.006338378
1141.519979	226.263306	5.10645E-06	0.005829112
1141.559979	223.090866	5.03485E-06	0.005747583
1141.599979	213.110254	4.8096E-06	0.005490641
1141.639979	210.67922	4.75474E-06	0.005428197
1141.679979	235.713035	5.31971E-06	0.006073412
1141.719979	262.291174	5.91955E-06	0.006758464
1141.759979	273.038207	6.16209E-06	0.00703563

1141.799979	252.724214	5.70363E-06	0.006512409
1141.839979	231.158367	5.21692E-06	0.005956891
1141.879979	232.46978	5.24652E-06	0.005990895
1141.919979	229.93827	5.18939E-06	0.005925864
1141.959979	237.234249	5.35405E-06	0.006114107
1141.999979	259.984722	5.86749E-06	0.006700677
1142.039979	283.855759	6.40623E-06	0.00731617
1142.079979	317.542538	7.16649E-06	0.008184708
1142.119979	378.108123	8.53337E-06	0.009746137
1142.159979	407.631775	9.19968E-06	0.010507508
1142.199979	402.432726	9.08235E-06	0.010373856
1142.239979	376.922208	8.50661E-06	0.009716589
1142.279979	353.600521	7.98027E-06	0.009115704
1142.319979	344.334619	7.77115E-06	0.008877143
1142.359979	344.506777	7.77504E-06	0.008881892
1142.399979	325.747205	7.35166E-06	0.008398537
1142.439979	285.739436	6.44874E-06	0.0073673
1142.479979	258.714714	5.83883E-06	0.006670747
1142.519979	229.635553	5.18255E-06	0.005921172
1142.559979	206.774083	4.6666E-06	0.005331874
1142.599979	235.667017	5.31868E-06	0.006077119
1142.639979	297.422232	6.71241E-06	0.007669863
1142.679979	350.916427	7.91969E-06	0.009049677
1142.719979	348.793357	7.87178E-06	0.008995241
1142.759979	321.916	7.2652E-06	0.008302375
1142.799979	294.007411	6.63534E-06	0.007582864
1142.839979	275.644423	6.22091E-06	0.007109505
1142.879979	279.350747	6.30456E-06	0.007205352
1142.919979	297.068407	6.70442E-06	0.007662616
1142.959979	314.270998	7.09266E-06	0.008106625
1142.999979	310.214809	7.00112E-06	0.008002276
1143.039979	330.825666	7.46627E-06	0.00853425
1143.079979	406.745014	9.17967E-06	0.010493095
1143.119979	477.230276	1.07704E-05	0.012311885
1143.159979	526.195753	1.18755E-05	0.013575602
1143.199979	537.813945	1.21377E-05	0.013875832
1143.239979	544.662296	1.22923E-05	0.014053014
1143.279979	507.102782	1.14446E-05	0.013084386
1143.319979	460.469038	1.03921E-05	0.011881547
1143.359979	422.791833	9.54182E-06	0.010909738
1143.399979	358.811555	8.09788E-06	0.009259112
1143.439979	304.022511	6.86136E-06	0.007845559
1143.479979	282.956994	6.38595E-06	0.007302201
1143.519979	268.595476	6.06183E-06	0.006931819
1143.559979	250.612026	5.65596E-06	0.006467934
1143.599979	247.809008	5.5927E-06	0.006395816

1143.639979	250.385949	5.65086E-06	0.006462552
1143.679979	264.021355	5.95859E-06	0.006814725
1143.719979	269.397129	6.07992E-06	0.006953724
1143.759979	254.105658	5.73481E-06	0.006559247
1143.799979	250.248925	5.64777E-06	0.006459919
1143.839979	277.6714	6.26666E-06	0.007168053
1143.879979	278.161945	6.27773E-06	0.007180967
1143.919979	286.140949	6.4578E-06	0.007387209
1143.959979	308.729798	6.9676E-06	0.007970657
1143.999979	307.32401	6.93587E-06	0.007934641
1144.039979	299.005192	6.74813E-06	0.007720131
1144.079979	333.516636	7.52701E-06	0.008611497
1144.119979	372.112445	8.39806E-06	0.009608388
1144.159979	434.033454	9.79553E-06	0.011207654
1144.199979	478.311968	1.07948E-05	0.01235145
1144.239979	507.457759	1.14526E-05	0.01310454
1144.279979	568.406312	1.28281E-05	0.014678982
1144.319979	557.712515	1.25868E-05	0.014403321
1144.359979	474.856818	1.07169E-05	0.012263942
1144.399979	398.607129	8.99601E-06	0.010295031
1144.439979	343.898589	7.76131E-06	0.008882356
1144.479979	283.664165	6.40191E-06	0.007326852
1144.519979	247.152343	5.57788E-06	0.006384
1144.559979	238.348166	5.37919E-06	0.006156801
1144.599979	266.482028	6.01413E-06	0.006883771
1144.639979	297.781326	6.72051E-06	0.007692564
1144.679979	336.75118	7.60001E-06	0.008699574
1144.719979	368.575866	8.31824E-06	0.00952206
1144.759979	391.307943	8.83128E-06	0.01010969
1144.799979	424.63797	9.58349E-06	0.010971176
1144.839979	417.675716	9.42636E-06	0.010791673
1144.879979	374.072001	8.44228E-06	0.009665402
1144.919979	327.702599	7.39579E-06	0.008467589
1144.959979	313.601784	7.07756E-06	0.008103518
1144.999979	324.084698	7.31414E-06	0.00837469
1145.039979	325.795277	7.35275E-06	0.008419188
1145.079979	371.587469	8.38621E-06	0.009602883
1145.119979	452.487733	1.0212E-05	0.011693986
1145.159979	538.103039	1.21442E-05	0.013907093
1145.199979	601.500135	1.3575E-05	0.015546113
1145.239979	618.512558	1.3959E-05	0.015986367
1145.279979	623.953807	1.40818E-05	0.016127567
1145.319979	612.714089	1.38281E-05	0.015837603
1145.359979	539.967336	1.21863E-05	0.013957713
1145.399979	420.188112	9.48306E-06	0.010861897
1145.439979	319.767017	7.2167E-06	0.008266292

1145.479979	249.190906	5.62389E-06	0.006442055
1145.519979	218.12468	4.92277E-06	0.005639132
1145.559979	227.119096	5.12576E-06	0.005871867
1145.599979	240.865329	5.43599E-06	0.006227476
1145.639979	255.081921	5.75684E-06	0.00659527
1145.679979	281.142012	6.34498E-06	0.007269321
1145.719979	317.376275	7.16274E-06	0.008206495
1145.759979	307.10581	6.93095E-06	0.007941206
1145.799979	293.584528	6.62579E-06	0.007591834
1145.839979	279.756975	6.31373E-06	0.007234519
1145.879979	283.813863	6.40528E-06	0.007339686
1145.919979	295.809253	6.676E-06	0.007650165
1145.959979	306.766827	6.9233E-06	0.007933825
1145.999979	313.109872	7.06645E-06	0.008098156
1146.039979	300.577436	6.78361E-06	0.007774293
1146.079979	317.041828	7.15519E-06	0.008200423
1146.119979	360.711145	8.14075E-06	0.009330274
1146.159979	414.045124	9.34442E-06	0.010710202
1146.199979	450.739234	1.01726E-05	0.011659784
1146.239979	453.625755	1.02377E-05	0.011734863
1146.279979	468.831564	1.05809E-05	0.012128646
1146.319979	487.456688	1.10012E-05	0.012610917
1146.359979	451.301738	1.01853E-05	0.011675965
1146.399979	384.745401	8.68317E-06	0.009954383
1146.439979	323.57125	7.30255E-06	0.008371938
1146.479979	260.23056	5.87304E-06	0.006733324
1146.519979	233.274845	5.26469E-06	0.00603607
1146.559979	258.21687	5.8276E-06	0.006681687
1146.599979	279.967351	6.31847E-06	0.007244761
1146.639979	291.424196	6.57704E-06	0.007541495
1146.679979	321.547107	7.25687E-06	0.008321308
1146.719979	332.691494	7.50838E-06	0.008610014
1146.759979	300.568054	6.7834E-06	0.007778934
1146.799979	277.595149	6.26494E-06	0.007184628
1146.839979	284.539943	6.42167E-06	0.007364628
1146.879979	295.944057	6.67905E-06	0.007660063
1146.919979	302.843844	6.83476E-06	0.007838927
1146.959979	294.406076	6.64434E-06	0.007620786
1146.999979	277.62859	6.26569E-06	0.007186747
1147.039979	306.100267	6.90826E-06	0.007924047
1147.079979	363.831186	8.21116E-06	0.009418861
1147.119979	422.138106	9.52707E-06	0.010928691
1147.159979	457.966864	1.03357E-05	0.011856672
1147.199979	498.193231	1.12435E-05	0.012898574
1147.239979	510.523384	1.15218E-05	0.013218272
1147.279979	498.511103	1.12507E-05	0.012907704

1147.319979	474.012731	1.06978E-05	0.012273808
1147.359979	420.727138	9.49523E-06	0.010894442
1147.399979	361.183423	8.15141E-06	0.009352924
1147.439979	315.36506	7.11735E-06	0.008166732
1147.479979	288.830215	6.5185E-06	0.007479843
1147.519979	273.273634	6.16741E-06	0.007077221
1147.559979	255.54011	5.76718E-06	0.00661819
1147.599979	252.391938	5.69613E-06	0.006536884
1147.639979	262.97693	5.93502E-06	0.00681127
1147.679979	253.308192	5.71681E-06	0.006561072
1147.719979	237.264089	5.35472E-06	0.006145719
1147.759979	253.000963	5.70988E-06	0.006553571
1147.799979	254.901291	5.75277E-06	0.006603026
1147.839979	253.237029	5.71521E-06	0.006560143
1147.879979	239.984095	5.41611E-06	0.006217041
1147.919979	242.395177	5.47052E-06	0.006279721
1147.959979	259.040699	5.84619E-06	0.00671119
1147.999979	263.80253	5.95366E-06	0.006834797
1148.039979	294.45671	6.64548E-06	0.007629274
1148.079979	329.550577	7.4375E-06	0.008538842
1148.119979	376.39228	8.49465E-06	0.009752877
1148.159979	427.523438	9.64861E-06	0.011078146
1148.199979	451.063137	1.01799E-05	0.011688523
1148.239979	473.39089	1.06838E-05	0.012267535
1148.279979	460.422563	1.03911E-05	0.011931887
1148.319979	383.395201	8.6527E-06	0.009936063
1148.359979	311.908173	7.03933E-06	0.008083688
1148.399979	274.041487	6.18473E-06	0.007102549
1148.439979	231.341182	5.22105E-06	0.005996061
1148.479979	212.165071	4.78827E-06	0.005499232
1148.519979	209.39369	4.72572E-06	0.005427588
1148.559979	214.502886	4.84103E-06	0.005560215
1148.599979	227.524995	5.13492E-06	0.005897972
1148.639979	256.150846	5.78097E-06	0.006640251
1148.679979	269.4741	6.08166E-06	0.006985875
1148.719979	272.032918	6.1394E-06	0.007052456
1148.759979	277.228616	6.25666E-06	0.007187405
1148.799979	273.465854	6.17174E-06	0.007090099
1148.839979	281.429627	6.35147E-06	0.007296828
1148.879979	279.397607	6.30561E-06	0.007244395
1148.919979	277.910515	6.27205E-06	0.007206087
1148.959979	287.592553	6.49056E-06	0.007457398
1148.999979	306.743644	6.92278E-06	0.00795427
1149.039979	317.996109	7.17673E-06	0.008246349
1149.079979	342.977095	7.74052E-06	0.008894471
1149.119979	401.528154	9.06193E-06	0.010413246

1149.159979	452.698118	1.02168E-05	0.011740699
1149.199979	473.958753	1.06966E-05	0.01229252
1149.239979	484.008069	1.09234E-05	0.012553594
1149.279979	468.571402	1.0575E-05	0.01215364
1149.319979	420.849038	9.49798E-06	0.010916214
1149.359979	360.541622	8.13692E-06	0.009352253
1149.399979	316.092067	7.13376E-06	0.008199541
1149.439979	272.487137	6.14966E-06	0.007068659
1149.479979	235.001744	5.30366E-06	0.006096453
1149.519979	204.531431	4.61599E-06	0.005306172
1149.559979	201.574203	4.54925E-06	0.005229635
1149.599979	219.661692	4.95746E-06	0.005699094
1149.639979	232.813387	5.25427E-06	0.006040523
1149.679979	252.175324	5.69125E-06	0.006543111
1149.719979	270.252981	6.09923E-06	0.00701241
1149.759979	264.389642	5.96691E-06	0.00686051
1149.799979	262.983158	5.93516E-06	0.006824251
1149.839979	243.266147	5.49018E-06	0.006312826
1149.879979	231.643718	5.22788E-06	0.00601143
1149.919979	219.752723	4.95951E-06	0.005703043
1149.959979	221.170564	4.99151E-06	0.005740039
1149.999979	245.88116	5.5492E-06	0.006381574
1150.039979	274.695863	6.1995E-06	0.007129676
1150.079979	328.60456	7.41615E-06	0.008529162
1150.119979	378.571705	8.54384E-06	0.009826437
1150.159979	424.336087	9.57667E-06	0.011014708
1150.199979	463.771862	1.04667E-05	0.012038781
1150.239979	438.154921	9.88855E-06	0.011374201
1150.279979	392.119465	8.84959E-06	0.010179506
1150.319979	380.278169	8.58235E-06	0.009872447
1150.359979	339.574718	7.66373E-06	0.008816046
1150.399979	278.819434	6.29257E-06	0.007238968
1150.439979	264.190378	5.96241E-06	0.006859394
1150.479979	256.854904	5.79686E-06	0.006669168
1150.519979	244.899944	5.52705E-06	0.006358982
1150.559979	239.639764	5.40834E-06	0.006222615
1150.599979	254.833419	5.75124E-06	0.006617371
1150.639979	286.500088	6.46591E-06	0.007439932
1150.679979	322.154265	7.27057E-06	0.008366103
1150.719979	358.547417	8.09192E-06	0.009311529
1150.759979	352.858442	7.96352E-06	0.009164104
1150.799979	310.81171	7.01459E-06	0.008072387
1150.839979	291.580867	6.58057E-06	0.007573188
1150.879979	287.576122	6.49019E-06	0.007469433
1150.919979	290.842174	6.5639E-06	0.007554527
1150.959979	295.410603	6.66701E-06	0.007673457

1150.999979	326.642666	7.37187E-06	0.008485022
1151.039979	354.414592	7.99864E-06	0.009206759
1151.079979	369.33559	8.33539E-06	0.0095947
1151.119979	366.8999	8.28042E-06	0.009531757
1151.159979	392.293201	8.85351E-06	0.010191808
1151.199979	458.016443	1.03368E-05	0.011899716
1151.239979	485.07877	1.09476E-05	0.01260326
1151.279979	481.292365	1.08621E-05	0.012505316
1151.319979	451.626332	1.01926E-05	0.011734918
1151.359979	406.259213	9.1687E-06	0.010556479
1151.399979	351.25714	7.92738E-06	0.00912759
1151.439979	282.544934	6.37665E-06	0.007342325
1151.479979	228.265306	5.15163E-06	0.005931999
1151.519979	235.529286	5.31557E-06	0.006120983
1151.559979	252.06844	5.68883E-06	0.006551033
1151.599979	234.620102	5.29505E-06	0.006097778
1151.639979	225.097592	5.08014E-06	0.005850491
1151.679979	232.823827	5.25451E-06	0.006051513
1151.719979	250.798461	5.66017E-06	0.006518933
1151.759979	281.208282	6.34648E-06	0.007309621
1151.799979	275.838952	6.2253E-06	0.007170301
1151.839979	248.291795	5.6036E-06	0.00645445
1151.879979	253.894101	5.73004E-06	0.006600314
1151.919979	268.439212	6.0583E-06	0.006978676
1151.959979	261.514777	5.90202E-06	0.006798896
1151.999979	272.945471	6.16E-06	0.007096319
1152.039979	298.825679	6.74408E-06	0.007769449
1152.079979	315.237115	7.11446E-06	0.00819643
1152.119979	353.573775	7.97967E-06	0.009193534
1152.159979	397.307807	8.96668E-06	0.010331054
1152.199979	413.643107	9.33535E-06	0.010756189
1152.239979	404.43861	9.12762E-06	0.010517204
1152.279979	410.615885	9.26703E-06	0.010678212
1152.319979	423.230624	9.55173E-06	0.011006644
1152.359979	402.788592	9.09038E-06	0.010475387
1152.399979	367.873047	8.30238E-06	0.009567665
1152.439979	344.39384	7.77249E-06	0.008957327
1152.479979	322.680221	7.28244E-06	0.00839287
1152.519979	315.602575	7.12271E-06	0.008209066
1152.559979	310.927274	7.0172E-06	0.008087739
1152.599979	313.407358	7.07317E-06	0.008152533
1152.639979	335.781225	7.57811E-06	0.008734838
1152.679979	345.127196	7.78904E-06	0.008978271
1152.719979	383.394955	8.65269E-06	0.009974129
1152.759979	430.070939	9.7061E-06	0.011188806
1152.799979	477.898841	1.07855E-05	0.012433537

1152.839979	543.34952	1.22626E-05	0.014136864
1152.879979	622.310207	1.40447E-05	0.016191824
1152.919979	695.895716	1.57054E-05	0.018107066
1152.959979	775.401655	1.74997E-05	0.020176494
1152.999979	867.122085	1.95697E-05	0.022563907
1153.039979	1007.112046	2.27291E-05	0.02620758
1153.079979	1196.463213	2.70025E-05	0.031136052
1153.119979	1399.597827	3.1587E-05	0.03642357
1153.159979	1680.604953	3.79289E-05	0.043738104
1153.199979	2058.061968	4.64476E-05	0.053563362
1153.239979	2425.494203	5.474E-05	0.063128386
1153.279979	2615.776777	5.90344E-05	0.068083236
1153.319979	2584.090789	5.83193E-05	0.067260848
1153.359979	2235.347158	5.04487E-05	0.058185479
1153.399979	1696.577157	3.82894E-05	0.044162974
1153.439979	1139.78201	2.57233E-05	0.029670274
1153.479979	730.099461	1.64773E-05	0.019006268
1153.519979	479.389965	1.08192E-05	0.012480122
1153.559979	351.402501	7.93066E-06	0.009148498
1153.599979	299.749365	6.76493E-06	0.007804018
1153.639979	280.222637	6.32423E-06	0.00729589
1153.679979	285.477368	6.44283E-06	0.00743296
1153.719979	335.168654	7.56429E-06	0.008727072
1153.759979	372.438623	8.40542E-06	0.009697838
1153.799979	384.565686	8.67911E-06	0.010013959
1153.839979	364.120201	8.21769E-06	0.009481894
1153.879979	369.40826	8.33703E-06	0.009619932
1153.919979	416.583568	9.40171E-06	0.010848822
1153.959979	434.053399	9.79598E-06	0.01130417
1153.999979	482.24849	1.08837E-05	0.012559763
1154.039979	577.702234	1.30379E-05	0.015046298
1154.079979	664.361467	1.49937E-05	0.017303944
1154.119979	783.726088	1.76876E-05	0.02041362
1154.159979	957.269572	2.16042E-05	0.02493475
1154.199979	1122.538438	2.53341E-05	0.029240651
1154.239979	1281.582652	2.89235E-05	0.033384701
1154.279979	1391.900038	3.14132E-05	0.03625968
1154.319979	1359.772555	3.06882E-05	0.035423971
1154.359979	1213.102515	2.7378E-05	0.031604107
1154.399979	970.643282	2.19061E-05	0.025288363
1154.439979	725.100421	1.63645E-05	0.018891841
1154.479979	511.110852	1.15351E-05	0.013316996
1154.519979	343.209659	7.74576E-06	0.008942639
1154.559979	250.958696	5.66379E-06	0.006539183
1154.599979	233.670185	5.27361E-06	0.006088911
1154.639979	238.001947	5.37137E-06	0.006202001

1154.679979	250.166655	5.64591E-06	0.006519223
1154.719979	258.436294	5.83255E-06	0.006734959
1154.759979	272.993903	6.16109E-06	0.007114583
1154.799979	308.730289	6.96761E-06	0.008046199
1154.839979	327.506646	7.39137E-06	0.008535848
1154.879979	316.12314	7.13446E-06	0.008239444
1154.919979	303.648044	6.85291E-06	0.007914567
1154.959979	332.787756	7.51056E-06	0.008674392
1154.999979	366.835	8.27895E-06	0.009562193
1155.039979	405.482232	9.15117E-06	0.010569966
1155.079979	466.897316	1.05372E-05	0.012171334
1155.119979	543.752693	1.22717E-05	0.014175333
1155.159979	687.810156	1.55229E-05	0.017931453
1155.199979	800.60911	1.80686E-05	0.020872884
1155.239979	846.682078	1.91084E-05	0.022074828
1155.279979	839.090918	1.89371E-05	0.021877668
1155.319979	787.066695	1.7763E-05	0.020521948
1155.359979	697.93484	1.57514E-05	0.018198557
1155.399979	560.096878	1.26406E-05	0.014604956
1155.439979	430.874529	9.72424E-06	0.011235773
1155.479979	335.433114	7.57026E-06	0.008747282
1155.519979	267.408534	6.03504E-06	0.006973607
1155.559979	254.722899	5.74874E-06	0.006643015
1155.599979	280.643132	6.33372E-06	0.007319252
1155.639979	285.728457	6.44849E-06	0.007452137
1155.679979	279.853481	6.3159E-06	0.007299163
1155.719979	282.606302	6.37803E-06	0.007371217
1155.759979	286.75051	6.47156E-06	0.00747957
1155.799979	301.229187	6.79832E-06	0.007857502
1155.839979	297.227666	6.70801E-06	0.007753391
1155.879979	268.870858	6.06804E-06	0.007013927
1155.919979	257.208569	5.80484E-06	0.00670993
1155.959979	258.284084	5.82911E-06	0.00673822
1155.999979	281.09275	6.34387E-06	0.007333516
1156.039979	316.034105	7.13245E-06	0.008245397
1156.079979	343.713286	7.75713E-06	0.008967863
1156.119979	384.653052	8.68108E-06	0.010036374
1156.159979	445.355338	1.0051E-05	0.011620621
1156.199979	476.403605	1.07518E-05	0.012431191
1156.239979	498.622106	1.12532E-05	0.013011407
1156.279979	518.38779	1.16993E-05	0.013527655
1156.319979	483.627594	1.09148E-05	0.012621003
1156.359979	434.446447	9.80485E-06	0.011337937
1156.399979	381.416654	8.60804E-06	0.00995434
1156.439979	319.5739	7.21234E-06	0.008340636
1156.479979	255.933187	5.77606E-06	0.006679893

1156.519979	234.896261	5.30128E-06	0.006131038
1156.559979	241.44787	5.44914E-06	0.00630226
1156.599979	253.788827	5.72766E-06	0.006624612
1156.639979	285.06537	6.43353E-06	0.007441276
1156.679979	283.307701	6.39386E-06	0.00739565
1156.719979	279.8927	6.31679E-06	0.007306755
1156.759979	289.370004	6.53068E-06	0.007554427
1156.799979	286.148412	6.45797E-06	0.007470581
1156.839979	250.481795	5.65303E-06	0.006539646
1156.879979	258.450126	5.83286E-06	0.006747918
1156.919979	280.952825	6.34071E-06	0.007335699
1156.959979	293.240487	6.61803E-06	0.007656795
1156.999979	321.929887	7.26551E-06	0.008406194
1157.039979	350.267778	7.90506E-06	0.009146466
1157.079979	382.621986	8.63525E-06	0.009991669
1157.119979	422.888557	9.54401E-06	0.01104356
1157.159979	449.061731	1.01347E-05	0.011727467
1157.199979	504.214498	1.13794E-05	0.013168263
1157.239979	539.153588	1.21679E-05	0.014081233
1157.279979	542.882601	1.22521E-05	0.014179115
1157.319979	499.42292	1.12713E-05	0.013044477
1157.359979	427.078897	9.63858E-06	0.011155302
1157.399979	374.982873	8.46284E-06	0.009794892
1157.439979	329.559332	7.4377E-06	0.008608686
1157.479979	267.111903	6.02834E-06	0.006977687
1157.519979	235.655853	5.31842E-06	0.006156182
1157.559979	240.606716	5.43016E-06	0.006285734
1157.599979	219.044452	4.94353E-06	0.005722628
1157.639979	210.262246	4.74533E-06	0.005493379
1157.679979	246.349068	5.55976E-06	0.006436417
1157.719979	277.279625	6.25781E-06	0.007244797
1157.759979	284.280555	6.41582E-06	0.007427975
1157.799979	285.856772	6.45139E-06	0.007469418
1157.839979	268.646376	6.06297E-06	0.007019954
1157.879979	235.995722	5.32609E-06	0.006166978
1157.919979	221.569602	5.00052E-06	0.005790199
1157.959979	248.85588	5.61633E-06	0.006503486
1157.999979	296.290666	6.68687E-06	0.007743393
1158.039979	298.188186	6.72969E-06	0.007793252
1158.079979	283.107027	6.38933E-06	0.007399357
1158.119979	305.058702	6.88475E-06	0.007973367
1158.159979	378.973875	8.55291E-06	0.009905641
1158.199979	426.242109	9.61969E-06	0.011141526
1158.239979	438.917451	9.90576E-06	0.011473242
1158.279979	425.413154	9.60098E-06	0.011120626
1158.319979	395.721461	8.93088E-06	0.010344819

1158.359979	364.182651	8.2191E-06	0.009520671
1158.399979	338.623698	7.64227E-06	0.0088528
1158.439979	293.556358	6.62516E-06	0.007674848
1158.479979	244.174779	5.51068E-06	0.006384018
1158.519979	237.841159	5.36774E-06	0.006218638
1158.559979	266.426992	6.01289E-06	0.006966289
1158.599979	276.866369	6.24849E-06	0.007239498
1158.639979	258.5139	5.8343E-06	0.006759852
1158.679979	279.199733	6.30115E-06	0.007301015
1158.719979	299.036545	6.74884E-06	0.007820014
1158.759979	274.151553	6.18722E-06	0.007169501
1158.799979	282.404566	6.37348E-06	0.007385586
1158.839979	292.355645	6.59806E-06	0.007646095
1158.879979	293.281331	6.61895E-06	0.00767057
1158.919979	303.81334	6.85664E-06	0.007946302
1158.959979	316.849298	7.15085E-06	0.008287546
1158.999979	339.323788	7.65807E-06	0.008875697
1159.039979	348.192999	7.85823E-06	0.009108004
1159.079979	369.152664	8.33126E-06	0.009656598
1159.119979	395.865399	8.93413E-06	0.010355729
1159.159979	463.003882	1.04494E-05	0.012112471
1159.199979	549.6815	1.24055E-05	0.014380508
1159.239979	553.767064	1.24978E-05	0.014487893
1159.279979	524.419957	1.18354E-05	0.013720575
1159.319979	484.176516	1.09272E-05	0.012668109
1159.359979	427.438765	9.6467E-06	0.011183995
1159.399979	370.113306	8.35294E-06	0.009684401
1159.439979	325.318928	7.342E-06	0.008512603
1159.479979	268.029779	6.04906E-06	0.007013763
1159.519979	214.519313	4.8414E-06	0.005613702
1159.559979	194.023964	4.37885E-06	0.00507754
1159.599979	211.340552	4.76966E-06	0.0055309
1159.639979	235.75575	5.32068E-06	0.006170072
1159.679979	249.990966	5.64195E-06	0.006542854
1159.719979	235.112986	5.30617E-06	0.006153674
1159.759979	216.426512	4.88444E-06	0.005664784
1159.799979	222.390487	5.01904E-06	0.005821087
1159.839979	217.359992	4.90551E-06	0.005689609
1159.879979	222.153543	5.0137E-06	0.005815286
1159.919979	219.21643	4.94741E-06	0.005738599
1159.959979	215.188792	4.85651E-06	0.005633359
1159.999979	211.575488	4.77496E-06	0.005538958
1160.039979	246.824433	5.57048E-06	0.006461984
1160.079979	288.372073	6.50816E-06	0.007549981
1160.119979	333.62438	7.52944E-06	0.008735051
1160.159979	380.090305	8.57811E-06	0.009951978

1160.199979	426.939612	9.63543E-06	0.011179028
1160.239979	473.830737	1.06937E-05	0.012407258
1160.279979	486.16314	1.0972E-05	0.012730621
1160.319979	453.205031	1.02282E-05	0.011867992
1160.359979	405.294409	9.14693E-06	0.010613732
1160.399979	313.785248	7.0817E-06	0.0082176
1160.439979	261.657104	5.90524E-06	0.006852672
1160.479979	261.915658	5.91107E-06	0.00685968
1160.519979	238.058038	5.37264E-06	0.006235054
1160.559979	231.629921	5.22756E-06	0.006066902
1160.599979	244.842932	5.52576E-06	0.006413202
1160.639979	249.594266	5.63299E-06	0.006537879
1160.679979	246.861856	5.57133E-06	0.006466529
1160.719979	251.213132	5.66953E-06	0.006580737
1160.759979	266.122675	6.00602E-06	0.006971545
1160.799979	294.950513	6.65662E-06	0.007727007
1160.839979	301.562626	6.80585E-06	0.007900501
1160.879979	289.796718	6.54031E-06	0.007592513
1160.919979	261.354161	5.8984E-06	0.00684757
1160.959979	255.424864	5.76458E-06	0.006692451
1160.999979	279.760751	6.31381E-06	0.007330334
1161.039979	343.051344	7.74219E-06	0.008988993
1161.079979	387.37673	8.74255E-06	0.010150804
1161.119979	427.769837	9.65417E-06	0.011209649
1161.159979	482.52236	1.08899E-05	0.012644867
1161.199979	516.607087	1.16591E-05	0.013538549
1161.239979	514.529796	1.16122E-05	0.013484575
1161.279979	498.522389	1.1251E-05	0.01306551
1161.319979	452.66352	1.0216E-05	0.011864028
1161.359979	400.679949	9.04279E-06	0.010501932
1161.399979	337.470914	7.61625E-06	0.008845511
1161.439979	291.715411	6.58361E-06	0.007646468
1161.479979	273.006303	6.16137E-06	0.00715631
1161.519979	258.465768	5.83321E-06	0.006775393
1161.559979	243.346432	5.49199E-06	0.006379276
1161.599979	244.105188	5.50911E-06	0.006399387
1161.639979	257.748656	5.81703E-06	0.006757292
1161.679979	257.125802	5.80297E-06	0.006741195
1161.719979	262.332965	5.92049E-06	0.006877951
1161.759979	261.379681	5.89898E-06	0.006853193
1161.799979	233.500004	5.26977E-06	0.006122419
1161.839979	205.798556	4.64459E-06	0.005396267
1161.879979	188.508611	4.25438E-06	0.004943075
1161.919978	206.295236	4.6558E-06	0.005409663
1161.959978	244.182693	5.51086E-06	0.006403403
1161.999978	261.989576	5.91274E-06	0.006870604

1162.039978	265.70827	5.99667E-06	0.006968365
1162.079978	288.242017	6.50522E-06	0.007559587
1162.119978	336.92778	7.60399E-06	0.008836749
1162.159978	403.992558	9.11755E-06	0.010596051
1162.199978	462.3645	1.04349E-05	0.012127467
1162.239978	455.20539	1.02734E-05	0.0119401
1162.279978	415.184561	9.37014E-06	0.010890723
1162.319978	395.726241	8.93099E-06	0.010380668
1162.359978	357.421799	8.06651E-06	0.009376191
1162.399978	299.409552	6.75726E-06	0.007854635
1162.439978	267.228563	6.03098E-06	0.007010648
1162.479978	268.05488	6.04963E-06	0.007032568
1162.519978	267.144201	6.02907E-06	0.007008917
1162.559978	259.523121	5.85708E-06	0.006809201
1162.599978	233.779373	5.27607E-06	0.006133964
1162.639978	229.845455	5.18729E-06	0.006030953
1162.679978	235.66758	5.31869E-06	0.006183933
1162.719978	247.224547	5.57951E-06	0.006487412
1162.759978	274.396281	6.19274E-06	0.007200672
1162.799978	279.529278	6.30859E-06	0.007335624
1162.839978	251.814818	5.68311E-06	0.006608547
1162.879978	239.594264	5.40731E-06	0.006288051
1162.919978	257.719619	5.81637E-06	0.006763976
1162.959978	281.261234	6.34767E-06	0.007382091
1162.999978	281.606809	6.35547E-06	0.007391415
1163.039978	272.134439	6.1417E-06	0.007143037
1163.079978	295.515492	6.66937E-06	0.007757014
1163.119978	356.167812	8.03821E-06	0.009349404
1163.159978	436.135039	9.84296E-06	0.011448938
1163.199978	533.912025	1.20497E-05	0.014016153
1163.239978	544.458859	1.22877E-05	0.014293518
1163.279978	491.858927	1.11006E-05	0.012913072
1163.319978	441.207641	9.95744E-06	0.011583691
1163.359978	409.85296	9.24981E-06	0.010760859
1163.399978	384.127803	8.66923E-06	0.010085781
1163.439978	344.264606	7.76957E-06	0.009039431
1163.479978	303.32025	6.84552E-06	0.00796462
1163.519978	247.034413	5.57522E-06	0.006486883
1163.559978	220.88286	4.98502E-06	0.005800368
1163.599978	224.78327	5.07305E-06	0.005902995
1163.639978	245.055984	5.53057E-06	0.006435595
1163.679978	269.009074	6.07116E-06	0.007064887
1163.719978	301.959648	6.81481E-06	0.007930529
1163.759978	336.115606	7.58566E-06	0.008827889
1163.799978	336.739837	7.59975E-06	0.008844588
1163.839978	317.815906	7.17266E-06	0.008347831

1163.879978	273.532798	6.17325E-06	0.007184927
1163.919978	252.108628	5.68974E-06	0.006622403
1163.959978	257.001384	5.80016E-06	0.006751158
1163.999978	271.3374	6.12371E-06	0.007127995
1164.039978	286.507289	6.46607E-06	0.007526764
1164.079978	300.269735	6.77667E-06	0.007888585
1164.119978	318.95903	7.19846E-06	0.008379872
1164.159978	349.113977	7.87902E-06	0.009172435
1164.199978	393.329839	8.87691E-06	0.010334494
1164.239978	432.775211	9.76713E-06	0.011371288
1164.279978	443.395201	1.00068E-05	0.011650731
1164.319978	444.190108	1.00248E-05	0.011672019
1164.359978	419.682245	9.47164E-06	0.011028403
1164.399978	346.21325	7.81355E-06	0.009098098
1164.439978	280.708278	6.33519E-06	0.007376954
1164.479978	256.568519	5.79039E-06	0.006742798
1164.519978	259.20509	5.8499E-06	0.006812323
1164.559978	258.700111	5.8385E-06	0.006799285
1164.599978	261.696004	5.90611E-06	0.006878261
1164.639978	282.598019	6.37784E-06	0.007427892
1164.679978	295.516735	6.6694E-06	0.007767718
1164.719978	297.0848	6.70479E-06	0.007809203
1164.759978	283.310005	6.39391E-06	0.007447373
1164.799978	290.0128	6.54518E-06	0.007623831
1164.839978	294.293163	6.64179E-06	0.007736619
1164.879978	277.51911	6.26322E-06	0.007295899
1164.919978	265.67562	5.99593E-06	0.006984777
1164.959978	266.854986	6.02255E-06	0.007016024
1164.999978	288.140143	6.50292E-06	0.007575904
1165.039978	333.144674	7.51861E-06	0.008759483
1165.079978	360.267679	8.13074E-06	0.009472962
1165.119978	390.126096	8.8046E-06	0.010258418
1165.159978	421.718363	9.5176E-06	0.011089522
1165.199978	467.944051	1.05608E-05	0.012305497
1165.239978	486.46256	1.09788E-05	0.012792916
1165.279978	465.368374	1.05027E-05	0.012238605
1165.319978	454.865732	1.02657E-05	0.011962809
1165.359978	453.869177	1.02432E-05	0.01193701
1165.399978	404.645376	9.13228E-06	0.010642762
1165.439978	320.221149	7.22695E-06	0.008422571
1165.479978	245.679635	5.54465E-06	0.006462175
1165.519978	209.575804	4.72983E-06	0.005512716
1165.559978	194.394023	4.3872E-06	0.005113547
1165.599978	207.805499	4.68988E-06	0.005466525
1165.639978	221.720024	5.00391E-06	0.00583276
1165.679978	243.394414	5.49307E-06	0.006403165

1165.719978	274.943243	6.20509E-06	0.007233393
1165.759978	296.795842	6.69827E-06	0.007808574
1165.799978	289.26692	6.52835E-06	0.007610752
1165.839978	265.051671	5.98185E-06	0.006973876
1165.879978	235.887909	5.32366E-06	0.00620675
1165.919978	217.945174	4.91872E-06	0.005734833
1165.959978	243.926347	5.50508E-06	0.0064187
1165.999978	244.97164	5.52867E-06	0.006446428
1166.039978	258.102575	5.82502E-06	0.006792201
1166.079978	290.538422	6.55705E-06	0.007646042
1166.119978	312.728261	7.05784E-06	0.00823029
1166.159978	314.69433	7.10221E-06	0.008282316
1166.199978	331.221376	7.47521E-06	0.008717584
1166.239978	361.323323	8.15456E-06	0.009510179
1166.279978	368.934803	8.32634E-06	0.009710849
1166.319978	374.273373	8.44683E-06	0.009851705
1166.359978	383.165831	8.64752E-06	0.01008612
1166.399978	356.250675	8.04008E-06	0.009377951
1166.439978	303.042647	6.83925E-06	0.007977575
1166.479978	243.916689	5.50486E-06	0.006421309
1166.519978	218.860878	4.93939E-06	0.005761891
1166.559978	223.615524	5.04669E-06	0.005887268
1166.599978	227.426633	5.1327E-06	0.00598781
1166.639978	226.591479	5.11385E-06	0.005966027
1166.679978	250.787947	5.65993E-06	0.006603332
1166.719978	264.829403	5.97683E-06	0.006973288
1166.759978	254.535149	5.7445E-06	0.006702457
1166.799978	258.515201	5.83433E-06	0.006807494
1166.839978	250.777431	5.6597E-06	0.006603961
1166.879978	255.596473	5.76846E-06	0.006731096
1166.919978	259.921905	5.86608E-06	0.006845241
1166.959978	265.214119	5.98551E-06	0.006984854
1166.999978	272.94625	6.16002E-06	0.007188739
1167.039978	303.247085	6.84386E-06	0.007987063
1167.079978	347.278053	7.83758E-06	0.009147085
1167.119978	377.48602	8.51933E-06	0.009943085
1167.159978	401.192044	9.05435E-06	0.01056787
1167.199978	428.600878	9.67292E-06	0.011290238
1167.239978	463.675191	1.04645E-05	0.012214587
1167.279978	467.760992	1.05567E-05	0.012322641
1167.319978	444.110939	1.0023E-05	0.011700008
1167.359978	379.936303	8.57463E-06	0.010009684
1167.399978	293.362669	6.62079E-06	0.007729106
1167.439978	245.32379	5.53662E-06	0.006463667
1167.479978	204.272318	4.61014E-06	0.005382248
1167.519978	195.873067	4.42058E-06	0.005161118

1167.559978	222.93246	5.03128E-06	0.005874315
1167.599978	219.050868	4.94367E-06	0.005772232
1167.639978	214.131745	4.83266E-06	0.005642801
1167.679978	225.687001	5.09344E-06	0.005947509
1167.719978	253.824975	5.72848E-06	0.006689256
1167.759978	261.626359	5.90454E-06	0.006895088
1167.799978	250.442376	5.65214E-06	0.006600564
1167.839978	261.479779	5.90123E-06	0.006891697
1167.879978	261.252163	5.8961E-06	0.006885934
1167.919978	256.521191	5.78933E-06	0.006761469
1167.959978	254.226533	5.73754E-06	0.006701216
1167.999978	255.909484	5.77552E-06	0.006745808
1168.039978	269.777833	6.08851E-06	0.007111623
1168.079978	319.530289	7.21135E-06	0.008423438
1168.119978	361.281516	8.15362E-06	0.009524407
1168.159978	373.487903	8.4291E-06	0.009846539
1168.199978	381.042362	8.5996E-06	0.010046047
1168.239978	402.897048	9.09283E-06	0.010622602
1168.279978	453.949849	1.0245E-05	0.011969047
1168.319978	450.634179	1.01702E-05	0.011882031
1168.359978	391.388168	8.83309E-06	0.010320224
1168.399978	331.456275	7.48051E-06	0.008740224
1168.439978	286.995838	6.4771E-06	0.007568098
1168.479978	260.357904	5.87592E-06	0.006865889
1168.519978	236.1291	5.3291E-06	0.006227166
1168.559978	239.903648	5.41429E-06	0.006326924
1168.599978	273.706087	6.17717E-06	0.007218635
1168.639978	265.456958	5.99099E-06	0.007001315
1168.679978	269.132957	6.07396E-06	0.007098511
1168.719978	308.384817	6.95982E-06	0.008134076
1168.759978	315.49983	7.12039E-06	0.008322029
1168.799978	294.085375	6.6371E-06	0.007757439
1168.839978	300.880076	6.79044E-06	0.007936943
1168.879978	328.749654	7.41942E-06	0.008672414
1168.919978	339.923336	7.6716E-06	0.008967482
1168.959978	350.360768	7.90715E-06	0.009243147
1168.999978	354.813246	8.00764E-06	0.009360932
1169.039978	370.578045	8.36343E-06	0.009777184
1169.079978	417.374712	9.41957E-06	0.011012226
1169.119978	505.209498	1.14019E-05	0.013330159
1169.159978	563.519479	1.27178E-05	0.014869201
1169.199978	558.192675	1.25976E-05	0.01472915
1169.239978	544.49628	1.22885E-05	0.014368232
1169.279978	522.202491	1.17854E-05	0.013780412
1169.319978	492.772077	1.11212E-05	0.013004217
1169.359978	451.82009	1.0197E-05	0.011923905

1169.399978	399.757788	9.02198E-06	0.010550299
1169.439978	338.455971	7.63848E-06	0.008932744
1169.479978	279.025861	6.29723E-06	0.007364479
1169.519978	256.179298	5.78161E-06	0.006761708
1169.559978	226.509846	5.11201E-06	0.005978804
1169.599978	221.249044	4.99328E-06	0.005840143
1169.639978	253.671832	5.72502E-06	0.006696212
1169.679978	283.507582	6.39837E-06	0.007484047
1169.719978	274.094272	6.18593E-06	0.007235801
1169.759978	253.15248	5.7133E-06	0.006683188
1169.799978	266.881249	6.02314E-06	0.007045867
1169.839978	291.448691	6.57759E-06	0.007694729
1169.879978	295.592973	6.67112E-06	0.007804412
1169.919978	276.613675	6.24279E-06	0.007303559
1169.959978	298.462025	6.73587E-06	0.007880701
1169.999978	338.52856	7.64012E-06	0.008938938
1170.039978	341.801881	7.71399E-06	0.009025679
1170.079978	344.12837	7.7665E-06	0.009087424
1170.119978	371.410272	8.38221E-06	0.009808194
1170.159978	421.625872	9.51551E-06	0.011134667
1170.199978	487.247887	1.09965E-05	0.012868111
1170.239978	516.886294	1.16654E-05	0.013651322
1170.279978	545.987172	1.23222E-05	0.014420389
1170.319978	559.503031	1.26272E-05	0.014777869
1170.359978	537.374422	1.21278E-05	0.014193883
1170.399978	483.16223	1.09043E-05	0.012762391
1170.439978	419.799903	9.4743E-06	0.011089098
1170.479978	345.571672	7.79907E-06	0.009128657
1170.519978	281.494665	6.35294E-06	0.007436246
1170.559978	269.991728	6.09334E-06	0.007132617
1170.599978	277.669459	6.26661E-06	0.007335697
1170.639978	277.656729	6.26633E-06	0.007335611
1170.679978	268.416776	6.05779E-06	0.007091737
1170.719978	273.368818	6.16955E-06	0.007222819
1170.759978	280.116053	6.32183E-06	0.007401345
1170.799978	266.401154	6.0123E-06	0.007039204
1170.839978	272.617213	6.15259E-06	0.007203699
1170.879978	293.984903	6.63483E-06	0.007768589
1170.919978	300.840859	6.78956E-06	0.00795003
1170.959978	313.231664	7.0692E-06	0.008277753
1170.999978	319.530674	7.21136E-06	0.008444505
1171.039978	314.48769	7.09755E-06	0.008311514
1171.079978	358.696846	8.09529E-06	0.00948023
1171.119978	427.771711	9.65421E-06	0.01130624
1171.159978	455.488783	1.02797E-05	0.012039229
1171.199978	484.107696	1.09256E-05	0.012796105

1171.239978	544.773932	1.22948E-05	0.014400148
1171.279978	558.056138	1.25945E-05	0.014751744
1171.319978	521.920123	1.1779E-05	0.01379699
1171.359978	448.830593	1.01295E-05	0.011865269
1171.399978	360.489063	8.13574E-06	0.009530201
1171.439978	300.568337	6.78341E-06	0.007946356
1171.479978	277.012163	6.25178E-06	0.007323834
1171.519978	253.146506	5.71316E-06	0.006693086
1171.559978	212.619845	4.79853E-06	0.00562177
1171.599978	198.984416	4.4908E-06	0.005261422
1171.639978	214.133644	4.8327E-06	0.005662182
1171.679978	213.411925	4.81641E-06	0.005643291
1171.719978	234.481231	5.29191E-06	0.006200642
1171.759978	249.594603	5.633E-06	0.006600527
1171.799978	248.40825	5.60623E-06	0.006569378
1171.839978	254.939077	5.75362E-06	0.006742322
1171.879978	263.817516	5.95399E-06	0.006977366
1171.919978	246.61962	5.56586E-06	0.006522744
1171.959978	226.558548	5.11311E-06	0.005992361
1171.999978	217.527044	4.90928E-06	0.005753679
1172.039978	252.161984	5.69094E-06	0.006670015
1172.079978	302.479599	6.82654E-06	0.008001255
1172.119978	340.164905	7.67705E-06	0.008998421
1172.159978	384.819215	8.68483E-06	0.010180014
1172.199978	410.996664	9.27562E-06	0.010872884
1172.239978	427.353538	9.64477E-06	0.01130599
1172.279978	434.675814	9.81003E-06	0.011500099
1172.319978	468.394049	1.0571E-05	0.012392596
1172.359978	439.361884	9.91579E-06	0.01162487
1172.399978	367.276644	8.28892E-06	0.009717932
1172.439978	299.408788	6.75724E-06	0.007922458
1172.479978	266.866785	6.02281E-06	0.007061626
1172.519978	259.658066	5.86012E-06	0.006871109
1172.559978	251.160832	5.66835E-06	0.006646481
1172.599978	252.867434	5.70687E-06	0.006691871
1172.639978	271.654197	6.13086E-06	0.007189288
1172.679978	292.50547	6.60144E-06	0.007741378
1172.719978	294.651331	6.64987E-06	0.007798436
1172.759978	281.48115	6.35264E-06	0.007450119
1172.799978	272.899719	6.15897E-06	0.007223236
1172.839978	280.481042	6.33007E-06	0.007424155
1172.879978	271.486739	6.12708E-06	0.007186327
1172.919978	276.389954	6.23774E-06	0.007316366
1172.959978	286.000611	6.45464E-06	0.007571029
1172.999978	304.069267	6.86242E-06	0.008049618
1173.039978	333.456351	7.52565E-06	0.008827883

1173.079978	345.543824	7.79844E-06	0.009148197
1173.119978	360.849651	8.14387E-06	0.009553741
1173.159978	390.592409	8.81513E-06	0.010341554
1173.199978	430.832398	9.72329E-06	0.01140736
1173.239978	471.391285	1.06386E-05	0.012481683
1173.279978	502.727314	1.13459E-05	0.013311865
1173.319978	493.810101	1.11446E-05	0.013076189
1173.359978	443.520091	1.00096E-05	0.0117449
1173.399978	384.00415	8.66644E-06	0.010169199
1173.439978	344.684724	7.77905E-06	0.009128253
1173.479978	299.544994	6.76031E-06	0.007933092
1173.519978	227.669427	5.13818E-06	0.006029759
1173.559978	193.897449	4.376E-06	0.005135493
1173.599978	208.837488	4.71317E-06	0.005531378
1173.639978	238.835007	5.39017E-06	0.006326123
1173.679978	245.521059	5.54107E-06	0.006503441
1173.719978	246.321849	5.55914E-06	0.006524875
1173.759978	230.889631	5.21086E-06	0.006116296
1173.799978	224.142672	5.05859E-06	0.00593777
1173.839978	221.689193	5.00322E-06	0.005872975
1173.879978	212.411102	4.79382E-06	0.005627372
1173.919978	205.237292	4.63192E-06	0.005437503
1173.959978	215.1239	4.85505E-06	0.005699631
1173.999978	233.581109	5.2716E-06	0.006188859
1174.039978	251.68272	5.68013E-06	0.006668698
1174.079978	280.852754	6.33846E-06	0.007441854
1174.119978	325.058365	7.33611E-06	0.008613479
1174.159978	351.415311	7.93095E-06	0.009312209
1174.199978	406.456865	9.17317E-06	0.01077113
1174.239978	423.25109	9.55219E-06	0.011216561
1174.279978	401.01852	9.05043E-06	0.010627738
1174.319978	398.121039	8.98504E-06	0.010551309
1174.359978	376.916181	8.50647E-06	0.009989662
1174.399978	319.579711	7.21247E-06	0.008470323
1174.439978	290.082757	6.54676E-06	0.007688781
1174.479978	271.557124	6.12867E-06	0.007197996
1174.519978	231.838412	5.23227E-06	0.006145406
1174.559978	207.932737	4.69275E-06	0.005511919
1174.599978	214.746018	4.84652E-06	0.005692721
1174.639978	219.92791	4.96347E-06	0.005830286
1174.679978	227.828876	5.14178E-06	0.006039946
1174.719978	247.985317	5.59668E-06	0.006574536
1174.759978	272.052039	6.13984E-06	0.007212833
1174.799978	280.548778	6.3316E-06	0.007438358
1174.839978	261.560662	5.90306E-06	0.006935151
1174.879978	257.539483	5.81231E-06	0.006828764

1174.919978	260.066156	5.86933E-06	0.006895994
1174.959978	248.41666	5.60642E-06	0.006587317
1174.999978	241.486876	5.45002E-06	0.006403776
1175.039978	281.114681	6.34437E-06	0.007454885
1175.079978	360.619241	8.13867E-06	0.009563593
1175.119978	424.45511	9.57936E-06	0.011256898
1175.159978	469.392587	1.05935E-05	0.012449101
1175.199978	511.246748	1.15381E-05	0.013559607
1175.239978	548.937377	1.23888E-05	0.014559757
1175.279978	598.092014	1.34981E-05	0.015864051
1175.319978	587.941725	1.3269E-05	0.015595351
1175.359978	538.081323	1.21437E-05	0.014273273
1175.399978	472.781774	1.067E-05	0.012541548
1175.439978	385.671093	8.70406E-06	0.010231099
1175.479978	300.829288	6.7893E-06	0.007980684
1175.519978	235.216295	5.3085E-06	0.006240253
1175.559978	225.30053	5.08472E-06	0.005977392
1175.599978	264.413023	5.96743E-06	0.007015315
1175.639978	277.335254	6.25907E-06	0.007358413
1175.679978	268.293034	6.055E-06	0.007118742
1175.719978	249.475075	5.6303E-06	0.006619662
1175.759978	250.139786	5.64531E-06	0.006637526
1175.799978	227.583231	5.13624E-06	0.006039187
1175.839978	195.70684	4.41683E-06	0.005193486
1175.879978	188.692795	4.25853E-06	0.005007524
1175.919978	206.25308	4.65484E-06	0.005473725
1175.959978	248.987956	5.61931E-06	0.006608085
1175.999978	273.532464	6.17325E-06	0.007259738
1176.039978	295.356088	6.66578E-06	0.007839218
1176.079978	323.047225	7.29073E-06	0.008574477
1176.119978	356.724304	8.05077E-06	0.009468672
1176.159978	391.146375	8.82763E-06	0.010382704
1176.199978	411.621599	9.28973E-06	0.010926576
1176.239978	435.590053	9.83066E-06	0.011563216
1176.279978	437.854356	9.88176E-06	0.01162372
1176.319978	427.291158	9.64337E-06	0.011343684
1176.359978	410.453235	9.26336E-06	0.010897043
1176.399978	373.775766	8.4356E-06	0.009923638
1176.439978	331.344327	7.47798E-06	0.008797395
1176.479978	291.573584	6.58041E-06	0.00774172
1176.519978	277.102437	6.25382E-06	0.007357739
1176.559978	276.264635	6.23491E-06	0.007335743
1176.599978	270.764882	6.11079E-06	0.007189951
1176.639978	255.522376	5.76678E-06	0.006785429
1176.679978	230.878813	5.21061E-06	0.006131224
1176.719978	247.476786	5.58521E-06	0.006572224

1176.759978	271.58546	6.12931E-06	0.007212721
1176.799978	247.737419	5.59109E-06	0.006579593
1176.839978	248.72621	5.6134E-06	0.006606078
1176.879978	284.612545	6.42331E-06	0.007559463
1176.919978	292.686664	6.60553E-06	0.007774181
1176.959978	270.664526	6.10852E-06	0.007189485
1176.999978	251.454257	5.67497E-06	0.006679442
1177.039978	291.747902	6.58434E-06	0.007750036
1177.079978	367.184367	8.28684E-06	0.009754273
1177.119978	427.491143	9.64788E-06	0.011356712
1177.159978	482.269989	1.08842E-05	0.0128124
1177.199978	535.993261	1.20966E-05	0.014240142
1177.239978	579.478704	1.3078E-05	0.015395976
1177.279978	582.95183	1.31564E-05	0.015488779
1177.319978	530.417031	1.19708E-05	0.014093431
1177.359978	463.681109	1.04646E-05	0.012320645
1177.399978	395.668055	8.92968E-06	0.010513801
1177.439978	339.076043	7.65247E-06	0.009010329
1177.479978	290.690157	6.56047E-06	0.007724824
1177.519978	249.869891	5.63922E-06	0.006640289
1177.559978	219.846745	4.96163E-06	0.005842623
1177.599978	213.935119	4.82822E-06	0.005685709
1177.639978	240.029607	5.41713E-06	0.006379433
1177.679978	260.533696	5.87988E-06	0.00692462
1177.719978	272.755297	6.15571E-06	0.007249699
1177.759978	252.042137	5.68824E-06	0.006699381
1177.799978	217.266069	4.90339E-06	0.005775216
1177.839978	224.766089	5.07266E-06	0.005974779
1177.879978	221.28952	4.9942E-06	0.005882564
1177.919978	204.84651	4.6231E-06	0.005445642
1177.959978	199.937033	4.5123E-06	0.005315309
1177.999978	208.159099	4.69786E-06	0.00553408
1178.039978	246.07539	5.55358E-06	0.006542338
1178.079978	290.50924	6.55639E-06	0.00772395
1178.119978	339.047644	7.65183E-06	0.009014777
1178.159978	380.071158	8.57768E-06	0.010105875
1178.199978	385.52855	8.70084E-06	0.010251332
1178.239978	413.036605	9.32166E-06	0.010983153
1178.279978	452.037444	1.02019E-05	0.012020642
1178.319978	453.067958	1.02251E-05	0.012048455
1178.359978	412.264757	9.30424E-06	0.010963746
1178.399978	384.747203	8.68321E-06	0.010232293
1178.439978	345.119858	7.78887E-06	0.009178721
1178.479978	291.743961	6.58425E-06	0.007759412
1178.519978	253.03476	5.71064E-06	0.006730106
1178.559978	235.140648	5.3068E-06	0.006254378

1178.599978	242.782271	5.47926E-06	0.006457853
1178.639978	230.48765	5.20179E-06	0.006131032
1178.679978	205.243685	4.63206E-06	0.005459721
1178.719978	230.117871	5.19344E-06	0.006121611
1178.759978	261.18282	5.89453E-06	0.006948239
1178.799978	250.086955	5.64411E-06	0.006653282
1178.839978	237.152399	5.3522E-06	0.006309387
1178.879978	233.46064	5.26888E-06	0.006211379
1178.919978	254.782754	5.75009E-06	0.006778898
1178.959978	284.598106	6.42298E-06	0.00757244
1178.999978	311.088122	7.02083E-06	0.008277553
1179.039978	310.01922	6.9967E-06	0.008249391
1179.079978	331.589165	7.48351E-06	0.008823652
1179.119978	359.687558	8.11765E-06	0.00957168
1179.159978	379.414379	8.56285E-06	0.010096975
1179.199978	422.075673	9.52566E-06	0.011232658
1179.239978	439.975199	9.92963E-06	0.011709414
1179.279978	436.071988	9.84154E-06	0.011605928
1179.319978	428.46837	9.66993E-06	0.011403947
1179.359978	388.62953	8.77083E-06	0.010343963
1179.399978	345.963861	7.80792E-06	0.009208664
1179.439978	307.836116	6.94743E-06	0.008194079
1179.479978	264.905614	5.97855E-06	0.007051581
1179.519978	230.903984	5.21118E-06	0.006146692
1179.559978	214.932204	4.85072E-06	0.005721716
1179.599978	201.766822	4.5536E-06	0.005371422
1179.639978	201.049416	4.53741E-06	0.005352505
1179.679978	225.509342	5.08943E-06	0.006003901
1179.719978	239.812237	5.41223E-06	0.006384914
1179.759978	239.564332	5.40663E-06	0.00637853
1179.799978	230.480675	5.20163E-06	0.00613688
1179.839978	210.036317	4.74023E-06	0.005592709
1179.879978	189.188593	4.26972E-06	0.005037761
1179.919978	203.736153	4.59804E-06	0.005425321
1179.959978	210.527077	4.7513E-06	0.005606347
1179.999978	243.623559	5.49824E-06	0.006487928
1180.039978	287.890745	6.49729E-06	0.007667066
1180.079978	294.90813	6.65567E-06	0.007854218
1180.119978	298.297403	6.73216E-06	0.007944753
1180.159978	328.532237	7.41451E-06	0.008750314
1180.199978	377.5059	8.51978E-06	0.010055047
1180.239978	402.632919	9.08686E-06	0.01072468
1180.279978	386.488915	8.72252E-06	0.010295012
1180.319978	352.005256	7.94427E-06	0.009376779
1180.359978	334.052017	7.53909E-06	0.008898839
1180.399978	301.11798	6.79581E-06	0.008021778

1180.439978	278.789957	6.2919E-06	0.007427211
1180.479978	254.886341	5.75243E-06	0.006790628
1180.519978	233.918001	5.2792E-06	0.006232205
1180.559978	216.718726	4.89104E-06	0.005774166
1180.599978	179.261796	4.04569E-06	0.00477634
1180.639978	180.298429	4.06908E-06	0.004804124
1180.679978	195.034525	4.40166E-06	0.005196949
1180.719978	195.991728	4.42326E-06	0.005222632
1180.759978	206.138158	4.65225E-06	0.005493192
1180.799978	223.627156	5.04695E-06	0.005959442
1180.839978	223.523606	5.04462E-06	0.005956885
1180.879978	227.443372	5.13308E-06	0.006061552
1180.919978	248.812393	5.61535E-06	0.006631278
1180.959978	257.371629	5.80852E-06	0.006859629
1180.999978	251.875176	5.68447E-06	0.006713361
1181.039978	284.658833	6.42435E-06	0.007587418
1181.079978	332.298967	7.49952E-06	0.008857539
1181.119978	358.313103	8.08663E-06	0.009551277
1181.159978	377.768831	8.52572E-06	0.010070235
1181.199978	401.687581	9.06553E-06	0.010708203
1181.239978	445.554927	1.00556E-05	0.011878022
1181.279978	426.624713	9.62833E-06	0.011373748
1181.319978	363.838353	8.21132E-06	0.009700202
1181.359978	339.608416	7.66449E-06	0.00905452
1181.399978	300.710035	6.78661E-06	0.008017697
1181.439978	255.635955	5.76935E-06	0.006816138
1181.479978	226.971411	5.12243E-06	0.006052047
1181.519978	215.177445	4.85626E-06	0.005737763
1181.559978	212.029562	4.78521E-06	0.005654015
1181.599978	221.613319	5.0015E-06	0.005909777
1181.639978	215.577489	4.86528E-06	0.005749014
1181.679978	230.076005	5.19249E-06	0.006135867
1181.719978	251.594167	5.67813E-06	0.00670996
1181.759978	248.996164	5.6195E-06	0.006640896
1181.799978	244.767136	5.52405E-06	0.006528326
1181.839978	226.590319	5.11383E-06	0.006043726
1181.879978	225.472611	5.0886E-06	0.006014118
1181.919978	234.240943	5.28649E-06	0.00624821
1181.959978	226.87587	5.12027E-06	0.006051957
1181.999978	222.765427	5.02751E-06	0.005942511
1182.039978	254.024341	5.73298E-06	0.006776606
1182.079978	310.091642	6.99834E-06	0.008272593
1182.119978	333.87261	7.53504E-06	0.008907321
1182.159978	354.74366	8.00607E-06	0.009464456
1182.199978	379.424843	8.56309E-06	0.010123285
1182.239978	397.996418	8.98222E-06	0.010619145

1182.279978	425.467667	9.60221E-06	0.011352504
1182.319978	444.691405	1.00361E-05	0.011865841
1182.359978	435.557494	9.82993E-06	0.011622511
1182.399978	385.873617	8.70863E-06	0.010297084
1182.439978	320.977818	7.24402E-06	0.008565622
1182.479978	270.576416	6.10653E-06	0.007220853
1182.519978	249.884508	5.63955E-06	0.006668875
1182.559978	230.335194	5.19834E-06	0.006147354
1182.599978	211.974478	4.78397E-06	0.005657521
1182.639978	201.937479	4.55745E-06	0.00538982
1182.679978	207.461153	4.68211E-06	0.005537437
1182.719978	220.876767	4.98488E-06	0.005895718
1182.759978	239.914975	5.41455E-06	0.006404109
1182.799978	232.97313	5.25788E-06	0.006219019
1182.839978	230.194001	5.19516E-06	0.006145041
1182.879978	259.757378	5.86236E-06	0.006934471
1182.919978	258.28646	5.82917E-06	0.006895436
1182.959978	241.95109	5.4605E-06	0.006459552
1182.999978	259.949879	5.86671E-06	0.006940314
1183.039978	277.031073	6.25221E-06	0.007396609
1183.079978	304.695041	6.87654E-06	0.0081355
1183.119978	347.412521	7.84062E-06	0.00927639
1183.159978	394.729436	8.90849E-06	0.010540173
1183.199978	432.427271	9.75928E-06	0.011547181
1183.239978	457.719529	1.03301E-05	0.012222978
1183.279978	453.33163	1.02311E-05	0.012106213
1183.319978	429.164286	9.68564E-06	0.011461211
1183.359978	380.478095	8.58686E-06	0.010161347
1183.399978	343.254658	7.74678E-06	0.009167539
1183.439978	299.673801	6.76322E-06	0.008003865
1183.479978	245.727281	5.54572E-06	0.006563251
1183.519978	206.865678	4.66867E-06	0.005525464
1183.559978	188.645034	4.25746E-06	0.005038954
1183.599978	195.566258	4.41366E-06	0.005224006
1183.639978	208.304087	4.70113E-06	0.005564449
1183.679978	236.340046	5.33387E-06	0.00631359
1183.719978	257.012182	5.80041E-06	0.006866058
1183.759978	265.998763	6.00322E-06	0.007106373
1183.799978	249.141998	5.62279E-06	0.006656256
1183.839978	234.667284	5.29611E-06	0.006269751
1183.879978	243.737873	5.50082E-06	0.006512316
1183.919978	241.147199	5.44236E-06	0.006443314
1183.959978	232.826002	5.25456E-06	0.006221187
1183.999978	227.318238	5.13026E-06	0.006074223
1184.039978	247.287617	5.58094E-06	0.006608053
1184.079978	303.13155	6.84126E-06	0.008100595

1184.119978	353.666009	7.98175E-06	0.009451349
1184.159978	380.461856	8.58649E-06	0.010167783
1184.199978	402.506511	9.08401E-06	0.010757286
1184.239978	399.128852	9.00778E-06	0.010667376
1184.279978	378.992394	8.55333E-06	0.010129538
1184.319978	376.265559	8.49179E-06	0.010056996
1184.359978	361.339986	8.15494E-06	0.009658385
1184.399978	327.878342	7.39976E-06	0.008764273
1184.439978	307.765231	6.94583E-06	0.008226922
1184.479978	263.107835	5.93798E-06	0.007033415
1184.519978	215.286829	4.85872E-06	0.005755255
1184.559978	188.923155	4.26373E-06	0.005050647
1184.599978	194.201789	4.38286E-06	0.00519194
1184.639978	199.506271	4.50258E-06	0.005333935
1184.679978	204.513038	4.61557E-06	0.005467979
1184.719978	220.70465	4.981E-06	0.005901086
1184.759978	236.05215	5.32737E-06	0.006311653
1184.799978	269.718212	6.08716E-06	0.007212072
1184.839978	271.623363	6.13016E-06	0.00726326
1184.879978	266.17463	6.00719E-06	0.0071178
1184.919978	266.193856	6.00762E-06	0.007118554
1184.959978	254.899198	5.75272E-06	0.006816743
1184.999978	261.803162	5.90853E-06	0.007001611
1185.039978	303.595228	6.85172E-06	0.008119564
1185.079978	363.970282	8.2143E-06	0.009734605
1185.119978	414.041441	9.34434E-06	0.011074162
1185.159978	423.919093	9.56726E-06	0.011338738
1185.199978	425.277766	9.59793E-06	0.011375463
1185.239978	449.107431	1.01357E-05	0.012013271
1185.279978	474.759326	1.07147E-05	0.012699868
1185.319978	447.99673	1.01107E-05	0.01198437
1185.359978	388.982797	8.7788E-06	0.010406038
1185.399978	348.629543	7.86808E-06	0.009326826
1185.439978	294.613166	6.64901E-06	0.007882001
1185.479978	225.762966	5.09516E-06	0.006040205
1185.519978	191.400061	4.31963E-06	0.005121011
1185.559978	190.886361	4.30804E-06	0.005107439
1185.599978	212.011592	4.78481E-06	0.005672866
1185.639978	233.511653	5.27003E-06	0.006248362
1185.679978	255.266366	5.76101E-06	0.00683071
1185.719978	249.967415	5.64142E-06	0.00668914
1185.759978	226.951097	5.12197E-06	0.006073427
1185.799978	205.995085	4.64902E-06	0.00551281
1185.839978	195.645056	4.41544E-06	0.005236001
1185.879978	198.495511	4.47977E-06	0.005312466
1185.919978	212.698174	4.8003E-06	0.005692773

1185.959978	219.428398	4.95219E-06	0.005873103
1185.999978	231.444379	5.22338E-06	0.006194925
1186.039978	249.251756	5.62526E-06	0.006671789
1186.079978	271.231522	6.12132E-06	0.007260372
1186.119978	284.402084	6.41856E-06	0.007613181
1186.159978	288.396558	6.50871E-06	0.00772037
1186.199978	323.227711	7.2948E-06	0.008653091
1186.239978	349.731324	7.89295E-06	0.009362931
1186.279978	329.639538	7.43951E-06	0.008825336
1186.319978	327.311896	7.38697E-06	0.008763314
1186.359978	344.510792	7.77513E-06	0.009224102
1186.399978	315.825405	7.12774E-06	0.00845635
1186.439978	252.895054	5.70749E-06	0.006771593
1186.479978	229.202041	5.17277E-06	0.006137389
1186.519978	210.418826	4.74886E-06	0.005634617
1186.559978	195.805702	4.41906E-06	0.005243482
1186.599978	211.57888	4.77504E-06	0.005666063
1186.639978	225.310681	5.08495E-06	0.006034003
1186.679978	233.997147	5.28099E-06	0.006266845
1186.719978	234.444042	5.29108E-06	0.006279025
1186.759978	233.225462	5.26357E-06	0.006246599
1186.799978	228.430843	5.15537E-06	0.006118388
1186.839978	208.514182	4.70587E-06	0.00558512
1186.879978	211.958746	4.78361E-06	0.005677575
1186.919978	240.040459	5.41738E-06	0.006429995
1186.959978	250.754483	5.65918E-06	0.006717219
1186.999978	260.285524	5.87428E-06	0.006972772
1187.039978	297.89428	6.72306E-06	0.00798054
1187.079978	334.931725	7.55894E-06	0.008973069
1187.119978	377.356788	8.51642E-06	0.010110009
1187.159978	423.996721	9.56902E-06	0.011359952
1187.199978	411.789045	9.29351E-06	0.011033249
1187.239978	396.02049	8.93763E-06	0.010611113
1187.279978	412.940589	9.31949E-06	0.011064849
1187.319978	397.487409	8.97074E-06	0.010651135
1187.359978	347.481809	7.84218E-06	0.009311491
1187.399978	306.346548	6.91381E-06	0.008209464
1187.439978	260.974548	5.88983E-06	0.006993822
1187.479978	224.641445	5.06984E-06	0.006020339
1187.519978	196.586925	4.43669E-06	0.005268662
1187.559978	184.481979	4.1635E-06	0.004944408
1187.599978	180.180584	4.06642E-06	0.004829286
1187.639978	207.854744	4.69099E-06	0.00557121
1187.679978	230.179262	5.19483E-06	0.00616979
1187.719978	242.928423	5.48256E-06	0.006511741
1187.759978	263.681226	5.95092E-06	0.007068262

1187.799978	253.049049	5.71096E-06	0.006783484
1187.839978	227.352582	5.13103E-06	0.006094844
1187.879978	216.803894	4.89296E-06	0.005812251
1187.919978	214.773787	4.84715E-06	0.005758021
1187.959978	220.531668	4.97709E-06	0.005912587
1187.999978	232.726159	5.25231E-06	0.006239739
1188.039978	249.316359	5.62672E-06	0.006684772
1188.079978	268.946453	6.06975E-06	0.007211345
1188.119978	299.892011	6.76814E-06	0.008041368
1188.159978	322.410329	7.27635E-06	0.00864547
1188.199978	336.562895	7.59576E-06	0.009025277
1188.239978	343.919212	7.76178E-06	0.009222854
1188.279978	352.655649	7.95895E-06	0.009457457
1188.319978	359.399912	8.11116E-06	0.009638648
1188.359978	357.16184	8.06065E-06	0.009578948
1188.399978	341.93894	7.71709E-06	0.009170984
1188.439978	311.930255	7.03983E-06	0.008366417
1188.479978	280.561921	6.33189E-06	0.007525327
1188.519978	268.424227	6.05796E-06	0.007200008
1188.559978	250.520427	5.6539E-06	0.006719996
1188.599978	219.776399	4.96005E-06	0.005895512
1188.639978	203.09441	4.58356E-06	0.0054482
1188.679978	213.367522	4.81541E-06	0.005723979
1188.719978	221.914623	5.0083E-06	0.005953471
1188.759978	213.27354	4.81329E-06	0.005721843
1188.799978	205.712825	4.64265E-06	0.005519185
1188.839978	227.792161	5.14095E-06	0.006111769
1188.879978	241.036644	5.43986E-06	0.006467342
1188.919978	243.001103	5.4842E-06	0.006520271
1188.959978	233.945428	5.27982E-06	0.006277498
1188.999978	258.29961	5.82946E-06	0.006931231
1189.039978	303.452584	6.8485E-06	0.008143143
1189.079978	330.452106	7.45784E-06	0.008867973
1189.119978	374.985723	8.46291E-06	0.01006341
1189.159978	421.309822	9.50838E-06	0.01130698
1189.199978	424.499692	9.58037E-06	0.011392972
1189.239978	417.511336	9.42265E-06	0.011205791
1189.279978	413.438468	9.33073E-06	0.011096851
1189.319978	405.140045	9.14345E-06	0.010874484
1189.359978	397.656175	8.97455E-06	0.010673966
1189.399978	362.417555	8.17926E-06	0.009728411
1189.439978	305.949039	6.90484E-06	0.008212897
1189.479978	247.981308	5.59659E-06	0.006657035
1189.519978	232.218967	5.24086E-06	0.006234106
1189.559978	219.362425	4.9507E-06	0.00588916
1189.599978	209.717986	4.73304E-06	0.005630428

1189.639978	218.013869	4.92027E-06	0.005853349
1189.679978	217.011407	4.89765E-06	0.00582663
1189.719978	250.270509	5.64826E-06	0.006719844
1189.759978	285.533796	6.4441E-06	0.007666932
1189.799978	258.319978	5.82992E-06	0.006936441
1189.839978	221.393077	4.99653E-06	0.005945075
1189.879978	232.455895	5.24621E-06	0.006242355
1189.919978	228.589455	5.15895E-06	0.006138732
1189.959978	229.365073	5.17645E-06	0.006159769
1189.999978	233.685178	5.27395E-06	0.006275999
1190.039978	263.756454	5.95262E-06	0.007083851
1190.079978	289.404042	6.53145E-06	0.007772943
1190.119978	301.436398	6.803E-06	0.008096386
1190.159978	332.049233	7.49389E-06	0.008918926
1190.199978	356.77243	8.05186E-06	0.00958332
1190.239978	383.91247	8.66437E-06	0.010312679
1190.279978	407.97036	9.20732E-06	0.010959292
1190.319978	388.341873	8.76434E-06	0.010432363
1190.359978	361.426098	8.15688E-06	0.009709628
1190.399978	319.580984	7.2125E-06	0.008585757
1190.439978	275.039868	6.20727E-06	0.007389378
1190.479978	246.070312	5.55346E-06	0.006611288
1190.519978	224.121572	5.05811E-06	0.006021783
1190.559978	217.448722	4.90751E-06	0.005842691
1190.599978	200.629906	4.52794E-06	0.005390962
1190.639978	187.136315	4.22341E-06	0.005028556
1190.679978	200.080449	4.51554E-06	0.00537656
1190.719978	231.119305	5.21604E-06	0.006210844
1190.759978	252.290002	5.69383E-06	0.00677999
1190.799978	267.200508	6.03034E-06	0.007180933
1190.839978	287.320783	6.48443E-06	0.007721918
1190.879978	284.874549	6.42922E-06	0.007656431
1190.919978	273.074709	6.16292E-06	0.00733954
1190.959978	274.725695	6.20018E-06	0.007384162
1190.999978	271.038693	6.11697E-06	0.007285306
1191.039978	287.584133	6.49037E-06	0.007730294
1191.079978	319.400034	7.20841E-06	0.008585797
1191.119978	339.205586	7.6554E-06	0.009118497
1191.159978	405.241585	9.14574E-06	0.010894037
1191.199978	501.471333	1.13175E-05	0.013481417
1191.239978	551.639659	1.24497E-05	0.014830626
1191.279978	584.509911	1.31916E-05	0.015714859
1191.319978	622.134332	1.40407E-05	0.016726973
1191.359978	612.647583	1.38266E-05	0.016472461
1191.399978	525.412716	1.18578E-05	0.014127422
1191.439978	409.71019	9.24659E-06	0.011016755

1191.479978	334.808529	7.55616E-06	0.009003016
1191.519978	287.692017	6.49281E-06	0.007736311
1191.559978	265.997203	6.00319E-06	0.007153157
1191.599978	248.852423	5.61625E-06	0.006692326
1191.639978	223.682172	5.0482E-06	0.006015631
1191.679978	224.042955	5.05634E-06	0.006025536
1191.719978	238.534261	5.38339E-06	0.006415489
1191.759978	233.603129	5.2721E-06	0.006283074
1191.799978	222.480158	5.02107E-06	0.005984108
1191.839978	224.965382	5.07716E-06	0.006051157
1191.879978	216.243535	4.88032E-06	0.00581675
1191.919978	191.445688	4.32066E-06	0.005149884
1191.959978	189.358745	4.27356E-06	0.005093916
1191.999978	208.668419	4.70936E-06	0.005613552
1192.039978	234.534364	5.29311E-06	0.006309603
1192.079978	282.895458	6.38456E-06	0.007610902
1192.119978	330.48887	7.45867E-06	0.008891634
1192.159978	392.484033	8.85782E-06	0.010559936
1192.199978	435.781972	9.83499E-06	0.011725277
1192.239978	462.168367	1.04305E-05	0.012435655
1192.279978	478.124501	1.07906E-05	0.012865421
1192.319978	444.100839	1.00227E-05	0.01195031
1192.359978	408.162091	9.21165E-06	0.010983603
1192.399978	387.902945	8.75443E-06	0.010438781
1192.439978	361.184993	8.15144E-06	0.009720106
1192.479978	306.982693	6.92817E-06	0.008261706
1192.519978	255.554338	5.76751E-06	0.006877865
1192.559978	237.420187	5.35824E-06	0.006390026
1192.599978	249.032911	5.62033E-06	0.006702801
1192.639978	255.82811	5.77368E-06	0.006885926
1192.679978	273.452648	6.17145E-06	0.007360559
1192.719978	288.064751	6.50122E-06	0.007754135
1192.759978	281.557953	6.35437E-06	0.007579239
1192.799978	248.583213	5.61018E-06	0.006691819
1192.839978	207.772503	4.68914E-06	0.005593389
1192.879978	200.259215	4.51957E-06	0.005391306
1192.919978	220.355236	4.97311E-06	0.005932523
1192.959978	231.793988	5.23127E-06	0.006240693
1192.999978	234.310256	5.28806E-06	0.006308651
1193.039978	262.506901	5.92442E-06	0.007068064
1193.079978	316.935456	7.15279E-06	0.008533853
1193.119978	377.860755	8.52779E-06	0.010174678
1193.159978	439.127118	9.91049E-06	0.011824797
1193.199978	499.434862	1.12715E-05	0.013449212
1193.239978	514.219137	1.16052E-05	0.0138478
1193.279978	481.234861	1.08608E-05	0.012959976

1193.319978	454.290058	1.02527E-05	0.012234744
1193.359978	407.629125	9.19962E-06	0.01097846
1193.399978	362.773376	8.18729E-06	0.009770711
1193.439978	312.792035	7.05928E-06	0.008424828
1193.479978	262.278119	5.91925E-06	0.007064509
1193.519978	239.963996	5.41565E-06	0.00646369
1193.559978	233.247767	5.26408E-06	0.006282992
1193.599978	212.091634	4.78661E-06	0.005713301
1193.639978	213.134395	4.81015E-06	0.005741583
1193.679978	212.330141	4.792E-06	0.005720109
1193.719978	222.056762	5.01151E-06	0.005982342
1193.759978	236.074521	5.32787E-06	0.006360202
1193.799978	233.371593	5.26687E-06	0.006287591
1193.839978	224.890781	5.07547E-06	0.006059301
1193.879978	228.931021	5.16665E-06	0.006168365
1193.919978	243.566248	5.49695E-06	0.00656292
1193.959978	267.392979	6.03469E-06	0.007205175
1193.999978	278.016426	6.27444E-06	0.007491685
1194.039978	274.25927	6.18965E-06	0.007390689
1194.079978	275.81255	6.22471E-06	0.007432796
1194.119978	301.012055	6.79342E-06	0.008112162
1194.159978	338.368697	7.63651E-06	0.009119215
1194.199978	376.767173	8.50311E-06	0.010154414
1194.239978	418.449909	9.44383E-06	0.011278201
1194.279978	431.383412	9.73572E-06	0.011627179
1194.319978	432.194926	9.75404E-06	0.011649442
1194.359978	399.84453	9.02393E-06	0.010777826
1194.399978	337.156017	7.60914E-06	0.009088359
1194.439978	291.523924	6.57929E-06	0.007858566
1194.479978	244.156891	5.51028E-06	0.00658192
1194.519978	228.20453	5.15026E-06	0.006152087
1194.559978	227.071692	5.12469E-06	0.006121752
1194.599978	213.396546	4.81606E-06	0.005753269
1194.639978	231.950852	5.23481E-06	0.00625371
1194.679978	263.907559	5.95603E-06	0.007115545
1194.719978	266.118249	6.00592E-06	0.00717539
1194.759978	257.351205	5.80806E-06	0.006939236
1194.799978	290.096917	6.54708E-06	0.007822455
1194.839978	306.252379	6.91169E-06	0.008258363
1194.879978	278.637136	6.28845E-06	0.007513945
1194.919978	246.084752	5.55379E-06	0.006636335
1194.959978	253.804098	5.728E-06	0.006844737
1194.999978	272.157363	6.14221E-06	0.007339944
1195.039978	303.278197	6.84457E-06	0.008179531
1195.079978	353.876827	7.98651E-06	0.009544515
1195.119978	405.928667	9.16124E-06	0.010948786

1195.159978	439.19723	9.91207E-06	0.011846509
1195.199978	469.560429	1.05973E-05	0.012665922
1195.239978	481.404787	1.08646E-05	0.012985847
1195.279978	462.706243	1.04426E-05	0.012481873
1195.319978	418.770511	9.45107E-06	0.011297049
1195.359978	377.216957	8.51326E-06	0.010176412
1195.399978	338.66395	7.64317E-06	0.00913665
1195.439978	297.072537	6.70451E-06	0.008014843
1195.479978	247.179797	5.5785E-06	0.00666899
1195.519978	222.01558	5.01058E-06	0.005990251
1195.559978	220.523617	4.97691E-06	0.005950195
1195.599978	207.597533	4.68519E-06	0.00560161
1195.639978	218.006663	4.92011E-06	0.005882676
1195.679978	253.977555	5.73192E-06	0.006853542
1195.719978	272.258901	6.1445E-06	0.007347106
1195.759978	271.33251	6.1236E-06	0.007322352
1195.799978	249.789675	5.63741E-06	0.006741209
1195.839978	232.544559	5.24821E-06	0.006276015
1195.879978	246.721113	5.56815E-06	0.006658841
1195.919978	238.225075	5.37641E-06	0.006429754
1195.959978	231.359698	5.22147E-06	0.006244665
1195.999978	248.994512	5.61946E-06	0.006720873
1196.039978	272.051679	6.13983E-06	0.007343479
1196.079978	310.948716	7.01768E-06	0.008393706
1196.119978	347.324861	7.83864E-06	0.009375952
1196.159978	407.31413	9.19251E-06	0.010995716
1196.199978	436.724077	9.85625E-06	0.011790051
1196.239978	437.288309	9.86899E-06	0.011805678
1196.279978	420.763224	9.49604E-06	0.011359922
1196.319978	395.320262	8.92183E-06	0.010673361
1196.359978	367.732087	8.2992E-06	0.009928832
1196.399978	356.734585	8.051E-06	0.009632219
1196.439978	333.851163	7.53456E-06	0.009014644
1196.479978	287.26353	6.48314E-06	0.007756945
1196.519978	263.809659	5.95382E-06	0.00712386
1196.559978	241.168459	5.44284E-06	0.00651268
1196.599978	220.217044	4.96999E-06	0.005947092
1196.639978	230.159956	5.19439E-06	0.006215814
1196.679978	241.367624	5.44733E-06	0.006518712
1196.719978	260.189137	5.87211E-06	0.007027267
1196.759978	277.781546	6.26914E-06	0.007502659
1196.799978	303.173798	6.84221E-06	0.008188757
1196.839978	300.918806	6.79132E-06	0.008128121
1196.879978	284.680777	6.42485E-06	0.007689773
1196.919978	242.674187	5.47682E-06	0.006555313
1196.959978	228.323009	5.15293E-06	0.006167854

1196.999978	269.929594	6.09193E-06	0.007292046
1197.039978	311.697463	7.03458E-06	0.008420671
1197.079978	354.042177	7.99024E-06	0.009564955
1197.119978	411.135344	9.27875E-06	0.011107779
1197.159978	422.49982	9.53523E-06	0.011415199
1197.199978	420.798877	9.49684E-06	0.011369622
1197.239978	409.119868	9.23327E-06	0.011054435
1197.279978	394.934954	8.91313E-06	0.010671514
1197.319978	378.134583	8.53397E-06	0.010217894
1197.359978	353.742608	7.98348E-06	0.009559097
1197.399978	317.001782	7.15429E-06	0.008566545
1197.439978	291.372068	6.57586E-06	0.0078742
1197.479978	254.113351	5.73498E-06	0.006867529
1197.519978	213.589374	4.82041E-06	0.005772543
1197.559978	203.857568	4.60078E-06	0.005509712
1197.599978	213.923339	4.82795E-06	0.005781955
1197.639978	219.671634	4.95768E-06	0.005937519
1197.679978	224.201817	5.05992E-06	0.006060168
1197.719978	233.706747	5.27444E-06	0.006317297
1197.759978	220.895965	4.98531E-06	0.00597121
1197.799978	206.873164	4.66884E-06	0.005592335
1197.839978	224.752551	5.07235E-06	0.006075866
1197.879978	243.68618	5.49966E-06	0.00658793
1197.919978	231.68684	5.22885E-06	0.006263743
1197.959978	223.56863	5.04563E-06	0.006044466
1197.999978	238.883289	5.39126E-06	0.006458733
1198.039978	246.885897	5.57187E-06	0.006675324
1198.079978	257.67532	5.81537E-06	0.006967282
1198.119978	281.096135	6.34395E-06	0.007600811
1198.159978	331.340941	7.4779E-06	0.008959725
1198.199978	393.177328	8.87346E-06	0.010632185
1198.239978	408.969096	9.22986E-06	0.011059591
1198.279978	403.210258	9.09989E-06	0.010904221
1198.319978	395.188179	8.91885E-06	0.010687632
1198.359978	348.184135	7.85803E-06	0.00941675
1198.399978	276.707454	6.2449E-06	0.00748389
1198.439978	244.089476	5.50876E-06	0.006601918
1198.479978	247.069076	5.576E-06	0.00668273
1198.519978	260.899812	5.88815E-06	0.00705706
1198.559978	248.138407	5.60014E-06	0.006712102
1198.599978	215.206038	4.8569E-06	0.005821481
1198.639978	214.055025	4.83092E-06	0.005790538
1198.679978	235.74027	5.32033E-06	0.006377372
1198.719978	242.784517	5.47931E-06	0.006568156
1198.759978	218.751782	4.93692E-06	0.005918186
1198.799978	214.192106	4.83402E-06	0.00579502

1198.839978	244.179324	5.51079E-06	0.006606552
1198.879978	247.248049	5.58004E-06	0.006689803
1198.919978	237.331617	5.35624E-06	0.006421708
1198.959978	249.444655	5.62962E-06	0.006749687
1198.999978	253.679964	5.7252E-06	0.006864519
1199.039978	289.095505	6.52448E-06	0.007823116
1199.079978	313.249671	7.06961E-06	0.008477026
1199.119978	323.57112	7.30255E-06	0.008756633
1199.159978	328.686698	7.418E-06	0.00889537
1199.199978	368.578819	8.31831E-06	0.009975318
1199.239978	369.547997	8.34018E-06	0.010001882
1199.279978	360.841688	8.14369E-06	0.00976657
1199.319978	358.454212	8.08981E-06	0.009702273
1199.359978	347.720579	7.84757E-06	0.00941206
1199.399978	308.980263	6.97325E-06	0.008363721
1199.439978	262.826769	5.93163E-06	0.007114639
1199.479978	234.26105	5.28695E-06	0.006341585
1199.519978	207.512134	4.68326E-06	0.005617664
1199.559978	187.415303	4.2297E-06	0.005073782
1199.599978	189.161502	4.26911E-06	0.005121226
1199.639978	202.529404	4.57081E-06	0.005483322
1199.679978	205.027786	4.62719E-06	0.005551149
1199.719978	221.898063	5.00793E-06	0.006008114
1199.759978	232.594243	5.24933E-06	0.006297934
1199.799978	246.395465	5.5608E-06	0.006671851
1199.839978	248.131702	5.59999E-06	0.006719088
1199.879978	225.287183	5.08442E-06	0.006100691
1199.919978	193.066469	4.35724E-06	0.005228341
1199.959978	198.628441	4.48277E-06	0.005379141
1199.999978	215.480711	4.8631E-06	0.005835719
1200.039978	229.874327	5.18794E-06	0.006225739
1200.079978	283.16805	6.39071E-06	0.007669361
1200.119978	335.149036	7.56385E-06	0.009077524
1200.159978	353.426529	7.97634E-06	0.009572889
1200.199978	369.580983	8.34093E-06	0.010010782
1200.239978	383.616623	8.65769E-06	0.010391309
1200.279978	355.87636	8.03163E-06	0.009640209
1200.319978	317.293611	7.16087E-06	0.008595341
1200.359978	277.194138	6.25589E-06	0.007509315
1200.399978	257.709016	5.81613E-06	0.006981687
1200.439978	257.558198	5.81273E-06	0.006977833
1200.479978	233.250376	5.26414E-06	0.00631949
1200.519978	205.548333	4.63894E-06	0.00556914
1200.559978	201.261703	4.5422E-06	0.005453179
1200.599978	191.504923	4.322E-06	0.005188992
1200.639978	209.79946	4.73488E-06	0.005684888

1200.679978	232.935809	5.25704E-06	0.006312019
1200.719978	240.217388	5.42137E-06	0.006509549
1200.759978	276.676532	6.2442E-06	0.00749779
1200.799978	296.848151	6.69945E-06	0.008044699
1200.839978	277.792921	6.2694E-06	0.007528545
1200.879978	237.110701	5.35126E-06	0.006426219
1200.919978	215.914396	4.87289E-06	0.005851948
1200.959978	215.04009	4.85316E-06	0.005828445
1200.999978	237.826982	5.36742E-06	0.006446276
1201.039978	274.09871	6.18603E-06	0.007429665
1201.079978	304.836212	6.87973E-06	0.008263104
1201.119978	327.217597	7.38485E-06	0.008870085
1201.159978	347.05517	7.83255E-06	0.009408148
1201.199978	383.226752	8.64889E-06	0.010389051
1201.239978	431.535125	9.73915E-06	0.011699052
1201.279978	437.663654	9.87746E-06	0.011865594
1201.319978	427.553434	9.64929E-06	0.011591879
1201.359978	391.217677	8.82924E-06	0.010607093
1201.399978	336.966408	7.60486E-06	0.009136482
1201.439978	294.980909	6.65731E-06	0.007998356
1201.479978	269.376969	6.07946E-06	0.007304353
1201.519978	259.469278	5.85586E-06	0.007035933
1201.559978	249.677442	5.63487E-06	0.006770637
1201.599978	240.377745	5.42499E-06	0.006518669
1201.639978	230.61749	5.20472E-06	0.006254194
1201.679978	228.842404	5.16465E-06	0.006206262
1201.719978	222.49097	5.02131E-06	0.00603421
1201.759978	209.651939	4.73155E-06	0.00568619
1201.799978	192.997978	4.3557E-06	0.005234675
1201.839978	188.546432	4.25523E-06	0.005114106
1201.879978	196.133384	4.42646E-06	0.00532007
1201.919978	201.176572	4.54028E-06	0.005457047
1201.959978	203.244058	4.58694E-06	0.005513313
1201.999978	214.851718	4.8489E-06	0.005828383
1202.039978	213.766597	4.82441E-06	0.005799139
1202.079978	221.957635	5.00927E-06	0.006021549
1202.119978	239.060376	5.39526E-06	0.006485749
1202.159978	278.959574	6.29573E-06	0.007568473
1202.199978	349.246073	7.882E-06	0.009475737
1202.239978	405.529419	9.15223E-06	0.011003182
1202.279978	415.329053	9.3734E-06	0.011269449
1202.319978	382.658075	8.63606E-06	0.010383307
1202.359978	317.155077	7.15775E-06	0.00860619
1202.399978	277.820012	6.27001E-06	0.007539061
1202.439978	258.881403	5.84259E-06	0.007025367
1202.479978	243.230058	5.48936E-06	0.00660085

1202.519978	242.677165	5.47689E-06	0.006586064
1202.559978	237.451385	5.35895E-06	0.006444455
1202.599978	208.107995	4.69671E-06	0.00564826
1202.639978	194.909827	4.39884E-06	0.005290225
1202.679978	203.170456	4.58527E-06	0.005514617
1202.719978	232.073608	5.23758E-06	0.00629934
1202.759978	254.438455	5.74232E-06	0.006906635
1202.799978	232.567169	5.24872E-06	0.006313157
1202.839978	226.501119	5.11181E-06	0.006148695
1202.879978	233.657096	5.27332E-06	0.006343165
1202.919978	248.843895	5.61606E-06	0.006755671
1202.959978	264.087888	5.9601E-06	0.007169757
1202.999978	285.708394	6.44804E-06	0.007756993
1203.039978	304.929528	6.88183E-06	0.008279122
1203.079978	318.819034	7.1953E-06	0.008656523
1203.119978	343.166787	7.7448E-06	0.009317919
1203.159978	351.879001	7.94142E-06	0.009554797
1203.199978	362.859433	8.18923E-06	0.009853284
1203.239978	388.792613	8.77451E-06	0.010557838
1203.279978	389.178829	8.78322E-06	0.010568678
1203.319978	369.636058	8.34217E-06	0.010038301
1203.359978	357.54723	8.06934E-06	0.009710324
1203.399978	313.745598	7.0808E-06	0.008521036
1203.439978	254.570617	5.7453E-06	0.006914129
1203.479978	217.35862	4.90548E-06	0.005903649
1203.519978	228.036455	5.14647E-06	0.006193874
1203.559978	230.944805	5.2121E-06	0.006273078
1203.599978	218.316585	4.9271E-06	0.005930259
1203.639978	211.147619	4.76531E-06	0.005735715
1203.679978	228.471834	5.15629E-06	0.006206524
1203.719978	248.340405	5.6047E-06	0.006746486
1203.759978	251.406549	5.6739E-06	0.006830008
1203.799978	236.279431	5.3325E-06	0.006419261
1203.839978	239.793702	5.41181E-06	0.006514953
1203.879978	245.406074	5.53847E-06	0.006667657
1203.919978	236.443795	5.33621E-06	0.006424366
1203.959978	224.386439	5.06409E-06	0.006096961
1203.999978	235.661007	5.31854E-06	0.006403523
1204.039978	261.872583	5.9101E-06	0.007115996
1204.079978	285.304438	6.43892E-06	0.007752979
1204.119978	320.882345	7.24187E-06	0.008720077
1204.159978	361.549042	8.15966E-06	0.009825534
1204.199978	360.337339	8.13231E-06	0.00979293
1204.239978	330.221015	7.45263E-06	0.008974753
1204.279978	302.402372	6.8248E-06	0.00821897
1204.319978	292.406964	6.59922E-06	0.00794757

1204.359978	295.825172	6.67636E-06	0.008040743
1204.399978	298.932465	6.74649E-06	0.008125472
1204.439978	277.489434	6.26255E-06	0.007542866
1204.479978	259.861297	5.86471E-06	0.007063923
1204.519978	234.786707	5.29881E-06	0.006382521
1204.559978	223.731266	5.0493E-06	0.006082188
1204.599978	215.429883	4.86195E-06	0.005856708
1204.639978	203.196534	4.58586E-06	0.005524314
1204.679978	211.937541	4.78314E-06	0.005762147
1204.719978	241.777268	5.45658E-06	0.006573646
1204.759978	242.517214	5.47328E-06	0.006593984
1204.799978	237.039377	5.34965E-06	0.006445256
1204.839978	227.139736	5.12623E-06	0.006176284
1204.879978	208.902466	4.71464E-06	0.005680573
1204.919978	198.72854	4.48503E-06	0.005404098
1204.959978	209.289732	4.72338E-06	0.005691481
1204.999978	247.925325	5.59533E-06	0.006742372
1205.039978	258.363087	5.8309E-06	0.007026462
1205.079978	272.273873	6.14484E-06	0.007405026
1205.119978	313.699545	7.07976E-06	0.008531962
1205.159978	377.283851	8.51477E-06	0.010261661
1205.199978	417.762188	9.42831E-06	0.011363
1205.239978	445.004755	1.00431E-05	0.012104391
1205.279978	434.807489	9.813E-06	0.011827412
1205.319978	409.862131	9.25002E-06	0.011149231
1205.359978	363.94694	8.21378E-06	0.009900556
1205.399978	325.808931	7.35305E-06	0.008863371
1205.439978	312.618039	7.05535E-06	0.008504805
1205.479978	273.597785	6.17472E-06	0.007443502
1205.519978	226.051646	5.10167E-06	0.006150166
1205.559978	198.690831	4.48418E-06	0.005405942
1205.599978	183.444215	4.14008E-06	0.004991281
1205.639978	193.585201	4.36895E-06	0.005267379
1205.679978	224.394739	5.06428E-06	0.006105897
1205.719978	248.936537	5.61815E-06	0.006773917
1205.759978	265.869805	6.00031E-06	0.007234935
1205.799978	262.98206	5.93514E-06	0.00715659
1205.839978	242.733871	5.47817E-06	0.006605791
1205.879978	225.158351	5.08151E-06	0.006127692
1205.919978	221.126564	4.99052E-06	0.006018166
1205.959978	227.491791	5.13417E-06	0.006191607
1205.999978	239.771693	5.41131E-06	0.006526044
1206.039978	256.298364	5.7843E-06	0.006976094
1206.079978	267.555196	6.03835E-06	0.007282731
1206.119978	276.701188	6.24476E-06	0.00753193
1206.159978	308.646403	6.96572E-06	0.008401772

1206.199978	321.639901	7.25896E-06	0.008755763
1206.239978	341.707298	7.71186E-06	0.009302351
1206.279978	344.474422	7.77431E-06	0.009377992
1206.319978	344.583269	7.77676E-06	0.009381266
1206.359978	333.237958	7.52072E-06	0.009072691
1206.399978	298.605125	6.7391E-06	0.008130052
1206.439978	292.310277	6.59704E-06	0.007958928
1206.479978	271.96437	6.13786E-06	0.007405202
1206.519978	249.537116	5.63171E-06	0.006794765
1206.559978	232.392879	5.24478E-06	0.006328146
1206.599978	224.885303	5.07535E-06	0.006123915
1206.639977	221.264722	4.99364E-06	0.006025522
1206.679977	229.289649	5.17475E-06	0.006244265
1206.719977	260.345797	5.87564E-06	0.007090255
1206.759977	272.94995	6.1601E-06	0.007433762
1206.799977	249.877991	5.6394E-06	0.006805626
1206.839977	241.552443	5.4515E-06	0.006579091
1206.879977	235.882962	5.32355E-06	0.006424886
1206.919977	259.845931	5.86436E-06	0.007077814
1206.959977	254.189306	5.7367E-06	0.006923966
1206.999977	250.865698	5.66169E-06	0.006833659
1207.039977	294.263089	6.64111E-06	0.008016083
1207.079977	349.598456	7.88995E-06	0.009523801
1207.119977	367.587799	8.29594E-06	0.0100142
1207.159977	391.669617	8.83944E-06	0.010670615
1207.199977	438.572464	9.89797E-06	0.011948829
1207.239977	457.65133	1.03286E-05	0.012469042
1207.279977	426.681341	9.6296E-06	0.011625628
1207.319977	386.690495	8.72707E-06	0.010536361
1207.359977	358.298352	8.08629E-06	0.009763069
1207.399977	324.04053	7.31314E-06	0.008829889
1207.439977	273.96389	6.18298E-06	0.007465581
1207.479977	224.562104	5.06805E-06	0.006119574
1207.519977	197.473843	4.45671E-06	0.005381566
1207.559977	193.381584	4.36435E-06	0.005270218
1207.599977	207.349376	4.67959E-06	0.005651069
1207.639977	221.156303	4.99119E-06	0.00602756
1207.679977	223.773102	5.05025E-06	0.006099082
1207.719977	239.771846	5.41132E-06	0.006535355
1207.759977	251.026431	5.66532E-06	0.006842343
1207.799977	240.615344	5.43035E-06	0.00655878
1207.839977	236.541255	5.33841E-06	0.006447941
1207.879977	237.526694	5.36065E-06	0.006475018
1207.919977	223.101724	5.0351E-06	0.006081992
1207.959977	228.738734	5.16231E-06	0.006235869
1207.999977	259.592429	5.85864E-06	0.007077236

1208.039977	265.658335	5.99554E-06	0.00724285
1208.079977	257.759371	5.81727E-06	0.007027727
1208.119977	279.091478	6.29871E-06	0.007609592
1208.159977	316.089408	7.1337E-06	0.008618648
1208.199977	342.135122	7.72151E-06	0.009329132
1208.239977	341.377857	7.70442E-06	0.009308792
1208.279977	346.774631	7.82622E-06	0.009456265
1208.319977	354.425033	7.99888E-06	0.009665206
1208.359977	340.127742	7.67621E-06	0.009275624
1208.399977	294.42735	6.64482E-06	0.008029595
1208.439977	259.208178	5.84997E-06	0.007069335
1208.479977	254.673566	5.74763E-06	0.006945893
1208.519977	225.221408	5.08293E-06	0.006142827
1208.559977	216.86815	4.89441E-06	0.005915191
1208.599977	213.968366	4.82897E-06	0.005836291
1208.639977	216.346244	4.88263E-06	0.005901346
1208.679977	242.773663	5.47906E-06	0.006622434
1208.719977	264.929723	5.97909E-06	0.007227051
1208.759977	268.745738	6.06522E-06	0.007331392
1208.799977	259.014724	5.8456E-06	0.007066163
1208.839977	249.851452	5.6388E-06	0.006816406
1208.879977	258.791544	5.84056E-06	0.007060542
1208.919977	253.991874	5.73224E-06	0.006929823
1208.959977	246.547633	5.56424E-06	0.006726939
1208.999977	255.233779	5.76027E-06	0.006964167
1209.039977	286.441881	6.46459E-06	0.007815953
1209.079977	314.261676	7.09245E-06	0.008575337
1209.119977	342.396863	7.72742E-06	0.009343378
1209.159977	398.565219	8.99506E-06	0.010876469
1209.199977	456.135409	1.02943E-05	0.012447917
1209.239977	466.697455	1.05327E-05	0.012736576
1209.279977	454.577819	1.02592E-05	0.012406231
1209.319977	422.282836	9.53034E-06	0.011525225
1209.359977	374.5857	8.45388E-06	0.010223781
1209.399977	326.130185	7.3603E-06	0.008901552
1209.439977	277.863669	6.271E-06	0.007584393
1209.479977	258.271324	5.82882E-06	0.007049846
1209.519977	240.564388	5.4292E-06	0.00656673
1209.559977	224.381154	5.06397E-06	0.006125176
1209.599977	223.347682	5.04065E-06	0.006097165
1209.639977	243.764083	5.50142E-06	0.006654733
1209.679977	234.747932	5.29793E-06	0.006408804
1209.719977	233.467812	5.26904E-06	0.006374067
1209.759977	252.113154	5.68984E-06	0.006883344
1209.799977	254.736594	5.74905E-06	0.006955201
1209.839977	231.873435	5.23306E-06	0.006331166

1209.879977	230.009114	5.19099E-06	0.006280469
1209.919977	236.466975	5.33673E-06	0.006457017
1209.959977	231.156078	5.21687E-06	0.006312205
1209.999977	228.839203	5.16458E-06	0.006249144
1210.039977	256.060832	5.77894E-06	0.006992744
1210.079977	287.314866	6.4843E-06	0.007846517
1210.119977	324.920981	7.33301E-06	0.008873827
1210.159977	343.234069	7.74631E-06	0.00937428
1210.199977	367.074391	8.28436E-06	0.01002573
1210.239977	383.13326	8.64678E-06	0.010464684
1210.279977	373.407156	8.42728E-06	0.010199368
1210.319977	376.085021	8.48772E-06	0.010272851
1210.359977	375.573876	8.47618E-06	0.010259228
1210.399977	327.79767	7.39794E-06	0.008954463
1210.439977	272.810086	6.15694E-06	0.007452611
1210.479977	218.458448	4.9303E-06	0.005968033
1210.519977	211.019133	4.76241E-06	0.00576499
1210.559977	229.200241	5.17273E-06	0.0062619
1210.599977	223.532258	5.04481E-06	0.006107249
1210.639977	236.996203	5.34867E-06	0.006475319
1210.679977	255.684626	5.77045E-06	0.006986163
1210.719977	252.60528	5.70095E-06	0.006902253
1210.759977	255.153481	5.75846E-06	0.006972111
1210.799977	271.541954	6.12832E-06	0.007420174
1210.839977	266.092626	6.00534E-06	0.007271506
1210.879977	251.623316	5.67879E-06	0.00687633
1210.919977	250.518475	5.65385E-06	0.006846364
1210.959977	249.936784	5.64073E-06	0.006830692
1210.999977	261.308322	5.89736E-06	0.007141709
1211.039977	267.676088	6.04108E-06	0.007315985
1211.079977	272.682409	6.15406E-06	0.007453061
1211.119977	317.584758	7.16745E-06	0.008680637
1211.159977	379.444856	8.56354E-06	0.010371819
1211.199977	419.949665	9.47768E-06	0.011479365
1211.239977	455.857133	1.02881E-05	0.01246131
1211.279977	442.727181	9.99174E-06	0.01210279
1211.319977	386.454384	8.72174E-06	0.010564814
1211.359977	335.95133	7.58195E-06	0.009184475
1211.399977	299.031067	6.74871E-06	0.008175393
1211.439977	263.020529	5.93601E-06	0.007191116
1211.479977	241.042275	5.43999E-06	0.006590437
1211.519977	224.443565	5.06538E-06	0.006136807
1211.559977	193.988863	4.37806E-06	0.00530428
1211.599977	178.299991	4.02398E-06	0.004875457
1211.639977	192.613089	4.34701E-06	0.00526701
1211.679977	215.832661	4.87104E-06	0.005902145

1211.719977	215.623243	4.86632E-06	0.005896613
1211.759977	219.841559	4.96152E-06	0.006012169
1211.799977	218.030077	4.92064E-06	0.005962826
1211.839977	199.789031	4.50896E-06	0.005464138
1211.879977	185.855637	4.1945E-06	0.005083234
1211.919977	173.625009	3.91847E-06	0.004748878
1211.959977	170.950327	3.85811E-06	0.004675876
1211.999977	179.856259	4.05911E-06	0.004919635
1212.039977	228.854568	5.16493E-06	0.0062601
1212.079977	271.084802	6.11801E-06	0.007415513
1212.119977	311.661607	7.03377E-06	0.008525771
1212.159977	347.970976	7.85322E-06	0.009519359
1212.199977	370.642321	8.36488E-06	0.010139908
1212.239977	397.473395	8.97042E-06	0.010874303
1212.279977	396.19437	8.94156E-06	0.010839668
1212.319977	361.008365	8.14746E-06	0.009877324
1212.359977	320.01384	7.22227E-06	0.008755987
1212.399977	271.675108	6.13133E-06	0.007433623
1212.439977	241.792906	5.45693E-06	0.006616199
1212.479977	243.238373	5.48955E-06	0.006655971
1212.519977	228.586639	5.15888E-06	0.006255248
1212.559977	211.204431	4.76659E-06	0.005779776
1212.599977	214.919337	4.85043E-06	0.005881631
1212.639977	220.961945	4.9868E-06	0.006047197
1212.679977	231.578515	5.2264E-06	0.006337956
1212.719977	259.007366	5.84544E-06	0.007088876
1212.759977	270.263417	6.09947E-06	0.007397192
1212.799977	275.285354	6.21281E-06	0.007534892
1212.839977	258.022988	5.82322E-06	0.007062633
1212.879977	232.109623	5.23839E-06	0.006353539
1212.919977	227.714722	5.1392E-06	0.006233443
1212.959977	239.895382	5.4141E-06	0.006567092
1212.999977	276.677871	6.24423E-06	0.007574256
1213.039977	313.064684	7.06543E-06	0.008570654
1213.079977	352.346319	7.95197E-06	0.00964637
1213.119977	399.274823	9.01108E-06	0.010931517
1213.159977	458.941664	1.03577E-05	0.012565516
1213.199977	505.37962	1.14057E-05	0.013837412
1213.239977	518.151357	1.1694E-05	0.014187573
1213.279977	495.08243	1.11733E-05	0.013556366
1213.319977	436.839766	9.85887E-06	0.011961958
1213.359977	414.637425	9.35779E-06	0.011354367
1213.399977	392.280773	8.85323E-06	0.01074251
1213.439977	339.314414	7.65785E-06	0.009292346
1213.479977	292.716015	6.60619E-06	0.008016483
1213.519977	275.000116	6.20637E-06	0.007531553

1213.559977	247.390721	5.58326E-06	0.006775626
1213.599977	233.351796	5.26643E-06	0.006391333
1213.639977	249.055013	5.62082E-06	0.006821658
1213.679977	255.517369	5.76667E-06	0.006998893
1213.719977	234.089585	5.28308E-06	0.006412175
1213.759977	215.392387	4.86111E-06	0.005900216
1213.799977	216.542537	4.88706E-06	0.005931917
1213.839977	229.865077	5.18773E-06	0.00629708
1213.879977	248.008749	5.59721E-06	0.006794344
1213.919977	249.489598	5.63063E-06	0.006835138
1213.959977	240.996384	5.43895E-06	0.006602671
1213.999977	224.205683	5.06001E-06	0.006142852
1214.039977	243.731174	5.50067E-06	0.006678037
1214.079977	284.947778	6.43087E-06	0.007807596
1214.119977	306.335496	6.91357E-06	0.008393898
1214.159977	349.575711	7.88944E-06	0.009579038
1214.199977	422.288529	9.53046E-06	0.011571889
1214.239977	441.465869	9.96327E-06	0.0120978
1214.279977	411.399218	9.28471E-06	0.011274234
1214.319977	377.482698	8.51926E-06	0.010345106
1214.359977	351.26559	7.92758E-06	0.00962693
1214.399977	311.514296	7.03044E-06	0.008537771
1214.439977	263.456034	5.94584E-06	0.007220861
1214.479977	222.523819	5.02205E-06	0.006099182
1214.519977	222.120268	5.01295E-06	0.006088322
1214.559977	225.366109	5.0862E-06	0.006177494
1214.599977	224.550491	5.06779E-06	0.00615534
1214.639977	218.626418	4.93409E-06	0.005993147
1214.679977	201.43266	4.54605E-06	0.005522001
1214.719977	202.80941	4.57713E-06	0.005559926
1214.759977	212.92589	4.80544E-06	0.005837457
1214.799977	211.221259	4.76697E-06	0.005790915
1214.839977	200.7394	4.53041E-06	0.005503722
1214.879977	198.351045	4.47651E-06	0.005438418
1214.919977	201.611068	4.55008E-06	0.005527984
1214.959977	218.340111	4.92763E-06	0.005986876
1214.999977	232.83903	5.25485E-06	0.006384646
1215.039977	266.251229	6.00892E-06	0.007301077
1215.079977	312.294219	7.04805E-06	0.008563939
1215.119977	348.535125	7.86595E-06	0.009558076
1215.159977	394.569522	8.90488E-06	0.010820859
1215.199977	438.387561	9.8938E-06	0.012022941
1215.239977	448.691085	1.01263E-05	0.012305924
1215.279977	441.152138	9.95619E-06	0.012099557
1215.319977	410.869616	9.27275E-06	0.011269364
1215.359977	363.17237	8.19629E-06	0.009961448

1215.399977	306.276233	6.91223E-06	0.008401122
1215.439977	251.790986	5.68257E-06	0.006906825
1215.479977	219.315002	4.94963E-06	0.006016181
1215.519977	211.719234	4.77821E-06	0.005808008
1215.559977	209.795179	4.73478E-06	0.005755415
1215.599977	220.882368	4.98501E-06	0.006059775
1215.639977	211.915227	4.78263E-06	0.005813958
1215.679977	231.005394	5.21347E-06	0.006337911
1215.719977	242.438966	5.47151E-06	0.006651824
1215.759977	211.992463	4.78437E-06	0.005816651
1215.799977	217.782407	4.91505E-06	0.005975712
1215.839977	231.753928	5.23036E-06	0.006359285
1215.879977	228.162338	5.14931E-06	0.006260938
1215.919977	230.555311	5.20331E-06	0.006326811
1215.959977	224.756211	5.07243E-06	0.006167877
1215.999977	237.472242	5.35942E-06	0.006517052
1216.039977	255.585837	5.76822E-06	0.007014382
1216.079977	278.66565	6.2891E-06	0.007648043
1216.119977	313.106021	7.06637E-06	0.00859355
1216.159977	359.930424	8.12313E-06	0.009879024
1216.199977	390.756537	8.81883E-06	0.010725462
1216.239977	384.846849	8.68546E-06	0.0105636
1216.279977	365.592609	8.25092E-06	0.010035424
1216.319977	376.170428	8.48964E-06	0.010326122
1216.359977	388.221914	8.76163E-06	0.010657293
1216.399977	337.015061	7.60596E-06	0.00925189
1216.439977	272.628485	6.15285E-06	0.007484567
1216.479977	241.50121	5.45035E-06	0.006630237
1216.519977	262.035973	5.91379E-06	0.00719424
1216.559977	263.420201	5.94503E-06	0.007232482
1216.599977	228.851139	5.16485E-06	0.006283558
1216.639977	187.885785	4.24032E-06	0.005158943
1216.679977	200.262503	4.51965E-06	0.005498962
1216.719977	246.993341	5.5743E-06	0.006782357
1216.759977	279.778542	6.31421E-06	0.00768288
1216.799977	279.730223	6.31312E-06	0.007681806
1216.839977	243.498507	5.49542E-06	0.006687049
1216.879977	223.533902	5.04485E-06	0.006138976
1216.919977	231.570067	5.22621E-06	0.006359884
1216.959977	212.908131	4.80504E-06	0.005847541
1216.999977	196.438292	4.43334E-06	0.005395373
1217.039977	228.347263	5.15348E-06	0.006271991
1217.079977	277.990735	6.27386E-06	0.007635794
1217.119977	309.183961	6.97785E-06	0.008492882
1217.159977	346.721095	7.82501E-06	0.009524292
1217.199977	369.120772	8.33054E-06	0.010139935

1217.239977	413.543228	9.33309E-06	0.011360616
1217.279977	450.329872	1.01633E-05	0.012371603
1217.319977	425.103932	9.594E-06	0.011678972
1217.359977	370.285547	8.35683E-06	0.010173269
1217.399977	317.33547	7.16182E-06	0.008718799
1217.439977	282.764672	6.3816E-06	0.007769221
1217.479977	250.720814	5.65842E-06	0.006889012
1217.519977	214.370775	4.83805E-06	0.005890422
1217.559977	184.06108	4.154E-06	0.005057747
1217.599977	184.503626	4.16399E-06	0.005070074
1217.639977	217.439833	4.90731E-06	0.005975342
1217.679977	227.84671	5.14218E-06	0.006261533
1217.719977	211.624553	4.77607E-06	0.005815918
1217.759977	212.906984	4.80501E-06	0.005851354
1217.799977	205.882228	4.64648E-06	0.005658477
1217.839977	201.971209	4.55821E-06	0.005551169
1217.879977	208.947384	4.71565E-06	0.005743097
1217.919977	223.956505	5.05439E-06	0.006155838
1217.959977	213.219246	4.81206E-06	0.005860898
1217.999977	195.03649	4.4017E-06	0.005361273
1218.039977	191.908851	4.33112E-06	0.005275472
1218.079977	225.799266	5.09597E-06	0.006207305
1218.119977	263.009814	5.93577E-06	0.007230474
1218.159977	291.921171	6.58825E-06	0.008025548
1218.199977	328.419044	7.41196E-06	0.00902925
1218.239977	372.484636	8.40646E-06	0.010241085
1218.279977	383.087828	8.64576E-06	0.010532955
1218.319977	366.105835	8.2625E-06	0.010066367
1218.359977	318.811723	7.19514E-06	0.008766266
1218.399977	272.274737	6.14486E-06	0.007486899
1218.439977	246.317416	5.55904E-06	0.006773358
1218.479977	220.958669	4.98673E-06	0.00607623
1218.519977	201.70571	4.55222E-06	0.005546967
1218.559977	178.325554	4.02456E-06	0.004904167
1218.599977	168.229936	3.79672E-06	0.004626677
1218.639977	173.554786	3.91689E-06	0.004773278
1218.679977	197.967509	4.46785E-06	0.00544488
1218.719977	215.817193	4.87069E-06	0.005936011
1218.759977	235.711042	5.31967E-06	0.006483401
1218.799977	241.365688	5.44729E-06	0.006639154
1218.839977	225.551228	5.09038E-06	0.006204355
1218.879977	219.592162	4.95589E-06	0.006040634
1218.919977	221.034381	4.98844E-06	0.006080507
1218.959977	234.220633	5.28603E-06	0.006443463
1218.999977	243.322615	5.49145E-06	0.00669408
1219.039977	277.468903	6.26209E-06	0.007633734

1219.079977	316.617091	7.14561E-06	0.008711066
1219.119977	351.603863	7.93521E-06	0.009673972
1219.159977	380.043384	8.57705E-06	0.010456796
1219.199977	384.665107	8.68136E-06	0.010584309
1219.239977	403.756743	9.11223E-06	0.011109992
1219.279977	431.491936	9.73817E-06	0.011873558
1219.319977	395.546167	8.92693E-06	0.010884779
1219.359977	367.907411	8.30316E-06	0.010124538
1219.399977	341.208037	7.70059E-06	0.009390099
1219.439977	295.134489	6.66077E-06	0.008122414
1219.479977	247.880457	5.59432E-06	0.006822157
1219.519977	221.335409	4.99523E-06	0.006091785
1219.559977	199.452448	4.50136E-06	0.005489683
1219.599977	193.584196	4.36893E-06	0.005328342
1219.639977	186.853124	4.21701E-06	0.00514324
1219.679977	203.260041	4.5873E-06	0.005595033
1219.719977	210.439235	4.74932E-06	0.005792841
1219.759977	217.208127	4.90208E-06	0.005979367
1219.799977	225.632084	5.0922E-06	0.006211468
1219.839977	225.502248	5.08927E-06	0.006208097
1219.879977	234.72102	5.29733E-06	0.006462103
1219.919977	246.607798	5.56559E-06	0.00678958
1219.959977	247.762984	5.59167E-06	0.006821608
1219.999977	242.325643	5.46895E-06	0.006672122
1220.039977	253.051498	5.71102E-06	0.006967673
1220.079977	260.598803	5.88135E-06	0.00717572
1220.119977	284.10871	6.41194E-06	0.007823333
1220.159977	323.255936	7.29544E-06	0.008901599
1220.199977	353.591253	7.98006E-06	0.009737272
1220.239977	366.077661	8.26186E-06	0.010081455
1220.279977	345.650887	7.80086E-06	0.009519232
1220.319977	310.582483	7.00941E-06	0.008553728
1220.359977	294.306537	6.64209E-06	0.008105739
1220.399977	282.603195	6.37796E-06	0.007783663
1220.439977	264.514781	5.96973E-06	0.007285697
1220.479977	232.843716	5.25496E-06	0.006413571
1220.519977	233.482192	5.26937E-06	0.006431369
1220.559977	231.774051	5.23082E-06	0.006384526
1220.599977	214.956385	4.85127E-06	0.005921455
1220.639977	200.039633	4.51462E-06	0.005510721
1220.679977	201.605918	4.54996E-06	0.005554051
1220.719977	215.180704	4.85633E-06	0.005928218
1220.759977	212.538467	4.7967E-06	0.005855616
1220.799977	234.166567	5.28481E-06	0.0064517
1220.839977	250.723078	5.65847E-06	0.006908087
1220.879977	240.053352	5.41767E-06	0.006614325

1220.919977	232.972258	5.25786E-06	0.006419426
1220.959977	240.95773	5.43808E-06	0.006639678
1220.999977	228.71262	5.16173E-06	0.006302466
1221.039977	253.76133	5.72704E-06	0.006992944
1221.079977	311.105086	7.02121E-06	0.008573457
1221.119977	340.163795	7.67702E-06	0.009374566
1221.159977	372.566009	8.4083E-06	0.010267874
1221.199977	410.80414	9.27128E-06	0.011322084
1221.239977	420.394786	9.48772E-06	0.011586789
1221.279977	432.921004	9.77042E-06	0.011932423
1221.319977	427.493602	9.64794E-06	0.011783216
1221.359977	371.982201	8.39512E-06	0.010253464
1221.399977	306.299179	6.91275E-06	0.008443228
1221.439977	257.395994	5.80907E-06	0.007095429
1221.479977	220.295527	4.97176E-06	0.006072909
1221.519977	204.185259	4.60818E-06	0.00562898
1221.559977	195.60473	4.41453E-06	0.005392609
1221.599977	194.020385	4.37877E-06	0.005349105
1221.639977	195.891621	4.421E-06	0.005400872
1221.679977	204.905367	4.62443E-06	0.005649572
1221.719977	210.316292	4.74655E-06	0.00579895
1221.759977	217.003123	4.89746E-06	0.005983518
1221.799977	220.502802	4.97644E-06	0.006080216
1221.839977	210.258197	4.74523E-06	0.005797917
1221.879977	203.733743	4.59799E-06	0.005618188
1221.919977	199.163979	4.49485E-06	0.005492351
1221.959977	190.649866	4.3027E-06	0.00525773
1221.999977	210.200057	4.74392E-06	0.005797073
1222.039977	224.963621	5.07712E-06	0.006204438
1222.079977	250.685886	5.65763E-06	0.006914078
1222.119977	285.959999	6.45372E-06	0.007887219
1222.159977	299.073157	6.74966E-06	0.00824917
1222.199977	324.533621	7.32427E-06	0.008951725
1222.239977	372.409747	8.40477E-06	0.010272645
1222.279977	396.399329	8.94618E-06	0.010934738
1222.319977	363.644237	8.20694E-06	0.010031511
1222.359977	321.731983	7.26104E-06	0.008875608
1222.399977	304.899922	6.88117E-06	0.008411538
1222.439977	282.453206	6.37458E-06	0.007792536
1222.479977	264.19402	5.96249E-06	0.007289026
1222.519977	242.735116	5.47819E-06	0.006697201
1222.559977	209.420803	4.72634E-06	0.005778229
1222.599977	208.679904	4.70961E-06	0.005757975
1222.639977	209.559397	4.72946E-06	0.005782431
1222.679977	212.620517	4.79855E-06	0.00586709
1222.719977	229.018828	5.16864E-06	0.006319794

1222.759977	236.650909	5.34088E-06	0.006530616
1222.799977	220.202846	4.96967E-06	0.006076914
1222.839977	205.610034	4.64033E-06	0.005674384
1222.879977	217.185869	4.90158E-06	0.005994047
1222.919977	237.231029	5.35397E-06	0.006547482
1222.959977	247.502462	5.58579E-06	0.006831193
1222.999977	265.676104	5.99594E-06	0.007333034
1223.039977	300.275485	6.7768E-06	0.008288297
1223.079977	337.410849	7.61489E-06	0.009313623
1223.119977	356.968547	8.05628E-06	0.009853801
1223.159977	379.887086	8.57352E-06	0.010486789
1223.199977	388.772175	8.77405E-06	0.010732413
1223.239977	413.270939	9.32695E-06	0.011409097
1223.279977	450.779658	1.01735E-05	0.012445001
1223.319977	408.018534	9.20841E-06	0.011264832
1223.359977	351.541059	7.93379E-06	0.009705884
1223.399977	301.87485	6.81289E-06	0.008334895
1223.439977	258.090784	5.82475E-06	0.007126231
1223.479977	231.940031	5.23456E-06	0.006404384
1223.519977	220.578735	4.97815E-06	0.006090872
1223.559977	209.9697	4.73872E-06	0.005798113
1223.599977	205.891322	4.64668E-06	0.005685678
1223.639977	209.032509	4.71757E-06	0.00577261
1223.679977	218.266188	4.92596E-06	0.006027803
1223.719977	215.495608	4.86344E-06	0.005951483
1223.759977	222.890365	5.03033E-06	0.006155911
1223.799977	240.611045	5.43026E-06	0.006645547
1223.839977	261.752697	5.90739E-06	0.007229705
1223.879977	249.795705	5.63754E-06	0.006899674
1223.919977	241.287305	5.44552E-06	0.006664879
1223.959977	227.307285	5.13001E-06	0.006278925
1223.999977	212.211534	4.78932E-06	0.005862126
1224.039977	227.826983	5.14174E-06	0.006293692
1224.079977	270.793476	6.11143E-06	0.007480881
1224.119977	290.266607	6.55091E-06	0.008019103
1224.159977	302.520178	6.82746E-06	0.008357902
1224.199977	323.763092	7.30688E-06	0.008945085
1224.239977	326.551498	7.36981E-06	0.009022419
1224.279977	317.026526	7.15485E-06	0.008759536
1224.319977	313.648305	7.07861E-06	0.008666478
1224.359977	296.101501	6.6826E-06	0.008181906
1224.399977	266.769731	6.02062E-06	0.007371649
1224.439977	259.152068	5.8487E-06	0.007161384
1224.479977	242.834291	5.48043E-06	0.006710679
1224.519977	214.447252	4.83978E-06	0.005926402
1224.559977	209.837027	4.73573E-06	0.005799185

1224.599977	210.591787	4.75276E-06	0.005820234
1224.639977	231.036375	5.21417E-06	0.00638548
1224.679977	259.977224	5.86732E-06	0.007185594
1224.719977	250.557508	5.65473E-06	0.006925466
1224.759977	256.028964	5.77822E-06	0.007076929
1224.799977	272.707415	6.15463E-06	0.007538186
1224.839977	272.550156	6.15108E-06	0.007534085
1224.879977	260.859506	5.88724E-06	0.007211157
1224.919977	246.847404	5.571E-06	0.006824032
1224.959977	239.353475	5.40187E-06	0.00661708
1224.999977	240.997272	5.43897E-06	0.006662741
1225.039977	261.928614	5.91136E-06	0.007241657
1225.079977	311.987511	7.04112E-06	0.008625939
1225.119977	355.382475	8.02049E-06	0.009826059
1225.159977	383.412627	8.65309E-06	0.010601418
1225.199977	406.030497	9.16354E-06	0.011227172
1225.239977	453.223585	1.02286E-05	0.01253252
1225.279977	501.729124	1.13233E-05	0.013874246
1225.319977	512.86652	1.15747E-05	0.01418269
1225.359977	466.985077	1.05392E-05	0.012914317
1225.399977	390.139093	8.8049E-06	0.010789519
1225.439977	293.45253	6.62281E-06	0.008115862
1225.479977	230.860397	5.2102E-06	0.006384993
1225.519977	218.305356	4.92685E-06	0.00603795
1225.559977	223.621574	5.04683E-06	0.00618519
1225.599977	220.160408	4.96871E-06	0.006089655
1225.639977	213.384807	4.8158E-06	0.005902434
1225.679977	231.009147	5.21355E-06	0.00639015
1225.719977	255.613137	5.76883E-06	0.007070973
1225.759977	314.224511	7.09161E-06	0.008692611
1225.799977	371.95555	8.39452E-06	0.010290001
1225.839977	400.768224	9.04478E-06	0.011087454
1225.879977	392.678917	8.86222E-06	0.010864013
1225.919977	363.941861	8.21366E-06	0.010069291
1225.959977	299.236253	6.75335E-06	0.008279331
1225.999977	251.577384	5.67775E-06	0.006960923
1226.039977	240.446727	5.42655E-06	0.006653164
1226.079977	238.120948	5.37406E-06	0.006589025
1226.119977	258.841851	5.8417E-06	0.007162625
1226.159977	300.645278	6.78515E-06	0.008319673
1226.199977	344.459836	7.77398E-06	0.009532453
1226.239977	406.152878	9.1663E-06	0.011240089
1226.279977	440.591949	9.94355E-06	0.012193572
1226.319977	430.65604	9.71931E-06	0.01191898
1226.359977	434.93428	9.81586E-06	0.012037779
1226.399977	394.627278	8.90619E-06	0.010922549

1226.439977	308.841747	6.97013E-06	0.008548444
1226.479977	257.34825	5.80799E-06	0.007123385
1226.519977	230.35014	5.19868E-06	0.006376287
1226.559977	203.246318	4.58699E-06	0.005626214
1226.599977	183.375231	4.13852E-06	0.005076313
1226.639977	193.604312	4.36938E-06	0.005359656
1226.679977	235.811843	5.32194E-06	0.006528323
1226.719977	277.790521	6.26935E-06	0.007690731
1226.759977	303.10757	6.84072E-06	0.008391916
1226.799977	316.238199	7.13706E-06	0.00875574
1226.839977	299.836663	6.7669E-06	0.008301898
1226.879977	261.679713	5.90575E-06	0.007245642
1226.919977	263.220132	5.94051E-06	0.007288533
1226.959977	257.392823	5.809E-06	0.007127407
1226.999977	249.838608	5.63851E-06	0.006918451
1227.039977	288.010772	6.5E-06	0.007975762
1227.079977	297.134661	6.70592E-06	0.008228695
1227.119977	287.308804	6.48416E-06	0.007956842
1227.159977	306.436678	6.91585E-06	0.008486853
1227.199977	342.301439	7.72527E-06	0.009480447
1227.239977	378.762749	8.54815E-06	0.010490629
1227.279977	402.492884	9.0837E-06	0.011148248
1227.319977	370.44701	8.36047E-06	0.010260976
1227.359977	305.763858	6.90066E-06	0.008469599
1227.399977	281.124844	6.3446E-06	0.007787357
1227.439977	263.519302	5.94726E-06	0.007299909
1227.479977	242.880477	5.48147E-06	0.0067284
1227.519977	223.05008	5.03393E-06	0.006179249
1227.559977	213.100079	4.80937E-06	0.005903793
1227.599977	212.949293	4.80597E-06	0.005899807
1227.639977	224.117436	5.05802E-06	0.006209426
1227.679977	222.29352	5.01686E-06	0.006159093
1227.719977	219.43309	4.9523E-06	0.006080037
1227.759977	223.875043	5.05255E-06	0.006203316
1227.799977	225.024365	5.07849E-06	0.006235366
1227.839977	226.586998	5.11375E-06	0.00627887
1227.879977	220.214011	4.96992E-06	0.00610247
1227.919977	206.195915	4.65355E-06	0.005714193
1227.959977	237.450018	5.35892E-06	0.006580535
1227.999977	252.731406	5.7038E-06	0.007004261
1228.039977	252.905634	5.70773E-06	0.007009318
1228.079977	266.276216	6.00948E-06	0.007380126
1228.119977	270.973222	6.11549E-06	0.007510553
1228.159977	273.826119	6.17987E-06	0.007589874
1228.199977	237.089611	5.35078E-06	0.006571831
1228.239977	245.678573	5.54462E-06	0.006810128

1228.279977	300.669617	6.78569E-06	0.008334733
1228.319977	327.746641	7.39679E-06	0.009085619
1228.359977	336.548277	7.59543E-06	0.009329917
1228.399977	322.46787	7.27765E-06	0.008939866
1228.439977	284.794591	6.42742E-06	0.007895696
1228.479977	258.56108	5.83536E-06	0.007168627
1228.519977	225.738082	5.09459E-06	0.006258811
1228.559977	210.551909	4.75186E-06	0.005837949
1228.599977	220.836109	4.98396E-06	0.006123297
1228.639977	268.756849	6.06547E-06	0.007452276
1228.679977	299.969836	6.7699E-06	0.008318042
1228.719977	307.303377	6.93541E-06	0.008521676
1228.759977	285.219475	6.43701E-06	0.007909536
1228.799977	262.399139	5.92198E-06	0.007276933
1228.839977	258.166816	5.82647E-06	0.007159794
1228.879977	250.853018	5.6614E-06	0.006957185
1228.919977	246.721792	5.56817E-06	0.006842832
1228.959977	249.170383	5.62343E-06	0.006910969
1228.999977	245.002452	5.52936E-06	0.006795588
1229.039977	246.509418	5.56337E-06	0.006837609
1229.079977	286.031393	6.45533E-06	0.007934117
1229.119977	330.148185	7.45098E-06	0.009158154
1229.159977	361.404489	8.1564E-06	0.010025515
1229.199977	410.744077	9.26992E-06	0.011394588
1229.239977	411.890503	9.29579E-06	0.011426763
1229.279977	381.444637	8.60867E-06	0.010582471
1229.319977	348.665483	7.86889E-06	0.009673389
1229.359977	305.587482	6.89668E-06	0.008478507
1229.399977	279.043143	6.29762E-06	0.007742288
1229.439977	268.810972	6.06669E-06	0.00745863
1229.479977	258.928917	5.84366E-06	0.007184669
1229.519977	221.267621	4.9937E-06	0.006139856
1229.559977	172.37938	3.89036E-06	0.004783434
1229.599977	176.040091	3.97298E-06	0.004885176
1229.639977	198.29474	4.47524E-06	0.005502929
1229.679977	218.867754	4.93954E-06	0.006074054
1229.719977	213.782304	4.82477E-06	0.005933115
1229.759977	206.398404	4.65812E-06	0.005728375
1229.799977	223.748852	5.0497E-06	0.006210121
1229.839977	226.624582	5.1146E-06	0.006290141
1229.879977	208.895425	4.71448E-06	0.005798243
1229.919977	202.183183	4.56299E-06	0.005612116
1229.959977	196.37005	4.4318E-06	0.005450935
1229.999977	196.452181	4.43365E-06	0.005453392
1230.039977	214.770269	4.84707E-06	0.005962085
1230.079977	232.696978	5.25165E-06	0.006459945

1230.119977	270.086104	6.09547E-06	0.007498156
1230.159977	299.473611	6.7587E-06	0.008314285
1230.199977	329.185276	7.42925E-06	0.009139467
1230.239977	365.093504	8.23965E-06	0.010136749
1230.279977	368.405554	8.3144E-06	0.01022904
1230.319977	321.133113	7.24753E-06	0.008916777
1230.359977	321.231211	7.24974E-06	0.008919791
1230.399977	339.749637	7.66768E-06	0.009434308
1230.439977	314.506292	7.09797E-06	0.008733625
1230.479977	249.563547	5.6323E-06	0.006930434
1230.519977	185.946	4.19654E-06	0.005163929
1230.559977	160.825742	3.62961E-06	0.004466456
1230.599977	164.208046	3.70595E-06	0.004560538
1230.639977	184.128642	4.15553E-06	0.005113958
1230.679977	223.232853	5.03805E-06	0.006200233
1230.719977	241.350516	5.44694E-06	0.006703664
1230.759977	241.509173	5.45053E-06	0.006708289
1230.799977	250.805026	5.66032E-06	0.006966722
1230.839977	267.1582	6.02939E-06	0.007421212
1230.879977	274.456295	6.1941E-06	0.007624189
1230.919977	272.191459	6.14298E-06	0.007561519
1230.959977	258.744482	5.8395E-06	0.007188194
1230.999977	247.232363	5.57969E-06	0.006868598
1231.039977	258.940602	5.84393E-06	0.00719411
1231.079977	278.402453	6.28316E-06	0.007735067
1231.119977	309.203652	6.9783E-06	0.008591119
1231.159977	343.854603	7.76032E-06	0.009554195
1231.199977	351.626578	7.93572E-06	0.009770461
1231.239977	339.246844	7.65633E-06	0.009426778
1231.279977	347.724717	7.84766E-06	0.00966267
1231.319977	339.256702	7.65655E-06	0.009427664
1231.359977	291.196986	6.57191E-06	0.008092387
1231.399977	248.021538	5.5975E-06	0.006892762
1231.439977	233.323265	5.26578E-06	0.006484493
1231.479977	225.582818	5.09109E-06	0.006269575
1231.519977	210.862096	4.75886E-06	0.005860636
1231.559977	189.046265	4.26651E-06	0.005254464
1231.599977	192.581632	4.3463E-06	0.005352902
1231.639977	203.715526	4.59758E-06	0.005662558
1231.679977	210.671054	4.75455E-06	0.005856087
1231.719977	202.455274	4.56913E-06	0.005627893
1231.759977	193.805732	4.37393E-06	0.005387626
1231.799977	216.099807	4.87707E-06	0.006007577
1231.839977	243.371381	5.49255E-06	0.006765946
1231.879977	235.770416	5.32101E-06	0.006554846
1231.919977	225.751045	5.09489E-06	0.006276493

1231.959977	229.769067	5.18557E-06	0.006388412
1231.999977	246.138026	5.55499E-06	0.006843751
1232.039977	236.656767	5.34101E-06	0.006580342
1232.079977	227.563459	5.13579E-06	0.006327704
1232.119977	245.598807	5.54282E-06	0.006829423
1232.159977	265.982184	6.00285E-06	0.007396468
1232.199977	288.552069	6.51222E-06	0.008024355
1232.239977	299.418492	6.75746E-06	0.00832681
1232.279977	289.000315	6.52233E-06	0.008037342
1232.319977	293.924914	6.63348E-06	0.008174565
1232.359977	297.421162	6.71238E-06	0.00827207
1232.399977	279.880309	6.31651E-06	0.007784465
1232.439977	263.874471	5.95528E-06	0.007339524
1232.479977	227.120448	5.12579E-06	0.006317436
1232.519977	200.786626	4.53147E-06	0.005585133
1232.559977	202.596386	4.57232E-06	0.005635656
1232.599977	203.188504	4.58568E-06	0.005652311
1232.639977	213.69896	4.82289E-06	0.005944884
1232.679977	229.162136	5.17187E-06	0.006375261
1232.719977	238.555561	5.38387E-06	0.0066368
1232.759977	239.896054	5.41412E-06	0.00667431
1232.799977	245.293288	5.53593E-06	0.006824692
1232.839977	239.976658	5.41594E-06	0.006676986
1232.879977	232.748798	5.25282E-06	0.006476092
1232.919977	237.088062	5.35075E-06	0.006597043
1232.959977	240.129637	5.41939E-06	0.006681893
1232.999977	243.603986	5.4978E-06	0.006778791
1233.039977	250.192533	5.6465E-06	0.006962357
1233.079977	261.550877	5.90284E-06	0.007278673
1233.119977	302.365918	6.82398E-06	0.008414783
1233.159977	358.136583	8.08264E-06	0.009967193
1233.199977	393.203138	8.87405E-06	0.010943475
1233.239977	378.931536	8.55196E-06	0.010546615
1233.279977	358.396551	8.08851E-06	0.009975399
1233.319977	360.887111	8.14472E-06	0.010045045
1233.359977	342.982406	7.74064E-06	0.00954699
1233.399977	297.834361	6.72171E-06	0.008290553
1233.439977	253.165693	5.7136E-06	0.007047379
1233.479977	251.020876	5.66519E-06	0.0069879
1233.519977	237.95297	5.37027E-06	0.006624332
1233.559977	191.445615	4.32066E-06	0.005329794
1233.599977	182.5763	4.12049E-06	0.00508304
1233.639977	199.283722	4.49756E-06	0.005548365
1233.679977	215.249564	4.85788E-06	0.005993073
1233.719977	212.638144	4.79895E-06	0.005920556
1233.759977	208.180696	4.69835E-06	0.005796634

1233.799977	199.399907	4.50018E-06	0.00555232
1233.839977	193.84078	4.37472E-06	0.0053977
1233.879977	211.574218	4.77494E-06	0.005891697
1233.919977	228.730816	5.16214E-06	0.006369663
1233.959977	224.080515	5.05719E-06	0.006240364
1233.999977	202.161344	4.5625E-06	0.005630125
1234.039977	206.562953	4.66184E-06	0.005752895
1234.079977	242.775109	5.4791E-06	0.006761643
1234.119977	278.886327	6.29408E-06	0.007767645
1234.159977	327.099176	7.38217E-06	0.009110782
1234.199977	358.358957	8.08766E-06	0.009981793
1234.239977	363.980461	8.21453E-06	0.010138704
1234.279977	380.825261	8.5947E-06	0.010608261
1234.319977	385.497371	8.70014E-06	0.010738755
1234.359977	355.373654	8.02029E-06	0.009899923
1234.399977	293.9361	6.63373E-06	0.008188674
1234.439977	239.184182	5.39805E-06	0.006663573
1234.479977	211.719817	4.77822E-06	0.005898619
1234.519977	198.699683	4.48438E-06	0.005536051
1234.559977	194.578375	4.39136E-06	0.005421401
1234.599977	199.099402	4.4934E-06	0.005547547
1234.639977	211.604679	4.77562E-06	0.005896175
1234.679977	217.468732	4.90797E-06	0.006059768
1234.719977	234.997249	5.30356E-06	0.006548412
1234.759977	257.509244	5.81162E-06	0.007175962
1234.799977	257.001471	5.80017E-06	0.007162044
1234.839977	246.895836	5.5721E-06	0.006880646
1234.879977	242.88689	5.48162E-06	0.006769141
1234.919977	252.444044	5.69731E-06	0.007035722
1234.959977	266.604672	6.0169E-06	0.007430626
1234.999977	296.229321	6.68548E-06	0.008256572
1235.039977	317.979156	7.17635E-06	0.008863075
1235.079977	307.512889	6.94014E-06	0.008571625
1235.119977	319.057002	7.20067E-06	0.008893694
1235.159977	321.811946	7.26285E-06	0.008970778
1235.199977	344.832945	7.7824E-06	0.009612819
1235.239977	385.670896	8.70405E-06	0.010751597
1235.279977	396.613739	8.95102E-06	0.011057015
1235.319977	368.85389	8.32452E-06	0.010283444
1235.359977	341.892128	7.71603E-06	0.009532074
1235.399977	314.078639	7.08832E-06	0.008756907
1235.439977	270.720664	6.10979E-06	0.007548277
1235.479977	220.873606	4.98481E-06	0.006158632
1235.519977	203.949093	4.60285E-06	0.005686909
1235.559977	192.426688	4.3428E-06	0.005365793
1235.599977	202.360542	4.567E-06	0.00564298

1235.639977	226.237588	5.10587E-06	0.006309014
1235.679977	241.755228	5.45608E-06	0.006741967
1235.719977	233.265734	5.26448E-06	0.006505426
1235.759977	219.11068	4.94502E-06	0.006110861
1235.799977	224.823386	5.07395E-06	0.006270388
1235.839977	233.156024	5.26201E-06	0.006502998
1235.879977	222.902542	5.0306E-06	0.006217218
1235.919977	205.178323	4.63059E-06	0.005723037
1235.959977	211.966758	4.78379E-06	0.005912579
1235.999977	229.503979	5.17959E-06	0.006401967
1236.039977	236.867761	5.34578E-06	0.006607592
1236.079977	256.574288	5.79052E-06	0.007157551
1236.119977	297.94925	6.7243E-06	0.008312041
1236.159977	299.126081	6.75086E-06	0.008345142
1236.199977	305.694741	6.8991E-06	0.008528673
1236.239977	353.506497	7.97815E-06	0.009862907
1236.279977	386.966339	8.73329E-06	0.010796793
1236.319977	370.198164	8.35486E-06	0.010329277
1236.359977	330.642703	7.46215E-06	0.009225898
1236.399977	310.418796	7.00572E-06	0.008661872
1236.439977	304.064383	6.86231E-06	0.008484834
1236.479977	254.806795	5.75063E-06	0.007110544
1236.519977	211.813608	4.78034E-06	0.005910984
1236.559977	191.229127	4.31578E-06	0.005336715
1236.599977	183.636941	4.14443E-06	0.005125002
1236.639977	176.125347	3.9749E-06	0.004915525
1236.679977	197.67379	4.46122E-06	0.005517104
1236.719977	237.108613	5.35121E-06	0.00661795
1236.759977	270.736642	6.11015E-06	0.007556788
1236.799977	282.323122	6.37164E-06	0.007880444
1236.839977	272.098797	6.14089E-06	0.007595299
1236.879977	288.185192	6.50394E-06	0.008044591
1236.919977	280.177126	6.32321E-06	0.007821302
1236.959977	254.85078	5.75163E-06	0.007114533
1236.999977	248.197576	5.60147E-06	0.006929023
1237.039977	272.265266	6.14465E-06	0.007601175
1237.079977	294.434163	6.64497E-06	0.008220358
1237.119977	319.951467	7.22086E-06	0.008933069
1237.159977	334.396015	7.54685E-06	0.009336664
1237.199977	382.962127	8.64292E-06	0.010693023
1237.239977	422.175457	9.52791E-06	0.011788314
1237.279977	432.63146	9.76389E-06	0.012080665
1237.319977	404.694895	9.1334E-06	0.011300938
1237.359977	341.258947	7.70174E-06	0.009529824
1237.399977	269.065063	6.07242E-06	0.007514017
1237.439977	244.378678	5.51529E-06	0.006824836

1237.479977	249.636476	5.63395E-06	0.006971897
1237.519977	224.910795	5.07592E-06	0.006281557
1237.559977	196.366239	4.43171E-06	0.00548451
1237.599977	179.355421	4.0478E-06	0.00500956
1237.639977	183.307061	4.13699E-06	0.005120098
1237.679977	203.293759	4.58806E-06	0.005678546
1237.719977	220.052687	4.96628E-06	0.006146867
1237.759977	228.525805	5.15751E-06	0.006383758
1237.799977	238.743146	5.3881E-06	0.00666939
1237.839977	234.588991	5.29435E-06	0.006553554
1237.879977	227.459176	5.13344E-06	0.006354579
1237.919977	230.816988	5.20922E-06	0.006448595
1237.959977	231.45786	5.22368E-06	0.006466709
1237.999977	212.280459	4.79087E-06	0.005931102
1238.039977	196.513872	4.43504E-06	0.005490762
1238.079977	227.547219	5.13542E-06	0.006358065
1238.119977	270.543537	6.10579E-06	0.007559702
1238.159977	301.814741	6.81154E-06	0.008433774
1238.199977	325.687386	7.35031E-06	0.009101154
1238.239977	334.702316	7.55377E-06	0.009353374
1238.279977	358.936509	8.1007E-06	0.010030931
1238.319977	375.751049	8.48018E-06	0.010501174
1238.359977	350.219179	7.90396E-06	0.009787947
1238.399977	319.982147	7.22155E-06	0.008943169
1238.439977	281.637376	6.35616E-06	0.007871727
1238.479977	230.014241	5.1911E-06	0.006429075
1238.519977	195.554866	4.4134E-06	0.005466085
1238.559977	182.028184	4.10812E-06	0.005088156
1238.599977	184.878076	4.17244E-06	0.005167985
1238.639977	184.110106	4.15511E-06	0.005146684
1238.679977	191.523137	4.32241E-06	0.005354083
1238.719977	238.5427	5.38358E-06	0.006668744
1238.759977	266.42878	6.01293E-06	0.007448573
1238.799977	266.110207	6.00574E-06	0.007439906
1238.839977	236.832378	5.34498E-06	0.006621571
1238.879977	219.061839	4.94392E-06	0.006124924
1238.919977	216.187944	4.87906E-06	0.006044766
1238.959977	217.854951	4.91668E-06	0.006091573
1238.999977	234.560096	5.29369E-06	0.006558888
1239.039977	259.349744	5.85316E-06	0.007252302
1239.079977	302.499217	6.82699E-06	0.008459182
1239.119977	332.693706	7.50843E-06	0.00930385
1239.159977	348.527215	7.86577E-06	0.009746952
1239.199977	370.010578	8.35062E-06	0.010348092
1239.239977	376.882968	8.50572E-06	0.010540633
1239.279977	370.09607	8.35255E-06	0.010351151

1239.319977	346.401872	7.81781E-06	0.009688765
1239.359977	328.342073	7.41022E-06	0.009183934
1239.399977	309.403817	6.98281E-06	0.008654499
1239.439977	275.432943	6.21614E-06	0.00770453
1239.479977	265.96483	6.00246E-06	0.007439924
1239.519977	267.207649	6.0305E-06	0.007474931
1239.559977	229.447902	5.17832E-06	0.006418838
1239.599977	204.107399	4.60642E-06	0.005710118
1239.639977	207.348705	4.67957E-06	0.005800984
1239.679977	199.265209	4.49714E-06	0.005575012
1239.719977	198.465807	4.4791E-06	0.005552826
1239.759977	220.336426	4.97269E-06	0.006164937
1239.799977	210.460855	4.74981E-06	0.005888812
1239.839977	206.722667	4.66544E-06	0.005784402
1239.879977	210.855387	4.75871E-06	0.005900232
1239.919977	208.982934	4.71645E-06	0.005848025
1239.959977	219.452546	4.95274E-06	0.006141197
1239.999977	214.803614	4.84782E-06	0.006011295
1240.039977	216.447732	4.88492E-06	0.006057501
1240.079977	219.69729	4.95826E-06	0.006148641
1240.119977	234.726776	5.29746E-06	0.006569481
1240.159977	294.388119	6.64393E-06	0.008239536
1240.199977	333.894578	7.53554E-06	0.009345571
1240.239977	340.690661	7.68891E-06	0.009536098
1240.279977	330.516605	7.4593E-06	0.00925162
1240.319977	310.032604	6.997E-06	0.008678524
1240.359977	304.010429	6.86109E-06	0.008510224
1240.399977	277.94742	6.27289E-06	0.007780888
1240.439977	249.194458	5.62397E-06	0.006976199
1240.479977	217.731709	4.9139E-06	0.006095596
1240.519977	179.286113	4.04624E-06	0.005019439
1240.559977	151.166148	3.41161E-06	0.004232306
1240.599977	153.711477	3.46905E-06	0.004303708
1240.639977	193.81749	4.37419E-06	0.005426796
1240.679977	217.855368	4.91669E-06	0.006100042
1240.719977	239.885542	5.41388E-06	0.006717112
1240.759977	268.85142	6.0676E-06	0.007528438
1240.799977	280.504546	6.3306E-06	0.007855004
1240.839977	262.236861	5.91832E-06	0.007343689
1240.879977	230.335672	5.19836E-06	0.006450535
1240.919977	204.992695	4.6264E-06	0.005740992
1240.959977	218.20775	4.92464E-06	0.006111287
1240.999977	244.244135	5.51225E-06	0.006840702
1241.039977	263.58615	5.94877E-06	0.007382664
1241.079977	272.989005	6.16098E-06	0.007646271
1241.119977	275.877188	6.22616E-06	0.007727416

1241.159977	273.364004	6.16944E-06	0.007657268
1241.199977	296.056724	6.68159E-06	0.008293187
1241.239977	318.202386	7.18138E-06	0.008913822
1241.279977	329.926666	7.44599E-06	0.009242552
1241.319977	337.361161	7.61377E-06	0.009451127
1241.359977	308.972684	6.97308E-06	0.008656106
1241.399977	288.305543	6.50665E-06	0.008077361
1241.439977	276.894505	6.24912E-06	0.007757911
1241.479977	257.751822	5.8171E-06	0.007221813
1241.519977	224.691381	5.07097E-06	0.006295712
1241.559977	185.31035	4.1822E-06	0.005192448
1241.599977	164.842548	3.72027E-06	0.004619083
1241.639977	173.677045	3.91965E-06	0.004866793
1241.679977	184.300617	4.15941E-06	0.005164654
1241.719977	188.126434	4.24575E-06	0.005272035
1241.759977	201.969607	4.55817E-06	0.005660156
1241.799977	197.211278	4.45078E-06	0.005526983
1241.839977	175.958151	3.97113E-06	0.004931508
1241.879977	157.278855	3.54956E-06	0.004408133
1241.919977	145.719145	3.28868E-06	0.004084275
1241.959977	163.033464	3.67944E-06	0.004569715
1241.999977	186.19725	4.20221E-06	0.005219148
1242.039977	212.466866	4.79508E-06	0.005955683
1242.079977	234.935637	5.30217E-06	0.006585719
1242.119977	260.54707	5.88018E-06	0.007303895
1242.159977	301.654735	6.80793E-06	0.008456535
1242.199977	312.702942	7.05727E-06	0.00876654
1242.239977	305.503863	6.8948E-06	0.008564992
1242.279977	325.028195	7.33543E-06	0.009112662
1242.319977	341.762577	7.71311E-06	0.009582145
1242.359977	325.165209	7.33853E-06	0.009117091
1242.399977	298.469067	6.73603E-06	0.008368845
1242.439977	253.374362	5.71831E-06	0.007104653
1242.479977	209.483003	4.72774E-06	0.005874122
1242.519977	195.292609	4.40748E-06	0.005476385
1242.559977	187.699159	4.23611E-06	0.005263619
1242.599977	199.851807	4.51038E-06	0.005604594
1242.639977	204.225491	4.60908E-06	0.005727433
1242.679977	199.336112	4.49874E-06	0.005590492
1242.719977	216.694092	4.89048E-06	0.006077502
1242.759977	237.04332	5.34974E-06	0.00664844
1242.799977	225.763995	5.09518E-06	0.006332288
1242.839977	210.365395	4.74765E-06	0.005900574
1242.879977	212.647765	4.79916E-06	0.005964785
1242.919977	234.617216	5.29498E-06	0.006581241
1242.959977	231.354368	5.22135E-06	0.006489924

1242.999977	228.055316	5.14689E-06	0.006397585
1243.039977	256.423663	5.78712E-06	0.007193628
1243.079977	286.752254	6.4716E-06	0.008044715
1243.119977	302.93968	6.83693E-06	0.00849912
1243.159977	322.839368	7.28603E-06	0.009057707
1243.199977	342.080626	7.72028E-06	0.009597856
1243.239977	363.877879	8.21222E-06	0.010209756
1243.279977	372.229931	8.40071E-06	0.010444436
1243.319977	348.57028	7.86675E-06	0.009780882
1243.359977	306.756373	6.92306E-06	0.008607861
1243.399977	253.035982	5.71067E-06	0.007100647
1243.439977	211.326571	4.76935E-06	0.005930396
1243.479977	201.739106	4.55297E-06	0.005661528
1243.519977	196.534711	4.43551E-06	0.005515651
1243.559977	204.122029	4.60675E-06	0.00572877
1243.599977	191.625508	4.32472E-06	0.005378223
1243.639977	180.140382	4.06552E-06	0.00505604
1243.679977	173.892903	3.92452E-06	0.004880848
1243.719977	197.332474	4.45352E-06	0.005538931
1243.759977	208.265258	4.70026E-06	0.005845991
1243.799977	211.6728	4.77716E-06	0.005941832
1243.839977	217.228283	4.90254E-06	0.006097975
1243.879977	210.174494	4.74335E-06	0.005900153
1243.919977	196.592702	4.43682E-06	0.005519053
1243.959977	206.119672	4.65183E-06	0.005786695
1243.999977	247.626577	5.58859E-06	0.006952202
1244.039977	253.195374	5.71427E-06	0.007108776
1244.079977	240.687508	5.43198E-06	0.00675782
1244.119977	263.901934	5.9559E-06	0.007409853
1244.159977	313.953012	7.08548E-06	0.008815473
1244.199977	345.374502	7.79462E-06	0.009698068
1244.239977	338.18533	7.63237E-06	0.009496502
1244.279977	332.819029	7.51126E-06	0.009346113
1244.319977	325.124808	7.33761E-06	0.00913034
1244.359977	315.152651	7.11256E-06	0.00885058
1244.399977	312.790811	7.05925E-06	0.008784534
1244.439977	272.861506	6.1581E-06	0.007663391
1244.479977	231.257306	5.21916E-06	0.006495134
1244.519977	197.221183	4.45101E-06	0.005539368
1244.559977	181.122307	4.08768E-06	0.005087361
1244.599977	194.373107	4.38673E-06	0.005459724
1244.639977	210.920126	4.76017E-06	0.005924702
1244.679977	209.609412	4.73059E-06	0.005888074
1244.719977	209.318473	4.72403E-06	0.00588009
1244.759977	243.161421	5.48781E-06	0.006831012
1244.799977	275.141957	6.20957E-06	0.007729673

1244.839977	270.683331	6.10895E-06	0.00760466
1244.879977	257.421325	5.80964E-06	0.007232305
1244.919977	215.347142	4.86009E-06	0.006050417
1244.959977	178.5109	4.02874E-06	0.005015623
1244.999977	213.466411	4.81764E-06	0.005997961
1245.039977	256.120814	5.78029E-06	0.007196692
1245.079977	269.060963	6.07233E-06	0.007560538
1245.119977	289.029525	6.52299E-06	0.00812191
1245.159977	317.922382	7.17507E-06	0.008934104
1245.199977	361.079365	8.14906E-06	0.010147207
1245.239977	401.877917	9.06982E-06	0.011294108
1245.279977	378.697131	8.54667E-06	0.010642993
1245.319977	341.729776	7.71237E-06	0.009604362
1245.359977	310.677295	7.01155E-06	0.008731908
1245.399977	286.867711	6.4742E-06	0.008062974
1245.439977	289.010617	6.52257E-06	0.008123466
1245.479977	268.719487	6.06462E-06	0.007553368
1245.519977	220.003418	4.96517E-06	0.006184219
1245.559977	204.832343	4.62278E-06	0.005757951
1245.599977	217.698849	4.91316E-06	0.006119832
1245.639977	224.538697	5.06753E-06	0.006312313
1245.679977	223.694399	5.04847E-06	0.006288779
1245.719977	232.855896	5.25523E-06	0.006546549
1245.759977	228.587658	5.15891E-06	0.006426757
1245.799977	223.100272	5.03506E-06	0.006272681
1245.839977	226.467525	5.11106E-06	0.006367559
1245.879977	227.738625	5.13974E-06	0.006403504
1245.919977	212.615108	4.79843E-06	0.005978456
1245.959977	215.35209	4.8602E-06	0.006055611
1245.999977	223.185874	5.03699E-06	0.006276095
1246.039977	217.075598	4.89909E-06	0.006104467
1246.079977	222.884322	5.03019E-06	0.006268017
1246.119977	237.407535	5.35796E-06	0.006676658
1246.159977	265.485054	5.99163E-06	0.007466527
1246.199977	303.229031	6.84346E-06	0.008528316
1246.239977	324.702264	7.32808E-06	0.009132544
1246.279977	314.24087	7.09198E-06	0.008838591
1246.319977	323.679383	7.30499E-06	0.009104358
1246.359977	323.875622	7.30942E-06	0.009110171
1246.399977	285.554515	6.44457E-06	0.008032509
1246.439977	248.191929	5.60135E-06	0.006981742
1246.479977	216.182264	4.87893E-06	0.006081492
1246.519977	197.469107	4.4566E-06	0.005555244
1246.559977	212.614664	4.79842E-06	0.005981514
1246.599977	224.494057	5.06652E-06	0.006315921
1246.639977	231.752504	5.23033E-06	0.00652034

1246.679977	215.812811	4.87059E-06	0.006072073
1246.719977	190.548081	4.3004E-06	0.005361401
1246.759977	198.983956	4.49079E-06	0.005598938
1246.799977	238.220912	5.37631E-06	0.006703188
1246.839977	258.912819	5.8433E-06	0.007285662
1246.879977	231.866759	5.23291E-06	0.00652481
1246.919977	207.760351	4.68886E-06	0.005846635
1246.959977	210.321326	4.74666E-06	0.005918894
1246.999977	229.328562	5.17563E-06	0.006454006
1247.039977	247.662887	5.58941E-06	0.006970213
1247.079977	250.299864	5.64892E-06	0.007044654
1247.119977	266.636778	6.01762E-06	0.007504695
1247.159977	328.81123	7.42081E-06	0.009254939
1247.199977	374.363613	8.44887E-06	0.010537425
1247.239977	373.384315	8.42676E-06	0.010510197
1247.279977	358.763042	8.09678E-06	0.010098954
1247.319977	331.724436	7.48656E-06	0.009338134
1247.359977	319.009042	7.19959E-06	0.00898048
1247.399977	310.402342	7.00535E-06	0.008738472
1247.439977	290.506881	6.55634E-06	0.008178635
1247.479977	253.519362	5.72158E-06	0.007137555
1247.519977	211.436655	4.77183E-06	0.005952954
1247.559977	188.417289	4.25232E-06	0.005305019
1247.599977	186.83319	4.21656E-06	0.005260586
1247.639977	187.692357	4.23596E-06	0.005284947
1247.679977	218.66481	4.93496E-06	0.006157251
1247.719977	240.702002	5.43231E-06	0.006778
1247.759977	233.792713	5.27638E-06	0.006583651
1247.799977	230.235051	5.19608E-06	0.006483674
1247.839977	242.446092	5.47167E-06	0.006827769
1247.879977	230.747391	5.20765E-06	0.006498519
1247.919977	223.958276	5.05443E-06	0.00630752
1247.959977	232.193345	5.24028E-06	0.00653966
1247.999977	234.270278	5.28715E-06	0.006598368
1248.039977	242.349484	5.46949E-06	0.006826143
1248.079977	256.870553	5.79721E-06	0.007235382
1248.119977	265.840207	5.99964E-06	0.007488275
1248.159977	301.134201	6.79618E-06	0.008482719
1248.199977	341.702373	7.71175E-06	0.009625802
1248.239977	353.56516	7.97947E-06	0.009960297
1248.279977	347.194021	7.83569E-06	0.009781129
1248.319977	357.220021	8.06196E-06	0.010063904
1248.359977	350.717747	7.91521E-06	0.009881033
1248.399977	337.218635	7.61055E-06	0.009501017
1248.439977	301.167157	6.79692E-06	0.008485551
1248.479977	263.02846	5.93619E-06	0.007411209

1248.519977	251.215308	5.66958E-06	0.007078583
1248.559977	253.628857	5.72405E-06	0.00714682
1248.599977	266.70763	6.01922E-06	0.007515598
1248.639977	297.030927	6.70357E-06	0.008370351
1248.679977	312.350664	7.04932E-06	0.008802344
1248.719977	308.15774	6.95469E-06	0.008684462
1248.759977	332.065264	7.49425E-06	0.00935852
1248.799977	348.820479	7.87239E-06	0.009831043
1248.839977	334.752607	7.5549E-06	0.009434861
1248.879977	322.89531	7.2873E-06	0.00910096
1248.919977	321.028316	7.24516E-06	0.009048627
1248.959977	332.833186	7.51158E-06	0.009381665
1248.999977	336.024161	7.5836E-06	0.009471913
1249.039977	337.942983	7.6269E-06	0.009526306
1249.079977	369.477859	8.3386E-06	0.010415579
1249.119977	386.363366	8.71968E-06	0.01089193
1249.159977	403.201742	9.0997E-06	0.011366983
1249.199977	442.790545	9.99317E-06	0.012483463
1249.239977	450.89572	1.01761E-05	0.012712376
1249.279977	416.621051	9.40256E-06	0.011746426
1249.319977	409.869692	9.25019E-06	0.011556445
1249.359977	380.026299	8.57666E-06	0.010715341
1249.399977	319.556759	7.21195E-06	0.009010611
1249.439977	282.292465	6.37095E-06	0.007960117
1249.479977	241.783578	5.45672E-06	0.006818061
1249.519977	219.864853	4.96204E-06	0.006200172
1249.559977	208.161386	4.69791E-06	0.005870323
1249.599977	205.063659	4.628E-06	0.00578315
1249.639977	221.165854	4.99141E-06	0.00623746
1249.679977	244.188577	5.511E-06	0.006886981
1249.719977	255.261084	5.76089E-06	0.007199496
1249.759977	257.270598	5.80624E-06	0.007256405
1249.799977	273.085196	6.16315E-06	0.007702708
1249.839977	300.147879	6.77392E-06	0.008466315
1249.879977	283.620614	6.40092E-06	0.008000385
1249.919977	265.243151	5.98617E-06	0.007482232
1249.959977	274.626692	6.19794E-06	0.007747179
1249.999977	292.787544	6.60781E-06	0.008259759
1250.039977	293.584129	6.62578E-06	0.008282496
1250.079977	281.121432	6.34452E-06	0.007931156
1250.119977	277.554393	6.26402E-06	0.007830772
1250.159977	312.676059	7.05666E-06	0.008821958
1250.199977	364.872606	8.23467E-06	0.01029498
1250.239977	374.156334	8.44419E-06	0.010557261
1250.279977	342.792517	7.73635E-06	0.009672603
1250.319977	314.670592	7.10168E-06	0.008879369

1250.359977	297.441937	6.71285E-06	0.008393479
1250.399977	290.197303	6.54935E-06	0.008189306
1250.439977	288.285397	6.5062E-06	0.008135612
1250.479977	260.523661	5.87966E-06	0.007352392
1250.519977	213.252858	4.81282E-06	0.006018528
1250.559977	187.614806	4.2342E-06	0.005295127
1250.599977	167.57238	3.78188E-06	0.004729613
1250.639977	163.019043	3.67911E-06	0.004601245
1250.679977	196.645304	4.43801E-06	0.005550531
1250.719977	214.373646	4.83811E-06	0.006051127
1250.759977	209.105546	4.71922E-06	0.005902613
1250.799977	220.338663	4.97274E-06	0.006219899
1250.839977	230.521445	5.20255E-06	0.006507555
1250.879977	239.335618	5.40147E-06	0.006756593
1250.919977	232.762216	5.25312E-06	0.006571232
1250.959977	257.889622	5.82021E-06	0.007280849
1250.999977	272.636771	6.15303E-06	0.007697443
1251.039977	277.485351	6.26246E-06	0.007834585
1251.079977	281.809372	6.36004E-06	0.007956925
1251.119977	265.605839	5.99435E-06	0.007499656
1251.159977	278.815688	6.29248E-06	0.007872901
1251.199977	360.69313	8.14034E-06	0.010185195
1251.239977	433.636856	9.78658E-06	0.01224536
1251.279977	442.650179	9.99E-06	0.012500284
1251.319977	419.890795	9.47635E-06	0.011857947
1251.359977	364.95962	8.23663E-06	0.010306989
1251.399976	288.06487	6.50122E-06	0.00813563
1251.439976	235.901367	5.32397E-06	0.006662623
1251.479976	199.6958	4.50686E-06	0.00564024
1251.519976	181.656239	4.09973E-06	0.005130892
1251.559976	185.733589	4.19175E-06	0.005246225
1251.599976	183.602688	4.14366E-06	0.005186201
1251.639976	169.418985	3.82355E-06	0.004785709
1251.679976	180.724977	4.07871E-06	0.005105241
1251.719976	207.733768	4.68826E-06	0.005868391
1251.759976	192.243093	4.33866E-06	0.005430959
1251.799976	177.195268	3.99905E-06	0.005006011
1251.839976	167.207483	3.77364E-06	0.004723993
1251.879976	172.151614	3.88522E-06	0.004863832
1251.919976	176.950015	3.99352E-06	0.004999562
1251.959976	187.486515	4.23131E-06	0.00529743
1251.999976	214.855156	4.84898E-06	0.006070925
1252.039976	236.06919	5.32775E-06	0.006670559
1252.079976	247.609642	5.5882E-06	0.006996879
1252.119976	251.619783	5.67871E-06	0.007110424
1252.159976	281.367672	6.35008E-06	0.007951312

1252.199976	301.449918	6.8033E-06	0.008519098
1252.239976	316.952737	7.15318E-06	0.0089575
1252.279976	338.319703	7.6354E-06	0.009561664
1252.319976	333.515251	7.52697E-06	0.009426181
1252.359976	312.364491	7.04963E-06	0.008828676
1252.399976	261.781226	5.90804E-06	0.007399226
1252.439976	222.298862	5.01698E-06	0.006283461
1252.479976	209.847186	4.73596E-06	0.005931693
1252.519976	205.603194	4.64018E-06	0.005811915
1252.559976	184.180928	4.15671E-06	0.005206525
1252.599976	187.301746	4.22714E-06	0.005294915
1252.639976	202.110379	4.56135E-06	0.005713729
1252.679976	185.785603	4.19292E-06	0.00525239
1252.719976	177.912823	4.01524E-06	0.005029977
1252.759976	208.89642	4.7145E-06	0.005906138
1252.799976	228.526036	5.15751E-06	0.006461334
1252.839976	229.050157	5.16934E-06	0.00647636
1252.879976	239.367916	5.4022E-06	0.006768309
1252.919976	242.481425	5.47247E-06	0.006856564
1252.959976	233.692063	5.2741E-06	0.006608242
1252.999976	235.41626	5.31302E-06	0.00665721
1253.039976	257.58705	5.81338E-06	0.007284399
1253.079976	274.594105	6.19721E-06	0.007765595
1253.119976	277.977447	6.27356E-06	0.007861528
1253.159976	304.5849	6.87406E-06	0.008614293
1253.199976	351.988845	7.9439E-06	0.009955293
1253.239976	391.599023	8.83784E-06	0.01107594
1253.279976	400.091496	9.02951E-06	0.011316501
1253.319976	393.432861	8.87923E-06	0.011128518
1253.359976	357.276282	8.06323E-06	0.010106127
1253.399976	295.720347	6.674E-06	0.008365187
1253.439976	257.348055	5.80799E-06	0.007279963
1253.479976	239.829671	5.41262E-06	0.006784613
1253.519976	220.391541	4.97393E-06	0.006234921
1253.559976	196.907313	4.44392E-06	0.005570725
1253.599976	205.925842	4.64746E-06	0.005826055
1253.639976	221.79179	5.00553E-06	0.006275135
1253.679976	218.670381	4.93509E-06	0.006187018
1253.719976	219.562136	4.95521E-06	0.006212448
1253.759976	229.962973	5.18994E-06	0.006506944
1253.799976	251.387691	5.67347E-06	0.007113397
1253.839976	260.992215	5.89023E-06	0.007385407
1253.879976	239.810471	5.41219E-06	0.006786235
1253.919976	216.45449	4.88508E-06	0.006125495
1253.959976	208.128384	4.69717E-06	0.00589006
1253.999976	192.665406	4.34819E-06	0.00545263

1254.039976	188.505131	4.2543E-06	0.00533506
1254.079976	213.987967	4.82941E-06	0.006056467
1254.119976	268.074403	6.05007E-06	0.007587508
1254.159976	296.927579	6.70124E-06	0.008404429
1254.199976	307.397287	6.93753E-06	0.008701048
1254.239976	330.345559	7.45544E-06	0.00935091
1254.279976	332.162753	7.49645E-06	0.009402648
1254.319976	300.561923	6.78326E-06	0.008508383
1254.359976	276.395436	6.23786E-06	0.007824522
1254.399976	265.223675	5.98573E-06	0.007508498
1254.439976	243.8304	5.50291E-06	0.006903073
1254.479976	229.819543	5.18671E-06	0.00650662
1254.519976	219.088391	4.94452E-06	0.006202999
1254.559976	204.249736	4.60963E-06	0.00578306
1254.599976	194.84789	4.39745E-06	0.005517035
1254.639976	208.041278	4.6952E-06	0.005890788
1254.679976	216.41079	4.88409E-06	0.00612797
1254.719976	204.954838	4.62555E-06	0.005803764
1254.759976	197.67831	4.46132E-06	0.005597891
1254.799976	183.690384	4.14564E-06	0.005201944
1254.839976	189.009596	4.26568E-06	0.00535275
1254.879976	212.218706	4.78948E-06	0.006010223
1254.919976	244.045713	5.50777E-06	0.006911813
1254.959976	254.580937	5.74554E-06	0.007210419
1254.999976	223.472392	5.04346E-06	0.006329543
1255.039976	218.00509	4.92007E-06	0.006174886
1255.079976	230.388665	5.19955E-06	0.006525853
1255.119976	261.424057	5.89998E-06	0.007405179
1255.159976	304.099219	6.8631E-06	0.008614283
1255.199976	333.903173	7.53573E-06	0.009458848
1255.239976	334.464757	7.5484E-06	0.009475058
1255.279976	323.879903	7.30952E-06	0.009175492
1255.319976	338.061229	7.62957E-06	0.009577553
1255.359976	325.435548	7.34463E-06	0.009220151
1255.399976	277.643052	6.26602E-06	0.007866358
1255.439976	240.879639	5.43632E-06	0.006824971
1255.479976	215.001882	4.85229E-06	0.006091957
1255.519976	187.190378	4.22463E-06	0.005304102
1255.559976	176.105268	3.97445E-06	0.004990161
1255.599976	178.253987	4.02294E-06	0.005051209
1255.639976	168.397108	3.80049E-06	0.004772045
1255.679976	180.812249	4.08068E-06	0.005124029
1255.719976	201.287451	4.54278E-06	0.005704456
1255.759976	193.921021	4.37653E-06	0.005495868
1255.799976	204.422763	4.61354E-06	0.00579368
1255.839976	221.786741	5.00542E-06	0.006286004

1255.879976	205.561736	4.63924E-06	0.005826331
1255.919976	190.310904	4.29505E-06	0.005394242
1255.959976	218.257747	4.92577E-06	0.006186574
1255.999976	254.447364	5.74252E-06	0.007212608
1256.039976	264.34192	5.96583E-06	0.00749332
1256.079976	264.815542	5.97652E-06	0.007506984
1256.119976	261.437728	5.90029E-06	0.007411466
1256.159976	280.59839	6.33271E-06	0.007954903
1256.199976	313.37711	7.07248E-06	0.008884455
1256.239976	329.001401	7.4251E-06	0.009327712
1256.279976	327.040572	7.38085E-06	0.009272414
1256.319976	298.389557	6.73424E-06	0.008460356
1256.359976	288.944868	6.52108E-06	0.008192828
1256.399976	276.796211	6.2469E-06	0.007848611
1256.439976	253.108195	5.7123E-06	0.007177161
1256.479976	244.434355	5.51654E-06	0.006931426
1256.519976	202.501849	4.57018E-06	0.005742528
1256.559976	179.07317	4.04143E-06	0.005078302
1256.599976	184.744083	4.16942E-06	0.005239289
1256.639976	186.267049	4.20379E-06	0.005282648
1256.679976	181.639753	4.09936E-06	0.005151579
1256.719976	184.20692	4.15729E-06	0.005224554
1256.759976	193.807327	4.37396E-06	0.00549702
1256.799976	195.683727	4.41631E-06	0.005550417
1256.839976	194.362278	4.38649E-06	0.005513111
1256.879976	194.791591	4.39617E-06	0.005525464
1256.919976	197.750051	4.46294E-06	0.005609562
1256.959976	210.795962	4.75737E-06	0.005979825
1256.999976	257.498003	5.81137E-06	0.007304894
1257.039976	295.709233	6.67375E-06	0.008389165
1257.079976	297.333957	6.71041E-06	0.008435526
1257.119976	303.465363	6.84879E-06	0.008609751
1257.159976	321.089252	7.24654E-06	0.009110057
1257.199976	343.36706	7.74932E-06	0.00974244
1257.239976	355.082576	8.01372E-06	0.010075168
1257.279976	351.888474	7.94163E-06	0.009984856
1257.319976	334.624484	7.55201E-06	0.009495291
1257.359976	308.683258	6.96655E-06	0.008759463
1257.399976	292.673995	6.60524E-06	0.008305434
1257.439976	282.610157	6.37812E-06	0.0080201
1257.479976	253.578757	5.72292E-06	0.007196456
1257.519976	214.556435	4.84224E-06	0.006089213
1257.559976	173.510482	3.91589E-06	0.004924466
1257.599976	139.403898	3.14615E-06	0.0039566
1257.639976	144.746737	3.26673E-06	0.004108373
1257.679976	159.445922	3.59847E-06	0.004525727

1257.719976	181.8083	4.10316E-06	0.005160626
1257.759976	203.214397	4.58627E-06	0.005768422
1257.799976	242.721363	5.47788E-06	0.006890081
1257.839976	249.55045	5.63201E-06	0.007084162
1257.879976	201.853897	4.55556E-06	0.005730349
1257.919976	169.02747	3.81471E-06	0.004798606
1257.959976	186.996896	4.22026E-06	0.005308918
1257.999976	228.861097	5.16508E-06	0.006497666
1258.039976	237.253017	5.35447E-06	0.006736137
1258.079976	223.266805	5.03882E-06	0.00633924
1258.119976	226.040316	5.10142E-06	0.006418192
1258.159976	232.595494	5.24936E-06	0.00660453
1258.199976	266.807044	6.02146E-06	0.007576205
1258.239976	315.226798	7.11423E-06	0.008951408
1258.279976	313.988068	7.08627E-06	0.008916516
1258.319976	308.030663	6.95182E-06	0.008747618
1258.359976	290.455206	6.55517E-06	0.008248763
1258.399976	249.404188	5.62871E-06	0.007083162
1258.439976	223.278304	5.03908E-06	0.00634138
1258.479976	201.962769	4.55802E-06	0.005736175
1258.519976	184.542891	4.16488E-06	0.00524158
1258.559976	171.734616	3.87581E-06	0.004877941
1258.599976	165.581348	3.73694E-06	0.004703313
1258.639976	162.972993	3.67807E-06	0.00462937
1258.679976	170.908601	3.85717E-06	0.004854941
1258.719976	187.09659	4.22251E-06	0.005314957
1258.759976	210.678144	4.75471E-06	0.005985041
1258.799976	226.530682	5.11248E-06	0.006435592
1258.839976	220.954806	4.98664E-06	0.006277385
1258.879976	223.948753	5.05421E-06	0.006362646
1258.919976	239.487899	5.40491E-06	0.006804347
1258.959976	241.589565	5.45234E-06	0.006864278
1258.999976	239.349059	5.40177E-06	0.006800834
1259.039976	239.03204	5.39462E-06	0.006792042
1259.079976	241.584997	5.45224E-06	0.006864802
1259.119976	267.171252	6.02968E-06	0.007592094
1259.159976	285.862457	6.45152E-06	0.008123493
1259.199976	331.678874	7.48553E-06	0.009425779
1259.239976	352.280923	7.95049E-06	0.010011575
1259.279976	354.824734	8.0079E-06	0.010084188
1259.319976	330.831671	7.46641E-06	0.009402599
1259.359976	300.142539	6.7738E-06	0.008530651
1259.399976	277.351356	6.25943E-06	0.007883131
1259.439976	247.668668	5.58954E-06	0.007039686
1259.479976	202.814165	4.57723E-06	0.005764934
1259.519976	176.75359	3.98908E-06	0.005024329

1259.559976	183.645676	4.14463E-06	0.005220406
1259.599976	185.739149	4.19187E-06	0.005280084
1259.639976	176.943001	3.99336E-06	0.005030192
1259.679976	188.732648	4.25943E-06	0.005365522
1259.719976	194.175856	4.38228E-06	0.005520444
1259.759976	193.042447	4.3567E-06	0.005488395
1259.799976	188.635304	4.25724E-06	0.005363266
1259.839976	183.69438	4.14573E-06	0.005222952
1259.879976	190.49976	4.29931E-06	0.00541662
1259.919976	194.272926	4.38447E-06	0.005524081
1259.959976	176.047735	3.97315E-06	0.005006013
1259.999976	183.701181	4.14588E-06	0.005223808
1260.039976	222.510237	5.02175E-06	0.006327601
1260.079976	253.981799	5.73202E-06	0.007222798
1260.119976	268.578968	6.06145E-06	0.007638158
1260.159976	279.438739	6.30654E-06	0.007947253
1260.199976	308.48848	6.96216E-06	0.008773708
1260.239976	321.016344	7.24489E-06	0.009130302
1260.279976	294.056736	6.63645E-06	0.008363786
1260.319976	259.249454	5.8509E-06	0.007374005
1260.359976	244.268518	5.5128E-06	0.006948113
1260.399976	247.251933	5.58013E-06	0.007033198
1260.439976	224.104401	5.05772E-06	0.006374958
1260.479976	192.539634	4.34535E-06	0.005477228
1260.519976	185.168607	4.179E-06	0.00526771
1260.559976	189.560279	4.27811E-06	0.005392816
1260.599976	192.17012	4.33701E-06	0.005467237
1260.639976	197.775798	4.46352E-06	0.005626897
1260.679976	203.676373	4.59669E-06	0.005794958
1260.719976	218.301481	4.92676E-06	0.006211265
1260.759976	230.611159	5.20457E-06	0.006561717
1260.799976	233.882657	5.27841E-06	0.006655014
1260.839976	210.686275	4.7549E-06	0.005995163
1260.879976	200.842762	4.53274E-06	0.005715243
1260.919976	210.840651	4.75838E-06	0.005999936
1260.959976	219.823367	4.96111E-06	0.006255758
1260.999976	231.070227	5.21493E-06	0.006576031
1261.039976	245.303932	5.53617E-06	0.006981329
1261.079976	261.501874	5.90173E-06	0.007442557
1261.119976	284.202782	6.41406E-06	0.0080889
1261.159976	315.506346	7.12054E-06	0.008980138
1261.199976	343.408876	7.75026E-06	0.009774628
1261.239976	351.634785	7.93591E-06	0.010009084
1261.279976	335.695794	7.57619E-06	0.009555692
1261.319976	315.723143	7.12543E-06	0.008987449
1261.359976	315.160695	7.11274E-06	0.008971723

1261.399976	310.134429	6.9993E-06	0.008828919
1261.439976	262.719126	5.9292E-06	0.007479336
1261.479976	235.391476	5.31246E-06	0.006701559
1261.519976	227.053287	5.12428E-06	0.006464377
1261.559976	223.146982	5.03612E-06	0.006353363
1261.599976	222.892815	5.03038E-06	0.006346328
1261.639976	220.107259	4.96751E-06	0.006267215
1261.679976	219.980944	4.96466E-06	0.006263817
1261.719976	220.735094	4.98168E-06	0.00628549
1261.759976	224.358403	5.06346E-06	0.006388867
1261.799976	218.849244	4.93912E-06	0.006232185
1261.839976	188.923061	4.26373E-06	0.005380145
1261.879976	189.505915	4.27688E-06	0.005396915
1261.919976	189.721972	4.28176E-06	0.005403239
1261.959976	178.98328	4.0394E-06	0.005097565
1261.999976	206.754161	4.66615E-06	0.005888686
1262.039976	220.44036	4.97503E-06	0.006278689
1262.079976	230.738242	5.20744E-06	0.006572207
1262.119976	254.018113	5.73283E-06	0.007235526
1262.159976	278.768813	6.29142E-06	0.007940783
1262.199976	297.624504	6.71697E-06	0.00847816
1262.239976	323.005685	7.28979E-06	0.009201462
1262.279976	346.991029	7.8311E-06	0.009885046
1262.319976	347.774968	7.8488E-06	0.009907693
1262.359976	299.22477	6.75309E-06	0.008524826
1262.399976	243.332608	5.49168E-06	0.006932694
1262.439976	238.671355	5.38648E-06	0.006800108
1262.479976	236.978758	5.34828E-06	0.006752097
1262.519976	208.832103	4.71305E-06	0.005950319
1262.559976	189.667567	4.28053E-06	0.005404429
1262.599976	208.323677	4.70158E-06	0.005936209
1262.639976	212.178059	4.78856E-06	0.006046231
1262.679976	178.974176	4.0392E-06	0.005100214
1262.719976	175.546304	3.96184E-06	0.005002689
1262.759976	186.030404	4.19845E-06	0.005301631
1262.799976	200.016665	4.5141E-06	0.005700402
1262.839976	215.859982	4.87166E-06	0.006152126
1262.879976	210.156147	4.74293E-06	0.005989753
1262.919976	223.843648	5.05184E-06	0.006380069
1262.959976	248.048165	5.5981E-06	0.007070178
1262.999976	264.330954	5.96558E-06	0.007534529
1263.039976	276.577861	6.24198E-06	0.007883867
1263.079976	300.719242	6.78681E-06	0.008572289
1263.119976	330.0064	7.44778E-06	0.009407446
1263.159976	368.978851	8.32734E-06	0.010518761
1263.199976	420.45892	9.48917E-06	0.011986722

1263.239976	417.604097	9.42474E-06	0.011905712
1263.279976	385.350441	8.69682E-06	0.010986522
1263.319976	339.584832	7.66396E-06	0.00968203
1263.359976	299.506843	6.75945E-06	0.008539621
1263.399976	278.922542	6.29489E-06	0.007952968
1263.439976	251.914434	5.68536E-06	0.007183108
1263.479976	246.063817	5.55332E-06	0.007016506
1263.519976	223.901202	5.05314E-06	0.006384741
1263.559976	190.854824	4.30733E-06	0.005442567
1263.599976	179.425011	4.04937E-06	0.005116787
1263.639976	198.574277	4.48154E-06	0.005663059
1263.679976	212.851205	4.80376E-06	0.006070409
1263.719976	201.438371	4.54618E-06	0.005745103
1263.759976	197.146239	4.44932E-06	0.005622867
1263.799976	222.26707	5.01626E-06	0.006339547
1263.839976	244.187989	5.51098E-06	0.006965
1263.879976	235.725966	5.32001E-06	0.00672385
1263.919976	226.292973	5.10712E-06	0.006454987
1263.959976	230.666518	5.20582E-06	0.006579951
1263.999976	236.500531	5.33749E-06	0.006746584
1264.039976	227.887507	5.1431E-06	0.006501089
1264.079976	237.551555	5.36121E-06	0.006776995
1264.119976	284.468591	6.42006E-06	0.008115726
1264.159976	318.220418	7.18179E-06	0.009078933
1264.199976	351.77671	7.93911E-06	0.010036623
1264.239976	369.635872	8.34217E-06	0.010546501
1264.279976	346.034722	7.80952E-06	0.009873422
1264.319976	329.083737	7.42696E-06	0.009390056
1264.359976	324.246923	7.3178E-06	0.009252335
1264.399976	275.810974	6.22467E-06	0.007870472
1264.439976	235.001217	5.30365E-06	0.006706147
1264.479976	214.751268	4.84664E-06	0.006128475
1264.519976	196.917075	4.44414E-06	0.005619709
1264.559976	186.281856	4.20412E-06	0.005316364
1264.599976	186.172576	4.20166E-06	0.005313414
1264.639976	197.644049	4.46055E-06	0.005640991
1264.679976	216.948907	4.89623E-06	0.00619217
1264.719976	220.490319	4.97616E-06	0.006293448
1264.759976	211.845284	4.78105E-06	0.006046884
1264.799976	233.120429	5.2612E-06	0.00665437
1264.839976	254.177317	5.73643E-06	0.007255663
1264.879976	266.141626	6.00645E-06	0.007597433
1264.919976	266.614347	6.01711E-06	0.007611168
1264.959976	241.40077	5.44808E-06	0.006891602
1264.999976	226.503729	5.11187E-06	0.00646652
1265.039976	253.252933	5.71557E-06	0.007230419

1265.079976	295.825674	6.67637E-06	0.008446146
1265.119976	321.816531	7.26295E-06	0.009188504
1265.159976	325.005831	7.33493E-06	0.009279858
1265.199976	332.174668	7.49672E-06	0.009484849
1265.239976	334.721397	7.5542E-06	0.00955787
1265.279976	326.72155	7.37365E-06	0.009329732
1265.319976	326.637198	7.37175E-06	0.009327618
1265.359976	345.048121	7.78726E-06	0.009853681
1265.399976	303.952821	6.85979E-06	0.00868038
1265.439976	214.893049	4.84984E-06	0.006137177
1265.479976	166.057042	3.74768E-06	0.004742609
1265.519976	160.482899	3.62188E-06	0.004583556
1265.559976	161.878921	3.65338E-06	0.004623574
1265.599976	168.176297	3.7955E-06	0.004803591
1265.639976	181.051043	4.08607E-06	0.005171493
1265.679976	183.097599	4.13226E-06	0.005230116
1265.719976	187.198702	4.22481E-06	0.005347431
1265.759976	191.917827	4.33132E-06	0.005482409
1265.799976	214.091183	4.83174E-06	0.006116016
1265.839976	221.694786	5.00334E-06	0.006333431
1265.879976	207.899835	4.69201E-06	0.005939521
1265.919976	201.351739	4.54423E-06	0.005752629
1265.959976	218.947038	4.94133E-06	0.006255526
1265.999976	240.734662	5.43305E-06	0.006878236
1266.039976	240.167118	5.42024E-06	0.006862237
1266.079976	246.818264	5.57034E-06	0.007052502
1266.119976	280.035147	6.32E-06	0.008001882
1266.159976	292.483219	6.60094E-06	0.008357845
1266.199976	282.667285	6.37941E-06	0.008077605
1266.239976	311.453391	7.02907E-06	0.008900488
1266.279976	352.309969	7.95115E-06	0.010068376
1266.319976	330.795045	7.46558E-06	0.009453817
1266.359976	297.344362	6.71065E-06	0.008498096
1266.399976	273.81423	6.17961E-06	0.007825853
1266.439976	249.1731	5.62349E-06	0.007121812
1266.479976	210.040583	4.74032E-06	0.006003525
1266.519976	181.413758	4.09426E-06	0.005185457
1266.559976	173.903299	3.92476E-06	0.004970938
1266.599976	176.119584	3.97477E-06	0.005034448
1266.639976	166.342267	3.75411E-06	0.00475511
1266.679976	172.452103	3.892E-06	0.004929923
1266.719976	189.483349	4.27638E-06	0.00541697
1266.759976	211.866764	4.78154E-06	0.006057061
1266.799976	224.478184	5.06616E-06	0.006417811
1266.839976	216.998908	4.89736E-06	0.006204175
1266.879976	207.498717	4.68296E-06	0.005932744

1266.919976	222.685683	5.02571E-06	0.006367167
1266.959976	236.800521	5.34426E-06	0.006770961
1266.999976	236.907856	5.34668E-06	0.006774244
1267.039976	226.75796	5.11761E-06	0.006484218
1267.079976	230.639449	5.20521E-06	0.006595419
1267.119976	263.700438	5.95135E-06	0.007541076
1267.159976	309.79962	6.99175E-06	0.008859661
1267.199976	321.637536	7.25891E-06	0.009198492
1267.239976	326.777269	7.37491E-06	0.009345778
1267.279976	344.787715	7.78138E-06	0.009861185
1267.319976	358.180895	8.08364E-06	0.010244563
1267.359976	342.891792	7.73859E-06	0.009807579
1267.399976	313.037062	7.06481E-06	0.008953941
1267.439976	288.135459	6.50282E-06	0.008241929
1267.479976	241.521862	5.45081E-06	0.006908795
1267.519976	204.915121	4.62465E-06	0.005861835
1267.559976	197.579465	4.45909E-06	0.005652168
1267.599976	186.706611	4.21371E-06	0.005341296
1267.639976	166.608902	3.76013E-06	0.004766492
1267.679976	177.122212	3.9974E-06	0.005067426
1267.719976	179.119033	4.04247E-06	0.005124716
1267.759976	175.223799	3.95456E-06	0.005013429
1267.799976	184.40403	4.16174E-06	0.005276257
1267.839976	193.002724	4.3558E-06	0.005522461
1267.879976	197.039546	4.44691E-06	0.005638146
1267.919976	191.505976	4.32202E-06	0.005479979
1267.959976	192.328611	4.34059E-06	0.005503693
1267.999976	230.384989	5.19947E-06	0.006592926
1268.039976	248.104717	5.59938E-06	0.007100235
1268.079976	234.624002	5.29514E-06	0.006714657
1268.119976	250.277409	5.64841E-06	0.007162865
1268.159976	291.323849	6.57477E-06	0.008337865
1268.199976	323.493486	7.3008E-06	0.009258871
1268.239976	315.125951	7.11195E-06	0.009019664
1268.279976	326.352723	7.36533E-06	0.009341296
1268.319976	319.755206	7.21643E-06	0.009152742
1268.359976	288.559545	6.51239E-06	0.008260051
1268.399976	249.163329	5.62327E-06	0.007132555
1268.439976	225.833363	5.09674E-06	0.006464914
1268.479976	218.164415	4.92367E-06	0.006245573
1268.519976	209.781743	4.73448E-06	0.006005785
1268.559976	192.947158	4.35455E-06	0.005524006
1268.599976	196.690191	4.43902E-06	0.005631345
1268.639976	203.402982	4.59052E-06	0.00582372
1268.679976	198.698803	4.48436E-06	0.005689212
1268.719976	214.692781	4.84532E-06	0.00614735

1268.759976	223.062825	5.03422E-06	0.006387213
1268.799976	217.511665	4.90894E-06	0.006228457
1268.839976	215.177468	4.85626E-06	0.006161811
1268.879976	215.94454	4.87357E-06	0.006183972
1268.919976	216.225074	4.8799E-06	0.006192201
1268.959976	215.547914	4.86462E-06	0.006173003
1268.999976	227.014663	5.1234E-06	0.0065016
1269.039976	224.113781	5.05794E-06	0.006418723
1269.079976	244.31536	5.51386E-06	0.006997526
1269.119976	279.724618	6.313E-06	0.008011948
1269.159976	297.177416	6.70688E-06	0.008512104
1269.199976	307.311593	6.93559E-06	0.008802657
1269.239976	330.012287	7.44792E-06	0.009453195
1269.279976	330.877775	7.46745E-06	0.009478285
1269.319976	309.76103	6.99087E-06	0.008873657
1269.359976	275.740444	6.22308E-06	0.007899326
1269.399976	248.145245	5.60029E-06	0.007109011
1269.439976	232.495487	5.2471E-06	0.006660878
1269.479976	230.574796	5.20375E-06	0.006606059
1269.519976	219.296328	4.94921E-06	0.006283124
1269.559976	195.031909	4.4016E-06	0.005588093
1269.599976	175.095045	3.95165E-06	0.005017016
1269.639976	170.960787	3.85835E-06	0.004898711
1269.679976	168.876711	3.81131E-06	0.004839147
1269.719976	181.157593	4.08847E-06	0.005191218
1269.759976	192.535336	4.34525E-06	0.00551743
1269.799976	188.82927	4.26161E-06	0.005411397
1269.839976	174.584159	3.94012E-06	0.005003324
1269.879976	159.790211	3.60624E-06	0.004579495
1269.919976	160.222182	3.61599E-06	0.00459202
1269.959976	173.341083	3.91207E-06	0.004968168
1269.999976	192.116985	4.33581E-06	0.005506482
1270.039976	216.950219	4.89626E-06	0.006218451
1270.079976	231.90522	5.23378E-06	0.006647316
1270.119976	238.162437	5.37499E-06	0.006826888
1270.159976	263.659	5.95042E-06	0.007557981
1270.199976	297.27252	6.70903E-06	0.008521806
1270.239976	307.051247	6.92972E-06	0.008802406
1270.279976	294.953753	6.6567E-06	0.008455867
1270.319976	269.294507	6.0776E-06	0.007720499
1270.359976	269.922762	6.09178E-06	0.007738754
1270.399976	276.297734	6.23566E-06	0.007921776
1270.439976	270.246415	6.09909E-06	0.007748522
1270.479976	254.201078	5.73696E-06	0.007288698
1270.519976	224.073982	5.05704E-06	0.006425067
1270.559976	202.037852	4.55971E-06	0.005793389

1270.599976	179.768106	4.05712E-06	0.005154971
1270.639976	194.833831	4.39713E-06	0.005587167
1270.679976	214.937419	4.85084E-06	0.006163863
1270.719976	201.006184	4.53643E-06	0.005764532
1270.759976	184.077711	4.15438E-06	0.005279217
1270.799976	203.63385	4.59573E-06	0.005840257
1270.839976	226.143855	5.10375E-06	0.006486052
1270.879976	223.731366	5.04931E-06	0.006417061
1270.919976	222.563659	5.02295E-06	0.00638377
1270.959976	240.033355	5.41722E-06	0.006885068
1270.999976	228.671619	5.1608E-06	0.006559377
1271.039976	232.203601	5.24051E-06	0.0066609
1271.079976	277.120336	6.25422E-06	0.007949614
1271.119976	314.406607	7.09572E-06	0.00901951
1271.159976	342.575881	7.73146E-06	0.009827923
1271.199976	372.759567	8.41266E-06	0.010694179
1271.239976	393.518154	8.88116E-06	0.011290081
1271.279976	396.278445	8.94345E-06	0.011369632
1271.319976	405.918199	9.16101E-06	0.011646573
1271.359976	381.176732	8.60263E-06	0.010937037
1271.399976	339.290956	7.65732E-06	0.009735522
1271.439976	278.48807	6.28509E-06	0.007991112
1271.479976	226.070002	5.10209E-06	0.006487199
1271.519976	215.383731	4.86091E-06	0.006180745
1271.559976	211.889274	4.78205E-06	0.006080658
1271.599976	194.755129	4.39535E-06	0.005589129
1271.639976	181.710359	4.10095E-06	0.005214932
1271.679976	171.137791	3.86234E-06	0.004911662
1271.719976	184.82975	4.17135E-06	0.005304789
1271.759976	200.65103	4.52841E-06	0.005759056
1271.799976	209.392165	4.72569E-06	0.006010132
1271.839976	221.622545	5.00171E-06	0.006361377
1271.879976	210.80507	4.75758E-06	0.006051067
1271.919976	168.135273	3.79458E-06	0.004826401
1271.959976	160.591759	3.62433E-06	0.004610006
1271.999976	190.128272	4.29093E-06	0.005458063
1272.039976	214.2519	4.83537E-06	0.00615078
1272.079976	225.275038	5.08414E-06	0.006467438
1272.119976	246.557878	5.56447E-06	0.007078671
1272.159976	270.476536	6.10428E-06	0.007765619
1272.199976	286.415555	6.464E-06	0.008223501
1272.239976	299.199706	6.75252E-06	0.008590827
1272.279976	312.246048	7.04696E-06	0.008965704
1272.319976	329.005925	7.42521E-06	0.009447237
1272.359976	313.889419	7.08405E-06	0.009013458
1272.399976	279.68877	6.31219E-06	0.008031625

1272.439976	240.864694	5.43598E-06	0.006916959
1272.479976	228.85941	5.16504E-06	0.006572408
1272.519976	213.461646	4.81753E-06	0.006130406
1272.559976	191.43847	4.3205E-06	0.005498095
1272.599976	171.130009	3.86217E-06	0.004914992
1272.639976	169.296348	3.82078E-06	0.004862481
1272.679976	181.500926	4.09622E-06	0.005213181
1272.719976	217.837236	4.91628E-06	0.006257052
1272.759976	229.022604	5.16872E-06	0.006578541
1272.799976	191.450083	4.32076E-06	0.005499465
1272.839976	190.748956	4.30494E-06	0.005479497
1272.879976	240.279094	5.42276E-06	0.006902528
1272.919976	244.674358	5.52196E-06	0.007029012
1272.959976	219.374705	4.95098E-06	0.006302401
1272.999976	212.072496	4.78618E-06	0.006092808
1273.039976	232.91361	5.25654E-06	0.00669178
1273.079976	261.023046	5.89093E-06	0.007499621
1273.119976	291.334196	6.57501E-06	0.008370773
1273.159976	331.331415	7.47769E-06	0.009520294
1273.199976	359.734154	8.1187E-06	0.010336727
1273.239976	365.514044	8.24914E-06	0.010503138
1273.279976	348.524869	7.86572E-06	0.010015265
1273.319976	335.266017	7.56649E-06	0.009634559
1273.359976	327.619386	7.39391E-06	0.009415113
1273.399976	318.113818	7.17939E-06	0.00914223
1273.439976	294.351828	6.64311E-06	0.008459603
1273.479976	268.492652	6.05951E-06	0.007716659
1273.519976	243.719169	5.5004E-06	0.007004872
1273.559976	228.13138	5.14861E-06	0.00655706
1273.599976	218.290775	4.92652E-06	0.006274414
1273.639976	200.301533	4.52053E-06	0.005757523
1273.679976	186.751702	4.21473E-06	0.005368212
1273.719976	184.227035	4.15775E-06	0.005295806
1273.759976	178.73727	4.03385E-06	0.005138158
1273.799976	195.508435	4.41235E-06	0.005620455
1273.839976	210.701363	4.75524E-06	0.00605741
1273.879976	194.844341	4.39737E-06	0.005601716
1273.919976	164.913838	3.72188E-06	0.004741372
1273.959976	165.890948	3.74393E-06	0.004769614
1273.999976	205.607602	4.64028E-06	0.005911713
1274.039976	224.853034	5.07462E-06	0.006465268
1274.079976	220.259932	4.97096E-06	0.0063334
1274.119976	232.046289	5.23696E-06	0.006672517
1274.159976	248.655037	5.6118E-06	0.007150328
1274.199976	288.155572	6.50327E-06	0.008286466
1274.239976	330.657596	7.46248E-06	0.009508992

1274.279976	355.146626	8.01516E-06	0.010213564
1274.319976	349.513725	7.88804E-06	0.010051884
1274.359976	317.086413	7.1562E-06	0.009119573
1274.399976	261.92697	5.91133E-06	0.007533395
1274.439976	220.095429	4.96725E-06	0.006330458
1274.479976	185.120022	4.1779E-06	0.005324651
1274.519976	177.119927	3.99735E-06	0.005094702
1274.559976	172.8628	3.90127E-06	0.004972406
1274.599976	168.404532	3.80066E-06	0.004844316
1274.639976	173.911209	3.92493E-06	0.005002877
1274.679976	187.461357	4.23074E-06	0.005392842
1274.719976	197.038151	4.44688E-06	0.005668522
1274.759976	198.112685	4.47113E-06	0.005699614
1274.799976	196.548123	4.43582E-06	0.00565478
1274.839976	187.001157	4.22036E-06	0.005380278
1274.879976	181.655303	4.09971E-06	0.005226635
1274.919976	168.000354	3.79153E-06	0.004833902
1274.959976	172.383107	3.89045E-06	0.004960164
1274.999976	219.392295	4.95138E-06	0.006313007
1275.039976	260.044506	5.86884E-06	0.007483009
1275.079976	271.387894	6.12485E-06	0.007809669
1275.119976	273.50781	6.17269E-06	0.007870921
1275.159976	290.95074	6.56635E-06	0.00837315
1275.199976	290.136432	6.54798E-06	0.008349978
1275.239976	306.625996	6.92012E-06	0.008824816
1275.279976	342.152016	7.72189E-06	0.009847577
1275.319976	364.658553	8.22984E-06	0.010495674
1275.359976	326.455002	7.36763E-06	0.009396386
1275.399976	304.773071	6.8783E-06	0.008772588
1275.439976	296.336964	6.68791E-06	0.008530031
1275.479976	252.977144	5.70934E-06	0.007282151
1275.519976	197.851094	4.46522E-06	0.005695482
1275.559976	154.089825	3.47759E-06	0.004435878
1275.599976	133.680527	3.01698E-06	0.003848464
1275.639976	148.100774	3.34243E-06	0.004263735
1275.679976	168.704283	3.80742E-06	0.00485705
1275.719976	181.00796	4.0851E-06	0.005211441
1275.759976	192.972523	4.35512E-06	0.005556089
1275.799976	201.196319	4.54072E-06	0.005793051
1275.839976	198.734818	4.48517E-06	0.005722357
1275.879976	192.721962	4.34947E-06	0.005549397
1275.919976	199.240306	4.49658E-06	0.005737271
1275.959976	212.500866	4.79585E-06	0.006119311
1275.999976	216.159062	4.87841E-06	0.00622485
1276.039976	195.713012	4.41697E-06	0.00563623
1276.079976	179.404287	4.0489E-06	0.005166726

1276.119976	197.251438	4.45169E-06	0.005680891
1276.159976	226.720497	5.11677E-06	0.006529812
1276.199976	270.141099	6.09671E-06	0.007780619
1276.239976	285.549344	6.44445E-06	0.008224666
1276.279976	271.548867	6.12848E-06	0.007821656
1276.319976	260.305142	5.87472E-06	0.007498028
1276.359976	243.865792	5.50371E-06	0.007024717
1276.399976	230.048624	5.19188E-06	0.006626912
1276.439976	216.609916	4.88858E-06	0.006239984
1276.479976	208.503223	4.70563E-06	0.006006639
1276.519976	214.476868	4.84044E-06	0.006178924
1276.559976	210.067318	4.74093E-06	0.006052077
1276.599976	198.155891	4.4721E-06	0.005709086
1276.639976	202.923082	4.57969E-06	0.005846617
1276.679976	212.9483	4.80595E-06	0.006135656
1276.719976	225.724346	5.09428E-06	0.006503974
1276.759976	229.796021	5.18618E-06	0.006621502
1276.799976	217.20456	4.902E-06	0.006258879
1276.839976	194.163669	4.382E-06	0.005595117
1276.879976	202.417794	4.56829E-06	0.005833155
1276.919976	236.164008	5.32989E-06	0.006805846
1276.959976	267.782141	6.04347E-06	0.007717269
1276.999976	281.66016	6.35668E-06	0.008117477
1277.039976	275.580292	6.21946E-06	0.007942503
1277.079976	273.746841	6.17808E-06	0.007889909
1277.119976	279.159058	6.30023E-06	0.008046151
1277.159976	284.966751	6.4313E-06	0.008213802
1277.199976	285.117469	6.4347E-06	0.008218404
1277.239976	283.796831	6.4049E-06	0.008180593
1277.279976	283.301376	6.39372E-06	0.008166567
1277.319976	289.333467	6.52985E-06	0.008340712
1277.359976	264.881204	5.978E-06	0.007636058
1277.399976	228.050853	5.14679E-06	0.00657451
1277.439976	205.221787	4.63157E-06	0.005916553
1277.479976	193.077568	4.35749E-06	0.005566608
1277.519976	187.157589	4.22389E-06	0.005396099
1277.559976	177.102627	3.99696E-06	0.005106356
1277.599976	185.350156	4.18309E-06	0.005344322
1277.639976	192.321755	4.34043E-06	0.005545512
1277.679976	192.728382	4.34961E-06	0.005557411
1277.719976	190.725037	4.3044E-06	0.005499816
1277.759976	181.151421	4.08834E-06	0.005223911
1277.799976	180.873374	4.08206E-06	0.005216056
1277.839976	200.550543	4.52615E-06	0.005783691
1277.879976	209.932956	4.73789E-06	0.00605446
1277.919976	204.562775	4.6167E-06	0.005899769

1277.959976	202.16645	4.56262E-06	0.00583084
1277.999976	211.033112	4.76272E-06	0.00608676
1278.039976	216.007495	4.87499E-06	0.00623043
1278.079976	238.091736	5.3734E-06	0.006867633
1278.119976	239.834528	5.41273E-06	0.00691812
1278.159976	235.965452	5.32541E-06	0.006806728
1278.199976	257.722307	5.81643E-06	0.007434565
1278.239976	278.415599	6.28345E-06	0.00803176
1278.279976	292.522294	6.60182E-06	0.008438975
1278.319976	286.869662	6.47425E-06	0.008276161
1278.359976	262.60671	5.92667E-06	0.007576415
1278.399976	246.206611	5.55654E-06	0.007103481
1278.439976	243.932067	5.50521E-06	0.007038077
1278.479976	230.15529	5.19428E-06	0.006640788
1278.519976	223.60277	5.0464E-06	0.006451927
1278.559976	202.739218	4.57554E-06	0.005850105
1278.599976	179.476804	4.05054E-06	0.005179022
1278.639976	172.046864	3.88286E-06	0.004964778
1278.679976	188.953958	4.26443E-06	0.005452838
1278.719976	214.881736	4.84958E-06	0.006201257
1278.759976	209.561384	4.72951E-06	0.006047906
1278.799976	198.381655	4.4772E-06	0.00572544
1278.839976	193.915301	4.3764E-06	0.005596713
1278.879976	199.4649	4.50164E-06	0.005757064
1278.919976	208.886613	4.71428E-06	0.006029187
1278.959976	217.303317	4.90423E-06	0.006272318
1278.999976	223.793899	5.05072E-06	0.006459866
1279.039976	207.750369	4.68864E-06	0.005996953
1279.079976	230.969875	5.21267E-06	0.00666742
1279.119976	263.053748	5.93676E-06	0.007593824
1279.159976	291.050037	6.56859E-06	0.008402282
1279.199976	338.833236	7.64699E-06	0.009782035
1279.239976	366.541215	8.27232E-06	0.010582288
1279.279976	360.200079	8.12921E-06	0.010399541
1279.319976	366.279048	8.26641E-06	0.010575381
1279.359976	318.250575	7.18247E-06	0.009188967
1279.399976	260.003844	5.86792E-06	0.007507423
1279.439976	230.960785	5.21246E-06	0.006669034
1279.479976	206.068796	4.65069E-06	0.005950459
1279.519976	196.643092	4.43796E-06	0.005678459
1279.559976	189.722218	4.28177E-06	0.005478777
1279.599976	190.629681	4.30225E-06	0.005505154
1279.639976	186.138292	4.20088E-06	0.005375617
1279.679976	184.781433	4.17026E-06	0.005336598
1279.719976	181.34161	4.09263E-06	0.005237417
1279.759976	182.025845	4.10807E-06	0.005257343

1279.799976	205.836353	4.64544E-06	0.005945234
1279.839976	221.996843	5.01016E-06	0.006412202
1279.879976	207.193022	4.67606E-06	0.005984793
1279.919976	199.208134	4.49585E-06	0.005754328
1279.959976	201.900698	4.55662E-06	0.005832288
1279.999976	196.339846	4.43112E-06	0.005671829
1280.039976	210.863303	4.75889E-06	0.006091571
1280.079976	221.610133	5.00143E-06	0.006402233
1280.119976	232.734301	5.25249E-06	0.006723816
1280.159976	275.059874	6.20772E-06	0.007946872
1280.199976	293.546056	6.62493E-06	0.00848123
1280.239976	283.961837	6.40862E-06	0.008204576
1280.279976	316.785459	7.14941E-06	0.009153242
1280.319976	326.237968	7.36274E-06	0.009426659
1280.359976	279.320695	6.30388E-06	0.008071234
1280.399976	231.387989	5.2221E-06	0.006686383
1280.439976	224.814304	5.07375E-06	0.006496627
1280.479976	214.953446	4.8512E-06	0.006211864
1280.519976	203.287312	4.58791E-06	0.005874912
1280.559976	192.391008	4.342E-06	0.005560188
1280.599976	186.836354	4.21664E-06	0.005399824
1280.639976	190.785656	4.30577E-06	0.005514137
1280.679976	210.823996	4.758E-06	0.00609348
1280.719976	217.740658	4.9141E-06	0.00629359
1280.759976	201.970818	4.5582E-06	0.00583796
1280.799976	195.588823	4.41417E-06	0.005653665
1280.839976	182.410025	4.11674E-06	0.005272885
1280.879976	179.299125	4.04653E-06	0.005183121
1280.919976	185.740769	4.19191E-06	0.005369502
1280.959976	184.035005	4.15341E-06	0.005320357
1280.999976	183.913492	4.15067E-06	0.00531701
1281.039976	194.467303	4.38886E-06	0.0056223
1281.079976	215.72285	4.86856E-06	0.00623702
1281.119976	258.752578	5.83969E-06	0.007481337
1281.159976	298.46564	6.73595E-06	0.008629834
1281.199976	313.623621	7.07805E-06	0.009068395
1281.239976	322.390793	7.27591E-06	0.009322188
1281.279976	312.838749	7.06033E-06	0.009046266
1281.319976	290.342687	6.55263E-06	0.008396016
1281.359976	252.205647	5.69193E-06	0.007293411
1281.399976	240.962486	5.43819E-06	0.006968494
1281.439976	219.38033	4.95111E-06	0.006344548
1281.479976	207.795442	4.68965E-06	0.006009697
1281.519976	211.50842	4.77345E-06	0.006117272
1281.559976	214.811698	4.848E-06	0.006213004
1281.599976	211.044842	4.76299E-06	0.006104245

1281.639976	199.128537	4.49405E-06	0.005759759
1281.679976	195.556808	4.41344E-06	0.005656624
1281.719976	210.526026	4.75128E-06	0.006089809
1281.759976	205.680393	4.64192E-06	0.005949827
1281.799976	179.881222	4.05967E-06	0.005203683
1281.839976	184.871186	4.17229E-06	0.005348202
1281.879976	200.110729	4.51622E-06	0.005789253
1281.919976	203.765167	4.5987E-06	0.00589516
1281.959976	197.776157	4.46353E-06	0.00572207
1281.999976	187.314799	4.22743E-06	0.00541957
1282.039976	180.547069	4.0747E-06	0.005223923
1282.079976	190.181145	4.29212E-06	0.005502846
1282.119976	217.274132	4.90357E-06	0.006286971
1282.159976	247.483122	5.58535E-06	0.007161311
1282.199976	284.423285	6.41904E-06	0.00823049
1282.239976	306.675447	6.92124E-06	0.008874688
1282.279976	293.580976	6.62571E-06	0.00849602
1282.319976	264.262834	5.96404E-06	0.007647813
1282.359976	251.788237	5.68251E-06	0.007287023
1282.399976	261.299217	5.89716E-06	0.007562517
1282.439976	246.118372	5.55455E-06	0.007123375
1282.479976	225.771266	5.09534E-06	0.006534675
1282.519976	214.720671	4.84595E-06	0.006215023
1282.559976	202.830548	4.5776E-06	0.00587105
1282.599976	203.966626	4.60324E-06	0.005904119
1282.639976	200.257013	4.51952E-06	0.005796919
1282.679976	202.691608	4.57447E-06	0.005867578
1282.719976	210.752295	4.75639E-06	0.006101111
1282.759976	213.604217	4.82075E-06	0.006183865
1282.799976	191.327268	4.31799E-06	0.005539117
1282.839976	170.466496	3.84719E-06	0.004935331
1282.879976	166.081841	3.74824E-06	0.004808537
1282.919976	171.73986	3.87593E-06	0.004972507
1282.959976	186.042122	4.19871E-06	0.005386779
1282.999976	207.582822	4.68486E-06	0.006010669
1283.039976	229.960402	5.18989E-06	0.006658831
1283.079976	262.996482	5.93546E-06	0.007615675
1283.119976	286.222226	6.45964E-06	0.008288489
1283.159976	288.556317	6.51231E-06	0.008356341
1283.199976	280.608908	6.33295E-06	0.008126444
1283.239976	268.476805	6.05915E-06	0.00777534
1283.279976	265.868723	6.00029E-06	0.007700048
1283.319976	279.428949	6.30632E-06	0.008093029
1283.359976	307.161788	6.93221E-06	0.008896526
1283.399976	298.545926	6.73777E-06	0.008647248
1283.439976	243.494924	5.49534E-06	0.007052941

1283.479976	204.025036	4.60456E-06	0.005909862
1283.519976	196.869963	4.44308E-06	0.005702783
1283.559976	196.641721	4.43793E-06	0.005696349
1283.599976	209.488199	4.72786E-06	0.006068677
1283.639976	204.022078	4.60449E-06	0.005910513
1283.679976	209.401064	4.72589E-06	0.006066531
1283.719976	211.462377	4.77241E-06	0.00612644
1283.759976	213.267651	4.81315E-06	0.006178934
1283.799976	219.32851	4.94994E-06	0.006354732
1283.839976	219.1193	4.94522E-06	0.006348868
1283.879976	204.021716	4.60449E-06	0.005911607
1283.919976	184.795607	4.17058E-06	0.00535469
1283.959976	161.167566	3.63733E-06	0.004670183
1283.999976	161.257251	3.63935E-06	0.004672927
1284.039976	176.211108	3.97684E-06	0.005106421
1284.079976	204.431126	4.61373E-06	0.005924393
1284.119976	241.01617	5.4394E-06	0.006984841
1284.159976	250.62114	5.65617E-06	0.007263427
1284.199976	246.374681	5.56033E-06	0.00714058
1284.239976	260.474803	5.87855E-06	0.007549473
1284.279976	293.760583	6.62977E-06	0.008514477
1284.319976	342.212036	7.72325E-06	0.009919123
1284.359976	324.533979	7.32428E-06	0.009407012
1284.399976	261.385434	5.89911E-06	0.007576811
1284.439976	218.072963	4.9216E-06	0.006321504
1284.479976	211.048464	4.76307E-06	0.006118068
1284.519976	221.477052	4.99843E-06	0.006420581
1284.559976	208.900573	4.71459E-06	0.00605618
1284.599976	193.4293	4.36543E-06	0.005607831
1284.639976	190.371132	4.29641E-06	0.005519342
1284.679976	195.471833	4.41153E-06	0.0056674
1284.719976	216.600053	4.88836E-06	0.006280176
1284.759976	238.633826	5.38563E-06	0.006919246
1284.799976	232.402407	5.245E-06	0.006738774
1284.839976	218.34761	4.9278E-06	0.006331436
1284.879976	208.483247	4.70518E-06	0.006045587
1284.919976	198.096774	4.47077E-06	0.005744579
1284.959976	200.46773	4.52428E-06	0.005813515
1284.999976	209.792766	4.73473E-06	0.006084129
1285.039976	215.789258	4.87006E-06	0.006258226
1285.079976	254.273978	5.73861E-06	0.007374572
1285.119976	293.822652	6.63117E-06	0.008521846
1285.159976	292.236476	6.59537E-06	0.008476106
1285.199976	299.639943	6.76246E-06	0.008691108
1285.239976	329.368516	7.43339E-06	0.009553688
1285.279976	343.468878	7.75161E-06	0.009962994

1285.319976	320.368515	7.23027E-06	0.009293212
1285.359976	294.731556	6.65168E-06	0.008549804
1285.399976	283.751696	6.40388E-06	0.008231548
1285.439976	257.668087	5.81521E-06	0.007475103
1285.479976	255.29773	5.76171E-06	0.007406568
1285.519976	255.119154	5.75768E-06	0.007401618
1285.559976	221.394038	4.99656E-06	0.006423371
1285.599976	194.618926	4.39228E-06	0.005646713
1285.639976	172.142195	3.88501E-06	0.004994724
1285.679976	165.000836	3.72384E-06	0.004787665
1285.719976	184.408135	4.16183E-06	0.005350954
1285.759976	199.92554	4.51204E-06	0.005801402
1285.799976	223.643074	5.04731E-06	0.006489835
1285.839976	237.694846	5.36444E-06	0.006897813
1285.879976	214.693796	4.84534E-06	0.006230526
1285.919976	200.147847	4.51706E-06	0.005808575
1285.959976	185.703911	4.19108E-06	0.005389559
1285.999976	176.565064	3.98483E-06	0.005124488
1286.039976	190.013666	4.28834E-06	0.005514982
1286.079976	218.528063	4.93187E-06	0.006342784
1286.119976	225.929248	5.09891E-06	0.006557808
1286.159976	255.9266	5.77591E-06	0.00742874
1286.199976	293.585067	6.62581E-06	0.008522112
1286.239976	310.455378	7.00655E-06	0.009012099
1286.279976	300.388892	6.77936E-06	0.008720154
1286.319976	274.371618	6.19219E-06	0.007965132
1286.359976	249.099935	5.62184E-06	0.007231708
1286.399976	244.511381	5.51828E-06	0.007098717
1286.439976	263.149072	5.93891E-06	0.007640049
1286.479976	250.908711	5.66266E-06	0.007284899
1286.519976	221.767753	5.00499E-06	0.006439019
1286.559976	201.472403	4.54695E-06	0.005849926
1286.599976	184.604648	4.16627E-06	0.005360323
1286.639976	177.361704	4.00281E-06	0.005150171
1286.679976	186.868827	4.21737E-06	0.005426404
1286.719976	209.647652	4.73146E-06	0.006088058
1286.759976	217.614947	4.91127E-06	0.006319621
1286.799976	214.391363	4.83851E-06	0.0062262
1286.839976	200.816569	4.53215E-06	0.005832152
1286.879976	184.913139	4.17323E-06	0.005370449
1286.919976	189.945165	4.2868E-06	0.005516766
1286.959976	198.828289	4.48728E-06	0.005774947
1286.999976	221.558439	5.00027E-06	0.006435341
1287.039976	235.562013	5.31631E-06	0.006842299
1287.079976	210.373076	4.74783E-06	0.006110833
1287.119976	219.62301	4.95659E-06	0.00637972

1287.159976	256.14352	5.7808E-06	0.007440818
1287.199976	291.39439	6.57637E-06	0.008465098
1287.239976	335.532271	7.5725E-06	0.00974762
1287.279976	344.167587	7.76738E-06	0.009998797
1287.319976	311.902166	7.0392E-06	0.009061699
1287.359976	266.793969	6.02117E-06	0.007751411
1287.399976	236.682001	5.34158E-06	0.006876754
1287.439976	232.574826	5.24889E-06	0.006757631
1287.479976	222.55132	5.02267E-06	0.006466591
1287.519976	207.709238	4.68771E-06	0.006035518
1287.559976	176.628321	3.98626E-06	0.005132543
1287.599976	157.804469	3.56143E-06	0.004585693
1287.639976	162.780223	3.67372E-06	0.004730432
1287.679976	177.069429	3.99621E-06	0.00514584
1287.719976	190.291494	4.29461E-06	0.00553026
1287.759976	188.445336	4.25295E-06	0.005476777
1287.799976	172.955856	3.90337E-06	0.005026763
1287.839976	170.683762	3.85209E-06	0.004960882
1287.879976	194.121236	4.38105E-06	0.005642261
1287.919976	191.006277	4.31075E-06	0.005551895
1287.959976	178.295973	4.02389E-06	0.005182612
1287.999976	169.805419	3.83227E-06	0.004935966
1288.039976	199.073948	4.49282E-06	0.005786934
1288.079976	240.952289	5.43796E-06	0.007004524
1288.119976	284.119218	6.41218E-06	0.008259651
1288.159976	288.674841	6.51499E-06	0.008392348
1288.199976	304.908185	6.88135E-06	0.008864559
1288.239976	323.366356	7.29793E-06	0.009401483
1288.279976	324.23358	7.3175E-06	0.009426989
1288.319976	328.272471	7.40865E-06	0.009544715
1288.359976	308.052007	6.9523E-06	0.008957071
1288.399976	255.430382	5.76471E-06	0.00742725
1288.439976	225.433046	5.08771E-06	0.006555209
1288.479976	228.422603	5.15518E-06	0.006642346
1288.519976	217.138614	4.90052E-06	0.006314413
1288.559976	207.187787	4.67594E-06	0.006025229
1288.599976	198.026127	4.46917E-06	0.005758977
1288.639976	194.089183	4.38032E-06	0.005644659
1288.679976	199.531989	4.50316E-06	0.005803131
1288.719976	203.914763	4.60207E-06	0.005930782
1288.759976	199.203416	4.49574E-06	0.005793934
1288.799976	216.558576	4.88743E-06	0.006298914
1288.839976	206.290763	4.6557E-06	0.006000446
1288.879976	185.450567	4.18536E-06	0.005394428
1288.919976	174.9185	3.94767E-06	0.005088227
1288.959976	195.601283	4.41445E-06	0.005690047

1288.999976	232.173088	5.23982E-06	0.006754132
1289.039976	263.542333	5.94778E-06	0.007666931
1289.079976	285.48444	6.44299E-06	0.008305524
1289.119976	299.46814	6.75858E-06	0.008712619
1289.159976	300.583303	6.78375E-06	0.008745334
1289.199976	334.744813	7.55472E-06	0.00973955
1289.239976	350.623663	7.91309E-06	0.010201869
1289.279976	367.497104	8.2939E-06	0.010693156
1289.319976	350.291324	7.90559E-06	0.010192832
1289.359976	293.556888	6.62517E-06	0.008542229
1289.399976	243.33034	5.49163E-06	0.007080904
1289.439976	220.59552	4.97853E-06	0.00641952
1289.479976	206.057442	4.65043E-06	0.005996636
1289.519976	187.542267	4.23257E-06	0.005457981
1289.559976	178.368744	4.02553E-06	0.005191168
1289.599976	187.525297	4.23218E-06	0.005457825
1289.639976	193.571672	4.36864E-06	0.005633977
1289.679976	211.736213	4.77859E-06	0.006162854
1289.719976	221.855924	5.00698E-06	0.006457601
1289.759976	204.300555	4.61078E-06	0.005946798
1289.799976	200.449249	4.52386E-06	0.005834875
1289.839976	204.697525	4.61974E-06	0.005958723
1289.879976	197.72015	4.46227E-06	0.005755791
1289.919976	189.796592	4.28344E-06	0.005525301
1289.959976	191.771364	4.32801E-06	0.005582963
1289.999976	207.584746	4.6849E-06	0.006043519
1290.039976	213.594845	4.82054E-06	0.006218687
1290.079976	207.879788	4.69156E-06	0.006052484
1290.119976	216.773109	4.89227E-06	0.006311612
1290.159976	243.292455	5.49077E-06	0.007083974
1290.199976	264.488321	5.96913E-06	0.007701375
1290.239976	264.170488	5.96196E-06	0.007692359
1290.279976	261.664339	5.9054E-06	0.007619619
1290.319976	275.75949	6.22351E-06	0.008030316
1290.359976	264.920547	5.97889E-06	0.007714917
1290.399976	247.759358	5.59158E-06	0.007215379
1290.439976	237.995352	5.37122E-06	0.006931242
1290.479976	216.263233	4.88076E-06	0.006298523
1290.519976	202.889003	4.57892E-06	0.005909191
1290.559976	198.178802	4.47262E-06	0.005772184
1290.599976	187.72551	4.2367E-06	0.005467889
1290.639976	176.573228	3.98501E-06	0.005143216
1290.679976	202.605395	4.57252E-06	0.005901662
1290.719976	222.638478	5.02464E-06	0.006485404
1290.759976	217.741758	4.91413E-06	0.00634296
1290.799976	188.638968	4.25732E-06	0.005495347

1290.839976	163.145593	3.68197E-06	0.004752833
1290.879976	183.552167	4.14252E-06	0.005347492
1290.919976	230.954224	5.21232E-06	0.006728682
1290.959976	240.433119	5.42624E-06	0.007005059
1290.999976	233.771823	5.2759E-06	0.006811192
1291.039976	242.024952	5.46217E-06	0.007051875
1291.079976	239.368215	5.40221E-06	0.006974682
1291.119976	257.081771	5.80198E-06	0.007491049
1291.159976	287.46514	6.48769E-06	0.008376643
1291.199976	319.507898	7.21085E-06	0.009310647
1291.239976	324.903734	7.33262E-06	0.009468178
1291.279976	312.316906	7.04856E-06	0.009101661
1291.319976	290.445371	6.55495E-06	0.008464535
1291.359976	279.823599	6.31523E-06	0.008155234
1291.399976	266.224806	6.00832E-06	0.007759148
1291.439976	244.841756	5.52574E-06	0.007136158
1291.479976	226.953962	5.12203E-06	0.006615005
1291.519976	202.821441	4.5774E-06	0.0059118
1291.559976	172.038749	3.88267E-06	0.005014708
1291.599976	153.175991	3.45697E-06	0.004465021
1291.639976	161.485672	3.64451E-06	0.004707391
1291.679976	176.285751	3.97852E-06	0.00513898
1291.719976	202.287866	4.56536E-06	0.005897161
1291.759976	228.259107	5.15149E-06	0.006654489
1291.799976	235.675857	5.31888E-06	0.006870924
1291.839976	213.82582	4.82575E-06	0.006234098
1291.879976	188.310998	4.24992E-06	0.005490383
1291.919976	193.175572	4.3597E-06	0.005632388
1291.959976	185.91721	4.19589E-06	0.005420925
1291.999976	194.466025	4.38883E-06	0.005670365
1292.039976	224.093785	5.05748E-06	0.006534472
1292.079976	233.520481	5.27023E-06	0.006809561
1292.119976	223.030072	5.03348E-06	0.006503858
1292.159976	228.974707	5.16764E-06	0.006677418
1292.199976	253.627846	5.72403E-06	0.007396588
1292.239976	277.834939	6.27035E-06	0.008102794
1292.279976	303.837028	6.85718E-06	0.008861394
1292.319976	312.124435	7.04421E-06	0.009103378
1292.359976	285.599892	6.44559E-06	0.008330025
1292.399976	231.623971	5.22743E-06	0.006755931
1292.439976	206.66555	4.66415E-06	0.006028139
1292.479976	239.261591	5.3998E-06	0.006979134
1292.519976	259.661129	5.86019E-06	0.007574413
1292.559976	231.448602	5.22347E-06	0.006751652
1292.599976	202.645972	4.57344E-06	0.005911625
1292.639976	186.996896	4.22026E-06	0.005455276

1292.679976	194.842366	4.39732E-06	0.005684329
1292.719976	213.337198	4.81472E-06	0.006224089
1292.759976	220.267175	4.97112E-06	0.006426469
1292.799976	229.826562	5.18687E-06	0.006705579
1292.839976	226.634539	5.11483E-06	0.006612651
1292.879976	215.98019	4.87437E-06	0.006301978
1292.919976	208.713533	4.71037E-06	0.006090136
1292.959976	224.114826	5.05796E-06	0.006539739
1292.999976	246.685976	5.56736E-06	0.007198595
1293.039976	242.832001	5.48038E-06	0.00708635
1293.079976	242.819075	5.48009E-06	0.007086192
1293.119976	258.981501	5.84485E-06	0.007558095
1293.159976	282.774325	6.38182E-06	0.008252718
1293.199976	301.662627	6.80811E-06	0.008804242
1293.239976	321.105977	7.24691E-06	0.009372
1293.279976	318.776889	7.19435E-06	0.009304309
1293.319976	314.818136	7.10501E-06	0.009189047
1293.359976	294.468406	6.64574E-06	0.008595336
1293.399976	267.070712	6.02741E-06	0.007795857
1293.439976	244.052764	5.50793E-06	0.007124178
1293.479976	226.393552	5.10939E-06	0.00660889
1293.519976	200.131244	4.51668E-06	0.00584242
1293.559976	159.957811	3.61002E-06	0.004669784
1293.599976	149.757633	3.37982E-06	0.004372137
1293.639976	168.269716	3.79761E-06	0.004912744
1293.679976	175.676343	3.96477E-06	0.005129144
1293.719976	173.998956	3.92691E-06	0.005080327
1293.759976	159.096599	3.59059E-06	0.00464536
1293.799976	172.043813	3.88279E-06	0.005023553
1293.839976	163.936608	3.69982E-06	0.004786976
1293.879976	169.844017	3.83314E-06	0.004959627
1293.919976	179.361317	4.04794E-06	0.005237704
1293.959976	178.189939	4.0215E-06	0.005203658
1293.999976	178.08988	4.01924E-06	0.005200897
1294.039976	198.304507	4.47546E-06	0.00579142
1294.079976	225.779418	5.09553E-06	0.006594019
1294.119976	252.620331	5.70129E-06	0.007378152
1294.159976	266.360756	6.01139E-06	0.007779702
1294.199976	287.072671	6.47883E-06	0.008384902
1294.239976	290.931485	6.56592E-06	0.008497874
1294.279976	291.140342	6.57063E-06	0.008504237
1294.319976	266.475879	6.01399E-06	0.007784027
1294.359976	256.856599	5.7969E-06	0.00750327
1294.399976	254.586845	5.74567E-06	0.007437196
1294.439976	228.782255	5.1633E-06	0.006683578
1294.479976	222.977087	5.03228E-06	0.006514189

1294.519976	210.541435	4.75163E-06	0.006151076
1294.559976	186.29255	4.20436E-06	0.005442801
1294.599976	170.207463	3.84135E-06	0.004973006
1294.639976	175.678931	3.96483E-06	0.005133026
1294.679976	198.946831	4.48995E-06	0.005813052
1294.719976	233.394148	5.26738E-06	0.006819783
1294.759976	240.101988	5.41877E-06	0.007016003
1294.799976	223.668405	5.04788E-06	0.006536001
1294.839976	226.814928	5.1189E-06	0.006628152
1294.879976	221.487784	4.99867E-06	0.006472679
1294.919976	220.36007	4.97322E-06	0.006439922
1294.959976	222.238366	5.01561E-06	0.006495015
1294.999976	222.557214	5.02281E-06	0.006504534
1295.039976	229.055593	5.16947E-06	0.006694665
1295.079976	242.427146	5.47124E-06	0.007085697
1295.119976	259.443092	5.85527E-06	0.007583276
1295.159976	274.663041	6.19876E-06	0.008028389
1295.199976	280.948391	6.34061E-06	0.008212363
1295.239976	302.332525	6.82322E-06	0.008837712
1295.279976	325.751706	7.35176E-06	0.00952259
1295.319976	301.563483	6.80587E-06	0.008815776
1295.359976	261.135303	5.89346E-06	0.007634152
1295.399976	235.426119	5.31324E-06	0.00688277
1295.439976	234.26081	5.28694E-06	0.006848914
1295.479976	230.908275	5.21128E-06	0.006751106
1295.519976	206.884951	4.66911E-06	0.006048919
1295.559976	186.001879	4.1978E-06	0.005438506
1295.599976	191.462301	4.32104E-06	0.005598336
1295.639976	212.582407	4.79769E-06	0.006216077
1295.679976	206.049701	4.65025E-06	0.006025242
1295.719976	177.354482	4.00264E-06	0.005186305
1295.759976	165.359574	3.73194E-06	0.004835692
1295.799976	164.993202	3.72367E-06	0.004825127
1295.839976	176.005268	3.97219E-06	0.005147327
1295.879976	183.581104	4.14317E-06	0.005369051
1295.919976	185.292111	4.18178E-06	0.005419259
1295.959976	210.6835	4.75483E-06	0.006162073
1295.999976	209.790209	4.73467E-06	0.006136136
1296.039976	203.290117	4.58797E-06	0.005946199
1296.079976	216.069593	4.87639E-06	0.006320191
1296.119975	223.077325	5.03454E-06	0.006525374
1296.159975	248.192395	5.60136E-06	0.007260254
1296.199975	282.477944	6.37513E-06	0.008263448
1296.239975	274.009058	6.184E-06	0.008015952
1296.279975	266.611758	6.01706E-06	0.007799789
1296.319975	289.080271	6.52414E-06	0.008457372

1296.359975	303.106829	6.8407E-06	0.008868008
1296.399975	282.173671	6.36827E-06	0.008255821
1296.439975	238.27077	5.37744E-06	0.006971527
1296.479975	215.126613	4.85511E-06	0.00629455
1296.519975	204.1892	4.60827E-06	0.005974709
1296.559975	188.890004	4.26298E-06	0.005527215
1296.599975	178.032453	4.01794E-06	0.005209667
1296.639975	167.743868	3.78575E-06	0.004908749
1296.679975	166.948567	3.7678E-06	0.004885626
1296.719975	187.30698	4.22726E-06	0.005481569
1296.759975	208.843059	4.7133E-06	0.006112015
1296.799975	223.74027	5.04951E-06	0.0065482
1296.839975	235.251965	5.30931E-06	0.006885324
1296.879975	229.45047	5.17838E-06	0.006715734
1296.919975	216.915149	4.89547E-06	0.006349036
1296.959975	215.851005	4.87146E-06	0.006318084
1296.999975	233.613128	5.27232E-06	0.006838203
1297.039975	248.243948	5.60252E-06	0.007266693
1297.079975	268.075066	6.05008E-06	0.007847439
1297.119975	324.740988	7.32895E-06	0.00950653
1297.159975	362.911244	8.1904E-06	0.010624261
1297.199975	369.176929	8.33181E-06	0.010808023
1297.239975	386.889719	8.73156E-06	0.011326931
1297.279975	390.865	8.82128E-06	0.011443668
1297.319975	383.651524	8.65848E-06	0.01123282
1297.359975	378.475192	8.54166E-06	0.011081605
1297.399975	343.275243	7.74724E-06	0.010051274
1297.439975	295.972018	6.67968E-06	0.008666479
1297.479975	256.767602	5.79489E-06	0.00751875
1297.519975	230.865325	5.21031E-06	0.00676048
1297.559975	205.53157	4.63856E-06	0.006018811
1297.599975	186.488596	4.20879E-06	0.005461323
1297.639975	185.200838	4.17972E-06	0.005423778
1297.679975	202.057407	4.56015E-06	0.005917621
1297.719975	202.645422	4.57342E-06	0.005935025
1297.759975	185.930366	4.19619E-06	0.005445647
1297.799975	193.340415	4.36342E-06	0.005662851
1297.839975	205.449922	4.63672E-06	0.006017719
1297.879975	199.575738	4.50415E-06	0.005845841
1297.919975	183.015424	4.1304E-06	0.005360933
1297.959975	168.728586	3.80797E-06	0.004942592
1297.999975	184.706792	4.16858E-06	0.00541081
1298.039975	222.560816	5.02289E-06	0.006519909
1298.079975	267.077304	6.02756E-06	0.007824258
1298.119975	299.592258	6.76138E-06	0.008777082
1298.159975	300.261785	6.77649E-06	0.008796968

1298.199975	296.412783	6.68962E-06	0.008684469
1298.239975	336.248013	7.58865E-06	0.009851888
1298.279975	379.411005	8.56278E-06	0.011116883
1298.319975	367.835398	8.30153E-06	0.010778046
1298.359975	348.514269	7.86548E-06	0.010212227
1298.399975	312.71042	7.05744E-06	0.009163378
1298.439975	268.048949	6.04949E-06	0.007854901
1298.479975	233.062837	5.2599E-06	0.006829879
1298.519975	218.087005	4.92192E-06	0.006391211
1298.559975	213.659744	4.822E-06	0.00626166
1298.599975	233.195521	5.2629E-06	0.006834399
1298.639975	245.977137	5.55136E-06	0.00720922
1298.679975	214.094969	4.83183E-06	0.006274995
1298.719975	201.228545	4.54145E-06	0.005898069
1298.759975	206.582375	4.66228E-06	0.006055178
1298.799975	215.536191	4.86435E-06	0.00631782
1298.839975	227.715492	5.13922E-06	0.006675026
1298.879975	225.895812	5.09815E-06	0.00662189
1298.919975	207.792944	4.6896E-06	0.006091412
1298.959975	194.984657	4.40053E-06	0.005716115
1298.999975	206.566186	4.66191E-06	0.006055822
1299.039975	229.82116	5.18674E-06	0.006737787
1299.079975	243.800806	5.50224E-06	0.007147856
1299.119975	269.734159	6.08752E-06	0.007908424
1299.159975	284.036409	6.41031E-06	0.008328013
1299.199975	284.676919	6.42476E-06	0.00834705
1299.239975	323.764889	7.30692E-06	0.009493446
1299.279975	351.698661	7.93735E-06	0.010312838
1299.319975	342.215504	7.72333E-06	0.010035073
1299.359975	302.843496	6.83476E-06	0.008880808
1299.399975	249.968253	5.64144E-06	0.007330481
1299.439975	206.094637	4.65127E-06	0.006044045
1299.479975	189.399727	4.27449E-06	0.005554612
1299.519975	190.816803	4.30647E-06	0.005596343
1299.559975	172.987912	3.9041E-06	0.005073607
1299.599975	178.993374	4.03963E-06	0.005249905
1299.639975	177.146473	3.99795E-06	0.005195895
1299.679975	162.979648	3.67822E-06	0.004780514
1299.719975	153.147891	3.45633E-06	0.004492267
1299.759975	159.085723	3.59034E-06	0.004666584
1299.799975	170.31158	3.8437E-06	0.004996035
1299.839975	179.819526	4.05828E-06	0.00527511
1299.879975	191.402243	4.31968E-06	0.005615068
1299.919975	200.043115	4.51469E-06	0.005868742
1299.959975	186.178426	4.20179E-06	0.005462156
1299.999975	184.143395	4.15586E-06	0.005402618

1300.039975	199.362651	4.49934E-06	0.005849318
1300.079975	205.30558	4.63346E-06	0.00602387
1300.119975	215.07237	4.85388E-06	0.006310631
1300.159975	224.058953	5.0567E-06	0.006574517
1300.199975	257.039219	5.80102E-06	0.007542482
1300.239975	263.23787	5.94091E-06	0.007724611
1300.279975	237.366268	5.35703E-06	0.006965634
1300.319975	230.107777	5.19321E-06	0.006752837
1300.359975	234.613482	5.2949E-06	0.006885275
1300.399975	259.029461	5.84593E-06	0.007602053
1300.439975	252.125229	5.69012E-06	0.007399653
1300.479975	208.760738	4.71144E-06	0.006127132
1300.519975	191.712932	4.32669E-06	0.005626952
1300.559975	201.89683	4.55653E-06	0.005926041
1300.599975	200.672015	4.52889E-06	0.005890271
1300.639975	191.726302	4.327E-06	0.005627863
1300.679975	176.611501	3.98588E-06	0.005184349
1300.719975	174.466072	3.93746E-06	0.005121528
1300.759975	194.188193	4.38256E-06	0.005700655
1300.799975	207.33009	4.67915E-06	0.00608664
1300.839975	206.823021	4.66771E-06	0.006071941
1300.879975	210.358836	4.74751E-06	0.006175935
1300.919975	214.738882	4.84636E-06	0.006304723
1300.959975	218.93668	4.9411E-06	0.006428168
1300.999975	215.331616	4.85973E-06	0.006322515
1301.039975	219.526888	4.95442E-06	0.006445893
1301.079975	249.706464	5.63553E-06	0.007332271
1301.119975	254.828904	5.75113E-06	0.007482915
1301.159975	252.495976	5.69848E-06	0.007414637
1301.199975	251.783772	5.68241E-06	0.00739395
1301.239975	277.118202	6.25417E-06	0.008138178
1301.279975	315.905412	7.12955E-06	0.009277534
1301.319975	311.579626	7.03192E-06	0.009150776
1301.359975	285.311839	6.43909E-06	0.008379575
1301.399975	284.005259	6.4096E-06	0.008341457
1301.439975	249.533237	5.63162E-06	0.007329212
1301.479975	203.98226	4.6036E-06	0.005991487
1301.519975	187.42063	4.22982E-06	0.005505199
1301.559975	175.071238	3.95111E-06	0.005142612
1301.599975	180.875421	4.08211E-06	0.005313269
1301.639975	178.634106	4.03152E-06	0.005247591
1301.679975	153.692897	3.46863E-06	0.004515052
1301.719975	135.331837	3.05425E-06	0.00397578
1301.759975	155.735452	3.51473E-06	0.004575338
1301.799975	170.146981	3.83998E-06	0.004998886
1301.839975	166.200263	3.75091E-06	0.004883083

1301.879975	176.081908	3.97392E-06	0.005173571
1301.919975	194.192597	4.38266E-06	0.005705868
1301.959975	195.049263	4.40199E-06	0.005731215
1301.999975	188.606237	4.25658E-06	0.005542067
1302.039975	191.330418	4.31806E-06	0.005622288
1302.079975	213.253706	4.81284E-06	0.006266701
1302.119975	228.941958	5.1669E-06	0.006727925
1302.159975	258.554612	5.83522E-06	0.007598387
1302.199975	277.18066	6.25558E-06	0.008146018
1302.239975	258.181002	5.82679E-06	0.007587873
1302.279975	266.508329	6.01472E-06	0.007832852
1302.319975	273.459679	6.1716E-06	0.008037403
1302.359975	242.518149	5.4733E-06	0.007128203
1302.399975	200.997585	4.53624E-06	0.005907993
1302.439975	199.63168	4.50541E-06	0.005868025
1302.479975	205.652278	4.64129E-06	0.006045181
1302.519975	181.527526	4.09682E-06	0.005336194
1302.559975	165.922254	3.74463E-06	0.004877611
1302.599975	158.922744	3.58666E-06	0.00467199
1302.639975	184.395702	4.16155E-06	0.005421007
1302.679975	214.102842	4.832E-06	0.006294553
1302.719975	201.836268	4.55516E-06	0.005934102
1302.759975	198.483663	4.4795E-06	0.005835713
1302.799975	203.80618	4.59962E-06	0.005992387
1302.839975	191.007101	4.31076E-06	0.005616236
1302.879975	185.934018	4.19627E-06	0.005467238
1302.919975	183.884842	4.15002E-06	0.00540715
1302.959975	179.395503	4.04871E-06	0.005275303
1302.999975	185.664339	4.19019E-06	0.005459812
1303.039975	216.454424	4.88507E-06	0.006365448
1303.079975	254.256576	5.73822E-06	0.007477355
1303.119975	262.661567	5.92791E-06	0.007724772
1303.159975	265.255854	5.98646E-06	0.007801309
1303.199975	278.549273	6.28647E-06	0.008192526
1303.239975	311.70452	7.03474E-06	0.00916795
1303.279975	349.646084	7.89103E-06	0.010284215
1303.319975	341.586444	7.70913E-06	0.010047463
1303.359975	296.906702	6.70077E-06	0.008733516
1303.399975	262.874134	5.9327E-06	0.007732685
1303.439975	241.925073	5.45991E-06	0.007116667
1303.479975	202.902549	4.57923E-06	0.005968932
1303.519975	192.878398	4.353E-06	0.005674218
1303.559975	197.475627	4.45675E-06	0.005809641
1303.599975	205.507272	4.63801E-06	0.006046113
1303.639975	190.216794	4.29293E-06	0.005596433
1303.679975	166.430001	3.75609E-06	0.004896744

1303.719975	161.007818	3.63372E-06	0.004737356
1303.759975	163.577695	3.69172E-06	0.004813118
1303.799975	157.561562	3.55594E-06	0.004636241
1303.839975	177.794037	4.01256E-06	0.005231741
1303.879975	178.580745	4.03032E-06	0.005255052
1303.919975	171.090771	3.86128E-06	0.005034801
1303.959975	164.013028	3.70155E-06	0.004826667
1303.999975	164.092649	3.70334E-06	0.004829159
1304.039975	197.892723	4.46616E-06	0.005824055
1304.079975	217.539961	4.90957E-06	0.006402477
1304.119975	222.510089	5.02174E-06	0.006548955
1304.159975	225.233952	5.08322E-06	0.006629328
1304.199975	223.841016	5.05178E-06	0.006588531
1304.239975	221.29046	4.99422E-06	0.006513658
1304.279975	246.808722	5.57013E-06	0.007265008
1304.319975	288.109489	6.50223E-06	0.008480988
1304.359975	275.655563	6.22116E-06	0.008114635
1304.399975	230.997538	5.21329E-06	0.006800219
1304.439975	199.038786	4.49203E-06	0.005859581
1304.479975	182.038869	4.10836E-06	0.005359278
1304.519975	180.311358	4.06938E-06	0.005308583
1304.559975	164.532998	3.71328E-06	0.004844197
1304.599975	147.795676	3.33554E-06	0.004351549
1304.639975	165.817232	3.74226E-06	0.004882307
1304.679975	185.48658	4.18617E-06	0.005461617
1304.719975	208.345166	4.70206E-06	0.006134872
1304.759975	211.33688	4.76958E-06	0.006223156
1304.799975	200.435882	4.52356E-06	0.005902339
1304.839975	194.474684	4.38902E-06	0.005726972
1304.879975	203.95932	4.60308E-06	0.006006464
1304.919975	214.51408	4.84128E-06	0.006317488
1304.959975	213.045989	4.80815E-06	0.006274445
1304.999975	196.412982	4.43277E-06	0.005784761
1305.039975	182.64112	4.12196E-06	0.005379317
1305.079975	193.824113	4.37434E-06	0.005708864
1305.119975	223.140205	5.03596E-06	0.006572537
1305.159975	262.654847	5.92775E-06	0.007736667
1305.199975	308.027445	6.95175E-06	0.009073424
1305.239975	315.372814	7.11753E-06	0.009290078
1305.279975	302.565297	6.82848E-06	0.008913075
1305.319975	287.484533	6.48813E-06	0.00846908
1305.359975	284.021633	6.40997E-06	0.008367322
1305.399975	277.658927	6.26638E-06	0.008180126
1305.439975	237.797269	5.36675E-06	0.007005974
1305.479975	213.427728	4.81677E-06	0.006288192
1305.519975	186.30079	4.20455E-06	0.005489123

1305.559975	172.715755	3.89795E-06	0.005089013
1305.599975	152.279655	3.43674E-06	0.004487007
1305.639975	136.020851	3.0698E-06	0.004008055
1305.679975	140.979281	3.18171E-06	0.00415429
1305.719975	154.945529	3.4969E-06	0.004565978
1305.759975	168.459995	3.80191E-06	0.004964379
1305.799975	171.140632	3.86241E-06	0.005043529
1305.839975	181.144361	4.08818E-06	0.005338504
1305.879975	183.60563	4.14372E-06	0.005411205
1305.919975	183.628587	4.14424E-06	0.005412048
1305.959975	203.65895	4.5963E-06	0.006002582
1305.999975	205.524723	4.63841E-06	0.006057759
1306.039975	190.322754	4.29532E-06	0.005609859
1306.079975	214.642293	4.84418E-06	0.006326883
1306.119975	259.939832	5.86648E-06	0.007662327
1306.159975	274.599369	6.19733E-06	0.008094698
1306.199975	290.155474	6.5484E-06	0.008553526
1306.239975	314.277135	7.0928E-06	0.009264895
1306.279975	357.331268	8.06447E-06	0.010534454
1306.319975	370.762684	8.3676E-06	0.01093076
1306.359975	358.799981	8.09762E-06	0.010578401
1306.399975	333.621491	7.52937E-06	0.009836372
1306.439975	284.212295	6.41428E-06	0.008379866
1306.479975	227.911693	5.14365E-06	0.006720075
1306.519975	174.083509	3.92882E-06	0.005133085
1306.559975	158.527149	3.57774E-06	0.004674528
1306.599975	182.139264	4.11063E-06	0.005370948
1306.639975	216.519543	4.88654E-06	0.006384954
1306.679975	228.124075	5.14844E-06	0.006727367
1306.719975	263.017504	5.93594E-06	0.00775661
1306.759975	290.219756	6.54986E-06	0.008559089
1306.799975	304.958728	6.88249E-06	0.008994043
1306.839975	324.233408	7.3175E-06	0.009562797
1306.879975	302.194057	6.8201E-06	0.008913051
1306.919975	246.660176	5.56678E-06	0.007275332
1306.959975	230.70071	5.20659E-06	0.00680481
1306.999975	255.703242	5.77087E-06	0.007542522
1307.039975	269.549934	6.08337E-06	0.007951203
1307.079975	285.329915	6.4395E-06	0.00841694
1307.119975	295.515811	6.66938E-06	0.00871768
1307.159975	296.976095	6.70234E-06	0.008761026
1307.199975	300.241602	6.77603E-06	0.008857632
1307.239975	326.93197	7.3784E-06	0.009645338
1307.279975	367.049406	8.28379E-06	0.010829238
1307.319975	373.94815	8.43949E-06	0.011033112
1307.359975	341.578533	7.70895E-06	0.010078375

1307.399975	294.720428	6.65143E-06	0.008696079
1307.439975	248.753303	5.61402E-06	0.007339988
1307.479975	219.068345	4.94407E-06	0.006464269
1307.519975	186.526009	4.20963E-06	0.005504178
1307.559975	171.095502	3.86139E-06	0.005048995
1307.599975	184.08929	4.15464E-06	0.005432606
1307.639975	174.83223	3.94572E-06	0.005159581
1307.679975	168.392756	3.80039E-06	0.004969694
1307.719975	171.372023	3.86763E-06	0.005057774
1307.759975	199.666892	4.5062E-06	0.005893033
1307.799975	241.252835	5.44474E-06	0.007120631
1307.839975	243.350153	5.49207E-06	0.007182754
1307.879975	233.881171	5.27837E-06	0.006903477
1307.919975	212.6688	4.79964E-06	0.006277543
1307.959975	187.920822	4.24111E-06	0.005547204
1307.999975	187.055494	4.22158E-06	0.005521829
1308.039975	195.916056	4.42155E-06	0.005783567
1308.079975	207.550163	4.68412E-06	0.006127201
1308.119975	223.181038	5.03689E-06	0.00658885
1308.159975	251.189521	5.669E-06	0.007415956
1308.199975	288.373911	6.5082E-06	0.008514024
1308.239975	305.278501	6.88971E-06	0.009013395
1308.279975	309.026754	6.9743E-06	0.009124341
1308.319975	287.843138	6.49622E-06	0.008499133
1308.359975	258.587549	5.83596E-06	0.007635538
1308.399975	230.556768	5.20335E-06	0.006808057
1308.439975	216.269451	4.8809E-06	0.006386365
1308.479975	209.580737	4.72995E-06	0.006189039
1308.519975	209.572934	4.72977E-06	0.006188997
1308.559975	198.533199	4.48062E-06	0.005863157
1308.599975	181.332401	4.09242E-06	0.00535534
1308.639975	176.262069	3.97799E-06	0.005205756
1308.679975	199.314807	4.49826E-06	0.00588678
1308.719975	206.819758	4.66763E-06	0.006108626
1308.759975	195.965068	4.42266E-06	0.005788199
1308.799975	187.679743	4.23567E-06	0.005543645
1308.839975	188.990349	4.26525E-06	0.005582528
1308.879975	200.945226	4.53505E-06	0.005935841
1308.919975	206.671567	4.66429E-06	0.006105182
1308.959975	226.223559	5.10555E-06	0.006682961
1308.999975	231.395667	5.22228E-06	0.006835962
1309.039975	240.401759	5.42553E-06	0.007102239
1309.079975	269.116911	6.07359E-06	0.00795082
1309.119975	312.921139	7.06219E-06	0.00924526
1309.159975	323.366787	7.29794E-06	0.009554168
1309.199975	315.768733	7.12646E-06	0.009329962

1309.239975	337.978954	7.62771E-06	0.009986508
1309.279975	331.38296	7.47885E-06	0.009791911
1309.319975	297.41748	6.7123E-06	0.008788546
1309.359975	277.451282	6.26169E-06	0.008198805
1309.399975	262.284201	5.91939E-06	0.007750848
1309.439975	226.750429	5.11744E-06	0.006700982
1309.479975	211.763028	4.7792E-06	0.006258262
1309.519975	200.510375	4.52524E-06	0.005925892
1309.559975	188.671707	4.25806E-06	0.005576182
1309.599975	181.708482	4.10091E-06	0.005370548
1309.639975	182.909275	4.12801E-06	0.005406204
1309.679975	196.66593	4.43848E-06	0.005812983
1309.719975	212.159342	4.78814E-06	0.006271124
1309.759975	210.102927	4.74173E-06	0.006210529
1309.799975	198.951354	4.49005E-06	0.005881074
1309.839975	178.639318	4.03164E-06	0.005280804
1309.879975	178.70214	4.03306E-06	0.005282822
1309.919975	190.18601	4.29223E-06	0.005622482
1309.959975	197.847235	4.46514E-06	0.00584915
1309.999975	193.765144	4.37301E-06	0.005728642
1310.039975	188.349515	4.25079E-06	0.0055687
1310.079975	187.992298	4.24272E-06	0.005558308
1310.119975	204.059447	4.60534E-06	0.006033545
1310.159975	248.070585	5.59861E-06	0.007335072
1310.199975	264.942142	5.97938E-06	0.007834177
1310.239975	262.384524	5.92165E-06	0.007758787
1310.279975	272.362707	6.14685E-06	0.00805409
1310.319975	283.113492	6.38948E-06	0.00837226
1310.359975	278.804836	6.29224E-06	0.008245095
1310.399975	269.640236	6.0854E-06	0.007974314
1310.439975	274.351162	6.19172E-06	0.008113882
1310.479975	241.536602	5.45114E-06	0.007143616
1310.519975	180.3682	4.07066E-06	0.00533468
1310.559975	151.71294	3.42395E-06	0.004487291
1310.599975	169.273761	3.82027E-06	0.00500685
1310.639975	186.096666	4.19994E-06	0.005504613
1310.679975	202.754232	4.57588E-06	0.005997515
1310.719975	203.496161	4.59262E-06	0.006019645
1310.759975	198.080727	4.47041E-06	0.005859629
1310.799975	203.430061	4.59113E-06	0.006018057
1310.839975	200.222358	4.51874E-06	0.005923345
1310.879975	186.340767	4.20545E-06	0.005512842
1310.919975	178.749546	4.03413E-06	0.005288419
1310.959975	197.34687	4.45384E-06	0.005838811
1310.999975	228.508504	5.15712E-06	0.006760982
1311.039975	248.431573	5.60675E-06	0.007350679

1311.079975	250.177937	5.64617E-06	0.007402577
1311.119975	256.076603	5.77929E-06	0.007577346
1311.159975	301.565054	6.8059E-06	0.008923628
1311.199975	340.45914	7.68369E-06	0.010074852
1311.239975	350.708314	7.915E-06	0.010378462
1311.279975	330.40634	7.45681E-06	0.009777967
1311.319975	301.301363	6.79995E-06	0.008916913
1311.359975	288.321076	6.50701E-06	0.008533026
1311.399975	262.440555	5.92292E-06	0.007767314
1311.439975	252.329517	5.69473E-06	0.007468291
1311.479975	246.265616	5.55787E-06	0.007289038
1311.519975	226.764242	5.11775E-06	0.006712035
1311.559975	196.266058	4.42945E-06	0.005809491
1311.599975	171.355212	3.86725E-06	0.005072283
1311.639975	172.029921	3.88248E-06	0.00509241
1311.679975	173.528142	3.91629E-06	0.005136917
1311.719975	171.564869	3.87198E-06	0.005078954
1311.759975	172.427816	3.89146E-06	0.005104656
1311.799975	160.513289	3.62256E-06	0.004752076
1311.839975	160.574611	3.62395E-06	0.004754036
1311.879975	182.295571	4.11416E-06	0.00539728
1311.919975	188.242141	4.24836E-06	0.005573512
1311.959975	168.989115	3.81385E-06	0.005003617
1311.999975	162.6843	3.67156E-06	0.004817084
1312.039975	169.15861	3.81767E-06	0.005008941
1312.079975	184.432747	4.16239E-06	0.005461389
1312.119975	204.257991	4.60982E-06	0.006048635
1312.159975	228.383698	5.1543E-06	0.006763269
1312.199975	270.554892	6.10605E-06	0.008012355
1312.239975	293.726887	6.62901E-06	0.008698848
1312.279975	299.648611	6.76265E-06	0.008874493
1312.319975	299.31005	6.75501E-06	0.008864736
1312.359975	292.248435	6.59564E-06	0.008655854
1312.399975	254.951011	5.75389E-06	0.007551404
1312.439975	210.066874	4.74092E-06	0.006222169
1312.479975	203.342145	4.58915E-06	0.006023166
1312.519975	220.542733	4.97734E-06	0.006532861
1312.559975	190.355279	4.29605E-06	0.005638828
1312.599975	153.223557	3.45804E-06	0.004539026
1312.639975	147.285458	3.32403E-06	0.004363252
1312.679975	169.105711	3.81648E-06	0.005009817
1312.719975	215.823887	4.87084E-06	0.006394055
1312.759975	245.656961	5.54414E-06	0.007278119
1312.799975	240.774675	5.43395E-06	0.007133688
1312.839975	211.486342	4.77295E-06	0.006266122
1312.879975	180.238146	4.06772E-06	0.005340433

1312.919975	174.312298	3.93399E-06	0.005165008
1312.959975	165.399814	3.73284E-06	0.004901074
1312.999975	165.02737	3.72444E-06	0.004890187
1313.039975	192.966912	4.35499E-06	0.005718282
1313.079975	234.716795	5.29723E-06	0.006955688
1313.119975	280.92793	6.34015E-06	0.00832538
1313.159975	318.932245	7.19786E-06	0.009451937
1313.199975	317.577494	7.16728E-06	0.009412074
1313.239975	293.825758	6.63124E-06	0.008708407
1313.279975	284.97466	6.43148E-06	0.008446335
1313.319975	270.394229	6.10242E-06	0.008014432
1313.359975	236.97701	5.34824E-06	0.007024166
1313.399975	232.294784	5.24257E-06	0.006885591
1313.439975	234.455481	5.29133E-06	0.006949849
1313.479975	242.890457	5.4817E-06	0.007200102
1313.519975	237.093615	5.35087E-06	0.007028478
1313.559975	203.805113	4.5996E-06	0.006041847
1313.599975	176.99782	3.99459E-06	0.005247299
1313.639975	169.127377	3.81697E-06	0.005014123
1313.679975	172.453026	3.89202E-06	0.005112875
1313.719975	169.023841	3.81463E-06	0.005011359
1313.759975	166.346207	3.7542E-06	0.004932121
1313.799975	171.103969	3.86158E-06	0.005073341
1313.839975	189.69358	4.28112E-06	0.005624706
1313.879975	203.446665	4.59151E-06	0.00603269
1313.919975	184.043646	4.15361E-06	0.005457509
1313.959975	173.415296	3.91374E-06	0.0051425
1313.999975	191.988507	4.33291E-06	0.005693448
1314.039975	205.062808	4.62798E-06	0.006081353
1314.079975	207.930847	4.69271E-06	0.006166596
1314.119975	220.223819	4.97014E-06	0.006531367
1314.159975	243.59452	5.49759E-06	0.007224711
1314.199975	264.453354	5.96834E-06	0.007843597
1314.239975	287.260406	6.48307E-06	0.008520306
1314.279975	296.872637	6.7E-06	0.008805678
1314.319975	286.426321	6.46424E-06	0.008496084
1314.359975	267.046107	6.02686E-06	0.007921462
1314.399975	238.259783	5.37719E-06	0.00706778
1314.439975	221.970587	5.00957E-06	0.006584775
1314.479975	213.747633	4.82399E-06	0.006341033
1314.519975	212.278053	4.79082E-06	0.006297629
1314.559975	205.401012	4.63561E-06	0.006093794
1314.599975	194.475469	4.38904E-06	0.005769832
1314.639975	200.895059	4.53392E-06	0.005960475
1314.679975	233.135307	5.26154E-06	0.00691724
1314.719975	265.820066	5.99919E-06	0.007887253

1314.759975	275.439096	6.21628E-06	0.008172912
1314.799975	294.488802	6.6462E-06	0.008738426
1314.839975	323.558906	7.30227E-06	0.009601322
1314.879975	331.191631	7.47453E-06	0.009828115
1314.919975	353.948831	7.98813E-06	0.010503754
1314.959975	399.279504	9.01118E-06	0.011849344
1314.999975	450.965236	1.01777E-05	0.013383619
1315.039975	514.61202	1.16141E-05	0.015272975
1315.079975	600.371982	1.35496E-05	0.017818754
1315.119975	716.782366	1.61768E-05	0.021274406
1315.159975	857.115127	1.93439E-05	0.025440316
1315.199975	1030.170528	2.32495E-05	0.03057776
1315.239975	1228.095451	2.77164E-05	0.036453722
1315.279975	1390.472234	3.1381E-05	0.041274829
1315.319975	1455.269669	3.28434E-05	0.043199592
1315.359975	1413.843774	3.19085E-05	0.041971144
1315.399975	1262.834935	2.85004E-05	0.037489459
1315.439975	1031.530996	2.32802E-05	0.030623729
1315.479975	733.561115	1.65555E-05	0.021778366
1315.519975	481.561287	1.08682E-05	0.014297291
1315.559975	329.30999	7.43207E-06	0.009777331
1315.599975	248.554536	5.60953E-06	0.007379897
1315.639975	242.309363	5.46858E-06	0.007194689
1315.679975	250.459932	5.65253E-06	0.007436923
1315.719975	244.797823	5.52475E-06	0.007269019
1315.759975	244.730185	5.52322E-06	0.007267231
1315.799975	254.462959	5.74287E-06	0.007556474
1315.839975	247.377983	5.58298E-06	0.007346304
1315.879975	232.63382	5.25022E-06	0.006908661
1315.919975	235.354236	5.31162E-06	0.006989663
1315.959975	267.484274	6.03675E-06	0.007944118
1315.999975	339.708409	7.66675E-06	0.010089437
1316.039975	393.150285	8.87285E-06	0.011677031
1316.079975	417.475962	9.42185E-06	0.012399909
1316.119975	471.50991	1.06413E-05	0.014005256
1316.159975	573.751966	1.29488E-05	0.01704267
1316.199975	645.660222	1.45717E-05	0.019179208
1316.239975	726.506185	1.63962E-05	0.021581377
1316.279975	805.592162	1.81811E-05	0.023931409
1316.319975	867.304984	1.95739E-05	0.02576547
1316.359975	837.06442	1.88914E-05	0.024867854
1316.399975	720.320949	1.62566E-05	0.021400241
1316.439975	583.579749	1.31706E-05	0.017338281
1316.479975	460.492479	1.03927E-05	0.013681747
1316.519975	339.138784	7.65389E-06	0.010076499
1316.559975	256.530778	5.78954E-06	0.00762228

1316.599975	226.773476	5.11796E-06	0.006738308
1316.639975	200.474085	4.52442E-06	0.005957033
1316.679975	185.126626	4.17805E-06	0.005501155
1316.719975	194.192166	4.38265E-06	0.005770718
1316.759975	210.219922	4.74437E-06	0.006247198
1316.799975	219.757536	4.95962E-06	0.006530829
1316.839975	254.981674	5.75458E-06	0.007577863
1316.879975	272.200562	6.14319E-06	0.008089841
1316.919975	258.20516	5.82733E-06	0.007674128
1316.959975	229.136006	5.17128E-06	0.006810369
1316.999975	204.493274	4.61513E-06	0.006078124
1317.039975	243.072524	5.48581E-06	0.007225029
1317.079975	284.896294	6.42971E-06	0.008468446
1317.119975	322.91013	7.28763E-06	0.009598685
1317.159975	366.221843	8.26512E-06	0.010886481
1317.199975	406.201055	9.16739E-06	0.012075289
1317.239975	455.889915	1.02888E-05	0.013552819
1317.279975	513.438014	1.15876E-05	0.015264088
1317.319975	564.005656	1.27288E-05	0.016767932
1317.359975	552.543263	1.24701E-05	0.016427653
1317.399975	465.557397	1.0507E-05	0.013841898
1317.439975	370.204168	8.35499E-06	0.011007201
1317.479975	291.124915	6.57028E-06	0.008656217
1317.519975	229.169686	5.17204E-06	0.006814267
1317.559975	197.674807	4.46125E-06	0.005877958
1317.599975	185.219048	4.18014E-06	0.005507747
1317.639975	168.541866	3.80376E-06	0.00501198
1317.679975	157.689892	3.55884E-06	0.004689414
1317.719975	163.308203	3.68564E-06	0.00485664
1317.759975	182.960853	4.12917E-06	0.005441257
1317.799975	186.626198	4.21189E-06	0.005550433
1317.839975	200.55934	4.52634E-06	0.005964998
1317.879975	219.833794	4.96134E-06	0.006538454
1317.919975	201.361212	4.54444E-06	0.005989211
1317.959975	193.266205	4.36175E-06	0.005748611
1317.999975	214.198949	4.83417E-06	0.006371438
1318.039975	196.279059	4.42974E-06	0.005838581
1318.079975	192.897335	4.35342E-06	0.005738161
1318.119975	226.783979	5.1182E-06	0.0067464
1318.159975	259.492182	5.85638E-06	0.007719642
1318.199975	299.51759	6.75969E-06	0.00891063
1318.239975	328.251983	7.40819E-06	0.009765772
1318.279975	337.292103	7.61221E-06	0.010035028
1318.319975	328.168551	7.40631E-06	0.009763883
1318.359975	316.674839	7.14691E-06	0.0094222
1318.399975	294.553078	6.64765E-06	0.008764265

1318.439975	281.843539	6.36082E-06	0.008386354
1318.479975	265.974617	6.00268E-06	0.007914409
1318.519975	227.986474	5.14534E-06	0.00678423
1318.559975	188.063982	4.24434E-06	0.00559642
1318.599975	178.610282	4.03099E-06	0.005315257
1318.639975	180.803094	4.08047E-06	0.005380676
1318.679975	177.229208	3.99982E-06	0.005274478
1318.719975	201.643129	4.5508E-06	0.006001237
1318.759975	242.716013	5.47776E-06	0.007223854
1318.799975	245.095379	5.53146E-06	0.007294891
1318.839975	228.260188	5.15151E-06	0.006794023
1318.879975	210.941637	4.76066E-06	0.006278738
1318.919975	198.208198	4.47328E-06	0.005899902
1318.959975	177.860018	4.01405E-06	0.005294375
1318.999975	199.40862	4.50037E-06	0.005935994
1319.039975	238.587465	5.38459E-06	0.007102485
1319.079975	238.318581	5.37852E-06	0.007094696
1319.119975	241.532241	5.45105E-06	0.007190584
1319.159975	251.887155	5.68474E-06	0.007499084
1319.199975	279.871393	6.31631E-06	0.008332473
1319.239975	309.575641	6.98669E-06	0.009217122
1319.279975	303.692468	6.85392E-06	0.009042234
1319.319975	286.002338	6.45467E-06	0.008515781
1319.359975	275.065771	6.20785E-06	0.008190391
1319.399975	266.600456	6.0168E-06	0.007938567
1319.439975	251.09944	5.66696E-06	0.00747722
1319.479975	222.393575	5.01911E-06	0.006622619
1319.519975	210.084982	4.74133E-06	0.006256274
1319.559975	212.399841	4.79357E-06	0.006325401
1319.599975	206.905069	4.66956E-06	0.00616195
1319.639975	182.915048	4.12814E-06	0.005447656
1319.679975	164.640298	3.7157E-06	0.004903538
1319.719975	171.123324	3.86202E-06	0.005096778
1319.759975	183.673061	4.14525E-06	0.005470729
1319.799975	191.967885	4.33245E-06	0.005717964
1319.839975	193.780344	4.37335E-06	0.005772125
1319.879975	202.505994	4.57028E-06	0.006032219
1319.919975	206.647286	4.66374E-06	0.006155765
1319.959975	208.256436	4.70006E-06	0.006203888
1319.999975	208.442577	4.70426E-06	0.006209621
1320.039975	192.108124	4.33561E-06	0.005723182
1320.079975	188.08325	4.24478E-06	0.005603445
1320.119975	212.110271	4.78703E-06	0.006319458
1320.159975	248.60088	5.61058E-06	0.007406857
1320.199975	244.368202	5.51505E-06	0.007280969
1320.239975	232.886091	5.25591E-06	0.006939069

1320.279975	252.449807	5.69744E-06	0.007522217
1320.319975	270.414577	6.10288E-06	0.008057755
1320.359975	271.236342	6.12143E-06	0.008082486
1320.399975	242.19791	5.46607E-06	0.007217398
1320.439975	225.226304	5.08304E-06	0.006711854
1320.479975	203.160855	4.58506E-06	0.006054477
1320.519975	185.327932	4.18259E-06	0.005523198
1320.559975	174.655124	3.94172E-06	0.005205281
1320.599975	163.321873	3.68595E-06	0.004867662
1320.639975	154.752334	3.49254E-06	0.004612394
1320.679975	157.856296	3.5626E-06	0.00470505
1320.719975	165.191424	3.72814E-06	0.004923829
1320.759975	166.810249	3.76467E-06	0.004972232
1320.799975	164.522554	3.71304E-06	0.00490419
1320.839975	180.640905	4.07681E-06	0.005384818
1320.879975	190.765867	4.30532E-06	0.005686811
1320.919975	190.969041	4.30991E-06	0.00569304
1320.959975	202.823316	4.57744E-06	0.006046615
1320.999975	202.712436	4.57494E-06	0.006043492
1321.039975	207.099329	4.67394E-06	0.006174466
1321.079975	211.082674	4.76384E-06	0.006293416
1321.119975	226.865328	5.12003E-06	0.00676418
1321.159975	265.053039	5.98188E-06	0.007903018
1321.199975	287.51811	6.48888E-06	0.008573112
1321.239975	302.157142	6.81927E-06	0.009009887
1321.279975	304.34325	6.8686E-06	0.009075348
1321.319975	309.373431	6.98213E-06	0.009225624
1321.359975	288.484052	6.51068E-06	0.008602956
1321.399975	241.315665	5.44616E-06	0.007196554
1321.439975	233.034683	5.25927E-06	0.006949807
1321.479975	229.021081	5.16869E-06	0.006830316
1321.519975	244.496719	5.51795E-06	0.007292082
1321.559975	223.949622	5.05423E-06	0.006679469
1321.599975	171.996043	3.88171E-06	0.005130069
1321.639975	164.039244	3.70214E-06	0.004892893
1321.679975	179.055003	4.04102E-06	0.005340938
1321.719975	206.177617	4.65314E-06	0.00615015
1321.759975	215.473712	4.86294E-06	0.006427642
1321.799975	201.596315	4.54975E-06	0.006013857
1321.839975	203.611752	4.59523E-06	0.006074164
1321.879975	197.573924	4.45897E-06	0.005894221
1321.919975	190.322277	4.29531E-06	0.005678054
1321.959975	188.214315	4.24773E-06	0.005615336
1321.999975	172.546815	3.89414E-06	0.005148055
1322.039975	179.861238	4.05922E-06	0.005366448
1322.079975	200.892516	4.53386E-06	0.005994131

1322.119975	223.562954	5.0455E-06	0.006670762
1322.159975	247.198558	5.57893E-06	0.007376234
1322.199975	255.623996	5.76908E-06	0.007627874
1322.239975	277.086645	6.25346E-06	0.008268574
1322.279975	308.495787	6.96232E-06	0.009206137
1322.319975	318.443102	7.18682E-06	0.009503272
1322.359975	299.830124	6.76675E-06	0.008948077
1322.399975	258.71484	5.83883E-06	0.007721273
1322.439975	236.85629	5.34552E-06	0.007069125
1322.479975	234.87743	5.30086E-06	0.007010276
1322.519975	235.284579	5.31005E-06	0.007022641
1322.559975	216.597488	4.8883E-06	0.006465075
1322.599975	188.818255	4.26137E-06	0.005636081
1322.639975	178.240245	4.02263E-06	0.005320497
1322.679975	168.850193	3.81071E-06	0.005040355
1322.719975	179.134965	4.04283E-06	0.005347528
1322.759975	220.678266	4.9804E-06	0.006587875
1322.799975	236.880771	5.34607E-06	0.00707178
1322.839975	232.732941	5.25246E-06	0.006948162
1322.879975	226.847307	5.11963E-06	0.006772653
1322.919975	225.621125	5.09195E-06	0.006736248
1322.959975	217.153631	4.90085E-06	0.006483635
1322.999975	204.998206	4.62652E-06	0.006120891
1323.039975	201.06491	4.53775E-06	0.006003631
1323.079975	208.728803	4.71072E-06	0.006232657
1323.119975	204.260443	4.60987E-06	0.006099416
1323.159975	215.001637	4.85229E-06	0.006420353
1323.199975	256.773281	5.79502E-06	0.007667964
1323.239975	286.589352	6.46792E-06	0.008558614
1323.279975	294.57699	6.64819E-06	0.00879742
1323.319975	284.384668	6.41817E-06	0.008493287
1323.359975	275.780466	6.22398E-06	0.008236567
1323.399975	260.449434	5.87798E-06	0.00777892
1323.439975	231.831554	5.23212E-06	0.00692439
1323.479975	210.454856	4.74967E-06	0.006286097
1323.519975	184.611616	4.16643E-06	0.005514349
1323.559975	170.016169	3.83703E-06	0.005078537
1323.599975	144.291451	3.25646E-06	0.004310246
1323.639975	141.800717	3.20024E-06	0.004235972
1323.679975	157.76094	3.56044E-06	0.004712889
1323.719975	174.991594	3.94932E-06	0.005227789
1323.759975	186.957447	4.21937E-06	0.005585432
1323.799975	186.754515	4.21479E-06	0.005579538
1323.839975	171.443975	3.86925E-06	0.00512227
1323.879975	145.807576	3.29067E-06	0.004356457
1323.919975	141.57848	3.19523E-06	0.004230228

1323.959975	159.296795	3.59511E-06	0.004759777
1323.999975	167.043889	3.76995E-06	0.004991411
1324.039975	176.702	3.98792E-06	0.005280163
1324.079975	182.225522	4.11258E-06	0.00544538
1324.119975	195.046353	4.40192E-06	0.005828676
1324.159975	203.619931	4.59542E-06	0.006085069
1324.199975	218.741806	4.9367E-06	0.006537175
1324.239975	254.26427	5.73839E-06	0.007599006
1324.279975	273.862696	6.1807E-06	0.008184977
1324.319975	265.01367	5.98099E-06	0.007920744
1324.359975	250.585933	5.65538E-06	0.007489753
1324.399975	244.846882	5.52585E-06	0.00731844
1324.439975	235.162791	5.3073E-06	0.007029196
1324.479975	219.405332	4.95167E-06	0.006558391
1324.519975	198.630957	4.48282E-06	0.00593759
1324.559975	166.267027	3.75242E-06	0.004970299
1324.599975	142.804136	3.22289E-06	0.004269041
1324.639975	143.12376	3.2301E-06	0.004278725
1324.679975	155.305401	3.50503E-06	0.004643039
1324.719975	179.732066	4.0563E-06	0.005373465
1324.759975	201.561457	4.54896E-06	0.006026282
1324.799975	206.435593	4.65896E-06	0.006172195
1324.839975	219.23766	4.94789E-06	0.006555161
1324.879975	210.452607	4.74962E-06	0.006292679
1324.919975	190.025163	4.2886E-06	0.005682056
1324.959975	179.337911	4.04741E-06	0.005362652
1324.999975	178.964289	4.03897E-06	0.005351641
1325.039975	180.92135	4.08314E-06	0.005410328
1325.079975	201.681702	4.55168E-06	0.006031334
1325.119975	250.812334	5.66049E-06	0.007500822
1325.159975	272.155853	6.14218E-06	0.008139369
1325.199975	285.498831	6.44331E-06	0.008538676
1325.239975	320.840685	7.24093E-06	0.009595966
1325.279975	344.448853	7.77373E-06	0.01030237
1325.319975	345.18217	7.79028E-06	0.010324615
1325.359975	303.755268	6.85533E-06	0.009085784
1325.399975	261.472999	5.90108E-06	0.007821293
1325.439975	228.930712	5.16665E-06	0.006848081
1325.479975	202.329319	4.56629E-06	0.006052527
1325.519975	183.274957	4.13626E-06	0.005482696
1325.559975	171.519654	3.87096E-06	0.005131189
1325.599975	150.695615	3.40099E-06	0.004508352
1325.639975	159.511004	3.59994E-06	0.004772226
1325.679975	170.761979	3.85386E-06	0.005108985
1325.719975	159.576871	3.60143E-06	0.004774485
1325.759975	141.928879	3.20314E-06	0.004246591

1325.799975	148.333866	3.34769E-06	0.004438366
1325.839975	181.20183	4.08947E-06	0.005421987
1325.879975	199.541683	4.50338E-06	0.005970939
1325.919975	196.207044	4.42812E-06	0.005871332
1325.959975	200.1445	4.51698E-06	0.005989338
1325.999975	200.449739	4.52387E-06	0.005998653
1326.039975	187.126219	4.22318E-06	0.005600103
1326.079975	188.426595	4.25253E-06	0.005639189
1326.119975	186.670939	4.2129E-06	0.005586815
1326.159975	196.614125	4.43731E-06	0.005884579
1326.199975	244.672103	5.52191E-06	0.007323155
1326.239975	298.348899	6.73332E-06	0.008929997
1326.279975	321.657366	7.25936E-06	0.009627942
1326.319975	313.177277	7.06797E-06	0.009374396
1326.359975	267.727239	6.04223E-06	0.008014173
1326.399975	241.397185	5.448E-06	0.007226225
1326.439975	228.218448	5.15057E-06	0.006831925
1326.479975	210.963638	4.76116E-06	0.006315577
1326.519975	195.272666	4.40703E-06	0.005846016
1326.559975	191.499954	4.32189E-06	0.005733243
1326.599975	184.445945	4.16269E-06	0.005522222
1326.639975	178.620141	4.03121E-06	0.005347961
1326.679975	185.976715	4.19724E-06	0.005568388
1326.719975	183.420311	4.13954E-06	0.005492012
1326.759975	180.865704	4.08189E-06	0.005415684
1326.799975	192.497111	4.34439E-06	0.005764139
1326.839975	198.935676	4.4897E-06	0.005957115
1326.879975	186.703271	4.21363E-06	0.005590985
1326.919975	176.192948	3.97643E-06	0.005276404
1326.959975	167.65726	3.78379E-06	0.005020939
1326.999975	171.901968	3.87959E-06	0.005148213
1327.039975	181.114294	4.0875E-06	0.005424272
1327.079975	207.268681	4.67777E-06	0.006207769
1327.119975	231.34498	5.22113E-06	0.006929071
1327.159975	251.988316	5.68703E-06	0.007547592
1327.199975	267.136078	6.02889E-06	0.008001542
1327.239975	289.292718	6.52893E-06	0.008665462
1327.279975	306.709887	6.92201E-06	0.009187452
1327.319975	326.546357	7.3697E-06	0.009781945
1327.359975	334.17864	7.54195E-06	0.010010878
1327.399975	301.011213	6.7934E-06	0.009017564
1327.439975	246.904473	5.57229E-06	0.007396881
1327.479975	214.872315	4.84937E-06	0.00643744
1327.519975	197.381794	4.45463E-06	0.005913613
1327.559975	181.255429	4.09068E-06	0.005430626
1327.599975	175.943415	3.9708E-06	0.005271631

1327.639975	181.824319	4.10352E-06	0.005447999
1327.679975	188.498126	4.25414E-06	0.005648137
1327.719975	179.992335	4.06218E-06	0.005393433
1327.759975	169.153547	3.81756E-06	0.005068803
1327.799975	169.364368	3.82232E-06	0.005075274
1327.839975	173.072754	3.90601E-06	0.005186558
1327.879975	170.070525	3.83825E-06	0.005096742
1327.919975	160.458475	3.62132E-06	0.004808829
1327.959975	139.762671	3.15425E-06	0.004188716
1327.999975	170.492798	3.84778E-06	0.005109858
1328.039975	230.81659	5.20921E-06	0.006918038
1328.079975	229.461631	5.17863E-06	0.006877634
1328.119975	225.192692	5.08229E-06	0.006749885
1328.159975	237.91276	5.36936E-06	0.007131369
1328.199975	241.326197	5.4464E-06	0.007233903
1328.239975	271.201781	6.12065E-06	0.008129687
1328.279975	280.995856	6.34169E-06	0.008423533
1328.319975	273.288766	6.16775E-06	0.008192741
1328.359975	256.556045	5.79011E-06	0.007691354
1328.399975	216.124498	4.87763E-06	0.006479442
1328.439975	192.502574	4.34451E-06	0.005771427
1328.479975	182.759559	4.12463E-06	0.005479487
1328.519975	167.808268	3.7872E-06	0.005031369
1328.559975	163.841611	3.69768E-06	0.004912586
1328.599975	166.828882	3.7651E-06	0.005002306
1328.639975	155.553183	3.51062E-06	0.004664348
1328.679975	139.503956	3.14841E-06	0.004183229
1328.719975	153.475261	3.46372E-06	0.004602318
1328.759975	178.628841	4.0314E-06	0.005356768
1328.799975	201.4522	4.5465E-06	0.006041383
1328.839975	221.479603	4.99849E-06	0.006642188
1328.879975	214.656566	4.8445E-06	0.006437759
1328.919975	192.79922	4.35121E-06	0.00578241
1328.959975	182.984803	4.12971E-06	0.005488222
1328.999975	192.272008	4.33931E-06	0.005766945
1329.039975	214.219749	4.83464E-06	0.006425432
1329.079975	210.64837	4.75404E-06	0.0063185
1329.119975	209.017641	4.71724E-06	0.006269774
1329.159975	242.700416	5.47741E-06	0.007280355
1329.199975	248.428853	5.60669E-06	0.007452416
1329.239975	249.847705	5.63871E-06	0.007495205
1329.279975	266.91623	6.02393E-06	0.008007486
1329.319975	264.477077	5.96888E-06	0.00793455
1329.359975	256.317017	5.78472E-06	0.007689973
1329.399975	235.738949	5.3203E-06	0.007072806
1329.439975	192.330597	4.34063E-06	0.005770612

1329.479975	167.078395	3.77073E-06	0.005013106
1329.519975	162.190691	3.66042E-06	0.004866599
1329.559975	160.951706	3.63246E-06	0.004829568
1329.599975	164.794944	3.71919E-06	0.004945038
1329.639975	159.253406	3.59413E-06	0.004778896
1329.679975	160.187418	3.61521E-06	0.004807068
1329.719975	172.176314	3.88578E-06	0.005166999
1329.759975	189.35288	4.27343E-06	0.005682637
1329.799975	190.532541	4.30005E-06	0.005718212
1329.839975	180.179471	4.0664E-06	0.005407661
1329.879975	183.807366	4.14828E-06	0.005516709
1329.919975	184.005592	4.15275E-06	0.005522825
1329.959975	183.638405	4.14446E-06	0.00551197
1329.999975	208.750202	4.7112E-06	0.006265898
1330.039975	209.627492	4.731E-06	0.00629242
1330.079975	219.494025	4.95367E-06	0.006588783
1330.119975	247.871785	5.59412E-06	0.007440852
1330.159975	274.672712	6.19898E-06	0.008245636
1330.199975	284.868579	6.42909E-06	0.008551971
1330.239975	290.167975	6.54869E-06	0.008711325
1330.279975	266.763225	6.02047E-06	0.008008917
1330.319975	243.693868	5.49983E-06	0.007316535
1330.359975	229.451847	5.17841E-06	0.006889148
1330.399975	220.435861	4.97493E-06	0.006618647
1330.439975	218.801974	4.93806E-06	0.006569787
1330.479975	201.953138	4.5578E-06	0.006064063
1330.519975	187.676089	4.23559E-06	0.005635534
1330.559975	174.994599	3.94938E-06	0.005254893
1330.599975	149.190291	3.36702E-06	0.004480153
1330.639975	157.414609	3.55263E-06	0.004727269
1330.679975	179.118601	4.04246E-06	0.005379217
1330.719975	190.813301	4.30639E-06	0.0057306
1330.759975	208.741446	4.711E-06	0.006269215
1330.799975	230.535103	5.20286E-06	0.006923961
1330.839975	231.35325	5.22132E-06	0.006948742
1330.879975	223.813023	5.05115E-06	0.006722472
1330.919975	214.054762	4.83092E-06	0.006429565
1330.959975	200.290885	4.52029E-06	0.00601632
1330.999975	209.672968	4.73203E-06	0.006298328
1331.039975	216.282202	4.88119E-06	0.006497056
1331.079975	208.966278	4.71608E-06	0.006277477
1331.119975	212.17385	4.78847E-06	0.006374026
1331.159975	211.751782	4.77894E-06	0.006361537
1331.199975	229.408154	5.17742E-06	0.006892185
1331.239975	264.131754	5.96109E-06	0.007935636
1331.279975	270.790151	6.11136E-06	0.008135927

1331.319975	252.359092	5.69539E-06	0.007582391
1331.359975	236.324335	5.33351E-06	0.007100823
1331.399975	240.040463	5.41738E-06	0.007212698
1331.439975	243.844106	5.50322E-06	0.00732721
1331.479975	225.04085	5.07886E-06	0.006762398
1331.519975	196.199791	4.42796E-06	0.005895912
1331.559975	167.260155	3.77483E-06	0.005026411
1331.599975	156.232726	3.52596E-06	0.004695162
1331.639975	168.18328	3.79566E-06	0.005054456
1331.679975	159.872361	3.6081E-06	0.00480483
1331.719975	154.092923	3.47766E-06	0.004631273
1331.759975	158.02427	3.56639E-06	0.004749572
1331.799975	160.123955	3.61377E-06	0.004812825
1331.839975	156.290704	3.52726E-06	0.004697751
1331.879975	153.131297	3.45596E-06	0.004602924
1331.919975	158.821507	3.58438E-06	0.004774108
1331.959975	152.621815	3.44446E-06	0.004587885
1331.999975	152.819145	3.44892E-06	0.004593955
1332.039975	168.414163	3.80087E-06	0.005062915
1332.079975	187.740436	4.23704E-06	0.005644076
1332.119975	215.210883	4.85701E-06	0.00647012
1332.159975	234.50155	5.29237E-06	0.007050288
1332.199975	268.870964	6.06804E-06	0.008083847
1332.239975	310.573419	7.00921E-06	0.009337949
1332.279975	313.0681	7.06551E-06	0.009413239
1332.319975	302.370831	6.82409E-06	0.009091869
1332.359975	301.201392	6.7977E-06	0.009056978
1332.399975	291.246106	6.57302E-06	0.00875789
1332.439975	251.648054	5.67935E-06	0.007567388
1332.479975	217.852866	4.91664E-06	0.006551319
1332.519975	205.031251	4.62727E-06	0.006165929
1332.559975	199.067713	4.49268E-06	0.005986767
1332.599975	173.147905	3.90771E-06	0.00520741
1332.639975	169.874603	3.83383E-06	0.005109119
1332.679975	181.539386	4.09709E-06	0.005460111
1332.719975	201.622639	4.55034E-06	0.006064332
1332.759975	242.583784	5.47478E-06	0.007296565
1332.799975	257.987599	5.82242E-06	0.007760122
1332.839975	217.539245	4.90956E-06	0.006543655
1332.879975	204.935376	4.62511E-06	0.006164711
1332.919975	201.183398	4.54043E-06	0.006052029
1332.959975	212.9423	4.80581E-06	0.006405954
1332.999975	230.523098	5.20259E-06	0.006935046
1333.039975	227.985958	5.14533E-06	0.006858925
1333.079975	221.646044	5.00224E-06	0.006668389
1333.119975	230.065049	5.19225E-06	0.006921889

1333.159975	232.333465	5.24344E-06	0.006990348
1333.199975	236.730916	5.34269E-06	0.00712287
1333.239975	254.413771	5.74176E-06	0.00765515
1333.279975	276.661364	6.24386E-06	0.008324816
1333.319975	279.748288	6.31353E-06	0.008417955
1333.359975	256.519901	5.7893E-06	0.007719217
1333.399975	242.154818	5.4651E-06	0.00728716
1333.439975	219.595182	4.95596E-06	0.006608472
1333.479975	200.868625	4.53333E-06	0.006045098
1333.519975	192.290433	4.33973E-06	0.005787113
1333.559975	176.209032	3.97679E-06	0.005303291
1333.599975	168.208456	3.79623E-06	0.005062653
1333.639975	171.254761	3.86498E-06	0.005154494
1333.679975	170.717028	3.85285E-06	0.005138463
1333.719975	158.172958	3.56974E-06	0.004761038
1333.759975	155.27828	3.50441E-06	0.004674048
1333.799975	159.022558	3.58892E-06	0.004786898
1333.839975	173.729727	3.92084E-06	0.00522977
1333.879975	188.778416	4.26047E-06	0.00568295
1333.919975	190.986484	4.3103E-06	0.005749594
1333.959975	185.544726	4.18749E-06	0.005585939
1333.999975	169.907678	3.83458E-06	0.005115329
1334.039975	162.240077	3.66153E-06	0.004884631
1334.079975	170.840126	3.85562E-06	0.00514371
1334.119975	191.993555	4.33303E-06	0.005780778
1334.159975	211.307902	4.76893E-06	0.006362509
1334.199975	214.785146	4.8474E-06	0.006467403
1334.239975	232.14656	5.23922E-06	0.006990383
1334.279975	233.093689	5.2606E-06	0.007019113
1334.319975	225.580209	5.09103E-06	0.006793065
1334.359975	241.671138	5.45418E-06	0.007277841
1334.399975	261.617773	5.90435E-06	0.007878763
1334.439975	245.220542	5.53429E-06	0.007385173
1334.479975	226.93133	5.12152E-06	0.006834571
1334.519975	192.257253	4.33898E-06	0.005790453
1334.559975	167.78226	3.78661E-06	0.005053461
1334.599975	172.64247	3.8963E-06	0.005200002
1334.639975	191.217934	4.31552E-06	0.005759669
1334.679975	204.308751	4.61096E-06	0.006154161
1334.719975	208.354985	4.70228E-06	0.006276229
1334.759975	221.673514	5.00286E-06	0.006677621
1334.799975	207.007201	4.67186E-06	0.006236004
1334.839975	179.001348	4.03981E-06	0.005392501
1334.879975	159.855172	3.60771E-06	0.004815858
1334.919975	154.514531	3.48718E-06	0.004655103
1334.959975	173.445808	3.91443E-06	0.005225608

1334.999975	194.915957	4.39898E-06	0.00587264
1335.039975	199.917665	4.51186E-06	0.006023518
1335.079975	210.104427	4.74176E-06	0.006330635
1335.119975	230.110383	5.19327E-06	0.00693364
1335.159975	241.166296	5.44279E-06	0.007266992
1335.199975	265.883528	6.00062E-06	0.008012029
1335.239975	286.538773	6.46678E-06	0.008634704
1335.279975	289.907191	6.5428E-06	0.008736472
1335.319975	275.023175	6.20689E-06	0.008288184
1335.359975	237.079585	5.35056E-06	0.007144918
1335.399975	202.666196	4.57389E-06	0.006107978
1335.439975	188.863375	4.26238E-06	0.005692157
1335.479975	183.559291	4.14268E-06	0.005532463
1335.519975	170.669611	3.85178E-06	0.005144123
1335.559975	164.020434	3.70171E-06	0.004943859
1335.599975	148.236333	3.34549E-06	0.004468233
1335.639975	139.090013	3.13907E-06	0.004192665
1335.679975	157.181977	3.54738E-06	0.004738162
1335.719975	183.801538	4.14814E-06	0.00554076
1335.759975	192.967565	4.35501E-06	0.005817247
1335.799975	186.485124	4.20871E-06	0.005621994
1335.839975	182.04774	4.10856E-06	0.005488384
1335.879975	195.133458	4.40389E-06	0.005883069
1335.919975	209.931224	4.73786E-06	0.006329396
1335.959975	213.5765	4.82012E-06	0.006439493
1335.999975	179.912855	4.06038E-06	0.005424671
1336.039975	159.164446	3.59212E-06	0.004799216
1336.079975	178.434168	4.02701E-06	0.005380408
1336.119975	211.140625	4.76515E-06	0.006366812
1336.159975	248.610149	5.61078E-06	0.007496906
1336.199975	258.578114	5.83575E-06	0.007797726
1336.239975	280.810673	6.33751E-06	0.008468429
1336.279975	286.377937	6.46315E-06	0.008636579
1336.319975	294.575552	6.64816E-06	0.008884069
1336.359975	286.631405	6.46887E-06	0.008644741
1336.399975	256.210516	5.78231E-06	0.007727485
1336.439975	234.448876	5.29118E-06	0.007071351
1336.479975	208.880299	4.71414E-06	0.00630035
1336.519975	197.297377	4.45273E-06	0.005951159
1336.559975	187.568402	4.23316E-06	0.005657869
1336.599975	182.011038	4.10774E-06	0.005490399
1336.639975	184.587425	4.16588E-06	0.005568283
1336.679975	197.345318	4.45381E-06	0.005953317
1336.719975	186.563537	4.21048E-06	0.005628232
1336.759975	167.5569	3.78153E-06	0.005054992
1336.799975	178.346299	4.02503E-06	0.005380657

1336.839975	182.169443	4.11131E-06	0.005496164
1336.879975	183.796434	4.14803E-06	0.005545418
1336.919975	196.098759	4.42568E-06	0.005916774
1336.959975	203.586891	4.59467E-06	0.006142893
1336.999975	197.730884	4.46251E-06	0.005966377
1337.039975	190.125825	4.29088E-06	0.005737071
1337.079975	218.140018	4.92312E-06	0.0065826
1337.119975	255.81365	5.77336E-06	0.007719672
1337.159975	269.694435	6.08663E-06	0.008138795
1337.199975	272.896243	6.15889E-06	0.008235665
1337.239975	299.938027	6.76918E-06	0.009052023
1337.279975	355.301323	8.01866E-06	0.010723188
1337.319975	369.091115	8.32987E-06	0.011139705
1337.359975	335.380502	7.56907E-06	0.010122572
1337.399975	299.747562	6.76488E-06	0.009047357
1337.439975	281.765584	6.35906E-06	0.008504857
1337.479975	253.887828	5.72989E-06	0.007663619
1337.519975	205.857607	4.64592E-06	0.00621401
1337.559975	169.234165	3.81938E-06	0.005108649
1337.599975	165.154532	3.72731E-06	0.004985647
1337.639975	156.582441	3.53385E-06	0.004727016
1337.679975	162.658864	3.67098E-06	0.004910602
1337.719975	159.857723	3.60777E-06	0.004826181
1337.759975	160.115924	3.61359E-06	0.004834121
1337.799975	160.827696	3.62966E-06	0.004855755
1337.839975	159.445097	3.59845E-06	0.004814155
1337.879975	168.325589	3.79887E-06	0.005082438
1337.919975	171.433658	3.86902E-06	0.005176438
1337.959975	175.517676	3.96119E-06	0.005299913
1337.999975	184.403894	4.16174E-06	0.005568407
1338.039975	188.00754	4.24307E-06	0.005677395
1338.079975	203.061546	4.58282E-06	0.006132175
1338.119975	219.585635	4.95574E-06	0.006631377
1338.159975	233.564348	5.27122E-06	0.007053738
1338.199975	251.723653	5.68105E-06	0.007602384
1338.239975	259.673285	5.86046E-06	0.007842708
1338.279975	273.581101	6.17434E-06	0.008263001
1338.319975	285.149391	6.43542E-06	0.008612657
1338.359975	280.962774	6.34094E-06	0.008486458
1338.399975	264.556367	5.97067E-06	0.007991143
1338.439975	238.017227	5.37172E-06	0.007189721
1338.479975	220.619394	4.97907E-06	0.006664389
1338.519975	193.86821	4.37534E-06	0.005856474
1338.559975	176.707797	3.98805E-06	0.005338243
1338.599975	168.44192	3.8015E-06	0.005088687
1338.639975	153.490632	3.46407E-06	0.004637142

1338.679975	144.949871	3.27132E-06	0.004379246
1338.719975	155.284208	3.50455E-06	0.004691609
1338.759975	173.4585	3.91472E-06	0.005240866
1338.799975	181.242103	4.09038E-06	0.005476203
1338.839975	171.638959	3.87365E-06	0.0051862
1338.879975	161.693671	3.6492E-06	0.004885842
1338.919975	162.736507	3.67274E-06	0.0049175
1338.959975	179.498483	4.05103E-06	0.005424168
1338.999975	203.692764	4.59706E-06	0.006155466
1339.039975	227.101557	5.12537E-06	0.00686307
1339.079975	249.940243	5.6408E-06	0.007553487
1339.119975	249.168176	5.62338E-06	0.007530379
1339.159975	254.047908	5.73351E-06	0.007678084
1339.199975	276.87581	6.2487E-06	0.008368261
1339.239975	292.744378	6.60683E-06	0.008848135
1339.279975	301.690126	6.80873E-06	0.00911879
1339.319975	304.575462	6.87384E-06	0.009206276
1339.359975	296.227607	6.68544E-06	0.008954217
1339.399975	265.340408	5.98836E-06	0.008020814
1339.439975	259.082746	5.84714E-06	0.007831889
1339.479975	256.271332	5.78369E-06	0.007747133
1339.519975	213.168043	4.81091E-06	0.006444304
1339.559975	174.649202	3.94159E-06	0.005279995
1339.599975	156.694042	3.53637E-06	0.004737316
1339.639975	159.620844	3.60242E-06	0.004825946
1339.679975	189.309338	4.27245E-06	0.005723713
1339.719975	197.410784	4.45529E-06	0.005968836
1339.759975	170.380719	3.84526E-06	0.005151719
1339.799975	180.207307	4.06703E-06	0.005449004
1339.839975	182.689246	4.12304E-06	0.005524216
1339.879975	182.529205	4.11943E-06	0.005519542
1339.919975	188.7534	4.2599E-06	0.005707927
1339.959975	192.845789	4.35226E-06	0.005831855
1339.999975	209.49753	4.72807E-06	0.00633561
1340.039975	216.664165	4.88981E-06	0.006552539
1340.079975	210.51408	4.75101E-06	0.006366733
1340.119975	211.801563	4.78007E-06	0.006405862
1340.159975	222.46551	5.02074E-06	0.00672859
1340.199975	241.56678	5.45183E-06	0.007306537
1340.239975	249.008716	5.61978E-06	0.007531854
1340.279975	254.158555	5.736E-06	0.007687852
1340.319975	239.548058	5.40627E-06	0.007246126
1340.359975	239.411403	5.40318E-06	0.007242209
1340.399975	237.090666	5.35081E-06	0.00717222
1340.439975	232.503686	5.24728E-06	0.00703367
1340.479975	207.81291	4.69005E-06	0.006286915

1340.519975	158.146313	3.56914E-06	0.004784506
1340.559975	160.30726	3.61791E-06	0.004850027
1340.599975	174.082315	3.9288E-06	0.005266943
1340.639975	185.297422	4.1819E-06	0.005606429
1340.679975	176.21713	3.97698E-06	0.005331851
1340.719975	165.397158	3.73278E-06	0.005004617
1340.759975	167.448841	3.77909E-06	0.005066849
1340.799975	176.775835	3.98958E-06	0.005349235
1340.839975	192.931591	4.3542E-06	0.005838282
1340.879974	214.944773	4.851E-06	0.006504614
1340.919974	229.707115	5.18417E-06	0.006951557
1340.959974	237.090964	5.35081E-06	0.007175226
1340.999974	230.413821	5.20012E-06	0.006973359
1341.039974	223.732831	5.04934E-06	0.006771365
1341.079974	235.671405	5.31878E-06	0.007132903
1341.119974	225.670657	5.09307E-06	0.006830421
1341.159974	216.324257	4.88214E-06	0.006547727
1341.199974	229.609098	5.18196E-06	0.006950041
1341.239974	282.740294	6.38105E-06	0.008558526
1341.279974	314.817455	7.10499E-06	0.009529783
1341.319974	313.080541	7.06579E-06	0.009477488
1341.359974	303.659854	6.85318E-06	0.009192581
1341.399974	278.421848	6.28359E-06	0.008428812
1341.439974	261.329407	5.89784E-06	0.007911599
1341.479974	234.476494	5.29181E-06	0.007098854
1341.519974	193.941553	4.37699E-06	0.00587182
1341.559974	165.207679	3.72851E-06	0.005002016
1341.599974	166.866521	3.76594E-06	0.005052392
1341.639974	165.169483	3.72765E-06	0.005001158
1341.679974	148.103652	3.34249E-06	0.004484556
1341.719974	158.294817	3.57249E-06	0.004793286
1341.759974	174.497107	3.93816E-06	0.005284061
1341.799974	193.192291	4.36008E-06	0.005850356
1341.839974	194.259384	4.38416E-06	0.005882846
1341.879974	192.201625	4.33772E-06	0.005820704
1341.919974	191.320369	4.31783E-06	0.005794188
1341.959974	174.69011	3.94251E-06	0.005290694
1341.999974	170.043112	3.83764E-06	0.005150108
1342.039974	172.116433	3.88443E-06	0.005213058
1342.079974	174.088686	3.92894E-06	0.005272951
1342.119974	190.764198	4.30528E-06	0.005778205
1342.159974	200.991397	4.5361E-06	0.006088166
1342.199974	247.30609	5.58135E-06	0.007491293
1342.239974	269.868374	6.09055E-06	0.008174984
1342.279974	259.665807	5.8603E-06	0.007866157
1342.319974	253.408237	5.71907E-06	0.007676823

1342.359974	246.00983	5.5521E-06	0.007452916
1342.399974	229.591448	5.18156E-06	0.006955725
1342.439974	200.814831	4.53211E-06	0.006084087
1342.479974	169.351012	3.82202E-06	0.005130981
1342.519974	162.65768	3.67096E-06	0.004928333
1342.559974	166.406737	3.75557E-06	0.005042076
1342.599974	176.810703	3.99037E-06	0.005357472
1342.639974	180.542012	4.07458E-06	0.005470696
1342.679974	162.804726	3.67428E-06	0.004933377
1342.719974	142.518746	3.21645E-06	0.004318791
1342.759974	156.2475	3.52629E-06	0.004734959
1342.799974	192.799944	4.35123E-06	0.005842826
1342.839974	205.903728	4.64696E-06	0.006240124
1342.879974	188.068217	4.24444E-06	0.00569977
1342.919974	183.057302	4.13135E-06	0.00554807
1342.959974	195.812102	4.41921E-06	0.005934817
1342.999974	206.471679	4.65978E-06	0.006258082
1343.039974	218.489706	4.93101E-06	0.006622541
1343.079974	228.205318	5.15028E-06	0.006917233
1343.119974	221.124701	4.99048E-06	0.006702809
1343.159974	222.358565	5.01832E-06	0.006740411
1343.199974	239.173488	5.39781E-06	0.007250342
1343.239974	254.322653	5.73971E-06	0.007709805
1343.279974	256.672975	5.79275E-06	0.007781287
1343.319974	250.7978	5.66016E-06	0.007603402
1343.359974	231.200272	5.21787E-06	0.007009475
1343.399974	213.644953	4.82167E-06	0.00647743
1343.439974	215.101053	4.85453E-06	0.006521771
1343.479974	198.487096	4.47958E-06	0.006018222
1343.519974	162.415059	3.66548E-06	0.004924648
1343.559974	148.61407	3.35401E-06	0.004506317
1343.599974	164.919192	3.722E-06	0.005000874
1343.639974	169.801837	3.83219E-06	0.005149085
1343.679974	176.258185	3.9779E-06	0.005345027
1343.719974	193.658097	4.37059E-06	0.005872854
1343.759974	211.764914	4.77924E-06	0.00642215
1343.799974	216.915926	4.89549E-06	0.00657856
1343.839974	192.953482	4.35469E-06	0.005852008
1343.879974	166.931817	3.76742E-06	0.005062958
1343.919974	152.849929	3.44961E-06	0.004636
1343.959974	153.977191	3.47505E-06	0.004670329
1343.999974	180.154528	4.06584E-06	0.005464485
1344.039974	202.613863	4.57271E-06	0.006145909
1344.079974	203.006737	4.58158E-06	0.006158009
1344.119974	202.60433	4.5725E-06	0.006145985
1344.159974	210.131924	4.74238E-06	0.006374524

1344.199974	232.968179	5.25777E-06	0.007067491
1344.239974	238.354708	5.37933E-06	0.007231115
1344.279974	236.243872	5.3317E-06	0.007167291
1344.319974	244.897618	5.527E-06	0.007430054
1344.359974	237.629312	5.36296E-06	0.007209752
1344.399974	234.325383	5.2884E-06	0.007109721
1344.439974	209.058839	4.71817E-06	0.006343292
1344.479974	180.026098	4.06294E-06	0.005462539
1344.519974	170.32224	3.84394E-06	0.005168248
1344.559974	178.007242	4.01738E-06	0.005401602
1344.599974	191.265907	4.31661E-06	0.005804107
1344.639974	207.649851	4.68637E-06	0.006301478
1344.679974	209.78534	4.73456E-06	0.006366472
1344.719974	208.715621	4.71042E-06	0.006334197
1344.759974	207.83076	4.69045E-06	0.00630753
1344.799974	215.593413	4.86564E-06	0.006543317
1344.839974	209.107759	4.71927E-06	0.006346664
1344.879974	199.899339	4.51145E-06	0.006067358
1344.919974	210.733259	4.75596E-06	0.00639638
1344.959974	210.651252	4.75411E-06	0.006394081
1344.999974	192.508794	4.34466E-06	0.005843561
1345.039974	185.121859	4.17794E-06	0.0056195
1345.079974	209.066806	4.71835E-06	0.006346554
1345.119974	257.714183	5.81625E-06	0.007823554
1345.159974	304.284075	6.86727E-06	0.009237574
1345.199974	324.939728	7.33344E-06	0.009864939
1345.239974	321.553474	7.25701E-06	0.009762425
1345.279974	296.727673	6.69673E-06	0.009008977
1345.319974	277.921794	6.27231E-06	0.008438261
1345.359974	274.837891	6.20271E-06	0.008344876
1345.399974	253.574567	5.72282E-06	0.007699488
1345.439974	222.391845	5.01907E-06	0.006752863
1345.479974	191.378094	4.31914E-06	0.005811312
1345.519974	164.953821	3.72278E-06	0.005009072
1345.559974	157.203341	3.54786E-06	0.004773859
1345.599974	148.938529	3.36134E-06	0.004523012
1345.639974	144.13743	3.25298E-06	0.004377341
1345.679974	170.795552	3.85462E-06	0.005187082
1345.719974	209.86452	4.73635E-06	0.006373801
1345.759974	217.500517	4.90868E-06	0.00660591
1345.799974	201.933422	4.55736E-06	0.00613329
1345.839974	172.459133	3.89216E-06	0.005238228
1345.879974	158.198297	3.57032E-06	0.004805216
1345.919974	162.259124	3.66196E-06	0.004928708
1345.959974	165.693733	3.73948E-06	0.005033186
1345.999974	169.292175	3.82069E-06	0.005142647

1346.039974	163.627989	3.69286E-06	0.004970732
1346.079974	163.353116	3.68665E-06	0.004962529
1346.119974	174.011075	3.92719E-06	0.005286466
1346.159974	208.032918	4.69501E-06	0.006320239
1346.199974	250.616106	5.65606E-06	0.007614183
1346.239974	269.523609	6.08277E-06	0.008188871
1346.279974	276.010895	6.22918E-06	0.008386222
1346.319974	267.175054	6.02977E-06	0.008117998
1346.359974	253.550613	5.72228E-06	0.007704254
1346.399974	235.026489	5.30422E-06	0.007141602
1346.439974	213.761589	4.8243E-06	0.006495632
1346.479974	182.830232	4.12622E-06	0.005555878
1346.519974	154.370242	3.48392E-06	0.00469117
1346.559974	135.712799	3.06285E-06	0.00412431
1346.599974	135.529013	3.0587E-06	0.004118847
1346.639974	156.047356	3.52177E-06	0.004742558
1346.679974	176.291649	3.97866E-06	0.005357978
1346.719974	173.217327	3.90927E-06	0.005264697
1346.759974	177.575139	4.00762E-06	0.005397307
1346.799974	191.701524	4.32644E-06	0.005826844
1346.839974	183.726703	4.14646E-06	0.005584612
1346.879974	180.881298	4.08224E-06	0.005498286
1346.919974	182.628859	4.12168E-06	0.005551572
1346.959974	172.811607	3.90012E-06	0.005253302
1346.999974	170.772158	3.85409E-06	0.005191459
1347.039974	173.436635	3.91422E-06	0.005272615
1347.079974	180.968637	4.08421E-06	0.005501758
1347.119974	201.718628	4.55251E-06	0.006132775
1347.159974	227.630909	5.13731E-06	0.006920782
1347.199974	259.377099	5.85378E-06	0.007886212
1347.239974	279.773775	6.3141E-06	0.008506614
1347.279974	272.87174	6.15834E-06	0.008297002
1347.319974	274.645554	6.19837E-06	0.008351184
1347.359974	268.774646	6.06587E-06	0.00817291
1347.399974	259.942444	5.86654E-06	0.007904574
1347.439974	248.884084	5.61697E-06	0.007568526
1347.479974	210.512618	4.75098E-06	0.006401846
1347.519974	180.21082	4.06711E-06	0.005480508
1347.559974	162.45199	3.66632E-06	0.00494058
1347.599974	143.38377	3.23597E-06	0.004360796
1347.639974	127.052484	2.8674E-06	0.00386422
1347.679974	138.109688	3.11694E-06	0.004200642
1347.719974	170.053859	3.83788E-06	0.005172386
1347.759974	188.27473	4.2491E-06	0.005726765
1347.799974	174.025585	3.92752E-06	0.005293505
1347.839974	172.442168	3.89178E-06	0.005245496

1347.879974	174.229488	3.93212E-06	0.005300022
1347.919974	168.80156	3.80962E-06	0.005135058
1347.959974	163.480343	3.68952E-06	0.00497333
1347.999974	164.539305	3.71342E-06	0.005005694
1348.039974	187.570298	4.2332E-06	0.005706523
1348.079974	215.381212	4.86085E-06	0.00655282
1348.119974	233.782531	5.27615E-06	0.007112878
1348.159974	241.875976	5.4588E-06	0.007359341
1348.199974	232.643364	5.25044E-06	0.007078639
1348.239974	226.65405	5.11527E-06	0.006896606
1348.279974	233.783119	5.27616E-06	0.00711374
1348.319974	238.270725	5.37744E-06	0.007250508
1348.359974	234.392258	5.28991E-06	0.007132699
1348.399974	237.249498	5.35439E-06	0.00721986
1348.439974	226.024729	5.10106E-06	0.006878478
1348.479974	176.080067	3.97388E-06	0.0053587
1348.519974	149.03374	3.36348E-06	0.004535725
1348.559974	150.52297	3.39709E-06	0.004581185
1348.599974	158.480591	3.57669E-06	0.004823519
1348.639974	176.604592	3.98572E-06	0.005375301
1348.679974	194.782094	4.39596E-06	0.005928744
1348.719974	197.50726	4.45746E-06	0.00601187
1348.759974	187.447768	4.23044E-06	0.005705841
1348.799974	191.337193	4.31821E-06	0.005824407
1348.839974	202.325319	4.5662E-06	0.006159074
1348.879974	203.465014	4.59192E-06	0.006193952
1348.919974	204.327212	4.61138E-06	0.006220383
1348.959974	199.708003	4.50713E-06	0.00607994
1348.999974	187.144571	4.22359E-06	0.005697626
1349.039974	170.464046	3.84714E-06	0.00518994
1349.079974	184.509906	4.16413E-06	0.005617747
1349.119974	212.044131	4.78554E-06	0.006456269
1349.159974	222.246932	5.0158E-06	0.006767122
1349.199974	218.755266	4.937E-06	0.006661003
1349.239974	210.852442	4.75865E-06	0.006420555
1349.279974	241.078024	5.4408E-06	0.007341156
1349.319974	251.509205	5.67621E-06	0.007659027
1349.359974	239.809063	5.41216E-06	0.007302947
1349.399974	232.343182	5.24366E-06	0.007075797
1349.439974	225.244063	5.08344E-06	0.006859804
1349.479974	194.672632	4.39349E-06	0.005928927
1349.519974	164.802501	3.71936E-06	0.005019355
1349.559974	159.13529	3.59146E-06	0.004846893
1349.599974	175.047501	3.95058E-06	0.0053317
1349.639974	192.995805	4.35565E-06	0.005878555
1349.679974	172.37894	3.89035E-06	0.005250731

1349.719974	160.891799	3.6311E-06	0.004900973
1349.759974	162.474618	3.66683E-06	0.004949335
1349.799974	161.226855	3.63867E-06	0.004911471
1349.839974	165.524713	3.73566E-06	0.005042546
1349.879974	167.181178	3.77305E-06	0.00509316
1349.919974	149.108003	3.36516E-06	0.004542697
1349.959974	155.554515	3.51065E-06	0.004739235
1349.999974	176.966563	3.99389E-06	0.00539175
1350.039974	192.059943	4.33453E-06	0.005851783
1350.079974	212.463707	4.79501E-06	0.006473647
1350.119974	237.384542	5.35744E-06	0.007233185
1350.159974	251.952954	5.68623E-06	0.007677316
1350.199974	223.245194	5.03833E-06	0.006802757
1350.239974	215.16211	4.85591E-06	0.006556643
1350.279974	220.952578	4.98659E-06	0.006733295
1350.319974	227.246116	5.12863E-06	0.006925289
1350.359974	226.900599	5.12083E-06	0.006914965
1350.399974	202.875159	4.57861E-06	0.006182954
1350.439974	187.470426	4.23095E-06	0.005713639
1350.479974	202.664687	4.57386E-06	0.006176906
1350.519974	184.070913	4.15422E-06	0.005610363
1350.559974	175.728087	3.96594E-06	0.005356237
1350.599974	175.988982	3.97183E-06	0.005364348
1350.639974	188.482799	4.25379E-06	0.005745345
1350.679974	212.831309	4.80331E-06	0.006487729
1350.719974	231.564793	5.22609E-06	0.007058991
1350.759974	226.074188	5.10218E-06	0.00689182
1350.799974	197.246008	4.45157E-06	0.006013177
1350.839974	173.957562	3.92598E-06	0.005303371
1350.879974	187.948898	4.24174E-06	0.005730088
1350.919974	194.48043	4.38915E-06	0.005929394
1350.959974	192.616413	4.34708E-06	0.005872737
1350.999974	194.552501	4.39078E-06	0.005931942
1351.039974	189.638842	4.27988E-06	0.005782295
1351.079974	183.043486	4.13104E-06	0.005581361
1351.119974	176.3301	3.97952E-06	0.005376815
1351.159974	184.860296	4.17204E-06	0.005637093
1351.199974	208.211575	4.69905E-06	0.00634935
1351.239974	245.396333	5.53825E-06	0.007483509
1351.279974	268.363791	6.0566E-06	0.008184158
1351.319974	268.010669	6.04863E-06	0.008173631
1351.359974	254.256919	5.73822E-06	0.007754407
1351.399974	226.904239	5.12091E-06	0.006920401
1351.439974	203.914917	4.60208E-06	0.006219429
1351.479974	191.978508	4.33269E-06	0.00585554
1351.519974	181.87712	4.10471E-06	0.005547602

1351.559974	171.797948	3.87724E-06	0.005240323
1351.599974	164.512672	3.71282E-06	0.00501825
1351.639974	165.806428	3.74202E-06	0.005057864
1351.679974	179.066112	4.04127E-06	0.005462507
1351.719974	188.232914	4.24815E-06	0.005742316
1351.759974	184.734117	4.16919E-06	0.005635746
1351.799974	182.224001	4.11254E-06	0.005559334
1351.839974	187.341146	4.22803E-06	0.005715618
1351.879974	194.895671	4.39852E-06	0.005946276
1351.919974	200.741009	4.53044E-06	0.006124799
1351.959974	209.48799	4.72785E-06	0.006391867
1351.999974	205.941859	4.64782E-06	0.006283854
1352.039974	188.787868	4.26068E-06	0.005760609
1352.079974	204.189884	4.60828E-06	0.006230765
1352.119974	213.020352	4.80757E-06	0.006500415
1352.159974	230.591348	5.20413E-06	0.00703681
1352.199974	261.355268	5.89842E-06	0.007975849
1352.239974	255.439298	5.76491E-06	0.007795541
1352.279974	242.279894	5.46792E-06	0.007394158
1352.319974	249.749685	5.6365E-06	0.007622355
1352.359974	244.850008	5.52592E-06	0.007473038
1352.399974	227.931828	5.1441E-06	0.006956886
1352.439974	201.579955	4.54938E-06	0.006152762
1352.479974	196.501467	4.43476E-06	0.00599793
1352.519974	194.528072	4.39023E-06	0.005937871
1352.559974	183.195943	4.13448E-06	0.005592128
1352.599974	153.196017	3.45742E-06	0.004676507
1352.639974	154.870881	3.49522E-06	0.004727774
1352.679974	171.670941	3.87437E-06	0.005240788
1352.719974	188.859751	4.2623E-06	0.0057657
1352.759974	202.558408	4.57146E-06	0.00618409
1352.799974	205.033866	4.62733E-06	0.00625985
1352.839974	196.939635	4.44465E-06	0.006012905
1352.879974	187.292864	4.22694E-06	0.005718541
1352.919974	184.356979	4.16068E-06	0.005629067
1352.959974	187.008812	4.22053E-06	0.005710206
1352.999974	186.305991	4.20467E-06	0.005688914
1353.039974	198.416545	4.47799E-06	0.006058893
1353.079974	209.246306	4.7224E-06	0.006389782
1353.119974	217.601109	4.91095E-06	0.00664511
1353.159974	234.942948	5.30234E-06	0.007174908
1353.199974	274.691417	6.1994E-06	0.008389032
1353.239974	329.414851	7.43443E-06	0.010060574
1353.279974	366.899801	8.28042E-06	0.011205723
1353.319974	365.824698	8.25615E-06	0.011173218
1353.359974	335.891348	7.5806E-06	0.01025928

1353.399974	297.485704	6.71384E-06	0.009086508
1353.439974	279.899189	6.31693E-06	0.008549592
1353.479974	269.466895	6.08149E-06	0.008231178
1353.519974	228.630836	5.15988E-06	0.006984
1353.559974	171.397995	3.86821E-06	0.00523586
1353.599974	128.982546	2.91096E-06	0.003940271
1353.639974	104.763575	2.36437E-06	0.003200503
1353.679974	101.608902	2.29317E-06	0.00310422
1353.719974	136.126246	3.07218E-06	0.004158871
1353.759974	163.484585	3.68962E-06	0.004994859
1353.799974	158.969854	3.58773E-06	0.004857066
1353.839974	153.81406	3.47137E-06	0.004699678
1353.879974	175.458818	3.95986E-06	0.005361177
1353.919974	193.476732	4.3665E-06	0.005911892
1353.959974	191.543278	4.32286E-06	0.005852986
1353.999974	196.567173	4.43625E-06	0.006006679
1354.039974	200.121568	4.51647E-06	0.006115474
1354.079974	188.606493	4.25659E-06	0.005763758
1354.119974	183.273309	4.13622E-06	0.005600943
1354.159974	214.007015	4.82984E-06	0.006540376
1354.199974	251.233084	5.66998E-06	0.007678288
1354.239974	273.97146	6.18315E-06	0.008373475
1354.279974	303.996027	6.86077E-06	0.009291399
1354.319974	315.540809	7.12132E-06	0.009644541
1354.359974	301.973066	6.81511E-06	0.009230114
1354.399974	273.033423	6.16198E-06	0.008345791
1354.439974	227.529516	5.13502E-06	0.006955082
1354.479974	201.694261	4.55196E-06	0.006165537
1354.519974	200.035919	4.51453E-06	0.006115024
1354.559974	180.179852	4.06641E-06	0.005508194
1354.599974	165.915793	3.74449E-06	0.005072284
1354.639974	169.504653	3.82548E-06	0.005182153
1354.679974	180.497273	4.07357E-06	0.005518386
1354.719974	182.209209	4.11221E-06	0.00557089
1354.759974	168.897388	3.81178E-06	0.005164045
1354.799974	168.560604	3.80418E-06	0.0051539
1354.839974	173.455996	3.91466E-06	0.005303738
1354.879974	192.297383	4.33988E-06	0.005880022
1354.919974	221.452617	4.99788E-06	0.006771724
1354.959974	214.379851	4.83825E-06	0.006555641
1354.999974	205.625262	4.64068E-06	0.006288116
1355.039974	181.621277	4.09894E-06	0.005554227
1355.079974	191.591808	4.32396E-06	0.005859312
1355.119974	231.021229	5.21383E-06	0.007065362
1355.159974	255.367622	5.76329E-06	0.007810182
1355.199974	280.333312	6.32673E-06	0.008573988

1355.239974	324.363679	7.32044E-06	0.009920948
1355.279974	337.641927	7.62011E-06	0.01032738
1355.319974	313.909059	7.08449E-06	0.009601751
1355.359974	282.586657	6.37759E-06	0.008643926
1355.399974	256.078471	5.77933E-06	0.00783331
1355.439974	234.020019	5.28151E-06	0.007158764
1355.479974	210.421769	4.74893E-06	0.006437074
1355.519974	210.623137	4.75347E-06	0.006443425
1355.559974	196.999593	4.44601E-06	0.006026828
1355.599974	184.484697	4.16356E-06	0.005644125
1355.639974	178.79127	4.03507E-06	0.005470102
1355.679974	184.975461	4.17464E-06	0.005659474
1355.719974	174.573449	3.93988E-06	0.005341373
1355.759974	180.317646	4.06952E-06	0.00551729
1355.799974	199.51966	4.50288E-06	0.006105006
1355.839974	193.682225	4.37114E-06	0.005926564
1355.879974	175.151515	3.95293E-06	0.005359693
1355.919974	162.326008	3.66347E-06	0.004967375
1355.959974	159.318587	3.5956E-06	0.004875488
1355.999974	152.753883	3.44744E-06	0.004674732
1356.039974	151.578461	3.42091E-06	0.004638897
1356.079974	165.914899	3.74447E-06	0.005077798
1356.119974	202.074983	4.56055E-06	0.006184654
1356.159974	222.239893	5.01564E-06	0.006802017
1356.199974	236.769324	5.34355E-06	0.007246928
1356.239974	261.564198	5.90314E-06	0.008006074
1356.279974	290.072756	6.54654E-06	0.008878938
1356.319974	286.998734	6.47716E-06	0.008785104
1356.359974	277.039712	6.2524E-06	0.008480506
1356.399974	269.210336	6.0757E-06	0.008241082
1356.439974	257.540673	5.81233E-06	0.007884083
1356.479974	231.562141	5.22603E-06	0.007089012
1356.519974	193.268872	4.36181E-06	0.005916881
1356.559974	169.591411	3.82744E-06	0.005192155
1356.599974	176.277718	3.97834E-06	0.005397019
1356.639974	190.033848	4.2888E-06	0.005818357
1356.679974	178.730255	4.03369E-06	0.00547243
1356.719974	175.26715	3.95554E-06	0.005366554
1356.759974	179.849681	4.05896E-06	0.00550703
1356.799974	182.68274	4.12289E-06	0.005593944
1356.839974	171.942246	3.8805E-06	0.005265213
1356.879974	171.83295	3.87803E-06	0.005262022
1356.919974	171.099965	3.86149E-06	0.00523973
1356.959974	185.934694	4.19629E-06	0.005694194
1356.999974	217.390239	4.90619E-06	0.006657706
1357.039974	224.397503	5.06434E-06	0.006872511

1357.079974	224.482861	5.06627E-06	0.006875327
1357.119974	256.134353	5.7806E-06	0.007844962
1357.159974	281.905305	6.36221E-06	0.008634537
1357.199974	320.798363	7.23997E-06	0.00982609
1357.239974	373.221278	8.42308E-06	0.011432147
1357.279974	382.127162	8.62408E-06	0.011705288
1357.319974	336.985335	7.60529E-06	0.010322812
1357.359974	301.483073	6.80405E-06	0.009235549
1357.399974	278.203725	6.27867E-06	0.008522667
1357.439974	263.772807	5.95298E-06	0.00808082
1357.479974	250.342786	5.64989E-06	0.00766961
1357.519974	219.581913	4.95566E-06	0.006727405
1357.559974	197.917478	4.46672E-06	0.006063843
1357.599974	183.970868	4.15197E-06	0.005636709
1357.639974	177.150937	3.99805E-06	0.005427912
1357.679974	174.315332	3.93405E-06	0.005341187
1357.719974	176.038417	3.97294E-06	0.005394143
1357.759974	170.379398	3.84523E-06	0.005220894
1357.799974	166.388519	3.75516E-06	0.005098752
1357.839974	177.035441	3.99544E-06	0.005425173
1357.879974	209.697634	4.73258E-06	0.00642628
1357.919974	231.608402	5.22708E-06	0.007097955
1357.959974	238.579559	5.38441E-06	0.007311811
1357.999974	227.7196	5.13931E-06	0.006979189
1358.039974	220.735372	4.98169E-06	0.006765334
1358.079974	221.722738	5.00397E-06	0.006795796
1358.119974	234.70044	5.29686E-06	0.007193774
1358.159974	254.81504	5.75082E-06	0.007810534
1358.199974	262.037808	5.91383E-06	0.008032161
1358.239974	283.81799	6.40538E-06	0.008700039
1358.279974	296.211853	6.68509E-06	0.009080222
1358.319974	295.175028	6.66169E-06	0.009048706
1358.359974	292.345169	6.59782E-06	0.008962219
1358.399974	274.008823	6.184E-06	0.008400342
1358.439974	254.331849	5.73992E-06	0.007797331
1358.479974	229.723506	5.18454E-06	0.007043093
1358.519974	214.847236	4.8488E-06	0.006587196
1358.559974	188.562648	4.2556E-06	0.005781483
1358.599974	188.041726	4.24384E-06	0.005765681
1358.639974	190.320197	4.29526E-06	0.005835714
1358.679974	156.601478	3.53428E-06	0.004801952
1358.719974	158.255834	3.57161E-06	0.004852823
1358.759974	177.690883	4.01024E-06	0.005448948
1358.799974	186.030819	4.19846E-06	0.005704863
1358.839974	184.375598	4.1611E-06	0.00565427
1358.879974	154.569946	3.48843E-06	0.004740355

1358.919974	143.0911	3.22937E-06	0.004388451
1358.959974	146.576226	3.30802E-06	0.004495468
1358.999974	155.159724	3.50174E-06	0.004758863
1359.039974	191.884864	4.33057E-06	0.005885423
1359.079974	218.058325	4.92127E-06	0.006688403
1359.119974	236.500026	5.33748E-06	0.00725427
1359.159974	253.417519	5.71928E-06	0.007773417
1359.199974	275.075695	6.20808E-06	0.008438016
1359.239974	298.964006	6.7472E-06	0.009171066
1359.279974	302.651399	6.83042E-06	0.009284454
1359.319974	320.194662	7.22635E-06	0.009822919
1359.359974	317.620617	7.16825E-06	0.009744239
1359.399974	273.035294	6.16203E-06	0.008376658
1359.439974	238.130167	5.37427E-06	0.007305992
1359.479974	220.178918	4.96913E-06	0.006755435
1359.519974	185.438299	4.18508E-06	0.005689705
1359.559974	161.027012	3.63416E-06	0.004940852
1359.599974	165.53419	3.73588E-06	0.005079297
1359.639974	160.845362	3.63006E-06	0.004935569
1359.679974	142.944649	3.22606E-06	0.004386411
1359.719974	138.570641	3.12735E-06	0.004252315
1359.759974	159.529452	3.60036E-06	0.004895622
1359.799974	175.44619	3.95958E-06	0.005384232
1359.839974	171.81821	3.8777E-06	0.005273048
1359.879974	169.397454	3.82306E-06	0.005198909
1359.919974	170.597794	3.85015E-06	0.005235902
1359.959974	171.424887	3.86882E-06	0.005261442
1359.999974	185.45361	4.18543E-06	0.005692184
1360.039974	199.070669	4.49275E-06	0.006110316
1360.079974	200.716951	4.5299E-06	0.006161029
1360.119974	201.591751	4.54965E-06	0.006188063
1360.159974	221.196702	4.9921E-06	0.006790057
1360.199974	237.360427	5.35689E-06	0.007286447
1360.239974	254.288593	5.73894E-06	0.007806335
1360.279974	273.755212	6.17827E-06	0.008404182
1360.319974	282.839655	6.3833E-06	0.008683326
1360.359974	278.882575	6.29399E-06	0.008562094
1360.399974	278.220035	6.27904E-06	0.008542004
1360.439974	246.588455	5.56516E-06	0.007571063
1360.479974	201.665294	4.5513E-06	0.006191959
1360.519974	169.88512	3.83407E-06	0.00521633
1360.559974	158.458108	3.57618E-06	0.004865606
1360.599974	149.359418	3.37083E-06	0.004586357
1360.639974	134.126558	3.02705E-06	0.004118725
1360.679974	140.16967	3.16343E-06	0.004304422
1360.719974	153.963045	3.47473E-06	0.004728137

1360.759974	161.471567	3.64419E-06	0.004958866
1360.799974	170.836388	3.85554E-06	0.005246618
1360.839974	158.506956	3.57728E-06	0.004868107
1360.879974	158.720129	3.58209E-06	0.004874798
1360.919974	173.069274	3.90593E-06	0.005315661
1360.959974	172.288636	3.88831E-06	0.00529184
1360.999974	158.831336	3.5846E-06	0.004878643
1361.039974	175.424363	3.95908E-06	0.005388471
1361.079974	213.488363	4.81813E-06	0.006557867
1361.119974	243.775952	5.50168E-06	0.007488452
1361.159974	245.509475	5.54081E-06	0.007541925
1361.199974	248.331903	5.60451E-06	0.007628852
1361.239974	263.021609	5.93603E-06	0.008080363
1361.279974	283.468628	6.39749E-06	0.008708778
1361.319974	278.436253	6.28392E-06	0.008554424
1361.359974	258.679323	5.83803E-06	0.007947663
1361.399974	232.740643	5.25263E-06	0.007150933
1361.439974	202.335442	4.56643E-06	0.006216919
1361.479974	192.789192	4.35098E-06	0.005923777
1361.519974	177.52559	4.00651E-06	0.005454937
1361.559974	174.35231	3.93489E-06	0.005357587
1361.599974	170.326507	3.84403E-06	0.005234034
1361.639974	175.687746	3.96503E-06	0.00539894
1361.679974	194.020771	4.37878E-06	0.005962495
1361.719974	209.886078	4.73684E-06	0.006450245
1361.759974	200.360146	4.52185E-06	0.006157674
1361.799974	184.706372	4.16857E-06	0.005676752
1361.839974	182.190605	4.11179E-06	0.005599598
1361.879974	179.890912	4.05989E-06	0.005529079
1361.919974	178.750789	4.03416E-06	0.005494198
1361.959974	192.547701	4.34553E-06	0.005918443
1361.999974	200.985876	4.53597E-06	0.006177993
1362.039974	183.739713	4.14675E-06	0.005648038
1362.079974	165.289719	3.73036E-06	0.005081047
1362.119974	165.942713	3.7451E-06	0.00510127
1362.159974	190.576475	4.30105E-06	0.005858712
1362.199974	232.328649	5.24333E-06	0.007142469
1362.239974	257.579354	5.81321E-06	0.007918983
1362.279974	287.641192	6.49166E-06	0.00884346
1362.319974	289.056129	6.52359E-06	0.008887223
1362.359974	259.498925	5.85653E-06	0.007978701
1362.399974	225.312197	5.08498E-06	0.00692778
1362.439974	195.274746	4.40708E-06	0.006004381
1362.479974	182.236765	4.11283E-06	0.005603648
1362.519974	195.989104	4.4232E-06	0.0060267
1362.559974	193.471437	4.36638E-06	0.005949456

1362.599974	172.978273	3.90388E-06	0.005319425
1362.639974	183.5063	4.14148E-06	0.005643348
1362.679974	182.976653	4.12953E-06	0.005627225
1362.719974	179.043127	4.04075E-06	0.005506416
1362.759974	176.57829	3.98513E-06	0.00543077
1362.799974	177.757732	4.01174E-06	0.005467205
1362.839974	187.162	4.22399E-06	0.005756616
1362.879974	180.495471	4.07353E-06	0.005551734
1362.919974	166.627007	3.76054E-06	0.005125314
1362.959974	183.181434	4.13415E-06	0.005634681
1362.999974	176.449461	3.98222E-06	0.005427764
1363.039974	170.300107	3.84344E-06	0.005238757
1363.079974	168.296366	3.79821E-06	0.00517727
1363.119974	161.205415	3.63818E-06	0.004959278
1363.159974	197.606345	4.4597E-06	0.006079284
1363.199974	260.346933	5.87567E-06	0.00800971
1363.239974	273.396194	6.17017E-06	0.008411424
1363.279974	266.239998	6.00867E-06	0.008191494
1363.319974	269.527986	6.08287E-06	0.0082929
1363.359974	237.643134	5.36327E-06	0.007312074
1363.399974	218.023813	4.92049E-06	0.006708601
1363.439974	220.821306	4.98363E-06	0.006794879
1363.479974	193.651526	4.37045E-06	0.005959015
1363.519974	161.562862	3.64625E-06	0.004971733
1363.559974	152.065435	3.43191E-06	0.004679608
1363.599974	164.329191	3.70868E-06	0.005057157
1363.639974	163.279234	3.68498E-06	0.005024993
1363.679974	154.832625	3.49436E-06	0.004765184
1363.719974	135.504198	3.05814E-06	0.004170448
1363.759974	126.455598	2.85393E-06	0.003892071
1363.799974	131.761752	2.97368E-06	0.004055504
1363.839974	136.26358	3.07528E-06	0.004194189
1363.879974	138.513297	3.12605E-06	0.00426356
1363.919974	144.743413	3.26666E-06	0.004455459
1363.959974	157.962378	3.56499E-06	0.004862505
1363.999974	159.286248	3.59487E-06	0.004903401
1364.039974	163.140153	3.68185E-06	0.005022185
1364.079974	176.474509	3.98278E-06	0.005432836
1364.119974	214.727415	4.8461E-06	0.00661066
1364.159974	226.180748	5.10458E-06	0.00696347
1364.199974	209.214292	4.72168E-06	0.006441309
1364.239974	200.493327	4.52486E-06	0.006172988
1364.279974	214.089398	4.8317E-06	0.006591791
1364.319974	233.927148	5.27941E-06	0.007202804
1364.359974	223.183645	5.03694E-06	0.006872205
1364.399974	200.804152	4.53187E-06	0.006183283

1364.439974	222.705734	5.02616E-06	0.006857891
1364.479974	215.979611	4.87436E-06	0.006650965
1364.519974	188.16349	4.24659E-06	0.005794554
1364.559974	185.278066	4.18147E-06	0.005705864
1364.599974	178.896355	4.03744E-06	0.005509493
1364.639974	179.064428	4.04123E-06	0.00551483
1364.679974	164.919756	3.72201E-06	0.005079351
1364.719974	157.496475	3.55448E-06	0.004850864
1364.759974	173.44799	3.91448E-06	0.005342325
1364.799974	189.01362	4.26577E-06	0.005821928
1364.839974	201.792889	4.55418E-06	0.006215733
1364.879974	190.652397	4.30276E-06	0.00587275
1364.919974	172.448937	3.89193E-06	0.005312176
1364.959974	166.098806	3.74862E-06	0.005116714
1364.999974	163.337189	3.68629E-06	0.00503179
1365.039974	156.273373	3.52687E-06	0.004814322
1365.079974	183.34063	4.13774E-06	0.00564835
1365.119974	207.310534	4.67871E-06	0.006387
1365.159974	205.617861	4.64051E-06	0.006335037
1365.199974	207.064606	4.67316E-06	0.006379798
1365.239974	215.918268	4.87297E-06	0.00665278
1365.279974	247.580688	5.58755E-06	0.007628572
1365.319974	273.114784	6.16382E-06	0.008415587
1365.359974	250.697461	5.65789E-06	0.00772506
1365.399974	211.879405	4.78182E-06	0.006529101
1365.439974	173.262131	3.91028E-06	0.005339259
1365.479974	148.806075	3.35835E-06	0.004585754
1365.519974	153.591226	3.46634E-06	0.004733357
1365.559974	186.602324	4.21135E-06	0.005750857
1365.599974	203.447367	4.59152E-06	0.006270185
1365.639974	172.937594	3.90296E-06	0.005330039
1365.679974	157.196844	3.54771E-06	0.004845042
1365.719974	153.817932	3.47146E-06	0.004741037
1365.759974	160.64605	3.62556E-06	0.004951641
1365.799974	168.58488	3.80473E-06	0.005196495
1365.839974	172.687492	3.89732E-06	0.00532311
1365.879974	174.776257	3.94446E-06	0.005387654
1365.919974	178.551198	4.02965E-06	0.005504182
1365.959974	171.246465	3.86479E-06	0.005279154
1365.999974	175.409741	3.95875E-06	0.005407657
1366.039974	200.052173	4.5149E-06	0.006167532
1366.079974	190.712231	4.30411E-06	0.005879758
1366.119974	173.739084	3.92105E-06	0.005356624
1366.159974	182.54139	4.1197E-06	0.005628176
1366.199974	195.990517	4.42323E-06	0.006043021
1366.239974	209.340715	4.72453E-06	0.006454839

1366.279974	235.342301	5.31135E-06	0.007256788
1366.319974	235.918606	5.32435E-06	0.007274772
1366.359974	217.027727	4.89801E-06	0.00669245
1366.399974	227.262205	5.12899E-06	0.007008254
1366.439974	248.270031	5.60311E-06	0.007656312
1366.479974	238.250581	5.37698E-06	0.007347541
1366.519974	213.261395	4.81301E-06	0.006577078
1366.559974	182.906355	4.12794E-06	0.00564108
1366.599974	167.722199	3.78526E-06	0.005172931
1366.639974	171.006559	3.85938E-06	0.005274383
1366.679974	176.056429	3.97335E-06	0.005430296
1366.719974	202.898291	4.57913E-06	0.006258391
1366.759974	222.837097	5.02912E-06	0.006873604
1366.799974	214.682795	4.84509E-06	0.006622271
1366.839974	184.241036	4.15806E-06	0.005683407
1366.879974	163.869981	3.69832E-06	0.005055156
1366.919974	148.872073	3.35984E-06	0.004592626
1366.959974	143.299681	3.23407E-06	0.00442085
1366.999974	145.525361	3.2843E-06	0.004489644
1367.039974	171.860284	3.87865E-06	0.005302266
1367.079974	214.974251	4.85167E-06	0.00663262
1367.119974	236.608428	5.33992E-06	0.007300315
1367.159974	227.543677	5.13534E-06	0.007020837
1367.199974	242.460809	5.472E-06	0.007481322
1367.239974	264.626198	5.97224E-06	0.008165492
1367.279974	302.326417	6.82309E-06	0.009329069
1367.319974	326.620867	7.37138E-06	0.010079032
1367.359974	305.746448	6.90027E-06	0.009435155
1367.399974	272.729301	6.15512E-06	0.008416512
1367.439974	255.505951	5.76641E-06	0.007885224
1367.479974	209.505059	4.72824E-06	0.00646577
1367.519974	170.912237	3.85725E-06	0.005274868
1367.559974	154.654418	3.49033E-06	0.004773242
1367.599974	142.915324	3.2254E-06	0.004411057
1367.639974	140.800974	3.17768E-06	0.004345925
1367.679974	142.789053	3.22255E-06	0.004407417
1367.719974	144.928521	3.27083E-06	0.004473586
1367.759974	149.080742	3.36454E-06	0.00460189
1367.799974	157.595899	3.55672E-06	0.004864881
1367.839974	171.511376	3.87077E-06	0.005294598
1367.879974	167.696317	3.78467E-06	0.005176977
1367.919974	153.733569	3.46955E-06	0.00474607
1367.959974	151.587063	3.42111E-06	0.00467994
1367.999974	174.169483	3.93076E-06	0.005377283
1368.039974	193.85106	4.37495E-06	0.005985104
1368.079974	211.892704	4.78212E-06	0.006542327

1368.119974	215.890905	4.87236E-06	0.006665969
1368.159974	220.897692	4.98535E-06	0.006820761
1368.199974	235.326164	5.31098E-06	0.007266488
1368.239974	253.016317	5.71023E-06	0.007812959
1368.279974	266.280501	6.00958E-06	0.008222788
1368.319974	257.892609	5.82028E-06	0.007964001
1368.359974	236.857375	5.34554E-06	0.007314624
1368.399974	229.162624	5.17188E-06	0.007077202
1368.439974	195.082939	4.40275E-06	0.006024899
1368.479974	162.637218	3.6705E-06	0.005023
1368.519974	176.589776	3.98539E-06	0.005454079
1368.559974	187.292827	4.22694E-06	0.005784818
1368.599974	193.207954	4.36043E-06	0.00596769
1368.639974	181.427624	4.09457E-06	0.00560399
1368.679974	190.519632	4.29976E-06	0.005884999
1368.719974	224.108861	5.05782E-06	0.006922746
1368.759974	240.026126	5.41706E-06	0.007414649
1368.799974	240.095744	5.41863E-06	0.007417016
1368.839974	222.779476	5.02782E-06	0.006882284
1368.879974	215.760125	4.86941E-06	0.006665632
1368.919974	225.011859	5.0782E-06	0.006951655
1368.959974	208.87485	4.71401E-06	0.006453297
1368.999974	193.068802	4.35729E-06	0.005965135
1369.039974	202.782471	4.57652E-06	0.006265436
1369.079974	215.232329	4.85749E-06	0.006650298
1369.119974	239.217738	5.39881E-06	0.00739162
1369.159974	261.704654	5.90631E-06	0.008086683
1369.199974	285.43044	6.44177E-06	0.008820068
1369.239974	298.697966	6.7412E-06	0.009230316
1369.279974	305.063761	6.88486E-06	0.009427307
1369.319974	307.191488	6.93288E-06	0.009493337
1369.359974	292.676025	6.60529E-06	0.00904502
1369.399974	261.805745	5.90859E-06	0.008091224
1369.439974	237.176993	5.35275E-06	0.007330276
1369.479974	220.396085	4.97403E-06	0.006811838
1369.519974	188.880821	4.26278E-06	0.005837958
1369.559974	179.505807	4.0512E-06	0.005548356
1369.599974	165.725543	3.74019E-06	0.005122571
1369.639974	158.274394	3.57203E-06	0.004892399
1369.679974	163.033959	3.67945E-06	0.005039668
1369.719974	156.876198	3.54048E-06	0.004849462
1369.759974	155.902646	3.51851E-06	0.004819508
1369.799974	150.131614	3.38826E-06	0.00464124
1369.839974	143.795569	3.24527E-06	0.004445495
1369.879974	155.241943	3.50359E-06	0.004799504
1369.919974	168.694556	3.8072E-06	0.005215561

1369.959974	160.454012	3.62122E-06	0.004960931
1369.999974	158.084357	3.56774E-06	0.004887809
1370.039974	175.904701	3.96992E-06	0.005438955
1370.079974	207.10768	4.67413E-06	0.006403934
1370.119974	221.101976	4.98996E-06	0.006836849
1370.159974	225.926983	5.09886E-06	0.00698625
1370.199974	230.760023	5.20793E-06	0.007135909
1370.239974	244.425343	5.51634E-06	0.007558709
1370.279974	277.677378	6.26679E-06	0.008587259
1370.319974	260.57623	5.88084E-06	0.008058636
1370.359974	223.36663	5.04107E-06	0.006908086
1370.399974	214.417387	4.8391E-06	0.006631505
1370.439974	209.34188	4.72455E-06	0.006474719
1370.479974	199.172145	4.49504E-06	0.006160359
1370.519974	184.33086	4.16009E-06	0.005701487
1370.559974	164.621757	3.71528E-06	0.005092019
1370.599974	162.838262	3.67503E-06	0.005037
1370.639974	167.484518	3.77989E-06	0.005180871
1370.679974	165.243325	3.72931E-06	0.005111693
1370.719974	177.181039	3.99873E-06	0.005481138
1370.759974	194.775218	4.39581E-06	0.006025594
1370.799974	208.97509	4.71628E-06	0.006465072
1370.839974	205.978952	4.64866E-06	0.006372566
1370.879974	197.846147	4.46511E-06	0.006121133
1370.919974	197.442253	4.456E-06	0.006108815
1370.959974	209.561755	4.72952E-06	0.006483978
1370.999974	216.665473	4.88984E-06	0.006703968
1371.039974	211.610284	4.77575E-06	0.006547743
1371.079974	205.743161	4.64334E-06	0.006366386
1371.119974	216.934199	4.8959E-06	0.00671287
1371.159974	235.82805	5.32231E-06	0.007297739
1371.199974	243.934557	5.50526E-06	0.007548817
1371.239974	273.81349	6.17959E-06	0.0084737
1371.279974	310.354449	7.00427E-06	0.009604812
1371.319974	320.265854	7.22795E-06	0.009911838
1371.359974	297.852554	6.72212E-06	0.009218443
1371.399974	261.094791	5.89255E-06	0.008081037
1371.439974	235.582086	5.31676E-06	0.007291617
1371.479974	229.48018	5.17905E-06	0.007102961
1371.519974	229.118285	5.17088E-06	0.007091966
1371.559974	207.708904	4.6877E-06	0.006429463
1371.599974	185.179877	4.17925E-06	0.005732262
1371.639974	179.483766	4.0507E-06	0.0055561
1371.679974	171.735348	3.87583E-06	0.005316395
1371.719974	162.963582	3.67786E-06	0.005044995
1371.759974	191.853861	4.32987E-06	0.005939548

1371.799974	220.241356	4.97054E-06	0.006818588
1371.839974	199.94634	4.51251E-06	0.006190442
1371.879974	177.860492	4.01406E-06	0.005506813
1371.919974	166.836	3.76526E-06	0.00516563
1371.959974	181.000061	4.08492E-06	0.005604346
1371.999974	190.409429	4.29728E-06	0.005895862
1372.039974	193.912062	4.37633E-06	0.006004493
1372.079974	206.251289	4.6548E-06	0.006386764
1372.119974	221.497793	4.9989E-06	0.006859086
1372.159974	251.106056	5.66711E-06	0.007776187
1372.199974	258.312118	5.82974E-06	0.007999576
1372.239974	250.218305	5.64708E-06	0.007749147
1372.279974	270.474939	6.10424E-06	0.00837673
1372.319974	284.862072	6.42894E-06	0.008822563
1372.359974	262.486112	5.92395E-06	0.008129786
1372.399974	240.356346	5.42451E-06	0.007444595
1372.439974	210.899679	4.75971E-06	0.006532419
1372.479974	176.99125	3.99445E-06	0.005482297
1372.519974	148.524139	3.35198E-06	0.004600664
1372.559974	142.683095	3.22016E-06	0.004419861
1372.599974	159.783248	3.60609E-06	0.004949713
1372.639974	173.929252	3.92534E-06	0.00538808
1372.679974	175.023355	3.95003E-06	0.005422132
1372.719974	166.586824	3.75963E-06	0.005160923
1372.759974	182.876738	4.12727E-06	0.005665756
1372.799974	197.327605	4.45341E-06	0.00611364
1372.839974	185.54932	4.18759E-06	0.005748891
1372.879974	184.509388	4.16412E-06	0.005716837
1372.919974	205.780325	4.64418E-06	0.006376081
1372.959974	207.628463	4.68589E-06	0.006433533
1372.999974	198.913868	4.48921E-06	0.006163684
1373.039974	198.09891	4.47082E-06	0.00613861
1373.079974	195.027594	4.4015E-06	0.006043613
1373.119974	212.288842	4.79106E-06	0.006578705
1373.159974	249.307032	5.62651E-06	0.007726102
1373.199974	276.355982	6.23697E-06	0.008564606
1373.239974	274.057292	6.18509E-06	0.008493615
1373.279974	268.480868	6.05924E-06	0.008321032
1373.319974	279.257298	6.30245E-06	0.008655278
1373.359974	305.251335	6.8891E-06	0.009461211
1373.399974	304.704001	6.87674E-06	0.009444521
1373.439974	263.030048	5.93622E-06	0.008153044
1373.479974	223.400917	5.04185E-06	0.006924877
1373.519974	197.037432	4.44686E-06	0.006107852
1373.559974	185.218124	4.18012E-06	0.005741639
1373.599974	171.666309	3.87427E-06	0.005321696

1373.639974	170.273563	3.84284E-06	0.005278675
1373.679974	158.720243	3.58209E-06	0.004920652
1373.719974	157.120238	3.54598E-06	0.00487119
1373.759974	155.915817	3.5188E-06	0.00483399
1373.799974	166.615122	3.76027E-06	0.005165861
1373.839974	189.483203	4.27637E-06	0.005875051
1373.879974	199.266344	4.49716E-06	0.006178563
1373.919974	182.660789	4.1224E-06	0.005663847
1373.959974	177.086821	3.9966E-06	0.005491172
1373.999974	182.274207	4.11367E-06	0.005652189
1374.039974	178.761652	4.0344E-06	0.005543429
1374.079974	179.227693	4.04492E-06	0.005558043
1374.119974	182.352124	4.11543E-06	0.005655099
1374.159974	191.637462	4.32499E-06	0.005943229
1374.199974	203.067695	4.58296E-06	0.006297897
1374.239974	230.119118	5.19347E-06	0.007137071
1374.279974	268.5021	6.05972E-06	0.00832775
1374.319974	292.963849	6.61179E-06	0.00908671
1374.359974	271.028926	6.11675E-06	0.00840661
1374.399974	244.732024	5.52326E-06	0.00759117
1374.439974	205.632971	4.64085E-06	0.006378569
1374.479974	168.432435	3.80129E-06	0.005224791
1374.519974	157.830031	3.562E-06	0.004896046
1374.559974	143.741236	3.24404E-06	0.004459127
1374.599974	131.624582	2.97058E-06	0.004083364
1374.639974	129.991623	2.93373E-06	0.004032822
1374.679974	153.822175	3.47155E-06	0.004772273
1374.719974	174.081203	3.92877E-06	0.005400959
1374.759974	183.721854	4.14635E-06	0.005700231
1374.799974	210.345656	4.74721E-06	0.006526462
1374.839974	225.785751	5.09567E-06	0.007005731
1374.879974	213.881164	4.827E-06	0.006636546
1374.919974	177.646781	4.00924E-06	0.005512385
1374.959974	168.542234	3.80376E-06	0.005230023
1374.999974	175.376506	3.958E-06	0.005442255
1375.039974	188.078586	4.24467E-06	0.005836593
1375.079974	204.378342	4.61253E-06	0.006342604
1375.119974	216.026799	4.87542E-06	0.006704293
1375.159974	248.626268	5.61115E-06	0.007716227
1375.199974	259.294043	5.85191E-06	0.00804754
1375.239974	274.985017	6.20603E-06	0.008534779
1375.279974	308.765873	6.96842E-06	0.009583523
1375.319974	321.807916	7.26276E-06	0.009988614
1375.359974	313.947119	7.08535E-06	0.009744906
1375.399974	291.693423	6.58311E-06	0.009054415
1375.439974	251.641569	5.6792E-06	0.007811398

1375.479974	203.267997	4.58748E-06	0.006309981
1375.519974	170.476352	3.84741E-06	0.005292195
1375.559974	156.227974	3.52585E-06	0.004850015
1375.599974	156.595459	3.53414E-06	0.004861565
1375.639974	171.197342	3.86369E-06	0.00531504
1375.679974	173.73967	3.92106E-06	0.005394127
1375.719974	163.440821	3.68863E-06	0.005074524
1375.759974	165.230308	3.72902E-06	0.005130234
1375.799974	170.298515	3.8434E-06	0.00528775
1375.839974	173.432057	3.91412E-06	0.005385203
1375.879974	170.887542	3.85669E-06	0.005306348
1375.919974	180.065731	4.06383E-06	0.005591509
1375.959974	178.843872	4.03626E-06	0.005553728
1375.999974	177.905559	4.01508E-06	0.005524751
1376.039974	208.879337	4.71412E-06	0.006486812
1376.079974	225.968031	5.09978E-06	0.00701771
1376.119974	228.830839	5.16439E-06	0.007106825
1376.159974	282.651752	6.37906E-06	0.008778602
1376.199974	298.047577	6.72652E-06	0.009257035
1376.239974	272.905855	6.15911E-06	0.008476407
1376.279974	295.039832	6.65864E-06	0.00916415
1376.319974	307.930538	6.94956E-06	0.009564823
1376.359974	272.914828	6.15931E-06	0.008477424
1376.399974	237.928521	5.36972E-06	0.007390876
1376.439974	201.866924	4.55586E-06	0.006270861
1376.479974	185.88957	4.19527E-06	0.005774703
1376.519974	187.179164	4.22437E-06	0.005814934
1376.559974	194.98329	4.4005E-06	0.006057554
1376.599974	197.65837	4.46087E-06	0.006140839
1376.639974	202.028817	4.55951E-06	0.006276802
1376.679974	200.230485	4.51892E-06	0.006221111
1376.719974	185.545961	4.18751E-06	0.005765034
1376.759974	194.647342	4.39292E-06	0.006047996
1376.799974	200.893804	4.53389E-06	0.006242264
1376.839974	195.911296	4.42145E-06	0.006087622
1376.879974	191.897564	4.33086E-06	0.005963075
1376.919974	170.508255	3.84813E-06	0.005298572
1376.959974	172.884992	3.90177E-06	0.005372586
1376.999974	177.427974	4.0043E-06	0.005513924
1377.039974	186.134757	4.2008E-06	0.005784673
1377.079974	203.789815	4.59925E-06	0.006333538
1377.119974	218.445569	4.93001E-06	0.006789218
1377.159974	228.571885	5.15855E-06	0.007104147
1377.199974	236.529333	5.33814E-06	0.007351683
1377.239974	241.780724	5.45665E-06	0.007515122
1377.279974	258.110507	5.82519E-06	0.008022924

1377.319974	270.071015	6.09513E-06	0.00839494
1377.359974	260.293117	5.87445E-06	0.008091237
1377.399974	225.056519	5.07921E-06	0.006996107
1377.439974	190.360073	4.29616E-06	0.005917705
1377.479974	161.944103	3.65485E-06	0.005034487
1377.519974	160.042727	3.61194E-06	0.004975521
1377.559974	162.804629	3.67427E-06	0.005061532
1377.599974	151.064122	3.40931E-06	0.004696661
1377.639974	142.234561	3.21004E-06	0.004422274
1377.679974	150.034564	3.38607E-06	0.004664922
1377.719974	167.113236	3.77151E-06	0.005196089
1377.759974	197.893293	4.46618E-06	0.006153319
1377.799974	199.798035	4.50916E-06	0.006212725
1377.839974	197.12554	4.44885E-06	0.006129802
1377.879974	201.39514	4.54521E-06	0.006262751
1377.919974	197.743284	4.46279E-06	0.006149368
1377.959974	209.168247	4.72064E-06	0.006504847
1377.999974	195.093337	4.40298E-06	0.006067313
1378.039974	167.124629	3.77177E-06	0.00519765
1378.079974	157.72889	3.55972E-06	0.004905581
1378.119974	177.640914	4.00911E-06	0.005525032
1378.159974	217.361524	4.90555E-06	0.006760628
1378.199974	259.921119	5.86606E-06	0.0080846
1378.239974	286.310416	6.46163E-06	0.008905673
1378.279974	304.80159	6.87895E-06	0.009481115
1378.319974	289.8966	6.54256E-06	0.009017745
1378.359974	245.16117	5.53295E-06	0.007626391
1378.399974	230.226052	5.19588E-06	0.007162003
1378.439974	209.739128	4.73352E-06	0.006524873
1378.479974	187.264107	4.22629E-06	0.005825856
1378.519974	162.452931	3.66634E-06	0.005054118
1378.559974	155.958134	3.51976E-06	0.004852197
1378.599974	149.832783	3.38152E-06	0.00466176
1378.639974	142.637253	3.21912E-06	0.004438013
1378.679974	160.718645	3.6272E-06	0.005000742
1378.719974	174.258749	3.93278E-06	0.005422199
1378.759974	190.370093	4.29639E-06	0.005923688
1378.799974	192.301497	4.33998E-06	0.00598396
1378.839974	173.041984	3.90532E-06	0.005384807
1378.879974	172.601245	3.89537E-06	0.005371247
1378.919974	177.206562	3.99931E-06	0.005514722
1378.959974	188.852115	4.26213E-06	0.005877306
1378.999974	181.220948	4.0899E-06	0.005639978
1379.039974	155.877083	3.51793E-06	0.004851364
1379.079974	169.28013	3.82042E-06	0.00526866
1379.119974	197.311931	4.45306E-06	0.006141298

1379.159974	224.102127	5.05767E-06	0.00697534
1379.199974	264.282836	5.9645E-06	0.008226232
1379.239974	286.519792	6.46635E-06	0.008918652
1379.279974	302.327024	6.8231E-06	0.009410965
1379.319974	304.800794	6.87893E-06	0.009488245
1379.359974	289.908274	6.54283E-06	0.009024912
1379.399974	259.230401	5.85047E-06	0.008070137
1379.439974	230.677903	5.20608E-06	0.007181473
1379.479974	200.388982	4.5225E-06	0.006238698
1379.519974	173.630245	3.91859E-06	0.005405777
1379.559974	168.445125	3.80157E-06	0.005244496
1379.599974	168.790323	3.80936E-06	0.005255396
1379.639974	163.260725	3.68457E-06	0.005083376
1379.679974	161.665413	3.64856E-06	0.00503385
1379.719974	162.787487	3.67389E-06	0.005068935
1379.759974	150.268643	3.39135E-06	0.004679254
1379.799974	151.067068	3.40937E-06	0.004704253
1379.839974	162.976529	3.67815E-06	0.005075263
1379.879974	176.893986	3.99225E-06	0.005508827
1379.919974	170.796866	3.85465E-06	0.005319105
1379.959974	165.303846	3.73068E-06	0.005148186
1379.999974	188.908582	4.2634E-06	0.005883497
1380.039974	207.84301	4.69073E-06	0.006473391
1380.079974	211.088166	4.76397E-06	0.006574654
1380.119974	224.252867	5.06107E-06	0.00698489
1380.159974	239.039491	5.39479E-06	0.007445671
1380.199974	235.815582	5.32203E-06	0.007345465
1380.239974	254.059505	5.73377E-06	0.007913977
1380.279974	278.675874	6.28933E-06	0.008681031
1380.319974	268.307301	6.05532E-06	0.008358282
1380.359974	249.630986	5.63382E-06	0.007776705
1380.399974	254.494028	5.74358E-06	0.007928432
1380.439974	221.922729	5.00849E-06	0.006913915
1380.479974	180.841918	4.08135E-06	0.005634222
1380.519974	174.194649	3.93133E-06	0.00542728
1380.559974	184.314569	4.15972E-06	0.005742747
1380.599974	178.614671	4.03108E-06	0.005565315
1380.639974	173.491964	3.91547E-06	0.005405857
1380.679974	149.54512	3.37503E-06	0.00465983
1380.719974	163.04982	3.67981E-06	0.005080783
1380.759974	199.002706	4.49121E-06	0.006201288
1380.799974	213.866839	4.82668E-06	0.006664675
1380.839974	195.351578	4.40881E-06	0.006087865
1380.879974	183.300528	4.13684E-06	0.005712476
1380.919974	182.18991	4.11177E-06	0.005678029
1380.959974	193.561761	4.36842E-06	0.006032612

1380.999974	201.782482	4.55395E-06	0.006289004
1381.039974	209.858014	4.7362E-06	0.006540886
1381.079974	206.235946	4.65446E-06	0.006428179
1381.119974	196.595991	4.4369E-06	0.006127888
1381.159974	214.037099	4.83052E-06	0.00667172
1381.199974	250.766895	5.65946E-06	0.007816845
1381.239974	275.226823	6.21149E-06	0.008579553
1381.279974	269.826654	6.08961E-06	0.008411459
1381.319974	270.122785	6.0963E-06	0.008420934
1381.359974	263.885596	5.95553E-06	0.008226731
1381.399974	237.073548	5.35042E-06	0.00739107
1381.439974	204.857969	4.62336E-06	0.006386893
1381.479974	176.493097	3.9832E-06	0.005502716
1381.519974	162.491668	3.66721E-06	0.005066325
1381.559974	149.907991	3.38321E-06	0.004674114
1381.599974	145.706945	3.2884E-06	0.004543257
1381.639974	139.218195	3.14196E-06	0.004341059
1381.679974	152.185201	3.43461E-06	0.004745529
1381.719974	180.411368	4.07163E-06	0.005625857
1381.759974	206.797405	4.66713E-06	0.006448853
1381.799974	210.598837	4.75292E-06	0.006567588
1381.839974	191.999429	4.33316E-06	0.005987733
1381.879974	178.486343	4.02819E-06	0.005566473
1381.919974	185.033287	4.17594E-06	0.00577082
1381.959974	183.224646	4.13513E-06	0.005714577
1381.999974	183.75011	4.14698E-06	0.005731132
1382.039974	195.596848	4.41435E-06	0.006100806
1382.079974	195.36303	4.40907E-06	0.006093689
1382.119974	197.4336	4.4558E-06	0.006158452
1382.159974	206.825911	4.66777E-06	0.006451609
1382.199974	229.795491	5.18616E-06	0.007168316
1382.239974	244.349605	5.51463E-06	0.007622542
1382.279974	224.05874	5.05669E-06	0.006989766
1382.319974	203.026127	4.58202E-06	0.006333813
1382.359974	202.487454	4.56986E-06	0.006317191
1382.399974	189.374332	4.27391E-06	0.00590826
1382.439974	175.997315	3.97201E-06	0.005491071
1382.479974	174.048354	3.92803E-06	0.005430421
1382.519974	170.28577	3.84311E-06	0.00531318
1382.559974	171.652474	3.87396E-06	0.005355978
1382.599974	179.126206	4.04263E-06	0.005589339
1382.639974	176.348297	3.97994E-06	0.005502818
1382.679974	178.189356	4.02149E-06	0.005560428
1382.719974	173.880235	3.92423E-06	0.005426118
1382.759974	171.830544	3.87798E-06	0.00536231
1382.799974	174.717932	3.94314E-06	0.005452574

1382.839974	179.136235	4.04286E-06	0.005590622
1382.879974	164.923552	3.72209E-06	0.00514721
1382.919974	152.309296	3.43741E-06	0.004753661
1382.959974	145.788599	3.29025E-06	0.004550278
1382.999974	162.073681	3.65778E-06	0.005058706
1383.039974	177.031237	3.99535E-06	0.005525727
1383.079974	200.4322	4.52348E-06	0.006256328
1383.119974	233.986271	5.28074E-06	0.007303903
1383.159974	248.40891	5.60624E-06	0.007754331
1383.199974	250.413966	5.65149E-06	0.007817147
1383.239974	249.615383	5.63347E-06	0.007792443
1383.279974	257.424551	5.80971E-06	0.00803646
1383.319974	270.379889	6.1021E-06	0.008441153
1383.359974	274.232828	6.18905E-06	0.008561688
1383.399974	238.119845	5.37403E-06	0.007434437
1383.439974	214.314401	4.83678E-06	0.006691391
1383.479974	194.064704	4.37977E-06	0.006059324
1383.519974	174.351045	3.93486E-06	0.005443958
1383.559974	169.355961	3.82213E-06	0.005288144
1383.599974	181.778452	4.10249E-06	0.0056762
1383.639974	193.642735	4.37025E-06	0.006046848
1383.679974	184.3216	4.15988E-06	0.005755945
1383.719974	167.257892	3.77478E-06	0.005223235
1383.759974	166.351405	3.75432E-06	0.005195077
1383.799974	162.336928	3.66372E-06	0.005069853
1383.839974	165.31575	3.73095E-06	0.005163033
1383.879974	169.006276	3.81424E-06	0.005278445
1383.919974	174.698397	3.9427E-06	0.005456381
1383.959974	179.235948	4.04511E-06	0.005598264
1383.999974	172.728413	3.89824E-06	0.005395164
1384.039974	159.975798	3.61043E-06	0.004996981
1384.079974	174.543504	3.9392E-06	0.005452173
1384.119974	196.778879	4.44103E-06	0.006146912
1384.159974	211.300996	4.76877E-06	0.006600739
1384.199974	227.705351	5.13899E-06	0.007113393
1384.239974	250.024192	5.6427E-06	0.007810848
1384.279974	242.297233	5.46831E-06	0.007569673
1384.319974	228.25285	5.15135E-06	0.007131115
1384.359974	240.641665	5.43095E-06	0.007518386
1384.399974	234.762303	5.29826E-06	0.007334908
1384.439974	218.015097	4.9203E-06	0.006811856
1384.479974	216.841406	4.89381E-06	0.00677538
1384.519974	221.782755	5.00533E-06	0.006929976
1384.559974	183.7905	4.1479E-06	0.00574301
1384.599974	138.722852	3.13078E-06	0.00433488
1384.639974	144.368545	3.2582E-06	0.00451143

1384.679974	188.339517	4.25056E-06	0.005885666
1384.719974	202.982494	4.58103E-06	0.006343447
1384.759974	207.523737	4.68352E-06	0.006485553
1384.799974	204.976796	4.62604E-06	0.006406141
1384.839974	179.921194	4.06057E-06	0.005623241
1384.879974	185.216299	4.18007E-06	0.005788901
1384.919974	205.119632	4.62926E-06	0.006411161
1384.959974	200.634837	4.52805E-06	0.006271166
1384.999974	196.605301	4.43711E-06	0.006145394
1385.039974	214.95773	4.8513E-06	0.00671924
1385.079974	235.826826	5.32228E-06	0.007371788
1385.119974	248.01186	5.59728E-06	0.007752907
1385.159974	253.280203	5.71618E-06	0.007917826
1385.199974	264.448217	5.96823E-06	0.008267189
1385.239974	261.648552	5.90504E-06	0.008179902
1385.279974	253.550277	5.72228E-06	0.007926955
1385.319974	268.914431	6.06902E-06	0.00840754
1385.359974	276.557712	6.24152E-06	0.008646755
1385.399974	265.314937	5.98779E-06	0.008295482
1385.439974	255.145896	5.75829E-06	0.007977762
1385.479974	240.918094	5.43719E-06	0.007533112
1385.519974	210.187767	4.74365E-06	0.006572415
1385.559974	172.404106	3.89092E-06	0.005391104
1385.599973	165.058694	3.72514E-06	0.005161561
1385.639973	170.884624	3.85663E-06	0.005343898
1385.679973	181.737892	4.10157E-06	0.005683465
1385.719973	200.527932	4.52564E-06	0.006271264
1385.759973	220.583891	4.97827E-06	0.006898689
1385.799973	220.377101	4.9736E-06	0.006892421
1385.839973	208.981123	4.71641E-06	0.006536193
1385.879973	198.38606	4.4773E-06	0.006204996
1385.919973	195.601731	4.41446E-06	0.006118086
1385.959973	196.811294	4.44176E-06	0.006156097
1385.999973	215.717876	4.86845E-06	0.006747674
1386.039973	245.242544	5.53478E-06	0.00767143
1386.079973	252.542344	5.69953E-06	0.007900003
1386.119973	257.630372	5.81436E-06	0.008059399
1386.159973	262.528257	5.9249E-06	0.008212855
1386.199973	275.868438	6.22597E-06	0.008630434
1386.239973	288.528183	6.51168E-06	0.00902675
1386.279973	283.447236	6.39701E-06	0.008868046
1386.319973	275.575911	6.21936E-06	0.008622029
1386.359973	287.586163	6.49042E-06	0.008998057
1386.399973	291.789646	6.58529E-06	0.00912984
1386.439973	296.530972	6.69229E-06	0.00927846
1386.479973	278.86172	6.29352E-06	0.00872584

1386.519973	225.571501	5.09083E-06	0.007058544
1386.559973	175.183751	3.95365E-06	0.005481977
1386.599973	172.392719	3.89066E-06	0.005394794
1386.639973	186.920879	4.21854E-06	0.005849602
1386.679973	189.284651	4.27189E-06	0.005923746
1386.719973	202.278684	4.56515E-06	0.006330582
1386.759973	221.83566	5.00652E-06	0.006942844
1386.799973	225.220869	5.08292E-06	0.007048995
1386.839973	215.995836	4.87473E-06	0.006760464
1386.879973	215.608138	4.86598E-06	0.006748524
1386.919973	226.561052	5.11317E-06	0.007091554
1386.959973	236.117688	5.32885E-06	0.007390898
1386.999973	221.857229	5.00701E-06	0.006944721
1387.039973	220.362488	4.97327E-06	0.00689813
1387.079973	238.859208	5.39072E-06	0.007477359
1387.119973	263.272729	5.9417E-06	0.008241849
1387.159973	290.406366	6.55407E-06	0.00909154
1387.199973	292.547873	6.6024E-06	0.009158846
1387.239973	300.304702	6.77746E-06	0.009401962
1387.279973	321.151698	7.24795E-06	0.010054931
1387.319973	340.434961	7.68314E-06	0.010658978
1387.359973	345.015955	7.78653E-06	0.010802719
1387.399973	340.984926	7.69555E-06	0.010676813
1387.439973	303.995991	6.86077E-06	0.009518901
1387.479973	270.541903	6.10575E-06	0.008471611
1387.519973	234.375982	5.28954E-06	0.007339342
1387.559973	208.960718	4.71595E-06	0.006543667
1387.599973	199.294449	4.4978E-06	0.006241145
1387.639973	180.368477	4.07067E-06	0.005648618
1387.679973	167.849737	3.78813E-06	0.005256719
1387.719973	197.627598	4.46018E-06	0.00618948
1387.759973	249.993311	5.642E-06	0.007829743
1387.799973	271.694218	6.13176E-06	0.008509656
1387.839973	282.086983	6.36631E-06	0.00883542
1387.879973	295.57314	6.67067E-06	0.009258095
1387.919973	286.329005	6.46205E-06	0.008968804
1387.959973	257.734873	5.81672E-06	0.00807337
1387.999973	212.007727	4.78472E-06	0.00664119
1388.039973	176.091452	3.97414E-06	0.005516264
1388.079973	188.623353	4.25697E-06	0.00590901
1388.119973	235.700349	5.31943E-06	0.007384005
1388.159973	263.626645	5.94969E-06	0.008259116
1388.199973	285.022856	6.43257E-06	0.008929692
1388.239973	306.731499	6.9225E-06	0.009610095
1388.279973	329.2126	7.42987E-06	0.010314739
1388.319973	325.225193	7.33988E-06	0.010190101

1388.359973	324.587124	7.32548E-06	0.010170402
1388.399973	309.150065	6.97709E-06	0.009686987
1388.439973	278.507817	6.28553E-06	0.008727086
1388.479973	233.440392	5.26842E-06	0.007315102
1388.519973	193.837733	4.37465E-06	0.006074286
1388.559973	180.662073	4.07729E-06	0.005661564
1388.599973	193.882363	4.37565E-06	0.006076034
1388.639973	190.214712	4.29288E-06	0.005961266
1388.679973	177.659989	4.00954E-06	0.005567966
1388.719973	179.731952	4.0563E-06	0.005633065
1388.759973	210.454812	4.74967E-06	0.006596154
1388.799973	257.657096	5.81496E-06	0.008075819
1388.839973	281.439647	6.3517E-06	0.008821496
1388.879973	284.344186	6.41725E-06	0.008912793
1388.919973	282.880262	6.38421E-06	0.008867162
1388.959973	264.808223	5.97635E-06	0.008300915
1388.999973	245.814876	5.5477E-06	0.007705754
1389.039973	244.459436	5.51711E-06	0.007663485
1389.079973	249.255975	5.62536E-06	0.007814075
1389.119973	252.719259	5.70352E-06	0.007922876
1389.159973	249.614444	5.63345E-06	0.007825764
1389.199973	251.518782	5.67643E-06	0.007885694
1389.239973	264.663078	5.97308E-06	0.008298037
1389.279973	265.251379	5.98635E-06	0.008316722
1389.319973	273.178821	6.16527E-06	0.008565526
1389.359973	261.797761	5.90841E-06	0.008208909
1389.399973	224.317464	5.06253E-06	0.007033883
1389.439973	193.681935	4.37113E-06	0.006073425
1389.479973	179.743392	4.05656E-06	0.005636506
1389.519973	178.82463	4.03582E-06	0.005607856
1389.559973	175.939937	3.97072E-06	0.005517553
1389.599973	169.331915	3.82159E-06	0.005310475
1389.639973	203.86395	4.60093E-06	0.00639363
1389.679973	224.606348	5.06905E-06	0.007044361
1389.719973	192.769503	4.35054E-06	0.006046031
1389.759973	169.756355	3.83116E-06	0.005324399
1389.799973	166.937606	3.76755E-06	0.00523614
1389.839973	188.461552	4.25331E-06	0.005911427
1389.879973	197.100972	4.44829E-06	0.006182595
1389.919973	203.230266	4.58662E-06	0.00637504
1389.959973	231.215252	5.21821E-06	0.007253098
1389.999973	225.766086	5.09523E-06	0.007082364
1390.039973	210.649358	4.75406E-06	0.006608337
1390.079973	219.117954	4.94519E-06	0.006874205
1390.119973	206.936699	4.67027E-06	0.00649224
1390.159973	191.773788	4.32807E-06	0.006016706

1390.199973	197.944392	4.46733E-06	0.006210481
1390.239973	213.701562	4.82295E-06	0.006705053
1390.279973	253.997921	5.73238E-06	0.007969612
1390.319973	276.532858	6.24096E-06	0.008676933
1390.359973	281.009852	6.342E-06	0.008817664
1390.399973	262.533606	5.92502E-06	0.008238145
1390.439973	223.467093	5.04334E-06	0.007012463
1390.479973	184.34662	4.16045E-06	0.005785017
1390.519973	174.496446	3.93814E-06	0.005476065
1390.559973	175.099243	3.95175E-06	0.00549514
1390.599973	162.208757	3.66083E-06	0.005090744
1390.639973	148.292804	3.34676E-06	0.004654141
1390.679973	154.404502	3.48469E-06	0.004846095
1390.719973	174.718305	3.94315E-06	0.005483816
1390.759973	209.77902	4.73442E-06	0.006584442
1390.799973	210.86387	4.7589E-06	0.006618683
1390.839973	205.836342	4.64544E-06	0.006461063
1390.879973	209.414781	4.7262E-06	0.006573577
1390.919973	212.790748	4.80239E-06	0.006679741
1390.959973	199.35262	4.49911E-06	0.006258083
1390.999973	173.251622	3.91005E-06	0.005438876
1391.039973	170.274034	3.84285E-06	0.005345555
1391.079973	191.874731	4.33035E-06	0.006023857
1391.119973	215.94766	4.87364E-06	0.006779815
1391.159973	230.283493	5.19718E-06	0.007230106
1391.199973	255.353781	5.76298E-06	0.008017456
1391.239973	277.295555	6.25817E-06	0.008706622
1391.279973	284.415363	6.41886E-06	0.008930429
1391.319973	266.218159	6.00817E-06	0.008359291
1391.359973	251.919826	5.68548E-06	0.007910549
1391.399973	242.405262	5.47075E-06	0.007612
1391.439973	232.726055	5.2523E-06	0.007308264
1391.479973	219.97029	4.96442E-06	0.006907895
1391.519973	200.001106	4.51375E-06	0.006280968
1391.559973	181.254217	4.09066E-06	0.005692392
1391.599973	165.93127	3.74484E-06	0.005211316
1391.639973	149.469692	3.37332E-06	0.004694451
1391.679973	154.447624	3.48567E-06	0.004850934
1391.719973	146.918254	3.31574E-06	0.004614582
1391.759973	149.634674	3.37705E-06	0.004700038
1391.799973	169.957178	3.8357E-06	0.005338523
1391.839973	177.742938	4.01141E-06	0.005583242
1391.879973	169.419393	3.82356E-06	0.005321936
1391.919973	165.210876	3.72858E-06	0.005189884
1391.959973	164.77565	3.71876E-06	0.005176361
1391.999973	168.179581	3.79558E-06	0.005283446

1392.039973	170.064084	3.83811E-06	0.005342802
1392.079973	160.153739	3.61445E-06	0.005031599
1392.119973	152.383682	3.43909E-06	0.004787622
1392.159973	175.827941	3.96819E-06	0.005524358
1392.199973	222.861354	5.02967E-06	0.007002307
1392.239973	270.491434	6.10461E-06	0.008499089
1392.279973	282.607134	6.37805E-06	0.00888003
1392.319973	267.116132	6.02844E-06	0.008393516
1392.359973	246.960485	5.57355E-06	0.007760394
1392.399973	234.209172	5.28577E-06	0.007359913
1392.439973	223.73027	5.04928E-06	0.00703082
1392.479973	187.478864	4.23114E-06	0.005891773
1392.519973	165.542327	3.73606E-06	0.005202538
1392.559973	163.7119	3.69475E-06	0.00514516
1392.599973	163.001743	3.67872E-06	0.005122989
1392.639973	180.741181	4.07908E-06	0.005680685
1392.679973	191.986885	4.33288E-06	0.00603431
1392.719973	188.917949	4.26361E-06	0.005938022
1392.759973	171.97045	3.88113E-06	0.005405487
1392.799973	177.988107	4.01694E-06	0.005594799
1392.839973	178.217721	4.02213E-06	0.005602177
1392.879973	182.826902	4.12615E-06	0.00574723
1392.919973	199.048336	4.49224E-06	0.006257336
1392.959973	194.400578	4.38735E-06	0.006111403
1392.999973	176.018675	3.9725E-06	0.005533687
1393.039973	177.710071	4.01067E-06	0.005587022
1393.079973	174.391184	3.93577E-06	0.005482837
1393.119973	180.645849	4.07693E-06	0.005679646
1393.159973	215.0009	4.85227E-06	0.00675999
1393.199973	234.844637	5.30012E-06	0.007384122
1393.239973	254.961965	5.75414E-06	0.008016893
1393.279973	256.198038	5.78203E-06	0.008055991
1393.319973	231.750531	5.23029E-06	0.007287463
1393.359973	213.596727	4.82058E-06	0.006716804
1393.399973	228.862175	5.1651E-06	0.007197051
1393.439973	221.124905	4.99048E-06	0.006953936
1393.479973	183.090104	4.13209E-06	0.005757983
1393.519973	155.976775	3.52018E-06	0.004905439
1393.559973	141.489833	3.19323E-06	0.004449955
1393.599973	137.235863	3.09722E-06	0.004316289
1393.639973	138.587934	3.12774E-06	0.004358939
1393.679973	137.648377	3.10653E-06	0.004329512
1393.719973	152.32825	3.43784E-06	0.004791381
1393.759973	166.31328	3.75346E-06	0.005231421
1393.799973	168.642661	3.80603E-06	0.005304844
1393.839973	174.710212	3.94297E-06	0.005495864

1393.879973	180.113768	4.06492E-06	0.005666006
1393.919973	190.933538	4.3091E-06	0.006006546
1393.959973	198.065576	4.47006E-06	0.006231091
1393.999973	214.258631	4.83552E-06	0.006740713
1394.039973	195.68263	4.41628E-06	0.006156477
1394.079973	162.480697	3.66696E-06	0.00511204
1394.119973	145.487573	3.28345E-06	0.004577526
1394.159973	141.195252	3.18658E-06	0.004442603
1394.199973	163.175796	3.68265E-06	0.005134351
1394.239973	189.780369	4.28308E-06	0.005971639
1394.279973	195.944055	4.42218E-06	0.006165763
1394.319973	213.489354	4.81816E-06	0.006718053
1394.359973	224.008389	5.05556E-06	0.007049267
1394.399973	219.777249	4.96007E-06	0.006916316
1394.439973	202.900594	4.57918E-06	0.006385397
1394.479973	185.622528	4.18924E-06	0.005841814
1394.519973	170.682855	3.85207E-06	0.005371795
1394.559973	160.35997	3.6191E-06	0.005047054
1394.599973	145.900623	3.29277E-06	0.004592102
1394.639973	149.594792	3.37615E-06	0.004708508
1394.679973	175.383551	3.95816E-06	0.00552037
1394.719973	182.451275	4.11767E-06	0.005742998
1394.759973	186.553167	4.21025E-06	0.005872281
1394.799973	202.047334	4.55993E-06	0.006360186
1394.839973	191.49151	4.3217E-06	0.006028075
1394.879973	176.237643	3.97744E-06	0.005548049
1394.919973	180.383297	4.071E-06	0.005678719
1394.959973	204.917231	4.6247E-06	0.006451266
1394.999973	230.665257	5.20579E-06	0.007262082
1395.039973	237.842962	5.36778E-06	0.007488274
1395.079973	221.236208	4.99299E-06	0.006965625
1395.119973	221.179595	4.99172E-06	0.006964042
1395.159973	235.171625	5.3075E-06	0.007404806
1395.199973	237.912562	5.36936E-06	0.007491324
1395.239973	235.028756	5.30427E-06	0.007400732
1395.279973	242.977489	5.48366E-06	0.007651246
1395.319973	253.657146	5.72469E-06	0.007987772
1395.359973	246.832511	5.57067E-06	0.007773084
1395.399973	230.637722	5.20517E-06	0.007263297
1395.439973	210.868762	4.75901E-06	0.006640919
1395.479973	188.947224	4.26428E-06	0.005950711
1395.519973	170.730428	3.85315E-06	0.005377145
1395.559973	159.924496	3.60927E-06	0.005036957
1395.599973	155.8673	3.51771E-06	0.004909313
1395.639973	160.797386	3.62897E-06	0.00506474
1395.679973	164.932537	3.7223E-06	0.005195136

1395.719973	173.902018	3.92473E-06	0.005477819
1395.759973	172.048748	3.8829E-06	0.005419597
1395.799973	151.721936	3.42415E-06	0.004779432
1395.839973	144.681355	3.26526E-06	0.004557776
1395.879973	165.03269	3.72456E-06	0.005199036
1395.919973	183.280642	4.13639E-06	0.005774068
1395.959973	187.907172	4.2408E-06	0.005919991
1395.999973	163.774634	3.69617E-06	0.005159847
1396.039973	158.442785	3.57583E-06	0.004992006
1396.079973	185.059758	4.17654E-06	0.005830785
1396.119973	191.958915	4.33225E-06	0.006048334
1396.159973	187.635868	4.23468E-06	0.005912291
1396.199973	218.367867	4.92826E-06	0.006880835
1396.239973	237.74763	5.36563E-06	0.007491711
1396.279973	242.048969	5.46271E-06	0.00762747
1396.319973	245.496792	5.54052E-06	0.00773634
1396.359973	239.883065	5.41383E-06	0.007559651
1396.399973	225.647737	5.09256E-06	0.007111244
1396.439973	223.468461	5.04337E-06	0.007042766
1396.479973	198.161157	4.47222E-06	0.006245367
1396.519973	175.469162	3.96009E-06	0.005530351
1396.559973	158.614215	3.5797E-06	0.004999268
1396.599973	147.839776	3.33654E-06	0.004659809
1396.639973	159.455687	3.59869E-06	0.005026078
1396.679973	184.677012	4.1679E-06	0.005821227
1396.719973	202.947026	4.58023E-06	0.006397301
1396.759973	195.381472	4.40949E-06	0.006158996
1396.799973	186.726335	4.21415E-06	0.005886329
1396.839973	194.786615	4.39606E-06	0.006140596
1396.879973	221.713239	5.00376E-06	0.006989651
1396.919973	215.469918	4.86286E-06	0.006793021
1396.959973	188.132601	4.24589E-06	0.005931339
1396.999973	197.647126	4.46062E-06	0.006231486
1397.039973	215.614277	4.86611E-06	0.006798156
1397.079973	228.879933	5.1655E-06	0.007216618
1397.119973	216.768918	4.89217E-06	0.006834952
1397.159973	216.459146	4.88518E-06	0.00682538
1397.199973	226.370694	5.10887E-06	0.007138115
1397.239973	217.455204	4.90766E-06	0.00685718
1397.279973	230.333501	5.19831E-06	0.007263489
1397.319973	244.639219	5.52117E-06	0.007714836
1397.359973	251.821086	5.68325E-06	0.007941548
1397.399973	234.290554	5.28761E-06	0.007388908
1397.439973	204.315156	4.61111E-06	0.006443747
1397.479973	177.959501	4.0163E-06	0.005612696
1397.519973	163.453469	3.68892E-06	0.005155335

1397.559973	161.891375	3.65366E-06	0.005106213
1397.599973	147.638625	3.332E-06	0.0046568
1397.639973	157.332365	3.55077E-06	0.004962701
1397.679973	197.578438	4.45907E-06	0.006232353
1397.719973	213.610862	4.8209E-06	0.006738268
1397.759973	195.931628	4.4219E-06	0.00618076
1397.799973	191.963863	4.33236E-06	0.006055769
1397.839973	205.210974	4.63133E-06	0.006473852
1397.879973	196.66048	4.43835E-06	0.006204285
1397.919973	186.141666	4.20096E-06	0.005872603
1397.959973	187.580103	4.23342E-06	0.005918154
1397.999973	178.333613	4.02474E-06	0.005626588
1398.039973	172.169007	3.88561E-06	0.005432245
1398.079973	152.579462	3.44351E-06	0.004814297
1398.119973	162.103246	3.65844E-06	0.005114944
1398.159973	215.063126	4.85368E-06	0.006786214
1398.199973	253.089455	5.71188E-06	0.007986346
1398.239973	253.233987	5.71514E-06	0.007991135
1398.279973	223.698124	5.04856E-06	0.007059293
1398.319973	222.963499	5.03198E-06	0.007036312
1398.359973	225.407146	5.08713E-06	0.007113632
1398.399973	216.07447	4.8765E-06	0.006819297
1398.439973	192.650153	4.34785E-06	0.006080201
1398.479973	164.205199	3.70588E-06	0.005182603
1398.519973	150.859875	3.4047E-06	0.004761537
1398.559973	145.042812	3.27341E-06	0.004578066
1398.599973	159.294133	3.59505E-06	0.005028032
1398.639973	168.111632	3.79405E-06	0.005306504
1398.679973	162.160819	3.65974E-06	0.00511881
1398.719973	167.142775	3.77218E-06	0.005276223
1398.759973	175.403512	3.95861E-06	0.005537149
1398.799973	195.233046	4.40614E-06	0.006163306
1398.839973	198.708026	4.48456E-06	0.006273187
1398.879973	185.027377	4.17581E-06	0.005841457
1398.919973	165.195101	3.72822E-06	0.005215486
1398.959973	173.009841	3.90459E-06	0.005462367
1398.999973	179.432011	4.04953E-06	0.005665293
1399.039973	189.823852	4.28406E-06	0.005993571
1399.079973	198.50202	4.47991E-06	0.006267758
1399.119973	198.575926	4.48158E-06	0.006270271
1399.159973	242.064275	5.46305E-06	0.007643686
1399.199973	275.139943	6.20953E-06	0.008688368
1399.239973	278.593983	6.28748E-06	0.008797691
1399.279973	291.312617	6.57452E-06	0.009199594
1399.319973	292.003161	6.5901E-06	0.009221665
1399.359973	284.516939	6.42115E-06	0.008985502

1399.399973	263.889689	5.95562E-06	0.008334298
1399.439973	252.724592	5.70364E-06	0.007981905
1399.479973	244.97195	5.52868E-06	0.007737271
1399.519973	198.644305	4.48313E-06	0.006274223
1399.559973	157.559091	3.55589E-06	0.00497668
1399.599973	157.235428	3.54858E-06	0.004966599
1399.639973	159.109043	3.59087E-06	0.005025924
1399.679973	157.825515	3.5619E-06	0.004985523
1399.719973	145.861199	3.29188E-06	0.004607716
1399.759973	138.726883	3.13087E-06	0.00438247
1399.799973	155.426326	3.50776E-06	0.004910156
1399.839973	185.750837	4.19214E-06	0.005868322
1399.879973	206.758126	4.66624E-06	0.00653218
1399.919973	214.81297	4.84803E-06	0.006786853
1399.959973	205.480316	4.6374E-06	0.006492181
1399.999973	203.734876	4.59801E-06	0.006437217
1400.039973	232.739187	5.2526E-06	0.007353849
1400.079973	229.106102	5.17061E-06	0.007239261
1400.119973	202.120259	4.56157E-06	0.006386749
1400.159973	200.115012	4.51632E-06	0.006323566
1400.199973	215.695364	4.86794E-06	0.006816095
1400.239973	238.11334	5.37389E-06	0.007524731
1400.279973	259.01875	5.84569E-06	0.008185606
1400.319973	251.634674	5.67904E-06	0.007952479
1400.359973	240.7157	5.43262E-06	0.007607621
1400.399973	234.202259	5.28562E-06	0.00740198
1400.439973	221.53041	4.99963E-06	0.007001686
1400.479973	197.76251	4.46322E-06	0.006250656
1400.519973	182.454811	4.11775E-06	0.005766992
1400.559973	168.856058	3.81085E-06	0.005337318
1400.599973	157.544939	3.55557E-06	0.004979931
1400.639973	162.593546	3.66951E-06	0.005139662
1400.679973	178.583176	4.03037E-06	0.005645263
1400.719973	223.627988	5.04697E-06	0.007069395
1400.759973	231.629867	5.22756E-06	0.007322562
1400.799973	207.677358	4.68699E-06	0.006565534
1400.839973	190.650968	4.30273E-06	0.006027432
1400.879973	178.292989	4.02382E-06	0.005636895
1400.919973	170.493254	3.8478E-06	0.005390453
1400.959973	181.328137	4.09232E-06	0.005733181
1400.999973	197.536346	4.45812E-06	0.006245826
1401.039973	205.921414	4.64736E-06	0.006511136
1401.079973	208.320924	4.70151E-06	0.006587196
1401.119973	216.747076	4.89168E-06	0.00685383
1401.159973	251.810545	5.68301E-06	0.007962811
1401.199973	258.991395	5.84508E-06	0.008190119

1401.239973	253.926009	5.73076E-06	0.008030165
1401.279973	249.261838	5.62549E-06	0.00788289
1401.319973	245.859145	5.5487E-06	0.007775502
1401.359973	234.075105	5.28275E-06	0.007403033
1401.399973	218.786519	4.93771E-06	0.006919702
1401.439973	203.749128	4.59833E-06	0.006444289
1401.479973	196.908966	4.44396E-06	0.006228122
1401.519973	197.725566	4.46239E-06	0.00625413
1401.559973	182.621861	4.12152E-06	0.005776559
1401.599973	167.903404	3.78935E-06	0.005311147
1401.639973	155.878973	3.51797E-06	0.004930929
1401.679973	148.615838	3.35405E-06	0.004701308
1401.719973	145.91611	3.29312E-06	0.004616037
1401.759973	183.362354	4.13823E-06	0.005800809
1401.799973	216.171163	4.87868E-06	0.006838936
1401.839973	217.992969	4.9198E-06	0.006896769
1401.879973	206.603379	4.66275E-06	0.006536617
1401.919973	194.295986	4.38499E-06	0.006147405
1401.959973	185.097818	4.1774E-06	0.005856547
1401.999973	197.777152	4.46355E-06	0.006257904
1402.039973	194.879327	4.39815E-06	0.006166389
1402.079973	205.382392	4.63519E-06	0.006498913
1402.119973	250.245771	5.6477E-06	0.007918751
1402.159973	295.652157	6.67246E-06	0.009355853
1402.199973	299.336875	6.75562E-06	0.009472725
1402.239973	257.519053	5.81185E-06	0.008149603
1402.279973	233.853387	5.27775E-06	0.007400876
1402.319973	247.501216	5.58576E-06	0.00783302
1402.359973	249.640929	5.63405E-06	0.007900963
1402.399973	224.445066	5.06541E-06	0.007103734
1402.439973	206.886798	4.66915E-06	0.006548198
1402.479973	188.668304	4.25798E-06	0.005971733
1402.519973	172.013816	3.88211E-06	0.00544474
1402.559973	146.899862	3.31533E-06	0.004649943
1402.599973	133.852551	3.02087E-06	0.004237066
1402.639973	144.684279	3.26532E-06	0.004580072
1402.679973	155.394657	3.50704E-06	0.004919256
1402.719973	174.506205	3.93836E-06	0.005524419
1402.759973	193.186448	4.35995E-06	0.006115962
1402.799973	192.384931	4.34186E-06	0.006090761
1402.839973	197.593033	4.4594E-06	0.006255824
1402.879973	189.093247	4.26757E-06	0.00598689
1402.919973	188.046156	4.24394E-06	0.005953908
1402.959973	204.812097	4.62232E-06	0.006484935
1402.999973	197.276875	4.45226E-06	0.006246527
1403.039973	185.684798	4.19065E-06	0.005879646

1403.079973	178.596143	4.03067E-06	0.005655347
1403.119973	175.98186	3.97167E-06	0.005572723
1403.159973	205.576835	4.63958E-06	0.006510077
1403.199973	239.358878	5.402E-06	0.007580081
1403.239973	238.447724	5.38143E-06	0.007551442
1403.279973	246.82528	5.5705E-06	0.007816975
1403.319973	247.176364	5.57843E-06	0.007828317
1403.359973	234.024629	5.28161E-06	0.007412
1403.399973	222.716674	5.02641E-06	0.007054057
1403.439973	232.940349	5.25714E-06	0.007378079
1403.479973	233.679178	5.27381E-06	0.007401692
1403.519973	213.694324	4.82278E-06	0.006768873
1403.559973	196.018413	4.42386E-06	0.006209156
1403.599973	187.338012	4.22796E-06	0.005934362
1403.639973	184.005613	4.15275E-06	0.005828966
1403.679973	182.841881	4.12649E-06	0.005792267
1403.719973	182.948478	4.12889E-06	0.005795809
1403.759973	181.670206	4.10004E-06	0.005755477
1403.799973	170.347643	3.84451E-06	0.005396922
1403.839973	155.44158	3.5081E-06	0.004924811
1403.879973	163.683105	3.6941E-06	0.005186073
1403.919973	161.621264	3.64757E-06	0.005120892
1403.959973	162.543581	3.66838E-06	0.005150262
1403.999973	183.401156	4.13911E-06	0.005811308
1404.039973	203.865044	4.60095E-06	0.006459918
1404.079973	210.288768	4.74592E-06	0.006663658
1404.119973	216.202964	4.8794E-06	0.006851263
1404.159973	212.917599	4.80525E-06	0.006747345
1404.199973	224.768724	5.07272E-06	0.007123109
1404.239973	254.927083	5.75335E-06	0.008079083
1404.279973	252.046414	5.68834E-06	0.007988017
1404.319973	253.250226	5.7155E-06	0.008026398
1404.359973	254.192999	5.73678E-06	0.008056507
1404.399973	229.394704	5.17712E-06	0.007270746
1404.439973	189.208854	4.27018E-06	0.005997212
1404.479973	157.15322	3.54673E-06	0.00498131
1404.519973	159.161231	3.59205E-06	0.005045102
1404.559973	175.109347	3.95197E-06	0.005550785
1404.599973	181.285853	4.09137E-06	0.005746737
1404.639973	174.040736	3.92786E-06	0.005517225
1404.679973	187.737176	4.23697E-06	0.005951582
1404.719973	204.260442	4.60987E-06	0.006475582
1404.759973	219.5067	4.95396E-06	0.006959125
1404.799973	220.71664	4.98127E-06	0.006997684
1404.839973	211.170163	4.76582E-06	0.006695209
1404.879973	202.52938	4.57081E-06	0.006421434

1404.919973	181.615229	4.0988E-06	0.00575849
1404.959973	173.230212	3.90956E-06	0.005492782
1404.999973	185.37212	4.18359E-06	0.005877945
1405.039973	197.588765	4.4593E-06	0.006265499
1405.079973	197.795383	4.46397E-06	0.00627223
1405.119973	226.97006	5.1224E-06	0.007197584
1405.159973	234.910487	5.3016E-06	0.0074496
1405.199973	221.320021	4.99488E-06	0.007018812
1405.239973	243.698499	5.49994E-06	0.007728729
1405.279973	273.823802	6.17982E-06	0.00868438
1405.319973	266.213448	6.00807E-06	0.008443256
1405.359973	254.057026	5.73371E-06	0.008057931
1405.399973	244.504573	5.51813E-06	0.007755176
1405.439973	207.91056	4.69225E-06	0.006594678
1405.479973	178.093361	4.01932E-06	0.005649072
1405.519973	164.225005	3.70633E-06	0.00520932
1405.559973	162.605454	3.66978E-06	0.005158094
1405.599973	173.522304	3.91616E-06	0.00550455
1405.639973	181.552891	4.0974E-06	0.005759463
1405.679973	177.796212	4.01261E-06	0.00564045
1405.719973	180.861178	4.08178E-06	0.005737847
1405.759973	189.133113	4.26847E-06	0.006000446
1405.799973	181.954533	4.10646E-06	0.005772862
1405.839973	162.645235	3.67068E-06	0.005160384
1405.879973	152.405504	3.43958E-06	0.004835637
1405.919973	147.173287	3.3215E-06	0.004669758
1405.959973	166.30669	3.75331E-06	0.005277004
1405.999973	181.807893	4.10315E-06	0.00576903
1406.039973	197.933643	4.46709E-06	0.006280902
1406.079973	214.562201	4.84237E-06	0.00680876
1406.119973	198.258915	4.47443E-06	0.006291582
1406.159973	181.226835	4.09004E-06	0.005751247
1406.199973	184.246566	4.15819E-06	0.005847244
1406.239973	214.990001	4.85202E-06	0.006823111
1406.279973	239.283972	5.40031E-06	0.007594342
1406.319973	267.201606	6.03037E-06	0.008480627
1406.359973	249.432814	5.62935E-06	0.007916894
1406.399973	214.293035	4.8363E-06	0.006801766
1406.439973	177.529747	4.0066E-06	0.005635041
1406.479973	164.656327	3.71606E-06	0.00522657
1406.519973	162.308752	3.66308E-06	0.005152199
1406.559973	155.584	3.51131E-06	0.004938874
1406.599973	148.123322	3.34294E-06	0.004702175
1406.639973	152.062107	3.43183E-06	0.004827349
1406.679973	174.758258	3.94405E-06	0.005548017
1406.719973	196.905132	4.44387E-06	0.006251287

1406.759973	198.634893	4.48291E-06	0.006306382
1406.799973	191.526765	4.32249E-06	0.006080882
1406.839973	207.755701	4.68876E-06	0.00659633
1406.879973	209.243706	4.72234E-06	0.006643764
1406.919973	199.189661	4.49543E-06	0.006324715
1406.959973	188.650231	4.25757E-06	0.005990235
1406.999973	188.872423	4.26259E-06	0.005997461
1407.039973	178.031178	4.01792E-06	0.005653368
1407.079973	174.759935	3.94409E-06	0.005549648
1407.119973	188.386464	4.25162E-06	0.00598254
1407.159973	214.894998	4.84988E-06	0.006824558
1407.199973	232.748982	5.25282E-06	0.007391769
1407.239973	238.045286	5.37235E-06	0.007560186
1407.279973	251.353642	5.6727E-06	0.007983079
1407.319973	245.528984	5.54125E-06	0.007798308
1407.359973	225.795396	5.09589E-06	0.007171748
1407.399973	220.212192	4.96988E-06	0.006994612
1407.439973	215.230463	4.85745E-06	0.006836572
1407.479973	208.69082	4.70986E-06	0.006629035
1407.519973	190.412641	4.29735E-06	0.006048603
1407.559973	163.061123	3.68006E-06	0.005179909
1407.599973	164.217528	3.70616E-06	0.005216792
1407.639973	170.328136	3.84407E-06	0.005411065
1407.679973	156.46621	3.53122E-06	0.004970834
1407.719973	147.549612	3.32999E-06	0.004687692
1407.759973	157.536237	3.55537E-06	0.005005112
1407.799973	184.303357	4.15947E-06	0.005855702
1407.839973	182.384051	4.11615E-06	0.005794886
1407.879973	167.668211	3.78404E-06	0.005327471
1407.919973	168.13548	3.79458E-06	0.00534247
1407.959973	187.215347	4.22519E-06	0.005948898
1407.999973	191.021636	4.31109E-06	0.006070018
1408.039973	194.326931	4.38569E-06	0.006175224
1408.079973	197.679279	4.46135E-06	0.006281932
1408.119973	204.665019	4.619E-06	0.006504112
1408.159973	231.868518	5.23295E-06	0.00736883
1408.199973	230.395674	5.19971E-06	0.007322231
1408.239973	246.501839	5.5632E-06	0.007834325
1408.279973	241.580062	5.45213E-06	0.007678119
1408.319973	240.054381	5.41769E-06	0.007629845
1408.359973	240.196308	5.4209E-06	0.007634573
1408.399973	224.091846	5.05744E-06	0.0071229
1408.439973	206.997873	4.67165E-06	0.006579744
1408.479973	184.319374	4.15983E-06	0.005859039
1408.519973	169.26951	3.82018E-06	0.005380796
1408.559973	159.187387	3.59264E-06	0.005060446

1408.599973	168.867916	3.81111E-06	0.005368335
1408.639973	176.690162	3.98765E-06	0.005617164
1408.679973	176.417742	3.9815E-06	0.005608663
1408.719973	199.750383	4.50809E-06	0.006350633
1408.759973	229.424663	5.1778E-06	0.00729427
1408.799973	230.599768	5.20432E-06	0.00733184
1408.839973	212.523	4.79635E-06	0.006757287
1408.879973	196.944316	4.44476E-06	0.006262132
1408.919973	211.043371	4.76295E-06	0.006710622
1408.959973	197.230137	4.45121E-06	0.006271576
1408.999973	183.841076	4.14904E-06	0.005845993
1409.039973	197.150104	4.4494E-06	0.006269387
1409.079973	231.286481	5.21981E-06	0.007355135
1409.119973	246.915114	5.57253E-06	0.007852364
1409.159973	247.730155	5.59092E-06	0.007878507
1409.199973	237.866153	5.36831E-06	0.007565019
1409.239973	215.270291	4.85835E-06	0.006846582
1409.279973	201.137391	4.53939E-06	0.006397272
1409.319973	218.960085	4.94162E-06	0.00696433
1409.359973	240.841892	5.43547E-06	0.007660528
1409.399973	239.700713	5.40971E-06	0.007624447
1409.439973	218.829552	4.93868E-06	0.00696077
1409.479973	194.034393	4.37909E-06	0.006172234
1409.519973	178.913516	4.03783E-06	0.0056914
1409.559973	190.617801	4.30198E-06	0.006063896
1409.599973	185.522342	4.18698E-06	0.005901968
1409.639973	182.21553	4.11235E-06	0.005796934
1409.679973	189.438736	4.27537E-06	0.006026901
1409.719973	182.23867	4.11287E-06	0.005797999
1409.759973	162.590657	3.66944E-06	0.005173036
1409.799973	158.927318	3.58677E-06	0.005056626
1409.839973	174.884677	3.9469E-06	0.005564502
1409.879973	210.618069	4.75336E-06	0.006701662
1409.919973	231.393352	5.22223E-06	0.00736292
1409.959973	196.05196	4.42462E-06	0.006238537
1409.999973	169.587916	3.82736E-06	0.005396582
1410.039973	167.843867	3.788E-06	0.005341235
1410.079973	167.404924	3.7781E-06	0.005327417
1410.119973	205.43418	4.63636E-06	0.006537828
1410.159973	227.568248	5.1359E-06	0.007242438
1410.199973	213.444221	4.81714E-06	0.006793129
1410.239973	222.884511	5.03019E-06	0.007093779
1410.279973	242.072357	5.46324E-06	0.007704692
1410.319973	237.938366	5.36994E-06	0.00757333
1410.359973	233.65158	5.27319E-06	0.007437097
1410.399973	225.594869	5.09136E-06	0.007180857

1410.439973	187.83047	4.23907E-06	0.005978957
1410.479973	158.002435	3.56589E-06	0.005029623
1410.519973	158.107492	3.56827E-06	0.00503311
1410.559973	167.729246	3.78542E-06	0.005339556
1410.599973	178.311733	4.02425E-06	0.005676603
1410.639973	176.444397	3.9821E-06	0.005617315
1410.679973	184.164426	4.15633E-06	0.005863258
1410.719973	189.298457	4.2722E-06	0.006026881
1410.759973	197.735601	4.46262E-06	0.006295682
1410.799973	222.404525	5.01936E-06	0.007081313
1410.839973	236.678345	5.3415E-06	0.007536003
1410.879973	242.157536	5.46516E-06	0.007710682
1410.919973	234.565938	5.29383E-06	0.007469166
1410.959973	229.568554	5.18104E-06	0.007310244
1410.999973	226.483178	5.11141E-06	0.007212199
1411.039973	225.394547	5.08684E-06	0.007177736
1411.079973	225.876534	5.09772E-06	0.007193289
1411.119973	228.968708	5.1675E-06	0.007291969
1411.159973	236.916781	5.34688E-06	0.007545306
1411.199973	243.897564	5.50443E-06	0.007767849
1411.239973	258.138757	5.82583E-06	0.008221647
1411.279973	266.276412	6.00949E-06	0.00848107
1411.319973	259.898042	5.86554E-06	0.008278149
1411.359973	240.710185	5.43249E-06	0.007667204
1411.399973	219.932774	4.96358E-06	0.007005592
1411.439973	198.28176	4.47494E-06	0.006316114
1411.479973	165.258265	3.72965E-06	0.005264325
1411.519973	137.315195	3.09901E-06	0.004374318
1411.559973	141.437883	3.19206E-06	0.004505778
1411.599973	146.373606	3.30345E-06	0.004663148
1411.639973	156.679201	3.53603E-06	0.004991603
1411.679973	187.603953	4.23396E-06	0.005976996
1411.719973	197.100097	4.44827E-06	0.006279718
1411.759973	187.297603	4.22705E-06	0.005967574
1411.799973	195.714973	4.41701E-06	0.006235941
1411.839973	214.46243	4.84012E-06	0.006833473
1411.879973	210.172243	4.74329E-06	0.006696963
1411.919973	183.248461	4.13566E-06	0.005839225
1411.959973	173.378796	3.91292E-06	0.005524883
1411.999973	184.095508	4.15478E-06	0.005866548
1412.039973	188.785335	4.26062E-06	0.006016169
1412.079973	199.95606	4.51273E-06	0.006372335
1412.119973	215.38847	4.86102E-06	0.00686434
1412.159973	223.891202	5.05291E-06	0.007135521
1412.199973	238.76746	5.38865E-06	0.00760985
1412.239973	256.045897	5.7786E-06	0.008160769

1412.279973	270.495705	6.10471E-06	0.008621561
1412.319973	269.769584	6.08832E-06	0.008598661
1412.359973	240.371969	5.42486E-06	0.007661856
1412.399973	241.129205	5.44195E-06	0.00768621
1412.439973	226.456139	5.1108E-06	0.007218698
1412.479973	203.764396	4.59868E-06	0.006495541
1412.519973	178.680204	4.03256E-06	0.005696076
1412.559973	158.886262	3.58584E-06	0.005065216
1412.599973	155.787184	3.5159E-06	0.00496656
1412.639973	170.183824	3.84081E-06	0.005425684
1412.679973	201.767061	4.5536E-06	0.006432782
1412.719973	220.665492	4.98011E-06	0.007035505
1412.759973	234.268799	5.28712E-06	0.007469432
1412.799973	233.979314	5.28059E-06	0.007460413
1412.839973	203.284431	4.58785E-06	0.006481893
1412.879973	186.599152	4.21128E-06	0.005950037
1412.919973	206.434814	4.65895E-06	0.006582718
1412.959973	228.671446	5.1608E-06	0.007291998
1412.999973	221.959427	5.00932E-06	0.007078162
1413.039973	206.369251	4.65747E-06	0.006581186
1413.079973	197.903713	4.46641E-06	0.006311396
1413.119973	165.936591	3.74496E-06	0.005292075
1413.159973	162.849133	3.67528E-06	0.005193756
1413.199973	196.092965	4.42555E-06	0.00625418
1413.239973	214.993911	4.85211E-06	0.0068572
1413.279973	223.301282	5.0396E-06	0.007122364
1413.319973	241.425346	5.44863E-06	0.007700663
1413.359973	260.234819	5.87314E-06	0.008300857
1413.399973	264.571091	5.971E-06	0.008439413
1413.439973	242.174841	5.46555E-06	0.007725225
1413.479973	192.498216	4.34442E-06	0.006140746
1413.519973	165.014536	3.72415E-06	0.005264158
1413.559973	158.069538	3.56741E-06	0.005042747
1413.599973	159.186435	3.59262E-06	0.005078522
1413.639973	169.493025	3.82522E-06	0.005407486
1413.679973	178.34811	4.02507E-06	0.005690159
1413.719973	189.726049	4.28185E-06	0.006053341
1413.759973	190.289473	4.29457E-06	0.006071489
1413.799973	185.563004	4.1879E-06	0.005920851
1413.839973	183.038971	4.13093E-06	0.00584048
1413.879973	185.615036	4.18907E-06	0.005922846
1413.919973	177.086833	3.9966E-06	0.005650877
1413.959973	148.826612	3.35881E-06	0.004749222
1413.999973	135.959003	3.06841E-06	0.004338725
1414.039973	153.778176	3.47056E-06	0.004907509
1414.079973	182.116559	4.11012E-06	0.005812034

1414.119973	210.214868	4.74426E-06	0.006708948
1414.159973	234.40855	5.29027E-06	0.007481294
1414.199973	239.62218	5.40794E-06	0.007647907
1414.239973	230.741558	5.20752E-06	0.007364677
1414.279973	247.393889	5.58334E-06	0.0078964
1414.319973	273.164577	6.16494E-06	0.008719203
1414.359973	252.747863	5.70417E-06	0.008067746
1414.399973	224.161269	5.05901E-06	0.00715546
1414.439973	201.40943	4.54553E-06	0.00642938
1414.479973	197.275745	4.45224E-06	0.006297603
1414.519973	201.05455	4.53752E-06	0.006418414
1414.559973	200.424452	4.5233E-06	0.00639848
1414.599973	189.724319	4.28181E-06	0.006057053
1414.639973	189.885307	4.28545E-06	0.006062364
1414.679973	180.702606	4.07821E-06	0.005769357
1414.719973	179.743931	4.05657E-06	0.005738911
1414.759973	178.093671	4.01933E-06	0.005686382
1414.799973	190.526295	4.29991E-06	0.006083517
1414.839973	194.551545	4.39076E-06	0.006212219
1414.879973	195.625058	4.41499E-06	0.006246674
1414.919973	215.88498	4.87222E-06	0.006893806
1414.959973	224.979951	5.07748E-06	0.007184437
1414.999973	206.412865	4.65845E-06	0.006591708
1415.039973	209.790394	4.73468E-06	0.006699757
1415.079973	224.272443	5.06152E-06	0.007162451
1415.119973	222.229772	5.01542E-06	0.007097416
1415.159973	236.543874	5.33847E-06	0.007554783
1415.199973	268.917268	6.06909E-06	0.008588973
1415.239973	279.261685	6.30255E-06	0.008919617
1415.279973	280.66526	6.33422E-06	0.0089647
1415.319973	280.323956	6.32652E-06	0.008954052
1415.359973	253.357619	5.71793E-06	0.008092927
1415.399973	214.122687	4.83245E-06	0.006839851
1415.439973	189.207124	4.27014E-06	0.006044129
1415.479973	180.772692	4.07979E-06	0.005774858
1415.519973	162.65744	3.67095E-06	0.005196306
1415.559973	159.961087	3.6101E-06	0.005110312
1415.599973	151.912019	3.42844E-06	0.004853303
1415.639973	130.38139	2.94253E-06	0.004165558
1415.679973	137.408077	3.10111E-06	0.004390178
1415.719973	151.078685	3.40964E-06	0.004827089
1415.759973	159.633122	3.6027E-06	0.005100555
1415.799973	173.318242	3.91155E-06	0.005537974
1415.839973	171.200012	3.86375E-06	0.005470446
1415.879973	173.268041	3.91042E-06	0.005536683
1415.919973	182.492887	4.11861E-06	0.005831623

1415.959973	194.142441	4.38152E-06	0.006204063
1415.999973	195.361275	4.40903E-06	0.006243189
1416.039973	183.020651	4.13052E-06	0.005848983
1416.079973	199.200463	4.49568E-06	0.006366238
1416.119973	214.794872	4.84762E-06	0.006864813
1416.159973	218.088463	4.92195E-06	0.006970273
1416.199973	246.511147	5.56341E-06	0.007878906
1416.239973	266.842358	6.02226E-06	0.008528966
1416.279973	272.039104	6.13954E-06	0.008695313
1416.319973	266.085075	6.00517E-06	0.008505242
1416.359973	231.800775	5.23142E-06	0.007409575
1416.399973	205.087557	4.62854E-06	0.006555865
1416.439973	196.177088	4.42744E-06	0.006271208
1416.479973	207.777081	4.68924E-06	0.006642214
1416.519973	200.49035	4.52479E-06	0.006409452
1416.559973	179.198478	4.04426E-06	0.005728937
1416.599973	162.033133	3.65686E-06	0.005180311
1416.639973	153.613624	3.46685E-06	0.004911272
1416.679973	159.347278	3.59625E-06	0.00509473
1416.719973	168.490004	3.80258E-06	0.005387198
1416.759973	174.135604	3.93E-06	0.005567864
1416.799973	192.987691	4.35546E-06	0.00617082
1416.839973	216.280834	4.88116E-06	0.006915819
1416.879973	197.93353	4.46708E-06	0.006329322
1416.919973	183.007607	4.13023E-06	0.005852201
1416.959973	187.040995	4.22125E-06	0.005981349
1416.999973	197.826044	4.46466E-06	0.006326421
1417.039973	203.152353	4.58487E-06	0.006496938
1417.079973	204.522828	4.6158E-06	0.006540951
1417.119973	201.430329	4.546E-06	0.00644223
1417.159973	201.118264	4.53896E-06	0.006432431
1417.199973	228.316276	5.15278E-06	0.00730252
1417.239973	260.559962	5.88048E-06	0.008334045
1417.279973	276.140554	6.23211E-06	0.008832641
1417.319973	277.732426	6.26803E-06	0.00888381
1417.359973	252.992654	5.70969E-06	0.008092689
1417.399973	222.762361	5.02744E-06	0.007125888
1417.439973	210.130216	4.74235E-06	0.006721991
1417.479973	195.709498	4.41689E-06	0.006260854
1417.519973	180.338666	4.06999E-06	0.005769296
1417.559973	144.972091	3.27182E-06	0.004637998
1417.599973	145.735755	3.28905E-06	0.004662561
1417.639973	166.43736	3.75626E-06	0.005325023
1417.679973	181.821809	4.10346E-06	0.0058174
1417.719973	188.621505	4.25692E-06	0.006035127
1417.759973	171.820652	3.87775E-06	0.005497723

1417.799973	171.968684	3.88109E-06	0.005502614
1417.839973	187.26163	4.22623E-06	0.005992124
1417.879973	183.746778	4.14691E-06	0.005879819
1417.919973	175.430353	3.95922E-06	0.005613855
1417.959973	171.604963	3.87288E-06	0.005491596
1417.999973	195.418779	4.41033E-06	0.006253847
1418.039973	232.760589	5.25308E-06	0.007449081
1418.079973	226.393949	5.1094E-06	0.007245532
1418.119973	190.4114	4.29732E-06	0.006094115
1418.159973	182.404232	4.11661E-06	0.005838011
1418.199973	205.436035	4.63641E-06	0.00657535
1418.239973	261.0078	5.89058E-06	0.00835426
1418.279973	282.916451	6.38503E-06	0.009055761
1418.319973	285.295083	6.43871E-06	0.009132155
1418.359973	281.710188	6.35781E-06	0.009017658
1418.399973	253.848591	5.72901E-06	0.008126026
1418.439973	218.24744	4.92554E-06	0.006986584
1418.479973	193.467909	4.3663E-06	0.006193511
1418.519973	181.259229	4.09077E-06	0.005802837
1418.559973	169.345999	3.8219E-06	0.005421599
1418.599973	157.801877	3.56137E-06	0.005052157
1418.639973	169.439959	3.82402E-06	0.005424913
1418.679973	194.578889	4.39137E-06	0.006229955
1418.719973	207.149091	4.67507E-06	0.00663261
1418.759973	205.532573	4.63858E-06	0.006581037
1418.799973	208.274289	4.70046E-06	0.006669013
1418.839973	189.259772	4.27133E-06	0.006060333
1418.879973	173.719039	3.9206E-06	0.005562856
1418.919973	177.050052	3.99577E-06	0.005669682
1418.959973	183.79862	4.14808E-06	0.005885958
1418.999973	193.740164	4.37245E-06	0.0062045
1419.039973	191.14981	4.31398E-06	0.006121717
1419.079973	203.499112	4.59269E-06	0.006517397
1419.119973	214.351237	4.83761E-06	0.006865147
1419.159973	223.033993	5.03357E-06	0.007143436
1419.199973	207.537625	4.68384E-06	0.006647299
1419.239973	202.637202	4.57324E-06	0.006490524
1419.279973	214.961322	4.85138E-06	0.006885463
1419.319973	229.647417	5.18282E-06	0.007356083
1419.359973	257.370979	5.8085E-06	0.008244359
1419.399973	260.670205	5.88296E-06	0.008350278
1419.439973	212.564671	4.79729E-06	0.006809463
1419.479973	194.391712	4.38715E-06	0.006227472
1419.519973	196.646856	4.43805E-06	0.006299894
1419.559973	187.386629	4.22906E-06	0.006003397
1419.599973	190.699009	4.30381E-06	0.00610969

1419.639973	185.878936	4.19503E-06	0.00595543
1419.679973	170.774895	3.85415E-06	0.005471662
1419.719973	172.442428	3.89179E-06	0.005525245
1419.759973	172.381099	3.8904E-06	0.005523436
1419.799973	176.11875	3.97475E-06	0.005643357
1419.839973	172.188521	3.88606E-06	0.005517576
1419.879973	164.930362	3.72225E-06	0.005285146
1419.919973	170.168012	3.84046E-06	0.005453139
1419.959973	166.852059	3.76562E-06	0.005347028
1419.999973	147.136722	3.32067E-06	0.004715353
1420.039973	134.604447	3.03783E-06	0.004313847
1420.079973	150.187327	3.38952E-06	0.004813388
1420.119973	182.234695	4.11278E-06	0.005840646
1420.159973	202.466199	4.56938E-06	0.006489251
1420.199973	241.98243	5.46121E-06	0.007756005
1420.239973	256.217636	5.78248E-06	0.008212502
1420.279973	231.935753	5.23447E-06	0.007434408
1420.319973	211.46965	4.77258E-06	0.006778584
1420.359973	214.4048	4.83882E-06	0.006872863
1420.399973	209.992484	4.73924E-06	0.006731613
1420.439973	217.966405	4.9192E-06	0.006987426
1420.479973	214.483952	4.8406E-06	0.006875981
1420.519973	204.544858	4.61629E-06	0.006557536
1420.559973	181.325023	4.09225E-06	0.005813291
1420.599973	168.813818	3.80989E-06	0.005412334
1420.639973	172.73992	3.8985E-06	0.005538364
1420.679973	180.22342	4.06739E-06	0.005778462
1420.719973	182.415412	4.11686E-06	0.005848908
1420.759973	173.010064	3.9046E-06	0.005547494
1420.799973	175.300878	3.9563E-06	0.005621106
1420.839973	185.9822	4.19736E-06	0.005963776
1420.879973	190.655343	4.30283E-06	0.006113799
1420.919973	188.355334	4.25092E-06	0.006040214
1420.959973	171.699102	3.87501E-06	0.005506234
1420.999973	196.086962	4.42541E-06	0.006288507
1421.039973	223.56762	5.04561E-06	0.007170013
1421.079973	227.601835	5.13666E-06	0.0072996
1421.119973	224.523895	5.06719E-06	0.007201087
1421.159973	218.101803	4.92225E-06	0.00699531
1421.199973	227.287211	5.12956E-06	0.007290125
1421.239973	235.596943	5.31709E-06	0.007556868
1421.279973	231.122347	5.21611E-06	0.007413552
1421.319973	224.750845	5.07231E-06	0.007209381
1421.359973	219.016949	4.94291E-06	0.007025651
1421.399973	199.79117	4.50901E-06	0.006409104
1421.439973	194.898257	4.39858E-06	0.006252321

1421.479973	211.253462	4.7677E-06	0.006777185
1421.519973	203.306291	4.58834E-06	0.006522417
1421.559973	180.467236	4.07289E-06	0.005789863
1421.599973	178.856846	4.03655E-06	0.005738359
1421.639973	182.019079	4.10792E-06	0.005839979
1421.679973	172.24399	3.88731E-06	0.005526506
1421.719973	160.385679	3.61968E-06	0.005146173
1421.759973	169.983342	3.83629E-06	0.00545428
1421.799973	174.499422	3.93821E-06	0.005599345
1421.839973	188.053622	4.24411E-06	0.006034443
1421.879973	188.380018	4.25147E-06	0.006045087
1421.919973	185.927321	4.19612E-06	0.005966548
1421.959973	193.212924	4.36055E-06	0.006200523
1421.999973	205.357266	4.63463E-06	0.00659044
1422.039973	204.15892	4.60758E-06	0.006552166
1422.079973	203.755893	4.59849E-06	0.006539416
1422.119973	219.929765	4.96351E-06	0.007058705
1422.159973	226.357572	5.10858E-06	0.007265211
1422.199973	211.370735	4.77034E-06	0.006784382
1422.239973	217.422578	4.90692E-06	0.006978825
1422.279973	228.882171	5.16555E-06	0.007346861
1422.319973	223.000803	5.03282E-06	0.007158277
1422.359973	237.394936	5.35767E-06	0.00762054
1422.399973	241.511885	5.45059E-06	0.007752915
1422.439973	213.934814	4.82821E-06	0.00686784
1422.479973	182.24127	4.11293E-06	0.005850563
1422.519973	180.174298	4.06628E-06	0.005784369
1422.559973	167.318985	3.77616E-06	0.005371809
1422.599973	155.621119	3.51215E-06	0.004996387
1422.639973	141.626219	3.19631E-06	0.004547193
1422.679973	152.280335	3.43676E-06	0.004889403
1422.719973	177.301584	4.00145E-06	0.005692943
1422.759973	205.652657	4.64129E-06	0.006603447
1422.799973	221.005305	4.98778E-06	0.007096616
1422.839973	215.530289	4.86422E-06	0.006921004
1422.879973	181.114547	4.0875E-06	0.005816026
1422.919973	158.939133	3.58703E-06	0.005104064
1422.959973	167.554606	3.78147E-06	0.005380886
1422.999973	192.704044	4.34906E-06	0.006188715
1423.039973	215.607079	4.86595E-06	0.006924443
1423.079973	207.713201	4.6878E-06	0.006671111
1423.119973	198.17228	4.47247E-06	0.006364865
1423.159973	204.647785	4.61862E-06	0.006573029
1423.199973	225.515712	5.08958E-06	0.007243484
1423.239973	261.990264	5.91276E-06	0.00841527
1423.279973	287.665738	6.49222E-06	0.00924024

1423.319973	279.431245	6.30637E-06	0.008975988
1423.359973	253.257983	5.71568E-06	0.00813547
1423.399973	232.867064	5.25549E-06	0.007480658
1423.439973	209.376394	4.72533E-06	0.006726229
1423.479973	204.774792	4.62148E-06	0.006578587
1423.519973	187.941876	4.24159E-06	0.006037983
1423.559973	162.382788	3.66475E-06	0.005216996
1423.599973	157.700724	3.55909E-06	0.005066714
1423.639973	162.372853	3.66453E-06	0.00521697
1423.679973	165.283615	3.73022E-06	0.005310641
1423.719973	173.737051	3.921E-06	0.005582411
1423.759973	185.857108	4.19454E-06	0.005972013
1423.799973	178.743117	4.03398E-06	0.005743585
1423.839973	190.875815	4.3078E-06	0.00613362
1423.879973	198.812926	4.48693E-06	0.006388851
1423.919973	185.046731	4.17625E-06	0.005946641
1423.959973	168.919078	3.81227E-06	0.005428517
1423.999973	172.93207	3.90284E-06	0.005557638
1424.039973	190.168018	4.29183E-06	0.006111734
1424.079973	219.935021	4.96363E-06	0.007068602
1424.119973	231.238185	5.21872E-06	0.007432089
1424.159973	216.554428	4.88733E-06	0.006960342
1424.199973	205.662894	4.64153E-06	0.00661046
1424.239973	199.472989	4.50183E-06	0.006411683
1424.279973	194.997583	4.40082E-06	0.006268005
1424.319973	193.62438	4.36983E-06	0.00622404
1424.359973	193.206214	4.3604E-06	0.006210772
1424.399973	189.670343	4.2806E-06	0.00609728
1424.439973	191.30756	4.31755E-06	0.006150084
1424.479973	186.960806	4.21944E-06	0.006010515
1424.519973	176.8008	3.99015E-06	0.005684045
1424.559973	162.965647	3.67791E-06	0.0052394
1424.599973	156.376166	3.52919E-06	0.005027687
1424.639973	168.729176	3.80798E-06	0.005425004
1424.679973	185.974211	4.19718E-06	0.005979637
1424.719973	194.546897	4.39065E-06	0.00625545
1424.759973	202.410888	4.56813E-06	0.006508491
1424.799973	205.493896	4.63771E-06	0.006607811
1424.839973	204.780725	4.62162E-06	0.006585063
1424.879973	185.510243	4.18671E-06	0.005965556
1424.919973	175.886804	3.96952E-06	0.005656249
1424.959973	172.161492	3.88545E-06	0.005536604
1424.999973	178.94845	4.03862E-06	0.005755029
1425.039973	201.708341	4.55228E-06	0.006487176
1425.079973	201.90303	4.55667E-06	0.006493619
1425.119973	198.208269	4.47328E-06	0.006374967

1425.159973	203.61961	4.59541E-06	0.006549196
1425.199973	219.329941	4.94997E-06	0.007054699
1425.239973	233.858605	5.27786E-06	0.007522221
1425.279973	235.384689	5.3123E-06	0.007571521
1425.319973	231.286797	5.21982E-06	0.007439915
1425.359973	219.303981	4.94939E-06	0.007054656
1425.399973	202.914695	4.5795E-06	0.006527622
1425.439973	191.565228	4.32336E-06	0.006162691
1425.479973	200.473344	4.5244E-06	0.006449447
1425.519973	219.695706	4.95823E-06	0.00706805
1425.559973	209.788178	4.73463E-06	0.006749495
1425.599973	194.30015	4.38508E-06	0.006251375
1425.639973	174.671885	3.9421E-06	0.005620017
1425.679973	162.763747	3.67335E-06	0.005237023
1425.719973	176.548716	3.98446E-06	0.005680722
1425.759973	184.328308	4.16003E-06	0.005931209
1425.799973	191.673909	4.32581E-06	0.006167744
1425.839973	196.711625	4.43951E-06	0.006330027
1425.879973	185.121725	4.17794E-06	0.00595724
1425.919973	174.007848	3.92711E-06	0.005599751
1425.959973	173.911947	3.92495E-06	0.005596822
1425.999973	181.081659	4.08676E-06	0.005827721
1426.039973	190.076816	4.28977E-06	0.006117382
1426.079973	184.591104	4.16596E-06	0.005940998
1426.119973	173.068287	3.90591E-06	0.005570297
1426.159973	183.205742	4.1347E-06	0.005896741
1426.199973	188.174412	4.24683E-06	0.006056835
1426.239973	208.755233	4.71131E-06	0.006719466
1426.279973	224.774628	5.07285E-06	0.007235305
1426.319973	207.884741	4.69167E-06	0.006691821
1426.359973	194.090947	4.38036E-06	0.006247973
1426.399973	207.996575	4.69419E-06	0.006695797
1426.439973	223.324519	5.04012E-06	0.007189433
1426.479973	224.525218	5.06722E-06	0.00722829
1426.519973	215.068845	4.8538E-06	0.006924049
1426.559973	205.490109	4.63763E-06	0.006615851
1426.599973	191.524106	4.32243E-06	0.006166382
1426.639973	158.28932	3.57237E-06	0.005096485
1426.679973	157.441779	3.55324E-06	0.005069339
1426.719973	158.168471	3.56964E-06	0.00509288
1426.759973	159.867948	3.608E-06	0.005147746
1426.799973	170.610043	3.85043E-06	0.005493795
1426.839973	197.037228	4.44686E-06	0.006344952
1426.879973	204.080465	4.60581E-06	0.006571941
1426.919973	210.429833	4.74911E-06	0.006776597
1426.959973	217.248297	4.90299E-06	0.006996373

1426.999973	208.121146	4.697E-06	0.006702625
1427.039973	207.695505	4.6874E-06	0.006689105
1427.079973	224.752438	5.07235E-06	0.007238648
1427.119973	229.174235	5.17214E-06	0.007381269
1427.159973	214.458938	4.84004E-06	0.006907511
1427.199973	216.172001	4.8787E-06	0.006962882
1427.239973	221.943681	5.00896E-06	0.007148988
1427.279973	230.989574	5.21311E-06	0.007440572
1427.319973	240.571462	5.42936E-06	0.007749438
1427.359973	232.302808	5.24275E-06	0.007483293
1427.399973	226.200157	5.10502E-06	0.007286909
1427.439973	196.990103	4.44579E-06	0.006346102
1427.479973	178.195724	4.02163E-06	0.005740795
1427.519973	185.809875	4.19347E-06	0.005986262
1427.559973	174.276317	3.93317E-06	0.005614841
1427.599973	172.234266	3.88709E-06	0.005549206
1427.639973	177.588538	4.00793E-06	0.005721875
1427.679973	166.64406	3.76092E-06	0.005369396
1427.719973	163.34715	3.68652E-06	0.005263315
1427.759973	159.137295	3.59151E-06	0.00512781
1427.799973	171.541806	3.87146E-06	0.00552767
1427.839973	190.40149	4.2971E-06	0.006135566
1427.879973	196.570729	4.43633E-06	0.006334543
1427.919973	177.172162	3.99853E-06	0.005709579
1427.959973	164.705308	3.71717E-06	0.005307969
1427.999973	171.949143	3.88065E-06	0.005541572
1428.039973	185.792269	4.19307E-06	0.005987875
1428.079973	192.20461	4.33779E-06	0.006194711
1428.119973	175.457736	3.95984E-06	0.005655122
1428.159973	179.444264	4.04981E-06	0.005783772
1428.199973	191.454213	4.32085E-06	0.006171045
1428.239973	193.500535	4.36704E-06	0.006237178
1428.279973	191.984326	4.33282E-06	0.006188478
1428.319973	183.81102	4.14836E-06	0.005925184
1428.359973	184.4694	4.16322E-06	0.005946573
1428.399973	214.651133	4.84438E-06	0.006919708
1428.439973	229.292941	5.17482E-06	0.007391923
1428.479973	218.580914	4.93307E-06	0.007046787
1428.519973	213.361167	4.81526E-06	0.006878701
1428.559973	169.411477	3.82338E-06	0.005461929
1428.599973	149.439743	3.37265E-06	0.004818163
1428.639973	167.990318	3.79131E-06	0.005416413
1428.679973	191.637889	4.325E-06	0.006179041
1428.719973	192.296452	4.33986E-06	0.006200449
1428.759973	181.365964	4.09318E-06	0.005848168
1428.799973	171.833821	3.87805E-06	0.005540958

1428.839973	161.07446	3.63523E-06	0.005194157
1428.879973	171.677975	3.87453E-06	0.005536242
1428.919973	193.57093	4.36863E-06	0.006242417
1428.959973	200.582885	4.52688E-06	0.006468725
1428.999973	212.84205	4.80355E-06	0.006864271
1429.039973	216.355117	4.88283E-06	0.006977764
1429.079973	200.224938	4.5188E-06	0.006457724
1429.119973	195.071739	4.4025E-06	0.006291697
1429.159973	214.281168	4.83603E-06	0.006911457
1429.199973	235.549763	5.31603E-06	0.00759767
1429.239973	246.397442	5.56085E-06	0.007947785
1429.279973	259.390297	5.85408E-06	0.008367116
1429.319973	245.564009	5.54204E-06	0.007921345
1429.359973	233.403781	5.2676E-06	0.007529294
1429.399973	219.102875	4.94485E-06	0.007068164
1429.439973	205.603482	4.64018E-06	0.006632865
1429.479973	192.348379	4.34103E-06	0.006205423
1429.519973	189.496016	4.27666E-06	0.006113573
1429.559973	201.262291	4.54221E-06	0.006493361
1429.599973	191.838407	4.32953E-06	0.00618949
1429.639973	182.71265	4.12357E-06	0.005895221
1429.679973	196.148478	4.4268E-06	0.006328904
1429.719973	186.390983	4.20658E-06	0.006014238
1429.759973	155.919335	3.51888E-06	0.005031157
1429.799973	139.841431	3.15603E-06	0.004512486
1429.839973	160.689436	3.62654E-06	0.005185367
1429.879973	186.522789	4.20956E-06	0.006019165
1429.919973	173.655597	3.91916E-06	0.005604092
1429.959973	154.678061	3.49087E-06	0.004991802
1429.999973	160.632942	3.62526E-06	0.005184124
1430.039973	169.944388	3.83541E-06	0.005484787
1430.079973	189.146009	4.26876E-06	0.006104671
1430.119973	201.689389	4.55185E-06	0.00650969
1430.159973	203.928337	4.60238E-06	0.006582138
1430.199973	209.745852	4.73367E-06	0.006770097
1430.239973	214.774389	4.84716E-06	0.0069326
1430.279973	240.1268	5.41933E-06	0.007751155
1430.319973	248.113485	5.59958E-06	0.008009185
1430.359972	228.420674	5.15514E-06	0.007373701
1430.399972	224.360981	5.06351E-06	0.007242851
1430.439972	198.061542	4.46997E-06	0.006394028
1430.479972	174.965083	3.94872E-06	0.005648562
1430.519972	175.804193	3.96766E-06	0.005675811
1430.559972	178.547222	4.02956E-06	0.00576453
1430.599972	178.169923	4.02105E-06	0.00575251
1430.639972	162.346046	3.66392E-06	0.005241756

1430.679972	169.223615	3.81914E-06	0.005463969
1430.719972	190.653694	4.30279E-06	0.006156085
1430.759972	226.816564	5.11893E-06	0.007323966
1430.799972	234.776057	5.29857E-06	0.007581192
1430.839972	200.269991	4.51981E-06	0.006467132
1430.879972	174.373689	3.93537E-06	0.005631044
1430.919972	174.405088	3.93608E-06	0.005632215
1430.959972	188.345932	4.25071E-06	0.006082589
1430.999972	207.650174	4.68638E-06	0.006706203
1431.039972	225.599979	5.09148E-06	0.007286108
1431.079972	211.586642	4.77522E-06	0.006833716
1431.119972	192.867851	4.35276E-06	0.00622932
1431.159972	205.217281	4.63147E-06	0.006628372
1431.199972	230.546936	5.20312E-06	0.00744671
1431.239972	217.451671	4.90758E-06	0.007023927
1431.279972	212.040132	4.78545E-06	0.006849319
1431.319972	218.579609	4.93304E-06	0.007060755
1431.359972	202.616496	4.57277E-06	0.006545283
1431.399972	191.165992	4.31435E-06	0.006175561
1431.439972	194.765393	4.39558E-06	0.006292014
1431.479972	176.396715	3.98103E-06	0.005698762
1431.519972	165.355843	3.73185E-06	0.005342219
1431.559972	173.180572	3.90844E-06	0.005595172
1431.599972	166.982489	3.76856E-06	0.005395074
1431.639972	149.444112	3.37275E-06	0.004828557
1431.679972	150.860909	3.40472E-06	0.00487447
1431.719972	160.749635	3.6279E-06	0.00519413
1431.759972	172.200014	3.88631E-06	0.005564269
1431.799972	180.535516	4.07444E-06	0.005833776
1431.839972	178.644874	4.03177E-06	0.005772844
1431.879972	172.686785	3.8973E-06	0.005580466
1431.919972	174.557604	3.93952E-06	0.00564108
1431.959972	172.725738	3.89818E-06	0.005582037
1431.999972	165.824395	3.74243E-06	0.005359153
1432.039972	178.304485	4.02408E-06	0.005762649
1432.079972	168.23194	3.79676E-06	0.005437265
1432.119972	170.092466	3.83875E-06	0.005497551
1432.159972	203.389476	4.59022E-06	0.006573925
1432.199972	237.328506	5.35617E-06	0.007671112
1432.239972	246.898055	5.57215E-06	0.007980649
1432.279972	261.474287	5.90111E-06	0.008452042
1432.319972	260.847914	5.88697E-06	0.008432031
1432.359972	251.819108	5.68321E-06	0.008140398
1432.399972	252.461036	5.69769E-06	0.008161377
1432.439972	240.971842	5.4384E-06	0.00779018
1432.479972	216.365375	4.88307E-06	0.006994893

1432.519972	199.901788	4.5115E-06	0.006462821
1432.559972	176.653684	3.98683E-06	0.00571137
1432.599972	172.808801	3.90005E-06	0.005587217
1432.639972	196.75839	4.44056E-06	0.006361728
1432.679972	191.498976	4.32187E-06	0.00619185
1432.719972	164.971249	3.72317E-06	0.005334262
1432.759972	148.010072	3.34038E-06	0.004785964
1432.799972	153.379928	3.46157E-06	0.004959739
1432.839972	180.046734	4.0634E-06	0.005822208
1432.879972	193.067683	4.35727E-06	0.006243443
1432.919972	197.194921	4.45041E-06	0.006377088
1432.959972	187.129271	4.22325E-06	0.006051744
1432.999972	169.358715	3.82219E-06	0.005477199
1433.039972	183.451542	4.14025E-06	0.005933138
1433.079972	207.003196	4.67177E-06	0.006695025
1433.119972	208.730973	4.71077E-06	0.006751095
1433.159972	201.94185	4.55755E-06	0.006531693
1433.199972	213.077739	4.80887E-06	0.006892069
1433.239972	237.284406	5.35518E-06	0.007675256
1433.279972	246.093021	5.55398E-06	0.007960404
1433.319972	259.370265	5.85363E-06	0.008390118
1433.359972	248.168817	5.60082E-06	0.008027998
1433.399972	218.784755	4.93767E-06	0.007077652
1433.439972	212.594538	4.79796E-06	0.006877591
1433.479972	201.029314	4.53695E-06	0.006503629
1433.519972	175.221015	3.95449E-06	0.005668846
1433.559972	157.777069	3.56081E-06	0.005104633
1433.599972	141.525007	3.19402E-06	0.00457895
1433.639972	157.590839	3.55661E-06	0.005098892
1433.679972	166.509626	3.75789E-06	0.005387612
1433.719972	171.569693	3.87209E-06	0.005551491
1433.759972	166.183697	3.75053E-06	0.005377366
1433.799972	161.184063	3.6377E-06	0.005215734
1433.839972	188.402284	4.25198E-06	0.006096655
1433.879972	206.929757	4.67012E-06	0.006696386
1433.919972	204.204235	4.60861E-06	0.006608371
1433.959972	203.755348	4.59847E-06	0.006594028
1433.999972	182.296669	4.11418E-06	0.005899737
1434.039972	168.879227	3.81137E-06	0.005465655
1434.079972	177.864832	4.01416E-06	0.005756629
1434.119972	195.31443	4.40797E-06	0.006321564
1434.159972	202.138585	4.56199E-06	0.006542618
1434.199972	213.118559	4.80979E-06	0.006898199
1434.239972	193.342269	4.36347E-06	0.006258257
1434.279972	175.541508	3.96173E-06	0.005682226
1434.319972	202.942301	4.58013E-06	0.006569365

1434.359972	237.580984	5.36187E-06	0.007690854
1434.399972	243.45645	5.49447E-06	0.007881272
1434.439972	227.502099	5.13441E-06	0.007364996
1434.479972	200.886785	4.53373E-06	0.006503552
1434.519972	154.391976	3.48441E-06	0.004998458
1434.559972	133.826317	3.02027E-06	0.004332764
1434.599972	145.631286	3.2867E-06	0.004715093
1434.639972	179.177714	4.04379E-06	0.005801385
1434.679972	193.968858	4.37761E-06	0.006280465
1434.719972	208.414999	4.70364E-06	0.006748401
1434.759972	210.522461	4.7512E-06	0.00681683
1434.799972	202.684975	4.57432E-06	0.006563231
1434.839972	200.103078	4.51605E-06	0.006479806
1434.879972	216.608935	4.88856E-06	0.0070145
1434.919972	227.958683	5.14471E-06	0.007382247
1434.959972	210.719549	4.75565E-06	0.006824163
1434.999972	185.629785	4.18941E-06	0.006011797
1435.039972	173.686089	3.91985E-06	0.005625146
1435.079972	161.857702	3.6529E-06	0.005242208
1435.119972	179.603491	4.0534E-06	0.005817116
1435.159972	201.069562	4.53786E-06	0.006512555
1435.199972	229.19273	5.17256E-06	0.007423659
1435.239972	237.028184	5.3494E-06	0.007677667
1435.279972	220.392545	4.97395E-06	0.007139015
1435.319972	220.059443	4.96644E-06	0.007128424
1435.359972	231.211979	5.21813E-06	0.007489898
1435.399972	232.018432	5.23633E-06	0.007516232
1435.439972	225.962975	5.09967E-06	0.00732027
1435.479972	213.645865	4.82169E-06	0.006921439
1435.519972	189.474687	4.27618E-06	0.006138541
1435.559972	171.295933	3.86591E-06	0.005549747
1435.599972	170.020881	3.83713E-06	0.00550859
1435.639972	172.138149	3.88492E-06	0.005577344
1435.679972	171.360817	3.86737E-06	0.005552313
1435.719972	192.81747	4.35162E-06	0.00624771
1435.759972	201.771003	4.55369E-06	0.006538007
1435.799972	192.016334	4.33354E-06	0.006222098
1435.839972	198.497267	4.47981E-06	0.006432286
1435.879972	192.985659	4.35542E-06	0.006253857
1435.919972	185.42223	4.18472E-06	0.006008925
1435.959972	176.111269	3.97459E-06	0.005707346
1435.999972	172.684829	3.89726E-06	0.00559646
1436.039972	152.950239	3.45187E-06	0.004957029
1436.079972	153.282881	3.45938E-06	0.004967948
1436.119972	183.708605	4.14605E-06	0.005954221
1436.159972	213.95172	4.82859E-06	0.006934631

1436.199972	224.695902	5.07107E-06	0.007283076
1436.239972	218.571358	4.93285E-06	0.007084758
1436.279972	225.800566	5.096E-06	0.007319289
1436.319972	228.294269	5.15228E-06	0.007400328
1436.359972	249.439319	5.6295E-06	0.008085986
1436.399972	235.567083	5.31642E-06	0.007636507
1436.439972	204.445005	4.61404E-06	0.00662779
1436.479972	197.623313	4.46008E-06	0.00640682
1436.519972	191.166345	4.31436E-06	0.006197662
1436.559972	166.398902	3.75539E-06	0.005394845
1436.599972	162.730123	3.67259E-06	0.005276046
1436.639972	185.793083	4.19309E-06	0.006023962
1436.679972	187.211434	4.2251E-06	0.006070118
1436.719972	161.71496	3.64968E-06	0.00524357
1436.759972	147.122591	3.32035E-06	0.004770549
1436.799972	183.136419	4.13313E-06	0.005938487
1436.839972	212.668814	4.79964E-06	0.006896313
1436.879972	206.954258	4.67067E-06	0.006711191
1436.919972	193.935376	4.37685E-06	0.006289185
1436.959972	194.227079	4.38343E-06	0.00629882
1436.999972	199.325123	4.49849E-06	0.006464331
1437.039972	177.415868	4.00403E-06	0.00575395
1437.079972	170.844314	3.85572E-06	0.005540975
1437.119972	199.117413	4.4938E-06	0.006458134
1437.159972	247.136819	5.57753E-06	0.008015808
1437.199972	273.683349	6.17665E-06	0.008877084
1437.239972	256.464548	5.78805E-06	0.008318813
1437.279972	261.688577	5.90595E-06	0.008488499
1437.319972	256.81633	5.79599E-06	0.008330688
1437.359972	227.526717	5.13496E-06	0.007380788
1437.399972	199.870007	4.51079E-06	0.006483806
1437.439972	198.157148	4.47213E-06	0.00642842
1437.479972	192.954459	4.35471E-06	0.006259813
1437.519972	171.038878	3.86011E-06	0.005548984
1437.559972	153.548994	3.46539E-06	0.004981702
1437.599972	146.079049	3.2968E-06	0.004739481
1437.639972	131.888613	2.97654E-06	0.004279196
1437.679972	149.139676	3.36587E-06	0.004839051
1437.719972	171.367464	3.86752E-06	0.005560418
1437.759972	180.335141	4.06991E-06	0.005851558
1437.799972	184.068967	4.15418E-06	0.00597288
1437.839972	169.560257	3.82674E-06	0.005502238
1437.879972	160.107086	3.61339E-06	0.005195627
1437.919972	147.49062	3.32866E-06	0.004786344
1437.959972	149.306601	3.36964E-06	0.00484541
1437.999972	163.402192	3.68776E-06	0.005302999

1438.039972	163.544463	3.69097E-06	0.005307763
1438.079972	170.36613	3.84493E-06	0.005529311
1438.119972	184.351666	4.16056E-06	0.005983385
1438.159972	180.439932	4.07228E-06	0.005856587
1438.199972	184.910997	4.17318E-06	0.006001873
1438.239972	207.78951	4.68952E-06	0.006744655
1438.279972	221.870456	5.00731E-06	0.00720191
1438.319972	214.923096	4.85051E-06	0.006976592
1438.359972	195.392241	4.40973E-06	0.00634278
1438.399972	172.81941	3.90029E-06	0.005610182
1438.439972	169.40354	3.8232E-06	0.005499446
1438.479972	181.229545	4.0901E-06	0.005883525
1438.519972	167.481778	3.77983E-06	0.005437362
1438.559972	159.778842	3.60599E-06	0.005187427
1438.599972	179.289559	4.04632E-06	0.00582103
1438.639972	198.279394	4.47489E-06	0.006437755
1438.679972	198.753176	4.48558E-06	0.006453317
1438.719972	203.172451	4.58532E-06	0.00659699
1438.759972	188.430301	4.25261E-06	0.006118484
1438.799972	186.33442	4.20531E-06	0.006050597
1438.839972	202.690702	4.57445E-06	0.006581897
1438.879972	196.365296	4.43169E-06	0.006376672
1438.919972	179.017212	4.04017E-06	0.00581348
1438.959972	171.532426	3.87125E-06	0.005570571
1438.999972	163.719625	3.69492E-06	0.005316995
1439.039972	165.497157	3.73504E-06	0.005374872
1439.079972	178.151829	4.02064E-06	0.005786021
1439.119972	190.986839	4.31031E-06	0.006203049
1439.159972	196.546477	4.43578E-06	0.006383797
1439.199972	200.263603	4.51967E-06	0.00650471
1439.239972	217.403621	4.9065E-06	0.007061626
1439.279972	243.05416	5.48539E-06	0.007895017
1439.319972	249.874027	5.63931E-06	0.00811677
1439.359972	234.71288	5.29714E-06	0.007624495
1439.399972	228.267337	5.15168E-06	0.007415322
1439.439972	232.93801	5.25709E-06	0.00756726
1439.479972	224.048329	5.05646E-06	0.007278671
1439.519972	209.935841	4.73796E-06	0.006820387
1439.559972	186.636528	4.21213E-06	0.006063609
1439.599972	156.683806	3.53614E-06	0.00509062
1439.639972	131.390426	2.9653E-06	0.004268963
1439.679972	135.465485	3.05727E-06	0.004401487
1439.719972	160.849481	3.63015E-06	0.005226398
1439.759972	198.08972	4.47061E-06	0.006436604
1439.799972	216.056596	4.8761E-06	0.007020604
1439.839972	191.239612	4.31601E-06	0.006214366

1439.879972	173.197055	3.90882E-06	0.005628226
1439.919972	149.062751	3.36414E-06	0.00484409
1439.959972	143.828461	3.24601E-06	0.004674122
1439.999972	160.601197	3.62455E-06	0.005219345
1440.039972	183.652694	4.14479E-06	0.005968657
1440.079972	188.449303	4.25304E-06	0.006124715
1440.119972	187.952374	4.24182E-06	0.006108734
1440.159972	194.847706	4.39744E-06	0.006333019
1440.199972	190.769854	4.30541E-06	0.006200651
1440.239972	202.553765	4.57136E-06	0.00658385
1440.279972	210.060373	4.74077E-06	0.006828036
1440.319972	226.771234	5.11791E-06	0.007371429
1440.359972	228.428861	5.15532E-06	0.007425518
1440.399972	212.565207	4.7973E-06	0.006910032
1440.439972	206.002688	4.64919E-06	0.006696884
1440.479972	205.177144	4.63056E-06	0.006670232
1440.519972	189.461428	4.27588E-06	0.006159491
1440.559972	179.270976	4.0459E-06	0.005828356
1440.599972	164.347212	3.70909E-06	0.005343312
1440.639972	157.361503	3.55143E-06	0.005116332
1440.679972	152.682058	3.44582E-06	0.004964326
1440.719972	148.354939	3.34816E-06	0.004823767
1440.759972	151.086652	3.40982E-06	0.004912725
1440.799972	142.426934	3.21438E-06	0.004631275
1440.839972	142.678809	3.22006E-06	0.004639594
1440.879972	158.119776	3.56854E-06	0.005141842
1440.919972	184.979763	4.17474E-06	0.00601546
1440.959972	202.017698	4.55926E-06	0.006569708
1440.999972	194.666908	4.39336E-06	0.006330833
1441.039972	172.752036	3.89877E-06	0.005618287
1441.079972	166.992905	3.7688E-06	0.005431138
1441.119972	172.249644	3.88743E-06	0.00560226
1441.159972	194.482235	4.38919E-06	0.006325529
1441.199972	222.271069	5.01635E-06	0.007229561
1441.239972	239.01623	5.39426E-06	0.007774428
1441.279972	249.089714	5.62161E-06	0.008102311
1441.319972	267.452757	6.03604E-06	0.00869986
1441.359972	261.742388	5.90716E-06	0.008514346
1441.399972	218.740509	4.93667E-06	0.007115714
1441.439972	184.269997	4.15872E-06	0.005994541
1441.479972	172.466471	3.89233E-06	0.005610713
1441.519972	172.696855	3.89753E-06	0.005618364
1441.559972	161.581767	3.64668E-06	0.005256901
1441.599972	153.428747	3.46267E-06	0.004991789
1441.639972	158.522012	3.57762E-06	0.005157641
1441.679972	177.63287	4.00893E-06	0.005779589

1441.719972	186.383242	4.20641E-06	0.006064466
1441.759972	186.840422	4.21673E-06	0.00607951
1441.799972	191.817828	4.32906E-06	0.00624164
1441.839972	183.979768	4.15217E-06	0.00598676
1441.879972	190.415595	4.29741E-06	0.006196356
1441.919972	194.762412	4.39552E-06	0.006337983
1441.959972	202.933361	4.57992E-06	0.006604066
1441.999972	211.041135	4.7629E-06	0.006868108
1442.039972	202.569939	4.57172E-06	0.006592605
1442.079972	173.750575	3.92131E-06	0.00565484
1442.119972	174.029529	3.9276E-06	0.005664076
1442.159972	192.596434	4.34663E-06	0.00626854
1442.199972	210.416129	4.7488E-06	0.006848718
1442.239972	242.852228	5.48084E-06	0.007904682
1442.279972	257.075676	5.80184E-06	0.008367878
1442.319972	249.624764	5.63368E-06	0.008125574
1442.359972	213.438726	4.81701E-06	0.006947869
1442.399972	182.425708	4.11709E-06	0.005938496
1442.439972	171.323359	3.86653E-06	0.005577237
1442.479972	153.111727	3.45552E-06	0.004984516
1442.519972	142.500866	3.21605E-06	0.004639211
1442.559972	149.61228	3.37654E-06	0.004870863
1442.599972	160.908296	3.63148E-06	0.005238767
1442.639972	177.745774	4.01147E-06	0.005787114
1442.679972	208.806368	4.71247E-06	0.006798584
1442.719972	207.496563	4.68291E-06	0.006756125
1442.759972	190.895224	4.30824E-06	0.006215755
1442.799972	183.033701	4.13082E-06	0.005959941
1442.839972	193.930187	4.37673E-06	0.006314927
1442.879972	203.180176	4.58549E-06	0.006616317
1442.919972	179.390511	4.04859E-06	0.005841797
1442.959972	175.246249	3.95506E-06	0.005706999
1442.999972	193.808379	4.37399E-06	0.00631166
1443.039972	211.532038	4.77398E-06	0.006889049
1443.079972	191.118102	4.31327E-06	0.006224393
1443.119972	182.726461	4.12388E-06	0.005951256
1443.159972	191.040697	4.31152E-06	0.006222217
1443.199972	215.955268	4.87381E-06	0.007033882
1443.239972	252.267409	5.69332E-06	0.008216833
1443.279972	274.780459	6.20141E-06	0.008950374
1443.319972	265.829241	5.9994E-06	0.008659048
1443.359972	232.161794	5.23957E-06	0.007562583
1443.399972	209.409669	4.72608E-06	0.00682163
1443.439972	186.474738	4.20848E-06	0.006074681
1443.479972	191.432915	4.32037E-06	0.006236374
1443.519972	188.335919	4.25048E-06	0.006135652

1443.559972	181.015241	4.08526E-06	0.00589732
1443.599972	181.751273	4.10187E-06	0.005921464
1443.639972	181.376158	4.09341E-06	0.005909406
1443.679972	160.647195	3.62558E-06	0.005234182
1443.719972	140.34926	3.16749E-06	0.004572965
1443.759972	157.558111	3.55587E-06	0.005133819
1443.799972	182.579701	4.12057E-06	0.005949278
1443.839972	189.663104	4.28043E-06	0.006180259
1443.879972	188.912494	4.26349E-06	0.006155971
1443.919972	180.971812	4.08428E-06	0.005897376
1443.959972	194.847307	4.39743E-06	0.006349716
1443.999972	203.866567	4.60098E-06	0.006643821
1444.039972	185.453712	4.18543E-06	0.006043931
1444.079972	172.013084	3.8821E-06	0.005606057
1444.119972	167.933465	3.79002E-06	0.00547325
1444.159972	195.600067	4.41442E-06	0.00637513
1444.199972	206.640685	4.66359E-06	0.00673516
1444.239972	203.332808	4.58894E-06	0.006627528
1444.279972	215.394337	4.86115E-06	0.007020862
1444.319972	217.264355	4.90335E-06	0.007082012
1444.359972	201.75058	4.55323E-06	0.006576503
1444.399972	210.107065	4.74182E-06	0.00684909
1444.439972	215.667007	4.8673E-06	0.007030528
1444.479972	190.796946	4.30602E-06	0.006219962
1444.519972	170.058674	3.83799E-06	0.005544049
1444.559972	187.00983	4.22055E-06	0.00609684
1444.599972	193.472835	4.36641E-06	0.006307719
1444.639972	177.094777	3.99678E-06	0.005773912
1444.679972	162.174767	3.66006E-06	0.005287613
1444.719972	172.565893	3.89457E-06	0.005626566
1444.759972	182.020778	4.10796E-06	0.00593501
1444.799972	193.224123	4.3608E-06	0.006300483
1444.839972	192.709532	4.34919E-06	0.006283877
1444.879972	195.406603	4.41005E-06	0.006372
1444.919972	205.258571	4.6324E-06	0.006693447
1444.959972	219.128938	4.94543E-06	0.007145955
1444.999972	250.957033	5.66375E-06	0.00818412
1445.039972	218.719591	4.9362E-06	0.007133001
1445.079972	185.855869	4.19451E-06	0.0060614
1445.119972	185.248393	4.1808E-06	0.006041755
1445.159972	197.56948	4.45887E-06	0.006443778
1445.199972	225.305769	5.08484E-06	0.007348607
1445.239972	253.724582	5.72621E-06	0.008275748
1445.279972	263.135284	5.9386E-06	0.008582935
1445.319972	257.460364	5.81052E-06	0.008398063
1445.359972	227.197775	5.12754E-06	0.007411137

1445.399972	224.34337	5.06312E-06	0.00731823
1445.439972	219.15963	4.94613E-06	0.007149331
1445.479972	190.338915	4.29568E-06	0.006209325
1445.519972	160.225912	3.61608E-06	0.00522711
1445.559972	146.01409	3.29533E-06	0.004763604
1445.599972	153.63183	3.46726E-06	0.005012266
1445.639972	169.15809	3.81766E-06	0.005518965
1445.679972	161.725586	3.64992E-06	0.005276618
1445.719972	147.0927	3.31968E-06	0.004799324
1445.759972	147.146644	3.32089E-06	0.004801217
1445.799972	167.947228	3.79034E-06	0.005480066
1445.839972	155.323618	3.50544E-06	0.005068302
1445.879972	139.983403	3.15923E-06	0.004567868
1445.919972	154.906234	3.49602E-06	0.005054962
1445.959972	181.80737	4.10314E-06	0.005932975
1445.999972	179.550347	4.0522E-06	0.005859483
1446.039972	165.639616	3.73826E-06	0.005405667
1446.079972	170.960385	3.85834E-06	0.005579465
1446.119972	187.703624	4.23621E-06	0.006126067
1446.159972	198.981234	4.49073E-06	0.006494313
1446.199972	198.506581	4.48002E-06	0.006479001
1446.239972	198.943646	4.48988E-06	0.006493445
1446.279972	211.828037	4.78066E-06	0.006914178
1446.319972	235.49603	5.31482E-06	0.007686927
1446.359972	241.673931	5.45424E-06	0.0078888
1446.399972	227.21579	5.12794E-06	0.007417058
1446.439972	196.104416	4.4258E-06	0.006401659
1446.479972	171.08572	3.86117E-06	0.0055851
1446.519972	170.680917	3.85203E-06	0.005572039
1446.559972	164.36082	3.70939E-06	0.005365862
1446.599972	161.291086	3.64012E-06	0.00526579
1446.639972	166.463281	3.75684E-06	0.005434801
1446.679972	178.119255	4.0199E-06	0.005815514
1446.719972	190.974065	4.31002E-06	0.00623539
1446.759972	193.108743	4.3582E-06	0.006305263
1446.799972	190.258167	4.29386E-06	0.006212359
1446.839972	176.337357	3.97969E-06	0.005757972
1446.879972	193.062283	4.35715E-06	0.006304268
1446.919972	211.760919	4.77915E-06	0.006915046
1446.959972	195.465873	4.41139E-06	0.006383108
1446.999972	179.183474	4.04392E-06	0.005851554
1447.039972	175.807529	3.96773E-06	0.005741465
1447.079972	185.981818	4.19735E-06	0.006073902
1447.119972	189.753064	4.28246E-06	0.006197237
1447.159972	205.054329	4.62779E-06	0.006697153
1447.199972	229.666003	5.18324E-06	0.007501187

1447.239972	219.384823	4.95121E-06	0.007165589
1447.279972	190.514256	4.29964E-06	0.006222785
1447.319972	188.327036	4.25028E-06	0.006151514
1447.359972	199.792316	4.50903E-06	0.006526196
1447.399972	203.589442	4.59473E-06	0.006650412
1447.439972	196.861069	4.44288E-06	0.006430802
1447.479972	184.998494	4.17516E-06	0.006043458
1447.519972	174.862489	3.9464E-06	0.005712497
1447.559972	163.26608	3.68469E-06	0.005333807
1447.599972	152.501809	3.44175E-06	0.004982282
1447.639972	155.853502	3.5174E-06	0.005091924
1447.679972	159.952691	3.60991E-06	0.005225994
1447.719972	167.841776	3.78796E-06	0.005483898
1447.759972	173.57555	3.91736E-06	0.005671395
1447.799972	165.811017	3.74212E-06	0.005417847
1447.839972	178.388176	4.02597E-06	0.005828964
1447.879972	181.550001	4.09733E-06	0.005932443
1447.919972	174.271583	3.93307E-06	0.005694766
1447.959972	179.158137	4.04335E-06	0.005854608
1447.999972	172.619369	3.89578E-06	0.005641087
1448.039972	164.512976	3.71283E-06	0.005376324
1448.079972	159.615901	3.60231E-06	0.005216431
1448.119972	149.096142	3.36489E-06	0.004872768
1448.159972	159.777327	3.60595E-06	0.005221995
1448.199972	187.18424	4.22449E-06	0.006117903
1448.239972	226.817014	5.11894E-06	0.007413459
1448.279972	241.619087	5.45301E-06	0.00789748
1448.319972	247.408087	5.58366E-06	0.00808692
1448.359972	238.660972	5.38625E-06	0.007801223
1448.399972	225.134003	5.08096E-06	0.007359264
1448.439972	195.280289	4.4072E-06	0.006383571
1448.479972	182.706327	4.12343E-06	0.005972702
1448.519972	196.626243	4.43758E-06	0.006427924
1448.559972	175.637075	3.96388E-06	0.005741924
1448.599972	149.966349	3.38453E-06	0.004902832
1448.639972	134.931636	3.04522E-06	0.004411426
1448.679972	118.01205	2.66337E-06	0.003858367
1448.719972	120.641803	2.72272E-06	0.003944455
1448.759972	134.651646	3.0389E-06	0.004402637
1448.799972	140.659237	3.17448E-06	0.004599191
1448.839972	149.715566	3.37887E-06	0.004895445
1448.879972	179.588448	4.05306E-06	0.005872399
1448.919972	194.418409	4.38775E-06	0.006357503
1448.959972	184.919987	4.17339E-06	0.00604707
1448.999972	173.498844	3.91563E-06	0.005673744
1449.039972	177.131132	3.9976E-06	0.005792686

1449.079972	208.699649	4.71006E-06	0.006825254
1449.119972	219.969653	4.96441E-06	0.007194024
1449.159972	233.611382	5.27228E-06	0.007640382
1449.199972	240.881127	5.43635E-06	0.00787836
1449.239972	234.468	5.29162E-06	0.007668822
1449.279972	233.172463	5.26238E-06	0.007626659
1449.319972	233.604571	5.27213E-06	0.007641003
1449.359972	243.447562	5.49427E-06	0.007963178
1449.399972	233.140953	5.26167E-06	0.007626259
1449.439972	226.665716	5.11553E-06	0.007414653
1449.479972	217.411408	4.90667E-06	0.007112124
1449.519972	196.304074	4.43031E-06	0.006421822
1449.559972	153.996824	3.47549E-06	0.005037937
1449.599972	127.456801	2.87652E-06	0.004169807
1449.639972	134.21693	3.02909E-06	0.004391089
1449.679972	161.894418	3.65373E-06	0.005296741
1449.719972	195.474901	4.4116E-06	0.006395579
1449.759972	184.120747	4.15535E-06	0.006024258
1449.799972	170.718005	3.85287E-06	0.005585887
1449.839972	173.909459	3.92489E-06	0.005690469
1449.879972	170.742141	3.85341E-06	0.005586985
1449.919972	153.005233	3.45311E-06	0.00500674
1449.959972	156.080498	3.52252E-06	0.005107512
1449.999972	169.912567	3.83469E-06	0.0055603
1450.039972	191.192309	4.31494E-06	0.006256841
1450.079972	207.16658	4.67546E-06	0.006779793
1450.119972	213.954786	4.82866E-06	0.007002138
1450.159972	203.721187	4.5977E-06	0.006667405
1450.199972	196.250168	4.42909E-06	0.00642307
1450.239972	203.526432	4.59331E-06	0.006661399
1450.279972	215.211994	4.85703E-06	0.00704406
1450.319972	227.548575	5.13545E-06	0.007448052
1450.359972	230.411695	5.20007E-06	0.007541975
1450.399972	219.780955	4.96015E-06	0.007194201
1450.439972	217.751736	4.91435E-06	0.007127975
1450.479972	193.254085	4.36148E-06	0.006326233
1450.519972	164.432651	3.71102E-06	0.005382903
1450.559972	153.670622	3.46813E-06	0.005030733
1450.599972	166.99733	3.7689E-06	0.005467162
1450.639972	170.420336	3.84615E-06	0.005579378
1450.679972	164.183066	3.70538E-06	0.005375325
1450.719972	161.926504	3.65446E-06	0.005301592
1450.759972	188.508921	4.25438E-06	0.00617209
1450.799972	196.186214	4.42765E-06	0.006423634
1450.839972	204.496041	4.61519E-06	0.006695903
1450.879972	202.188115	4.5631E-06	0.006620516

1450.919972	190.197239	4.29249E-06	0.006228055
1450.959972	186.939957	4.21897E-06	0.006121563
1450.999972	196.41441	4.4328E-06	0.006431992
1451.039972	186.961359	4.21946E-06	0.006122601
1451.079972	183.56584	4.14283E-06	0.006011571
1451.119972	204.191159	4.60831E-06	0.006687211
1451.159972	225.039381	5.07883E-06	0.007370188
1451.199972	222.047225	5.0113E-06	0.007272393
1451.239972	200.654056	4.52848E-06	0.006571915
1451.279972	212.519897	4.79628E-06	0.006960742
1451.319972	229.297398	5.17492E-06	0.007510469
1451.359972	241.304704	5.44591E-06	0.007903977
1451.399972	235.685175	5.31909E-06	0.007720121
1451.439972	223.610056	5.04657E-06	0.00732479
1451.479972	219.595995	4.95598E-06	0.0071935
1451.519972	195.96025	4.42255E-06	0.006419419
1451.559972	174.569176	3.93978E-06	0.005718832
1451.599972	186.289805	4.2043E-06	0.006102964
1451.639972	196.221935	4.42846E-06	0.006428523
1451.679972	187.45862	4.23068E-06	0.006141593
1451.719972	175.42284	3.95905E-06	0.005747431
1451.759972	168.590791	3.80486E-06	0.005523742
1451.799972	173.893454	3.92453E-06	0.005697637
1451.839972	175.773232	3.96696E-06	0.005759387
1451.879972	167.925874	3.78985E-06	0.005502412
1451.919972	164.244018	3.70676E-06	0.005381917
1451.959972	174.492738	3.93806E-06	0.005717903
1451.999972	168.02312	3.79205E-06	0.005506053
1452.039972	166.287115	3.75287E-06	0.005449315
1452.079972	191.792732	4.32849E-06	0.006285321
1452.119972	218.193789	4.92433E-06	0.007150718
1452.159972	223.348039	5.04065E-06	0.007319836
1452.199972	213.458131	4.81745E-06	0.006995905
1452.239972	219.226093	4.94763E-06	0.007185143
1452.279972	240.955549	5.43803E-06	0.007897544
1452.319972	241.497459	5.45026E-06	0.007915523
1452.359972	229.418065	5.17765E-06	0.007519806
1452.399972	217.122566	4.90015E-06	0.007116983
1452.439972	211.788732	4.77978E-06	0.006942339
1452.479972	199.784751	4.50886E-06	0.006549034
1452.519972	192.995802	4.35565E-06	0.006326663
1452.559972	167.285963	3.77541E-06	0.005484011
1452.599972	144.618792	3.26384E-06	0.004741061
1452.639972	137.174939	3.09585E-06	0.004497152
1452.679972	151.223478	3.4129E-06	0.004957856
1452.719972	185.861514	4.19464E-06	0.006093631

1452.759972	188.134285	4.24593E-06	0.006168315
1452.799972	179.704471	4.05568E-06	0.005892091
1452.839972	163.659628	3.69357E-06	0.005366166
1452.879972	154.024906	3.47613E-06	0.005050396
1452.919972	161.009477	3.63376E-06	0.005279562
1452.959972	194.656779	4.39313E-06	0.006383045
1452.999972	219.726356	4.95892E-06	0.007205307
1453.039972	209.982546	4.73901E-06	0.006885976
1453.079972	175.660419	3.96441E-06	0.005760606
1453.119972	168.370522	3.79989E-06	0.005521693
1453.159972	212.889969	4.80463E-06	0.006981896
1453.199972	248.105977	5.59941E-06	0.008137057
1453.239972	244.321315	5.51399E-06	0.008013153
1453.279972	246.422083	5.5614E-06	0.008082276
1453.319972	230.235807	5.1961E-06	0.007551598
1453.359972	213.938195	4.82829E-06	0.007017239
1453.399972	216.662979	4.88978E-06	0.007106809
1453.439972	222.252523	5.01593E-06	0.007290353
1453.479972	229.649747	5.18287E-06	0.007533205
1453.519972	213.501591	4.81843E-06	0.007003689
1453.559972	185.042689	4.17616E-06	0.006070293
1453.599972	157.535121	3.55535E-06	0.005168054
1453.639972	160.258131	3.6168E-06	0.005257529
1453.679972	158.797601	3.58384E-06	0.005209757
1453.719972	158.290319	3.57239E-06	0.005193258
1453.759972	162.634636	3.67044E-06	0.005335935
1453.799972	178.976128	4.03924E-06	0.00587225
1453.839972	173.745654	3.9212E-06	0.005700794
1453.879972	174.997887	3.94946E-06	0.005742039
1453.919972	176.197421	3.97653E-06	0.005781557
1453.959972	161.317024	3.6407E-06	0.005293433
1453.999972	169.443137	3.8241E-06	0.005560235
1454.039972	179.367917	4.04808E-06	0.005886076
1454.079972	189.635248	4.2798E-06	0.006223176
1454.119972	196.487064	4.43444E-06	0.006448207
1454.159972	183.515358	4.14169E-06	0.006022674
1454.199972	162.08538	3.65804E-06	0.005319523
1454.239972	169.574487	3.82706E-06	0.005565464
1454.279972	183.408193	4.13927E-06	0.006019654
1454.319972	191.774875	4.32809E-06	0.00629443
1454.359972	199.050415	4.49229E-06	0.006533408
1454.399972	189.072911	4.26711E-06	0.006206088
1454.439972	193.757165	4.37283E-06	0.006360018
1454.479972	177.613353	4.00849E-06	0.005830263
1454.519972	155.915157	3.51879E-06	0.005118147
1454.559972	153.947048	3.47437E-06	0.00505368

1454.599972	169.196084	3.81852E-06	0.005554419
1454.639972	176.61526	3.98596E-06	0.005798137
1454.679972	178.109429	4.01968E-06	0.005847351
1454.719972	162.499958	3.6674E-06	0.005335037
1454.759972	156.144391	3.52396E-06	0.005126518
1454.799972	179.591242	4.05312E-06	0.005896485
1454.839972	193.467178	4.36628E-06	0.006352246
1454.879972	173.754339	3.92139E-06	0.005705157
1454.919972	166.847095	3.76551E-06	0.005478511
1454.959972	155.699646	3.51392E-06	0.005112619
1454.999972	163.062969	3.6801E-06	0.005354551
1455.039972	172.486002	3.89277E-06	0.005664134
1455.079972	173.392167	3.91322E-06	0.005694048
1455.119972	185.206613	4.17986E-06	0.006082191
1455.159972	196.934482	4.44454E-06	0.006467512
1455.199972	199.110756	4.49365E-06	0.006539163
1455.239972	212.344027	4.79231E-06	0.006973959
1455.279972	241.926386	5.45994E-06	0.007945744
1455.319972	273.929777	6.18221E-06	0.008997099
1455.359972	264.485159	5.96906E-06	0.008687133
1455.399972	237.13668	5.35184E-06	0.007789074
1455.439972	218.109739	4.92243E-06	0.007164306
1455.479972	196.591983	4.43681E-06	0.006457684
1455.519972	164.517129	3.71292E-06	0.005404233
1455.559972	141.638091	3.19657E-06	0.004652806
1455.599972	146.09361	3.29713E-06	0.004799301
1455.639972	172.814721	3.90019E-06	0.005677269
1455.679972	165.30595	3.73073E-06	0.005430742
1455.719972	169.23063	3.8193E-06	0.005559831
1455.759972	201.338111	4.54392E-06	0.006614858
1455.799972	195.524259	4.41271E-06	0.006424023
1455.839972	156.374979	3.52917E-06	0.0051379
1455.879972	151.983789	3.43006E-06	0.004993759
1455.919972	171.801172	3.87731E-06	0.005645058
1455.959972	168.827161	3.81019E-06	0.00554749
1455.999972	167.944631	3.79028E-06	0.005518642
1456.039972	165.320209	3.73105E-06	0.005432553
1456.079972	166.9977	3.76891E-06	0.005487828
1456.119972	184.259257	4.15847E-06	0.006055238
1456.159972	204.932633	4.62504E-06	0.006734804
1456.199972	204.850308	4.62319E-06	0.006732283
1456.239972	207.957328	4.69331E-06	0.006834582
1456.279972	207.640853	4.68616E-06	0.006824368
1456.319972	222.319382	5.01744E-06	0.007306996
1456.359972	234.117869	5.28371E-06	0.00769499
1456.399972	233.96239	5.28021E-06	0.007690091

1456.439972	233.54484	5.27078E-06	0.007676577
1456.479972	197.00158	4.44605E-06	0.006475585
1456.519972	186.551544	4.21021E-06	0.006132253
1456.559972	178.434599	4.02702E-06	0.005865597
1456.599972	171.796646	3.87721E-06	0.005647545
1456.639972	163.500539	3.68998E-06	0.005374972
1456.679972	171.738055	3.87589E-06	0.005645929
1456.719972	185.487833	4.1862E-06	0.006098124
1456.759972	195.320783	4.40812E-06	0.00642157
1456.799972	200.64957	4.52838E-06	0.006596946
1456.839972	192.969585	4.35505E-06	0.006344618
1456.879972	183.017445	4.13045E-06	0.006017568
1456.919972	180.12702	4.06522E-06	0.005922694
1456.959972	183.626481	4.14419E-06	0.006037925
1456.999972	188.563506	4.25562E-06	0.006200432
1457.039972	182.968255	4.12934E-06	0.006016611
1457.079972	186.500827	4.20906E-06	0.006132943
1457.119972	178.317107	4.02437E-06	0.005863988
1457.159972	165.452755	3.73404E-06	0.005441091
1457.199972	189.784406	4.28317E-06	0.006241435
1457.239972	215.214003	4.85708E-06	0.007077932
1457.279972	214.98966	4.85202E-06	0.007070747
1457.319972	214.696043	4.84539E-06	0.007061285
1457.359972	213.707027	4.82307E-06	0.007028949
1457.399972	215.060966	4.85363E-06	0.007073675
1457.439972	225.82586	5.09658E-06	0.007427952
1457.479972	212.118238	4.78721E-06	0.006977267
1457.519972	192.452477	4.34338E-06	0.006330569
1457.559972	166.035784	3.7472E-06	0.005461763
1457.599972	178.991823	4.0396E-06	0.005888115
1457.639972	195.286907	4.40735E-06	0.006424335
1457.679972	189.844095	4.28452E-06	0.006245454
1457.719972	185.384804	4.18388E-06	0.006098921
1457.759972	185.782177	4.19284E-06	0.006112162
1457.799972	169.409252	3.82333E-06	0.005573652
1457.839972	156.863032	3.54018E-06	0.005161016
1457.879972	161.493015	3.64467E-06	0.005313495
1457.919972	175.546729	3.96185E-06	0.005776053
1457.959972	198.79098	4.48644E-06	0.006541043
1457.999972	194.944081	4.39962E-06	0.006414641
1458.039972	168.961251	3.81322E-06	0.005559827
1458.079972	152.069305	3.43199E-06	0.005004119
1458.119972	175.373885	3.95794E-06	0.005771158
1458.159972	211.117376	4.76463E-06	0.006947586
1458.199972	225.786101	5.09568E-06	0.007430517
1458.239972	209.029588	4.71751E-06	0.006879257

1458.279972	209.867955	4.73643E-06	0.006907037
1458.319972	241.930856	5.46004E-06	0.007962489
1458.359972	228.416638	5.15505E-06	0.007517912
1458.399972	213.687926	4.82264E-06	0.007033336
1458.439972	213.262084	4.81303E-06	0.007019513
1458.479972	193.18369	4.35989E-06	0.006358808
1458.519972	171.420816	3.86873E-06	0.005642619
1458.559972	157.310283	3.55027E-06	0.005178288
1458.599972	167.095558	3.77111E-06	0.005500547
1458.639972	183.202174	4.13462E-06	0.006030919
1458.679972	179.418645	4.04923E-06	0.005906529
1458.719972	180.88068	4.08223E-06	0.005954823
1458.759972	185.819929	4.1937E-06	0.006117597
1458.799972	205.460548	4.63696E-06	0.006764395
1458.839972	244.015356	5.50709E-06	0.008033958
1458.879972	255.289879	5.76154E-06	0.008405391
1458.919972	246.042322	5.55283E-06	0.008101138
1458.959972	228.669372	5.16075E-06	0.007529327
1458.999972	207.183413	4.67584E-06	0.006822052
1459.039972	192.050847	4.33432E-06	0.006323946
1459.079972	195.276739	4.40712E-06	0.006430346
1459.119972	192.10329	4.3355E-06	0.00632602
1459.159972	228.975855	5.16767E-06	0.007540452
1459.199972	279.789196	6.31445E-06	0.009214049
1459.239972	272.175158	6.14261E-06	0.008963548
1459.279972	254.025414	5.733E-06	0.008366052
1459.319972	257.660711	5.81504E-06	0.008486009
1459.359972	258.137335	5.8258E-06	0.008501939
1459.399972	258.538901	5.83486E-06	0.008515399
1459.439972	253.541271	5.72207E-06	0.008351023
1459.479972	233.910682	5.27904E-06	0.007704651
1459.519972	221.808292	5.0059E-06	0.007306217
1459.559972	209.066718	4.71834E-06	0.006886707
1459.599972	182.229705	4.11267E-06	0.006002854
1459.639972	164.149424	3.70462E-06	0.005407417
1459.679972	148.295886	3.34683E-06	0.004885303
1459.719972	146.493461	3.30615E-06	0.004826058
1459.759972	166.207281	3.75107E-06	0.005475657
1459.799972	181.668782	4.10001E-06	0.005985196
1459.839972	194.853398	4.39757E-06	0.006419748
1459.879972	199.122926	4.49393E-06	0.006560594
1459.919972	202.977285	4.58091E-06	0.006687769
1459.959972	196.270146	4.42954E-06	0.006466957
1459.999972	198.946924	4.48995E-06	0.006555334
1460.039972	196.024266	4.42399E-06	0.006459209
1460.079972	193.112242	4.35827E-06	0.006363429

1460.119972	197.969846	4.4679E-06	0.006523675
1460.159972	221.962648	5.00939E-06	0.007314508
1460.199972	242.169205	5.46542E-06	0.007980609
1460.239972	263.470756	5.94617E-06	0.008682832
1460.279972	280.968391	6.34107E-06	0.009259731
1460.319972	259.317695	5.85244E-06	0.008546434
1460.359972	257.065469	5.80161E-06	0.008472438
1460.399972	270.758711	6.11065E-06	0.008923989
1460.439972	243.298294	5.4909E-06	0.008019135
1460.479972	205.781161	4.64419E-06	0.006782753
1460.519972	187.03547	4.22113E-06	0.006165045
1460.559972	178.590025	4.03053E-06	0.005886828
1460.599972	156.148091	3.52404E-06	0.00514722
1460.639972	142.069647	3.20631E-06	0.00468327
1460.679972	175.652959	3.96424E-06	0.00579049
1460.719972	209.212053	4.72162E-06	0.006896971
1460.759972	234.768486	5.2984E-06	0.007739687
1460.799972	239.875969	5.41367E-06	0.007908284
1460.839972	231.423127	5.2229E-06	0.007629818
1460.879972	220.90126	4.98543E-06	0.00728312
1460.919972	204.570588	4.61687E-06	0.006744882
1460.959972	175.945966	3.97086E-06	0.005801261
1460.999972	176.940836	3.99331E-06	0.005834223
1461.039972	184.294224	4.15926E-06	0.006076851
1461.079972	195.068499	4.40242E-06	0.006432294
1461.119972	212.905353	4.80498E-06	0.007020648
1461.159972	222.851484	5.02945E-06	0.007348827
1461.199972	211.085212	4.7639E-06	0.006961009
1461.239972	210.776276	4.75693E-06	0.006951012
1461.279972	225.3925	5.08679E-06	0.007433231
1461.319972	255.561279	5.76766E-06	0.0084284
1461.359972	272.809978	6.15694E-06	0.008997507
1461.399972	245.98002	5.55143E-06	0.008112854
1461.439972	199.322909	4.49844E-06	0.006574201
1461.479972	159.351751	3.59635E-06	0.005255989
1461.519972	147.670706	3.33272E-06	0.00487084
1461.559972	151.392199	3.41671E-06	0.004993728
1461.599972	162.472133	3.66677E-06	0.005359351
1461.639972	159.974198	3.61039E-06	0.005277097
1461.679972	153.931325	3.47402E-06	0.005077899
1461.719972	146.251739	3.3007E-06	0.004824696
1461.759972	164.645105	3.71581E-06	0.005431623
1461.799972	178.451113	4.02739E-06	0.005887243
1461.839972	165.309985	3.73082E-06	0.005453856
1461.879972	153.479649	3.46382E-06	0.005063692
1461.919972	154.566449	3.48835E-06	0.005099688

1461.959972	146.255555	3.30078E-06	0.004825614
1461.999972	144.693731	3.26554E-06	0.004774213
1462.039972	162.400165	3.66515E-06	0.005358589
1462.079972	177.790071	4.01247E-06	0.005866558
1462.119972	170.203777	3.84126E-06	0.005616386
1462.159972	173.942982	3.92565E-06	0.005739929
1462.199972	174.404368	3.93606E-06	0.005755312
1462.239972	176.209629	3.97681E-06	0.005815045
1462.279972	178.652195	4.03193E-06	0.005895812
1462.319972	202.284481	4.56528E-06	0.006675899
1462.359972	220.165868	4.96884E-06	0.007266228
1462.399972	223.459254	5.04316E-06	0.007375123
1462.439972	226.977333	5.12256E-06	0.00749144
1462.479972	216.029589	4.87549E-06	0.007130302
1462.519972	191.899632	4.33091E-06	0.006334039
1462.559972	178.445559	4.02727E-06	0.005890121
1462.599972	163.101213	3.68097E-06	0.005383783
1462.639972	158.492813	3.57696E-06	0.005231808
1462.679972	163.094988	3.68083E-06	0.005383872
1462.719972	152.654392	3.4452E-06	0.005039358
1462.759972	146.093504	3.29713E-06	0.004822905
1462.799972	170.129576	3.83959E-06	0.005616549
1462.839972	194.426822	4.38794E-06	0.006418858
1462.879972	186.220632	4.20274E-06	0.006148105
1462.919972	173.675813	3.91962E-06	0.005734092
1462.959972	166.663856	3.76137E-06	0.005502735
1462.999972	170.128721	3.83957E-06	0.005617288
1463.039972	190.418077	4.29747E-06	0.006287372
1463.079972	210.091823	4.74148E-06	0.006937164
1463.119972	216.305157	4.88171E-06	0.007142522
1463.159972	206.089567	4.65115E-06	0.006805383
1463.199972	212.195382	4.78895E-06	0.007007198
1463.239972	228.596863	5.15911E-06	0.00754902
1463.279972	254.001352	5.73246E-06	0.008388189
1463.319972	254.606114	5.74611E-06	0.008408391
1463.359972	236.455195	5.33646E-06	0.007809168
1463.399972	231.085211	5.21527E-06	0.007632028
1463.439972	216.639992	4.88926E-06	0.007155143
1463.479972	207.447076	4.68179E-06	0.006851708
1463.519972	205.84014	4.64553E-06	0.006798819
1463.559972	189.403357	4.27457E-06	0.006256089
1463.599972	172.250442	3.88745E-06	0.005689675
1463.639972	167.730774	3.78545E-06	0.005540536
1463.679972	184.071413	4.15424E-06	0.006080471
1463.719972	193.701581	4.37157E-06	0.006398761
1463.759972	188.507701	4.25436E-06	0.006227356

1463.799972	175.760887	3.96668E-06	0.005806424
1463.839972	166.178994	3.75043E-06	0.005490027
1463.879972	156.323016	3.52799E-06	0.005164558
1463.919972	155.474751	3.50885E-06	0.005136674
1463.959972	159.46275	3.59885E-06	0.005268575
1463.999972	162.090524	3.65816E-06	0.005355542
1464.039972	166.568392	3.75922E-06	0.005503643
1464.079972	162.951116	3.67758E-06	0.005384271
1464.119972	167.366939	3.77724E-06	0.005530331
1464.159972	197.405036	4.45516E-06	0.006523062
1464.199972	211.403427	4.77108E-06	0.006985817
1464.239972	207.862078	4.69116E-06	0.00686898
1464.279972	207.333988	4.67924E-06	0.006851716
1464.319972	219.612673	4.95635E-06	0.007257685
1464.359972	219.921885	4.96333E-06	0.007268103
1464.399972	207.910774	4.69226E-06	0.00687134
1464.439972	189.430191	4.27518E-06	0.006260738
1464.479972	174.271549	3.93307E-06	0.005759897
1464.519972	156.753027	3.5377E-06	0.005181029
1464.559972	155.707876	3.51411E-06	0.005146625
1464.599972	173.692981	3.92001E-06	0.005741245
1464.639972	178.44471	4.02725E-06	0.005898469
1464.679972	192.686261	4.34866E-06	0.006369396
1464.719972	202.658649	4.57372E-06	0.006699224
1464.759972	201.571382	4.54919E-06	0.006663465
1464.799972	179.24284	4.04526E-06	0.005925499
1464.839972	173.633527	3.91867E-06	0.00574022
1464.879972	182.527124	4.11938E-06	0.006034402
1464.919972	173.503197	3.91573E-06	0.005736224
1464.959972	171.22086	3.86422E-06	0.005660922
1464.999972	191.146663	4.31391E-06	0.006319884
1465.039972	215.859928	4.87166E-06	0.007137174
1465.079972	206.60468	4.66278E-06	0.006831345
1465.119972	188.361374	4.25105E-06	0.006228304
1465.159972	190.741293	4.30477E-06	0.00630717
1465.199972	226.455718	5.11079E-06	0.00748833
1465.239972	233.816665	5.27692E-06	0.007731949
1465.279972	229.736892	5.18484E-06	0.007597245
1465.319972	227.133018	5.12608E-06	0.007511341
1465.359972	224.280261	5.06169E-06	0.007417202
1465.399972	222.234578	5.01552E-06	0.00734975
1465.439972	204.404184	4.61312E-06	0.006760247
1465.479972	199.856255	4.51048E-06	0.006610014
1465.519972	184.167699	4.15641E-06	0.0060913
1465.559972	170.249535	3.84229E-06	0.005631114
1465.599972	186.818548	4.21623E-06	0.006179313

1465.639972	182.679828	4.12283E-06	0.006042583
1465.679972	152.36418	3.43865E-06	0.005039957
1465.719972	147.844059	3.33663E-06	0.004890572
1465.759972	169.616294	3.828E-06	0.005610934
1465.799972	178.941511	4.03846E-06	0.005919576
1465.839972	166.736569	3.76301E-06	0.005515974
1465.879972	154.465908	3.48608E-06	0.005110175
1465.919972	143.645127	3.24187E-06	0.004752323
1465.959972	153.248916	3.45861E-06	0.005070191
1465.999972	181.891521	4.10504E-06	0.006017986
1466.039972	191.547995	4.32297E-06	0.006337649
1466.079972	187.69777	4.23608E-06	0.006210428
1466.119972	196.988736	4.44576E-06	0.00651802
1466.159972	190.401426	4.29709E-06	0.006300229
1466.199972	196.549607	4.43585E-06	0.006503844
1466.239972	218.348476	4.92782E-06	0.007225368
1466.279972	221.684201	5.0031E-06	0.007335951
1466.319972	201.668712	4.55138E-06	0.006673782
1466.359972	199.876399	4.51093E-06	0.00661465
1466.399972	197.132707	4.44901E-06	0.006524029
1466.439972	178.296285	4.0239E-06	0.005900806
1466.479972	161.431398	3.64328E-06	0.0053428
1466.519972	170.365138	3.8449E-06	0.005638628
1466.559972	182.326894	4.11486E-06	0.006034695
1466.599972	198.030394	4.46927E-06	0.006554631
1466.639972	185.756103	4.19226E-06	0.006148531
1466.679972	163.832983	3.69748E-06	0.005423023
1466.719972	165.543124	3.73608E-06	0.00547978
1466.759972	186.218596	4.20269E-06	0.006164344
1466.799972	197.468076	4.45658E-06	0.006536911
1466.839972	204.476701	4.61475E-06	0.006769106
1466.879972	199.298554	4.49789E-06	0.006597866
1466.919972	190.904922	4.30846E-06	0.006320163
1466.959972	174.569807	3.9398E-06	0.005779525
1466.999972	178.786209	4.03496E-06	0.00591928
1467.039972	203.55457	4.59394E-06	0.006739498
1467.079972	213.113548	4.80968E-06	0.007056179
1467.119972	214.30251	4.83651E-06	0.007095739
1467.159972	217.906854	4.91785E-06	0.007215279
1467.199972	240.790954	5.43432E-06	0.007973229
1467.239972	255.890449	5.77509E-06	0.008473444
1467.279972	290.560171	6.55754E-06	0.009621745
1467.319972	286.259757	6.46048E-06	0.009479597
1467.359972	239.506019	5.40532E-06	0.007931546
1467.399972	216.896773	4.89506E-06	0.007183008
1467.439972	211.417956	4.77141E-06	0.007001756

1467.479972	215.70626	4.86819E-06	0.007143971
1467.519972	193.645303	4.3703E-06	0.00641351
1467.559972	163.219837	3.68364E-06	0.005405969
1467.599972	166.065381	3.74786E-06	0.005500366
1467.639972	170.417803	3.84609E-06	0.005644679
1467.679972	182.365956	4.11575E-06	0.006040597
1467.719972	189.245689	4.27101E-06	0.006268649
1467.759972	180.929196	4.08332E-06	0.005993334
1467.799972	196.422208	4.43298E-06	0.006506722
1467.839972	199.67263	4.50633E-06	0.006614576
1467.879972	188.493526	4.25404E-06	0.006244415
1467.919972	168.052215	3.7927E-06	0.005567387
1467.959972	151.056671	3.40914E-06	0.005004479
1467.999972	136.682332	3.08473E-06	0.004528383
1468.039972	140.185352	3.16379E-06	0.004644567
1468.079972	152.002841	3.43049E-06	0.005036237
1468.119972	154.108933	3.47802E-06	0.005106156
1468.159972	179.583046	4.05294E-06	0.005950363
1468.199972	205.152662	4.63001E-06	0.00679778
1468.239972	223.558356	5.0454E-06	0.007407859
1468.279972	241.702665	5.45489E-06	0.008009309
1468.319972	276.255652	6.23471E-06	0.009154542
1468.359972	281.043537	6.34276E-06	0.009313457
1468.399972	270.118783	6.0962E-06	0.008951667
1468.439972	250.35188	5.65009E-06	0.008296823
1468.479972	225.858454	5.09731E-06	0.007485299
1468.519972	194.924197	4.39917E-06	0.006460266
1468.559972	173.094706	3.90651E-06	0.005736939
1468.599972	165.455044	3.73409E-06	0.005483884
1468.639972	182.100017	4.10974E-06	0.006035734
1468.679972	210.850835	4.75861E-06	0.006988875
1468.719972	220.979487	4.9872E-06	0.007324799
1468.759972	214.383407	4.83833E-06	0.007106353
1468.799972	226.920177	5.12127E-06	0.007522125
1468.839972	243.857973	5.50353E-06	0.008083812
1468.879972	262.973916	5.93495E-06	0.008717737
1468.919972	258.306083	5.82961E-06	0.008563228
1468.959972	244.147306	5.51006E-06	0.008094064
1468.999972	208.215262	4.69913E-06	0.00690302
1469.039972	179.00729	4.03995E-06	0.005934841
1469.079972	184.3772	4.16114E-06	0.006113042
1469.119972	210.576215	4.75241E-06	0.006981863
1469.159972	224.360785	5.06351E-06	0.007439107
1469.199972	220.275228	4.97131E-06	0.007303841
1469.239972	219.738791	4.9592E-06	0.007286253
1469.279972	250.407495	5.65135E-06	0.008303413

1469.319972	285.475482	6.44278E-06	0.009466511
1469.359972	331.322341	7.47748E-06	0.010987115
1469.399972	348.475561	7.86461E-06	0.011556255
1469.439972	293.865461	6.63213E-06	0.009745523
1469.479972	239.325405	5.40124E-06	0.007937016
1469.519972	189.07922	4.26725E-06	0.006270816
1469.559972	165.842372	3.74283E-06	0.005500315
1469.599972	161.463493	3.64401E-06	0.005355231
1469.639972	161.840638	3.65252E-06	0.005367886
1469.679972	164.635013	3.71558E-06	0.005460718
1469.719972	167.697841	3.78471E-06	0.005562459
1469.759972	166.965811	3.76819E-06	0.005538329
1469.799972	171.154807	3.86273E-06	0.005677434
1469.839972	187.214794	4.22518E-06	0.006210334
1469.879972	230.769793	5.20815E-06	0.007655359
1469.919972	247.378678	5.58299E-06	0.008206552
1469.959972	217.666821	4.91244E-06	0.007221086
1469.999972	202.967128	4.58069E-06	0.006733607
1470.039972	177.981416	4.01679E-06	0.005904846
1470.079972	155.98251	3.52031E-06	0.005175134
1470.119972	157.650977	3.55796E-06	0.005230632
1470.159972	166.292153	3.75298E-06	0.005517484
1470.199972	195.152316	4.40432E-06	0.006475225
1470.239972	223.228754	5.03796E-06	0.007407013
1470.279972	239.501051	5.40521E-06	0.007947165
1470.319972	234.011009	5.2813E-06	0.007765205
1470.359972	241.629564	5.45324E-06	0.00801823
1470.399972	251.542968	5.67697E-06	0.008347423
1470.439972	225.966613	5.09975E-06	0.007498879
1470.479972	194.541331	4.39053E-06	0.006456182
1470.519972	179.777262	4.05732E-06	0.005966374
1470.559972	185.35001	4.18309E-06	0.006151487
1470.599972	176.591826	3.98543E-06	0.005860976
1470.639972	180.485778	4.07331E-06	0.005990376
1470.679972	188.614634	4.25677E-06	0.006260346
1470.719972	194.432938	4.38808E-06	0.006453638
1470.759972	195.579672	4.41396E-06	0.006491877
1470.799972	210.336331	4.747E-06	0.006981885
1470.839972	234.881515	5.30095E-06	0.007796847
1470.879972	228.837836	5.16455E-06	0.007596435
1470.919972	205.711651	4.64263E-06	0.00682893
1470.959972	180.880876	4.08223E-06	0.006004796
1470.999972	157.181961	3.54738E-06	0.005218193
1471.039972	156.198418	3.52518E-06	0.005185682
1471.079972	165.293319	3.73044E-06	0.005487776
1471.119972	178.668439	4.0323E-06	0.005931994

1471.159972	189.758921	4.28259E-06	0.006300382
1471.199972	220.251121	4.97076E-06	0.007312983
1471.239972	236.062758	5.32761E-06	0.007838189
1471.279972	237.91211	5.36934E-06	0.00789981
1471.319972	240.291916	5.42305E-06	0.007979047
1471.359972	219.440784	4.95247E-06	0.00728687
1471.399972	186.385932	4.20647E-06	0.006189401
1471.439972	171.780737	3.87685E-06	0.005704555
1471.479972	168.703664	3.80741E-06	0.005602523
1471.519972	186.588358	4.21104E-06	0.006196628
1471.559972	190.224575	4.2931E-06	0.00631756
1471.599972	173.92811	3.92532E-06	0.005776494
1471.639972	189.037189	4.26631E-06	0.006278467
1471.679972	189.109068	4.26793E-06	0.006281025
1471.719972	180.118883	4.06503E-06	0.005982589
1471.759972	178.643379	4.03173E-06	0.005933742
1471.799972	172.816674	3.90023E-06	0.005740361
1471.839972	175.43682	3.95936E-06	0.005827551
1471.879972	185.904683	4.19561E-06	0.006175434
1471.919972	189.497883	4.2767E-06	0.006294965
1471.959972	175.257845	3.95533E-06	0.005822081
1471.999972	162.549971	3.66853E-06	0.005400071
1472.039972	160.841918	3.62998E-06	0.005343473
1472.079972	173.933502	3.92544E-06	0.005778557
1472.119972	181.464913	4.09541E-06	0.006028935
1472.159972	196.128235	4.42634E-06	0.006516282
1472.199972	214.4278	4.83934E-06	0.007124471
1472.239972	214.00926	4.82989E-06	0.007110758
1472.279972	202.367	4.56714E-06	0.006724111
1472.319972	191.077675	4.31236E-06	0.006349169
1472.359972	186.961506	4.21946E-06	0.006212565
1472.399972	195.577663	4.41392E-06	0.006499049
1472.439972	210.642363	4.7539E-06	0.006999839
1472.479972	214.190738	4.83399E-06	0.007117948
1472.519972	198.945353	4.48992E-06	0.006611496
1472.559972	181.617021	4.09884E-06	0.006035792
1472.599972	159.962739	3.61014E-06	0.005316286
1472.639972	145.786344	3.29019E-06	0.004845272
1472.679972	152.161203	3.43407E-06	0.005057281
1472.719972	176.217051	3.97697E-06	0.005856968
1472.759972	188.33328	4.25042E-06	0.006259848
1472.799972	192.341194	4.34087E-06	0.006393237
1472.839972	181.860934	4.10435E-06	0.006045048
1472.879972	172.217448	3.88671E-06	0.005724654
1472.919972	177.890626	4.01474E-06	0.005913396
1472.959972	185.056585	4.17647E-06	0.006151772

1472.999972	200.101356	4.51601E-06	0.006652081
1473.039972	222.975472	5.03225E-06	0.007412699
1473.079972	217.399188	4.9064E-06	0.007227515
1473.119972	215.216265	4.85713E-06	0.007155137
1473.159972	219.609917	4.95629E-06	0.007301408
1473.199972	223.161559	5.03645E-06	0.007419691
1473.239972	229.14266	5.17143E-06	0.007618758
1473.279972	238.700281	5.38713E-06	0.007936755
1473.319972	239.857706	5.41325E-06	0.007975456
1473.359972	249.979441	5.64169E-06	0.008312237
1473.399972	262.426559	5.9226E-06	0.008726361
1473.439972	242.111201	5.46411E-06	0.008051042
1473.479972	209.340481	4.72452E-06	0.00696149
1473.519972	192.677561	4.34846E-06	0.006407549
1473.559972	187.714092	4.23645E-06	0.006242657
1473.599972	179.266278	4.04579E-06	0.005961876
1473.639972	173.267477	3.91041E-06	0.00576253
1473.679972	165.116683	3.72645E-06	0.0054916
1473.719972	156.086866	3.52266E-06	0.005191419
1473.759972	168.472995	3.8022E-06	0.005603531
1473.799972	179.914365	4.06042E-06	0.005984242
1473.839972	171.438648	3.86913E-06	0.005702481
1473.879972	160.555628	3.62352E-06	0.005340629
1473.919972	144.704797	3.26579E-06	0.004813507
1473.959972	140.82433	3.17821E-06	0.004684553
1473.999972	147.698412	3.33335E-06	0.004913354
1474.039972	151.426042	3.41747E-06	0.005037495
1474.079972	182.746176	4.12433E-06	0.006079587
1474.119972	189.986829	4.28774E-06	0.00632064
1474.159972	188.106445	4.2453E-06	0.006258252
1474.199972	192.429137	4.34286E-06	0.006402241
1474.239972	212.421353	4.79405E-06	0.007067586
1474.279972	202.550927	4.57129E-06	0.006739365
1474.319972	176.912179	3.99266E-06	0.005886461
1474.359972	167.671392	3.78411E-06	0.00557914
1474.399972	159.411747	3.5977E-06	0.00530445
1474.439972	163.328876	3.68611E-06	0.005434941
1474.479972	172.03721	3.88264E-06	0.005724875
1474.519972	160.82943	3.6297E-06	0.00535206
1474.559972	166.544652	3.75868E-06	0.0055424
1474.599972	181.51823	4.09661E-06	0.006040866
1474.639972	193.904697	4.37616E-06	0.006453259
1474.679972	212.220403	4.78952E-06	0.007063008
1474.719972	204.036408	4.60482E-06	0.006790816
1474.759972	195.314169	4.40797E-06	0.006500696
1474.799972	180.643275	4.07687E-06	0.006012563

1474.839972	156.001305	3.52073E-06	0.005192517
1474.879972	155.86039	3.51755E-06	0.005187967
1474.919972	172.722316	3.8981E-06	0.005749389
1474.959972	195.478166	4.41167E-06	0.006507037
1474.999972	216.81374	4.89318E-06	0.007217446
1475.039972	221.613826	5.00152E-06	0.007377435
1475.079971	224.600539	5.06892E-06	0.007477064
1475.119971	210.330622	4.74687E-06	0.007002202
1475.159971	199.316343	4.49829E-06	0.006635701
1475.199971	207.090594	4.67375E-06	0.00689471
1475.239971	209.228983	4.72201E-06	0.006966093
1475.279971	235.641979	5.31811E-06	0.007845703
1475.319971	279.885362	6.31662E-06	0.00931904
1475.359971	257.635321	5.81447E-06	0.008578437
1475.399971	233.781291	5.27612E-06	0.007784385
1475.439971	205.083879	4.62846E-06	0.006829011
1475.479971	182.025795	4.10807E-06	0.006061373
1475.519971	181.687432	4.10043E-06	0.00605027
1475.559971	175.807734	3.96774E-06	0.005854632
1475.599971	171.501248	3.87054E-06	0.005711375
1475.639971	169.726921	3.8305E-06	0.005652439
1475.679971	182.435841	4.11732E-06	0.006075851
1475.719971	175.986058	3.97176E-06	0.005861206
1475.759971	190.602726	4.30164E-06	0.006348185
1475.799971	226.936508	5.12164E-06	0.007558517
1475.839971	211.83216	4.78076E-06	0.007055632
1475.879971	177.991164	4.01701E-06	0.005928629
1475.919971	161.322723	3.64083E-06	0.005373572
1475.959971	159.128926	3.59132E-06	0.005300642
1475.999971	164.23782	3.70662E-06	0.005470969
1476.039971	173.460269	3.91476E-06	0.005778337
1476.079971	184.899698	4.17293E-06	0.006159576
1476.119971	192.945836	4.35452E-06	0.006427792
1476.159971	198.327473	4.47597E-06	0.006607255
1476.199971	219.066344	4.94402E-06	0.007298366
1476.239971	220.873664	4.98481E-06	0.007358777
1476.279971	216.044438	4.87582E-06	0.007198078
1476.319971	222.76571	5.02751E-06	0.007422216
1476.359971	226.599715	5.11404E-06	0.007550164
1476.399971	231.709265	5.22936E-06	0.00772062
1476.439971	232.980431	5.25804E-06	0.007763186
1476.479971	212.416789	4.79395E-06	0.007078173
1476.519971	192.010141	4.3334E-06	0.006398354
1476.559971	172.481396	3.89266E-06	0.005747753
1476.599971	170.083451	3.83855E-06	0.005667998
1476.639971	185.025684	4.17577E-06	0.006166112

1476.679971	187.660782	4.23524E-06	0.006254098
1476.719971	208.134879	4.69731E-06	0.006936618
1476.759971	245.235382	5.53462E-06	0.008173307
1476.799971	275.465176	6.21687E-06	0.009181067
1476.839971	280.86657	6.33877E-06	0.009361345
1476.879971	265.608653	5.99442E-06	0.008853035
1476.919971	287.879596	6.49704E-06	0.00959561
1476.959971	334.229322	7.54309E-06	0.011140842
1476.999971	373.729016	8.43454E-06	0.01245782
1477.039971	399.944308	9.02619E-06	0.013332037
1477.079971	428.75116	9.67632E-06	0.014292693
1477.119971	467.797978	1.05575E-05	0.015594766
1477.159971	548.397446	1.23766E-05	0.018282169
1477.199971	625.024174	1.41059E-05	0.020837272
1477.239971	704.125702	1.58911E-05	0.023475022
1477.279971	795.117284	1.79447E-05	0.026509331
1477.319971	892.726146	2.01476E-05	0.029764431
1477.359971	924.017717	2.08538E-05	0.030808559
1477.399971	889.259045	2.00693E-05	0.029650439
1477.439971	828.521494	1.86986E-05	0.027626024
1477.479971	701.804375	1.58387E-05	0.023401432
1477.519971	513.177227	1.15817E-05	0.017112186
1477.559971	371.402235	8.38203E-06	0.012384954
1477.599971	290.460879	6.5553E-06	0.009686107
1477.639971	234.832379	5.29984E-06	0.007831255
1477.679971	202.231169	4.56408E-06	0.006744243
1477.719971	207.847497	4.69083E-06	0.006931731
1477.759971	226.11756	5.10316E-06	0.007541243
1477.799971	212.968355	4.8064E-06	0.007102896
1477.839971	212.667695	4.79961E-06	0.007093061
1477.879971	216.781223	4.89245E-06	0.007230454
1477.919971	242.602498	5.4752E-06	0.008091908
1477.959971	255.736132	5.77161E-06	0.008530206
1477.999971	264.759889	5.97526E-06	0.008831437
1478.039971	280.41852	6.32866E-06	0.009354006
1478.079971	293.14534	6.61588E-06	0.009778803
1478.119971	312.356771	7.04946E-06	0.010419944
1478.159971	362.162567	8.1735E-06	0.012081747
1478.199971	416.571713	9.40144E-06	0.013897213
1478.239971	508.72107	1.14811E-05	0.016971859
1478.279971	556.82744	1.25668E-05	0.018577278
1478.319971	589.271212	1.3299E-05	0.019660222
1478.359971	637.556431	1.43888E-05	0.021271768
1478.399971	632.562704	1.42761E-05	0.021105725
1478.439971	547.636918	1.23594E-05	0.018272635
1478.479971	458.326746	1.03438E-05	0.015293096

1478.519971	359.11922	8.10482E-06	0.011983139
1478.559971	292.851487	6.60925E-06	0.009772173
1478.599971	244.581662	5.51987E-06	0.008161676
1478.639971	203.789509	4.59925E-06	0.006800628
1478.679971	195.025243	4.40145E-06	0.006508333
1478.719971	179.234928	4.04508E-06	0.005981545
1478.759971	161.353922	3.64153E-06	0.005384954
1478.799971	169.070552	3.81569E-06	0.005642638
1478.839971	173.508123	3.91584E-06	0.005790896
1478.879971	177.063631	3.99608E-06	0.005909722
1478.919971	173.981388	3.92652E-06	0.005807005
1478.959971	187.325543	4.22768E-06	0.006252564
1478.999971	228.307406	5.15258E-06	0.007620666
1479.039971	236.857255	5.34554E-06	0.007906265
1479.079971	231.911042	5.23391E-06	0.00774137
1479.119971	248.791872	5.61489E-06	0.00830509
1479.159971	277.523608	6.26332E-06	0.009264454
1479.199971	313.630376	7.0782E-06	0.010470074
1479.239971	338.441797	7.63816E-06	0.011298671
1479.279971	340.042199	7.67428E-06	0.011352407
1479.319971	336.720091	7.5993E-06	0.011241801
1479.359971	353.793311	7.98462E-06	0.011812131
1479.399971	367.817897	8.30114E-06	0.012280703
1479.439971	355.512908	8.02343E-06	0.011870185
1479.479971	308.994284	6.97357E-06	0.010317258
1479.519971	235.283598	5.31002E-06	0.007856285
1479.559971	185.74868	4.19209E-06	0.006202447
1479.599971	167.356752	3.77701E-06	0.005588462
1479.639971	158.714369	3.58196E-06	0.005300015
1479.679971	170.718727	3.85288E-06	0.005701035
1479.719971	174.083163	3.92881E-06	0.005813545
1479.759971	171.899549	3.87953E-06	0.005740778
1479.799971	180.50011	4.07364E-06	0.006028167
1479.839971	192.452443	4.34338E-06	0.006427513
1479.879971	194.954855	4.39986E-06	0.006511264
1479.919971	188.122551	4.24566E-06	0.006283243
1479.959971	167.264016	3.77492E-06	0.005586724
1479.999971	166.68455	3.76184E-06	0.00556752
1480.039971	180.334517	4.0699E-06	0.006023613
1480.079971	201.360399	4.54442E-06	0.006726111
1480.119971	219.24381	4.94803E-06	0.007323674
1480.159971	224.024974	5.05593E-06	0.007483588
1480.199971	250.115884	5.64477E-06	0.008355384
1480.239971	271.770133	6.13347E-06	0.009079012
1480.279971	268.15273	6.05183E-06	0.008958408
1480.319971	261.448519	5.90053E-06	0.008734671

1480.359971	260.449739	5.87799E-06	0.008701538
1480.399971	255.765248	5.77227E-06	0.008545262
1480.439971	255.924036	5.77585E-06	0.008550798
1480.479971	225.348218	5.0858E-06	0.007529418
1480.519971	186.044081	4.19876E-06	0.006216342
1480.559971	173.172818	3.90827E-06	0.005786427
1480.599971	177.103127	3.99697E-06	0.005917915
1480.639971	171.042844	3.8602E-06	0.005715565
1480.679971	161.736342	3.65016E-06	0.005404725
1480.719971	165.837516	3.74272E-06	0.005541923
1480.759971	173.612062	3.91818E-06	0.005801888
1480.799971	174.645689	3.94151E-06	0.005836588
1480.839971	189.686328	4.28096E-06	0.006339411
1480.879971	198.585513	4.4818E-06	0.006637006
1480.919971	188.127949	4.24579E-06	0.006287669
1480.959971	183.214768	4.1349E-06	0.006123625
1480.999971	166.293987	3.75302E-06	0.005558228
1481.039971	156.510898	3.53223E-06	0.005231378
1481.079971	168.494507	3.80269E-06	0.005632083
1481.119971	210.609367	4.75316E-06	0.00704
1481.159971	236.31088	5.33321E-06	0.007899333
1481.199971	233.75771	5.27559E-06	0.007814198
1481.239971	253.066649	5.71136E-06	0.008459897
1481.279971	272.98171	6.16082E-06	0.009125895
1481.319971	255.796393	5.77297E-06	0.008551613
1481.359971	217.929315	4.91836E-06	0.007285863
1481.399971	219.160178	4.94614E-06	0.007327212
1481.439971	187.442612	4.23032E-06	0.006266963
1481.479971	155.84045	3.5171E-06	0.005210516
1481.519971	148.072201	3.34178E-06	0.004950919
1481.559971	158.768865	3.58319E-06	0.005308714
1481.599971	160.906861	3.63144E-06	0.005380347
1481.639971	153.648687	3.46764E-06	0.005137789
1481.679971	171.678234	3.87454E-06	0.005740826
1481.719971	192.629823	4.34739E-06	0.00644161
1481.759971	177.107113	3.99706E-06	0.005922685
1481.799971	158.470758	3.57646E-06	0.005299605
1481.839971	157.91054	3.56382E-06	0.005281012
1481.879971	169.061194	3.81548E-06	0.005654077
1481.919971	171.217515	3.86414E-06	0.005726347
1481.959971	173.045126	3.90539E-06	0.005787628
1481.999971	192.192717	4.33752E-06	0.006428207
1482.039971	195.921308	4.42167E-06	0.006553093
1482.079971	168.067146	3.79304E-06	0.005621591
1482.119971	167.761686	3.78615E-06	0.005611525
1482.159971	179.448561	4.0499E-06	0.006002606

1482.199971	195.790074	4.41871E-06	0.006549411
1482.239971	197.036975	4.44685E-06	0.006591299
1482.279971	190.839141	4.30697E-06	0.006384141
1482.319971	203.62026	4.59543E-06	0.006811891
1482.359971	195.4005	4.40992E-06	0.006537085
1482.399971	181.595912	4.09837E-06	0.006075419
1482.439971	182.801731	4.12558E-06	0.006115925
1482.479971	180.116515	4.06498E-06	0.00602625
1482.519971	172.217037	3.8867E-06	0.005762108
1482.559971	183.792965	4.14795E-06	0.006149586
1482.599971	186.246543	4.20333E-06	0.00623185
1482.639971	179.486519	4.05076E-06	0.00600582
1482.679971	166.786677	3.76414E-06	0.005581019
1482.719971	157.191027	3.54758E-06	0.005260071
1482.759971	181.081078	4.08675E-06	0.006059666
1482.799971	189.012354	4.26575E-06	0.006325247
1482.839971	204.392853	4.61286E-06	0.006840136
1482.879971	222.925217	5.03111E-06	0.007460535
1482.919971	203.482975	4.59233E-06	0.006810054
1482.959971	193.401081	4.36479E-06	0.006472813
1482.999971	200.055447	4.51497E-06	0.006695705
1483.039971	192.741456	4.34991E-06	0.006451085
1483.079971	178.194184	4.02159E-06	0.005964346
1483.119971	179.062414	4.04119E-06	0.005993569
1483.159971	193.588445	4.36902E-06	0.006479958
1483.199971	190.861065	4.30747E-06	0.006388837
1483.239971	198.876454	4.48836E-06	0.006657322
1483.279971	218.234218	4.92524E-06	0.007305513
1483.319971	217.557428	4.90997E-06	0.007283054
1483.359971	211.81027	4.78026E-06	0.00709085
1483.399971	203.227925	4.58657E-06	0.00680372
1483.439971	173.340605	3.91206E-06	0.0058033
1483.479971	165.342322	3.73155E-06	0.005535674
1483.519971	162.285621	3.66256E-06	0.005433482
1483.559971	145.558471	3.28505E-06	0.004873572
1483.599971	123.128145	2.77883E-06	0.004122673
1483.639971	125.436427	2.83093E-06	0.004200074
1483.679971	144.315563	3.257E-06	0.004832348
1483.719971	164.272629	3.7074E-06	0.00550075
1483.759971	185.196463	4.17963E-06	0.006201562
1483.799971	196.564091	4.43618E-06	0.0065824
1483.839971	195.35719	4.40894E-06	0.006542161
1483.879971	188.969131	4.26477E-06	0.006328407
1483.919971	171.226566	3.86435E-06	0.005734379
1483.959971	163.03708	3.67952E-06	0.00546026
1483.999971	153.493005	3.46412E-06	0.005140759

1484.039971	150.220027	3.39026E-06	0.005031277
1484.079971	155.36641	3.5064E-06	0.005203783
1484.119971	167.846224	3.78806E-06	0.005621929
1484.159971	192.093223	4.33528E-06	0.006434244
1484.199971	199.849091	4.51032E-06	0.00669421
1484.239971	177.495502	4.00583E-06	0.005945607
1484.279971	166.597424	3.75987E-06	0.005580702
1484.319971	203.708858	4.59743E-06	0.00682405
1484.359971	230.376559	5.19928E-06	0.0077176
1484.399971	215.572974	4.86518E-06	0.007221876
1484.439971	197.661793	4.46095E-06	0.006622014
1484.479971	188.142973	4.24612E-06	0.006303287
1484.519971	169.23822	3.81947E-06	0.005670081
1484.559971	157.99832	3.5658E-06	0.005293647
1484.599971	175.052012	3.95068E-06	0.00586518
1484.639971	180.068087	4.06389E-06	0.006033407
1484.679971	174.767337	3.94426E-06	0.005855957
1484.719971	182.013442	4.10779E-06	0.006098918
1484.759971	197.689236	4.46157E-06	0.006624362
1484.799971	201.407377	4.54548E-06	0.006749134
1484.839971	178.829903	4.03594E-06	0.005992728
1484.879971	152.328779	3.43785E-06	0.005104792
1484.919971	139.029856	3.13771E-06	0.004659249
1484.959971	152.915391	3.45109E-06	0.005124727
1484.999971	175.207969	3.9542E-06	0.005871987
1485.039971	196.38601	4.43216E-06	0.006581933
1485.079971	197.790024	4.46385E-06	0.006629167
1485.119971	231.104423	5.2157E-06	0.007745948
1485.159971	250.599538	5.65568E-06	0.008399593
1485.199971	219.053259	4.94373E-06	0.007342423
1485.239971	212.67214	4.79971E-06	0.007128727
1485.279971	229.066585	5.16971E-06	0.007678472
1485.319971	244.03171	5.50746E-06	0.008180334
1485.359971	207.028748	4.67235E-06	0.006940122
1485.399971	164.79873	3.71928E-06	0.005524615
1485.439971	167.551158	3.7814E-06	0.005617037
1485.479971	184.944959	4.17395E-06	0.006200319
1485.519971	191.744334	4.3274E-06	0.006428443
1485.559971	178.812969	4.03556E-06	0.005995066
1485.599971	158.938488	3.58702E-06	0.005328877
1485.639971	149.952422	3.38422E-06	0.005027728
1485.679971	152.642182	3.44492E-06	0.005118051
1485.719971	169.653962	3.82885E-06	0.005688604
1485.759971	180.418216	4.07179E-06	0.006049699
1485.799971	193.05083	4.35689E-06	0.006473465
1485.839971	202.483357	4.56977E-06	0.006789943

1485.879971	185.373858	4.18363E-06	0.006216372
1485.919971	162.296082	3.6628E-06	0.005442622
1485.959971	159.075756	3.59012E-06	0.005334772
1485.999971	164.905243	3.72168E-06	0.005530419
1486.039971	179.581206	4.0529E-06	0.006022768
1486.079971	184.652757	4.16736E-06	0.006193024
1486.119971	181.570937	4.0978E-06	0.006089827
1486.159971	181.363998	4.09313E-06	0.00608305
1486.199971	181.225108	4.09E-06	0.006078555
1486.239971	180.125183	4.06517E-06	0.006041825
1486.279971	195.833921	4.4197E-06	0.00656891
1486.319971	215.345003	4.86004E-06	0.00722357
1486.359971	224.716925	5.07155E-06	0.007538146
1486.399971	205.889762	4.64665E-06	0.006906773
1486.439971	192.204944	4.3378E-06	0.006447876
1486.479971	180.481552	4.07322E-06	0.006054756
1486.519971	194.874033	4.39804E-06	0.006537768
1486.559971	208.488862	4.7053E-06	0.006994715
1486.599971	202.048325	4.55995E-06	0.00677882
1486.639971	178.48007	4.02805E-06	0.005988255
1486.679971	168.751402	3.80848E-06	0.005661997
1486.719971	170.818361	3.85513E-06	0.005731502
1486.759971	176.19782	3.97654E-06	0.00591216
1486.799971	170.596764	3.85013E-06	0.005724375
1486.839971	186.612675	4.21159E-06	0.006261958
1486.879971	199.409929	4.5004E-06	0.006691561
1486.919971	179.15983	4.04339E-06	0.006012194
1486.959971	143.732651	3.24385E-06	0.004823469
1486.999971	142.261654	3.21065E-06	0.004774233
1487.039971	182.283679	4.11389E-06	0.006117517
1487.079971	192.113091	4.33572E-06	0.00644757
1487.119971	160.34196	3.61869E-06	0.005381433
1487.159971	164.222517	3.70627E-06	0.005511822
1487.199971	195.527529	4.41278E-06	0.006562692
1487.239971	249.615447	5.63347E-06	0.008378326
1487.279971	283.62694	6.40106E-06	0.009520176
1487.319971	280.220778	6.32419E-06	0.009406098
1487.359971	264.939983	5.97933E-06	0.008893411
1487.399971	241.705789	5.45496E-06	0.008113712
1487.439971	230.223909	5.19583E-06	0.00772849
1487.479971	215.12375	4.85504E-06	0.00722178
1487.519971	208.51802	4.70596E-06	0.007000211
1487.559971	188.60137	4.25647E-06	0.006331755
1487.599971	159.556036	3.60096E-06	0.005356784
1487.639971	166.610915	3.76018E-06	0.005593789
1487.679971	185.854203	4.19447E-06	0.00624003

1487.719971	183.396153	4.139E-06	0.006157667
1487.759971	180.407525	4.07155E-06	0.006057484
1487.799971	185.076541	4.17692E-06	0.006214421
1487.839971	200.888587	4.53378E-06	0.006745533
1487.879971	205.551774	4.63902E-06	0.006902301
1487.919971	195.875701	4.42064E-06	0.006577561
1487.959971	179.984182	4.06199E-06	0.006044082
1487.999971	176.14718	3.9754E-06	0.00591539
1488.039971	188.369655	4.25124E-06	0.006326016
1488.079971	207.075528	4.67341E-06	0.006954402
1488.119971	208.218785	4.69921E-06	0.006992985
1488.159971	199.719582	4.50739E-06	0.006707722
1488.199971	195.426566	4.41051E-06	0.006563714
1488.239971	215.716286	4.86842E-06	0.007245371
1488.279971	239.662664	5.40885E-06	0.008049887
1488.319971	228.542769	5.15789E-06	0.007676594
1488.359971	215.356834	4.8603E-06	0.007233882
1488.399971	210.788235	4.7572E-06	0.007080612
1488.439971	202.467239	4.5694E-06	0.006801283
1488.479971	190.331218	4.29551E-06	0.006393781
1488.519971	196.375255	4.43192E-06	0.006596995
1488.559971	194.307904	4.38526E-06	0.006527721
1488.599971	160.156384	3.61451E-06	0.005380554
1488.639971	143.713208	3.24341E-06	0.004828265
1488.679971	151.774975	3.42535E-06	0.00509925
1488.719971	182.270772	4.1136E-06	0.006123995
1488.759971	206.924713	4.67E-06	0.006952513
1488.799971	189.435923	4.2753E-06	0.006365074
1488.839971	187.490545	4.2314E-06	0.006299878
1488.879971	179.420513	4.04927E-06	0.006028879
1488.919971	177.82128	4.01318E-06	0.005975302
1488.959971	187.028287	4.22097E-06	0.006284852
1488.999971	188.579793	4.25598E-06	0.006337159
1489.039971	204.315389	4.61111E-06	0.006866133
1489.079971	204.259038	4.60984E-06	0.006864423
1489.119971	182.378271	4.11602E-06	0.006129253
1489.159971	210.330716	4.74687E-06	0.007068851
1489.199971	240.158105	5.42003E-06	0.008071514
1489.239971	223.06939	5.03437E-06	0.007497378
1489.279971	214.446294	4.83975E-06	0.007207749
1489.319971	224.172023	5.05925E-06	0.007534843
1489.359971	238.487486	5.38233E-06	0.008016227
1489.399971	225.852467	5.09718E-06	0.007591733
1489.439971	197.700243	4.46182E-06	0.006645612
1489.479971	184.133886	4.15565E-06	0.00618975
1489.519971	189.735297	4.28206E-06	0.006378216

1489.559971	181.172515	4.08881E-06	0.00609053
1489.599971	170.044493	3.83767E-06	0.005716589
1489.639971	168.942574	3.8128E-06	0.005679697
1489.679971	157.204308	3.54788E-06	0.005285209
1489.719971	141.808079	3.20041E-06	0.004767716
1489.759971	159.182786	3.59253E-06	0.005352013
1489.799971	188.568714	4.25573E-06	0.006340191
1489.839971	214.596288	4.84314E-06	0.007215503
1489.879971	211.54573	4.77429E-06	0.007113123
1489.919971	175.580896	3.96262E-06	0.005903981
1489.959971	151.210086	3.4126E-06	0.005084639
1489.999971	144.207244	3.25456E-06	0.004849289
1490.039971	163.59556	3.69212E-06	0.005501412
1490.079971	179.589476	4.05308E-06	0.00603942
1490.119971	174.187807	3.93118E-06	0.005857924
1490.159971	180.395831	4.07128E-06	0.006066862
1490.199971	189.580479	4.27857E-06	0.006375921
1490.239971	213.598152	4.82061E-06	0.00718387
1490.279971	234.045386	5.28208E-06	0.007871776
1490.319971	263.415994	5.94493E-06	0.008859851
1490.359971	264.13796	5.96123E-06	0.008884372
1490.399971	232.846313	5.25502E-06	0.007832077
1490.439971	203.406562	4.5906E-06	0.006842018
1490.479971	201.240121	4.54171E-06	0.006769327
1490.519971	201.580184	4.54938E-06	0.006780948
1490.559971	184.960816	4.17431E-06	0.006222056
1490.599971	160.328246	3.61839E-06	0.005393565
1490.639971	139.100716	3.13931E-06	0.00467958
1490.679971	147.939353	3.33879E-06	0.00497706
1490.719971	170.695278	3.85235E-06	0.005742782
1490.759971	201.854061	4.55556E-06	0.006791254
1490.799971	208.27486	4.70047E-06	0.007007466
1490.839971	196.016894	4.42383E-06	0.00659522
1490.879971	188.503672	4.25427E-06	0.006342599
1490.919971	182.35788	4.11556E-06	0.006135976
1490.959971	163.792687	3.69657E-06	0.005511442
1490.999971	161.411334	3.64283E-06	0.005431458
1491.039971	182.345775	4.11529E-06	0.006136062
1491.079971	199.077348	4.4929E-06	0.006699271
1491.119971	192.0367	4.334E-06	0.006462515
1491.159971	178.732213	4.03374E-06	0.006014947
1491.199971	182.955839	4.12906E-06	0.006157252
1491.239971	206.816714	4.66757E-06	0.00696046
1491.279971	214.321721	4.83694E-06	0.007213236
1491.319971	228.648833	5.16029E-06	0.007695637
1491.359971	225.899692	5.09824E-06	0.007603313

1491.399971	204.078323	4.60576E-06	0.006869036
1491.439971	194.326385	4.38568E-06	0.006540972
1491.479971	179.947119	4.06116E-06	0.006057133
1491.519971	173.82719	3.92304E-06	0.005851289
1491.559971	165.930878	3.74483E-06	0.005585637
1491.599971	166.645744	3.76096E-06	0.005609851
1491.639971	159.244563	3.59393E-06	0.005360847
1491.679971	148.699021	3.35593E-06	0.005005973
1491.719971	149.651821	3.37743E-06	0.005038184
1491.759971	156.806975	3.53892E-06	0.005279212
1491.799971	175.264968	3.95549E-06	0.005900794
1491.839971	167.371036	3.77733E-06	0.005635174
1491.879971	152.966059	3.45223E-06	0.005150314
1491.919971	160.678665	3.62629E-06	0.00541014
1491.959971	167.515708	3.7806E-06	0.005640498
1491.999971	166.753777	3.7634E-06	0.005614993
1492.039971	174.16585	3.93068E-06	0.005864733
1492.079971	195.887563	4.42091E-06	0.00659635
1492.119971	194.512312	4.38987E-06	0.006550216
1492.159971	187.78307	4.238E-06	0.006323777
1492.199971	192.573408	4.34611E-06	0.006485271
1492.239971	215.598962	4.86577E-06	0.007260894
1492.279971	223.569577	5.04565E-06	0.007529528
1492.319971	227.420778	5.13257E-06	0.007659437
1492.359971	217.416137	4.90678E-06	0.007322681
1492.399971	227.90378	5.14347E-06	0.007676116
1492.439971	232.709151	5.25192E-06	0.007838177
1492.479971	187.581913	4.23346E-06	0.006318358
1492.519971	146.328004	3.30242E-06	0.004928927
1492.559971	137.420395	3.10139E-06	0.004629006
1492.599971	153.532228	3.46501E-06	0.005171872
1492.639971	177.663069	4.00961E-06	0.005984901
1492.679971	170.202212	3.84123E-06	0.005733722
1492.719971	150.33786	3.39292E-06	0.005064674
1492.759971	154.525731	3.48743E-06	0.005205897
1492.799971	186.20148	4.20231E-06	0.006273205
1492.839971	202.434799	4.56867E-06	0.006820295
1492.879971	181.131444	4.08788E-06	0.006102721
1492.919971	166.59195	3.75975E-06	0.005613003
1492.959971	171.451276	3.86942E-06	0.005776884
1492.999971	180.530795	4.07433E-06	0.006082972
1493.039971	183.25174	4.13574E-06	0.00617482
1493.079971	193.441233	4.3657E-06	0.006518338
1493.119971	210.685503	4.75488E-06	0.007099604
1493.159971	200.408177	4.52293E-06	0.006753463
1493.199971	175.305676	3.9564E-06	0.005907704

1493.239971	174.359274	3.93505E-06	0.005875968
1493.279971	192.992141	4.35556E-06	0.006504076
1493.319971	197.607996	4.45974E-06	0.006659815
1493.359971	190.034717	4.28882E-06	0.00640475
1493.399971	206.272771	4.65529E-06	0.006952209
1493.439971	213.92644	4.82802E-06	0.007210361
1493.479971	204.923525	4.62484E-06	0.006907104
1493.519971	187.782211	4.23798E-06	0.006329512
1493.559971	173.28196	3.91073E-06	0.005840913
1493.599971	158.673405	3.58104E-06	0.005348638
1493.639971	142.670405	3.21987E-06	0.00480933
1493.679971	151.263874	3.41381E-06	0.005099147
1493.719971	173.135686	3.90743E-06	0.005836608
1493.759971	186.649199	4.21241E-06	0.006292333
1493.799971	182.772988	4.12493E-06	0.006161823
1493.839971	173.330646	3.91183E-06	0.00584365
1493.879971	171.333582	3.86676E-06	0.005776476
1493.919971	159.871927	3.60809E-06	0.005390193
1493.959971	161.35572	3.64157E-06	0.005440366
1493.999971	176.336587	3.97967E-06	0.005945629
1494.039971	185.894854	4.19539E-06	0.006268077
1494.079971	186.09999	4.20002E-06	0.006275162
1494.119971	180.27055	4.06846E-06	0.00607876
1494.159971	195.540786	4.41308E-06	0.006593852
1494.199971	207.447749	4.68181E-06	0.006995556
1494.239971	218.665901	4.93498E-06	0.007374052
1494.279971	225.987801	5.10023E-06	0.007621171
1494.319971	223.5004	5.04409E-06	0.007537488
1494.359971	230.276033	5.19701E-06	0.007766203
1494.399971	241.037257	5.43988E-06	0.008129349
1494.439971	237.345984	5.35657E-06	0.00800507
1494.479971	212.379194	4.7931E-06	0.007163196
1494.519971	191.902491	4.33097E-06	0.006472724
1494.559971	173.406159	3.91354E-06	0.005849013
1494.599971	172.971338	3.90372E-06	0.005834503
1494.639971	172.4426	3.89179E-06	0.005816824
1494.679971	178.84278	4.03623E-06	0.006032876
1494.719971	193.278758	4.36203E-06	0.006520017
1494.759971	205.170832	4.63042E-06	0.006921366
1494.799971	204.719906	4.62024E-06	0.006906339
1494.839971	201.254298	4.54203E-06	0.006789607
1494.879971	199.206831	4.49582E-06	0.006720712
1494.919971	194.719789	4.39455E-06	0.006569507
1494.959971	185.791475	4.19305E-06	0.006268449
1494.999971	183.250237	4.1357E-06	0.006182875
1495.039971	186.816742	4.21619E-06	0.006303378

1495.079971	205.849243	4.64573E-06	0.006945739
1495.119971	209.737501	4.73348E-06	0.007077125
1495.159971	215.89342	4.87241E-06	0.007285038
1495.199971	208.167641	4.69805E-06	0.00702453
1495.239971	211.344225	4.76974E-06	0.007131913
1495.279971	219.644951	4.95708E-06	0.007412223
1495.319971	232.945026	5.25724E-06	0.007861263
1495.359971	232.983886	5.25812E-06	0.007862785
1495.399971	229.915395	5.18887E-06	0.007759436
1495.439971	215.359314	4.86036E-06	0.007268376
1495.479971	193.525399	4.3676E-06	0.006531656
1495.519971	165.319878	3.73104E-06	0.005579844
1495.559971	155.583776	3.51131E-06	0.005251373
1495.599971	156.560909	3.53336E-06	0.005284496
1495.639971	173.192872	3.90872E-06	0.005846041
1495.679971	185.695726	4.19089E-06	0.006268236
1495.719971	176.136358	3.97515E-06	0.005945715
1495.759971	175.387593	3.95825E-06	0.005920597
1495.799971	186.375158	4.20623E-06	0.006291675
1495.839971	186.426897	4.2074E-06	0.00629359
1495.879971	183.762474	4.14726E-06	0.006203808
1495.919971	179.83894	4.05871E-06	0.006071512
1495.959971	173.575333	3.91735E-06	0.005860204
1495.999971	176.937985	3.99324E-06	0.005973893
1496.039971	165.922756	3.74465E-06	0.005602139
1496.079971	179.162137	4.04344E-06	0.006049309
1496.119971	200.044572	4.51473E-06	0.006754574
1496.159971	205.43476	4.63638E-06	0.006936761
1496.199971	182.725958	4.12387E-06	0.006170135
1496.239971	196.013706	4.42376E-06	0.006619001
1496.279971	206.10454	4.65149E-06	0.006959935
1496.319971	191.727762	4.32703E-06	0.006474619
1496.359971	205.528843	4.6385E-06	0.006940865
1496.399971	211.972044	4.78391E-06	0.007158648
1496.439971	203.599289	4.59495E-06	0.00687607
1496.479971	182.938885	4.12868E-06	0.006178481
1496.519971	166.549107	3.75878E-06	0.005625091
1496.559971	151.462534	3.4183E-06	0.005115689
1496.599971	150.035735	3.3861E-06	0.005067634
1496.639971	146.573923	3.30797E-06	0.004950839
1496.679971	143.166064	3.23106E-06	0.004835861
1496.719971	161.379582	3.64211E-06	0.005451222
1496.759971	197.969317	4.46789E-06	0.006687362
1496.799971	230.74648	5.20763E-06	0.007794775
1496.839971	248.011434	5.59727E-06	0.008378221
1496.879971	242.564618	5.47435E-06	0.008194438

1496.919971	210.939928	4.76062E-06	0.007126268
1496.959971	185.403527	4.1843E-06	0.006263729
1496.999971	178.345111	4.025E-06	0.006025426
1497.039971	178.546494	4.02955E-06	0.006032391
1497.079971	189.575403	4.27845E-06	0.006405186
1497.119971	199.109894	4.49363E-06	0.006727508
1497.159971	209.033962	4.71761E-06	0.00706301
1497.199971	214.076909	4.83142E-06	0.007233598
1497.239971	211.303146	4.76882E-06	0.007140064
1497.279971	197.799394	4.46406E-06	0.006683943
1497.319971	180.523759	4.07417E-06	0.006100336
1497.359971	180.958002	4.08397E-06	0.006115173
1497.399971	184.919964	4.17339E-06	0.006249228
1497.439971	201.802484	4.5544E-06	0.006819942
1497.479971	215.014523	4.85258E-06	0.007266639
1497.519971	189.510854	4.277E-06	0.006404887
1497.559971	151.965216	3.42964E-06	0.005136096
1497.599971	133.760597	3.01879E-06	0.00452094
1497.639971	138.002756	3.11453E-06	0.004664445
1497.679971	153.313033	3.46006E-06	0.005182065
1497.719971	155.996818	3.52063E-06	0.005272919
1497.759971	153.734557	3.46957E-06	0.00519659
1497.799971	148.218146	3.34508E-06	0.005010256
1497.839971	154.414809	3.48493E-06	0.005219863
1497.879971	184.806328	4.17082E-06	0.00624739
1497.919971	202.928161	4.57981E-06	0.006860183
1497.959971	192.099204	4.33541E-06	0.006494273
1497.999971	177.67866	4.00996E-06	0.00600692
1498.039971	170.756892	3.85375E-06	0.005773064
1498.079971	159.955606	3.60998E-06	0.005408032
1498.119971	161.135507	3.6366E-06	0.005448069
1498.159971	185.700235	4.191E-06	0.006278782
1498.199971	223.953713	5.05432E-06	0.007572387
1498.239971	227.711288	5.13913E-06	0.007699645
1498.279971	199.570032	4.50402E-06	0.006748279
1498.319971	181.606065	4.0986E-06	0.006141008
1498.359971	208.231364	4.69949E-06	0.00704153
1498.399971	218.745779	4.93679E-06	0.007397282
1498.439971	192.890116	4.35326E-06	0.006523101
1498.479971	181.348331	4.09278E-06	0.006132948
1498.519971	181.810191	4.1032E-06	0.006148731
1498.559971	189.548394	4.27784E-06	0.006410605
1498.599971	184.346392	4.16044E-06	0.006234837
1498.639971	183.472337	4.14072E-06	0.006205441
1498.679971	170.630603	3.8509E-06	0.005771259
1498.719971	148.227567	3.34529E-06	0.005013653

1498.759971	147.215016	3.32244E-06	0.004979537
1498.799971	158.060288	3.5672E-06	0.00534652
1498.839971	171.611866	3.87304E-06	0.005805068
1498.879971	184.200847	4.15716E-06	0.006231079
1498.919971	183.639787	4.14449E-06	0.006212265
1498.959971	170.592471	3.85003E-06	0.005771048
1498.999971	161.04319	3.63452E-06	0.005448146
1499.039971	183.423476	4.13961E-06	0.006205444
1499.079971	196.363752	4.43166E-06	0.006643407
1499.119971	193.684826	4.3712E-06	0.006552948
1499.159971	183.961773	4.15176E-06	0.006224154
1499.199971	195.416276	4.41027E-06	0.006611881
1499.239971	210.382216	4.74803E-06	0.007118442
1499.279971	214.278867	4.83598E-06	0.007250481
1499.319971	240.578912	5.42953E-06	0.008140604
1499.359971	269.071804	6.07258E-06	0.009104977
1499.399971	256.559619	5.79019E-06	0.008681816
1499.439971	218.282087	4.92632E-06	0.007386725
1499.479971	197.27407	4.4522E-06	0.006675986
1499.519971	182.058381	4.1088E-06	0.006161234
1499.559971	181.967135	4.10674E-06	0.00615831
1499.599971	177.449063	4.00478E-06	0.006005565
1499.639971	177.846667	4.01375E-06	0.006019182
1499.679971	162.822204	3.67467E-06	0.00551083
1499.719971	157.403059	3.55237E-06	0.005327557
1499.759971	183.642405	4.14455E-06	0.006215835
1499.799971	210.482345	4.75029E-06	0.00712449
1499.839971	199.163762	4.49485E-06	0.006741554
1499.879971	172.669517	3.89691E-06	0.005844898
1499.919971	165.514404	3.73543E-06	0.005602845
1499.959971	164.374448	3.7097E-06	0.005564405
1499.999971	167.88689	3.78897E-06	0.00568346
1500.039971	159.30366	3.59526E-06	0.005393036
1500.079971	160.234815	3.61628E-06	0.005424704
1500.119971	167.71318	3.78505E-06	0.005678033
1500.159971	185.230415	4.18039E-06	0.006271257
1500.199971	202.122097	4.56161E-06	0.006843333
1500.239971	206.690201	4.66471E-06	0.006998184
1500.279971	218.631708	4.93421E-06	0.007402701
1500.319971	225.541617	5.09016E-06	0.007636869
1500.359971	225.081412	5.07977E-06	0.00762149
1500.399971	221.849117	5.00683E-06	0.007512241
1500.439971	206.837377	4.66803E-06	0.007004101
1500.479971	197.256676	4.45181E-06	0.006679849
1500.519971	182.976425	4.12952E-06	0.006196432
1500.559971	175.734078	3.96607E-06	0.005951331

1500.599971	176.15228	3.97551E-06	0.005965653
1500.639971	169.801924	3.83219E-06	0.005750742
1500.679971	165.470733	3.73444E-06	0.005604205
1500.719971	179.709761	4.0558E-06	0.006086619
1500.759971	195.029364	4.40154E-06	0.006605657
1500.799971	189.309345	4.27245E-06	0.00641209
1500.839971	177.894525	4.01483E-06	0.00602562
1500.879971	169.108853	3.81655E-06	0.005728185
1500.919971	168.449913	3.80168E-06	0.005706017
1500.959971	166.800174	3.76445E-06	0.005650285
1500.999971	174.541743	3.93916E-06	0.005912685
1501.039971	202.154727	4.56235E-06	0.006848271
1501.079971	215.943712	4.87355E-06	0.007315586
1501.119971	215.677	4.86753E-06	0.007306746
1501.159971	208.94049	4.7155E-06	0.007078714
1501.199971	214.525276	4.84154E-06	0.007268115
1501.239971	232.081206	5.23775E-06	0.007863119
1501.279971	248.345857	5.60482E-06	0.008414404
1501.319971	237.911432	5.36933E-06	0.008061082
1501.359971	208.114058	4.69684E-06	0.007051654
1501.399971	186.854694	4.21705E-06	0.006331479
1501.439971	175.645846	3.96408E-06	0.005951831
1501.479971	181.895042	4.10512E-06	0.006163752
1501.519971	176.204245	3.97668E-06	0.005971071
1501.559971	155.314145	3.50522E-06	0.005263304
1501.599971	139.211025	3.1418E-06	0.004717725
1501.639971	141.508827	3.19366E-06	0.004795723
1501.679971	150.180678	3.38937E-06	0.005089747
1501.719971	154.11766	3.47822E-06	0.005223314
1501.759971	146.379917	3.30359E-06	0.0049612
1501.799971	142.668949	3.21984E-06	0.004835555
1501.839971	146.725259	3.31138E-06	0.00497317
1501.879971	159.18103	3.59249E-06	0.005395495
1501.919971	174.95469	3.94848E-06	0.005930306
1501.959971	170.065064	3.83813E-06	0.00576472
1501.999971	180.717646	4.07855E-06	0.006125975
1502.039971	175.806416	3.96771E-06	0.005959653
1502.079971	167.290943	3.77552E-06	0.005671138
1502.119971	190.085265	4.28996E-06	0.006444034
1502.159971	191.459188	4.32097E-06	0.006490784
1502.199971	188.925279	4.26378E-06	0.006405051
1502.239971	199.923356	4.51199E-06	0.006778094
1502.279971	217.540068	4.90958E-06	0.007375558
1502.319971	238.321105	5.37858E-06	0.008080341
1502.359971	231.808337	5.23159E-06	0.007859733
1502.399971	216.895695	4.89503E-06	0.007354299

1502.439971	192.431111	4.3429E-06	0.00652495
1502.479971	174.053172	3.92814E-06	0.005901948
1502.519971	164.393923	3.71014E-06	0.005574562
1502.559971	155.466997	3.50867E-06	0.005271992
1502.599971	146.731567	3.31153E-06	0.004975901
1502.639971	135.059112	3.0481E-06	0.004580191
1502.679971	139.774895	3.15452E-06	0.004740241
1502.719971	150.741706	3.40203E-06	0.005112299
1502.759971	166.683033	3.7618E-06	0.005653088
1502.799971	179.62636	4.05392E-06	0.006092226
1502.839971	187.646143	4.23491E-06	0.006364395
1502.879971	187.546444	4.23266E-06	0.006361183
1502.919971	185.860068	4.1946E-06	0.006304152
1502.959971	181.33506	4.09248E-06	0.006150833
1502.999971	169.565394	3.82685E-06	0.005751763
1503.039971	161.785405	3.65127E-06	0.005488007
1503.079971	162.018751	3.65654E-06	0.005496068
1503.119971	191.431789	4.32035E-06	0.006494003
1503.159971	196.610822	4.43723E-06	0.00666987
1503.199971	184.485047	4.16357E-06	0.006258679
1503.239971	223.821897	5.05135E-06	0.007593389
1503.279971	254.838551	5.75135E-06	0.008645891
1503.319971	246.324215	5.55919E-06	0.008357248
1503.359971	213.382872	4.81575E-06	0.007239812
1503.399971	202.179597	4.56291E-06	0.006859882
1503.439971	195.112677	4.40342E-06	0.00662028
1503.479971	179.921581	4.06058E-06	0.006105
1503.519971	167.423792	3.77852E-06	0.005681083
1503.559971	162.215652	3.66098E-06	0.005504505
1503.599971	156.947125	3.54208E-06	0.005325868
1503.639971	142.55119	3.21718E-06	0.004837483
1503.679971	139.143865	3.14028E-06	0.004721981
1503.719971	133.625443	3.01574E-06	0.004534829
1503.759971	130.535601	2.94601E-06	0.004430087
1503.799971	154.252817	3.48127E-06	0.005235136
1503.839971	180.795914	4.08031E-06	0.006136136
1503.879971	167.226242	3.77406E-06	0.005675738
1503.919971	152.366596	3.4387E-06	0.005171532
1503.959971	148.338261	3.34779E-06	0.005034939
1503.999971	153.790222	3.47083E-06	0.00522013
1504.039971	154.15658	3.4791E-06	0.005232704
1504.079971	168.871652	3.8112E-06	0.005732347
1504.119971	175.946035	3.97086E-06	0.005972645
1504.159971	161.449521	3.64369E-06	0.005480694
1504.199971	167.072965	3.7706E-06	0.005671743
1504.239971	193.129997	4.35868E-06	0.006556493

1504.279971	213.653074	4.82185E-06	0.007253416
1504.319971	225.627521	5.0921E-06	0.007660146
1504.359971	222.436835	5.02009E-06	0.007552022
1504.399971	216.49183	4.88592E-06	0.007350376
1504.439971	206.022329	4.64964E-06	0.0069951
1504.479971	188.721564	4.25918E-06	0.006407855
1504.519971	152.341232	3.43813E-06	0.005172734
1504.559971	141.788831	3.19998E-06	0.004814556
1504.599971	160.448009	3.62109E-06	0.005448289
1504.639971	167.203019	3.77354E-06	0.005677818
1504.679971	164.117769	3.70391E-06	0.005573198
1504.719971	158.770509	3.58323E-06	0.005391757
1504.759971	159.116875	3.59105E-06	0.005403663
1504.799971	181.001455	4.08495E-06	0.006147034
1504.839971	190.981207	4.31018E-06	0.006486131
1504.879971	178.346105	4.02502E-06	0.006057177
1504.919971	149.255746	3.36849E-06	0.005069314
1504.959971	126.411942	2.85294E-06	0.004293563
1504.999971	125.651072	2.83577E-06	0.004267833
1505.039971	141.485971	3.19314E-06	0.004805805
1505.079971	164.982901	3.72343E-06	0.005604066
1505.119971	187.970888	4.24224E-06	0.006385082
1505.159971	208.8099	4.71255E-06	0.00709314
1505.199971	212.692319	4.80017E-06	0.007225215
1505.239971	212.633622	4.79884E-06	0.007223413
1505.279971	211.16972	4.76581E-06	0.007173873
1505.319971	202.064976	4.56033E-06	0.006864748
1505.359971	202.885238	4.57884E-06	0.006892798
1505.399971	211.382496	4.77061E-06	0.007181674
1505.439971	192.893749	4.35334E-06	0.006553697
1505.479971	185.507881	4.18665E-06	0.006302924
1505.519971	182.629712	4.1217E-06	0.006205299
1505.559971	181.189639	4.0892E-06	0.006156532
1505.599971	185.996225	4.19768E-06	0.00632002
1505.639971	178.996375	4.0397E-06	0.006082332
1505.679971	160.947666	3.63236E-06	0.005469179
1505.719971	174.989904	3.94928E-06	0.005946507
1505.759971	199.387541	4.4999E-06	0.006775768
1505.799971	217.19012	4.90168E-06	0.007380947
1505.839971	194.10792	4.38075E-06	0.006596702
1505.879971	159.362555	3.59659E-06	0.005416034
1505.919971	150.301182	3.39209E-06	0.005108214
1505.959971	159.524867	3.60025E-06	0.005421838
1505.999971	169.127755	3.81698E-06	0.005748369
1506.039971	167.433293	3.77874E-06	0.005690928
1506.079971	158.973983	3.58782E-06	0.005403546

1506.119971	168.637252	3.80591E-06	0.005732154
1506.159971	173.913166	3.92498E-06	0.005911645
1506.199971	178.849164	4.03638E-06	0.00607959
1506.239971	177.786302	4.01239E-06	0.006043621
1506.279971	202.436097	4.5687E-06	0.006881742
1506.319971	255.099773	5.75725E-06	0.008672255
1506.359971	264.60956	5.97187E-06	0.008995785
1506.399971	217.208979	4.9021E-06	0.007384529
1506.439971	181.605937	4.09859E-06	0.006174284
1506.479971	159.873852	3.60813E-06	0.005435576
1506.519971	153.172249	3.45688E-06	0.005207865
1506.559971	163.96367	3.70043E-06	0.005574922
1506.599971	168.304881	3.79841E-06	0.005722679
1506.639971	167.584195	3.78214E-06	0.005698326
1506.679971	171.663579	3.87421E-06	0.005837191
1506.719971	174.874562	3.94668E-06	0.005946534
1506.759971	175.717914	3.96571E-06	0.005975371
1506.799971	173.455949	3.91466E-06	0.005898608
1506.839971	170.045243	3.83768E-06	0.005782776
1506.879971	165.36138	3.73198E-06	0.00562364
1506.919971	177.153338	3.9981E-06	0.006024823
1506.959971	158.23446	3.57113E-06	0.005381552
1506.999971	144.351041	3.2578E-06	0.004909507
1507.039971	166.712363	3.76247E-06	0.005670186
1507.079971	189.692108	4.28109E-06	0.00645194
1507.119971	189.002084	4.26551E-06	0.006428641
1507.159971	204.385116	4.61269E-06	0.006952058
1507.199971	214.552492	4.84215E-06	0.00729809
1507.239971	204.245098	4.60953E-06	0.006947664
1507.279971	216.065854	4.87631E-06	0.007349957
1507.319971	235.89484	5.32382E-06	0.008024697
1507.359971	219.820016	4.96103E-06	0.00747806
1507.399971	188.174122	4.24683E-06	0.006401668
1507.439971	180.853893	4.08162E-06	0.006152798
1507.479971	179.77965	4.05738E-06	0.006116413
1507.519971	179.076917	4.04152E-06	0.006092667
1507.559971	175.687318	3.96502E-06	0.005977503
1507.599971	166.385894	3.7551E-06	0.005661185
1507.639971	151.579094	3.42093E-06	0.005157529
1507.679971	160.122519	3.61374E-06	0.005448367
1507.719971	164.404607	3.71038E-06	0.005594219
1507.759971	152.188755	3.43469E-06	0.005178685
1507.799971	139.338777	3.14468E-06	0.004741552
1507.839971	145.754927	3.28949E-06	0.004960018
1507.879971	165.546045	3.73614E-06	0.005633656
1507.919971	168.242152	3.79699E-06	0.005725559

1507.959971	168.255006	3.79728E-06	0.005726148
1507.999971	174.298712	3.93368E-06	0.005931988
1508.039971	171.759064	3.87636E-06	0.00584571
1508.079971	172.690033	3.89737E-06	0.005877551
1508.119971	172.539665	3.89398E-06	0.005872589
1508.159971	179.377496	4.0483E-06	0.006105484
1508.199971	198.090289	4.47062E-06	0.006742592
1508.239971	209.693688	4.73249E-06	0.007137737
1508.279971	233.995189	5.28095E-06	0.007965144
1508.319971	239.935724	5.41502E-06	0.008167575
1508.359971	224.46414	5.06584E-06	0.007641115
1508.399971	225.528754	5.08987E-06	0.00767756
1508.439971	218.352389	4.92791E-06	0.007433455
1508.479971	193.249525	4.36137E-06	0.006579043
1508.519971	176.195063	3.97648E-06	0.005998595
1508.559971	156.957479	3.54231E-06	0.00534379
1508.599971	135.963917	3.06852E-06	0.004629163
1508.639971	139.856537	3.15637E-06	0.004761822
1508.679971	146.711545	3.31108E-06	0.004995353
1508.719971	172.510914	3.89333E-06	0.005873946
1508.759971	208.427864	4.70393E-06	0.007097096
1508.799971	222.070437	5.01182E-06	0.007561835
1508.839971	224.615982	5.06927E-06	0.007648717
1508.879971	213.716591	4.82329E-06	0.007277759
1508.919971	209.797224	4.73483E-06	0.007144481
1508.959971	212.909081	4.80506E-06	0.007250645
1508.999971	216.73354	4.89137E-06	0.007381083
1509.039971	220.582949	4.97825E-06	0.007512378
1509.079971	218.487673	4.93096E-06	0.007441217
1509.119971	211.528749	4.77391E-06	0.007204402
1509.159971	214.361933	4.83785E-06	0.00730109
1509.199971	226.671967	5.11567E-06	0.00772057
1509.239971	231.007496	5.21352E-06	0.007868449
1509.279971	238.771412	5.38874E-06	0.008133115
1509.319971	216.155269	4.87832E-06	0.007362951
1509.359971	187.123645	4.22312E-06	0.006374208
1509.399971	171.828266	3.87792E-06	0.005853339
1509.439971	170.205192	3.84129E-06	0.005798203
1509.479971	195.289502	4.40741E-06	0.0066529
1509.519971	188.141548	4.24609E-06	0.006409562
1509.559971	158.804336	3.58399E-06	0.005410252
1509.599971	127.793111	2.88411E-06	0.004353856
1509.639971	119.531612	2.69766E-06	0.004072498
1509.679971	132.655239	2.99384E-06	0.004519746
1509.719971	149.394636	3.37163E-06	0.005090215
1509.759971	154.207775	3.48025E-06	0.005254349

1509.799971	167.718569	3.78517E-06	0.005714856
1509.839971	190.033783	4.2888E-06	0.006475398
1509.879971	184.461461	4.16304E-06	0.006285688
1509.919971	186.798902	4.21579E-06	0.006365507
1509.959971	195.472388	4.41154E-06	0.006661248
1509.999971	191.097555	4.31281E-06	0.006512336
1510.039971	193.739634	4.37243E-06	0.00660255
1510.079971	185.812768	4.19354E-06	0.006332574
1510.119971	178.806027	4.0354E-06	0.006093943
1510.159971	183.94347	4.15135E-06	0.006269199
1510.199971	180.647874	4.07697E-06	0.006157041
1510.239971	192.063661	4.33461E-06	0.0065463
1510.279971	208.726147	4.71066E-06	0.007114413
1510.319971	243.385808	5.49288E-06	0.008296004
1510.359971	256.338587	5.7852E-06	0.008737742
1510.399971	230.847922	5.20992E-06	0.007869057
1510.439971	211.984151	4.78419E-06	0.007226227
1510.479971	194.396685	4.38726E-06	0.006626872
1510.519971	166.29777	3.75311E-06	0.005669146
1510.559971	163.38965	3.68748E-06	0.005570155
1510.599971	174.957525	3.94855E-06	0.005964676
1510.639971	161.815764	3.65196E-06	0.005516791
1510.679971	145.013419	3.27275E-06	0.004944079
1510.719971	146.34375	3.30277E-06	0.004989568
1510.759971	139.769443	3.1544E-06	0.004765544
1510.799971	146.2833	3.30141E-06	0.004987771
1510.839971	156.697146	3.53644E-06	0.005342989
1510.879971	159.302319	3.59523E-06	0.005431963
1510.919971	179.41499	4.04915E-06	0.006117936
1510.959971	181.909483	4.10544E-06	0.006203161
1510.999971	172.481858	3.89268E-06	0.005881832
1511.039971	174.540701	3.93914E-06	0.005952199
1511.079971	174.64395	3.94147E-06	0.005955877
1511.119971	168.005515	3.79165E-06	0.005729639
1511.159971	175.697945	3.96526E-06	0.005992139
1511.199971	215.733042	4.86879E-06	0.007357722
1511.239971	241.049978	5.44016E-06	0.008221391
1511.279971	215.192877	4.8566E-06	0.007339688
1511.319971	196.9722	4.44539E-06	0.006718404
1511.359971	194.466149	4.38883E-06	0.006633102
1511.399971	186.732201	4.21429E-06	0.006369471
1511.439971	163.748136	3.69557E-06	0.005585628
1511.479971	150.786431	3.40304E-06	0.005143626
1511.519971	160.540811	3.62318E-06	0.005476513
1511.559971	158.843505	3.58488E-06	0.005418756
1511.599971	150.467288	3.39584E-06	0.005133147

1511.639971	171.279384	3.86554E-06	0.0058433
1511.679971	181.448741	4.09505E-06	0.006190398
1511.719971	174.147958	3.93028E-06	0.005941478
1511.759971	175.325271	3.95685E-06	0.005981803
1511.799971	171.216534	3.86412E-06	0.005841775
1511.839971	162.805003	3.67428E-06	0.005554927
1511.879971	158.158713	3.56942E-06	0.005396537
1511.919971	178.446587	4.02729E-06	0.006088942
1511.959971	191.476789	4.32136E-06	0.00653373
1511.999971	183.796308	4.14803E-06	0.006271816
1512.039971	185.773654	4.19265E-06	0.006339458
1512.079971	185.245493	4.18073E-06	0.006321602
1512.119971	191.408339	4.31982E-06	0.006532085
1512.159971	191.660135	4.3255E-06	0.006540851
1512.199971	191.677696	4.3259E-06	0.006541624
1512.239971	182.737058	4.12412E-06	0.00623666
1512.279971	176.682948	3.98749E-06	0.006030198
1512.319971	205.770159	4.64395E-06	0.007023132
1512.359971	219.391645	4.95136E-06	0.007488244
1512.399971	201.253199	4.542E-06	0.006869327
1512.439971	193.859306	4.37513E-06	0.006617128
1512.479971	186.366461	4.20603E-06	0.006361538
1512.519971	198.750811	4.48553E-06	0.006784452
1512.559971	185.343328	4.18294E-06	0.006326949
1512.599971	158.997646	3.58836E-06	0.005427746
1512.639971	165.240565	3.72925E-06	0.005641012
1512.679971	182.548486	4.11987E-06	0.006232037
1512.719971	175.771486	3.96692E-06	0.006000835
1512.759971	146.720579	3.31128E-06	0.00500917
1512.799971	139.906616	3.1575E-06	0.004776662
1512.839971	155.820806	3.51666E-06	0.005320142
1512.879971	174.193538	3.93131E-06	0.005947593
1512.919971	186.861825	4.21721E-06	0.006380303
1512.959971	195.41654	4.41028E-06	0.006672576
1512.999971	209.552562	4.72931E-06	0.007155445
1513.039971	210.437856	4.74929E-06	0.007185865
1513.079971	192.833384	4.35198E-06	0.006584895
1513.119971	179.985306	4.06202E-06	0.00614632
1513.159971	184.41505	4.16199E-06	0.006297758
1513.199971	191.637444	4.32499E-06	0.006544575
1513.239971	192.849247	4.35234E-06	0.006586133
1513.279971	196.512767	4.43502E-06	0.006711426
1513.319971	212.746229	4.80139E-06	0.007266033
1513.359971	223.267988	5.03885E-06	0.00762559
1513.399971	212.130488	4.78749E-06	0.007245387
1513.439971	195.836911	4.41977E-06	0.006689051

1513.479971	179.502547	4.05112E-06	0.006131293
1513.519971	176.096829	3.97426E-06	0.006015122
1513.559971	177.047736	3.99572E-06	0.006047763
1513.599971	166.808308	3.76463E-06	0.005698146
1513.639971	157.373269	3.5517E-06	0.005375988
1513.679971	139.886388	3.15704E-06	0.00477875
1513.719971	143.640107	3.24176E-06	0.004907113
1513.759971	169.900722	3.83442E-06	0.005804396
1513.799971	186.935241	4.21887E-06	0.006386522
1513.839971	172.210194	3.88654E-06	0.005883606
1513.879971	165.15566	3.72733E-06	0.005642735
1513.919971	160.320622	3.61821E-06	0.005477685
1513.959971	160.99607	3.63346E-06	0.005500909
1513.999971	161.363354	3.64175E-06	0.005513604
1514.039971	176.32845	3.97949E-06	0.006025103
1514.079971	177.190852	3.99895E-06	0.006054731
1514.119971	166.442188	3.75637E-06	0.005687592
1514.159971	167.323757	3.77626E-06	0.005717868
1514.199971	188.23249	4.24815E-06	0.006432541
1514.239971	205.123644	4.62935E-06	0.007009954
1514.279971	229.32099	5.17546E-06	0.007837088
1514.319971	240.986972	5.43874E-06	0.008235993
1514.359971	223.760966	5.04997E-06	0.007647477
1514.399971	209.515538	4.72847E-06	0.007160801
1514.439971	194.078141	4.38007E-06	0.006633358
1514.479971	171.443347	3.86924E-06	0.005859883
1514.519971	154.450008	3.48572E-06	0.005279195
1514.559971	148.079051	3.34194E-06	0.005061565
1514.599971	142.943704	3.22604E-06	0.004886161
1514.639971	135.345228	3.05455E-06	0.004626548
1514.679971	136.186746	3.07355E-06	0.004655437
1514.719971	143.862499	3.24678E-06	0.004917957
1514.759971	164.370053	3.7096E-06	0.005619158
1514.799971	168.705084	3.80744E-06	0.005767508
1514.839971	160.01861	3.6114E-06	0.005470689
1514.879971	159.112279	3.59094E-06	0.005439847
1514.919971	162.709155	3.67212E-06	0.005562966
1514.959971	158.189174	3.57011E-06	0.005408573
1514.999971	154.223915	3.48062E-06	0.005273138
1515.039971	161.704624	3.64945E-06	0.00552906
1515.079971	171.447126	3.86932E-06	0.005862334
1515.119971	183.843459	4.14909E-06	0.00628637
1515.159971	187.731962	4.23685E-06	0.006419504
1515.199971	197.610922	4.4598E-06	0.006757494
1515.239971	232.411092	5.24519E-06	0.007947729
1515.279971	258.029665	5.82337E-06	0.008824036

1515.319971	247.688771	5.58999E-06	0.008470624
1515.359971	231.235315	5.21866E-06	0.007908147
1515.399971	215.003596	4.85233E-06	0.007353223
1515.439971	215.580249	4.86535E-06	0.00737314
1515.479971	228.451878	5.15584E-06	0.007813573
1515.519971	205.842636	4.64558E-06	0.007040472
1515.559971	176.41106	3.98135E-06	0.006033978
1515.599971	145.850485	3.29164E-06	0.004988813
1515.639971	148.108469	3.3426E-06	0.005066181
1515.679971	163.328852	3.6861E-06	0.005586955
1515.719971	156.429319	3.53039E-06	0.005351085
1515.759971	146.104437	3.29737E-06	0.004998027
1515.799971	167.787891	3.78674E-06	0.005739939
1515.839971	202.717944	4.57506E-06	0.006935061
1515.879971	200.742336	4.53047E-06	0.006867656
1515.919971	170.03976	3.83756E-06	0.005817435
1515.959971	164.996755	3.72375E-06	0.005645051
1515.999971	160.690282	3.62656E-06	0.005497859
1516.039971	151.878682	3.42769E-06	0.005196516
1516.079971	158.456529	3.57614E-06	0.005421719
1516.119971	160.189274	3.61525E-06	0.005481151
1516.159971	174.59785	3.94043E-06	0.005974323
1516.199971	209.740428	4.73355E-06	0.007177007
1516.239971	217.158455	4.90096E-06	0.007431037
1516.279971	217.047277	4.89845E-06	0.007427429
1516.319971	217.109357	4.89986E-06	0.007429749
1516.359971	223.30048	5.03958E-06	0.007641819
1516.399971	220.592776	4.97847E-06	0.007549354
1516.439971	222.526148	5.02211E-06	0.007615721
1516.479971	222.691116	5.02583E-06	0.007621568
1516.519971	205.320006	4.63379E-06	0.00702723
1516.559971	178.215449	4.02207E-06	0.006099717
1516.599971	153.760052	3.47015E-06	0.00526283
1516.639971	151.995905	3.43034E-06	0.005202584
1516.679971	154.794593	3.4935E-06	0.005298519
1516.719971	159.028942	3.58906E-06	0.005443602
1516.759971	165.722194	3.74012E-06	0.005672863
1516.799971	165.814988	3.74221E-06	0.005676189
1516.839971	183.868988	4.14967E-06	0.006294381
1516.879971	167.484935	3.7799E-06	0.005733657
1516.919971	146.894179	3.3152E-06	0.005028889
1516.959971	134.648373	3.03883E-06	0.004609778
1516.999971	129.624496	2.92544E-06	0.004437899
1517.039971	148.399689	3.34917E-06	0.005080831
1517.079971	164.612952	3.71509E-06	0.005636081
1517.119971	171.806185	3.87743E-06	0.005882521

1517.159971	181.107952	4.08735E-06	0.00620117
1517.199971	205.441856	4.63654E-06	0.007034553
1517.239971	217.446111	4.90746E-06	0.007445788
1517.279971	224.529564	5.06732E-06	0.007688542
1517.319971	223.691732	5.04841E-06	0.007660055
1517.359971	224.785852	5.0731E-06	0.007697724
1517.399971	210.302938	4.74624E-06	0.007201951
1517.439971	194.170811	4.38216E-06	0.006649672
1517.479971	168.358323	3.79961E-06	0.005765836
1517.519971	152.351235	3.43836E-06	0.005217773
1517.559971	148.817268	3.3586E-06	0.005096875
1517.599971	143.112551	3.22985E-06	0.004901622
1517.639971	137.4955	3.10308E-06	0.004709361
1517.679971	137.402784	3.10099E-06	0.00470631
1517.719971	142.473776	3.21543E-06	0.004880129
1517.759971	142.423118	3.21429E-06	0.004878523
1517.799971	145.731152	3.28895E-06	0.004991967
1517.839971	170.78634	3.85441E-06	0.005850377
1517.879971	170.756211	3.85373E-06	0.005849499
1517.919971	163.513866	3.69028E-06	0.00560155
1517.959971	166.927587	3.76732E-06	0.005718646
1517.999971	178.862127	4.03667E-06	0.006127664
1518.039971	190.515809	4.29968E-06	0.006527081
1518.079971	186.19444	4.20215E-06	0.006379198
1518.119971	180.766027	4.07964E-06	0.006193379
1518.159971	173.746753	3.92122E-06	0.005953043
1518.199971	196.129804	4.42638E-06	0.006720125
1518.239971	217.070768	4.89898E-06	0.007437835
1518.279971	241.592398	5.4524E-06	0.008278276
1518.319971	242.635174	5.47594E-06	0.008314226
1518.359971	231.20548	5.21799E-06	0.007922781
1518.399971	250.83271	5.66094E-06	0.008595579
1518.439971	241.354052	5.44702E-06	0.00827098
1518.479971	196.435773	4.43328E-06	0.00673185
1518.519971	171.21235	3.86402E-06	0.005867598
1518.559971	147.991586	3.33996E-06	0.005071936
1518.599971	147.396371	3.32653E-06	0.00505167
1518.639971	158.176261	3.56982E-06	0.005421268
1518.679971	162.107652	3.65854E-06	0.005556157
1518.719971	169.887088	3.83411E-06	0.005822947
1518.759971	174.700521	3.94275E-06	0.005988087
1518.799971	162.664516	3.67111E-06	0.005575684
1518.839971	151.536577	3.41997E-06	0.005194386
1518.879971	158.809368	3.58411E-06	0.005443827
1518.919971	159.905264	3.60884E-06	0.005481538
1518.959971	166.379381	3.75495E-06	0.00570362

1518.999971	177.969106	4.01651E-06	0.006101086
1519.039971	188.753073	4.25989E-06	0.006470949
1519.079971	182.577836	4.12053E-06	0.006259411
1519.119971	190.271057	4.29415E-06	0.006523333
1519.159971	208.032972	4.69501E-06	0.007132478
1519.199971	224.536833	5.06748E-06	0.007698521
1519.239971	240.477531	5.42724E-06	0.008245284
1519.279971	242.744593	5.47841E-06	0.008323235
1519.319971	234.682329	5.29645E-06	0.008047007
1519.359971	260.970699	5.88975E-06	0.008948643
1519.399971	262.997697	5.93549E-06	0.009018386
1519.439971	233.227325	5.26362E-06	0.007997748
1519.479971	205.477671	4.63734E-06	0.007046353
1519.519971	169.221958	3.8191E-06	0.005803205
1519.559971	146.488527	3.30604E-06	0.005023729
1519.599971	144.955233	3.27144E-06	0.004971277
1519.639971	149.87427	3.38245E-06	0.005140112
1519.679971	142.941633	3.22599E-06	0.004902478
1519.719971	131.255644	2.96226E-06	0.004501801
1519.759971	129.855246	2.93065E-06	0.004453888
1519.799971	152.969004	3.4523E-06	0.005246801
1519.83997	173.814157	3.92274E-06	0.005961942
1519.87997	170.534422	3.84872E-06	0.005849599
1519.91997	174.527343	3.93884E-06	0.00598672
1519.95997	172.861289	3.90124E-06	0.005929726
1519.99997	167.513161	3.78054E-06	0.005746419
1520.03997	167.540414	3.78115E-06	0.005747505
1520.07997	173.869536	3.92399E-06	0.005964784
1520.11997	186.604801	4.21141E-06	0.006401849
1520.15997	198.37621	4.47707E-06	0.00680587
1520.19997	207.717029	4.68788E-06	0.007126521
1520.23997	217.222608	4.90241E-06	0.007452842
1520.27997	211.980789	4.78411E-06	0.007273188
1520.31997	215.040432	4.85316E-06	0.007378361
1520.35997	215.465336	4.86275E-06	0.007393134
1520.39997	218.075529	4.92166E-06	0.007482893
1520.43997	214.620303	4.84368E-06	0.007364527
1520.47997	199.455267	4.50143E-06	0.00684433
1520.51997	193.590088	4.36906E-06	0.006643241
1520.55997	180.988275	4.08465E-06	0.00621096
1520.59997	149.676188	3.37798E-06	0.005136561
1520.63997	140.149327	3.16298E-06	0.004809746
1520.67997	158.092225	3.56792E-06	0.005425666
1520.71997	172.336471	3.88939E-06	0.005914679
1520.75997	178.867457	4.03679E-06	0.006138988
1520.79997	174.852041	3.94617E-06	0.006001331

1520.83997	165.02888	3.72447E-06	0.005664326
1520.87997	155.017826	3.49854E-06	0.005320854
1520.91997	150.965582	3.40708E-06	0.0051819
1520.95997	150.487307	3.39629E-06	0.005165619
1520.99997	150.810056	3.40357E-06	0.005176834
1521.03997	150.975408	3.4073E-06	0.005182647
1521.07997	170.014325	3.83699E-06	0.005836363
1521.11997	197.236318	4.45135E-06	0.006771036
1521.15997	219.949835	4.96396E-06	0.007550979
1521.19997	208.948127	4.71567E-06	0.007173474
1521.23997	181.744778	4.10173E-06	0.00623971
1521.27997	189.491648	4.27656E-06	0.006505849
1521.31997	224.17667	5.05936E-06	0.007696898
1521.35997	249.110736	5.62208E-06	0.008553211
1521.39997	271.140517	6.11926E-06	0.009309848
1521.43997	245.512674	5.54088E-06	0.008430115
1521.47997	210.601836	4.75299E-06	0.007231579
1521.51997	204.125067	4.60682E-06	0.007009366
1521.55997	173.816888	3.92281E-06	0.005968783
1521.59997	152.309659	3.43742E-06	0.005230373
1521.63997	161.523377	3.64536E-06	0.005546922
1521.67997	154.901093	3.4959E-06	0.005319644
1521.71997	143.369149	3.23564E-06	0.004923741
1521.75997	157.140454	3.54644E-06	0.005396832
1521.79997	167.764184	3.7862E-06	0.005761845
1521.83997	159.100972	3.59069E-06	0.005464451
1521.87997	159.392483	3.59727E-06	0.005474608
1521.91997	162.922455	3.67693E-06	0.005595998
1521.95997	172.359457	3.88991E-06	0.005920292
1521.99997	182.631601	4.12174E-06	0.006273289
1522.03997	205.460953	4.63697E-06	0.00705765
1522.07997	213.292556	4.81372E-06	0.007326861
1522.11997	188.309973	4.24989E-06	0.006468848
1522.15997	176.044113	3.97307E-06	0.006047649
1522.19997	175.129819	3.95244E-06	0.006016398
1522.23997	188.648579	4.25754E-06	0.006480991
1522.27997	198.714981	4.48472E-06	0.006827
1522.31997	198.918979	4.48932E-06	0.006834188
1522.35997	207.112574	4.67424E-06	0.007115879
1522.39997	213.810698	4.82541E-06	0.007346203
1522.43997	199.082446	4.49301E-06	0.006840343
1522.47997	181.408011	4.09413E-06	0.006233225
1522.51997	183.214954	4.13491E-06	0.006295477
1522.55997	175.155694	3.95302E-06	0.00601871
1522.59997	181.722517	4.10122E-06	0.006244524
1522.63997	177.888701	4.0147E-06	0.006112943

1522.67997	167.697266	3.78469E-06	0.005762877
1522.71997	169.575932	3.82709E-06	0.00582759
1522.75997	163.948137	3.70008E-06	0.005634335
1522.79997	175.049898	3.95063E-06	0.006016023
1522.83997	196.886634	4.44346E-06	0.006766674
1522.87997	186.578585	4.21082E-06	0.006412572
1522.91997	171.776906	3.87677E-06	0.005904004
1522.95997	165.175021	3.72777E-06	0.005677245
1522.99997	164.160066	3.70486E-06	0.005642508
1523.03997	168.670061	3.80665E-06	0.005797678
1523.07997	188.481864	4.25377E-06	0.006478837
1523.11997	195.767925	4.41821E-06	0.006729463
1523.15997	190.159913	4.29164E-06	0.006536861
1523.19997	187.503409	4.23169E-06	0.006445711
1523.23997	197.478337	4.45681E-06	0.006788793
1523.27997	221.749942	5.00459E-06	0.007623388
1523.31997	225.123727	5.08073E-06	0.007739576
1523.35997	207.585403	4.68491E-06	0.00713681
1523.39997	195.836612	4.41976E-06	0.006733062
1523.43997	170.899424	3.85696E-06	0.00587585
1523.47997	158.659003	3.58071E-06	0.005455144
1523.51997	167.400369	3.77799E-06	0.005755848
1523.55997	167.747587	3.78583E-06	0.005767938
1523.59997	159.640331	3.60286E-06	0.005489317
1523.63997	144.129191	3.2528E-06	0.004956089
1523.67997	148.409592	3.3494E-06	0.00510341
1523.71997	158.091834	3.56791E-06	0.0054365
1523.75997	170.987307	3.85895E-06	0.005880106
1523.79997	172.895829	3.90202E-06	0.005945895
1523.83997	179.774265	4.05725E-06	0.006182607
1523.87997	173.170922	3.90823E-06	0.005955668
1523.91997	150.948281	3.40669E-06	0.005191527
1523.95997	141.019674	3.18262E-06	0.004850182
1523.99997	131.110593	2.95898E-06	0.004509491
1524.03997	141.274472	3.18837E-06	0.0048592
1524.07997	176.230883	3.97729E-06	0.006061701
1524.11997	183.965344	4.15184E-06	0.006327905
1524.15997	173.895117	3.92457E-06	0.005981673
1524.19997	178.25995	4.02308E-06	0.006131977
1524.23997	201.541071	4.5485E-06	0.006933007
1524.27997	219.83432	4.96135E-06	0.007562493
1524.31997	222.870637	5.02988E-06	0.007667146
1524.35997	217.368328	4.9057E-06	0.007478053
1524.39997	203.103743	4.58377E-06	0.006987497
1524.43997	191.827716	4.32928E-06	0.006599734
1524.47997	186.298908	4.20451E-06	0.006409686

1524.51997	208.497184	4.70549E-06	0.007173615
1524.55997	204.289348	4.61053E-06	0.007029023
1524.59997	169.089135	3.81611E-06	0.005818035
1524.63997	163.163545	3.68237E-06	0.005614294
1524.67997	182.374764	4.11594E-06	0.006275498
1524.71997	186.624634	4.21186E-06	0.006421904
1524.75997	176.128084	3.97497E-06	0.006060868
1524.79997	184.860632	4.17205E-06	0.006361537
1524.83997	209.331312	4.72432E-06	0.007203826
1524.87997	199.409192	4.50039E-06	0.006862551
1524.91997	152.9723	3.45237E-06	0.005264591
1524.95997	124.469593	2.80911E-06	0.004283773
1524.99997	121.131909	2.73378E-06	0.004169012
1525.03997	134.759324	3.04133E-06	0.00463815
1525.07997	157.929599	3.56425E-06	0.005435768
1525.11997	159.242839	3.59389E-06	0.005481112
1525.15997	168.88026	3.81139E-06	0.005812983
1525.19997	183.950124	4.1515E-06	0.006331865
1525.23997	208.845974	4.71336E-06	0.007189009
1525.27997	220.487758	4.9761E-06	0.007589948
1525.31997	202.147055	4.56218E-06	0.00695878
1525.35997	203.484333	4.59236E-06	0.007004999
1525.39997	213.404828	4.81625E-06	0.007346707
1525.43997	212.319112	4.79175E-06	0.007309522
1525.47997	186.018566	4.19818E-06	0.006404239
1525.51997	180.477753	4.07313E-06	0.006213643
1525.55997	179.192072	4.04412E-06	0.006169541
1525.59997	154.126223	3.47841E-06	0.005306669
1525.63997	122.042375	2.75433E-06	0.00420211
1525.67997	120.925287	2.72912E-06	0.004163757
1525.71997	156.966898	3.54252E-06	0.0054049
1525.75997	177.654726	4.00942E-06	0.006117412
1525.79997	169.796326	3.83207E-06	0.005846967
1525.83997	161.966729	3.65536E-06	0.0055775
1525.87997	166.731161	3.76289E-06	0.005741719
1525.91997	170.626267	3.8508E-06	0.005876008
1525.95997	180.942222	4.08361E-06	0.006231431
1525.99997	179.790916	4.05763E-06	0.006191944
1526.03997	173.369932	3.91272E-06	0.005970964
1526.07997	174.60648	3.94063E-06	0.006013709
1526.11997	158.696783	3.58157E-06	0.005465898
1526.15997	148.823457	3.35874E-06	0.005125972
1526.19997	171.489442	3.87028E-06	0.005906818
1526.23997	180.458451	4.0727E-06	0.006215911
1526.27997	199.720243	4.50741E-06	0.006879566
1526.31997	206.253344	4.65485E-06	0.007104792

1526.35997	202.507185	4.57031E-06	0.006975931
1526.39997	199.0398	4.49205E-06	0.006856667
1526.43997	204.189522	4.60827E-06	0.007034252
1526.47997	181.141287	4.08811E-06	0.006240413
1526.51997	150.177236	3.38929E-06	0.00517382
1526.55997	139.248902	3.14265E-06	0.004797449
1526.59997	142.243707	3.21024E-06	0.004900756
1526.63997	147.09463	3.31972E-06	0.005068019
1526.67997	142.983076	3.22693E-06	0.004926488
1526.71997	171.865439	3.87876E-06	0.005921786
1526.75997	178.301712	4.02402E-06	0.006143715
1526.79997	144.047562	3.25095E-06	0.004963555
1526.83997	137.54568	3.10421E-06	0.004739639
1526.87997	164.324379	3.70857E-06	0.005662545
1526.91997	170.777276	3.85421E-06	0.005885063
1526.95997	148.985157	3.36239E-06	0.005134231
1526.99997	134.522773	3.03599E-06	0.004635959
1527.03997	156.730633	3.53719E-06	0.005401434
1527.07997	184.06244	4.15403E-06	0.00634354
1527.11997	179.958527	4.06141E-06	0.006202265
1527.15997	170.638478	3.85107E-06	0.005881204
1527.19997	179.858246	4.05915E-06	0.006199134
1527.23997	180.055853	4.06361E-06	0.006206107
1527.27997	176.397379	3.98104E-06	0.006080167
1527.31997	179.422193	4.04931E-06	0.00618459
1527.35997	193.022974	4.35626E-06	0.006653577
1527.39997	209.248472	4.72245E-06	0.007213065
1527.43997	189.370591	4.27383E-06	0.006528019
1527.47997	173.463966	3.91484E-06	0.00597984
1527.51997	158.875552	3.5856E-06	0.005477075
1527.55997	140.717161	3.17579E-06	0.00485121
1527.59997	145.754739	3.28948E-06	0.005025012
1527.63997	161.665128	3.64856E-06	0.005573681
1527.67997	144.653108	3.26462E-06	0.004987293
1527.71997	128.761416	2.90597E-06	0.004439502
1527.75997	151.252885	3.41357E-06	0.005215111
1527.79997	172.31724	3.88896E-06	0.005941553
1527.83997	155.645672	3.51271E-06	0.005366853
1527.87997	153.86957	3.47262E-06	0.005305749
1527.91997	165.512551	3.73539E-06	0.005707374
1527.95997	163.281119	3.68503E-06	0.005630574
1527.99997	148.41271	3.34947E-06	0.005117987
1528.03997	135.82496	3.06538E-06	0.004684023
1528.07997	160.163682	3.61467E-06	0.005523507
1528.11997	183.268261	4.13611E-06	0.006320471
1528.15997	181.171263	4.08878E-06	0.006248315

1528.19997	183.954835	4.1516E-06	0.006344482
1528.23997	200.169553	4.51755E-06	0.006903897
1528.27997	204.131466	4.60696E-06	0.007040729
1528.31997	206.835146	4.66798E-06	0.007134169
1528.35997	205.612603	4.64039E-06	0.007092186
1528.39997	193.554285	4.36825E-06	0.006676434
1528.43997	194.34745	4.38615E-06	0.006703969
1528.47997	195.626979	4.41503E-06	0.006748283
1528.51997	167.223807	3.77401E-06	0.005768647
1528.55997	161.892374	3.65369E-06	0.005584877
1528.59997	167.470967	3.77959E-06	0.005777476
1528.63997	161.373193	3.64197E-06	0.005567258
1528.67997	167.270496	3.77506E-06	0.005770862
1528.71997	172.80226	3.89991E-06	0.005961865
1528.75997	180.255996	4.06813E-06	0.006219189
1528.79997	173.285766	3.91082E-06	0.005978859
1528.83997	151.491894	3.41896E-06	0.005227044
1528.87997	162.065477	3.65759E-06	0.005592019
1528.91997	178.052974	4.01841E-06	0.006143824
1528.95997	170.428593	3.84634E-06	0.005880894
1528.99997	154.892411	3.49571E-06	0.005344934
1529.03997	155.583178	3.5113E-06	0.005368911
1529.07997	179.844097	4.05883E-06	0.006206277
1529.11997	195.668822	4.41597E-06	0.006752552
1529.15997	194.479391	4.38913E-06	0.00671168
1529.19997	176.681696	3.98746E-06	0.006097623
1529.23997	170.0257	3.83724E-06	0.005868066
1529.27997	186.185523	4.20195E-06	0.006425955
1529.31997	200.721205	4.53E-06	0.006927816
1529.35997	207.319133	4.6789E-06	0.007155729
1529.39997	186.864339	4.21727E-06	0.006449889
1529.43997	190.012819	4.28832E-06	0.006558735
1529.47997	188.061216	4.24428E-06	0.006491541
1529.51997	165.555301	3.73635E-06	0.005714826
1529.55997	155.751564	3.5151E-06	0.00537655
1529.59997	156.589182	3.534E-06	0.005405606
1529.63997	151.517033	3.41953E-06	0.005230647
1529.67997	149.909379	3.38325E-06	0.005175283
1529.71997	149.000783	3.36274E-06	0.005144051
1529.75997	140.213112	3.16441E-06	0.004840795
1529.79997	146.10129	3.2973E-06	0.005044213
1529.83997	150.612667	3.39912E-06	0.005200107
1529.87997	152.341348	3.43813E-06	0.005259929
1529.91997	164.106487	3.70365E-06	0.005666295
1529.95997	189.383122	4.27411E-06	0.006539222
1529.99997	191.978671	4.33269E-06	0.006629017

1530.03997	192.998922	4.35572E-06	0.006664421
1530.07997	180.578498	4.07541E-06	0.006235696
1530.11997	156.622106	3.53474E-06	0.005408581
1530.15997	162.232489	3.66136E-06	0.005602469
1530.19997	163.148522	3.68203E-06	0.00563425
1530.23997	161.950115	3.65499E-06	0.00559301
1530.27997	165.017604	3.72422E-06	0.005699095
1530.31997	176.629813	3.98629E-06	0.006100297
1530.35997	180.17079	4.0662E-06	0.006222755
1530.39997	188.523142	4.2547E-06	0.0065114
1530.43997	177.051713	3.99581E-06	0.006115348
1530.47997	144.520626	3.26163E-06	0.004991858
1530.51997	150.76596	3.40258E-06	0.005207713
1530.55997	163.61319	3.69252E-06	0.005651626
1530.59997	152.912622	3.45102E-06	0.005282139
1530.63997	146.459192	3.30538E-06	0.005059347
1530.67997	153.340671	3.46069E-06	0.005297202
1530.71997	150.320789	3.39253E-06	0.005193015
1530.75997	169.821087	3.83263E-06	0.00586683
1530.79997	180.797352	4.08034E-06	0.006246191
1530.83997	156.430261	3.53041E-06	0.005404497
1530.87997	144.543661	3.26215E-06	0.004993959
1530.91997	155.756358	3.5152E-06	0.005381496
1530.95997	174.830296	3.94568E-06	0.006040672
1530.99997	186.24337	4.20325E-06	0.006435181
1531.03997	173.826277	3.92302E-06	0.006006296
1531.07997	171.101536	3.86152E-06	0.005912301
1531.11997	188.907078	4.26337E-06	0.00652773
1531.15997	199.54231	4.50339E-06	0.006895414
1531.19997	188.212966	4.2477E-06	0.006504085
1531.23997	175.49145	3.9606E-06	0.006064625
1531.27997	173.47909	3.91518E-06	0.005995239
1531.31997	181.687146	4.10043E-06	0.006279064
1531.35997	183.786365	4.1478E-06	0.006351778
1531.39997	206.249317	4.65476E-06	0.007128299
1531.43997	201.875634	4.55605E-06	0.00697732
1531.47997	175.80581	3.96769E-06	0.006076441
1531.51997	164.128532	3.70415E-06	0.005672983
1531.55997	155.943587	3.51943E-06	0.005390217
1531.59997	160.270811	3.61709E-06	0.005539933
1531.63997	161.694044	3.64921E-06	0.005589275
1531.67997	161.434186	3.64334E-06	0.005580438
1531.71997	159.45395	3.59865E-06	0.00551213
1531.75997	156.904227	3.54111E-06	0.00542413
1531.79997	164.209524	3.70598E-06	0.00567682
1531.83997	173.071284	3.90598E-06	0.005983333

1531.87997	169.010451	3.81433E-06	0.005843096
1531.91997	171.031125	3.85993E-06	0.00591311
1531.95997	169.361351	3.82225E-06	0.005855534
1531.99997	157.354432	3.55127E-06	0.005440546
1532.03997	162.104971	3.65848E-06	0.005604943
1532.07997	168.960876	3.81321E-06	0.005842145
1532.11997	172.953663	3.90332E-06	0.00598036
1532.15997	170.023523	3.83719E-06	0.005879195
1532.19997	203.506321	4.59285E-06	0.007037171
1532.23997	216.977734	4.89689E-06	0.007503203
1532.27997	204.403796	4.61311E-06	0.007068574
1532.31997	201.143561	4.53953E-06	0.006956012
1532.35997	191.518756	4.32231E-06	0.006623337
1532.39997	167.851789	3.78818E-06	0.005805009
1532.43997	151.6261	3.42199E-06	0.005243994
1532.47997	162.209786	3.66085E-06	0.005610178
1532.51997	146.104608	3.29738E-06	0.005053297
1532.55997	125.844957	2.84015E-06	0.004352693
1532.59997	128.18314	2.89291E-06	0.004433681
1532.63997	149.730765	3.37921E-06	0.00517912
1532.67997	173.060866	3.90574E-06	0.005986254
1532.71997	175.158736	3.95309E-06	0.006058978
1532.75997	169.581815	3.82723E-06	0.005866218
1532.79997	168.885912	3.81152E-06	0.005842297
1532.83997	168.03418	3.7923E-06	0.005812985
1532.87997	164.435509	3.71108E-06	0.005688641
1532.91997	162.602081	3.6697E-06	0.00562536
1532.95997	170.503454	3.84803E-06	0.005898869
1532.99997	183.084647	4.13197E-06	0.006334303
1533.03997	200.98433	4.53594E-06	0.006953772
1533.07997	211.82974	4.7807E-06	0.007329199
1533.11997	202.92615	4.57976E-06	0.007021322
1533.15997	197.711945	4.46208E-06	0.006841087
1533.19997	190.663015	4.303E-06	0.006597357
1533.23997	192.377887	4.3417E-06	0.006656869
1533.27997	203.743252	4.5982E-06	0.00705033
1533.31997	208.929088	4.71524E-06	0.007229969
1533.35997	203.505508	4.59284E-06	0.007042471
1533.39997	214.317606	4.83685E-06	0.007416825
1533.43997	220.343606	4.97285E-06	0.007625564
1533.47997	192.50293	4.34452E-06	0.006662239
1533.51997	177.342083	4.00236E-06	0.006137705
1533.55997	182.715436	4.12363E-06	0.006323838
1533.59997	172.37851	3.89034E-06	0.00596623
1533.63997	157.845144	3.56235E-06	0.005463355
1533.67997	152.453098	3.44065E-06	0.005276862

1533.71997	148.93743	3.36131E-06	0.005155309
1533.75997	156.172705	3.5246E-06	0.005405891
1533.79997	176.43631	3.98192E-06	0.006107471
1533.83997	160.91696	3.63167E-06	0.005570403
1533.87997	153.874229	3.47273E-06	0.005326746
1533.91997	165.624899	3.73792E-06	0.005733675
1533.95997	161.569178	3.64639E-06	0.005593418
1533.99997	178.704387	4.03311E-06	0.006186789
1534.03997	197.298182	4.45275E-06	0.006830689
1534.07997	198.478925	4.47939E-06	0.006871747
1534.11997	178.925062	4.03809E-06	0.006194914
1534.15997	187.962667	4.24206E-06	0.006507992
1534.19997	203.08023	4.58324E-06	0.007031603
1534.23997	207.973121	4.69366E-06	0.007201206
1534.27997	211.650711	4.77666E-06	0.007328736
1534.31997	215.685818	4.86773E-06	0.007468653
1534.35997	211.768391	4.77932E-06	0.007333194
1534.39997	192.703653	4.34905E-06	0.006673187
1534.43997	165.618209	3.73777E-06	0.005735387
1534.47997	148.181932	3.34426E-06	0.0051317
1534.51997	140.744194	3.1764E-06	0.00487425
1534.55997	141.019041	3.1826E-06	0.004883896
1534.59997	146.733414	3.31157E-06	0.005081933
1534.63997	156.616938	3.53463E-06	0.005424379
1534.67997	159.156899	3.59195E-06	0.005512493
1534.71997	156.648919	3.53535E-06	0.005425769
1534.75997	155.803538	3.51627E-06	0.005396629
1534.79997	147.704036	3.33347E-06	0.005116216
1534.83997	141.639216	3.1966E-06	0.004906269
1534.87997	146.306457	3.30193E-06	0.005068071
1534.91997	139.093728	3.13915E-06	0.004818347
1534.95997	134.289829	3.03073E-06	0.004652056
1534.99997	165.615922	3.73772E-06	0.005737401
1535.03997	184.940966	4.17386E-06	0.006407042
1535.07997	194.278692	4.3846E-06	0.006730711
1535.11997	184.999914	4.17519E-06	0.006409418
1535.15997	171.396584	3.86818E-06	0.005938278
1535.19997	175.634195	3.96382E-06	0.006085255
1535.23997	195.362447	4.40906E-06	0.006768963
1535.27997	222.144661	5.0135E-06	0.007697119
1535.31997	209.627979	4.73101E-06	0.007263616
1535.35997	185.970411	4.19709E-06	0.006444049
1535.39997	192.937263	4.35433E-06	0.006685631
1535.43997	184.518031	4.16431E-06	0.006394056
1535.47997	150.990192	3.40764E-06	0.00523236
1535.51997	134.045622	3.02522E-06	0.00464529

1535.55997	152.649945	3.4451E-06	0.005290152
1535.59997	177.118032	3.99731E-06	0.006138265
1535.63997	183.543623	4.14232E-06	0.006361118
1535.67997	165.298041	3.73055E-06	0.005728926
1535.71997	161.222865	3.63858E-06	0.005587833
1535.75997	161.646128	3.64813E-06	0.005602649
1535.79997	174.853976	3.94621E-06	0.00606059
1535.83997	179.810458	4.05807E-06	0.006232549
1535.87997	182.313498	4.11456E-06	0.006319473
1535.91997	193.830176	4.37448E-06	0.006718847
1535.95997	194.141458	4.3815E-06	0.006729812
1535.99997	178.233253	4.02248E-06	0.006178523
1536.03997	168.722976	3.80784E-06	0.005848998
1536.07997	151.311218	3.41488E-06	0.005245534
1536.11997	164.237515	3.70661E-06	0.005693801
1536.15997	190.57326	4.30097E-06	0.006606983
1536.19997	195.260301	4.40675E-06	0.006769654
1536.23997	182.441726	4.11746E-06	0.0063254
1536.27997	182.37379	4.11592E-06	0.006323209
1536.31997	195.002941	4.40094E-06	0.006761259
1536.35997	209.404575	4.72597E-06	0.00726079
1536.39997	206.68418	4.66457E-06	0.007166651
1536.43997	193.567486	4.36855E-06	0.006712013
1536.47997	167.918809	3.78969E-06	0.005822788
1536.51997	171.491195	3.87032E-06	0.00594682
1536.55997	171.425335	3.86883E-06	0.005944691
1536.59997	169.18372	3.81824E-06	0.005867109
1536.63997	165.917239	3.74452E-06	0.005753981
1536.67997	156.768213	3.53804E-06	0.005436836
1536.71997	149.235214	3.36803E-06	0.00517572
1536.75997	159.162779	3.59208E-06	0.005520168
1536.79997	175.169967	3.95334E-06	0.006075496
1536.83997	179.207693	4.04447E-06	0.0062157
1536.87997	175.709819	3.96553E-06	0.006094537
1536.91997	178.616965	4.03114E-06	0.006195534
1536.95997	184.686399	4.16811E-06	0.006406226
1536.99997	166.656524	3.76121E-06	0.005780973
1537.03997	149.906721	3.38319E-06	0.005200092
1537.07997	161.778569	3.65112E-06	0.005612059
1537.11997	188.09764	4.2451E-06	0.006525231
1537.15997	213.971793	4.82905E-06	0.007423015
1537.19997	220.68941	4.98065E-06	0.007656259
1537.23997	201.932061	4.55733E-06	0.007005703
1537.27997	196.096577	4.42563E-06	0.006803427
1537.31997	195.170437	4.40472E-06	0.006771472
1537.35997	204.850485	4.62319E-06	0.007107507

1537.39997	200.495159	4.5249E-06	0.006956576
1537.43997	187.989789	4.24267E-06	0.006522847
1537.47997	179.568478	4.05261E-06	0.006230807
1537.51997	170.007861	3.83684E-06	0.005899219
1537.55997	169.050358	3.81523E-06	0.005866147
1537.59997	171.575926	3.87223E-06	0.00595394
1537.63997	168.966467	3.81334E-06	0.005863541
1537.67997	171.417451	3.86865E-06	0.00594875
1537.71997	189.707909	4.28144E-06	0.006583661
1537.75997	186.309049	4.20474E-06	0.006465874
1537.79997	168.036816	3.79236E-06	0.005831886
1537.83997	148.091105	3.34221E-06	0.005139784
1537.87997	135.823819	3.06535E-06	0.004714147
1537.91997	137.352803	3.09986E-06	0.004767339
1537.95997	147.882415	3.3375E-06	0.005132942
1537.99997	160.111154	3.61349E-06	0.005557541
1538.03997	150.844936	3.40436E-06	0.005236042
1538.07997	159.078442	3.59018E-06	0.005521982
1538.11997	164.183309	3.70539E-06	0.005699332
1538.15997	160.646733	3.62557E-06	0.005576711
1538.19997	168.359917	3.79965E-06	0.00584462
1538.23997	178.830701	4.03596E-06	0.006208275
1538.27997	175.967508	3.97134E-06	0.006109035
1538.31997	157.602957	3.55688E-06	0.005471618
1538.35997	165.982502	3.74599E-06	0.005762687
1538.39997	192.272547	4.33932E-06	0.006675615
1538.43997	178.760892	4.03438E-06	0.006206658
1538.47997	147.65104	3.33228E-06	0.005126643
1538.51997	145.488958	3.28348E-06	0.005051704
1538.55997	154.477086	3.48633E-06	0.005363932
1538.59997	147.578845	3.33065E-06	0.005124536
1538.63997	138.43099	3.12419E-06	0.004807011
1538.67997	157.306898	3.5502E-06	0.005462618
1538.71997	179.855476	4.05909E-06	0.006245799
1538.75997	190.593959	4.30144E-06	0.006618884
1538.79997	184.998988	4.17517E-06	0.006424751
1538.83997	181.329946	4.09236E-06	0.006297494
1538.87997	160.576946	3.624E-06	0.005576898
1538.91997	151.311854	3.4149E-06	0.005255254
1538.95997	166.660589	3.7613E-06	0.005788486
1538.99997	157.927786	3.56421E-06	0.005485319
1539.03997	156.255087	3.52646E-06	0.005427362
1539.07997	160.175681	3.61494E-06	0.005563685
1539.11997	156.54331	3.53296E-06	0.005437656
1539.15997	165.713576	3.73992E-06	0.005756342
1539.19997	188.815839	4.26131E-06	0.006559009

1539.23997	212.100716	4.78682E-06	0.007368061
1539.27997	229.761485	5.1854E-06	0.007981777
1539.31997	227.87094	5.14273E-06	0.007916307
1539.35997	215.828834	4.87096E-06	0.007498155
1539.39997	197.85178	4.46524E-06	0.006873789
1539.43997	193.381307	4.36435E-06	0.00671865
1539.47997	188.421113	4.2524E-06	0.006546488
1539.51997	164.25386	3.70698E-06	0.005706971
1539.55997	150.316291	3.39243E-06	0.005222848
1539.59997	130.907114	2.95439E-06	0.004548581
1539.63997	136.402706	3.07842E-06	0.004739657
1539.67997	159.882534	3.60833E-06	0.005555667
1539.71997	167.939735	3.79017E-06	0.005835794
1539.75997	174.115184	3.92954E-06	0.006050544
1539.79997	158.396952	3.5748E-06	0.005504475
1539.83997	143.749913	3.24424E-06	0.004995603
1539.87997	141.240661	3.1876E-06	0.004908529
1539.91997	128.81392	2.90715E-06	0.004476779
1539.95997	115.057282	2.59668E-06	0.003998787
1539.99997	98.775046	2.22922E-06	0.003432991
1540.03997	103.502571	2.33591E-06	0.003597393
1540.07997	129.000023	2.91135E-06	0.004483713
1540.11997	174.444647	3.93697E-06	0.00606341
1540.15997	228.10163	5.14794E-06	0.007928645
1540.19997	247.40092	5.58349E-06	0.008599698
1540.23997	216.218662	4.87975E-06	0.007515992
1540.27997	216.340155	4.8825E-06	0.007520411
1540.31997	229.394137	5.17711E-06	0.0079744
1540.35997	219.582856	4.95568E-06	0.00763353
1540.39997	176.905862	3.99252E-06	0.006150076
1540.43997	163.486996	3.68967E-06	0.005683721
1540.47997	175.656823	3.96433E-06	0.006106971
1540.51997	156.782576	3.53836E-06	0.005450921
1540.55997	159.825029	3.60703E-06	0.005556843
1540.59997	176.929948	3.99306E-06	0.006151712
1540.63997	179.862267	4.05924E-06	0.006253829
1540.67997	166.35064	3.7543E-06	0.005784178
1540.71997	159.484312	3.59934E-06	0.005545573
1540.75997	157.803659	3.56141E-06	0.005487276
1540.79997	154.006017	3.4757E-06	0.00535536
1540.83997	159.936083	3.60953E-06	0.005561715
1540.87997	165.132091	3.7268E-06	0.005742553
1540.91997	162.546287	3.66844E-06	0.005652778
1540.95997	169.772503	3.83153E-06	0.005904233
1540.99997	179.197248	4.04423E-06	0.006232162
1541.03997	166.776062	3.7639E-06	0.005800326

1541.07997	156.404837	3.52984E-06	0.005439765
1541.11997	166.258741	3.75223E-06	0.005782634
1541.15997	184.454323	4.16288E-06	0.00641566
1541.19997	207.039904	4.6726E-06	0.007201414
1541.23997	208.336434	4.70186E-06	0.007246699
1541.27997	197.939314	4.46721E-06	0.006885228
1541.31997	191.202734	4.31518E-06	0.006651072
1541.35997	183.536698	4.14217E-06	0.006384571
1541.39997	181.960283	4.10659E-06	0.006329898
1541.43997	176.710933	3.98812E-06	0.006147447
1541.47997	162.245076	3.66165E-06	0.005644353
1541.51997	155.642746	3.51264E-06	0.005414805
1541.55997	128.719554	2.90502E-06	0.004478264
1541.59997	106.817678	2.41073E-06	0.003716375
1541.63997	124.420215	2.80799E-06	0.004328911
1541.67997	130.234546	2.93921E-06	0.004531325
1541.71997	131.30162	2.96329E-06	0.004568571
1541.75997	140.03316	3.16035E-06	0.004872506
1541.79997	153.709977	3.46902E-06	0.005348535
1541.83997	170.211876	3.84144E-06	0.005922893
1541.87997	163.51143	3.69023E-06	0.005689884
1541.91997	150.882247	3.4052E-06	0.005250549
1541.95997	137.305275	3.09879E-06	0.004778208
1541.99997	121.700721	2.74662E-06	0.004235281
1542.03997	134.346016	3.032E-06	0.004675469
1542.07997	165.085115	3.72574E-06	0.005745391
1542.11997	181.969955	4.10681E-06	0.006333191
1542.15997	174.225602	3.93203E-06	0.006063818
1542.19997	181.951715	4.1064E-06	0.006332885
1542.23997	206.371836	4.65752E-06	0.007183021
1542.27997	199.153637	4.49462E-06	0.006931963
1542.31997	176.979306	3.99418E-06	0.006160298
1542.35997	159.686114	3.60389E-06	0.005558501
1542.39997	170.06409	3.83811E-06	0.0059199
1542.43997	169.743219	3.83087E-06	0.005908884
1542.47997	156.029414	3.52137E-06	0.005431637
1542.51997	142.919837	3.2255E-06	0.004975401
1542.55997	151.145637	3.41115E-06	0.005261898
1542.59997	159.302358	3.59523E-06	0.005546005
1542.63997	175.361335	3.95766E-06	0.006105246
1542.67997	198.007621	4.46876E-06	0.006893861
1542.71997	183.621932	4.14409E-06	0.006393172
1542.75997	159.883431	3.60835E-06	0.005566812
1542.79997	179.469785	4.05038E-06	0.006248931
1542.83997	180.217871	4.06727E-06	0.006275141
1542.87997	159.557604	3.60099E-06	0.0055559

1542.91997	138.209557	3.1192E-06	0.004812672
1542.95997	151.873465	3.42757E-06	0.005288607
1542.99997	173.834956	3.92321E-06	0.006053517
1543.03997	168.540128	3.80372E-06	0.005869286
1543.07997	170.613091	3.8505E-06	0.005941629
1543.11997	181.119393	4.08761E-06	0.006307676
1543.15997	174.526403	3.93882E-06	0.006078226
1543.19997	172.831316	3.90056E-06	0.006019347
1543.23997	203.54598	4.59375E-06	0.007089257
1543.27997	223.903853	5.0532E-06	0.007798499
1543.31997	203.611014	4.59522E-06	0.00709189
1543.35997	182.393017	4.11636E-06	0.00635302
1543.39997	181.881694	4.10482E-06	0.006335374
1543.43997	179.659477	4.05466E-06	0.006258131
1543.47997	164.919236	3.722E-06	0.005744829
1543.51997	141.411091	3.19145E-06	0.004926069
1543.55997	139.822036	3.15559E-06	0.00487084
1543.59997	139.58013	3.15013E-06	0.004862539
1543.63997	120.294937	2.71489E-06	0.004190811
1543.67997	96.435357	2.17641E-06	0.003359683
1543.71997	86.836951	1.95979E-06	0.003025365
1543.75997	102.163812	2.30569E-06	0.00355944
1543.79997	136.858216	3.0887E-06	0.004768334
1543.83997	151.144601	3.41112E-06	0.005266228
1543.87997	154.851082	3.49477E-06	0.00539551
1543.91997	153.545857	3.46532E-06	0.005350171
1543.95997	152.748811	3.44733E-06	0.005322536
1543.99997	151.768362	3.4252E-06	0.00528851
1544.03997	149.771296	3.38013E-06	0.005219055
1544.07997	164.464728	3.71174E-06	0.005731223
1544.11997	176.26524	3.97806E-06	0.006142603
1544.15997	164.418006	3.71069E-06	0.005729892
1544.19997	153.630756	3.46723E-06	0.0053541
1544.23997	155.801776	3.51623E-06	0.005429901
1544.27997	166.235521	3.7517E-06	0.005793682
1544.31997	177.903872	4.01504E-06	0.00620051
1544.35997	166.410564	3.75565E-06	0.005800083
1544.39997	162.376786	3.66462E-06	0.005659636
1544.43997	165.254773	3.72957E-06	0.005760097
1544.47997	145.529005	3.28439E-06	0.00507267
1544.51997	124.467125	2.80905E-06	0.004338633
1544.55997	128.157606	2.89234E-06	0.004467391
1544.59997	135.602434	3.06036E-06	0.004727029
1544.63997	140.022576	3.16011E-06	0.004881239
1544.67997	138.79952	3.13251E-06	0.004838728
1544.71997	142.139542	3.20789E-06	0.004955294

1544.75997	166.708868	3.76239E-06	0.005811985
1544.79997	183.574298	4.14302E-06	0.006400131
1544.83997	197.295135	4.45268E-06	0.006878672
1544.87997	184.767444	4.16994E-06	0.006442063
1544.91997	187.917265	4.24103E-06	0.006552053
1544.95997	205.302158	4.63338E-06	0.007158392
1544.99997	187.979584	4.24244E-06	0.006554566
1545.03997	161.684332	3.64899E-06	0.005637836
1545.07997	154.254269	3.4813E-06	0.005378893
1545.11997	150.028175	3.38593E-06	0.005231663
1545.15997	175.890252	3.9696E-06	0.006133664
1545.19997	193.06846	4.35729E-06	0.006732879
1545.23997	203.992903	4.60384E-06	0.007114031
1545.27997	215.185913	4.85645E-06	0.007504569
1545.31997	215.248713	4.85786E-06	0.007506954
1545.35997	196.775016	4.44094E-06	0.006862848
1545.39997	171.892307	3.87937E-06	0.005995178
1545.43997	153.23734	3.45835E-06	0.005344677
1545.47997	136.334727	3.07688E-06	0.004755264
1545.51997	114.562652	2.58552E-06	0.003995972
1545.55997	105.360187	2.37783E-06	0.003675083
1545.59997	118.138237	2.66622E-06	0.004120903
1545.63997	133.493508	3.01276E-06	0.004656646
1545.67997	146.349092	3.3029E-06	0.005105219
1545.71997	150.592536	3.39866E-06	0.005253382
1545.75997	167.89401	3.78913E-06	0.005857092
1545.79997	177.199359	3.99914E-06	0.006181875
1545.83997	155.303966	3.50499E-06	0.00541816
1545.87997	138.699624	3.13026E-06	0.004839002
1545.91997	157.927017	3.56419E-06	0.005509957
1545.95997	163.315972	3.68581E-06	0.005698121
1545.99997	153.74369	3.46978E-06	0.005364281
1546.03997	167.213651	3.77378E-06	0.005834413
1546.07997	185.237362	4.18055E-06	0.006463463
1546.11997	190.018737	4.28846E-06	0.006630471
1546.15997	175.349921	3.9574E-06	0.006118779
1546.19997	166.691276	3.76199E-06	0.005816789
1546.23997	175.744353	3.96631E-06	0.00613286
1546.27997	164.670996	3.7164E-06	0.005746587
1546.31997	156.925665	3.54159E-06	0.005476437
1546.35997	167.546493	3.78129E-06	0.005847237
1546.39997	185.9915	4.19757E-06	0.006491121
1546.43997	190.208853	4.29275E-06	0.006638478
1546.47997	191.497208	4.32183E-06	0.006683616
1546.51997	175.924006	3.97036E-06	0.006140241
1546.55997	150.125952	3.38813E-06	0.005239952

1546.59997	123.072103	2.77757E-06	0.004295783
1546.63997	114.497358	2.58405E-06	0.003996589
1546.67997	124.28205	2.80487E-06	0.004338241
1546.71997	137.301378	3.0987E-06	0.004792822
1546.75997	142.606001	3.21842E-06	0.004978121
1546.79997	158.082421	3.5677E-06	0.005518518
1546.83997	148.593018	3.35354E-06	0.005187386
1546.87997	130.271401	2.94004E-06	0.004547895
1546.91997	123.808951	2.7942E-06	0.004322397
1546.95997	137.868479	3.1115E-06	0.004813365
1546.99997	166.257775	3.75221E-06	0.005804663
1547.03997	186.933457	4.21883E-06	0.006526695
1547.07997	186.093196	4.19986E-06	0.006497526
1547.11997	172.135771	3.88486E-06	0.006010352
1547.15997	161.412715	3.64286E-06	0.005636087
1547.19997	161.641833	3.64803E-06	0.005644233
1547.23997	180.065027	4.06382E-06	0.0062877
1547.27997	200.023716	4.51426E-06	0.006984819
1547.31997	213.696982	4.82284E-06	0.007462482
1547.35997	214.610701	4.84346E-06	0.007494583
1547.39997	206.074072	4.6508E-06	0.007196655
1547.43997	188.438299	4.25279E-06	0.006580937
1547.47997	160.073258	3.61263E-06	0.005590473
1547.51997	136.515535	3.08097E-06	0.004767856
1547.55997	132.951489	3.00053E-06	0.0046435
1547.59997	127.242203	2.87168E-06	0.004444211
1547.63997	126.37288	2.85206E-06	0.004413962
1547.67997	142.1412	3.20793E-06	0.004964847
1547.71997	162.11883	3.6588E-06	0.005662792
1547.75997	167.627858	3.78313E-06	0.005855373
1547.79997	175.112182	3.95204E-06	0.006116964
1547.83997	167.22805	3.7741E-06	0.005841709
1547.87997	156.442787	3.5307E-06	0.005465093
1547.91997	152.030387	3.43111E-06	0.00531109
1547.95997	157.419574	3.55274E-06	0.0054995
1547.99997	157.487733	3.55428E-06	0.005502023
1548.03997	149.941908	3.38398E-06	0.005238536
1548.07997	146.627132	3.30917E-06	0.00512286
1548.11997	144.336029	3.25746E-06	0.005042944
1548.15997	169.06488	3.81556E-06	0.005907095
1548.19997	187.976586	4.24237E-06	0.006568037
1548.23997	194.757001	4.39539E-06	0.006805125
1548.27997	193.733488	4.37229E-06	0.006769537
1548.31997	184.272473	4.15877E-06	0.006439111
1548.35997	181.586415	4.09815E-06	0.006345415
1548.39997	189.426718	4.2751E-06	0.00661956

1548.43997	197.873511	4.46573E-06	0.006914914
1548.47997	190.803975	4.30618E-06	0.006668033
1548.51997	169.629185	3.82829E-06	0.00592819
1548.55997	178.015248	4.01756E-06	0.006221427
1548.59997	195.101519	4.40317E-06	0.006818748
1548.63997	196.549937	4.43586E-06	0.006869547
1548.67997	194.62273	4.39236E-06	0.006802366
1548.71997	168.950692	3.81298E-06	0.005905241
1548.75997	158.108226	3.56828E-06	0.005526413
1548.79997	151.991593	3.43024E-06	0.005312753
1548.83997	151.390278	3.41667E-06	0.005291872
1548.87997	157.453917	3.55352E-06	0.005503969
1548.91997	158.599567	3.57937E-06	0.00554416
1548.95997	142.749571	3.22166E-06	0.004990221
1548.99997	131.334171	2.96403E-06	0.004591281
1549.03997	131.857564	2.97584E-06	0.004609698
1549.07997	126.50961	2.85515E-06	0.004422849
1549.11997	136.578958	3.0824E-06	0.004775003
1549.15997	156.143302	3.52394E-06	0.005459142
1549.19997	185.901358	4.19553E-06	0.006499722
1549.23997	220.189842	4.96938E-06	0.007698759
1549.27997	248.690884	5.61261E-06	0.008695499
1549.31997	238.433704	5.38112E-06	0.008337071
1549.35997	237.886451	5.36877E-06	0.008318151
1549.39997	259.235569	5.85059E-06	0.009064897
1549.43997	270.498514	6.10477E-06	0.009458982
1549.47997	245.822068	5.54786E-06	0.008596301
1549.51997	198.062998	4.47001E-06	0.006926364
1549.55997	190.682945	4.30345E-06	0.006668451
1549.59997	177.521941	4.00642E-06	0.006208353
1549.63997	152.887336	3.45045E-06	0.005346962
1549.67997	146.167897	3.29881E-06	0.005112093
1549.71997	143.216156	3.23219E-06	0.005008988
1549.75997	151.605992	3.42154E-06	0.00530256
1549.79997	178.459506	4.02758E-06	0.006241947
1549.83997	238.282112	5.3777E-06	0.008334567
1549.87997	286.067257	6.45614E-06	0.010006241
1549.91997	255.954756	5.77654E-06	0.008953178
1549.95997	199.705054	4.50706E-06	0.00698577
1549.99997	163.074444	3.68036E-06	0.005704563
1550.03997	157.152833	3.54672E-06	0.005497559
1550.07997	166.527874	3.7583E-06	0.005825669
1550.11997	160.34663	3.6188E-06	0.005609574
1550.15997	172.139349	3.88495E-06	0.006022287
1550.19997	200.463439	4.52418E-06	0.007013385
1550.23997	219.596417	4.95599E-06	0.007682966

1550.27997	230.419141	5.20024E-06	0.008061826
1550.31997	248.254264	5.60275E-06	0.00868606
1550.35997	262.955373	5.93454E-06	0.009200668
1550.39997	269.064161	6.0724E-06	0.009414654
1550.43997	255.565047	5.76775E-06	0.008942546
1550.47997	226.452746	5.11072E-06	0.007924074
1550.51997	188.695526	4.2586E-06	0.006603037
1550.55997	155.466723	3.50867E-06	0.005440399
1550.59997	132.871991	2.99874E-06	0.00464984
1550.63997	144.89789	3.27014E-06	0.005070815
1550.67997	173.930382	3.92537E-06	0.006086987
1550.71997	173.801085	3.92245E-06	0.006082619
1550.75997	161.311216	3.64057E-06	0.005645649
1550.79997	166.679616	3.76173E-06	0.005833686
1550.83997	192.478595	4.34397E-06	0.006736808
1550.87997	207.308403	4.67866E-06	0.007256043
1550.91997	187.009702	4.22055E-06	0.006545733
1550.95997	161.531577	3.64554E-06	0.005654091
1550.99997	160.396238	3.61992E-06	0.005614495
1551.03997	165.132608	3.72681E-06	0.005780436
1551.07997	168.143926	3.79477E-06	0.005885998
1551.11997	166.947417	3.76777E-06	0.005844264
1551.15997	178.959189	4.03886E-06	0.006264917
1551.19997	195.738083	4.41754E-06	0.006852482
1551.23997	207.607071	4.6854E-06	0.007268184
1551.27997	209.637391	4.73122E-06	0.007339453
1551.31997	208.184367	4.69843E-06	0.00728877
1551.35997	212.994452	4.80699E-06	0.007457369
1551.39997	223.08196	5.03465E-06	0.007810754
1551.43997	212.526535	4.79643E-06	0.00744137
1551.47997	178.643122	4.03173E-06	0.006255143
1551.51997	173.195645	3.90878E-06	0.006064557
1551.55997	159.711324	3.60446E-06	0.005592539
1551.59997	125.818965	2.83956E-06	0.004405859
1551.63997	115.456153	2.60568E-06	0.004043084
1551.67997	134.467067	3.03473E-06	0.004708937
1551.71997	157.309287	3.55025E-06	0.005508996
1551.75997	167.088454	3.77095E-06	0.005851615
1551.79997	173.065257	3.90584E-06	0.006061085
1551.83997	173.431169	3.9141E-06	0.006074057
1551.87997	182.942307	4.12875E-06	0.006407329
1551.91997	178.522439	4.029E-06	0.00625269
1551.95997	176.692689	3.98771E-06	0.006188763
1551.99997	181.683143	4.10034E-06	0.006363721
1552.03997	190.228696	4.2932E-06	0.006663213
1552.07997	214.426125	4.8393E-06	0.007510979

1552.11997	225.586139	5.09116E-06	0.007902099
1552.15997	205.092851	4.62866E-06	0.007184421
1552.19997	176.851906	3.9913E-06	0.006195298
1552.23997	167.203096	3.77354E-06	0.005857441
1552.27997	182.755752	4.12454E-06	0.006402445
1552.31997	199.975872	4.51318E-06	0.007005895
1552.35997	216.984745	4.89704E-06	0.007601974
1552.39997	221.955073	5.00922E-06	0.007776308
1552.43997	199.354392	4.49915E-06	0.006984662
1552.47997	171.967436	3.88107E-06	0.006025276
1552.51997	165.12452	3.72663E-06	0.005785668
1552.55997	149.958461	3.38435E-06	0.005254412
1552.59997	156.394809	3.52961E-06	0.005480077
1552.63997	172.995508	3.90427E-06	0.006061922
1552.67997	162.973893	3.67809E-06	0.005710903
1552.71997	160.208957	3.61569E-06	0.005614159
1552.75997	164.393444	3.71013E-06	0.005760943
1552.79997	173.824015	3.92297E-06	0.006091581
1552.83997	177.982438	4.01682E-06	0.006237472
1552.87997	180.586092	4.07558E-06	0.006328881
1552.91997	193.667003	4.37079E-06	0.006787494
1552.95997	187.540587	4.23253E-06	0.006572949
1552.99997	160.399018	3.61998E-06	0.005621833
1553.03997	143.559114	3.23993E-06	0.00503174
1553.07997	162.127128	3.65898E-06	0.005682694
1553.11997	184.136274	4.1557E-06	0.006454299
1553.15997	179.622537	4.05383E-06	0.006296247
1553.19997	165.521698	3.73559E-06	0.005802125
1553.23997	170.538844	3.84882E-06	0.005978148
1553.27997	193.335591	4.36331E-06	0.00677745
1553.31997	207.964516	4.69347E-06	0.00729046
1553.35997	210.754117	4.75643E-06	0.007388443
1553.39997	205.65942	4.64145E-06	0.007210023
1553.43997	196.401434	4.43251E-06	0.006885633
1553.47997	171.390199	3.86804E-06	0.00600892
1553.51997	152.513183	3.44201E-06	0.005347231
1553.55997	140.380136	3.16818E-06	0.004921964
1553.59997	139.408949	3.14627E-06	0.004888038
1553.63997	134.101766	3.02649E-06	0.004702076
1553.67997	130.051449	2.93508E-06	0.004560175
1553.71997	142.687672	3.22026E-06	0.005003385
1553.75997	156.02768	3.52133E-06	0.005471298
1553.79997	162.272529	3.66226E-06	0.005690427
1553.83997	164.85049	3.72045E-06	0.005780978
1553.87997	165.717631	3.74002E-06	0.005811536
1553.91997	160.47071	3.6216E-06	0.005627677

1553.95997	152.85224	3.44966E-06	0.005360637
1553.99997	139.78256	3.1547E-06	0.0049024
1554.03997	139.509502	3.14854E-06	0.004892949
1554.07997	146.422291	3.30455E-06	0.005135531
1554.11997	162.039799	3.65701E-06	0.005683436
1554.15997	169.66135	3.82902E-06	0.00595091
1554.19997	167.561124	3.78162E-06	0.005877396
1554.23997	195.598711	4.41439E-06	0.006861022
1554.27997	198.147573	4.47191E-06	0.006950607
1554.31997	176.824171	3.99068E-06	0.006202786
1554.35997	165.069112	3.72538E-06	0.005790581
1554.39997	171.950337	3.88068E-06	0.006032128
1554.43997	169.966262	3.8359E-06	0.005962679
1554.47997	164.525373	3.71311E-06	0.005771953
1554.51997	150.385294	3.39399E-06	0.00527602
1554.55997	138.076731	3.1162E-06	0.004844319
1554.59997	143.988074	3.24961E-06	0.005051844
1554.63997	152.745176	3.44725E-06	0.005359226
1554.67997	143.668991	3.24241E-06	0.005040908
1554.71997	126.146883	2.84696E-06	0.004426225
1554.75997	127.954335	2.88775E-06	0.00448976
1554.79997	136.260055	3.0752E-06	0.00478132
1554.83997	157.450482	3.55344E-06	0.005525028
1554.87997	187.31672	4.22748E-06	0.00657322
1554.91997	181.023719	4.08545E-06	0.006352553
1554.95997	141.223457	3.18722E-06	0.004955994
1554.99997	137.344699	3.09968E-06	0.00482
1555.03997	144.02087	3.25035E-06	0.005054425
1555.07997	150.916684	3.40598E-06	0.00529657
1555.11997	162.125138	3.65894E-06	0.005690088
1555.15997	156.506481	3.53213E-06	0.005493032
1555.19997	160.301642	3.61778E-06	0.005626379
1555.23997	155.848909	3.51729E-06	0.005470234
1555.27997	153.944514	3.47431E-06	0.00540353
1555.31997	156.409294	3.52994E-06	0.005490186
1555.35997	166.524742	3.75823E-06	0.005845403
1555.39997	177.873368	4.01435E-06	0.006243926
1555.43997	164.833709	3.72007E-06	0.005786341
1555.47997	151.616532	3.42177E-06	0.005322501
1555.51997	146.785007	3.31273E-06	0.005153023
1555.55997	148.269511	3.34624E-06	0.005205271
1555.59997	131.102663	2.9588E-06	0.004602716
1555.63997	117.51595	2.65217E-06	0.004125824
1555.67997	132.575789	2.99205E-06	0.004654674
1555.71997	165.026762	3.72442E-06	0.005794161
1555.75997	179.182572	4.0439E-06	0.006291339

1555.79997	178.045763	4.01824E-06	0.006251585
1555.83997	186.458407	4.20811E-06	0.00654714
1555.87997	191.126924	4.31347E-06	0.006711239
1555.91997	182.901699	4.12784E-06	0.006422583
1555.95997	173.360565	3.91251E-06	0.006087703
1555.99997	160.810191	3.62926E-06	0.005647132
1556.03997	147.839009	3.33652E-06	0.005191759
1556.07997	153.790394	3.47083E-06	0.005400897
1556.11997	162.395997	3.66505E-06	0.00570326
1556.15997	169.340995	3.82179E-06	0.005947317
1556.19997	173.379235	3.91293E-06	0.006089298
1556.23997	181.089673	4.08694E-06	0.006360262
1556.27997	187.92955	4.24131E-06	0.006600663
1556.31997	182.740806	4.12421E-06	0.006418583
1556.35997	187.673254	4.23552E-06	0.006592
1556.39997	189.476784	4.27623E-06	0.00665552
1556.43997	185.828721	4.1939E-06	0.006527546
1556.47997	172.693511	3.89745E-06	0.006066306
1556.51997	143.531785	3.23931E-06	0.005042055
1556.55997	133.203769	3.00622E-06	0.004679367
1556.59997	144.410048	3.25913E-06	0.005073167
1556.63997	149.969657	3.38461E-06	0.005268613
1556.67997	139.081554	3.13888E-06	0.004886227
1556.71997	146.23988	3.30043E-06	0.005137846
1556.75997	154.61085	3.48935E-06	0.005432083
1556.79997	148.396038	3.34909E-06	0.005213866
1556.83997	160.672177	3.62615E-06	0.005645331
1556.87997	179.169851	4.04361E-06	0.006295422
1556.91997	182.291022	4.11405E-06	0.006405254
1556.95997	167.895545	3.78917E-06	0.005899584
1556.99997	145.205227	3.27708E-06	0.005102413
1557.03997	144.409614	3.25912E-06	0.005074586
1557.07997	167.519724	3.78069E-06	0.005886832
1557.11997	183.460476	4.14045E-06	0.006447173
1557.15997	190.733072	4.30458E-06	0.006702919
1557.19997	191.958197	4.33223E-06	0.006746147
1557.23997	177.428443	4.00431E-06	0.006235676
1557.27997	159.026194	3.589E-06	0.005589077
1557.31997	137.678949	3.10722E-06	0.004838939
1557.35997	131.902829	2.97686E-06	0.004636047
1557.39997	137.837459	3.1108E-06	0.004844759
1557.43997	142.636098	3.2191E-06	0.005013552
1557.47997	155.095408	3.50029E-06	0.005451627
1557.51997	156.163847	3.5244E-06	0.005489324
1557.55997	141.271343	3.1883E-06	0.004965964
1557.59997	137.036193	3.09272E-06	0.004817214

1557.63997	126.761922	2.86084E-06	0.004456159
1557.67997	111.728091	2.52155E-06	0.003927764
1557.71997	121.075052	2.7325E-06	0.004256462
1557.75997	142.788087	3.22253E-06	0.005019925
1557.79997	146.954501	3.31656E-06	0.005166535
1557.83997	145.493588	3.28359E-06	0.005115304
1557.87997	137.526738	3.10379E-06	0.004835327
1557.91997	146.409222	3.30425E-06	0.005147761
1557.95997	149.364309	3.37094E-06	0.005251796
1557.99997	138.54679	3.12681E-06	0.004871567
1558.03997	140.571619	3.17251E-06	0.004942891
1558.07997	139.320619	3.14427E-06	0.004899028
1558.11997	137.347072	3.09973E-06	0.004829754
1558.15997	125.230718	2.82628E-06	0.004403801
1558.19997	125.29942	2.82783E-06	0.00440633
1558.23997	145.452993	3.28267E-06	0.00511519
1558.27997	163.162676	3.68235E-06	0.005738139
1558.31997	171.90764	3.87972E-06	0.006045839
1558.35997	178.436095	4.02705E-06	0.0062756
1558.39997	179.171243	4.04365E-06	0.006301617
1558.43997	178.255197	4.02297E-06	0.00626956
1558.47997	154.989918	3.49791E-06	0.005451417
1558.51997	142.193671	3.20911E-06	0.005001467
1558.55997	157.56566	3.55604E-06	0.005542298
1558.59997	153.747885	3.46988E-06	0.005408148
1558.63997	136.658423	3.08419E-06	0.004807142
1558.67997	124.585742	2.81173E-06	0.004382582
1558.71997	133.83946	3.02057E-06	0.004708223
1558.75997	152.574826	3.4434E-06	0.005367436
1558.79997	145.683151	3.28787E-06	0.005125125
1558.83997	156.506819	3.53214E-06	0.005506042
1558.87997	166.987112	3.76867E-06	0.005874899
1558.91997	159.221189	3.5934E-06	0.005601824
1558.95997	155.250029	3.50378E-06	0.005462248
1558.99997	154.440959	3.48552E-06	0.005433921
1559.03997	159.366536	3.59668E-06	0.005607369
1559.07997	160.13238	3.61396E-06	0.00563446
1559.11997	147.983734	3.33979E-06	0.005207128
1559.15997	135.510969	3.05829E-06	0.004768369
1559.19997	139.741495	3.15377E-06	0.004917359
1559.23997	155.461862	3.50856E-06	0.005470683
1559.27997	162.6834	3.67154E-06	0.005724955
1559.31997	162.376341	3.66461E-06	0.005714296
1559.35997	167.457771	3.77929E-06	0.005893271
1559.39997	183.350161	4.13796E-06	0.006452731
1559.43997	190.302548	4.29486E-06	0.006697582

1559.47997	173.931706	3.9254E-06	0.006121577
1559.51997	154.031667	3.47628E-06	0.005421328
1559.55997	152.153385	3.43389E-06	0.005355357
1559.59997	143.110485	3.2298E-06	0.005037203
1559.63997	131.096157	2.95866E-06	0.004614441
1559.67997	129.838523	2.93027E-06	0.004570291
1559.71997	134.313563	3.03127E-06	0.004727932
1559.75997	137.408452	3.10112E-06	0.004836999
1559.79997	133.000911	3.00165E-06	0.004681966
1559.83997	133.2189	3.00657E-06	0.00468976
1559.87997	126.890656	2.86375E-06	0.004467099
1559.91997	131.814784	2.97488E-06	0.004640569
1559.95997	126.32702	2.85102E-06	0.004447485
1559.99997	131.351795	2.96443E-06	0.004624506
1560.03997	150.33248	3.39279E-06	0.005292895
1560.07997	142.563023	3.21745E-06	0.005019478
1560.11997	140.208492	3.16431E-06	0.004936704
1560.15997	153.526531	3.46488E-06	0.005405767
1560.19997	146.599462	3.30855E-06	0.005161993
1560.23997	144.338624	3.25752E-06	0.005082516
1560.27997	171.402048	3.86831E-06	0.00603564
1560.31997	198.119158	4.47127E-06	0.006976617
1560.35997	205.940497	4.64779E-06	0.007252226
1560.39997	200.18892	4.51799E-06	0.007049864
1560.43997	192.355421	4.34119E-06	0.006774173
1560.47997	175.088565	3.95151E-06	0.006166244
1560.51997	151.625697	3.42198E-06	0.005340069
1560.55997	124.093392	2.80062E-06	0.004370528
1560.59997	113.510164	2.56177E-06	0.003997892
1560.63997	133.313404	3.0087E-06	0.004695494
1560.67997	161.385172	3.64224E-06	0.005684369
1560.71997	185.232677	4.18044E-06	0.006524502
1560.75997	181.386838	4.09365E-06	0.006389202
1560.79997	181.418423	4.09436E-06	0.006390479
1560.83997	178.374671	4.02567E-06	0.006283423
1560.87997	178.109131	4.01968E-06	0.00627423
1560.91997	167.167643	3.77274E-06	0.005888947
1560.95997	159.006226	3.58855E-06	0.005601581
1560.99997	171.017954	3.85964E-06	0.006024893
1561.03997	172.763049	3.89902E-06	0.006086528
1561.07997	164.319894	3.70847E-06	0.00578922
1561.11997	171.444722	3.86927E-06	0.006040392
1561.15997	171.844254	3.87829E-06	0.006054624
1561.19997	159.409913	3.59766E-06	0.005616666
1561.23997	162.565342	3.66887E-06	0.005727992
1561.27997	181.806226	4.10311E-06	0.006406109

1561.31997	186.792909	4.21566E-06	0.006581988
1561.35997	190.955195	4.30959E-06	0.006728826
1561.39997	193.14063	4.35891E-06	0.00680601
1561.43997	182.947383	4.12887E-06	0.006446979
1561.47997	163.560471	3.69133E-06	0.005763941
1561.51997	143.417355	3.23673E-06	0.005054218
1561.55997	133.163569	3.00532E-06	0.004692982
1561.59997	124.650819	2.8132E-06	0.004393086
1561.63997	133.973543	3.0236E-06	0.004721769
1561.67997	137.278448	3.09818E-06	0.004838371
1561.71997	148.253481	3.34587E-06	0.005225319
1561.75997	158.015536	3.56619E-06	0.005569534
1561.79997	158.800525	3.58391E-06	0.005597345
1561.83997	150.097101	3.38748E-06	0.005290706
1561.87997	159.065767	3.58989E-06	0.005606982
1561.91997	173.000758	3.90439E-06	0.006098339
1561.95997	171.39074	3.86805E-06	0.00604174
1561.99997	158.584272	3.57903E-06	0.005590439
1562.03997	141.312235	3.18922E-06	0.00498169
1562.07997	141.970525	3.20408E-06	0.005005025
1562.11997	154.452938	3.48579E-06	0.005445219
1562.15997	169.982607	3.83627E-06	0.005992868
1562.19997	159.51912	3.60012E-06	0.005624114
1562.23997	159.255435	3.59417E-06	0.005614961
1562.27997	187.481307	4.23119E-06	0.006610306
1562.31997	215.014801	4.85258E-06	0.00758129
1562.35997	219.016382	4.94289E-06	0.007722581
1562.39997	204.387638	4.61274E-06	0.007206952
1562.43997	178.474505	4.02792E-06	0.006293385
1562.47997	162.190175	3.66041E-06	0.005719312
1562.51997	152.996657	3.45292E-06	0.005395259
1562.55997	139.237927	3.14241E-06	0.004910198
1562.59997	151.758069	3.42497E-06	0.005351855
1562.63997	171.548989	3.87162E-06	0.006049951
1562.67997	193.847637	4.37487E-06	0.006836524
1562.71997	198.144695	4.47185E-06	0.006988249
1562.75997	153.431252	3.46273E-06	0.005411415
1562.79997	120.464497	2.71872E-06	0.004248809
1562.83997	133.969923	3.02351E-06	0.004725269
1562.87997	141.686814	3.19767E-06	0.004997581
1562.91997	138.443565	3.12448E-06	0.00488331
1562.95997	140.452608	3.16982E-06	0.004954301
1562.99997	148.40665	3.34933E-06	0.005235005
1563.03997	156.744649	3.53751E-06	0.005529267
1563.07997	162.334396	3.66366E-06	0.005726595
1563.11997	164.679174	3.71658E-06	0.00580946

1563.15997	174.916242	3.94762E-06	0.006170755
1563.19997	173.025523	3.90495E-06	0.00610421
1563.23997	168.243607	3.79702E-06	0.005935659
1563.27997	173.606292	3.91805E-06	0.006125013
1563.31997	170.57502	3.84964E-06	0.00601822
1563.35997	166.760623	3.76355E-06	0.005883791
1563.39997	150.168634	3.3891E-06	0.005298514
1563.43997	139.16682	3.1408E-06	0.004910454
1563.47997	152.095535	3.43258E-06	0.005366777
1563.51997	173.516982	3.91604E-06	0.006122801
1563.55997	162.838594	3.67504E-06	0.005746146
1563.59997	133.593185	3.01501E-06	0.004714273
1563.63997	115.640433	2.60984E-06	0.004080856
1563.67997	122.222196	2.75838E-06	0.004313231
1563.71997	141.350012	3.19007E-06	0.004988381
1563.75997	161.629905	3.64776E-06	0.005704224
1563.79997	164.317481	3.70842E-06	0.005799222
1563.83997	157.54047	3.55547E-06	0.005560184
1563.87997	167.558741	3.78157E-06	0.005913918
1563.91997	173.940731	3.9256E-06	0.006139324
1563.95997	165.609538	3.73758E-06	0.00584542
1563.99997	143.963358	3.24905E-06	0.005081518
1564.03997	155.259353	3.50399E-06	0.005480376
1564.07997	172.994711	3.90425E-06	0.006106559
1564.11997	161.145133	3.63682E-06	0.005688425
1564.15997	135.674131	3.06198E-06	0.004789421
1564.19997	128.409018	2.89801E-06	0.004533071
1564.23997	145.107153	3.27487E-06	0.005122677
1564.27997	163.550624	3.69111E-06	0.005773929
1564.31997	162.601255	3.66968E-06	0.00574056
1564.35997	155.415893	3.50752E-06	0.005487024
1564.39997	160.815526	3.62938E-06	0.005677806
1564.43997	153.597748	3.46649E-06	0.005423111
1564.47997	126.096026	2.84581E-06	0.004452215
1564.51997	124.754093	2.81553E-06	0.004404947
1564.559969	142.766433	3.22204E-06	0.005041074
1564.599969	140.426556	3.16923E-06	0.00495858
1564.639969	122.38238	2.762E-06	0.004321535
1564.679969	122.161263	2.75701E-06	0.004313838
1564.719969	129.588335	2.92463E-06	0.004576224
1564.759969	141.308561	3.18914E-06	0.004990235
1564.799969	142.861628	3.22419E-06	0.005045209
1564.839969	134.588229	3.03747E-06	0.004753153
1564.879969	135.166548	3.05052E-06	0.004773699
1564.919969	144.271766	3.25601E-06	0.005095399
1564.959969	152.322166	3.4377E-06	0.005379862

1564.999969	144.701123	3.2657E-06	0.005110825
1565.039969	135.656179	3.06157E-06	0.004791481
1565.079969	140.504065	3.17098E-06	0.004962839
1565.119969	143.95228	3.2488E-06	0.005084766
1565.159969	149.975993	3.38475E-06	0.005297674
1565.199969	164.045314	3.70227E-06	0.0057948
1565.239969	185.736204	4.19181E-06	0.006561185
1565.279969	212.300105	4.79132E-06	0.007499754
1565.319969	216.770698	4.89221E-06	0.007657878
1565.359969	223.411626	5.04209E-06	0.007892685
1565.399969	201.239475	4.54169E-06	0.007109569
1565.439969	161.844818	3.65261E-06	0.005717945
1565.479969	145.138354	3.27557E-06	0.00512784
1565.519969	140.645609	3.17418E-06	0.004969235
1565.559969	148.348905	3.34803E-06	0.005241539
1565.599969	170.065421	3.83814E-06	0.006008991
1565.639969	165.808375	3.74206E-06	0.005858725
1565.679969	148.63387	3.35446E-06	0.00525201
1565.719969	134.458328	3.03454E-06	0.004751235
1565.759969	123.950137	2.79738E-06	0.004380029
1565.799969	124.956421	2.82009E-06	0.004415701
1565.839969	142.873388	3.22445E-06	0.005048978
1565.879969	159.390474	3.59722E-06	0.005632816
1565.919969	141.332456	3.18968E-06	0.004994778
1565.959969	123.462816	2.78638E-06	0.004363365
1565.999969	126.94531	2.86498E-06	0.004486557
1566.039969	119.582649	2.69881E-06	0.00422645
1566.079969	123.184786	2.78011E-06	0.004353873
1566.119969	145.668569	3.28754E-06	0.005148677
1566.159969	166.100562	3.74866E-06	0.005870999
1566.199969	184.459858	4.163E-06	0.006520094
1566.239969	194.725539	4.39468E-06	0.00688313
1566.279969	186.833137	4.21656E-06	0.006604319
1566.319969	183.187982	4.1343E-06	0.006475633
1566.359969	207.588914	4.68499E-06	0.007338385
1566.399969	198.02871	4.46923E-06	0.007000605
1566.439969	191.31896	4.3178E-06	0.006763578
1566.479969	168.197511	3.79598E-06	0.005946332
1566.519969	155.328487	3.50555E-06	0.00549151
1566.559969	150.525525	3.39715E-06	0.005321841
1566.599969	137.124379	3.09471E-06	0.004848167
1566.639969	139.291242	3.14361E-06	0.004924904
1566.679969	137.663601	3.10688E-06	0.00486748
1566.719969	129.831775	2.93012E-06	0.004590681
1566.759969	133.708831	3.01762E-06	0.004727889
1566.799969	170.127196	3.83953E-06	0.006015782

1566.839969	187.135179	4.22338E-06	0.006617361
1566.879969	172.031546	3.88251E-06	0.006083431
1566.919969	153.104676	3.45536E-06	0.005414271
1566.959969	152.308066	3.43738E-06	0.005386238
1566.999969	159.722952	3.60472E-06	0.005648603
1567.039969	164.087727	3.70323E-06	0.005803112
1567.079969	158.217341	3.57075E-06	0.005595643
1567.119969	147.938174	3.33876E-06	0.005232235
1567.159969	147.452071	3.32779E-06	0.005215176
1567.199969	148.522011	3.35193E-06	0.005253152
1567.239969	147.993104	3.34E-06	0.005234579
1567.279969	154.910926	3.49612E-06	0.005479405
1567.319969	159.871479	3.60808E-06	0.005655011
1567.359969	177.859157	4.01403E-06	0.006291435
1567.399969	176.803491	3.99021E-06	0.006254253
1567.439969	160.481744	3.62185E-06	0.005677032
1567.479969	156.198869	3.52519E-06	0.005525666
1567.519969	152.050928	3.43158E-06	0.005379067
1567.559969	141.408235	3.19139E-06	0.00500269
1567.599969	128.207844	2.89347E-06	0.004535807
1567.639969	120.766891	2.72554E-06	0.004272666
1567.679969	139.964921	3.15881E-06	0.004952008
1567.719969	149.856225	3.38205E-06	0.005302101
1567.759969	155.705271	3.51405E-06	0.005509189
1567.799969	169.563777	3.82682E-06	0.005999686
1567.839969	171.401933	3.8683E-06	0.00606488
1567.879969	162.73543	3.67271E-06	0.005758372
1567.919969	161.464703	3.64403E-06	0.005713553
1567.959969	144.128375	3.25278E-06	0.005100224
1567.999969	137.302588	3.09873E-06	0.004858806
1568.039969	149.306749	3.36965E-06	0.005283739
1568.079969	159.824656	3.60702E-06	0.005656096
1568.119969	149.868102	3.38231E-06	0.005303875
1568.159969	150.58583	3.39851E-06	0.005329411
1568.199969	169.79153	3.83196E-06	0.006009277
1568.239969	166.463014	3.75684E-06	0.005891624
1568.279969	199.413191	4.50048E-06	0.007058009
1568.319969	229.769981	5.18559E-06	0.008132662
1568.359969	233.021123	5.25896E-06	0.008247946
1568.399969	220.981454	4.98724E-06	0.007821993
1568.439969	200.304477	4.52059E-06	0.007090279
1568.479969	183.434293	4.13986E-06	0.006493282
1568.519969	151.437451	3.41773E-06	0.005360781
1568.559969	140.187897	3.16385E-06	0.004962682
1568.599969	156.62537	3.53482E-06	0.005544713
1568.639969	148.060304	3.34151E-06	0.005241634

1568.679969	143.344724	3.23509E-06	0.005074822
1568.719969	142.040876	3.20566E-06	0.00502879
1568.759969	131.985678	2.97873E-06	0.004672917
1568.799969	151.909385	3.42838E-06	0.005378448
1568.839969	176.152334	3.97551E-06	0.006236943
1568.879969	159.710063	3.60443E-06	0.005654924
1568.919969	133.306418	3.00854E-06	0.004720159
1568.959969	148.613607	3.354E-06	0.005262295
1568.999969	179.764997	4.05705E-06	0.006365504
1569.039969	186.639801	4.2122E-06	0.006609111
1569.079969	191.451137	4.32079E-06	0.006779658
1569.119969	179.290015	4.04633E-06	0.006349171
1569.159969	180.606057	4.07603E-06	0.006395939
1569.199969	181.464198	4.09539E-06	0.006426492
1569.239969	195.200687	4.40541E-06	0.006913142
1569.279969	192.455451	4.34345E-06	0.006816091
1569.319969	202.091786	4.56093E-06	0.007157559
1569.359969	208.142097	4.69748E-06	0.007372033
1569.399969	215.270046	4.85835E-06	0.007624687
1569.439969	191.642537	4.32511E-06	0.006787993
1569.479969	150.786029	3.40303E-06	0.005340988
1569.519969	146.241798	3.30047E-06	0.005180159
1569.559969	152.517143	3.4421E-06	0.005402582
1569.599969	154.799363	3.49361E-06	0.005483564
1569.639969	152.062475	3.43184E-06	0.00538675
1569.679969	141.162321	3.18584E-06	0.005000744
1569.719969	127.977307	2.88827E-06	0.004533774
1569.759969	125.190084	2.82537E-06	0.004435146
1569.799969	136.434247	3.07913E-06	0.00483362
1569.839969	158.808498	3.58409E-06	0.005626442
1569.879969	156.982351	3.54287E-06	0.005561885
1569.919969	153.395581	3.46192E-06	0.005434945
1569.959969	156.663784	3.53568E-06	0.005550881
1569.999969	162.91609	3.67679E-06	0.005772559
1570.039969	148.814262	3.35853E-06	0.005273027
1570.079969	145.791388	3.29031E-06	0.005166048
1570.119969	149.935101	3.38383E-06	0.005313013
1570.159969	149.292385	3.36932E-06	0.005290373
1570.199969	155.237758	3.5035E-06	0.005501196
1570.239969	168.633025	3.80581E-06	0.005976039
1570.279969	193.498024	4.36698E-06	0.006857383
1570.319969	207.832986	4.6905E-06	0.007365587
1570.359969	208.464282	4.70475E-06	0.007388149
1570.399969	202.566647	4.57165E-06	0.007179314
1570.439969	186.688532	4.2133E-06	0.006616735
1570.479969	155.302515	3.50496E-06	0.005504472

1570.519969	163.185924	3.68288E-06	0.005784035
1570.559969	171.810674	3.87753E-06	0.00608989
1570.599969	162.44713	3.66621E-06	0.005758142
1570.639969	146.064768	3.29648E-06	0.005177581
1570.679969	145.01935	3.27288E-06	0.005140654
1570.719969	149.027001	3.36333E-06	0.005282852
1570.759969	161.444806	3.64358E-06	0.005723197
1570.799969	165.52463	3.73566E-06	0.005867975
1570.839969	162.74647	3.67296E-06	0.005769634
1570.879969	164.463672	3.71172E-06	0.00583066
1570.919969	156.409631	3.52995E-06	0.005545265
1570.959969	147.128019	3.32047E-06	0.005216332
1570.999969	142.620527	3.21875E-06	0.005056651
1571.039969	142.708044	3.22072E-06	0.005059883
1571.079969	141.751867	3.19914E-06	0.005026108
1571.119969	139.128544	3.13994E-06	0.004933218
1571.159969	150.343215	3.39304E-06	0.005331004
1571.199969	169.886312	3.8341E-06	0.006024134
1571.239969	168.095314	3.79368E-06	0.005960777
1571.279969	175.298027	3.95623E-06	0.006216349
1571.319969	191.711921	4.32667E-06	0.006798585
1571.359969	183.460379	4.14045E-06	0.00650613
1571.399969	180.606951	4.07605E-06	0.006405101
1571.439969	178.354236	4.02521E-06	0.006325371
1571.479969	169.850316	3.83329E-06	0.006023931
1571.519969	158.632129	3.58011E-06	0.005626208
1571.559969	149.849417	3.38189E-06	0.005314847
1571.599969	139.648149	3.15166E-06	0.004953155
1571.639969	136.785501	3.08706E-06	0.004851744
1571.679969	138.833042	3.13327E-06	0.004924495
1571.719969	148.198806	3.34464E-06	0.005256838
1571.759969	144.121707	3.25263E-06	0.005112348
1571.799969	146.698908	3.31079E-06	0.0052039
1571.839969	161.406576	3.64272E-06	0.005725775
1571.879969	182.27705	4.11374E-06	0.006466304
1571.919969	179.724423	4.05613E-06	0.006375912
1571.959969	152.967136	3.45226E-06	0.005426807
1571.999969	135.705664	3.06269E-06	0.004814545
1572.039969	143.042588	3.22827E-06	0.005074973
1572.079969	146.898104	3.31529E-06	0.005211894
1572.119969	147.691599	3.33319E-06	0.00524018
1572.159969	155.14137	3.50132E-06	0.005504642
1572.199969	153.761258	3.47018E-06	0.005455813
1572.239969	144.706509	3.26582E-06	0.00513466
1572.279969	154.279112	3.48186E-06	0.005474466
1572.319969	171.689308	3.87479E-06	0.006092407

1572.359969	159.390114	3.59721E-06	0.005656114
1572.399969	165.085683	3.72575E-06	0.005858375
1572.439969	180.269747	4.06844E-06	0.006397373
1572.479969	174.219419	3.93189E-06	0.006182818
1572.519969	153.496199	3.4642E-06	0.005447516
1572.559969	141.71533	3.19832E-06	0.005029546
1572.599969	146.593197	3.3084E-06	0.005202796
1572.639969	147.629057	3.33178E-06	0.005239694
1572.679969	136.466566	3.07986E-06	0.004843635
1572.719969	139.368332	3.14535E-06	0.004946753
1572.759969	130.717259	2.95011E-06	0.004639809
1572.799969	129.407045	2.92054E-06	0.00459342
1572.839969	144.904204	3.27029E-06	0.005143637
1572.879969	151.938219	3.42903E-06	0.005393459
1572.919969	152.81182	3.44875E-06	0.005424608
1572.959969	157.503867	3.55464E-06	0.005591311
1572.999969	156.751725	3.53767E-06	0.005564752
1573.039969	146.807632	3.31324E-06	0.005211865
1573.079969	133.94977	3.02306E-06	0.004755515
1573.119969	133.906673	3.02209E-06	0.004754106
1573.159969	141.362463	3.19035E-06	0.005018937
1573.199969	148.809724	3.35843E-06	0.005283479
1573.239969	165.331098	3.73129E-06	0.005870219
1573.279969	175.619847	3.9635E-06	0.006235688
1573.319969	157.424778	3.55286E-06	0.005589782
1573.359969	142.00236	3.2048E-06	0.005042297
1573.399969	136.501621	3.08065E-06	0.004847097
1573.439969	151.708162	3.42384E-06	0.00538721
1573.479969	160.682739	3.62639E-06	0.005706045
1573.519969	155.243424	3.50363E-06	0.005513028
1573.559969	153.990507	3.47535E-06	0.005468674
1573.599969	143.696641	3.24303E-06	0.005103237
1573.639969	146.972417	3.31696E-06	0.005219705
1573.679969	151.345123	3.41565E-06	0.005375138
1573.719969	150.27089	3.3914E-06	0.005337121
1573.759969	146.394591	3.30392E-06	0.00519958
1573.799969	140.170888	3.16346E-06	0.004978656
1573.839969	133.884789	3.02159E-06	0.004755504
1573.879969	136.075708	3.07104E-06	0.004833447
1573.919969	141.655401	3.19697E-06	0.005031767
1573.959969	153.076434	3.45472E-06	0.005437594
1573.999969	145.303704	3.2793E-06	0.005161622
1574.039969	150.090235	3.38733E-06	0.005331789
1574.079969	159.823208	3.60699E-06	0.005677686
1574.119969	169.126995	3.81696E-06	0.006008354
1574.159969	175.936531	3.97064E-06	0.006250426

1574.199969	164.633098	3.71554E-06	0.005849002
1574.239969	157.941591	3.56452E-06	0.005611413
1574.279969	150.420077	3.39477E-06	0.005344321
1574.319969	139.03561	3.13784E-06	0.004939964
1574.359969	130.387653	2.94267E-06	0.004632818
1574.399969	129.888789	2.93141E-06	0.00461521
1574.439969	131.187604	2.96072E-06	0.004661478
1574.479969	144.222782	3.25491E-06	0.005124786
1574.519969	153.840804	3.47197E-06	0.00546669
1574.559969	144.273045	3.25604E-06	0.005126833
1574.599969	133.280034	3.00794E-06	0.00473631
1574.639969	129.068309	2.91289E-06	0.004586756
1574.679969	114.584487	2.58601E-06	0.004072142
1574.719969	110.527987	2.49446E-06	0.00392808
1574.759969	117.666925	2.65558E-06	0.004181899
1574.799969	131.613452	2.97033E-06	0.004677679
1574.839969	128.535664	2.90087E-06	0.004568407
1574.879969	130.452555	2.94413E-06	0.004636655
1574.919969	146.47287	3.30569E-06	0.005206195
1574.959969	157.912751	3.56387E-06	0.005612954
1574.999969	146.949587	3.31645E-06	0.005223405
1575.039969	140.320045	3.16683E-06	0.004987881
1575.079969	158.379756	3.57441E-06	0.005629982
1575.119969	173.386877	3.9131E-06	0.006163602
1575.159969	187.89777	4.24059E-06	0.006679609
1575.199969	207.107999	4.67414E-06	0.007362704
1575.239969	191.018447	4.31102E-06	0.006790891
1575.279969	161.034244	3.63432E-06	0.005725069
1575.319969	173.932639	3.92542E-06	0.006183788
1575.359969	184.96413	4.17438E-06	0.006576156
1575.399969	190.989235	4.31036E-06	0.006790543
1575.439969	183.803299	4.14818E-06	0.006535216
1575.479969	178.730027	4.03369E-06	0.006354994
1575.519969	162.398715	3.66511E-06	0.005774458
1575.559969	124.263299	2.80445E-06	0.004418578
1575.599969	114.151098	2.57623E-06	0.00405911
1575.639969	136.103237	3.07166E-06	0.004839831
1575.679969	127.878471	2.88604E-06	0.004547474
1575.719969	123.13117	2.7789E-06	0.004378767
1575.759969	123.879467	2.79579E-06	0.004405489
1575.799969	135.953042	3.06827E-06	0.004834981
1575.839969	150.531512	3.39729E-06	0.00535358
1575.879969	151.262086	3.41377E-06	0.005379699
1575.919969	148.962136	3.36187E-06	0.005298035
1575.959969	146.306376	3.30193E-06	0.005203711
1575.999969	150.744573	3.4021E-06	0.005361702

1576.039969	137.042546	3.09286E-06	0.00487447
1576.079969	126.620218	2.85764E-06	0.004503872
1576.119969	137.489604	3.10295E-06	0.00489062
1576.159969	161.367566	3.64184E-06	0.005740124
1576.199969	181.737441	4.10156E-06	0.00646488
1576.239969	204.758556	4.62112E-06	0.007283987
1576.279969	199.792594	4.50904E-06	0.00710751
1576.319969	181.69028	4.1005E-06	0.006463695
1576.359969	179.991963	4.06217E-06	0.006403439
1576.399969	197.110013	4.4485E-06	0.007012613
1576.439969	192.019295	4.33361E-06	0.006831673
1576.479969	148.748814	3.35705E-06	0.005292328
1576.519969	125.759249	2.83821E-06	0.004474496
1576.559969	139.282709	3.14342E-06	0.004955785
1576.599969	145.553505	3.28494E-06	0.005179036
1576.639969	145.386738	3.28118E-06	0.005173233
1576.679969	138.76043	3.13163E-06	0.004937578
1576.719969	144.336311	3.25747E-06	0.005136117
1576.759969	162.344541	3.66389E-06	0.005777075
1576.799969	173.561447	3.91704E-06	0.006176389
1576.839969	171.206392	3.86389E-06	0.006092736
1576.879969	173.62228	3.91841E-06	0.006178867
1576.919969	178.506779	4.02865E-06	0.006352858
1576.959969	187.68036	4.23568E-06	0.006679505
1576.999969	176.110351	3.97457E-06	0.006267889
1577.039969	161.500649	3.64484E-06	0.005748066
1577.079969	149.987331	3.38501E-06	0.005338424
1577.119969	129.031775	2.91207E-06	0.00459268
1577.159969	130.216562	2.93881E-06	0.004634968
1577.199969	167.479474	3.77978E-06	0.005961466
1577.239969	191.66014	4.3255E-06	0.006822355
1577.279969	169.496366	3.8253E-06	0.006033564
1577.319969	145.975322	3.29446E-06	0.005196417
1577.359969	158.485796	3.5768E-06	0.005641907
1577.399969	179.947783	4.06117E-06	0.006406091
1577.439969	171.192666	3.86358E-06	0.006094566
1577.479969	170.782543	3.85432E-06	0.006080119
1577.519969	177.672612	4.00982E-06	0.006325576
1577.559969	163.093737	3.6808E-06	0.00580668
1577.599969	157.240686	3.5487E-06	0.005598434
1577.639969	161.614876	3.64742E-06	0.00575432
1577.679969	159.369824	3.59675E-06	0.005674528
1577.719969	160.024282	3.61153E-06	0.005697975
1577.759969	157.476379	3.55402E-06	0.005607394
1577.799969	158.958318	3.58747E-06	0.005660307
1577.839969	161.365902	3.6418E-06	0.005746183

1577.879969	159.861336	3.60785E-06	0.005692751
1577.919969	157.960335	3.56494E-06	0.005625197
1577.959969	137.514806	3.10352E-06	0.004897227
1577.999969	123.621664	2.78997E-06	0.004402571
1578.039969	130.897028	2.95416E-06	0.004661788
1578.079969	150.487108	3.39628E-06	0.005359608
1578.119969	159.666047	3.60344E-06	0.005686661
1578.159969	175.117429	3.95216E-06	0.006237135
1578.199969	196.934775	4.44454E-06	0.007014379
1578.239969	184.923636	4.17347E-06	0.006586735
1578.279969	165.764088	3.74106E-06	0.005904447
1578.319969	177.043645	3.99563E-06	0.00630638
1578.359969	174.796072	3.9449E-06	0.006226478
1578.399969	149.19131	3.36704E-06	0.005314536
1578.439969	135.924984	3.06764E-06	0.004842082
1578.479969	150.798198	3.40331E-06	0.005372049
1578.519969	146.511502	3.30656E-06	0.005219472
1578.559969	139.014494	3.13736E-06	0.004952516
1578.599969	130.973141	2.95588E-06	0.004666154
1578.639969	134.154713	3.02769E-06	0.004779625
1578.679969	154.215149	3.48042E-06	0.005494471
1578.719969	154.919579	3.49632E-06	0.005519709
1578.759969	137.462694	3.10234E-06	0.004897853
1578.799969	139.664584	3.15204E-06	0.004976433
1578.839969	153.972326	3.47494E-06	0.005486376
1578.879969	149.845806	3.38181E-06	0.005339474
1578.919969	134.120394	3.02691E-06	0.004779249
1578.959969	126.497895	2.85488E-06	0.004507743
1578.999969	123.466844	2.78647E-06	0.004399843
1579.039969	133.516633	3.01328E-06	0.004758097
1579.079969	162.628289	3.67029E-06	0.005795688
1579.119969	214.119228	4.83237E-06	0.007630896
1579.159969	228.445146	5.15569E-06	0.008141657
1579.199969	210.192642	4.74376E-06	0.007491338
1579.239969	188.409614	4.25214E-06	0.006715153
1579.279969	162.208446	3.66082E-06	0.005781458
1579.319969	167.753798	3.78597E-06	0.005979257
1579.359969	193.748817	4.37264E-06	0.006905974
1579.399969	184.159134	4.15622E-06	0.006564326
1579.439969	163.120034	3.68139E-06	0.005814538
1579.479969	153.265257	3.45898E-06	0.005463395
1579.519969	158.876241	3.58562E-06	0.005663551
1579.559969	165.637969	3.73822E-06	0.00590474
1579.599969	153.03359	3.45375E-06	0.005455551
1579.639969	136.381007	3.07793E-06	0.00486202
1579.679969	138.328762	3.12189E-06	0.004931583

1579.719969	127.183584	2.87036E-06	0.004534359
1579.759969	114.672937	2.58801E-06	0.004088432
1579.799969	126.891606	2.86377E-06	0.004524179
1579.839969	131.120845	2.95921E-06	0.004675086
1579.879969	146.021964	3.29551E-06	0.005206514
1579.919969	161.334594	3.6411E-06	0.005752642
1579.959969	151.241977	3.41332E-06	0.00539291
1579.999969	148.592368	3.35352E-06	0.005298566
1580.039969	143.602207	3.2409E-06	0.005120754
1580.079969	146.518153	3.30671E-06	0.005224867
1580.119969	166.553758	3.75889E-06	0.005939491
1580.159969	173.480996	3.91522E-06	0.006186681
1580.199969	187.250047	4.22597E-06	0.006677882
1580.239969	201.143204	4.53952E-06	0.007173534
1580.279969	205.448225	4.63668E-06	0.007327253
1580.319969	202.395888	4.56779E-06	0.007218575
1580.359969	217.17944	4.90144E-06	0.007746036
1580.399969	201.397684	4.54527E-06	0.007183337
1580.439969	168.236434	3.79686E-06	0.006000712
1580.479969	148.878299	3.35998E-06	0.005310375
1580.519969	142.285997	3.2112E-06	0.005075361
1580.559969	155.035248	3.49893E-06	0.005530268
1580.599969	153.824371	3.4716E-06	0.005487214
1580.639969	136.826551	3.08798E-06	0.004880992
1580.679969	135.639666	3.0612E-06	0.004838775
1580.719969	145.608745	3.28619E-06	0.005194541
1580.759969	155.927428	3.51906E-06	0.005562797
1580.799969	151.64358	3.42238E-06	0.005410105
1580.839969	158.366462	3.57411E-06	0.005650097
1580.879969	172.591465	3.89515E-06	0.006157763
1580.919969	164.280074	3.70757E-06	0.005861375
1580.959969	143.910752	3.24787E-06	0.005134745
1580.999969	127.174305	2.87015E-06	0.004537702
1581.039969	131.437402	2.96636E-06	0.004689932
1581.079969	157.501495	3.55459E-06	0.00562009
1581.119969	162.005292	3.65623E-06	0.005780944
1581.159969	145.731665	3.28896E-06	0.005200373
1581.199969	139.345205	3.14483E-06	0.004972601
1581.239969	154.967552	3.4974E-06	0.005530232
1581.279969	183.56099	4.14272E-06	0.006550794
1581.319969	182.748917	4.12439E-06	0.006521978
1581.359969	177.325576	4.00199E-06	0.006328589
1581.399969	180.296229	4.06903E-06	0.006434771
1581.439969	166.294056	3.75303E-06	0.005935184
1581.479969	154.196966	3.48001E-06	0.005503567
1581.519969	159.707389	3.60437E-06	0.005700388

1581.559969	154.954533	3.49711E-06	0.005530886
1581.599969	155.161299	3.50177E-06	0.005538406
1581.639969	166.222338	3.75141E-06	0.005933375
1581.679969	171.430082	3.86894E-06	0.006119422
1581.719969	159.673885	3.60362E-06	0.005699913
1581.759969	149.625367	3.37684E-06	0.005341344
1581.799969	147.951316	3.33906E-06	0.005281717
1581.839969	149.941135	3.38396E-06	0.005352887
1581.879969	140.345386	3.1674E-06	0.005010446
1581.919969	153.038933	3.45388E-06	0.005463755
1581.959969	158.767093	3.58315E-06	0.005668403
1581.999969	153.104895	3.45536E-06	0.005466386
1582.039969	153.396771	3.46195E-06	0.005476946
1582.079969	162.920131	3.67688E-06	0.005817119
1582.119969	169.937396	3.83525E-06	0.006067826
1582.159969	160.666097	3.62601E-06	0.005736928
1582.199969	151.350976	3.41578E-06	0.005404448
1582.239969	145.69719	3.28818E-06	0.005202694
1582.279969	173.466185	3.91489E-06	0.006194452
1582.319969	191.065868	4.31209E-06	0.006823107
1582.359969	174.365591	3.93519E-06	0.006226885
1582.399969	159.475616	3.59914E-06	0.005695283
1582.439969	149.422629	3.37226E-06	0.0053364
1582.479969	130.604984	2.94757E-06	0.004664475
1582.519969	125.818718	2.83955E-06	0.00449365
1582.559969	135.838554	3.06569E-06	0.004851633
1582.599969	141.80206	3.20027E-06	0.005064755
1582.639969	153.646194	3.46758E-06	0.005487932
1582.679969	169.831061	3.83285E-06	0.006066176
1582.719969	167.140442	3.77213E-06	0.005970221
1582.759969	142.356974	3.2128E-06	0.005085089
1582.799969	137.88043	3.11177E-06	0.004925308
1582.839969	148.215802	3.34502E-06	0.005294638
1582.879969	153.162478	3.45666E-06	0.005471484
1582.919969	159.156397	3.59194E-06	0.005685751
1582.959969	162.400772	3.66516E-06	0.0058018
1582.999969	147.934969	3.33869E-06	0.00528514
1583.039969	137.392036	3.10075E-06	0.004908606
1583.079969	140.013358	3.15991E-06	0.005002385
1583.119969	153.47698	3.46376E-06	0.00548355
1583.159969	174.023622	3.92747E-06	0.006217814
1583.199969	185.215766	4.18006E-06	0.006617874
1583.239969	194.961527	4.40001E-06	0.006966272
1583.279969	184.625797	4.16675E-06	0.006597127
1583.319969	183.24653	4.13562E-06	0.006548008
1583.359969	194.966641	4.40013E-06	0.006966983

1583.399969	193.712062	4.37181E-06	0.006922326
1583.439969	171.335352	3.8668E-06	0.006122846
1583.479969	141.414255	3.19152E-06	0.005053712
1583.519969	131.471029	2.96712E-06	0.004698491
1583.559969	149.534092	3.37478E-06	0.00534416
1583.599969	146.403024	3.30411E-06	0.005232392
1583.639969	136.960061	3.091E-06	0.004895028
1583.679969	138.880062	3.13433E-06	0.004963775
1583.719969	125.328731	2.82849E-06	0.004479544
1583.759969	130.33686	2.94152E-06	0.004658664
1583.799969	140.963946	3.18136E-06	0.005038638
1583.839969	160.215626	3.61584E-06	0.005726917
1583.879969	156.287362	3.52719E-06	0.005586643
1583.919969	141.074079	3.18385E-06	0.005042956
1583.959969	131.795838	2.97445E-06	0.004711407
1583.999969	137.559787	3.10453E-06	0.00491758
1584.039969	150.935563	3.40641E-06	0.005395882
1584.079969	153.161914	3.45665E-06	0.005475612
1584.119969	146.631856	3.30928E-06	0.005242291
1584.159969	140.659058	3.17448E-06	0.005028883
1584.199969	154.6618	3.4905E-06	0.005529652
1584.239969	173.165195	3.9081E-06	0.006191364
1584.279969	188.548379	4.25527E-06	0.006741546
1584.319969	168.002081	3.79157E-06	0.006007065
1584.359969	143.89265	3.24746E-06	0.00514514
1584.399969	132.265161	2.98504E-06	0.004729498
1584.439969	126.668014	2.85872E-06	0.004529471
1584.479969	140.828632	3.17831E-06	0.005035962
1584.519969	139.740459	3.15375E-06	0.004997176
1584.559969	134.229796	3.02938E-06	0.004800234
1584.599969	128.780341	2.90639E-06	0.00460547
1584.639969	136.757156	3.08642E-06	0.004890862
1584.679969	146.418494	3.30446E-06	0.005236514
1584.719969	140.399822	3.16863E-06	0.005021389
1584.759969	135.90646	3.06722E-06	0.004860807
1584.799969	149.584225	3.37591E-06	0.005350138
1584.839969	148.047036	3.34122E-06	0.005295292
1584.879969	157.715146	3.55941E-06	0.005641239
1584.919969	154.580632	3.48867E-06	0.005529262
1584.959969	148.736992	3.35679E-06	0.005320373
1584.999969	151.268811	3.41393E-06	0.005411073
1585.039969	150.179304	3.38934E-06	0.005372236
1585.079969	164.010134	3.70148E-06	0.005867142
1585.119969	165.300354	3.7306E-06	0.005913447
1585.159969	145.788242	3.29024E-06	0.005215553
1585.199969	144.020834	3.25035E-06	0.005152454

1585.239969	162.825319	3.67474E-06	0.005825346
1585.279969	195.993728	4.42331E-06	0.007012178
1585.319969	220.89214	4.98523E-06	0.007903181
1585.359969	224.677068	5.07065E-06	0.008038803
1585.399969	199.428413	4.50082E-06	0.007135602
1585.439969	176.039509	3.97297E-06	0.0062989
1585.479969	165.609537	3.73758E-06	0.005925853
1585.519969	136.136116	3.0724E-06	0.004871356
1585.559969	120.715833	2.72439E-06	0.004319681
1585.599969	130.360463	2.94205E-06	0.004664921
1585.639969	144.29804	3.25661E-06	0.005163804
1585.679969	151.275665	3.41408E-06	0.00541364
1585.719969	146.181262	3.29911E-06	0.005231461
1585.759969	133.915037	3.02228E-06	0.004792604
1585.799969	132.115659	2.98167E-06	0.004728326
1585.839969	138.322506	3.12175E-06	0.00495059
1585.879969	146.307327	3.30195E-06	0.0052365
1585.919969	152.122161	3.43319E-06	0.005444757
1585.959969	157.108914	3.54573E-06	0.005623385
1585.999969	160.122651	3.61375E-06	0.0057314
1586.039969	164.661655	3.71618E-06	0.005894017
1586.079969	166.602048	3.75998E-06	0.005963623
1586.119969	150.890169	3.40538E-06	0.005401343
1586.159969	155.634256	3.51245E-06	0.005571305
1586.199969	175.457219	3.95983E-06	0.006281074
1586.239969	179.089865	4.04181E-06	0.006411279
1586.279969	170.085568	3.83859E-06	0.006089085
1586.319969	170.281936	3.84303E-06	0.006096269
1586.359969	185.820215	4.1937E-06	0.006652723
1586.399969	176.902654	3.99245E-06	0.006333617
1586.439969	175.834201	3.96833E-06	0.006295522
1586.479969	172.222607	3.88682E-06	0.006166369
1586.519969	154.051373	3.47672E-06	0.005515894
1586.559969	133.806869	3.01983E-06	0.004791149
1586.599969	121.490506	2.74187E-06	0.004350253
1586.639969	127.279106	2.87251E-06	0.004557643
1586.679969	128.494352	2.89994E-06	0.004601274
1586.719969	141.42423	3.19175E-06	0.00506441
1586.759969	151.061591	3.40925E-06	0.005409661
1586.799969	164.413275	3.71058E-06	0.005887946
1586.839969	169.084667	3.81601E-06	0.00605539
1586.879969	158.519568	3.57757E-06	0.005677168
1586.919969	146.389531	3.30381E-06	0.005242879
1586.959969	144.400875	3.25893E-06	0.005171786
1586.999969	150.303445	3.39214E-06	0.005383325
1587.039969	149.585148	3.37593E-06	0.005357733

1587.079969	128.681707	2.90417E-06	0.004609145
1587.119969	128.622145	2.90282E-06	0.004607128
1587.159969	150.903895	3.40569E-06	0.005405376
1587.199969	154.921007	3.49635E-06	0.005549409
1587.239969	138.695585	3.13017E-06	0.004968325
1587.279969	155.503329	3.50949E-06	0.005570549
1587.319969	192.416536	4.34257E-06	0.006893053
1587.359969	169.411126	3.82337E-06	0.006069069
1587.399969	133.875847	3.02139E-06	0.004796157
1587.439969	126.035405	2.84444E-06	0.004515383
1587.479969	144.069149	3.25144E-06	0.005161596
1587.519969	167.256324	3.77474E-06	0.005992479
1587.559969	161.714889	3.64968E-06	0.005794086
1587.599969	147.413801	3.32692E-06	0.005281825
1587.639969	140.637006	3.17398E-06	0.00503914
1587.679969	141.283938	3.18858E-06	0.005062447
1587.719969	138.479741	3.12529E-06	0.004962093
1587.759969	141.290798	3.18874E-06	0.005062948
1587.799969	153.038947	3.45388E-06	0.005484064
1587.839969	155.595696	3.51158E-06	0.005575824
1587.879969	156.264272	3.52667E-06	0.005599924
1587.919969	166.766539	3.76369E-06	0.005976436
1587.959969	150.893313	3.40545E-06	0.005407721
1587.999969	163.318762	3.68588E-06	0.005853173
1588.039969	180.054126	4.06357E-06	0.006453113
1588.079969	185.400743	4.18424E-06	0.006644902
1588.119969	178.283286	4.02361E-06	0.006389968
1588.159969	156.425571	3.53031E-06	0.005606693
1588.199969	163.177838	3.6827E-06	0.005848858
1588.239969	164.92146	3.72205E-06	0.005911505
1588.279969	156.893308	3.54086E-06	0.005623882
1588.319969	154.423706	3.48513E-06	0.005535498
1588.359969	158.259101	3.57169E-06	0.005673125
1588.399969	164.543609	3.71352E-06	0.005898555
1588.439969	179.986071	4.06203E-06	0.006452299
1588.479969	172.797085	3.89979E-06	0.006194738
1588.519969	144.536919	3.262E-06	0.005181747
1588.559969	143.653562	3.24206E-06	0.005150208
1588.599969	166.550839	3.75882E-06	0.005971262
1588.639969	174.537525	3.93907E-06	0.006257762
1588.679969	155.06145	3.49952E-06	0.005559619
1588.719969	126.560093	2.85629E-06	0.004537837
1588.759969	131.785326	2.97421E-06	0.004725308
1588.799969	150.829452	3.40401E-06	0.005408292
1588.839969	166.718021	3.76259E-06	0.005978159
1588.879969	149.799219	3.38076E-06	0.005371621

1588.919969	133.603554	3.01525E-06	0.004790985
1588.959969	124.430622	2.80823E-06	0.004462158
1588.999969	117.021867	2.64102E-06	0.004196582
1589.039969	142.463135	3.21519E-06	0.005109073
1589.079969	168.632655	3.8058E-06	0.006047727
1589.119969	163.930629	3.69969E-06	0.005879245
1589.159969	162.951076	3.67758E-06	0.005844261
1589.199969	167.82348	3.78754E-06	0.006019162
1589.239969	163.922304	3.6995E-06	0.00587939
1589.279969	148.735218	3.35675E-06	0.00533481
1589.319969	160.561043	3.62364E-06	0.005759122
1589.359969	193.295579	4.36241E-06	0.006933443
1589.399969	202.413414	4.56819E-06	0.007260679
1589.439969	183.850648	4.14925E-06	0.006594989
1589.479969	149.77299	3.38017E-06	0.005372709
1589.519969	133.597105	3.0151E-06	0.004792563
1589.559969	123.128751	2.77884E-06	0.00441714
1589.599969	121.09585	2.73296E-06	0.004344321
1589.639969	121.771329	2.74821E-06	0.004368663
1589.679969	124.041487	2.79944E-06	0.004450219
1589.719969	120.68439	2.72368E-06	0.004329886
1589.759969	141.399706	3.19119E-06	0.005073233
1589.799969	180.808894	4.0806E-06	0.006487346
1589.839969	177.930103	4.01563E-06	0.006384216
1589.879969	151.194958	3.41226E-06	0.005425083
1589.919969	135.330328	3.05422E-06	0.004855961
1589.959969	134.048799	3.02529E-06	0.004810097
1589.999969	130.554676	2.94644E-06	0.004684835
1590.039969	134.195013	3.02859E-06	0.004815586
1590.079969	136.966943	3.09115E-06	0.004915181
1590.119969	147.635347	3.33192E-06	0.005298159
1590.159969	163.680058	3.69403E-06	0.0058741
1590.199969	159.833509	3.60722E-06	0.005736201
1590.239969	153.574341	3.46596E-06	0.005511706
1590.279969	156.20229	3.52527E-06	0.005606163
1590.319969	151.508419	3.41933E-06	0.005437835
1590.359969	157.17914	3.54731E-06	0.005641507
1590.399969	177.770354	4.01203E-06	0.006380731
1590.439969	164.473569	3.71194E-06	0.005903617
1590.479969	153.356081	3.46103E-06	0.005504704
1590.519969	151.815601	3.42627E-06	0.005449546
1590.559969	150.721113	3.40157E-06	0.005410394
1590.599969	149.32362	3.37003E-06	0.005360363
1590.639969	153.140922	3.45618E-06	0.005497534
1590.679969	155.579748	3.51122E-06	0.005585224
1590.719969	150.227452	3.39042E-06	0.005393216

1590.759969	169.946232	3.83545E-06	0.00610128
1590.799969	173.017562	3.90477E-06	0.006211701
1590.839969	163.833193	3.69749E-06	0.00588211
1590.879969	178.696007	4.03292E-06	0.006415892
1590.919969	210.388686	4.74818E-06	0.007553974
1590.959969	213.291875	4.8137E-06	0.007658405
1590.999969	187.136985	4.22342E-06	0.006719463
1591.039969	160.85444	3.63026E-06	0.00577589
1591.079969	141.103671	3.18451E-06	0.005066815
1591.119969	143.6392	3.24174E-06	0.005157992
1591.159969	159.28926	3.59494E-06	0.005720119
1591.199969	181.402073	4.09399E-06	0.00651436
1591.239969	193.287215	4.36222E-06	0.006941344
1591.279969	217.494395	4.90855E-06	0.00781087
1591.319969	229.140033	5.17137E-06	0.008229307
1591.359969	217.390595	4.9062E-06	0.007807535
1591.399969	196.120444	4.42617E-06	0.007043799
1591.439969	170.250289	3.84231E-06	0.006114809
1591.479969	148.544403	3.35244E-06	0.005335342
1591.519969	133.857949	3.02099E-06	0.004807962
1591.559969	137.988283	3.1142E-06	0.004956441
1591.599969	147.642931	3.3321E-06	0.005303363
1591.639969	141.846042	3.20127E-06	0.005095265
1591.679969	147.573078	3.33052E-06	0.00530112
1591.719969	172.998958	3.90435E-06	0.006214625
1591.759969	177.202812	3.99922E-06	0.006365799
1591.799969	150.469513	3.39589E-06	0.005405573
1591.839969	116.805741	2.63614E-06	0.004196318
1591.879969	98.1222	2.21448E-06	0.003525189
1591.919969	103.508182	2.33604E-06	0.003718782
1591.959969	137.062298	3.09331E-06	0.004924418
1591.999969	148.863893	3.35965E-06	0.005348564
1592.039969	152.860762	3.44985E-06	0.005492306
1592.079969	153.110487	3.45549E-06	0.005501417
1592.119969	155.206016	3.50278E-06	0.005576852
1592.159969	169.997573	3.83661E-06	0.006108494
1592.199969	180.325396	4.06969E-06	0.006479765
1592.239969	180.5921	4.07571E-06	0.006489512
1592.279969	187.587707	4.23359E-06	0.006741066
1592.319969	166.258114	3.75221E-06	0.005974725
1592.359969	143.728995	3.24376E-06	0.005165239
1592.399969	141.591842	3.19553E-06	0.005088563
1592.439969	139.274708	3.14324E-06	0.005005415
1592.479969	125.638858	2.83549E-06	0.004515467
1592.519969	110.370442	2.49091E-06	0.003966819
1592.559969	110.391756	2.49139E-06	0.003967685

1592.599969	138.508694	3.12595E-06	0.004978385
1592.639969	145.224195	3.27751E-06	0.00521989
1592.679969	137.552842	3.10438E-06	0.004944278
1592.719969	155.518437	3.50983E-06	0.005590184
1592.759969	173.959228	3.92602E-06	0.006253203
1592.799969	154.285306	3.482E-06	0.005546137
1592.839969	144.345674	3.25768E-06	0.005188964
1592.879969	145.899041	3.29274E-06	0.005244937
1592.919969	149.465314	3.37322E-06	0.005373276
1592.959969	150.479344	3.39611E-06	0.005409866
1592.999969	151.081975	3.40971E-06	0.005431668
1593.039969	157.695228	3.55896E-06	0.005669568
1593.079969	176.592531	3.98545E-06	0.006349137
1593.119969	183.27343	4.13623E-06	0.006589504
1593.159969	186.341758	4.20547E-06	0.006699993
1593.199969	185.405646	4.18435E-06	0.006666502
1593.239969	177.751542	4.0116E-06	0.006391449
1593.279969	159.529794	3.60037E-06	0.00573639
1593.319969	148.95179	3.36163E-06	0.005356159
1593.359969	156.279133	3.527E-06	0.005619784
1593.399969	160.856126	3.6303E-06	0.005784518
1593.439969	165.23418	3.72911E-06	0.005942105
1593.479969	146.3961	3.30396E-06	0.005264788
1593.519969	128.963366	2.91052E-06	0.004637977
1593.559969	117.202722	2.6451E-06	0.004215129
1593.599969	118.94474	2.68442E-06	0.004277887
1593.639969	122.972997	2.77533E-06	0.004422876
1593.679969	123.650981	2.79063E-06	0.004447372
1593.719969	120.040017	2.70914E-06	0.004317604
1593.759969	125.727924	2.8375E-06	0.0045223
1593.799969	143.445411	3.23736E-06	0.005159709
1593.839969	154.082232	3.47742E-06	0.005542453
1593.879969	150.453278	3.39552E-06	0.005412053
1593.919969	145.573004	3.28538E-06	0.005236633
1593.959969	136.655822	3.08413E-06	0.004915982
1593.999969	128.469645	2.89938E-06	0.004621613
1594.039969	126.576905	2.85666E-06	0.004553637
1594.079969	152.309545	3.43741E-06	0.005479513
1594.119969	168.504912	3.80292E-06	0.006062313
1594.159969	162.569036	3.66896E-06	0.005848904
1594.199969	165.600811	3.73738E-06	0.005958131
1594.239969	158.616934	3.57976E-06	0.005707002
1594.279969	167.627749	3.78312E-06	0.00603136
1594.319969	179.727296	4.05619E-06	0.006466872
1594.359969	181.55162	4.09737E-06	0.006532678
1594.399969	189.384499	4.27414E-06	0.006814696

1594.439969	187.030505	4.22102E-06	0.00673016
1594.479969	163.127877	3.68157E-06	0.005870188
1594.519969	151.241697	3.41331E-06	0.005442598
1594.559969	138.208346	3.11917E-06	0.004973703
1594.599969	127.637658	2.8806E-06	0.004593411
1594.639969	113.983926	2.57246E-06	0.004102145
1594.679969	118.491016	2.67418E-06	0.004264457
1594.719969	132.903387	2.99944E-06	0.004783274
1594.759969	137.581438	3.10502E-06	0.004951764
1594.799969	132.677548	2.99435E-06	0.004775385
1594.839969	134.117575	3.02685E-06	0.004827336
1594.879969	135.646764	3.06136E-06	0.004882499
1594.919969	127.963533	2.88796E-06	0.004606063
1594.959969	117.95416	2.66206E-06	0.004245881
1594.999969	125.378171	2.82961E-06	0.004513229
1595.039969	139.749411	3.15395E-06	0.005030676
1595.079969	158.897783	3.5861E-06	0.005720119
1595.119969	170.898877	3.85695E-06	0.006152297
1595.159969	172.694875	3.89748E-06	0.006217109
1595.199969	173.200116	3.90889E-06	0.006235454
1595.239969	189.414395	4.27482E-06	0.006819362
1595.279969	185.619141	4.18917E-06	0.006682892
1595.319969	176.046481	3.97312E-06	0.006338404
1595.359969	155.455517	3.50841E-06	0.005597184
1595.399969	139.154884	3.14053E-06	0.005010405
1595.439969	137.761664	3.10909E-06	0.004960365
1595.479969	133.775904	3.01914E-06	0.004816971
1595.519969	131.433835	2.96628E-06	0.004732757
1595.559969	115.43454	2.6052E-06	0.004156748
1595.599969	104.984461	2.36935E-06	0.00378054
1595.639969	120.400411	2.71727E-06	0.004335784
1595.679969	126.772504	2.86108E-06	0.004565366
1595.719969	138.639511	3.1289E-06	0.004992849
1595.759969	138.052674	3.11566E-06	0.00497184
1595.799969	134.988823	3.04651E-06	0.00486162
1595.839969	141.4666	3.1927E-06	0.005095045
1595.879969	141.244097	3.18768E-06	0.005087159
1595.919969	125.788872	2.83888E-06	0.004530625
1595.959969	118.408186	2.67231E-06	0.004264896
1595.999969	138.231678	3.1197E-06	0.004979035
1596.039969	170.960885	3.85835E-06	0.006158079
1596.079969	179.871679	4.05945E-06	0.006479212
1596.119969	163.827651	3.69736E-06	0.005901433
1596.159969	145.226483	3.27756E-06	0.005231509
1596.199969	153.6671	3.46805E-06	0.005535705
1596.239969	157.311259	3.5503E-06	0.005667124

1596.279969	170.221375	3.84166E-06	0.006132364
1596.319969	175.899778	3.96981E-06	0.006337092
1596.359969	164.838533	3.72018E-06	0.00593874
1596.399969	174.094214	3.92906E-06	0.006272358
1596.439969	195.353162	4.40885E-06	0.007038462
1596.479969	174.547332	3.93929E-06	0.006288998
1596.519969	125.607384	2.83478E-06	0.004525789
1596.559969	119.933154	2.70672E-06	0.004321448
1596.599969	135.712395	3.06284E-06	0.00489013
1596.639969	151.907703	3.42835E-06	0.005473833
1596.679969	158.783802	3.58353E-06	0.005721749
1596.719969	130.602394	2.94751E-06	0.004706355
1596.759969	126.621681	2.85767E-06	0.004563021
1596.799969	153.908213	3.47349E-06	0.005546475
1596.839969	180.553154	4.07483E-06	0.006506857
1596.879969	175.010081	3.94973E-06	0.006307251
1596.919969	157.556932	3.55584E-06	0.005678393
1596.959969	155.100601	3.5004E-06	0.005590006
1596.999969	146.558463	3.30762E-06	0.00528227
1597.039969	142.712534	3.22082E-06	0.005143783
1597.079969	149.020846	3.36319E-06	0.005371288
1597.119969	159.784162	3.60611E-06	0.005759384
1597.159969	166.121835	3.74914E-06	0.005987974
1597.199969	178.642697	4.03172E-06	0.006439458
1597.239969	170.517721	3.84835E-06	0.006146734
1597.279969	161.004445	3.63365E-06	0.00580395
1597.319969	180.212663	4.06715E-06	0.006496538
1597.359969	193.72912	4.3722E-06	0.006983971
1597.399969	175.114024	3.95208E-06	0.006313052
1597.439969	161.930602	3.65455E-06	0.005837921
1597.479969	150.566047	3.39807E-06	0.005428342
1597.519969	135.038002	3.04762E-06	0.004868633
1597.559969	126.63266	2.85792E-06	0.004565703
1597.599969	123.259608	2.7818E-06	0.0044442
1597.639969	120.306495	2.71515E-06	0.004337832
1597.679969	117.084668	2.64244E-06	0.00422177
1597.719969	120.33429	2.71578E-06	0.004339052
1597.759969	134.465795	3.03471E-06	0.004848731
1597.799969	136.2844	3.07575E-06	0.004914432
1597.839969	135.173692	3.05068E-06	0.004874502
1597.879969	129.851709	2.93057E-06	0.004682703
1597.919969	125.626106	2.83521E-06	0.004530433
1597.959969	139.260814	3.14292E-06	0.005022264
1597.999969	159.148344	3.59176E-06	0.005739627
1598.039969	161.077396	3.63529E-06	0.005809343
1598.079969	148.375388	3.34863E-06	0.005351372

1598.119969	157.284999	3.5497E-06	0.005672852
1598.159969	163.909372	3.69921E-06	0.005911923
1598.199969	162.658906	3.67098E-06	0.005866968
1598.239969	161.470352	3.64416E-06	0.005824244
1598.279969	166.105301	3.74877E-06	0.005991576
1598.319969	169.346239	3.82191E-06	0.006108633
1598.359969	167.606979	3.78266E-06	0.006046046
1598.399969	155.855634	3.51744E-06	0.005622283
1598.439969	128.447783	2.89889E-06	0.004633698
1598.479969	119.394672	2.69457E-06	0.004307218
1598.519969	125.694842	2.83676E-06	0.004534614
1598.559969	126.903674	2.86404E-06	0.004578338
1598.599969	140.976565	3.18164E-06	0.005086177
1598.639969	158.921484	3.58664E-06	0.005733741
1598.679969	148.590469	3.35348E-06	0.005361141
1598.719969	140.665535	3.17463E-06	0.005075337
1598.759969	136.119943	3.07204E-06	0.004911451
1598.799969	138.507302	3.12592E-06	0.004997716
1598.839969	149.823272	3.3813E-06	0.005406162
1598.879969	170.471503	3.8473E-06	0.006151378
1598.919969	178.183266	4.02135E-06	0.006429814
1598.959969	173.824859	3.92298E-06	0.006272696
1598.999969	161.792703	3.65144E-06	0.005838646
1599.039969	134.356797	3.03225E-06	0.004848682
1599.079969	135.868664	3.06637E-06	0.004903365
1599.119969	134.614761	3.03807E-06	0.004858235
1599.159969	133.021294	3.00211E-06	0.004800847
1599.199969	159.740862	3.60513E-06	0.005765322
1599.239969	170.286098	3.84312E-06	0.006146071
1599.279969	169.747244	3.83096E-06	0.006126776
1599.319969	172.895518	3.90201E-06	0.006240564
1599.359969	175.265253	3.95549E-06	0.006326257
1599.399969	172.093481	3.88391E-06	0.006211926
1599.439969	158.334972	3.5734E-06	0.005715438
1599.479969	158.22361	3.57089E-06	0.005711561
1599.519969	151.426046	3.41747E-06	0.005466319
1599.559969	131.57167	2.96939E-06	0.004749716
1599.599969	126.089603	2.84567E-06	0.004551928
1599.639969	117.444473	2.65056E-06	0.004239939
1599.679969	127.694578	2.88189E-06	0.0046101
1599.719969	142.832016	3.22352E-06	0.005156729
1599.759969	136.457007	3.07964E-06	0.004926692
1599.799969	127.529323	2.87816E-06	0.004604479
1599.839969	135.769311	3.06412E-06	0.004902108
1599.879969	160.127066	3.61384E-06	0.005781718
1599.919969	140.438562	3.1695E-06	0.005070951

1599.959969	103.824053	2.34316E-06	0.003748969
1599.999969	99.989115	2.25662E-06	0.003610584
1600.039969	114.778452	2.59039E-06	0.004144727
1600.079969	145.781466	3.29008E-06	0.005264399
1600.119969	181.997941	4.10744E-06	0.006572397
1600.159969	167.252308	3.77465E-06	0.006040046
1600.199969	141.202465	3.18674E-06	0.005099426
1600.239969	148.300611	3.34694E-06	0.005355904
1600.279969	173.321913	3.91163E-06	0.00625971
1600.319969	182.515	4.11911E-06	0.006591893
1600.359969	176.011847	3.97234E-06	0.006357177
1600.399969	180.515447	4.07398E-06	0.006520001
1600.439969	182.029338	4.10815E-06	0.006574845
1600.479969	162.707943	3.67209E-06	0.005877109
1600.519969	138.115024	3.11706E-06	0.004988923
1600.559969	130.493463	2.94506E-06	0.004713738
1600.599969	133.685206	3.01709E-06	0.004829152
1600.639969	145.33247	3.27995E-06	0.005250021
1600.679969	141.98223	3.20434E-06	0.005129125
1600.719969	132.26228	2.98498E-06	0.00477811
1600.759969	127.383553	2.87487E-06	0.004601976
1600.799969	128.100698	2.89105E-06	0.004628
1600.839969	124.250861	2.80417E-06	0.004489026
1600.879969	107.672895	2.43003E-06	0.003890182
1600.919969	101.749049	2.29633E-06	0.003676247
1600.959969	120.340268	2.71591E-06	0.004348067
1600.999969	127.717284	2.8824E-06	0.004614724
1601.039969	128.941195	2.91002E-06	0.004659063
1601.079969	141.457955	3.19251E-06	0.005111462
1601.119969	146.521069	3.30678E-06	0.005294546
1601.159969	162.735963	3.67272E-06	0.005880619
1601.199969	179.746632	4.05663E-06	0.006495478
1601.239969	174.243706	3.93244E-06	0.006296776
1601.279969	169.645433	3.82866E-06	0.006130758
1601.319969	175.471418	3.96015E-06	0.00634146
1601.359969	180.197245	4.0668E-06	0.006512412
1601.399969	162.12447	3.65892E-06	0.0058594
1601.439969	143.645999	3.24189E-06	0.005191692
1601.479969	134.467064	3.03473E-06	0.004860066
1601.519969	124.41226	2.80781E-06	0.004496766
1601.559969	107.22451	2.41991E-06	0.003875628
1601.599969	104.203852	2.35174E-06	0.00376654
1601.639969	109.510726	2.4715E-06	0.00395846
1601.679969	108.751895	2.45438E-06	0.003931129
1601.719969	122.101057	2.75565E-06	0.004413781
1601.759969	157.980017	3.56539E-06	0.005710897

1601.799969	167.941499	3.79021E-06	0.006071151
1601.839969	141.642818	3.19668E-06	0.005120572
1601.879969	139.162998	3.14072E-06	0.005031048
1601.919969	141.73986	3.19887E-06	0.005124336
1601.959969	135.241732	3.05222E-06	0.00488953
1601.999969	138.478099	3.12526E-06	0.005006663
1602.039969	142.88291	3.22467E-06	0.005166047
1602.079969	153.100969	3.45528E-06	0.005535628
1602.119969	169.722067	3.83039E-06	0.006136745
1602.159969	177.327413	4.00203E-06	0.006411897
1602.199969	173.543912	3.91664E-06	0.006275247
1602.239969	159.853748	3.60768E-06	0.005780363
1602.279969	152.235387	3.43574E-06	0.005505018
1602.319969	154.435345	3.48539E-06	0.005584711
1602.359969	155.016783	3.49851E-06	0.005605877
1602.399969	160.195171	3.61538E-06	0.005793288
1602.439969	148.16801	3.34395E-06	0.005358472
1602.479969	148.608594	3.35389E-06	0.00537454
1602.519969	148.211283	3.34492E-06	0.005360305
1602.559969	146.44687	3.3051E-06	0.005296624
1602.599969	135.143688	3.05E-06	0.004887938
1602.639969	126.779258	2.86123E-06	0.004585524
1602.679969	112.947282	2.54906E-06	0.004085332
1602.719969	118.599343	2.67662E-06	0.004289875
1602.759969	132.35766	2.98713E-06	0.004787649
1602.799969	150.158453	3.38887E-06	0.005431676
1602.839969	141.69278	3.19781E-06	0.005125576
1602.879969	124.170472	2.80235E-06	0.004491838
1602.919969	124.086548	2.80046E-06	0.004488914
1602.959969	137.318411	3.09909E-06	0.00496771
1602.999969	138.37207	3.12286E-06	0.005005952
1603.039969	151.51309	3.41944E-06	0.005481498
1603.079969	158.071411	3.56745E-06	0.00571891
1603.119969	153.797358	3.47099E-06	0.005564417
1603.159969	146.912006	3.3156E-06	0.005315436
1603.199969	158.71697	3.58202E-06	0.005742696
1603.239969	176.636044	3.98643E-06	0.006391203
1603.279969	179.057318	4.04107E-06	0.006478973
1603.319969	173.970382	3.92627E-06	0.006295066
1603.359969	171.579469	3.87231E-06	0.006208706
1603.399969	160.47672	3.62174E-06	0.005807091
1603.439969	149.283982	3.36913E-06	0.0054022
1603.479969	146.378852	3.30357E-06	0.005297203
1603.519969	147.120276	3.3203E-06	0.005324167
1603.559969	142.397268	3.21371E-06	0.005153373
1603.599969	151.733532	3.42441E-06	0.005491391

1603.639969	154.023996	3.47611E-06	0.005574424
1603.679969	170.39669	3.84562E-06	0.006167137
1603.719969	172.023917	3.88234E-06	0.006226187
1603.759969	139.275372	3.14325E-06	0.00504102
1603.799969	109.098774	2.46221E-06	0.003948888
1603.839969	100.327722	2.26426E-06	0.003631506
1603.879969	100.093363	2.25897E-06	0.003623113
1603.919969	110.368385	2.49086E-06	0.003995141
1603.959969	129.484628	2.92229E-06	0.004687232
1603.999969	155.32519	3.50547E-06	0.005622779
1604.039969	162.174886	3.66006E-06	0.005870885
1604.079969	171.501857	3.87056E-06	0.006208685
1604.119969	172.067699	3.88333E-06	0.006229324
1604.159969	157.21978	3.54823E-06	0.005691931
1604.199969	154.057949	3.47687E-06	0.0055776
1604.239969	140.656512	3.17442E-06	0.005092534
1604.279969	149.381049	3.37132E-06	0.005408545
1604.319969	168.535394	3.80361E-06	0.006102206
1604.359969	161.949141	3.65497E-06	0.005863882
1604.399969	155.67468	3.51336E-06	0.005636836
1604.439969	130.817657	2.95237E-06	0.004736904
1604.479969	128.873215	2.90849E-06	0.004666612
1604.519969	145.099211	3.27469E-06	0.005254301
1604.559969	152.76959	3.4478E-06	0.005532197
1604.599969	133.749056	3.01853E-06	0.004843533
1604.639969	121.444446	2.74083E-06	0.004398049
1604.679969	109.797221	2.47797E-06	0.003976349
1604.719969	123.584374	2.78913E-06	0.004475768
1604.759969	153.729186	3.46945E-06	0.00556764
1604.799969	159.448902	3.59854E-06	0.005774936
1604.839969	154.999433	3.49812E-06	0.005613925
1604.879969	150.894292	3.40547E-06	0.005465377
1604.919969	129.380535	2.91994E-06	0.004686268
1604.959969	118.448276	2.67321E-06	0.004290399
1604.999969	111.100741	2.50739E-06	0.004024359
1605.039969	106.917923	2.41299E-06	0.003872943
1605.079969	117.94551	2.66187E-06	0.004272508
1605.119969	140.431185	3.16934E-06	0.005087165
1605.159969	152.743571	3.44721E-06	0.005533323
1605.199969	168.107872	3.79396E-06	0.006090065
1605.239969	162.209674	3.66085E-06	0.005876537
1605.279969	146.23342	3.30028E-06	0.005297881
1605.319969	149.159111	3.36631E-06	0.00540401
1605.359969	141.036734	3.183E-06	0.005109865
1605.399969	115.958095	2.61701E-06	0.004201352
1605.439969	116.200927	2.62249E-06	0.004210255

1605.479969	134.750972	3.04114E-06	0.004882492
1605.519969	144.497339	3.2611E-06	0.005235767
1605.559969	142.956933	3.22634E-06	0.005180081
1605.599969	159.830307	3.60715E-06	0.005791636
1605.639969	184.845871	4.17171E-06	0.00669827
1605.679969	182.42909	4.11717E-06	0.006610858
1605.719969	162.178412	3.66014E-06	0.005877161
1605.759969	151.764932	3.42512E-06	0.005499926
1605.799969	145.424898	3.28204E-06	0.005270296
1605.839969	156.924714	3.54157E-06	0.005687198
1605.879969	148.882131	3.36006E-06	0.005395857
1605.919969	124.383412	2.80716E-06	0.004508075
1605.959969	119.167086	2.68944E-06	0.004319125
1605.999969	127.823596	2.8848E-06	0.00463299
1606.039969	146.643182	3.30953E-06	0.005315241
1606.079969	151.706868	3.42381E-06	0.005498917
1606.119969	141.973982	3.20416E-06	0.005146257
1606.159969	144.187259	3.25411E-06	0.005226614
1606.199969	153.671023	3.46814E-06	0.005570528
1606.239969	165.653625	3.73857E-06	0.006005043
1606.279969	178.26721	4.02324E-06	0.006462454
1606.319969	173.327178	3.91175E-06	0.006283527
1606.359969	158.418322	3.57528E-06	0.005743188
1606.399969	153.799848	3.47105E-06	0.005575892
1606.439969	159.443245	3.59841E-06	0.005780633
1606.479969	155.797471	3.51613E-06	0.005648595
1606.519969	153.06376	3.45444E-06	0.00554962
1606.559969	150.790346	3.40313E-06	0.005467329
1606.599969	135.010412	3.047E-06	0.004895305
1606.639969	135.610582	3.06054E-06	0.004917189
1606.679969	142.618246	3.2187E-06	0.005171413
1606.719969	148.337896	3.34778E-06	0.005378944
1606.759969	151.822227	3.42642E-06	0.005505428
1606.799969	133.754392	3.01865E-06	0.004850367
1606.839969	126.098985	2.84588E-06	0.004572871
1606.879969	160.341078	3.61867E-06	0.005814776
1606.919969	173.342014	3.91209E-06	0.006286412
1606.959969	159.320294	3.59564E-06	0.005778045
1606.999969	156.122816	3.52347E-06	0.005662223
1607.039969	150.665972	3.40032E-06	0.005464452
1607.079969	146.928564	3.31597E-06	0.005329034
1607.119969	146.699382	3.3108E-06	0.005320854
1607.159969	142.261486	3.21064E-06	0.005160018
1607.199969	141.425717	3.19178E-06	0.005129831
1607.239969	155.650978	3.51283E-06	0.005645954
1607.279969	177.289651	4.00118E-06	0.006431017

1607.319969	175.727307	3.96592E-06	0.006374503
1607.359969	168.408292	3.80074E-06	0.006109158
1607.399969	167.114353	3.77154E-06	0.00606237
1607.439969	166.086147	3.74833E-06	0.00602522
1607.479969	157.991615	3.56565E-06	0.005731712
1607.519969	141.612935	3.19601E-06	0.005137645
1607.559969	143.875044	3.24706E-06	0.005219843
1607.599969	135.380595	3.05535E-06	0.004911783
1607.639969	117.530222	2.65249E-06	0.004264254
1607.679969	125.354351	2.82907E-06	0.004548244
1607.719969	133.330148	3.00908E-06	0.004837751
1607.759969	138.03158	3.11518E-06	0.005008463
1607.799969	144.607627	3.26359E-06	0.005247204
1607.839969	145.079591	3.27424E-06	0.005264461
1607.879969	139.644095	3.15157E-06	0.005067351
1607.919969	142.470411	3.21536E-06	0.00517004
1607.959969	144.700566	3.26569E-06	0.005251099
1607.999969	144.791855	3.26775E-06	0.005254543
1608.039969	144.913883	3.2705E-06	0.005259102
1608.079969	146.289828	3.30156E-06	0.005309169
1608.119969	173.185186	3.90855E-06	0.006285415
1608.159969	206.862341	4.66859E-06	0.007507847
1608.199969	201.722384	4.55259E-06	0.00732148
1608.239969	170.127196	3.83953E-06	0.006174892
1608.279969	172.64027	3.89625E-06	0.006266262
1608.319969	174.867732	3.94652E-06	0.006347269
1608.359969	174.388707	3.93571E-06	0.006330039
1608.399969	168.527221	3.80342E-06	0.006117428
1608.439969	137.26881	3.09797E-06	0.004982892
1608.479969	134.081535	3.02603E-06	0.004867314
1608.519969	142.123214	3.20752E-06	0.005159365
1608.559969	136.88887	3.08939E-06	0.004969471
1608.599969	150.030222	3.38597E-06	0.005446676
1608.639969	170.849537	3.85584E-06	0.006202652
1608.679969	156.316415	3.52784E-06	0.005675172
1608.719969	151.622566	3.42191E-06	0.005504895
1608.759969	147.129408	3.32051E-06	0.005341897
1608.799969	165.768435	3.74116E-06	0.006018782
1608.839969	175.219785	3.95447E-06	0.006362104
1608.879969	157.837169	3.56217E-06	0.005731096
1608.919969	143.436725	3.23717E-06	0.005208343
1608.959969	151.799303	3.4259E-06	0.005512134
1608.999969	153.715623	3.46915E-06	0.005581858
1609.039969	129.878993	2.93119E-06	0.004716399
1609.079969	129.275436	2.91757E-06	0.004694598
1609.119969	149.392268	3.37158E-06	0.005425269

1609.159969	147.208371	3.32229E-06	0.005346093
1609.199969	142.215854	3.20961E-06	0.00516491
1609.239969	153.126467	3.45585E-06	0.005561294
1609.279969	173.34182	3.91208E-06	0.006295638
1609.319968	193.231821	4.36097E-06	0.007018201
[truncated: 553,051 more chars]
