# Supplementary material for: Scutellaria barbata D. Don Inhibits the Main Proteases (Mpro and TMPRSS2) of Severe Acute Respiratory Syndrome Coronavirus 2 (SARS-CoV-2) Infection
Source: Viruses. 2021 May 2;13(5):826. doi: 10.3390/v13050826 (PMC8147405; doi:10.3390/v13050826)
Supplement: Supplementary file 1 [file viruses-13-00826-s001.zip › viruses-1186444-suppl/viruses-1186444-suppl.pdf]

Figure S1

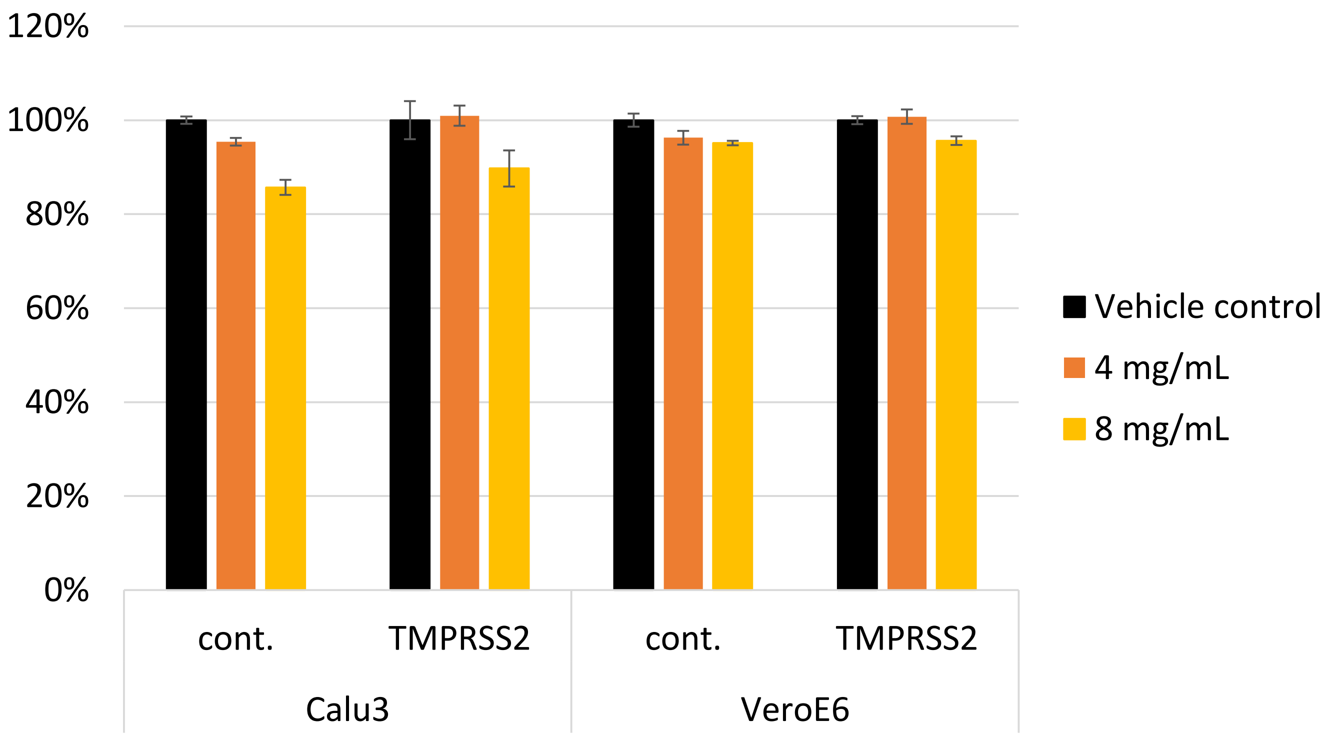

Supplementary Figure S1. *Scutellaria barbata* showed no cytotoxicity. Calu3 and VeroE6 cells with and without TMPRSS2 over-expression were treated with Scutellaria barbata. After 24 hours treatment, the cell viability measured by CCK8 assay demonstrated no cytotoxicity.
